# Epithelial-mesenchymal transition spectrum quantification and its efficacy in deciphering survival and drug responses of cancer patients

**DOI:** 10.15252/emmm.201404208

**Published:** 2014-09-11

**Authors:** Tuan Zea Tan, Qing Hao Miow, Yoshio Miki, Tetsuo Noda, Seiichi Mori, Ruby Yun-Ju Huang, Jean Paul Thiery

**Affiliations:** 1 Cancer Science Institute of Singapore, National University of SingaporeSingapore; 2 Institute of Molecular and Cell Biology, A*STARSingapore; 3 Cancer Institute of Japanese Foundation for Cancer ResearchKyoto, Japan; 4 Department of Obstetrics and Gynaecology, National University Health SystemSingapore; 5 Department of Biochemistry, Yong Loo Lin School of Medicine, National University of SingaporeSingapore

**Keywords:** drug response, epithelial-mesenchymal transition, gene expression signature, microarray, prognosis

## Abstract

Epithelial-mesenchymal transition (EMT) is a reversible and dynamic process hypothesized to be co-opted by carcinoma during invasion and metastasis. Yet, there is still no quantitative measure to assess the interplay between EMT and cancer progression. Here, we derived a method for universal EMT scoring from cancer-specific transcriptomic EMT signatures of ovarian, breast, bladder, lung, colorectal and gastric cancers. We show that EMT scoring exhibits good correlation with previously published, cancer-specific EMT signatures. This universal and quantitative EMT scoring was used to establish an EMT spectrum across various cancers, with good correlation noted between cell lines and tumours. We show correlations between EMT and poorer disease-free survival in ovarian and colorectal, but not breast, carcinomas, despite previous notions. Importantly, we found distinct responses between epithelial- and mesenchymal-like ovarian cancers to therapeutic regimes administered with or without paclitaxel*in vivo* and demonstrated that mesenchymal-like tumours do not always show resistance to chemotherapy. EMT scoring is thus a promising, versatile tool for the objective and systematic investigation of EMT roles and dynamics in cancer progression, treatment response and survival.

## Introduction

Accumulating evidence indicates that epithelial-mesenchymal transition (EMT) is of paramount importance in a plethora of cancer-related events, including cancer invasion, metastasis, resistance to cell death, refractory responses to chemotherapy and immunotherapy, immunosuppression and the acquisition of stem cell-like properties (Lee*et al*, 2006; Onder*et al*, 2008; Thiery*et al*, 2009; Jordan*et al*, 2011; Huang*et al*, 2012; Lee & Nelson, 2012; Frisch*et al*, 2013; Tam & Weinberg, 2013). In EMT, polarized epithelial (Epi) cells progressively alter their junctional and polarity complexes to acquire morphological and biochemical characteristics typical of mesenchymal (Mes) cells (Thiery*et al*, 2009). EMT was first described as a mechanism driving critical morphogenetic steps (for example, gastrulation) in the development of most metazoans (Jordan*et al*, 2011; Lim & Thiery, 2012) and, more recently, in wound-healing and carcinoma progression (Thiery*et al*, 2009). However, despite its potential involvement in invasion and metastasis, the role of EMT in human tumours is still inadequately documented (Wang*et al*, 2004; Chaffer & Weinberg, 2011; Kong*et al*, 2011). This is so even after the identification of a transitioned or ‘EMTed’ phenotype—either partially or completely—in circulating tumour cells (CTCs) (Jordan*et al*, 2011; Valastyan & Weinberg, 2011; Yu*et al*, 2013). Initially believed to be a binary process, EMT is now well documented to be a dynamic course, with the existence of intermediate states (Jordan*et al*, 2011; Kong*et al*, 2011; Huang*et al*, 2013; Tam & Weinberg, 2013). Cells stuck or transitioning in these intermediate or ‘metastable’ states of EMT (Jordan*et al*, 2011)—often called ‘fused cells’ (Kong*et al*, 2011)—have attributes of both Epi and Mes phenotypes and exhibit stem cell-like properties. They also display high plasticity between the Epi and Mes states, which is critical for metastasis, and hence, it is becoming increasingly clear that these intermediate phenotypes must also be quantitatively assessed and considered in the design of new therapeutic strategies (Chaffer & Weinberg, 2011; Valastyan & Weinberg, 2011).

Numerous signalling pathways initiate and execute the biochemical programs that lead to EMT in a context-dependent manner, including those associated with surface tyrosine or serine/threonine kinases, WNT signalling, cytokine receptors and downstream transcriptional regulators such as*SNAIL*,*ZEB* and*TWIST* (Thiery*et al*, 2009; Jordan*et al*, 2011; Lee & Nelson, 2012; Frisch*et al*, 2013; Tam & Weinberg, 2013). These diverse mechanisms nonetheless converge and generate similar EMTed phenotypic endpoints (Thiery*et al*, 2009; Tam & Weinberg, 2013), and this convergence likely reflects a series of molecular features common to all cancers undergoing EMT (Jordan*et al*, 2011). Thus, we sought to establish a generic EMT signature to capture a set of universal molecular features exhibited by a broad spectrum of cancers during EMT. Here, we developed an approach to quantitatively estimate the EMT status amongst clinical samples and cell lines using transcriptomics. We first established bladder, breast, colorectal, gastric, lung and ovarian cancer-specific EMT signatures and, from these, derived a generic EMT signature. We posit that this generic EMT signature exemplifies the common molecular features of EMT in tumours and cell lines of different origins and believe that this signature will be important in the future objective and systematic study of the role EMT and its dynamic nature in cancer progression, treatment response and survival.

## Results

### Cancer-specific EMT signature

**Figure 1:**
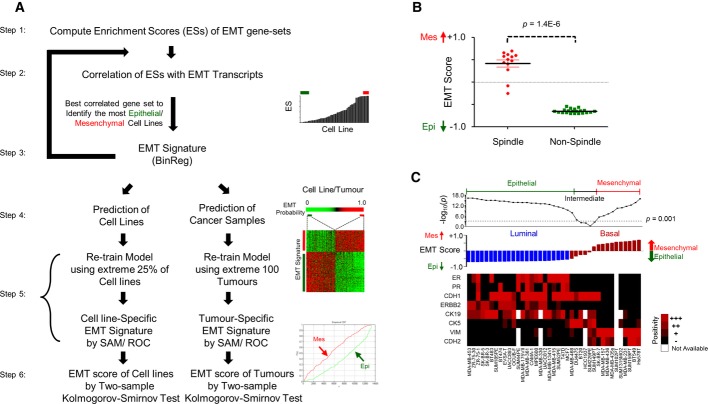
Derivation and application of cancer-specific epithelial-mesenchymal transition (EMT) signature A six-step scheme illustrating the generation of a cancer-specific EMT signature. Note that tumours and cell lines have their own cancer-specific EMT signatures. (Top right panel) Red and green bars on sample enrichment score (ES) bar chart indicate, respectively, mesenchymal-like (Mes) and epithelial-like (Epi) samples selected for building the BinReg EMT signature. (Middle right panel) Heatmap of the EMT signature from Significance Analysis of Microarray (SAM)/Receiver Operating Characteristics (ROC) analysis. The colour bar shows the EMT phenotype probability of cell line or tumour samples, sorted from most Epi to most Mes. Red and green bars indicate Mes and Epi samples selected for SAM/ROC analysis. (Bottom right panel) Plots of empirical cumulative distribution function of Mes (red) and Epi (green) gene sets.Dot plot of EMT score (mean ± SEM) for breast cancer cell lines (*n *= 34) with spindle- and non-spindle-like morphologies. Mann–Whitney*U-*test*P*-value is shown.Immunohistochemistry staining heatmap of Oestrogen Receptor (ER), Progesterone Receptor (PR), and Epi (CDH1, ERBB2, CK19) as well as Mes (CK5, VIM, CDH2) markers (black = low, red = high, white = no data). Breast cancer cell lines (*n *= 39) are aligned from the most Epi to most Mes based on the EMT score, as shown by the bar chart. Dot plot is the*-*log_10_*P*-value of two-sample Kolmogorov–Smirnov test. Arbitrary threshold of*P *< 0.001 was used to define Epi, intermediate and Mes cell lines. Breast cancer cell line microarrays and subtype are from GSE16795 (Hollestelle*et al*, 2010). Subtype colour code: blue, Luminal; maroon, Basal.

We first generated EMT signatures specific to bladder, breast, colorectal, gastric, lung and ovarian cancer according to the six-step scheme depicted in Fig1A (see Materials and Methods). First, we curated published EMT signatures (Subramanian*et al*, 2005; Lee*et al*, 2006; Carretero*et al*, 2010) and applied single-sample gene set enrichment analysis (ssGSEA) (Verhaak*et al*, 2013) to provide a gross assessment of the EMT phenotype of each cell line or tumour. An EMT signature that correlated best with known EMT transcripts was next established, and the most Epi and most Mes cell lines or tumours were chosen to build the EMT signature using BinReg (Gatza*et al*, 2010). This BinReg EMT signature was then used to predict the EMT phenotype in cell lines and tumours. The most Epi and most Mes cell lines or tumours were again selected to generate the final EMT signature. Finally, we computed an EMT score of a given sample using a two-sample Kolmogorov–Smirnov test (2KS). Samples with a positive (high) EMT score were more Mes, whereas those with a negative (low) score were more Epi. We developed a cancer-specific EMT signature for tumours and cell lines separately, acknowledging the limitations that cell lines mimic only certain aspects of cancer biology, do not propagate in a stromal microenvironment, and often accumulate additional mutations to survive in artificial culture systems (Borrell, 2010; Gillet*et al*, 2013).

To first ensure the validity of these cancer-specific EMT signatures, we verified our breast cancer-specific EMT signature on the GSE16795 breast cancer cell line data set (Hollestelle*et al*, 2010). EMT scores for breast cancer cell lines with a spindle-like morphology were significantly higher than those for cell lines without a spindle-like morphology (Fig1B;*P *= 1.4E-6); this is consistent with the reported spindle-shaped morphology of Mes cells (Lee & Nelson, 2012). In addition, cell lines with a high EMT score displayed a significantly higher positive staining for VIM and CDH2, known markers of an EMTed phenotype (Thiery*et al*, 2009) (*P *= 2.1E-5 and*P *= 9.1E-6, respectively; Fig1C). Conversely, immunohistochemistry for known Epi markers, CDH1 and CK19, was significantly enriched in Luminal cell lines with a low EMT score (*P *= 0.035 and*P *= 0.005, respectively). Cell lines with an intermediate EMT score were of a mixed Basal–Luminal phenotype, with enriched expression of CK5, a myoepithelial or basal marker (*P *= 0.0002). Basal cell lines had an intermediate-to-high EMT score, whereas Luminal cell lines had a lower EMT score (*P *= 1.6E-7; Fig1C). The bladder cancer-specific EMT signature was validated (Supplementary Text, Supplementary Fig S1), whereas the ovarian cancer-specific EMT signature was already assessed in a previous study (Miow*et al*, 2014). These results corroborate the cancer-specific EMT signature scoring, which forms the basis of the generic EMT signature.

### Generic EMT signature

**Figure 2:**
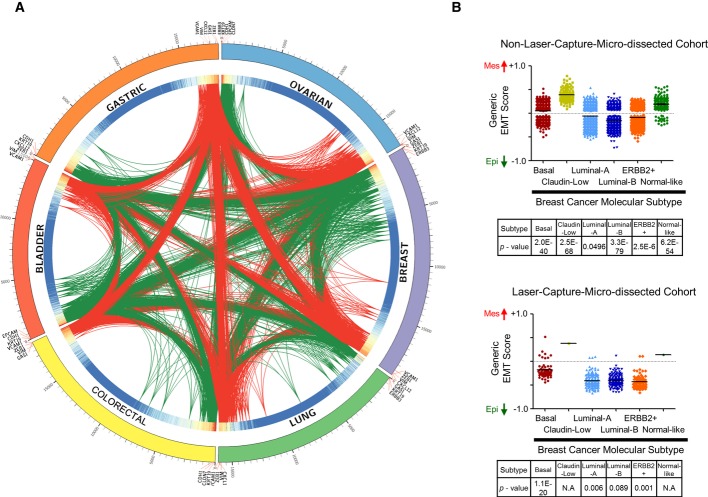
Derivation and application of generic epithelial-mesenchymal transition (EMT) signature Circos plot illustrating the generic EMT signature: the overlap of ovarian (blue), breast (purple), lung (green), colorectal (yellow), bladder (red) and gastric (orange) cancer-specific EMT signatures is shown. Links indicate overlapping genes (red = mesenchymal, green = epithelial). Heatmap on the inner ring indicates weight computed based on Significance Analysis of Microarray (SAM) fold-change, false discovery rate, Receiver Operating Characteristics (ROC) and number of samples of a gene in each cancer-specific EMT signature (red = high, blue = low weight). On the outermost ring, genes are represented by ticks and aligned from the highest SAM fold-change to the lowest for each cancer type. Selected genes are labelled.EMT score (mean ± SEM;*y*-axis) of breast cancer molecular subtypes as predicted using ssGSEA and signature from Prat*et al* (2010) in non-laser-capture micro-dissected (non-LCM) cohort (*n *= 3,992; upper panel) and LCM cohort (*n *= 417; lower panel). The Mann–Whitney*U*-test*P*-value of binary comparison for each subtype is given. Colour code: maroon, Basal; yellow, Claudin-low; light blue, Luminal-A; dark blue, Luminal-B; orange, ERBB2+; green, Normal-like. N.A, not applicable. Note that no*P*-value is available for Claudin-low and Normal-like subtypes in lower panel because*n* < 3.

To quantitatively score any cancer for its EMT status, we derived a generic EMT signature for tumours and cell lines based on the weighted sum of the significance analysis of microarray (SAM) and receiver operating characteristic (ROC) results from each of the cancer-specific EMT signatures (Fig2A; see Material and Methods). Genes that were present in all six of the cancer-specific EMT signatures with a high*z*-transformed weighted sum (*P *< 0.001) were included in the generic EMT signature (Fig2A). As illustrated by the interconnecting links in the heatmap, we noted a high overlap of genes amongst the cancer-specific EMT signatures. A total of 315 genes (Epi: 145, Mes: 170) and 218 genes (Epi: 170, Mes: 48) were selected for tumour and cell line generic EMT signatures, respectively (Supplementary Table S1A and B). Amongst these, 88 Epi and 30 Mes genes were up-regulated in both signatures (Supplementary Table S1A and B). Known EMT transcripts—*CDH1*,*EPCAM*,*GRHL2*,*KRT19*,*RAB25*,*CDH2*,*VIM*,*ZEB1*,*ZEB2*,*SNAI2* and*TWIST1* (Thiery*et al*, 2009; Cieply*et al*, 2012; Huang*et al*, 2012; Zhang*et al*, 2013)—were consistently selected in the generic EMT signature; this successful identification of genes relevant to EMT lends support to the validity of our strategy. Furthermore, the expression of miRNAs reported to suppress EMT, such as those from the miR-200 (miR-200a, miR-200b, miR-200c, miR-141, miR-429) and miR-34 (miR-34a, miR-34b, miR-34c) families (Zhang & Ma, 2012; Hao*et al*, 2014), was significantly and consistently anti-correlated with the generic EMT score (Supplementary Text, Supplementary Fig S2). This suggests the potential to incorporate miRNAs in the generic EMT signature.

Functional annotation analyses on gene ontology and KEGG pathway (Huang da*et al*, 2009) for all 315 genes in the generic EMT signature revealed a significant enrichment in EMT-related biological processes, for example, cell adhesion (FDR* *= 1.2E-9) and cell migration (FDR* *= 6.0E-4; Supplementary Table S2). The generic EMT signature was then compared with published cancer-specific EMT signatures (Supplementary Table S1C). By comparing the enrichment score from ssGSEA, the generic EMT signature was found to strongly correlate with the six cancer-specific EMT signatures that were used for its derivation (*Rho* ∈ [+0.73, +0.97] and with the majority of published cancer-specific EMT signatures (*Rho* ∈ [+0.32, +0.84]; Supplementary Table S1C) for each respective cancer type despite the small overlap in the signature genes. Surprisingly, EMT scores computed from the generic EMT signatures of tumour and cell lines were strongly correlated (*Rho* > +0.89), even though the cell line generic EMT signature does not include stromal components. This indicates that stroma-related genes have a limited influence on the generic EMT score of tumours. We noted, however, that the generic EMT signature had a marginal or no correlation with four of the published EMT signatures, probably due to the small number of genes in these signatures, or because the signature was derived from non-malignant cells. Overall, these results demonstrate the consistency of the generic EMT signature with previously reported EMT-related genes and cancer-specific EMT signatures. Furthermore, the generic EMT signature is both versatile for the quantitation of EMT in all cancer types and not strikingly sensitive to the presence of stroma, two important advantages for this system of classification.

To assess the utility of this generic EMT signature, we computed the EMT scores for laser-capture-micro-dissected (LCM) and non-LCM breast carcinoma (Fig2B). Consistent with previous reports (Blick*et al*, 2008; Taube*et al*, 2010), we observed that Luminal-A, Luminal-B and ERBB2+ breast cancers were more Epi (*P *= 0.0496,*P *= 3.34E-79 and*P *= 2.48E-6, respectively), whereas Basal and Claudin-Low breast cancers were more Mes (*P *= 1.98E-40 and*P *= 2.47E-68, respectively) in both non-LCM and LCM cohorts. Of note, the high similarity between the EMT profiles of breast cancer subtypes in LCM and non-LCM cohorts indicates that EMT scoring is able to capture an overall EMT status of a sample, even in the presence of stroma. To further ensure the validity of the generic EMT signature, we computed the EMT scores for a panel of*in vitro* functional studies across various cancers (Supplementary Fig S3, Supplementary Table S3). In each functional study, the generic EMT score accurately reflected the EMT phenotype regardless of the cancer type (Supplementary Fig S3). For example, consistently higher EMT scores were found for cell lines with*CDH1* or*NOTCH3* knockdown, cell lines treated with TGFβ, and cell lines constitutively expressing EMT inducers,*TWIST1*,*SNAIL*,*GSC*, as compared with control cell lines (Supplementary Fig S3;*P *< 0.05). Conversely, cell lines with over-expressed*GRHL2*—a transcription factor commonly under-expressed in EMTed cells (Cieply*et al*, 2012)—displayed a lower EMT score, indicating a more Epi phenotype. Thus, the EMT score could routinely identify the Epi or Mes phenotype of a cell line under different interventions, which is in full agreement with previous EMT studies (Onder*et al*, 2008; Hellner*et al*, 2009; Malizia*et al*, 2009; Yanagawa*et al*, 2009; Maupin*et al*, 2010; Taube*et al*, 2010; Ohashi*et al*, 2011; Cieply*et al*, 2012; D'Amato*et al*, 2012; Cai*et al*, 2013; Deshiere*et al*, 2013), thus again validating our generic EMT scoring method.

Pancreatic cancer was not included in our original derivation of the EMT signature. As EMT has been implicated in pancreatic cancers, it is important that this generic EMT signature can also accurately estimate the EMT status in pancreatic cancers. We found that the generic EMT score correlates positively with the immunofluorescence staining of EMT markers such as ZEB1, VIM and metastatic ability in various pancreatic cancer cell lines (Supplementary Fig S4, Supplementary Text). The data thus validate the generic EMT signature in pancreatic cancers.

Finally, with the aim of developing a smaller, more cost-effective EMT signature, we explored the possibility of reducing the number of genes in our generic EMT signature (Supplementary Text, Supplementary Fig S5). We identified a 40–50% smaller generic EMT signature that has an overall correlation of 0.85–0.88 with the full generic EMT signature and has good concordance (75.08–95.8%) in estimating EMT status (Supplementary Text, Supplementary Tables S3 and S4A). However, the following analyses continue to use the full generic EMT signature.

### Application of the generic EMT signature

#### A spectrum of EMT is found in multiple cancers

**Figure 3:**
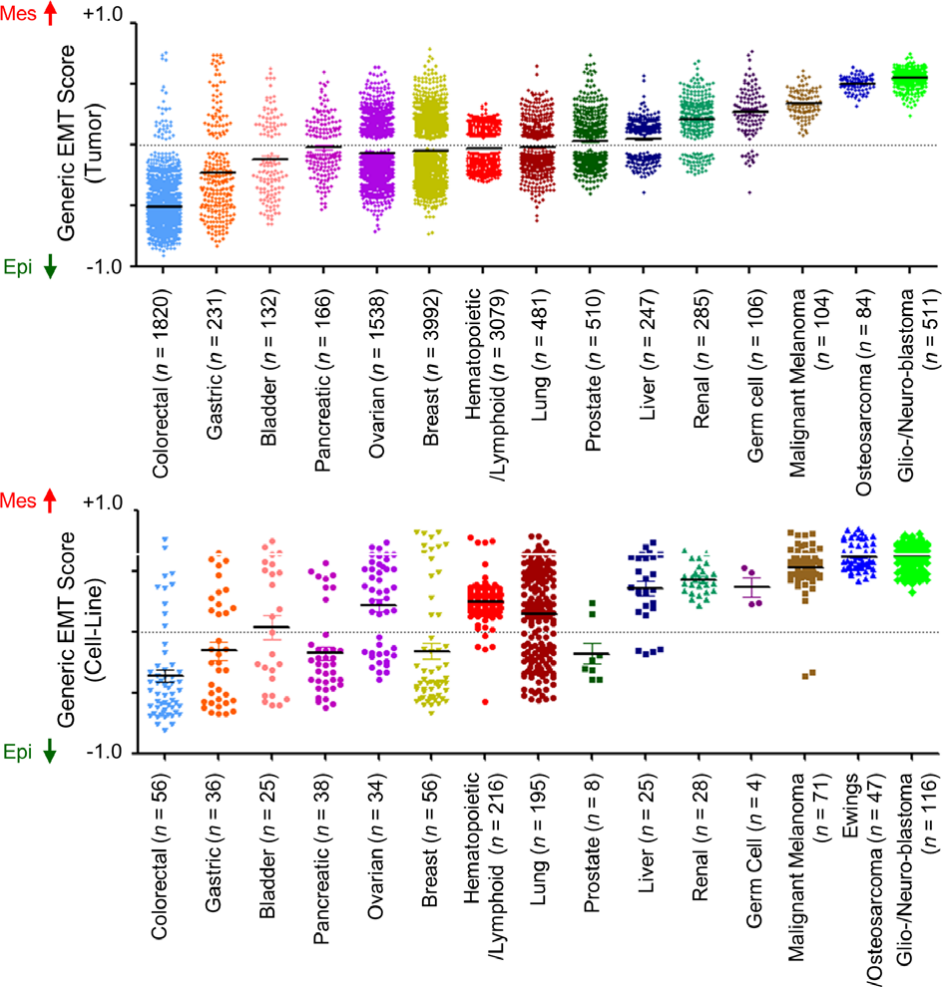
Epithelial-mesenchymal transition (EMT) scores in different cancers types Scatter plot of EMT scores (mean ± SEM;*y*-axis) of various cancers in clinical samples (upper panel) and cell lines (lower panel) sorted by cancer type and mean EMT score. EMT score nearer to +1.0 is more mesenchymal-like (Mes), whereas EMT score nearer to −1.0 is more epithelial-like (Epi). EMT scores of overlapping cell lines in Cancer Cell Line Encyclopedia (CCLE) (Barretina*et al*, 2012) and SANGER/COSMIC (Garnett*et al*, 2012) collections were averaged.

We next performed generic EMT scoring on multiple clinical samples and cell lines (Fig3, Supplementary Fig S6, Supplementary Table S4A–D). A wide range of EMT scores was observed in bladder, breast, gastric, lung, ovarian and prostate cancers. Surprisingly, haematopoietic and lymphoid malignancies, such as lymphoma, acute myeloid leukaemia and multiple myeloma, also displayed a spectrum of EMT scoring, albeit over a narrower range. Colorectal cancer was predominantly Epi (*P *< 1E-50), whereas renal carcinoma exhibited strong Mes features (*P *= 2.47E-53), perhaps reflecting that kidney epithelium derives from the condensation of mesodermal Mes cells. Interestingly, although hepatocytes originate from the primitive Epi endoderm, liver carcinoma displayed an extensive range in EMT score. Other tumours that were primarily Mes included germ cell tumours (*P *= 1.9E-22), malignant melanoma (*P *= 1.38E-42), sarcoma (*P *= 1.7E-34), and glioblastoma and neuroblastoma (*P *< 1E-50). A similar mean and dispersion of the EMT score was seen in cell lines (Fig3), with a wide spectrum noted for cell lines derived from bladder, breast, gastric, liver, lung and prostate carcinoma. Colorectal carcinoma cell lines were predominantly Epi (*P *= 2.61E-17), whereas renal carcinoma (*P *= 7.92E-5), malignant melanoma (*P *= 8.17E-9), sarcoma (*P *= 1.51E-7) and glioblastoma (*P *= 5.67E-19) cell lines were generally Mes, mimicking the observations in tumours. In concordance with clinical samples, germ cell tumour cell lines showed a tendency to be Mes (*P *= 0.58); the lack of significance was presumably because of the limited number of cell lines. Note that the tumours and cell lines in Fig3 were not paired. As a result, the composition of histology, grade, stage of tumours and cell lines are different and that leads to the difference in EMT score distribution, such as is the case in prostate cancer. These results show that each cancer type has a characteristic EMT spectrum.

#### EMT status does not necessarily correlate with poorer survival

**Figure 4:**
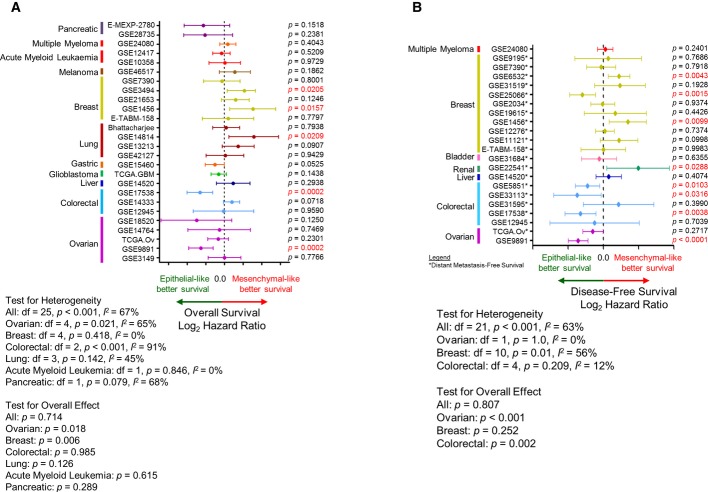
Correlation of Epithelial-mesenchymal transition (EMT) scores and survival A, B Plot of log_2_ hazard ratio (HR; mean ± 95% confidence interval) comparing (A) overall survival (OS) and (B) disease-free survival (DFS) of Epi and Mes tumours in different cancers and cohorts. DFS includes progression-free, recurrence-free and distant metastasis-free survival (cohorts inclusive of distant metastasis-free survival were indicated with *). Corresponding*P*-values from the log-rank test are given next to each cohort, and those with significant differences (*P* < 0.05) are marked red. Log_2_ HR < 0.0 indicates Epi tumours with survival benefit, whereas log_2_ HR > 0.0 indicates Mes tumours with survival benefit. Meta-analysis*P*-value for effect or heterogeneity was computed using DerSimonian–Laird binary random effect (for overall) or Peto fixed effect method (for individual cancer).

To investigate if an EMTed status universally correlates with poor survival, we performed Kaplan–Meier analyses by cohort and by cancer type comparing Epi and Mes tumours (Fig4). Intriguingly, a transitioned status did not universally correlate with overall survival (OS) or disease-free survival (DFS), as shown in the hazard ratio (HR) plots (Fig4). In order to include as many data as possible, we adopted a broad definition of DFS which encompasses progression-free, (local) recurrence-free, and distant recurrence/metastasis-free survival. In general, patients with Epi ovarian cancer (cohort mean HR [μ_HR_] = 0.68,*P *= 0.018), gastric cancer, (μ_HR_* *= 0.7013), pancreatic cancer (μ_HR_* *= 0.6006) and glioblastoma (μ_HR_* *= 0.81) showed better OS. There was no correlation between EMT status and OS for patients with acute myeloid leukaemia, colorectal or lung cancer. Surprisingly, patients with Mes breast cancer (μ_HR_* *= 1.48;*P *= 0.006) and malignant melanoma (μ_HR_* *= 1.48) had better OS (Fig4A), which is in stark contrast with previous reports (Thiery*et al*, 2009; Hrstka*et al*, 2010; Loboda*et al*, 2011; Cieply*et al*, 2012; Huang*et al*, 2012; Byers*et al*, 2013; Frisch*et al*, 2013). Equally intriguing results were observed for DFS (Fig4B), with poorer DFS for patients with ovarian and colorectal cancers (μ_HR_* *= 0.5165,*P *< 0.001; and μ_HR_* *= 0.7669,*P *= 0.002, respectively), and a marginal correlation noted for patients with bladder carcinoma (μ_HR_* *= 0.8473). For liver and renal carcinoma, patients with Mes tumours had better DFS than their Epi counterparts (μ_HR_* *= 1.238 and 3.948, respectively). The result for DFS in patients with breast cancer was unclear (HR* *= 0.4432–2.622;*P *= 0.252). Overall, the EMT status is unlikely to be the sole prognostic factor for survival where the composition of histotype or molecular subtype may play a role; this suggests the requirement for stratification of cancers in addition to deciphering the EMT status. This is exemplified by the stratification of breast cancer molecular subtypes (Prat & Perou, 2011), where there is a correlation for better DFS for patients with Epi breast cancers that are of a Basal and Claudin-Low subtypes, but no correlation for other subtypes (Supplementary Fig S7). However, this correlation of EMT and DFS in Basal and Claudin-Low subtypes was not coherent in all breast cancer cohorts probably due to small sample sizes.

#### EMT status does not necessarily translate to chemotherapeutic resistance

**Figure 5:**
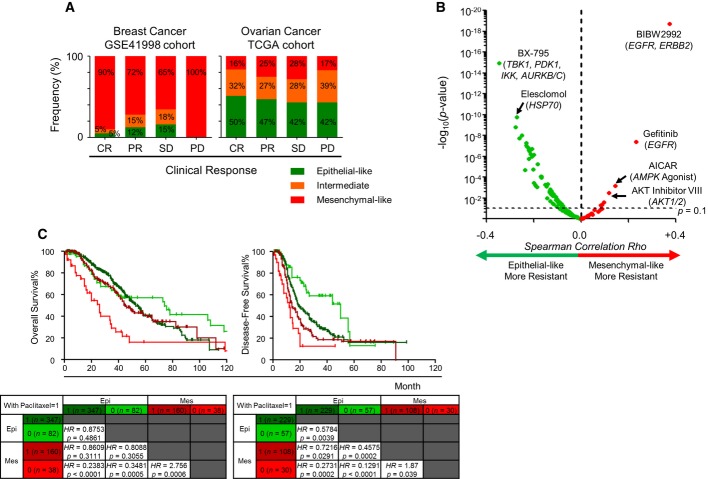
Generic epithelial-mesenchymal transition (EMT) and drug sensitivity Bar plots of breast (*n *= 270; left panel) and ovarian (*n *= 328; right panel) cancers stratified by EMT status and clinical response based on response evaluation criteria in solid tumours (RECIST). Regimen was neoadjuvant doxorubicin and cyclophosphamide for breast cancer, and platinum-based adjuvant/progression/recurrence chemotherapy for ovarian cancer. Percentage distribution of EMT status is given in each clinical response group. Abbreviation: CR, complete response; PR, partial response; SD, stable disease; PD, progressive disease. Green, epithelial-like (Epi); orange, intermediate; red, mesenchymal-like (Mes).Volcano plot of EMT correlation with drug sensitivity regardless of cancer type.*Rho* ∈ [−1.0, +1.0] (*x*-axis) and*-*log_10_*P*-value (*y*-axis) were computed by Spearman's correlation coefficient test. Dashed line of*P*-value = 0.1 is plotted. Red and green indicate higher drug resistance in Mes tumours (*Rho* ∈ [0, +1.0]) and Epi tumours (*Rho* ∈ [−1.0, 0]), respectively.Kaplan–Meier analysis comparing overall survival (left panel) and disease-free survival (right panel) of Epi (green) and Mes (red) ovarian cancer patients who underwent a treatment regimen with (dark colour) or without (light colour) paclitaxel.*P*-value reported was computed by log-rank test. Abbreviation: HR = hazard ratio.

To investigate the association between EMT and chemotherapeutic resistance, we compared the clinical outcomes of patients using the response evaluation criteria in solid tumours (RECIST) available for breast (Horak*et al*, 2013) and ovarian cancer (The Cancer Genome Atlas Research, 2011) cohorts (Fig5A). In these cohorts, patients with breast cancer had been treated with sequential neoadjuvant therapy (doxorubicin and cyclophosphamide), whereas patients with ovarian cancer had undergone primarily platinum-based therapy. Without considering the treatment regimen, there was no significant difference between the RECIST groups in terms of EMT score. Thus, we categorized tumours into Epi, intermediate and Mes based on 2KS (*P *< 0.05) and observed an enrichment of Mes breast cancers in the progressive disease (PD) category (*P *= 0.3303). Analyses with another 11 breast cancer cohorts (GSE48905, GSE33658, GSE23428, GSE22226, GSE18864, GSE28796, GSE16646, GSE22513, GSE4779, GSE18728 and GSE50948) (Farmer*et al*, 2009; Bauer*et al*, 2010; Korde*et al*, 2010; Silver*et al*, 2010; Lehmann*et al*, 2011; Massarweh*et al*, 2011; Carey*et al*, 2012; Esserman*et al*, 2012; Evans*et al*, 2012; Knudsen*et al*, 2014; Prat*et al*, 2014), within which patients had been administered with different neoadjuvant treatment regimens, including fulvestrant, anastrazole, carboplatin, doxorubicin and other drugs (Supplementary Fig S8A), showed a similar distribution of Epi, intermediate and Mes breast cancers in each clinical response group. Notably, the worst response group (PD or residual disease) comprised mostly patients with Mes breast cancers. Thus, there was a trend towards either an increasing proportion of Mes or a decreasing proportion of Epi breast cancers amongst chemo-resistant patients. We also noted a trend towards a decrease in the Epi proportion amongst patients with ovarian cancer and a change from complete response (CR) to PD (50–42%), albeit there was no significant enrichment in PD for patients with Mes ovarian cancers (*P *= 0.556).

Since the distribution of the EMT score did not allow us to segregate certain other cancers into Epi, intermediate and Mes groups, we next investigated the EMT score profiles of responders and non-responders in these cancers (Supplementary Fig S8B). This was performed using cohorts of predominantly Epi colorectal cancers (GSE19862, GSE35452, GSE46862) (Gim*et al*, 2014), a cohort of head and neck cancers (GSE32877) (Tomkiewicz*et al*, 2012) and a cohort of predominantly Mes melanoma (GSE22968) (Beasley*et al*, 2011). There was no significant difference between responders and non-responders in terms of EMT score, albeit there was a slight trend towards responders tending to have a higher EMT score in predominantly Epi colorectal cancer, and a slight trend that responders tended to have lower EMT score in predominantly Mes melanoma (Supplementary Fig S8B). These data suggest that EMT may correlate with chemotherapeutic resistance; however, the results remain inconclusive at this time. The lack of a conclusive result might be because the majority of these patients had been treated with more than one chemotherapeutic compound, which may confound the role of EMT and chemotherapeutic resistance in these patients. Furthermore, as these data are from relatively small cohorts, further study is required to validate the current observations.

Thus, to search for an association between EMT and chemotherapeutics, and to explore the potential therapeutic options for Epi and Mes cancers, we analysed drug sensitivity data from the SANGER/COSMIC (Garnett*et al*, 2012) database (April 16, 2013) in cell line models. We correlated the EMT score with the half-maximal inhibitory concentration (IC_50_) of 138 compounds (Fig5B, Supplementary Fig S9, Supplementary Table S5) using the Spearman's correlation coefficient test, as it measures the overall trend and requires no definition of sensitive or resistant categories. We employed a less stringent threshold (*P *< 0.1) because of the limited samples for certain drugs. Surprisingly, the EMT status did not systematically translate to cellular chemotherapeutic resistance (Fig5B, Supplementary Fig S9), contradicting previous associations between cellular phenotype and attaining resistance (Witta*et al*, 2006; Arumugam*et al*, 2009; Hrstka*et al*, 2010; Sethi*et al*, 2010; Marchini*et al*, 2013). Regardless of the cancer type, Mes and Epi cell lines were preferentially sensitive to certain compounds. Mes cell lines were more resistant to Afatinib and Gefinitib (against*EGFR*), but were more sensitive to the PDK1 kinase inhibitor, BX-795 and the HSP-90 inhibitor, Elesclomol (Fig5B). Intriguingly, Epi cell lines were resistant to 64 compounds, whereas Mes cell lines were resistant to only 7, albeit the correlation was weak (*Rho* ∈ [−0.35, +0.37]). When stratified by cancer type, we observed a similar pattern of preferential sensitivity to certain compounds. Notably, Mes pancreatic cancer, malignant melanoma, renal cancer and liver cancer cell lines were more sensitive to compounds targeting microtubule dynamics, such as Vinblastine and Docetaxel. Comparatively, Mes breast, lung and uterine cancer cell lines were more resistant to Afatinib and Gefinitib (Supplementary Fig S9). Previous observations reported that EMT is associated with*EGFR* inhibitor resistance in non-small cell lung cancer (NSCLC) (Byers*et al*, 2013). Using our generic EMT score, we observed that Epi cell lines not limited to NSCLC were more sensitive to inhibitors of*EGFR* or both*EGFR* and*ERBB2* (Erlotinib, Lapatinib, BIBW2992 and Gefitinib) in the SANGER/COSMIC (Garnett*et al*, 2012) and Cancer Cell Line Encyclopedia (CCLE) (Barretina*et al*, 2012) databases (Supplementary Table S5). Cell lines with sensitizing*EGFR* activating mutations (L861Q, G719S, exon 19 in-frame deletion) (Carr*et al*, 2004) exhibited significantly lower EMT scores compared with wild-type cell lines (*P *= 0.0056). On the other hand, cell lines harbouring the secondary gatekeeper*EGFR*-T790M mutation, which confers resistance to*EGFR* inhibitors (Gazdar, 2009), were more Mes (EMT score = +0.23). Hence, a higher prevalence of sensitizing*EGFR* mutations could account for the higher response rate of Epi cancers to*EGFR* inhibitors. Although it is too preliminary to conclude if Epi or Mes is resistant to certain compounds (due to the modest correlation and*P*-values), these results suggest that Epi and Mes cell lines have differential responses to certain compounds. In addition, we show that Epi and Mes cell lines also have preferential responses to certain compounds and that EMT is not the only mechanism driving resistance in all chemo- or targeted therapies.

These intriguing preferential drug sensitivities of Epi and Mes cancers in the correlation analysis of EMT score and the IC_50_ of 138 compounds (EMT score–IC_50_; Supplementary Table S5) prompted us to investigate the relevance of these findings, as cell lines do not fully exemplify the behaviours of primary tumours. We selected ovarian cancer as a model for this pilot study because the first-line treatment for ovarian cancer is primarily cisplatin/carboplatin and paclitaxel, which could provide a less convoluted mechanism of interaction between EMT and the drugs. Provocatively, in the EMT score–IC_50_ correlation analysis, we found that Mes ovarian cancers have preferential sensitivity to cisplatin (*Rho* = −0.37) and paclitaxel (*Rho* = −1.0; Supplementary Table S5). A Kaplan–Meier analysis was then performed to stratify the treatment regimens into Epi versus Mes ovarian cancer patients (Fig5C); this was performed using the 2KS criteria described for Fig5A. Because few patients were treated without cis-/carboplatin (*n *< 10), we focused our analysis on the effect of paclitaxel. Surprisingly, patients with Epi and Mes ovarian cancers, who had received a regimen with paclitaxel, had significantly different OS and DFS outcomes as compared with those who had received a regimen without paclitaxel (Fig5C). Epi patients receiving a regimen containing paclitaxel showed poorer DFS (*P *= 0.0039), whereas Mes patients treated with paclitaxel showed better DFS (*P *= 0.039) and OS (*P *= 0.0006); these results for the Mes patients mirrored those garnered from the EMT score–IC_50_ analysis, indicating that Mes is more sensitive to paclitaxel. We found no significant difference in DFS or OS outcome for ovarian cancer patients who exhibited an intermediate EMTed phenotype. Such differential therapeutic response in Epi and Mes tumours is also observed in glioma (Desmedt*et al*, 2009) and multiple myeloma (Erdem-Eraslan*et al*, 2013) (Supplementary Fig S10, Supplementary Information). Even though glioma patients receiving radiotherapy and chemotherapy generally have better OS, the benefit is greater in patients with Mes glioma (*P *= 0.0117). In contrast, patients with Epi multiple myeloma have better DFS rates when administered with bortezomib instead of dexamethasone (*P *= 0.0349). However, we observed no difference in patients with ER+ breast cancers (Mulligan*et al*, 2007) who were administered with letrozole or tamoxifen in terms of EMT stratification. Overall, these results provide*in vivo* evidence for the findings of the EMT score–IC_50_ correlation analysis and show the preferential drug sensitivity in patients with Epi and Mes tumours as well as their differential responses to particular chemotherapeutic regimens.

## Discussion

Increasing evidence points to the role of EMT in cancer progression, metastasis and drug resistance. However, the difficulty in making an adequate assessment of EMT in tumours has caused dispute as to whether EMT exists in cancer (Jordan*et al*, 2011). To address this issue, we developed a generic EMT signature to quantitatively estimate the extent of EMT in tumours and cell lines. To the best of our knowledge, this is the first time a generic EMT signature has been sought in order to capture the universal features of EMT in tumours or in cells. Previous reports indicate that intermediate states of EMT display the highest plasticity (Jordan*et al*, 2011; Huang*et al*, 2013) and thus represent an appropriate stage within which to induce or reverse EMT. The change in EMT score captures and reflects this phenotypic transition in the cell or tissue; this method is judiciously illustrated in a previous application where Epi MCF7 breast cancer cells displayed a shift in the EMT spectrum when transfected with*SNAI1* (Akalay*et al*, 2013). Having the capacity to monitor such a transition would be instrumental for assessing the effectiveness of EMT reversion therapy and for identifying an intermediate state of EMT that would have an improved chemotherapeutic response. It is important to note that full reversion of EMT may not be desirable, as Mes micro-metastases must re-acquire an Epi phenotype to proliferate at the metastatic site (Thiery, 2002); in agreement was the recent demonstration that reversing EMT may promote metastatic colonization (Tsai*et al*, 2012). The main challenge, therefore, is that we do not know the precise intermediate states and under what conditions or context cancer cells in the primary tumour or at the distant metastatic sites can exit dormancy and resume growth or become chemo-resistant. Here, we validated the efficacy of our generic EMT scoring system to reflect the EMT transition in a panel of functional studies across multiple cancer types. The spectrum of EMT identified across the various tumours implies a causal role of the EMT status in the differential characteristics of these cancers and in their responses to treatment. Remarkably, the EMT spectrum was highly similar between cell lines and tumours in a given cancer type, which verifies the capacity of the EMT score to capture the EMT phenotype rather than the influence of the stroma.

The type of cancer is generally considered to be a good indicator of the EMT status (Fig3). For example, colorectal carcinoma is primarily Epi, whereas glioblastoma, neuroblastoma, osteosarcoma, malignant melanoma and germ cell tumours are primarily Mes. It is unclear, however, whether these phenotypic traits are inherent or acquired. Inherent EMT traits could be a reflection of the cell of origin or the lineage of the cancer. Indeed, melanoma and neuroblastoma are derived from transformed melanocytes and sympathetic neural progenitor cells, respectively (Nakaya & Sheng, 2013), which originate from the neural crest and delaminate through an EMT before colonizing different embryonic sites where they undergo differentiation into melanocytes, glial cells and neurons of the peripheral nervous system. Thus, these neural crest cell derivatives maintain an intrinsic Mes phenotype. Another example is found in breast cancer. The most EMTed breast cancers belong to the Claudin-Low subtype and are likely derived from the highly plastic cells of the basal layer of the mammary gland; the less plastic luminal cells, in contrast, are thought to give rise to the Basal subtype (Taddei*et al*, 2008; Lim*et al*, 2009; Molyneux*et al*, 2010) (Fig2B). In the case of acquired EMT, the process may be triggered by changes in the tumour microenvironment (Valastyan & Weinberg, 2011; Lee & Nelson, 2012; Tam & Weinberg, 2013; Van den Eynden*et al*, 2013) or the influence of drug treatment or cytotoxic stress (Frisch*et al*, 2013; Marchini*et al*, 2013), amongst other factors. This is exemplified in pancreatic carcinoma, which derives from the same endodermal anlage as the colon yet exhibits a relatively Mes phenotype as compared with colon carcinoma. Although pancreatic carcinoma comprises a large fraction of stromal cells (Beatty*et al*, 2011), pancreatic carcinoma cell lines exhibit the same EMT spectrum as colon cells (Fig3), supporting the notion that the EMT score still arises from the contributions of the pancreatic carcinoma cells not just the stromal cells. In a similar way, liver carcinoma shows a wide spectrum of EMT scores. As the liver also derives from the primitive endoderm, it would be expected that liver carcinoma would exhibit an Epi phenotype. In this case, in addition to the role of the stroma, it is intriguing to consider that the cell of origin may have undergone an E- to N-cadherin switch.

Although many reports have associated the EMT status with survival (Witta*et al*, 2006; Arumugam*et al*, 2009; Hrstka*et al*, 2010; Sethi*et al*, 2010; Loboda*et al*, 2011; Cieply*et al*, 2012; Byers*et al*, 2013; Marchini*et al*, 2013), our EMT scoring does not wholly support these findings. We show that the EMT status is linked to OS in ovarian cancer, gastric cancer and glioblastoma, but not in other carcinoma types. In terms of DFS, patients with Epi ovarian and colorectal cancers have a better prognosis. The discrepancy in the reported correlations between EMT status and survival is intriguing, as EMT was posited to be involved in cancer progression, metastasis and drug resistance, all of which are strongly connected with poorer survival. It is noteworthy that most breast carcinoma of the lobular histotype, which are notoriously known for not expressing E-cadherin, are not more aggressive than E-cadherin-positive invasive ductal carcinoma (Ferlicot*et al*, 2004). Here, we also showed that patients with Mes breast cancers appear to have better OS and DFS than those with Epi breast cancers, seemingly in opposition to what has been previously reported (Hrstka*et al*, 2010; Taube*et al*, 2010; Cai*et al*, 2013). On closer look, this difference likely arises from the distribution of patients with luminal and triple-negative breast cancers in the cohort. As shown in Fig4B, a breast cancer cohort (GSE25066) with a lower percentage of Luminal-B and ERBB2+ breast cancers would show a better DFS for Epi breast cancers. Even though Luminal-B and ERBB2+ breast cancer subtypes are of the Epi type (Blick*et al*, 2008) (Fig2B), they have poor OS and DFS, similar to that of the Mes type, triple-negative breast cancers (Prat & Perou, 2011; Ishitobi*et al*, 2013). Consequently, the more prevalent Epi Luminal-B and ERBB2+ breast cancers give rise to poorer survival curves in Epi breast cancer cohorts, suggesting that heterogeneity within a cancer type could mask and perplex the role of EMT. Thus, stratification by molecular subtypes may be required to study the role of EMT. Indeed, when stratified by breast cancer molecular subtype (Prat & Perou, 2011), patients with Epi breast cancers show better DFS if their cancers are of the Basal and Claudin-Low subtypes, but not the other subtypes (Supplementary Fig S7).

The crosstalk between stromal and cancer cells plays a major role in metastasis (Park*et al*, 2011) and hence may influence the results of EMT scoring. In breast cancer, mammary Epi cells can adopt a stromal gene expression pattern indistinguishable from reactive stroma when undergoing EMT (Farmer*et al*, 2009). As there is no distinction between reactive stroma and EMT-induced stromal expression, this may generate a high EMT score in some Epi tumours. The EMT scores of LCM and non-LCM breast cancer cohorts (Fig2B) showed a marginally lower EMT score for Epi, Luminal-A subtype, suggesting that stromal contribution may, to some extent, obscure a precise assessment of the EMT score. However, assessing the stromal contribution is non-trivial given the RNA instability and labour-intensive procedure of segregating stromal from cancer cells (Park*et al*, 2011). It is therefore difficult to quantify the influence of stroma in our EMT scoring. Nevertheless, in addition to the minute EMT score differences in LCM and non-LCM breast cancer cohorts, we have also shown a strong correlation of the generic EMT score computed using tumour- and cell line-specific signatures (Supplementary Table S1C). This result indicates that whereas stroma may obscure a precise assessment of EMT by transcriptome, the influence is not overwhelmingly striking. Thus, we believe the EMT scoring is relatively independent of stromal influence, but likely not of clonal heterogeneity. Others have shown that, although there is a higher proportion of EMT carcinoma cells in basal-like tumours, such cells are also seen in luminal breast tumours (Sarrio*et al*, 2008). It would thus be useful to analyse the phenotype and clonogenicity of these EMTed cells and of CTCs in addition to EMT scoring (Thiery & Lim, 2013). On-going studies on CTCs in our laboratory are exploring whether the EMT score reflects the propensity of a cancer to disseminate and become refractory to therapy. CTCs exhibit a wide spectrum of EMT phenotypes, irrespective of the primary tumour (Valastyan & Weinberg, 2011; Thiery & Lim, 2013; Yu*et al*, 2013). Thus, the capacity of a primary tumour to metastasize may reside in a small subset of cells, the phenotype of which is not known and cannot be assessed by an EMT scoring method.

Our findings are apparently discrepant with previous connections between EMT status and drug resistance (Witta*et al*, 2006; Arumugam*et al*, 2009; Hrstka*et al*, 2010; Sethi*et al*, 2010; Marchini*et al*, 2013). Whilst we acknowledge the limitations of cell lines and IC_50_ as a drug assay (Haibe-Kains*et al*, 2013), we believe our results give a bird's-eye view of EMT and drug resistance. By assessing OS and DFS outcomes of ovarian cancer patients through EMT status and treatment regimen, we found that Epi ovarian cancers are more resistant to paclitaxel, whereas Mes ovarian cancers have a preferential sensitivity to paclitaxel. This shows that cancers with different degrees of EMT respond distinctly to particular compounds—in accordance with our previous work in ovarian cancer (Miow*et al*, 2014)—and is supportive of the utility of the EMT score-IC_50_ correlation analysis in cell lines. More importantly, these results identify that patients with Mes, but not Epi, ovarian cancer would benefit from therapeutic regimens that contain paclitaxel. In line with this, the Japanese Gynecologic Oncology Group demonstrated a survival advantage for a weekly administration of paclitaxel compared with a once in 3 week administration of paclitaxel in combination with carboplatin in relapsed patients with ovarian cancer (Baird*et al*, 2010), where relapsed ovarian cancer is shown to be enriched for Mes (Tan*et al*, 2013). Similar to our findings, gastric cancer patients, who have an enrichment for Mes, respond differently to chemotherapy from the subtype of patients not enriched for Mes and are more sensitive to cisplatin (Tan*et al*, 2011). Overall, our data indicate that not all Mes carcinoma are resistant to chemotherapy and that the EMT status does not necessarily translate to a propensity towards drug resistance. Indeed, testicular carcinoma, a highly Mes, germ cell tumour, is extraordinarily sensitive to cisplatin (Masters & Koberle, 2003; Eckstein, 2011). Furthermore, even though EMT is often linked with the acquisition of stem cell-like features,*Prrx1* uncouples EMT and stemness, resulting in a drug-resistant, metastatic colonization (Ocana*et al*, 2012). Thus, we postulate that it is not solely the acquisition of EMT but the EMT stem cell-like phenotype that engenders drug resistance (Brabletz, 2012). Frisch*et al* (2013) proposed a similar concept, suggesting that EMT is acquired by triggering EMT inducers to repress cell polarity and that stem cell-like features are acquired by engaging additional programs such as the WNT and Hippo pathways. It is likely that the present generic EMT signature estimates the degree of EMT but cannot estimate the degree or behaviour of a cancer stem cell-like phenotype. This distinction is evident in the minute differences between control and*HMGA2*-knockdown—a gene implicated in stemness (Copley*et al*, 2013)—MDA-MB-231 breast cancer cells (Supplementary Fig S3) and may explain the limited correlation between generic EMT score and therapeutic resistance, as cancer stem cells may have an impact on tumour progression in breast (Sarrio*et al*, 2008) and colon (Brabletz, 2012) Epi tumours. In our preliminary analysis, although there are some moderate correlations between stemness and generic EMT score, the correlation was not consistent across cancer types, which may suggest that different cancers enrol distinct programs to acquire stemness (Supplementary Text, Supplementary Fig S11). In addition, the existence of different types of stem cells within a cancer—as shown in breast cancers—has to be taken into account when considering the correlation of stemness and EMT (Liu*et al*, 2014). Finally, the lower sensitivity of Mes cell lines to various compounds (*EGFR* inhibitors) may be due to a lower prevalence of the targeted mutations in these cell lines. However, it is still unknown whether an*EGFR* mutation is the main driver in these cancers and whether these mutations—acquired or inherent—play a role in initiating or regulating EMT.

Overall, we demonstrate the feasibility of applying a generic EMT score for the examination of the EMT spectrum in different cancers, as well as its correlation with survival and chemotherapeutic resistance. We believe the proposed generic EMT score is a promising, general-purpose tool with which to estimate EMT phenotypes, regardless of cancer type, to systematically investigate EMT and to more objectively assess the impact of EMT effectors or drugs on phenotype changes. It also offers a more objective EMT scoring*in vitro* as opposed to estimations by visual inspection or marker assessment.

## Materials and Methods

### Data pre-processing for Affymetrix microarray expression data

Pre-processing and quality checks were performed as described (Tan*et al*, 2013) (Supplementary Materials and Methods). Data sets on the Affymetrix U133A or U133Plus2 platforms for bladder (*n *= 132), breast (*n *= 3992), colorectal (*n *= 1820), gastric (*n *= 231) and ovarian (*n *= 1538) cancers, as well as NSCLC and lung adenocarcinoma (*n *= 481) were downloaded from Gene Expression Omnibus (GEO), Array Express, Expression Project for Oncology (ExpO) and The Cancer Genome Atlas (TCGA) (Supplementary Table S6). An LCM breast cancer cohort (*n *= 417) was provided by the Japanese Foundation for Cancer Research (http://www.jfcr.or.jp/english; Supplementary Materials and Methods; GSE54002). Normalization was performed independently on each cohort using R version 3.01, Bioconductor Affy Package 1.38.1, Robust Multichip Average (Gautier*et al*, 2004), and ComBat (Johnson*et al*, 2007) was applied for batch adjustment on the compiled, normalized data sets separately. Normal tissues were removed from the batch-adjusted data. Cell line collections (Supplementary Table S7), including SANGER/COSMIC (Garnett*et al*, 2012), CCLE (Barretina*et al*, 2012) data sets and validation data sets (Supplementary Table S8), were subjected to the same normalization procedure.

### Predictive modelling and validation by BinReg

Expression data analysis based on a binary regression model using the BinReg v2.0 (Profiler, http://dig.genome.duke.edu/software.html) was described previously (Gatza*et al*, 2010; Tan*et al*, 2013). Details are given in the Supplementary Materials and Methods.

### Generation of cancer-specific EMT signature

Aside from an ovarian- and breast cancer-specific EMT signatures, which we derived previously from CDH2 and CDH1 immunofluorescence staining (Akalay*et al*, 2013; Miow*et al*, 2014), we devised a strategy to generate cancer-specific EMT signatures for the other types of cancer (bladder, colorectal, gastric and lung), as depicted by the six-step scheme in Fig1A:

1. Published EMT gene sets from Molecular Signature database v4.0 (Subramanian*et al*, 2005) and previous literature (Lee*et al*, 2006; Carretero*et al*, 2010) (Supplementary Table S9) were collated.
2. ssGSEA score (Verhaak*et al*, 2013) was computed for EMT gene sets on cancer cell lines and correlated with gene expression of known Mes and Epi markers (*TWIST1*,*SNAI1*,*SNAI2*,*VIM*,*CDH2*,*ZEB1* and*CDH1*,*DDR1*,*ERBB2*,*ERBB3*,*KRT19*) (Thiery*et al*, 2009).
3. The gene set that best correlated with the enrichment score was chosen to rank the cell lines. The 10–20 most Mes and most Epi cell lines were selected for BinReg modelling. Two data sets, GSE9691 (Onder*et al*, 2008) and GSE24202 (Taube*et al*, 2010), were used for BinReg parameter settings and to ensure validity of the derived EMT signature. (Note: Steps 1–3 can be recursive to identify an initial BinReg EMT signature of sufficient accuracy in predicting the EMT status.)
4. The BinReg EMT signature was then used to predict the EMT status of cell lines and tumour samples specific to a particular cancer type.
5. The extreme 25% of the most Mes and Epi cell lines or the extreme 100 Mes and Epi tumours were chosen to generate the EMT signatures for cell lines and tumours, respectively; this prevented the signature from over-fitting the training data. EMT signatures were generated using SAM/ROC (Tusher*et al*, 2001; Verhaak*et al*, 2013), with applied thresholds of: SAM*q*% = 0, and ROC > 0.8–0.85 or < 0.15–0.2.
6. Using this SAM/ROC-derived EMT signature, we then computed the EMT score of a sample using a two-sample Kolmogorov–Smirnov test (2KS).

The final cancer-specific EMT signature (generated by SAM/ROC) is a refinement of the initial EMT signature (generated by BinReg). Although it seems redundant to have an initial followed by a refined final EMT signature, the benefit of this approach is threefold. First, since some of the collected, published EMT signatures are derived from different cell types and from a relatively smaller number of cell lines, these published EMT signatures may not be applicable universally, as they may be cell line-specific or cancer-specific. In this study, we used a large panel of cell lines to derive an EMT signature specific to each cancer type, and hence, it is less likely that the derived signature contains features unique to a single cell line. Second, to ensure accuracy of the final EMT signature, we validated the initial EMT signature on two independent functional EMT studies. Third, regenerating the EMT signature by SAM/ROC from the most Epi or most Mes tumours ensured the additional changes sometimes acquired in cell lines would not be included and distort the EMT signature for tumours in general.

### Derivation of generic EMT signature

We derived a generic EMT signature from the overlap between specific EMT signatures generated for bladder, breast, colorectal, gastric, lung and ovarian cancer types. We weighted the genes that were selected in six cancer-specific EMT signatures using the formula: for gene*g*, the weight of the gene is given by:


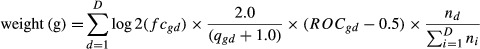


where*D* is the total number of diseases (*D *= 6 in this case),*fc*_*gd*_ and*q*_*gd*_ are the fold-change and*q*-value% of the gene,*g*, of disease,*d*, as computed by SAM.*ROC*_*gd*_ is the ROC value of gene,*g*, of disease,*d*, and*n*_*d*_ is the number of samples in disease,*d*. The formula will give higher weights to genes that have a large fold-change, a small*q*-value%, a large ROC value and a large number of samples. We ranked and selected the genes with a*z*-transformed weight > 3.09 or*P *< 0.001 (Supplementary Table S1A and B).

### Computation of EMT score

To compute the EMT score of a sample, we adopted a similar approach to that used in ssGSEA (Verhaak*et al*, 2013). The empirical cumulative distribution function (ECDF) was estimated for Epi and Mes gene sets. The 2KS test was employed to compute the difference between Mes ECDF (ECDF_Mes_) and Epi ECDF (ECDF_Epi_). The 2KS score was then taken as the EMT score. A sample with a positive EMT score exhibits a more Mes phenotype, whereas a negative EMT score reflects a more Epi phenotype. Note that the 2KS test allows segregation of samples into Epi (2KS score ECDF_Epi_ > ECDF_Mes_;*P *< 0.05), intermediate Epi (2KS score ECDF_Epi_ > ECDF_Mes_;*P* ≥ 0.05), intermediate Mes (2KS score ECDF_Epi_ < ECDF_Mes_,*P* ≥ 0.05) and Mes (2KS score ECDF_Epi_ < ECDF_Mes_,*P *< 0.05). The EMT signature is given in Supplementary Table S1A and B. The Matlab® R2012a script for computing the EMT score and computation of the EMT score can be requested through http://www.csi.nus.edu.sg/bioinfo/index.php.

**The paper explained**

**Problem**

During epithelial-mesenchymal transition (EMT), epithelial cells lose polarity and acquire migratory properties reminiscent of mesenchymal cells. EMT is a dynamic process, not a binary process, with intermediary states, and is thus still not easily ascertained in cultures or*in vivo*. Consequently, its role in cancer remains controversial.

**Results**

We used gene expression to establish an EMT scoring method and quantitatively estimated the degree of EMT (−1.0 to +1.0) in a large collection of cell lines and tumours reflecting epithelial and mesenchymal states as well as the potential intermediate states that occur during transition.

**Impact**

We applied EMT scoring to ascertain its efficacy in correlating EMT status with patient survival rates and responses to treatment. Such versatile EMT scoring may enable the objective and systematic investigation of EMT across many parameters of cancer progression, survival and throughout the clinical response to therapy.

### Statistical analysis

DerSimonian–Laird binary random or Peto fixed effect meta-analysis was conducted using OpenMeta[Analyst] software with the default settings. The log-rank test in the Kaplan–Meier analyses was computed by GraphPad Prism® version 5.0 (GraphPad Software, La Jolla, CA). Mann–Whitney, Fisher's exact and Spearman's correlation coefficient tests were computed by Matlab® R2012a, statistics toolbox version 8.0 (MathWorks, Natick, MA).

## For more information

Gene Expression Omnibus (GEO): http://www.ncbi.nlm.nih.gov/gds

ArrayExpress: http://www.ebi.ac.uk/arrayexpress/

The Cancer Genome Atlas (TCGA): https://tcga-data.nci.nih.gov/tcga/

Molecular Signature Database (MSigDB): http://www.broadinstitute.org/gsea/msigdb/index.jsp

Cancer Cell Line Encyclopedia (CCLE): http://www.broadinstitute.org/ccle/home

SANGER COSMIC cell line: http://cancer.sanger.ac.uk/cancergenome/projects/cell_lines/

Compute Generic EMT score: http://www.csi.nus.edu.sg/bioinfo/index.php

BinReg/Profiler software: http://dig.genome.duke.edu/software.html

DAVID functional annotation tool: http://david.abcc.ncifcrf.gov/summary.jsp
