# Supplementary material for: Epithelial-mesenchymal transition spectrum quantification and its efficacy in deciphering survival and drug responses of cancer patients
Source: EMBO Mol Med. 2014 Sep 11;6(10):1279–93. doi: 10.15252/emmm.201404208 (PMC4287932; doi:10.15252/emmm.201404208)
Supplement: Supplementary file 1 [file emmm0006-1279-sd1.pdf]

Epithelial-Mesenchymal Transition Spectrum Quantification and its Efficacy in Deciphering Survival and Drug Responses of Cancer Patients

Tuan Zea Tan, Qing Hao Miow, Yoshio Miki, Tetsuo Noda, Seiichi Mori, Ruby Yun-Ju Huang*, Jean Paul Thiery*

*Co-senior authors

Table of Contents

Supplementary Text 2

Verifying bladder cancer-specific EMT signature 2

Verifying Generic EMT signature on Pancreatic Cancer 3

Generic EMT signature and miRNA 3

Effect of reducing the number of genes in the generic EMT signature 4

Generic EMT signature and stemness 5

Supplementary Figure Legends 7

Figure S1: Validation of bladder-specific EMT signature 7

Figure S2: Correlation of generic EMT signature with miRNA implicated in EMT. 8

Figure S3: Validation of generic epithelial-mesenchymal transition (EMT) signature. 9

Figure S4: Validation of generic EMT signature on pancreatic cancer cell lines. 10

Figure S5: Effect of reducing genes in generic EMT signature. 11

Figure S6: Epithelial-mesenchymal transition (EMT) spectrum in different cell lines. 12

Figure S7. Correlation of Epithelial-Mesenchymal Transition and survival in breast cancer subtypes. 13

Figure S8. Generic epithelial-mesenchymal transition (EMT) and drug sensitivity. 14

Figure S9: Epithelial-mesenchymal transition (EMT) and drug sensitivity. 15

Figure S10. Differential treatment response in Epi and Mes tumours. 17

Figure S11: Correlation of generic EMT signature and stemness. 18

Supplementary Materials and Methods 19

Quality control of Affymetrix microarray expression data 19

Clinical tissue samples of Japanese Foundation for Cancer Research 19

RNA isolation and microarray analysis 19

Breast Cancer Subtype Prediction 20

Predictive modelling and validation by BinReg 20

Protein expression quantification from western blot 21

Supplementary References 22

Supplementary Text

Verifying bladder cancer-specific EMT signature

Breast and ovarian cancer-specific EMT signatures were previously verified (Akalay *et al*, 2013; Miow *et al*, 2014) and assessed again in Fig 1C. Hence, because of the relative lack of data in other cancers studying EMT parameters of multiple cell lines, we focused on verifying the bladder cancer-specific EMT signature (Fig S1). The EMT scores of bladder cancer cell lines were computed based on microarray gene expression of BLA-40 (Lee *et al*, 2007) and CCLE (Barretina *et al*, 2012). Data for the immunofluorescence staining of EMT markers, cell line morphology and invasion assays were taken or inferred from (Baumgart *et al*, 2007; Black *et al*, 2008; Chen *et al*, 2009) based solely on cell line name. Consistent with breast cancer data (Fig 1C), cell lines with high positive staining for VIM and CDH2 (Thiery *et al*, 2009) had a significantly greater EMT score (p=0.0324 and p=0.0022, respectively; Fig S1). Conversely, cell lines with high positive staining for known Epi markers, CDH1 and Plakoglobin, had a significantly lower EMT score (p=0.0025 and p=0.0062, respectively). No nuclear localisation of β-catenin was found in cell lines with high CDH1 staining intensity (Black *et al*, 2008). Importantly, not only did the EMT score provide a good indication as to whether a cell line had an Epi or Mes morphology (p=7.54E-4), but it could identify any intermediate phenotypic cell lines that displayed both Epi and Mes morphologies. The invasion assay also affirmed that cell lines with higher EMT scores were more invasive (p=0.0016). However, one caveat of this approach was that these experimental data were taken or inferred and combined from publications based solely on cell line names: it is possible that different cell lines were labelled with the same name, or the same cell line was cultured differently by different laboratories. In addition, we found one discrepancy for the highly metastatic UMUC14 bladder cancer cell line (Karashima *et al*, 2003): Chen and colleagues previously reported that it has an Epi morphology (Chen *et al*, 2009); yet, our EMT scoring revealed otherwise. Nevertheless, these results indicate the overall congruency between the bladder cancer-specific EMT signature scores and the published literature.

Verifying Generic EMT signature on Pancreatic Cancer

EMT has been implicated in pancreatic cancer progression and drug resistance (Arumugam *et al*, 2009; Hotz *et al*, 2007; Nakajima *et al*, 2004). Since the generic EMT signature was not derived from a pancreatic cancer-specific EMT signature, it was important to ascertain that the generic EMT scoring was capable of quantitating the EMT status accurately in pancreatic cancer. In Fig S3, we have validated the applicability of the generic EMT signature in pancreatic cancer where the change in EMT status of a pancreatic cancer cell line, PANC-1, induced with TGFβ, was accurately reflected. To further study the applicability of the generic EMT signature in pancreatic cancer, we performed additional validation of the generic EMT scoring in this cancer type. Fig S4 shows the relative protein expression normalized to β-actin, as inferred from western blot (Hotz *et al*, 2007; Zhu *et al*, 2012) (Supplementary Materials and Methods), immunofluorescence (IF) staining, and migration assays (Arumugam *et al*, 2009) of seven pancreatic cancer cell lines. EMT scores were computed based on gene expression data from SANGER COSMIC (Garnett *et al*, 2012) and CCLE (Barretina *et al*, 2012) collections. Note that the experiment data from publications was matched to the computed EMT score based solely on the cell line name. We observed that well-differentiated cell lines CAPAN-1 and HPAF-II were identified as Epi whereas undifferentiated cell lines MIAPaCa-2 and PANC-1 were identified as Mes. Western blot analysis of Epi cell lines showed strong relative protein expression of CDH1, the prototypic adhesion molecule of epithelial cells, whereas the relative expression of Mes markers (CDH2, SNAI1, SNAI2, TWIST1) were not limited to Mes cell lines (Hotz *et al*, 2007). However, positive IF for ZEB1 and VIM (Arumugam *et al*, 2009) was observed for the Mes cell lines, affirming the accuracy of generic EMT scoring. Migration assays conducted previously (Arumugam *et al*, 2009) uphold the belief that Mes cell lines are more invasive, a finding unambiguously reflected by the EMT score.

Generic EMT signature and miRNA

Expression levels of miRNAs have been implicated in promoting and suppressing EMT (Hao *et al*, 2014; Lim & Thiery, 2012; Zhang & Ma, 2012). In order to investigate whether a generic EMT

signature for miRNA can be established, we collected six datasets from GEO and TCGA comprising mRNA and miRNA expression profiles. We correlated the EMT score computed from the mRNA generic EMT signature with the expression of EMT-regulating miRNA (Hao *et al*, 2014; Zhang & Ma, 2012) in bladder (GSE40355) (Hecker *et al*, 2013), pancreas (GSE32688) (Donahue *et al*, 2012), prostate (GSE21034) (Taylor *et al*, 2010), breast (TCGA) (The Cancer Genome Atlas, 2012), ovarian cancer (TCGA) (The Cancer Genome Atlas, 2011) and multiple myeloma (GSE17498; Fig S2) (Lionetti *et al*, 2009). While it appears that miRNAs regulating EMT are cancer-specific, miR-200 (miR-200a, miR-200b, miR-200c, miR-141, miR-429) and miR-34 (miR-34a, miR-34b, miR-34c) families show consistently negative correlations with the EMT score across all cancer types, implicating a role in suppressing EMT universally. On the other hand, miR-155 and miR-214 exhibited consistent positive correlations with the generic EMT score across all cancers, suggesting a role in promoting EMT universally. However, we also noted that miRNA previously reported to promote or suppress EMT (Hao *et al*, 2014; Zhang & Ma, 2012) were not in perfect concordance with our results (Fig S2). For example, miR-143, previously reported to suppress metastasis and stem cell characteristics (Hao *et al*, 2014; Zhang & Ma, 2012), had a positive correlation with generic EMT score, suggestive of a role in promoting EMT (Fig S2). This discrepancy could stem from the platform-specific, cross-hybridization problem related to the short, closely related nucleotide sequences between miRNA family members that makes it technically challenging to measure miRNA expression (Mestdagh *et al*, 2014).

This assessment is preliminary, as only small cohorts were analysed. Further investigations re required whenever more datasets including both miRNA and mRNA gene expression profiles become available.

Effect of reducing the number of genes in the generic EMT signature

If a biomarker or gene-based scoring system is to be quick, cheap and cost-effective in the clinical setting, the system needs to have as few biomarkers or genes as possible (CMTP, 2013). Thus, we sought to identify the utility and reliability of a smaller, generic EMT signature. To do this, we

applied an increasingly stringent degree of *z*-transformed weight thresholds and subsequently computed the EMT score of these new generic EMT signatures. The EMT score from these new signature was then correlated with the reference EMT score (Table E1A) across 17 cancer types (Table E4A) using the Spearman Correlation Coefficient test (Fig S5). The correlation decreased sharply to less than 0.85 when the *z*-score threshold was raised to above 5.0. At a threshold of 5.0, the number of genes in the generic EMT signature was 62 Epi and 24 Mes genes, which was much lower than the original 145 Epi and 170 Mes genes in the initial reference signature (corresponding to 57% and 86% reductions, respectively). When segregating a tumour to Epi, intermediate or Mes, the EMT scoring using these 86 genes was able to estimate EMT in the different cancer types with an overall concordance of 75.08% with reference to the full EMT score. We then repeated the same analysis for cell lines across 20 types of cancer (Tables E4B, E4C). Using the same threshold of 5.0, the number of genes significantly reduced to 87 Epi and 18 Mes genes, with an overall correlation of 0.88. Using this reduced signature for cell lines, we repeated the validation analysis presented in Fig S3 and Table E3. This reduced set accurately segregated Epi and Mes samples within the various datasets (Table E3). These results indicate that concordance between the full (tumour: 315 genes; cell line: 218 genes) and reduced (tumour: 86 genes; cell lines: 105 genes) EMT signatures. Notably, molecular diagnostics technologies are now capable of simultaneously assaying up to 800 genes (nCounter® from NanoString Technologies, Inc., Seattle, WA), which could be a potential platform for the proposed generic EMT scoring method using either the full or the reduced EMT signature.

Generic EMT signature and stemness

EMT is often associated with the acquisition of stemness (Frisch *et al*, 2013; Huang *et al*, 2012; Tam & Weinberg, 2013; Thiery *et al*, 2009), and therefore, we assessed if scoring with the generic EMT signature could also universally quantitate the stemness phenotype in different cancer types. Using Spearman Correlation Coefficient test, we computed the correlation of the generic EMT score and ssGSEA (Verhaak *et al*, 2013) enrichment score of 21 published stem cell-related signatures (*Beier Glioma stem cell, Ben-Porath embryonic stem cell v1, Ben-Porath embryonic stem cell v2, Ben-Porath NANOG targets, Ben-Porath NOS targets, Ben-Porath OCT4 targets, Ben-Porath SOX2*

targets, *Bhattacharya embryonic stem cell*, *BIOCARTA stem pathway, Boquest stem cell cultured vs fresh*, *Boquest stem cell*, *Conrad stem cell*, *Gal leukemic stem cell*, *Gentles leukemic stem cell*, *Hoebeke lymphoid stem cell*, *Jaatinen hematopoietic stem cell*, *Lee neural crest stem cell*, *Oswald hematopoietic stem cell in collagen gel*, *Pece mammary stem cell*, *Wong embryonic stem cell core, Yamashita liver cancer stem cell*) found in the Molecular Signature Database (Msigdb v4.0) (Subramanian *et al*, 2005), as well as published stem cell markers (Medema, 2013) (Fig S11). The generic EMT score does not correlate universally with stemness in all cancer types. Positive correlations between generic EMT score and stem cell signatures, such as *Boquest stem cell* and *Pece mammary stem cell*, or gene expression of stem cell markers, such as *CD44* and *CXCR4*, were observed in the majority of the cancer types. However, this result is limited by the fact that these stem cell signatures were derived from different cell types, with no consideration given to the different types of stem cells. For example, in breast cancer, there exist at least two types of breast cancer stem cells (Liu *et al*, 2014). Thus, although we observed a correlation of generic EMT score with stemness in cases, the correlation is not universal in all cancers or in all types of stem cell.

Supplementary Figure Legends

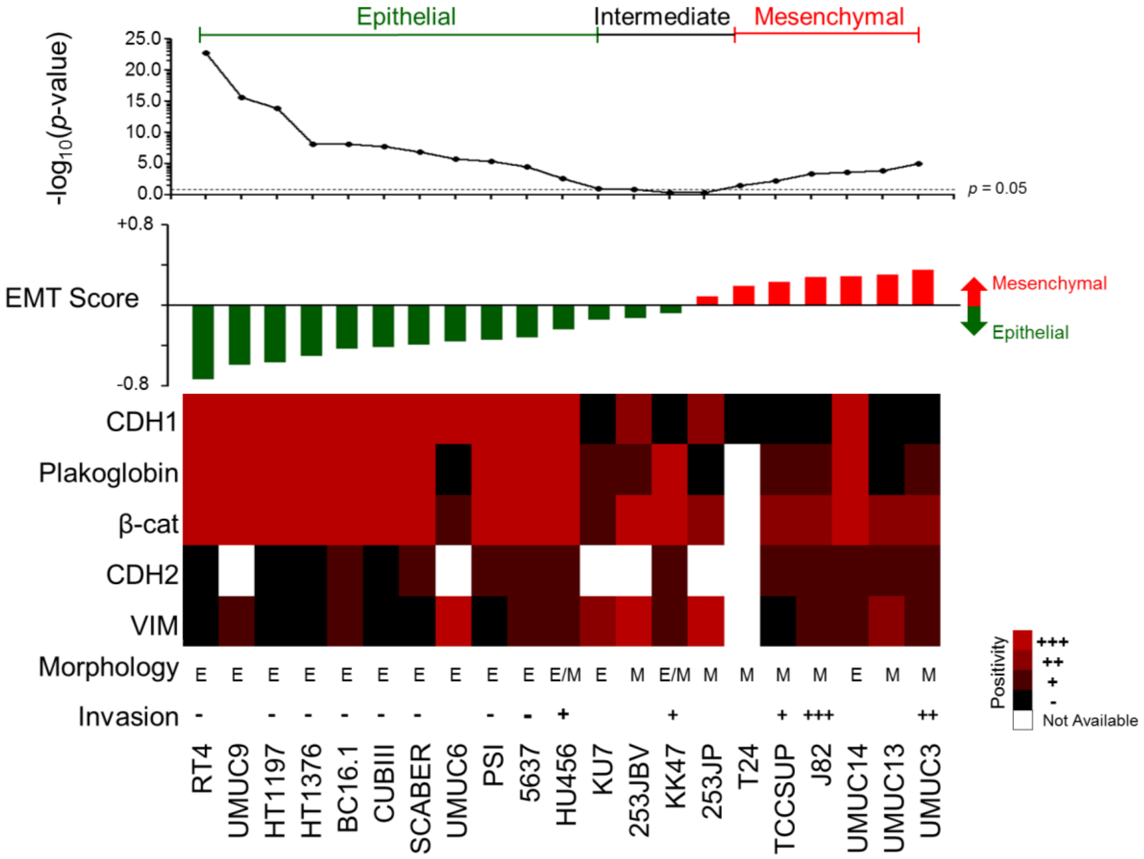

Figure S1: Validation of bladder-specific EMT signature.
Immunofluorescence staining heatmap of Epi (CDH1, Plakoglobin) and Mes (VIM, CDH2) markers, as well as non-nuclear β-catenin (black = low, red = high, white = no data). Bladder cancer cell lines (n=21) are aligned from the most Epi to most Mes based on the EMT score computed from gene expression data BLA-40 (Lee *et al*, 2007) and CCLE (Barretina *et al*, 2012), as shown by the bar chart. The dot plot is the $-\log_{10}$ p-value of two-sample Kolmogorov-Smirnov test. An arbitrary threshold of $p<0.05$ was used to define Epi, intermediate, and Mes cell lines. Reported morphology (E=Epi, M=Mes) and 16-h invasion assay measuring number of cells that had traversed the membrane grid/mm (-, 0-4; +, 5-20; ++, 21-50; +++, >51 cells) were given. The staining, morphology and invasion data were taken or inferred from the literature (Baumgart *et al*, 2007; Black *et al*, 2008; Chen *et al*, 2009).

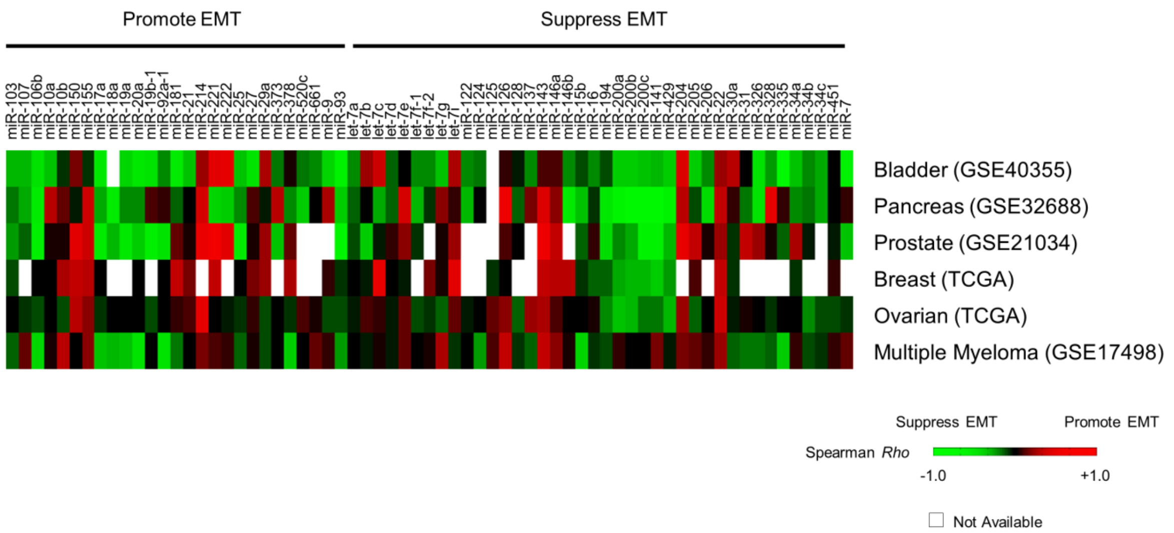

Figure S2: Correlation of generic EMT signature with miRNA implicated in EMT.
Heatmap of Spearman correlation coefficient *Rho* correlating generic EMT scores with miRNA expressions reported to regulate EMT.

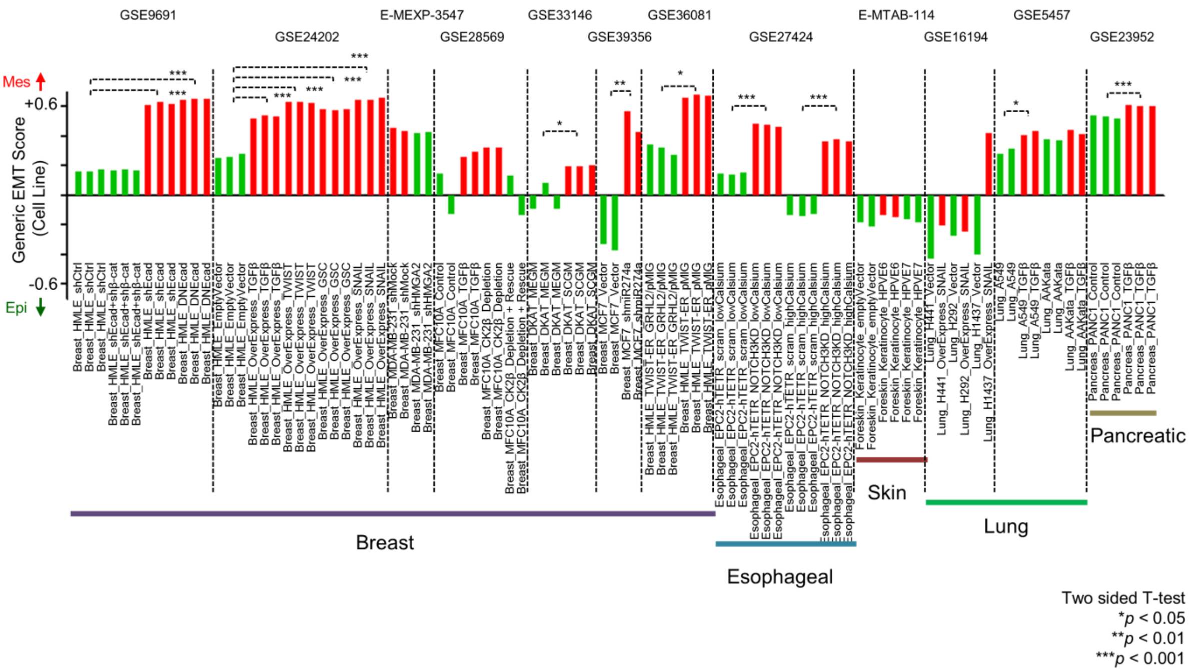

Figure S3: Validation of generic epithelial-mesenchymal transition (EMT) signature.
Bar chart of EMT scoring: in breast, GSE9691 (Onder *et al*, 2008), GSE24202 (Taube *et al*, 2010), E-MEXP-3547, GSE28569 (Deshiere *et al*, 2013), GSE33146 (D'Amato *et al*, 2012), GSE39356 (Cai *et al*, 2013), GSE36081 (Cieply *et al*, 2012); in oesophageal, GSE27424 (Ohashi *et al*, 2011); in skin, E-MTAB-114 (Hellner *et al*, 2009); in lung, GSE16194 (Yanagawa *et al*, 2009), GSE5457 (Malizia *et al*, 2009); and in pancreas, GSE23952 (Maupin *et al*, 2010). Dataset is separated by vertical dashed lines, with the dataset ID at the top of the chart. Cell line names and functional interventions are given at the bottom of chart. Coloured bars at the bottom-most region of the figure indicate the different cancer types. * indicates $p<0.05$, ** indicates $p<0.01$, and *** indicates $p<0.001$; computed by Mann Whitney U test. Colour code: green, more epithelial-like (Epi); red, more mesenchymal-like (Mes). EMT score and additional information are given in Tables E3 and E8.

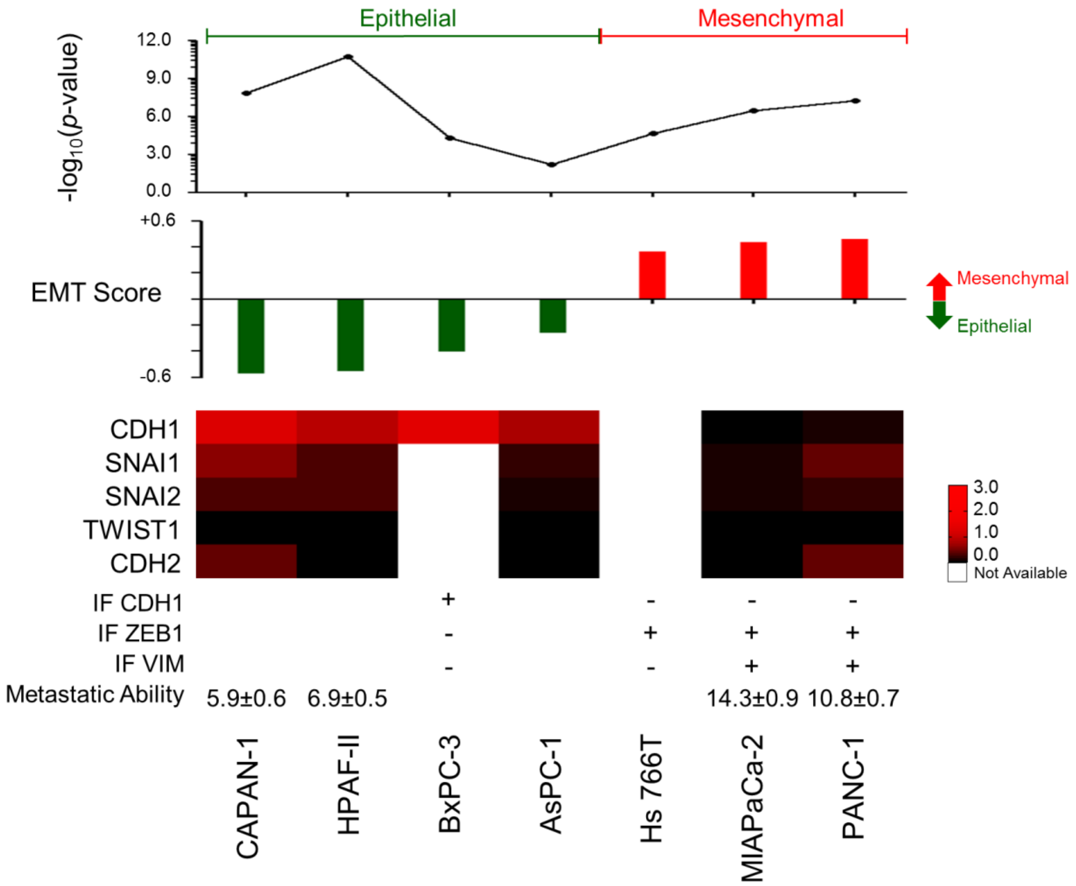

Figure S4: Validation of generic EMT signature on pancreatic cancer cell lines.
Normalized protein expression (relative to β-actin expression) estimated from western blot from the literature (Hotz *et al*, 2007; Zhu *et al*, 2012) for Epi (CDH1) and Mes (CDH2, SNAI1, SNAI2, VIM, and TWIST1) markers (black: no expression; red: high expression). Top panel shows the two-sample Kolmogorov-Smirnov test *p*-value for EMT scoring. Second panel is the EMT score computed using the generic EMT signature and data from SANGER COSMIC (Garnett *et al*, 2012) and CCLE (Barretina *et al*, 2012). The metastatic ability (in nude mouse) and immunofluorescence (IF) staining (-: no expression, +: positive) data was adapted from the literature (Arumugam *et al*, 2009).

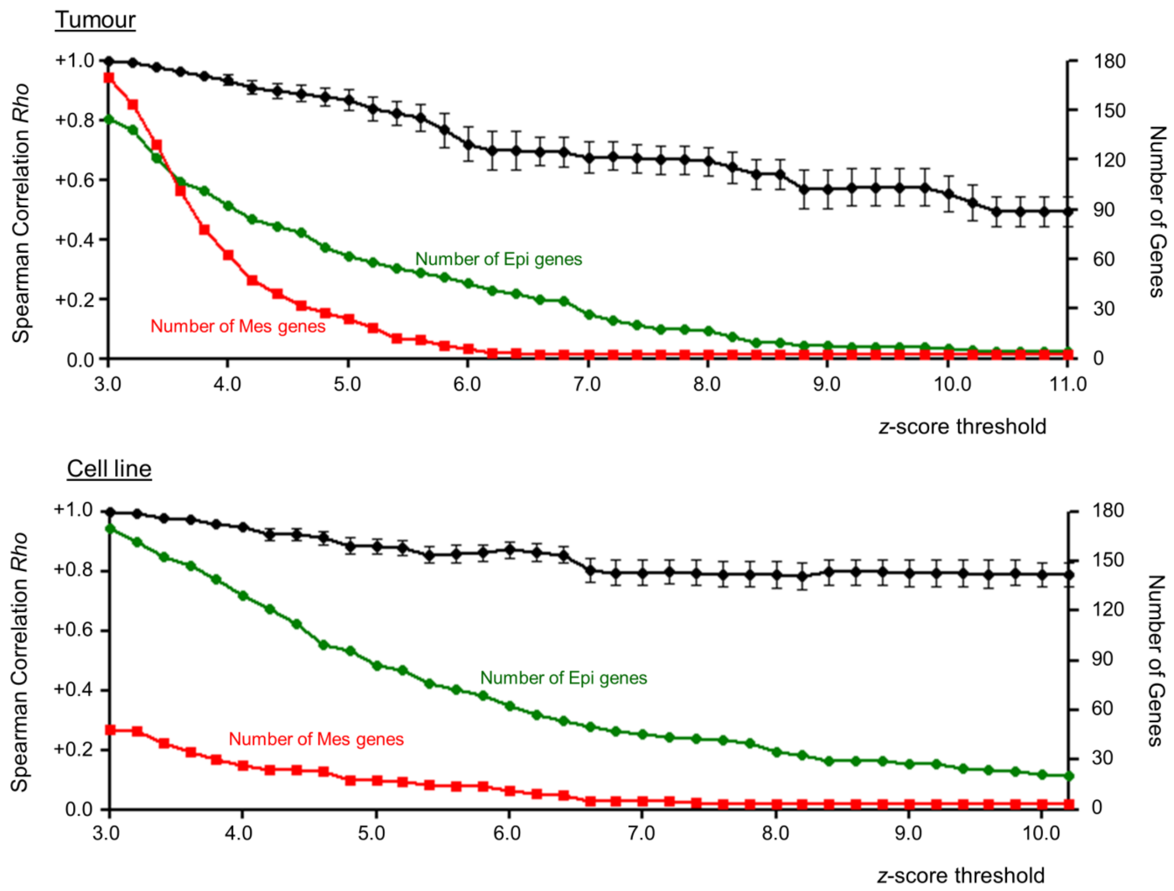

Figure S5: Effect of reducing genes in generic EMT signature.
Plot of Spearman correlation coefficient *Rho* (mean ± SEM from 17 cancer types in tumour and 20 cancer types in cell lines; black; left *y*-axis), as well as number of genes in generic EMT signature (Epi genes, green; Mes genes, red; right *y*-axis) against the *z*-transformed weight threshold (*x*-axis), computed based on Significance Analysis of Microarray (SAM) fold-change, false discovery rate, Receiver Operating Characteristics (ROC), and number of samples of a gene in each cancer-specific EMT signature.

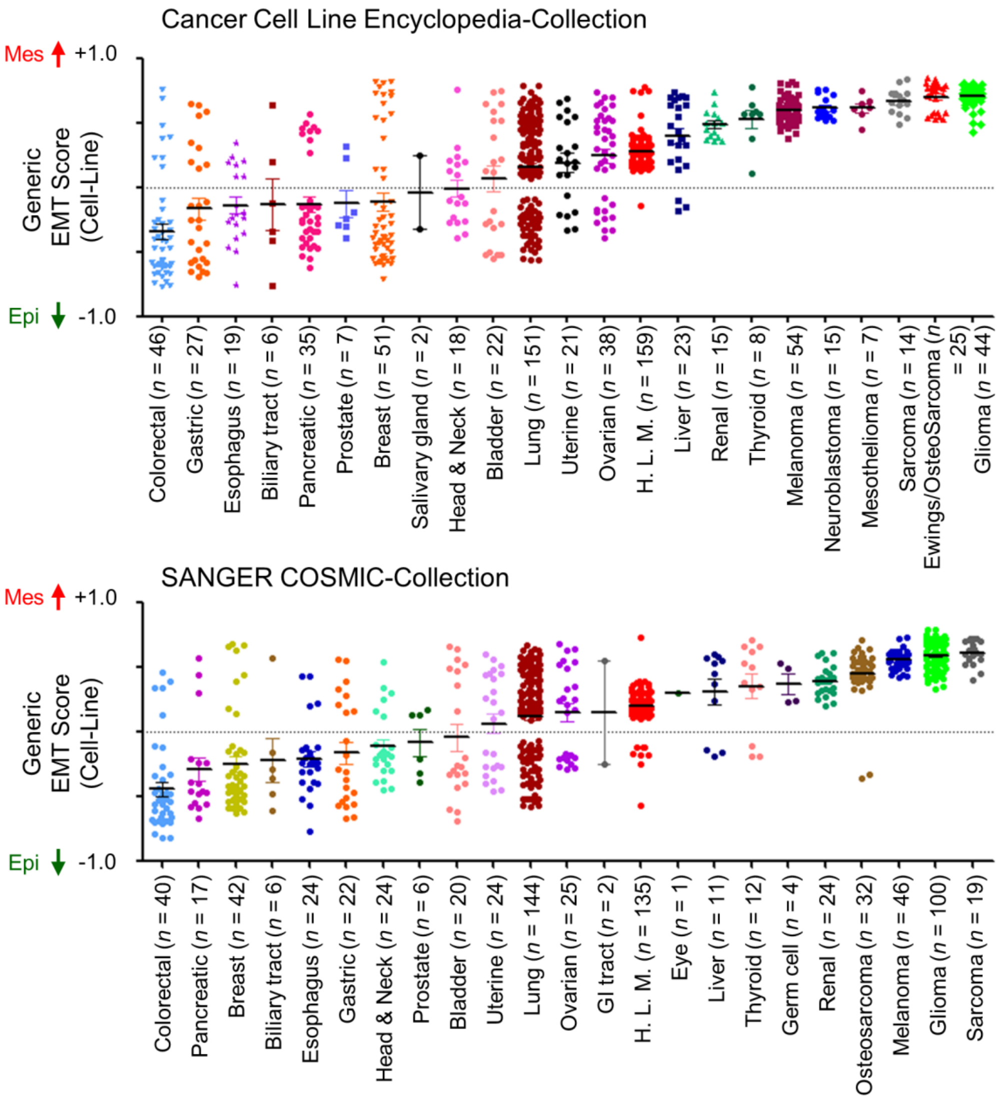

Figure S6: Epithelial-mesenchymal transition (EMT) spectrum in different cell lines.
Scatter plot of EMT scores for various cancer cell lines sourced from the Cancer Cell Line Encyclopedia (CCLE) (Barretina *et al*, 2012) (upper panel) and SANGER COSMIC (Garnett *et al*, 2012) (lower panel) cell line collections. Cell lines are grouped according to cancer/lineage and the groups are sorted by mean EMT score, as indicated by the horizontal centre line. The bars above and below this line indicate the ± square error of the mean. EMT score nearer to +1.0 is more mesenchymal-like (Mes) whereas EMT score nearer to -1.0 is more epithelial-like (Epi). Abbreviations: H. L. M., hematopoietic/lymphoid malignancy.

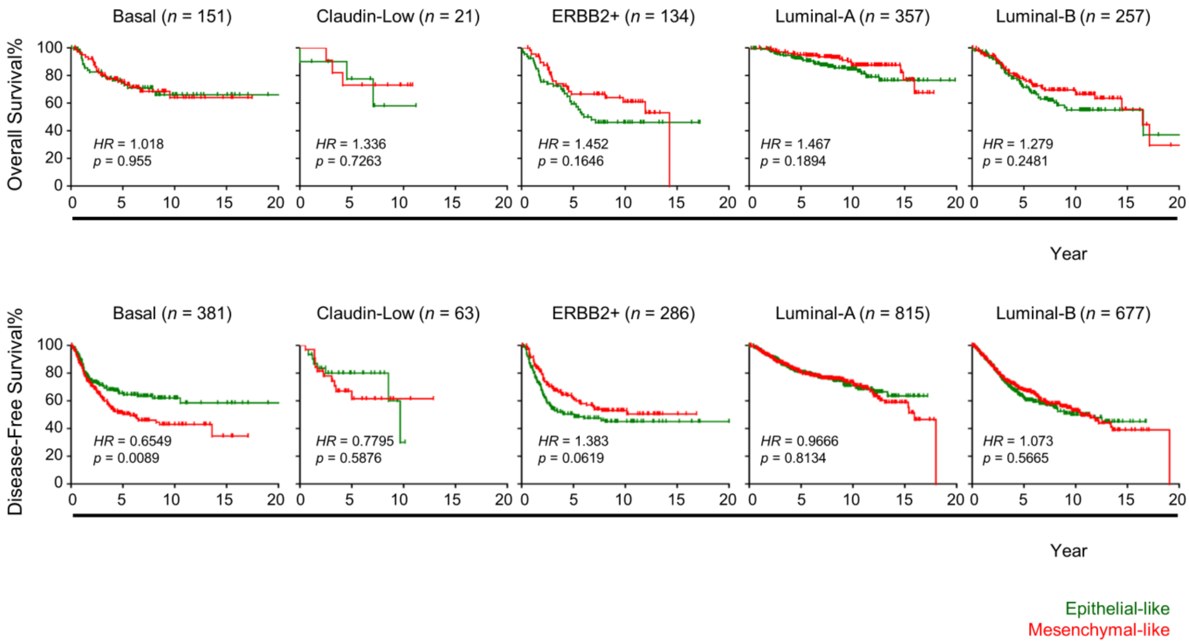

Figure S7. Correlation of Epithelial-Mesenchymal Transition and survival in breast cancer subtypes.

Kaplan–Meier analysis of overall survival (upper panel) and disease-free survival (lower panel) comparing mesenchymal-like (Mes; red) and epithelial-like (Epi; green) breast cancers in five molecular subtypes: Basal, Claudin-Low, ERBB2+, Luminal-A and Luminal-B. The molecular subtypes were predicted based on a subtype signature (Prat *et al*, 2010) and ssGSEA (Verhaak *et al*, 2013), as described in (Akalay *et al*, 2013). Median of EMT score was used to categorize breast cancer into Mes (≥ median) or Epi (< median). Hazard ratio (HR) and log-rank test *p*-values are shown. The number of samples (*n*) is given in parentheses.

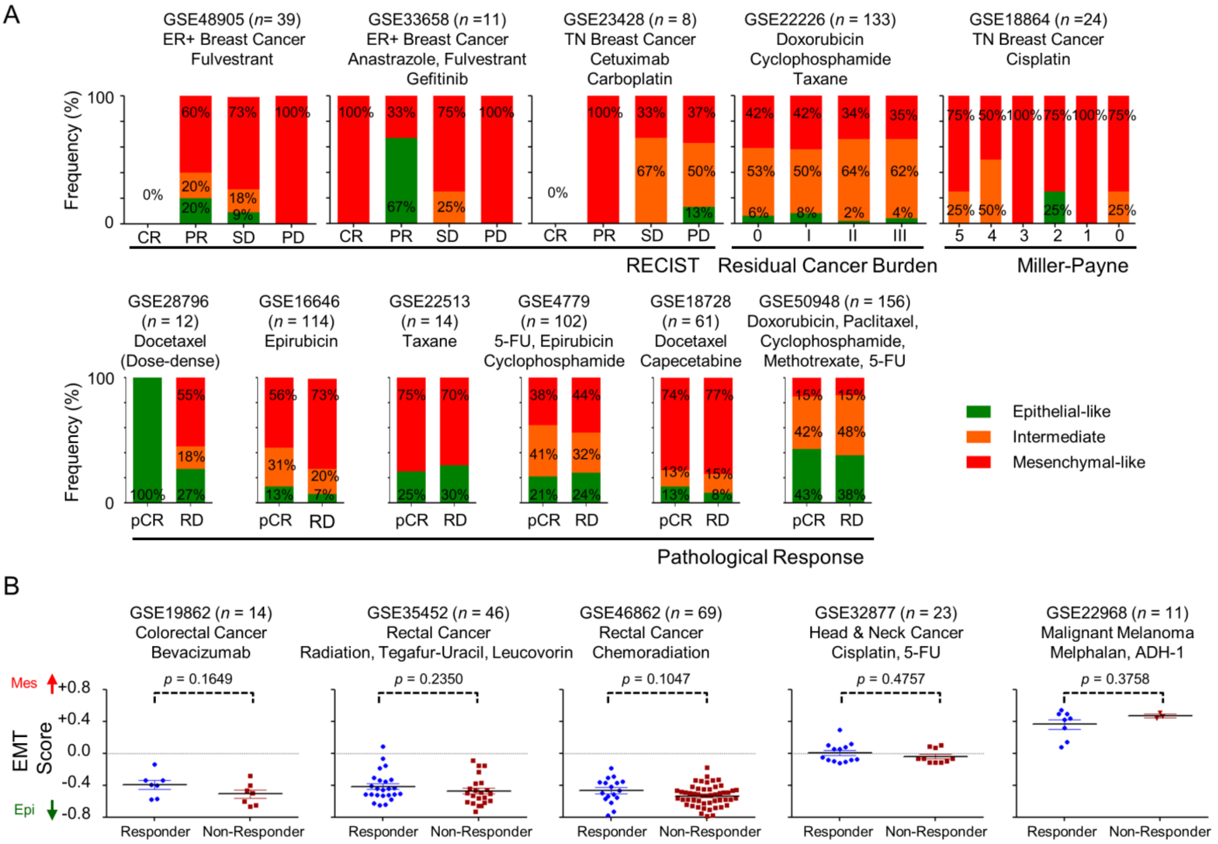

Figure S8. Generic epithelial-mesenchymal transition (EMT) and drug sensitivity.

A. Bar plots of 11 breast cancers cohorts (Bauer *et al*, 2010; Carey *et al*, 2012; Esserman *et al*, 2012; Evans *et al*, 2012; Farmer *et al*, 2009; Knudsen *et al*, 2014; Korde *et al*, 2010; Lehmann *et al*, 2011; Massarweh *et al*, 2011; Prat *et al*, 2014; Silver *et al*, 2010) and EMT status in clinical response to treatment. Percentage distributions of EMT status are given in each clinical response group. The bars are aligned from most sensitive to most resistant according to different assessment criteria (response evaluation criteria in solid tumour (RECIST), residual cancer burden (RCB) grading, Miller-Payne grading, and pathological response), as given in the original publications. The number of samples is given in parentheses, and the neoadjuvant treatment regimen is also indicated. Cohorts comprise a mix (ER+, HER2+ or ER-/PR-/HER2-) of breast cancer subtypes, unless specified. Abbreviation: CR, complete response; PR, partial response; SD, stable disease; PD, progressive disease; pCR, pathologic complete response; RD, residual disease; TN, triple negative; 5-FU, Fluorouracil. Green, epithelial-like (Epi); orange, intermediate; red, mesenchymal-like (Mes).

B. Dot plot of EMT score (mean ± SEM; *y*-axis) for responders (blue) and non-responders (maroon) in 3 recurrent colorectal cancer cohorts (Gim J, 2014), 1 head & neck cancer cohort (Tomkiewicz *et al*, 2012), and 1 malignant melanoma cohort (Beasley *et al*, 2011). Clinical response was evaluated based on RECIST or radiological examination, and the *p*-value was evaluated using Mann-Whitney *U*-test.

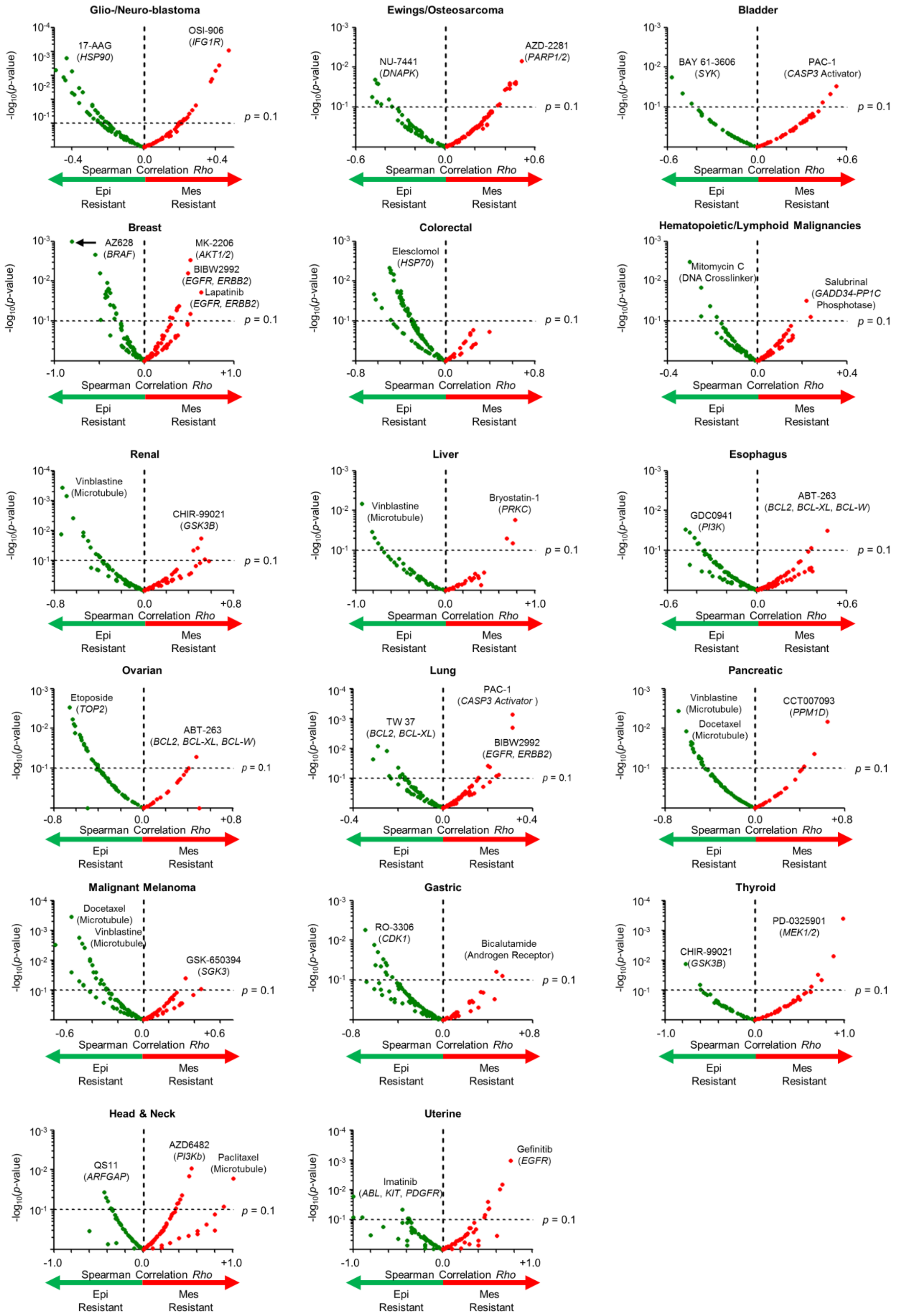

Figure S9: Epithelial-mesenchymal transition (EMT) and drug sensitivity.
Volcano plot of EMT correlation with drug sensitivity in 17 different cancers. *Rho* $\in [-1, +1]$ (*x*-axis) and -log$_{10}$ *p*-value (*y*-axis) were computed by Spearman correlation coefficient test. A dashed line corresponding to *p*=0.1 is plotted. Red indicates higher drug resistance in mesenchymal-like

(Mes) tumours (*Rho* $\in [0, +1]$), whereas green indicates higher drug resistance in epithelial-like (Epi) tumours (*Rho* $\in [-1,0]$). Selected compounds are labelled in different plots.

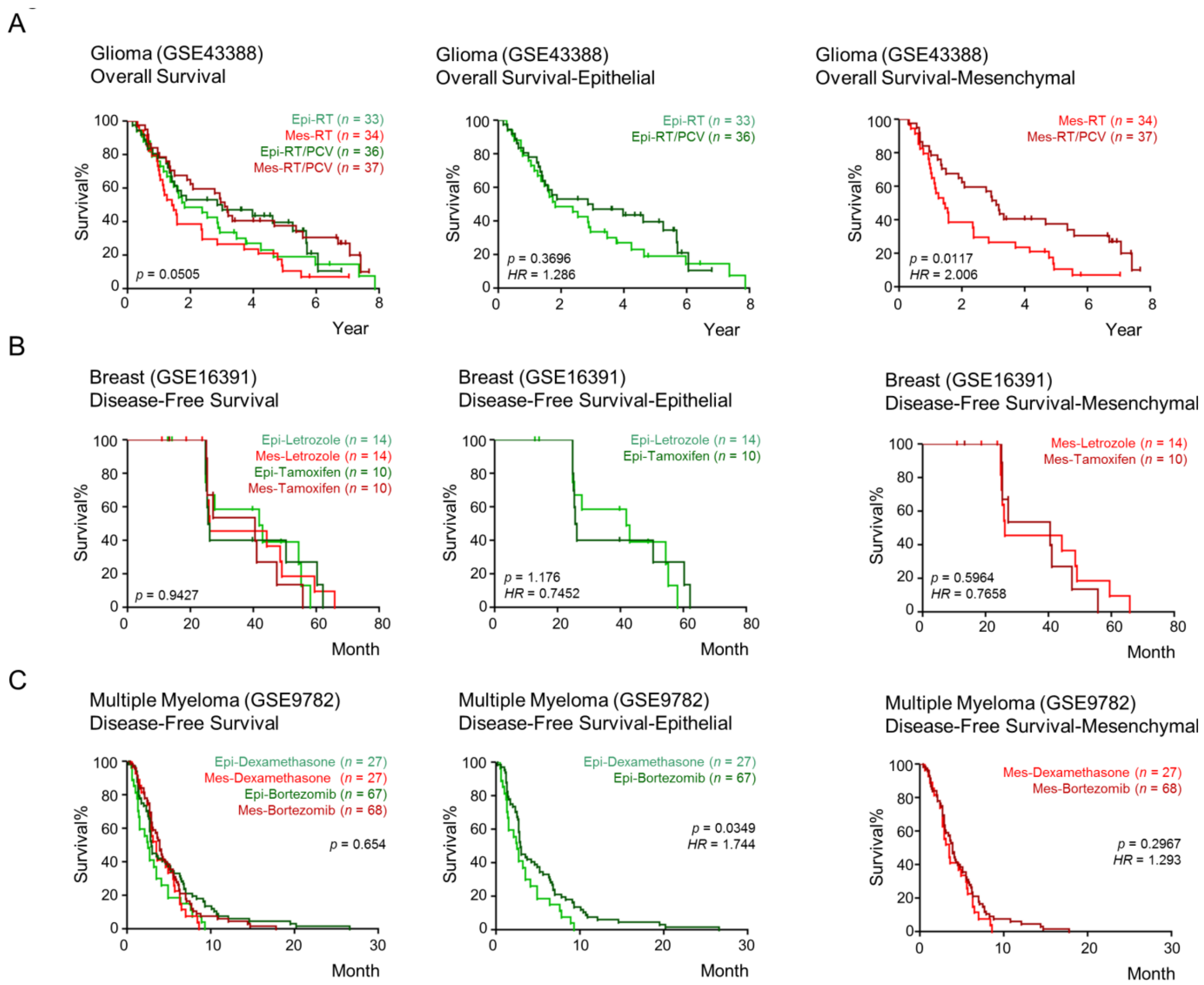

Figure S10. Differential treatment response in Epi and Mes tumours.

Kaplan–Meier analyses of overall survival or disease-free survival in patients with (A) Epi and Mes glioma (GSE43388) (Erdem-Eraslan *et al*, 2013) who underwent radiotherapy alone (RT) or radiotherapy and procarbazine, vincristine (RT/PCV), (B) ER+ breast cancer (GSE16391) (Desmedt *et al*, 2009), who underwent letrozole or tamoxifen, and (C) multiple myeloma (GSE9782) (Mulligan *et al*, 2007), who underwent dexamethasone or bortezomib. Left panels are the combined survival plots; middle panels are the survival plots of Epi tumours, and right panels are the survival plots of Mes tumours. Colour code: Green, epithelial-like (Epi); Red, mesenchymal-like (Mes). Light or dark colour indicates different treatment regimens. Epi and Mes are defined based on median EMT score, and *p*-value was evaluated using the log-rank test.

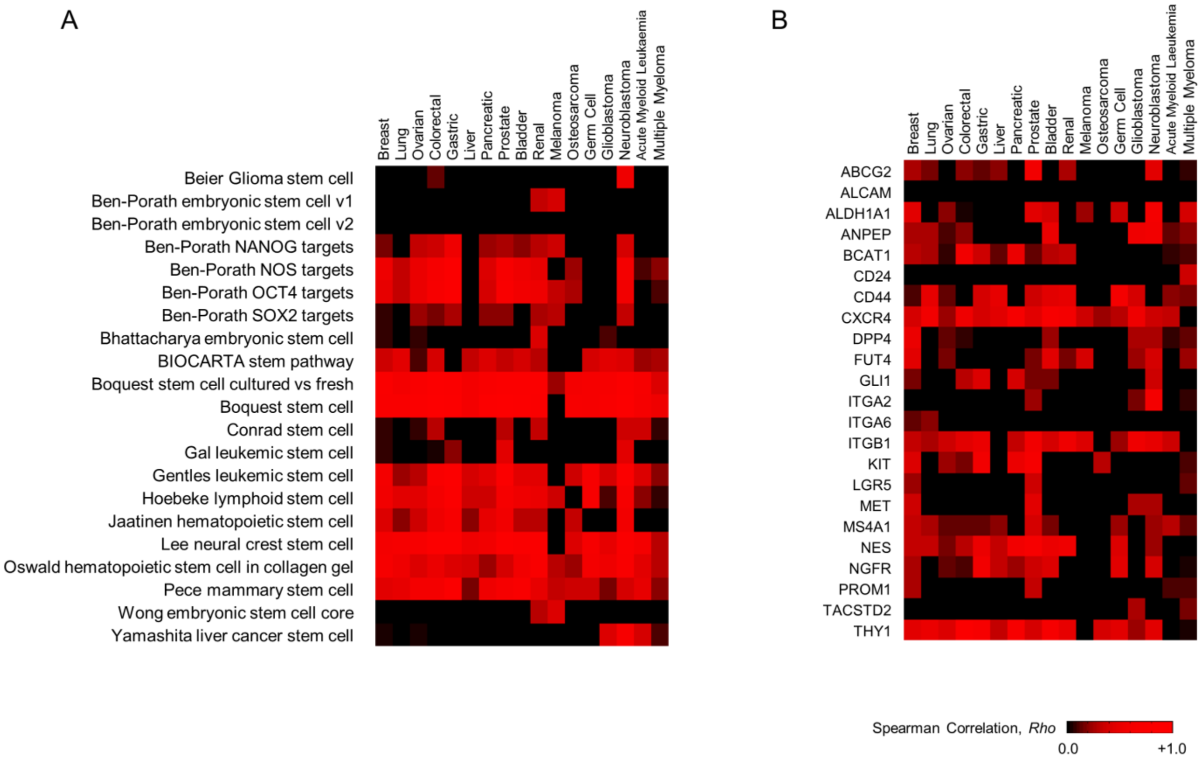

Figure S11: Correlation of generic EMT signature and stemness.
Heatmap of Spearman correlation coefficient *Rho* (red: positive correlation, black: no correlation) showing correlations of the generic EMT signature and the enrichment score of stemness-related gene sets from (A) Molecular Signature Database v4.0 (Subramanian *et al*, 2005) and (B) published stem cell markers.

Supplementary Materials and Methods

Quality control of Affymetrix microarray expression data

The quality of the Affymetrix chip (Affymetrix, Santa Clara, CA) was confirmed using the Bioconductor AffyQCReport package (Gautier *et al,* 2004) and the following criteria: average perfect-match intensity, kernel density plot, GAPDH 3':5' ratio, β-actin 3':5' ratio, and centre of intensity for positive and negative controls. All chips passed at least one of the criteria, and hence, none of the samples was discarded.

Clinical tissue samples of Japanese Foundation for Cancer Research

Clinical tissues were obtained from patients with breast cancer who had never received chemotherapy prior to sample collection and who underwent surgical resections of malignant lesions at the Cancer Institute Hospital, Japanese Foundation for Cancer Research (JFCR; Tokyo, Japan) between 2001 and 2007. This study was approved by the institutional review board at JFCR and informed consent was obtained from each patient. Each tissue sample was immediately embedded in a plastic cryomold containing Tissue-Tek OCT compound (Sakura Finetek, Tokyo, Japan), frozen with liquid nitrogen and kept in a -145°C freezer. Frozen tissue specimens were cut (10-μm-thick) in series using a cryostat (CM3050S, Leica Microsystems, Wetzlar, Germany) and placed onto plain, uncoated glass slides and stored at -20°C. Subsequently, sections were stained with haematoxylin and eosin and covered by micro-cover glasses. The prepared slides were pathologically examined to distinguish clusters of mammary tumour cells from normal and stromal cells.

RNA isolation and microarray analysis

Frozen OCT-embedded tissues were washed with 70% ethanol and water, and stained with Mayer's haematoxylin for 30 sec. Stained sections were water-washed and then dried for the subsequent dissection process. Cancer cells were isolated by laser capture micro-dissection (LCM, Leica Microsystems). Total RNA was extracted using RNeasy Micro Kit (Qiagen, Hilden, Germany)

according to the manufacturer's procedure. The total RNA concentration for each sample was measured using a NanoDrop spectrophotometer (NanoDrop Technologies, Wilmington, DE). Any possible degradation of the total RNA was validated using a BioAnalyzer 2100 (Agilent Technologies, Santa Clara, CA). Total RNA with the desired concentration and quality was amplified and biotinylated with GeneChip Two-Cycle Target Labeling and Control Reagents (Affymetrix). Concentration and size of cRNA were validated with the BioAnalyzer. Biotinylated cRNA was fragmented following the Two-Cycle assay protocol. The fragmented cRNA was validated in terms of fragment size, again using the BioAnalyzer, and applied to GeneChip Human Genome U133 Plus 2.0 (Affymetrix) and incubated at 45°C for 16 h. After hybridization, the microarray was washed and stained with a streptavidin phycoerythrin (SAPE) conjugate using a fully automated system (GeneChip Fluidic Station 450; Affymetrix), according to the manufacturer's procedure. The hybridized probe arrays were scanned using the GeneChip Scanner 3000 (Affymetrix). The microarray data were deposited at Gene Expression Omnibus (GEO) with the accession number GSE54002.

Breast Cancer Subtype Prediction

For each breast cancer sample, subtype signature (Prat *et al*, 2010) scores for Basal, Claudin-Low, Luminal, ERBB2+, and Normal-like subtypes were computed using single-sample Gene Set Enrichment Analysis (ssGSEA) (Verhaak *et al*, 2013). The subtype of each breast cancer sample was inferred based on the maximum subtype signature score. For Luminal breast cancers, the PAM50 signature (Parker *et al*, 2009) was applied to further classify them into Luminal-A and Luminal-B subtypes.

Predictive modelling and validation by BinReg

Expression data analysis based on a binary regression model using the BinReg v2.0 (renamed to Profiler, http://dig.genome.duke.edu/software.html) has been described previously (Gatza *et al*, 2010; Tan *et al*, 2013). Briefly, BinReg uses a binary probit regression model and Bayesian statistics to produce fitted regression coefficients on a set of genes that are most correlated with the binary

response/phenotype of interest (e.g., epithelial vs. mesenchymal). The regression coefficients of these genes indicate the discriminating power of the genes and are weights for the overall meta-gene profile. The overall meta-gene profile is used for comparison and predicts the status of the EMT phenotype of the new sample or dataset. To predict the status of the phenotype on a dataset, a Bayesian probit regression model was fit to assign the probability that the sample exhibited evidence of a phenotype, based on the concordance of its gene expression values with the signature (Gatza *et al,* 2010).

Protein expression quantification from western blot

Western blot images were downloaded from the journal website (Hotz *et al*, 2007) and processed using ImageJ 1.46r software (NIH, Bethesda, MD). A rectangular selection tool was used to mark the regions of interest, and quantitation was performed using the area under the curve (AUC) of the intensity histogram. Regions of interest with no unimodal peaks in the intensity histogram were considered as ‘no expression’. The AUC was normalized to that of β-actin and the normalized AUC was taken as the protein expression.

Supplementary References

Akalay I, Janji B, Hasmim M, Noman MZ, Andre F, De Cremoux P, Bertheau P, Badoual C, Vielh P, Larsen AK et al (2013) Epithelial-to-mesenchymal transition and autophagy induction in breast carcinoma promote escape from T-cell-mediated lysis. *Cancer Res* 73**:** 2418-2427

Arumugam T, Ramachandran V, Fournier KF, Wang H, Marquis L, Abbruzzese JL, Gallick GE, Logsdon CD, McConkey DJ, Choi W (2009) Epithelial to mesenchymal transition contributes to drug resistance in pancreatic cancer. *Cancer Res* 69**:** 5820-5828

Barretina J, Caponigro G, Stransky N, Venkatesan K, Margolin AA, Kim S, Wilson CJ, Lehar J, Kryukov GV, Sonkin D et al (2012) The Cancer Cell Line Encyclopedia enables predictive modelling of anticancer drug sensitivity. *Nature* 483**:** 603-607

Bauer JA, Chakravarthy AB, Rosenbluth JM, Mi D, Seeley EH, De Matos Granja-Ingram N, Olivares MG, Kelley MC, Mayer IA, Meszoely IM et al (2010) Identification of markers of taxane sensitivity using proteomic and genomic analyses of breast tumors from patients receiving neoadjuvant paclitaxel and radiation. *Clin Cancer Res* 16**:** 681-690

Baumgart E, Cohen MS, Silva Neto B, Jacobs MA, Wotkowicz C, Rieger-Christ KM, Biolo A, Zeheb R, Loda M, Libertino JA et al (2007) Identification and prognostic significance of an epithelial-mesenchymal transition expression profile in human bladder tumors. *Clin Cancer Res* 13**:** 1685-1694

Beasley GM, Riboh JC, Augustine CK, Zager JS, Hochwald SN, Grobmyer SR, Peterson B, Royal R, Ross MI, Tyler DS (2011) Prospective multicenter phase II trial of systemic ADH-1 in combination with melphalan via isolated limb infusion in patients with advanced extremity melanoma. *J Clin Oncol* 29**:** 1210-1215

Black PC, Brown GA, Inamoto T, Shrader M, Arora A, Siefker-Radtke AO, Adam L, Theodorescu D, Wu X, Munsell MF et al (2008) Sensitivity to epidermal growth factor receptor inhibitor requires E-cadherin expression in urothelial carcinoma cells. *Clin Cancer Res* 14**:** 1478-1486

Cai J, Guan H, Fang L, Yang Y, Zhu X, Yuan J, Wu J, Li M (2013) MicroRNA-374a activates Wnt/beta-catenin signaling to promote breast cancer metastasis. *J Clin Invest* 123**:** 566-579

The Cancer Genome Atlas (2012) Comprehensive molecular portraits of human breast tumours. *Nature* 490**:** 61-70

The Cancer Genome Atlas (2011) Integrated genomic analyses of ovarian carcinoma. *Nature* 474**:** 609-615

Carey LA, Rugo HS, Marcom PK, Mayer EL, Esteva FJ, Ma CX, Liu MC, Storniolo AM, Rimawi MF, Forero-Torres A et al (2012) TBCRC 001: randomized phase II study of cetuximab in combination with carboplatin in stage IV triple-negative breast cancer. *J Clin Oncol* 30**:** 2615-2623

Chen LM, Verity NJ, Chai KX (2009) Loss of prostasin (PRSS8) in human bladder transitional cell carcinoma cell lines is associated with epithelial-mesenchymal transition (EMT). *BMC Cancer* 9**:** 377

Cieply B, Riley Pt, Pifer PM, Widmeyer J, Addison JB, Ivanov AV, Denvir J, Frisch SM (2012) Suppression of the epithelial-mesenchymal transition by Grainyhead-like-2. *Cancer Res* 72**:** 2440-2453

CMTP, Center for Medical Technology and Policy (2013) What are the Evidence Gaps Affecting Clinical Use of Next Generation Sequencing Tests in Oncology?, Deverka P (ed). CMTP, Center for Medical Technology Policy

D'Amato NC, Ostrander JH, Bowie ML, Sistrunk C, Borowsky A, Cardiff RD, Bell K, Young LJ, Simin K, Bachelder RE et al (2012) Evidence for phenotypic plasticity in aggressive triple-negative breast cancer: human biology is recapitulated by a novel model system. *PLoS One* 7**:** e45684

Deshiere A, Duchemin-Pelletier E, Spreux E, Ciais D, Combes F, Vandenbrouck Y, Coute Y, Mikaelian I, Giusiano S, Charpin C et al (2013) Unbalanced expression of CK2 kinase subunits is sufficient to drive epithelial-to-mesenchymal transition by Snail1 induction. *Oncogene* 32**:** 1373-1383

Desmedt C, Giobbie-Hurder A, Neven P, Paridaens R, Christiaens MR, Smeets A, Lallemand F, Haibe-Kains B, Viale G, Gelber RD et al (2009) The Gene expression Grade Index: a potential predictor of relapse for endocrine-treated breast cancer patients in the BIG 1-98 trial. *BMC Med Genomics* 2**:** 40

Donahue TR, Tran LM, Hill R, Li Y, Kovochich A, Calvopina JH, Patel SG, Wu N, Hindoyan A, Farrell JJ et al (2012) Integrative survival-based molecular profiling of human pancreatic cancer. *Clin Cancer Res* 18**:** 1352-1363

Erdem-Eraslan L, Gravendeel LA, de Rooi J, Eilers PH, Idbaih A, Spliet WG, den Dunnen WF, Teepen JL, Wesseling P, Sillevis Smitt PA et al (2013) Intrinsic molecular subtypes of glioma are prognostic and predict benefit from adjuvant procarbazine, lomustine, and vincristine chemotherapy in combination with other prognostic factors in anaplastic oligodendroglial brain tumors: a report from EORTC study 26951. *J Clin Oncol* 31**:** 328-336

Esserman LJ, Berry DA, Cheang MC, Yau C, Perou CM, Carey L, DeMichele A, Gray JW, Conway-Dorsey K, Lenburg ME et al (2012) Chemotherapy response and recurrence-free survival in neoadjuvant breast cancer depends on biomarker profiles: results from the I-SPY 1 TRIAL (CALGB 150007/150012; ACRIN 6657). *Breast Cancer Res Treat* 132**:** 1049-1062

Evans AL, Faial T, Gilchrist MJ, Down T, Vallier L, Pedersen RA, Wardle FC, Smith JC (2012) Genomic targets of Brachyury (T) in differentiating mouse embryonic stem cells. *PLoS One* 7**:** e33346

Farmer P, Bonnefoi H, Anderle P, Cameron D, Wirapati P, Becette V, Andre S, Piccart M, Campone M, Brain E et al (2009) A stroma-related gene signature predicts resistance to neoadjuvant chemotherapy in breast cancer. *Nat Med* 15**:** 68-74

Frisch SM, Schaller M, Cieply B (2013) Mechanisms that link the oncogenic epithelial-mesenchymal transition to suppression of anoikis. *J Cell Sci* 126**:** 21-29

Garnett MJ, Edelman EJ, Heidorn SJ, Greenman CD, Dastur A, Lau KW, Greninger P, Thompson IR, Luo X, Soares J et al (2012) Systematic identification of genomic markers of drug sensitivity in cancer cells. *Nature* 483**:** 570-575

Gatza ML, Lucas JE, Barry WT, Kim JW, Wang Q, Crawford MD, Datto MB, Kelley M, Mathey-Prevot B, Potti A et al (2010) A pathway-based classification of human breast cancer. *Proc Natl Acad Sci U S A* 107**:** 6994-6999

Gautier L, Cope L, Bolstad BM, Irizarry RA (2004) affy--analysis of Affymetrix GeneChip data at the probe level. *Bioinformatics* 20**:** 307-315

Gim J CY, Hong SH, Kim HC, Chun HK, Park T*, Park WY, Lee WY (2014) Predicting multi-class response to preoperative chemoradiotherapy in rectal cancer patients, manuscript in preparation.

Hao J, Zhang Y, Deng M, Ye R, Zhao S, Wang Y, Li J, Zhao Z (2014) MicroRNA control of epithelial-mesenchymal transition in cancer stem cells. *Int J Cancer* 135**:** 1019-1027

Hecker N, Stephan C, Mollenkopf HJ, Jung K, Preissner R, Meyer HA (2013) A new algorithm for integrated analysis of miRNA-mRNA interactions based on individual classification reveals insights into bladder cancer. *PLoS One* 8**:** e64543

Hellner K, Mar J, Fang F, Quackenbush J, Munger K (2009) HPV16 E7 oncogene expression in normal human epithelial cells causes molecular changes indicative of an epithelial to mesenchymal transition. *Virology* 391**:** 57-63

Hotz B, Arndt M, Dullat S, Bhargava S, Buhr HJ, Hotz HG (2007) Epithelial to mesenchymal transition: expression of the regulators snail, slug, and twist in pancreatic cancer. *Clin Cancer Res* 13**:** 4769-4776

Huang RY, Chung VY, Thiery JP (2012) Targeting pathways contributing to epithelial-mesenchymal transition (EMT) in epithelial ovarian cancer. *Curr Drug Targets* 13**:** 1649-1653

Karashima T, Sweeney P, Kamat A, Huang S, Kim SJ, Bar-Eli M, McConkey DJ, Dinney CP (2003) Nuclear factor-kappaB mediates angiogenesis and metastasis of human bladder cancer through the regulation of interleukin-8. *Clin Cancer Res* 9**:** 2786-2797

Knudsen S, Jensen T, Hansen A, Mazin W, Lindemann J, Kuter I, Laing N, Anderson E (2014) Development and validation of a gene expression score that predicts response to fulvestrant in breast cancer patients. *PLoS One* 9**:** e87415

Korde LA, Lusa L, McShane L, Lebowitz PF, Lukes L, Camphausen K, Parker JS, Swain SM, Hunter K, Zujewski JA (2010) Gene expression pathway analysis to predict response to neoadjuvant docetaxel and capecitabine for breast cancer. *Breast Cancer Res Treat* 119**:** 685-699

Lee JK, Havaleshko DM, Cho H, Weinstein JN, Kaldjian EP, Karpovich J, Grimshaw A, Theodorescu D (2007) A strategy for predicting the chemosensitivity of human cancers and its application to drug discovery. *Proc Natl Acad Sci U S A* 104**:** 13086-13091

Lehmann BD, Bauer JA, Chen X, Sanders ME, Chakravarthy AB, Shyr Y, Pietenpol JA (2011) Identification of human triple-negative breast cancer subtypes and preclinical models for selection of targeted therapies. *J Clin Invest* 121**:** 2750-2767

Lim J, Thiery JP (2012) Epithelial-mesenchymal transitions: insights from development. *Development* 139**:** 3471-3486

Lionetti M, Biasiolo M, Agnelli L, Todoerti K, Mosca L, Fabris S, Sales G, Deliliers GL, Bicciato S, Lombardi L et al (2009) Identification of microRNA expression patterns and definition of a microRNA/mRNA regulatory network in distinct molecular groups of multiple myeloma. *Blood* 114**:** e20-26

Liu S, Cong Y, Wang D, Sun Y, Deng L, Liu Y, Martin-Trevino R, Shang L, McDermott SP, Landis MD et al (2014) Breast Cancer Stem Cells Transition between Epithelial and Mesenchymal States Reflective of their Normal Counterparts. *Stem Cell Reports* 2**:** 78-91

Malizia AP, Lacey N, Walls D, Egan JJ, Doran PP (2009) CUX1/Wnt signaling regulates epithelial mesenchymal transition in EBV infected epithelial cells. *Exp Cell Res* 315**:** 1819-1831

Massarweh S, Tham YL, Huang J, Sexton K, Weiss H, Tsimelzon A, Beyer A, Rimawi M, Cai WY, Hilsenbeck S et al (2011) A phase II neoadjuvant trial of anastrozole, fulvestrant, and gefitinib in patients with newly diagnosed estrogen receptor positive breast cancer. *Breast Cancer Res Treat* 129**:** 819-827

Maupin KA, Sinha A, Eugster E, Miller J, Ross J, Paulino V, Keshamouni VG, Tran N, Berens M, Webb C et al (2010) Glycogene expression alterations associated with pancreatic cancer epithelial-mesenchymal transition in complementary model systems. *PLoS One* 5**:** e13002

Medema JP (2013) Cancer stem cells: the challenges ahead. *Nat Cell Biol* 15**:** 338-344

Mestdagh P, Hartmann N, Baeriswyl L, Andreasen D, Bernard N, Chen C, Cheo D, D'Andrade P, DeMayo M, Dennis L et al (2014) Evaluation of quantitative miRNA expression platforms in the microRNA quality control (miRQC) study. *Nat Methods* 10.1038/nmeth.3014

Miow QH, Tan TZ, Ye J, Lau JA, Yokomizo T, Thiery J-P, Mori S (2014) Epithelial-Mesenchymal Status Renders Differential Responses to Cisplatin in Ovarian Cancer. *Oncogene, In Press* doi: 10.1038/onc.2014.136

Mulligan G, Mitsiades C, Bryant B, Zhan F, Chng WJ, Roels S, Koenig E, Fergus A, Huang Y, Richardson P et al (2007) Gene expression profiling and correlation with outcome in clinical trials of the proteasome inhibitor bortezomib. *Blood* 109**:** 3177-3188

Nakajima S, Doi R, Toyoda E, Tsuji S, Wada M, Koizumi M, Tulachan SS, Ito D, Kami K, Mori T et al (2004) N-cadherin expression and epithelial-mesenchymal transition in pancreatic carcinoma. *Clin Cancer Res* 10**:** 4125-4133

Ohashi S, Natsuizaka M, Naganuma S, Kagawa S, Kimura S, Itoh H, Kalman RA, Nakagawa M, Darling DS, Basu D et al (2011) A NOTCH3-mediated squamous cell differentiation program limits expansion of EMT-competent cells that express the ZEB transcription factors. *Cancer Res* 71**:** 6836-6847

Onder TT, Gupta PB, Mani SA, Yang J, Lander ES, Weinberg RA (2008) Loss of E-cadherin promotes metastasis via multiple downstream transcriptional pathways. *Cancer Res* 68**:** 3645-3654

Parker JS, Mullins M, Cheang MC, Leung S, Voduc D, Vickery T, Davies S, Fauron C, He X, Hu Z et al (2009) Supervised risk predictor of breast cancer based on intrinsic subtypes. *J Clin Oncol* 27**:** 1160-1167

Prat A, Bianchini G, Thomas M, Belousov A, Cheang MC, Koehler A, Gomez P, Semiglazov V, Eiermann W, Tjulandin S et al (2014) Research-based PAM50 subtype predictor identifies higher responses and improved survival outcomes in HER2-positive breast cancer in the NOAH study. *Clin Cancer Res* 20**:** 511-521

Prat A, Parker JS, Karginova O, Fan C, Livasy C, Herschkowitz JI, He X, Perou CM (2010) Phenotypic and molecular characterization of the claudin-low intrinsic subtype of breast cancer. *Breast Cancer Res* 12**:** R68

Silver DP, Richardson AL, Eklund AC, Wang ZC, Szallasi Z, Li Q, Juul N, Leong CO, Calogrias D, Buraimoh A et al (2010) Efficacy of neoadjuvant Cisplatin in triple-negative breast cancer. *J Clin Oncol* 28**:** 1145-1153

Subramanian A, Tamayo P, Mootha VK, Mukherjee S, Ebert BL, Gillette MA, Paulovich A, Pomeroy SL, Golub TR, Lander ES et al (2005) Gene set enrichment analysis: a knowledge-based approach for interpreting genome-wide expression profiles. *Proc Natl Acad Sci U S A* 102**:** 15545-15550

Tam WL, Weinberg RA (2013) The epigenetics of epithelial-mesenchymal plasticity in cancer. *Nat Med* 19**:** 1438-1449

Tan TZ, Miow QH, Huang RY, Wong MK, Ye J, Lau JA, Wu MC, Bin Abdul Hadi LH, Soong R, Choolani M et al (2013) Functional genomics identifies five distinct molecular subtypes with clinical relevance and pathways for growth control in epithelial ovarian cancer. *EMBO Mol Med* 5**:** 983-998

Taube JH, Herschkowitz JI, Komurov K, Zhou AY, Gupta S, Yang J, Hartwell K, Onder TT, Gupta PB, Evans KW et al (2010) Core epithelial-to-mesenchymal transition interactome gene-expression signature is associated with claudin-low and metaplastic breast cancer subtypes. *Proc Natl Acad Sci U S A* 107**:** 15449-15454

Taylor BS, Schultz N, Hieronymus H, Gopalan A, Xiao Y, Carver BS, Arora VK, Kaushik P, Cerami E, Reva B et al (2010) Integrative genomic profiling of human prostate cancer. *Cancer Cell* 18**:** 11-22

Thiery JP, Acloque H, Huang RY, Nieto MA (2009) Epithelial-mesenchymal transitions in development and disease. *Cell* 139**:** 871-890

Tomkiewicz C, Hans S, Mucchielli MH, Agier N, Delacroix H, Marisa L, Brasnu D, Aggerbeck LP, Badoual C, Barouki R et al (2012) A head and neck cancer tumor response-specific gene signature for cisplatin, 5-fluorouracil induction chemotherapy fails with added taxanes. *PLoS One* 7**:** e47170

Verhaak RG, Tamayo P, Yang JY, Hubbard D, Zhang H, Creighton CJ, Fereday S, Lawrence M, Carter SL, Mermel CH et al (2013) Prognostically relevant gene signatures of high-grade serous ovarian carcinoma. *J Clin Invest* 123**:** 517-525

Yanagawa J, Walser TC, Zhu LX, Hong L, Fishbein MC, Mah V, Chia D, Goodglick L, Elashoff DA, Luo J et al (2009) Snail promotes CXCR2 ligand-dependent tumor progression in non-small cell lung carcinoma. *Clin Cancer Res* 15**:** 6820-6829

Zhang J, Ma L (2012) MicroRNA control of epithelial-mesenchymal transition and metastasis. *Cancer Metastasis Rev* 31**:** 653-662

Zhu L, Qin H, Li PY, Xu SN, Pang HF, Zhao HZ, Li DM, Zhao Q (2012) Response gene to complement-32 enhances metastatic phenotype by mediating transforming growth factor beta-induced epithelial-mesenchymal transition in human pancreatic cancer cell line BxPC-3. *J Exp Clin Cancer Res* 31**:** 29
