# Supplementary material for: Epithelial-mesenchymal transition spectrum quantification and its efficacy in deciphering survival and drug responses of cancer patients
Source: EMBO Mol Med. 2014 Sep 11;6(10):1279–93. doi: 10.15252/emmm.201404208 (PMC4287932; doi:10.15252/emmm.201404208)
Supplement: Supplementary file 2 [file emmm0006-1279-sd2.pdf]

Table S1A. Generic EMT signature for tumour

Index	Gene Symbol	Gene Title	GO Molecular Function	Weight	Weight.Ztransformed	Category	In Generic Cell Line EMT Signature = 1
1	KRT19	Keratin, type I cytoskeletal 19	structural constituent of cytoskeleton	2.37	12.68	Epi	1
2	AGR2	Anterior gradient protein 2 homolog	oxidoreductase activity	2.34	12.52	Epi	1
3	RAB25	Ras-related protein Rab-25	---	2.31	12.37	Epi	1
4	CDH1	cadherin 1, type 1, E-cadherin (epithelial)	calcium ion binding	2.09	11.12	Epi	1
5	ERBB3	Receptor tyrosine-protein kinase erbB-3	non-membrane spanning protein tyrosine kinase activity;transmembrane receptor protein kinase activity;transmembrane receptor protein tyrosine kinase activity;transmembrane receptor protein kinase activity;growth factor activity	1.95	10.31	Epi	1
6	FXYD3	FXYD domain-containing ion transport regulator 3	ion channel activity;protein binding	1.91	10.10	Epi	1
7	SLC44A4	solute carrier family 44, member 4	phospholipid metabolic process	1.86	9.84	Epi	0
8	S100P	Protein S100-P	calcium ion binding;receptor binding;calmodulin binding	1.72	9.05	Epi	1
9	SCNN1A	Amiloride-sensitive sodium channel subunit alpha	ion channel activity	1.68	8.78	Epi	1
10	GALNT3	Polypeptide N-acetylgalactosaminyltransferase 3	transferase activity, transferring glycosyl groups	1.67	8.75	Epi	1
11	PRSS8	Prostasin	serine-type peptidase activity	1.61	8.39	Epi	1
12	ELF3	ETS-related transcription factor Elf-3	transcription factor activity;receptor binding;transcription factor activity	1.59	8.32	Epi	1
13	CEACAM6	Carcinoembryonic antigen-related cell adhesion molecule 6	---	1.58	8.24	Epi	1
14	TMPRSS4	Transmembrane protease serine 4	serine-type peptidase activity	1.56	8.14	Epi	1
15	CLDN7	Claudin-7	---	1.56	8.14	Epi	1
16	TACSTD2	Tumour-associated calcium signal transducer 2	receptor activity	1.56	8.10	Epi	1
17	CLDN3	Claudin-3	---	1.54	8.03	Epi	0
18	EPCAM	Epithelial cell adhesion molecule	receptor activity	1.52	7.92	Epi	1
19	SPINT1	Kunitz-type protease inhibitor 1	protein binding;serine-type endopeptidase inhibitor activity	1.46	7.56	Epi	1
20	TSPAN1	Tetraspanin-1	receptor activity;receptor binding	1.43	7.41	Epi	1
21	PLS1	Phospholipid scramblase 1	---	1.43	7.39	Epi	1
22	TMEM30B	Cell cycle control protein 50B	---	1.43	7.38	Epi	1
23	PRR15L	Proline-rich protein 15-like protein	---	1.41	7.26	Epi	0
24	KRT8	Keratin, type II cytoskeletal 8	structural constituent of cytoskeleton	1.40	7.20	Epi	1
25	ST14	Suppressor of Tumourigenicity 14 protein	serine-type peptidase activity;receptor activity;calcium ion binding;hormone activity;calmodulin binding;calcium-dependent phospholipid binding;peptidase inhibitor activity	1.38	7.11	Epi	1
26	RBM47	RNA-binding protein 47	RNA splicing factor activity, transesterification mechanism;structural constituent of ribosome;DNA replication origin binding;single-stranded DNA binding;poly(A) RNA binding	1.37	7.03	Epi	1
27	S100A14	Protein S100-A14	calcium ion binding;receptor binding;calmodulin binding	1.36	7.01	Epi	1
28	C1orf106	Uncharacterized protein C1orf106	---	1.36	7.00	Epi	1
29	NQO1	NAD(P)H dehydrogenase [quinone] 1	---	1.36	7.00	Epi	0
30	TOX3	TOX high mobility group box family member 3	transcription factor activity;chromatin binding;receptor binding;transcription factor activity	1.36	6.99	Epi	1
31	PTK6	Protein-tyrosine kinase 6	non-membrane spanning protein tyrosine kinase activity;transmembrane receptor protein kinase activity;transmembrane receptor protein tyrosine kinase activity;transmembrane receptor protein kinase activity;growth factor activity	1.35	6.95	Epi	1
32	TFF1	Trefoil factor 1	cytokine activity;hormone activity;growth factor activity	1.34	6.87	Epi	0

33	CLDN4	Claudin-4	---	1.34	6.87	Epi	1
34	GPRC5A	Retinoic acid-induced protein 3	G-protein coupled receptor activity	1.34	6.87	Epi	0
35	TJP3	Tight junction protein ZO-3	---	1.33	6.85	Epi	0
36	KRT18	Keratin, type I cytoskeletal 18	structural constituent of cytoskeleton	1.31	6.73	Epi	1
37	MAP7	Ensconsin	structural constituent of cytoskeleton;microtubule binding	1.28	6.56	Epi	1
38	CKMT1A	creatine kinase, mitochondrial 1A	nucleotide binding	1.27	6.51	Epi	1
39	ESRP1	Epithelial splicing regulatory protein 1	structural constituent of ribosome;nucleic acid binding	1.27	6.47	Epi	1
40	MUC1	mucin 1, cell surface associated	RNA polymerase II core promoter proximal region sequence-specific DNA binding	1.25	6.40	Epi	0
41	SPINT2	Kunitz-type protease inhibitor 2	protein binding;serine-type endopeptidase inhibitor activity	1.23	6.25	Epi	1
42	ESRP2	Epithelial splicing regulatory protein 2	structural constituent of ribosome;nucleic acid binding	1.22	6.20	Epi	1
43	CDS1	Phosphatidate cytidylyltransferase 1	nucleotidyltransferase activity	1.21	6.14	Epi	1
44	PPAP2C	Lipid phosphate phosphohydrolase 2	pyrophosphatase activity	1.20	6.08	Epi	0
45	CEACAM7	Carcinoembryonic antigen-related cell adhesion molecule 7	---	1.19	6.05	Epi	0
46	TTC39A	Tetratricopeptide repeat protein 39A	---	1.19	6.02	Epi	1
47	OVOL2	Transcription factor Ovo-like 2	transcription factor activity;transcription factor activity	1.17	5.90	Epi	1
48	EHF	ETS homologous factor	transcription factor activity;receptor binding;transcription factor activity	1.16	5.88	Epi	1
49	AP1M2	AP-1 complex subunit mu-2	---	1.15	5.83	Epi	1
50	CEACAM5	Carcinoembryonic antigen-related cell adhesion molecule 5	---	1.14	5.75	Epi	0
51	LAD1	Ladinin-1	structural molecule activity	1.13	5.70	Epi	1
52	ARHGAP8	Rho GTPase-activating protein 8	protein binding;small GTPase regulator activity	1.12	5.63	Epi	1
53	TFF3	trefoil factor 3 (intestinal)	molecular_function	1.09	5.49	Epi	0
54	JUP	junction plakoglobin	transcription coactivator activity	1.09	5.47	Epi	1
55	CD24	CD24 Molecular	signal transducer activity	1.08	5.45	Epi	0
56	TMC5	Transmembrane channel-like protein 5	ion channel activity	1.07	5.39	Epi	0
57	MLPH	Melanophilin	motor activity;structural constituent of cytoskeleton;actin binding;small GTPase regulator activity	1.07	5.38	Epi	0
58	ELMO3	engulfment and cell motility 3	binding	1.06	5.33	Epi	1
59	ERBB2	Receptor tyrosine-protein kinase erbB-2	non-membrane spanning protein tyrosine kinase activity;transmembrane receptor protein kinase activity;transmembrane receptor protein tyrosine kinase activity;transmembrane receptor protein kinase activity;growth factor activity	1.03	5.16	Epi	1
60	LLGL2	Lethal(2) giant larvae protein homolog 2	---	1.02	5.11	Epi	1
61	DDR1	Epithelial discoidin domain-containing receptor 1	non-membrane spanning protein tyrosine kinase activity;transmembrane receptor protein kinase activity;transmembrane receptor protein tyrosine kinase activity;transmembrane receptor protein kinase activity;growth factor activity	1.02	5.10	Epi	1
62	FA2H	Fatty acid 2-hydroxylase	oxidoreductase activity	1.02	5.06	Epi	1
63	CBLC	Signal transduction protein CBL-C	ligase activity	1.00	4.95	Epi	1
64	TMPRSS2	Transmembrane protease serine 2	serine-type peptidase activity;receptor activity;calcium ion binding;hormone activity;calmodulin binding;calcium-dependent phospholipid binding;peptidase inhibitor activity	1.00	4.95	Epi	1
65	LSR	Lipolysis-stimulated lipoprotein receptor	---	0.99	4.93	Epi	1
66	PERP	p53 apoptosis effector related to PMP-22	---	0.98	4.86	Epi	1
67	POF1B	Protein POF1B	---	0.97	4.81	Epi	1
68	MYO5C	Unconventional myosin-Vc	motor activity;structural constituent of cytoskeleton;protein binding;small GTPase regulator activity	0.97	4.80	Epi	1
69	RAB11FIP1	Rab11 family-interacting protein 1	---	0.96	4.76	Epi	1

70	MAPK13	Mitogen-activated protein kinase 13	protein kinase activity	0.96	4.75	Epi	1
71	KRT7	Keratin, type II cytoskeletal 7	structural constituent of cytoskeleton	0.96	4.74	Epi	1
72	CEACAM1	Carcinoembryonic antigen-related cell adhesion molecule 1	---	0.95	4.69	Epi	1
73	CXADR	Coxsackievirus and adenovirus receptor	receptor activity	0.95	4.68	Epi	0
74	ATP2C2	Calcium-transporting ATPase type 2C member 2	hydrolase activity;cation transmembrane transporter activity;ion channel activity	0.94	4.65	Epi	1
75	RNF128	E3 ubiquitin-protein ligase RNF128	ubiquitin-protein ligase activity	0.94	4.64	Epi	0
76	MPZL2	Myelin protein zero-like protein 2	voltage-gated sodium channel activity;voltage-gated sodium channel activity;cation channel activity	0.94	4.61	Epi	1
77	EPS8L1	Epidermal growth factor receptor kinase substrate 8-like protein 1	---	0.93	4.58	Epi	1
78	GALNT7	N-acetylgalactosaminyltransferase 7	transferase activity, transferring glycosyl groups	0.91	4.45	Epi	0
79	CORO2A	Coronin-2A	structural constituent of cytoskeleton;actin binding	0.90	4.43	Epi	0
80	BCAS1	Breast carcinoma-amplified sequence 1	---	0.90	4.41	Epi	0
81	TPD52	Tumour protein D52	---	0.89	4.36	Epi	0
82	ARHGAP32	Rho GTPase-activating protein 32	protein binding;small GTPase regulator activity	0.87	4.26	Epi	0
83	FUT2	Galactoside 2-alpha-L-fucosyltransferase 2	transferase activity, transferring glycosyl groups	0.87	4.25	Epi	0
84	OR7E14P	olfactory receptor, family 7, subfamily E, member 14 pseudogene	---	0.87	4.22	Epi	1
85	GALE	UDP-glucose 4-epimerase	oxidoreductase activity;hydro-lyase activity;racemase and epimerase activity	0.86	4.16	Epi	0
86	GRHL2	Grainyhead-like protein 2 homolog	transcription factor activity;transcription factor activity	0.85	4.14	Epi	1
87	BIK	Bcl-2-interacting killer	---	0.85	4.10	Epi	1
88	RAPGEFL1	Rap guanine nucleotide exchange factor-like 1	protein binding;small GTPase regulator activity;guanyl-nucleotide exchange factor activity	0.84	4.07	Epi	0
89	STYK1	Tyrosine-protein kinase STYK1	non-membrane spanning protein tyrosine kinase activity;transmembrane receptor protein kinase activity;transmembrane receptor protein tyrosine kinase activity;transmembrane receptor protein kinase activity	0.84	4.05	Epi	0
90	F11R	Junctional adhesion molecule A	receptor activity	0.84	4.04	Epi	1
91	PKP3	Plakophilin-3	structural constituent of cytoskeleton;intermediate filament binding	0.83	4.03	Epi	1
92	CYB561	Cytochrome b561	oxidoreductase activity	0.83	4.03	Epi	1
93	SH3YL1	SH3 domain-containing YSC84-like protein 1	structural constituent of cytoskeleton;actin binding	0.82	3.95	Epi	1
94	GDF15	Growth/differentiation factor 15	growth factor activity	0.81	3.93	Epi	0
95	PSCA	prostate stem cell antigen	---	0.81	3.92	Epi	0
96	EZR	Ezrin	structural constituent of cytoskeleton	0.81	3.88	Epi	0
97	TJP2	Tight junction protein ZO-2	---	0.80	3.87	Epi	1
98	FGFR3	Fibroblast growth factor receptor 3	non-membrane spanning protein tyrosine kinase activity;transmembrane receptor protein kinase activity;transmembrane receptor protein tyrosine kinase activity;transmembrane receptor protein kinase activity;growth factor activity	0.80	3.86	Epi	0
99	FUT3	Galactoside 3(4)-L-fucosyltransferase	transferase activity, transferring glycosyl groups	0.80	3.85	Epi	0
100	BSPRY	B box and SPRY domain-containing protein	ubiquitin-protein ligase activity;transcription factor activity;RNA binding;transcription factor activity;transcription cofactor activity	0.80	3.84	Epi	1
101	TOM1L1	TOM1-like protein 1	structural constituent of cytoskeleton;transmembrane transporter activity;protein binding;kinase activator activity;kinase regulator activity	0.80	3.82	Epi	0
102	IRF6	Interferon regulatory factor 6	transcription factor activity;transcription factor activity	0.78	3.74	Epi	1
103	EPB41L4B	Band 4.1-like protein 4B	---	0.78	3.74	Epi	0
104	SPDEF	SAM pointed domain-containing Ets transcription factor	transcription factor activity;receptor binding;transcription factor activity	0.78	3.72	Epi	0

105	OCLN	Occludin	transcription factor activity;transcription factor activity;transcription cofactor activity	0.77	3.70	Epi	1
106	LRRC1	Leucine-rich repeat-containing protein 1	adenylate cyclase activity;receptor activity;growth factor activity;kinase regulator activity	0.77	3.65	Epi	0
107	C19orf21	Uncharacterized protein C19orf21	---	0.76	3.64	Epi	1
108	ABHD11	Abhydrolase domain-containing protein 11	serine-type peptidase activity	0.75	3.57	Epi	0
109	EPS8L2	Epidermal growth factor receptor kinase substrate 8-like protein 2	---	0.75	3.55	Epi	1
110	MYO6	Unconventional myosin-VI	motor activity;structural constituent of cytoskeleton;protein binding;small GTPase regulator activity	0.75	3.55	Epi	1
111	TSPAN8	Tetraspanin-8	receptor activity;receptor binding	0.74	3.53	Epi	0
112	MST1R	Macrophage-stimulating protein receptor	non-membrane spanning protein tyrosine kinase activity;transmembrane receptor protein kinase activity;transmembrane receptor protein tyrosine kinase activity;transmembrane receptor protein kinase activity;growth factor activity	0.74	3.50	Epi	1
113	SLC16A5	Monocarboxylate transporter 6	transmembrane transporter activity	0.74	3.50	Epi	0
114	GPR56	G-protein coupled receptor 56	G-protein coupled receptor activity	0.73	3.47	Epi	1
115	AZGP1	Zinc-alpha-2-glycoprotein	receptor activity	0.73	3.47	Epi	0
116	TOB1	Protein Tob1	---	0.73	3.43	Epi	1
117	SLC35A3	UDP-N-acetylglucosamine transporter	transmembrane transporter activity	0.72	3.42	Epi	0
118	TRPM4	Transient receptor potential cation channel subfamily M member 4	receptor activity;ion channel activity	0.72	3.42	Epi	0
119	PHLDA2	Pleckstrin homology-like domain family A member 2	---	0.72	3.42	Epi	0
120	VAMP8	Vesicle-associated membrane protein 8	SNAP receptor activity	0.72	3.42	Epi	1
121	SLC22A18	Solute carrier family 22 member 18	transmembrane transporter activity	0.72	3.42	Epi	0
122	AKR1B10	Aldo-keto reductase family 1 member B10	oxidoreductase activity;transporter activity	0.72	3.40	Epi	0
123	VAV3	Guanine nucleotide exchange factor VAV3	receptor binding;small GTPase regulator activity;guanyl-nucleotide exchange factor activity	0.72	3.38	Epi	0
124	SPAG1	Sperm-associated antigen 1	---	0.72	3.38	Epi	0
125	ABCC3	ATP-binding cassette, sub-family C (CFTR/MRP), member	nucleotide binding	0.72	3.37	Epi	1
126	SYNGR2	Synaptogyrin-2	---	0.72	3.37	Epi	1
127	STAP2	Signal-transducing adaptor protein 2	transmembrane receptor protein kinase activity;transmembrane receptor protein kinase activity	0.70	3.28	Epi	1
128	C4orf19	Uncharacterized protein C4orf19	---	0.70	3.28	Epi	1
129	PPL	Periplakin	structural constituent of cytoskeleton;intermediate filament binding	0.70	3.27	Epi	1
130	PLLP	Plasmolipin	structural constituent of myelin sheath	0.70	3.26	Epi	0
131	DSG2	Desmoglein-2	calcium ion binding	0.70	3.26	Epi	0
132	HDHD3	Haloacid dehalogenase-like hydrolase domain-containing protein 3	hydrolase activity	0.69	3.25	Epi	0
133	CD2AP	CD2-associated protein	receptor binding	0.69	3.25	Epi	0
134	MANSC1	MANSC domain-containing protein 1	---	0.69	3.24	Epi	1
135	DHCR24	Delta(24)-sterol reductase	oxidoreductase activity	0.69	3.24	Epi	0
136	EPN3	Epsin-3	---	0.69	3.23	Epi	1
137	TUFT1	Tuftelin	---	0.69	3.22	Epi	0
138	GMDS	GDP-mannose 4,6 dehydratase	oxidoreductase activity;hydro-lyase activity;racemase and epimerase activity	0.69	3.22	Epi	0
139	EXPH5	Exophilin-5	---	0.68	3.18	Epi	1
140	DSP	Dentin phosphoprotein	---	0.68	3.17	Epi	1
141	SDC4	Syndecan-4	structural constituent of cytoskeleton;receptor binding	0.68	3.17	Epi	0
142	IL20RA	Interleukin-20 receptor subunit alpha	interferon receptor activity	0.67	3.13	Epi	1

143	FAM174B	family with sequence similarity 174, member B	---	0.67	3.13	Epi	0
144	PTPRF	Receptor-type tyrosine-protein phosphatase F	phosphoprotein phosphatase activity;phosphoprotein phosphatase activity;receptor activity	0.67	3.11	Epi	0
145	SORD	Sorbitol dehydrogenase	oxidoreductase activity	0.67	3.10	Epi	0
146	GAS1	Growth arrest-specific protein 1	---	1.62	8.43	Mes	0
147	CXCL12	SDF-1-alpha(3-67)	---	1.38	7.13	Mes	0
148	ZEB1	Zinc finger E-box-binding homeobox 1	---	1.28	6.56	Mes	1
149	GLYR1	Putative oxidoreductase GLYR1	oxidoreductase activity	1.21	6.18	Mes	1
150	FHL1	Four and a half LIM domains protein 1	structural constituent of cytoskeleton;transcription factor activity;RNA binding;transcription factor activity	1.21	6.15	Mes	1
151	FERMT2	Fermitin family homolog 2	---	1.19	6.06	Mes	1
152	C1S	Complement C1s subcomponent	oxidoreductase activity;serine-type peptidase activity;calcium ion binding;calmodulin binding;calcium-dependent phospholipid binding	1.15	5.84	Mes	0
153	FYN	Tyrosine-protein kinase Fyn	non-membrane spanning protein tyrosine kinase activity;transmembrane receptor protein kinase activity;transmembrane receptor protein tyrosine kinase activity;transmembrane receptor protein kinase activity	1.15	5.84	Mes	0
154	WIPF1	WAS/WASL-interacting protein family member 1	---	1.15	5.80	Mes	0
155	CYP1B1	Cytochrome P450, family 1, subfamily B, polypeptide 1, isoform CRA_a	oxidoreductase activity	1.12	5.65	Mes	0
156	SERPING1	Plasma protease C1 inhibitor	protein binding;serine-type endopeptidase inhibitor activity	1.12	5.63	Mes	0
157	SERPINF1	Pigment epithelium-derived factor	protein binding;serine-type endopeptidase inhibitor activity	1.09	5.48	Mes	0
158	VCAM1	Vascular cell adhesion protein 1	phosphoprotein phosphatase activity;phosphoprotein phosphatase activity;receptor activity	1.08	5.40	Mes	0
159	MAP1B	MAP1 light chain LC1	structural constituent of cytoskeleton;microtubule binding	1.07	5.39	Mes	1
160	TCF4	Transcription factor 4	transcription factor activity;transcription factor activity	1.06	5.33	Mes	0
161	SRPX	Sushi repeat-containing protein SRPX	serine-type peptidase activity;metallopeptidase activity;receptor activity;lipid transporter activity	1.06	5.31	Mes	1
162	EMP3	Epithelial membrane protein 3	structural constituent of cytoskeleton	1.06	5.29	Mes	1
163	DPT	Dermatopontin	---	1.05	5.24	Mes	0
164	CALD1	Caldesmon	structural constituent of cytoskeleton;actin binding	1.04	5.21	Mes	1
165	PTGIS	Prostacyclin synthase	oxidoreductase activity;isomerase activity	1.02	5.11	Mes	0
166	VIM	Vimentin	structural constituent of cytoskeleton	1.01	5.04	Mes	1
167	CD163	Scavenger receptor cysteine-rich type 1 protein M130	oxidoreductase activity;serine-type peptidase activity;receptor activity	1.01	5.04	Mes	0
168	C1R	Complement C1r subcomponent heavy chain	oxidoreductase activity;serine-type peptidase activity;calcium ion binding;calmodulin binding;calcium-dependent phospholipid binding	1.01	5.02	Mes	0
169	FBN1	Fibrillin-1	receptor activity;extracellular matrix structural constituent;calcium ion binding;receptor binding;calcium-dependent phospholipid binding	1.01	5.02	Mes	0
170	FN1	Anastellin	hydrolase activity;deaminase activity;receptor binding;kinase regulator activity	1.00	4.94	Mes	0
171	FXYD6	FXYD domain-containing ion transport regulator 6	ion channel activity;protein binding	0.98	4.86	Mes	0

172	IGF1	Insulin-like growth factor I	hormone activity;growth factor activity	0.97	4.82	Mes	0
173	NAP1L3	Nucleosome assembly protein 1-like 3	protein binding;phosphatase inhibitor activity;phosphatase regulator activity	0.97	4.81	Mes	0
174	MRC1	Macrophage mannose receptor 1	receptor activity	0.96	4.74	Mes	0
175	QKI	Protein quaking	RNA splicing factor activity, transesterification mechanism;transcription factor activity;mRNA binding;transcription factor activity;transcription cofactor activity	0.95	4.67	Mes	0
176	MS4A4A	Membrane-spanning 4-domains subfamily A member 4A	receptor activity	0.94	4.65	Mes	0
177	DCN	Decorin	receptor activity	0.94	4.64	Mes	0
178	LOX	Protein-lysine 6-oxidase	oxidoreductase activity;serine-type peptidase activity;receptor activity	0.92	4.53	Mes	0
179	RECK	Reversion-inducing cysteine-rich protein with Kazal motifs	protein binding;serine-type endopeptidase inhibitor activity	0.92	4.53	Mes	1
180	ANK2	Ankyrin-2	structural constituent of cytoskeleton	0.92	4.53	Mes	1
181	LY96	Lymphocyte antigen 96	---	0.92	4.50	Mes	0
182	ZFPM2	Zinc finger protein ZFPM2	transcription factor activity;transcription factor activity	0.91	4.46	Mes	0
183	CSRP2	Cysteine and glycine-rich protein 2	structural constituent of cytoskeleton	0.91	4.45	Mes	0
184	EFEMP1	EGF-containing fibulin-like extracellular matrix protein 1	receptor activity;extracellular matrix structural constituent;calcium ion binding;receptor binding;calmodulin binding;calcium-dependent phospholipid binding	0.90	4.44	Mes	0
185	RARRES2	Retinoic acid receptor responder protein 2	---	0.90	4.38	Mes	0
186	PTPRC	Receptor-type tyrosine-protein phosphatase C	phosphoprotein phosphatase activity;phosphoprotein phosphatase activity;receptor activity	0.89	4.34	Mes	0
187	PLEKHO1	Pleckstrin homology domain-containing family O member 1	---	0.89	4.33	Mes	0
188	RGS2	Regulator of G-protein signaling 2	protein binding;small GTPase regulator activity	0.89	4.32	Mes	0
189	F13A1	Coagulation factor XIII A chain	acyltransferase activity	0.88	4.31	Mes	0
190	JAM2	Junctional adhesion molecule B	receptor activity	0.87	4.25	Mes	0
191	CHRDL1	Chordin-like protein 1	receptor binding	0.87	4.22	Mes	0
192	TUBA1A	Tubulin alpha-1A chain	structural constituent of cytoskeleton	0.87	4.21	Mes	1
193	AP1S2	AP-1 complex subunit sigma-2	---	0.86	4.19	Mes	1
194	MYLK	Myosin light chain kinase, smooth muscle	protein kinase activity	0.86	4.17	Mes	0
195	DDR2	Discoidin domain-containing receptor 2	non-membrane spanning protein tyrosine kinase activity;transmembrane receptor protein kinase activity;transmembrane receptor protein tyrosine kinase activity;transmembrane receptor protein kinase activity;growth factor activity	0.86	4.17	Mes	0
196	DSE	Dermatan-sulfate epimerase	---	0.86	4.17	Mes	0
197	SACS	Sacsin	---	0.86	4.17	Mes	1
198	GLIPR1	Glioma pathogenesis-related protein 1	---	0.86	4.16	Mes	0
199	CXCL13	C-X-C motif chemokine 13	chemokine activity	0.85	4.15	Mes	0
200	FLRT2	Leucine-rich repeat transmembrane protein FLRT2	receptor activity	0.85	4.13	Mes	0
201	PTX3	Pentraxin-related protein PTX3	---	0.85	4.12	Mes	0
202	AKT3	RAC-gamma serine/threonine-protein kinase	protein kinase activity;calcium ion binding;calmodulin binding;calcium-dependent phospholipid binding	0.84	4.10	Mes	0
203	COL6A2	Collagen alpha-2(VI) chain	receptor activity;extracellular matrix structural constituent;transmembrane transporter activity	0.84	4.07	Mes	0
204	DPYSL3	Dihydropyrimidinase-related protein 3	hydrolase activity	0.84	4.06	Mes	0
205	CDH11	Cadherin-11	G-protein coupled receptor activity;calcium ion binding	0.83	4.04	Mes	0
206	PDZRN3	E3 ubiquitin-protein ligase PDZRN3	---	0.83	4.02	Mes	0
207	ZEB2	Zinc finger E-box-binding homeobox 2	---	0.83	4.01	Mes	0

208	CCL2	C-C motif chemokine 2	chemokine activity	0.83	4.01	Mes	0
209	MAFB	Transcription factor MafB	transcription factor activity;transcription factor activity	0.83	3.99	Mes	0
210	SFRP1	Secreted frizzled-related protein 1	G-protein coupled receptor activity;receptor binding	0.82	3.98	Mes	0
211	C14orf139	chromosome 14 open reading frame 139	---	0.82	3.97	Mes	0
212	MFAP4	Microfibril-associated glycoprotein 4	hydrolase activity;deaminase activity;receptor binding;kinase regulator activity	0.82	3.96	Mes	0
213	MAF	Transcription factor Maf	transcription factor activity;transcription factor activity	0.82	3.95	Mes	0
214	UCHL1	Ubiquitin carboxyl-terminal hydrolase isozyme L1	cysteine-type peptidase activity	0.82	3.94	Mes	0
215	TUBB6	Tubulin beta-6 chain	structural constituent of cytoskeleton	0.82	3.93	Mes	1
216	SRGN	Serglycin	---	0.81	3.91	Mes	0
217	HEG1	Protein HEG homolog 1	---	0.81	3.91	Mes	0
218	KCNJ8	ATP-sensitive inward rectifier potassium channel 8	voltage-gated potassium channel activity;voltage-gated potassium channel activity;cation channel activity	0.80	3.86	Mes	0
219	AKAP12	A-kinase anchor protein 12	---	0.80	3.85	Mes	1
220	EVI2A	Protein EVI2A	receptor activity	0.80	3.84	Mes	0
221	COL14A1	Collagen alpha-1(XIV) chain	receptor activity;extracellular matrix structural constituent;transmembrane transporter activity	0.80	3.84	Mes	0
222	AXL	Tyrosine-protein kinase receptor UFO	non-membrane spanning protein tyrosine kinase activity;transmembrane receptor protein kinase activity;transmembrane receptor protein tyrosine kinase activity;transmembrane receptor protein kinase activity;growth factor activity	0.80	3.82	Mes	1
223	ECM2	Extracellular matrix protein 2	receptor activity	0.79	3.81	Mes	0
224	FSTL1	Follistatin-related protein 1	protein binding	0.79	3.79	Mes	1
225	PLN	Cardiac phospholamban	---	0.79	3.77	Mes	0
226	MYL9	Myosin regulatory light polypeptide 9	structural constituent of cytoskeleton;calcium ion binding;calmodulin binding	0.79	3.77	Mes	1
227	OLFML3	Olfactomedin-like protein 3	receptor activity;structural molecule activity	0.78	3.76	Mes	0
228	STON1	Stonin-1	---	0.78	3.75	Mes	0
229	SLIT2	Slit homolog 2 protein	receptor activity	0.78	3.75	Mes	0
230	BICC1	Protein bicaudal C homolog 1	lipid transporter activity;RNA binding	0.78	3.75	Mes	0
231	SOBP	Sine oculis-binding protein homolog	---	0.78	3.75	Mes	1
232	CLIC4	Chloride intracellular channel protein 4	oxidoreductase activity;transferase activity;racemase and epimerase activity;structural constituent of cytoskeleton;anion channel activity;translation factor activity, nucleic acid binding;receptor binding;translation elongation factor activity	0.78	3.74	Mes	0
233	ENPP2	Ectonucleotide pyrophosphatase/phosphodiesterase family member 2	pyrophosphatase activity;nucleotide phosphatase activity;nucleotide phosphatase activity	0.78	3.73	Mes	0
234	SAMSN1	SAM domain-containing protein SAMSN-1	---	0.78	3.72	Mes	0
235	TPM2	Tropomyosin beta chain	motor activity;structural constituent of cytoskeleton	0.77	3.70	Mes	1
236	ASPN	Asporin	receptor activity	0.77	3.70	Mes	0
237	COL6A1	Collagen alpha-1(VI) chain	receptor activity;extracellular matrix structural constituent;transmembrane transporter activity	0.77	3.69	Mes	0
238	IGFBP5	Insulin-like growth factor-binding protein 5	---	0.77	3.68	Mes	0
239	MOXD1	DBH-like monooxygenase protein 1	oxidoreductase activity	0.77	3.68	Mes	0
240	AKAP2	A-kinase anchor protein 2	---	0.77	3.68	Mes	0
241	SLC2A3	Solute carrier family 2, facilitated glucose transporter member 3	carbohydrate transmembrane transporter activity;transmembrane transporter activity	0.77	3.67	Mes	0

242	OLFML2B	Olfactomedin-like protein 2B	receptor activity;structural molecule activity	0.77	3.65	Mes	0
243	ANGPTL2	Angiopoietin-related protein 2	hydrolase activity;deaminase activity;receptor binding;kinase regulator activity	0.76	3.64	Mes	0
244	PCOLCE	Procollagen C-endopeptidase enhancer 1	oxidoreductase activity;serine-type peptidase activity;metallopeptidase activity;receptor activity;lipid transporter activity;transmembrane transporter activity;receptor binding;enzyme regulator activity	0.76	3.62	Mes	0
245	COLEC12	Collectin-12	receptor activity;transmembrane transporter activity	0.76	3.62	Mes	0
246	CTSK	Cathepsin K	cysteine-type peptidase activity	0.76	3.62	Mes	0
247	TAGLN	Transgelin	structural constituent of cytoskeleton;actin binding	0.76	3.60	Mes	0
248	CDH2	Cadherin-2	G-protein coupled receptor activity;calcium ion binding	0.76	3.60	Mes	0
249	IL10RA	Interleukin-10 receptor subunit alpha	interferon receptor activity	0.76	3.60	Mes	0
250	C1orf54	chromosome 1 open reading frame 54	---	0.76	3.60	Mes	0
251	CEP170	Centrosomal protein of 170 kDa	---	0.76	3.59	Mes	1
252	TNS1	Tensin-1	phosphoprotein phosphatase activity;phosphoprotein phosphatase activity;structural constituent of cytoskeleton;actin binding	0.75	3.59	Mes	0
253	CLEC2B	C-type lectin domain family 2 member B	receptor activity;receptor binding	0.75	3.58	Mes	0
254	JAM3	Junctional adhesion molecule C	receptor activity	0.75	3.57	Mes	0
255	SEPT6	Septin-6	GTPase activity;structural constituent of cytoskeleton;protein binding	0.75	3.56	Mes	0
256	GREM1	Gremlin-1	---	0.75	3.55	Mes	0
257	VCAN	Versican core protein	---	0.75	3.54	Mes	0
258	ZCCHC24	zinc finger, CCHC domain containing 24	---	0.75	3.54	Mes	0
259	CRYAB	Alpha-crystallin B chain	structural molecule activity	0.75	3.54	Mes	0
260	SFRP4	Secreted frizzled-related protein 4	G-protein coupled receptor activity;receptor binding	0.74	3.53	Mes	0
261	RUNX1T1	Protein CBFA2T1	transcription factor activity;transcription factor activity;transcription cofactor activity	0.74	3.52	Mes	0
262	FGL2	Fibroleukin	hydrolase activity;deaminase activity;receptor binding;kinase regulator activity	0.74	3.51	Mes	0
263	MS4A6A	Membrane-spanning 4-domains subfamily A member 6A	receptor activity	0.74	3.51	Mes	0
264	PTRF	Polymerase I and transcript release factor	transcription factor activity;transcription factor activity	0.74	3.48	Mes	1
265	GIMAP4	GTPase IMAP family member 4	---	0.73	3.48	Mes	0
266	TWIST1	Twist-related protein 1	transcription factor activity;transcription factor activity	0.73	3.48	Mes	0
267	GFPT2	Glucosamine--fructose-6-phosphate aminotransferase [isomerizing] 2	transaminase activity	0.73	3.47	Mes	1
268	LHFP	Lipoma HMGIC fusion partner	---	0.73	3.47	Mes	1
269	CXCR4	C-X-C chemokine receptor type 4	G-protein coupled receptor activity	0.73	3.47	Mes	0
270	SPOCK1	Testican-1	calcium ion binding;protein binding;cysteine-type endopeptidase inhibitor activity	0.73	3.46	Mes	0
271	SPARC	SPARC	growth factor activity	0.73	3.46	Mes	1
272	VSIG4	V-set and immunoglobulin domain-containing protein 4	---	0.73	3.44	Mes	0
273	GPM6B	Neuronal membrane glycoprotein M6-b	structural constituent of myelin sheath	0.72	3.42	Mes	0
274	TRPC1	Short transient receptor potential channel 1	ion channel activity	0.72	3.41	Mes	1

275	SNAI2	Zinc finger protein SNAI2	transcription factor activity;transcription factor activity	0.72	3.39	Mes	0
276	GUCY1B3	guanylate cyclase 1, soluble, beta 3	nucleotide binding	0.72	3.37	Mes	0
277	PLXNC1	Plexin-C1	transmembrane receptor protein kinase activity;transmembrane receptor protein tyrosine kinase activity;transmembrane receptor protein kinase activity;receptor binding	0.71	3.36	Mes	0
278	SYT11	Synaptotagmin-11	---	0.71	3.36	Mes	0
279	FLI1	Friend Leukaemia integration 1 transcription factor	transcription factor activity;receptor binding;transcription factor activity	0.71	3.36	Mes	0
280	MYH10	Myosin-10	motor activity;structural constituent of cytoskeleton;protein binding;small GTPase regulator activity	0.71	3.35	Mes	0
281	CSF2RB	Cytokine receptor common subunit beta	cytokine receptor activity	0.71	3.34	Mes	0
282	TNC	Tenascin	hydrolase activity;deaminase activity;receptor binding;kinase regulator activity	0.71	3.34	Mes	0
283	PMP22	Peripheral myelin protein 22	structural constituent of cytoskeleton	0.71	3.34	Mes	1
284	COL5A2	Collagen alpha-2(V) chain	receptor activity;extracellular matrix structural constituent;transmembrane transporter activity	0.71	3.34	Mes	1
285	MMP2	72 kDa type IV collagenase	metallopeptidase activity	0.71	3.33	Mes	0
286	GNG11	Guanine nucleotide-binding protein G(I)/G(S)/G(O) subunit gamma-11	GTPase activity;protein binding	0.71	3.33	Mes	0
287	CAV1	Caveolin-1	structural molecule activity;protein binding;small GTPase regulator activity	0.71	3.31	Mes	0
288	CDK14	Cyclin-dependent kinase 14	non-membrane spanning protein tyrosine kinase activity	0.70	3.30	Mes	0
289	SDC2	Syndecan-2	structural constituent of cytoskeleton;receptor binding	0.70	3.30	Mes	0
290	PTGDS	Prostaglandin-H2 D-isomerase	isomerase activity;binding	0.70	3.30	Mes	0
291	NR3C1	Glucocorticoid receptor	ligand-dependent nuclear receptor activity;transcription factor activity;transcription factor activity	0.70	3.29	Mes	0
292	SYNM	Probable asparagine--tRNA ligase, mitochondrial	aminoacyl-tRNA ligase activity;RNA binding	0.70	3.29	Mes	0
293	FAP	Glomulin	structural molecule activity;receptor binding	0.70	3.28	Mes	0
294	NUAK1	NUAK family SNF1-like kinase 1	protein kinase activity	0.70	3.28	Mes	0
295	WWTR1	WW domain-containing transcription regulator protein 1	transcription factor activity;protein binding;kinase regulator activity;transcription factor activity;transcription cofactor activity	0.70	3.26	Mes	0
296	FBLN1	Fibulin-1	receptor activity;extracellular matrix structural constituent;calcium ion binding;receptor binding;calmodulin binding;calcium-dependent phospholipid binding	0.70	3.26	Mes	0
297	MPDZ	Multiple PDZ domain protein	structural constituent of cytoskeleton	0.69	3.23	Mes	0
298	SYNE1	Nesprin-1	structural constituent of cytoskeleton;calcium ion binding;actin binding	0.69	3.23	Mes	0
299	EFEMP2	EGF-containing fibulin-like extracellular matrix protein 2	receptor activity;extracellular matrix structural constituent;calcium ion binding;receptor binding;calmodulin binding;calcium-dependent phospholipid binding	0.69	3.22	Mes	0
300	GIMAP6	GTPase IMAP family member 6	---	0.69	3.20	Mes	0
301	KIAA1462	---	cell adhesion	0.69	3.20	Mes	0
302	CCL8	C-C motif chemokine 8	chemokine activity	0.69	3.20	Mes	0
303	COL15A1	Collagen alpha-1(XV) chain	receptor activity;extracellular matrix structural constituent;transmembrane transporter activity	0.69	3.20	Mes	0
304	CHN1	N-chimaerin	protein binding;small GTPase regulator activity	0.68	3.20	Mes	1

305	CRISPLD2	Cysteine-rich secretory protein LCCL domain-containing 2	---	0.68	3.19	Mes	0
306	PDGFC	Platelet-derived growth factor C	oxidoreductase activity;serine-type peptidase activity;metallopeptidase activity;receptor activity;lipid transporter activity;transmembrane transporter activity;growth factor activity;enzyme regulator activity	0.68	3.18	Mes	0
307	GEM	GTP-binding protein GEM	GTPase activity;protein binding	0.68	3.17	Mes	0
308	ISLR	Immunoglobulin superfamily containing leucine-rich repeat protein	receptor activity	0.68	3.14	Mes	0
309	GZMK	Granzyme K	serine-type peptidase activity;calcium ion binding;growth factor activity;calmodulin binding;calcium-dependent phospholipid binding	0.67	3.14	Mes	0
310	SPARCL1	SPARC-like protein 1	growth factor activity	0.67	3.14	Mes	0
311	BNC2	Zinc finger protein basonuclin-2	transcription factor activity;transcription factor activity	0.67	3.12	Mes	0
312	BGN	Biglycan	receptor activity	0.67	3.12	Mes	0
313	MEOX2	Homeobox protein MOX-2	transcription factor activity;transcription factor activity	0.67	3.10	Mes	0
314	ITM2A	Integral membrane protein 2A	---	0.67	3.10	Mes	0
315	IFFO1	Intermediate filament family orphan 1	---	0.67	3.10	Mes	0

Table S1B. Generic EMT signature for cell line

Index	Gene Symbol	Gene Title	GO Molecular Function	Weight	Weight.Ztransformed	Epi/Mes	In Generic Tumour EMT Signature = 1
1	CDH1	cadherin 1, type 1, E-cadherin (epithelial)	calcium ion binding	4.99	18.83	Epi	1
2	AGR2	Anterior gradient protein 2 homolog	oxidoreductase activity	4.65	17.53	Epi	1
3	EPCAM	Epithelial cell adhesion molecule	receptor activity	4.44	16.72	Epi	1
4	KRT19	Keratin, type I cytoskeletal 19	structural constituent of cytoskeleton	4.39	16.52	Epi	1
5	RAB25	Ras-related protein Rab-25	---	4.36	16.41	Epi	1
6	TACSTD2	Tumour-associated calcium signal transducer 2	receptor activity	4.07	15.26	Epi	1
7	S100P	Protein S100-P	calcium ion binding;receptor binding;calmodulin binding	3.69	13.79	Epi	1
8	CEACAM6	Carcinoembryonic antigen-related cell adhesion molecule 6	---	3.57	13.34	Epi	1
9	GALNT3	Polypeptide N-acetylgalactosaminyltransferase 3	transferase activity, transferring glycosyl groups	3.54	13.22	Epi	1
10	FXYD3	FXYD domain-containing ion transport regulator 3	ion channel activity;protein binding	3.48	13.01	Epi	1
11	SPINT2	Kunitz-type protease inhibitor 2	protein binding;serine-type endopeptidase inhibitor activity	3.33	12.40	Epi	1
12	TMEM30B	Cell cycle control protein 50B	---	3.21	11.94	Epi	1
13	SCNN1A	Amiloride-sensitive sodium channel subunit alpha	ion channel activity	3.08	11.45	Epi	1
14	ST14	Suppressor of Tumourigenicity 14 protein	serine-type peptidase activity;receptor activity;calcium ion binding;hormone activity;calmodulin binding;calcium-dependent phospholipid binding;peptidase inhibitor activity	3.03	11.26	Epi	1
15	ESRP1	Epithelial splicing regulatory protein 1	structural constituent of ribosome;nucleic acid binding	3.02	11.24	Epi	1
16	S100A14	Protein S100-A14	calcium ion binding;receptor binding;calmodulin binding	2.93	10.88	Epi	1
17	CLDN7	Claudin-7	---	2.90	10.76	Epi	1
18	ERBB3	Receptor tyrosine-protein kinase erbB-3	non-membrane spanning protein tyrosine kinase activity;transmembrane receptor protein kinase activity;transmembrane receptor protein tyrosine kinase activity;transmembrane receptor protein kinase activity;growth factor activity	2.89	10.72	Epi	1
19	RBM47	RNA-binding protein 47	RNA splicing factor activity, transesterification mechanism;structural constituent of ribosome;DNA replication origin binding;single-stranded DNA binding;poly(A) RNA binding	2.82	10.44	Epi	1
20	SPINT1	Kunitz-type protease inhibitor 1	protein binding;serine-type endopeptidase inhibitor activity	2.79	10.32	Epi	1
21	ELF3	ETS-related transcription factor Elf-3	transcription factor activity;receptor binding;transcription factor activity	2.73	10.08	Epi	1
22	CLDN4	Claudin-4	---	2.68	9.91	Epi	1
23	PRSS8	Prostasin	serine-type peptidase activity	2.66	9.84	Epi	1
24	SH3YL1	SH3 domain-containing YSC84-like protein 1	structural constituent of cytoskeleton;actin binding	2.64	9.75	Epi	1
25	EHF	ETS homologous factor	transcription factor activity;receptor binding;transcription factor activity	2.57	9.46	Epi	1
26	LCN2	Neutrophil gelatinase-associated lipocalin	isomerase activity;binding	2.54	9.35	Epi	0
27	JUP	junction plakoglobin	transcription coactivator activity	2.52	9.28	Epi	1

28	VAMP8	Vesicle-associated membrane protein 8	SNAP receptor activity	2.52	9.28	Epi	1
29	KRT8	Keratin, type II cytoskeletal 8	structural constituent of cytoskeleton	2.41	8.86	Epi	1
30	C1orf106	Uncharacterized protein C1orf106	---	2.29	8.39	Epi	1
31	KRT7	Keratin, type II cytoskeletal 7	structural constituent of cytoskeleton	2.29	8.38	Epi	1
32	DSP	Dentin phosphoprotein	---	2.28	8.35	Epi	1
33	CDS1	Phosphatidate cytidylyltransferase 1	nucleotidyltransferase activity	2.26	8.28	Epi	1
34	ITGB4	Integrin beta-4	receptor activity	2.22	8.11	Epi	0
35	TMPRSS4	Transmembrane protease serine 4	serine-type peptidase activity	2.20	8.03	Epi	1
36	LSR	Lipolysis-stimulated lipoprotein receptor	---	2.18	7.95	Epi	1
37	SORL1	Sortilin-related receptor	receptor activity;transmembrane transporter activity;lipid binding	2.16	7.90	Epi	0
38	GRHL2	Grainyhead-like protein 2 homolog	transcription factor activity;transcription factor activity	2.16	7.89	Epi	1
39	PPL	Periplakin	structural constituent of cytoskeleton;intermediate filament binding	2.15	7.84	Epi	1
40	C1orf116	Specifically androgen-regulated gene protein	---	2.14	7.83	Epi	0
41	TSPAN1	Tetraspanin-1	receptor activity;receptor binding	2.13	7.76	Epi	1
42	MAP7	Ensconsin	structural constituent of cytoskeleton;microtubule binding	2.09	7.61	Epi	1
43	SLPI	Antileukoproteinase	protein binding;serine-type endopeptidase inhibitor activity	2.03	7.41	Epi	0
44	TOX3	TOX high mobility group box family member 3	transcription factor activity;chromatin binding;receptor binding;transcription factor activity	1.99	7.24	Epi	1
45	ARHGAP8	Rho GTPase-activating protein 8	protein binding;small GTPase regulator activity	1.96	7.13	Epi	1
46	F11R	Junctional adhesion molecule A	receptor activity	1.96	7.11	Epi	1
47	LAD1	Ladinin-1	structural molecule activity	1.89	6.86	Epi	1
48	GPX2	Glutathione peroxidase 2	oxidoreductase activity;peroxidase activity	1.85	6.70	Epi	0
49	CTSH	Cathepsin H	cysteine-type peptidase activity	1.84	6.65	Epi	0
50	GPR56	G-protein coupled receptor 56	G-protein coupled receptor activity	1.83	6.62	Epi	1
51	FA2H	Fatty acid 2-hydroxylase	oxidoreductase activity	1.80	6.50	Epi	1
52	KLF5	Krueppel-like factor 5	transcription factor activity;transcription factor activity	1.79	6.48	Epi	0
53	AREG	Amphiregulin	growth factor activity	1.78	6.42	Epi	0
54	KRT18	Keratin, type I cytoskeletal 18	structural constituent of cytoskeleton	1.78	6.42	Epi	1
55	SCEL	Sciellin	---	1.77	6.40	Epi	0
56	CDH3	Cadherin-15	G-protein coupled receptor activity;calcium ion binding	1.76	6.34	Epi	0
57	UGT1A1	UDP-glucuronosyltransferase 1-1	transferase activity, transferring glycosyl groups	1.75	6.33	Epi	0
58	MPZL2	Myelin protein zero-like protein 2	voltage-gated sodium channel activity;voltage-gated sodium channel activity;cation channel activity	1.71	6.15	Epi	1
59	AIM1	Absent in melanoma 1 protein	structural molecule activity	1.71	6.13	Epi	0
60	OVOL2	Transcription factor Ovo-like 2	transcription factor activity;transcription factor activity	1.69	6.07	Epi	1
61	LLGL2	Lethal(2) giant larvae protein homolog 2	---	1.68	6.05	Epi	1
62	ESRP2	Epithelial splicing regulatory protein 2	structural constituent of ribosome;nucleic acid binding	1.68	6.02	Epi	1

63	MYO5C	Unconventional myosin-Vc	motor activity;structural constituent of cytoskeleton;protein binding;small GTPase regulator activity	1.67	6.01	Epi	1
64	DDR1	Epithelial discoidin domain-containing receptor 1	non-membrane spanning protein tyrosine kinase activity;transmembrane receptor protein kinase activity;transmembrane receptor protein tyrosine kinase activity;transmembrane receptor protein kinase activity;growth factor activity	1.67	5.98	Epi	1
65	VGLL1	Transcription cofactor vestigial-like protein 1	transcription factor activity;transcription factor activity	1.65	5.91	Epi	0
66	IRF6	Interferon regulatory factor 6	transcription factor activity;transcription factor activity	1.64	5.89	Epi	1
67	SFN	Oligoribonuclease, mitochondrial	exoribonuclease activity;nucleic acid binding	1.64	5.88	Epi	0
68	TSPAN13	Tetraspanin-13	receptor activity;receptor binding	1.63	5.85	Epi	0
69	KCNK1	Potassium channel subfamily K member 1	voltage-gated potassium channel activity;voltage-gated potassium channel activity;cation channel activity	1.62	5.81	Epi	0
70	MYO1D	Unconventional myosin-Id	motor activity;structural constituent of cytoskeleton;protein binding;enzyme regulator activity	1.60	5.72	Epi	0
71	PKP3	Plakophilin-3	structural constituent of cytoskeleton;intermediate filament binding	1.59	5.70	Epi	1
72	ITGB6	Integrin beta-6	receptor activity	1.59	5.69	Epi	0
73	LY75	Lymphocyte antigen 75	receptor activity	1.58	5.65	Epi	0
74	MAPK13	Mitogen-activated protein kinase 13	protein kinase activity	1.57	5.59	Epi	1
75	TTC39A	Tetratricopeptide repeat protein 39A	---	1.56	5.57	Epi	1
76	ELMO3	engulfment and cell motility 3	binding	1.54	5.49	Epi	1
77	CEACAM1	Carcinoembryonic antigen-related cell adhesion molecule 1	---	1.51	5.37	Epi	1
78	DTX4	Protein deltex-4	transcription factor activity;transcription factor activity;transcription cofactor activity	1.51	5.36	Epi	0
79	ERBB2	Receptor tyrosine-protein kinase erbB-2	non-membrane spanning protein tyrosine kinase activity;transmembrane receptor protein kinase activity;transmembrane receptor protein tyrosine kinase activity;transmembrane receptor protein kinase activity;growth factor activity	1.50	5.32	Epi	1
80	RAB11FIP1	Rab11 family-interacting protein 1	---	1.50	5.32	Epi	1
81	ATP2C2	Calcium-transporting ATPase type 2C member 2	hydrolase activity;cation transmembrane transporter activity;ion channel activity	1.48	5.28	Epi	1
82	MST1R	Macrophage-stimulating protein receptor	non-membrane spanning protein tyrosine kinase activity;transmembrane receptor protein kinase activity;transmembrane receptor protein tyrosine kinase activity;transmembrane receptor protein kinase activity;growth factor activity	1.48	5.28	Epi	1
83	AP1M2	AP-1 complex subunit mu-2	---	1.48	5.27	Epi	1
84	TGFA	Protransforming growth factor alpha	cytokine activity;growth factor activity	1.47	5.21	Epi	0

85	MYO6	Unconventional myosin-VI	motor activity;structural constituent of cytoskeleton;protein binding;small GTPase regulator activity	1.45	5.14	Epi	1
86	PTK6	Protein-tyrosine kinase 6	non-membrane spanning protein tyrosine kinase activity;transmembrane receptor protein kinase activity;transmembrane receptor protein tyrosine kinase activity;transmembrane receptor protein kinase activity;growth factor activity	1.44	5.09	Epi	1
87	OAS1	2'-5'-oligoadenylate synthase 1	nucleotidyltransferase activity;nucleic acid binding	1.43	5.08	Epi	0
88	FBP1	fructose-1,6-bisphosphatase 1	protein binding	1.41	5.00	Epi	0
89	AQP3	Aquaporin-3	transmembrane transporter activity	1.40	4.97	Epi	0
90	CBLC	Signal transduction protein CBL-C	ligase activity	1.40	4.94	Epi	1
91	EPHA1	Ephrin type-A receptor 1	non-membrane spanning protein tyrosine kinase activity;transmembrane receptor protein kinase activity;transmembrane receptor protein tyrosine kinase activity;transmembrane receptor protein kinase activity;growth factor activity	1.39	4.93	Epi	0
92	BSPRY	B box and SPRY domain-containing protein	ubiquitin-protein ligase activity;transcription factor activity;RNA binding;transcription factor activity;transcription cofactor activity	1.39	4.92	Epi	1
93	SH2D3A	SH2 domain-containing protein 3A	---	1.39	4.90	Epi	0
94	EPS8L1	Epidermal growth factor receptor kinase substrate 8-like protein 1	---	1.39	4.90	Epi	1
95	GRB7	Growth factor receptor-bound protein 7	---	1.37	4.83	Epi	0
96	C4orf19	Uncharacterized protein C4orf19	---	1.36	4.81	Epi	1
97	KLK6	Kallikrein-6	serine-type peptidase activity	1.35	4.75	Epi	0
98	TJP2	Tight junction protein ZO-2	---	1.35	4.75	Epi	1
99	PLS1	Phospholipid scramblase 1	---	1.35	4.75	Epi	1
100	DENND2D	DENN domain-containing protein 2D	---	1.34	4.73	Epi	0
101	EPS8L2	Epidermal growth factor receptor kinase substrate 8-like protein 2	---	1.31	4.59	Epi	1
102	IL20RA	Interleukin-20 receptor subunit alpha	interferon receptor activity	1.31	4.59	Epi	1
103	HES1	Transcription factor HES-1	transcription factor activity;transcription factor activity	1.30	4.57	Epi	0
104	IL1RN	Interleukin-1 receptor antagonist protein	hematopoietin/interferon-class (D200-domain) cytokine receptor binding	1.29	4.54	Epi	0
105	EXPH5	Exophilin-5	---	1.28	4.50	Epi	1
106	ARHGDIB	Rho GDP-dissociation inhibitor 2	receptor binding;small GTPase regulator activity	1.28	4.49	Epi	0
107	C19orf21	Uncharacterized protein C19orf21	---	1.27	4.47	Epi	1
108	CAMK2N1	Calcium/calmodulin-dependent protein kinase II inhibitor 1	---	1.27	4.46	Epi	0
109	HPGD	15-hydroxyprostaglandin dehydrogenase [NAD(+)]	oxidoreductase activity	1.27	4.44	Epi	0
110	SYNGR2	Synaptogyrin-2	---	1.27	4.44	Epi	1
111	C10orf116	chromosome 10 open reading frame 116	molecular_function	1.26	4.43	Epi	0
112	PERP	p53 apoptosis effector related to PMP-22	---	1.26	4.42	Epi	1
113	MANSC1	MANSC domain-containing protein 1	---	1.25	4.37	Epi	1

114	DSC2	Desmocollin-2	calcium ion binding	1.24	4.33	Epi	0
115	POF1B	Protein POF1B	---	1.23	4.30	Epi	1
116	SERINC5	Serine incorporator 5	---	1.22	4.27	Epi	0
117	BIK	Bcl-2-interacting killer	---	1.22	4.25	Epi	1
118	ANXA9	Annexin A9	calcium ion binding;calcium-dependent phospholipid binding	1.22	4.25	Epi	0
119	MALL	MAL-like protein	structural constituent of myelin sheath	1.21	4.24	Epi	0
120	EPN3	Epsin-3	---	1.21	4.23	Epi	1
121	STAP2	Signal-transducing adaptor protein 2	transmembrane receptor protein kinase activity;transmembrane receptor protein kinase activity	1.21	4.23	Epi	1
122	FOXA1	Hepatocyte nuclear factor 3-alpha	transcription factor activity;transcription factor activity	1.21	4.20	Epi	0
123	PYCARD	Apoptosis-associated speck-like protein containing a CARD	cysteine-type peptidase activity;protein binding;peptidase inhibitor activity	1.21	4.20	Epi	0
124	ZNF165	Zinc finger protein 165	transcription factor activity;transcription factor activity	1.20	4.17	Epi	0
125	SLC37A1	Glycerol-3-phosphate transporter	cation transmembrane transporter activity	1.19	4.14	Epi	0
126	ANK3	Ankyrin-3	structural constituent of cytoskeleton	1.16	4.02	Epi	0
127	TSPAN15	Tetraspanin-15	receptor activity;receptor binding	1.16	4.01	Epi	0
128	HNMT	Histamine N-methyltransferase	---	1.16	4.01	Epi	0
129	ABCC3	ATP-binding cassette, sub-family C (CFTR/MRP), member	nucleotide binding	1.16	4.01	Epi	1
130	SDC1	Syndecan-1	structural constituent of cytoskeleton;receptor binding	1.15	4.00	Epi	0
131	CKMT1A	creatine kinase, mitochondrial 1A	nucleotide binding	1.15	3.99	Epi	1
132	TOB1	Protein Tob1	---	1.15	3.98	Epi	1
133	B3GNT3	UDP-GlcNAc:betaGal beta-1,3-N-acetylglucosaminyltransferase 3	transferase activity, transferring glycosyl groups	1.14	3.96	Epi	0
134	TMC6	Transmembrane channel-like protein 6	ion channel activity	1.14	3.93	Epi	0
135	CD9	CD9 antigen	receptor activity;receptor binding	1.12	3.89	Epi	0
136	ADAP1	Arf-GAP with dual PH domain-containing protein 1	nucleic acid binding;protein binding;small GTPase regulator activity	1.12	3.88	Epi	0
137	ATP1B1	Sodium/potassium-transporting ATPase subunit beta-1	cation transmembrane transporter activity;hydrogen ion transmembrane transporter activity	1.12	3.86	Epi	0
138	SHANK2	SH3 and multiple ankyrin repeat domains protein 2	---	1.10	3.81	Epi	0
139	CYB561	Cytochrome b561	oxidoreductase activity	1.10	3.81	Epi	1
140	ERMP1	Endoplasmic reticulum metallopeptidase 1	metallopeptidase activity	1.10	3.78	Epi	0
141	RAB20	Ras-related protein Rab-20	---	1.09	3.75	Epi	0
142	MYH14	myosin, heavy chain 14, non-muscle	microfilament motor activity	1.08	3.73	Epi	0
143	CAPN1	Calpain-1 catalytic subunit	cysteine-type peptidase activity;calcium ion binding;calmodulin binding;calcium-dependent phospholipid binding	1.07	3.69	Epi	0
144	ALDH3B2	Aldehyde dehydrogenase family 3 member B2	oxidoreductase activity	1.06	3.65	Epi	0
145	TRIM31	E3 ubiquitin-protein ligase TRIM31	ubiquitin-protein ligase activity;structural constituent of cytoskeleton;transcription factor activity;RNA binding;cytoskeletal protein binding;transcription factor activity;transcription cofactor activity	1.06	3.65	Epi	0

146	ARAP2	Arf-GAP with Rho-GAP domain, ANK repeat and PH domain-containing protein 2	nucleic acid binding;protein binding;small GTPase regulator activity	1.06	3.64	Epi	0
147	SSH3	Protein phosphatase Slingshot homolog 3	phosphoprotein phosphatase activity;phosphoprotein phosphatase activity;protein binding;kinase inhibitor activity;kinase regulator activity	1.05	3.61	Epi	0
148	ICA1	Islet cell autoantigen 1	---	1.04	3.55	Epi	0
149	ARHGEF5	Rho guanine nucleotide exchange factor 5	---	1.03	3.54	Epi	0
150	ALOX5	Arachidonate 5-lipoxygenase	oxidoreductase activity	1.03	3.52	Epi	0
151	RHOD	Rho-related GTP-binding protein RhoD	GTPase activity;protein binding	1.01	3.44	Epi	0
152	TMPRSS2	Transmembrane protease serine 2	serine-type peptidase activity;receptor activity;calcium ion binding;hormone activity;calmodulin binding;calcium-dependent phospholipid binding;peptidase inhibitor activity	1.01	3.43	Epi	1
153	MTUS1	Microtubule-associated Tumour suppressor 1	---	1.00	3.41	Epi	0
154	CYP4F3	Leukotriene-B(4) omega-hydroxylase 2	oxidoreductase activity	0.99	3.37	Epi	0
155	PPFIBP2	Liprin-beta-2	---	0.99	3.36	Epi	0
156	RABGAP1L	Rab GTPase-activating protein 1-like	hydrolase activity;protein binding;small GTPase regulator activity	0.99	3.36	Epi	0
157	PLXNB2	Plexin-B2	transmembrane receptor protein kinase activity;transmembrane receptor protein tyrosine kinase activity;transmembrane receptor protein kinase activity;receptor binding	0.98	3.33	Epi	0
158	MGST2	Microsomal glutathione S-transferase 2	transferase activity	0.97	3.31	Epi	0
159	OR7E14P	olfactory receptor, family 7, subfamily E, member 14 pseudogene	---	0.97	3.30	Epi	1
160	EVPL	Envoplakin	structural constituent of cytoskeleton;intermediate filament binding	0.97	3.30	Epi	0
161	CD46	Membrane cofactor protein	serine-type peptidase activity;metallopeptidase activity;receptor activity;lipid transporter activity	0.97	3.28	Epi	0
162	KRT15	Keratin, type I cytoskeletal 15	structural constituent of cytoskeleton	0.95	3.22	Epi	0
163	CNKSR1	Connector enhancer of kinase suppressor of ras 1	protein binding;kinase regulator activity	0.95	3.20	Epi	0
164	BLNK	B-cell linker protein	receptor binding	0.94	3.17	Epi	0
165	COMT	Catechol O-methyltransferase	methyltransferase activity	0.93	3.16	Epi	0
166	ANXA4	annexin A4	phospholipase inhibitor activity	0.93	3.15	Epi	0
167	TNFSF13	Tumour necrosis factor ligand superfamily member 13	---	0.93	3.15	Epi	0
168	OCLN	Occludin	transcription factor activity;transcription factor activity;transcription cofactor activity	0.93	3.14	Epi	1
169	SLC9A3R1	Na(+)/H(+) exchange regulatory cofactor NHE-RF1	---	0.93	3.14	Epi	0
170	XBP1	X-box-binding protein 1	transcription factor activity;transcription factor activity	0.92	3.11	Epi	0
171	VIM	Vimentin	structural constituent of cytoskeleton	3.30	12.31	Mes	1
172	ZEB1	Zinc finger E-box-binding homeobox 1	---	3.07	11.40	Mes	1
173	EMP3	Epithelial membrane protein 3	structural constituent of cytoskeleton	2.78	10.27	Mes	1
174	SACS	Sacsin	---	2.07	7.56	Mes	1

175	AXL	Tyrosine-protein kinase receptor UFO	non-membrane spanning protein tyrosine kinase activity;transmembrane receptor protein kinase activity;transmembrane receptor protein tyrosine kinase activity;transmembrane receptor protein kinase activity;growth factor activity	2.00	7.29	Mes	1
176	LOXL2	Lysyl oxidase homolog 2	oxidoreductase activity;serine-type peptidase activity;receptor activity	1.82	6.57	Mes	0
177	SPARC	SPARC	growth factor activity	1.80	6.51	Mes	1
178	FHL1	Four and a half LIM domains protein 1	structural constituent of cytoskeleton;transcription factor activity;RNA binding;transcription factor activity	1.79	6.45	Mes	1
179	FERMT2	Fermitin family homolog 2	---	1.78	6.42	Mes	1
180	TUBA1A	Tubulin alpha-1A chain	structural constituent of cytoskeleton	1.75	6.32	Mes	1
181	TMEM158	transmembrane protein 158 (gene/pseudogene)	peptide binding	1.69	6.09	Mes	0
182	CALD1	Caldesmon	structural constituent of cytoskeleton;actin binding	1.65	5.94	Mes	1
183	LGALS1	Galectin-1	receptor binding	1.65	5.92	Mes	0
184	PMP22	Peripheral myelin protein 22	structural constituent of cytoskeleton	1.64	5.89	Mes	1
185	MSN	Moesin	structural constituent of cytoskeleton	1.54	5.51	Mes	0
186	GLYR1	Putative oxidoreductase GLYR1	oxidoreductase activity	1.51	5.38	Mes	1
187	MAP1B	MAP1 light chain LC1	structural constituent of cytoskeleton;microtubule binding	1.47	5.24	Mes	1
188	AP1S2	AP-1 complex subunit sigma-2	---	1.44	5.13	Mes	1
189	GJA1	Gap junction alpha-1 protein	gap junction channel activity	1.36	4.80	Mes	0
190	DENND5A	DENN domain-containing protein 5A	ion channel activity;receptor binding;small GTPase regulator activity	1.35	4.75	Mes	0
191	C12orf24	Protein FAM216A	---	1.33	4.70	Mes	0
192	TPM2	Tropomyosin beta chain	motor activity;structural constituent of cytoskeleton	1.32	4.65	Mes	1
193	TUBB6	Tubulin beta-6 chain	structural constituent of cytoskeleton	1.31	4.63	Mes	1
194	SRPX	Sushi repeat-containing protein SRPX	serine-type peptidase activity;metallopeptidase activity;receptor activity;lipid transporter activity	1.28	4.48	Mes	1
195	ANK2	Ankyrin-2	structural constituent of cytoskeleton	1.20	4.18	Mes	1
196	CHN1	N-chimaerin	protein binding;small GTPase regulator activity	1.17	4.06	Mes	1
197	SH2B3	SH2B adapter protein 3	---	1.17	4.06	Mes	0
198	LEPRE1	Prolyl 3-hydroxylase 1	---	1.13	3.90	Mes	0
199	ETV1	ETS translocation variant 1	transcription factor activity;receptor binding;transcription factor activity	1.11	3.84	Mes	0
200	SOBP	Sine oculis-binding protein homolog	---	1.10	3.81	Mes	1
201	AKAP12	A-kinase anchor protein 12	---	1.10	3.78	Mes	1
202	TGFB1I1	Transforming growth factor beta-1-induced transcript 1 protein	structural constituent of cytoskeleton	1.09	3.75	Mes	0
203	SERPINE1	Plasminogen activator inhibitor 1	protein binding;serine-type endopeptidase inhibitor activity	1.07	3.70	Mes	0
204	SOAT1	Sterol O-acyltransferase 1	acyltransferase activity	1.07	3.69	Mes	0
205	COL5A2	Collagen alpha-2(V) chain	receptor activity;extracellular matrix structural constituent;transmembrane transporter activity	1.05	3.61	Mes	1
206	LHFP	Lipoma HMGIC fusion partner	---	1.05	3.60	Mes	1

207	CEP170	Centrosomal protein of 170 kDa	---	1.04	3.57	Mes	1
208	POPDC3	Popeye domain-containing protein 3	---	1.03	3.54	Mes	0
209	TRPC1	Short transient receptor potential channel 1	ion channel activity	1.03	3.53	Mes	1
210	KDELC1	KDEL motif-containing protein 1	---	1.01	3.44	Mes	0
211	MYL9	Myosin regulatory light polypeptide 9	structural constituent of cytoskeleton;calcium ion binding;calmodulin binding	0.99	3.36	Mes	1
212	BAG2	BAG family molecular chaperone regulator 2	---	0.99	3.36	Mes	0
213	FSTL1	Follistatin-related protein 1	protein binding	0.98	3.34	Mes	1
214	MXRA7	Matrix-remodeling-associated protein 7	---	0.98	3.34	Mes	0
215	GFPT2	Glucosamine--fructose-6-phosphate aminotransferase [isomerizing] 2	transaminase activity	0.98	3.33	Mes	1
216	RECK	Reversion-inducing cysteine-rich protein with Kazal motifs	protein binding;serine-type endopeptidase inhibitor activity	0.97	3.29	Mes	1
217	TMEFF1	Tomoregulin-1	hydrolase activity;receptor activity;structural molecule activity;receptor binding	0.95	3.22	Mes	0
218	PTRF	Polymerase I and transcript release factor	transcription factor activity;transcription factor activity	0.93	3.14	Mes	1

Table S1C. Generic EMT signature compared against disease-specific EMT signature

**Source**	This Study	This Study	This Study	Akalay et al.	This Study	This Study	This Study	Miow et al.	Chung et al.	Joyce et al.	Loboda et al.	REACTOME	Alonso et al.	Gene Ontology GO:0001837	Gotzmann et al.	Jechlinger et al.	Sarrio et al.	Anastassiou et al.	Byers et al.	Becker-Santos et al.	Kalluri & Weinberg	Carretero et al.	Jordan et al.	Choi et al.
**Overlap% relative to Tumour generic EMT signature**	100	37.46	44.76	18.41	33.34	44.45	39.05	28.57	5.08	4.45	8.89	0.32	2.22	0	4.76	6.35	5.08	7.3	10.79	6.03	4.13	4.45	6.35	11.12
**Enrichment Score Corrleation (Spearman Correlation test on respective Tumour)**	+1.0	[+0.89,+0.97]	+0.96	+0.83	+0.91	+0.92	+0.91	+0.73	+0.44	+0.59	+0.80	[-0.32,+0.1]	+0.12	[+0.23,+0.56]	+0.37	+0.46	+0.05	+0.84	+0.82	+0.16	[+0.59,+0.77]	[+0.69,+0.86]	+0.80	+0.59
**Category\Cancer deriving EMT Signature**	**Tumour Generic**	**Cell-line Generic**	**Bladder Tumour**	**Breast Tumour**	**Colorectal Tumour**	**Gastric Tumour**	**Lung Tumour**	**Ovarian Tumour**	**40 Head & Neck Tumour**	**Colon cancer cell line (CACO-H & CACO-K)**	**92 Lung cancer cell lines - full list not available**	**Human Kidney HEK293T**	**34 Melanoma**	**Murine Endocardium**	**Hepatocytes cell line (MMH-RT)**	**Mammary Epithelium cell line (EpH4)**	**Breast cancer cell line (MCF10A)**	**Xenograft-neuroblastoma cell line (NGP)**	**54 NSCLC cell lines**	**Prostate cancer cell line (PrEC)**	**Curated**	**Curated fr Msigdb**	**Breast cancer cell lines (SUM149, SUM229)**	**15 Breast cancer cell lines**
Epi	KRT19	1	1	1	1	1	1	1	0	0	1	0	0	0	1	0	1	0	1	0	0	0	0	0
Epi	AGR2	1	1	1	0	1	0	0	0	0	0	0	0	0	0	0	0	0	0	0	0	0	0	1
Epi	RAB25	1	1	1	1	1	1	1	0	0	1	0	0	0	0	0	0	0	1	0	0	0	0	1
Epi	CDH1	1	1	1	1	1	1	1	1	0	1	0	0	0	1	1	1	0	1	1	0	0	0	1
Epi	ERBB3	1	1	1	0	1	1	1	0	0	1	0	0	0	0	0	0	0	0	0	0	0	0	1
Epi	FXYD3	1	1	1	1	1	0	1	0	0	1	0	0	0	0	0	1	0	1	0	0	0	0	1
Epi	SLC44A4	0	1	1	1	1	0	0	0	0	0	0	0	0	0	0	0	0	0	0	0	0	0	0
Epi	S100P	1	1	0	0	1	0	0	0	0	0	0	0	0	0	0	0	0	0	0	0	0	0	0
Epi	SCNN1A	1	0	1	0	1	0	0	0	0	0	0	0	0	0	0	0	0	1	0	0	0	0	0
Epi	GALNT3	1	0	0	1	1	1	1	0	0	1	0	0	0	0	0	0	0	1	0	0	0	0	1
Epi	PRSS8	1	0	1	1	1	0	1	0	0	1	0	0	0	0	0	0	0	1	0	0	0	0	0
Epi	ELF3	1	1	0	1	1	1	1	0	0	1	0	0	0	0	0	0	0	0	0	0	0	0	1
Epi	CEACAM6	1	0	0	1	1	0	0	0	1	1	0	0	0	0	0	0	0	0	0	0	0	0	0
Epi	TMPRSS4	1	0	0	1	1	0	1	0	0	1	0	0	0	0	0	0	0	0	0	0	0	0	0
Epi	CLDN7	1	0	1	1	1	1	1	0	0	1	0	0	0	0	0	0	0	1	0	0	0	0	1
Epi	TACSTD2	1	1	0	0	1	1	1	0	0	0	0	0	0	0	0	1	0	1	0	0	0	0	1
Epi	CLDN3	0	0	0	1	0	0	0	0	0	0	0	0	0	0	0	0	0	0	0	0	0	0	1
Epi	EPCAM	1	0	1	1	1	1	1	0	1	0	0	0	0	0	1	0	0	0	0	0	0	0	0
Epi	SPINT1	1	0	1	1	1	1	1	0	0	1	0	0	0	0	0	0	0	0	0	0	0	0	1
Epi	TSPAN1	1	0	1	1	1	0	0	0	0	0	0	0	0	0	0	0	0	0	0	0	0	0	1
Epi	PLS1	1	1	0	1	1	1	1	0	0	0	0	0	0	0	0	0	0	0	0	0	0	0	0
Epi	TMEM30B	1	1	0	1	1	1	1	0	0	0	0	0	0	0	0	0	0	1	0	0	0	0	0
Epi	PRR15L	0	0	1	1	1	0	1	0	0	0	0	0	0	0	0	0	0	0	0	0	0	0	0
Epi	KRT8	1	1	1	1	1	1	0	0	0	0	0	0	0	0	0	0	0	0	1	0	0	0	1
Epi	ST14	1	0	0	1	1	1	1	0	0	1	0	0	0	0	0	0	0	1	0	0	0	0	1
Epi	RBM47	1	1	1	1	1	0	1	0	0	0	0	0	0	0	0	0	0	0	0	0	0	0	1
Epi	S100A14	1	0	0	0	1	0	1	0	1	0	0	0	0	0	0	0	0	1	0	0	0	0	1
Epi	C1orf106	1	1	0	1	1	1	1	0	0	0	0	0	0	0	0	0	0	0	0	0	0	0	0
Epi	NQO1	0	1	0	0	1	0	1	0	0	0	0	0	0	0	0	0	0	0	0	0	0	0	0
Epi	TOX3	1	0	1	0	1	0	0	0	0	1	0	0	0	0	0	0	0	0	0	0	0	0	0
Epi	PTK6	1	1	1	1	1	0	1	0	0	0	0	0	0	0	0	0	0	0	0	0	0	0	0
Epi	TFF1	0	0	0	0	1	0	0	0	1	0	0	0	0	0	0	0	0	0	0	0	0	0	0
Epi	CLDN4	1	1	0	1	1	1	1	0	0	1	0	0	0	0	0	0	0	1	0	0	0	0	1
Epi	GPRC5A	0	0	0	1	1	0	0	0	0	0	0	0	0	0	0	0	0	0	0	0	0	0	0
Epi	TJP3	0	0	1	1	1	0	1	0	0	0	0	0	0	0	0	0	0	1	0	0	0	0	0
Epi	KRT18	1	0	1	0	1	1	0	0	0	0	0	0	0	0	0	0	0	0	1	0	0	0	0
Epi	MAP7	1	1	1	1	1	1	1	0	0	1	0	0	0	0	0	0	0	0	0	0	0	0	0
Epi	CKMT1A	1	0	0	1	1	0	1	0	0	0	0	0	0	0	0	0	0	0	0	0	0	0	1
Epi	ESRP1	1	0	0	1	1	1	1	0	0	0	0	0	0	0	0	0	0	0	0	0	0	0	0
Epi	MUC1	0	0	0	0	1	0	1	0	0	0	0	0	0	0	0	0	0	1	0	0	0	0	0
Epi	SPINT2	1	0	1	1	1	1	0	0	0	1	0	0	0	0	0	0	0	1	0	0	0	0	1
Epi	ESRP2	1	1	1	0	1	1	1	0	0	0	0	0	0	0	0	0	0	0	0	0	0	0	0
Epi	CDS1	1	0	1	1	1	1	0	0	0	0	0	0	0	0	0	0	0	1	0	0	0	0	0
Epi	PPAP2C	0	0	0	1	1	1	1	0	0	0	0	0	0	0	0	0	0	0	0	0	0	0	0
Epi	CEACAM7	0	0	0	1	0	0	0	0	0	0	0	0	0	0	0	0	0	0	0	0	0	0	0
Epi	TTC39A	1	0	0	1	1	0	0	0	0	0	0	0	0	0	0	0	0	0	0	0	0	0	0
Epi	OVOL2	1	0	0	1	1	1	1	0	0	0	0	0	0	0	0	0	0	0	0	0	0	0	0
Epi	EHF	1	0	0	0	0	0	1	0	0	0	0	0	0	0	0	0	0	0	0	0	0	0	0
Epi	AP1M2	1	1	1	1	1	1	1	0	0	1	0	0	0	0	0	0	0	1	0	0	0	0	0
Epi	CEACAM5	0	0	0	1	0	0	0	0	0	1	0	0	0	0	0	0	0	0	0	0	0	0	0
Epi	LAD1	1	1	0	1	1	1	1	0	0	0	0	0	0	0	0	0	0	0	0	0	0	0	1
Epi	ARHGAP8	1	0	1	0	0	1	1	0	0	1	0	0	0	0	0	0	0	0	0	0	0	0	0
Epi	TFF3	0	0	1	0	0	0	0	0	0	0	0	0	0	0	0	0	0	0	0	0	0	0	0
Epi	JUP	1	0	0	1	1	1	1	1	0	0	0	0	0	0	1	0	0	0	1	0	0	0	0
Epi	CD24	0	0	0	1	0	0	1	0	0	1	0	0	0	0	0	0	0	0	0	0	0	0	1
Epi	TMC5	0	0	0	1	1	0	1	0	0	0	0	0	0	0	0	0	0	0	0	0	0	0	0
Epi	MLPH	0	0	1	0	0	0	0	0	0	0	0	0	0	0	0	0	0	0	0	0	0	0	1
Epi	ELMO3	1	1	0	1	1	0	1	0	0	0	0	0	0	0	0	0	0	1	0	0	0	0	0
Epi	ERBB2	1	1	0	1	1	1	0	0	0	0	0	0	0	0	0	0	0	0	0	0	0	0	0

Epi	LLGL2	1	1	1	1	1	1	1	0	0	0	0	0	0	0	0	0	0	0	0	0	0	0	1
Epi	DDR1	1	1	1	1	1	1	0	1	0	0	0	0	0	0	0	0	0	0	0	0	0	0	0
Epi	FA2H	1	0	0	1	1	0	0	0	0	0	0	0	0	0	0	0	0	0	0	0	0	0	0
Epi	CBLC	1	0	0	1	1	1	1	0	0	0	0	0	0	0	0	0	0	0	0	0	0	0	0
Epi	TMPRSS2	1	0	0	1	1	0	0	0	0	0	0	0	0	0	0	0	0	0	0	0	0	0	0
Epi	LSR	1	0	0	1	1	1	1	0	0	0	0	0	0	0	0	0	0	0	0	0	0	0	0
Epi	PERP	1	0	0	0	1	0	1	0	0	0	0	0	0	0	0	0	0	0	0	0	0	0	0
Epi	POF1B	1	1	0	1	1	0	0	0	0	0	0	0	0	0	0	0	0	0	0	0	0	0	0
Epi	MYO5C	1	0	0	0	1	0	1	0	0	0	0	0	0	0	0	0	0	0	0	0	0	0	0
Epi	RAB11FIP1	1	1	0	0	1	0	0	0	0	0	0	0	0	0	0	0	0	0	0	0	0	0	0
Epi	MAPK13	1	1	0	0	1	1	1	0	0	1	0	0	0	0	0	0	0	1	0	0	0	0	0
Epi	KRT7	1	1	0	0	0	0	0	0	0	0	0	0	0	0	0	0	0	0	0	0	0	0	0
Epi	CEACAM1	1	0	0	1	0	0	0	1	0	0	0	0	0	0	0	0	0	0	0	0	0	0	0
Epi	CXADR	0	1	0	1	1	1	0	0	0	0	0	0	0	0	0	0	0	0	0	0	0	0	0
Epi	ATP2C2	1	1	1	1	0	0	0	0	0	1	0	0	0	0	0	0	0	0	0	0	0	0	0
Epi	RNF128	0	1	0	0	1	0	0	0	0	0	0	0	0	0	0	0	0	0	0	0	0	0	0
Epi	MPZL2	1	1	0	0	0	0	1	0	1	0	0	0	0	0	0	0	0	1	0	0	0	0	0
Epi	EPS8L1	1	0	1	1	1	0	1	0	0	0	0	0	0	0	0	0	0	0	0	0	0	0	0
Epi	GALNT7	0	0	1	0	1	0	0	0	0	0	0	0	0	0	0	0	0	0	0	0	0	0	0
Epi	CORO2A	0	0	0	1	1	0	0	0	0	0	0	0	0	0	0	0	0	0	0	0	0	0	0
Epi	BCAS1	0	1	0	0	1	0	0	0	0	0	0	0	0	0	0	0	0	0	0	0	0	0	0
Epi	TPD52	0	0	0	1	1	0	0	0	0	0	0	0	0	1	0	0	0	0	0	0	0	0	1
Epi	ARHGAP32	0	1	1	1	0	1	1	0	0	0	0	0	0	0	0	0	0	0	0	0	0	0	0
Epi	FUT2	0	0	0	1	1	0	1	0	0	0	0	0	0	0	0	0	0	0	0	0	0	0	0
Epi	OR7E14P	1	1	0	0	1	0	0	0	0	0	0	0	0	0	0	0	0	0	0	0	0	0	0
Epi	GALE	0	0	0	1	1	0	1	0	0	0	0	0	0	0	0	0	0	0	0	0	0	0	0
Epi	GRHL2	1	1	0	0	1	1	1	0	0	0	0	0	0	0	0	0	0	1	0	0	0	0	1
Epi	BIK	1	0	0	0	1	0	0	0	0	0	0	0	0	0	0	0	0	0	0	0	0	0	0
Epi	RAPGEFL1	0	1	0	1	1	0	0	0	0	0	0	0	0	0	0	0	0	0	0	0	0	0	0
Epi	STYK1	0	0	0	1	1	0	0	0	0	0	0	0	0	0	0	0	0	0	0	0	0	0	0
Epi	F11R	1	0	0	1	1	1	1	0	0	0	1	0	0	0	0	1	0	1	0	0	0	0	0
Epi	PKP3	1	0	0	1	1	1	1	0	0	1	0	0	0	0	0	0	0	0	0	0	0	0	1
Epi	CYB561	1	0	1	0	1	0	1	0	0	0	0	0	0	0	0	0	0	0	0	0	0	0	0
Epi	SH3YL1	1	1	0	0	1	1	0	0	0	0	0	0	0	0	0	0	0	1	0	0	0	0	1
Epi	GDF15	0	0	0	0	0	0	0	0	0	0	0	0	0	0	0	0	0	0	0	0	0	0	0
Epi	PSCA	0	1	0	0	0	0	0	0	0	0	0	0	0	0	0	0	0	0	0	0	0	0	0
Epi	EZR	0	0	0	1	1	0	0	0	0	0	0	0	0	0	0	0	0	0	0	0	0	0	0
Epi	TJP2	1	1	0	0	0	0	1	0	0	0	0	0	0	0	0	0	0	0	0	0	0	0	0
Epi	FGFR3	0	1	0	0	0	0	0	0	0	0	0	0	0	0	0	0	0	0	0	0	0	0	0
Epi	FUT3	0	0	0	1	1	0	0	0	0	0	0	0	0	0	0	0	0	0	0	0	0	0	0
Epi	BSPRY	1	0	0	0	1	0	1	0	0	0	0	0	0	0	0	0	0	1	0	0	0	0	0
Epi	TOM1L1	0	0	1	0	1	1	0	0	0	0	0	0	0	0	0	0	0	0	0	0	0	0	1
Epi	IRF6	1	0	0	1	1	0	1	0	0	0	0	0	0	0	1	0	0	0	1	0	0	0	0
Epi	EPB41L4B	0	0	0	0	1	1	0	0	0	0	0	0	0	0	0	0	0	0	0	0	0	0	0
Epi	SPDEF	0	0	1	0	0	0	1	0	0	0	0	0	0	0	0	0	0	0	0	0	0	0	1
Epi	OCLN	1	0	0	0	1	0	0	0	0	0	0	0	0	0	0	0	0	0	0	0	0	0	1
Epi	LRRC1	0	1	1	1	1	0	0	0	0	0	0	0	0	0	0	0	0	0	0	0	0	0	1
Epi	C19orf21	1	0	0	1	1	0	1	0	0	0	0	0	0	0	0	0	0	0	0	0	0	0	0
Epi	ABHD11	0	0	1	0	1	0	1	0	0	0	0	0	0	0	0	0	0	0	0	0	0	0	0
Epi	EPS8L2	1	0	0	1	1	0	1	0	0	0	0	0	0	0	0	0	0	0	0	0	0	0	0
Epi	MYO6	1	0	0	1	1	0	1	0	0	0	0	0	0	0	0	0	0	0	0	0	0	0	0
Epi	TSPAN8	0	0	0	1	1	0	0	0	1	0	0	0	0	0	0	0	0	0	0	0	0	0	0
Epi	MST1R	1	0	0	1	1	0	1	0	0	0	0	0	0	0	0	0	0	0	0	0	0	0	0
Epi	SLC16A5	0	0	0	1	1	0	0	0	0	0	0	0	0	0	0	0	0	0	0	0	0	0	0
Epi	GPR56	1	1	0	0	1	0	0	1	0	0	0	0	0	0	0	0	0	1	0	0	0	0	0
Epi	AZGP1	0	0	0	0	0	0	0	0	0	0	0	0	0	0	0	0	0	0	0	0	0	0	0
Epi	TOB1	1	0	1	0	0	0	0	0	0	0	0	0	0	1	0	0	0	0	0	0	0	0	0
Epi	SLC35A3	0	0	0	1	1	0	0	0	0	0	0	0	0	0	0	0	0	0	0	0	0	0	0
Epi	TRPM4	0	0	0	0	1	0	1	0	0	0	0	0	0	0	0	0	0	0	0	0	0	0	0
Epi	PHLDA2	0	0	0	0	1	0	0	0	0	0	0	0	0	0	0	0	0	0	0	0	0	0	0
Epi	VAMP8	1	0	0	0	1	0	1	0	0	0	0	0	0	0	1	0	0	0	0	0	0	0	1
Epi	SLC22A18	0	0	0	0	1	0	0	0	0	0	0	0	0	1	0	0	0	0	0	0	0	0	0
Epi	AKR1B10	0	0	0	0	1	0	0	0	0	0	0	0	0	0	0	0	0	0	0	0	0	0	0
Epi	VAV3	0	0	0	0	0	0	0	0	0	0	0	0	0	0	0	1	0	0	0	0	0	0	0
Epi	SPAG1	0	0	0	0	1	0	0	0	0	0	0	0	0	0	0	0	0	0	0	0	0	0	0
Epi	ABCC3	1	0	0	1	1	0	0	0	0	0	0	0	0	0	0	0	0	0	0	0	0	0	0
Epi	SYNGR2	1	0	0	1	1	0	1	0	0	0	0	0	0	0	0	0	0	0	0	0	0	0	0
Epi	STAP2	1	1	0	0	1	0	0	0	0	0	0	0	0	0	0	0	0	1	0	0	0	0	0
Epi	C4orf19	1	0	0	0	1	0	0	0	0	0	0	0	0	0	0	0	0	0	0	0	0	0	0
Epi	PPL	1	0	0	0	0	1	0	0	0	0	0	0	0	0	0	0	0	0	0	0	0	0	0
Epi	PLLP	0	0	0	0	1	0	0	0	0	0	0	0	0	0	0	0	0	0	0	0	0	0	0
Epi	DSG2	0	0	0	1	1	0	0	0	0	0	0	1	0	0	0	0	0	0	0	0	0	0	0
Epi	HDHD3	0	0	0	0	1	1	1	0	0	0	0	0	0	0	0	0	0	0	0	0	0	0	0
Epi	CD2AP	0	0	0	1	1	0	1	0	0	0	0	0	0	0	0	0	0	0	0	0	0	0	0
Epi	MANSC1	1	1	0	1	0	0	0	0	0	0	0	0	0	0	0	0	0	0	0	0	0	0	0

Epi	DHCR24	0	0	1	0	1	0	0	0	0	0	0	0	0	0	0	0	0	0	0	0	0	0	0
Epi	EPN3	1	1	1	0	1	0	0	0	0	0	0	0	0	0	0	0	0	1	0	0	0	0	0
Epi	TUFT1	0	0	0	0	1	0	0	0	0	0	0	0	0	0	0	0	0	0	0	0	0	0	0
Epi	GMDS	0	0	0	1	1	0	0	0	0	0	0	0	0	0	0	0	0	0	0	0	0	0	0
Epi	EXPH5	1	0	0	0	1	0	0	0	0	0	0	0	0	0	0	0	0	0	0	0	0	0	0
Epi	DSP	1	0	0	0	1	0	0	0	0	1	0	0	0	0	0	1	0	1	0	0	0	0	0
Epi	SDC4	0	0	0	0	1	0	0	0	0	0	0	0	0	1	0	0	0	0	0	0	0	0	0
Epi	IL20RA	1	1	0	0	0	0	0	0	0	0	0	0	0	0	0	0	0	0	0	0	0	0	0
Epi	FAM174B	0	1	1	0	0	0	0	0	0	0	0	0	0	0	0	0	0	0	0	0	0	0	0
Epi	PTPRF	0	1	0	0	1	1	0	1	0	0	0	0	0	0	0	0	0	0	0	0	0	0	0
Epi	SORD	0	0	0	0	0	0	0	0	0	0	0	0	0	0	0	0	0	0	0	0	0	0	0
Mes	GAS1	0	1	0	0	0	1	0	0	0	0	0	0	0	0	1	1	0	0	1	0	1	1	0
Mes	CXCL12	0	1	0	1	0	1	0	0	0	0	0	0	0	0	0	0	0	0	0	1	0	1	0
Mes	ZEB1	1	1	0	0	1	1	1	0	0	0	0	0	0	0	0	0	0	1	0	0	0	0	0
Mes	GLYR1	1	1	0	1	1	1	1	0	0	0	0	0	0	0	0	0	0	0	0	0	0	0	0
Mes	FHL1	1	0	0	0	0	0	1	0	0	0	0	0	0	1	0	0	0	0	0	0	0	1	0
Mes	FERMT2	1	1	0	0	0	1	1	0	0	0	0	0	0	0	0	0	0	0	0	0	0	0	0
Mes	C1S	0	1	1	1	0	1	0	0	0	0	0	0	0	0	0	1	0	0	0	0	0	1	0
Mes	FYN	0	1	1	0	1	1	1	0	1	0	0	0	0	0	0	0	0	0	0	0	0	0	0
Mes	WIPF1	0	1	1	0	0	1	0	0	0	0	0	0	0	0	0	0	0	0	0	0	0	0	0
Mes	CYP1B1	0	0	0	0	1	0	0	1	0	0	0	0	0	1	1	0	0	0	1	0	1	0	0
Mes	SERPING1	0	1	0	1	0	1	0	0	0	0	0	0	0	0	0	0	0	0	0	0	0	0	0
Mes	SERPINF1	0	1	0	0	0	1	0	0	0	0	0	0	0	0	0	0	1	0	0	0	0	0	0
Mes	VCAM1	0	1	0	0	0	1	0	1	0	0	0	0	0	0	0	0	0	0	0	0	0	0	0
Mes	MAP1B	1	1	0	0	1	0	1	0	0	0	0	0	0	0	0	0	0	0	0	0	0	0	0
Mes	TCF4	0	1	0	0	0	1	1	0	0	0	0	0	0	0	0	0	0	0	0	0	0	0	0
Mes	SRPX	1	1	0	0	0	0	0	0	0	0	0	0	0	0	0	0	0	0	0	0	0	0	0
Mes	EMP3	1	1	1	0	0	1	0	0	0	0	0	0	0	0	0	0	0	0	0	0	0	0	0
Mes	DPT	0	1	0	0	0	1	0	0	0	0	0	0	0	0	0	0	0	0	0	0	0	0	0
Mes	CALD1	1	1	0	0	0	1	1	0	0	0	0	0	0	0	0	1	0	0	0	0	0	0	0
Mes	PTGIS	0	0	0	0	0	1	0	0	0	0	0	0	0	0	0	0	0	0	0	0	0	0	0
Mes	VIM	1	1	1	0	0	1	0	0	0	0	0	0	0	0	1	0	0	1	1	1	1	1	0
Mes	CD163	0	1	0	1	0	0	0	0	0	0	0	0	0	0	0	0	0	0	0	0	0	0	0
Mes	C1R	0	1	1	0	0	1	0	0	0	0	0	0	0	0	0	0	0	0	0	0	0	0	0
Mes	FBN1	0	1	0	0	0	1	0	0	0	0	0	0	0	0	0	0	1	0	0	0	0	0	0
Mes	FN1	0	1	0	0	0	1	0	0	1	0	0	0	0	1	0	0	1	1	0	1	0	0	0
Mes	FXYD6	0	1	1	1	1	0	0	0	0	0	0	0	0	0	0	0	0	0	0	0	0	0	0
Mes	IGF1	0	0	0	0	0	0	0	0	0	0	0	0	0	0	0	0	0	0	0	0	0	0	0
Mes	NAP1L3	0	0	0	0	0	1	1	0	0	0	0	0	0	0	0	0	0	0	0	0	0	0	0
Mes	MRC1	0	1	0	0	0	0	0	0	0	0	0	0	0	1	0	0	0	0	0	0	0	0	0
Mes	QKI	0	1	0	1	1	0	0	0	0	0	0	0	0	0	0	0	0	0	0	0	0	0	0
Mes	MS4A4A	0	1	0	0	0	0	0	0	0	0	0	0	0	0	0	0	0	0	0	0	0	0	0
Mes	DCN	0	1	0	0	0	0	0	1	0	0	0	0	0	0	1	0	1	0	1	0	1	0	0
Mes	LOX	0	1	0	0	0	1	0	0	0	0	0	0	0	0	0	0	1	0	0	0	0	0	0
Mes	RECK	1	1	0	0	0	1	1	0	0	0	0	0	0	0	0	0	0	0	0	0	0	0	0
Mes	ANK2	1	1	0	0	1	1	0	0	0	0	0	0	0	0	0	0	0	0	0	0	0	0	0
Mes	LY96	0	1	0	0	0	0	0	0	0	0	0	0	0	0	0	0	0	0	0	0	0	1	0
Mes	ZFPM2	0	0	0	0	0	0	0	0	0	0	0	0	0	0	0	0	0	0	0	0	0	0	0
Mes	CSRP2	0	0	0	1	0	0	0	0	0	0	0	0	0	0	0	0	0	0	0	0	0	0	0
Mes	EFEMP1	0	1	0	0	0	0	0	0	0	0	0	0	0	0	0	0	0	0	0	0	0	0	0
Mes	RARRES2	0	0	0	0	0	1	0	0	0	0	0	0	0	0	0	0	0	0	0	0	0	0	0
Mes	PTPRC	0	1	0	0	0	1	0	0	0	0	0	0	0	0	0	0	0	0	0	0	0	0	0
Mes	PLEKHO1	0	1	1	0	1	1	0	0	0	0	0	0	0	0	0	0	0	0	0	0	0	0	0
Mes	RGS2	0	1	0	0	0	0	0	0	0	0	0	0	0	0	0	1	0	0	0	0	0	1	0
Mes	F13A1	0	1	0	0	0	1	0	0	0	0	0	0	0	0	0	0	0	0	0	0	0	0	0
Mes	JAM2	0	1	0	0	1	1	1	0	0	0	0	0	0	0	0	0	0	0	0	0	0	1	0
Mes	CHRDL1	0	0	0	0	0	0	0	0	0	0	0	0	0	0	0	0	0	0	0	0	0	0	0
Mes	TUBA1A	1	0	0	0	0	1	1	0	1	0	0	1	0	0	0	1	0	0	0	0	0	0	0
Mes	AP1S2	1	1	0	1	1	1	0	0	0	1	0	0	0	0	0	0	0	0	0	0	0	0	1
Mes	MYLK	0	0	0	0	0	1	0	0	0	0	0	0	0	0	0	0	0	0	0	0	0	0	0
Mes	DDR2	0	1	1	0	1	1	0	0	0	0	0	0	0	0	1	0	0	0	1	1	1	0	0
Mes	DSE	0	1	0	0	0	0	0	0	0	0	0	0	0	0	0	0	0	0	0	0	0	0	0
Mes	SACS	1	1	0	0	0	0	1	0	0	0	0	0	0	0	0	0	0	0	0	0	0	0	1
Mes	GLIPR1	0	1	1	0	0	1	0	0	0	0	0	0	0	0	0	0	0	0	0	0	0	0	0
Mes	CXCL13	0	0	0	0	0	0	0	0	0	0	0	0	0	0	0	0	0	0	0	0	0	0	0
Mes	FLRT2	0	0	0	0	1	0	0	0	0	0	0	0	0	0	0	0	0	0	0	0	0	0	0
Mes	PTX3	0	0	1	0	0	0	0	0	0	0	0	0	0	0	0	0	0	0	0	0	0	0	0
Mes	AKT3	0	1	0	0	0	0	0	0	0	0	0	0	0	0	0	0	0	0	0	1	0	0	0
Mes	COL6A2	0	1	0	0	0	1	0	1	0	0	0	0	0	0	1	0	1	0	1	0	1	0	0
Mes	DPYSL3	0	0	0	0	0	0	0	0	0	0	0	0	0	0	0	0	0	0	0	0	0	1	0
Mes	CDH11	0	0	0	0	0	1	0	0	0	0	0	0	0	0	0	0	1	0	0	1	0	0	0
Mes	PDZRN3	0	0	0	0	0	1	0	0	0	0	0	0	0	0	0	0	0	0	0	0	0	0	0
Mes	ZEB2	0	1	0	0	0	1	0	0	0	0	0	0	0	0	0	0	0	0	0	0	0	0	0
Mes	CCL2	0	0	0	0	0	1	0	0	0	0	0	0	0	1	1	0	0	0	0	1	0	1	0
Mes	MAFB	0	1	0	1	0	0	0	0	0	0	0	0	0	0	0	1	0	0	0	0	0	0	0

Mes	SFRP1	0	0	0	0	0	0	0	0	0	0	0	0	0	0	0	0	0	0	0	0	0	0	0
Mes	C14orf139	0	1	1	0	0	1	0	0	0	0	0	0	0	0	0	0	0	0	0	0	0	0	0
Mes	MFAP4	0	0	0	0	0	0	1	0	0	0	0	0	0	0	0	0	0	0	0	0	0	0	0
Mes	MAF	0	1	0	0	0	0	0	0	0	0	0	0	0	0	0	0	0	0	0	0	0	0	0
Mes	UCHL1	0	0	0	0	0	0	0	0	0	0	0	0	0	0	0	0	0	0	0	0	0	0	0
Mes	TUBB6	1	0	0	0	0	0	0	0	0	0	0	0	0	0	0	0	0	0	0	0	0	0	0
Mes	SRGN	0	1	0	0	0	0	0	0	0	0	0	0	0	0	0	0	0	0	0	0	0	0	0
Mes	HEG1	0	1	0	1	0	1	0	0	0	0	0	0	0	0	0	0	0	0	0	0	0	0	0
Mes	KCNJ8	0	1	0	1	1	0	1	0	0	0	0	0	0	0	0	0	0	0	0	0	0	0	0
Mes	AKAP12	1	0	0	0	0	1	0	0	1	1	0	0	0	0	0	0	0	0	0	0	0	1	0
Mes	EVI2A	0	1	0	0	0	1	0	0	0	0	0	0	0	0	0	0	0	0	0	0	0	0	0
Mes	COL14A1	0	0	0	0	0	0	0	0	0	0	0	0	0	0	0	0	0	0	0	0	0	0	0
Mes	AXL	1	1	0	0	1	0	0	0	0	0	0	0	0	0	0	0	0	1	0	0	0	1	0
Mes	ECM2	0	0	0	1	0	1	0	0	0	0	0	0	0	0	0	0	0	0	0	0	0	0	0
Mes	FSTL1	1	0	0	0	0	0	0	0	0	0	0	0	0	0	0	0	0	0	0	0	0	0	0
Mes	PLN	0	0	0	0	0	0	0	0	0	0	0	0	0	0	0	0	0	0	0	0	0	0	0
Mes	MYL9	1	0	0	0	0	1	0	0	0	0	0	0	0	0	0	0	0	0	0	0	0	0	0
Mes	OLFML3	0	0	0	0	0	0	0	0	0	0	0	0	0	0	0	0	0	0	0	0	0	0	0
Mes	STON1	0	0	0	0	0	1	0	0	0	0	0	0	0	0	0	0	0	0	0	0	0	0	0
Mes	SLIT2	0	1	0	0	1	1	0	0	0	0	0	0	0	0	0	0	0	0	0	0	0	1	0
Mes	BICC1	0	0	0	0	0	1	0	0	0	0	0	0	0	0	0	0	0	0	0	0	0	0	0
Mes	SOBP	1	0	0	0	0	0	0	0	0	0	0	0	0	0	0	0	0	0	0	0	0	0	0
Mes	CLIC4	0	1	0	0	0	1	0	0	0	0	0	1	0	0	0	0	0	0	0	0	0	0	0
Mes	ENPP2	0	0	0	0	0	0	0	0	0	0	0	0	0	0	0	0	0	0	0	0	0	0	0
Mes	SAMSN1	0	1	0	0	0	0	0	0	0	0	0	0	0	0	0	0	0	0	0	0	0	0	0
Mes	TPM2	1	1	0	0	0	1	0	0	1	0	0	0	0	0	0	0	0	0	0	0	0	0	0
Mes	ASPN	0	0	0	0	0	0	0	0	0	0	0	0	0	0	0	0	1	0	0	0	0	0	0
Mes	COL6A1	0	1	0	0	0	1	0	0	0	0	0	0	0	0	1	0	0	0	1	0	1	1	0
Mes	IGFBP5	0	0	0	0	0	0	1	0	0	0	0	0	0	0	0	0	0	0	0	0	0	0	0
Mes	MOXD1	0	0	1	0	0	0	0	0	0	0	0	0	0	0	0	0	0	0	0	0	0	1	0
Mes	AKAP2	0	1	0	0	0	1	0	0	0	0	0	0	0	0	0	1	0	0	0	0	0	0	0
Mes	SLC2A3	0	0	0	0	0	0	0	0	0	0	0	0	0	0	0	0	0	0	0	0	0	1	0
Mes	OLFML2B	0	1	0	0	0	1	0	0	0	0	0	0	0	0	0	0	1	0	0	0	0	0	0
Mes	ANGPTL2	0	1	0	0	0	1	0	0	0	1	0	0	0	0	0	0	0	0	0	0	0	0	0
Mes	PCOLCE	0	1	0	0	0	0	0	1	0	0	0	0	0	0	1	0	1	0	1	1	0	0	0
Mes	COLEC12	0	0	0	0	0	0	0	0	0	0	0	0	0	0	0	0	0	0	0	0	0	0	0
Mes	CTSK	0	1	0	0	0	1	0	0	0	0	0	0	0	0	0	0	1	0	0	0	0	0	0
Mes	TAGLN	0	0	0	0	0	1	0	0	0	0	0	0	0	0	0	0	0	0	0	0	0	0	0
Mes	CDH2	0	0	0	0	0	0	1	0	0	0	0	1	0	1	1	0	0	0	1	1	1	1	0
Mes	IL10RA	0	1	0	0	0	1	0	0	0	0	0	0	0	0	0	0	0	0	0	0	0	0	0
Mes	C1orf54	0	1	0	1	1	1	0	0	0	0	0	0	0	0	0	0	0	0	0	0	0	0	0
Mes	CEP170	1	1	0	1	0	0	1	0	0	0	0	0	0	0	0	0	0	0	0	0	0	0	0
Mes	TNS1	0	0	0	0	0	0	0	0	0	0	0	0	0	0	0	0	0	0	0	0	0	0	0
Mes	CLEC2B	0	0	0	1	0	0	0	0	0	0	0	0	0	0	0	0	0	0	0	0	0	0	0
Mes	JAM3	0	0	0	0	0	1	0	0	1	0	0	0	0	0	0	0	0	0	0	0	0	0	0
Mes	SEPT6	0	0	0	0	0	0	0	0	0	0	0	0	0	0	0	0	0	0	0	0	0	0	0
Mes	GREM1	0	0	0	0	0	0	0	0	0	0	0	0	0	0	0	0	1	0	0	0	0	0	0
Mes	VCAN	0	0	0	0	0	0	0	0	0	0	0	1	0	0	0	1	1	0	0	0	0	0	0
Mes	ZCCHC24	0	1	0	0	0	1	0	0	0	0	0	0	0	0	0	0	0	0	0	0	0	0	0
Mes	CRYAB	0	0	0	0	0	0	0	0	0	0	0	0	0	0	0	0	0	0	0	0	0	0	0
Mes	SFRP4	0	0	0	0	0	0	0	0	0	0	0	0	0	0	0	0	1	0	0	0	0	0	0
Mes	RUNX1T1	0	0	0	0	1	1	0	0	0	0	0	0	0	0	0	0	0	0	0	0	0	0	0
Mes	FGL2	0	0	0	0	0	0	0	0	0	0	0	0	0	0	0	0	0	0	0	0	0	0	0
Mes	MS4A6A	0	1	0	1	0	0	0	0	0	0	0	0	0	0	0	0	0	0	0	0	0	0	0
Mes	PTRF	1	1	0	0	0	1	0	0	0	0	0	0	0	0	0	0	0	0	0	0	0	0	0
Mes	GIMAP4	0	1	0	1	0	0	0	0	0	0	0	0	0	0	0	0	0	0	0	0	0	0	0
Mes	TWIST1	0	0	0	0	0	0	0	0	0	0	0	0	0	0	0	0	0	0	0	1	0	0	0
Mes	GFPT2	1	1	0	0	0	1	0	0	0	0	0	0	0	0	0	0	0	0	0	0	0	0	0
Mes	LHFP	1	0	0	0	0	0	0	0	1	0	0	0	0	0	0	0	0	0	0	0	0	0	0
Mes	CXCR4	0	0	0	0	0	1	0	0	0	0	0	0	0	0	0	0	0	0	0	1	0	0	0
Mes	SPOCK1	0	0	0	0	0	0	0	0	0	0	0	0	0	0	0	0	1	0	0	0	0	0	0
Mes	SPARC	1	1	0	0	0	1	0	1	1	0	0	1	0	0	1	0	1	0	1	0	1	0	0
Mes	VSIG4	0	1	0	1	0	0	0	0	0	0	0	0	0	0	0	0	0	0	0	0	0	0	0
Mes	GPM6B	0	0	0	0	0	0	0	0	0	0	0	0	0	0	0	0	0	0	0	0	0	0	0
Mes	TRPC1	1	0	0	0	0	0	1	0	0	0	0	0	0	0	0	0	0	0	0	0	0	0	0
Mes	SNAI2	0	0	0	0	0	0	0	0	0	0	0	0	0	0	0	0	1	0	0	1	0	0	0
Mes	GUCY1B3	0	1	0	1	0	0	0	0	0	0	0	0	0	0	0	0	0	0	0	0	0	0	0
Mes	PLXNC1	0	1	0	0	0	0	0	0	0	0	0	0	0	0	0	0	0	0	0	0	0	0	0
Mes	SYT11	0	1	1	0	1	1	1	0	0	0	0	0	0	0	0	0	0	0	0	0	0	0	0
Mes	FLI1	0	1	0	0	0	1	0	0	0	0	0	0	0	0	0	0	0	0	0	0	0	0	0
Mes	MYH10	0	0	0	1	0	0	0	0	0	0	0	0	0	0	0	0	0	0	0	0	0	0	0
Mes	CSF2RB	0	0	0	0	0	1	0	0	0	0	0	0	0	0	0	0	0	0	0	0	0	0	0
Mes	TNC	0	1	0	0	0	0	0	1	0	0	0	0	0	1	1	0	0	0	1	0	1	0	0
Mes	PMP22	1	1	0	0	0	1	0	1	0	0	0	0	0	0	1	0	0	0	1	0	1	1	0
Mes	COL5A2	1	1	0	0	0	1	0	0	0	0	0	0	0	1	0	0	1	0	0	0	0	0	0

Mes	MMP2	0	0	0	0	0	1	0	1	0	0	0	1	0	0	1	0	1	1	1	1	1	0	0
Mes	GNG11	0	0	0	0	0	0	0	0	0	0	0	0	0	0	0	0	0	0	0	0	0	1	0
Mes	CAV1	0	0	0	0	0	0	0	0	0	0	0	0	0	0	0	0	0	0	0	0	0	0	0
Mes	CDK14	0	1	0	1	0	0	0	0	0	0	0	0	0	0	0	0	0	0	0	0	0	0	0
Mes	SDC2	0	0	0	0	0	0	0	0	0	0	0	0	0	0	1	0	0	0	1	0	1	0	0
Mes	PTGDS	0	0	0	0	0	0	0	0	0	0	0	0	0	0	0	0	0	0	0	0	0	0	0
Mes	NR3C1	0	1	0	1	0	0	0	0	0	0	0	0	0	0	0	0	0	0	0	0	0	0	0
Mes	SYNM	0	0	0	0	0	0	0	0	0	0	0	0	0	0	0	0	0	0	0	0	0	0	0
Mes	FAP	0	1	0	0	0	0	0	0	0	0	0	0	0	0	0	0	1	0	0	0	0	0	0
Mes	NUAK1	0	0	0	0	0	0	0	0	0	0	0	0	0	0	0	0	1	0	0	0	0	0	0
Mes	WWTR1	0	0	0	0	0	0	0	0	0	0	0	0	0	0	0	0	0	0	0	0	0	0	0
Mes	FBLN1	0	0	0	0	0	1	0	0	0	0	0	0	0	0	0	0	0	0	0	0	0	0	0
Mes	MPDZ	0	0	0	1	0	0	1	0	0	0	0	0	0	1	0	0	0	0	0	0	0	0	0
Mes	SYNE1	0	0	0	0	1	0	0	0	0	0	0	0	0	0	0	0	0	0	0	0	0	0	0
Mes	EFEMP2	0	0	0	0	0	1	0	0	0	0	0	0	0	0	0	0	0	0	0	0	0	0	0
Mes	GIMAP6	0	0	0	0	0	0	0	0	0	0	0	0	0	0	0	0	0	0	0	0	0	0	0
Mes	KIAA1462	0	0	0	0	0	0	1	0	0	0	0	0	0	0	0	0	0	0	0	0	0	0	0
Mes	CCL8	0	1	0	0	0	0	0	0	0	0	0	0	0	0	0	0	0	0	0	0	0	0	0
Mes	COL15A1	0	0	0	0	0	0	0	0	0	0	0	0	0	0	0	0	0	0	0	0	0	0	0
Mes	CHN1	1	1	0	0	0	1	0	0	0	0	0	0	0	0	0	0	0	0	0	0	0	0	1
Mes	CRISPLD2	0	0	0	0	0	1	0	0	0	0	0	0	0	0	0	0	1	0	0	0	0	0	0
Mes	PDGFC	0	1	0	0	0	0	0	0	0	0	0	0	0	0	0	0	0	0	0	1	0	0	0
Mes	GEM	0	1	0	0	0	0	0	0	0	0	0	0	0	0	0	1	0	0	0	0	0	0	0
Mes	ISLR	0	1	0	0	0	0	0	1	0	0	0	0	0	0	0	0	0	0	0	0	0	0	0
Mes	GZMK	0	0	0	0	0	0	0	0	0	0	0	0	0	0	0	0	0	0	0	0	0	0	0
Mes	SPARCL1	0	0	0	0	0	0	0	0	0	0	0	0	0	0	0	0	0	0	0	0	0	0	0
Mes	BNC2	0	1	0	0	1	1	0	0	0	0	0	0	0	0	0	0	0	0	0	0	0	0	0
Mes	BGN	0	0	0	0	0	1	0	0	0	0	0	0	0	0	0	0	1	0	0	0	0	0	0
Mes	MEOX2	0	0	0	0	0	0	0	0	0	0	0	0	0	0	0	0	0	0	0	0	0	0	0
Mes	ITM2A	0	0	0	0	0	0	0	0	0	0	0	0	0	0	0	0	0	0	0	0	0	1	0
Mes	IFFO1	0	1	1	1	0	1	0	0	0	0	0	0	0	0	0	0	0	0	0	0	0	0	0

Source	References
Miow et al.	Miow QH, Tan TZ, Ye J, Lau JA, Yokomizo T, Thiery JP, Mori S. Epithelial-Mesenchymal Status Renders Differential Responses to Cisplatin in Ovarian Cancer, *Oncogene*, accepted for publication, 2014.
Akalay et al.	Akalay I, Janji B, Hasmim M, Noman MZ, André F, De Cremoux P, Bertheau P, Badoual C, Vielh P, Larsen AK, Sabbah M, Tan TZ, Keira JH, Hung NT, Thiery JP, Mami-Chouaib F, Chouaib S. Epithelial-to-mesenchymal transition and autophagy induction in breast carcinoma promote escape from T-cell-mediated lysis. *Cancer Res*. 2013 Apr 15;73(8):2418-27.
Chung et al.	Chung CH, Parker JS, Ely K, Carter J, Yi Y, Murphy BA, Ang KK, El-Naggar AK, Zanation AM, Cmelak AJ, Levy S, Slebos RJ, Yarbrough WG. Gene expression profiles identify epithelial-to-mesenchymal transition and activation of nuclear factor-kappaB signaling as characteristics of a high-risk head and neck squamous cell carcinoma. *Cancer Res*. 2006 Aug 15;66(16):8210-8.
Joyce et al.	Joyce T, Cantarella D, Isella C, Medico E, Pintzas A. A molecular signature for Epithelial to Mesenchymal transition in a human colon cancer cell system is revealed by large-scale microarray analysis. *Clin Exp Metastasis*.2009;26(6):569-87.
Loboda et al.	Loboda A, Nebozhyn MV, Watters JW, Buser CA, Shaw PM, Huang PS, Van't Veer L, Tollenaar RA, Jackson DB, Agrawal D, Dai H, Yeatman TJ. EMT is the dominantprogram in human colon cancer. *BMC Med Genomics*. 2011 Jan 20;4:9.
REACTOME	REACTOME
Alonso et al.	Alonso SR, Tracey L, Ortiz P, Pérez-Gómez B, Palacios J, Pollán M, Linares J, Serrano S, Sáez-Castillo AI, Sánchez L, Pajares R, Sánchez-Aguilera A, Artiga MJ, Piris MA, Rodríguez-Peralto JL. A high-throughput study in melanoma identifies epithelial-mesenchymal transition as a major determinant of metastasis. *Cancer Res*. 2007 Apr 1;67(7):3450-60.
Gene Ontology GO:0001837	Gene Ontology GO:0001837
Gotzmann et al.	Gotzmann J, Fischer AN, Zojer M, Mikula M, Proell V, Huber H, Jechlinger M, Waerner T, Weith A, Beug H, Mikulits W. A crucial function of PDGF in TGF-beta-mediated cancer progression of hepatocytes. *Oncogene*. 2006 May 25;25(22):3170-85.
Jechlinger et al.	Jechlinger M, Grunert S, Tamir IH, Janda E, Lüdemann S, Waerner T, Seither P, Weith A, Beug H, Kraut N. Expression profiling of epithelial plasticity in Tumour progression. Oncogene. 2003 Oct 16;22(46):7155-69.
Sarrio et al.	Sarrió D, Rodriguez-Pinilla SM, Hardisson D, Cano A, Moreno-Bueno G, Palacios J. Epithelial-mesenchymal transition in breast cancer relates to the basal-like phenotype. *Cancer Res*. 2008 Feb 15;68(4):989-97.
Anastassiou et al.	Anastassiou D, Rumjantseva V, Cheng W, Huang J, Canoll PD, Yamashiro DJ, Kandel JJ. Human cancer cells express Slug-based epithelial-mesenchymal transition gene expression signature obtained in vivo. *BMC Cancer*. 2011 Dec 30;11:529.
Byers et al.	Byers LA, Diao L, Wang J, Saintigny P, Girard L, Peyton M, Shen L, Fan Y, Giri U, Tumula PK, Nilsson MB, Gudikote J, Tran H, Cardnell RJ, Bearss DJ, Warner SL, Foulks JM, Kanner SB, Gandhi V, Krett N, Rosen ST, Kim ES, Herbst RS, Blumenschein GR, Lee JJ, Lippman SM, Ang KK, Mills GB, Hong WK, Weinstein JN, Wistuba II, Coombes KR, Minna JD, Heymach JV. An epithelial-mesenchymal transition gene signature predicts resistance to EGFR and PI3K inhibitors and identifies Axl as a therapeutic target for overcoming EGFR inhibitor resistance. *Clin Cancer Res*. 2013 Jan 1;19(1):279-90.
Becker-Santos et al.	Becker-Santos DD, Guo Y, Ghaffari M, Vickers ED, Lehman M, Altamirano-Dimas M, Oloumi A, Furukawa J, Sharma M, Wang Y, Dedhar S, Cox ME. Integrin-linked kinase as a target for ERG-mediated invasive properties in prostate cancer models. *Carcinogenesis*. 2012 Dec;33(12):2558-67.
Kalluri & Weinberg	Kalluri R, Weinberg RA. The basics of epithelial-mesenchymal transition. *J Clin Invest*. 2009 Jun;119(6):1420-8.
Carretero et al.	Carretero J, Shimamura T, Rikova K, Jackson AL, Wilkerson MD, Borgman CL, Buttarazzi MS, Sanofsky BA, McNamara KL, Brandstetter KA, Walton ZE, Gu TL, Silva JC, Crosby K, Shapiro GI, Maira SM, Ji H, Castrillon DH, Kim CF, García-Echeverría C, Bardeesy N, Sharpless NE, Hayes ND, Kim WY, Engelman JA, Wong KK. Integrative genomic and proteomic analyses identify targets for Lkb1-deficient metastatic lung Tumours. Cancer Cell. 2010 Jun 15;17(6):547-59.
Choi et al.	Choi YL, Bocanegra M, Kwon MJ, Shin YK, Nam SJ, Yang JH, Kao J, Godwin AK, Pollack JR. LYN is a mediator of epithelial-mesenchymal transition and a target of dasatinib in breast cancer. *Cancer Res*. 2010 Mar 15;70(6):2296-306.
Jordan et al.	Jordan NV, Prat A, Abell AN, Zawistowski JS, Sciaky N, Karginova OA, Zhou B, Golitz BT, Perou CM, Johnson GL. SWI/SNF chromatin-remodeling factor Smarcd3/Baf60c controls epithelial-mesenchymal transition by inducing Wnt5a signaling. Mol Cell Biol. 2013 Aug;33(15):3011-25.

Table S2. Gene Ontology & KEGG pathway analysis of generic tumour-specific EMT signature.

Category	Term	Count	%	P-Value	Genes	Fold Enrichment	False Discover Rate (corrected by Benjamin's method)
GOTERM_BP_FAT	GO:0007155~cell adhesion	44	1.32	7.21E-13	CLDN7, PLXNC1, CCL2, MPZL2, CLDN4, CLDN3, TNC, FERMT2, CDH1, SPOCK1, CDH2, CXADR, CD2AP, DDR2, CXCL12, ISLR, VCAM1, AZGP1, SRPX, EZR, COL6A2, COL6A1, CD24, CEACAM1, FN1, DPT, FLRT2, F11R, PTPRC, GMDS, PTPRF, COL15A1, ECM2, JUP, DDR1, COL14A1, DSG2, PKP3, GPR56, VCAN, JAM2, PERP, MFAP4, CDH11	3.51	1.24E-09
GOTERM_BP_FAT	GO:0022610~biological adhesion	44	1.32	7.47E-13	CLDN7, PLXNC1, CCL2, MPZL2, CLDN4, CLDN3, TNC, FERMT2, CDH1, SPOCK1, CDH2, CXADR, CD2AP, DDR2, CXCL12, ISLR, VCAM1, AZGP1, SRPX, EZR, COL6A2, COL6A1, CD24, CEACAM1, FN1, DPT, FLRT2, F11R, PTPRC, GMDS, PTPRF, COL15A1, ECM2, JUP, DDR1, COL14A1, DSG2, PKP3, GPR56, VCAN, JAM2, PERP, MFAP4, CDH11	3.51	6.41E-10
GOTERM_BP_FAT	GO:0006928~cell motion	29	0.87	2.64E-08	CCL2, SORD, ENPP2, CALD1, ERBB2, VIM, SPOCK1, ZEB2, CDH2, CXCL12, CD2AP, VCAM1, OVOL2, CXCR4, SPINT2, CD24, CEACAM1, TWIST1, FN1, VAV3, S100P, IGF1, GAS1, SLIT2, TNS1, FYN, VCAN, MST1R, MYH10	3.41	1.51E-05
GOTERM_BP_FAT	GO:0043062~extracellular structure organization	16	0.48	2.16E-07	RECK, MYO6, ELF3, ERBB2, TNC, MAP1B, CDH1, DCN, CDH2, ECM2, COL5A2, COL14A1, CRISPLD2, COL6A2, LOX, DPT	5.49	9.27E-05
GOTERM_BP_FAT	GO:0016337~cell-cell adhesion	20	0.60	5.12E-07	CLDN7, PTPRC, MPZL2, CLDN4, PTPRF, GMDS, CLDN3, CDH1, CDH2, CD2AP, VCAM1, JUP, EZR, COL14A1, DSG2, COL6A2, CD24, JAM2, CEACAM1, CDH11	4.05	1.76E-04
GOTERM_BP_FAT	GO:0016477~cell migration	19	0.57	2.24E-06	S100P, VAV3, CCL2, ZEB2, CDH2, CXCL12, CD2AP, SLIT2, VCAM1, TNS1, OVOL2, CXCR4, FYN, VCAN, CD24, CEACAM1, MYH10, TWIST1, FN1	3.85	6.41E-04
GOTERM_BP_FAT	GO:0051674~localization of cell	20	0.60	2.51E-06	S100P, VAV3, CCL2, SORD, ZEB2, CDH2, CXCL12, CD2AP, SLIT2, VCAM1, TNS1, OVOL2, CXCR4, FYN, VCAN, CD24, CEACAM1, MYH10, TWIST1, FN1	3.64	6.17E-04
GOTERM_BP_FAT	GO:0048870~cell motility	20	0.60	2.51E-06	S100P, VAV3, CCL2, SORD, ZEB2, CDH2, CXCL12, CD2AP, SLIT2, VCAM1, TNS1, OVOL2, CXCR4, FYN, VCAN, CD24, CEACAM1, MYH10, TWIST1, FN1	3.64	6.17E-04
GOTERM_BP_FAT	GO:0009611~response to wounding	27	0.81	2.83E-06	CCL2, ELF3, ERBB3, TNC, ERBB2, F13A1, CCL8, C1R, C1S, CXCR4, LOX, CD24, PTX3, FN1, F11R, LY96, EFEMP2, MAP1B, IGF1, SERPING1, CD163, CXCL13, DSP, PLLP, VCAN, VSIG4, MYH10	2.85	6.08E-04
GOTERM_BP_FAT	GO:0007517~muscle organ development	15	0.45	2.55E-05	CAV1, CRYAB, ERBB3, ERBB2, FHL1, TNC, IGF1, CXADR, CSRP2, MEOX2, TAGLN, PLN, ZFPM2, MYH10, IGFBP5	3.97	4.85E-03
GOTERM_BP_FAT	GO:0060056~mammary gland involution	4	0.12	5.44E-05	CAV1, ELF3, ERBB3, ERBB2	44.72	9.30E-03
GOTERM_BP_FAT	GO:0030198~extracellular matrix organization	10	0.30	9.83E-05	RECK, COL14A1, ELF3, CRISPLD2, COL6A2, DCN, LOX, ECM2, COL5A2, DPT	5.38	1.52E-02
GOTERM_BP_FAT	GO:0060443~mammary gland morphogenesis	4	0.12	1.07E-04	CAV1, ELF3, ERBB3, ERBB2	37.27	1.53E-02
GOTERM_BP_FAT	GO:0001503~ossification	10	0.30	2.12E-04	CTSK, CHRDL1, TUFT1, IGF1, SPARC, WWTR1, COL5A2, MMP2, CDH11, IGFBP5	4.86	2.77E-02
GOTERM_BP_FAT	GO:0008285~negative regulation of cell proliferation	18	0.54	2.59E-04	CAV1, PTPRF, ERBB2, GAS1, ZEB1, CXADR, DDR1, AZGP1, OVOL2, IRF6, CD24, PMP22, VSIG4, EMP3, IGFBP5, DHCR24, TOB1, DPT	2.79	3.13E-02
GOTERM_BP_FAT	GO:0014706~striated muscle tissue development	10	0.30	2.75E-04	CAV1, MEOX2, ERBB3, TNC, ERBB2, PLN, ZFPM2, CXADR, MYH10, IGFBP5	4.70	3.10E-02
GOTERM_BP_FAT	GO:0060348~bone development	10	0.30	3.51E-04	CTSK, CHRDL1, TUFT1, IGF1, SPARC, WWTR1, COL5A2, MMP2, CDH11, IGFBP5	4.54	3.70E-02
GOTERM_BP_FAT	GO:0060537~muscle tissue development	10	0.30	3.96E-04	CAV1, MEOX2, ERBB3, TNC, ERBB2, PLN, ZFPM2, CXADR, MYH10, IGFBP5	4.47	3.92E-02
GOTERM_BP_FAT	GO:0001568~blood vessel development	14	0.42	4.54E-04	RECK, CAV1, COL15A1, CDH2, MMP2, CXCL12, SLIT2, BGN, MEOX2, CXCR4, QKI, ZFPM2, LOX, CEACAM1	3.19	4.25E-02
GOTERM_BP_FAT	GO:0022604~regulation of cell morphogenesis	10	0.30	5.58E-04	EZR, PTPRF, FYN, MAP1B, FERMT2, CDH2, AKAP2, SLIT2, MYH10, FN1	4.27	4.93E-02
GOTERM_BP_FAT	GO:0001944~vasculature development	14	0.42	5.72E-04	RECK, CAV1, COL15A1, CDH2, MMP2, CXCL12, SLIT2, BGN, MEOX2, CXCR4, QKI, ZFPM2, LOX, CEACAM1	3.12	4.80E-02
KEGG_PATHWAY	hsa04670:Leukocyte transendothelial migration	15	0.45	7.33E-08	F11R, CLDN7, VAV3, OCLN, CLDN4, CLDN3, MMP2, CXCL12, MYL9, VCAM1, EZR, MAPK13, CXCR4, JAM2, JAM3	6.22	7.18E-06
KEGG_PATHWAY	hsa04514:Cell adhesion molecules (CAMs)	15	0.45	3.05E-07	F11R, CLDN7, PTPRC, OCLN, PTPRF, CLDN4, CLDN3, CDH1, CDH2, SDC4, SDC2, VCAM1, VCAN, JAM2, JAM3	5.56	1.49E-05
KEGG_PATHWAY	hsa04530:Tight junction	14	0.42	2.33E-06	F11R, CLDN7, OCLN, CLDN4, MPDZ, CLDN3, MYL9, LLGL2, TJP3, JAM2, JAM3, TJP2, AKT3, MYH10	5.11	7.62E-05
KEGG_PATHWAY	hsa05130:Pathogenic Escherichia coli infection	8	0.24	1.31E-04	EZR, KRT18, OCLN, LY96, FYN, TUBB6, CDH1, TUBA1A	6.86	3.21E-03
KEGG_PATHWAY	hsa04510:Focal adhesion	14	0.42	1.81E-04	CAV1, VAV3, ERBB2, TNC, IGF1, COL5A2, MYL9, FYN, COL6A2, COL6A1, PDGFC, AKT3, MYLK, FN1	3.41	3.53E-03

Table S3. EMT score (generic cell line EMT signature) of functional studies data.

Index	Sample	Source	Desc.	Label	Tissue	EMT score *p*-value	EMT Score	EMT score *p*-value from Reduced Set	EMT Score from Reduced Set	Reference
1	GSM245051	GSE9691	HMLE_shCntrl_rep1	Breast_HMLE_shCtrl	Breast	1.32E-01	1.60E-01	1.63E-01	2.32E-01	
2	GSM245052	GSE9691	HMLE_shCntrl_rep2	Breast_HMLE_shCtrl	Breast	1.38E-01	1.58E-01	1.32E-01	2.46E-01	
3	GSM245053	GSE9691	HMLE_shCntrl_rep3	Breast_HMLE_shCtrl	Breast	1.01E-01	1.70E-01	8.88E-02	2.69E-01	
4	GSM245054	GSE9691	HMLE_shEcad+shBcat_rep1	Breast_HMLE_shEcad+shBcat	Breast	1.06E-01	1.68E-01	8.13E-02	2.73E-01	
5	GSM245055	GSE9691	HMLE_shEcad+shBcat_rep2	Breast_HMLE_shEcad+shBcat	Breast	8.57E-02	1.76E-01	3.99E-02	3.10E-01	Onder TT, Gupta PB, Mani SA, et al. 2008 Cancer Res **68** 3645-54
6	GSM245056	GSE9691	HMLE_shEcad+shBcat_rep3	Breast_HMLE_shEcad+shBcat	Breast	1.06E-01	1.68E-01	3.13E-02	3.21E-01	
7	GSM245057	GSE9691	HMLE_shEcad_rep1	Breast_HMLE_shEcad	Breast	2.01E-13	6.07E-01	2.16E-10	8.14E-01	
8	GSM245058	GSE9691	HMLE_shEcad_rep2	Breast_HMLE_shEcad	Breast	3.52E-14	6.25E-01	4.03E-10	8.03E-01	
9	GSM245059	GSE9691	HMLE_shEcad_rep3	Breast_HMLE_shEcad	Breast	1.45E-13	6.11E-01	1.15E-10	8.26E-01	
10	GSM245060	GSE9691	HMLE_DNEcad_rep1	Breast_HMLE_DNEcad	Breast	4.91E-15	6.45E-01	4.14E-11	8.44E-01	
11	GSM245061	GSE9691	HMLE_DNEcad_rep2	Breast_HMLE_DNEcad	Breast	4.12E-15	6.46E-01	7.91E-11	8.32E-01	
12	GSM245062	GSE9691	HMLE_DNEcad_rep3	Breast_HMLE_DNEcad	Breast	3.78E-15	6.47E-01	1.47E-11	8.62E-01	
13	GSM595326	GSE24202	HMLE, pWZL, rep1	Breast_HMLE_EmptyVector	Breast	6.36E-03	2.53E-01	6.03E-03	3.90E-01	
14	GSM595327	GSE24202	HMLE, pWZL, rep2	Breast_HMLE_EmptyVector	Breast	4.82E-03	2.59E-01	8.11E-03	3.79E-01	
15	GSM595328	GSE24202	HMLE, pWZL, rep3	Breast_HMLE_EmptyVector	Breast	2.02E-03	2.80E-01	1.43E-02	3.56E-01	
16	GSM595329	GSE24202	HMLE, TGFb, rep1	Breast_HMLE_OverExpress_TGFβ	Breast	7.66E-10	5.14E-01	1.74E-06	6.29E-01	
17	GSM595330	GSE24202	HMLE, TGFb, rep2	Breast_HMLE_OverExpress_TGFβ	Breast	1.51E-10	5.34E-01	1.74E-06	6.29E-01	
18	GSM595331	GSE24202	HMLE, TGFb, rep3	Breast_HMLE_OverExpress_TGFβ	Breast	1.87E-10	5.31E-01	1.07E-06	6.40E-01	
19	GSM595332	GSE24202	HMLE, Twist, rep1	Breast_HMLE_OverExpress_TWIST	Breast	2.73E-14	6.28E-01	1.37E-09	7.80E-01	
20	GSM595333	GSE24202	HMLE, Twist, rep2	Breast_HMLE_OverExpress_TWIST	Breast	3.52E-14	6.25E-01	1.37E-09	7.80E-01	Taube JH, Herschkowitz JI, Komurov K, et al. 2010 Proc Natl Acad Sci USA 107 15449-54
21	GSM595334	GSE24202	HMLE, Twist, rep3	Breast_HMLE_OverExpress_TWIST	Breast	6.33E-14	6.19E-01	1.37E-09	7.80E-01	
22	GSM595335	GSE24202	HMLE, Gsc, rep1	Breast_HMLE_OverExpress_GSC	Breast	3.05E-12	5.78E-01	2.27E-08	7.24E-01	
23	GSM595336	GSE24202	HMLE, Gsc, rep2	Breast_HMLE_OverExpress_GSC	Breast	4.86E-12	5.73E-01	1.29E-08	7.36E-01	
24	GSM595337	GSE24202	HMLE, Gsc, rep3	Breast_HMLE_OverExpress_GSC	Breast	3.05E-12	5.78E-01	1.29E-08	7.36E-01	
25	GSM595338	GSE24202	HMLE, Snail, rep1	Breast_HMLE_OverExpress_SNAIL	Breast	5.35E-15	6.44E-01	4.03E-10	8.03E-01	
26	GSM595339	GSE24202	HMLE, Snail, rep2	Breast_HMLE_OverExpress_SNAIL	Breast	5.35E-15	6.44E-01	2.16E-10	8.14E-01	
27	GSM595340	GSE24202	HMLE, Snail, rep3	Breast_HMLE_OverExpress_SNAIL	Breast	1.57E-15	6.56E-01	1.50E-10	8.21E-01	
28	MDAcontrol1	E-MEXP-3547	MDA-MB-231_shMock(1)	Breast_MDA-MB-231_shMock	Breast	7.05E-08	4.55E-01	1.01E-05	5.85E-01	
29	MDAcontrol2	E-MEXP-3547	MDA-MB-231_shMock(2)	Breast_MDA-MB-231_shMock	Breast	2.99E-07	4.35E-01	1.01E-05	5.85E-01	
30	MDAhmga1	E-MEXP-3547	MDA-MB-231_shHMGA2(1)	Breast_MDA-MB-231_shHMGA2	Breast	7.99E-07	4.20E-01	2.42E-05	5.62E-01	---
31	MDAhmga2	E-MEXP-3547	MDA-MB-231_shHMGA2(2)	Breast_MDA-MB-231_shHMGA2	Breast	5.37E-07	4.26E-01	2.42E-05	5.62E-01	
32	GSM707310	GSE28569	MCF10A cells, control	Breast_MFC10A_Control	Breast	2.04E-01	1.43E-01	1.24E-01	2.55E-01	
33	GSM707311	GSE28569	MCF10A cells, control	Breast_MFC10A_Control	Breast	2.94E-01	-1.25E-01	1.11E-03	4.60E-01	
34	GSM707312	GSE28569	MCF10A cells, TGFbeta1	Breast_MFC10A_TGFβ	Breast	5.46E-03	2.58E-01	1.61E-04	5.21E-01	
35	GSM707313	GSE28569	MCF10A cells, TGFbeta1	Breast_MFC10A_TGFβ	Breast	1.13E-03	2.95E-01	8.65E-02	2.76E-01	Deshiere A, Duchemin-Pelletier E, Spreux E, et al. 2013 32 1373-83
36	GSM707314	GSE28569	MCF10A cells, CK2beta depletion	Breast_MFC10A_CK2beta_Depletion	Breast	3.16E-04	3.21E-01	1.63E-01	2.38E-01	
37	GSM707315	GSE28569	MCF10A cells, CK2beta depletion	Breast_MFC10A_CK2beta_Depletion	Breast	3.12E-04	3.22E-01	2.51E-04	5.08E-01	
38	GSM707316	GSE28569	MCF10A cells, CK2beta depletion and rescue	Breast_MFC10A_CK2beta_Depletion&Rescue	Breast	2.47E-01	1.34E-01	1.16E-04	5.31E-01	
39	GSM707317	GSE28569	MCF10A cells, CK2beta depletion and rescue	Breast_MFC10A_CK2beta_Depletion&Rescue	Breast	2.44E-01	-1.34E-01	2.86E-01	1.97E-01	
40	GSM820817	GSE33146	DKAT MEGM rep1	Breast_DKAT_MEGM	Breast	5.23E-01	-9.03E-02	6.54E-01	1.12E-01	
41	GSM820818	GSE33146	DKAT MEGM rep2	Breast_DKAT_MEGM	Breast	5.75E-01	8.34E-02	6.54E-01	1.12E-01	
42	GSM820819	GSE33146	DKAT MEGM rep3	Breast_DKAT_MEGM	Breast	4.96E-01	-9.40E-02	6.54E-01	1.12E-01	D'Amato NC, Ostrander JH, Bowie ML, et al. 2012 PLoS One 7 e45684
43	GSM820820	GSE33146	DKAT SCGM day14 rep1	Breast_DKAT_SCGM	Breast	4.58E-02	1.97E-01	1.42E-01	2.41E-01	
44	GSM820821	GSE33146	DKAT SCGM day14 rep2	Breast_DKAT_SCGM	Breast	5.06E-02	1.94E-01	7.84E-02	2.75E-01	
45	GSM820822	GSE33146	DKAT SCGM day14 rep3	Breast_DKAT_SCGM	Breast	4.21E-02	2.00E-01	9.63E-02	2.64E-01	
46	GSM966553	GSE39356	MCF_7_vector Rep_1	Breast_MCF7_Vector	Breast	1.61E-04	-3.35E-01	1.84E-02	-3.52E-01	
47	GSM966554	GSE39356	MCF_7_vector Rep_2	Breast_MCF7_Vector	Breast	1.69E-05	-3.75E-01	5.76E-03	-4.00E-01	Cai J, Guan H, Fang L, et al. 2013 J Clin Invest. 123 566-79
48	GSM966555	GSE39356	MCF_7_374a Rep_1	Breast_MCF7_shmiR274a	Breast	2.10E-11	5.62E-01	1.21E-08	7.53E-01	
49	GSM966556	GSE39356	MCF_7_374a Rep_2	Breast_MCF7_shmiR274a	Breast	6.63E-07	4.27E-01	3.30E-06	6.26E-01	
50	GSM880848	GSE36081	HMLE+Twist-ER cells, pMIG	Breast_HMLE_TWIST-ER_pMIG	Breast	7.47E-16	6.72E-01	6.09E-10	7.98E-01	
51	GSM880849	GSE36081	HMLE+Twist-ER cells, pMIG	Breast_HMLE_TWIST-ER_pMIG	Breast	5.09E-16	6.76E-01	6.09E-10	7.98E-01	
52	GSM880850	GSE36081	HMLE+Twist-ER cells, pMIG	Breast_HMLE_TWIST-ER_pMIG	Breast	5.19E-15	6.53E-01	1.15E-09	7.86E-01	Cieply B, Riley P 4th, Pifer PM, et al. 2012 Cancer Res 72 2440-53
53	GSM880851	GSE36081	HMLE+Twist-ER cells, GRHL2/pMIG	Breast_HMLE_TWIST-ER_GRHL2/pMIG	Breast	3.15E-03	2.73E-01	6.54E-03	3.89E-01	
54	GSM880852	GSE36081	HMLE+Twist-ER cells, GRHL2/pMIG	Breast_HMLE_TWIST-ER_GRHL2/pMIG	Breast	4.19E-04	3.18E-01	1.07E-02	3.69E-01	
55	GSM880853	GSE36081	HMLE+Twist-ER cells, GRHL2/pMIG	Breast_HMLE_TWIST-ER_GRHL2/pMIG	Breast	1.46E-04	3.39E-01	4.76E-03	4.01E-01	
56	GSM678008	GSE27424	EPC2-hTERT_scram_lowCalsium	Esophageal_EPC2-hTETR_scram_lowCalsium	Esophageal	1.92E-01	1.44E-01	1.30E-01	2.46E-01	
57	GSM678009	GSE27424	EPC2-hTERT_scram_lowCalsium	Esophageal_EPC2-hTETR_scram_lowCalsium	Esophageal	2.19E-01	1.38E-01	1.17E-01	2.52E-01	
58	GSM678010	GSE27424	EPC2-hTERT_scram_lowCalsium	Esophageal_EPC2-hTETR_scram_lowCalsium	Esophageal	1.54E-01	1.53E-01	8.47E-02	2.71E-01	
59	GSM678011	GSE27424	EPC2-hTERT_Notch3KD_lowCalsium	Esophageal_EPC2-hTETR_NOTCH3KD_lowCalsiu	Esophageal	1.16E-08	4.80E-01	6.25E-05	5.36E-01	
60	GSM678012	GSE27424	EPC2-hTERT_Notch3KD_lowCalsium	Esophageal_EPC2-hTETR_NOTCH3KD_lowCalsiu	Esophageal	1.82E-08	4.74E-01	6.25E-05	5.36E-01	
61	GSM678013	GSE27424	EPC2-hTERT_Notch3KD_lowCalsium	Esophageal_EPC2-hTETR_NOTCH3KD_lowCalsiu	Esophageal	4.39E-08	4.62E-01	8.08E-05	5.29E-01	Ohashi S, Natsuizaka M, Naganuma S, et al. Cancer Res 71 6836-47
62	GSM678014	GSE27424	EPC2-hTERT_scram_highCalsium	Esophageal_EPC2-hTETR_scram_highCalsium	Esophageal	2.49E-01	-1.32E-01	3.92E-01	1.67E-01	
63	GSM678015	GSE27424	EPC2-hTERT_scram_highCalsium	Esophageal_EPC2-hTETR_scram_highCalsium	Esophageal	1.92E-01	-1.44E-01	3.92E-01	1.67E-01	
64	GSM678016	GSE27424	EPC2-hTERT_scram_highCalsium	Esophageal_EPC2-hTETR_scram_highCalsium	Esophageal	2.63E-01	-1.30E-01	3.92E-01	1.67E-01	
65	GSM678017	GSE27424	EPC2-hTERT_Notch3KD_highCalsium	Esophageal_EPC2-hTETR_NOTCH3KD_highCalsi	Esophageal	3.04E-05	3.62E-01	8.92E-04	4.57E-01	
66	GSM678018	GSE27424	EPC2-hTERT_Notch3KD_highCalsium	Esophageal_EPC2-hTETR_NOTCH3KD_highCalsi	Esophageal	1.11E-05	3.79E-01	1.26E-03	4.46E-01	
67	GSM678019	GSE27424	EPC2-hTERT_Notch3KD_highCalsium	Esophageal_EPC2-hTETR_NOTCH3KD_highCalsi	Esophageal	3.66E-05	3.59E-01	1.01E-03	4.53E-01	
68	JQ2008041701	E-MTAB-114	Keratinocyte_emptyVector	Foreskin_Keratinocyte_emptyVector	Foreskin	6.77E-02	-1.84E-01	2.83E-01	-1.94E-01	
69	JQ2008041702	E-MTAB-114	Keratinocyte_emptyVector	Foreskin_Keratinocyte_emptyVector	Foreskin	2.91E-02	-2.11E-01	1.56E-01	-2.35E-01	
70	JQ2008041703	E-MTAB-114	Keratinocyte_HPV16_E6	Foreskin_Keratinocyte_HPVE6	Foreskin	2.17E-01	-1.39E-01	2.72E-01	1.97E-01	Hellner K, Mar J, Fang F, et al. 2009 Virology 391 57-63
71	JQ2008041704	E-MTAB-114	Keratinocyte_HPV16_E6	Foreskin_Keratinocyte_HPVE6	Foreskin	1.73E-01	-1.49E-01	4.71E-01	1.50E-01	
72	JQ2008041705	E-MTAB-114	Keratinocyte_HPV16_E7	Foreskin_Keratinocyte_HPVE7	Foreskin	1.29E-01	-1.60E-01	3.92E-01	1.67E-01	
73	JQ2008041706	E-MTAB-114	Keratinocyte_HPV16_E7	Foreskin_Keratinocyte_HPVE7	Foreskin	6.77E-02	-1.84E-01	2.99E-01	1.90E-01	
74	GSM406502	GSE16194	H441 vector control	Lung_H441_Vector	Lung	3.59E-07	-4.32E-01	4.99E-04	-4.75E-01	
75	GSM406503	GSE16194	H441 Snail over-expressing	Lung_H441_OverExpress_SNAIL	Lung	3.85E-02	-2.03E-01	5.70E-01	1.29E-01	
76	GSM406504	GSE16194	H292 vector control	Lung_H292_Vector	Lung	2.63E-03	-2.74E-01	3.65E-03	-4.08E-01	Yanagawa J, Walser TC, Zhu LX, et al. 2009 Clin Cancer Res 15 6820-9
77	GSM406505	GSE16194	H292 Snail over-expressing	Lung_H292_OverExpress_SNAIL	Lung	7.56E-03	-2.48E-01	8.96E-03	-3.74E-01	
78	GSM406506	GSE16194	H1437 vector control	Lung_H1437_Vector	Lung	3.14E-06	-3.99E-01	9.38E-05	-5.25E-01	
79	GSM406507	GSE16194	H1437 Snail over-expressing	Lung_H1437_OverExpress_SNAIL	Lung	1.09E-06	4.16E-01	3.58E-06	6.11E-01	
80	GSM124905	GSE5457	A549_rep1	Lung_A549	Lung	2.02E-03	2.80E-01	1.91E-04	5.05E-01	
81	GSM124906	GSE5457	A549_rep2	Lung_A549	Lung	4.81E-04	3.10E-01	5.94E-05	5.38E-01	
82	GSM124907	GSE5457	A549_exposure_TGFbeta1_rep1	Lung_A549_TGFβ	Lung	2.40E-06	4.04E-01	1.13E-05	5.83E-01	
83	GSM124908	GSE5457	A549_exposure_TGFbeta1_rep2	Lung_A549_TGFβ	Lung	3.74E-07	4.32E-01	1.76E-05	5.71E-01	Malizia AP, Keating DT, Smith SM, et al. 2008 Am J Physiol Lung Cell Mol Physiol. 295 L451-60
84	GSM124909	GSE5457	AAKata_rep1	Lung_AAKata	Lung	1.51E-05	3.74E-01	1.58E-07	6.83E-01	
85	GSM124910	GSE5457	AAKata_rep2	Lung_AAKata	Lung	2.14E-05	3.68E-01	1.97E-07	6.78E-01	
86	GSM124992	GSE5457	AAkata_exposure_TGFbeta1_rep1	Lung_AAKata_TGFβ	Lung	2.48E-07	4.38E-01	1.27E-05	5.80E-01	
87	GSM124993	GSE5457	AAkata_exposure_TGFbeta1_rep2	Lung_AAKata_TGFβ	Lung	1.39E-06	4.12E-01	1.13E-05	5.83E-01	
88	GSM590166	GSE23952	Panc-1 Untreated Control 1	Pancreas_PANC1_Control	Pancreas	1.50E-10	5.34E-01	1.65E-06	6.29E-01	
89	GSM590167	GSE23952	Panc-1 Untreated Control 2	Pancreas_PANC1_Control	Pancreas	2.46E-10	5.28E-01	1.38E-06	6.33E-01	
90	GSM590168	GSE23952	Panc-1 Untreated Control 3	Pancreas_PANC1_Control	Pancreas	5.95E-10	5.17E-01	2.23E-06	6.22E-01	Maupin KA, Sinha A, Eugster E, et al. 2010 PLoS One 5 e13002
91	GSM590169	GSE23952	Panc-1 TGFb Treated 1 48 hr	Pancreas_PANC1_TGFβ	Pancreas	2.30E-13	6.05E-01	1.53E-08	7.31E-01	
92	GSM590170	GSE23952	Panc-1 TGFb Treated 2 48 hr	Pancreas_PANC1_TGFβ	Pancreas	5.52E-13	5.96E-01	1.53E-08	7.31E-01	
93	GSM590171	GSE23952	Panc-1 TGFb Treated 3 48 hr	Pancreas_PANC1_TGFβ	Pancreas	3.15E-13	6.02E-01	2.67E-08	7.20E-01	

Table S4A. EMT score (tumour generic EMT signature) of clinical samples.

Index	Sample	Source	Disease	Label	Generic.EMT. Tumour.pv	Generic.EMT.Tumour.Ksscore	Generic.EMT.Tumour.pv.from Reduced Set	Generic.EMT.Tumour .Ksscore from Reduced Set
1	GSM158711	GSE6891	Acute Myeloid Leukaemia	Blood	6.73E-02	1.30E-01	1.96E-03	4.05E-01
2	GSM158712	GSE6891	Acute Myeloid Leukaemia	Blood	1.16E-01	1.16E-01	3.55E-01	1.65E-01
3	GSM158713	GSE6891	Acute Myeloid Leukaemia	Blood	2.40E-02	1.53E-01	8.89E-02	2.52E-01
4	GSM158714	GSE6891	Acute Myeloid Leukaemia	Blood	2.24E-03	1.95E-01	3.16E-02	3.02E-01
5	GSM158715	GSE6891	Acute Myeloid Leukaemia	Blood	3.05E-02	-1.48E-01	2.19E-01	2.00E-01
6	GSM158716	GSE6891	Acute Myeloid Leukaemia	Blood	5.46E-02	1.35E-01	2.26E-01	1.98E-01
7	GSM158717	GSE6891	Acute Myeloid Leukaemia	Blood	4.59E-02	1.39E-01	9.82E-02	2.47E-01
8	GSM158718	GSE6891	Acute Myeloid Leukaemia	Blood	5.01E-02	1.37E-01	2.55E-02	3.11E-01
9	GSM158719	GSE6891	Acute Myeloid Leukaemia	Blood	1.39E-01	1.11E-01	3.23E-01	1.72E-01
10	GSM158720	GSE6891	Acute Myeloid Leukaemia	Blood	2.77E-02	1.50E-01	6.43E-02	2.69E-01
11	GSM158721	GSE6891	Acute Myeloid Leukaemia	Blood	3.50E-02	1.45E-01	8.89E-02	2.52E-01
12	GSM158722	GSE6891	Acute Myeloid Leukaemia	Blood	4.59E-02	1.39E-01	1.55E-01	2.22E-01
13	GSM158723	GSE6891	Acute Myeloid Leukaemia	Blood	1.01E-02	1.70E-01	2.67E-02	3.09E-01
14	GSM158724	GSE6891	Acute Myeloid Leukaemia	Blood	8.91E-02	1.23E-01	5.54E-02	2.76E-01
15	GSM158725	GSE6891	Acute Myeloid Leukaemia	Blood	5.47E-03	1.81E-01	6.43E-02	2.69E-01
16	GSM158726	GSE6891	Acute Myeloid Leukaemia	Blood	9.63E-02	1.21E-01	1.96E-01	2.07E-01
17	GSM158727	GSE6891	Acute Myeloid Leukaemia	Blood	1.21E-01	-1.15E-01	1.16E-01	2.38E-01
18	GSM158728	GSE6891	Acute Myeloid Leukaemia	Blood	1.30E-01	1.13E-01	1.02E-01	2.45E-01
19	GSM158729	GSE6891	Acute Myeloid Leukaemia	Blood	1.25E-02	1.66E-01	1.75E-01	2.14E-01
20	GSM158730	GSE6891	Acute Myeloid Leukaemia	Blood	1.79E-02	1.59E-01	2.67E-02	3.09E-01
21	GSM158731	GSE6891	Acute Myeloid Leukaemia	Blood	8.56E-03	1.73E-01	9.34E-03	3.51E-01
22	GSM158732	GSE6891	Acute Myeloid Leukaemia	Blood	1.35E-04	2.36E-01	7.97E-06	5.56E-01
23	GSM158733	GSE6891	Acute Myeloid Leukaemia	Blood	2.52E-02	1.52E-01	2.05E-02	3.20E-01
24	GSM158734	GSE6891	Acute Myeloid Leukaemia	Blood	1.16E-01	1.16E-01	3.72E-02	2.94E-01
25	GSM158735	GSE6891	Acute Myeloid Leukaemia	Blood	3.05E-02	1.48E-01	1.16E-01	2.38E-01
26	GSM158736	GSE6891	Acute Myeloid Leukaemia	Blood	2.38E-03	1.94E-01	3.72E-02	2.94E-01
27	GSM158737	GSE6891	Acute Myeloid Leukaemia	Blood	1.06E-02	1.69E-01	5.31E-02	2.78E-01
28	GSM158738	GSE6891	Acute Myeloid Leukaemia	Blood	1.39E-01	1.11E-01	1.55E-01	2.22E-01
29	GSM158739	GSE6891	Acute Myeloid Leukaemia	Blood	9.54E-03	1.71E-01	2.05E-02	3.20E-01
30	GSM158740	GSE6891	Acute Myeloid Leukaemia	Blood	1.31E-02	1.65E-01	5.75E-04	4.43E-01
31	GSM158741	GSE6891	Acute Myeloid Leukaemia	Blood	1.39E-01	-1.11E-01	1.80E-01	2.12E-01
32	GSM158742	GSE6891	Acute Myeloid Leukaemia	Blood	2.53E-03	1.93E-01	1.04E-02	3.47E-01
33	GSM158743	GSE6891	Acute Myeloid Leukaemia	Blood	1.95E-01	1.01E-01	1.19E-02	3.42E-01
34	GSM158744	GSE6891	Acute Myeloid Leukaemia	Blood	4.59E-02	1.39E-01	3.16E-02	3.02E-01
35	GSM158745	GSE6891	Acute Myeloid Leukaemia	Blood	1.34E-01	1.12E-01	8.56E-02	2.54E-01
36	GSM158746	GSE6891	Acute Myeloid Leukaemia	Blood	5.94E-02	-1.33E-01	9.82E-02	2.47E-01
37	GSM158747	GSE6891	Acute Myeloid Leukaemia	Blood	1.01E-02	1.70E-01	4.55E-02	2.85E-01
38	GSM158748	GSE6891	Acute Myeloid Leukaemia	Blood	1.44E-01	1.10E-01	1.80E-02	3.25E-01
39	GSM158749	GSE6891	Acute Myeloid Leukaemia	Blood	1.04E-01	1.19E-01	8.56E-02	2.54E-01
40	GSM158750	GSE6891	Acute Myeloid Leukaemia	Blood	5.16E-03	1.82E-01	9.85E-03	3.49E-01
41	GSM158751	GSE6891	Acute Myeloid Leukaemia	Blood	6.13E-03	1.79E-01	8.56E-02	2.54E-01
42	GSM158752	GSE6891	Acute Myeloid Leukaemia	Blood	2.91E-02	1.49E-01	2.02E-01	2.05E-01
43	GSM158753	GSE6891	Acute Myeloid Leukaemia	Blood	8.91E-02	1.23E-01	4.20E-03	3.80E-01
44	GSM158754	GSE6891	Acute Myeloid Leukaemia	Blood	2.52E-02	1.52E-01	7.73E-02	2.60E-01
45	GSM158755	GSE6891	Acute Myeloid Leukaemia	Blood	5.94E-02	1.33E-01	3.89E-02	2.92E-01
46	GSM158756	GSE6891	Acute Myeloid Leukaemia	Blood	5.16E-03	1.82E-01	3.21E-03	3.89E-01
47	GSM158757	GSE6891	Acute Myeloid Leukaemia	Blood	6.73E-02	1.30E-01	1.43E-02	3.34E-01
48	GSM158758	GSE6891	Acute Myeloid Leukaemia	Blood	1.60E-01	1.07E-01	3.46E-01	1.67E-01
49	GSM158759	GSE6891	Acute Myeloid Leukaemia	Blood	1.08E-01	-1.18E-01	2.67E-02	3.09E-01
50	GSM158760	GSE6891	Acute Myeloid Leukaemia	Blood	9.73E-04	2.08E-01	3.72E-02	2.94E-01
51	GSM158761	GSE6891	Acute Myeloid Leukaemia	Blood	1.04E-01	1.19E-01	6.43E-02	2.69E-01
52	GSM158762	GSE6891	Acute Myeloid Leukaemia	Blood	1.63E-03	2.00E-01	2.44E-03	3.98E-01
53	GSM158763	GSE6891	Acute Myeloid Leukaemia	Blood	5.01E-02	1.37E-01	1.19E-02	3.42E-01
54	GSM158764	GSE6891	Acute Myeloid Leukaemia	Blood	1.01E-02	1.70E-01	5.10E-02	2.80E-01
55	GSM158765	GSE6891	Acute Myeloid Leukaemia	Blood	8.91E-02	-1.23E-01	1.75E-01	2.14E-01
56	GSM158766	GSE6891	Acute Myeloid Leukaemia	Blood	9.26E-02	1.22E-01	1.02E-01	2.45E-01
57	GSM158767	GSE6891	Acute Myeloid Leukaemia	Blood	5.69E-02	1.34E-01	6.43E-02	2.69E-01
58	GSM158768	GSE6891	Acute Myeloid Leukaemia	Blood	8.91E-02	-1.23E-01	2.02E-01	2.05E-01
59	GSM158769	GSE6891	Acute Myeloid Leukaemia	Blood	1.53E-02	1.62E-01	1.25E-02	3.40E-01
60	GSM158770	GSE6891	Acute Myeloid Leukaemia	Blood	1.83E-01	1.03E-01	1.80E-01	2.12E-01
61	GSM158771	GSE6891	Acute Myeloid Leukaemia	Blood	1.16E-01	-1.16E-01	9.23E-02	2.50E-01
62	GSM158772	GSE6891	Acute Myeloid Leukaemia	Blood	1.38E-02	1.64E-01	3.72E-02	2.94E-01
63	GSM158773	GSE6891	Acute Myeloid Leukaemia	Blood	5.94E-02	-1.33E-01	1.12E-01	2.40E-01
64	GSM158774	GSE6891	Acute Myeloid Leukaemia	Blood	4.95E-04	2.18E-01	3.97E-03	3.82E-01
65	GSM158775	GSE6891	Acute Myeloid Leukaemia	Blood	1.97E-03	1.97E-01	5.10E-02	2.80E-01
66	GSM158776	GSE6891	Acute Myeloid Leukaemia	Blood	3.19E-02	-1.47E-01	1.02E-01	2.45E-01
67	GSM158777	GSE6891	Acute Myeloid Leukaemia	Blood	1.70E-02	1.60E-01	3.72E-02	2.94E-01
68	GSM158778	GSE6891	Acute Myeloid Leukaemia	Blood	1.88E-02	1.58E-01	5.31E-02	2.78E-01
69	GSM158779	GSE6891	Acute Myeloid Leukaemia	Blood	4.34E-03	1.85E-01	2.05E-02	3.20E-01

70	GSM158780	GSE6891	Acute Myeloid Leukaemia	Blood	8.57E-02	-1.24E-01	2.02E-01	2.05E-01
71	GSM158781	GSE6891	Acute Myeloid Leukaemia	Blood	5.69E-02	1.34E-01	7.43E-02	2.62E-01
72	GSM158782	GSE6891	Acute Myeloid Leukaemia	Blood	1.25E-01	1.14E-01	1.02E-01	2.45E-01
73	GSM158783	GSE6891	Acute Myeloid Leukaemia	Blood	1.12E-02	1.68E-01	3.16E-02	3.02E-01
74	GSM158784	GSE6891	Acute Myeloid Leukaemia	Blood	5.69E-02	1.34E-01	1.19E-02	3.42E-01
75	GSM158785	GSE6891	Acute Myeloid Leukaemia	Blood	3.05E-02	1.48E-01	4.06E-02	2.90E-01
76	GSM158786	GSE6891	Acute Myeloid Leukaemia	Blood	4.20E-02	-1.41E-01	3.23E-01	1.72E-01
77	GSM158787	GSE6891	Acute Myeloid Leukaemia	Blood	5.47E-03	1.81E-01	2.55E-02	3.11E-01
78	GSM158788	GSE6891	Acute Myeloid Leukaemia	Blood	1.06E-02	1.69E-01	2.15E-02	3.18E-01
79	GSM158789	GSE6891	Acute Myeloid Leukaemia	Blood	7.25E-03	1.76E-01	1.47E-03	4.14E-01
80	GSM158790	GSE6891	Acute Myeloid Leukaemia	Blood	1.18E-02	1.67E-01	3.89E-02	2.92E-01
81	GSM158791	GSE6891	Acute Myeloid Leukaemia	Blood	6.51E-04	2.14E-01	8.56E-02	2.54E-01
82	GSM158792	GSE6891	Acute Myeloid Leukaemia	Blood	4.59E-02	1.39E-01	7.43E-02	2.62E-01
83	GSM158793	GSE6891	Acute Myeloid Leukaemia	Blood	1.53E-03	2.01E-01	4.36E-02	2.87E-01
84	GSM158794	GSE6891	Acute Myeloid Leukaemia	Blood	4.20E-02	1.41E-01	2.55E-02	3.11E-01
85	GSM158795	GSE6891	Acute Myeloid Leukaemia	Blood	1.95E-01	-1.01E-01	2.55E-02	3.11E-01
86	GSM158796	GSE6891	Acute Myeloid Leukaemia	Blood	3.19E-02	1.47E-01	2.26E-01	1.98E-01
87	GSM158797	GSE6891	Acute Myeloid Leukaemia	Blood	2.40E-02	1.53E-01	3.16E-02	3.02E-01
88	GSM158798	GSE6891	Acute Myeloid Leukaemia	Blood	4.39E-02	1.40E-01	4.55E-02	2.85E-01
89	GSM158799	GSE6891	Acute Myeloid Leukaemia	Blood	2.64E-02	1.51E-01	1.37E-01	2.29E-01
90	GSM158800	GSE6891	Acute Myeloid Leukaemia	Blood	5.79E-03	1.80E-01	3.16E-02	3.02E-01
91	GSM158801	GSE6891	Acute Myeloid Leukaemia	Blood	2.28E-01	-9.62E-02	1.75E-01	2.14E-01
92	GSM158802	GSE6891	Acute Myeloid Leukaemia	Blood	1.77E-01	-1.04E-01	2.08E-01	2.03E-01
93	GSM158803	GSE6891	Acute Myeloid Leukaemia	Blood	1.74E-03	1.99E-01	9.85E-03	3.49E-01
94	GSM158804	GSE6891	Acute Myeloid Leukaemia	Blood	1.46E-02	1.63E-01	2.26E-01	1.98E-01
95	GSM158805	GSE6891	Acute Myeloid Leukaemia	Blood	5.94E-02	1.33E-01	1.58E-02	3.30E-01
96	GSM158806	GSE6891	Acute Myeloid Leukaemia	Blood	1.44E-01	-1.10E-01	8.89E-02	2.52E-01
97	GSM158807	GSE6891	Acute Myeloid Leukaemia	Blood	8.10E-03	1.74E-01	8.12E-03	3.56E-01
98	GSM158808	GSE6891	Acute Myeloid Leukaemia	Blood	1.25E-01	-1.14E-01	1.37E-01	2.29E-01
99	GSM158809	GSE6891	Acute Myeloid Leukaemia	Blood	6.46E-02	-1.31E-01	6.31E-03	3.65E-01
100	GSM158810	GSE6891	Acute Myeloid Leukaemia	Blood	5.01E-02	1.37E-01	1.55E-01	2.22E-01
101	GSM158811	GSE6891	Acute Myeloid Leukaemia	Blood	7.92E-02	1.26E-01	1.55E-01	2.22E-01
102	GSM158812	GSE6891	Acute Myeloid Leukaemia	Blood	3.50E-02	-1.45E-01	8.56E-02	2.54E-01
103	GSM158813	GSE6891	Acute Myeloid Leukaemia	Blood	1.65E-01	1.06E-01	3.55E-01	1.65E-01
104	GSM158814	GSE6891	Acute Myeloid Leukaemia	Blood	5.23E-02	1.36E-01	8.56E-02	2.54E-01
105	GSM158815	GSE6891	Acute Myeloid Leukaemia	Blood	6.46E-02	-1.31E-01	1.32E-01	2.31E-01
106	GSM158816	GSE6891	Acute Myeloid Leukaemia	Blood	2.42E-01	-9.42E-02	8.56E-02	2.54E-01
107	GSM158817	GSE6891	Acute Myeloid Leukaemia	Blood	1.21E-01	1.15E-01	6.43E-02	2.69E-01
108	GSM158818	GSE6891	Acute Myeloid Leukaemia	Blood	5.79E-03	1.80E-01	3.72E-02	2.94E-01
109	GSM158819	GSE6891	Acute Myeloid Leukaemia	Blood	1.46E-02	-1.63E-01	1.76E-01	-2.14E-01
110	GSM158820	GSE6891	Acute Myeloid Leukaemia	Blood	2.40E-02	1.53E-01	2.15E-02	3.18E-01
111	GSM158821	GSE6891	Acute Myeloid Leukaemia	Blood	2.52E-02	1.52E-01	5.10E-02	2.80E-01
112	GSM158822	GSE6891	Acute Myeloid Leukaemia	Blood	1.08E-01	-1.18E-01	1.02E-01	2.45E-01
113	GSM158823	GSE6891	Acute Myeloid Leukaemia	Blood	4.79E-02	-1.38E-01	1.02E-01	2.45E-01
114	GSM158824	GSE6891	Acute Myeloid Leukaemia	Blood	2.91E-02	1.49E-01	2.55E-02	3.11E-01
115	GSM158825	GSE6891	Acute Myeloid Leukaemia	Blood	5.47E-03	1.81E-01	6.17E-02	2.71E-01
116	GSM158826	GSE6891	Acute Myeloid Leukaemia	Blood	9.54E-03	1.71E-01	1.60E-01	2.20E-01
117	GSM158827	GSE6891	Acute Myeloid Leukaemia	Blood	4.59E-02	-1.39E-01	1.16E-01	2.38E-01
118	GSM158828	GSE6891	Acute Myeloid Leukaemia	Blood	5.94E-02	1.33E-01	5.31E-02	2.78E-01
119	GSM158829	GSE6891	Acute Myeloid Leukaemia	Blood	9.26E-02	-1.22E-01	2.26E-01	1.98E-01
120	GSM158830	GSE6891	Acute Myeloid Leukaemia	Blood	2.38E-03	1.94E-01	3.72E-02	2.94E-01
121	GSM158831	GSE6891	Acute Myeloid Leukaemia	Blood	5.46E-02	-1.35E-01	1.80E-01	2.12E-01
122	GSM158832	GSE6891	Acute Myeloid Leukaemia	Blood	1.01E-02	1.70E-01	1.16E-01	2.38E-01
123	GSM158833	GSE6891	Acute Myeloid Leukaemia	Blood	8.24E-02	1.25E-01	1.37E-01	2.29E-01
124	GSM158834	GSE6891	Acute Myeloid Leukaemia	Blood	3.34E-02	1.46E-01	7.43E-02	2.62E-01
125	GSM158835	GSE6891	Acute Myeloid Leukaemia	Blood	1.79E-02	1.59E-01	1.25E-02	3.40E-01
126	GSM158836	GSE6891	Acute Myeloid Leukaemia	Blood	2.40E-02	1.53E-01	1.19E-02	3.42E-01
127	GSM158837	GSE6891	Acute Myeloid Leukaemia	Blood	1.18E-02	1.67E-01	8.56E-02	2.54E-01
128	GSM158838	GSE6891	Acute Myeloid Leukaemia	Blood	1.71E-01	-1.05E-01	2.15E-02	3.18E-01
129	GSM158839	GSE6891	Acute Myeloid Leukaemia	Blood	6.48E-03	1.78E-01	1.25E-02	3.40E-01
130	GSM158840	GSE6891	Acute Myeloid Leukaemia	Blood	7.25E-03	1.76E-01	3.72E-02	2.94E-01
131	GSM158841	GSE6891	Acute Myeloid Leukaemia	Blood	6.46E-02	1.31E-01	6.17E-02	2.71E-01
132	GSM158842	GSE6891	Acute Myeloid Leukaemia	Blood	1.65E-01	1.06E-01	4.55E-02	2.85E-01
133	GSM158843	GSE6891	Acute Myeloid Leukaemia	Blood	1.31E-02	1.65E-01	9.34E-03	3.51E-01
134	GSM158844	GSE6891	Acute Myeloid Leukaemia	Blood	9.63E-02	1.21E-01	1.32E-01	2.31E-01
135	GSM158845	GSE6891	Acute Myeloid Leukaemia	Blood	1.18E-02	1.67E-01	6.17E-02	2.71E-01
136	GSM158846	GSE6891	Acute Myeloid Leukaemia	Blood	1.16E-01	1.16E-01	8.56E-02	2.54E-01
137	GSM158847	GSE6891	Acute Myeloid Leukaemia	Blood	4.20E-02	1.41E-01	3.16E-02	3.02E-01
138	GSM158848	GSE6891	Acute Myeloid Leukaemia	Blood	2.21E-01	9.72E-02	4.55E-02	2.85E-01
139	GSM158849	GSE6891	Acute Myeloid Leukaemia	Blood	8.24E-02	1.25E-01	4.55E-02	2.85E-01
140	GSM158850	GSE6891	Acute Myeloid Leukaemia	Blood	2.14E-01	9.82E-02	4.55E-02	2.85E-01
141	GSM158851	GSE6891	Acute Myeloid Leukaemia	Blood	5.69E-02	1.34E-01	1.02E-01	2.45E-01
142	GSM158852	GSE6891	Acute Myeloid Leukaemia	Blood	1.46E-02	1.63E-01	4.36E-02	2.87E-01
143	GSM158853	GSE6891	Acute Myeloid Leukaemia	Blood	1.71E-01	1.05E-01	2.19E-01	2.00E-01

144	GSM158854	GSE6891	Acute Myeloid Leukaemia	Blood	1.39E-01	-1.11E-01	6.43E-02	2.69E-01
145	GSM158855	GSE6891	Acute Myeloid Leukaemia	Blood	4.20E-02	-1.41E-01	1.02E-01	2.45E-01
146	GSM158856	GSE6891	Acute Myeloid Leukaemia	Blood	5.46E-02	1.35E-01	8.56E-02	2.54E-01
147	GSM158857	GSE6891	Acute Myeloid Leukaemia	Blood	5.01E-02	1.37E-01	9.82E-02	2.47E-01
148	GSM158858	GSE6891	Acute Myeloid Leukaemia	Blood	1.21E-01	-1.15E-01	5.31E-02	2.78E-01
149	GSM158859	GSE6891	Acute Myeloid Leukaemia	Blood	1.12E-01	-1.17E-01	2.58E-01	1.89E-01
150	GSM158860	GSE6891	Acute Myeloid Leukaemia	Blood	1.54E-01	1.08E-01	1.21E-01	2.36E-01
151	GSM158861	GSE6891	Acute Myeloid Leukaemia	Blood	4.39E-02	1.40E-01	2.58E-01	1.89E-01
152	GSM158862	GSE6891	Acute Myeloid Leukaemia	Blood	1.21E-01	1.15E-01	1.12E-01	2.40E-01
153	GSM158863	GSE6891	Acute Myeloid Leukaemia	Blood	7.92E-02	-1.26E-01	2.26E-01	1.98E-01
154	GSM158864	GSE6891	Acute Myeloid Leukaemia	Blood	3.56E-01	-8.04E-02	2.02E-01	2.05E-01
155	GSM158865	GSE6891	Acute Myeloid Leukaemia	Blood	1.79E-02	1.59E-01	1.12E-01	2.40E-01
156	GSM158866	GSE6891	Acute Myeloid Leukaemia	Blood	1.97E-02	1.57E-01	8.89E-02	2.52E-01
157	GSM158867	GSE6891	Acute Myeloid Leukaemia	Blood	3.50E-02	1.45E-01	1.32E-01	2.31E-01
158	GSM158868	GSE6891	Acute Myeloid Leukaemia	Blood	9.54E-03	1.71E-01	7.32E-04	4.36E-01
159	GSM158869	GSE6891	Acute Myeloid Leukaemia	Blood	1.35E-03	2.03E-01	4.75E-02	2.83E-01
160	GSM158870	GSE6891	Acute Myeloid Leukaemia	Blood	2.53E-03	1.93E-01	2.37E-04	4.69E-01
161	GSM158871	GSE6891	Acute Myeloid Leukaemia	Blood	9.63E-02	1.21E-01	3.16E-02	3.02E-01
162	GSM158872	GSE6891	Acute Myeloid Leukaemia	Blood	2.14E-01	-9.82E-02	7.43E-02	2.62E-01
163	GSM158873	GSE6891	Acute Myeloid Leukaemia	Blood	8.91E-02	1.23E-01	1.75E-01	2.14E-01
164	GSM158874	GSE6891	Acute Myeloid Leukaemia	Blood	3.19E-02	1.47E-01	2.67E-02	3.09E-01
165	GSM158875	GSE6891	Acute Myeloid Leukaemia	Blood	2.07E-02	1.56E-01	1.47E-03	4.14E-01
166	GSM158876	GSE6891	Acute Myeloid Leukaemia	Blood	2.35E-01	9.52E-02	1.80E-01	2.12E-01
167	GSM158877	GSE6891	Acute Myeloid Leukaemia	Blood	1.97E-03	1.97E-01	1.43E-02	3.34E-01
168	GSM158878	GSE6891	Acute Myeloid Leukaemia	Blood	4.23E-01	7.34E-02	1.18E-01	2.37E-01
169	GSM158879	GSE6891	Acute Myeloid Leukaemia	Blood	4.20E-02	1.41E-01	3.72E-02	2.94E-01
170	GSM158880	GSE6891	Acute Myeloid Leukaemia	Blood	2.88E-01	-8.83E-02	1.37E-01	2.29E-01
171	GSM158881	GSE6891	Acute Myeloid Leukaemia	Blood	4.01E-02	1.42E-01	1.50E-02	3.32E-01
172	GSM158882	GSE6891	Acute Myeloid Leukaemia	Blood	1.89E-01	-1.02E-01	3.72E-02	2.94E-01
173	GSM158883	GSE6891	Acute Myeloid Leukaemia	Blood	3.42E-03	1.88E-01	4.88E-03	3.74E-01
174	GSM158884	GSE6891	Acute Myeloid Leukaemia	Blood	1.18E-02	1.67E-01	9.51E-03	3.50E-01
175	GSM158885	GSE6891	Acute Myeloid Leukaemia	Blood	5.94E-02	1.33E-01	1.21E-01	2.36E-01
176	GSM158886	GSE6891	Acute Myeloid Leukaemia	Blood	1.08E-01	1.18E-01	4.55E-02	2.85E-01
177	GSM158887	GSE6891	Acute Myeloid Leukaemia	Blood	2.64E-01	-9.13E-02	3.32E-01	1.70E-01
178	GSM158888	GSE6891	Acute Myeloid Leukaemia	Blood	7.97E-04	2.11E-01	3.89E-02	2.92E-01
179	GSM158889	GSE6891	Acute Myeloid Leukaemia	Blood	1.49E-01	-1.09E-01	9.82E-02	2.47E-01
180	GSM158890	GSE6891	Acute Myeloid Leukaemia	Blood	1.49E-01	1.09E-01	1.55E-01	2.22E-01
181	GSM158891	GSE6891	Acute Myeloid Leukaemia	Blood	5.94E-02	1.33E-01	3.16E-02	3.02E-01
182	GSM158892	GSE6891	Acute Myeloid Leukaemia	Blood	7.01E-02	1.29E-01	8.56E-02	2.54E-01
183	GSM158893	GSE6891	Acute Myeloid Leukaemia	Blood	9.54E-03	1.71E-01	5.31E-02	2.78E-01
184	GSM158894	GSE6891	Acute Myeloid Leukaemia	Blood	2.64E-01	-9.13E-02	4.55E-02	2.85E-01
185	GSM158895	GSE6891	Acute Myeloid Leukaemia	Blood	4.09E-03	1.86E-01	5.31E-02	2.78E-01
186	GSM158896	GSE6891	Acute Myeloid Leukaemia	Blood	9.54E-03	1.71E-01	2.05E-02	3.20E-01
187	GSM158897	GSE6891	Acute Myeloid Leukaemia	Blood	3.67E-02	1.44E-01	2.05E-02	3.20E-01
188	GSM158898	GSE6891	Acute Myeloid Leukaemia	Blood	4.34E-03	1.85E-01	2.05E-02	3.20E-01
189	GSM158899	GSE6891	Acute Myeloid Leukaemia	Blood	1.25E-01	1.14E-01	2.58E-01	1.89E-01
190	GSM158900	GSE6891	Acute Myeloid Leukaemia	Blood	4.09E-03	1.86E-01	7.70E-03	3.58E-01
191	GSM158901	GSE6891	Acute Myeloid Leukaemia	Blood	5.16E-03	1.82E-01	2.15E-02	3.18E-01
192	GSM158902	GSE6891	Acute Myeloid Leukaemia	Blood	3.84E-02	1.43E-01	2.67E-02	3.09E-01
193	GSM158903	GSE6891	Acute Myeloid Leukaemia	Blood	1.97E-02	1.57E-01	2.05E-02	3.20E-01
194	GSM158904	GSE6891	Acute Myeloid Leukaemia	Blood	1.53E-02	1.62E-01	5.31E-02	2.78E-01
195	GSM158905	GSE6891	Acute Myeloid Leukaemia	Blood	3.86E-03	1.87E-01	2.55E-02	3.11E-01
196	GSM158906	GSE6891	Acute Myeloid Leukaemia	Blood	1.01E-02	1.70E-01	1.02E-01	2.45E-01
197	GSM158907	GSE6891	Acute Myeloid Leukaemia	Blood	1.97E-02	1.57E-01	3.72E-02	2.94E-01
198	GSM158908	GSE6891	Acute Myeloid Leukaemia	Blood	1.16E-01	-1.16E-01	2.08E-01	2.03E-01
199	GSM158909	GSE6891	Acute Myeloid Leukaemia	Blood	1.97E-03	1.97E-01	5.10E-02	2.80E-01
200	GSM158910	GSE6891	Acute Myeloid Leukaemia	Blood	1.34E-01	-1.12E-01	1.16E-01	2.38E-01
201	GSM158911	GSE6891	Acute Myeloid Leukaemia	Blood	1.85E-03	1.98E-01	7.43E-02	2.62E-01
202	GSM158912	GSE6891	Acute Myeloid Leukaemia	Blood	1.34E-01	-1.12E-01	4.77E-01	1.40E-01
203	GSM158913	GSE6891	Acute Myeloid Leukaemia	Blood	1.79E-02	1.59E-01	9.34E-03	3.51E-01
204	GSM158914	GSE6891	Acute Myeloid Leukaemia	Blood	1.53E-03	2.01E-01	4.55E-02	2.85E-01
205	GSM158915	GSE6891	Acute Myeloid Leukaemia	Blood	8.24E-02	1.25E-01	4.55E-02	2.85E-01
206	GSM158916	GSE6891	Acute Myeloid Leukaemia	Blood	1.04E-01	1.19E-01	3.72E-02	2.94E-01
207	GSM158917	GSE6891	Acute Myeloid Leukaemia	Blood	1.44E-03	2.02E-01	9.34E-03	3.51E-01
208	GSM158918	GSE6891	Acute Myeloid Leukaemia	Blood	4.34E-03	1.85E-01	2.25E-02	3.16E-01
209	GSM158919	GSE6891	Acute Myeloid Leukaemia	Blood	1.31E-02	1.65E-01	6.67E-03	3.63E-01
210	GSM158920	GSE6891	Acute Myeloid Leukaemia	Blood	4.79E-02	1.38E-01	6.43E-02	2.69E-01
211	GSM158921	GSE6891	Acute Myeloid Leukaemia	Blood	3.67E-02	1.44E-01	1.58E-02	3.30E-01
212	GSM158922	GSE6891	Acute Myeloid Leukaemia	Blood	1.97E-02	1.57E-01	4.75E-02	2.83E-01
213	GSM158923	GSE6891	Acute Myeloid Leukaemia	Blood	5.16E-03	1.82E-01	1.58E-02	3.30E-01
214	GSM158924	GSE6891	Acute Myeloid Leukaemia	Blood	1.25E-02	1.66E-01	2.05E-02	3.20E-01
215	GSM158925	GSE6891	Acute Myeloid Leukaemia	Blood	1.61E-02	1.61E-01	6.17E-02	2.71E-01
216	GSM158926	GSE6891	Acute Myeloid Leukaemia	Blood	3.50E-02	1.45E-01	2.44E-03	3.98E-01
217	GSM158927	GSE6891	Acute Myeloid Leukaemia	Blood	1.39E-01	1.11E-01	1.96E-01	2.07E-01

218	GSM158928	GSE6891	Acute Myeloid Leukaemia	Blood	3.49E-04	2.23E-01	5.98E-03	3.67E-01
219	GSM158929	GSE6891	Acute Myeloid Leukaemia	Blood	7.30E-02	1.28E-01	1.02E-01	2.45E-01
220	GSM158930	GSE6891	Acute Myeloid Leukaemia	Blood	2.77E-02	1.50E-01	3.89E-02	2.92E-01
221	GSM158931	GSE6891	Acute Myeloid Leukaemia	Blood	1.12E-02	1.68E-01	9.85E-03	3.49E-01
222	GSM158932	GSE6891	Acute Myeloid Leukaemia	Blood	1.88E-02	1.58E-01	3.72E-02	2.94E-01
223	GSM158933	GSE6891	Acute Myeloid Leukaemia	Blood	5.46E-02	1.35E-01	5.31E-02	2.78E-01
224	GSM158934	GSE6891	Acute Myeloid Leukaemia	Blood	4.39E-02	1.40E-01	1.96E-01	2.07E-01
225	GSM158935	GSE6891	Acute Myeloid Leukaemia	Blood	6.46E-02	1.31E-01	6.17E-02	2.71E-01
226	GSM158936	GSE6891	Acute Myeloid Leukaemia	Blood	2.64E-02	1.51E-01	8.03E-02	2.58E-01
227	GSM158937	GSE6891	Acute Myeloid Leukaemia	Blood	6.46E-02	-1.31E-01	2.86E-01	1.82E-01
228	GSM158938	GSE6891	Acute Myeloid Leukaemia	Blood	7.92E-02	1.26E-01	1.37E-01	2.29E-01
229	GSM158939	GSE6891	Acute Myeloid Leukaemia	Blood	8.56E-03	1.73E-01	2.15E-02	3.18E-01
230	GSM158940	GSE6891	Acute Myeloid Leukaemia	Blood	6.46E-02	1.31E-01	2.55E-02	3.11E-01
231	GSM158941	GSE6891	Acute Myeloid Leukaemia	Blood	7.01E-02	1.29E-01	1.43E-02	3.34E-01
232	GSM158942	GSE6891	Acute Myeloid Leukaemia	Blood	3.63E-03	1.88E-01	2.55E-02	3.11E-01
233	GSM158943	GSE6891	Acute Myeloid Leukaemia	Blood	7.30E-02	-1.28E-01	1.12E-01	2.40E-01
234	GSM158944	GSE6891	Acute Myeloid Leukaemia	Blood	6.48E-03	1.78E-01	4.36E-02	2.87E-01
235	GSM158945	GSE6891	Acute Myeloid Leukaemia	Blood	2.91E-02	1.49E-01	5.31E-02	2.78E-01
236	GSM158946	GSE6891	Acute Myeloid Leukaemia	Blood	3.23E-03	1.89E-01	3.72E-02	2.94E-01
237	GSM158947	GSE6891	Acute Myeloid Leukaemia	Blood	1.60E-01	1.07E-01	8.56E-02	2.54E-01
238	GSM158948	GSE6891	Acute Myeloid Leukaemia	Blood	6.86E-03	-1.77E-01	8.56E-02	2.54E-01
239	GSM158949	GSE6891	Acute Myeloid Leukaemia	Blood	4.39E-02	1.40E-01	2.67E-02	3.09E-01
240	GSM158950	GSE6891	Acute Myeloid Leukaemia	Blood	1.79E-02	1.59E-01	9.27E-04	4.29E-01
241	GSM158951	GSE6891	Acute Myeloid Leukaemia	Blood	5.94E-02	1.33E-01	8.56E-02	2.54E-01
242	GSM158952	GSE6891	Acute Myeloid Leukaemia	Blood	8.56E-03	1.73E-01	1.72E-02	3.27E-01
243	GSM158953	GSE6891	Acute Myeloid Leukaemia	Blood	1.21E-01	1.15E-01	9.34E-03	3.51E-01
244	GSM158954	GSE6891	Acute Myeloid Leukaemia	Blood	9.11E-04	2.09E-01	2.15E-02	3.18E-01
245	GSM158955	GSE6891	Acute Myeloid Leukaemia	Blood	1.21E-01	1.15E-01	1.75E-01	2.14E-01
246	GSM158956	GSE6891	Acute Myeloid Leukaemia	Blood	3.84E-02	1.43E-01	1.32E-01	2.31E-01
247	GSM158957	GSE6891	Acute Myeloid Leukaemia	Blood	5.94E-02	1.33E-01	9.82E-02	2.47E-01
248	GSM158958	GSE6891	Acute Myeloid Leukaemia	Blood	1.04E-01	1.19E-01	3.16E-02	3.02E-01
249	GSM158959	GSE6891	Acute Myeloid Leukaemia	Blood	2.53E-03	1.93E-01	9.34E-03	3.51E-01
250	GSM158960	GSE6891	Acute Myeloid Leukaemia	Blood	1.21E-01	1.15E-01	8.56E-02	2.54E-01
251	GSM158961	GSE6891	Acute Myeloid Leukaemia	Blood	5.16E-03	1.82E-01	3.16E-02	3.02E-01
252	GSM158962	GSE6891	Acute Myeloid Leukaemia	Blood	5.23E-02	1.36E-01	2.67E-02	3.09E-01
253	GSM158963	GSE6891	Acute Myeloid Leukaemia	Blood	4.39E-02	1.40E-01	2.19E-01	2.00E-01
254	GSM158964	GSE6891	Acute Myeloid Leukaemia	Blood	9.73E-04	2.08E-01	2.55E-02	3.11E-01
255	GSM158965	GSE6891	Acute Myeloid Leukaemia	Blood	9.26E-02	1.22E-01	5.31E-02	2.78E-01
256	GSM158966	GSE6891	Acute Myeloid Leukaemia	Blood	6.86E-03	1.77E-01	1.25E-02	3.40E-01
257	GSM158967	GSE6891	Acute Myeloid Leukaemia	Blood	6.46E-02	-1.31E-01	3.16E-02	3.02E-01
258	GSM158968	GSE6891	Acute Myeloid Leukaemia	Blood	6.46E-02	1.31E-01	6.43E-02	2.69E-01
259	GSM158969	GSE6891	Acute Myeloid Leukaemia	Blood	1.97E-03	1.97E-01	7.70E-03	3.58E-01
260	GSM158970	GSE6891	Acute Myeloid Leukaemia	Blood	7.01E-02	1.29E-01	2.02E-01	2.05E-01
261	GSM158971	GSE6891	Acute Myeloid Leukaemia	Blood	4.79E-02	1.38E-01	4.36E-02	2.87E-01
262	GSM158972	GSE6891	Acute Myeloid Leukaemia	Blood	8.24E-02	1.25E-01	4.55E-02	2.85E-01
263	GSM158973	GSE6891	Acute Myeloid Leukaemia	Blood	1.95E-01	-1.01E-01	3.46E-01	1.67E-01
264	GSM158974	GSE6891	Acute Myeloid Leukaemia	Blood	7.25E-03	1.76E-01	8.56E-02	2.54E-01
265	GSM158975	GSE6891	Acute Myeloid Leukaemia	Blood	8.57E-02	1.24E-01	1.02E-01	2.45E-01
266	GSM158976	GSE6891	Acute Myeloid Leukaemia	Blood	4.34E-03	1.85E-01	1.72E-02	3.27E-01
267	GSM158977	GSE6891	Acute Myeloid Leukaemia	Blood	1.49E-01	1.09E-01	1.21E-01	2.36E-01
268	GSM158978	GSE6891	Acute Myeloid Leukaemia	Blood	9.26E-02	1.22E-01	8.56E-02	2.54E-01
269	GSM158979	GSE6891	Acute Myeloid Leukaemia	Blood	9.73E-04	2.08E-01	1.50E-02	3.32E-01
270	GSM158980	GSE6891	Acute Myeloid Leukaemia	Blood	8.52E-04	2.10E-01	3.16E-02	3.02E-01
271	GSM158981	GSE6891	Acute Myeloid Leukaemia	Blood	5.94E-02	-1.33E-01	2.58E-01	1.89E-01
272	GSM158982	GSE6891	Acute Myeloid Leukaemia	Blood	2.64E-02	1.51E-01	1.55E-01	2.22E-01
273	GSM158983	GSE6891	Acute Myeloid Leukaemia	Blood	2.64E-02	1.51E-01	4.55E-02	2.85E-01
274	GSM158984	GSE6891	Acute Myeloid Leukaemia	Blood	1.95E-01	1.01E-01	2.81E-01	1.83E-01
275	GSM158985	GSE6891	Acute Myeloid Leukaemia	Blood	1.83E-01	-1.03E-01	2.86E-01	1.82E-01
276	GSM158986	GSE6891	Acute Myeloid Leukaemia	Blood	6.08E-04	2.15E-01	3.72E-02	2.94E-01
277	GSM158987	GSE6891	Acute Myeloid Leukaemia	Blood	2.69E-03	1.92E-01	4.36E-02	2.87E-01
278	GSM158988	GSE6891	Acute Myeloid Leukaemia	Blood	1.30E-01	1.13E-01	5.31E-02	2.78E-01
279	GSM158989	GSE6891	Acute Myeloid Leukaemia	Blood	4.60E-03	1.84E-01	1.72E-02	3.27E-01
280	GSM158990	GSE6891	Acute Myeloid Leukaemia	Blood	2.42E-01	-9.42E-02	1.55E-01	2.22E-01
281	GSM158991	GSE6891	Acute Myeloid Leukaemia	Blood	3.56E-01	8.04E-02	1.21E-01	2.36E-01
282	GSM158992	GSE6891	Acute Myeloid Leukaemia	Blood	4.01E-02	1.42E-01	3.15E-01	1.74E-01
283	GSM158993	GSE6891	Acute Myeloid Leukaemia	Blood	1.70E-02	1.60E-01	9.85E-03	3.49E-01
284	GSM158994	GSE6891	Acute Myeloid Leukaemia	Blood	1.83E-01	-1.03E-01	1.32E-01	2.31E-01
285	GSM158995	GSE6891	Acute Myeloid Leukaemia	Blood	7.92E-02	1.26E-01	8.56E-02	2.54E-01
286	GSM158996	GSE6891	Acute Myeloid Leukaemia	Blood	7.30E-02	-1.28E-01	3.23E-01	1.72E-01
287	GSM158997	GSE6891	Acute Myeloid Leukaemia	Blood	4.39E-02	1.40E-01	1.16E-01	2.38E-01
288	GSM158998	GSE6891	Acute Myeloid Leukaemia	Blood	8.91E-02	1.23E-01	3.72E-02	2.94E-01
289	GSM158999	GSE6891	Acute Myeloid Leukaemia	Blood	4.20E-02	1.41E-01	1.37E-01	2.29E-01
290	GSM159000	GSE6891	Acute Myeloid Leukaemia	Blood	3.56E-01	-8.04E-02	3.55E-01	1.65E-01
291	GSM159001	GSE6891	Acute Myeloid Leukaemia	Blood	2.86E-03	1.91E-01	6.17E-02	2.71E-01

292	GSM159002	GSE6891	Acute Myeloid Leukaemia	Blood	8.24E-02	-1.25E-01	2.02E-01	2.05E-01
293	GSM159003	GSE6891	Acute Myeloid Leukaemia	Blood	4.79E-02	1.38E-01	2.15E-02	3.18E-01
294	GSM159004	GSE6891	Acute Myeloid Leukaemia	Blood	1.70E-02	1.60E-01	4.36E-02	2.87E-01
295	GSM159005	GSE6891	Acute Myeloid Leukaemia	Blood	1.16E-01	1.16E-01	8.35E-02	2.56E-01
296	GSM159006	GSE6891	Acute Myeloid Leukaemia	Blood	2.64E-02	1.51E-01	4.55E-02	2.85E-01
297	GSM159007	GSE6891	Acute Myeloid Leukaemia	Blood	2.44E-04	2.28E-01	2.15E-02	3.18E-01
298	GSM159008	GSE6891	Acute Myeloid Leukaemia	Blood	1.97E-02	1.57E-01	4.36E-02	2.87E-01
299	GSM159009	GSE6891	Acute Myeloid Leukaemia	Blood	5.79E-03	1.80E-01	1.72E-02	3.27E-01
300	GSM159010	GSE6891	Acute Myeloid Leukaemia	Blood	1.25E-01	1.14E-01	3.16E-02	3.02E-01
301	GSM159011	GSE6891	Acute Myeloid Leukaemia	Blood	4.34E-03	1.85E-01	3.40E-03	3.87E-01
302	GSM159012	GSE6891	Acute Myeloid Leukaemia	Blood	1.44E-03	2.02E-01	3.72E-02	2.94E-01
303	GSM159013	GSE6891	Acute Myeloid Leukaemia	Blood	9.63E-02	1.21E-01	5.10E-02	2.80E-01
304	GSM159014	GSE6891	Acute Myeloid Leukaemia	Blood	2.18E-02	-1.55E-01	8.35E-02	-2.56E-01
305	GSM159015	GSE6891	Acute Myeloid Leukaemia	Blood	8.24E-02	-1.25E-01	9.82E-02	2.47E-01
306	GSM159016	GSE6891	Acute Myeloid Leukaemia	Blood	2.86E-03	1.91E-01	1.58E-02	3.30E-01
307	GSM159017	GSE6891	Acute Myeloid Leukaemia	Blood	5.23E-02	1.36E-01	1.75E-01	2.14E-01
308	GSM159018	GSE6891	Acute Myeloid Leukaemia	Blood	2.24E-03	1.95E-01	5.54E-02	2.76E-01
309	GSM159019	GSE6891	Acute Myeloid Leukaemia	Blood	2.52E-02	1.52E-01	7.43E-02	2.62E-01
310	GSM159020	GSE6891	Acute Myeloid Leukaemia	Blood	1.12E-02	1.68E-01	3.03E-03	3.91E-01
311	GSM159021	GSE6891	Acute Myeloid Leukaemia	Blood	2.53E-03	1.93E-01	7.43E-02	2.62E-01
312	GSM159022	GSE6891	Acute Myeloid Leukaemia	Blood	7.92E-02	1.26E-01	3.16E-02	3.02E-01
313	GSM159023	GSE6891	Acute Myeloid Leukaemia	Blood	3.34E-02	1.46E-01	9.23E-02	2.50E-01
314	GSM159024	GSE6891	Acute Myeloid Leukaemia	Blood	6.97E-04	2.13E-01	2.15E-02	3.18E-01
315	GSM159025	GSE6891	Acute Myeloid Leukaemia	Blood	7.67E-03	1.75E-01	8.89E-02	2.52E-01
316	GSM159026	GSE6891	Acute Myeloid Leukaemia	Blood	5.39E-05	2.48E-01	9.34E-03	3.51E-01
317	GSM159027	GSE6891	Acute Myeloid Leukaemia	Blood	3.34E-02	1.46E-01	8.56E-02	2.54E-01
318	GSM159028	GSE6891	Acute Myeloid Leukaemia	Blood	6.13E-03	1.79E-01	4.36E-02	2.87E-01
319	GSM159029	GSE6891	Acute Myeloid Leukaemia	Blood	3.84E-02	1.43E-01	4.55E-02	2.85E-01
320	GSM159030	GSE6891	Acute Myeloid Leukaemia	Blood	9.04E-03	1.72E-01	4.36E-02	2.87E-01
321	GSM159031	GSE6891	Acute Myeloid Leukaemia	Blood	5.94E-02	1.33E-01	6.43E-02	2.69E-01
322	GSM159032	GSE6891	Acute Myeloid Leukaemia	Blood	1.83E-01	1.03E-01	2.02E-01	2.05E-01
323	GSM159033	GSE6891	Acute Myeloid Leukaemia	Blood	4.59E-02	1.39E-01	3.15E-01	1.74E-01
324	GSM159034	GSE6891	Acute Myeloid Leukaemia	Blood	6.73E-02	1.30E-01	2.55E-02	3.11E-01
325	GSM159035	GSE6891	Acute Myeloid Leukaemia	Blood	9.04E-03	1.72E-01	1.66E-02	3.29E-01
326	GSM159036	GSE6891	Acute Myeloid Leukaemia	Blood	2.52E-02	1.52E-01	2.15E-02	3.18E-01
327	GSM159037	GSE6891	Acute Myeloid Leukaemia	Blood	8.91E-02	1.23E-01	2.86E-01	1.82E-01
328	GSM159038	GSE6891	Acute Myeloid Leukaemia	Blood	9.26E-02	1.22E-01	2.15E-02	3.18E-01
329	GSM159039	GSE6891	Acute Myeloid Leukaemia	Blood	1.00E-01	-1.20E-01	1.32E-01	2.31E-01
330	GSM159040	GSE6891	Acute Myeloid Leukaemia	Blood	5.69E-02	1.34E-01	1.96E-01	2.07E-01
331	GSM159041	GSE6891	Acute Myeloid Leukaemia	Blood	2.49E-01	9.33E-02	2.15E-02	3.18E-01
332	GSM159042	GSE6891	Acute Myeloid Leukaemia	Blood	6.73E-02	-1.30E-01	3.72E-02	2.94E-01
333	GSM159043	GSE6891	Acute Myeloid Leukaemia	Blood	8.57E-02	1.24E-01	3.72E-02	2.94E-01
334	GSM159044	GSE6891	Acute Myeloid Leukaemia	Blood	6.19E-02	1.32E-01	1.02E-01	2.45E-01
335	GSM159045	GSE6891	Acute Myeloid Leukaemia	Blood	6.19E-02	1.32E-01	3.16E-02	3.02E-01
336	GSM159046	GSE6891	Acute Myeloid Leukaemia	Blood	1.06E-02	1.69E-01	3.97E-03	3.82E-01
337	GSM159047	GSE6891	Acute Myeloid Leukaemia	Blood	2.69E-03	1.92E-01	2.80E-02	3.07E-01
338	GSM159048	GSE6891	Acute Myeloid Leukaemia	Blood	1.39E-01	-1.11E-01	1.19E-02	3.42E-01
339	GSM159049	GSE6891	Acute Myeloid Leukaemia	Blood	7.60E-02	1.27E-01	9.82E-02	2.47E-01
340	GSM159050	GSE6891	Acute Myeloid Leukaemia	Blood	1.25E-01	1.14E-01	2.51E-01	1.91E-01
341	GSM159051	GSE6891	Acute Myeloid Leukaemia	Blood	8.24E-02	1.25E-01	4.55E-02	2.85E-01
342	GSM159052	GSE6891	Acute Myeloid Leukaemia	Blood	4.79E-02	1.38E-01	2.67E-02	3.09E-01
343	GSM159053	GSE6891	Acute Myeloid Leukaemia	Blood	1.53E-02	1.62E-01	1.55E-01	2.22E-01
344	GSM159054	GSE6891	Acute Myeloid Leukaemia	Blood	1.88E-02	1.58E-01	1.06E-01	2.43E-01
345	GSM159055	GSE6891	Acute Myeloid Leukaemia	Blood	1.61E-02	-1.61E-01	7.43E-02	2.62E-01
346	GSM159056	GSE6891	Acute Myeloid Leukaemia	Blood	1.25E-01	1.14E-01	1.72E-02	3.27E-01
347	GSM159057	GSE6891	Acute Myeloid Leukaemia	Blood	4.79E-02	1.38E-01	2.05E-02	3.20E-01
348	GSM159058	GSE6891	Acute Myeloid Leukaemia	Blood	1.71E-01	-1.05E-01	1.55E-01	2.22E-01
349	GSM159059	GSE6891	Acute Myeloid Leukaemia	Blood	3.04E-03	1.90E-01	5.38E-04	4.45E-01
350	GSM159060	GSE6891	Acute Myeloid Leukaemia	Blood	6.86E-03	1.77E-01	1.72E-02	3.27E-01
351	GSM159061	GSE6891	Acute Myeloid Leukaemia	Blood	6.08E-04	2.15E-01	8.56E-02	2.54E-01
352	GSM159062	GSE6891	Acute Myeloid Leukaemia	Blood	1.25E-02	1.66E-01	2.67E-02	3.09E-01
353	GSM159063	GSE6891	Acute Myeloid Leukaemia	Blood	2.64E-02	1.51E-01	8.56E-02	2.54E-01
354	GSM159064	GSE6891	Acute Myeloid Leukaemia	Blood	6.13E-03	1.79E-01	6.17E-02	2.71E-01
355	GSM159065	GSE6891	Acute Myeloid Leukaemia	Blood	1.00E-01	-1.20E-01	9.82E-02	2.47E-01
356	GSM159066	GSE6891	Acute Myeloid Leukaemia	Blood	2.40E-02	-1.53E-01	1.16E-01	2.38E-01
357	GSM159067	GSE6891	Acute Myeloid Leukaemia	Blood	1.30E-01	-1.13E-01	8.56E-02	2.54E-01
358	GSM159068	GSE6891	Acute Myeloid Leukaemia	Blood	1.16E-01	-1.16E-01	3.23E-01	1.72E-01
359	GSM159069	GSE6891	Acute Myeloid Leukaemia	Blood	4.39E-02	1.40E-01	1.21E-01	2.36E-01
360	GSM159070	GSE6891	Acute Myeloid Leukaemia	Blood	1.54E-01	1.08E-01	3.89E-02	2.92E-01
361	GSM159071	GSE6891	Acute Myeloid Leukaemia	Blood	1.60E-01	1.07E-01	8.56E-02	2.54E-01
362	GSM159072	GSE6891	Acute Myeloid Leukaemia	Blood	4.60E-03	1.84E-01	5.31E-02	2.78E-01
363	GSM159073	GSE6891	Acute Myeloid Leukaemia	Blood	3.67E-02	1.44E-01	1.19E-02	3.42E-01
364	GSM159074	GSE6891	Acute Myeloid Leukaemia	Blood	5.01E-02	-1.37E-01	1.75E-01	2.14E-01
365	GSM159075	GSE6891	Acute Myeloid Leukaemia	Blood	1.38E-02	1.64E-01	7.43E-02	2.62E-01

366	GSM159076	GSE6891	Acute Myeloid Leukaemia	Blood	2.53E-03	1.93E-01	5.10E-02	2.80E-01
367	GSM159077	GSE6891	Acute Myeloid Leukaemia	Blood	1.25E-01	1.14E-01	2.15E-02	3.18E-01
368	GSM159078	GSE6891	Acute Myeloid Leukaemia	Blood	1.70E-02	1.60E-01	8.56E-02	2.54E-01
369	GSM159079	GSE6891	Acute Myeloid Leukaemia	Blood	2.42E-05	2.58E-01	1.36E-07	6.45E-01
370	GSM159080	GSE6891	Acute Myeloid Leukaemia	Blood	7.67E-03	1.75E-01	2.67E-02	3.09E-01
371	GSM159081	GSE6891	Acute Myeloid Leukaemia	Blood	7.30E-02	1.28E-01	2.86E-01	1.82E-01
372	GSM159082	GSE6891	Acute Myeloid Leukaemia	Blood	1.25E-01	1.14E-01	6.43E-02	2.69E-01
373	GSM159083	GSE6891	Acute Myeloid Leukaemia	Blood	4.39E-02	1.40E-01	4.06E-02	2.90E-01
374	GSM159084	GSE6891	Acute Myeloid Leukaemia	Blood	1.18E-02	1.67E-01	2.15E-02	3.18E-01
375	GSM159085	GSE6891	Acute Myeloid Leukaemia	Blood	5.69E-02	-1.34E-01	5.31E-02	2.78E-01
376	GSM159086	GSE6891	Acute Myeloid Leukaemia	Blood	1.49E-01	-1.09E-01	4.88E-03	3.74E-01
377	GSM159087	GSE6891	Acute Myeloid Leukaemia	Blood	3.84E-02	1.43E-01	1.32E-01	2.31E-01
378	GSM159088	GSE6891	Acute Myeloid Leukaemia	Blood	5.46E-02	1.35E-01	2.44E-03	3.98E-01
379	GSM159089	GSE6891	Acute Myeloid Leukaemia	Blood	1.53E-02	1.62E-01	4.55E-02	2.85E-01
380	GSM159090	GSE6891	Acute Myeloid Leukaemia	Blood	5.46E-02	-1.35E-01	2.02E-01	2.05E-01
381	GSM159091	GSE6891	Acute Myeloid Leukaemia	Blood	1.65E-01	1.06E-01	2.51E-01	1.91E-01
382	GSM159092	GSE6891	Acute Myeloid Leukaemia	Blood	7.60E-02	-1.27E-01	4.67E-01	1.42E-01
383	GSM159093	GSE6891	Acute Myeloid Leukaemia	Blood	5.23E-02	1.36E-01	1.43E-02	3.34E-01
384	GSM159094	GSE6891	Acute Myeloid Leukaemia	Blood	3.84E-02	-1.43E-01	1.16E-01	2.38E-01
385	GSM159095	GSE6891	Acute Myeloid Leukaemia	Blood	1.79E-02	1.59E-01	1.06E-01	2.43E-01
386	GSM159096	GSE6891	Acute Myeloid Leukaemia	Blood	7.25E-03	1.76E-01	2.05E-02	3.20E-01
387	GSM159097	GSE6891	Acute Myeloid Leukaemia	Blood	7.92E-02	1.26E-01	1.55E-01	2.22E-01
388	GSM159098	GSE6891	Acute Myeloid Leukaemia	Blood	1.97E-02	1.57E-01	1.02E-01	2.45E-01
389	GSM159099	GSE6891	Acute Myeloid Leukaemia	Blood	4.09E-03	1.86E-01	4.50E-04	4.50E-01
390	GSM159100	GSE6891	Acute Myeloid Leukaemia	Blood	1.34E-01	-1.12E-01	2.15E-02	3.18E-01
391	GSM159101	GSE6891	Acute Myeloid Leukaemia	Blood	1.16E-01	1.16E-01	1.42E-01	2.27E-01
392	GSM159102	GSE6891	Acute Myeloid Leukaemia	Blood	4.59E-02	1.39E-01	3.72E-02	2.94E-01
393	GSM159103	GSE6891	Acute Myeloid Leukaemia	Blood	1.08E-01	-1.18E-01	1.37E-01	2.29E-01
394	GSM159104	GSE6891	Acute Myeloid Leukaemia	Blood	4.95E-04	2.18E-01	5.10E-02	2.80E-01
395	GSM159105	GSE6891	Acute Myeloid Leukaemia	Blood	1.46E-02	1.63E-01	3.16E-02	3.02E-01
396	GSM159106	GSE6891	Acute Myeloid Leukaemia	Blood	2.35E-01	9.52E-02	1.16E-01	2.38E-01
397	GSM159107	GSE6891	Acute Myeloid Leukaemia	Blood	2.24E-03	1.95E-01	1.89E-02	3.23E-01
398	GSM159108	GSE6891	Acute Myeloid Leukaemia	Blood	1.38E-02	1.64E-01	4.88E-03	3.74E-01
399	GSM159109	GSE6891	Acute Myeloid Leukaemia	Blood	2.96E-01	-8.73E-02	2.51E-01	1.91E-01
400	GSM159110	GSE6891	Acute Myeloid Leukaemia	Blood	3.50E-02	1.45E-01	2.51E-01	1.91E-01
401	GSM159111	GSE6891	Acute Myeloid Leukaemia	Blood	1.30E-01	1.13E-01	8.89E-02	2.52E-01
402	GSM159112	GSE6891	Acute Myeloid Leukaemia	Blood	8.57E-02	1.24E-01	3.72E-02	2.94E-01
403	GSM159113	GSE6891	Acute Myeloid Leukaemia	Blood	1.30E-01	1.13E-01	9.23E-02	2.50E-01
404	GSM159115	GSE6891	Acute Myeloid Leukaemia	Blood	7.45E-04	2.12E-01	9.85E-03	3.49E-01
405	GSM159116	GSE6891	Acute Myeloid Leukaemia	Blood	3.04E-03	1.90E-01	2.08E-03	4.03E-01
406	GSM159117	GSE6891	Acute Myeloid Leukaemia	Blood	8.24E-02	-1.25E-01	2.32E-01	1.96E-01
407	GSM159118	GSE6891	Acute Myeloid Leukaemia	Blood	1.25E-02	1.66E-01	1.80E-02	3.25E-01
408	GSM159119	GSE6891	Acute Myeloid Leukaemia	Blood	1.79E-02	1.59E-01	4.36E-02	2.87E-01
409	GSM159120	GSE6891	Acute Myeloid Leukaemia	Blood	1.18E-02	1.67E-01	8.03E-02	2.58E-01
410	GSM159121	GSE6891	Acute Myeloid Leukaemia	Blood	1.34E-01	-1.12E-01	1.43E-02	3.34E-01
411	GSM159122	GSE6891	Acute Myeloid Leukaemia	Blood	5.68E-04	2.16E-01	1.50E-02	3.32E-01
412	GSM159123	GSE6891	Acute Myeloid Leukaemia	Blood	1.85E-03	1.98E-01	4.88E-03	3.74E-01
413	GSM159124	GSE6891	Acute Myeloid Leukaemia	Blood	4.60E-03	1.84E-01	2.15E-02	3.18E-01
414	GSM159125	GSE6891	Acute Myeloid Leukaemia	Blood	7.67E-03	1.75E-01	7.70E-03	3.58E-01
415	GSM159126	GSE6891	Acute Myeloid Leukaemia	Blood	2.64E-01	9.13E-02	2.67E-02	3.09E-01
416	GSM159127	GSE6891	Acute Myeloid Leukaemia	Blood	1.25E-01	1.14E-01	1.02E-01	2.45E-01
417	GSM159128	GSE6891	Acute Myeloid Leukaemia	Blood	2.91E-02	1.49E-01	8.56E-02	2.54E-01
418	GSM159129	GSE6891	Acute Myeloid Leukaemia	Blood	4.79E-02	1.38E-01	1.72E-02	3.27E-01
419	GSM159130	GSE6891	Acute Myeloid Leukaemia	Blood	1.44E-01	-1.10E-01	3.89E-02	2.92E-01
420	GSM159131	GSE6891	Acute Myeloid Leukaemia	Blood	4.39E-02	-1.40E-01	1.16E-01	2.38E-01
421	GSM159132	GSE6891	Acute Myeloid Leukaemia	Blood	6.46E-02	1.31E-01	5.10E-02	2.80E-01
422	GSM159133	GSE6891	Acute Myeloid Leukaemia	Blood	2.07E-02	1.56E-01	3.72E-02	2.94E-01
423	GSM159134	GSE6891	Acute Myeloid Leukaemia	Blood	1.44E-01	1.10E-01	5.31E-02	2.78E-01
424	GSM159135	GSE6891	Acute Myeloid Leukaemia	Blood	5.01E-02	-1.37E-01	1.02E-01	2.45E-01
425	GSM159136	GSE6891	Acute Myeloid Leukaemia	Blood	1.49E-01	1.09E-01	2.15E-02	3.18E-01
426	GSM159137	GSE6891	Acute Myeloid Leukaemia	Blood	7.01E-02	1.29E-01	2.67E-02	3.09E-01
427	GSM159138	GSE6891	Acute Myeloid Leukaemia	Blood	4.87E-03	1.83E-01	2.15E-02	3.18E-01
428	GSM159139	GSE6891	Acute Myeloid Leukaemia	Blood	3.86E-03	1.87E-01	3.51E-04	4.58E-01
429	GSM159140	GSE6891	Acute Myeloid Leukaemia	Blood	1.11E-03	2.06E-01	5.31E-02	2.78E-01
430	GSM159141	GSE6891	Acute Myeloid Leukaemia	Blood	1.61E-02	-1.61E-01	2.94E-01	1.80E-01
431	GSM159142	GSE6891	Acute Myeloid Leukaemia	Blood	2.64E-01	9.13E-02	1.21E-01	2.36E-01
432	GSM159143	GSE6891	Acute Myeloid Leukaemia	Blood	9.63E-02	1.21E-01	7.43E-02	2.62E-01
433	GSM159144	GSE6891	Acute Myeloid Leukaemia	Blood	1.25E-02	1.66E-01	1.25E-01	2.34E-01
434	GSM159145	GSE6891	Acute Myeloid Leukaemia	Blood	7.01E-02	-1.29E-01	5.77E-02	2.74E-01
435	GSM159146	GSE6891	Acute Myeloid Leukaemia	Blood	1.70E-02	1.60E-01	5.31E-02	2.78E-01
436	GSM159147	GSE6891	Acute Myeloid Leukaemia	Blood	3.47E-01	8.13E-02	1.60E-01	2.20E-01
437	GSM159148	GSE6891	Acute Myeloid Leukaemia	Blood	5.46E-02	1.35E-01	3.89E-02	2.92E-01
438	GSM159149	GSE6891	Acute Myeloid Leukaemia	Blood	1.74E-03	1.99E-01	8.56E-02	2.54E-01
439	GSM159150	GSE6891	Acute Myeloid Leukaemia	Blood	3.84E-02	1.43E-01	1.02E-01	2.45E-01

440	GSM159151	GSE6891	Acute Myeloid Leukaemia	Blood	5.16E-03	1.82E-01	4.55E-02	2.85E-01
441	GSM159152	GSE6891	Acute Myeloid Leukaemia	Blood	1.39E-01	-1.11E-01	7.43E-02	2.62E-01
442	GSM159153	GSE6891	Acute Myeloid Leukaemia	Blood	4.01E-02	1.42E-01	1.55E-01	2.22E-01
443	GSM159154	GSE6891	Acute Myeloid Leukaemia	Blood	1.16E-01	1.16E-01	2.55E-02	3.11E-01
444	GSM159155	GSE6891	Acute Myeloid Leukaemia	Blood	1.49E-01	-1.09E-01	3.89E-02	2.92E-01
445	GSM159156	GSE6891	Acute Myeloid Leukaemia	Blood	4.01E-02	1.42E-01	2.15E-02	3.18E-01
446	GSM159157	GSE6891	Acute Myeloid Leukaemia	Blood	5.69E-02	1.34E-01	4.75E-02	2.83E-01
447	GSM159158	GSE6891	Acute Myeloid Leukaemia	Blood	1.08E-01	1.18E-01	9.58E-02	2.49E-01
448	GSM159159	GSE6891	Acute Myeloid Leukaemia	Blood	2.07E-02	1.56E-01	5.31E-02	2.78E-01
449	GSM159160	GSE6891	Acute Myeloid Leukaemia	Blood	2.49E-01	9.33E-02	5.31E-02	2.78E-01
450	GSM159161	GSE6891	Acute Myeloid Leukaemia	Blood	2.91E-02	-1.49E-01	8.89E-02	2.52E-01
451	GSM159162	GSE6891	Acute Myeloid Leukaemia	Blood	3.19E-02	-1.47E-01	3.72E-02	2.94E-01
452	GSM159163	GSE6891	Acute Myeloid Leukaemia	Blood	8.57E-02	1.24E-01	1.65E-01	2.18E-01
453	GSM159164	GSE6891	Acute Myeloid Leukaemia	Blood	5.69E-02	1.34E-01	1.32E-01	2.31E-01
454	GSM159165	GSE6891	Acute Myeloid Leukaemia	Blood	4.79E-02	1.38E-01	3.72E-02	2.94E-01
455	GSM159166	GSE6891	Acute Myeloid Leukaemia	Blood	6.86E-03	1.77E-01	3.16E-02	3.02E-01
456	GSM159167	GSE6891	Acute Myeloid Leukaemia	Blood	2.29E-02	1.54E-01	3.72E-02	2.94E-01
457	GSM159168	GSE6891	Acute Myeloid Leukaemia	Blood	2.77E-02	1.50E-01	1.89E-02	3.23E-01
458	GSM159169	GSE6891	Acute Myeloid Leukaemia	Blood	1.97E-02	1.57E-01	4.88E-03	3.74E-01
459	GSM159170	GSE6891	Acute Myeloid Leukaemia	Blood	5.16E-03	1.82E-01	3.89E-02	2.92E-01
460	GSM159171	GSE6891	Acute Myeloid Leukaemia	Blood	2.29E-02	1.54E-01	9.82E-02	2.47E-01
461	GSM361321	GSE14468	Acute Myeloid Leukaemia	Blood	8.24E-02	-1.25E-01	1.60E-01	2.20E-01
462	GSM361322	GSE14468	Acute Myeloid Leukaemia	Blood	1.60E-01	-1.07E-01	6.17E-02	2.71E-01
463	GSM361323	GSE14468	Acute Myeloid Leukaemia	Blood	2.86E-03	1.91E-01	6.17E-02	2.71E-01
464	GSM361324	GSE14468	Acute Myeloid Leukaemia	Blood	6.48E-03	1.78E-01	9.82E-02	2.47E-01
465	GSM361325	GSE14468	Acute Myeloid Leukaemia	Blood	6.73E-02	-1.30E-01	2.02E-01	2.05E-01
466	GSM361326	GSE14468	Acute Myeloid Leukaemia	Blood	6.19E-02	-1.32E-01	1.80E-01	2.12E-01
467	GSM361327	GSE14468	Acute Myeloid Leukaemia	Blood	5.16E-03	-1.82E-01	1.71E-01	-2.16E-01
468	GSM361328	GSE14468	Acute Myeloid Leukaemia	Blood	1.97E-03	1.97E-01	3.72E-02	2.94E-01
469	GSM361329	GSE14468	Acute Myeloid Leukaemia	Blood	8.52E-04	2.10E-01	6.17E-02	2.71E-01
470	GSM361330	GSE14468	Acute Myeloid Leukaemia	Blood	3.34E-02	1.46E-01	9.34E-03	3.51E-01
471	GSM361331	GSE14468	Acute Myeloid Leukaemia	Blood	5.01E-02	-1.37E-01	3.16E-02	3.02E-01
472	GSM361332	GSE14468	Acute Myeloid Leukaemia	Blood	9.54E-03	1.71E-01	8.56E-02	2.54E-01
473	GSM361333	GSE14468	Acute Myeloid Leukaemia	Blood	8.24E-02	-1.25E-01	6.17E-02	2.71E-01
474	GSM361334	GSE14468	Acute Myeloid Leukaemia	Blood	8.24E-02	-1.25E-01	1.16E-01	2.38E-01
475	GSM361335	GSE14468	Acute Myeloid Leukaemia	Blood	5.16E-03	1.82E-01	4.36E-02	2.87E-01
476	GSM361336	GSE14468	Acute Myeloid Leukaemia	Blood	7.67E-03	1.75E-01	3.72E-02	2.94E-01
477	GSM361337	GSE14468	Acute Myeloid Leukaemia	Blood	8.91E-02	-1.23E-01	3.72E-02	2.94E-01
478	GSM361338	GSE14468	Acute Myeloid Leukaemia	Blood	8.56E-03	1.73E-01	5.38E-04	4.45E-01
479	GSM361339	GSE14468	Acute Myeloid Leukaemia	Blood	1.60E-01	-1.07E-01	7.43E-02	2.62E-01
480	GSM361340	GSE14468	Acute Myeloid Leukaemia	Blood	2.07E-02	1.56E-01	5.31E-02	2.78E-01
481	GSM361341	GSE14468	Acute Myeloid Leukaemia	Blood	2.77E-02	-1.50E-01	1.37E-01	2.29E-01
482	GSM361342	GSE14468	Acute Myeloid Leukaemia	Blood	1.08E-01	1.18E-01	3.55E-01	1.65E-01
483	GSM361343	GSE14468	Acute Myeloid Leukaemia	Blood	8.24E-02	1.25E-01	3.16E-02	3.02E-01
484	GSM361344	GSE14468	Acute Myeloid Leukaemia	Blood	3.05E-02	1.48E-01	1.96E-01	2.07E-01
485	GSM361345	GSE14468	Acute Myeloid Leukaemia	Blood	3.04E-01	8.63E-02	7.43E-02	2.62E-01
486	GSM361346	GSE14468	Acute Myeloid Leukaemia	Blood	4.01E-02	1.42E-01	2.36E-02	3.14E-01
487	GSM361347	GSE14468	Acute Myeloid Leukaemia	Blood	8.91E-02	1.23E-01	4.55E-02	2.85E-01
488	GSM361348	GSE14468	Acute Myeloid Leukaemia	Blood	2.86E-03	1.91E-01	5.10E-02	2.80E-01
489	GSM361349	GSE14468	Acute Myeloid Leukaemia	Blood	4.39E-02	1.40E-01	2.67E-02	3.09E-01
490	GSM361350	GSE14468	Acute Myeloid Leukaemia	Blood	2.91E-02	1.49E-01	2.67E-02	3.09E-01
491	GSM361351	GSE14468	Acute Myeloid Leukaemia	Blood	2.21E-01	9.72E-02	7.43E-02	2.62E-01
492	GSM361352	GSE14468	Acute Myeloid Leukaemia	Blood	6.46E-02	-1.31E-01	1.16E-01	2.38E-01
493	GSM361353	GSE14468	Acute Myeloid Leukaemia	Blood	2.38E-03	1.94E-01	4.88E-03	3.74E-01
494	GSM361354	GSE14468	Acute Myeloid Leukaemia	Blood	2.10E-03	1.96E-01	9.34E-03	3.51E-01
495	GSM361355	GSE14468	Acute Myeloid Leukaemia	Blood	9.54E-03	1.71E-01	1.16E-01	2.38E-01
496	GSM361356	GSE14468	Acute Myeloid Leukaemia	Blood	1.97E-02	1.57E-01	1.84E-03	4.07E-01
497	GSM361357	GSE14468	Acute Myeloid Leukaemia	Blood	2.40E-02	1.53E-01	2.05E-02	3.20E-01
498	GSM361358	GSE14468	Acute Myeloid Leukaemia	Blood	2.86E-03	1.91E-01	3.89E-02	2.92E-01
499	GSM361359	GSE14468	Acute Myeloid Leukaemia	Blood	2.86E-03	1.91E-01	4.36E-02	2.87E-01
500	GSM361360	GSE14468	Acute Myeloid Leukaemia	Blood	2.91E-02	1.49E-01	3.72E-02	2.94E-01
501	GSM361361	GSE14468	Acute Myeloid Leukaemia	Blood	1.01E-02	1.70E-01	5.31E-02	2.78E-01
502	GSM361362	GSE14468	Acute Myeloid Leukaemia	Blood	7.45E-04	-2.12E-01	4.50E-01	1.45E-01
503	GSM361363	GSE14468	Acute Myeloid Leukaemia	Blood	3.03E-04	2.25E-01	4.55E-02	2.85E-01
504	GSM361364	GSE14468	Acute Myeloid Leukaemia	Blood	3.19E-02	1.47E-01	2.67E-02	3.09E-01
505	GSM361365	GSE14468	Acute Myeloid Leukaemia	Blood	1.38E-02	1.64E-01	1.43E-02	3.34E-01
506	GSM361366	GSE14468	Acute Myeloid Leukaemia	Blood	2.29E-02	1.54E-01	4.36E-02	2.87E-01
507	GSM361367	GSE14468	Acute Myeloid Leukaemia	Blood	4.39E-02	1.40E-01	1.16E-01	2.38E-01
508	GSM361368	GSE14468	Acute Myeloid Leukaemia	Blood	6.86E-03	1.77E-01	5.31E-02	2.78E-01
509	GSM361369	GSE14468	Acute Myeloid Leukaemia	Blood	4.20E-02	1.41E-01	2.86E-01	1.82E-01
510	GSM361370	GSE14468	Acute Myeloid Leukaemia	Blood	4.34E-03	1.85E-01	2.67E-02	3.09E-01
511	GSM361371	GSE14468	Acute Myeloid Leukaemia	Blood	3.05E-02	1.48E-01	6.31E-03	3.65E-01
512	GSM361372	GSE14468	Acute Myeloid Leukaemia	Blood	2.01E-01	-1.00E-01	2.55E-02	3.11E-01
513	GSM361373	GSE14468	Acute Myeloid Leukaemia	Blood	6.73E-02	-1.30E-01	5.31E-02	2.78E-01

514	GSM361374	GSE14468	Acute Myeloid Leukaemia	Blood	2.18E-02	1.55E-01	2.08E-03	4.03E-01
515	GSM361375	GSE14468	Acute Myeloid Leukaemia	Blood	7.01E-02	-1.29E-01	2.86E-01	1.82E-01
516	GSM361376	GSE14468	Acute Myeloid Leukaemia	Blood	3.42E-03	1.88E-01	5.31E-02	2.78E-01
517	GSM361377	GSE14468	Acute Myeloid Leukaemia	Blood	1.08E-01	-1.18E-01	7.43E-02	2.62E-01
518	GSM361378	GSE14468	Acute Myeloid Leukaemia	Blood	9.26E-02	1.22E-01	6.69E-02	2.67E-01
519	GSM361379	GSE14468	Acute Myeloid Leukaemia	Blood	1.74E-03	1.99E-01	2.15E-02	3.18E-01
520	GSM361380	GSE14468	Acute Myeloid Leukaemia	Blood	7.30E-02	-1.28E-01	8.89E-02	2.52E-01
521	GSM361381	GSE14468	Acute Myeloid Leukaemia	Blood	2.07E-02	1.56E-01	1.55E-01	2.22E-01
522	GSM361382	GSE14468	Acute Myeloid Leukaemia	Blood	1.16E-01	1.16E-01	1.96E-01	2.07E-01
523	GSM361383	GSE14468	Acute Myeloid Leukaemia	Blood	1.97E-02	1.57E-01	8.56E-02	2.54E-01
524	GSM361384	GSE14468	Acute Myeloid Leukaemia	Blood	8.57E-02	1.24E-01	8.03E-02	2.58E-01
525	GSM361385	GSE14468	Acute Myeloid Leukaemia	Blood	7.67E-03	1.75E-01	3.72E-02	2.94E-01
526	GSM311598	GSE12417	Acute Myeloid Leukaemia	Blood	4.39E-02	1.40E-01	1.75E-01	2.14E-01
527	GSM311599	GSE12417	Acute Myeloid Leukaemia	Blood	3.23E-03	1.89E-01	1.50E-02	3.32E-01
528	GSM311600	GSE12417	Acute Myeloid Leukaemia	Blood	1.88E-02	1.58E-01	5.54E-02	2.76E-01
529	GSM311601	GSE12417	Acute Myeloid Leukaemia	Blood	2.69E-03	1.92E-01	7.73E-02	2.60E-01
530	GSM311602	GSE12417	Acute Myeloid Leukaemia	Blood	1.71E-01	1.05E-01	5.16E-03	3.72E-01
531	GSM311603	GSE12417	Acute Myeloid Leukaemia	Blood	3.19E-02	1.47E-01	1.16E-01	2.38E-01
532	GSM311604	GSE12417	Acute Myeloid Leukaemia	Blood	3.21E-01	-8.43E-02	5.31E-02	2.78E-01
533	GSM311605	GSE12417	Acute Myeloid Leukaemia	Blood	1.79E-02	1.59E-01	3.16E-02	3.02E-01
534	GSM311606	GSE12417	Acute Myeloid Leukaemia	Blood	5.25E-01	-6.35E-02	4.55E-02	2.85E-01
535	GSM311607	GSE12417	Acute Myeloid Leukaemia	Blood	6.13E-03	1.79E-01	2.05E-02	3.20E-01
536	GSM311608	GSE12417	Acute Myeloid Leukaemia	Blood	1.79E-02	1.59E-01	9.82E-02	2.47E-01
537	GSM311609	GSE12417	Acute Myeloid Leukaemia	Blood	5.46E-02	1.35E-01	1.80E-02	3.25E-01
538	GSM311610	GSE12417	Acute Myeloid Leukaemia	Blood	2.53E-03	1.93E-01	6.31E-03	3.65E-01
539	GSM311611	GSE12417	Acute Myeloid Leukaemia	Blood	8.56E-03	1.73E-01	8.56E-02	2.54E-01
540	GSM311612	GSE12417	Acute Myeloid Leukaemia	Blood	5.16E-03	1.82E-01	1.02E-01	2.45E-01
541	GSM311613	GSE12417	Acute Myeloid Leukaemia	Blood	6.86E-03	1.77E-01	2.55E-02	3.11E-01
542	GSM311614	GSE12417	Acute Myeloid Leukaemia	Blood	7.60E-02	1.27E-01	5.39E-02	2.77E-01
543	GSM311615	GSE12417	Acute Myeloid Leukaemia	Blood	1.60E-01	-1.07E-01	1.32E-01	2.31E-01
544	GSM311616	GSE12417	Acute Myeloid Leukaemia	Blood	1.44E-01	1.10E-01	1.21E-01	2.36E-01
545	GSM311617	GSE12417	Acute Myeloid Leukaemia	Blood	3.67E-02	1.44E-01	7.43E-02	2.62E-01
546	GSM311618	GSE12417	Acute Myeloid Leukaemia	Blood	1.16E-01	1.16E-01	1.02E-01	2.45E-01
547	GSM311619	GSE12417	Acute Myeloid Leukaemia	Blood	7.92E-02	-1.26E-01	3.16E-02	3.02E-01
548	GSM311620	GSE12417	Acute Myeloid Leukaemia	Blood	1.12E-01	1.17E-01	5.10E-02	2.80E-01
549	GSM311621	GSE12417	Acute Myeloid Leukaemia	Blood	1.53E-02	1.62E-01	7.43E-02	2.62E-01
550	GSM311622	GSE12417	Acute Myeloid Leukaemia	Blood	5.79E-03	1.80E-01	2.08E-03	4.03E-01
551	GSM311623	GSE12417	Acute Myeloid Leukaemia	Blood	1.04E-03	2.07E-01	2.25E-02	3.16E-01
552	GSM311624	GSE12417	Acute Myeloid Leukaemia	Blood	2.21E-01	9.72E-02	6.17E-02	2.71E-01
553	GSM311625	GSE12417	Acute Myeloid Leukaemia	Blood	6.86E-03	1.77E-01	6.43E-02	2.69E-01
554	GSM311626	GSE12417	Acute Myeloid Leukaemia	Blood	3.49E-04	2.23E-01	4.21E-04	4.52E-01
555	GSM311627	GSE12417	Acute Myeloid Leukaemia	Blood	1.12E-02	1.68E-01	2.55E-02	3.11E-01
556	GSM311628	GSE12417	Acute Myeloid Leukaemia	Blood	1.97E-02	1.57E-01	1.80E-01	2.12E-01
557	GSM311629	GSE12417	Acute Myeloid Leukaemia	Blood	1.61E-02	1.61E-01	2.55E-02	3.11E-01
558	GSM311630	GSE12417	Acute Myeloid Leukaemia	Blood	2.64E-02	1.51E-01	1.75E-01	2.14E-01
559	GSM311631	GSE12417	Acute Myeloid Leukaemia	Blood	1.44E-01	-1.10E-01	8.56E-02	2.54E-01
560	GSM311632	GSE12417	Acute Myeloid Leukaemia	Blood	1.01E-02	1.70E-01	3.72E-02	2.94E-01
561	GSM311633	GSE12417	Acute Myeloid Leukaemia	Blood	5.23E-02	-1.36E-01	3.55E-01	1.65E-01
562	GSM311634	GSE12417	Acute Myeloid Leukaemia	Blood	4.79E-02	1.38E-01	1.43E-02	3.34E-01
563	GSM311635	GSE12417	Acute Myeloid Leukaemia	Blood	8.61E-07	2.96E-01	5.95E-07	6.14E-01
564	GSM311636	GSE12417	Acute Myeloid Leukaemia	Blood	4.34E-03	1.85E-01	2.55E-02	3.11E-01
565	GSM311637	GSE12417	Acute Myeloid Leukaemia	Blood	1.25E-02	1.66E-01	1.43E-02	3.34E-01
566	GSM311638	GSE12417	Acute Myeloid Leukaemia	Blood	1.30E-01	-1.13E-01	1.37E-01	2.29E-01
567	GSM311639	GSE12417	Acute Myeloid Leukaemia	Blood	3.50E-02	1.45E-01	2.05E-02	3.20E-01
568	GSM311640	GSE12417	Acute Myeloid Leukaemia	Blood	6.19E-02	1.32E-01	8.56E-02	2.54E-01
569	GSM311641	GSE12417	Acute Myeloid Leukaemia	Blood	5.69E-02	-1.34E-01	5.31E-02	2.78E-01
570	GSM311642	GSE12417	Acute Myeloid Leukaemia	Blood	1.63E-03	2.00E-01	9.34E-03	3.51E-01
571	GSM311643	GSE12417	Acute Myeloid Leukaemia	Blood	9.26E-02	1.22E-01	1.80E-01	2.12E-01
572	GSM311644	GSE12417	Acute Myeloid Leukaemia	Blood	7.25E-03	1.76E-01	8.56E-02	2.54E-01
573	GSM311645	GSE12417	Acute Myeloid Leukaemia	Blood	2.91E-02	1.49E-01	9.23E-02	2.50E-01
574	GSM311646	GSE12417	Acute Myeloid Leukaemia	Blood	5.01E-02	1.37E-01	1.02E-01	2.45E-01
575	GSM311647	GSE12417	Acute Myeloid Leukaemia	Blood	3.63E-03	1.88E-01	2.05E-02	3.20E-01
576	GSM311648	GSE12417	Acute Myeloid Leukaemia	Blood	8.91E-02	-1.23E-01	1.71E-01	-2.16E-01
577	GSM311649	GSE12417	Acute Myeloid Leukaemia	Blood	4.59E-02	1.39E-01	1.55E-01	2.22E-01
578	GSM311650	GSE12417	Acute Myeloid Leukaemia	Blood	2.57E-01	9.23E-02	1.16E-01	2.38E-01
579	GSM311651	GSE12417	Acute Myeloid Leukaemia	Blood	3.21E-01	-8.43E-02	3.97E-01	1.56E-01
580	GSM311652	GSE12417	Acute Myeloid Leukaemia	Blood	1.61E-02	1.61E-01	6.43E-02	2.69E-01
581	GSM311653	GSE12417	Acute Myeloid Leukaemia	Blood	1.18E-02	1.67E-01	7.73E-02	2.60E-01
582	GSM311654	GSE12417	Acute Myeloid Leukaemia	Blood	6.46E-02	-1.31E-01	5.31E-02	2.78E-01
583	GSM311655	GSE12417	Acute Myeloid Leukaemia	Blood	4.79E-02	1.38E-01	1.75E-01	2.14E-01
584	GSM311656	GSE12417	Acute Myeloid Leukaemia	Blood	2.91E-02	1.49E-01	3.16E-02	3.02E-01
585	GSM311657	GSE12417	Acute Myeloid Leukaemia	Blood	1.34E-01	1.12E-01	1.16E-01	2.38E-01
586	GSM311658	GSE12417	Acute Myeloid Leukaemia	Blood	1.08E-01	1.18E-01	6.17E-02	2.71E-01
587	GSM311659	GSE12417	Acute Myeloid Leukaemia	Blood	1.54E-01	1.08E-01	7.43E-02	2.62E-01

588	GSM311660	GSE12417	Acute Myeloid Leukaemia	Blood	2.07E-02	1.56E-01	5.54E-02	2.76E-01
589	GSM311661	GSE12417	Acute Myeloid Leukaemia	Blood	7.60E-02	1.27E-01	5.54E-02	2.76E-01
590	GSM311662	GSE12417	Acute Myeloid Leukaemia	Blood	1.97E-02	1.57E-01	6.17E-02	2.71E-01
591	GSM311663	GSE12417	Acute Myeloid Leukaemia	Blood	1.44E-01	-1.10E-01	8.89E-02	2.52E-01
592	GSM311664	GSE12417	Acute Myeloid Leukaemia	Blood	1.53E-02	1.62E-01	4.55E-02	2.85E-01
593	GSM311665	GSE12417	Acute Myeloid Leukaemia	Blood	1.00E-01	1.20E-01	3.30E-02	3.00E-01
594	GSM311666	GSE12417	Acute Myeloid Leukaemia	Blood	1.12E-01	1.17E-01	1.60E-01	2.20E-01
595	GSM311667	GSE12417	Acute Myeloid Leukaemia	Blood	4.20E-02	1.41E-01	6.43E-02	2.69E-01
596	GSM311668	GSE12417	Acute Myeloid Leukaemia	Blood	1.16E-01	1.16E-01	1.80E-01	2.12E-01
597	GSM311669	GSE12417	Acute Myeloid Leukaemia	Blood	1.71E-01	-1.05E-01	5.54E-02	2.76E-01
598	GSM311670	GSE12417	Acute Myeloid Leukaemia	Blood	1.46E-02	1.63E-01	7.43E-02	2.62E-01
599	GSM311671	GSE12417	Acute Myeloid Leukaemia	Blood	3.84E-02	1.43E-01	5.77E-02	2.74E-01
600	GSM311672	GSE12417	Acute Myeloid Leukaemia	Blood	2.28E-01	-9.62E-02	3.16E-02	3.02E-01
601	GSM311673	GSE12417	Acute Myeloid Leukaemia	Blood	5.46E-02	-1.35E-01	5.04E-01	1.34E-01
602	GSM311674	GSE12417	Acute Myeloid Leukaemia	Blood	2.42E-01	9.42E-02	3.55E-01	1.65E-01
603	GSM311675	GSE12417	Acute Myeloid Leukaemia	Blood	2.07E-02	1.56E-01	4.55E-02	2.85E-01
604	GSM311676	GSE12417	Acute Myeloid Leukaemia	Blood	4.34E-03	1.85E-01	3.03E-03	3.91E-01
605	GSM311677	GSE12417	Acute Myeloid Leukaemia	Blood	4.03E-01	7.54E-02	2.26E-01	1.98E-01
606	GSM311678	GSE12417	Acute Myeloid Leukaemia	Blood	1.44E-01	-1.10E-01	1.55E-01	2.22E-01
607	GSM311679	GSE12417	Acute Myeloid Leukaemia	Blood	2.64E-02	1.51E-01	2.26E-01	1.98E-01
608	GSM311680	GSE12417	Acute Myeloid Leukaemia	Blood	5.23E-02	-1.36E-01	1.55E-01	2.22E-01
609	GSM311681	GSE12417	Acute Myeloid Leukaemia	Blood	5.46E-02	1.35E-01	1.96E-01	2.07E-01
610	GSM311682	GSE12417	Acute Myeloid Leukaemia	Blood	6.19E-02	-1.32E-01	1.80E-01	2.12E-01
611	GSM311683	GSE12417	Acute Myeloid Leukaemia	Blood	8.10E-03	1.74E-01	6.17E-02	2.71E-01
612	GSM311684	GSE12417	Acute Myeloid Leukaemia	Blood	7.60E-02	1.27E-01	8.89E-02	2.52E-01
613	GSM311685	GSE12417	Acute Myeloid Leukaemia	Blood	1.31E-02	-1.65E-01	1.25E-01	-2.34E-01
614	GSM311686	GSE12417	Acute Myeloid Leukaemia	Blood	2.72E-01	-9.03E-02	1.80E-01	2.12E-01
615	GSM311687	GSE12417	Acute Myeloid Leukaemia	Blood	7.60E-02	1.27E-01	2.55E-02	3.11E-01
616	GSM311688	GSE12417	Acute Myeloid Leukaemia	Blood	6.48E-03	1.78E-01	3.89E-02	2.92E-01
617	GSM311689	GSE12417	Acute Myeloid Leukaemia	Blood	2.07E-02	1.56E-01	2.26E-01	1.98E-01
618	GSM311690	GSE12417	Acute Myeloid Leukaemia	Blood	5.39E-05	2.48E-01	5.98E-03	3.67E-01
619	GSM311691	GSE12417	Acute Myeloid Leukaemia	Blood	1.12E-01	-1.17E-01	1.16E-01	2.38E-01
620	GSM311692	GSE12417	Acute Myeloid Leukaemia	Blood	7.60E-02	1.27E-01	3.55E-01	1.65E-01
621	GSM311693	GSE12417	Acute Myeloid Leukaemia	Blood	1.39E-01	1.11E-01	8.56E-02	2.54E-01
622	GSM311694	GSE12417	Acute Myeloid Leukaemia	Blood	1.95E-01	-1.01E-01	7.43E-02	2.62E-01
623	GSM311695	GSE12417	Acute Myeloid Leukaemia	Blood	1.26E-03	2.04E-01	6.31E-03	3.65E-01
624	GSM311696	GSE12417	Acute Myeloid Leukaemia	Blood	1.60E-01	-1.07E-01	1.16E-01	2.38E-01
625	GSM311697	GSE12417	Acute Myeloid Leukaemia	Blood	3.86E-03	1.87E-01	5.45E-03	3.70E-01
626	GSM311698	GSE12417	Acute Myeloid Leukaemia	Blood	2.18E-02	1.55E-01	1.50E-02	3.32E-01
627	GSM311699	GSE12417	Acute Myeloid Leukaemia	Blood	4.39E-02	1.40E-01	1.80E-02	3.25E-01
628	GSM311700	GSE12417	Acute Myeloid Leukaemia	Blood	1.65E-01	1.06E-01	7.43E-02	2.62E-01
629	GSM311701	GSE12417	Acute Myeloid Leukaemia	Blood	7.30E-02	-1.28E-01	7.43E-02	2.62E-01
630	GSM311702	GSE12417	Acute Myeloid Leukaemia	Blood	1.25E-01	-1.14E-01	5.31E-02	2.78E-01
631	GSM311703	GSE12417	Acute Myeloid Leukaemia	Blood	1.04E-01	-1.19E-01	4.55E-02	2.85E-01
632	GSM311704	GSE12417	Acute Myeloid Leukaemia	Blood	4.09E-03	1.86E-01	6.17E-02	2.71E-01
633	GSM311705	GSE12417	Acute Myeloid Leukaemia	Blood	1.34E-01	-1.12E-01	8.56E-02	2.54E-01
634	GSM311706	GSE12417	Acute Myeloid Leukaemia	Blood	1.65E-01	1.06E-01	1.02E-01	2.45E-01
635	GSM311707	GSE12417	Acute Myeloid Leukaemia	Blood	5.46E-02	1.35E-01	1.16E-01	2.38E-01
636	GSM311708	GSE12417	Acute Myeloid Leukaemia	Blood	4.20E-02	1.41E-01	1.02E-01	2.45E-01
637	GSM311709	GSE12417	Acute Myeloid Leukaemia	Blood	1.54E-01	1.08E-01	2.08E-02	3.19E-01
638	GSM311710	GSE12417	Acute Myeloid Leukaemia	Blood	7.30E-02	1.28E-01	1.37E-01	2.29E-01
639	GSM311711	GSE12417	Acute Myeloid Leukaemia	Blood	6.73E-02	-1.30E-01	1.46E-01	2.25E-01
640	GSM311712	GSE12417	Acute Myeloid Leukaemia	Blood	6.73E-02	1.30E-01	7.73E-02	2.60E-01
641	GSM311713	GSE12417	Acute Myeloid Leukaemia	Blood	3.19E-02	1.47E-01	3.72E-02	2.94E-01
642	GSM311714	GSE12417	Acute Myeloid Leukaemia	Blood	4.59E-02	-1.39E-01	5.31E-02	2.78E-01
643	GSM311715	GSE12417	Acute Myeloid Leukaemia	Blood	1.38E-02	1.64E-01	5.31E-02	2.78E-01
644	GSM311716	GSE12417	Acute Myeloid Leukaemia	Blood	4.60E-03	1.84E-01	2.55E-02	3.11E-01
645	GSM311717	GSE12417	Acute Myeloid Leukaemia	Blood	1.79E-02	-1.59E-01	2.81E-01	-1.83E-01
646	GSM311718	GSE12417	Acute Myeloid Leukaemia	Blood	1.44E-01	1.10E-01	2.51E-01	1.91E-01
647	GSM311719	GSE12417	Acute Myeloid Leukaemia	Blood	2.91E-02	1.49E-01	1.75E-01	2.14E-01
648	GSM311720	GSE12417	Acute Myeloid Leukaemia	Blood	2.77E-02	1.50E-01	6.31E-03	3.65E-01
649	GSM311721	GSE12417	Acute Myeloid Leukaemia	Blood	5.69E-02	1.34E-01	4.55E-02	2.85E-01
650	GSM311722	GSE12417	Acute Myeloid Leukaemia	Blood	9.54E-03	1.71E-01	3.89E-02	2.92E-01
651	GSM311723	GSE12417	Acute Myeloid Leukaemia	Blood	1.88E-02	1.58E-01	1.96E-01	2.07E-01
652	GSM311724	GSE12417	Acute Myeloid Leukaemia	Blood	2.40E-02	1.53E-01	1.80E-02	3.25E-01
653	GSM311725	GSE12417	Acute Myeloid Leukaemia	Blood	2.42E-01	-9.42E-02	1.80E-01	2.12E-01
654	GSM311726	GSE12417	Acute Myeloid Leukaemia	Blood	4.39E-02	1.40E-01	3.97E-03	3.82E-01
655	GSM311727	GSE12417	Acute Myeloid Leukaemia	Blood	3.42E-03	1.88E-01	5.10E-02	2.80E-01
656	GSM311728	GSE12417	Acute Myeloid Leukaemia	Blood	4.01E-02	1.42E-01	1.25E-02	3.40E-01
657	GSM311729	GSE12417	Acute Myeloid Leukaemia	Blood	2.01E-01	1.00E-01	2.86E-01	1.82E-01
658	GSM311730	GSE12417	Acute Myeloid Leukaemia	Blood	2.28E-01	9.62E-02	9.85E-03	3.49E-01
659	GSM311731	GSE12417	Acute Myeloid Leukaemia	Blood	1.65E-01	-1.06E-01	3.23E-01	1.72E-01
660	GSM311732	GSE12417	Acute Myeloid Leukaemia	Blood	8.24E-02	1.25E-01	8.56E-02	2.54E-01
661	GSM311733	GSE12417	Acute Myeloid Leukaemia	Blood	6.73E-02	1.30E-01	2.08E-01	2.03E-01

662	GSM311734	GSE12417	Acute Myeloid Leukaemia	Blood	4.79E-02	1.38E-01	1.66E-02	3.29E-01
663	GSM311735	GSE12417	Acute Myeloid Leukaemia	Blood	1.21E-01	1.15E-01	5.31E-02	2.78E-01
664	GSM311736	GSE12417	Acute Myeloid Leukaemia	Blood	6.13E-03	1.79E-01	9.82E-02	2.47E-01
665	GSM311737	GSE12417	Acute Myeloid Leukaemia	Blood	2.64E-02	1.51E-01	3.21E-03	3.89E-01
666	GSM311738	GSE12417	Acute Myeloid Leukaemia	Blood	1.46E-02	1.63E-01	1.21E-01	2.36E-01
667	GSM311739	GSE12417	Acute Myeloid Leukaemia	Blood	8.24E-02	-1.25E-01	5.31E-02	2.78E-01
668	GSM311740	GSE12417	Acute Myeloid Leukaemia	Blood	1.65E-01	1.06E-01	2.26E-01	1.98E-01
669	GSM311741	GSE12417	Acute Myeloid Leukaemia	Blood	5.46E-02	1.35E-01	2.08E-02	3.19E-01
670	GSM311742	GSE12417	Acute Myeloid Leukaemia	Blood	1.77E-01	1.04E-01	3.72E-02	2.94E-01
671	GSM311743	GSE12417	Acute Myeloid Leukaemia	Blood	1.97E-02	1.57E-01	6.17E-02	2.71E-01
672	GSM311744	GSE12417	Acute Myeloid Leukaemia	Blood	3.05E-02	1.48E-01	2.05E-02	3.20E-01
673	GSM311745	GSE12417	Acute Myeloid Leukaemia	Blood	2.40E-02	-1.53E-01	5.31E-02	2.78E-01
674	GSM311746	GSE12417	Acute Myeloid Leukaemia	Blood	1.21E-01	-1.15E-01	1.02E-01	2.45E-01
675	GSM311747	GSE12417	Acute Myeloid Leukaemia	Blood	2.91E-02	1.49E-01	1.55E-01	2.22E-01
676	GSM311748	GSE12417	Acute Myeloid Leukaemia	Blood	1.04E-03	2.07E-01	4.55E-02	2.85E-01
677	GSM311749	GSE12417	Acute Myeloid Leukaemia	Blood	3.94E-01	7.64E-02	2.21E-01	-1.99E-01
678	GSM311750	GSE12417	Acute Myeloid Leukaemia	Blood	4.20E-02	-1.41E-01	2.58E-01	1.89E-01
679	GSM311751	GSE12417	Acute Myeloid Leukaemia	Blood	4.87E-03	1.83E-01	8.56E-02	2.54E-01
680	GSM311752	GSE12417	Acute Myeloid Leukaemia	Blood	4.79E-02	1.38E-01	2.19E-01	2.00E-01
681	GSM311753	GSE12417	Acute Myeloid Leukaemia	Blood	4.20E-02	1.41E-01	6.17E-02	2.71E-01
682	GSM311754	GSE12417	Acute Myeloid Leukaemia	Blood	5.94E-02	1.33E-01	3.89E-02	2.92E-01
683	GSM311755	GSE12417	Acute Myeloid Leukaemia	Blood	4.01E-02	1.42E-01	6.17E-02	2.71E-01
684	GSM311756	GSE12417	Acute Myeloid Leukaemia	Blood	4.39E-02	1.40E-01	8.56E-02	2.54E-01
685	GSM311757	GSE12417	Acute Myeloid Leukaemia	Blood	4.79E-02	1.38E-01	8.89E-02	2.52E-01
686	GSM311758	GSE12417	Acute Myeloid Leukaemia	Blood	1.65E-01	1.06E-01	1.89E-02	3.23E-01
687	GSM311759	GSE12417	Acute Myeloid Leukaemia	Blood	1.04E-01	1.19E-01	2.58E-01	1.89E-01
688	GSM311760	GSE12417	Acute Myeloid Leukaemia	Blood	1.01E-02	1.70E-01	1.47E-03	4.14E-01
689	GSM316652	GSE12417	Acute Myeloid Leukaemia	Blood	1.97E-03	1.97E-01	1.50E-02	3.32E-01
690	GSM316653	GSE12417	Acute Myeloid Leukaemia	Blood	5.94E-02	1.33E-01	8.56E-02	2.54E-01
691	GSM316654	GSE12417	Acute Myeloid Leukaemia	Blood	3.84E-02	-1.43E-01	3.97E-01	1.56E-01
692	GSM316655	GSE12417	Acute Myeloid Leukaemia	Blood	7.60E-02	1.27E-01	8.56E-02	2.54E-01
693	GSM316656	GSE12417	Acute Myeloid Leukaemia	Blood	1.44E-01	1.10E-01	2.02E-01	2.05E-01
694	GSM316657	GSE12417	Acute Myeloid Leukaemia	Blood	2.64E-02	-1.51E-01	5.31E-02	2.78E-01
695	GSM316658	GSE12417	Acute Myeloid Leukaemia	Blood	3.67E-02	1.44E-01	2.02E-01	2.05E-01
696	GSM316659	GSE12417	Acute Myeloid Leukaemia	Blood	7.01E-02	1.29E-01	1.32E-01	2.31E-01
697	GSM316660	GSE12417	Acute Myeloid Leukaemia	Blood	3.84E-02	1.43E-01	1.16E-01	2.38E-01
698	GSM316661	GSE12417	Acute Myeloid Leukaemia	Blood	2.29E-02	1.54E-01	2.44E-03	3.98E-01
699	GSM316662	GSE12417	Acute Myeloid Leukaemia	Blood	2.69E-03	1.92E-01	6.17E-02	2.71E-01
700	GSM316663	GSE12417	Acute Myeloid Leukaemia	Blood	2.77E-02	1.50E-01	7.73E-02	2.60E-01
701	GSM316664	GSE12417	Acute Myeloid Leukaemia	Blood	4.79E-02	1.38E-01	7.43E-02	2.62E-01
702	GSM316665	GSE12417	Acute Myeloid Leukaemia	Blood	2.24E-03	-1.95E-01	1.55E-01	2.22E-01
703	GSM316666	GSE12417	Acute Myeloid Leukaemia	Blood	6.86E-03	1.77E-01	6.17E-02	2.71E-01
704	GSM316667	GSE12417	Acute Myeloid Leukaemia	Blood	6.73E-02	1.30E-01	2.26E-01	1.98E-01
705	GSM316668	GSE12417	Acute Myeloid Leukaemia	Blood	1.82E-04	2.32E-01	1.43E-02	3.34E-01
706	GSM316669	GSE12417	Acute Myeloid Leukaemia	Blood	3.34E-02	1.46E-01	9.82E-02	2.47E-01
707	GSM316670	GSE12417	Acute Myeloid Leukaemia	Blood	4.39E-02	1.40E-01	8.35E-02	2.56E-01
708	GSM316671	GSE12417	Acute Myeloid Leukaemia	Blood	1.01E-02	1.70E-01	1.89E-02	3.23E-01
709	GSM316672	GSE12417	Acute Myeloid Leukaemia	Blood	7.92E-02	-1.26E-01	4.55E-02	2.85E-01
710	GSM316673	GSE12417	Acute Myeloid Leukaemia	Blood	8.24E-02	1.25E-01	5.31E-02	2.78E-01
711	GSM316674	GSE12417	Acute Myeloid Leukaemia	Blood	1.97E-03	1.97E-01	5.10E-02	2.80E-01
712	GSM316675	GSE12417	Acute Myeloid Leukaemia	Blood	1.79E-02	1.59E-01	9.82E-02	2.47E-01
713	GSM316676	GSE12417	Acute Myeloid Leukaemia	Blood	7.67E-03	1.75E-01	1.67E-03	4.10E-01
714	GSM316677	GSE12417	Acute Myeloid Leukaemia	Blood	1.49E-01	-1.09E-01	6.69E-02	2.67E-01
715	GSM316678	GSE12417	Acute Myeloid Leukaemia	Blood	5.46E-02	1.35E-01	1.43E-02	3.34E-01
716	GSM316679	GSE12417	Acute Myeloid Leukaemia	Blood	8.24E-02	-1.25E-01	8.56E-02	2.54E-01
717	GSM316680	GSE12417	Acute Myeloid Leukaemia	Blood	1.54E-01	1.08E-01	2.44E-03	3.98E-01
718	GSM316681	GSE12417	Acute Myeloid Leukaemia	Blood	2.38E-03	1.94E-01	1.80E-02	3.25E-01
719	GSM316682	GSE12417	Acute Myeloid Leukaemia	Blood	1.83E-01	1.03E-01	7.70E-03	3.58E-01
720	GSM316683	GSE12417	Acute Myeloid Leukaemia	Blood	1.25E-01	-1.14E-01	3.16E-02	3.02E-01
721	GSM316684	GSE12417	Acute Myeloid Leukaemia	Blood	1.00E-01	-1.20E-01	1.75E-01	2.14E-01
722	GSM316685	GSE12417	Acute Myeloid Leukaemia	Blood	1.12E-02	1.68E-01	1.60E-01	2.20E-01
723	GSM316686	GSE12417	Acute Myeloid Leukaemia	Blood	1.46E-02	1.63E-01	1.80E-02	3.25E-01
724	GSM316687	GSE12417	Acute Myeloid Leukaemia	Blood	6.46E-02	1.31E-01	1.12E-01	2.40E-01
725	GSM316688	GSE12417	Acute Myeloid Leukaemia	Blood	6.73E-02	1.30E-01	6.69E-02	2.67E-01
726	GSM316689	GSE12417	Acute Myeloid Leukaemia	Blood	6.19E-02	-1.32E-01	4.55E-02	2.85E-01
727	GSM316690	GSE12417	Acute Myeloid Leukaemia	Blood	4.60E-03	1.84E-01	9.85E-03	3.49E-01
728	GSM316691	GSE12417	Acute Myeloid Leukaemia	Blood	4.01E-02	1.42E-01	4.55E-02	2.85E-01
729	GSM316692	GSE12417	Acute Myeloid Leukaemia	Blood	2.07E-02	1.56E-01	1.75E-01	2.14E-01
730	GSM316693	GSE12417	Acute Myeloid Leukaemia	Blood	2.21E-01	9.72E-02	2.67E-02	3.09E-01
731	GSM316694	GSE12417	Acute Myeloid Leukaemia	Blood	2.24E-03	1.95E-01	1.25E-03	4.20E-01
732	GSM316695	GSE12417	Acute Myeloid Leukaemia	Blood	5.69E-02	1.34E-01	1.80E-01	2.12E-01
733	GSM316696	GSE12417	Acute Myeloid Leukaemia	Blood	7.30E-02	1.28E-01	1.02E-01	2.45E-01
734	GSM316697	GSE12417	Acute Myeloid Leukaemia	Blood	1.53E-02	1.62E-01	1.37E-01	2.29E-01
735	GSM316698	GSE12417	Acute Myeloid Leukaemia	Blood	7.60E-02	1.27E-01	2.67E-02	3.09E-01

736	GSM316699	GSE12417	Acute Myeloid Leukaemia	Blood	1.18E-03	2.05E-01	3.97E-03	3.82E-01
737	GSM316700	GSE12417	Acute Myeloid Leukaemia	Blood	2.40E-02	1.53E-01	7.43E-02	2.62E-01
738	GSM316701	GSE12417	Acute Myeloid Leukaemia	Blood	1.77E-01	-1.04E-01	6.17E-02	2.71E-01
739	GSM316702	GSE12417	Acute Myeloid Leukaemia	Blood	2.77E-02	1.50E-01	3.30E-02	3.00E-01
740	GSM316703	GSE12417	Acute Myeloid Leukaemia	Blood	1.97E-02	1.57E-01	6.43E-02	2.69E-01
741	GSM316704	GSE12417	Acute Myeloid Leukaemia	Blood	3.49E-04	2.23E-01	3.40E-03	3.87E-01
742	GSM316705	GSE12417	Acute Myeloid Leukaemia	Blood	4.79E-02	1.38E-01	9.82E-02	2.47E-01
743	GSM316706	GSE12417	Acute Myeloid Leukaemia	Blood	2.08E-01	-9.92E-02	1.75E-01	2.14E-01
744	GSM316707	GSE12417	Acute Myeloid Leukaemia	Blood	7.01E-02	1.29E-01	2.02E-01	2.05E-01
745	GSM316708	GSE12417	Acute Myeloid Leukaemia	Blood	9.26E-02	1.22E-01	3.89E-02	2.92E-01
746	GSM316709	GSE12417	Acute Myeloid Leukaemia	Blood	9.63E-02	1.21E-01	1.21E-01	2.36E-01
747	GSM316710	GSE12417	Acute Myeloid Leukaemia	Blood	3.13E-01	8.53E-02	4.43E-02	2.87E-01
748	GSM316711	GSE12417	Acute Myeloid Leukaemia	Blood	4.79E-02	1.38E-01	2.32E-01	1.96E-01
749	GSM316712	GSE12417	Acute Myeloid Leukaemia	Blood	7.92E-02	1.26E-01	8.56E-02	2.54E-01
750	GSM316713	GSE12417	Acute Myeloid Leukaemia	Blood	3.19E-02	1.47E-01	2.55E-02	3.11E-01
751	GSM316714	GSE12417	Acute Myeloid Leukaemia	Blood	4.20E-02	1.41E-01	6.17E-02	2.71E-01
752	GSM316715	GSE12417	Acute Myeloid Leukaemia	Blood	3.34E-02	1.46E-01	3.72E-02	2.94E-01
753	GSM316716	GSE12417	Acute Myeloid Leukaemia	Blood	2.10E-03	1.96E-01	7.04E-03	3.61E-01
754	GSM316717	GSE12417	Acute Myeloid Leukaemia	Blood	3.67E-02	1.44E-01	1.32E-01	2.31E-01
755	GSM316718	GSE12417	Acute Myeloid Leukaemia	Blood	5.68E-04	2.16E-01	4.44E-03	3.78E-01
756	GSM316719	GSE12417	Acute Myeloid Leukaemia	Blood	2.40E-02	1.53E-01	1.02E-01	2.45E-01
757	GSM316720	GSE12417	Acute Myeloid Leukaemia	Blood	4.59E-02	1.39E-01	8.56E-02	2.54E-01
758	GSM316721	GSE12417	Acute Myeloid Leukaemia	Blood	1.25E-02	1.66E-01	8.89E-02	2.52E-01
759	GSM316722	GSE12417	Acute Myeloid Leukaemia	Blood	8.24E-02	-1.25E-01	1.55E-01	2.22E-01
760	GSM316723	GSE12417	Acute Myeloid Leukaemia	Blood	7.01E-02	1.29E-01	2.08E-01	2.03E-01
761	GSM316724	GSE12417	Acute Myeloid Leukaemia	Blood	1.49E-01	-1.09E-01	1.65E-01	-2.18E-01
762	GSM316725	GSE12417	Acute Myeloid Leukaemia	Blood	3.05E-02	-1.48E-01	3.46E-01	1.67E-01
763	GSM316726	GSE12417	Acute Myeloid Leukaemia	Blood	4.01E-02	1.42E-01	1.37E-01	2.29E-01
764	GSM316727	GSE12417	Acute Myeloid Leukaemia	Blood	6.51E-04	2.14E-01	2.44E-03	3.98E-01
765	GSM316728	GSE12417	Acute Myeloid Leukaemia	Blood	2.77E-02	1.50E-01	5.76E-03	3.69E-01
766	GSM316729	GSE12417	Acute Myeloid Leukaemia	Blood	6.46E-02	1.31E-01	1.50E-02	3.32E-01
767	GSM316730	GSE12417	Acute Myeloid Leukaemia	Blood	1.18E-03	2.05E-01	3.21E-03	3.89E-01
768	GSM262030	GSE10358	Acute Myeloid Leukaemia	Blood	1.38E-02	1.64E-01	4.55E-02	2.85E-01
769	GSM262031	GSE10358	Acute Myeloid Leukaemia	Blood	1.95E-01	1.01E-01	2.04E-01	-2.05E-01
770	GSM262032	GSE10358	Acute Myeloid Leukaemia	Blood	1.26E-03	2.04E-01	6.86E-04	4.38E-01
771	GSM262033	GSE10358	Acute Myeloid Leukaemia	Blood	9.63E-02	1.21E-01	2.58E-01	1.89E-01
772	GSM262034	GSE10358	Acute Myeloid Leukaemia	Blood	1.31E-02	1.65E-01	4.55E-02	2.85E-01
773	GSM262035	GSE10358	Acute Myeloid Leukaemia	Blood	3.84E-02	1.43E-01	3.72E-02	2.94E-01
774	GSM262036	GSE10358	Acute Myeloid Leukaemia	Blood	8.91E-02	-1.23E-01	2.32E-01	1.96E-01
775	GSM262037	GSE10358	Acute Myeloid Leukaemia	Blood	8.10E-03	1.74E-01	2.55E-02	3.11E-01
776	GSM262038	GSE10358	Acute Myeloid Leukaemia	Blood	1.31E-02	1.65E-01	6.17E-02	2.71E-01
777	GSM262039	GSE10358	Acute Myeloid Leukaemia	Blood	5.46E-02	1.35E-01	3.72E-02	2.94E-01
778	GSM262040	GSE10358	Acute Myeloid Leukaemia	Blood	8.24E-02	1.25E-01	2.26E-01	1.98E-01
779	GSM262041	GSE10358	Acute Myeloid Leukaemia	Blood	1.04E-01	1.19E-01	7.43E-02	2.62E-01
780	GSM262042	GSE10358	Acute Myeloid Leukaemia	Blood	5.30E-04	2.17E-01	1.80E-02	3.25E-01
781	GSM262043	GSE10358	Acute Myeloid Leukaemia	Blood	1.53E-03	2.01E-01	1.19E-02	3.42E-01
782	GSM262044	GSE10358	Acute Myeloid Leukaemia	Blood	1.44E-01	1.10E-01	2.25E-02	3.16E-01
783	GSM262045	GSE10358	Acute Myeloid Leukaemia	Blood	4.59E-02	1.39E-01	7.43E-02	2.62E-01
784	GSM262046	GSE10358	Acute Myeloid Leukaemia	Blood	1.16E-01	1.16E-01	2.86E-01	1.82E-01
785	GSM262047	GSE10358	Acute Myeloid Leukaemia	Blood	2.77E-02	-1.50E-01	1.32E-01	2.31E-01
786	GSM262048	GSE10358	Acute Myeloid Leukaemia	Blood	3.04E-03	1.90E-01	1.43E-02	3.34E-01
787	GSM262049	GSE10358	Acute Myeloid Leukaemia	Blood	1.70E-02	1.60E-01	1.43E-02	3.34E-01
788	GSM262050	GSE10358	Acute Myeloid Leukaemia	Blood	1.34E-01	-1.12E-01	1.65E-01	-2.18E-01
789	GSM262051	GSE10358	Acute Myeloid Leukaemia	Blood	1.60E-01	1.07E-01	7.43E-02	2.62E-01
790	GSM262052	GSE10358	Acute Myeloid Leukaemia	Blood	4.33E-01	7.24E-02	2.66E-01	1.87E-01
791	GSM262053	GSE10358	Acute Myeloid Leukaemia	Blood	7.30E-02	1.28E-01	2.32E-01	1.96E-01
792	GSM262054	GSE10358	Acute Myeloid Leukaemia	Blood	1.53E-02	1.62E-01	3.16E-02	3.02E-01
793	GSM262055	GSE10358	Acute Myeloid Leukaemia	Blood	8.24E-02	1.25E-01	1.16E-01	2.38E-01
794	GSM262056	GSE10358	Acute Myeloid Leukaemia	Blood	7.01E-02	-1.29E-01	9.82E-02	2.47E-01
795	GSM262057	GSE10358	Acute Myeloid Leukaemia	Blood	4.60E-03	1.84E-01	1.80E-02	3.25E-01
796	GSM262058	GSE10358	Acute Myeloid Leukaemia	Blood	2.07E-02	1.56E-01	8.56E-02	2.54E-01
797	GSM262059	GSE10358	Acute Myeloid Leukaemia	Blood	6.19E-02	-1.32E-01	1.80E-01	2.12E-01
798	GSM262060	GSE10358	Acute Myeloid Leukaemia	Blood	2.35E-01	9.52E-02	2.19E-01	2.00E-01
799	GSM262061	GSE10358	Acute Myeloid Leukaemia	Blood	4.20E-02	-1.41E-01	1.55E-01	2.22E-01
800	GSM262062	GSE10358	Acute Myeloid Leukaemia	Blood	2.40E-02	-1.53E-01	2.26E-01	1.98E-01
801	GSM262063	GSE10358	Acute Myeloid Leukaemia	Blood	1.16E-01	-1.16E-01	3.16E-02	3.02E-01
802	GSM262064	GSE10358	Acute Myeloid Leukaemia	Blood	1.89E-01	-1.02E-01	5.54E-02	2.76E-01
803	GSM262065	GSE10358	Acute Myeloid Leukaemia	Blood	4.34E-03	1.85E-01	1.80E-02	3.25E-01
804	GSM262066	GSE10358	Acute Myeloid Leukaemia	Blood	5.01E-02	1.37E-01	3.30E-02	3.00E-01
805	GSM262067	GSE10358	Acute Myeloid Leukaemia	Blood	1.12E-01	1.17E-01	1.89E-02	3.23E-01
806	GSM262068	GSE10358	Acute Myeloid Leukaemia	Blood	3.21E-01	8.43E-02	4.20E-03	3.80E-01
807	GSM262069	GSE10358	Acute Myeloid Leukaemia	Blood	6.51E-04	2.14E-01	1.80E-02	3.25E-01
808	GSM262070	GSE10358	Acute Myeloid Leukaemia	Blood	1.97E-02	1.57E-01	1.43E-02	3.34E-01
809	GSM262071	GSE10358	Acute Myeloid Leukaemia	Blood	7.60E-02	1.27E-01	6.43E-02	2.69E-01

810	GSM262072	GSE10358	Acute Myeloid Leukaemia	Blood	3.56E-01	-8.04E-02	7.83E-02	-2.59E-01
811	GSM262073	GSE10358	Acute Myeloid Leukaemia	Blood	1.01E-02	1.70E-01	9.85E-03	3.49E-01
812	GSM262074	GSE10358	Acute Myeloid Leukaemia	Blood	4.09E-03	1.86E-01	6.86E-04	4.38E-01
813	GSM262075	GSE10358	Acute Myeloid Leukaemia	Blood	1.77E-01	1.04E-01	2.02E-01	2.05E-01
814	GSM262076	GSE10358	Acute Myeloid Leukaemia	Blood	9.63E-02	1.21E-01	1.16E-01	2.38E-01
815	GSM262077	GSE10358	Acute Myeloid Leukaemia	Blood	1.46E-02	1.63E-01	1.29E-01	2.32E-01
816	GSM262078	GSE10358	Acute Myeloid Leukaemia	Blood	1.04E-01	1.19E-01	9.34E-03	3.51E-01
817	GSM262079	GSE10358	Acute Myeloid Leukaemia	Blood	8.57E-02	1.24E-01	2.19E-01	2.00E-01
818	GSM262080	GSE10358	Acute Myeloid Leukaemia	Blood	7.30E-02	-1.28E-01	2.39E-01	-1.94E-01
819	GSM262090	GSE10358	Acute Myeloid Leukaemia	Blood	1.00E-01	1.20E-01	2.26E-01	1.98E-01
820	GSM262091	GSE10358	Acute Myeloid Leukaemia	Blood	1.25E-02	1.66E-01	8.89E-02	2.52E-01
821	GSM262092	GSE10358	Acute Myeloid Leukaemia	Blood	8.10E-03	1.74E-01	9.27E-04	4.29E-01
822	GSM262093	GSE10358	Acute Myeloid Leukaemia	Blood	1.97E-03	1.97E-01	4.36E-02	2.87E-01
823	GSM262094	GSE10358	Acute Myeloid Leukaemia	Blood	1.60E-01	1.07E-01	1.55E-01	2.22E-01
824	GSM262095	GSE10358	Acute Myeloid Leukaemia	Blood	2.77E-02	1.50E-01	7.70E-03	3.58E-01
825	GSM262096	GSE10358	Acute Myeloid Leukaemia	Blood	2.01E-01	1.00E-01	2.26E-01	1.98E-01
826	GSM262097	GSE10358	Acute Myeloid Leukaemia	Blood	9.26E-02	-1.22E-01	3.16E-02	3.02E-01
827	GSM262098	GSE10358	Acute Myeloid Leukaemia	Blood	5.16E-03	1.82E-01	2.44E-03	3.98E-01
828	GSM262099	GSE10358	Acute Myeloid Leukaemia	Blood	6.86E-03	1.77E-01	2.67E-02	3.09E-01
829	GSM262100	GSE10358	Acute Myeloid Leukaemia	Blood	6.51E-04	2.14E-01	3.16E-02	3.02E-01
830	GSM262101	GSE10358	Acute Myeloid Leukaemia	Blood	9.26E-02	-1.22E-01	2.26E-01	1.98E-01
831	GSM262102	GSE10358	Acute Myeloid Leukaemia	Blood	1.70E-02	1.60E-01	2.15E-02	3.18E-01
832	GSM262103	GSE10358	Acute Myeloid Leukaemia	Blood	2.44E-04	2.28E-01	2.55E-02	3.11E-01
833	GSM262104	GSE10358	Acute Myeloid Leukaemia	Blood	7.25E-03	1.76E-01	2.15E-02	3.18E-01
834	GSM262105	GSE10358	Acute Myeloid Leukaemia	Blood	3.63E-03	1.88E-01	7.81E-04	4.34E-01
835	GSM262106	GSE10358	Acute Myeloid Leukaemia	Blood	3.05E-02	1.48E-01	2.15E-02	3.18E-01
836	GSM262107	GSE10358	Acute Myeloid Leukaemia	Blood	6.48E-03	1.78E-01	5.10E-02	2.80E-01
837	GSM262108	GSE10358	Acute Myeloid Leukaemia	Blood	1.31E-02	1.65E-01	1.42E-01	2.27E-01
838	GSM262109	GSE10358	Acute Myeloid Leukaemia	Blood	1.53E-02	1.62E-01	3.72E-02	2.94E-01
839	GSM262110	GSE10358	Acute Myeloid Leukaemia	Blood	3.34E-02	1.46E-01	2.67E-02	3.09E-01
840	GSM262111	GSE10358	Acute Myeloid Leukaemia	Blood	1.06E-02	1.69E-01	1.89E-02	3.23E-01
841	GSM262112	GSE10358	Acute Myeloid Leukaemia	Blood	1.04E-01	1.19E-01	4.55E-02	2.85E-01
842	GSM262113	GSE10358	Acute Myeloid Leukaemia	Blood	1.89E-01	1.02E-01	1.75E-01	2.14E-01
843	GSM262114	GSE10358	Acute Myeloid Leukaemia	Blood	5.23E-02	1.36E-01	1.37E-01	2.29E-01
844	GSM262115	GSE10358	Acute Myeloid Leukaemia	Blood	1.77E-01	1.04E-01	2.02E-01	2.05E-01
845	GSM262116	GSE10358	Acute Myeloid Leukaemia	Blood	1.88E-02	1.58E-01	5.31E-02	2.78E-01
846	GSM262117	GSE10358	Acute Myeloid Leukaemia	Blood	1.25E-01	1.14E-01	1.16E-01	2.38E-01
847	GSM262118	GSE10358	Acute Myeloid Leukaemia	Blood	1.60E-01	-1.07E-01	5.54E-02	2.76E-01
848	GSM262119	GSE10358	Acute Myeloid Leukaemia	Blood	4.62E-04	2.19E-01	9.34E-03	3.51E-01
849	GSM262120	GSE10358	Acute Myeloid Leukaemia	Blood	1.21E-01	1.15E-01	3.30E-02	3.00E-01
850	GSM262121	GSE10358	Acute Myeloid Leukaemia	Blood	4.79E-02	-1.38E-01	5.54E-02	2.76E-01
851	GSM262122	GSE10358	Acute Myeloid Leukaemia	Blood	1.04E-01	-1.19E-01	8.89E-02	2.52E-01
852	GSM262123	GSE10358	Acute Myeloid Leukaemia	Blood	1.16E-01	-1.16E-01	6.17E-02	2.71E-01
853	GSM262124	GSE10358	Acute Myeloid Leukaemia	Blood	9.04E-03	1.72E-01	1.72E-02	3.27E-01
854	GSM262125	GSE10358	Acute Myeloid Leukaemia	Blood	6.86E-03	1.77E-01	1.43E-02	3.34E-01
855	GSM262126	GSE10358	Acute Myeloid Leukaemia	Blood	4.87E-03	1.83E-01	2.67E-02	3.09E-01
856	GSM262127	GSE10358	Acute Myeloid Leukaemia	Blood	4.79E-02	1.38E-01	4.88E-03	3.74E-01
857	GSM262128	GSE10358	Acute Myeloid Leukaemia	Blood	9.63E-02	-1.21E-01	1.75E-01	2.14E-01
858	GSM262129	GSE10358	Acute Myeloid Leukaemia	Blood	5.01E-02	1.37E-01	6.17E-02	2.71E-01
859	GSM262130	GSE10358	Acute Myeloid Leukaemia	Blood	2.91E-02	1.49E-01	3.16E-02	3.02E-01
860	GSM262131	GSE10358	Acute Myeloid Leukaemia	Blood	8.10E-03	1.74E-01	1.80E-02	3.25E-01
861	GSM262132	GSE10358	Acute Myeloid Leukaemia	Blood	5.46E-02	1.35E-01	2.08E-03	4.03E-01
862	GSM262133	GSE10358	Acute Myeloid Leukaemia	Blood	6.51E-04	2.14E-01	4.55E-02	2.85E-01
863	GSM262134	GSE10358	Acute Myeloid Leukaemia	Blood	7.01E-02	-1.29E-01	1.55E-01	2.22E-01
864	GSM262135	GSE10358	Acute Myeloid Leukaemia	Blood	4.79E-02	1.38E-01	1.02E-01	2.45E-01
865	GSM262136	GSE10358	Acute Myeloid Leukaemia	Blood	7.01E-02	1.29E-01	4.55E-02	2.85E-01
866	GSM262137	GSE10358	Acute Myeloid Leukaemia	Blood	5.79E-03	1.80E-01	3.40E-03	3.87E-01
867	GSM262138	GSE10358	Acute Myeloid Leukaemia	Blood	3.34E-02	1.46E-01	4.55E-02	2.85E-01
868	GSM262139	GSE10358	Acute Myeloid Leukaemia	Blood	5.46E-02	-1.35E-01	6.43E-02	2.69E-01
869	GSM262140	GSE10358	Acute Myeloid Leukaemia	Blood	7.25E-03	1.76E-01	1.32E-01	2.31E-01
870	GSM262141	GSE10358	Acute Myeloid Leukaemia	Blood	1.21E-01	1.15E-01	8.56E-02	2.54E-01
871	GSM262142	GSE10358	Acute Myeloid Leukaemia	Blood	2.29E-02	1.54E-01	6.17E-02	2.71E-01
872	GSM262143	GSE10358	Acute Myeloid Leukaemia	Blood	3.19E-02	1.47E-01	7.43E-02	2.62E-01
873	GSM262144	GSE10358	Acute Myeloid Leukaemia	Blood	1.30E-01	1.13E-01	1.80E-02	3.25E-01
874	GSM262145	GSE10358	Acute Myeloid Leukaemia	Blood	1.08E-01	1.18E-01	6.17E-02	2.71E-01
875	GSM262146	GSE10358	Acute Myeloid Leukaemia	Blood	5.94E-02	-1.33E-01	1.16E-01	2.38E-01
876	GSM262147	GSE10358	Acute Myeloid Leukaemia	Blood	1.39E-01	1.11E-01	8.56E-02	2.54E-01
877	GSM262148	GSE10358	Acute Myeloid Leukaemia	Blood	2.64E-02	1.51E-01	1.02E-01	2.45E-01
878	GSM262149	GSE10358	Acute Myeloid Leukaemia	Blood	3.86E-03	1.87E-01	1.57E-03	4.12E-01
879	GSM262150	GSE10358	Acute Myeloid Leukaemia	Blood	1.31E-02	1.65E-01	8.56E-02	2.54E-01
880	GSM262151	GSE10358	Acute Myeloid Leukaemia	Blood	2.10E-03	-1.96E-01	8.89E-02	2.52E-01
881	GSM262152	GSE10358	Acute Myeloid Leukaemia	Blood	1.00E-01	1.20E-01	9.85E-03	3.49E-01
882	GSM262153	GSE10358	Acute Myeloid Leukaemia	Blood	1.00E-01	1.20E-01	1.80E-01	2.12E-01
883	GSM262154	GSE10358	Acute Myeloid Leukaemia	Blood	1.30E-01	-1.13E-01	9.82E-02	2.47E-01

884	GSM262155	GSE10358	Acute Myeloid Leukaemia	Blood	1.04E-01	1.19E-01	1.21E-01	2.36E-01
885	GSM262156	GSE10358	Acute Myeloid Leukaemia	Blood	1.61E-02	1.61E-01	9.85E-03	3.49E-01
886	GSM262157	GSE10358	Acute Myeloid Leukaemia	Blood	6.13E-03	1.79E-01	1.25E-02	3.40E-01
887	GSM262158	GSE10358	Acute Myeloid Leukaemia	Blood	4.31E-04	2.20E-01	1.57E-03	4.12E-01
888	GSM262159	GSE10358	Acute Myeloid Leukaemia	Blood	2.77E-02	1.50E-01	5.31E-02	2.78E-01
889	GSM262160	GSE10358	Acute Myeloid Leukaemia	Blood	1.61E-02	1.61E-01	3.16E-02	3.02E-01
890	GSM262161	GSE10358	Acute Myeloid Leukaemia	Blood	1.08E-01	1.18E-01	6.69E-02	2.67E-01
891	GSM262162	GSE10358	Acute Myeloid Leukaemia	Blood	4.39E-02	-1.40E-01	6.17E-02	2.71E-01
892	GSM262163	GSE10358	Acute Myeloid Leukaemia	Blood	5.46E-02	-1.35E-01	1.16E-01	2.38E-01
893	GSM262164	GSE10358	Acute Myeloid Leukaemia	Blood	1.70E-02	1.60E-01	5.31E-02	2.78E-01
894	GSM262165	GSE10358	Acute Myeloid Leukaemia	Blood	1.26E-03	2.04E-01	9.27E-04	4.29E-01
895	GSM262166	GSE10358	Acute Myeloid Leukaemia	Blood	1.61E-02	1.61E-01	6.17E-02	2.71E-01
896	GSM262167	GSE10358	Acute Myeloid Leukaemia	Blood	9.63E-02	-1.21E-01	1.16E-01	2.38E-01
897	GSM262168	GSE10358	Acute Myeloid Leukaemia	Blood	5.01E-02	-1.37E-01	1.60E-01	2.20E-01
898	GSM262169	GSE10358	Acute Myeloid Leukaemia	Blood	7.01E-02	-1.29E-01	8.56E-02	2.54E-01
899	GSM262170	GSE10358	Acute Myeloid Leukaemia	Blood	1.53E-02	1.62E-01	1.06E-01	2.43E-01
900	GSM262171	GSE10358	Acute Myeloid Leukaemia	Blood	3.86E-03	1.87E-01	3.30E-02	3.00E-01
901	GSM262172	GSE10358	Acute Myeloid Leukaemia	Blood	1.53E-03	2.01E-01	2.15E-02	3.18E-01
902	GSM262173	GSE10358	Acute Myeloid Leukaemia	Blood	1.01E-02	1.70E-01	1.19E-02	3.42E-01
903	GSM262174	GSE10358	Acute Myeloid Leukaemia	Blood	2.52E-02	1.52E-01	2.25E-02	3.16E-01
904	GSM262175	GSE10358	Acute Myeloid Leukaemia	Blood	2.14E-01	-9.82E-02	1.16E-01	2.38E-01
905	GSM262176	GSE10358	Acute Myeloid Leukaemia	Blood	1.25E-01	1.14E-01	1.02E-01	2.45E-01
906	GSM262177	GSE10358	Acute Myeloid Leukaemia	Blood	3.67E-02	1.44E-01	7.73E-02	2.60E-01
907	GSM262178	GSE10358	Acute Myeloid Leukaemia	Blood	3.67E-02	-1.44E-01	1.96E-01	2.07E-01
908	GSM262179	GSE10358	Acute Myeloid Leukaemia	Blood	8.57E-02	-1.24E-01	3.15E-01	1.74E-01
909	GSM262180	GSE10358	Acute Myeloid Leukaemia	Blood	1.38E-02	1.64E-01	8.56E-02	2.54E-01
910	GSM262181	GSE10358	Acute Myeloid Leukaemia	Blood	3.23E-03	1.89E-01	2.25E-02	3.16E-01
911	GSM262182	GSE10358	Acute Myeloid Leukaemia	Blood	4.87E-03	1.83E-01	4.55E-02	2.85E-01
912	GSM262183	GSE10358	Acute Myeloid Leukaemia	Blood	1.71E-01	1.05E-01	1.37E-01	2.29E-01
913	GSM262184	GSE10358	Acute Myeloid Leukaemia	Blood	5.46E-02	-1.35E-01	2.21E-01	-1.99E-01
914	GSM262185	GSE10358	Acute Myeloid Leukaemia	Blood	5.46E-02	1.35E-01	7.43E-02	2.62E-01
915	GSM262186	GSE10358	Acute Myeloid Leukaemia	Blood	1.25E-01	1.14E-01	1.02E-01	2.45E-01
916	GSM262187	GSE10358	Acute Myeloid Leukaemia	Blood	7.30E-02	1.28E-01	1.16E-01	2.38E-01
917	GSM262188	GSE10358	Acute Myeloid Leukaemia	Blood	1.53E-02	1.62E-01	4.88E-03	3.74E-01
918	GSM262189	GSE10358	Acute Myeloid Leukaemia	Blood	1.30E-01	-1.13E-01	1.16E-01	2.38E-01
919	GSM262190	GSE10358	Acute Myeloid Leukaemia	Blood	1.77E-01	1.04E-01	6.43E-02	2.69E-01
920	GSM262191	GSE10358	Acute Myeloid Leukaemia	Blood	9.63E-02	1.21E-01	1.37E-01	2.29E-01
921	GSM262192	GSE10358	Acute Myeloid Leukaemia	Blood	1.79E-02	1.59E-01	9.85E-03	3.49E-01
922	GSM262193	GSE10358	Acute Myeloid Leukaemia	Blood	1.08E-01	-1.18E-01	3.15E-01	1.74E-01
923	GSM262194	GSE10358	Acute Myeloid Leukaemia	Blood	3.84E-02	1.43E-01	8.56E-02	2.54E-01
924	GSM262195	GSE10358	Acute Myeloid Leukaemia	Blood	1.06E-02	1.69E-01	2.55E-02	3.11E-01
925	GSM262196	GSE10358	Acute Myeloid Leukaemia	Blood	2.69E-03	1.92E-01	5.98E-03	3.67E-01
926	GSM262197	GSE10358	Acute Myeloid Leukaemia	Blood	4.34E-03	1.85E-01	1.19E-02	3.42E-01
927	GSM262198	GSE10358	Acute Myeloid Leukaemia	Blood	1.88E-02	1.58E-01	6.17E-02	2.71E-01
928	GSM262199	GSE10358	Acute Myeloid Leukaemia	Blood	5.94E-02	1.33E-01	5.31E-02	2.78E-01
929	GSM262200	GSE10358	Acute Myeloid Leukaemia	Blood	1.04E-03	2.07E-01	1.33E-03	4.18E-01
930	GSM262201	GSE10358	Acute Myeloid Leukaemia	Blood	1.49E-01	1.09E-01	8.56E-02	2.54E-01
931	GSM262202	GSE10358	Acute Myeloid Leukaemia	Blood	1.30E-01	-1.13E-01	3.97E-01	1.56E-01
932	GSM262203	GSE10358	Acute Myeloid Leukaemia	Blood	5.46E-02	1.35E-01	9.82E-02	2.47E-01
933	GSM262204	GSE10358	Acute Myeloid Leukaemia	Blood	1.71E-01	-1.05E-01	1.46E-01	-2.25E-01
934	GSM262205	GSE10358	Acute Myeloid Leukaemia	Blood	2.08E-01	9.92E-02	1.02E-01	2.45E-01
935	GSM262206	GSE10358	Acute Myeloid Leukaemia	Blood	6.73E-02	-1.30E-01	5.31E-02	2.78E-01
936	GSM262207	GSE10358	Acute Myeloid Leukaemia	Blood	7.01E-02	1.29E-01	2.55E-02	3.11E-01
937	GSM262208	GSE10358	Acute Myeloid Leukaemia	Blood	4.34E-03	1.85E-01	1.43E-02	3.34E-01
938	GSM262209	GSE10358	Acute Myeloid Leukaemia	Blood	1.79E-02	1.59E-01	7.43E-02	2.62E-01
939	GSM262210	GSE10358	Acute Myeloid Leukaemia	Blood	8.52E-04	2.10E-01	2.25E-02	3.16E-01
940	GSM262211	GSE10358	Acute Myeloid Leukaemia	Blood	6.73E-02	1.30E-01	7.43E-02	2.62E-01
941	GSM262212	GSE10358	Acute Myeloid Leukaemia	Blood	1.77E-01	-1.04E-01	3.72E-01	1.61E-01
942	GSM262213	GSE10358	Acute Myeloid Leukaemia	Blood	6.19E-02	1.32E-01	8.89E-02	2.52E-01
943	GSM262214	GSE10358	Acute Myeloid Leukaemia	Blood	1.60E-01	1.07E-01	1.75E-01	2.14E-01
944	GSM262215	GSE10358	Acute Myeloid Leukaemia	Blood	7.45E-04	2.12E-01	4.36E-02	2.87E-01
945	GSM262216	GSE10358	Acute Myeloid Leukaemia	Blood	6.13E-03	1.79E-01	3.16E-02	3.02E-01
946	GSM262217	GSE10358	Acute Myeloid Leukaemia	Blood	7.30E-02	1.28E-01	8.12E-03	3.56E-01
947	GSM262218	GSE10358	Acute Myeloid Leukaemia	Blood	3.19E-02	1.47E-01	9.85E-03	3.49E-01
948	GSM262219	GSE10358	Acute Myeloid Leukaemia	Blood	8.24E-02	1.25E-01	1.06E-01	2.43E-01
949	GSM262220	GSE10358	Acute Myeloid Leukaemia	Blood	4.60E-03	1.84E-01	8.56E-02	2.54E-01
950	GSM262221	GSE10358	Acute Myeloid Leukaemia	Blood	1.65E-01	1.06E-01	1.16E-01	2.38E-01
951	GSM262222	GSE10358	Acute Myeloid Leukaemia	Blood	1.38E-02	1.64E-01	4.36E-02	2.87E-01
952	GSM726172	GSE10358	Acute Myeloid Leukaemia	Blood	2.86E-03	1.91E-01	3.97E-03	3.82E-01
953	GSM726173	GSE10358	Acute Myeloid Leukaemia	Blood	3.67E-02	1.44E-01	1.37E-01	2.29E-01
954	GSM726174	GSE10358	Acute Myeloid Leukaemia	Blood	1.12E-07	3.16E-01	2.11E-05	5.32E-01
955	GSM726175	GSE10358	Acute Myeloid Leukaemia	Blood	5.01E-02	1.37E-01	2.55E-02	3.11E-01
956	GSM726176	GSE10358	Acute Myeloid Leukaemia	Blood	1.31E-02	1.65E-01	7.81E-04	4.34E-01
957	GSM726177	GSE10358	Acute Myeloid Leukaemia	Blood	3.42E-03	1.88E-01	1.50E-02	3.32E-01

958	GSM726178	GSE10358	Acute Myeloid Leukaemia	Blood	2.96E-01	-8.73E-02	3.89E-02	2.92E-01
959	GSM726179	GSE10358	Acute Myeloid Leukaemia	Blood	4.98E-05	2.49E-01	5.80E-05	5.07E-01
960	GSM726180	GSE10358	Acute Myeloid Leukaemia	Blood	1.39E-01	-1.11E-01	7.43E-02	2.62E-01
961	GSM726181	GSE10358	Acute Myeloid Leukaemia	Blood	4.59E-02	-1.39E-01	1.55E-01	2.22E-01
962	GSM726182	GSE10358	Acute Myeloid Leukaemia	Blood	8.24E-02	-1.25E-01	2.02E-01	2.05E-01
963	GSM726183	GSE10358	Acute Myeloid Leukaemia	Blood	6.46E-02	-1.31E-01	4.55E-02	2.85E-01
964	GSM726184	GSE10358	Acute Myeloid Leukaemia	Blood	5.69E-02	1.34E-01	1.66E-02	3.29E-01
965	GSM726185	GSE10358	Acute Myeloid Leukaemia	Blood	8.57E-02	1.24E-01	2.55E-02	3.11E-01
966	GSM726186	GSE10358	Acute Myeloid Leukaemia	Blood	5.69E-02	1.34E-01	4.36E-02	2.87E-01
967	GSM726187	GSE10358	Acute Myeloid Leukaemia	Blood	2.40E-02	-1.53E-01	2.86E-01	1.82E-01
968	GSM726188	GSE10358	Acute Myeloid Leukaemia	Blood	5.01E-02	-1.37E-01	9.58E-02	2.49E-01
969	GSM726189	GSE10358	Acute Myeloid Leukaemia	Blood	1.25E-02	1.66E-01	1.46E-02	3.34E-01
970	GSM726190	GSE10358	Acute Myeloid Leukaemia	Blood	4.79E-02	-1.38E-01	5.31E-02	2.78E-01
971	GSM726191	GSE10358	Acute Myeloid Leukaemia	Blood	1.38E-02	1.64E-01	9.82E-02	2.47E-01
972	GSM726192	GSE10358	Acute Myeloid Leukaemia	Blood	5.23E-02	1.36E-01	6.09E-02	2.71E-01
973	GSM726193	GSE10358	Acute Myeloid Leukaemia	Blood	1.25E-01	-1.14E-01	1.16E-01	2.38E-01
974	GSM726194	GSE10358	Acute Myeloid Leukaemia	Blood	2.69E-03	1.92E-01	9.85E-03	3.49E-01
975	GSM726195	GSE10358	Acute Myeloid Leukaemia	Blood	1.04E-01	-1.19E-01	8.89E-02	2.52E-01
976	GSM726196	GSE10358	Acute Myeloid Leukaemia	Blood	1.74E-03	1.99E-01	3.21E-03	3.89E-01
977	GSM726197	GSE10358	Acute Myeloid Leukaemia	Blood	7.60E-02	-1.27E-01	2.26E-01	1.98E-01
978	GSM726198	GSE10358	Acute Myeloid Leukaemia	Blood	3.50E-02	1.45E-01	7.43E-02	2.62E-01
979	GSM726199	GSE10358	Acute Myeloid Leukaemia	Blood	1.38E-02	1.64E-01	4.88E-03	3.74E-01
980	GSM726200	GSE10358	Acute Myeloid Leukaemia	Blood	2.84E-06	2.83E-01	4.21E-04	4.52E-01
981	GSM726201	GSE10358	Acute Myeloid Leukaemia	Blood	5.46E-02	-1.35E-01	1.32E-01	2.31E-01
982	GSM726202	GSE10358	Acute Myeloid Leukaemia	Blood	2.82E-04	2.26E-01	5.98E-03	3.67E-01
983	GSM726203	GSE10358	Acute Myeloid Leukaemia	Blood	4.39E-02	-1.40E-01	2.02E-01	2.05E-01
984	GSM726204	GSE10358	Acute Myeloid Leukaemia	Blood	6.13E-03	1.79E-01	2.15E-02	3.18E-01
985	GSM726205	GSE10358	Acute Myeloid Leukaemia	Blood	2.40E-02	1.53E-01	2.67E-02	3.09E-01
986	GSM726206	GSE10358	Acute Myeloid Leukaemia	Blood	2.14E-01	-9.82E-02	1.02E-01	2.45E-01
987	GSM726207	GSE10358	Acute Myeloid Leukaemia	Blood	4.34E-03	1.85E-01	5.31E-02	2.78E-01
988	GSM726208	GSE10358	Acute Myeloid Leukaemia	Blood	5.46E-02	1.35E-01	8.56E-02	2.54E-01
989	GSM726209	GSE10358	Acute Myeloid Leukaemia	Blood	5.79E-03	1.80E-01	1.04E-02	3.47E-01
990	GSM726210	GSE10358	Acute Myeloid Leukaemia	Blood	2.29E-02	1.54E-01	1.43E-02	3.34E-01
991	GSM726211	GSE10358	Acute Myeloid Leukaemia	Blood	3.67E-02	1.44E-01	1.19E-02	3.42E-01
992	GSM726212	GSE10358	Acute Myeloid Leukaemia	Blood	2.91E-02	1.49E-01	1.32E-01	2.31E-01
993	GSM726213	GSE10358	Acute Myeloid Leukaemia	Blood	1.18E-03	2.05E-01	2.15E-02	3.18E-01
994	GSM726214	GSE10358	Acute Myeloid Leukaemia	Blood	3.50E-02	1.45E-01	1.80E-02	3.25E-01
995	GSM726215	GSE10358	Acute Myeloid Leukaemia	Blood	8.24E-02	1.25E-01	1.16E-01	2.38E-01
996	GSM726216	GSE10358	Acute Myeloid Leukaemia	Blood	6.73E-02	-1.30E-01	5.31E-02	2.78E-01
997	GSM726217	GSE10358	Acute Myeloid Leukaemia	Blood	4.34E-03	-1.85E-01	1.07E-01	-2.43E-01
998	GSM726218	GSE10358	Acute Myeloid Leukaemia	Blood	7.30E-02	1.28E-01	2.59E-03	3.96E-01
999	GSM726219	GSE10358	Acute Myeloid Leukaemia	Blood	6.73E-02	-1.30E-01	1.16E-01	2.38E-01
1000	GSM726220	GSE10358	Acute Myeloid Leukaemia	Blood	1.70E-02	1.60E-01	8.56E-02	2.54E-01
1001	GSM726221	GSE10358	Acute Myeloid Leukaemia	Blood	3.74E-04	2.22E-01	4.82E-04	4.49E-01
1002	GSM726222	GSE10358	Acute Myeloid Leukaemia	Blood	1.77E-01	1.04E-01	4.22E-01	1.51E-01
1003	GSM726223	GSE10358	Acute Myeloid Leukaemia	Blood	1.88E-02	1.58E-01	2.67E-02	3.09E-01
1004	GSM726224	GSE10358	Acute Myeloid Leukaemia	Blood	1.12E-02	1.68E-01	1.17E-03	4.22E-01
1005	GSM726225	GSE10358	Acute Myeloid Leukaemia	Blood	1.46E-02	1.63E-01	1.50E-02	3.32E-01
1006	GSM726226	GSE10358	Acute Myeloid Leukaemia	Blood	9.63E-02	1.21E-01	8.56E-02	2.54E-01
1007	GSM726227	GSE10358	Acute Myeloid Leukaemia	Blood	4.59E-02	1.39E-01	3.16E-02	3.02E-01
1008	GSM726228	GSE10358	Acute Myeloid Leukaemia	Blood	2.64E-02	1.51E-01	1.25E-02	3.40E-01
1009	GSM726229	GSE10358	Acute Myeloid Leukaemia	Blood	7.25E-03	1.76E-01	2.15E-02	3.18E-01
1010	GSM726230	GSE10358	Acute Myeloid Leukaemia	Blood	1.97E-03	1.97E-01	1.43E-02	3.34E-01
1011	GSM726231	GSE10358	Acute Myeloid Leukaemia	Blood	2.18E-02	-1.55E-01	2.51E-01	1.91E-01
1012	GSM726232	GSE10358	Acute Myeloid Leukaemia	Blood	1.18E-03	2.05E-01	2.44E-03	3.98E-01
1013	GSM726233	GSE10358	Acute Myeloid Leukaemia	Blood	6.73E-02	1.30E-01	4.36E-02	2.87E-01
1014	GSM726234	GSE10358	Acute Myeloid Leukaemia	Blood	1.06E-02	1.69E-01	2.55E-02	3.11E-01
1015	GSM726235	GSE10358	Acute Myeloid Leukaemia	Blood	4.39E-02	-1.40E-01	2.51E-01	1.91E-01
1016	GSM726236	GSE10358	Acute Myeloid Leukaemia	Blood	1.25E-01	-1.14E-01	6.43E-02	2.69E-01
1017	GSM726237	GSE10358	Acute Myeloid Leukaemia	Blood	4.79E-02	1.38E-01	1.12E-01	2.40E-01
1018	GSM726238	GSE10358	Acute Myeloid Leukaemia	Blood	1.79E-02	1.59E-01	9.85E-03	3.49E-01
1019	GSM726239	GSE10358	Acute Myeloid Leukaemia	Blood	1.30E-01	1.13E-01	4.55E-02	2.85E-01
1020	GSM726240	GSE10358	Acute Myeloid Leukaemia	Blood	1.61E-02	1.61E-01	3.30E-02	3.00E-01
1021	GSM726241	GSE10358	Acute Myeloid Leukaemia	Blood	5.23E-02	1.36E-01	9.85E-03	3.49E-01
1022	GSM726242	GSE10358	Acute Myeloid Leukaemia	Blood	5.79E-03	1.80E-01	6.17E-02	2.71E-01
1023	GSM726243	GSE10358	Acute Myeloid Leukaemia	Blood	1.46E-02	1.63E-01	9.82E-02	2.47E-01
1024	GSM726244	GSE10358	Acute Myeloid Leukaemia	Blood	1.54E-01	1.08E-01	8.03E-02	2.58E-01
1025	GSM726245	GSE10358	Acute Myeloid Leukaemia	Blood	2.91E-02	1.49E-01	2.26E-01	1.98E-01
1026	GSM726246	GSE10358	Acute Myeloid Leukaemia	Blood	3.13E-01	-8.53E-02	6.43E-02	2.69E-01
1027	GSM726247	GSE10358	Acute Myeloid Leukaemia	Blood	1.53E-03	2.01E-01	1.72E-02	3.27E-01
1028	GSM726248	GSE10358	Acute Myeloid Leukaemia	Blood	4.39E-02	1.40E-01	9.82E-02	2.47E-01
1029	GSM726249	GSE10358	Acute Myeloid Leukaemia	Blood	2.91E-02	1.49E-01	3.16E-02	3.02E-01
1030	GSM726250	GSE10358	Acute Myeloid Leukaemia	Blood	1.61E-02	-1.61E-01	3.58E-01	-1.65E-01
1031	GSM726251	GSE10358	Acute Myeloid Leukaemia	Blood	1.97E-02	1.57E-01	8.56E-02	2.54E-01

1032	GSM726252	GSE10358	Acute Myeloid Leukaemia	Blood	1.00E-01	-1.20E-01	6.43E-02	2.69E-01
1033	GSM726253	GSE10358	Acute Myeloid Leukaemia	Blood	2.40E-02	-1.53E-01	1.37E-01	2.29E-01
1034	GSM726254	GSE10358	Acute Myeloid Leukaemia	Blood	2.24E-03	1.95E-01	5.98E-03	3.67E-01
1035	GSM726255	GSE10358	Acute Myeloid Leukaemia	Blood	1.30E-01	1.13E-01	2.32E-01	1.96E-01
1036	GSM726256	GSE10358	Acute Myeloid Leukaemia	Blood	3.50E-02	-1.45E-01	1.55E-01	2.22E-01
1037	GSM726257	GSE10358	Acute Myeloid Leukaemia	Blood	7.25E-03	1.76E-01	1.72E-02	3.27E-01
1038	GSM726258	GSE10358	Acute Myeloid Leukaemia	Blood	6.86E-03	1.77E-01	1.43E-02	3.34E-01
1039	GSM726259	GSE10358	Acute Myeloid Leukaemia	Blood	5.47E-03	1.81E-01	2.25E-02	3.16E-01
1040	GSM726260	GSE10358	Acute Myeloid Leukaemia	Blood	1.79E-02	-1.59E-01	2.51E-01	1.91E-01
1041	GSM726261	GSE10358	Acute Myeloid Leukaemia	Blood	1.12E-01	1.17E-01	2.15E-02	3.18E-01
1042	GSM726262	GSE10358	Acute Myeloid Leukaemia	Blood	8.10E-03	1.74E-01	6.31E-03	3.65E-01
1043	GSM400688	GSE16015	Acute Myeloid Leukaemia	Blood	9.54E-03	1.71E-01	3.72E-02	2.94E-01
1044	GSM400689	GSE16015	Acute Myeloid Leukaemia	Blood	4.23E-01	7.34E-02	1.16E-01	2.38E-01
1045	GSM400690	GSE16015	Acute Myeloid Leukaemia	Blood	3.67E-02	1.44E-01	2.26E-01	1.98E-01
1046	GSM400691	GSE16015	Acute Myeloid Leukaemia	Blood	1.21E-01	1.15E-01	7.04E-03	3.61E-01
1047	GSM400692	GSE16015	Acute Myeloid Leukaemia	Blood	2.40E-02	1.53E-01	2.25E-02	3.16E-01
1048	GSM400693	GSE16015	Acute Myeloid Leukaemia	Blood	8.57E-02	1.24E-01	4.36E-02	2.87E-01
1049	GSM400694	GSE16015	Acute Myeloid Leukaemia	Blood	6.13E-03	1.79E-01	3.72E-02	2.94E-01
1050	GSM400695	GSE16015	Acute Myeloid Leukaemia	Blood	4.20E-02	1.41E-01	1.06E-01	2.43E-01
1051	GSM400696	GSE16015	Acute Myeloid Leukaemia	Blood	1.88E-02	1.58E-01	4.44E-03	3.78E-01
1052	GSM400697	GSE16015	Acute Myeloid Leukaemia	Blood	1.60E-01	1.07E-01	1.80E-02	3.25E-01
1053	GSM400698	GSE16015	Acute Myeloid Leukaemia	Blood	1.06E-02	-1.69E-01	2.32E-01	1.96E-01
1054	GSM400699	GSE16015	Acute Myeloid Leukaemia	Blood	1.79E-02	-1.59E-01	3.55E-01	1.65E-01
1055	GSM400700	GSE16015	Acute Myeloid Leukaemia	Blood	4.60E-03	1.84E-01	4.88E-03	3.74E-01
1056	GSM400701	GSE16015	Acute Myeloid Leukaemia	Blood	1.04E-03	2.07E-01	4.20E-03	3.80E-01
1057	GSM400702	GSE16015	Acute Myeloid Leukaemia	Blood	2.64E-02	1.51E-01	4.20E-03	3.80E-01
1058	GSM400703	GSE16015	Acute Myeloid Leukaemia	Blood	1.31E-02	1.65E-01	2.30E-03	4.00E-01
1059	GSM400704	GSE16015	Acute Myeloid Leukaemia	Blood	3.86E-03	1.87E-01	1.16E-01	2.38E-01
1060	GSM400705	GSE16015	Acute Myeloid Leukaemia	Blood	6.73E-02	-1.30E-01	2.46E-01	-1.92E-01
1061	GSM400706	GSE16015	Acute Myeloid Leukaemia	Blood	9.54E-03	1.71E-01	7.64E-05	5.00E-01
1062	GSM400707	GSE16015	Acute Myeloid Leukaemia	Blood	1.08E-01	1.18E-01	1.25E-01	2.34E-01
1063	GSM400708	GSE16015	Acute Myeloid Leukaemia	Blood	8.56E-03	1.73E-01	9.82E-02	2.47E-01
1064	GSM400709	GSE16015	Acute Myeloid Leukaemia	Blood	8.57E-02	1.24E-01	2.26E-01	1.98E-01
1065	GSM400710	GSE16015	Acute Myeloid Leukaemia	Blood	2.07E-02	1.56E-01	4.75E-02	2.83E-01
1066	GSM400711	GSE16015	Acute Myeloid Leukaemia	Blood	1.85E-03	1.98E-01	3.97E-03	3.82E-01
1067	GSM400712	GSE16015	Acute Myeloid Leukaemia	Blood	3.23E-03	1.89E-01	5.45E-03	3.70E-01
1068	GSM400713	GSE16015	Acute Myeloid Leukaemia	Blood	2.64E-02	1.51E-01	1.32E-01	2.31E-01
1069	GSM400714	GSE16015	Acute Myeloid Leukaemia	Blood	5.69E-02	-1.34E-01	5.31E-02	2.78E-01
1070	GSM400715	GSE16015	Acute Myeloid Leukaemia	Blood	1.06E-02	1.69E-01	7.70E-03	3.58E-01
1071	GSM400716	GSE16015	Acute Myeloid Leukaemia	Blood	1.00E-04	2.40E-01	4.21E-04	4.52E-01
1072	GSM400717	GSE16015	Acute Myeloid Leukaemia	Blood	1.46E-02	1.63E-01	1.25E-02	3.40E-01
1073	GSM400718	GSE16015	Acute Myeloid Leukaemia	Blood	4.39E-02	1.40E-01	1.55E-01	2.22E-01
1074	GSM400719	GSE16015	Acute Myeloid Leukaemia	Blood	1.39E-01	1.11E-01	4.75E-02	2.83E-01
1075	GSM400720	GSE16015	Acute Myeloid Leukaemia	Blood	1.12E-01	-1.17E-01	7.43E-02	2.62E-01
1076	GSM400721	GSE16015	Acute Myeloid Leukaemia	Blood	2.42E-01	-9.42E-02	1.16E-01	2.38E-01
1077	GSM400722	GSE16015	Acute Myeloid Leukaemia	Blood	7.92E-02	1.26E-01	2.67E-02	3.09E-01
1078	GSM400723	GSE16015	Acute Myeloid Leukaemia	Blood	1.18E-03	2.05E-01	3.16E-02	3.02E-01
1079	GSM400724	GSE16015	Acute Myeloid Leukaemia	Blood	2.52E-02	-1.52E-01	4.41E-01	1.47E-01
1080	GSM400725	GSE16015	Acute Myeloid Leukaemia	Blood	2.77E-02	1.50E-01	9.85E-03	3.49E-01
1081	GSM400726	GSE16015	Acute Myeloid Leukaemia	Blood	4.01E-02	1.42E-01	1.43E-02	3.34E-01
1082	GSM400727	GSE16015	Acute Myeloid Leukaemia	Blood	1.74E-03	1.99E-01	2.44E-03	3.98E-01
1083	GSM400728	GSE16015	Acute Myeloid Leukaemia	Blood	9.26E-02	1.22E-01	2.15E-02	3.18E-01
1084	GSM400729	GSE16015	Acute Myeloid Leukaemia	Blood	1.00E-04	2.40E-01	4.88E-03	3.74E-01
1085	GSM400730	GSE16015	Acute Myeloid Leukaemia	Blood	5.46E-02	1.35E-01	1.32E-01	2.31E-01
1086	GSM400731	GSE16015	Acute Myeloid Leukaemia	Blood	1.61E-02	1.61E-01	4.36E-02	2.87E-01
1087	GSM400732	GSE16015	Acute Myeloid Leukaemia	Blood	2.38E-03	1.94E-01	1.80E-02	3.25E-01
1088	GSM400733	GSE16015	Acute Myeloid Leukaemia	Blood	5.94E-02	1.33E-01	2.51E-01	1.91E-01
1089	GSM400734	GSE16015	Acute Myeloid Leukaemia	Blood	1.61E-02	1.61E-01	5.10E-02	2.80E-01
1090	GSM400735	GSE16015	Acute Myeloid Leukaemia	Blood	3.67E-02	1.44E-01	6.43E-02	2.69E-01
1091	GSM400736	GSE16015	Acute Myeloid Leukaemia	Blood	4.20E-02	1.41E-01	2.15E-02	3.18E-01
1092	GSM400737	GSE16015	Acute Myeloid Leukaemia	Blood	1.70E-02	1.60E-01	1.16E-01	2.38E-01
1093	GSM400738	GSE16015	Acute Myeloid Leukaemia	Blood	2.27E-04	-2.29E-01	1.92E-01	-2.09E-01
1094	GSM400739	GSE16015	Acute Myeloid Leukaemia	Blood	5.23E-02	-1.36E-01	1.10E-01	2.41E-01
1095	GSM400740	GSE16015	Acute Myeloid Leukaemia	Blood	7.30E-02	1.28E-01	2.15E-02	3.18E-01
1096	GSM400741	GSE16015	Acute Myeloid Leukaemia	Blood	5.69E-02	1.34E-01	3.72E-02	2.94E-01
1097	GSM400742	GSE16015	Acute Myeloid Leukaemia	Blood	1.06E-02	1.69E-01	2.15E-02	3.18E-01
1098	GSM400743	GSE16015	Acute Myeloid Leukaemia	Blood	2.24E-03	1.95E-01	4.36E-02	2.87E-01
1099	GSM400744	GSE16015	Acute Myeloid Leukaemia	Blood	9.26E-02	-1.22E-01	3.16E-02	3.02E-01
1100	GSM400745	GSE16015	Acute Myeloid Leukaemia	Blood	1.53E-02	1.62E-01	5.31E-02	2.78E-01
1101	GSM400746	GSE16015	Acute Myeloid Leukaemia	Blood	2.18E-02	1.55E-01	1.19E-02	3.42E-01
1102	GSM400747	GSE16015	Acute Myeloid Leukaemia	Blood	7.25E-03	1.76E-01	7.43E-02	2.62E-01
1103	GSM400748	GSE16015	Acute Myeloid Leukaemia	Blood	8.24E-02	1.25E-01	4.55E-02	2.85E-01
1104	GSM400749	GSE16015	Acute Myeloid Leukaemia	Blood	4.79E-02	-1.38E-01	3.72E-02	2.94E-01
1105	GSM400750	GSE16015	Acute Myeloid Leukaemia	Blood	3.04E-03	1.90E-01	3.72E-02	2.94E-01

1106	GSM400751	GSE16015	Acute Myeloid Leukaemia	Blood	2.24E-03	1.95E-01	4.55E-02	2.85E-01
1107	GSM400752	GSE16015	Acute Myeloid Leukaemia	Blood	7.30E-02	1.28E-01	2.26E-01	1.98E-01
1108	GSM400753	GSE16015	Acute Myeloid Leukaemia	Blood	8.56E-03	1.73E-01	4.36E-02	2.87E-01
1109	GSM400754	GSE16015	Acute Myeloid Leukaemia	Blood	2.52E-02	1.52E-01	1.16E-01	2.38E-01
1110	GSM400755	GSE16015	Acute Myeloid Leukaemia	Blood	8.91E-02	-1.23E-01	7.73E-02	2.60E-01
1111	GSM400756	GSE16015	Acute Myeloid Leukaemia	Blood	3.50E-02	1.45E-01	4.55E-02	2.85E-01
1112	GSM400757	GSE16015	Acute Myeloid Leukaemia	Blood	1.83E-01	-1.03E-01	6.17E-02	2.71E-01
1113	GSM400758	GSE16015	Acute Myeloid Leukaemia	Blood	2.10E-03	1.96E-01	3.72E-02	2.94E-01
1114	GSM400759	GSE16015	Acute Myeloid Leukaemia	Blood	3.67E-02	1.44E-01	3.30E-02	3.00E-01
1115	GSM400760	GSE16015	Acute Myeloid Leukaemia	Blood	1.04E-01	1.19E-01	5.54E-02	2.76E-01
1116	GSM400761	GSE16015	Acute Myeloid Leukaemia	Blood	2.49E-01	-9.33E-02	7.43E-02	2.62E-01
1117	GSM400762	GSE16015	Acute Myeloid Leukaemia	Blood	6.13E-03	1.79E-01	2.55E-02	3.11E-01
1118	GSM400763	GSE16015	Acute Myeloid Leukaemia	Blood	4.59E-02	1.39E-01	1.75E-01	2.14E-01
1119	GSM400764	GSE16015	Acute Myeloid Leukaemia	Blood	1.61E-02	1.61E-01	3.89E-02	2.92E-01
1120	GSM400765	GSE16015	Acute Myeloid Leukaemia	Blood	1.83E-01	1.03E-01	5.31E-02	2.78E-01
1121	GSM400766	GSE16015	Acute Myeloid Leukaemia	Blood	7.01E-02	1.29E-01	5.31E-02	2.78E-01
1122	GSM400767	GSE16015	Acute Myeloid Leukaemia	Blood	2.28E-01	9.62E-02	1.16E-01	2.38E-01
1123	GSM400768	GSE16015	Acute Myeloid Leukaemia	Blood	4.79E-02	1.38E-01	1.12E-01	2.40E-01
1124	GSM400769	GSE16015	Acute Myeloid Leukaemia	Blood	1.97E-02	1.57E-01	1.02E-01	2.45E-01
1125	GSM400770	GSE16015	Acute Myeloid Leukaemia	Blood	6.73E-02	1.30E-01	4.55E-02	2.85E-01
1126	GSM400771	GSE16015	Acute Myeloid Leukaemia	Blood	1.12E-02	1.68E-01	4.55E-02	2.85E-01
1127	GSM400772	GSE16015	Acute Myeloid Leukaemia	Blood	1.25E-01	1.14E-01	5.31E-02	2.78E-01
1128	GSM400773	GSE16015	Acute Myeloid Leukaemia	Blood	8.24E-02	1.25E-01	7.43E-02	2.62E-01
1129	GSM400774	GSE16015	Acute Myeloid Leukaemia	Blood	4.79E-02	1.38E-01	7.43E-02	2.62E-01
1130	GSM400775	GSE16015	Acute Myeloid Leukaemia	Blood	3.67E-02	1.44E-01	7.43E-02	2.62E-01
1131	GSM400776	GSE16015	Acute Myeloid Leukaemia	Blood	1.44E-01	-1.10E-01	8.89E-02	2.52E-01
1132	GSM400777	GSE16015	Acute Myeloid Leukaemia	Blood	1.88E-02	1.58E-01	4.55E-02	2.85E-01
1133	GSM400778	GSE16015	Acute Myeloid Leukaemia	Blood	7.01E-02	-1.29E-01	2.86E-01	1.82E-01
1134	GSM400779	GSE16015	Acute Myeloid Leukaemia	Blood	1.38E-02	1.64E-01	4.55E-02	2.85E-01
1135	GSM400780	GSE16015	Acute Myeloid Leukaemia	Blood	9.63E-02	1.21E-01	5.54E-02	2.76E-01
1136	GSM400781	GSE16015	Acute Myeloid Leukaemia	Blood	1.53E-02	1.62E-01	1.19E-02	3.42E-01
1137	GSM400782	GSE16015	Acute Myeloid Leukaemia	Blood	7.60E-02	1.27E-01	1.75E-01	2.14E-01
1138	GSM400783	GSE16015	Acute Myeloid Leukaemia	Blood	4.79E-02	1.38E-01	1.32E-01	2.31E-01
1139	GSM400784	GSE16015	Acute Myeloid Leukaemia	Blood	5.23E-02	1.36E-01	7.43E-02	2.62E-01
1140	GSM400785	GSE16015	Acute Myeloid Leukaemia	Blood	1.25E-01	-1.14E-01	4.55E-02	2.85E-01
1141	GSM400786	GSE16015	Acute Myeloid Leukaemia	Blood	1.61E-02	1.61E-01	1.32E-01	2.31E-01
1142	GSM400787	GSE16015	Acute Myeloid Leukaemia	Blood	2.21E-01	9.72E-02	1.75E-01	2.14E-01
1143	GSM400788	GSE16015	Acute Myeloid Leukaemia	Blood	3.67E-02	1.44E-01	1.02E-01	2.45E-01
1144	GSM400789	GSE16015	Acute Myeloid Leukaemia	Blood	3.30E-01	8.33E-02	1.02E-01	2.45E-01
1145	GSM400790	GSE16015	Acute Myeloid Leukaemia	Blood	3.50E-02	-1.45E-01	3.01E-01	1.78E-01
1146	GSM400791	GSE16015	Acute Myeloid Leukaemia	Blood	4.39E-02	1.40E-01	1.32E-01	2.31E-01
1147	GSM400792	GSE16015	Acute Myeloid Leukaemia	Blood	2.52E-02	1.52E-01	2.19E-01	2.00E-01
1148	GSM400793	GSE16015	Acute Myeloid Leukaemia	Blood	8.57E-02	-1.24E-01	2.02E-01	2.05E-01
1149	GSM400794	GSE16015	Acute Myeloid Leukaemia	Blood	1.12E-02	1.68E-01	2.05E-02	3.20E-01
1150	GSM592391	GSE24080	Multiple Myeloma	Blood	2.21E-01	-9.72E-02	2.66E-01	1.87E-01
1151	GSM592392	GSE24080	Multiple Myeloma	Blood	1.74E-03	-1.99E-01	1.16E-01	2.38E-01
1152	GSM592393	GSE24080	Multiple Myeloma	Blood	2.91E-02	-1.49E-01	1.19E-02	3.42E-01
1153	GSM592394	GSE24080	Multiple Myeloma	Blood	5.47E-03	-1.81E-01	2.19E-01	2.00E-01
1154	GSM592395	GSE24080	Multiple Myeloma	Blood	2.44E-04	-2.28E-01	9.58E-02	-2.49E-01
1155	GSM592396	GSE24080	Multiple Myeloma	Blood	3.86E-03	-1.87E-01	1.96E-01	2.07E-01
1156	GSM592397	GSE24080	Multiple Myeloma	Blood	3.67E-02	-1.44E-01	1.32E-01	2.31E-01
1157	GSM592398	GSE24080	Multiple Myeloma	Blood	7.96E-05	-2.43E-01	1.32E-01	2.31E-01
1158	GSM592399	GSE24080	Multiple Myeloma	Blood	1.54E-01	-1.08E-01	2.26E-01	1.98E-01
1159	GSM592400	GSE24080	Multiple Myeloma	Blood	8.86E-06	-2.70E-01	2.88E-01	-1.81E-01
1160	GSM592401	GSE24080	Multiple Myeloma	Blood	7.45E-04	-2.12E-01	7.24E-02	-2.63E-01
1161	GSM592402	GSE24080	Multiple Myeloma	Blood	4.39E-02	-1.40E-01	4.55E-02	2.85E-01
1162	GSM592403	GSE24080	Multiple Myeloma	Blood	1.44E-01	-1.10E-01	3.15E-01	1.74E-01
1163	GSM592404	GSE24080	Multiple Myeloma	Blood	3.21E-01	-8.43E-02	1.34E-01	2.30E-01
1164	GSM592405	GSE24080	Multiple Myeloma	Blood	3.19E-02	-1.47E-01	6.17E-02	2.71E-01
1165	GSM592406	GSE24080	Multiple Myeloma	Blood	6.48E-03	-1.78E-01	6.17E-02	2.71E-01
1166	GSM592407	GSE24080	Multiple Myeloma	Blood	1.70E-02	-1.60E-01	9.82E-02	2.47E-01
1167	GSM592408	GSE24080	Multiple Myeloma	Blood	6.48E-03	-1.78E-01	5.31E-02	2.78E-01
1168	GSM592409	GSE24080	Multiple Myeloma	Blood	6.13E-03	-1.79E-01	1.32E-01	2.31E-01
1169	GSM592410	GSE24080	Multiple Myeloma	Blood	4.62E-04	-2.19E-01	2.53E-01	-1.90E-01
1170	GSM592411	GSE24080	Multiple Myeloma	Blood	2.88E-01	-8.83E-02	2.15E-02	3.18E-01
1171	GSM592412	GSE24080	Multiple Myeloma	Blood	2.01E-01	-1.00E-01	2.05E-02	3.20E-01
1172	GSM592413	GSE24080	Multiple Myeloma	Blood	1.12E-02	-1.68E-01	2.19E-01	2.00E-01
1173	GSM592414	GSE24080	Multiple Myeloma	Blood	3.84E-02	-1.43E-01	3.15E-01	1.74E-01
1174	GSM592415	GSE24080	Multiple Myeloma	Blood	2.77E-02	-1.50E-01	7.43E-02	2.62E-01
1175	GSM592416	GSE24080	Multiple Myeloma	Blood	2.69E-03	-1.92E-01	2.19E-01	2.00E-01
1176	GSM592417	GSE24080	Multiple Myeloma	Blood	1.88E-02	-1.58E-01	2.19E-01	2.00E-01
1177	GSM592418	GSE24080	Multiple Myeloma	Blood	1.12E-02	-1.68E-01	1.14E-01	-2.39E-01
1178	GSM592419	GSE24080	Multiple Myeloma	Blood	2.21E-01	9.72E-02	1.75E-01	2.14E-01
1179	GSM592420	GSE24080	Multiple Myeloma	Blood	7.67E-03	-1.75E-01	1.55E-01	2.22E-01

1180	GSM592421	GSE24080	Multiple Myeloma	Blood	4.79E-02	-1.38E-01	2.88E-01	-1.81E-01
1181	GSM592422	GSE24080	Multiple Myeloma	Blood	4.09E-03	-1.86E-01	3.46E-01	1.67E-01
1182	GSM592423	GSE24080	Multiple Myeloma	Blood	1.04E-03	-2.07E-01	8.67E-02	-2.54E-01
1183	GSM592424	GSE24080	Multiple Myeloma	Blood	2.01E-01	-1.00E-01	1.12E-01	2.40E-01
1184	GSM592425	GSE24080	Multiple Myeloma	Blood	1.49E-01	-1.09E-01	2.67E-02	3.09E-01
1185	GSM592426	GSE24080	Multiple Myeloma	Blood	3.49E-04	-2.23E-01	6.96E-02	-2.65E-01
1186	GSM592427	GSE24080	Multiple Myeloma	Blood	2.18E-02	-1.55E-01	1.12E-01	2.40E-01
1187	GSM592428	GSE24080	Multiple Myeloma	Blood	4.79E-02	-1.38E-01	1.55E-01	2.22E-01
1188	GSM592429	GSE24080	Multiple Myeloma	Blood	1.12E-02	-1.68E-01	2.19E-01	2.00E-01
1189	GSM592430	GSE24080	Multiple Myeloma	Blood	3.63E-03	-1.88E-01	3.78E-01	1.60E-01
1190	GSM592431	GSE24080	Multiple Myeloma	Blood	2.10E-03	-1.96E-01	5.31E-02	2.78E-01
1191	GSM592432	GSE24080	Multiple Myeloma	Blood	5.47E-03	-1.81E-01	2.19E-01	2.00E-01
1192	GSM592433	GSE24080	Multiple Myeloma	Blood	1.11E-03	-2.06E-01	6.43E-02	2.69E-01
1193	GSM592434	GSE24080	Multiple Myeloma	Blood	1.65E-01	-1.06E-01	2.26E-01	1.98E-01
1194	GSM592435	GSE24080	Multiple Myeloma	Blood	1.06E-02	-1.69E-01	1.96E-01	2.07E-01
1195	GSM592436	GSE24080	Multiple Myeloma	Blood	5.16E-03	-1.82E-01	1.96E-01	2.07E-01
1196	GSM592437	GSE24080	Multiple Myeloma	Blood	1.16E-01	-1.16E-01	1.80E-02	3.25E-01
1197	GSM592438	GSE24080	Multiple Myeloma	Blood	3.23E-03	-1.89E-01	1.32E-01	2.31E-01
1198	GSM592439	GSE24080	Multiple Myeloma	Blood	1.44E-01	-1.10E-01	6.67E-03	3.63E-01
1199	GSM592440	GSE24080	Multiple Myeloma	Blood	3.23E-03	-1.89E-01	3.15E-01	1.74E-01
1200	GSM592441	GSE24080	Multiple Myeloma	Blood	1.04E-01	-1.19E-01	5.54E-02	2.76E-01
1201	GSM592442	GSE24080	Multiple Myeloma	Blood	8.24E-02	-1.25E-01	9.27E-04	4.29E-01
1202	GSM592443	GSE24080	Multiple Myeloma	Blood	1.21E-01	-1.15E-01	2.51E-01	1.91E-01
1203	GSM592444	GSE24080	Multiple Myeloma	Blood	2.49E-01	9.33E-02	1.43E-02	3.34E-01
1204	GSM592445	GSE24080	Multiple Myeloma	Blood	1.63E-03	-2.00E-01	6.01E-02	-2.72E-01
1205	GSM592446	GSE24080	Multiple Myeloma	Blood	1.38E-02	-1.64E-01	2.15E-02	3.18E-01
1206	GSM592447	GSE24080	Multiple Myeloma	Blood	3.63E-03	-1.88E-01	1.55E-01	2.22E-01
1207	GSM592448	GSE24080	Multiple Myeloma	Blood	6.46E-02	1.31E-01	1.89E-02	3.23E-01
1208	GSM592449	GSE24080	Multiple Myeloma	Blood	1.34E-01	-1.12E-01	4.55E-02	2.85E-01
1209	GSM592450	GSE24080	Multiple Myeloma	Blood	1.35E-05	-2.65E-01	1.42E-01	2.27E-01
1210	GSM592451	GSE24080	Multiple Myeloma	Blood	7.92E-02	-1.26E-01	1.89E-02	3.23E-01
1211	GSM592452	GSE24080	Multiple Myeloma	Blood	7.30E-02	-1.28E-01	4.06E-02	2.90E-01
1212	GSM592453	GSE24080	Multiple Myeloma	Blood	3.19E-02	-1.47E-01	8.12E-03	3.56E-01
1213	GSM592454	GSE24080	Multiple Myeloma	Blood	4.59E-02	1.39E-01	3.06E-04	4.62E-01
1214	GSM592455	GSE24080	Multiple Myeloma	Blood	1.25E-01	1.14E-01	1.02E-01	2.45E-01
1215	GSM592456	GSE24080	Multiple Myeloma	Blood	5.47E-03	-1.81E-01	2.67E-02	3.09E-01
1216	GSM592457	GSE24080	Multiple Myeloma	Blood	9.26E-02	-1.22E-01	4.55E-02	2.85E-01
1217	GSM592458	GSE24080	Multiple Myeloma	Blood	8.24E-02	1.25E-01	4.36E-02	2.87E-01
1218	GSM592459	GSE24080	Multiple Myeloma	Blood	1.97E-03	-1.97E-01	8.56E-02	2.54E-01
1219	GSM592460	GSE24080	Multiple Myeloma	Blood	9.26E-02	-1.22E-01	8.89E-02	2.52E-01
1220	GSM592461	GSE24080	Multiple Myeloma	Blood	4.25E-05	-2.51E-01	7.24E-02	-2.63E-01
1221	GSM592462	GSE24080	Multiple Myeloma	Blood	2.64E-02	-1.51E-01	3.72E-02	2.94E-01
1222	GSM592463	GSE24080	Multiple Myeloma	Blood	2.07E-02	-1.56E-01	4.55E-02	2.85E-01
1223	GSM592464	GSE24080	Multiple Myeloma	Blood	2.38E-03	-1.94E-01	2.08E-01	2.03E-01
1224	GSM592465	GSE24080	Multiple Myeloma	Blood	1.31E-02	-1.65E-01	7.43E-02	2.62E-01
1225	GSM592466	GSE24080	Multiple Myeloma	Blood	3.23E-03	-1.89E-01	2.44E-03	3.98E-01
1226	GSM592467	GSE24080	Multiple Myeloma	Blood	1.46E-02	-1.63E-01	3.89E-02	2.92E-01
1227	GSM592468	GSE24080	Multiple Myeloma	Blood	1.16E-01	-1.16E-01	2.46E-01	1.92E-01
1228	GSM592469	GSE24080	Multiple Myeloma	Blood	9.11E-04	-2.09E-01	1.37E-01	2.29E-01
1229	GSM592470	GSE24080	Multiple Myeloma	Blood	6.73E-02	1.30E-01	1.98E-02	3.21E-01
1230	GSM592471	GSE24080	Multiple Myeloma	Blood	5.39E-05	-2.48E-01	1.66E-02	-3.29E-01
1231	GSM592472	GSE24080	Multiple Myeloma	Blood	8.91E-02	-1.23E-01	3.30E-02	3.00E-01
1232	GSM592473	GSE24080	Multiple Myeloma	Blood	1.65E-06	-2.89E-01	1.75E-01	2.14E-01
1233	GSM592474	GSE24080	Multiple Myeloma	Blood	4.59E-02	-1.39E-01	8.56E-02	2.54E-01
1234	GSM592475	GSE24080	Multiple Myeloma	Blood	4.98E-05	-2.49E-01	2.02E-01	2.05E-01
1235	GSM592476	GSE24080	Multiple Myeloma	Blood	5.69E-02	1.34E-01	7.70E-03	3.58E-01
1236	GSM592477	GSE24080	Multiple Myeloma	Blood	2.29E-02	-1.54E-01	4.20E-03	3.80E-01
1237	GSM592478	GSE24080	Multiple Myeloma	Blood	2.08E-01	-9.92E-02	1.50E-02	3.32E-01
1238	GSM592479	GSE24080	Multiple Myeloma	Blood	4.94E-01	-6.65E-02	3.87E-01	1.58E-01
1239	GSM592480	GSE24080	Multiple Myeloma	Blood	1.39E-01	-1.11E-01	5.16E-03	3.72E-01
1240	GSM592481	GSE24080	Multiple Myeloma	Blood	4.33E-01	7.24E-02	6.69E-02	2.67E-01
1241	GSM592482	GSE24080	Multiple Myeloma	Blood	5.23E-02	-1.36E-01	4.88E-03	3.74E-01
1242	GSM592483	GSE24080	Multiple Myeloma	Blood	4.34E-03	-1.85E-01	2.51E-01	1.91E-01
1243	GSM592484	GSE24080	Multiple Myeloma	Blood	1.74E-05	-2.62E-01	1.96E-01	2.07E-01
1244	GSM592485	GSE24080	Multiple Myeloma	Blood	2.29E-02	-1.54E-01	6.43E-02	2.69E-01
1245	GSM592486	GSE24080	Multiple Myeloma	Blood	2.91E-02	-1.49E-01	1.32E-01	2.31E-01
1246	GSM592487	GSE24080	Multiple Myeloma	Blood	2.18E-02	-1.55E-01	9.23E-02	2.50E-01
1247	GSM592488	GSE24080	Multiple Myeloma	Blood	3.49E-04	-2.23E-01	1.92E-01	-2.09E-01
1248	GSM592489	GSE24080	Multiple Myeloma	Blood	1.57E-04	-2.34E-01	2.58E-01	1.89E-01
1249	GSM592490	GSE24080	Multiple Myeloma	Blood	1.11E-03	-2.06E-01	1.86E-01	-2.10E-01
1250	GSM592491	GSE24080	Multiple Myeloma	Blood	5.46E-02	1.35E-01	3.03E-03	3.91E-01
1251	GSM592492	GSE24080	Multiple Myeloma	Blood	7.25E-03	-1.76E-01	8.56E-02	2.54E-01
1252	GSM592493	GSE24080	Multiple Myeloma	Blood	5.68E-01	5.95E-02	2.19E-01	2.00E-01
1253	GSM592494	GSE24080	Multiple Myeloma	Blood	7.92E-02	-1.26E-01	9.82E-02	2.47E-01

1254	GSM592495	GSE24080	Multiple Myeloma	Blood	9.26E-02	-1.22E-01	3.72E-02	2.94E-01
1255	GSM592496	GSE24080	Multiple Myeloma	Blood	4.87E-03	-1.83E-01	7.43E-02	2.62E-01
1256	GSM592497	GSE24080	Multiple Myeloma	Blood	9.54E-03	-1.71E-01	1.19E-02	3.42E-01
1257	GSM592498	GSE24080	Multiple Myeloma	Blood	4.43E-01	-7.14E-02	8.89E-02	2.52E-01
1258	GSM592499	GSE24080	Multiple Myeloma	Blood	3.84E-01	7.74E-02	4.67E-01	1.42E-01
1259	GSM592500	GSE24080	Multiple Myeloma	Blood	6.48E-03	-1.78E-01	1.96E-01	2.07E-01
1260	GSM592501	GSE24080	Multiple Myeloma	Blood	7.30E-02	-1.28E-01	1.65E-01	2.18E-01
1261	GSM592502	GSE24080	Multiple Myeloma	Blood	1.39E-01	-1.11E-01	1.75E-01	2.14E-01
1262	GSM592503	GSE24080	Multiple Myeloma	Blood	7.30E-02	-1.28E-01	5.31E-02	2.78E-01
1263	GSM592504	GSE24080	Multiple Myeloma	Blood	1.30E-01	-1.13E-01	2.19E-01	2.00E-01
1264	GSM592505	GSE24080	Multiple Myeloma	Blood	1.49E-01	1.09E-01	7.43E-02	2.62E-01
1265	GSM592506	GSE24080	Multiple Myeloma	Blood	1.70E-02	-1.60E-01	1.75E-01	2.14E-01
1266	GSM592507	GSE24080	Multiple Myeloma	Blood	2.64E-01	-9.13E-02	5.07E-01	-1.34E-01
1267	GSM592508	GSE24080	Multiple Myeloma	Blood	4.20E-02	1.41E-01	4.88E-03	3.74E-01
1268	GSM592509	GSE24080	Multiple Myeloma	Blood	1.60E-01	-1.07E-01	2.25E-02	3.16E-01
1269	GSM592510	GSE24080	Multiple Myeloma	Blood	1.89E-01	-1.02E-01	1.12E-01	2.40E-01
1270	GSM592511	GSE24080	Multiple Myeloma	Blood	4.01E-02	1.42E-01	7.70E-03	3.58E-01
1271	GSM592512	GSE24080	Multiple Myeloma	Blood	3.67E-02	-1.44E-01	1.25E-02	3.40E-01
1272	GSM592513	GSE24080	Multiple Myeloma	Blood	2.38E-03	-1.94E-01	3.97E-01	1.56E-01
1273	GSM592514	GSE24080	Multiple Myeloma	Blood	1.98E-06	-2.87E-01	4.44E-03	-3.78E-01
1274	GSM592515	GSE24080	Multiple Myeloma	Blood	4.34E-03	-1.85E-01	7.73E-02	2.60E-01
1275	GSM592516	GSE24080	Multiple Myeloma	Blood	4.87E-03	-1.83E-01	8.56E-02	2.54E-01
1276	GSM592517	GSE24080	Multiple Myeloma	Blood	1.38E-02	-1.64E-01	1.02E-01	2.45E-01
1277	GSM592518	GSE24080	Multiple Myeloma	Blood	1.38E-02	-1.64E-01	2.81E-01	-1.83E-01
1278	GSM592519	GSE24080	Multiple Myeloma	Blood	8.57E-02	-1.24E-01	2.67E-02	3.09E-01
1279	GSM592520	GSE24080	Multiple Myeloma	Blood	1.79E-02	-1.59E-01	7.43E-02	2.62E-01
1280	GSM592521	GSE24080	Multiple Myeloma	Blood	1.38E-02	1.64E-01	4.21E-04	4.52E-01
1281	GSM592522	GSE24080	Multiple Myeloma	Blood	2.01E-01	1.00E-01	9.34E-03	3.51E-01
1282	GSM592523	GSE24080	Multiple Myeloma	Blood	6.19E-02	-1.32E-01	3.46E-02	2.98E-01
1283	GSM592524	GSE24080	Multiple Myeloma	Blood	2.29E-02	-1.54E-01	1.32E-01	2.31E-01
1284	GSM592525	GSE24080	Multiple Myeloma	Blood	9.54E-03	-1.71E-01	1.43E-02	3.34E-01
1285	GSM592526	GSE24080	Multiple Myeloma	Blood	1.63E-03	-2.00E-01	1.46E-01	2.25E-01
1286	GSM592527	GSE24080	Multiple Myeloma	Blood	1.26E-04	-2.37E-01	7.43E-02	2.62E-01
1287	GSM592528	GSE24080	Multiple Myeloma	Blood	5.69E-02	-1.34E-01	1.80E-01	2.12E-01
1288	GSM592529	GSE24080	Multiple Myeloma	Blood	1.21E-01	1.15E-01	1.89E-02	3.23E-01
1289	GSM592530	GSE24080	Multiple Myeloma	Blood	2.38E-03	-1.94E-01	1.02E-01	2.45E-01
1290	GSM592531	GSE24080	Multiple Myeloma	Blood	1.89E-01	-1.02E-01	3.15E-01	1.74E-01
1291	GSM592532	GSE24080	Multiple Myeloma	Blood	4.01E-02	-1.42E-01	8.89E-02	2.52E-01
1292	GSM592533	GSE24080	Multiple Myeloma	Blood	2.21E-01	-9.72E-02	1.75E-01	2.14E-01
1293	GSM592534	GSE24080	Multiple Myeloma	Blood	3.66E-01	-7.94E-02	1.58E-02	3.30E-01
1294	GSM592535	GSE24080	Multiple Myeloma	Blood	3.67E-02	-1.44E-01	5.54E-02	2.76E-01
1295	GSM592536	GSE24080	Multiple Myeloma	Blood	1.12E-02	1.68E-01	1.09E-02	3.45E-01
1296	GSM592537	GSE24080	Multiple Myeloma	Blood	1.65E-01	1.06E-01	7.81E-04	4.34E-01
1297	GSM592538	GSE24080	Multiple Myeloma	Blood	6.46E-02	-1.31E-01	1.32E-01	2.31E-01
1298	GSM592539	GSE24080	Multiple Myeloma	Blood	1.26E-04	-2.37E-01	1.46E-01	-2.25E-01
1299	GSM592540	GSE24080	Multiple Myeloma	Blood	2.80E-01	-8.93E-02	6.67E-03	3.63E-01
1300	GSM592541	GSE24080	Multiple Myeloma	Blood	2.40E-02	-1.53E-01	2.19E-01	2.00E-01
1301	GSM592542	GSE24080	Multiple Myeloma	Blood	7.60E-02	-1.27E-01	4.06E-02	2.90E-01
1302	GSM592543	GSE24080	Multiple Myeloma	Blood	7.92E-02	1.26E-01	4.36E-02	2.87E-01
1303	GSM592544	GSE24080	Multiple Myeloma	Blood	1.21E-01	-1.15E-01	2.67E-02	3.09E-01
1304	GSM592545	GSE24080	Multiple Myeloma	Blood	5.27E-06	-2.76E-01	2.15E-01	-2.01E-01
1305	GSM592546	GSE24080	Multiple Myeloma	Blood	1.53E-02	-1.62E-01	7.43E-02	2.62E-01
1306	GSM592547	GSE24080	Multiple Myeloma	Blood	9.26E-02	1.22E-01	4.06E-02	2.90E-01
1307	GSM592548	GSE24080	Multiple Myeloma	Blood	1.39E-01	-1.11E-01	5.31E-02	2.78E-01
1308	GSM592549	GSE24080	Multiple Myeloma	Blood	2.01E-01	-1.00E-01	2.25E-02	3.16E-01
1309	GSM592550	GSE24080	Multiple Myeloma	Blood	3.34E-02	-1.46E-01	1.37E-01	2.29E-01
1310	GSM592551	GSE24080	Multiple Myeloma	Blood	5.47E-03	-1.81E-01	9.82E-02	2.47E-01
1311	GSM592552	GSE24080	Multiple Myeloma	Blood	5.94E-02	-1.33E-01	5.79E-01	1.20E-01
1312	GSM592553	GSE24080	Multiple Myeloma	Blood	1.71E-01	-1.05E-01	1.65E-01	2.18E-01
1313	GSM592554	GSE24080	Multiple Myeloma	Blood	7.01E-02	-1.29E-01	1.16E-01	2.38E-01
1314	GSM592555	GSE24080	Multiple Myeloma	Blood	8.24E-02	1.25E-01	2.15E-02	3.18E-01
1315	GSM592556	GSE24080	Multiple Myeloma	Blood	1.74E-05	-2.62E-01	1.96E-01	2.07E-01
1316	GSM592557	GSE24080	Multiple Myeloma	Blood	1.65E-01	-1.06E-01	3.30E-02	3.00E-01
1317	GSM592558	GSE24080	Multiple Myeloma	Blood	3.66E-01	7.94E-02	1.96E-01	2.07E-01
1318	GSM592559	GSE24080	Multiple Myeloma	Blood	1.12E-01	-1.17E-01	1.19E-02	3.42E-01
1319	GSM592560	GSE24080	Multiple Myeloma	Blood	2.52E-02	1.52E-01	1.17E-03	4.22E-01
1320	GSM592561	GSE24080	Multiple Myeloma	Blood	7.92E-02	-1.26E-01	2.15E-01	2.01E-01
1321	GSM592562	GSE24080	Multiple Myeloma	Blood	3.38E-01	-8.23E-02	1.57E-01	2.21E-01
1322	GSM592563	GSE24080	Multiple Myeloma	Blood	7.30E-02	1.28E-01	6.31E-03	3.65E-01
1323	GSM592564	GSE24080	Multiple Myeloma	Blood	1.53E-02	-1.62E-01	3.89E-02	2.92E-01
1324	GSM592565	GSE24080	Multiple Myeloma	Blood	1.46E-02	-1.63E-01	9.82E-02	2.47E-01
1325	GSM592566	GSE24080	Multiple Myeloma	Blood	4.79E-02	-1.38E-01	3.78E-01	1.60E-01
1326	GSM592567	GSE24080	Multiple Myeloma	Blood	6.51E-04	2.14E-01	6.86E-04	4.38E-01
1327	GSM592568	GSE24080	Multiple Myeloma	Blood	1.74E-03	-1.99E-01	1.42E-01	-2.27E-01

1328	GSM592569	GSE24080	Multiple Myeloma	Blood	7.01E-02	-1.29E-01	8.89E-02	2.52E-01
1329	GSM592570	GSE24080	Multiple Myeloma	Blood	2.64E-02	-1.51E-01	1.06E-01	2.43E-01
1330	GSM592571	GSE24080	Multiple Myeloma	Blood	2.77E-02	1.50E-01	1.50E-02	3.32E-01
1331	GSM592572	GSE24080	Multiple Myeloma	Blood	3.47E-01	-8.13E-02	1.32E-01	2.31E-01
1332	GSM592573	GSE24080	Multiple Myeloma	Blood	1.83E-01	1.03E-01	4.55E-02	2.85E-01
1333	GSM592574	GSE24080	Multiple Myeloma	Blood	4.20E-02	-1.41E-01	3.55E-01	1.65E-01
1334	GSM592575	GSE24080	Multiple Myeloma	Blood	5.46E-02	1.35E-01	2.15E-02	3.18E-01
1335	GSM592576	GSE24080	Multiple Myeloma	Blood	3.94E-01	-7.64E-02	5.31E-02	2.78E-01
1336	GSM592577	GSE24080	Multiple Myeloma	Blood	3.50E-02	-1.45E-01	1.32E-01	2.31E-01
1337	GSM592578	GSE24080	Multiple Myeloma	Blood	2.18E-02	-1.55E-01	3.72E-02	2.94E-01
1338	GSM592579	GSE24080	Multiple Myeloma	Blood	5.68E-04	-2.16E-01	1.58E-02	-3.30E-01
1339	GSM592580	GSE24080	Multiple Myeloma	Blood	1.21E-01	1.15E-01	5.16E-03	3.72E-01
1340	GSM592581	GSE24080	Multiple Myeloma	Blood	1.71E-01	-1.05E-01	7.70E-03	3.58E-01
1341	GSM592582	GSE24080	Multiple Myeloma	Blood	1.65E-01	1.06E-01	4.75E-02	2.83E-01
1342	GSM592583	GSE24080	Multiple Myeloma	Blood	5.69E-02	-1.34E-01	7.43E-02	2.62E-01
1343	GSM592584	GSE24080	Multiple Myeloma	Blood	5.01E-02	-1.37E-01	1.47E-03	4.14E-01
1344	GSM592585	GSE24080	Multiple Myeloma	Blood	6.48E-03	-1.78E-01	7.73E-02	2.60E-01
1345	GSM592586	GSE24080	Multiple Myeloma	Blood	3.19E-02	-1.47E-01	9.82E-02	2.47E-01
1346	GSM592587	GSE24080	Multiple Myeloma	Blood	6.13E-03	-1.79E-01	2.51E-01	1.91E-01
1347	GSM592588	GSE24080	Multiple Myeloma	Blood	1.71E-01	1.05E-01	1.58E-02	3.30E-01
1348	GSM592589	GSE24080	Multiple Myeloma	Blood	5.16E-03	-1.82E-01	6.17E-02	2.71E-01
1349	GSM592590	GSE24080	Multiple Myeloma	Blood	3.23E-03	-1.89E-01	4.36E-02	2.87E-01
1350	GSM592591	GSE24080	Multiple Myeloma	Blood	1.88E-02	-1.58E-01	2.02E-01	2.05E-01
1351	GSM592592	GSE24080	Multiple Myeloma	Blood	4.25E-05	-2.51E-01	1.75E-01	2.14E-01
1352	GSM592593	GSE24080	Multiple Myeloma	Blood	2.10E-03	-1.96E-01	1.32E-01	2.31E-01
1353	GSM592594	GSE24080	Multiple Myeloma	Blood	3.84E-02	-1.43E-01	1.37E-01	2.29E-01
1354	GSM592595	GSE24080	Multiple Myeloma	Blood	6.86E-03	-1.77E-01	2.61E-01	-1.88E-01
1355	GSM592596	GSE24080	Multiple Myeloma	Blood	5.16E-03	-1.82E-01	3.72E-02	2.94E-01
1356	GSM592597	GSE24080	Multiple Myeloma	Blood	6.48E-03	-1.78E-01	5.54E-02	2.76E-01
1357	GSM592598	GSE24080	Multiple Myeloma	Blood	4.62E-04	-2.19E-01	1.29E-01	-2.32E-01
1358	GSM592599	GSE24080	Multiple Myeloma	Blood	9.26E-02	-1.22E-01	1.55E-01	2.22E-01
1359	GSM592600	GSE24080	Multiple Myeloma	Blood	3.67E-02	-1.44E-01	1.06E-01	2.43E-01
1360	GSM592601	GSE24080	Multiple Myeloma	Blood	7.92E-02	-1.26E-01	9.27E-04	4.29E-01
1361	GSM592602	GSE24080	Multiple Myeloma	Blood	7.01E-02	-1.29E-01	3.97E-03	3.82E-01
1362	GSM592603	GSE24080	Multiple Myeloma	Blood	1.71E-01	1.05E-01	9.85E-03	3.49E-01
1363	GSM592604	GSE24080	Multiple Myeloma	Blood	4.39E-02	-1.40E-01	1.96E-01	2.07E-01
1364	GSM592605	GSE24080	Multiple Myeloma	Blood	1.60E-01	1.07E-01	4.88E-03	3.74E-01
1365	GSM592606	GSE24080	Multiple Myeloma	Blood	2.40E-02	-1.53E-01	5.54E-02	2.76E-01
1366	GSM592607	GSE24080	Multiple Myeloma	Blood	3.34E-02	-1.46E-01	1.12E-01	2.40E-01
1367	GSM592608	GSE24080	Multiple Myeloma	Blood	1.04E-01	-1.19E-01	2.02E-01	2.05E-01
1368	GSM592609	GSE24080	Multiple Myeloma	Blood	4.79E-02	-1.38E-01	8.89E-02	2.52E-01
1369	GSM592610	GSE24080	Multiple Myeloma	Blood	8.57E-02	-1.24E-01	2.59E-03	3.96E-01
1370	GSM592611	GSE24080	Multiple Myeloma	Blood	7.36E-05	-2.44E-01	2.73E-01	-1.85E-01
1371	GSM592612	GSE24080	Multiple Myeloma	Blood	8.10E-03	-1.74E-01	6.43E-02	2.69E-01
1372	GSM592613	GSE24080	Multiple Myeloma	Blood	3.67E-02	-1.44E-01	1.21E-01	2.36E-01
1373	GSM592614	GSE24080	Multiple Myeloma	Blood	2.53E-03	-1.93E-01	2.66E-01	1.87E-01
1374	GSM592615	GSE24080	Multiple Myeloma	Blood	6.19E-02	-1.32E-01	3.21E-03	3.89E-01
1375	GSM592616	GSE24080	Multiple Myeloma	Blood	5.16E-03	-1.82E-01	5.31E-02	2.78E-01
1376	GSM592617	GSE24080	Multiple Myeloma	Blood	1.88E-02	-1.58E-01	1.32E-01	2.31E-01
1377	GSM592618	GSE24080	Multiple Myeloma	Blood	9.26E-02	1.22E-01	8.12E-03	3.56E-01
1378	GSM592619	GSE24080	Multiple Myeloma	Blood	3.50E-02	-1.45E-01	1.37E-01	2.29E-01
1379	GSM592620	GSE24080	Multiple Myeloma	Blood	3.05E-02	-1.48E-01	1.75E-01	2.14E-01
1380	GSM592621	GSE24080	Multiple Myeloma	Blood	1.38E-02	1.64E-01	9.89E-04	4.27E-01
1381	GSM592622	GSE24080	Multiple Myeloma	Blood	8.57E-02	1.24E-01	7.43E-02	2.62E-01
1382	GSM592623	GSE24080	Multiple Myeloma	Blood	1.79E-02	-1.59E-01	7.73E-02	2.60E-01
1383	GSM592624	GSE24080	Multiple Myeloma	Blood	8.91E-02	-1.23E-01	2.15E-01	-2.01E-01
1384	GSM592625	GSE24080	Multiple Myeloma	Blood	1.26E-03	-2.04E-01	1.34E-01	-2.30E-01
1385	GSM592626	GSE24080	Multiple Myeloma	Blood	3.84E-01	-7.74E-02	2.58E-01	1.89E-01
1386	GSM592627	GSE24080	Multiple Myeloma	Blood	1.44E-03	-2.02E-01	1.46E-01	2.25E-01
1387	GSM592628	GSE24080	Multiple Myeloma	Blood	2.57E-01	-9.23E-02	1.16E-01	2.38E-01
1388	GSM592629	GSE24080	Multiple Myeloma	Blood	5.01E-02	-1.37E-01	1.46E-01	2.25E-01
1389	GSM592630	GSE24080	Multiple Myeloma	Blood	3.23E-03	-1.89E-01	1.75E-01	2.14E-01
1390	GSM592631	GSE24080	Multiple Myeloma	Blood	1.38E-02	-1.64E-01	7.73E-02	2.60E-01
1391	GSM592632	GSE24080	Multiple Myeloma	Blood	1.44E-03	-2.02E-01	2.08E-01	-2.03E-01
1392	GSM592633	GSE24080	Multiple Myeloma	Blood	2.49E-01	9.33E-02	5.16E-03	3.72E-01
1393	GSM592634	GSE24080	Multiple Myeloma	Blood	5.23E-02	-1.36E-01	9.34E-03	3.51E-01
1394	GSM592635	GSE24080	Multiple Myeloma	Blood	8.24E-02	1.25E-01	1.19E-02	3.42E-01
1395	GSM592636	GSE24080	Multiple Myeloma	Blood	3.34E-02	-1.46E-01	1.16E-01	2.38E-01
1396	GSM592637	GSE24080	Multiple Myeloma	Blood	4.60E-03	1.84E-01	1.71E-04	4.78E-01
1397	GSM592638	GSE24080	Multiple Myeloma	Blood	4.23E-01	-7.34E-02	9.34E-03	3.51E-01
1398	GSM592639	GSE24080	Multiple Myeloma	Blood	6.19E-02	-1.32E-01	6.17E-02	2.71E-01
1399	GSM592640	GSE24080	Multiple Myeloma	Blood	4.23E-01	7.34E-02	6.31E-03	3.65E-01
1400	GSM592641	GSE24080	Multiple Myeloma	Blood	4.87E-03	-1.83E-01	2.15E-02	3.18E-01
1401	GSM592642	GSE24080	Multiple Myeloma	Blood	6.73E-02	-1.30E-01	5.31E-02	2.78E-01

1402	GSM592643	GSE24080	Multiple Myeloma	Blood	2.88E-01	8.83E-02	1.04E-02	3.47E-01
1403	GSM592644	GSE24080	Multiple Myeloma	Blood	6.86E-03	-1.77E-01	5.31E-02	2.78E-01
1404	GSM592645	GSE24080	Multiple Myeloma	Blood	2.10E-03	-1.96E-01	1.32E-01	2.31E-01
1405	GSM592646	GSE24080	Multiple Myeloma	Blood	1.63E-03	-2.00E-01	1.37E-01	2.29E-01
1406	GSM592647	GSE24080	Multiple Myeloma	Blood	1.46E-02	-1.63E-01	3.03E-03	3.91E-01
1407	GSM592648	GSE24080	Multiple Myeloma	Blood	2.38E-03	-1.94E-01	1.50E-02	3.32E-01
1408	GSM592649	GSE24080	Multiple Myeloma	Blood	1.63E-03	-2.00E-01	4.36E-02	2.87E-01
1409	GSM592650	GSE24080	Multiple Myeloma	Blood	1.21E-01	-1.15E-01	1.72E-02	3.27E-01
1410	GSM592651	GSE24080	Multiple Myeloma	Blood	6.19E-02	-1.32E-01	6.69E-02	2.67E-01
1411	GSM592652	GSE24080	Multiple Myeloma	Blood	3.19E-02	-1.47E-01	1.21E-01	2.36E-01
1412	GSM592653	GSE24080	Multiple Myeloma	Blood	2.91E-02	-1.49E-01	8.12E-03	3.56E-01
1413	GSM592654	GSE24080	Multiple Myeloma	Blood	1.53E-02	-1.62E-01	4.36E-02	2.87E-01
1414	GSM592655	GSE24080	Multiple Myeloma	Blood	1.04E-01	-1.19E-01	3.81E-01	-1.59E-01
1415	GSM592656	GSE24080	Multiple Myeloma	Blood	4.39E-02	-1.40E-01	9.82E-02	2.47E-01
1416	GSM592657	GSE24080	Multiple Myeloma	Blood	2.64E-01	9.13E-02	8.56E-02	2.54E-01
1417	GSM592658	GSE24080	Multiple Myeloma	Blood	4.03E-01	-7.54E-02	3.89E-02	2.92E-01
1418	GSM592659	GSE24080	Multiple Myeloma	Blood	2.10E-03	1.96E-01	2.37E-04	4.69E-01
1419	GSM592660	GSE24080	Multiple Myeloma	Blood	1.31E-02	-1.65E-01	3.78E-01	1.60E-01
1420	GSM592661	GSE24080	Multiple Myeloma	Blood	1.97E-02	-1.57E-01	2.02E-01	2.05E-01
1421	GSM592662	GSE24080	Multiple Myeloma	Blood	1.65E-01	-1.06E-01	2.86E-01	1.82E-01
1422	GSM592663	GSE24080	Multiple Myeloma	Blood	3.67E-02	-1.44E-01	9.82E-02	2.47E-01
1423	GSM592664	GSE24080	Multiple Myeloma	Blood	5.94E-02	-1.33E-01	1.80E-01	2.12E-01
1424	GSM592665	GSE24080	Multiple Myeloma	Blood	7.25E-03	-1.76E-01	1.37E-01	2.29E-01
1425	GSM592666	GSE24080	Multiple Myeloma	Blood	1.21E-01	-1.15E-01	1.37E-01	2.29E-01
1426	GSM592667	GSE24080	Multiple Myeloma	Blood	6.19E-02	1.32E-01	2.05E-02	3.20E-01
1427	GSM592668	GSE24080	Multiple Myeloma	Blood	3.67E-02	-1.44E-01	1.96E-01	2.07E-01
1428	GSM592669	GSE24080	Multiple Myeloma	Blood	2.64E-02	-1.51E-01	1.92E-01	-2.09E-01
1429	GSM592670	GSE24080	Multiple Myeloma	Blood	2.91E-02	-1.49E-01	3.15E-01	1.74E-01
1430	GSM592671	GSE24080	Multiple Myeloma	Blood	5.01E-02	-1.37E-01	7.73E-02	2.60E-01
1431	GSM592672	GSE24080	Multiple Myeloma	Blood	2.29E-02	-1.54E-01	8.56E-02	2.54E-01
1432	GSM592673	GSE24080	Multiple Myeloma	Blood	8.57E-02	-1.24E-01	3.16E-02	3.02E-01
1433	GSM592674	GSE24080	Multiple Myeloma	Blood	2.29E-02	-1.54E-01	1.75E-01	2.14E-01
1434	GSM592675	GSE24080	Multiple Myeloma	Blood	2.62E-05	-2.57E-01	4.24E-02	-2.89E-01
1435	GSM592676	GSE24080	Multiple Myeloma	Blood	5.69E-02	-1.34E-01	2.59E-03	3.96E-01
1436	GSM592677	GSE24080	Multiple Myeloma	Blood	9.04E-03	-1.72E-01	4.55E-02	2.85E-01
1437	GSM592678	GSE24080	Multiple Myeloma	Blood	7.45E-04	-2.12E-01	6.96E-02	-2.65E-01
1438	GSM592679	GSE24080	Multiple Myeloma	Blood	5.79E-03	-1.80E-01	7.43E-02	2.62E-01
1439	GSM592680	GSE24080	Multiple Myeloma	Blood	1.01E-02	1.70E-01	4.21E-04	4.52E-01
1440	GSM592681	GSE24080	Multiple Myeloma	Blood	9.63E-02	1.21E-01	2.44E-03	3.98E-01
1441	GSM592682	GSE24080	Multiple Myeloma	Blood	1.97E-03	-1.97E-01	1.76E-01	-2.14E-01
1442	GSM592683	GSE24080	Multiple Myeloma	Blood	1.39E-01	-1.11E-01	1.25E-01	2.34E-01
1443	GSM592684	GSE24080	Multiple Myeloma	Blood	4.87E-03	-1.83E-01	3.78E-01	1.60E-01
1444	GSM592685	GSE24080	Multiple Myeloma	Blood	4.13E-01	-7.44E-02	3.94E-01	1.57E-01
1445	GSM592686	GSE24080	Multiple Myeloma	Blood	3.50E-02	1.45E-01	6.67E-03	3.63E-01
1446	GSM592687	GSE24080	Multiple Myeloma	Blood	3.86E-03	-1.87E-01	2.46E-01	-1.92E-01
1447	GSM592688	GSE24080	Multiple Myeloma	Blood	1.44E-01	-1.10E-01	1.96E-03	4.05E-01
1448	GSM592689	GSE24080	Multiple Myeloma	Blood	1.44E-01	-1.10E-01	1.89E-02	3.23E-01
1449	GSM592690	GSE24080	Multiple Myeloma	Blood	7.30E-02	-1.28E-01	1.55E-01	2.22E-01
1450	GSM592691	GSE24080	Multiple Myeloma	Blood	5.69E-02	-1.34E-01	2.26E-01	1.98E-01
1451	GSM592692	GSE24080	Multiple Myeloma	Blood	1.49E-01	-1.09E-01	1.32E-01	2.31E-01
1452	GSM592693	GSE24080	Multiple Myeloma	Blood	1.70E-02	-1.60E-01	1.12E-01	2.40E-01
1453	GSM592694	GSE24080	Multiple Myeloma	Blood	2.40E-02	-1.53E-01	6.17E-02	2.71E-01
1454	GSM592695	GSE24080	Multiple Myeloma	Blood	1.83E-01	1.03E-01	1.75E-01	2.14E-01
1455	GSM592696	GSE24080	Multiple Myeloma	Blood	1.31E-02	-1.65E-01	2.86E-01	1.82E-01
1456	GSM592697	GSE24080	Multiple Myeloma	Blood	2.42E-01	9.42E-02	1.12E-01	2.40E-01
1457	GSM592698	GSE24080	Multiple Myeloma	Blood	1.54E-01	-1.08E-01	8.89E-02	2.52E-01
1458	GSM592699	GSE24080	Multiple Myeloma	Blood	8.24E-02	-1.25E-01	1.37E-01	2.29E-01
1459	GSM592700	GSE24080	Multiple Myeloma	Blood	1.60E-01	1.07E-01	2.44E-03	3.98E-01
1460	GSM592701	GSE24080	Multiple Myeloma	Blood	2.88E-01	-8.83E-02	4.55E-02	2.85E-01
1461	GSM592702	GSE24080	Multiple Myeloma	Blood	5.69E-02	-1.34E-01	2.51E-01	1.91E-01
1462	GSM592703	GSE24080	Multiple Myeloma	Blood	3.42E-03	-1.88E-01	3.46E-01	1.67E-01
1463	GSM592704	GSE24080	Multiple Myeloma	Blood	1.30E-01	-1.13E-01	3.30E-02	3.00E-01
1464	GSM592705	GSE24080	Multiple Myeloma	Blood	2.52E-02	-1.52E-01	3.46E-01	1.67E-01
1465	GSM592706	GSE24080	Multiple Myeloma	Blood	1.53E-02	-1.62E-01	1.46E-01	2.25E-01
1466	GSM592707	GSE24080	Multiple Myeloma	Blood	7.67E-03	-1.75E-01	3.15E-01	1.74E-01
1467	GSM592708	GSE24080	Multiple Myeloma	Blood	7.25E-03	-1.76E-01	2.19E-01	2.00E-01
1468	GSM592709	GSE24080	Multiple Myeloma	Blood	1.04E-01	-1.19E-01	1.60E-01	2.20E-01
1469	GSM592710	GSE24080	Multiple Myeloma	Blood	9.73E-04	-2.08E-01	4.22E-01	1.51E-01
1470	GSM592711	GSE24080	Multiple Myeloma	Blood	2.77E-02	-1.50E-01	1.12E-01	2.40E-01
1471	GSM592712	GSE24080	Multiple Myeloma	Blood	5.23E-02	-1.36E-01	1.75E-01	2.14E-01
1472	GSM592713	GSE24080	Multiple Myeloma	Blood	2.27E-04	-2.29E-01	1.12E-01	2.40E-01
1473	GSM592714	GSE24080	Multiple Myeloma	Blood	2.52E-02	-1.52E-01	1.29E-01	-2.32E-01
1474	GSM592715	GSE24080	Multiple Myeloma	Blood	6.48E-03	-1.78E-01	6.17E-02	2.71E-01
1475	GSM592716	GSE24080	Multiple Myeloma	Blood	3.04E-03	-1.90E-01	5.54E-02	2.76E-01

1476	GSM592717	GSE24080	Multiple Myeloma	Blood	5.23E-02	-1.36E-01	3.90E-01	-1.57E-01
1477	GSM592718	GSE24080	Multiple Myeloma	Blood	1.16E-01	-1.16E-01	1.12E-01	2.40E-01
1478	GSM592719	GSE24080	Multiple Myeloma	Blood	3.23E-03	-1.89E-01	2.32E-01	1.96E-01
1479	GSM592720	GSE24080	Multiple Myeloma	Blood	6.46E-02	-1.31E-01	1.75E-01	2.14E-01
1480	GSM592721	GSE24080	Multiple Myeloma	Blood	9.26E-02	-1.22E-01	1.02E-01	2.45E-01
1481	GSM592722	GSE24080	Multiple Myeloma	Blood	7.01E-02	-1.29E-01	1.37E-01	2.29E-01
1482	GSM592723	GSE24080	Multiple Myeloma	Blood	4.01E-02	-1.42E-01	2.51E-01	1.91E-01
1483	GSM592724	GSE24080	Multiple Myeloma	Blood	1.12E-01	1.17E-01	2.55E-02	3.11E-01
1484	GSM592725	GSE24080	Multiple Myeloma	Blood	1.38E-02	-1.64E-01	3.15E-01	1.74E-01
1485	GSM592726	GSE24080	Multiple Myeloma	Blood	6.73E-02	-1.30E-01	3.72E-02	2.94E-01
1486	GSM592727	GSE24080	Multiple Myeloma	Blood	1.31E-02	-1.65E-01	4.22E-01	1.51E-01
1487	GSM592728	GSE24080	Multiple Myeloma	Blood	2.14E-01	9.82E-02	1.12E-01	2.40E-01
1488	GSM592729	GSE24080	Multiple Myeloma	Blood	2.64E-02	-1.51E-01	5.31E-02	2.78E-01
1489	GSM592730	GSE24080	Multiple Myeloma	Blood	1.60E-01	1.07E-01	1.80E-02	3.25E-01
1490	GSM592731	GSE24080	Multiple Myeloma	Blood	1.12E-01	-1.17E-01	3.72E-02	2.94E-01
1491	GSM592732	GSE24080	Multiple Myeloma	Blood	1.30E-01	1.13E-01	7.43E-02	2.62E-01
1492	GSM592733	GSE24080	Multiple Myeloma	Blood	2.42E-01	9.42E-02	3.72E-02	2.94E-01
1493	GSM592734	GSE24080	Multiple Myeloma	Blood	2.14E-01	-9.82E-02	1.75E-01	2.14E-01
1494	GSM592735	GSE24080	Multiple Myeloma	Blood	2.91E-02	-1.49E-01	2.19E-01	2.00E-01
1495	GSM592736	GSE24080	Multiple Myeloma	Blood	6.19E-02	1.32E-01	2.59E-03	3.96E-01
1496	GSM592737	GSE24080	Multiple Myeloma	Blood	4.87E-03	-1.83E-01	1.16E-01	2.38E-01
1497	GSM592738	GSE24080	Multiple Myeloma	Blood	2.77E-02	-1.50E-01	3.49E-01	-1.67E-01
1498	GSM592739	GSE24080	Multiple Myeloma	Blood	2.69E-03	-1.92E-01	2.19E-01	2.00E-01
1499	GSM592740	GSE24080	Multiple Myeloma	Blood	1.25E-01	1.14E-01	5.31E-02	2.78E-01
1500	GSM592741	GSE24080	Multiple Myeloma	Blood	2.49E-01	-9.33E-02	6.17E-02	2.71E-01
1501	GSM592742	GSE24080	Multiple Myeloma	Blood	1.89E-01	1.02E-01	4.55E-02	2.85E-01
1502	GSM592743	GSE24080	Multiple Myeloma	Blood	5.47E-03	-1.81E-01	1.32E-01	2.31E-01
1503	GSM592744	GSE24080	Multiple Myeloma	Blood	6.48E-03	-1.78E-01	2.19E-01	2.00E-01
1504	GSM592745	GSE24080	Multiple Myeloma	Blood	2.38E-03	-1.94E-01	1.96E-01	2.07E-01
1505	GSM592746	GSE24080	Multiple Myeloma	Blood	6.97E-04	-2.13E-01	1.46E-01	-2.25E-01
1506	GSM592747	GSE24080	Multiple Myeloma	Blood	1.46E-04	-2.35E-01	1.55E-01	2.22E-01
1507	GSM592748	GSE24080	Multiple Myeloma	Blood	3.84E-01	-7.74E-02	3.90E-01	1.57E-01
1508	GSM592749	GSE24080	Multiple Myeloma	Blood	8.10E-03	-1.74E-01	6.17E-02	2.71E-01
1509	GSM592750	GSE24080	Multiple Myeloma	Blood	1.04E-03	-2.07E-01	5.04E-01	1.34E-01
1510	GSM592751	GSE24080	Multiple Myeloma	Blood	2.28E-01	-9.62E-02	2.28E-01	1.97E-01
1511	GSM592752	GSE24080	Multiple Myeloma	Blood	2.91E-02	-1.49E-01	9.82E-02	2.47E-01
1512	GSM592753	GSE24080	Multiple Myeloma	Blood	1.79E-02	-1.59E-01	1.12E-01	2.40E-01
1513	GSM592754	GSE24080	Multiple Myeloma	Blood	2.14E-01	-9.82E-02	3.16E-02	3.02E-01
1514	GSM592755	GSE24080	Multiple Myeloma	Blood	6.13E-03	-1.79E-01	1.80E-01	2.12E-01
1515	GSM592756	GSE24080	Multiple Myeloma	Blood	1.31E-02	-1.65E-01	1.32E-01	2.31E-01
1516	GSM592757	GSE24080	Multiple Myeloma	Blood	1.21E-01	-1.15E-01	1.16E-01	2.38E-01
1517	GSM592758	GSE24080	Multiple Myeloma	Blood	2.07E-02	-1.56E-01	2.86E-01	1.82E-01
1518	GSM592759	GSE24080	Multiple Myeloma	Blood	7.30E-02	1.28E-01	8.12E-03	3.56E-01
1519	GSM592760	GSE24080	Multiple Myeloma	Blood	1.04E-01	-1.19E-01	5.54E-02	2.76E-01
1520	GSM592761	GSE24080	Multiple Myeloma	Blood	2.21E-01	9.72E-02	2.55E-02	3.11E-01
1521	GSM592762	GSE24080	Multiple Myeloma	Blood	5.01E-02	-1.37E-01	3.15E-01	1.74E-01
1522	GSM592763	GSE24080	Multiple Myeloma	Blood	2.64E-02	-1.51E-01	2.19E-01	2.00E-01
1523	GSM592764	GSE24080	Multiple Myeloma	Blood	1.39E-01	1.11E-01	2.05E-02	3.20E-01
1524	GSM592765	GSE24080	Multiple Myeloma	Blood	2.01E-01	-1.00E-01	6.43E-02	2.69E-01
1525	GSM592766	GSE24080	Multiple Myeloma	Blood	5.23E-02	-1.36E-01	4.22E-01	1.51E-01
1526	GSM592767	GSE24080	Multiple Myeloma	Blood	4.01E-02	-1.42E-01	3.15E-01	1.74E-01
1527	GSM592768	GSE24080	Multiple Myeloma	Blood	9.26E-02	-1.22E-01	3.15E-01	1.74E-01
1528	GSM592769	GSE24080	Multiple Myeloma	Blood	5.94E-02	1.33E-01	1.80E-02	3.25E-01
1529	GSM592770	GSE24080	Multiple Myeloma	Blood	2.01E-01	1.00E-01	2.47E-02	3.12E-01
1530	GSM592771	GSE24080	Multiple Myeloma	Blood	5.01E-02	-1.37E-01	5.93E-01	-1.17E-01
1531	GSM592772	GSE24080	Multiple Myeloma	Blood	1.12E-01	-1.17E-01	2.02E-01	2.05E-01
1532	GSM592773	GSE24080	Multiple Myeloma	Blood	9.63E-02	-1.21E-01	1.32E-01	2.31E-01
1533	GSM592774	GSE24080	Multiple Myeloma	Blood	2.91E-02	-1.49E-01	4.75E-02	2.83E-01
1534	GSM592775	GSE24080	Multiple Myeloma	Blood	3.34E-02	-1.46E-01	2.67E-02	3.09E-01
1535	GSM592776	GSE24080	Multiple Myeloma	Blood	3.34E-02	-1.46E-01	2.19E-01	2.00E-01
1536	GSM592777	GSE24080	Multiple Myeloma	Blood	3.05E-02	-1.48E-01	2.02E-01	2.05E-01
1537	GSM592778	GSE24080	Multiple Myeloma	Blood	1.97E-02	-1.57E-01	1.12E-01	2.40E-01
1538	GSM592779	GSE24080	Multiple Myeloma	Blood	7.92E-02	-1.26E-01	5.10E-02	2.80E-01
1539	GSM592780	GSE24080	Multiple Myeloma	Blood	4.09E-03	-1.86E-01	3.15E-01	1.74E-01
1540	GSM592781	GSE24080	Multiple Myeloma	Blood	2.29E-02	1.54E-01	6.86E-04	4.38E-01
1541	GSM592782	GSE24080	Multiple Myeloma	Blood	1.21E-01	1.15E-01	1.96E-01	2.07E-01
1542	GSM592783	GSE24080	Multiple Myeloma	Blood	1.44E-01	-1.10E-01	1.16E-01	2.38E-01
1543	GSM592784	GSE24080	Multiple Myeloma	Blood	2.07E-02	-1.56E-01	2.26E-01	1.98E-01
1544	GSM592785	GSE24080	Multiple Myeloma	Blood	2.96E-01	-8.73E-02	2.93E-02	3.05E-01
1545	GSM592786	GSE24080	Multiple Myeloma	Blood	1.77E-01	1.04E-01	8.56E-02	2.54E-01
1546	GSM592787	GSE24080	Multiple Myeloma	Blood	1.65E-01	1.06E-01	3.30E-02	3.00E-01
1547	GSM592788	GSE24080	Multiple Myeloma	Blood	1.44E-01	-1.10E-01	1.32E-01	2.31E-01
1548	GSM592789	GSE24080	Multiple Myeloma	Blood	8.91E-02	-1.23E-01	1.32E-01	2.31E-01
1549	GSM592790	GSE24080	Multiple Myeloma	Blood	1.83E-01	-1.03E-01	1.86E-01	2.10E-01

1550	GSM592791	GSE24080	Multiple Myeloma	Blood	1.71E-01	1.05E-01	1.57E-03	4.12E-01
1551	GSM592792	GSE24080	Multiple Myeloma	Blood	3.04E-01	8.63E-02	1.89E-02	3.23E-01
1552	GSM592793	GSE24080	Multiple Myeloma	Blood	1.08E-01	-1.18E-01	3.89E-02	2.92E-01
1553	GSM592794	GSE24080	Multiple Myeloma	Blood	1.88E-02	-1.58E-01	3.15E-01	1.74E-01
1554	GSM592795	GSE24080	Multiple Myeloma	Blood	8.10E-03	-1.74E-01	1.96E-01	2.07E-01
1555	GSM592796	GSE24080	Multiple Myeloma	Blood	3.67E-02	-1.44E-01	1.42E-01	2.27E-01
1556	GSM592797	GSE24080	Multiple Myeloma	Blood	6.73E-02	-1.30E-01	1.04E-02	3.47E-01
1557	GSM592798	GSE24080	Multiple Myeloma	Blood	2.08E-01	-9.92E-02	1.12E-01	2.40E-01
1558	GSM592799	GSE24080	Multiple Myeloma	Blood	3.67E-02	-1.44E-01	1.55E-01	2.22E-01
1559	GSM592800	GSE24080	Multiple Myeloma	Blood	2.08E-01	-9.92E-02	1.16E-01	2.38E-01
1560	GSM592801	GSE24080	Multiple Myeloma	Blood	2.21E-01	-9.72E-02	3.23E-01	1.72E-01
1561	GSM592802	GSE24080	Multiple Myeloma	Blood	1.95E-01	-1.01E-01	7.43E-02	2.62E-01
1562	GSM592803	GSE24080	Multiple Myeloma	Blood	7.01E-02	-1.29E-01	8.56E-02	2.54E-01
1563	GSM592804	GSE24080	Multiple Myeloma	Blood	2.28E-01	9.62E-02	3.89E-02	2.92E-01
1564	GSM592805	GSE24080	Multiple Myeloma	Blood	1.77E-01	-1.04E-01	2.51E-01	1.91E-01
1565	GSM592806	GSE24080	Multiple Myeloma	Blood	1.54E-01	-1.08E-01	2.19E-01	2.00E-01
1566	GSM592807	GSE24080	Multiple Myeloma	Blood	2.77E-02	-1.50E-01	6.43E-02	2.69E-01
1567	GSM592808	GSE24080	Multiple Myeloma	Blood	2.40E-02	-1.53E-01	1.55E-01	2.22E-01
1568	GSM592809	GSE24080	Multiple Myeloma	Blood	2.21E-01	-9.72E-02	1.86E-01	2.10E-01
1569	GSM592810	GSE24080	Multiple Myeloma	Blood	5.16E-03	-1.82E-01	1.12E-01	2.40E-01
1570	GSM592811	GSE24080	Multiple Myeloma	Blood	1.44E-01	-1.10E-01	2.39E-01	1.94E-01
1571	GSM592812	GSE24080	Multiple Myeloma	Blood	1.04E-01	-1.19E-01	3.16E-02	3.02E-01
1572	GSM592813	GSE24080	Multiple Myeloma	Blood	2.77E-02	-1.50E-01	2.67E-02	3.09E-01
1573	GSM592814	GSE24080	Multiple Myeloma	Blood	1.01E-02	-1.70E-01	1.10E-01	-2.41E-01
1574	GSM592815	GSE24080	Multiple Myeloma	Blood	1.61E-02	-1.61E-01	5.17E-02	2.79E-01
1575	GSM592816	GSE24080	Multiple Myeloma	Blood	1.89E-01	1.02E-01	1.72E-02	3.27E-01
1576	GSM592817	GSE24080	Multiple Myeloma	Blood	9.26E-02	-1.22E-01	6.27E-01	1.11E-01
1577	GSM592818	GSE24080	Multiple Myeloma	Blood	4.20E-02	-1.41E-01	2.51E-01	1.91E-01
1578	GSM592819	GSE24080	Multiple Myeloma	Blood	1.49E-01	1.09E-01	1.17E-03	4.22E-01
1579	GSM592820	GSE24080	Multiple Myeloma	Blood	1.49E-01	1.09E-01	6.69E-02	2.67E-01
1580	GSM592821	GSE24080	Multiple Myeloma	Blood	9.27E-05	-2.41E-01	3.87E-01	1.58E-01
1581	GSM592822	GSE24080	Multiple Myeloma	Blood	7.60E-02	-1.27E-01	2.67E-02	3.09E-01
1582	GSM592823	GSE24080	Multiple Myeloma	Blood	9.04E-03	-1.72E-01	1.12E-01	2.40E-01
1583	GSM592824	GSE24080	Multiple Myeloma	Blood	2.53E-03	-1.93E-01	1.55E-01	2.22E-01
1584	GSM592825	GSE24080	Multiple Myeloma	Blood	4.01E-02	1.42E-01	2.55E-02	3.11E-01
1585	GSM592826	GSE24080	Multiple Myeloma	Blood	1.46E-02	-1.63E-01	9.82E-02	2.47E-01
1586	GSM592827	GSE24080	Multiple Myeloma	Blood	1.34E-01	-1.12E-01	4.55E-02	2.85E-01
1587	GSM592828	GSE24080	Multiple Myeloma	Blood	2.77E-02	-1.50E-01	8.56E-02	2.54E-01
1588	GSM592829	GSE24080	Multiple Myeloma	Blood	3.86E-03	-1.87E-01	1.96E-01	2.07E-01
1589	GSM592830	GSE24080	Multiple Myeloma	Blood	5.23E-02	1.36E-01	7.70E-03	3.58E-01
1590	GSM592831	GSE24080	Multiple Myeloma	Blood	5.94E-02	-1.33E-01	3.40E-03	3.87E-01
1591	GSM592832	GSE24080	Multiple Myeloma	Blood	1.38E-02	-1.64E-01	2.19E-01	2.00E-01
1592	GSM592833	GSE24080	Multiple Myeloma	Blood	1.00E-01	-1.20E-01	3.16E-02	3.02E-01
1593	GSM592834	GSE24080	Multiple Myeloma	Blood	1.39E-01	-1.11E-01	3.72E-02	2.94E-01
1594	GSM592835	GSE24080	Multiple Myeloma	Blood	4.79E-02	-1.38E-01	1.89E-02	3.23E-01
1595	GSM592836	GSE24080	Multiple Myeloma	Blood	6.08E-04	-2.15E-01	3.30E-02	-3.00E-01
1596	GSM592837	GSE24080	Multiple Myeloma	Blood	4.34E-03	-1.85E-01	1.75E-01	2.14E-01
1597	GSM592838	GSE24080	Multiple Myeloma	Blood	3.05E-02	-1.48E-01	1.02E-01	2.45E-01
1598	GSM592839	GSE24080	Multiple Myeloma	Blood	6.86E-03	-1.77E-01	1.32E-01	2.31E-01
1599	GSM592840	GSE24080	Multiple Myeloma	Blood	1.34E-01	-1.12E-01	3.78E-01	1.60E-01
1600	GSM592841	GSE24080	Multiple Myeloma	Blood	7.30E-02	-1.28E-01	5.04E-01	1.34E-01
1601	GSM592842	GSE24080	Multiple Myeloma	Blood	1.25E-02	-1.66E-01	3.87E-01	1.58E-01
1602	GSM592843	GSE24080	Multiple Myeloma	Blood	1.18E-02	-1.67E-01	6.43E-02	2.69E-01
1603	GSM592844	GSE24080	Multiple Myeloma	Blood	1.70E-02	-1.60E-01	1.32E-01	2.31E-01
1604	GSM592845	GSE24080	Multiple Myeloma	Blood	1.65E-01	-1.06E-01	2.32E-01	1.96E-01
1605	GSM592846	GSE24080	Multiple Myeloma	Blood	1.77E-01	-1.04E-01	1.12E-01	2.40E-01
1606	GSM592847	GSE24080	Multiple Myeloma	Blood	5.69E-02	-1.34E-01	5.54E-02	2.76E-01
1607	GSM592848	GSE24080	Multiple Myeloma	Blood	7.45E-04	-2.12E-01	8.56E-02	2.54E-01
1608	GSM592849	GSE24080	Multiple Myeloma	Blood	4.83E-06	-2.77E-01	1.29E-01	-2.32E-01
1609	GSM592850	GSE24080	Multiple Myeloma	Blood	8.57E-02	-1.24E-01	2.02E-01	2.05E-01
1610	GSM592851	GSE24080	Multiple Myeloma	Blood	1.71E-01	1.05E-01	1.84E-03	4.07E-01
1611	GSM592852	GSE24080	Multiple Myeloma	Blood	1.54E-01	1.08E-01	2.44E-03	3.98E-01
1612	GSM592853	GSE24080	Multiple Myeloma	Blood	1.53E-03	-2.01E-01	5.89E-01	1.18E-01
1613	GSM592854	GSE24080	Multiple Myeloma	Blood	2.62E-04	-2.27E-01	3.16E-02	3.02E-01
1614	GSM592855	GSE24080	Multiple Myeloma	Blood	5.01E-02	-1.37E-01	4.88E-03	3.74E-01
1615	GSM592856	GSE24080	Multiple Myeloma	Blood	4.79E-02	1.38E-01	4.88E-03	3.74E-01
1616	GSM592857	GSE24080	Multiple Myeloma	Blood	3.19E-02	-1.47E-01	1.16E-01	2.38E-01
1617	GSM592858	GSE24080	Multiple Myeloma	Blood	2.24E-03	-1.95E-01	7.24E-02	-2.63E-01
1618	GSM592859	GSE24080	Multiple Myeloma	Blood	2.14E-01	9.82E-02	1.16E-01	2.38E-01
1619	GSM592860	GSE24080	Multiple Myeloma	Blood	3.23E-03	-1.89E-01	2.19E-01	2.00E-01
1620	GSM592861	GSE24080	Multiple Myeloma	Blood	1.16E-01	-1.16E-01	1.75E-01	2.14E-01
1621	GSM592862	GSE24080	Multiple Myeloma	Blood	1.74E-03	-1.99E-01	3.32E-01	-1.70E-01
1622	GSM592863	GSE24080	Multiple Myeloma	Blood	1.21E-01	1.15E-01	3.30E-02	3.00E-01
1623	GSM592864	GSE24080	Multiple Myeloma	Blood	1.35E-03	-2.03E-01	3.72E-02	2.94E-01

1624	GSM592865	GSE24080	Multiple Myeloma	Blood	7.25E-03	-1.76E-01	9.94E-02	-2.47E-01
1625	GSM592866	GSE24080	Multiple Myeloma	Blood	1.08E-01	-1.18E-01	3.30E-02	3.00E-01
1626	GSM592867	GSE24080	Multiple Myeloma	Blood	1.83E-01	-1.03E-01	3.97E-03	3.82E-01
1627	GSM592868	GSE24080	Multiple Myeloma	Blood	7.25E-03	-1.76E-01	1.16E-01	2.38E-01
1628	GSM592869	GSE24080	Multiple Myeloma	Blood	2.44E-04	2.28E-01	6.67E-03	3.63E-01
1629	GSM592870	GSE24080	Multiple Myeloma	Blood	2.08E-01	9.92E-02	6.86E-04	4.38E-01
1630	GSM592871	GSE24080	Multiple Myeloma	Blood	2.18E-02	-1.55E-01	1.02E-01	2.45E-01
1631	GSM592872	GSE24080	Multiple Myeloma	Blood	5.01E-02	-1.37E-01	7.43E-02	2.62E-01
1632	GSM592873	GSE24080	Multiple Myeloma	Blood	4.60E-05	-2.50E-01	1.98E-01	-2.07E-01
1633	GSM592874	GSE24080	Multiple Myeloma	Blood	1.57E-04	-2.34E-01	4.06E-01	1.54E-01
1634	GSM592875	GSE24080	Multiple Myeloma	Blood	1.97E-03	-1.97E-01	1.75E-01	2.14E-01
1635	GSM592876	GSE24080	Multiple Myeloma	Blood	2.91E-02	1.49E-01	9.34E-03	3.51E-01
1636	GSM592877	GSE24080	Multiple Myeloma	Blood	1.79E-02	-1.59E-01	1.75E-01	2.14E-01
1637	GSM592878	GSE24080	Multiple Myeloma	Blood	9.54E-03	-1.71E-01	8.56E-02	2.54E-01
1638	GSM592879	GSE24080	Multiple Myeloma	Blood	8.91E-02	1.23E-01	1.50E-02	3.32E-01
1639	GSM592880	GSE24080	Multiple Myeloma	Blood	5.01E-02	-1.37E-01	2.86E-01	1.82E-01
1640	GSM592881	GSE24080	Multiple Myeloma	Blood	2.08E-01	-9.92E-02	1.43E-02	3.34E-01
1641	GSM592882	GSE24080	Multiple Myeloma	Blood	1.18E-02	-1.67E-01	1.76E-01	-2.14E-01
1642	GSM592883	GSE24080	Multiple Myeloma	Blood	2.69E-03	-1.92E-01	6.17E-02	2.71E-01
1643	GSM592884	GSE24080	Multiple Myeloma	Blood	5.79E-03	-1.80E-01	6.17E-02	2.71E-01
1644	GSM592885	GSE24080	Multiple Myeloma	Blood	5.46E-02	1.35E-01	3.97E-03	3.82E-01
1645	GSM592886	GSE24080	Multiple Myeloma	Blood	5.69E-02	-1.34E-01	9.85E-03	3.49E-01
1646	GSM592887	GSE24080	Multiple Myeloma	Blood	2.86E-03	-1.91E-01	1.32E-01	2.31E-01
1647	GSM592888	GSE24080	Multiple Myeloma	Blood	1.08E-01	-1.18E-01	1.47E-03	4.14E-01
1648	GSM592889	GSE24080	Multiple Myeloma	Blood	7.92E-02	-1.26E-01	1.50E-02	3.32E-01
1649	GSM592890	GSE24080	Multiple Myeloma	Blood	3.67E-02	1.44E-01	6.86E-04	4.38E-01
1650	GSM592891	GSE24080	Multiple Myeloma	Blood	3.67E-02	-1.44E-01	2.67E-02	3.09E-01
1651	GSM592892	GSE24080	Multiple Myeloma	Blood	2.18E-02	-1.55E-01	2.21E-01	-1.99E-01
1652	GSM592893	GSE24080	Multiple Myeloma	Blood	7.97E-04	-2.11E-01	1.75E-01	2.14E-01
1653	GSM592894	GSE24080	Multiple Myeloma	Blood	1.12E-01	-1.17E-01	5.54E-02	2.76E-01
1654	GSM592895	GSE24080	Multiple Myeloma	Blood	5.94E-02	-1.33E-01	2.19E-01	2.00E-01
1655	GSM592896	GSE24080	Multiple Myeloma	Blood	1.21E-01	-1.15E-01	1.19E-02	3.42E-01
1656	GSM592897	GSE24080	Multiple Myeloma	Blood	1.18E-02	-1.67E-01	1.75E-01	2.14E-01
1657	GSM592898	GSE24080	Multiple Myeloma	Blood	3.63E-03	-1.88E-01	1.55E-01	2.22E-01
1658	GSM592899	GSE24080	Multiple Myeloma	Blood	1.44E-03	-2.02E-01	2.19E-01	2.00E-01
1659	GSM592900	GSE24080	Multiple Myeloma	Blood	2.69E-03	-1.92E-01	1.55E-01	2.22E-01
1660	GSM592901	GSE24080	Multiple Myeloma	Blood	2.28E-01	-9.62E-02	7.73E-02	2.60E-01
1661	GSM592902	GSE24080	Multiple Myeloma	Blood	2.07E-02	-1.56E-01	6.17E-02	2.71E-01
1662	GSM592903	GSE24080	Multiple Myeloma	Blood	5.91E-07	-3.00E-01	1.92E-01	-2.09E-01
1663	GSM592904	GSE24080	Multiple Myeloma	Blood	1.89E-01	1.02E-01	1.21E-01	2.36E-01
1664	GSM592905	GSE24080	Multiple Myeloma	Blood	6.97E-04	-2.13E-01	6.69E-02	2.67E-01
1665	GSM592906	GSE24080	Multiple Myeloma	Blood	7.01E-02	-1.29E-01	9.85E-03	3.49E-01
1666	GSM592907	GSE24080	Multiple Myeloma	Blood	3.84E-02	-1.43E-01	1.55E-01	2.22E-01
1667	GSM592908	GSE24080	Multiple Myeloma	Blood	8.24E-02	-1.25E-01	5.04E-01	1.34E-01
1668	GSM592909	GSE24080	Multiple Myeloma	Blood	4.59E-02	-1.39E-01	1.75E-01	2.14E-01
1669	GSM592910	GSE24080	Multiple Myeloma	Blood	2.35E-01	9.52E-02	3.97E-03	3.82E-01
1670	GSM592911	GSE24080	Multiple Myeloma	Blood	2.18E-02	-1.55E-01	2.19E-01	2.00E-01
1671	GSM592912	GSE24080	Multiple Myeloma	Blood	3.50E-02	1.45E-01	6.17E-02	2.71E-01
1672	GSM592913	GSE24080	Multiple Myeloma	Blood	1.12E-02	-1.68E-01	4.06E-02	2.90E-01
1673	GSM592914	GSE24080	Multiple Myeloma	Blood	4.39E-02	-1.40E-01	3.72E-02	2.94E-01
1674	GSM592915	GSE24080	Multiple Myeloma	Blood	6.51E-04	-2.14E-01	6.43E-02	2.69E-01
1675	GSM592916	GSE24080	Multiple Myeloma	Blood	9.63E-02	-1.21E-01	8.56E-02	2.54E-01
1676	GSM592917	GSE24080	Multiple Myeloma	Blood	4.59E-02	-1.39E-01	2.26E-01	1.98E-01
1677	GSM592918	GSE24080	Multiple Myeloma	Blood	2.53E-03	-1.93E-01	6.17E-02	2.71E-01
1678	GSM592919	GSE24080	Multiple Myeloma	Blood	4.09E-03	-1.86E-01	3.87E-01	1.58E-01
1679	GSM592920	GSE24080	Multiple Myeloma	Blood	7.25E-03	-1.76E-01	1.21E-01	2.36E-01
1680	GSM592921	GSE24080	Multiple Myeloma	Blood	1.18E-02	-1.67E-01	9.27E-04	4.29E-01
1681	GSM592922	GSE24080	Multiple Myeloma	Blood	1.21E-01	1.15E-01	1.32E-01	2.31E-01
1682	GSM592923	GSE24080	Multiple Myeloma	Blood	1.18E-03	-2.05E-01	9.94E-02	-2.47E-01
1683	GSM592924	GSE24080	Multiple Myeloma	Blood	1.60E-01	-1.07E-01	7.70E-03	3.58E-01
1684	GSM592925	GSE24080	Multiple Myeloma	Blood	3.84E-02	1.43E-01	9.34E-03	3.51E-01
1685	GSM592926	GSE24080	Multiple Myeloma	Blood	4.60E-03	-1.84E-01	7.73E-02	2.60E-01
1686	GSM592927	GSE24080	Multiple Myeloma	Blood	1.77E-01	-1.04E-01	4.88E-03	3.74E-01
1687	GSM592928	GSE24080	Multiple Myeloma	Blood	7.01E-02	-1.29E-01	1.12E-01	2.40E-01
1688	GSM592929	GSE24080	Multiple Myeloma	Blood	6.48E-03	-1.78E-01	4.36E-02	2.87E-01
1689	GSM592930	GSE24080	Multiple Myeloma	Blood	4.31E-04	-2.20E-01	6.01E-02	-2.72E-01
1690	GSM592931	GSE24080	Multiple Myeloma	Blood	7.92E-02	-1.26E-01	1.16E-01	2.38E-01
1691	GSM592932	GSE24080	Multiple Myeloma	Blood	2.72E-01	-9.03E-02	9.82E-02	2.47E-01
1692	GSM592933	GSE24080	Multiple Myeloma	Blood	1.65E-01	-1.06E-01	1.55E-01	2.22E-01
1693	GSM592934	GSE24080	Multiple Myeloma	Blood	2.72E-01	9.03E-02	1.04E-02	3.47E-01
1694	GSM592935	GSE24080	Multiple Myeloma	Blood	3.23E-03	-1.89E-01	2.02E-01	2.05E-01
1695	GSM592936	GSE24080	Multiple Myeloma	Blood	4.20E-02	-1.41E-01	6.69E-02	2.67E-01
1696	GSM592937	GSE24080	Multiple Myeloma	Blood	1.88E-02	-1.58E-01	1.16E-01	2.38E-01
1697	GSM592938	GSE24080	Multiple Myeloma	Blood	3.42E-03	-1.88E-01	8.89E-02	2.52E-01

1698	GSM592939	GSE24080	Multiple Myeloma	Blood	2.40E-02	-1.53E-01	8.56E-02	2.54E-01
1699	GSM592940	GSE24080	Multiple Myeloma	Blood	4.09E-03	-1.86E-01	1.96E-01	2.07E-01
1700	GSM592941	GSE24080	Multiple Myeloma	Blood	6.46E-02	-1.31E-01	5.77E-02	2.74E-01
1701	GSM592942	GSE24080	Multiple Myeloma	Blood	9.73E-04	-2.08E-01	1.75E-01	2.14E-01
1702	GSM592943	GSE24080	Multiple Myeloma	Blood	1.34E-01	-1.12E-01	1.96E-01	2.07E-01
1703	GSM592944	GSE24080	Multiple Myeloma	Blood	9.11E-04	-2.09E-01	1.16E-01	2.38E-01
1704	GSM592945	GSE24080	Multiple Myeloma	Blood	9.04E-03	-1.72E-01	1.75E-01	2.14E-01
1705	GSM592946	GSE24080	Multiple Myeloma	Blood	6.28E-06	-2.74E-01	6.01E-02	-2.72E-01
1706	GSM592947	GSE24080	Multiple Myeloma	Blood	1.46E-02	1.63E-01	1.96E-03	4.05E-01
1707	GSM592948	GSE24080	Multiple Myeloma	Blood	4.01E-02	-1.42E-01	3.16E-02	3.02E-01
1708	GSM592949	GSE24080	Multiple Myeloma	Blood	1.46E-02	1.63E-01	9.85E-03	3.49E-01
1709	GSM38162	GSE2113	Multiple Myeloma	Blood	2.40E-02	-1.53E-01	1.21E-01	2.36E-01
1710	GSM38163	GSE2113	Multiple Myeloma	Blood	1.89E-01	-1.02E-01	1.96E-03	4.05E-01
1711	GSM38164	GSE2113	Multiple Myeloma	Blood	1.97E-02	-1.57E-01	4.41E-01	-1.47E-01
1712	GSM38165	GSE2113	Multiple Myeloma	Blood	1.61E-02	-1.61E-01	2.15E-01	-2.01E-01
1713	GSM38166	GSE2113	Multiple Myeloma	Blood	1.00E-01	1.20E-01	7.43E-02	2.62E-01
1714	GSM38167	GSE2113	Multiple Myeloma	Blood	6.73E-02	-1.30E-01	1.12E-01	2.40E-01
1715	GSM38168	GSE2113	Multiple Myeloma	Blood	4.01E-02	-1.42E-01	4.55E-02	2.85E-01
1716	GSM38169	GSE2113	Multiple Myeloma	Blood	7.67E-03	-1.75E-01	6.10E-01	-1.14E-01
1717	GSM38170	GSE2113	Multiple Myeloma	Blood	1.97E-02	-1.57E-01	1.75E-01	2.14E-01
1718	GSM38171	GSE2113	Multiple Myeloma	Blood	7.60E-02	-1.27E-01	1.75E-01	2.14E-01
1719	GSM38172	GSE2113	Multiple Myeloma	Blood	2.64E-02	-1.51E-01	1.75E-01	2.14E-01
1720	GSM38173	GSE2113	Multiple Myeloma	Blood	3.05E-02	-1.48E-01	1.12E-01	2.40E-01
1721	GSM38174	GSE2113	Multiple Myeloma	Blood	5.94E-02	-1.33E-01	6.96E-02	2.65E-01
1722	GSM38175	GSE2113	Multiple Myeloma	Blood	4.79E-02	-1.38E-01	6.17E-02	2.71E-01
1723	GSM38176	GSE2113	Multiple Myeloma	Blood	1.49E-01	-1.09E-01	6.43E-02	2.69E-01
1724	GSM38177	GSE2113	Multiple Myeloma	Blood	9.54E-03	-1.71E-01	5.31E-02	2.78E-01
1725	GSM38178	GSE2113	Multiple Myeloma	Blood	7.92E-02	-1.26E-01	1.75E-01	2.14E-01
1726	GSM38179	GSE2113	Multiple Myeloma	Blood	1.46E-02	-1.63E-01	4.36E-02	2.87E-01
1727	GSM38180	GSE2113	Multiple Myeloma	Blood	4.60E-03	-1.84E-01	9.82E-02	2.47E-01
1728	GSM38181	GSE2113	Multiple Myeloma	Blood	1.63E-03	-2.00E-01	2.51E-01	1.91E-01
1729	GSM38182	GSE2113	Multiple Myeloma	Blood	1.12E-02	-1.68E-01	2.19E-01	2.00E-01
1730	GSM38183	GSE2113	Multiple Myeloma	Blood	5.01E-02	1.37E-01	5.98E-03	3.67E-01
1731	GSM38184	GSE2113	Multiple Myeloma	Blood	1.25E-01	1.14E-01	4.75E-02	2.83E-01
1732	GSM38185	GSE2113	Multiple Myeloma	Blood	1.04E-01	-1.19E-01	7.43E-02	2.62E-01
1733	GSM38186	GSE2113	Multiple Myeloma	Blood	1.77E-01	-1.04E-01	5.98E-03	3.67E-01
1734	GSM38187	GSE2113	Multiple Myeloma	Blood	2.27E-04	-2.29E-01	1.96E-01	2.07E-01
1735	GSM38188	GSE2113	Multiple Myeloma	Blood	7.67E-03	-1.75E-01	2.46E-01	-1.92E-01
1736	GSM38189	GSE2113	Multiple Myeloma	Blood	1.18E-02	-1.67E-01	1.96E-01	2.07E-01
1737	GSM38190	GSE2113	Multiple Myeloma	Blood	1.77E-01	1.04E-01	5.31E-02	2.78E-01
1738	GSM38191	GSE2113	Multiple Myeloma	Blood	2.29E-02	-1.54E-01	1.37E-01	2.29E-01
1739	GSM38192	GSE2113	Multiple Myeloma	Blood	1.65E-01	1.06E-01	3.03E-03	3.91E-01
1740	GSM38193	GSE2113	Multiple Myeloma	Blood	1.21E-01	-1.15E-01	1.16E-01	2.38E-01
1741	GSM38194	GSE2113	Multiple Myeloma	Blood	1.16E-01	-1.16E-01	4.55E-02	2.85E-01
1742	GSM38195	GSE2113	Multiple Myeloma	Blood	4.09E-03	-1.86E-01	5.31E-02	2.78E-01
1743	GSM38196	GSE2113	Multiple Myeloma	Blood	1.30E-01	-1.13E-01	5.16E-03	3.72E-01
1744	GSM38197	GSE2113	Multiple Myeloma	Blood	5.46E-02	-1.35E-01	5.54E-02	2.76E-01
1745	GSM38198	GSE2113	Multiple Myeloma	Blood	1.06E-02	-1.69E-01	1.16E-01	2.38E-01
1746	GSM38199	GSE2113	Multiple Myeloma	Blood	3.50E-02	-1.45E-01	1.12E-01	2.40E-01
1747	GSM38200	GSE2113	Multiple Myeloma	Blood	2.29E-02	-1.54E-01	3.15E-01	1.74E-01
1748	GSM658418	GSE26760	Multiple Myeloma	Blood	1.70E-02	-1.60E-01	6.17E-02	2.71E-01
1749	GSM658419	GSE26760	Multiple Myeloma	Blood	3.19E-02	-1.47E-01	8.56E-02	2.54E-01
1750	GSM658420	GSE26760	Multiple Myeloma	Blood	1.04E-03	-2.07E-01	1.16E-01	2.38E-01
1751	GSM658421	GSE26760	Multiple Myeloma	Blood	7.60E-02	-1.27E-01	8.89E-02	2.52E-01
1752	GSM658422	GSE26760	Multiple Myeloma	Blood	7.01E-02	-1.29E-01	1.55E-01	2.22E-01
1753	GSM658423	GSE26760	Multiple Myeloma	Blood	2.40E-02	-1.53E-01	9.82E-02	2.47E-01
1754	GSM658424	GSE26760	Multiple Myeloma	Blood	2.77E-02	-1.50E-01	3.30E-02	3.00E-01
1755	GSM658425	GSE26760	Multiple Myeloma	Blood	4.25E-05	-2.51E-01	2.46E-01	-1.92E-01
1756	GSM658426	GSE26760	Multiple Myeloma	Blood	1.44E-01	-1.10E-01	2.19E-01	2.00E-01
1757	GSM658427	GSE26760	Multiple Myeloma	Blood	3.47E-01	8.13E-02	6.43E-02	2.69E-01
1758	GSM658428	GSE26760	Multiple Myeloma	Blood	1.61E-02	-1.61E-01	6.31E-03	3.65E-01
1759	GSM658429	GSE26760	Multiple Myeloma	Blood	1.35E-04	-2.36E-01	1.86E-01	-2.10E-01
1760	GSM658430	GSE26760	Multiple Myeloma	Blood	3.05E-02	-1.48E-01	8.56E-02	2.54E-01
1761	GSM658431	GSE26760	Multiple Myeloma	Blood	2.80E-01	8.93E-02	1.02E-01	2.45E-01
1762	GSM658432	GSE26760	Multiple Myeloma	Blood	1.31E-02	1.65E-01	9.89E-04	4.27E-01
1763	GSM658433	GSE26760	Multiple Myeloma	Blood	5.23E-02	-1.36E-01	1.16E-01	2.38E-01
1764	GSM658434	GSE26760	Multiple Myeloma	Blood	6.19E-02	-1.32E-01	8.56E-02	2.54E-01
1765	GSM658435	GSE26760	Multiple Myeloma	Blood	5.16E-03	-1.82E-01	1.86E-01	2.10E-01
1766	GSM658436	GSE26760	Multiple Myeloma	Blood	3.13E-01	8.53E-02	3.97E-03	3.82E-01
1767	GSM658437	GSE26760	Multiple Myeloma	Blood	4.79E-02	-1.38E-01	2.51E-01	1.91E-01
1768	GSM658438	GSE26760	Multiple Myeloma	Blood	7.25E-03	-1.76E-01	3.46E-01	1.67E-01
1769	GSM658439	GSE26760	Multiple Myeloma	Blood	2.29E-02	-1.54E-01	3.78E-01	1.60E-01
1770	GSM658440	GSE26760	Multiple Myeloma	Blood	7.92E-02	1.26E-01	1.25E-02	3.40E-01
1771	GSM658441	GSE26760	Multiple Myeloma	Blood	1.01E-02	-1.70E-01	5.31E-02	2.78E-01

1772	GSM658442	GSE26760	Multiple Myeloma	Blood	5.23E-02	-1.36E-01	3.46E-01	1.67E-01
1773	GSM658443	GSE26760	Multiple Myeloma	Blood	8.10E-03	-1.74E-01	9.82E-02	2.47E-01
1774	GSM658444	GSE26760	Multiple Myeloma	Blood	1.35E-03	-2.03E-01	1.14E-01	-2.39E-01
1775	GSM658445	GSE26760	Multiple Myeloma	Blood	3.04E-01	-8.63E-02	7.70E-03	3.58E-01
1776	GSM658446	GSE26760	Multiple Myeloma	Blood	6.86E-03	-1.77E-01	1.80E-01	2.12E-01
1777	GSM658447	GSE26760	Multiple Myeloma	Blood	1.21E-01	1.15E-01	9.34E-03	3.51E-01
1778	GSM658448	GSE26760	Multiple Myeloma	Blood	1.16E-01	1.16E-01	2.44E-03	3.98E-01
1779	GSM658449	GSE26760	Multiple Myeloma	Blood	7.30E-02	-1.28E-01	3.46E-01	1.67E-01
1780	GSM658450	GSE26760	Multiple Myeloma	Blood	8.56E-03	-1.73E-01	6.17E-02	2.71E-01
1781	GSM658451	GSE26760	Multiple Myeloma	Blood	1.11E-03	-2.06E-01	2.46E-01	-1.92E-01
1782	GSM658452	GSE26760	Multiple Myeloma	Blood	4.87E-03	-1.83E-01	2.46E-01	-1.92E-01
1783	GSM658453	GSE26760	Multiple Myeloma	Blood	6.84E-06	-2.73E-01	9.94E-02	-2.47E-01
1784	GSM658454	GSE26760	Multiple Myeloma	Blood	2.49E-01	-9.33E-02	3.16E-02	3.02E-01
1785	GSM658455	GSE26760	Multiple Myeloma	Blood	7.92E-02	-1.26E-01	6.69E-02	2.67E-01
1786	GSM658456	GSE26760	Multiple Myeloma	Blood	7.25E-03	-1.76E-01	1.55E-01	2.22E-01
1787	GSM658457	GSE26760	Multiple Myeloma	Blood	5.46E-02	-1.35E-01	2.15E-02	3.18E-01
1788	GSM658458	GSE26760	Multiple Myeloma	Blood	3.50E-02	-1.45E-01	2.73E-01	-1.85E-01
1789	GSM658459	GSE26760	Multiple Myeloma	Blood	8.91E-02	-1.23E-01	3.89E-02	2.92E-01
1790	GSM658460	GSE26760	Multiple Myeloma	Blood	3.86E-03	-1.87E-01	3.15E-01	1.74E-01
1791	GSM658461	GSE26760	Multiple Myeloma	Blood	1.12E-01	-1.17E-01	3.16E-02	3.02E-01
1792	GSM658462	GSE26760	Multiple Myeloma	Blood	8.57E-02	-1.24E-01	2.15E-02	3.18E-01
1793	GSM658463	GSE26760	Multiple Myeloma	Blood	1.77E-01	1.04E-01	1.57E-03	4.12E-01
1794	GSM658464	GSE26760	Multiple Myeloma	Blood	3.34E-02	-1.46E-01	1.89E-02	3.23E-01
1795	GSM658465	GSE26760	Multiple Myeloma	Blood	7.25E-03	-1.76E-01	1.75E-01	2.14E-01
1796	GSM658466	GSE26760	Multiple Myeloma	Blood	1.97E-03	-1.97E-01	2.02E-01	2.05E-01
1797	GSM658467	GSE26760	Multiple Myeloma	Blood	1.12E-01	-1.17E-01	7.43E-02	2.62E-01
1798	GSM658468	GSE26760	Multiple Myeloma	Blood	1.26E-03	-2.04E-01	2.08E-01	2.03E-01
1799	GSM658469	GSE26760	Multiple Myeloma	Blood	3.34E-02	-1.46E-01	2.08E-01	2.03E-01
1800	GSM658470	GSE26760	Multiple Myeloma	Blood	2.49E-01	9.33E-02	4.88E-03	3.74E-01
1801	GSM658471	GSE26760	Multiple Myeloma	Blood	2.53E-03	-1.93E-01	3.72E-02	2.94E-01
1802	GSM658472	GSE26760	Multiple Myeloma	Blood	1.77E-01	1.04E-01	1.43E-02	3.34E-01
1803	GSM658473	GSE26760	Multiple Myeloma	Blood	1.08E-01	1.18E-01	8.56E-02	2.54E-01
1804	GSM658474	GSE26760	Multiple Myeloma	Blood	2.52E-02	-1.52E-01	5.31E-02	2.78E-01
1805	GSM658475	GSE26760	Multiple Myeloma	Blood	7.36E-05	-2.44E-01	1.57E-01	-2.21E-01
1806	GSM658476	GSE26760	Multiple Myeloma	Blood	1.04E-03	-2.07E-01	3.15E-01	1.74E-01
1807	GSM658477	GSE26760	Multiple Myeloma	Blood	7.30E-02	-1.28E-01	7.73E-02	2.60E-01
1808	GSM658478	GSE26760	Multiple Myeloma	Blood	1.65E-01	-1.06E-01	6.17E-02	2.71E-01
1809	GSM658479	GSE26760	Multiple Myeloma	Blood	1.01E-02	-1.70E-01	2.51E-01	1.91E-01
1810	GSM658480	GSE26760	Multiple Myeloma	Blood	1.49E-01	1.09E-01	1.80E-01	2.12E-01
1811	GSM658481	GSE26760	Multiple Myeloma	Blood	1.46E-04	-2.35E-01	1.80E-01	2.12E-01
1812	GSM658482	GSE26760	Multiple Myeloma	Blood	2.14E-01	-9.82E-02	8.12E-03	3.56E-01
1813	GSM658483	GSE26760	Multiple Myeloma	Blood	2.64E-02	-1.51E-01	1.19E-02	3.42E-01
1814	GSM658484	GSE26760	Multiple Myeloma	Blood	4.31E-04	-2.20E-01	2.19E-01	2.00E-01
1815	GSM658485	GSE26760	Multiple Myeloma	Blood	6.46E-02	-1.31E-01	2.19E-01	2.00E-01
1816	GSM658486	GSE26760	Multiple Myeloma	Blood	3.05E-02	-1.48E-01	7.43E-02	2.62E-01
1817	GSM658487	GSE26760	Multiple Myeloma	Blood	2.86E-03	-1.91E-01	3.10E-01	-1.76E-01
1818	GSM658488	GSE26760	Multiple Myeloma	Blood	7.01E-02	-1.29E-01	3.16E-02	3.02E-01
1819	GSM658489	GSE26760	Multiple Myeloma	Blood	2.29E-02	-1.54E-01	1.19E-02	3.42E-01
1820	GSM658490	GSE26760	Multiple Myeloma	Blood	3.23E-03	-1.89E-01	5.31E-02	2.78E-01
1821	GSM658491	GSE26760	Multiple Myeloma	Blood	1.16E-01	-1.16E-01	3.55E-01	1.65E-01
1822	GSM658492	GSE26760	Multiple Myeloma	Blood	2.07E-02	-1.56E-01	1.72E-02	3.27E-01
1823	GSM658493	GSE26760	Multiple Myeloma	Blood	3.94E-01	-7.64E-02	3.89E-02	2.92E-01
1824	GSM658494	GSE26760	Multiple Myeloma	Blood	2.07E-02	-1.56E-01	1.12E-01	2.40E-01
1825	GSM658495	GSE26760	Multiple Myeloma	Blood	1.83E-01	-1.03E-01	8.56E-02	2.54E-01
1826	GSM658496	GSE26760	Multiple Myeloma	Blood	1.97E-02	-1.57E-01	2.86E-01	1.82E-01
1827	GSM658497	GSE26760	Multiple Myeloma	Blood	3.23E-03	-1.89E-01	2.15E-01	-2.01E-01
1828	GSM658498	GSE26760	Multiple Myeloma	Blood	1.31E-02	-1.65E-01	3.10E-01	-1.76E-01
1829	GSM658499	GSE26760	Multiple Myeloma	Blood	2.91E-02	-1.49E-01	3.15E-01	1.74E-01
1830	GSM658500	GSE26760	Multiple Myeloma	Blood	4.59E-02	-1.39E-01	1.16E-01	2.38E-01
1831	GSM658501	GSE26760	Multiple Myeloma	Blood	6.73E-02	-1.30E-01	1.80E-02	3.25E-01
1832	GSM658502	GSE26760	Multiple Myeloma	Blood	9.26E-02	-1.22E-01	3.49E-01	1.67E-01
1833	GSM658503	GSE26760	Multiple Myeloma	Blood	1.44E-03	-2.02E-01	1.60E-01	2.20E-01
1834	GSM658504	GSE26760	Multiple Myeloma	Blood	1.08E-01	1.18E-01	4.88E-03	3.74E-01
1835	GSM658505	GSE26760	Multiple Myeloma	Blood	5.69E-02	-1.34E-01	1.80E-01	2.12E-01
1836	GSM658506	GSE26760	Multiple Myeloma	Blood	2.14E-01	9.82E-02	3.89E-02	2.92E-01
1837	GSM658507	GSE26760	Multiple Myeloma	Blood	6.13E-03	-1.79E-01	1.43E-02	3.34E-01
1838	GSM658508	GSE26760	Multiple Myeloma	Blood	2.52E-02	-1.52E-01	7.43E-02	2.62E-01
1839	GSM658509	GSE26760	Multiple Myeloma	Blood	1.53E-02	-1.62E-01	1.75E-01	2.14E-01
1840	GSM658510	GSE26760	Multiple Myeloma	Blood	3.19E-02	-1.47E-01	1.32E-01	2.31E-01
1841	GSM658511	GSE26760	Multiple Myeloma	Blood	1.08E-01	1.18E-01	2.59E-03	3.96E-01
1842	GSM658512	GSE26760	Multiple Myeloma	Blood	5.69E-02	1.34E-01	3.89E-02	2.92E-01
1843	GSM658513	GSE26760	Multiple Myeloma	Blood	6.46E-02	1.31E-01	8.12E-03	3.56E-01
1844	GSM658514	GSE26760	Multiple Myeloma	Blood	1.25E-01	-1.14E-01	2.67E-02	3.09E-01
1845	GSM658515	GSE26760	Multiple Myeloma	Blood	3.50E-02	1.45E-01	1.25E-02	3.40E-01

1846	GSM658516	GSE26760	Multiple Myeloma	Blood	7.60E-02	-1.27E-01	3.97E-03	3.82E-01
1847	GSM658517	GSE26760	Multiple Myeloma	Blood	6.86E-03	1.77E-01	6.86E-04	4.38E-01
1848	GSM658518	GSE26760	Multiple Myeloma	Blood	7.01E-02	1.29E-01	5.10E-02	2.80E-01
1849	GSM658519	GSE26760	Multiple Myeloma	Blood	1.04E-01	-1.19E-01	1.96E-01	2.07E-01
1850	GSM658520	GSE26760	Multiple Myeloma	Blood	1.06E-02	1.69E-01	6.86E-04	4.38E-01
1851	GSM658521	GSE26760	Multiple Myeloma	Blood	5.79E-03	-1.80E-01	2.53E-01	-1.90E-01
1852	GSM658522	GSE26760	Multiple Myeloma	Blood	1.34E-01	-1.12E-01	7.29E-03	3.60E-01
1853	GSM658523	GSE26760	Multiple Myeloma	Blood	2.38E-03	-1.94E-01	1.02E-01	2.45E-01
1854	GSM658524	GSE26760	Multiple Myeloma	Blood	6.48E-03	-1.78E-01	2.08E-01	-2.03E-01
1855	GSM658525	GSE26760	Multiple Myeloma	Blood	7.30E-02	-1.28E-01	2.19E-01	2.00E-01
1856	GSM658526	GSE26760	Multiple Myeloma	Blood	3.84E-02	-1.43E-01	1.02E-01	2.45E-01
1857	GSM658527	GSE26760	Multiple Myeloma	Blood	2.21E-01	9.72E-02	3.97E-03	3.82E-01
1858	GSM658528	GSE26760	Multiple Myeloma	Blood	2.28E-01	9.62E-02	2.55E-02	3.11E-01
1859	GSM658529	GSE26760	Multiple Myeloma	Blood	9.73E-04	-2.08E-01	1.37E-01	2.29E-01
1860	GSM658530	GSE26760	Multiple Myeloma	Blood	2.07E-02	-1.56E-01	2.51E-01	1.91E-01
1861	GSM658531	GSE26760	Multiple Myeloma	Blood	1.04E-01	1.19E-01	8.56E-02	2.54E-01
1862	GSM658532	GSE26760	Multiple Myeloma	Blood	3.86E-03	-1.87E-01	2.26E-01	1.98E-01
1863	GSM658533	GSE26760	Multiple Myeloma	Blood	5.01E-02	-1.37E-01	3.46E-01	1.67E-01
1864	GSM658534	GSE26760	Multiple Myeloma	Blood	2.72E-01	-9.03E-02	3.40E-03	3.87E-01
1865	GSM658535	GSE26760	Multiple Myeloma	Blood	2.77E-02	-1.50E-01	3.15E-01	1.74E-01
1866	GSM658536	GSE26760	Multiple Myeloma	Blood	1.54E-01	-1.08E-01	1.19E-02	3.42E-01
1867	GSM658537	GSE26760	Multiple Myeloma	Blood	3.84E-01	7.74E-02	2.15E-02	3.18E-01
1868	GSM658538	GSE26760	Multiple Myeloma	Blood	4.59E-02	-1.39E-01	5.10E-02	2.80E-01
1869	GSM658539	GSE26760	Multiple Myeloma	Blood	1.77E-01	-1.04E-01	1.80E-02	3.25E-01
1870	GSM658540	GSE26760	Multiple Myeloma	Blood	2.10E-03	-1.96E-01	8.56E-02	2.54E-01
1871	GSM658541	GSE26760	Multiple Myeloma	Blood	4.13E-01	-7.44E-02	1.98E-02	3.21E-01
1872	GSM658542	GSE26760	Multiple Myeloma	Blood	1.44E-03	-2.02E-01	3.26E-01	-1.72E-01
1873	GSM658543	GSE26760	Multiple Myeloma	Blood	2.72E-01	9.03E-02	3.16E-02	3.02E-01
1874	GSM658544	GSE26760	Multiple Myeloma	Blood	2.11E-04	-2.30E-01	6.69E-02	-2.67E-01
1875	GSM658545	GSE26760	Multiple Myeloma	Blood	8.91E-02	-1.23E-01	2.55E-02	3.11E-01
1876	GSM658546	GSE26760	Multiple Myeloma	Blood	9.63E-02	-1.21E-01	1.21E-01	2.36E-01
1877	GSM658547	GSE26760	Multiple Myeloma	Blood	1.12E-01	-1.17E-01	6.43E-02	2.69E-01
1878	GSM658548	GSE26760	Multiple Myeloma	Blood	3.84E-01	-7.74E-02	4.36E-02	2.87E-01
1879	GSM658549	GSE26760	Multiple Myeloma	Blood	9.27E-05	-2.41E-01	2.19E-01	2.00E-01
1880	GSM658550	GSE26760	Multiple Myeloma	Blood	2.49E-01	9.33E-02	1.02E-01	2.45E-01
1881	GSM658551	GSE26760	Multiple Myeloma	Blood	5.23E-02	-1.36E-01	5.31E-02	2.78E-01
1882	GSM658552	GSE26760	Multiple Myeloma	Blood	2.21E-01	-9.72E-02	3.72E-02	2.94E-01
1883	GSM658553	GSE26760	Multiple Myeloma	Blood	7.92E-02	-1.26E-01	5.10E-02	2.80E-01
1884	GSM658554	GSE26760	Multiple Myeloma	Blood	4.79E-02	-1.38E-01	1.02E-01	2.45E-01
1885	GSM658555	GSE26760	Multiple Myeloma	Blood	8.52E-04	-2.10E-01	3.46E-01	1.67E-01
1886	GSM658556	GSE26760	Multiple Myeloma	Blood	2.14E-01	9.82E-02	1.19E-02	3.42E-01
1887	GSM658557	GSE26760	Multiple Myeloma	Blood	2.77E-02	-1.50E-01	5.31E-02	2.78E-01
1888	GSM658558	GSE26760	Multiple Myeloma	Blood	1.79E-02	-1.59E-01	2.02E-01	2.05E-01
1889	GSM658559	GSE26760	Multiple Myeloma	Blood	1.95E-01	1.01E-01	1.04E-02	3.47E-01
1890	GSM658560	GSE26760	Multiple Myeloma	Blood	4.31E-04	-2.20E-01	3.18E-01	-1.74E-01
1891	GSM658561	GSE26760	Multiple Myeloma	Blood	1.54E-01	1.08E-01	6.17E-02	2.71E-01
1892	GSM658562	GSE26760	Multiple Myeloma	Blood	2.44E-04	-2.28E-01	8.89E-02	-2.52E-01
1893	GSM658563	GSE26760	Multiple Myeloma	Blood	1.65E-01	-1.06E-01	1.46E-01	2.25E-01
1894	GSM658564	GSE26760	Multiple Myeloma	Blood	6.86E-03	-1.77E-01	1.96E-01	2.07E-01
1895	GSM658565	GSE26760	Multiple Myeloma	Blood	4.60E-03	-1.84E-01	1.80E-01	-2.12E-01
1896	GSM658566	GSE26760	Multiple Myeloma	Blood	3.67E-02	1.44E-01	7.81E-04	4.34E-01
1897	GSM658567	GSE26760	Multiple Myeloma	Blood	8.91E-02	-1.23E-01	1.37E-01	2.29E-01
1898	GSM658568	GSE26760	Multiple Myeloma	Blood	1.77E-01	-1.04E-01	1.86E-01	2.10E-01
1899	GSM658569	GSE26760	Multiple Myeloma	Blood	1.25E-02	-1.66E-01	1.42E-01	2.27E-01
1900	GSM658570	GSE26760	Multiple Myeloma	Blood	2.80E-01	-8.93E-02	1.10E-01	2.41E-01
1901	GSM658571	GSE26760	Multiple Myeloma	Blood	8.57E-02	-1.24E-01	4.55E-02	2.85E-01
1902	GSM658572	GSE26760	Multiple Myeloma	Blood	5.79E-03	-1.80E-01	5.31E-02	2.78E-01
1903	GSM658573	GSE26760	Multiple Myeloma	Blood	6.46E-02	-1.31E-01	3.72E-01	1.61E-01
1904	GSM658574	GSE26760	Multiple Myeloma	Blood	3.19E-02	-1.47E-01	1.16E-01	2.38E-01
1905	GSM658575	GSE26760	Multiple Myeloma	Blood	4.39E-02	-1.40E-01	7.43E-02	2.62E-01
1906	GSM658576	GSE26760	Multiple Myeloma	Blood	3.34E-02	-1.46E-01	2.28E-01	-1.97E-01
1907	GSM658577	GSE26760	Multiple Myeloma	Blood	8.59E-05	-2.42E-01	3.61E-02	-2.96E-01
1908	GSM658578	GSE26760	Multiple Myeloma	Blood	4.79E-02	-1.38E-01	3.72E-02	2.94E-01
1909	GSM658579	GSE26760	Multiple Myeloma	Blood	1.04E-01	-1.19E-01	6.31E-03	3.65E-01
1910	GSM658580	GSE26760	Multiple Myeloma	Blood	1.46E-02	-1.63E-01	9.82E-02	2.47E-01
1911	GSM658581	GSE26760	Multiple Myeloma	Blood	6.73E-02	-1.30E-01	1.96E-01	2.07E-01
1912	GSM658582	GSE26760	Multiple Myeloma	Blood	1.65E-01	1.06E-01	4.55E-02	2.85E-01
1913	GSM658583	GSE26760	Multiple Myeloma	Blood	3.23E-03	-1.89E-01	1.55E-01	2.22E-01
1914	GSM658584	GSE26760	Multiple Myeloma	Blood	2.24E-03	-1.95E-01	8.56E-02	2.54E-01
1915	GSM658585	GSE26760	Multiple Myeloma	Blood	5.16E-03	-1.82E-01	3.46E-01	1.67E-01
1916	GSM658586	GSE26760	Multiple Myeloma	Blood	6.19E-02	1.32E-01	1.32E-01	2.31E-01
1917	GSM658587	GSE26760	Multiple Myeloma	Blood	7.01E-02	-1.29E-01	2.51E-01	1.91E-01
1918	GSM658588	GSE26760	Multiple Myeloma	Blood	3.94E-01	7.64E-02	8.56E-02	2.54E-01
1919	GSM658589	GSE26760	Multiple Myeloma	Blood	7.01E-02	-1.29E-01	8.56E-02	2.54E-01

1920	GSM658590	GSE26760	Multiple Myeloma	Blood	1.85E-03	-1.98E-01	7.43E-02	2.62E-01
1921	GSM658591	GSE26760	Multiple Myeloma	Blood	1.16E-01	1.16E-01	3.30E-02	3.00E-01
1922	GSM658592	GSE26760	Multiple Myeloma	Blood	5.23E-02	-1.36E-01	3.16E-02	3.02E-01
1923	GSM658593	GSE26760	Multiple Myeloma	Blood	1.04E-01	-1.19E-01	3.78E-01	1.60E-01
1924	GSM658594	GSE26760	Multiple Myeloma	Blood	1.16E-01	-1.16E-01	6.31E-03	3.65E-01
1925	GSM658595	GSE26760	Multiple Myeloma	Blood	3.34E-02	-1.46E-01	8.56E-02	2.54E-01
1926	GSM658596	GSE26760	Multiple Myeloma	Blood	2.07E-02	-1.56E-01	3.89E-02	2.92E-01
1927	GSM658597	GSE26760	Multiple Myeloma	Blood	2.10E-03	-1.96E-01	7.73E-02	2.60E-01
1928	GSM658598	GSE26760	Multiple Myeloma	Blood	7.92E-02	-1.26E-01	4.96E-02	2.81E-01
1929	GSM658599	GSE26760	Multiple Myeloma	Blood	2.29E-02	-1.54E-01	1.21E-01	2.36E-01
1930	GSM658600	GSE26760	Multiple Myeloma	Blood	7.45E-04	-2.12E-01	3.15E-01	1.74E-01
1931	GSM658601	GSE26760	Multiple Myeloma	Blood	1.97E-02	-1.57E-01	2.19E-01	2.00E-01
1932	GSM658602	GSE26760	Multiple Myeloma	Blood	1.46E-04	2.35E-01	2.59E-03	3.96E-01
1933	GSM658603	GSE26760	Multiple Myeloma	Blood	6.19E-02	-1.32E-01	1.86E-01	-2.10E-01
1934	GSM658604	GSE26760	Multiple Myeloma	Blood	9.04E-03	-1.72E-01	6.43E-02	2.69E-01
1935	GSM658605	GSE26760	Multiple Myeloma	Blood	2.69E-03	-1.92E-01	8.56E-02	2.54E-01
1936	GSM658606	GSE26760	Multiple Myeloma	Blood	1.12E-02	-1.68E-01	1.75E-01	2.14E-01
1937	GSM658607	GSE26760	Multiple Myeloma	Blood	1.70E-02	-1.60E-01	1.16E-01	2.38E-01
1938	GSM658608	GSE26760	Multiple Myeloma	Blood	1.96E-04	-2.31E-01	1.71E-01	-2.16E-01
1939	GSM658609	GSE26760	Multiple Myeloma	Blood	7.25E-03	-1.76E-01	1.96E-01	2.07E-01
1940	GSM658610	GSE26760	Multiple Myeloma	Blood	2.52E-02	-1.52E-01	2.26E-01	1.98E-01
1941	GSM658611	GSE26760	Multiple Myeloma	Blood	1.12E-02	-1.68E-01	1.37E-01	2.29E-01
1942	GSM658612	GSE26760	Multiple Myeloma	Blood	4.95E-04	-2.18E-01	2.19E-01	2.00E-01
1943	GSM658613	GSE26760	Multiple Myeloma	Blood	1.88E-02	-1.58E-01	8.56E-02	2.54E-01
1944	GSM658614	GSE26760	Multiple Myeloma	Blood	5.01E-02	-1.37E-01	1.32E-01	2.31E-01
1945	GSM658615	GSE26760	Multiple Myeloma	Blood	1.39E-01	1.11E-01	4.75E-02	2.83E-01
1946	GSM658616	GSE26760	Multiple Myeloma	Blood	5.94E-02	1.33E-01	1.47E-03	4.14E-01
1947	GSM658617	GSE26760	Multiple Myeloma	Blood	7.01E-02	-1.29E-01	1.65E-01	2.18E-01
1948	GSM658618	GSE26760	Multiple Myeloma	Blood	8.91E-02	-1.23E-01	8.56E-02	2.54E-01
1949	GSM658619	GSE26760	Multiple Myeloma	Blood	2.29E-02	-1.54E-01	3.72E-02	2.94E-01
1950	GSM658620	GSE26760	Multiple Myeloma	Blood	1.71E-01	-1.05E-01	7.73E-02	2.60E-01
1951	GSM658621	GSE26760	Multiple Myeloma	Blood	4.39E-02	-1.40E-01	1.12E-01	2.40E-01
1952	GSM658622	GSE26760	Multiple Myeloma	Blood	2.29E-02	-1.54E-01	1.75E-01	2.14E-01
1953	GSM658623	GSE26760	Multiple Myeloma	Blood	4.31E-04	-2.20E-01	2.51E-01	1.91E-01
1954	GSM658624	GSE26760	Multiple Myeloma	Blood	5.01E-02	-1.37E-01	7.73E-02	2.60E-01
1955	GSM658625	GSE26760	Multiple Myeloma	Blood	1.00E-04	-2.40E-01	1.14E-01	-2.39E-01
1956	GSM658626	GSE26760	Multiple Myeloma	Blood	4.79E-02	1.38E-01	1.43E-02	3.34E-01
1957	GSM658627	GSE26760	Multiple Myeloma	Blood	1.70E-02	-1.60E-01	6.17E-02	2.71E-01
1958	GSM658628	GSE26760	Multiple Myeloma	Blood	2.64E-02	-1.51E-01	1.96E-01	2.07E-01
1959	GSM658629	GSE26760	Multiple Myeloma	Blood	1.77E-01	1.04E-01	3.30E-02	3.00E-01
1960	GSM658630	GSE26760	Multiple Myeloma	Blood	1.49E-01	-1.09E-01	1.32E-01	2.31E-01
1961	GSM658631	GSE26760	Multiple Myeloma	Blood	5.47E-03	-1.81E-01	1.21E-01	2.36E-01
1962	GSM658632	GSE26760	Multiple Myeloma	Blood	3.04E-01	8.63E-02	4.96E-02	2.81E-01
1963	GSM658633	GSE26760	Multiple Myeloma	Blood	4.01E-02	-1.42E-01	1.32E-01	2.31E-01
1964	GSM658634	GSE26760	Multiple Myeloma	Blood	6.81E-05	-2.45E-01	2.02E-01	2.05E-01
1965	GSM658635	GSE26760	Multiple Myeloma	Blood	3.19E-02	-1.47E-01	3.78E-01	1.60E-01
1966	GSM658636	GSE26760	Multiple Myeloma	Blood	1.12E-01	1.17E-01	2.44E-03	3.98E-01
1967	GSM658637	GSE26760	Multiple Myeloma	Blood	6.46E-02	-1.31E-01	2.26E-01	1.98E-01
1968	GSM658638	GSE26760	Multiple Myeloma	Blood	4.39E-02	-1.40E-01	3.16E-02	3.02E-01
1969	GSM658639	GSE26760	Multiple Myeloma	Blood	1.89E-01	1.02E-01	7.81E-04	4.34E-01
1970	GSM658640	GSE26760	Multiple Myeloma	Blood	8.57E-02	-1.24E-01	2.55E-02	3.11E-01
1971	GSM658641	GSE26760	Multiple Myeloma	Blood	2.10E-03	-1.96E-01	8.56E-02	2.54E-01
1972	GSM658642	GSE26760	Multiple Myeloma	Blood	3.86E-03	-1.87E-01	2.26E-01	1.98E-01
1973	GSM658643	GSE26760	Multiple Myeloma	Blood	1.26E-03	-2.04E-01	7.43E-02	2.62E-01
1974	GSM658644	GSE26760	Multiple Myeloma	Blood	1.12E-02	-1.68E-01	1.96E-01	2.07E-01
1975	GSM658645	GSE26760	Multiple Myeloma	Blood	4.34E-03	-1.85E-01	3.15E-01	1.74E-01
1976	GSM658646	GSE26760	Multiple Myeloma	Blood	2.23E-05	-2.59E-01	3.77E-02	-2.94E-01
1977	GSM658647	GSE26760	Multiple Myeloma	Blood	2.86E-03	-1.91E-01	1.80E-01	2.12E-01
1978	GSM658648	GSE26760	Multiple Myeloma	Blood	2.64E-02	-1.51E-01	3.46E-01	1.67E-01
1979	GSM658649	GSE26760	Multiple Myeloma	Blood	1.35E-03	-2.03E-01	4.34E-01	-1.48E-01
1980	GSM658650	GSE26760	Multiple Myeloma	Blood	1.18E-02	-1.67E-01	1.32E-01	2.31E-01
1981	GSM658651	GSE26760	Multiple Myeloma	Blood	4.33E-01	-7.24E-02	2.15E-02	3.18E-01
1982	GSM658652	GSE26760	Multiple Myeloma	Blood	2.64E-02	-1.51E-01	1.17E-03	4.22E-01
1983	GSM658653	GSE26760	Multiple Myeloma	Blood	1.79E-02	-1.59E-01	2.19E-01	2.00E-01
1984	GSM658654	GSE26760	Multiple Myeloma	Blood	2.86E-03	-1.91E-01	3.72E-02	2.94E-01
1985	GSM658655	GSE26760	Multiple Myeloma	Blood	6.73E-02	1.30E-01	3.21E-03	3.89E-01
1986	GSM658656	GSE26760	Multiple Myeloma	Blood	1.04E-01	-1.19E-01	9.82E-02	2.47E-01
1987	GSM658657	GSE26760	Multiple Myeloma	Blood	1.44E-03	-2.02E-01	3.46E-01	1.67E-01
1988	GSM658658	GSE26760	Multiple Myeloma	Blood	4.87E-03	-1.83E-01	8.56E-02	2.54E-01
1989	GSM658659	GSE26760	Multiple Myeloma	Blood	1.00E-01	-1.20E-01	1.43E-02	3.34E-01
1990	GSM658660	GSE26760	Multiple Myeloma	Blood	7.01E-02	-1.29E-01	2.02E-01	2.05E-01
1991	GSM658661	GSE26760	Multiple Myeloma	Blood	1.08E-01	-1.18E-01	3.30E-02	3.00E-01
1992	GSM658662	GSE26760	Multiple Myeloma	Blood	1.50E-06	-2.90E-01	3.60E-03	-3.85E-01
1993	GSM658663	GSE26760	Multiple Myeloma	Blood	4.94E-01	6.65E-02	8.12E-03	3.56E-01

1994	GSM658664	GSE26760	Multiple Myeloma	Blood	3.21E-01	8.43E-02	3.72E-02	2.94E-01
1995	GSM658665	GSE26760	Multiple Myeloma	Blood	7.01E-02	-1.29E-01	1.75E-01	2.14E-01
1996	GSM658666	GSE26760	Multiple Myeloma	Blood	6.48E-03	-1.78E-01	1.02E-01	2.45E-01
1997	GSM658667	GSE26760	Multiple Myeloma	Blood	3.25E-04	2.24E-01	1.83E-04	4.76E-01
1998	GSM658668	GSE26760	Multiple Myeloma	Blood	1.39E-01	-1.11E-01	9.85E-03	3.49E-01
1999	GSM658669	GSE26760	Multiple Myeloma	Blood	1.21E-01	1.15E-01	2.15E-02	3.18E-01
2000	GSM658670	GSE26760	Multiple Myeloma	Blood	3.04E-03	-1.90E-01	1.16E-01	2.38E-01
2001	GSM658671	GSE26760	Multiple Myeloma	Blood	5.01E-02	-1.37E-01	8.56E-02	2.54E-01
2002	GSM658672	GSE26760	Multiple Myeloma	Blood	1.38E-02	-1.64E-01	1.12E-01	2.40E-01
2003	GSM658673	GSE26760	Multiple Myeloma	Blood	4.34E-03	1.85E-01	1.57E-03	4.12E-01
2004	GSM658674	GSE26760	Multiple Myeloma	Blood	5.68E-04	-2.16E-01	1.76E-01	-2.14E-01
2005	GSM658675	GSE26760	Multiple Myeloma	Blood	5.16E-03	-1.82E-01	1.16E-01	2.38E-01
2006	GSM658676	GSE26760	Multiple Myeloma	Blood	2.28E-01	-9.62E-02	2.94E-01	1.80E-01
2007	GSM658677	GSE26760	Multiple Myeloma	Blood	1.71E-01	1.05E-01	3.40E-03	3.87E-01
2008	GSM658678	GSE26760	Multiple Myeloma	Blood	2.08E-01	9.92E-02	1.25E-02	3.40E-01
2009	GSM658679	GSE26760	Multiple Myeloma	Blood	3.63E-03	-1.88E-01	1.98E-01	-2.07E-01
2010	GSM658680	GSE26760	Multiple Myeloma	Blood	1.95E-01	1.01E-01	9.82E-02	2.47E-01
2011	GSM658681	GSE26760	Multiple Myeloma	Blood	8.10E-03	-1.74E-01	1.32E-01	2.31E-01
2012	GSM658682	GSE26760	Multiple Myeloma	Blood	2.07E-02	-1.56E-01	5.31E-02	2.78E-01
2013	GSM658683	GSE26760	Multiple Myeloma	Blood	1.18E-03	2.05E-01	5.37E-05	5.09E-01
2014	GSM658684	GSE26760	Multiple Myeloma	Blood	1.89E-01	1.02E-01	8.56E-02	2.54E-01
2015	GSM658685	GSE26760	Multiple Myeloma	Blood	1.06E-02	1.69E-01	2.55E-02	3.11E-01
2016	GSM658686	GSE26760	Multiple Myeloma	Blood	1.26E-04	-2.37E-01	7.24E-02	-2.63E-01
2017	GSM658687	GSE26760	Multiple Myeloma	Blood	6.51E-04	-2.14E-01	1.75E-01	2.14E-01
2018	GSM658688	GSE26760	Multiple Myeloma	Blood	6.73E-02	-1.30E-01	2.02E-01	2.05E-01
2019	GSM658689	GSE26760	Multiple Myeloma	Blood	4.25E-05	-2.51E-01	2.51E-01	1.91E-01
2020	GSM658690	GSE26760	Multiple Myeloma	Blood	3.84E-02	-1.43E-01	3.30E-02	3.00E-01
2021	GSM658691	GSE26760	Multiple Myeloma	Blood	1.12E-02	-1.68E-01	1.16E-01	2.38E-01
2022	GSM658692	GSE26760	Multiple Myeloma	Blood	1.30E-01	1.13E-01	1.84E-03	4.07E-01
2023	GSM658693	GSE26760	Multiple Myeloma	Blood	1.79E-02	1.59E-01	1.51E-04	4.81E-01
2024	GSM658694	GSE26760	Multiple Myeloma	Blood	1.46E-02	-1.63E-01	1.80E-01	2.12E-01
2025	GSM658695	GSE26760	Multiple Myeloma	Blood	4.79E-02	-1.38E-01	2.46E-01	-1.92E-01
2026	GSM658696	GSE26760	Multiple Myeloma	Blood	8.24E-02	-1.25E-01	1.16E-01	2.38E-01
2027	GSM658697	GSE26760	Multiple Myeloma	Blood	1.21E-01	-1.15E-01	8.56E-02	2.54E-01
2028	GSM658698	GSE26760	Multiple Myeloma	Blood	2.57E-01	-9.23E-02	1.18E-01	2.37E-01
2029	GSM658699	GSE26760	Multiple Myeloma	Blood	2.07E-02	1.56E-01	7.32E-04	4.36E-01
2030	GSM658700	GSE26760	Multiple Myeloma	Blood	3.74E-04	-2.22E-01	2.08E-01	2.03E-01
2031	GSM658701	GSE26760	Multiple Myeloma	Blood	4.39E-02	-1.40E-01	1.12E-01	2.40E-01
2032	GSM658702	GSE26760	Multiple Myeloma	Blood	5.47E-03	-1.81E-01	7.73E-02	2.60E-01
2033	GSM658703	GSE26760	Multiple Myeloma	Blood	2.80E-01	8.93E-02	3.21E-03	3.89E-01
2034	GSM658704	GSE26760	Multiple Myeloma	Blood	1.35E-04	-2.36E-01	9.23E-02	-2.50E-01
2035	GSM658705	GSE26760	Multiple Myeloma	Blood	3.93E-05	-2.52E-01	3.46E-01	1.67E-01
2036	GSM658706	GSE26760	Multiple Myeloma	Blood	8.24E-02	-1.25E-01	3.15E-01	1.74E-01
2037	GSM658707	GSE26760	Multiple Myeloma	Blood	1.79E-02	-1.59E-01	2.58E-01	1.89E-01
2038	GSM658708	GSE26760	Multiple Myeloma	Blood	3.05E-02	1.48E-01	7.32E-04	4.36E-01
2039	GSM658709	GSE26760	Multiple Myeloma	Blood	4.01E-02	-1.42E-01	2.15E-01	2.01E-01
2040	GSM658710	GSE26760	Multiple Myeloma	Blood	1.31E-02	-1.65E-01	1.21E-01	2.36E-01
2041	GSM658711	GSE26760	Multiple Myeloma	Blood	2.86E-03	-1.91E-01	1.75E-01	2.14E-01
2042	GSM658712	GSE26760	Multiple Myeloma	Blood	2.01E-01	1.00E-01	2.55E-02	3.11E-01
2043	GSM658713	GSE26760	Multiple Myeloma	Blood	9.73E-04	-2.08E-01	1.51E-01	-2.23E-01
2044	GSM658714	GSE26760	Multiple Myeloma	Blood	6.28E-06	-2.74E-01	6.26E-02	-2.70E-01
2045	GSM658715	GSE26760	Multiple Myeloma	Blood	7.92E-02	-1.26E-01	3.72E-02	2.94E-01
2046	GSM658716	GSE26760	Multiple Myeloma	Blood	8.10E-03	-1.74E-01	5.10E-02	2.80E-01
2047	GSM658717	GSE26760	Multiple Myeloma	Blood	2.40E-02	-1.53E-01	5.54E-02	2.76E-01
2048	GSM658718	GSE26760	Multiple Myeloma	Blood	5.79E-03	-1.80E-01	3.01E-01	1.78E-01
2049	GSM658719	GSE26760	Multiple Myeloma	Blood	1.61E-02	-1.61E-01	1.12E-01	2.40E-01
2050	GSM658720	GSE26760	Multiple Myeloma	Blood	4.59E-02	-1.39E-01	8.56E-02	2.54E-01
2051	GSM658721	GSE26760	Multiple Myeloma	Blood	1.31E-02	-1.65E-01	6.17E-02	2.71E-01
2052	GSM392759	GSE21349	Multiple Myeloma	Blood	7.92E-02	1.26E-01	2.15E-02	3.18E-01
2053	GSM392760	GSE21349	Multiple Myeloma	Blood	1.21E-01	-1.15E-01	4.75E-02	2.83E-01
2054	GSM392761	GSE21349	Multiple Myeloma	Blood	1.08E-01	1.18E-01	6.17E-02	2.71E-01
2055	GSM392762	GSE21349	Multiple Myeloma	Blood	3.42E-03	-1.88E-01	6.43E-02	2.69E-01
2056	GSM392763	GSE21349	Multiple Myeloma	Blood	3.30E-01	-8.33E-02	7.73E-02	2.60E-01
2057	GSM392764	GSE21349	Multiple Myeloma	Blood	1.00E-04	-2.40E-01	2.21E-01	-1.99E-01
2058	GSM392765	GSE21349	Multiple Myeloma	Blood	5.16E-03	-1.82E-01	7.43E-02	2.62E-01
2059	GSM392766	GSE21349	Multiple Myeloma	Blood	1.63E-03	-2.00E-01	5.31E-02	2.78E-01
2060	GSM392767	GSE21349	Multiple Myeloma	Blood	2.21E-01	-9.72E-02	3.89E-02	2.92E-01
2061	GSM392768	GSE21349	Multiple Myeloma	Blood	1.06E-02	-1.69E-01	2.88E-01	-1.81E-01
2062	GSM392769	GSE21349	Multiple Myeloma	Blood	1.89E-01	-1.02E-01	3.16E-02	3.02E-01
2063	GSM392770	GSE21349	Multiple Myeloma	Blood	3.84E-02	-1.43E-01	5.10E-02	2.80E-01
2064	GSM392771	GSE21349	Multiple Myeloma	Blood	2.42E-01	9.42E-02	8.56E-03	3.54E-01
2065	GSM392773	GSE21349	Multiple Myeloma	Blood	1.79E-02	-1.59E-01	4.55E-02	2.85E-01
2066	GSM392775	GSE21349	Multiple Myeloma	Blood	7.67E-03	-1.75E-01	3.16E-02	3.02E-01
2067	GSM392777	GSE21349	Multiple Myeloma	Blood	9.26E-02	-1.22E-01	1.06E-01	2.43E-01

2068	GSM392778	GSE21349	Multiple Myeloma	Blood	5.69E-02	-1.34E-01	4.36E-02	2.87E-01
2069	GSM392779	GSE21349	Multiple Myeloma	Blood	7.60E-02	1.27E-01	3.30E-02	3.00E-01
2070	GSM392780	GSE21349	Multiple Myeloma	Blood	4.79E-02	1.38E-01	5.31E-02	2.78E-01
2071	GSM392781	GSE21349	Multiple Myeloma	Blood	6.73E-02	1.30E-01	3.03E-03	3.91E-01
2072	GSM392782	GSE21349	Multiple Myeloma	Blood	4.79E-02	-1.38E-01	1.72E-02	3.27E-01
2073	GSM392783	GSE21349	Multiple Myeloma	Blood	3.63E-03	-1.88E-01	1.80E-01	2.12E-01
2074	GSM392784	GSE21349	Multiple Myeloma	Blood	3.42E-03	-1.88E-01	3.78E-01	1.60E-01
2075	GSM392785	GSE21349	Multiple Myeloma	Blood	1.35E-03	-2.03E-01	1.96E-01	2.07E-01
2076	GSM392786	GSE21349	Multiple Myeloma	Blood	1.60E-01	-1.07E-01	6.43E-02	2.69E-01
2077	GSM392787	GSE21349	Multiple Myeloma	Blood	2.64E-02	1.51E-01	2.49E-03	3.97E-01
2078	GSM392788	GSE21349	Multiple Myeloma	Blood	2.62E-04	-2.27E-01	1.16E-01	2.38E-01
2079	GSM392789	GSE21349	Multiple Myeloma	Blood	1.04E-01	1.19E-01	2.26E-01	1.98E-01
2080	GSM392790	GSE21349	Multiple Myeloma	Blood	8.91E-02	1.23E-01	3.89E-02	2.92E-01
2081	GSM392791	GSE21349	Multiple Myeloma	Blood	1.60E-01	1.07E-01	5.38E-04	4.45E-01
2082	GSM392792	GSE21349	Multiple Myeloma	Blood	1.12E-02	-1.68E-01	6.43E-02	2.69E-01
2083	GSM392793	GSE21349	Multiple Myeloma	Blood	9.26E-02	1.22E-01	1.17E-03	4.22E-01
2084	GSM392794	GSE21349	Multiple Myeloma	Blood	1.26E-04	-2.37E-01	2.19E-01	2.00E-01
2085	GSM392795	GSE21349	Multiple Myeloma	Blood	4.31E-04	-2.20E-01	1.80E-01	2.12E-01
2086	GSM392796	GSE21349	Multiple Myeloma	Blood	2.72E-01	9.03E-02	3.89E-02	2.92E-01
2087	GSM392797	GSE21349	Multiple Myeloma	Blood	1.49E-01	1.09E-01	1.25E-02	3.40E-01
2088	GSM392798	GSE21349	Multiple Myeloma	Blood	1.11E-03	-2.06E-01	1.12E-01	2.40E-01
2089	GSM392799	GSE21349	Multiple Myeloma	Blood	9.26E-02	-1.22E-01	1.50E-02	3.32E-01
2090	GSM392800	GSE21349	Multiple Myeloma	Blood	9.63E-02	1.21E-01	2.80E-02	3.07E-01
2091	GSM392801	GSE21349	Multiple Myeloma	Blood	4.98E-05	-2.49E-01	1.98E-01	-2.07E-01
2092	GSM392802	GSE21349	Multiple Myeloma	Blood	1.25E-02	-1.66E-01	5.31E-02	2.78E-01
2093	GSM392803	GSE21349	Multiple Myeloma	Blood	1.95E-01	1.01E-01	6.67E-03	3.63E-01
2094	GSM392804	GSE21349	Multiple Myeloma	Blood	2.40E-02	-1.53E-01	3.15E-01	1.74E-01
2095	GSM392805	GSE21349	Multiple Myeloma	Blood	2.07E-02	-1.56E-01	2.19E-01	2.00E-01
2096	GSM392806	GSE21349	Multiple Myeloma	Blood	6.51E-04	2.14E-01	1.08E-04	4.90E-01
2097	GSM392807	GSE21349	Multiple Myeloma	Blood	1.08E-01	1.18E-01	3.16E-02	3.02E-01
2098	GSM392808	GSE21349	Multiple Myeloma	Blood	2.10E-03	-1.96E-01	1.75E-01	2.14E-01
2099	GSM392809	GSE21349	Multiple Myeloma	Blood	4.59E-02	-1.39E-01	3.16E-02	3.02E-01
2100	GSM392810	GSE21349	Multiple Myeloma	Blood	2.62E-04	-2.27E-01	1.12E-01	2.40E-01
2101	GSM392811	GSE21349	Multiple Myeloma	Blood	1.00E-01	1.20E-01	2.08E-03	4.03E-01
2102	GSM392812	GSE21349	Multiple Myeloma	Blood	5.69E-02	1.34E-01	2.55E-02	3.11E-01
2103	GSM392813	GSE21349	Multiple Myeloma	Blood	3.34E-02	-1.46E-01	1.32E-01	2.31E-01
2104	GSM392814	GSE21349	Multiple Myeloma	Blood	3.93E-05	-2.52E-01	2.47E-02	-3.12E-01
2105	GSM392815	GSE21349	Multiple Myeloma	Blood	1.38E-02	-1.64E-01	1.75E-01	2.14E-01
2106	GSM392816	GSE21349	Multiple Myeloma	Blood	3.05E-02	-1.48E-01	1.71E-01	2.16E-01
2107	GSM392817	GSE21349	Multiple Myeloma	Blood	3.23E-03	-1.89E-01	1.75E-01	2.14E-01
2108	GSM392818	GSE21349	Multiple Myeloma	Blood	5.79E-03	-1.80E-01	1.75E-01	2.14E-01
2109	GSM392819	GSE21349	Multiple Myeloma	Blood	6.08E-04	-2.15E-01	6.01E-02	-2.72E-01
2110	GSM392820	GSE21349	Multiple Myeloma	Blood	1.97E-03	-1.97E-01	1.37E-01	2.29E-01
2111	GSM392821	GSE21349	Multiple Myeloma	Blood	7.60E-02	-1.27E-01	2.51E-01	1.91E-01
2112	GSM392822	GSE21349	Multiple Myeloma	Blood	4.83E-06	-2.77E-01	1.29E-01	-2.32E-01
2113	GSM392823	GSE21349	Multiple Myeloma	Blood	5.23E-02	-1.36E-01	8.89E-02	2.52E-01
2114	GSM392824	GSE21349	Multiple Myeloma	Blood	2.52E-02	-1.52E-01	1.21E-01	2.36E-01
2115	GSM392826	GSE21349	Multiple Myeloma	Blood	2.52E-02	-1.52E-01	6.17E-02	2.71E-01
2116	GSM392827	GSE21349	Multiple Myeloma	Blood	5.16E-03	-1.82E-01	1.55E-01	2.22E-01
2117	GSM392828	GSE21349	Multiple Myeloma	Blood	1.70E-02	-1.60E-01	1.80E-02	3.25E-01
2118	GSM392829	GSE21349	Multiple Myeloma	Blood	4.20E-02	-1.41E-01	3.16E-02	3.02E-01
2119	GSM392830	GSE21349	Multiple Myeloma	Blood	1.31E-02	-1.65E-01	3.78E-01	1.60E-01
2120	GSM392831	GSE21349	Multiple Myeloma	Blood	1.71E-01	-1.05E-01	3.16E-02	3.02E-01
2121	GSM392832	GSE21349	Multiple Myeloma	Blood	3.84E-01	-7.74E-02	2.17E-01	2.01E-01
2122	GSM392833	GSE21349	Multiple Myeloma	Blood	2.01E-01	1.00E-01	8.56E-03	3.54E-01
2123	GSM392834	GSE21349	Multiple Myeloma	Blood	1.12E-02	-1.68E-01	1.76E-01	-2.14E-01
2124	GSM392835	GSE21349	Multiple Myeloma	Blood	3.05E-02	-1.48E-01	3.89E-02	2.92E-01
2125	GSM392836	GSE21349	Multiple Myeloma	Blood	5.16E-03	-1.82E-01	1.60E-01	2.20E-01
2126	GSM392837	GSE21349	Multiple Myeloma	Blood	3.23E-03	-1.89E-01	3.78E-01	1.60E-01
2127	GSM392838	GSE21349	Multiple Myeloma	Blood	4.60E-03	-1.84E-01	2.19E-01	2.00E-01
2128	GSM392839	GSE21349	Multiple Myeloma	Blood	5.68E-04	-2.16E-01	1.02E-01	2.45E-01
2129	GSM392840	GSE21349	Multiple Myeloma	Blood	1.95E-01	-1.01E-01	1.21E-01	2.36E-01
2130	GSM392841	GSE21349	Multiple Myeloma	Blood	7.30E-02	1.28E-01	1.19E-02	3.42E-01
2131	GSM392842	GSE21349	Multiple Myeloma	Blood	1.18E-03	-2.05E-01	1.34E-01	-2.30E-01
2132	GSM392843	GSE21349	Multiple Myeloma	Blood	6.19E-02	-1.32E-01	2.67E-02	3.09E-01
2133	GSM392844	GSE21349	Multiple Myeloma	Blood	2.18E-02	-1.55E-01	3.89E-02	2.92E-01
2134	GSM392845	GSE21349	Multiple Myeloma	Blood	1.26E-03	-2.04E-01	2.46E-01	-1.92E-01
2135	GSM392846	GSE21349	Multiple Myeloma	Blood	2.80E-01	8.93E-02	5.10E-02	2.80E-01
2136	GSM392847	GSE21349	Multiple Myeloma	Blood	7.30E-02	-1.28E-01	4.20E-03	3.80E-01
2137	GSM392848	GSE21349	Multiple Myeloma	Blood	1.16E-01	-1.16E-01	3.72E-02	2.94E-01
2138	GSM392849	GSE21349	Multiple Myeloma	Blood	9.27E-05	2.41E-01	3.95E-06	5.72E-01
2139	GSM392850	GSE21349	Multiple Myeloma	Blood	9.63E-02	1.21E-01	3.93E-04	4.54E-01
2140	GSM392851	GSE21349	Multiple Myeloma	Blood	3.23E-03	1.89E-01	1.58E-05	5.40E-01
2141	GSM392852	GSE21349	Multiple Myeloma	Blood	9.54E-03	-1.71E-01	1.74E-02	-3.27E-01

2142	GSM392853	GSE21349	Multiple Myeloma	Blood	9.04E-03	1.72E-01	2.44E-03	3.98E-01
2143	GSM392854	GSE21349	Multiple Myeloma	Blood	2.07E-02	-1.56E-01	1.46E-01	2.25E-01
2144	GSM392855	GSE21349	Multiple Myeloma	Blood	4.79E-02	-1.38E-01	8.56E-02	2.54E-01
2145	GSM392856	GSE21349	Multiple Myeloma	Blood	1.18E-02	-1.67E-01	1.55E-01	2.22E-01
2146	GSM392857	GSE21349	Multiple Myeloma	Blood	1.70E-02	-1.60E-01	2.26E-01	1.98E-01
2147	GSM392858	GSE21349	Multiple Myeloma	Blood	1.06E-02	-1.69E-01	2.32E-01	1.96E-01
2148	GSM392859	GSE21349	Multiple Myeloma	Blood	2.52E-02	-1.52E-01	1.02E-01	2.45E-01
2149	GSM392860	GSE21349	Multiple Myeloma	Blood	9.26E-02	-1.22E-01	9.23E-02	2.50E-01
2150	GSM392861	GSE21349	Multiple Myeloma	Blood	1.31E-02	-1.65E-01	1.80E-02	3.25E-01
2151	GSM392862	GSE21349	Multiple Myeloma	Blood	1.97E-02	-1.57E-01	1.96E-01	2.07E-01
2152	GSM392863	GSE21349	Multiple Myeloma	Blood	7.92E-02	-1.26E-01	3.16E-02	3.02E-01
2153	GSM392864	GSE21349	Multiple Myeloma	Blood	2.42E-01	9.42E-02	1.67E-03	4.10E-01
2154	GSM392865	GSE21349	Multiple Myeloma	Blood	4.59E-02	1.39E-01	7.70E-03	3.58E-01
2155	GSM392866	GSE21349	Multiple Myeloma	Blood	4.87E-03	-1.83E-01	1.32E-01	2.31E-01
2156	GSM392867	GSE21349	Multiple Myeloma	Blood	2.91E-02	-1.49E-01	7.43E-02	2.62E-01
2157	GSM392868	GSE21349	Multiple Myeloma	Blood	3.74E-04	-2.22E-01	6.69E-02	-2.67E-01
2158	GSM392869	GSE21349	Multiple Myeloma	Blood	2.88E-01	-8.83E-02	5.54E-02	2.76E-01
2159	GSM392870	GSE21349	Multiple Myeloma	Blood	5.47E-03	-1.81E-01	2.15E-02	3.18E-01
2160	GSM392871	GSE21349	Multiple Myeloma	Blood	2.96E-01	8.73E-02	1.31E-04	4.85E-01
2161	GSM392872	GSE21349	Multiple Myeloma	Blood	1.16E-01	-1.16E-01	7.70E-03	3.58E-01
2162	GSM392873	GSE21349	Multiple Myeloma	Blood	1.04E-01	1.19E-01	1.75E-01	2.14E-01
2163	GSM392875	GSE21349	Multiple Myeloma	Blood	8.24E-02	1.25E-01	7.70E-03	3.58E-01
2164	GSM392876	GSE21349	Multiple Myeloma	Blood	6.13E-03	-1.79E-01	6.17E-02	2.71E-01
2165	GSM392877	GSE21349	Multiple Myeloma	Blood	1.16E-01	-1.16E-01	3.72E-02	2.94E-01
2166	GSM392878	GSE21349	Multiple Myeloma	Blood	2.07E-02	-1.56E-01	1.18E-01	-2.37E-01
2167	GSM392879	GSE21349	Multiple Myeloma	Blood	4.79E-02	1.38E-01	5.16E-03	3.72E-01
2168	GSM392880	GSE21349	Multiple Myeloma	Blood	8.13E-06	-2.71E-01	2.73E-01	-1.85E-01
2169	GSM392881	GSE21349	Multiple Myeloma	Blood	3.34E-02	1.46E-01	5.16E-03	3.72E-01
2170	GSM392882	GSE21349	Multiple Myeloma	Blood	2.01E-01	-1.00E-01	2.15E-02	3.18E-01
2171	GSM392883	GSE21349	Multiple Myeloma	Blood	3.19E-02	-1.47E-01	5.31E-02	2.78E-01
2172	GSM392884	GSE21349	Multiple Myeloma	Blood	1.97E-02	-1.57E-01	2.51E-01	1.91E-01
2173	GSM392885	GSE21349	Multiple Myeloma	Blood	5.01E-02	1.37E-01	1.96E-03	4.05E-01
2174	GSM392886	GSE21349	Multiple Myeloma	Blood	5.46E-02	-1.35E-01	1.21E-01	2.36E-01
2175	GSM392887	GSE21349	Multiple Myeloma	Blood	4.39E-02	1.40E-01	1.02E-01	2.45E-01
2176	GSM392888	GSE21349	Multiple Myeloma	Blood	6.19E-02	-1.32E-01	8.56E-02	2.54E-01
2177	GSM392890	GSE21349	Multiple Myeloma	Blood	4.23E-01	7.34E-02	1.65E-01	2.18E-01
2178	GSM392891	GSE21349	Multiple Myeloma	Blood	1.60E-01	1.07E-01	9.27E-04	4.29E-01
2179	GSM392892	GSE21349	Multiple Myeloma	Blood	1.25E-02	-1.66E-01	3.15E-01	1.74E-01
2180	GSM392893	GSE21349	Multiple Myeloma	Blood	1.12E-02	-1.68E-01	1.02E-01	2.45E-01
2181	GSM392894	GSE21349	Multiple Myeloma	Blood	1.46E-02	-1.63E-01	8.56E-02	2.54E-01
2182	GSM392895	GSE21349	Multiple Myeloma	Blood	1.01E-02	-1.70E-01	1.16E-01	2.38E-01
2183	GSM392896	GSE21349	Multiple Myeloma	Blood	1.25E-02	-1.66E-01	3.89E-02	2.92E-01
2184	GSM392897	GSE21349	Multiple Myeloma	Blood	2.01E-01	-1.00E-01	1.89E-02	3.23E-01
2185	GSM392898	GSE21349	Multiple Myeloma	Blood	2.40E-02	-1.53E-01	7.43E-02	2.62E-01
2186	GSM392899	GSE21349	Multiple Myeloma	Blood	1.21E-01	-1.15E-01	4.36E-02	2.87E-01
2187	GSM392900	GSE21349	Multiple Myeloma	Blood	1.61E-02	-1.61E-01	2.19E-01	2.00E-01
2188	GSM392901	GSE21349	Multiple Myeloma	Blood	1.34E-01	-1.12E-01	9.82E-02	2.47E-01
2189	GSM392902	GSE21349	Multiple Myeloma	Blood	1.30E-01	-1.13E-01	4.20E-03	3.80E-01
2190	GSM392903	GSE21349	Multiple Myeloma	Blood	1.57E-04	-2.34E-01	1.37E-01	2.29E-01
2191	GSM392904	GSE21349	Multiple Myeloma	Blood	1.38E-02	-1.64E-01	8.89E-02	2.52E-01
2192	GSM392905	GSE21349	Multiple Myeloma	Blood	8.57E-02	-1.24E-01	2.44E-03	3.98E-01
2193	GSM392906	GSE21349	Multiple Myeloma	Blood	7.01E-02	1.29E-01	1.50E-02	3.32E-01
2194	GSM392907	GSE21349	Multiple Myeloma	Blood	3.13E-01	-8.53E-02	1.32E-01	2.31E-01
2195	GSM392908	GSE21349	Multiple Myeloma	Blood	4.60E-03	-1.84E-01	1.55E-01	2.22E-01
2196	GSM392909	GSE21349	Multiple Myeloma	Blood	7.01E-02	-1.29E-01	8.89E-02	2.52E-01
2197	GSM392910	GSE21349	Multiple Myeloma	Blood	6.73E-02	-1.30E-01	2.51E-01	1.91E-01
2198	GSM392911	GSE21349	Multiple Myeloma	Blood	2.52E-02	-1.52E-01	2.08E-01	-2.03E-01
2199	GSM392912	GSE21349	Multiple Myeloma	Blood	1.83E-01	1.03E-01	1.80E-02	3.25E-01
2200	GSM392913	GSE21349	Multiple Myeloma	Blood	2.35E-01	-9.52E-02	9.85E-03	3.49E-01
2201	GSM392914	GSE21349	Multiple Myeloma	Blood	7.60E-02	1.27E-01	4.70E-03	3.76E-01
2202	GSM392915	GSE21349	Multiple Myeloma	Blood	7.67E-03	-1.75E-01	1.16E-01	2.38E-01
2203	GSM392916	GSE21349	Multiple Myeloma	Blood	3.84E-01	7.74E-02	1.98E-02	3.21E-01
2204	GSM392917	GSE21349	Multiple Myeloma	Blood	1.04E-01	1.19E-01	4.20E-03	3.80E-01
2205	GSM392918	GSE21349	Multiple Myeloma	Blood	1.74E-03	-1.99E-01	7.73E-02	-2.60E-01
2206	GSM392919	GSE21349	Multiple Myeloma	Blood	1.18E-02	-1.67E-01	4.55E-02	2.85E-01
2207	GSM392920	GSE21349	Multiple Myeloma	Blood	1.08E-04	-2.39E-01	9.58E-02	-2.49E-01
2208	GSM392922	GSE21349	Multiple Myeloma	Blood	1.60E-01	1.07E-01	1.43E-02	3.34E-01
2209	GSM392923	GSE21349	Multiple Myeloma	Blood	9.04E-03	-1.72E-01	3.89E-02	2.92E-01
2210	GSM392924	GSE21349	Multiple Myeloma	Blood	9.65E-06	-2.69E-01	1.51E-01	-2.23E-01
2211	GSM392926	GSE21349	Multiple Myeloma	Blood	8.57E-02	-1.24E-01	2.94E-01	1.80E-01
2212	GSM392927	GSE21349	Multiple Myeloma	Blood	3.63E-03	-1.88E-01	1.32E-01	2.31E-01
2213	GSM392928	GSE21349	Multiple Myeloma	Blood	3.84E-02	-1.43E-01	4.88E-03	3.74E-01
2214	GSM392929	GSE21349	Multiple Myeloma	Blood	1.71E-01	1.05E-01	1.96E-03	4.05E-01
2215	GSM392930	GSE21349	Multiple Myeloma	Blood	9.04E-03	-1.72E-01	1.14E-01	-2.39E-01

2216	GSM392931	GSE21349	Multiple Myeloma	Blood	3.50E-02	1.45E-01	2.37E-04	4.69E-01
2217	GSM392932	GSE21349	Multiple Myeloma	Blood	3.05E-02	-1.48E-01	6.43E-02	2.69E-01
2218	GSM392933	GSE21349	Multiple Myeloma	Blood	3.74E-04	-2.22E-01	1.32E-01	2.31E-01
2219	GSM392934	GSE21349	Multiple Myeloma	Blood	1.82E-04	-2.32E-01	1.37E-01	2.29E-01
2220	GSM392935	GSE21349	Multiple Myeloma	Blood	2.64E-02	-1.51E-01	1.19E-02	3.42E-01
2221	GSM392936	GSE21349	Multiple Myeloma	Blood	2.01E-01	1.00E-01	2.08E-02	3.19E-01
2222	GSM392937	GSE21349	Multiple Myeloma	Blood	9.11E-04	-2.09E-01	2.39E-01	1.94E-01
2223	GSM392938	GSE21349	Multiple Myeloma	Blood	2.44E-04	-2.28E-01	1.57E-01	-2.21E-01
2224	GSM392939	GSE21349	Multiple Myeloma	Blood	1.53E-03	2.01E-01	4.50E-04	4.50E-01
2225	GSM392940	GSE21349	Multiple Myeloma	Blood	4.95E-04	-2.18E-01	1.96E-01	2.07E-01
2226	GSM392941	GSE21349	Multiple Myeloma	Blood	4.60E-03	-1.84E-01	1.92E-01	2.09E-01
2227	GSM392942	GSE21349	Multiple Myeloma	Blood	3.67E-02	-1.44E-01	3.46E-01	1.67E-01
2228	GSM392943	GSE21349	Multiple Myeloma	Blood	8.24E-02	1.25E-01	1.57E-03	4.12E-01
2229	GSM392944	GSE21349	Multiple Myeloma	Blood	1.30E-01	1.13E-01	1.19E-02	3.42E-01
2230	GSM392945	GSE21349	Multiple Myeloma	Blood	1.38E-02	-1.64E-01	5.10E-02	2.80E-01
2231	GSM392947	GSE21349	Multiple Myeloma	Blood	6.86E-03	-1.77E-01	2.19E-01	2.00E-01
2232	GSM392948	GSE21349	Multiple Myeloma	Blood	4.01E-02	-1.42E-01	2.32E-01	1.96E-01
2233	GSM392949	GSE21349	Multiple Myeloma	Blood	7.60E-02	1.27E-01	5.10E-02	2.80E-01
2234	GSM392950	GSE21349	Multiple Myeloma	Blood	3.63E-03	-1.88E-01	1.02E-01	2.45E-01
2235	GSM392951	GSE21349	Multiple Myeloma	Blood	3.25E-04	-2.24E-01	4.80E-01	-1.39E-01
2236	GSM392953	GSE21349	Multiple Myeloma	Blood	5.16E-03	-1.82E-01	2.66E-01	1.87E-01
2237	GSM392954	GSE21349	Multiple Myeloma	Blood	1.34E-01	-1.12E-01	7.70E-03	3.58E-01
2238	GSM392955	GSE21349	Multiple Myeloma	Blood	2.77E-02	-1.50E-01	9.94E-02	-2.47E-01
2239	GSM392956	GSE21349	Multiple Myeloma	Blood	1.11E-03	-2.06E-01	2.51E-01	1.91E-01
2240	GSM392957	GSE21349	Multiple Myeloma	Blood	3.05E-02	-1.48E-01	4.22E-01	1.51E-01
2241	GSM392958	GSE21349	Multiple Myeloma	Blood	5.23E-02	-1.36E-01	7.43E-02	2.62E-01
2242	GSM392959	GSE21349	Multiple Myeloma	Blood	1.18E-03	-2.05E-01	1.60E-01	-2.20E-01
2243	GSM392960	GSE21349	Multiple Myeloma	Blood	8.57E-02	1.24E-01	5.16E-03	3.72E-01
2244	GSM392961	GSE21349	Multiple Myeloma	Blood	4.62E-04	-2.19E-01	6.17E-02	2.71E-01
2245	GSM392962	GSE21349	Multiple Myeloma	Blood	2.53E-03	-1.93E-01	2.19E-01	2.00E-01
2246	GSM392963	GSE21349	Multiple Myeloma	Blood	1.31E-02	-1.65E-01	3.23E-01	1.72E-01
2247	GSM392964	GSE21349	Multiple Myeloma	Blood	4.09E-03	-1.86E-01	3.58E-01	-1.65E-01
2248	GSM392965	GSE21349	Multiple Myeloma	Blood	4.62E-04	-2.19E-01	2.08E-02	-3.19E-01
2249	GSM392966	GSE21349	Multiple Myeloma	Blood	1.53E-03	-2.01E-01	2.26E-01	1.98E-01
2250	GSM392967	GSE21349	Multiple Myeloma	Blood	5.69E-02	-1.34E-01	6.69E-02	2.67E-01
2251	GSM392968	GSE21349	Multiple Myeloma	Blood	1.60E-01	1.07E-01	9.82E-02	2.47E-01
2252	GSM392969	GSE21349	Multiple Myeloma	Blood	2.21E-01	-9.72E-02	5.45E-03	3.70E-01
2253	GSM392970	GSE21349	Multiple Myeloma	Blood	6.19E-02	-1.32E-01	2.55E-02	3.11E-01
2254	GSM392972	GSE21349	Multiple Myeloma	Blood	1.46E-04	-2.35E-01	1.02E-01	2.45E-01
2255	GSM392973	GSE21349	Multiple Myeloma	Blood	1.11E-03	-2.06E-01	1.55E-01	2.22E-01
2256	GSM392974	GSE21349	Multiple Myeloma	Blood	3.21E-01	-8.43E-02	1.37E-01	2.29E-01
2257	GSM392975	GSE21349	Multiple Myeloma	Blood	7.67E-03	-1.75E-01	1.16E-01	2.38E-01
2258	GSM392976	GSE21349	Multiple Myeloma	Blood	3.47E-01	8.13E-02	1.74E-02	3.27E-01
2259	GSM392977	GSE21349	Multiple Myeloma	Blood	6.48E-03	-1.78E-01	2.26E-01	1.98E-01
2260	GSM392978	GSE21349	Multiple Myeloma	Blood	3.71E-06	-2.80E-01	1.51E-01	-2.23E-01
2261	GSM392979	GSE21349	Multiple Myeloma	Blood	5.30E-04	-2.17E-01	2.25E-02	3.16E-01
2262	GSM392980	GSE21349	Multiple Myeloma	Blood	5.23E-02	-1.36E-01	2.19E-01	2.00E-01
2263	GSM392981	GSE21349	Multiple Myeloma	Blood	2.91E-02	-1.49E-01	8.56E-02	2.54E-01
2264	GSM392982	GSE21349	Multiple Myeloma	Blood	3.23E-03	-1.89E-01	2.08E-01	2.03E-01
2265	GSM392983	GSE21349	Multiple Myeloma	Blood	1.63E-03	-2.00E-01	1.32E-01	2.31E-01
2266	GSM392984	GSE21349	Multiple Myeloma	Blood	1.85E-03	-1.98E-01	8.56E-02	2.54E-01
2267	GSM392985	GSE21349	Multiple Myeloma	Blood	4.34E-03	-1.85E-01	6.17E-02	2.71E-01
2268	GSM392986	GSE21349	Multiple Myeloma	Blood	6.30E-05	-2.46E-01	6.43E-02	2.69E-01
2269	GSM392987	GSE21349	Multiple Myeloma	Blood	7.25E-03	-1.76E-01	1.72E-02	3.27E-01
2270	GSM392988	GSE21349	Multiple Myeloma	Blood	4.20E-02	-1.41E-01	8.89E-02	2.52E-01
2271	GSM392989	GSE21349	Multiple Myeloma	Blood	5.94E-02	-1.33E-01	1.06E-01	2.43E-01
2272	GSM392990	GSE21349	Multiple Myeloma	Blood	3.50E-02	-1.45E-01	2.39E-01	1.94E-01
2273	GSM392991	GSE21349	Multiple Myeloma	Blood	1.74E-03	-1.99E-01	3.15E-01	1.74E-01
2274	GSM392992	GSE21349	Multiple Myeloma	Blood	2.29E-02	-1.54E-01	2.21E-01	-1.99E-01
2275	GSM392993	GSE21349	Multiple Myeloma	Blood	8.91E-02	-1.23E-01	1.19E-02	3.42E-01
2276	GSM392994	GSE21349	Multiple Myeloma	Blood	1.69E-04	-2.33E-01	6.17E-02	2.71E-01
2277	GSM392995	GSE21349	Multiple Myeloma	Blood	2.91E-02	-1.49E-01	1.16E-01	2.38E-01
2278	GSM392996	GSE21349	Multiple Myeloma	Blood	1.95E-01	1.01E-01	1.19E-02	3.42E-01
2279	GSM392997	GSE21349	Multiple Myeloma	Blood	2.08E-01	9.92E-02	5.16E-03	3.72E-01
2280	GSM392998	GSE21349	Multiple Myeloma	Blood	5.23E-02	1.36E-01	2.44E-03	3.98E-01
2281	GSM392999	GSE21349	Multiple Myeloma	Blood	1.01E-02	-1.70E-01	1.06E-01	2.43E-01
2282	GSM393000	GSE21349	Multiple Myeloma	Blood	6.81E-05	2.45E-01	4.20E-08	6.69E-01
2283	GSM393001	GSE21349	Multiple Myeloma	Blood	3.34E-05	-2.54E-01	1.75E-01	2.14E-01
2284	GSM393002	GSE21349	Multiple Myeloma	Blood	1.61E-02	-1.61E-01	1.55E-01	2.22E-01
2285	GSM393003	GSE21349	Multiple Myeloma	Blood	1.08E-04	-2.39E-01	1.66E-02	-3.29E-01
2286	GSM393004	GSE21349	Multiple Myeloma	Blood	4.83E-06	-2.77E-01	6.96E-02	-2.65E-01
2287	GSM393005	GSE21349	Multiple Myeloma	Blood	5.79E-03	-1.80E-01	2.19E-01	2.00E-01
2288	GSM393006	GSE21349	Multiple Myeloma	Blood	1.31E-02	-1.65E-01	2.55E-02	3.11E-01
2289	GSM393007	GSE21349	Multiple Myeloma	Blood	9.63E-02	1.21E-01	2.44E-03	3.98E-01

2290	GSM393008	GSE21349	Multiple Myeloma	Blood	1.79E-02	-1.59E-01	1.12E-01	2.40E-01
2291	GSM393009	GSE21349	Multiple Myeloma	Blood	1.88E-02	1.58E-01	2.75E-03	3.94E-01
2292	GSM393010	GSE21349	Multiple Myeloma	Blood	7.01E-02	-1.29E-01	4.55E-02	2.85E-01
2293	GSM393011	GSE21349	Multiple Myeloma	Blood	7.97E-04	-2.11E-01	2.02E-01	2.05E-01
2294	GSM393012	GSE21349	Multiple Myeloma	Blood	2.49E-01	-9.33E-02	1.98E-01	2.07E-01
2295	GSM393013	GSE21349	Multiple Myeloma	Blood	3.67E-02	-1.44E-01	8.56E-02	2.54E-01
2296	GSM393014	GSE21349	Multiple Myeloma	Blood	3.67E-02	-1.44E-01	5.54E-02	2.76E-01
2297	GSM393015	GSE21349	Multiple Myeloma	Blood	4.03E-01	7.54E-02	3.30E-02	3.00E-01
2298	GSM393016	GSE21349	Multiple Myeloma	Blood	6.48E-03	-1.78E-01	2.32E-01	1.96E-01
2299	GSM538443	GSE21349	Multiple Myeloma	Blood	3.34E-02	-1.46E-01	2.39E-01	-1.94E-01
2300	GSM538444	GSE21349	Multiple Myeloma	Blood	2.29E-02	1.54E-01	2.75E-03	3.94E-01
2301	GSM538445	GSE21349	Multiple Myeloma	Blood	8.57E-02	-1.24E-01	1.96E-01	2.07E-01
2302	GSM538446	GSE21349	Multiple Myeloma	Blood	2.01E-01	1.00E-01	5.39E-02	2.77E-01
2303	GSM538447	GSE21349	Multiple Myeloma	Blood	1.77E-01	-1.04E-01	4.55E-02	2.85E-01
2304	GSM538448	GSE21349	Multiple Myeloma	Blood	2.64E-02	-1.51E-01	2.26E-01	1.98E-01
2305	GSM538449	GSE21349	Multiple Myeloma	Blood	1.00E-01	-1.20E-01	2.19E-01	2.00E-01
2306	GSM538450	GSE21349	Multiple Myeloma	Blood	4.95E-04	2.18E-01	2.35E-03	3.99E-01
2307	GSM538451	GSE21349	Multiple Myeloma	Blood	2.77E-02	-1.50E-01	9.23E-02	-2.50E-01
2308	GSM538452	GSE21349	Multiple Myeloma	Blood	1.77E-01	-1.04E-01	3.78E-01	1.60E-01
2309	GSM538453	GSE21349	Multiple Myeloma	Blood	5.23E-02	-1.36E-01	3.16E-02	3.02E-01
2310	GSM493958	GSE19784	Multiple Myeloma	Blood	6.73E-02	-1.30E-01	1.75E-01	2.14E-01
2311	GSM493959	GSE19784	Multiple Myeloma	Blood	5.23E-02	-1.36E-01	5.31E-02	2.78E-01
2312	GSM493960	GSE19784	Multiple Myeloma	Blood	1.77E-01	1.04E-01	2.55E-02	3.11E-01
2313	GSM493961	GSE19784	Multiple Myeloma	Blood	2.88E-01	8.83E-02	2.08E-02	3.19E-01
2314	GSM493962	GSE19784	Multiple Myeloma	Blood	3.74E-04	-2.22E-01	5.54E-02	-2.76E-01
2315	GSM493963	GSE19784	Multiple Myeloma	Blood	5.79E-03	1.80E-01	2.15E-02	3.18E-01
2316	GSM493964	GSE19784	Multiple Myeloma	Blood	7.92E-02	-1.26E-01	1.12E-01	2.40E-01
2317	GSM493965	GSE19784	Multiple Myeloma	Blood	2.10E-03	-1.96E-01	1.71E-01	-2.16E-01
2318	GSM493966	GSE19784	Multiple Myeloma	Blood	7.67E-03	-1.75E-01	1.51E-01	-2.23E-01
2319	GSM493967	GSE19784	Multiple Myeloma	Blood	4.34E-03	-1.85E-01	1.60E-01	2.20E-01
2320	GSM493968	GSE19784	Multiple Myeloma	Blood	6.97E-04	-2.13E-01	1.32E-02	-3.38E-01
2321	GSM493969	GSE19784	Multiple Myeloma	Blood	1.97E-02	-1.57E-01	3.32E-01	1.70E-01
2322	GSM493970	GSE19784	Multiple Myeloma	Blood	1.01E-02	-1.70E-01	2.58E-01	1.89E-01
2323	GSM493971	GSE19784	Multiple Myeloma	Blood	1.16E-01	-1.16E-01	5.54E-02	2.76E-01
2324	GSM493972	GSE19784	Multiple Myeloma	Blood	4.20E-02	-1.41E-01	1.02E-01	2.45E-01
2325	GSM493973	GSE19784	Multiple Myeloma	Blood	2.29E-02	1.54E-01	8.70E-04	4.31E-01
2326	GSM493974	GSE19784	Multiple Myeloma	Blood	1.46E-02	-1.63E-01	1.50E-02	3.32E-01
2327	GSM493975	GSE19784	Multiple Myeloma	Blood	1.16E-01	1.16E-01	7.70E-03	3.58E-01
2328	GSM493976	GSE19784	Multiple Myeloma	Blood	7.60E-02	-1.27E-01	3.16E-02	3.02E-01
2329	GSM493977	GSE19784	Multiple Myeloma	Blood	4.34E-03	-1.85E-01	1.43E-02	3.34E-01
2330	GSM493978	GSE19784	Multiple Myeloma	Blood	1.16E-01	1.16E-01	6.43E-02	2.69E-01
2331	GSM493979	GSE19784	Multiple Myeloma	Blood	5.01E-02	-1.37E-01	4.88E-03	3.74E-01
2332	GSM493980	GSE19784	Multiple Myeloma	Blood	4.09E-03	-1.86E-01	8.35E-02	-2.56E-01
2333	GSM493981	GSE19784	Multiple Myeloma	Blood	1.01E-02	-1.70E-01	7.70E-03	3.58E-01
2334	GSM493982	GSE19784	Multiple Myeloma	Blood	2.52E-02	-1.52E-01	3.72E-02	2.94E-01
2335	GSM493983	GSE19784	Multiple Myeloma	Blood	2.08E-01	9.92E-02	2.44E-03	3.98E-01
2336	GSM493984	GSE19784	Multiple Myeloma	Blood	1.65E-01	-1.06E-01	5.31E-02	2.78E-01
2337	GSM493985	GSE19784	Multiple Myeloma	Blood	7.60E-02	-1.27E-01	8.89E-02	2.52E-01
2338	GSM493986	GSE19784	Multiple Myeloma	Blood	5.23E-02	1.36E-01	2.44E-03	3.98E-01
2339	GSM493987	GSE19784	Multiple Myeloma	Blood	2.14E-01	9.82E-02	8.03E-02	2.58E-01
2340	GSM493988	GSE19784	Multiple Myeloma	Blood	1.00E-01	1.20E-01	1.43E-02	3.34E-01
2341	GSM493989	GSE19784	Multiple Myeloma	Blood	2.53E-03	-1.93E-01	2.55E-02	3.11E-01
2342	GSM493990	GSE19784	Multiple Myeloma	Blood	4.01E-02	-1.42E-01	1.96E-03	4.05E-01
2343	GSM493991	GSE19784	Multiple Myeloma	Blood	1.57E-04	-2.34E-01	7.43E-02	2.62E-01
2344	GSM493992	GSE19784	Multiple Myeloma	Blood	4.20E-02	-1.41E-01	1.16E-01	2.38E-01
2345	GSM493993	GSE19784	Multiple Myeloma	Blood	6.81E-05	-2.45E-01	8.03E-02	-2.58E-01
2346	GSM493994	GSE19784	Multiple Myeloma	Blood	4.59E-02	1.39E-01	5.16E-03	3.72E-01
2347	GSM493995	GSE19784	Multiple Myeloma	Blood	4.95E-04	-2.18E-01	1.14E-01	-2.39E-01
2348	GSM493996	GSE19784	Multiple Myeloma	Blood	9.26E-02	-1.22E-01	1.17E-03	4.22E-01
2349	GSM493997	GSE19784	Multiple Myeloma	Blood	8.91E-02	-1.23E-01	3.55E-01	1.65E-01
2350	GSM493998	GSE19784	Multiple Myeloma	Blood	9.54E-03	-1.71E-01	1.80E-01	2.12E-01
2351	GSM493999	GSE19784	Multiple Myeloma	Blood	2.86E-03	-1.91E-01	1.75E-01	2.14E-01
2352	GSM494000	GSE19784	Multiple Myeloma	Blood	7.25E-03	1.76E-01	1.50E-02	3.32E-01
2353	GSM494001	GSE19784	Multiple Myeloma	Blood	5.23E-02	1.36E-01	6.31E-03	3.65E-01
2354	GSM494002	GSE19784	Multiple Myeloma	Blood	2.64E-02	-1.51E-01	1.80E-01	2.12E-01
2355	GSM494003	GSE19784	Multiple Myeloma	Blood	1.89E-01	1.02E-01	3.97E-03	3.82E-01
2356	GSM494004	GSE19784	Multiple Myeloma	Blood	4.60E-03	-1.84E-01	1.51E-01	-2.23E-01
2357	GSM494005	GSE19784	Multiple Myeloma	Blood	4.20E-02	-1.41E-01	1.55E-01	2.22E-01
2358	GSM494006	GSE19784	Multiple Myeloma	Blood	7.60E-02	-1.27E-01	2.44E-03	3.98E-01
2359	GSM494007	GSE19784	Multiple Myeloma	Blood	3.04E-01	-8.63E-02	6.31E-03	3.65E-01
2360	GSM494008	GSE19784	Multiple Myeloma	Blood	3.05E-02	-1.48E-01	1.19E-02	3.42E-01
2361	GSM494009	GSE19784	Multiple Myeloma	Blood	2.64E-02	-1.51E-01	5.16E-03	3.72E-01
2362	GSM494010	GSE19784	Multiple Myeloma	Blood	4.98E-05	-2.49E-01	4.24E-02	-2.89E-01
2363	GSM494011	GSE19784	Multiple Myeloma	Blood	2.10E-03	-1.96E-01	3.72E-02	2.94E-01

2364	GSM494012	GSE19784	Multiple Myeloma	Blood	2.08E-01	-9.92E-02	8.56E-02	2.54E-01
2365	GSM494013	GSE19784	Multiple Myeloma	Blood	3.23E-03	-1.89E-01	3.78E-01	1.60E-01
2366	GSM494014	GSE19784	Multiple Myeloma	Blood	2.29E-02	1.54E-01	8.70E-04	4.31E-01
2367	GSM494015	GSE19784	Multiple Myeloma	Blood	5.79E-03	-1.80E-01	2.25E-02	3.16E-01
2368	GSM494016	GSE19784	Multiple Myeloma	Blood	6.46E-02	-1.31E-01	2.55E-02	3.11E-01
2369	GSM494017	GSE19784	Multiple Myeloma	Blood	6.51E-04	-2.14E-01	2.51E-01	1.91E-01
2370	GSM494018	GSE19784	Multiple Myeloma	Blood	9.04E-03	-1.72E-01	1.75E-01	2.14E-01
2371	GSM494019	GSE19784	Multiple Myeloma	Blood	1.70E-02	-1.60E-01	6.17E-02	2.71E-01
2372	GSM494020	GSE19784	Multiple Myeloma	Blood	4.79E-02	-1.38E-01	4.36E-02	2.87E-01
2373	GSM494021	GSE19784	Multiple Myeloma	Blood	5.46E-02	-1.35E-01	1.37E-01	2.29E-01
2374	GSM494022	GSE19784	Multiple Myeloma	Blood	1.60E-01	-1.07E-01	8.70E-04	4.31E-01
2375	GSM494023	GSE19784	Multiple Myeloma	Blood	3.13E-01	-8.53E-02	1.80E-01	2.12E-01
2376	GSM494024	GSE19784	Multiple Myeloma	Blood	1.83E-01	1.03E-01	4.88E-03	3.74E-01
2377	GSM494025	GSE19784	Multiple Myeloma	Blood	1.39E-01	-1.11E-01	3.30E-02	3.00E-01
2378	GSM494026	GSE19784	Multiple Myeloma	Blood	1.63E-03	-2.00E-01	1.43E-02	3.34E-01
2379	GSM494027	GSE19784	Multiple Myeloma	Blood	1.12E-01	1.17E-01	1.80E-02	3.25E-01
2380	GSM494028	GSE19784	Multiple Myeloma	Blood	9.04E-03	-1.72E-01	1.80E-02	3.25E-01
2381	GSM494029	GSE19784	Multiple Myeloma	Blood	1.88E-02	-1.58E-01	1.16E-01	2.38E-01
2382	GSM494030	GSE19784	Multiple Myeloma	Blood	9.26E-02	1.22E-01	8.12E-03	3.56E-01
2383	GSM494031	GSE19784	Multiple Myeloma	Blood	1.65E-01	-1.06E-01	1.32E-01	2.31E-01
2384	GSM494032	GSE19784	Multiple Myeloma	Blood	9.54E-03	-1.71E-01	1.16E-01	2.38E-01
2385	GSM494033	GSE19784	Multiple Myeloma	Blood	2.53E-03	-1.93E-01	3.72E-02	2.94E-01
2386	GSM494034	GSE19784	Multiple Myeloma	Blood	1.97E-02	-1.57E-01	7.43E-02	2.62E-01
2387	GSM494035	GSE19784	Multiple Myeloma	Blood	1.71E-01	1.05E-01	7.70E-03	3.58E-01
2388	GSM494036	GSE19784	Multiple Myeloma	Blood	1.89E-01	1.02E-01	6.69E-02	2.67E-01
2389	GSM494037	GSE19784	Multiple Myeloma	Blood	4.23E-01	-7.34E-02	2.19E-01	2.00E-01
2390	GSM494038	GSE19784	Multiple Myeloma	Blood	2.40E-02	1.53E-01	2.59E-03	3.96E-01
2391	GSM494039	GSE19784	Multiple Myeloma	Blood	1.44E-03	-2.02E-01	3.46E-01	1.67E-01
2392	GSM494040	GSE19784	Multiple Myeloma	Blood	2.77E-02	1.50E-01	3.21E-03	3.89E-01
2393	GSM494041	GSE19784	Multiple Myeloma	Blood	1.06E-02	-1.69E-01	1.55E-01	2.22E-01
2394	GSM494042	GSE19784	Multiple Myeloma	Blood	2.52E-02	-1.52E-01	1.80E-01	2.12E-01
2395	GSM494043	GSE19784	Multiple Myeloma	Blood	1.79E-02	1.59E-01	1.25E-02	3.40E-01
2396	GSM494044	GSE19784	Multiple Myeloma	Blood	9.11E-04	-2.09E-01	1.75E-01	2.14E-01
2397	GSM494045	GSE19784	Multiple Myeloma	Blood	3.19E-02	-1.47E-01	1.17E-03	4.22E-01
2398	GSM494046	GSE19784	Multiple Myeloma	Blood	2.38E-03	-1.94E-01	4.75E-02	2.83E-01
2399	GSM494047	GSE19784	Multiple Myeloma	Blood	1.60E-05	-2.63E-01	7.43E-02	2.62E-01
2400	GSM494048	GSE19784	Multiple Myeloma	Blood	1.46E-02	-1.63E-01	1.10E-01	2.41E-01
2401	GSM494049	GSE19784	Multiple Myeloma	Blood	2.44E-04	-2.28E-01	8.56E-02	2.54E-01
2402	GSM494050	GSE19784	Multiple Myeloma	Blood	3.03E-04	-2.25E-01	3.72E-02	2.94E-01
2403	GSM494051	GSE19784	Multiple Myeloma	Blood	3.50E-02	-1.45E-01	7.43E-02	2.62E-01
2404	GSM494052	GSE19784	Multiple Myeloma	Blood	3.05E-02	-1.48E-01	1.65E-01	-2.18E-01
2405	GSM494053	GSE19784	Multiple Myeloma	Blood	3.63E-03	-1.88E-01	1.02E-01	2.45E-01
2406	GSM494054	GSE19784	Multiple Myeloma	Blood	2.91E-02	1.49E-01	4.21E-04	4.52E-01
2407	GSM494055	GSE19784	Multiple Myeloma	Blood	1.04E-01	-1.19E-01	4.06E-02	2.90E-01
2408	GSM494056	GSE19784	Multiple Myeloma	Blood	9.63E-02	1.21E-01	4.20E-03	3.80E-01
2409	GSM494057	GSE19784	Multiple Myeloma	Blood	7.01E-02	-1.29E-01	9.23E-02	2.50E-01
2410	GSM494058	GSE19784	Multiple Myeloma	Blood	1.04E-01	-1.19E-01	3.72E-02	2.94E-01
2411	GSM494059	GSE19784	Multiple Myeloma	Blood	4.39E-02	-1.40E-01	6.17E-02	2.71E-01
2412	GSM494060	GSE19784	Multiple Myeloma	Blood	4.01E-02	-1.42E-01	9.85E-03	3.49E-01
2413	GSM494061	GSE19784	Multiple Myeloma	Blood	5.69E-02	-1.34E-01	1.43E-02	3.34E-01
2414	GSM494062	GSE19784	Multiple Myeloma	Blood	1.25E-01	-1.14E-01	8.56E-02	2.54E-01
2415	GSM494063	GSE19784	Multiple Myeloma	Blood	8.91E-02	-1.23E-01	1.37E-01	2.29E-01
2416	GSM494064	GSE19784	Multiple Myeloma	Blood	4.01E-02	-1.42E-01	1.02E-01	2.45E-01
2417	GSM494065	GSE19784	Multiple Myeloma	Blood	3.19E-02	-1.47E-01	1.96E-01	2.07E-01
2418	GSM494066	GSE19784	Multiple Myeloma	Blood	9.04E-03	-1.72E-01	1.16E-01	2.38E-01
2419	GSM494067	GSE19784	Multiple Myeloma	Blood	1.65E-01	1.06E-01	2.08E-03	4.03E-01
2420	GSM494068	GSE19784	Multiple Myeloma	Blood	1.01E-02	-1.70E-01	6.69E-02	2.67E-01
2421	GSM494069	GSE19784	Multiple Myeloma	Blood	6.19E-02	-1.32E-01	2.75E-03	3.94E-01
2422	GSM494070	GSE19784	Multiple Myeloma	Blood	7.30E-02	1.28E-01	3.21E-03	3.89E-01
2423	GSM494071	GSE19784	Multiple Myeloma	Blood	4.01E-02	-1.42E-01	6.17E-02	2.71E-01
2424	GSM494072	GSE19784	Multiple Myeloma	Blood	1.21E-01	-1.15E-01	7.43E-02	2.62E-01
2425	GSM494073	GSE19784	Multiple Myeloma	Blood	1.00E-01	-1.20E-01	6.31E-03	3.65E-01
2426	GSM494074	GSE19784	Multiple Myeloma	Blood	1.83E-01	1.03E-01	1.19E-02	3.42E-01
2427	GSM494075	GSE19784	Multiple Myeloma	Blood	2.42E-05	-2.58E-01	2.46E-01	-1.92E-01
2428	GSM494076	GSE19784	Multiple Myeloma	Blood	3.13E-01	8.53E-02	5.77E-02	2.74E-01
2429	GSM494077	GSE19784	Multiple Myeloma	Blood	1.14E-06	-2.93E-01	7.24E-02	-2.63E-01
2430	GSM494078	GSE19784	Multiple Myeloma	Blood	1.44E-01	1.10E-01	2.15E-02	3.18E-01
2431	GSM494079	GSE19784	Multiple Myeloma	Blood	3.42E-03	-1.88E-01	1.32E-01	2.31E-01
2432	GSM494080	GSE19784	Multiple Myeloma	Blood	1.01E-02	-1.70E-01	7.43E-02	2.62E-01
2433	GSM494081	GSE19784	Multiple Myeloma	Blood	3.47E-01	8.13E-02	1.25E-03	4.20E-01
2434	GSM494082	GSE19784	Multiple Myeloma	Blood	3.23E-03	-1.89E-01	2.26E-01	1.98E-01
2435	GSM494083	GSE19784	Multiple Myeloma	Blood	1.97E-02	-1.57E-01	1.98E-01	-2.07E-01
2436	GSM494084	GSE19784	Multiple Myeloma	Blood	1.04E-03	-2.07E-01	2.28E-01	-1.97E-01
2437	GSM494085	GSE19784	Multiple Myeloma	Blood	5.30E-04	-2.17E-01	2.94E-01	-1.80E-01

2438	GSM494086	GSE19784	Multiple Myeloma	Blood	1.25E-02	1.66E-01	5.38E-04	4.45E-01
2439	GSM494087	GSE19784	Multiple Myeloma	Blood	1.12E-02	-1.68E-01	1.21E-01	2.36E-01
2440	GSM494088	GSE19784	Multiple Myeloma	Blood	2.64E-01	-9.13E-02	5.38E-04	4.45E-01
2441	GSM494089	GSE19784	Multiple Myeloma	Blood	9.11E-04	-2.09E-01	3.46E-01	1.67E-01
2442	GSM494090	GSE19784	Multiple Myeloma	Blood	5.01E-02	1.37E-01	3.30E-02	3.00E-01
2443	GSM494091	GSE19784	Multiple Myeloma	Blood	1.44E-01	-1.10E-01	1.92E-01	2.09E-01
2444	GSM494092	GSE19784	Multiple Myeloma	Blood	6.48E-03	-1.78E-01	8.89E-02	2.52E-01
2445	GSM494093	GSE19784	Multiple Myeloma	Blood	1.97E-03	-1.97E-01	6.17E-02	2.71E-01
2446	GSM494094	GSE19784	Multiple Myeloma	Blood	2.24E-03	-1.95E-01	2.80E-02	-3.07E-01
2447	GSM494095	GSE19784	Multiple Myeloma	Blood	4.01E-02	1.42E-01	3.06E-04	4.62E-01
2448	GSM494096	GSE19784	Multiple Myeloma	Blood	6.08E-04	-2.15E-01	1.32E-01	2.31E-01
2449	GSM494097	GSE19784	Multiple Myeloma	Blood	6.48E-03	-1.78E-01	2.02E-01	2.05E-01
2450	GSM494098	GSE19784	Multiple Myeloma	Blood	2.77E-02	-1.50E-01	6.31E-03	3.65E-01
2451	GSM494099	GSE19784	Multiple Myeloma	Blood	3.05E-02	-1.48E-01	2.19E-01	2.00E-01
2452	GSM494100	GSE19784	Multiple Myeloma	Blood	1.00E-01	1.20E-01	3.89E-02	2.92E-01
2453	GSM494101	GSE19784	Multiple Myeloma	Blood	2.24E-03	-1.95E-01	2.86E-01	1.82E-01
2454	GSM494102	GSE19784	Multiple Myeloma	Blood	1.83E-01	-1.03E-01	1.78E-01	2.13E-01
2455	GSM494103	GSE19784	Multiple Myeloma	Blood	1.65E-01	1.06E-01	3.40E-03	3.87E-01
2456	GSM494104	GSE19784	Multiple Myeloma	Blood	1.44E-01	1.10E-01	2.55E-02	3.11E-01
2457	GSM494105	GSE19784	Multiple Myeloma	Blood	2.64E-01	9.13E-02	3.46E-02	2.98E-01
2458	GSM494106	GSE19784	Multiple Myeloma	Blood	1.88E-02	-1.58E-01	1.42E-01	2.27E-01
2459	GSM494107	GSE19784	Multiple Myeloma	Blood	6.13E-03	-1.79E-01	2.19E-01	2.00E-01
2460	GSM494108	GSE19784	Multiple Myeloma	Blood	1.70E-02	-1.60E-01	3.16E-02	3.02E-01
2461	GSM494109	GSE19784	Multiple Myeloma	Blood	6.86E-03	-1.77E-01	1.12E-01	2.40E-01
2462	GSM494110	GSE19784	Multiple Myeloma	Blood	8.57E-02	-1.24E-01	6.43E-02	2.69E-01
2463	GSM494111	GSE19784	Multiple Myeloma	Blood	1.83E-01	1.03E-01	4.55E-02	2.85E-01
2464	GSM494112	GSE19784	Multiple Myeloma	Blood	5.01E-02	-1.37E-01	7.73E-02	2.60E-01
2465	GSM494113	GSE19784	Multiple Myeloma	Blood	9.04E-03	-1.72E-01	2.15E-02	3.18E-01
2466	GSM494114	GSE19784	Multiple Myeloma	Blood	3.74E-04	-2.22E-01	2.25E-02	3.16E-01
2467	GSM494115	GSE19784	Multiple Myeloma	Blood	2.18E-02	-1.55E-01	4.55E-02	2.85E-01
2468	GSM494116	GSE19784	Multiple Myeloma	Blood	4.39E-02	-1.40E-01	1.50E-02	3.32E-01
2469	GSM494117	GSE19784	Multiple Myeloma	Blood	2.21E-01	-9.72E-02	6.17E-02	2.71E-01
2470	GSM494118	GSE19784	Multiple Myeloma	Blood	6.46E-02	1.31E-01	3.93E-04	4.54E-01
2471	GSM494119	GSE19784	Multiple Myeloma	Blood	4.59E-02	-1.39E-01	3.16E-02	3.02E-01
2472	GSM494120	GSE19784	Multiple Myeloma	Blood	8.57E-02	1.24E-01	2.55E-02	3.11E-01
2473	GSM494121	GSE19784	Multiple Myeloma	Blood	8.24E-02	-1.25E-01	3.30E-02	3.00E-01
2474	GSM494122	GSE19784	Multiple Myeloma	Blood	2.62E-04	-2.27E-01	2.47E-02	-3.12E-01
2475	GSM494123	GSE19784	Multiple Myeloma	Blood	3.34E-02	-1.46E-01	1.37E-01	2.29E-01
2476	GSM494124	GSE19784	Multiple Myeloma	Blood	1.60E-01	-1.07E-01	6.78E-02	2.66E-01
2477	GSM494125	GSE19784	Multiple Myeloma	Blood	2.21E-01	-9.72E-02	4.96E-02	2.81E-01
2478	GSM494126	GSE19784	Multiple Myeloma	Blood	4.31E-04	-2.20E-01	1.46E-01	-2.25E-01
2479	GSM494127	GSE19784	Multiple Myeloma	Blood	1.60E-01	1.07E-01	2.55E-02	3.11E-01
2480	GSM494128	GSE19784	Multiple Myeloma	Blood	4.79E-02	-1.38E-01	3.97E-03	3.82E-01
2481	GSM494129	GSE19784	Multiple Myeloma	Blood	1.16E-01	-1.16E-01	2.19E-01	2.00E-01
2482	GSM494130	GSE19784	Multiple Myeloma	Blood	2.72E-01	-9.03E-02	2.15E-02	3.18E-01
2483	GSM494131	GSE19784	Multiple Myeloma	Blood	1.70E-02	-1.60E-01	1.16E-01	2.38E-01
2484	GSM494132	GSE19784	Multiple Myeloma	Blood	4.60E-03	-1.84E-01	1.96E-01	2.07E-01
2485	GSM494133	GSE19784	Multiple Myeloma	Blood	1.18E-02	-1.67E-01	1.21E-01	2.36E-01
2486	GSM494134	GSE19784	Multiple Myeloma	Blood	9.27E-05	-2.41E-01	1.60E-01	-2.20E-01
2487	GSM494135	GSE19784	Multiple Myeloma	Blood	1.34E-01	1.12E-01	9.89E-04	4.27E-01
2488	GSM494136	GSE19784	Multiple Myeloma	Blood	9.63E-02	-1.21E-01	2.91E-01	1.80E-01
2489	GSM494137	GSE19784	Multiple Myeloma	Blood	4.25E-05	-2.51E-01	2.53E-01	-1.90E-01
2490	GSM494138	GSE19784	Multiple Myeloma	Blood	1.61E-02	-1.61E-01	8.89E-02	2.52E-01
2491	GSM494139	GSE19784	Multiple Myeloma	Blood	3.05E-02	-1.48E-01	1.88E-01	2.10E-01
2492	GSM494140	GSE19784	Multiple Myeloma	Blood	1.79E-02	-1.59E-01	1.16E-01	2.38E-01
2493	GSM494141	GSE19784	Multiple Myeloma	Blood	1.04E-01	1.19E-01	3.61E-02	2.96E-01
2494	GSM494142	GSE19784	Multiple Myeloma	Blood	2.77E-02	1.50E-01	1.67E-03	4.10E-01
2495	GSM494143	GSE19784	Multiple Myeloma	Blood	1.38E-02	-1.64E-01	3.49E-01	-1.67E-01
2496	GSM494144	GSE19784	Multiple Myeloma	Blood	1.61E-02	-1.61E-01	4.06E-02	2.90E-01
2497	GSM494145	GSE19784	Multiple Myeloma	Blood	1.69E-04	-2.33E-01	4.43E-02	-2.87E-01
2498	GSM494146	GSE19784	Multiple Myeloma	Blood	6.46E-02	-1.31E-01	1.02E-01	2.45E-01
2499	GSM494147	GSE19784	Multiple Myeloma	Blood	6.73E-02	-1.30E-01	1.86E-01	2.10E-01
2500	GSM494148	GSE19784	Multiple Myeloma	Blood	1.83E-01	1.03E-01	4.36E-02	2.87E-01
2501	GSM494149	GSE19784	Multiple Myeloma	Blood	1.75E-08	3.34E-01	3.57E-05	5.19E-01
2502	GSM494150	GSE19784	Multiple Myeloma	Blood	4.03E-07	-3.04E-01	5.76E-03	-3.69E-01
2503	GSM494151	GSE19784	Multiple Myeloma	Blood	6.86E-03	-1.77E-01	3.89E-02	2.92E-01
2504	GSM494152	GSE19784	Multiple Myeloma	Blood	1.71E-01	1.05E-01	1.09E-02	3.45E-01
2505	GSM494153	GSE19784	Multiple Myeloma	Blood	2.64E-02	-1.51E-01	1.80E-01	2.12E-01
2506	GSM494154	GSE19784	Multiple Myeloma	Blood	1.34E-01	1.12E-01	2.44E-03	3.98E-01
2507	GSM494155	GSE19784	Multiple Myeloma	Blood	2.49E-01	9.33E-02	1.12E-01	2.40E-01
2508	GSM494156	GSE19784	Multiple Myeloma	Blood	9.04E-03	-1.72E-01	2.55E-02	3.11E-01
2509	GSM494157	GSE19784	Multiple Myeloma	Blood	1.71E-01	-1.05E-01	5.16E-03	3.72E-01
2510	GSM494158	GSE19784	Multiple Myeloma	Blood	7.60E-02	-1.27E-01	1.50E-02	3.32E-01
2511	GSM494159	GSE19784	Multiple Myeloma	Blood	1.95E-01	-1.01E-01	3.72E-02	2.94E-01

2512	GSM494160	GSE19784	Multiple Myeloma	Blood	2.38E-03	-1.94E-01	9.82E-02	2.47E-01
2513	GSM494161	GSE19784	Multiple Myeloma	Blood	1.30E-01	-1.13E-01	9.34E-03	3.51E-01
2514	GSM494162	GSE19784	Multiple Myeloma	Blood	3.19E-02	-1.47E-01	3.72E-02	2.94E-01
2515	GSM494163	GSE19784	Multiple Myeloma	Blood	3.86E-03	-1.87E-01	2.04E-01	-2.05E-01
2516	GSM494164	GSE19784	Multiple Myeloma	Blood	4.59E-02	1.39E-01	7.70E-03	3.58E-01
2517	GSM494165	GSE19784	Multiple Myeloma	Blood	1.61E-02	-1.61E-01	2.58E-01	1.89E-01
2518	GSM494166	GSE19784	Multiple Myeloma	Blood	1.70E-02	-1.60E-01	2.26E-01	1.98E-01
2519	GSM494167	GSE19784	Multiple Myeloma	Blood	2.86E-03	-1.91E-01	1.02E-01	2.45E-01
2520	GSM494168	GSE19784	Multiple Myeloma	Blood	1.60E-01	1.07E-01	4.50E-04	4.50E-01
2521	GSM494169	GSE19784	Multiple Myeloma	Blood	2.14E-01	9.82E-02	6.67E-03	3.63E-01
2522	GSM494170	GSE19784	Multiple Myeloma	Blood	5.23E-02	-1.36E-01	3.72E-02	2.94E-01
2523	GSM494171	GSE19784	Multiple Myeloma	Blood	9.63E-02	-1.21E-01	1.50E-02	3.32E-01
2524	GSM494172	GSE19784	Multiple Myeloma	Blood	3.19E-02	1.47E-01	4.21E-04	4.52E-01
2525	GSM494173	GSE19784	Multiple Myeloma	Blood	2.72E-01	9.03E-02	2.32E-01	1.96E-01
2526	GSM494174	GSE19784	Multiple Myeloma	Blood	4.01E-02	-1.42E-01	1.37E-01	2.29E-01
2527	GSM494175	GSE19784	Multiple Myeloma	Blood	8.10E-03	-1.74E-01	1.42E-01	-2.27E-01
2528	GSM494176	GSE19784	Multiple Myeloma	Blood	1.26E-04	-2.37E-01	2.86E-01	1.82E-01
2529	GSM494177	GSE19784	Multiple Myeloma	Blood	1.12E-01	1.17E-01	3.30E-02	3.00E-01
2530	GSM494178	GSE19784	Multiple Myeloma	Blood	1.44E-01	1.10E-01	5.16E-03	3.72E-01
2531	GSM494179	GSE19784	Multiple Myeloma	Blood	1.18E-03	-2.05E-01	2.08E-01	-2.03E-01
2532	GSM494180	GSE19784	Multiple Myeloma	Blood	1.12E-02	-1.68E-01	3.78E-01	1.60E-01
2533	GSM494181	GSE19784	Multiple Myeloma	Blood	2.28E-01	-9.62E-02	2.08E-01	2.03E-01
2534	GSM494182	GSE19784	Multiple Myeloma	Blood	1.04E-01	-1.19E-01	6.17E-02	2.71E-01
2535	GSM494183	GSE19784	Multiple Myeloma	Blood	2.29E-02	-1.54E-01	1.75E-01	2.14E-01
2536	GSM494184	GSE19784	Multiple Myeloma	Blood	1.61E-02	-1.61E-01	1.96E-01	2.07E-01
2537	GSM494185	GSE19784	Multiple Myeloma	Blood	2.52E-02	-1.52E-01	1.32E-01	2.31E-01
2538	GSM494186	GSE19784	Multiple Myeloma	Blood	5.47E-03	-1.81E-01	5.31E-02	2.78E-01
2539	GSM494187	GSE19784	Multiple Myeloma	Blood	6.48E-03	-1.78E-01	2.02E-01	2.05E-01
2540	GSM494188	GSE19784	Multiple Myeloma	Blood	4.60E-03	-1.84E-01	3.49E-01	-1.67E-01
2541	GSM494189	GSE19784	Multiple Myeloma	Blood	1.97E-03	-1.97E-01	1.71E-01	-2.16E-01
2542	GSM494190	GSE19784	Multiple Myeloma	Blood	6.19E-02	-1.32E-01	3.16E-02	3.02E-01
2543	GSM494191	GSE19784	Multiple Myeloma	Blood	1.79E-02	-1.59E-01	1.12E-01	2.40E-01
2544	GSM494192	GSE19784	Multiple Myeloma	Blood	9.54E-03	-1.71E-01	3.18E-01	-1.74E-01
2545	GSM494193	GSE19784	Multiple Myeloma	Blood	8.24E-02	-1.25E-01	9.82E-02	2.47E-01
2546	GSM494194	GSE19784	Multiple Myeloma	Blood	1.53E-03	-2.01E-01	1.96E-01	2.07E-01
2547	GSM494195	GSE19784	Multiple Myeloma	Blood	5.16E-03	-1.82E-01	1.12E-01	2.40E-01
2548	GSM494196	GSE19784	Multiple Myeloma	Blood	1.53E-03	-2.01E-01	3.55E-01	1.65E-01
2549	GSM494197	GSE19784	Multiple Myeloma	Blood	1.61E-02	-1.61E-01	3.46E-01	1.67E-01
2550	GSM494198	GSE19784	Multiple Myeloma	Blood	1.06E-02	-1.69E-01	1.16E-01	2.38E-01
2551	GSM494199	GSE19784	Multiple Myeloma	Blood	6.97E-04	-2.13E-01	8.35E-02	-2.56E-01
2552	GSM494200	GSE19784	Multiple Myeloma	Blood	1.34E-01	-1.12E-01	7.43E-02	2.62E-01
2553	GSM494201	GSE19784	Multiple Myeloma	Blood	2.18E-02	-1.55E-01	1.55E-01	2.22E-01
2554	GSM494202	GSE19784	Multiple Myeloma	Blood	7.30E-02	1.28E-01	9.85E-03	3.49E-01
2555	GSM494203	GSE19784	Multiple Myeloma	Blood	4.01E-02	1.42E-01	1.16E-01	2.38E-01
2556	GSM494204	GSE19784	Multiple Myeloma	Blood	3.84E-02	-1.43E-01	8.56E-02	2.54E-01
2557	GSM494205	GSE19784	Multiple Myeloma	Blood	3.34E-02	1.46E-01	1.57E-03	4.12E-01
2558	GSM494206	GSE19784	Multiple Myeloma	Blood	2.35E-01	-9.52E-02	1.06E-01	2.43E-01
2559	GSM494207	GSE19784	Multiple Myeloma	Blood	1.65E-01	-1.06E-01	9.85E-03	3.49E-01
2560	GSM494208	GSE19784	Multiple Myeloma	Blood	2.08E-01	9.92E-02	6.69E-02	2.67E-01
2561	GSM494209	GSE19784	Multiple Myeloma	Blood	8.24E-02	-1.25E-01	6.43E-02	2.69E-01
2562	GSM494210	GSE19784	Multiple Myeloma	Blood	1.61E-02	-1.61E-01	1.37E-01	2.29E-01
2563	GSM494211	GSE19784	Multiple Myeloma	Blood	3.84E-02	-1.43E-01	3.32E-01	-1.70E-01
2564	GSM494212	GSE19784	Multiple Myeloma	Blood	1.04E-01	-1.19E-01	3.72E-02	2.94E-01
2565	GSM494213	GSE19784	Multiple Myeloma	Blood	5.69E-02	1.34E-01	5.16E-03	3.72E-01
2566	GSM494214	GSE19784	Multiple Myeloma	Blood	4.79E-02	-1.38E-01	1.25E-02	3.40E-01
2567	GSM494215	GSE19784	Multiple Myeloma	Blood	4.20E-02	-1.41E-01	7.43E-02	2.62E-01
2568	GSM494216	GSE19784	Multiple Myeloma	Blood	2.91E-02	-1.49E-01	2.15E-02	3.18E-01
2569	GSM494217	GSE19784	Multiple Myeloma	Blood	9.04E-03	-1.72E-01	1.96E-01	2.07E-01
2570	GSM494218	GSE19784	Multiple Myeloma	Blood	1.65E-01	1.06E-01	1.19E-02	3.42E-01
2571	GSM494219	GSE19784	Multiple Myeloma	Blood	3.13E-01	8.53E-02	2.75E-03	3.94E-01
2572	GSM494220	GSE19784	Multiple Myeloma	Blood	5.23E-02	-1.36E-01	3.10E-01	-1.76E-01
2573	GSM494221	GSE19784	Multiple Myeloma	Blood	3.75E-01	7.84E-02	1.04E-02	3.47E-01
2574	GSM494222	GSE19784	Multiple Myeloma	Blood	1.11E-03	-2.06E-01	1.98E-01	-2.07E-01
2575	GSM494223	GSE19784	Multiple Myeloma	Blood	4.09E-03	-1.86E-01	1.80E-02	3.25E-01
2576	GSM494224	GSE19784	Multiple Myeloma	Blood	2.07E-02	-1.56E-01	3.89E-02	2.92E-01
2577	GSM494225	GSE19784	Multiple Myeloma	Blood	1.39E-01	1.11E-01	8.56E-03	3.54E-01
2578	GSM494226	GSE19784	Multiple Myeloma	Blood	7.60E-02	1.27E-01	5.16E-03	3.72E-01
2579	GSM494227	GSE19784	Multiple Myeloma	Blood	4.79E-02	-1.38E-01	7.73E-02	2.60E-01
2580	GSM494228	GSE19784	Multiple Myeloma	Blood	3.04E-03	-1.90E-01	7.43E-02	2.62E-01
2581	GSM494229	GSE19784	Multiple Myeloma	Blood	2.53E-03	-1.93E-01	1.96E-01	2.07E-01
2582	GSM494230	GSE19784	Multiple Myeloma	Blood	7.67E-03	-1.75E-01	1.96E-01	2.07E-01
2583	GSM494231	GSE19784	Multiple Myeloma	Blood	1.46E-02	-1.63E-01	2.19E-01	2.00E-01
2584	GSM494232	GSE19784	Multiple Myeloma	Blood	3.21E-01	8.43E-02	2.19E-01	2.00E-01
2585	GSM494233	GSE19784	Multiple Myeloma	Blood	1.01E-02	-1.70E-01	2.86E-01	1.82E-01

2586	GSM494234	GSE19784	Multiple Myeloma	Blood	2.38E-03	-1.94E-01	1.12E-01	2.40E-01
2587	GSM494235	GSE19784	Multiple Myeloma	Blood	1.82E-04	-2.32E-01	8.67E-02	-2.54E-01
2588	GSM494236	GSE19784	Multiple Myeloma	Blood	1.25E-02	-1.66E-01	1.25E-01	2.34E-01
2589	GSM494237	GSE19784	Multiple Myeloma	Blood	3.93E-05	-2.52E-01	2.94E-01	-1.80E-01
2590	GSM494238	GSE19784	Multiple Myeloma	Blood	1.35E-03	-2.03E-01	7.24E-02	-2.63E-01
2591	GSM494239	GSE19784	Multiple Myeloma	Blood	8.24E-02	1.25E-01	7.70E-03	3.58E-01
2592	GSM494240	GSE19784	Multiple Myeloma	Blood	1.04E-03	-2.07E-01	2.73E-01	-1.85E-01
2593	GSM494241	GSE19784	Multiple Myeloma	Blood	1.83E-01	1.03E-01	7.32E-04	4.36E-01
2594	GSM494242	GSE19784	Multiple Myeloma	Blood	1.25E-01	1.14E-01	5.75E-04	4.43E-01
2595	GSM494243	GSE19784	Multiple Myeloma	Blood	1.44E-01	-1.10E-01	6.86E-04	4.38E-01
2596	GSM494244	GSE19784	Multiple Myeloma	Blood	3.30E-01	8.33E-02	1.19E-02	3.42E-01
2597	GSM494245	GSE19784	Multiple Myeloma	Blood	9.26E-02	1.22E-01	8.12E-03	3.56E-01
2598	GSM494246	GSE19784	Multiple Myeloma	Blood	2.53E-03	-1.93E-01	8.56E-02	2.54E-01
2599	GSM494247	GSE19784	Multiple Myeloma	Blood	1.97E-02	-1.57E-01	5.31E-02	2.78E-01
2600	GSM494248	GSE19784	Multiple Myeloma	Blood	4.59E-02	-1.39E-01	1.32E-01	2.31E-01
2601	GSM494249	GSE19784	Multiple Myeloma	Blood	1.00E-01	-1.20E-01	5.54E-02	2.76E-01
2602	GSM494250	GSE19784	Multiple Myeloma	Blood	9.26E-02	-1.22E-01	2.55E-02	3.11E-01
2603	GSM494251	GSE19784	Multiple Myeloma	Blood	3.13E-01	8.53E-02	8.56E-03	3.54E-01
2604	GSM494252	GSE19784	Multiple Myeloma	Blood	3.13E-01	-8.53E-02	6.43E-02	2.69E-01
2605	GSM494253	GSE19784	Multiple Myeloma	Blood	7.25E-03	-1.76E-01	1.37E-01	2.29E-01
2606	GSM494254	GSE19784	Multiple Myeloma	Blood	1.77E-01	-1.04E-01	4.75E-02	2.83E-01
2607	GSM494255	GSE19784	Multiple Myeloma	Blood	4.02E-04	-2.21E-01	2.51E-01	1.91E-01
2608	GSM494256	GSE19784	Multiple Myeloma	Blood	7.67E-03	-1.75E-01	2.86E-01	1.82E-01
2609	GSM494257	GSE19784	Multiple Myeloma	Blood	1.82E-04	-2.32E-01	2.26E-01	1.98E-01
2610	GSM494258	GSE19784	Multiple Myeloma	Blood	2.40E-02	-1.53E-01	3.81E-01	-1.59E-01
2611	GSM494259	GSE19784	Multiple Myeloma	Blood	4.79E-02	-1.38E-01	2.08E-01	2.03E-01
2612	GSM494260	GSE19784	Multiple Myeloma	Blood	1.53E-02	-1.62E-01	9.82E-02	2.47E-01
2613	GSM494261	GSE19784	Multiple Myeloma	Blood	9.26E-02	-1.22E-01	6.17E-02	2.71E-01
2614	GSM494262	GSE19784	Multiple Myeloma	Blood	3.50E-02	-1.45E-01	1.75E-01	2.14E-01
2615	GSM494263	GSE19784	Multiple Myeloma	Blood	3.04E-03	-1.90E-01	1.12E-01	2.40E-01
2616	GSM494264	GSE19784	Multiple Myeloma	Blood	7.97E-04	-2.11E-01	7.24E-02	-2.63E-01
2617	GSM494265	GSE19784	Multiple Myeloma	Blood	4.34E-03	-1.85E-01	1.12E-01	2.40E-01
2618	GSM494266	GSE19784	Multiple Myeloma	Blood	1.69E-04	-2.33E-01	8.35E-02	-2.56E-01
2619	GSM494267	GSE19784	Multiple Myeloma	Blood	2.38E-03	-1.94E-01	9.94E-02	-2.47E-01
2620	GSM494268	GSE19784	Multiple Myeloma	Blood	2.80E-01	-8.93E-02	1.12E-01	2.40E-01
2621	GSM494269	GSE19784	Multiple Myeloma	Blood	1.11E-03	-2.06E-01	4.24E-02	-2.89E-01
2622	GSM494270	GSE19784	Multiple Myeloma	Blood	3.23E-03	-1.89E-01	3.78E-01	1.60E-01
2623	GSM494271	GSE19784	Multiple Myeloma	Blood	1.79E-02	-1.59E-01	7.73E-02	2.60E-01
2624	GSM494272	GSE19784	Multiple Myeloma	Blood	2.62E-04	-2.27E-01	1.71E-01	-2.16E-01
2625	GSM494273	GSE19784	Multiple Myeloma	Blood	2.96E-01	-8.73E-02	1.51E-01	2.23E-01
2626	GSM494274	GSE19784	Multiple Myeloma	Blood	2.64E-02	-1.51E-01	3.72E-02	2.94E-01
2627	GSM494275	GSE19784	Multiple Myeloma	Blood	2.72E-01	9.03E-02	3.46E-02	2.98E-01
2628	GSM494276	GSE19784	Multiple Myeloma	Blood	2.64E-01	-9.13E-02	4.75E-02	2.83E-01
2629	GSM494277	GSE19784	Multiple Myeloma	Blood	2.52E-02	-1.52E-01	2.08E-01	2.03E-01
2630	GSM147556	GSE6401	Multiple Myeloma	Blood	2.18E-02	-1.55E-01	1.55E-01	2.22E-01
2631	GSM147557	GSE6401	Multiple Myeloma	Blood	1.49E-01	-1.09E-01	1.17E-03	4.22E-01
2632	GSM147558	GSE6401	Multiple Myeloma	Blood	3.34E-02	-1.46E-01	2.66E-01	-1.87E-01
2633	GSM147559	GSE6401	Multiple Myeloma	Blood	1.25E-02	-1.66E-01	1.71E-01	-2.16E-01
2634	GSM147560	GSE6401	Multiple Myeloma	Blood	5.46E-02	1.35E-01	3.72E-02	2.94E-01
2635	GSM147561	GSE6401	Multiple Myeloma	Blood	1.30E-01	-1.13E-01	1.12E-01	2.40E-01
2636	GSM147562	GSE6401	Multiple Myeloma	Blood	4.79E-02	-1.38E-01	3.72E-02	2.94E-01
2637	GSM147563	GSE6401	Multiple Myeloma	Blood	3.34E-02	-1.46E-01	6.03E-01	-1.15E-01
2638	GSM147564	GSE6401	Multiple Myeloma	Blood	8.24E-02	-1.25E-01	1.75E-01	2.14E-01
2639	GSM147565	GSE6401	Multiple Myeloma	Blood	8.57E-02	-1.24E-01	6.17E-02	2.71E-01
2640	GSM147566	GSE6401	Multiple Myeloma	Blood	1.18E-02	-1.67E-01	1.75E-01	2.14E-01
2641	GSM147567	GSE6401	Multiple Myeloma	Blood	1.12E-02	-1.68E-01	1.12E-01	2.40E-01
2642	GSM147568	GSE6401	Multiple Myeloma	Blood	6.46E-02	-1.31E-01	7.73E-02	2.60E-01
2643	GSM147569	GSE6401	Multiple Myeloma	Blood	8.24E-02	-1.25E-01	1.50E-02	3.32E-01
2644	GSM147570	GSE6401	Multiple Myeloma	Blood	8.24E-02	1.25E-01	3.16E-02	3.02E-01
2645	GSM147571	GSE6401	Multiple Myeloma	Blood	1.61E-02	-1.61E-01	4.36E-02	2.87E-01
2646	GSM147572	GSE6401	Multiple Myeloma	Blood	2.21E-01	-9.72E-02	2.58E-01	1.89E-01
2647	GSM147573	GSE6401	Multiple Myeloma	Blood	4.01E-02	-1.42E-01	4.36E-02	2.87E-01
2648	GSM147574	GSE6401	Multiple Myeloma	Blood	2.52E-02	-1.52E-01	9.82E-02	2.47E-01
2649	GSM147575	GSE6401	Multiple Myeloma	Blood	2.69E-03	-1.92E-01	2.32E-01	1.96E-01
2650	GSM147576	GSE6401	Multiple Myeloma	Blood	4.01E-02	-1.42E-01	2.39E-01	1.94E-01
2651	GSM147577	GSE6401	Multiple Myeloma	Blood	3.34E-02	1.46E-01	2.44E-03	3.98E-01
2652	GSM147578	GSE6401	Multiple Myeloma	Blood	1.46E-02	1.63E-01	2.15E-02	3.18E-01
2653	GSM147579	GSE6401	Multiple Myeloma	Blood	1.60E-01	-1.07E-01	7.43E-02	2.62E-01
2654	GSM147580	GSE6401	Multiple Myeloma	Blood	9.26E-02	1.22E-01	5.98E-03	3.67E-01
2655	GSM147581	GSE6401	Multiple Myeloma	Blood	1.26E-03	-2.04E-01	8.89E-02	2.52E-01
2656	GSM147582	GSE6401	Multiple Myeloma	Blood	1.61E-02	-1.61E-01	1.75E-01	2.14E-01
2657	GSM147583	GSE6401	Multiple Myeloma	Blood	1.70E-02	-1.60E-01	1.75E-01	2.14E-01
2658	GSM147584	GSE6401	Multiple Myeloma	Blood	8.57E-02	1.24E-01	7.43E-02	2.62E-01
2659	GSM147585	GSE6401	Multiple Myeloma	Blood	3.05E-02	-1.48E-01	1.37E-01	2.29E-01

2660	GSM147586	GSE6401	Multiple Myeloma	Blood	7.01E-02	1.29E-01	2.44E-03	3.98E-01
2661	GSM147587	GSE6401	Multiple Myeloma	Blood	1.49E-01	-1.09E-01	7.43E-02	2.62E-01
2662	GSM147588	GSE6401	Multiple Myeloma	Blood	2.57E-01	9.23E-02	4.55E-02	2.85E-01
2663	GSM147589	GSE6401	Multiple Myeloma	Blood	1.12E-02	-1.68E-01	4.36E-02	2.87E-01
2664	GSM147590	GSE6401	Multiple Myeloma	Blood	1.25E-01	-1.14E-01	3.21E-03	3.89E-01
2665	GSM147591	GSE6401	Multiple Myeloma	Blood	2.01E-01	-1.00E-01	1.80E-02	3.25E-01
2666	GSM147592	GSE6401	Multiple Myeloma	Blood	2.77E-02	-1.50E-01	9.82E-02	2.47E-01
2667	GSM147593	GSE6401	Multiple Myeloma	Blood	8.24E-02	-1.25E-01	1.12E-01	2.40E-01
2668	GSM147594	GSE6401	Multiple Myeloma	Blood	1.30E-01	-1.13E-01	3.15E-01	1.74E-01
2669	GSM147595	GSE6401	Multiple Myeloma	Blood	2.77E-02	-1.50E-01	1.12E-01	2.40E-01
2670	GSM147596	GSE6401	Multiple Myeloma	Blood	5.47E-03	-1.81E-01	1.43E-02	3.34E-01
2671	GSM147597	GSE6401	Multiple Myeloma	Blood	8.10E-03	-1.74E-01	3.72E-02	2.94E-01
2672	GSM147598	GSE6401	Multiple Myeloma	Blood	1.16E-01	-1.16E-01	1.19E-02	3.42E-01
2673	GSM147599	GSE6401	Multiple Myeloma	Blood	1.53E-02	-1.62E-01	3.78E-01	1.60E-01
2674	GSM147600	GSE6401	Multiple Myeloma	Blood	2.53E-03	-1.93E-01	3.78E-01	1.60E-01
2675	GSM147601	GSE6401	Multiple Myeloma	Blood	1.00E-01	-1.20E-01	3.72E-02	2.94E-01
2676	GSM147602	GSE6401	Multiple Myeloma	Blood	5.69E-02	-1.34E-01	1.89E-02	3.23E-01
2677	GSM147603	GSE6401	Multiple Myeloma	Blood	5.23E-02	-1.36E-01	7.73E-02	2.60E-01
2678	GSM147604	GSE6401	Multiple Myeloma	Blood	1.00E-01	-1.20E-01	4.50E-01	-1.45E-01
2679	GSM147605	GSE6401	Multiple Myeloma	Blood	1.01E-02	-1.70E-01	3.40E-01	-1.69E-01
2680	GSM147606	GSE6401	Multiple Myeloma	Blood	1.83E-01	-1.03E-01	4.36E-02	2.87E-01
2681	GSM147607	GSE6401	Multiple Myeloma	Blood	2.08E-01	-9.92E-02	1.72E-02	3.27E-01
2682	GSM147608	GSE6401	Multiple Myeloma	Blood	1.34E-01	-1.12E-01	3.55E-01	1.65E-01
2683	GSM147609	GSE6401	Multiple Myeloma	Blood	5.46E-02	-1.35E-01	1.12E-01	2.40E-01
2684	GSM147610	GSE6401	Multiple Myeloma	Blood	2.72E-01	-9.03E-02	2.15E-02	3.18E-01
2685	GSM147611	GSE6401	Multiple Myeloma	Blood	5.01E-02	-1.37E-01	1.37E-01	2.29E-01
2686	GSM147612	GSE6401	Multiple Myeloma	Blood	1.31E-02	-1.65E-01	1.19E-02	3.42E-01
2687	GSM147613	GSE6401	Multiple Myeloma	Blood	2.27E-04	-2.29E-01	8.56E-02	2.54E-01
2688	GSM147614	GSE6401	Multiple Myeloma	Blood	4.79E-02	-1.38E-01	8.56E-02	2.54E-01
2689	GSM147615	GSE6401	Multiple Myeloma	Blood	4.39E-02	-1.40E-01	2.51E-01	1.91E-01
2690	GSM147616	GSE6401	Multiple Myeloma	Blood	3.03E-04	-2.25E-01	3.49E-01	-1.67E-01
2691	GSM147617	GSE6401	Multiple Myeloma	Blood	1.25E-02	-1.66E-01	2.08E-01	2.03E-01
2692	GSM147618	GSE6401	Multiple Myeloma	Blood	4.01E-02	-1.42E-01	1.06E-01	2.43E-01
2693	GSM147619	GSE6401	Multiple Myeloma	Blood	9.04E-03	-1.72E-01	2.19E-01	2.00E-01
2694	GSM147620	GSE6401	Multiple Myeloma	Blood	4.23E-01	7.34E-02	1.06E-01	2.43E-01
2695	GSM147621	GSE6401	Multiple Myeloma	Blood	1.71E-01	-1.05E-01	2.25E-02	3.16E-01
2696	GSM147622	GSE6401	Multiple Myeloma	Blood	2.91E-02	-1.49E-01	1.37E-01	2.29E-01
2697	GSM147623	GSE6401	Multiple Myeloma	Blood	4.87E-03	-1.83E-01	3.78E-01	1.60E-01
2698	GSM147624	GSE6401	Multiple Myeloma	Blood	1.70E-02	-1.60E-01	2.39E-01	1.94E-01
2699	GSM147625	GSE6401	Multiple Myeloma	Blood	1.71E-01	-1.05E-01	4.55E-02	2.85E-01
2700	GSM147626	GSE6401	Multiple Myeloma	Blood	2.44E-04	-2.28E-01	1.32E-01	2.31E-01
2701	GSM147627	GSE6401	Multiple Myeloma	Blood	1.70E-02	-1.60E-01	2.05E-02	3.20E-01
2702	GSM147628	GSE6401	Multiple Myeloma	Blood	3.13E-01	-8.53E-02	1.16E-01	2.38E-01
2703	GSM147629	GSE6401	Multiple Myeloma	Blood	1.25E-01	-1.14E-01	1.06E-01	2.43E-01
2704	GSM147630	GSE6401	Multiple Myeloma	Blood	2.57E-01	-9.23E-02	4.55E-02	2.85E-01
2705	GSM147631	GSE6401	Multiple Myeloma	Blood	3.19E-02	-1.47E-01	5.54E-02	2.76E-01
2706	GSM147632	GSE6401	Multiple Myeloma	Blood	2.91E-02	-1.49E-01	9.82E-02	2.47E-01
2707	GSM147633	GSE6401	Multiple Myeloma	Blood	7.30E-02	-1.28E-01	2.55E-02	3.11E-01
2708	GSM147634	GSE6401	Multiple Myeloma	Blood	6.46E-02	-1.31E-01	2.19E-01	2.00E-01
2709	GSM147635	GSE6401	Multiple Myeloma	Blood	3.75E-01	-7.84E-02	4.75E-02	2.83E-01
2710	GSM147636	GSE6401	Multiple Myeloma	Blood	1.89E-01	1.02E-01	5.16E-03	3.72E-01
2711	GSM147637	GSE6401	Multiple Myeloma	Blood	1.16E-01	-1.16E-01	2.26E-01	1.98E-01
2712	GSM147638	GSE6401	Multiple Myeloma	Blood	1.70E-02	-1.60E-01	3.46E-01	1.67E-01
2713	GSM147639	GSE6401	Multiple Myeloma	Blood	9.26E-02	-1.22E-01	2.86E-01	1.82E-01
2714	GSM147640	GSE6401	Multiple Myeloma	Blood	4.53E-01	7.04E-02	1.58E-02	3.30E-01
2715	GSM147641	GSE6401	Multiple Myeloma	Blood	3.75E-01	7.84E-02	1.09E-02	3.45E-01
2716	GSM147642	GSE6401	Multiple Myeloma	Blood	2.42E-01	-9.42E-02	5.31E-02	2.78E-01
2717	GSM147643	GSE6401	Multiple Myeloma	Blood	2.07E-02	-1.56E-01	3.78E-01	1.60E-01
2718	GSM147644	GSE6401	Multiple Myeloma	Blood	2.49E-01	-9.33E-02	2.55E-02	3.11E-01
2719	GSM147645	GSE6401	Multiple Myeloma	Blood	6.48E-03	-1.78E-01	1.38E-01	-2.28E-01
2720	GSM147646	GSE6401	Multiple Myeloma	Blood	4.79E-02	-1.38E-01	1.43E-02	3.34E-01
2721	GSM147647	GSE6401	Multiple Myeloma	Blood	1.06E-02	-1.69E-01	1.46E-01	-2.25E-01
2722	GSM147648	GSE6401	Multiple Myeloma	Blood	1.21E-01	-1.15E-01	2.15E-01	2.01E-01
2723	GSM147649	GSE6401	Multiple Myeloma	Blood	2.64E-01	-9.13E-02	2.25E-02	3.16E-01
2724	GSM147650	GSE6401	Multiple Myeloma	Blood	1.06E-02	-1.69E-01	2.67E-02	3.09E-01
2725	GSM147651	GSE6401	Multiple Myeloma	Blood	1.30E-01	-1.13E-01	8.89E-02	2.52E-01
2726	GSM147652	GSE6401	Multiple Myeloma	Blood	7.36E-05	-2.44E-01	4.67E-01	1.42E-01
2727	GSM147653	GSE6401	Multiple Myeloma	Blood	3.49E-04	-2.23E-01	1.80E-01	2.12E-01
2728	GSM147654	GSE6401	Multiple Myeloma	Blood	3.50E-02	-1.45E-01	3.16E-02	3.02E-01
2729	GSM147655	GSE6401	Multiple Myeloma	Blood	5.23E-02	-1.36E-01	1.55E-01	2.22E-01
2730	GSM147656	GSE6401	Multiple Myeloma	Blood	3.62E-05	-2.53E-01	8.35E-02	-2.56E-01
2731	GSM147657	GSE6401	Multiple Myeloma	Blood	4.98E-05	-2.49E-01	1.55E-01	2.22E-01
2732	GSM148911	GSE6477	Multiple Myeloma	Blood	1.61E-02	-1.61E-01	4.36E-02	2.87E-01
2733	GSM148914	GSE6477	Multiple Myeloma	Blood	1.60E-05	-2.63E-01	1.46E-01	-2.25E-01

2734	GSM148915	GSE6477	Multiple Myeloma	Blood	1.97E-02	-1.57E-01	1.96E-03	4.05E-01
2735	GSM148916	GSE6477	Multiple Myeloma	Blood	1.74E-03	-1.99E-01	1.92E-01	-2.09E-01
2736	GSM148917	GSE6477	Multiple Myeloma	Blood	1.18E-02	-1.67E-01	1.10E-01	-2.41E-01
2737	GSM148918	GSE6477	Multiple Myeloma	Blood	1.25E-02	-1.66E-01	6.17E-02	2.71E-01
2738	GSM148919	GSE6477	Multiple Myeloma	Blood	1.53E-02	-1.62E-01	3.78E-01	1.60E-01
2739	GSM148920	GSE6477	Multiple Myeloma	Blood	5.46E-02	-1.35E-01	9.58E-02	-2.49E-01
2740	GSM148921	GSE6477	Multiple Myeloma	Blood	8.13E-06	-2.71E-01	4.96E-02	-2.81E-01
2741	GSM148922	GSE6477	Multiple Myeloma	Blood	1.61E-02	-1.61E-01	1.72E-02	3.27E-01
2742	GSM148923	GSE6477	Multiple Myeloma	Blood	1.16E-04	-2.38E-01	2.26E-01	1.98E-01
2743	GSM148924	GSE6477	Multiple Myeloma	Blood	8.10E-03	-1.74E-01	1.96E-01	2.07E-01
2744	GSM148925	GSE6477	Multiple Myeloma	Blood	1.16E-01	-1.16E-01	2.21E-01	-1.99E-01
2745	GSM148926	GSE6477	Multiple Myeloma	Blood	1.61E-02	-1.61E-01	1.75E-01	2.14E-01
2746	GSM148927	GSE6477	Multiple Myeloma	Blood	3.19E-02	-1.47E-01	1.19E-02	3.42E-01
2747	GSM148928	GSE6477	Multiple Myeloma	Blood	3.84E-02	-1.43E-01	1.55E-01	2.22E-01
2748	GSM148929	GSE6477	Multiple Myeloma	Blood	2.77E-02	-1.50E-01	8.56E-02	2.54E-01
2749	GSM148930	GSE6477	Multiple Myeloma	Blood	3.19E-02	-1.47E-01	2.26E-01	1.98E-01
2750	GSM148931	GSE6477	Multiple Myeloma	Blood	5.83E-05	-2.47E-01	1.29E-01	-2.32E-01
2751	GSM148932	GSE6477	Multiple Myeloma	Blood	9.11E-04	-2.09E-01	3.01E-01	-1.78E-01
2752	GSM148933	GSE6477	Multiple Myeloma	Blood	3.42E-03	-1.88E-01	1.75E-01	2.14E-01
2753	GSM148934	GSE6477	Multiple Myeloma	Blood	1.71E-01	1.05E-01	3.72E-02	2.94E-01
2754	GSM148935	GSE6477	Multiple Myeloma	Blood	1.35E-03	-2.03E-01	2.15E-02	3.18E-01
2755	GSM148936	GSE6477	Multiple Myeloma	Blood	3.21E-01	-8.43E-02	6.17E-02	2.71E-01
2756	GSM148938	GSE6477	Multiple Myeloma	Blood	8.24E-02	-1.25E-01	7.43E-02	2.62E-01
2757	GSM148939	GSE6477	Multiple Myeloma	Blood	6.08E-04	-2.15E-01	2.02E-01	2.05E-01
2758	GSM148940	GSE6477	Multiple Myeloma	Blood	4.39E-02	-1.40E-01	8.56E-02	2.54E-01
2759	GSM148941	GSE6477	Multiple Myeloma	Blood	5.69E-02	-1.34E-01	3.87E-01	1.58E-01
2760	GSM148942	GSE6477	Multiple Myeloma	Blood	5.46E-02	-1.35E-01	9.82E-02	2.47E-01
2761	GSM148943	GSE6477	Multiple Myeloma	Blood	2.40E-02	1.53E-01	2.15E-02	3.18E-01
2762	GSM148944	GSE6477	Multiple Myeloma	Blood	2.69E-03	-1.92E-01	3.63E-01	1.63E-01
2763	GSM148945	GSE6477	Multiple Myeloma	Blood	2.52E-02	-1.52E-01	1.10E-01	-2.41E-01
2764	GSM148946	GSE6477	Multiple Myeloma	Blood	4.34E-03	-1.85E-01	3.72E-02	2.94E-01
2765	GSM148947	GSE6477	Multiple Myeloma	Blood	2.18E-02	-1.55E-01	3.15E-01	1.74E-01
2766	GSM148949	GSE6477	Multiple Myeloma	Blood	5.69E-02	-1.34E-01	2.02E-01	2.05E-01
2767	GSM148950	GSE6477	Multiple Myeloma	Blood	7.25E-03	-1.76E-01	1.12E-01	2.40E-01
2768	GSM148951	GSE6477	Multiple Myeloma	Blood	5.47E-03	-1.81E-01	1.96E-01	2.07E-01
2769	GSM148952	GSE6477	Multiple Myeloma	Blood	1.85E-03	-1.98E-01	3.15E-01	1.74E-01
2770	GSM148955	GSE6477	Multiple Myeloma	Blood	1.79E-02	-1.59E-01	5.77E-02	2.74E-01
2771	GSM148956	GSE6477	Multiple Myeloma	Blood	9.63E-02	-1.21E-01	6.17E-02	2.71E-01
2772	GSM148957	GSE6477	Multiple Myeloma	Blood	1.08E-01	-1.18E-01	6.17E-02	2.71E-01
2773	GSM148958	GSE6477	Multiple Myeloma	Blood	1.34E-01	1.12E-01	1.17E-03	4.22E-01
2774	GSM148959	GSE6477	Multiple Myeloma	Blood	6.48E-03	-1.78E-01	1.75E-01	2.14E-01
2775	GSM148960	GSE6477	Multiple Myeloma	Blood	9.63E-02	-1.21E-01	3.15E-01	1.74E-01
2776	GSM148961	GSE6477	Multiple Myeloma	Blood	4.20E-02	1.41E-01	1.50E-02	3.32E-01
2777	GSM148962	GSE6477	Multiple Myeloma	Blood	3.67E-02	-1.44E-01	3.30E-02	3.00E-01
2778	GSM148964	GSE6477	Multiple Myeloma	Blood	1.60E-01	-1.07E-01	2.55E-02	3.11E-01
2779	GSM148965	GSE6477	Multiple Myeloma	Blood	2.49E-01	-9.33E-02	3.16E-02	3.02E-01
2780	GSM148967	GSE6477	Multiple Myeloma	Blood	9.54E-03	-1.71E-01	1.55E-01	2.22E-01
2781	GSM148968	GSE6477	Multiple Myeloma	Blood	1.79E-02	-1.59E-01	1.60E-01	2.20E-01
2782	GSM148969	GSE6477	Multiple Myeloma	Blood	9.04E-03	-1.72E-01	9.82E-02	2.47E-01
2783	GSM148971	GSE6477	Multiple Myeloma	Blood	5.01E-02	1.37E-01	6.86E-04	4.38E-01
2784	GSM148973	GSE6477	Multiple Myeloma	Blood	2.52E-02	-1.52E-01	2.04E-01	-2.05E-01
2785	GSM148974	GSE6477	Multiple Myeloma	Blood	3.50E-02	-1.45E-01	1.75E-01	2.14E-01
2786	GSM148975	GSE6477	Multiple Myeloma	Blood	6.46E-02	-1.31E-01	1.21E-01	2.36E-01
2787	GSM148978	GSE6477	Multiple Myeloma	Blood	2.57E-01	-9.23E-02	1.32E-01	2.31E-01
2788	GSM148979	GSE6477	Multiple Myeloma	Blood	1.18E-03	-2.05E-01	2.32E-01	1.96E-01
2789	GSM148980	GSE6477	Multiple Myeloma	Blood	1.77E-01	-1.04E-01	1.43E-02	3.34E-01
2790	GSM148981	GSE6477	Multiple Myeloma	Blood	1.18E-02	-1.67E-01	6.17E-02	2.71E-01
2791	GSM148983	GSE6477	Multiple Myeloma	Blood	8.56E-03	-1.73E-01	2.19E-01	2.00E-01
2792	GSM148984	GSE6477	Multiple Myeloma	Blood	5.01E-02	1.37E-01	4.88E-03	3.74E-01
2793	GSM148986	GSE6477	Multiple Myeloma	Blood	2.28E-01	-9.62E-02	3.55E-01	1.65E-01
2794	GSM148987	GSE6477	Multiple Myeloma	Blood	1.12E-02	-1.68E-01	3.15E-01	1.74E-01
2795	GSM148988	GSE6477	Multiple Myeloma	Blood	1.53E-02	-1.62E-01	1.32E-01	2.31E-01
2796	GSM148989	GSE6477	Multiple Myeloma	Blood	1.30E-01	-1.13E-01	1.19E-02	3.42E-01
2797	GSM148990	GSE6477	Multiple Myeloma	Blood	1.97E-02	-1.57E-01	8.56E-02	2.54E-01
2798	GSM148991	GSE6477	Multiple Myeloma	Blood	3.84E-02	-1.43E-01	5.31E-02	2.78E-01
2799	GSM148992	GSE6477	Multiple Myeloma	Blood	2.62E-05	-2.57E-01	1.29E-01	-2.32E-01
2800	GSM148993	GSE6477	Multiple Myeloma	Blood	3.86E-03	-1.87E-01	1.16E-01	2.38E-01
2801	GSM148994	GSE6477	Multiple Myeloma	Blood	1.44E-03	-2.02E-01	2.26E-01	1.98E-01
2802	GSM148995	GSE6477	Multiple Myeloma	Blood	1.53E-02	-1.62E-01	1.32E-01	2.31E-01
2803	GSM148997	GSE6477	Multiple Myeloma	Blood	3.50E-02	-1.45E-01	1.16E-01	2.38E-01
2804	GSM148998	GSE6477	Multiple Myeloma	Blood	1.12E-02	-1.68E-01	3.01E-01	1.78E-01
2805	GSM148999	GSE6477	Multiple Myeloma	Blood	1.25E-02	-1.66E-01	1.55E-01	2.22E-01
2806	GSM149000	GSE6477	Multiple Myeloma	Blood	5.46E-02	-1.35E-01	1.55E-01	2.22E-01
2807	GSM149001	GSE6477	Multiple Myeloma	Blood	1.26E-03	-2.04E-01	1.51E-01	-2.23E-01

2808	GSM149002	GSE6477	Multiple Myeloma	Blood	2.64E-02	-1.51E-01	5.31E-02	2.78E-01
2809	GSM149004	GSE6477	Multiple Myeloma	Blood	2.18E-02	-1.55E-01	1.55E-01	2.22E-01
2810	GSM149005	GSE6477	Multiple Myeloma	Blood	1.12E-02	-1.68E-01	2.51E-01	1.91E-01
2811	GSM149006	GSE6477	Multiple Myeloma	Blood	4.39E-02	-1.40E-01	6.43E-02	2.69E-01
2812	GSM149007	GSE6477	Multiple Myeloma	Blood	7.60E-02	-1.27E-01	1.46E-01	2.25E-01
2813	GSM149008	GSE6477	Multiple Myeloma	Blood	5.79E-03	-1.80E-01	8.89E-02	2.52E-01
2814	GSM149009	GSE6477	Multiple Myeloma	Blood	1.38E-02	-1.64E-01	2.08E-01	-2.03E-01
2815	GSM149010	GSE6477	Multiple Myeloma	Blood	4.23E-01	7.34E-02	1.19E-02	3.42E-01
2816	GSM149012	GSE6477	Multiple Myeloma	Blood	1.85E-03	-1.98E-01	1.06E-01	2.43E-01
2817	GSM149014	GSE6477	Multiple Myeloma	Blood	2.07E-02	-1.56E-01	7.70E-03	3.58E-01
2818	GSM149016	GSE6477	Multiple Myeloma	Blood	3.25E-04	-2.24E-01	1.51E-01	-2.23E-01
2819	GSM149017	GSE6477	Multiple Myeloma	Blood	1.25E-02	1.66E-01	3.60E-03	3.85E-01
2820	GSM149018	GSE6477	Multiple Myeloma	Blood	9.11E-04	-2.09E-01	2.32E-01	1.96E-01
2821	GSM149019	GSE6477	Multiple Myeloma	Blood	2.91E-02	-1.49E-01	1.43E-02	3.34E-01
2822	GSM149020	GSE6477	Multiple Myeloma	Blood	1.16E-01	-1.16E-01	7.43E-02	2.62E-01
2823	GSM149021	GSE6477	Multiple Myeloma	Blood	2.69E-03	-1.92E-01	1.75E-01	2.14E-01
2824	GSM149023	GSE6477	Multiple Myeloma	Blood	2.18E-02	-1.55E-01	2.26E-01	1.98E-01
2825	GSM149024	GSE6477	Multiple Myeloma	Blood	6.97E-04	-2.13E-01	2.08E-01	-2.03E-01
2826	GSM149025	GSE6477	Multiple Myeloma	Blood	6.97E-04	-2.13E-01	5.31E-02	2.78E-01
2827	GSM149026	GSE6477	Multiple Myeloma	Blood	2.35E-01	9.52E-02	4.20E-03	3.80E-01
2828	GSM149027	GSE6477	Multiple Myeloma	Blood	5.46E-02	-1.35E-01	9.23E-02	2.50E-01
2829	GSM149028	GSE6477	Multiple Myeloma	Blood	4.43E-01	-7.14E-02	9.23E-02	2.50E-01
2830	GSM149030	GSE6477	Multiple Myeloma	Blood	4.83E-01	6.75E-02	1.86E-01	2.10E-01
2831	GSM149031	GSE6477	Multiple Myeloma	Blood	8.56E-03	-1.73E-01	7.43E-02	2.62E-01
2832	GSM149032	GSE6477	Multiple Myeloma	Blood	3.19E-02	-1.47E-01	2.51E-01	1.91E-01
2833	GSM149033	GSE6477	Multiple Myeloma	Blood	1.01E-02	-1.70E-01	6.17E-02	2.71E-01
2834	GSM149034	GSE6477	Multiple Myeloma	Blood	4.60E-03	-1.84E-01	1.55E-01	2.22E-01
2835	GSM149035	GSE6477	Multiple Myeloma	Blood	1.97E-02	-1.57E-01	5.41E-01	1.27E-01
2836	GSM149036	GSE6477	Multiple Myeloma	Blood	2.18E-02	-1.55E-01	1.98E-01	2.07E-01
2837	GSM149037	GSE6477	Multiple Myeloma	Blood	7.30E-02	-1.28E-01	3.87E-01	1.58E-01
2838	GSM149038	GSE6477	Multiple Myeloma	Blood	2.40E-02	-1.53E-01	2.51E-01	1.91E-01
2839	GSM149039	GSE6477	Multiple Myeloma	Blood	3.63E-03	-1.88E-01	2.68E-01	-1.86E-01
2840	GSM149040	GSE6477	Multiple Myeloma	Blood	4.59E-02	-1.39E-01	8.56E-02	2.54E-01
2841	GSM149042	GSE6477	Multiple Myeloma	Blood	1.79E-02	-1.59E-01	1.14E-01	-2.39E-01
2842	GSM149043	GSE6477	Multiple Myeloma	Blood	2.40E-02	-1.53E-01	8.56E-02	2.54E-01
2843	GSM149044	GSE6477	Multiple Myeloma	Blood	1.12E-02	-1.68E-01	2.94E-01	1.80E-01
2844	GSM149047	GSE6477	Multiple Myeloma	Blood	2.29E-02	-1.54E-01	2.02E-01	2.05E-01
2845	GSM149048	GSE6477	Multiple Myeloma	Blood	1.12E-01	-1.17E-01	1.16E-01	2.38E-01
2846	GSM149049	GSE6477	Multiple Myeloma	Blood	1.54E-01	-1.08E-01	3.30E-02	3.00E-01
2847	GSM149050	GSE6477	Multiple Myeloma	Blood	3.75E-01	7.84E-02	1.37E-01	2.29E-01
2848	GSM149051	GSE6477	Multiple Myeloma	Blood	2.29E-02	-1.54E-01	9.82E-02	2.47E-01
2849	GSM149052	GSE6477	Multiple Myeloma	Blood	5.69E-02	-1.34E-01	4.06E-02	2.90E-01
2850	GSM149054	GSE6477	Multiple Myeloma	Blood	2.07E-02	-1.56E-01	2.51E-01	1.91E-01
2851	GSM149055	GSE6477	Multiple Myeloma	Blood	1.65E-01	-1.06E-01	1.75E-01	2.14E-01
2852	GSM149056	GSE6477	Multiple Myeloma	Blood	2.64E-02	-1.51E-01	8.89E-02	2.52E-01
2853	GSM149058	GSE6477	Multiple Myeloma	Blood	1.18E-02	-1.67E-01	3.78E-01	1.60E-01
2854	GSM149059	GSE6477	Multiple Myeloma	Blood	8.57E-02	-1.24E-01	2.02E-01	2.05E-01
2855	GSM149060	GSE6477	Multiple Myeloma	Blood	1.00E-01	-1.20E-01	1.19E-02	3.42E-01
2856	GSM149061	GSE6477	Multiple Myeloma	Blood	5.68E-04	-2.16E-01	2.26E-01	1.98E-01
2857	GSM341943	GSE13591	Multiple Myeloma	Blood	2.18E-02	-1.55E-01	4.38E-01	1.48E-01
2858	GSM341944	GSE13591	Multiple Myeloma	Blood	7.30E-02	1.28E-01	7.43E-02	2.62E-01
2859	GSM341945	GSE13591	Multiple Myeloma	Blood	3.19E-02	-1.47E-01	5.65E-01	-1.23E-01
2860	GSM341946	GSE13591	Multiple Myeloma	Blood	2.40E-02	-1.53E-01	1.75E-01	2.14E-01
2861	GSM341947	GSE13591	Multiple Myeloma	Blood	2.29E-02	-1.54E-01	1.12E-01	2.40E-01
2862	GSM341948	GSE13591	Multiple Myeloma	Blood	2.01E-01	-1.00E-01	2.81E-01	1.83E-01
2863	GSM341949	GSE13591	Multiple Myeloma	Blood	9.04E-03	-1.72E-01	1.75E-01	2.14E-01
2864	GSM341950	GSE13591	Multiple Myeloma	Blood	1.16E-01	1.16E-01	5.31E-02	2.78E-01
2865	GSM341951	GSE13591	Multiple Myeloma	Blood	2.64E-02	-1.51E-01	1.80E-01	2.12E-01
2866	GSM341952	GSE13591	Multiple Myeloma	Blood	3.04E-01	8.63E-02	4.55E-02	2.85E-01
2867	GSM341953	GSE13591	Multiple Myeloma	Blood	5.69E-02	-1.34E-01	1.12E-01	2.40E-01
2868	GSM341954	GSE13591	Multiple Myeloma	Blood	2.57E-01	-9.23E-02	4.36E-02	2.87E-01
2869	GSM341955	GSE13591	Multiple Myeloma	Blood	6.08E-04	-2.15E-01	1.02E-01	2.45E-01
2870	GSM341956	GSE13591	Multiple Myeloma	Blood	6.97E-04	-2.13E-01	3.55E-01	1.65E-01
2871	GSM341957	GSE13591	Multiple Myeloma	Blood	2.88E-01	-8.83E-02	2.32E-01	1.96E-01
2872	GSM341958	GSE13591	Multiple Myeloma	Blood	1.63E-03	-2.00E-01	3.15E-01	1.74E-01
2873	GSM341959	GSE13591	Multiple Myeloma	Blood	9.54E-03	-1.71E-01	2.66E-01	1.87E-01
2874	GSM341960	GSE13591	Multiple Myeloma	Blood	2.28E-01	-9.62E-02	4.55E-02	2.85E-01
2875	GSM341961	GSE13591	Multiple Myeloma	Blood	1.30E-01	-1.13E-01	8.56E-02	2.54E-01
2876	GSM341962	GSE13591	Multiple Myeloma	Blood	1.06E-02	-1.69E-01	3.16E-02	3.02E-01
2877	GSM341963	GSE13591	Multiple Myeloma	Blood	1.00E-01	-1.20E-01	6.43E-02	2.69E-01
2878	GSM341964	GSE13591	Multiple Myeloma	Blood	2.40E-02	-1.53E-01	1.37E-01	2.29E-01
2879	GSM341965	GSE13591	Multiple Myeloma	Blood	5.16E-03	-1.82E-01	2.02E-01	2.05E-01
2880	GSM341966	GSE13591	Multiple Myeloma	Blood	1.25E-02	-1.66E-01	7.43E-02	2.62E-01
2881	GSM341967	GSE13591	Multiple Myeloma	Blood	1.18E-03	-2.05E-01	2.02E-01	2.05E-01

2882	GSM341968	GSE13591	Multiple Myeloma	Blood	2.80E-01	-8.93E-02	8.12E-03	3.56E-01
2883	GSM341969	GSE13591	Multiple Myeloma	Blood	1.18E-02	-1.67E-01	2.08E-01	2.03E-01
2884	GSM341970	GSE13591	Multiple Myeloma	Blood	4.20E-02	-1.41E-01	4.55E-02	2.85E-01
2885	GSM341971	GSE13591	Multiple Myeloma	Blood	4.20E-02	-1.41E-01	2.21E-01	-1.99E-01
2886	GSM341972	GSE13591	Multiple Myeloma	Blood	2.08E-01	-9.92E-02	1.96E-03	4.05E-01
2887	GSM341973	GSE13591	Multiple Myeloma	Blood	7.92E-02	-1.26E-01	7.43E-02	2.62E-01
2888	GSM341974	GSE13591	Multiple Myeloma	Blood	1.16E-01	-1.16E-01	3.89E-02	2.92E-01
2889	GSM341975	GSE13591	Multiple Myeloma	Blood	8.24E-02	1.25E-01	2.15E-02	3.18E-01
2890	GSM341976	GSE13591	Multiple Myeloma	Blood	5.01E-02	-1.37E-01	6.17E-02	2.71E-01
2891	GSM341977	GSE13591	Multiple Myeloma	Blood	9.54E-03	-1.71E-01	9.82E-02	2.47E-01
2892	GSM341978	GSE13591	Multiple Myeloma	Blood	4.39E-02	1.40E-01	5.98E-03	3.67E-01
2893	GSM341979	GSE13591	Multiple Myeloma	Blood	6.97E-04	-2.13E-01	1.21E-01	2.36E-01
2894	GSM341980	GSE13591	Multiple Myeloma	Blood	1.04E-01	1.19E-01	3.97E-03	3.82E-01
2895	GSM341981	GSE13591	Multiple Myeloma	Blood	3.86E-03	-1.87E-01	2.15E-02	3.18E-01
2896	GSM341982	GSE13591	Multiple Myeloma	Blood	8.91E-02	-1.23E-01	1.80E-02	3.25E-01
2897	GSM341983	GSE13591	Multiple Myeloma	Blood	2.18E-02	-1.55E-01	1.21E-01	2.36E-01
2898	GSM341984	GSE13591	Multiple Myeloma	Blood	2.18E-02	-1.55E-01	2.02E-01	2.05E-01
2899	GSM341985	GSE13591	Multiple Myeloma	Blood	1.60E-01	-1.07E-01	3.89E-02	2.92E-01
2900	GSM341986	GSE13591	Multiple Myeloma	Blood	6.51E-04	-2.14E-01	1.32E-01	2.31E-01
2901	GSM341987	GSE13591	Multiple Myeloma	Blood	2.77E-02	-1.50E-01	7.73E-02	2.60E-01
2902	GSM341988	GSE13591	Multiple Myeloma	Blood	3.50E-02	-1.45E-01	9.82E-02	2.47E-01
2903	GSM341989	GSE13591	Multiple Myeloma	Blood	1.89E-01	1.02E-01	4.20E-03	3.80E-01
2904	GSM341990	GSE13591	Multiple Myeloma	Blood	3.94E-01	-7.64E-02	2.80E-02	3.07E-01
2905	GSM341991	GSE13591	Multiple Myeloma	Blood	8.59E-05	-2.42E-01	3.72E-01	-1.61E-01
2906	GSM341992	GSE13591	Multiple Myeloma	Blood	3.08E-05	-2.55E-01	1.51E-01	-2.23E-01
2907	GSM341993	GSE13591	Multiple Myeloma	Blood	1.65E-01	1.06E-01	2.15E-02	3.18E-01
2908	GSM341994	GSE13591	Multiple Myeloma	Blood	4.59E-02	-1.39E-01	4.55E-02	2.85E-01
2909	GSM341995	GSE13591	Multiple Myeloma	Blood	6.13E-03	-1.79E-01	7.43E-02	2.62E-01
2910	GSM341996	GSE13591	Multiple Myeloma	Blood	9.04E-03	-1.72E-01	2.26E-01	1.98E-01
2911	GSM341997	GSE13591	Multiple Myeloma	Blood	6.13E-03	-1.79E-01	1.42E-01	-2.27E-01
2912	GSM341998	GSE13591	Multiple Myeloma	Blood	3.50E-02	-1.45E-01	1.32E-01	2.31E-01
2913	GSM341999	GSE13591	Multiple Myeloma	Blood	2.40E-02	-1.53E-01	4.36E-02	2.87E-01
2914	GSM342000	GSE13591	Multiple Myeloma	Blood	3.04E-03	-1.90E-01	2.51E-01	1.91E-01
2915	GSM342001	GSE13591	Multiple Myeloma	Blood	7.01E-02	1.29E-01	3.16E-02	3.02E-01
2916	GSM342002	GSE13591	Multiple Myeloma	Blood	1.34E-01	-1.12E-01	7.43E-02	2.62E-01
2917	GSM342003	GSE13591	Multiple Myeloma	Blood	7.30E-02	1.28E-01	9.34E-03	3.51E-01
2918	GSM342004	GSE13591	Multiple Myeloma	Blood	1.38E-02	-1.64E-01	1.75E-01	2.14E-01
2919	GSM342005	GSE13591	Multiple Myeloma	Blood	1.34E-01	-1.12E-01	2.67E-02	3.09E-01
2920	GSM342006	GSE13591	Multiple Myeloma	Blood	1.12E-01	-1.17E-01	3.15E-01	1.74E-01
2921	GSM342007	GSE13591	Multiple Myeloma	Blood	4.87E-03	-1.83E-01	3.72E-02	2.94E-01
2922	GSM342008	GSE13591	Multiple Myeloma	Blood	7.67E-03	-1.75E-01	3.78E-01	1.60E-01
2923	GSM342009	GSE13591	Multiple Myeloma	Blood	5.79E-03	-1.80E-01	3.78E-01	1.60E-01
2924	GSM342010	GSE13591	Multiple Myeloma	Blood	6.73E-02	-1.30E-01	4.15E-01	-1.52E-01
2925	GSM342011	GSE13591	Multiple Myeloma	Blood	1.38E-02	-1.64E-01	3.81E-01	-1.59E-01
2926	GSM342012	GSE13591	Multiple Myeloma	Blood	2.14E-01	-9.82E-02	3.72E-02	2.94E-01
2927	GSM342013	GSE13591	Multiple Myeloma	Blood	1.00E-01	-1.20E-01	3.15E-01	1.74E-01
2928	GSM342014	GSE13591	Multiple Myeloma	Blood	2.21E-01	-9.72E-02	4.75E-02	2.83E-01
2929	GSM342015	GSE13591	Multiple Myeloma	Blood	5.46E-02	-1.35E-01	3.16E-02	3.02E-01
2930	GSM342016	GSE13591	Multiple Myeloma	Blood	2.91E-02	-1.49E-01	2.51E-01	1.91E-01
2931	GSM342017	GSE13591	Multiple Myeloma	Blood	2.64E-02	-1.51E-01	1.55E-01	2.22E-01
2932	GSM342018	GSE13591	Multiple Myeloma	Blood	1.83E-01	-1.03E-01	4.55E-02	2.85E-01
2933	GSM342019	GSE13591	Multiple Myeloma	Blood	7.60E-02	-1.27E-01	2.15E-02	3.18E-01
2934	GSM342020	GSE13591	Multiple Myeloma	Blood	3.05E-02	-1.48E-01	2.19E-01	2.00E-01
2935	GSM342021	GSE13591	Multiple Myeloma	Blood	2.72E-01	-9.03E-02	1.25E-01	2.34E-01
2936	GSM342022	GSE13591	Multiple Myeloma	Blood	4.59E-02	-1.39E-01	2.26E-01	1.98E-01
2937	GSM342023	GSE13591	Multiple Myeloma	Blood	8.10E-03	-1.74E-01	3.46E-01	1.67E-01
2938	GSM342024	GSE13591	Multiple Myeloma	Blood	3.38E-01	-8.23E-02	2.93E-02	3.05E-01
2939	GSM342025	GSE13591	Multiple Myeloma	Blood	8.56E-03	-1.73E-01	3.78E-01	1.60E-01
2940	GSM342026	GSE13591	Multiple Myeloma	Blood	1.95E-01	-1.01E-01	5.31E-02	2.78E-01
2941	GSM342027	GSE13591	Multiple Myeloma	Blood	1.38E-02	-1.64E-01	1.57E-01	-2.21E-01
2942	GSM342028	GSE13591	Multiple Myeloma	Blood	2.77E-02	-1.50E-01	1.92E-01	-2.09E-01
2943	GSM342029	GSE13591	Multiple Myeloma	Blood	7.01E-02	-1.29E-01	2.26E-01	1.98E-01
2944	GSM342030	GSE13591	Multiple Myeloma	Blood	2.44E-04	-2.28E-01	1.80E-01	2.12E-01
2945	GSM342031	GSE13591	Multiple Myeloma	Blood	6.30E-05	-2.46E-01	1.16E-01	2.38E-01
2946	GSM342032	GSE13591	Multiple Myeloma	Blood	8.24E-02	-1.25E-01	2.55E-02	3.11E-01
2947	GSM342033	GSE13591	Multiple Myeloma	Blood	2.91E-02	1.49E-01	6.67E-03	3.63E-01
2948	GSM342034	GSE13591	Multiple Myeloma	Blood	5.68E-04	-2.16E-01	1.96E-01	2.07E-01
2949	GSM342035	GSE13591	Multiple Myeloma	Blood	5.94E-02	-1.33E-01	3.63E-01	-1.63E-01
2950	GSM342036	GSE13591	Multiple Myeloma	Blood	8.10E-03	-1.74E-01	1.75E-01	2.14E-01
2951	GSM342037	GSE13591	Multiple Myeloma	Blood	1.70E-02	-1.60E-01	8.89E-02	2.52E-01
2952	GSM342038	GSE13591	Multiple Myeloma	Blood	2.62E-04	-2.27E-01	9.82E-02	2.47E-01
2953	GSM342039	GSE13591	Multiple Myeloma	Blood	4.01E-02	-1.42E-01	5.51E-01	1.25E-01
2954	GSM342040	GSE13591	Multiple Myeloma	Blood	4.20E-02	-1.41E-01	7.43E-02	2.62E-01
2955	GSM342041	GSE13591	Multiple Myeloma	Blood	2.69E-03	-1.92E-01	3.90E-01	-1.57E-01

2956	GSM342042	GSE13591	Multiple Myeloma	Blood	6.46E-02	-1.31E-01	2.15E-02	3.18E-01
2957	GSM342043	GSE13591	Multiple Myeloma	Blood	4.87E-03	-1.83E-01	2.21E-01	-1.99E-01
2958	GSM342044	GSE13591	Multiple Myeloma	Blood	2.18E-02	-1.55E-01	1.80E-01	2.12E-01
2959	GSM342045	GSE13591	Multiple Myeloma	Blood	2.96E-01	8.73E-02	2.81E-01	1.83E-01
2960	GSM342046	GSE13591	Multiple Myeloma	Blood	8.24E-02	-1.25E-01	1.12E-01	2.40E-01
2961	GSM342047	GSE13591	Multiple Myeloma	Blood	5.46E-02	-1.35E-01	8.03E-02	2.58E-01
2962	GSM342048	GSE13591	Multiple Myeloma	Blood	3.84E-02	-1.43E-01	2.19E-01	2.00E-01
2963	GSM342049	GSE13591	Multiple Myeloma	Blood	1.89E-01	-1.02E-01	8.56E-02	2.54E-01
2964	GSM342050	GSE13591	Multiple Myeloma	Blood	9.04E-03	-1.72E-01	4.36E-02	2.87E-01
2965	GSM342051	GSE13591	Multiple Myeloma	Blood	7.01E-02	-1.29E-01	8.12E-03	3.56E-01
2966	GSM342052	GSE13591	Multiple Myeloma	Blood	2.77E-02	-1.50E-01	9.82E-02	2.47E-01
2967	GSM342053	GSE13591	Multiple Myeloma	Blood	1.08E-01	-1.18E-01	1.19E-02	3.42E-01
2968	GSM342054	GSE13591	Multiple Myeloma	Blood	4.20E-02	-1.41E-01	4.06E-02	2.90E-01
2969	GSM342055	GSE13591	Multiple Myeloma	Blood	5.01E-02	-1.37E-01	8.89E-02	2.52E-01
2970	GSM342056	GSE13591	Multiple Myeloma	Blood	7.92E-02	-1.26E-01	1.16E-01	2.38E-01
2971	GSM342057	GSE13591	Multiple Myeloma	Blood	3.67E-02	-1.44E-01	1.19E-02	3.42E-01
2972	GSM342058	GSE13591	Multiple Myeloma	Blood	1.97E-02	-1.57E-01	6.17E-02	2.71E-01
2973	GSM342059	GSE13591	Multiple Myeloma	Blood	2.77E-02	-1.50E-01	1.02E-01	2.45E-01
2974	GSM342060	GSE13591	Multiple Myeloma	Blood	2.18E-02	-1.55E-01	2.05E-02	3.20E-01
2975	GSM342061	GSE13591	Multiple Myeloma	Blood	2.72E-01	-9.03E-02	1.34E-01	2.30E-01
2976	GSM342062	GSE13591	Multiple Myeloma	Blood	3.84E-01	7.74E-02	7.04E-03	3.61E-01
2977	GSM342063	GSE13591	Multiple Myeloma	Blood	3.67E-02	-1.44E-01	9.34E-03	3.51E-01
2978	GSM342064	GSE13591	Multiple Myeloma	Blood	3.05E-02	-1.48E-01	5.31E-02	2.78E-01
2979	GSM342065	GSE13591	Multiple Myeloma	Blood	1.00E-01	-1.20E-01	1.72E-02	3.27E-01
2980	GSM342066	GSE13591	Multiple Myeloma	Blood	5.16E-03	-1.82E-01	3.55E-01	1.65E-01
2981	GSM342067	GSE13591	Multiple Myeloma	Blood	2.53E-03	-1.93E-01	3.97E-03	3.82E-01
2982	GSM342068	GSE13591	Multiple Myeloma	Blood	1.25E-02	-1.66E-01	4.36E-02	2.87E-01
2983	GSM342069	GSE13591	Multiple Myeloma	Blood	6.86E-03	-1.77E-01	1.21E-01	2.36E-01
2984	GSM342070	GSE13591	Multiple Myeloma	Blood	3.50E-02	-1.45E-01	1.55E-01	2.22E-01
2985	GSM342071	GSE13591	Multiple Myeloma	Blood	1.04E-01	-1.19E-01	3.16E-02	3.02E-01
2986	GSM342072	GSE13591	Multiple Myeloma	Blood	3.34E-02	-1.46E-01	1.02E-01	2.45E-01
2987	GSM342073	GSE13591	Multiple Myeloma	Blood	8.57E-02	-1.24E-01	6.69E-02	2.67E-01
2988	GSM342074	GSE13591	Multiple Myeloma	Blood	7.01E-02	-1.29E-01	2.46E-01	1.92E-01
2989	GSM342075	GSE13591	Multiple Myeloma	Blood	1.01E-02	-1.70E-01	7.43E-02	2.62E-01
2990	GSM432969	GSE17306	Multiple Myeloma	Blood	4.09E-03	-1.86E-01	8.56E-02	2.54E-01
2991	GSM432970	GSE17306	Multiple Myeloma	Blood	2.52E-02	-1.52E-01	1.55E-01	2.22E-01
2992	GSM432971	GSE17306	Multiple Myeloma	Blood	9.26E-02	-1.22E-01	4.55E-02	2.85E-01
2993	GSM432972	GSE17306	Multiple Myeloma	Blood	3.19E-02	-1.47E-01	4.88E-03	3.74E-01
2994	GSM432973	GSE17306	Multiple Myeloma	Blood	6.46E-02	-1.31E-01	7.73E-02	2.60E-01
2995	GSM432974	GSE17306	Multiple Myeloma	Blood	3.34E-02	-1.46E-01	1.32E-01	2.31E-01
2996	GSM432975	GSE17306	Multiple Myeloma	Blood	1.61E-02	-1.61E-01	3.30E-02	3.00E-01
2997	GSM432976	GSE17306	Multiple Myeloma	Blood	1.12E-01	-1.17E-01	1.72E-02	3.27E-01
2998	GSM432977	GSE17306	Multiple Myeloma	Blood	1.74E-03	-1.99E-01	1.72E-02	3.27E-01
2999	GSM432978	GSE17306	Multiple Myeloma	Blood	1.97E-02	-1.57E-01	1.32E-01	2.31E-01
3000	GSM432979	GSE17306	Multiple Myeloma	Blood	1.83E-01	1.03E-01	1.37E-01	2.29E-01
3001	GSM432980	GSE17306	Multiple Myeloma	Blood	3.50E-02	-1.45E-01	3.72E-02	2.94E-01
3002	GSM432981	GSE17306	Multiple Myeloma	Blood	7.25E-03	-1.76E-01	1.75E-01	2.14E-01
3003	GSM432982	GSE17306	Multiple Myeloma	Blood	1.26E-03	-2.04E-01	2.15E-01	-2.01E-01
3004	GSM432983	GSE17306	Multiple Myeloma	Blood	2.35E-01	9.52E-02	3.16E-02	3.02E-01
3005	GSM432984	GSE17306	Multiple Myeloma	Blood	3.56E-01	8.04E-02	1.16E-01	2.38E-01
3006	GSM432985	GSE17306	Multiple Myeloma	Blood	4.79E-02	-1.38E-01	4.88E-03	3.74E-01
3007	GSM432986	GSE17306	Multiple Myeloma	Blood	1.04E-01	-1.19E-01	9.23E-02	2.50E-01
3008	GSM432987	GSE17306	Multiple Myeloma	Blood	2.10E-03	-1.96E-01	9.58E-02	-2.49E-01
3009	GSM432988	GSE17306	Multiple Myeloma	Blood	4.20E-02	-1.41E-01	1.80E-02	3.25E-01
3010	GSM432990	GSE17306	Multiple Myeloma	Blood	1.97E-02	-1.57E-01	2.26E-01	1.98E-01
3011	GSM432991	GSE17306	Multiple Myeloma	Blood	2.52E-02	-1.52E-01	3.72E-02	2.94E-01
3012	GSM432992	GSE17306	Multiple Myeloma	Blood	2.27E-04	-2.29E-01	1.06E-01	2.43E-01
3013	GSM432993	GSE17306	Multiple Myeloma	Blood	1.31E-02	-1.65E-01	1.19E-02	3.42E-01
3014	GSM432994	GSE17306	Multiple Myeloma	Blood	1.30E-01	1.13E-01	1.43E-02	3.34E-01
3015	GSM432995	GSE17306	Multiple Myeloma	Blood	1.08E-04	-2.39E-01	1.96E-01	2.07E-01
3016	GSM432996	GSE17306	Multiple Myeloma	Blood	1.04E-01	-1.19E-01	7.43E-02	2.62E-01
3017	GSM432997	GSE17306	Multiple Myeloma	Blood	4.87E-03	-1.83E-01	2.88E-01	-1.81E-01
3018	GSM432998	GSE17306	Multiple Myeloma	Blood	5.94E-02	-1.33E-01	2.19E-01	2.00E-01
3019	GSM432999	GSE17306	Multiple Myeloma	Blood	6.13E-03	-1.79E-01	3.46E-01	1.67E-01
3020	GSM433000	GSE17306	Multiple Myeloma	Blood	5.69E-02	-1.34E-01	3.72E-02	2.94E-01
3021	GSM433001	GSE17306	Multiple Myeloma	Blood	4.13E-01	7.44E-02	5.17E-02	2.79E-01
3022	GSM433002	GSE17306	Multiple Myeloma	Blood	5.46E-02	-1.35E-01	1.96E-01	2.07E-01
3023	GSM433003	GSE17306	Multiple Myeloma	Blood	2.42E-01	9.42E-02	9.82E-02	2.47E-01
3024	GSM433004	GSE17306	Multiple Myeloma	Blood	9.73E-04	-2.08E-01	3.40E-01	-1.69E-01
3025	GSM433005	GSE17306	Multiple Myeloma	Blood	9.63E-02	-1.21E-01	2.59E-03	3.96E-01
3026	GSM433006	GSE17306	Multiple Myeloma	Blood	1.79E-02	-1.59E-01	2.61E-01	-1.88E-01
3027	GSM433007	GSE17306	Multiple Myeloma	Blood	1.83E-01	-1.03E-01	5.77E-02	2.74E-01
3028	GSM433008	GSE17306	Multiple Myeloma	Blood	7.92E-02	-1.26E-01	6.69E-02	2.67E-01
3029	GSM433009	GSE17306	Multiple Myeloma	Blood	1.18E-02	-1.67E-01	3.15E-01	1.74E-01

3030	GSM433010	GSE17306	Multiple Myeloma	Blood	3.62E-05	-2.53E-01	6.96E-02	-2.65E-01
3031	GSM433011	GSE17306	Multiple Myeloma	Blood	1.08E-01	-1.18E-01	1.02E-01	2.45E-01
3032	GSM433012	GSE17306	Multiple Myeloma	Blood	2.64E-01	9.13E-02	1.16E-01	2.38E-01
3033	GSM433013	GSE17306	Multiple Myeloma	Blood	1.44E-03	-2.02E-01	2.19E-01	2.00E-01
3034	GSM433014	GSE17306	Multiple Myeloma	Blood	3.19E-02	-1.47E-01	1.55E-01	2.22E-01
3035	GSM433015	GSE17306	Multiple Myeloma	Blood	2.21E-01	9.72E-02	1.19E-02	3.42E-01
3036	GSM433016	GSE17306	Multiple Myeloma	Blood	4.60E-03	-1.84E-01	7.43E-02	2.62E-01
3037	GSM433017	GSE17306	Multiple Myeloma	Blood	2.72E-01	9.03E-02	1.75E-01	2.14E-01
3038	GSM433018	GSE17306	Multiple Myeloma	Blood	8.91E-02	-1.23E-01	9.34E-03	3.51E-01
3039	GSM433019	GSE17306	Multiple Myeloma	Blood	2.08E-01	9.92E-02	1.80E-01	2.12E-01
3040	GSM433020	GSE17306	Multiple Myeloma	Blood	2.38E-03	-1.94E-01	1.96E-01	2.07E-01
3041	GSM442690	GSE17498	Multiple Myeloma	Blood	3.67E-02	-1.44E-01	4.41E-01	-1.47E-01
3042	GSM442691	GSE17498	Multiple Myeloma	Blood	3.67E-02	-1.44E-01	1.71E-01	-2.16E-01
3043	GSM442692	GSE17498	Multiple Myeloma	Blood	1.95E-01	-1.01E-01	1.80E-02	3.25E-01
3044	GSM442693	GSE17498	Multiple Myeloma	Blood	8.57E-02	-1.24E-01	5.62E-01	-1.23E-01
3045	GSM442694	GSE17498	Multiple Myeloma	Blood	1.08E-01	-1.18E-01	1.75E-01	2.14E-01
3046	GSM442695	GSE17498	Multiple Myeloma	Blood	9.63E-02	1.21E-01	3.89E-02	2.92E-01
3047	GSM442696	GSE17498	Multiple Myeloma	Blood	7.01E-02	-1.29E-01	3.30E-02	3.00E-01
3048	GSM442697	GSE17498	Multiple Myeloma	Blood	2.35E-01	9.52E-02	9.85E-03	3.49E-01
3049	GSM442698	GSE17498	Multiple Myeloma	Blood	1.04E-01	1.19E-01	2.15E-02	3.18E-01
3050	GSM442699	GSE17498	Multiple Myeloma	Blood	3.34E-02	-1.46E-01	5.31E-02	2.78E-01
3051	GSM442700	GSE17498	Multiple Myeloma	Blood	1.83E-01	-1.03E-01	6.96E-02	2.65E-01
3052	GSM442701	GSE17498	Multiple Myeloma	Blood	8.24E-02	-1.25E-01	5.31E-02	2.78E-01
3053	GSM442702	GSE17498	Multiple Myeloma	Blood	8.91E-02	-1.23E-01	9.82E-02	2.47E-01
3054	GSM442703	GSE17498	Multiple Myeloma	Blood	1.06E-02	-1.69E-01	2.26E-01	1.98E-01
3055	GSM442704	GSE17498	Multiple Myeloma	Blood	9.63E-02	-1.21E-01	1.02E-01	2.45E-01
3056	GSM442705	GSE17498	Multiple Myeloma	Blood	1.97E-02	1.57E-01	1.84E-03	4.07E-01
3057	GSM442706	GSE17498	Multiple Myeloma	Blood	4.60E-03	-1.84E-01	8.89E-02	2.52E-01
3058	GSM442707	GSE17498	Multiple Myeloma	Blood	8.24E-02	-1.25E-01	2.26E-01	1.98E-01
3059	GSM442708	GSE17498	Multiple Myeloma	Blood	4.20E-02	-1.41E-01	1.75E-01	2.14E-01
3060	GSM442709	GSE17498	Multiple Myeloma	Blood	1.89E-01	-1.02E-01	4.20E-03	3.80E-01
3061	GSM442710	GSE17498	Multiple Myeloma	Blood	3.67E-02	-1.44E-01	9.82E-02	2.47E-01
3062	GSM442711	GSE17498	Multiple Myeloma	Blood	5.01E-02	-1.37E-01	1.12E-01	2.40E-01
3063	GSM442712	GSE17498	Multiple Myeloma	Blood	6.73E-02	-1.30E-01	1.89E-02	3.23E-01
3064	GSM442713	GSE17498	Multiple Myeloma	Blood	1.54E-01	-1.08E-01	1.96E-01	2.07E-01
3065	GSM442714	GSE17498	Multiple Myeloma	Blood	1.63E-03	-2.00E-01	4.31E-01	1.49E-01
3066	GSM442715	GSE17498	Multiple Myeloma	Blood	2.18E-02	-1.55E-01	3.23E-01	1.72E-01
3067	GSM442716	GSE17498	Multiple Myeloma	Blood	6.97E-04	-2.13E-01	1.32E-01	2.31E-01
3068	GSM442717	GSE17498	Multiple Myeloma	Blood	2.57E-01	-9.23E-02	4.55E-02	2.85E-01
3069	GSM442718	GSE17498	Multiple Myeloma	Blood	1.60E-01	-1.07E-01	2.58E-01	1.89E-01
3070	GSM442719	GSE17498	Multiple Myeloma	Blood	2.49E-01	-9.33E-02	1.16E-01	2.38E-01
3071	GSM442720	GSE17498	Multiple Myeloma	Blood	3.50E-02	-1.45E-01	1.80E-02	3.25E-01
3072	GSM442721	GSE17498	Multiple Myeloma	Blood	4.34E-03	-1.85E-01	9.85E-03	3.49E-01
3073	GSM442722	GSE17498	Multiple Myeloma	Blood	3.19E-02	-1.47E-01	7.43E-02	2.62E-01
3074	GSM442723	GSE17498	Multiple Myeloma	Blood	2.14E-01	-9.82E-02	2.58E-01	1.89E-01
3075	GSM442724	GSE17498	Multiple Myeloma	Blood	1.16E-01	-1.16E-01	2.55E-02	3.11E-01
3076	GSM442725	GSE17498	Multiple Myeloma	Blood	9.04E-03	-1.72E-01	1.75E-01	2.14E-01
3077	GSM442726	GSE17498	Multiple Myeloma	Blood	8.91E-02	-1.23E-01	2.58E-01	1.89E-01
3078	GSM442727	GSE17498	Multiple Myeloma	Blood	4.39E-02	-1.40E-01	2.51E-01	1.91E-01
3079	GSM825626	GSE33382	Osteosarcoma	Bone	7.29E-22	5.76E-01	3.14E-10	8.15E-01
3080	GSM825627	GSE33382	Osteosarcoma	Bone	4.94E-19	5.36E-01	1.64E-08	7.38E-01
3081	GSM825628	GSE33382	Osteosarcoma	Bone	4.04E-19	5.38E-01	3.14E-10	8.15E-01
3082	GSM825629	GSE33382	Osteosarcoma	Bone	3.08E-19	5.39E-01	6.81E-09	7.56E-01
3083	GSM825630	GSE33382	Osteosarcoma	Bone	1.00E-15	4.85E-01	6.81E-09	7.56E-01
3084	GSM825631	GSE33382	Osteosarcoma	Bone	1.20E-10	3.95E-01	1.54E-07	6.90E-01
3085	GSM825632	GSE33382	Osteosarcoma	Bone	1.60E-24	6.12E-01	1.14E-11	8.75E-01
3086	GSM825633	GSE33382	Osteosarcoma	Bone	1.04E-10	3.96E-01	8.91E-08	7.02E-01
3087	GSM825634	GSE33382	Osteosarcoma	Bone	8.99E-20	5.47E-01	3.14E-10	8.15E-01
3088	GSM825635	GSE33382	Osteosarcoma	Bone	2.39E-17	5.11E-01	8.10E-10	7.98E-01
3089	GSM825636	GSE33382	Osteosarcoma	Bone	2.06E-14	4.64E-01	2.77E-09	7.74E-01
3090	GSM825637	GSE33382	Osteosarcoma	Bone	1.02E-10	3.96E-01	6.76E-08	7.08E-01
3091	GSM825638	GSE33382	Osteosarcoma	Bone	1.82E-17	5.13E-01	1.23E-08	7.44E-01
3092	GSM825639	GSE33382	Osteosarcoma	Bone	1.01E-20	5.61E-01	3.14E-10	8.15E-01
3093	GSM825640	GSE33382	Osteosarcoma	Bone	1.27E-17	5.15E-01	1.11E-09	7.92E-01
3094	GSM825641	GSE33382	Osteosarcoma	Bone	6.90E-15	4.72E-01	6.81E-09	7.56E-01
3095	GSM825642	GSE33382	Osteosarcoma	Bone	4.81E-20	5.51E-01	3.14E-10	8.15E-01
3096	GSM825643	GSE33382	Osteosarcoma	Bone	1.35E-16	4.99E-01	1.23E-08	7.44E-01
3097	GSM825644	GSE33382	Osteosarcoma	Bone	4.04E-19	5.38E-01	1.51E-09	7.86E-01
3098	GSM825645	GSE33382	Osteosarcoma	Bone	1.33E-18	5.30E-01	8.10E-10	7.98E-01
3099	GSM825646	GSE33382	Osteosarcoma	Bone	5.84E-14	4.56E-01	3.14E-10	8.15E-01
3100	GSM825647	GSE33382	Osteosarcoma	Bone	2.48E-22	5.83E-01	4.32E-10	8.10E-01
3101	GSM825648	GSE33382	Osteosarcoma	Bone	5.68E-16	4.89E-01	3.14E-10	8.15E-01
3102	GSM825649	GSE33382	Osteosarcoma	Bone	5.26E-16	4.90E-01	1.23E-08	7.44E-01
3103	GSM825650	GSE33382	Osteosarcoma	Bone	6.41E-11	4.00E-01	2.63E-07	6.79E-01

3104	GSM825651	GSE33382	Osteosarcoma	Bone	8.36E-19	5.33E-01	5.92E-10	8.04E-01
3105	GSM825652	GSE33382	Osteosarcoma	Bone	5.78E-15	4.73E-01	5.12E-08	7.14E-01
3106	GSM825653	GSE33382	Osteosarcoma	Bone	3.05E-26	6.33E-01	1.58E-13	9.46E-01
3107	GSM825654	GSE33382	Osteosarcoma	Bone	1.74E-11	4.11E-01	1.17E-07	6.96E-01
3108	GSM825655	GSE33382	Osteosarcoma	Bone	1.23E-18	5.31E-01	2.26E-11	8.63E-01
3109	GSM825656	GSE33382	Osteosarcoma	Bone	6.29E-21	5.63E-01	3.14E-10	8.15E-01
3110	GSM825657	GSE33382	Osteosarcoma	Bone	2.91E-20	5.54E-01	8.58E-11	8.39E-01
3111	GSM825658	GSE33382	Osteosarcoma	Bone	2.41E-13	4.45E-01	2.19E-08	7.32E-01
3112	GSM825659	GSE33382	Osteosarcoma	Bone	2.19E-21	5.70E-01	8.10E-10	7.98E-01
3113	GSM825660	GSE33382	Osteosarcoma	Bone	1.33E-18	5.30E-01	1.23E-08	7.44E-01
3114	GSM825661	GSE33382	Osteosarcoma	Bone	2.87E-10	3.87E-01	1.60E-06	6.37E-01
3115	GSM825662	GSE33382	Osteosarcoma	Bone	6.58E-19	5.35E-01	1.23E-08	7.44E-01
3116	GSM825663	GSE33382	Osteosarcoma	Bone	1.39E-20	5.59E-01	4.76E-13	9.29E-01
3117	GSM825664	GSE33382	Osteosarcoma	Bone	1.47E-11	4.13E-01	3.43E-07	6.73E-01
3118	GSM825665	GSE33382	Osteosarcoma	Bone	1.27E-17	5.15E-01	6.17E-11	8.45E-01
3119	GSM825666	GSE33382	Osteosarcoma	Bone	1.13E-18	5.31E-01	4.32E-10	8.10E-01
3120	GSM825667	GSE33382	Osteosarcoma	Bone	1.23E-20	5.59E-01	2.26E-11	8.63E-01
3121	GSM825668	GSE33382	Osteosarcoma	Bone	2.24E-16	4.96E-01	1.65E-10	8.27E-01
3122	GSM825669	GSE33382	Osteosarcoma	Bone	3.02E-24	6.08E-01	2.85E-12	8.99E-01
3123	GSM825670	GSE33382	Osteosarcoma	Bone	6.85E-18	5.19E-01	2.77E-09	7.74E-01
3124	GSM825671	GSE33382	Osteosarcoma	Bone	9.57E-17	5.02E-01	1.23E-08	7.44E-01
3125	GSM825672	GSE33382	Osteosarcoma	Bone	6.76E-17	5.04E-01	4.32E-10	8.10E-01
3126	GSM825673	GSE33382	Osteosarcoma	Bone	1.63E-13	4.48E-01	8.91E-08	7.02E-01
3127	GSM825674	GSE33382	Osteosarcoma	Bone	2.22E-13	4.46E-01	1.54E-07	6.90E-01
3128	GSM825675	GSE33382	Osteosarcoma	Bone	1.32E-21	5.73E-01	1.65E-10	8.27E-01
3129	GSM825676	GSE33382	Osteosarcoma	Bone	1.05E-15	4.85E-01	1.65E-10	8.27E-01
3130	GSM825677	GSE33382	Osteosarcoma	Bone	5.36E-15	4.74E-01	6.81E-09	7.56E-01
3131	GSM825678	GSE33382	Osteosarcoma	Bone	3.78E-12	4.24E-01	5.12E-08	7.14E-01
3132	GSM825679	GSE33382	Osteosarcoma	Bone	9.81E-18	5.17E-01	2.85E-12	8.99E-01
3133	GSM825680	GSE33382	Osteosarcoma	Bone	2.21E-16	4.96E-01	3.75E-09	7.68E-01
3134	GSM825681	GSE33382	Osteosarcoma	Bone	5.36E-14	4.57E-01	1.17E-07	6.96E-01
3135	GSM825682	GSE33382	Osteosarcoma	Bone	1.13E-20	5.60E-01	2.05E-09	7.80E-01
3136	GSM825683	GSE33382	Osteosarcoma	Bone	2.78E-19	5.40E-01	8.12E-12	8.81E-01
3137	GSM825684	GSE33382	Osteosarcoma	Bone	1.99E-15	4.81E-01	5.06E-09	7.62E-01
3138	GSM825685	GSE33382	Osteosarcoma	Bone	5.68E-16	4.89E-01	3.86E-08	7.20E-01
3139	GSM825686	GSE33382	Osteosarcoma	Bone	5.67E-17	5.05E-01	8.58E-11	8.39E-01
3140	GSM825687	GSE33382	Osteosarcoma	Bone	4.46E-22	5.79E-01	4.32E-10	8.10E-01
3141	GSM825688	GSE33382	Osteosarcoma	Bone	3.03E-15	4.78E-01	2.91E-08	7.26E-01
3142	GSM825689	GSE33382	Osteosarcoma	Bone	1.13E-18	5.31E-01	1.19E-10	8.33E-01
3143	GSM825690	GSE33382	Osteosarcoma	Bone	9.38E-19	5.32E-01	3.75E-09	7.68E-01
3144	GSM825691	GSE33382	Osteosarcoma	Bone	4.54E-19	5.37E-01	3.17E-11	8.57E-01
3145	GSM825692	GSE33382	Osteosarcoma	Bone	1.98E-12	4.29E-01	1.24E-06	6.43E-01
3146	GSM825693	GSE33382	Osteosarcoma	Bone	7.55E-14	4.54E-01	1.51E-09	7.86E-01
3147	GSM825694	GSE33382	Osteosarcoma	Bone	9.71E-17	5.02E-01	3.75E-09	7.68E-01
3148	GSM825695	GSE33382	Osteosarcoma	Bone	3.32E-15	4.77E-01	1.11E-09	7.92E-01
3149	GSM825696	GSE33382	Osteosarcoma	Bone	3.97E-20	5.52E-01	2.26E-11	8.63E-01
3150	GSM825697	GSE33382	Osteosarcoma	Bone	2.18E-15	4.80E-01	2.01E-07	6.85E-01
3151	GSM825698	GSE33382	Osteosarcoma	Bone	1.77E-09	3.71E-01	7.47E-07	6.55E-01
3152	GSM825699	GSE33382	Osteosarcoma	Bone	2.64E-07	3.22E-01	1.54E-07	6.90E-01
3153	GSM825700	GSE33382	Osteosarcoma	Bone	2.76E-22	5.82E-01	6.17E-11	8.45E-01
3154	GSM825701	GSE33382	Osteosarcoma	Bone	2.66E-16	4.95E-01	9.15E-09	7.50E-01
3155	GSM825702	GSE33382	Osteosarcoma	Bone	5.35E-12	4.21E-01	1.11E-09	7.92E-01
3156	GSM825703	GSE33382	Osteosarcoma	Bone	1.89E-16	4.97E-01	8.10E-10	7.98E-01
3157	GSM825704	GSE33382	Osteosarcoma	Bone	7.74E-12	4.18E-01	5.06E-09	7.62E-01
3158	GSM825705	GSE33382	Osteosarcoma	Bone	1.65E-17	5.14E-01	1.14E-11	8.75E-01
3159	GSM825706	GSE33382	Osteosarcoma	Bone	1.92E-18	5.28E-01	2.77E-09	7.74E-01
3160	GSM825707	GSE33382	Osteosarcoma	Bone	4.43E-12	4.22E-01	2.77E-09	7.74E-01
3161	GSM825708	GSE33382	Osteosarcoma	Bone	9.38E-19	5.32E-01	5.06E-09	7.62E-01
3162	GSM825709	GSE33382	Osteosarcoma	Bone	1.78E-19	5.43E-01	2.05E-09	7.80E-01
3163	AA05	TCGA.GBM	Glioblastoma	CNS	5.51E-25	5.91E-01	4.69E-12	8.29E-01
3164	AA07	TCGA.GBM	Glioblastoma	CNS	6.64E-25	5.90E-01	4.87E-11	7.91E-01
3165	AB05	TCGA.GBM	Glioblastoma	CNS	1.69E-24	5.85E-01	1.15E-11	8.14E-01
3166	AB07	TCGA.GBM	Glioblastoma	CNS	7.74E-20	5.25E-01	2.63E-12	8.38E-01
3167	AC05	TCGA.GBM	Glioblastoma	CNS	1.59E-28	6.33E-01	8.34E-12	8.20E-01
3168	AC07	TCGA.GBM	Glioblastoma	CNS	1.71E-20	5.34E-01	5.66E-10	7.49E-01
3169	AD05	TCGA.GBM	Glioblastoma	CNS	3.15E-23	5.69E-01	1.45E-10	7.72E-01
3170	AD07	TCGA.GBM	Glioblastoma	CNS	7.84E-28	6.25E-01	1.30E-11	8.12E-01
3171	AE05	TCGA.GBM	Glioblastoma	CNS	1.84E-21	5.47E-01	3.51E-09	7.16E-01
3172	AE07	TCGA.GBM	Glioblastoma	CNS	1.16E-27	6.23E-01	1.45E-10	7.72E-01
3173	AF05	TCGA.GBM	Glioblastoma	CNS	1.08E-19	5.23E-01	5.66E-10	7.49E-01
3174	AF07	TCGA.GBM	Glioblastoma	CNS	1.44E-20	5.35E-01	2.03E-11	8.05E-01
3175	AG05	TCGA.GBM	Glioblastoma	CNS	1.08E-19	5.23E-01	2.21E-10	7.65E-01
3176	AG07	TCGA.GBM	Glioblastoma	CNS	5.51E-25	5.91E-01	1.45E-10	7.72E-01
3177	AH05	TCGA.GBM	Glioblastoma	CNS	2.58E-29	6.42E-01	1.66E-12	8.45E-01

3178	AH07	TCGA.GBM	Glioblastoma	CNS	3.87E-29	6.40E-01	1.80E-11	8.07E-01
3179	BA10	TCGA.GBM	Glioblastoma	CNS	2.63E-23	5.70E-01	6.33E-10	7.47E-01
3180	BA11	TCGA.GBM	Glioblastoma	CNS	2.03E-24	5.84E-01	1.45E-10	7.72E-01
3181	BB08	TCGA.GBM	Glioblastoma	CNS	2.09E-19	5.19E-01	5.48E-11	7.89E-01
3182	BB09	TCGA.GBM	Glioblastoma	CNS	2.10E-27	6.20E-01	3.15E-11	7.98E-01
3183	BB10	TCGA.GBM	Glioblastoma	CNS	5.84E-35	7.02E-01	1.27E-14	9.18E-01
3184	BB11	TCGA.GBM	Glioblastoma	CNS	9.26E-23	5.63E-01	1.97E-10	7.67E-01
3185	BC08	TCGA.GBM	Glioblastoma	CNS	1.22E-26	6.11E-01	2.03E-11	8.05E-01
3186	BC09	TCGA.GBM	Glioblastoma	CNS	2.94E-24	5.82E-01	5.48E-11	7.89E-01
3187	BC10	TCGA.GBM	Glioblastoma	CNS	2.03E-24	5.84E-01	3.35E-10	7.58E-01
3188	BD08	TCGA.GBM	Glioblastoma	CNS	3.21E-22	5.57E-01	1.42E-09	7.32E-01
3189	BD09	TCGA.GBM	Glioblastoma	CNS	2.15E-25	5.96E-01	1.42E-09	7.32E-01
3190	BD10	TCGA.GBM	Glioblastoma	CNS	9.20E-22	5.51E-01	2.47E-10	7.63E-01
3191	BE08	TCGA.GBM	Glioblastoma	CNS	6.29E-31	6.60E-01	1.20E-13	8.85E-01
3192	BE09	TCGA.GBM	Glioblastoma	CNS	5.41E-23	5.66E-01	9.51E-10	7.40E-01
3193	BE10	TCGA.GBM	Glioblastoma	CNS	1.55E-21	5.48E-01	1.29E-10	7.74E-01
3194	BF08	TCGA.GBM	Glioblastoma	CNS	1.50E-19	5.21E-01	1.45E-10	7.72E-01
3195	BF09	TCGA.GBM	Glioblastoma	CNS	6.16E-18	4.98E-01	3.90E-09	7.14E-01
3196	BF10	TCGA.GBM	Glioblastoma	CNS	1.27E-19	5.22E-01	5.48E-11	7.89E-01
3197	BG03	TCGA.GBM	Glioblastoma	CNS	2.44E-24	5.83E-01	3.55E-11	7.96E-01
3198	BG08	TCGA.GBM	Glioblastoma	CNS	7.37E-24	5.77E-01	4.87E-11	7.91E-01
3199	BG09	TCGA.GBM	Glioblastoma	CNS	3.83E-22	5.56E-01	5.66E-10	7.49E-01
3200	BG10	TCGA.GBM	Glioblastoma	CNS	4.69E-20	5.28E-01	8.42E-11	7.82E-01
3201	BH03	TCGA.GBM	Glioblastoma	CNS	6.16E-21	5.40E-01	3.35E-10	7.58E-01
3202	BH09	TCGA.GBM	Glioblastoma	CNS	6.13E-24	5.78E-01	2.21E-10	7.65E-01
3203	BH10	TCGA.GBM	Glioblastoma	CNS	4.52E-23	5.67E-01	8.42E-11	7.82E-01
3204	DA01	TCGA.GBM	Glioblastoma	CNS	1.47E-25	5.98E-01	7.38E-12	8.22E-01
3205	DA04	TCGA.GBM	Glioblastoma	CNS	4.69E-20	5.28E-01	3.35E-10	7.58E-01
3206	DA05	TCGA.GBM	Glioblastoma	CNS	1.84E-21	5.47E-01	5.06E-10	7.51E-01
3207	DA06	TCGA.GBM	Glioblastoma	CNS	1.28E-15	4.63E-01	3.21E-08	6.74E-01
3208	DB03	TCGA.GBM	Glioblastoma	CNS	1.06E-23	5.75E-01	2.12E-09	7.25E-01
3209	DB04	TCGA.GBM	Glioblastoma	CNS	4.73E-19	5.14E-01	2.12E-09	7.25E-01
3210	DB05	TCGA.GBM	Glioblastoma	CNS	4.56E-25	5.92E-01	3.35E-10	7.58E-01
3211	DB06	TCGA.GBM	Glioblastoma	CNS	2.19E-21	5.46E-01	1.42E-09	7.32E-01
3212	DC01	TCGA.GBM	Glioblastoma	CNS	3.43E-17	4.87E-01	3.51E-09	7.16E-01
3213	DC02	TCGA.GBM	Glioblastoma	CNS	2.64E-26	6.07E-01	3.56E-13	8.69E-01
3214	DC03	TCGA.GBM	Glioblastoma	CNS	9.29E-30	6.47E-01	5.72E-13	8.62E-01
3215	DC04	TCGA.GBM	Glioblastoma	CNS	4.52E-23	5.67E-01	3.35E-10	7.58E-01
3216	DC05	TCGA.GBM	Glioblastoma	CNS	2.63E-23	5.70E-01	9.16E-13	8.54E-01
3217	DC06	TCGA.GBM	Glioblastoma	CNS	1.40E-24	5.86E-01	1.66E-12	8.45E-01
3218	DD01	TCGA.GBM	Glioblastoma	CNS	3.97E-20	5.29E-01	3.55E-11	7.96E-01
3219	DD02	TCGA.GBM	Glioblastoma	CNS	1.89E-22	5.60E-01	4.69E-12	8.29E-01
3220	DD03	TCGA.GBM	Glioblastoma	CNS	2.63E-23	5.70E-01	3.35E-10	7.58E-01
3221	DD04	TCGA.GBM	Glioblastoma	CNS	4.57E-22	5.55E-01	1.42E-09	7.32E-01
3222	DD05	TCGA.GBM	Glioblastoma	CNS	1.39E-32	6.78E-01	5.72E-13	8.62E-01
3223	DD06	TCGA.GBM	Glioblastoma	CNS	1.22E-25	5.99E-01	3.55E-11	7.96E-01
3224	DE01	TCGA.GBM	Glioblastoma	CNS	3.21E-22	5.57E-01	9.51E-10	7.40E-01
3225	DE02	TCGA.GBM	Glioblastoma	CNS	2.56E-27	6.19E-01	7.38E-12	8.22E-01
3226	DE03	TCGA.GBM	Glioblastoma	CNS	1.08E-19	5.23E-01	3.35E-10	7.58E-01
3227	DE04	TCGA.GBM	Glioblastoma	CNS	7.57E-30	6.48E-01	1.66E-12	8.45E-01
3228	DE05	TCGA.GBM	Glioblastoma	CNS	2.84E-20	5.31E-01	5.18E-09	7.09E-01
3229	DF02	TCGA.GBM	Glioblastoma	CNS	2.03E-24	5.84E-01	2.03E-11	8.05E-01
3230	DF03	TCGA.GBM	Glioblastoma	CNS	1.44E-20	5.35E-01	8.51E-10	7.42E-01
3231	DF04	TCGA.GBM	Glioblastoma	CNS	1.58E-22	5.61E-01	3.75E-10	7.56E-01
3232	DF05	TCGA.GBM	Glioblastoma	CNS	1.40E-29	6.45E-01	3.56E-13	8.69E-01
3233	DF06	TCGA.GBM	Glioblastoma	CNS	8.66E-21	5.38E-01	2.47E-10	7.63E-01
3234	DG01	TCGA.GBM	Glioblastoma	CNS	1.47E-25	5.98E-01	2.63E-12	8.38E-01
3235	DG02	TCGA.GBM	Glioblastoma	CNS	4.56E-25	5.92E-01	8.51E-10	7.42E-01
3236	DG03	TCGA.GBM	Glioblastoma	CNS	2.40E-20	5.32E-01	1.81E-08	6.85E-01
3237	DG04	TCGA.GBM	Glioblastoma	CNS	2.90E-19	5.17E-01	5.18E-09	7.09E-01
3238	DG05	TCGA.GBM	Glioblastoma	CNS	4.25E-24	5.80E-01	3.15E-11	7.98E-01
3239	DG06	TCGA.GBM	Glioblastoma	CNS	1.69E-24	5.85E-01	2.03E-11	8.05E-01
3240	DH01	TCGA.GBM	Glioblastoma	CNS	1.83E-23	5.72E-01	5.48E-11	7.89E-01
3241	DH02	TCGA.GBM	Glioblastoma	CNS	1.27E-23	5.74E-01	1.15E-11	8.14E-01
3242	DH03	TCGA.GBM	Glioblastoma	CNS	8.32E-26	6.01E-01	3.55E-11	7.96E-01
3243	DH05	TCGA.GBM	Glioblastoma	CNS	2.64E-26	6.07E-01	5.66E-10	7.49E-01
3244	EA02	TCGA.GBM	Glioblastoma	CNS	7.70E-19	5.11E-01	1.42E-09	7.32E-01
3245	EA04	TCGA.GBM	Glioblastoma	CNS	7.72E-22	5.52E-01	1.80E-11	8.07E-01
3246	EA05	TCGA.GBM	Glioblastoma	CNS	1.01E-36	7.20E-01	3.93E-14	9.02E-01
3247	EA06	TCGA.GBM	Glioblastoma	CNS	7.30E-21	5.39E-01	4.87E-11	7.91E-01
3248	EA07	TCGA.GBM	Glioblastoma	CNS	4.25E-24	5.80E-01	1.66E-12	8.45E-01
3249	EA08	TCGA.GBM	Glioblastoma	CNS	5.26E-18	4.99E-01	4.87E-11	7.91E-01
3250	EA09	TCGA.GBM	Glioblastoma	CNS	8.01E-25	5.89E-01	1.29E-10	7.74E-01
3251	EA10	TCGA.GBM	Glioblastoma	CNS	1.58E-22	5.61E-01	2.03E-11	8.05E-01

3252	EA11	TCGA.GBM	Glioblastoma	CNS	9.65E-25	5.88E-01	3.15E-11	7.98E-01
3253	EB01	TCGA.GBM	Glioblastoma	CNS	1.42E-27	6.22E-01	1.15E-11	8.14E-01
3254	EB02	TCGA.GBM	Glioblastoma	CNS	9.14E-20	5.24E-01	1.29E-10	7.74E-01
3255	EB03	TCGA.GBM	Glioblastoma	CNS	1.53E-23	5.73E-01	5.66E-10	7.49E-01
3256	EB05	TCGA.GBM	Glioblastoma	CNS	4.25E-24	5.80E-01	2.63E-12	8.38E-01
3257	EB08	TCGA.GBM	Glioblastoma	CNS	5.60E-27	6.15E-01	1.30E-11	8.12E-01
3258	EB09	TCGA.GBM	Glioblastoma	CNS	1.50E-19	5.21E-01	3.15E-09	7.18E-01
3259	EB10	TCGA.GBM	Glioblastoma	CNS	7.74E-20	5.25E-01	3.15E-11	7.98E-01
3260	EB11	TCGA.GBM	Glioblastoma	CNS	2.40E-20	5.32E-01	4.87E-11	7.91E-01
3261	EB12	TCGA.GBM	Glioblastoma	CNS	3.79E-27	6.17E-01	9.16E-13	8.54E-01
3262	EC01	TCGA.GBM	Glioblastoma	CNS	6.80E-27	6.14E-01	1.45E-10	7.72E-01
3263	EC02	TCGA.GBM	Glioblastoma	CNS	8.01E-25	5.89E-01	1.66E-12	8.45E-01
3264	EC03	TCGA.GBM	Glioblastoma	CNS	2.90E-19	5.17E-01	4.87E-11	7.91E-01
3265	EC06	TCGA.GBM	Glioblastoma	CNS	2.56E-27	6.19E-01	4.69E-12	8.29E-01
3266	EC08	TCGA.GBM	Glioblastoma	CNS	1.01E-25	6.00E-01	8.51E-10	7.42E-01
3267	EC12	TCGA.GBM	Glioblastoma	CNS	9.65E-25	5.88E-01	1.30E-11	8.12E-01
3268	FA02	TCGA.GBM	Glioblastoma	CNS	7.30E-21	5.39E-01	1.90E-09	7.27E-01
3269	FA03	TCGA.GBM	Glioblastoma	CNS	3.10E-21	5.44E-01	9.51E-10	7.40E-01
3270	FA04	TCGA.GBM	Glioblastoma	CNS	2.64E-26	6.07E-01	1.45E-10	7.72E-01
3271	FA05	TCGA.GBM	Glioblastoma	CNS	2.63E-23	5.70E-01	2.47E-10	7.63E-01
3272	FA06	TCGA.GBM	Glioblastoma	CNS	1.58E-22	5.61E-01	2.12E-09	7.25E-01
3273	FA07	TCGA.GBM	Glioblastoma	CNS	1.89E-22	5.60E-01	2.12E-09	7.25E-01
3274	FA08	TCGA.GBM	Glioblastoma	CNS	2.38E-18	5.04E-01	5.06E-10	7.51E-01
3275	FA09	TCGA.GBM	Glioblastoma	CNS	6.48E-23	5.65E-01	7.38E-12	8.22E-01
3276	FA10	TCGA.GBM	Glioblastoma	CNS	1.03E-20	5.37E-01	3.35E-10	7.58E-01
3277	FB01	TCGA.GBM	Glioblastoma	CNS	2.38E-18	5.04E-01	1.81E-08	6.85E-01
3278	FB02	TCGA.GBM	Glioblastoma	CNS	1.10E-21	5.50E-01	3.75E-10	7.56E-01
3279	FB03	TCGA.GBM	Glioblastoma	CNS	2.17E-26	6.08E-01	2.03E-11	8.05E-01
3280	FB04	TCGA.GBM	Glioblastoma	CNS	1.16E-24	5.87E-01	3.15E-11	7.98E-01
3281	FB05	TCGA.GBM	Glioblastoma	CNS	6.13E-24	5.78E-01	8.42E-11	7.82E-01
3282	FB06	TCGA.GBM	Glioblastoma	CNS	2.20E-23	5.71E-01	3.75E-10	7.56E-01
3283	FB07	TCGA.GBM	Glioblastoma	CNS	6.80E-27	6.14E-01	5.72E-13	8.62E-01
3284	FB08	TCGA.GBM	Glioblastoma	CNS	5.19E-21	5.41E-01	3.55E-11	7.96E-01
3285	FB09	TCGA.GBM	Glioblastoma	CNS	1.69E-24	5.85E-01	9.46E-11	7.80E-01
3286	FB10	TCGA.GBM	Glioblastoma	CNS	6.35E-17	4.83E-01	1.05E-07	6.50E-01
3287	FC01	TCGA.GBM	Glioblastoma	CNS	1.06E-23	5.75E-01	1.45E-10	7.72E-01
3288	FC02	TCGA.GBM	Glioblastoma	CNS	3.21E-22	5.57E-01	1.42E-09	7.32E-01
3289	FC03	TCGA.GBM	Glioblastoma	CNS	1.10E-21	5.50E-01	5.06E-10	7.51E-01
3290	FC04	TCGA.GBM	Glioblastoma	CNS	3.78E-25	5.93E-01	2.03E-11	8.05E-01
3291	FC05	TCGA.GBM	Glioblastoma	CNS	8.84E-24	5.76E-01	1.29E-10	7.74E-01
3292	FC06	TCGA.GBM	Glioblastoma	CNS	9.29E-30	6.47E-01	3.56E-13	8.69E-01
3293	FC07	TCGA.GBM	Glioblastoma	CNS	1.22E-20	5.36E-01	1.29E-10	7.74E-01
3294	FC08	TCGA.GBM	Glioblastoma	CNS	8.26E-27	6.13E-01	3.55E-11	7.96E-01
3295	FC09	TCGA.GBM	Glioblastoma	CNS	2.94E-24	5.82E-01	8.42E-11	7.82E-01
3296	FC10	TCGA.GBM	Glioblastoma	CNS	2.79E-18	5.03E-01	1.37E-08	6.90E-01
3297	FD01	TCGA.GBM	Glioblastoma	CNS	3.35E-20	5.30E-01	1.24E-08	6.92E-01
3298	FD02	TCGA.GBM	Glioblastoma	CNS	8.66E-21	5.38E-01	1.45E-10	7.72E-01
3299	FD03	TCGA.GBM	Glioblastoma	CNS	1.10E-21	5.50E-01	1.29E-10	7.74E-01
3300	FD04	TCGA.GBM	Glioblastoma	CNS	6.87E-26	6.02E-01	6.33E-10	7.47E-01
3301	FD05	TCGA.GBM	Glioblastoma	CNS	4.56E-25	5.92E-01	1.15E-11	8.14E-01
3302	FD06	TCGA.GBM	Glioblastoma	CNS	1.59E-28	6.33E-01	7.38E-12	8.22E-01
3303	FD07	TCGA.GBM	Glioblastoma	CNS	1.84E-21	5.47E-01	5.48E-11	7.89E-01
3304	FD08	TCGA.GBM	Glioblastoma	CNS	8.44E-18	4.96E-01	7.36E-08	6.58E-01
3305	FD09	TCGA.GBM	Glioblastoma	CNS	1.06E-23	5.75E-01	8.42E-11	7.82E-01
3306	FD10	TCGA.GBM	Glioblastoma	CNS	2.64E-26	6.07E-01	2.03E-11	8.05E-01
3307	FE01	TCGA.GBM	Glioblastoma	CNS	7.74E-20	5.25E-01	1.80E-11	8.07E-01
3308	FE02	TCGA.GBM	Glioblastoma	CNS	1.27E-23	5.74E-01	9.16E-13	8.54E-01
3309	FE03	TCGA.GBM	Glioblastoma	CNS	1.50E-19	5.21E-01	1.29E-10	7.74E-01
3310	FE04	TCGA.GBM	Glioblastoma	CNS	5.19E-21	5.41E-01	1.29E-10	7.74E-01
3311	FE05	TCGA.GBM	Glioblastoma	CNS	2.63E-23	5.70E-01	5.72E-13	8.62E-01
3312	FE06	TCGA.GBM	Glioblastoma	CNS	3.41E-19	5.16E-01	1.29E-10	7.74E-01
3313	FE07	TCGA.GBM	Glioblastoma	CNS	1.22E-20	5.36E-01	6.33E-10	7.47E-01
3314	FE08	TCGA.GBM	Glioblastoma	CNS	1.00E-26	6.12E-01	7.38E-12	8.22E-01
3315	FE09	TCGA.GBM	Glioblastoma	CNS	4.25E-24	5.80E-01	3.15E-11	7.98E-01
3316	FE10	TCGA.GBM	Glioblastoma	CNS	7.37E-24	5.77E-01	4.69E-12	8.29E-01
3317	FF01	TCGA.GBM	Glioblastoma	CNS	5.54E-20	5.27E-01	8.51E-10	7.42E-01
3318	FF02	TCGA.GBM	Glioblastoma	CNS	4.57E-22	5.55E-01	7.38E-12	8.22E-01
3319	FF03	TCGA.GBM	Glioblastoma	CNS	2.84E-20	5.31E-01	1.59E-09	7.30E-01
3320	FF04	TCGA.GBM	Glioblastoma	CNS	7.74E-20	5.25E-01	3.35E-10	7.58E-01
3321	FF05	TCGA.GBM	Glioblastoma	CNS	8.70E-29	6.36E-01	1.30E-11	8.12E-01
3322	FF06	TCGA.GBM	Glioblastoma	CNS	4.25E-24	5.80E-01	4.69E-12	8.29E-01
3323	FF07	TCGA.GBM	Glioblastoma	CNS	1.03E-20	5.37E-01	3.75E-10	7.56E-01
3324	FF09	TCGA.GBM	Glioblastoma	CNS	7.57E-30	6.48E-01	1.94E-13	8.78E-01
3325	FF10	TCGA.GBM	Glioblastoma	CNS	4.56E-25	5.92E-01	7.38E-12	8.22E-01

3326	FG01	TCGA.GBM	Glioblastoma	CNS	1.45E-30	6.56E-01	1.66E-12	8.45E-01
3327	FG03	TCGA.GBM	Glioblastoma	CNS	1.78E-25	5.97E-01	2.03E-11	8.05E-01
3328	FG08	TCGA.GBM	Glioblastoma	CNS	5.10E-31	6.61E-01	1.94E-13	8.78E-01
3329	FH02	TCGA.GBM	Glioblastoma	CNS	6.64E-25	5.90E-01	3.15E-11	7.98E-01
3330	FH03	TCGA.GBM	Glioblastoma	CNS	6.55E-20	5.26E-01	1.42E-09	7.32E-01
3331	FH04	TCGA.GBM	Glioblastoma	CNS	7.37E-24	5.77E-01	3.35E-10	7.58E-01
3332	FH05	TCGA.GBM	Glioblastoma	CNS	9.20E-22	5.51E-01	8.51E-10	7.42E-01
3333	FH07	TCGA.GBM	Glioblastoma	CNS	2.60E-25	5.95E-01	8.42E-11	7.82E-01
3334	FH08	TCGA.GBM	Glioblastoma	CNS	1.16E-27	6.23E-01	1.66E-12	8.45E-01
3335	FH09	TCGA.GBM	Glioblastoma	CNS	3.27E-18	5.02E-01	3.90E-09	7.14E-01
3336	GA01	TCGA.GBM	Glioblastoma	CNS	9.28E-40	7.50E-01	4.00E-15	9.34E-01
3337	GA02	TCGA.GBM	Glioblastoma	CNS	3.35E-20	5.30E-01	3.15E-11	7.98E-01
3338	GA03	TCGA.GBM	Glioblastoma	CNS	7.72E-22	5.52E-01	1.37E-08	6.90E-01
3339	GA04	TCGA.GBM	Glioblastoma	CNS	8.84E-24	5.76E-01	8.51E-10	7.42E-01
3340	GA05	TCGA.GBM	Glioblastoma	CNS	3.53E-24	5.81E-01	5.48E-11	7.89E-01
3341	GA06	TCGA.GBM	Glioblastoma	CNS	1.32E-22	5.62E-01	6.16E-11	7.87E-01
3342	GA07	TCGA.GBM	Glioblastoma	CNS	3.10E-21	5.44E-01	1.45E-10	7.72E-01
3343	GA09	TCGA.GBM	Glioblastoma	CNS	1.11E-22	5.63E-01	5.66E-10	7.49E-01
3344	GA10	TCGA.GBM	Glioblastoma	CNS	7.74E-20	5.25E-01	2.47E-10	7.63E-01
3345	GA11	TCGA.GBM	Glioblastoma	CNS	2.90E-19	5.17E-01	1.15E-11	8.14E-01
3346	GA12	TCGA.GBM	Glioblastoma	CNS	8.26E-27	6.13E-01	2.63E-12	8.38E-01
3347	GB01	TCGA.GBM	Glioblastoma	CNS	6.48E-23	5.65E-01	3.15E-11	7.98E-01
3348	GB02	TCGA.GBM	Glioblastoma	CNS	1.22E-26	6.11E-01	4.69E-12	8.29E-01
3349	GB03	TCGA.GBM	Glioblastoma	CNS	1.30E-21	5.49E-01	1.29E-10	7.74E-01
3350	GB04	TCGA.GBM	Glioblastoma	CNS	9.26E-23	5.63E-01	5.48E-11	7.89E-01
3351	GB07	TCGA.GBM	Glioblastoma	CNS	1.50E-19	5.21E-01	9.46E-11	7.80E-01
3352	GB08	TCGA.GBM	Glioblastoma	CNS	6.48E-22	5.53E-01	5.76E-09	7.07E-01
3353	GB09	TCGA.GBM	Glioblastoma	CNS	3.53E-24	5.81E-01	1.29E-10	7.74E-01
3354	GB10	TCGA.GBM	Glioblastoma	CNS	5.28E-13	4.21E-01	1.37E-08	6.90E-01
3355	GB11	TCGA.GBM	Glioblastoma	CNS	3.83E-22	5.56E-01	8.42E-11	7.82E-01
3356	GB12	TCGA.GBM	Glioblastoma	CNS	5.51E-25	5.91E-01	2.21E-10	7.65E-01
3357	GC01	TCGA.GBM	Glioblastoma	CNS	4.60E-27	6.16E-01	2.63E-12	8.38E-01
3358	GC04	TCGA.GBM	Glioblastoma	CNS	5.41E-23	5.66E-01	3.75E-10	7.56E-01
3359	GC05	TCGA.GBM	Glioblastoma	CNS	3.10E-21	5.44E-01	5.66E-10	7.49E-01
3360	GC06	TCGA.GBM	Glioblastoma	CNS	1.83E-23	5.72E-01	2.21E-10	7.65E-01
3361	GC07	TCGA.GBM	Glioblastoma	CNS	1.48E-26	6.10E-01	2.03E-11	8.05E-01
3362	GC08	TCGA.GBM	Glioblastoma	CNS	1.10E-21	5.50E-01	1.59E-09	7.30E-01
3363	GC09	TCGA.GBM	Glioblastoma	CNS	2.20E-23	5.71E-01	7.38E-12	8.22E-01
3364	GC10	TCGA.GBM	Glioblastoma	CNS	1.55E-21	5.48E-01	5.06E-10	7.51E-01
3365	GD01	TCGA.GBM	Glioblastoma	CNS	6.48E-23	5.65E-01	6.33E-10	7.47E-01
3366	GD02	TCGA.GBM	Glioblastoma	CNS	3.27E-18	5.02E-01	5.66E-10	7.49E-01
3367	GD03	TCGA.GBM	Glioblastoma	CNS	2.84E-20	5.31E-01	1.15E-11	8.14E-01
3368	GD04	TCGA.GBM	Glioblastoma	CNS	3.11E-27	6.18E-01	2.63E-12	8.38E-01
3369	GD05	TCGA.GBM	Glioblastoma	CNS	3.13E-25	5.94E-01	1.45E-10	7.72E-01
3370	GD06	TCGA.GBM	Glioblastoma	CNS	5.27E-28	6.27E-01	7.38E-12	8.22E-01
3371	GD07	TCGA.GBM	Glioblastoma	CNS	4.37E-21	5.42E-01	8.51E-10	7.42E-01
3372	GD10	TCGA.GBM	Glioblastoma	CNS	4.56E-25	5.92E-01	9.16E-13	8.54E-01
3373	GD11	TCGA.GBM	Glioblastoma	CNS	1.84E-21	5.47E-01	1.45E-10	7.72E-01
3374	GD12	TCGA.GBM	Glioblastoma	CNS	7.75E-23	5.64E-01	4.69E-12	8.29E-01
3375	GE01	TCGA.GBM	Glioblastoma	CNS	1.77E-19	5.20E-01	3.35E-10	7.58E-01
3376	GE03	TCGA.GBM	Glioblastoma	CNS	2.61E-21	5.45E-01	8.51E-10	7.42E-01
3377	GE04	TCGA.GBM	Glioblastoma	CNS	9.20E-22	5.51E-01	8.42E-11	7.82E-01
3378	GE06	TCGA.GBM	Glioblastoma	CNS	9.01E-33	6.80E-01	6.43E-14	8.94E-01
3379	GE07	TCGA.GBM	Glioblastoma	CNS	1.40E-24	5.86E-01	3.15E-11	7.98E-01
3380	GE08	TCGA.GBM	Glioblastoma	CNS	2.46E-19	5.18E-01	1.42E-09	7.32E-01
3381	GE09	TCGA.GBM	Glioblastoma	CNS	1.03E-20	5.37E-01	4.87E-11	7.91E-01
3382	GE10	TCGA.GBM	Glioblastoma	CNS	1.83E-23	5.72E-01	2.21E-10	7.65E-01
3383	GE11	TCGA.GBM	Glioblastoma	CNS	3.13E-25	5.94E-01	2.63E-12	8.38E-01
3384	GF02	TCGA.GBM	Glioblastoma	CNS	5.44E-22	5.54E-01	9.51E-10	7.40E-01
3385	GF03	TCGA.GBM	Glioblastoma	CNS	4.25E-24	5.80E-01	2.36E-09	7.23E-01
3386	GF04	TCGA.GBM	Glioblastoma	CNS	2.61E-21	5.45E-01	1.80E-11	8.07E-01
3387	GF05	TCGA.GBM	Glioblastoma	CNS	5.60E-27	6.15E-01	2.63E-12	8.38E-01
3388	GF06	TCGA.GBM	Glioblastoma	CNS	6.16E-21	5.40E-01	1.45E-10	7.72E-01
3389	GF07	TCGA.GBM	Glioblastoma	CNS	3.15E-23	5.69E-01	1.45E-10	7.72E-01
3390	GF08	TCGA.GBM	Glioblastoma	CNS	2.94E-24	5.82E-01	3.15E-11	7.98E-01
3391	HA01	TCGA.GBM	Glioblastoma	CNS	1.16E-27	6.23E-01	2.03E-11	8.05E-01
3392	HA02	TCGA.GBM	Glioblastoma	CNS	5.19E-21	5.41E-01	1.42E-09	7.32E-01
3393	HA03	TCGA.GBM	Glioblastoma	CNS	2.60E-25	5.95E-01	3.35E-10	7.58E-01
3394	HA04	TCGA.GBM	Glioblastoma	CNS	6.16E-30	6.49E-01	5.72E-13	8.62E-01
3395	HA05	TCGA.GBM	Glioblastoma	CNS	1.10E-21	5.50E-01	1.45E-10	7.72E-01
3396	HA06	TCGA.GBM	Glioblastoma	CNS	2.56E-27	6.19E-01	4.15E-12	8.31E-01
3397	HA07	TCGA.GBM	Glioblastoma	CNS	2.03E-24	5.84E-01	3.15E-11	7.98E-01
3398	HA08	TCGA.GBM	Glioblastoma	CNS	7.57E-30	6.48E-01	5.48E-11	7.89E-01
3399	HA09	TCGA.GBM	Glioblastoma	CNS	6.16E-30	6.49E-01	1.66E-12	8.45E-01

3400	HA10	TCGA.GBM	Glioblastoma	CNS	4.57E-22	5.55E-01	2.03E-11	8.05E-01
3401	HB02	TCGA.GBM	Glioblastoma	CNS	1.72E-29	6.44E-01	4.15E-12	8.31E-01
3402	HB03	TCGA.GBM	Glioblastoma	CNS	1.50E-19	5.21E-01	3.35E-10	7.58E-01
3403	HB04	TCGA.GBM	Glioblastoma	CNS	6.16E-21	5.40E-01	3.35E-10	7.58E-01
3404	HB05	TCGA.GBM	Glioblastoma	CNS	2.56E-27	6.19E-01	7.38E-12	8.22E-01
3405	HB06	TCGA.GBM	Glioblastoma	CNS	1.83E-23	5.72E-01	3.15E-11	7.98E-01
3406	HB07	TCGA.GBM	Glioblastoma	CNS	5.27E-28	6.27E-01	5.72E-13	8.62E-01
3407	HB09	TCGA.GBM	Glioblastoma	CNS	2.63E-23	5.70E-01	3.15E-11	7.98E-01
3408	HB10	TCGA.GBM	Glioblastoma	CNS	3.68E-21	5.43E-01	2.21E-10	7.65E-01
3409	HC01	TCGA.GBM	Glioblastoma	CNS	2.19E-21	5.46E-01	1.42E-09	7.32E-01
3410	HC02	TCGA.GBM	Glioblastoma	CNS	9.65E-25	5.88E-01	8.42E-11	7.82E-01
3411	HC04	TCGA.GBM	Glioblastoma	CNS	6.48E-23	5.65E-01	3.35E-10	7.58E-01
3412	HC05	TCGA.GBM	Glioblastoma	CNS	7.72E-22	5.52E-01	2.12E-09	7.25E-01
3413	HC06	TCGA.GBM	Glioblastoma	CNS	1.47E-18	5.07E-01	3.75E-10	7.56E-01
3414	HC07	TCGA.GBM	Glioblastoma	CNS	3.83E-22	5.56E-01	9.46E-11	7.80E-01
3415	HC08	TCGA.GBM	Glioblastoma	CNS	7.72E-22	5.52E-01	5.48E-11	7.89E-01
3416	HC09	TCGA.GBM	Glioblastoma	CNS	3.68E-21	5.43E-01	2.47E-10	7.63E-01
3417	HC10	TCGA.GBM	Glioblastoma	CNS	2.69E-22	5.58E-01	9.46E-11	7.80E-01
3418	HC11	TCGA.GBM	Glioblastoma	CNS	7.72E-22	5.52E-01	3.55E-11	7.96E-01
3419	HD01	TCGA.GBM	Glioblastoma	CNS	1.83E-23	5.72E-01	5.48E-11	7.89E-01
3420	HD02	TCGA.GBM	Glioblastoma	CNS	8.44E-18	4.96E-01	4.64E-08	6.67E-01
3421	HD03	TCGA.GBM	Glioblastoma	CNS	1.53E-23	5.73E-01	1.30E-11	8.12E-01
3422	HH04	TCGA.GBM	Glioblastoma	CNS	4.60E-27	6.16E-01	8.42E-11	7.82E-01
3423	HH05	TCGA.GBM	Glioblastoma	CNS	2.94E-24	5.82E-01	8.42E-11	7.82E-01
3424	HH06	TCGA.GBM	Glioblastoma	CNS	4.73E-19	5.14E-01	3.35E-10	7.58E-01
3425	APEEK_B09_46409	TCGA.GBM	Glioblastoma	CNS	3.53E-24	5.81E-01	8.42E-11	7.82E-01
3426	APEEK_B12_46409	TCGA.GBM	Glioblastoma	CNS	2.19E-21	5.46E-01	8.42E-11	7.82E-01
3427	APEEK_C01_46408	TCGA.GBM	Glioblastoma	CNS	1.11E-22	5.63E-01	1.29E-10	7.74E-01
3428	APEEK_C02_46408	TCGA.GBM	Glioblastoma	CNS	1.27E-19	5.22E-01	2.22E-08	6.81E-01
3429	APEEK_C03_46408	TCGA.GBM	Glioblastoma	CNS	1.10E-21	5.50E-01	5.66E-10	7.49E-01
3430	APEEK_C04_46408	TCGA.GBM	Glioblastoma	CNS	1.27E-23	5.74E-01	8.42E-11	7.82E-01
3431	APEEK_C05_46410	TCGA.GBM	Glioblastoma	CNS	9.26E-23	5.63E-01	8.42E-11	7.82E-01
3432	APEEK_C06_46410	TCGA.GBM	Glioblastoma	CNS	2.61E-21	5.45E-01	2.21E-10	7.65E-01
3433	APEEK_C07_46410	TCGA.GBM	Glioblastoma	CNS	7.21E-18	4.97E-01	1.81E-08	6.85E-01
3434	APEEK_C08_46411	TCGA.GBM	Glioblastoma	CNS	3.36E-16	4.72E-01	3.35E-10	7.58E-01
3435	APEEK_C09_46409	TCGA.GBM	Glioblastoma	CNS	8.01E-25	5.89E-01	5.72E-13	8.62E-01
3436	APEEK_C10_46409	TCGA.GBM	Glioblastoma	CNS	2.44E-24	5.83E-01	1.30E-11	8.12E-01
3437	APEEK_C11_46410	TCGA.GBM	Glioblastoma	CNS	7.75E-23	5.64E-01	1.42E-09	7.32E-01
3438	APEEK_C12_46410	TCGA.GBM	Glioblastoma	CNS	3.78E-25	5.93E-01	3.15E-11	7.98E-01
3439	APEEK_D01_46412	TCGA.GBM	Glioblastoma	CNS	2.51E-36	7.16E-01	3.13E-13	8.71E-01
3440	APEEK_D02_46412	TCGA.GBM	Glioblastoma	CNS	5.81E-29	6.38E-01	2.03E-11	8.05E-01
3441	APEEK_D03_46412	TCGA.GBM	Glioblastoma	CNS	1.47E-25	5.98E-01	9.16E-13	8.54E-01
3442	APEEK_D04_46412	TCGA.GBM	Glioblastoma	CNS	9.88E-18	4.95E-01	3.35E-10	7.58E-01
3443	APEEK_D05_46411	TCGA.GBM	Glioblastoma	CNS	2.60E-25	5.95E-01	1.66E-12	8.45E-01
3444	APEEK_D06_46411	TCGA.GBM	Glioblastoma	CNS	1.72E-29	6.44E-01	1.27E-14	9.18E-01
3445	APEEK_D07_46411	TCGA.GBM	Glioblastoma	CNS	6.13E-24	5.78E-01	4.69E-12	8.29E-01
3446	APEEK_D08_46411	TCGA.GBM	Glioblastoma	CNS	1.40E-24	5.86E-01	1.45E-10	7.72E-01
3447	APEEK_D09_46402	TCGA.GBM	Glioblastoma	CNS	1.27E-23	5.74E-01	1.29E-10	7.74E-01
3448	APEEK_D10_46402	TCGA.GBM	Glioblastoma	CNS	5.27E-28	6.27E-01	2.03E-11	8.05E-01
3449	APEEK_D11_46401	TCGA.GBM	Glioblastoma	CNS	2.56E-27	6.19E-01	2.03E-11	8.05E-01
3450	APEEK_D12_46401	TCGA.GBM	Glioblastoma	CNS	6.64E-25	5.90E-01	3.75E-10	7.56E-01
3451	APEEK_E01_46402	TCGA.GBM	Glioblastoma	CNS	9.20E-22	5.51E-01	8.51E-10	7.42E-01
3452	APEEK_E02_46403	TCGA.GBM	Glioblastoma	CNS	3.13E-25	5.94E-01	5.48E-11	7.89E-01
3453	APEEK_E03_46402	TCGA.GBM	Glioblastoma	CNS	3.78E-25	5.93E-01	9.46E-11	7.80E-01
3454	APEEK_E04_46402	TCGA.GBM	Glioblastoma	CNS	4.57E-22	5.55E-01	2.90E-08	6.76E-01
3455	APEEK_E05_46400	TCGA.GBM	Glioblastoma	CNS	1.72E-18	5.06E-01	5.06E-10	7.51E-01
3456	APEEK_E06_46400	TCGA.GBM	Glioblastoma	CNS	1.06E-23	5.75E-01	2.03E-11	8.05E-01
3457	APEEK_E07_46400	TCGA.GBM	Glioblastoma	CNS	3.83E-22	5.56E-01	1.45E-10	7.72E-01
3458	APEEK_E08_46400	TCGA.GBM	Glioblastoma	CNS	1.55E-21	5.48E-01	1.97E-10	7.67E-01
3459	APEEK_E09_46401	TCGA.GBM	Glioblastoma	CNS	3.87E-26	6.05E-01	3.15E-11	7.98E-01
3460	APEEK_E10_46401	TCGA.GBM	Glioblastoma	CNS	3.83E-22	5.56E-01	8.51E-10	7.42E-01
3461	APEEK_E11_46400	TCGA.GBM	Glioblastoma	CNS	2.58E-29	6.42E-01	1.20E-13	8.85E-01
3462	APEEK_E12_46401	TCGA.GBM	Glioblastoma	CNS	7.75E-23	5.64E-01	3.35E-10	7.58E-01
3463	APEEK_F01_46405	TCGA.GBM	Glioblastoma	CNS	7.37E-24	5.77E-01	1.66E-12	8.45E-01
3464	APEEK_F02_46405	TCGA.GBM	Glioblastoma	CNS	1.89E-22	5.60E-01	1.42E-09	7.32E-01
3465	APEEK_F03_46405	TCGA.GBM	Glioblastoma	CNS	3.78E-23	5.68E-01	9.16E-13	8.54E-01
3466	APEEK_F04_46404	TCGA.GBM	Glioblastoma	CNS	1.53E-23	5.73E-01	5.48E-11	7.89E-01
3467	APEEK_F05_46406	TCGA.GBM	Glioblastoma	CNS	1.11E-22	5.63E-01	8.42E-11	7.82E-01
3468	APEEK_F06_46406	TCGA.GBM	Glioblastoma	CNS	2.63E-23	5.70E-01	1.15E-11	8.14E-01
3469	APEEK_F07_46405	TCGA.GBM	Glioblastoma	CNS	5.54E-20	5.27E-01	1.59E-09	7.30E-01
3470	APEEK_F08_46405	TCGA.GBM	Glioblastoma	CNS	3.78E-25	5.93E-01	8.42E-11	7.82E-01
3471	APEEK_F09_46403	TCGA.GBM	Glioblastoma	CNS	1.27E-23	5.74E-01	2.03E-11	8.05E-01
3472	FEAST_A01_51636	TCGA.GBM	Glioblastoma	CNS	4.60E-27	6.16E-01	5.48E-11	7.89E-01
3473	FEAST_A02_51640	TCGA.GBM	Glioblastoma	CNS	9.65E-25	5.88E-01	1.30E-11	8.12E-01

3474	FEAST_A03_51639	TCGA.GBM	Glioblastoma	CNS	6.13E-24	5.78E-01	1.15E-11	8.14E-01
3475	FEAST_A04_51638	TCGA.GBM	Glioblastoma	CNS	3.10E-21	5.44E-01	5.48E-11	7.89E-01
3476	FEAST_A05_51641	TCGA.GBM	Glioblastoma	CNS	4.56E-25	5.92E-01	2.03E-11	8.05E-01
3477	FEAST_A06_51628	TCGA.GBM	Glioblastoma	CNS	1.55E-21	5.48E-01	3.35E-10	7.58E-01
3478	FEAST_A07_51646	TCGA.GBM	Glioblastoma	CNS	1.08E-19	5.23E-01	2.12E-09	7.25E-01
3479	FEAST_A08_51646	TCGA.GBM	Glioblastoma	CNS	2.03E-24	5.84E-01	8.42E-11	7.82E-01
3480	FEAST_A09_51638	TCGA.GBM	Glioblastoma	CNS	7.75E-23	5.64E-01	3.55E-11	7.96E-01
3481	FEAST_A10_51647	TCGA.GBM	Glioblastoma	CNS	4.73E-19	5.14E-01	1.29E-10	7.74E-01
3482	FEAST_A11_51634	TCGA.GBM	Glioblastoma	CNS	9.46E-32	6.69E-01	6.16E-11	7.87E-01
3483	FEAST_A12_51637	TCGA.GBM	Glioblastoma	CNS	1.27E-23	5.74E-01	2.63E-12	8.38E-01
3484	FEAST_B01_51643	TCGA.GBM	Glioblastoma	CNS	1.83E-23	5.72E-01	3.55E-11	7.96E-01
3485	FEAST_B02_51636	TCGA.GBM	Glioblastoma	CNS	3.68E-21	5.43E-01	7.38E-12	8.22E-01
3486	FEAST_B03_51636	TCGA.GBM	Glioblastoma	CNS	2.44E-24	5.83E-01	9.51E-10	7.40E-01
3487	FEAST_B04_51639	TCGA.GBM	Glioblastoma	CNS	2.16E-17	4.90E-01	8.51E-10	7.42E-01
3488	FEAST_B05_51639	TCGA.GBM	Glioblastoma	CNS	1.71E-20	5.34E-01	3.51E-09	7.16E-01
3489	FEAST_B06_51646	TCGA.GBM	Glioblastoma	CNS	5.10E-24	5.79E-01	1.15E-11	8.14E-01
3490	FEAST_B07_51631	TCGA.GBM	Glioblastoma	CNS	5.26E-18	4.99E-01	1.45E-10	7.72E-01
3491	FEAST_B08_51642	TCGA.GBM	Glioblastoma	CNS	2.56E-27	6.19E-01	2.03E-11	8.05E-01
3492	FEAST_B09_51635	TCGA.GBM	Glioblastoma	CNS	2.94E-17	4.88E-01	5.18E-09	7.09E-01
3493	FEAST_B10_51640	TCGA.GBM	Glioblastoma	CNS	1.01E-25	6.00E-01	2.12E-09	7.25E-01
3494	FEAST_B11_51629	TCGA.GBM	Glioblastoma	CNS	1.22E-26	6.11E-01	5.48E-11	7.89E-01
3495	FEAST_B12_51644	TCGA.GBM	Glioblastoma	CNS	2.10E-27	6.20E-01	2.03E-11	8.05E-01
3496	FEAST_C01_51641	TCGA.GBM	Glioblastoma	CNS	6.80E-27	6.14E-01	1.15E-11	8.14E-01
3497	FEAST_C02_51639	TCGA.GBM	Glioblastoma	CNS	1.47E-25	5.98E-01	2.63E-12	8.38E-01
3498	FEAST_C03_51642	TCGA.GBM	Glioblastoma	CNS	2.64E-26	6.07E-01	4.15E-12	8.31E-01
3499	FEAST_C04_51632	TCGA.GBM	Glioblastoma	CNS	7.75E-31	6.59E-01	4.69E-12	8.29E-01
3500	FEAST_C05_51638	TCGA.GBM	Glioblastoma	CNS	1.16E-27	6.23E-01	7.38E-12	8.22E-01
3501	FEAST_C06_51633	TCGA.GBM	Glioblastoma	CNS	4.25E-24	5.80E-01	2.03E-11	8.05E-01
3502	FEAST_C07_51642	TCGA.GBM	Glioblastoma	CNS	2.02E-20	5.33E-01	5.06E-10	7.51E-01
3503	FEAST_C08_51646	TCGA.GBM	Glioblastoma	CNS	8.84E-24	5.76E-01	5.48E-11	7.89E-01
3504	FEAST_C09_51640	TCGA.GBM	Glioblastoma	CNS	1.22E-26	6.11E-01	3.55E-11	7.96E-01
3505	FEAST_C10_51632	TCGA.GBM	Glioblastoma	CNS	6.55E-20	5.26E-01	1.29E-10	7.74E-01
3506	FEAST_C11_51634	TCGA.GBM	Glioblastoma	CNS	1.44E-20	5.35E-01	1.42E-09	7.32E-01
3507	FEAST_C12_51631	TCGA.GBM	Glioblastoma	CNS	5.54E-20	5.27E-01	1.80E-11	8.07E-01
3508	FEAST_D01_51645	TCGA.GBM	Glioblastoma	CNS	1.53E-23	5.73E-01	3.75E-10	7.56E-01
3509	FEAST_D02_51643	TCGA.GBM	Glioblastoma	CNS	8.84E-24	5.76E-01	4.69E-12	8.29E-01
3510	FEAST_D03_51643	TCGA.GBM	Glioblastoma	CNS	6.48E-23	5.65E-01	2.03E-11	8.05E-01
3511	FEAST_D04_51629	TCGA.GBM	Glioblastoma	CNS	3.21E-22	5.57E-01	5.66E-10	7.49E-01
3512	FEAST_D05_51631	TCGA.GBM	Glioblastoma	CNS	1.55E-21	5.48E-01	8.42E-11	7.82E-01
3513	FEAST_D06_51644	TCGA.GBM	Glioblastoma	CNS	1.08E-19	5.23E-01	2.03E-11	8.05E-01
3514	FEAST_D07_51646	TCGA.GBM	Glioblastoma	CNS	1.16E-24	5.87E-01	2.21E-10	7.65E-01
3515	FEAST_D08_51633	TCGA.GBM	Glioblastoma	CNS	2.09E-19	5.19E-01	8.51E-10	7.42E-01
3516	FEAST_D09_51644	TCGA.GBM	Glioblastoma	CNS	1.89E-22	5.60E-01	4.87E-11	7.91E-01
3517	FEAST_D10_51638	TCGA.GBM	Glioblastoma	CNS	9.65E-25	5.88E-01	4.87E-11	7.91E-01
3518	FEAST_D11_51629	TCGA.GBM	Glioblastoma	CNS	4.69E-20	5.28E-01	5.66E-10	7.49E-01
3519	NIDUS_A01_40038	TCGA.GBM	Glioblastoma	CNS	4.52E-23	5.67E-01	2.03E-11	8.05E-01
3520	NIDUS_A02_40037	TCGA.GBM	Glioblastoma	CNS	5.54E-20	5.27E-01	5.48E-11	7.89E-01
3521	NIDUS_A03_40038	TCGA.GBM	Glioblastoma	CNS	3.78E-25	5.93E-01	8.42E-11	7.82E-01
3522	NIDUS_A04_40037	TCGA.GBM	Glioblastoma	CNS	6.64E-25	5.90E-01	4.69E-12	8.29E-01
3523	NIDUS_A05_40037	TCGA.GBM	Glioblastoma	CNS	1.11E-22	5.63E-01	2.03E-11	8.05E-01
3524	NIDUS_A06_40037	TCGA.GBM	Glioblastoma	CNS	9.65E-25	5.88E-01	2.03E-11	8.05E-01
3525	NIDUS_A07_40037	TCGA.GBM	Glioblastoma	CNS	4.73E-19	5.14E-01	5.76E-09	7.07E-01
3526	NIDUS_A08_40041	TCGA.GBM	Glioblastoma	CNS	4.69E-20	5.28E-01	1.28E-09	7.34E-01
3527	NIDUS_A09_40041	TCGA.GBM	Glioblastoma	CNS	3.83E-22	5.56E-01	1.28E-09	7.34E-01
3528	NIDUS_A10_40041	TCGA.GBM	Glioblastoma	CNS	6.13E-24	5.78E-01	1.45E-10	7.72E-01
3529	NIDUS_A11_40040	TCGA.GBM	Glioblastoma	CNS	2.09E-19	5.19E-01	5.66E-10	7.49E-01
3530	NIDUS_A12_40040	TCGA.GBM	Glioblastoma	CNS	7.65E-32	6.70E-01	4.69E-12	8.29E-01
3531	NIDUS_B01_40040	TCGA.GBM	Glioblastoma	CNS	7.75E-23	5.64E-01	3.15E-11	7.98E-01
3532	NIDUS_B02_40040	TCGA.GBM	Glioblastoma	CNS	3.78E-25	5.93E-01	2.63E-12	8.38E-01
3533	NIDUS_B03_40040	TCGA.GBM	Glioblastoma	CNS	1.78E-25	5.97E-01	3.35E-10	7.58E-01
3534	NIDUS_B04_40039	TCGA.GBM	Glioblastoma	CNS	7.30E-21	5.39E-01	1.15E-11	8.14E-01
3535	NIDUS_B05_40039	TCGA.GBM	Glioblastoma	CNS	4.37E-21	5.42E-01	1.29E-10	7.74E-01
3536	NIDUS_B06_40039	TCGA.GBM	Glioblastoma	CNS	2.20E-23	5.71E-01	1.29E-10	7.74E-01
3537	NIDUS_B07_40039	TCGA.GBM	Glioblastoma	CNS	2.64E-26	6.07E-01	2.63E-12	8.38E-01
3538	NIDUS_B10_40038	TCGA.GBM	Glioblastoma	CNS	2.44E-24	5.83E-01	5.48E-11	7.89E-01
3539	NIDUS_B11_40038	TCGA.GBM	Glioblastoma	CNS	8.66E-21	5.38E-01	5.66E-10	7.49E-01
3540	NIDUS_C01_40044	TCGA.GBM	Glioblastoma	CNS	2.19E-21	5.46E-01	3.51E-09	7.16E-01
3541	NIDUS_C02_40044	TCGA.GBM	Glioblastoma	CNS	1.58E-22	5.61E-01	8.51E-10	7.42E-01
3542	NIDUS_C04_40043	TCGA.GBM	Glioblastoma	CNS	3.53E-24	5.81E-01	1.28E-09	7.34E-01
3543	NIDUS_C05_40043	TCGA.GBM	Glioblastoma	CNS	2.03E-24	5.84E-01	2.03E-11	8.05E-01
3544	NIDUS_C06_40043	TCGA.GBM	Glioblastoma	CNS	2.15E-25	5.96E-01	4.69E-12	8.29E-01
3545	SOCKS_E01_5882	TCGA.GBM	Glioblastoma	CNS	2.44E-24	5.83E-01	2.21E-10	7.65E-01
3546	SOCKS_E02_5882	TCGA.GBM	Glioblastoma	CNS	8.66E-21	5.38E-01	4.69E-12	8.29E-01
3547	SOCKS_E03_5882	TCGA.GBM	Glioblastoma	CNS	1.06E-28	6.35E-01	1.45E-10	7.72E-01

3548	SOCKS_E04_58823	TCGA.GBM	Glioblastoma	CNS	4.60E-27	6.16E-01	5.72E-13	8.62E-01
3549	SOCKS_E05_58821	TCGA.GBM	Glioblastoma	CNS	5.68E-26	6.03E-01	5.18E-09	7.09E-01
3550	SOCKS_E06_58820	TCGA.GBM	Glioblastoma	CNS	2.19E-21	5.46E-01	3.55E-11	7.96E-01
3551	SOCKS_E07_58821	TCGA.GBM	Glioblastoma	CNS	4.57E-22	5.55E-01	8.42E-11	7.82E-01
3552	SOCKS_E08_58821	TCGA.GBM	Glioblastoma	CNS	1.84E-21	5.47E-01	5.48E-11	7.89E-01
3553	SOCKS_E09_58821	TCGA.GBM	Glioblastoma	CNS	2.19E-21	5.46E-01	5.66E-10	7.49E-01
3554	SOCKS_E10_58821	TCGA.GBM	Glioblastoma	CNS	2.16E-17	4.90E-01	2.21E-10	7.65E-01
3555	SOCKS_E11_58822	TCGA.GBM	Glioblastoma	CNS	3.35E-20	5.30E-01	2.21E-10	7.65E-01
3556	SOCKS_E12_58822	TCGA.GBM	Glioblastoma	CNS	4.25E-24	5.80E-01	2.47E-10	7.63E-01
3557	SOCKS_F01_58825	TCGA.GBM	Glioblastoma	CNS	1.53E-23	5.73E-01	2.03E-11	8.05E-01
3558	SOCKS_F02_58825	TCGA.GBM	Glioblastoma	CNS	6.16E-21	5.40E-01	9.51E-10	7.40E-01
3559	SOCKS_F03_58826	TCGA.GBM	Glioblastoma	CNS	8.32E-26	6.01E-01	2.21E-10	7.65E-01
3560	SOCKS_F04_58826	TCGA.GBM	Glioblastoma	CNS	1.32E-22	5.62E-01	1.42E-09	7.32E-01
3561	SOCKS_F05_58826	TCGA.GBM	Glioblastoma	CNS	6.48E-23	5.65E-01	1.15E-11	8.14E-01
3562	SOCKS_F06_58826	TCGA.GBM	Glioblastoma	CNS	1.22E-25	5.99E-01	8.42E-11	7.82E-01
3563	SOCKS_F07_58826	TCGA.GBM	Glioblastoma	CNS	9.65E-25	5.88E-01	2.03E-11	8.05E-01
3564	SOCKS_F08_58827	TCGA.GBM	Glioblastoma	CNS	5.41E-23	5.66E-01	8.42E-11	7.82E-01
3565	SOCKS_F09_58824	TCGA.GBM	Glioblastoma	CNS	4.25E-24	5.80E-01	3.35E-10	7.58E-01
3566	SOCKS_F10_58824	TCGA.GBM	Glioblastoma	CNS	6.16E-21	5.40E-01	5.48E-11	7.89E-01
3567	SOCKS_F11_58824	TCGA.GBM	Glioblastoma	CNS	1.10E-21	5.50E-01	2.21E-10	7.65E-01
3568	SOCKS_G01_58824	TCGA.GBM	Glioblastoma	CNS	1.30E-21	5.49E-01	9.46E-11	7.80E-01
3569	SOCKS_G02_58825	TCGA.GBM	Glioblastoma	CNS	3.68E-21	5.43E-01	2.21E-10	7.65E-01
3570	SOCKS_G03_58825	TCGA.GBM	Glioblastoma	CNS	1.00E-26	6.12E-01	6.43E-14	8.94E-01
3571	SOCKS_G04_58825	TCGA.GBM	Glioblastoma	CNS	1.89E-22	5.60E-01	4.69E-12	8.29E-01
3572	SOCKS_G05_58819	TCGA.GBM	Glioblastoma	CNS	1.03E-20	5.37E-01	4.15E-12	8.31E-01
3573	SOCKS_G06_58819	TCGA.GBM	Glioblastoma	CNS	3.21E-22	5.57E-01	2.03E-11	8.05E-01
3574	GSM478151	GSE19274	Neuroblastoma	CNS	5.16E-14	4.39E-01	5.11E-07	6.18E-01
3575	GSM478152	GSE19274	Neuroblastoma	CNS	1.75E-18	5.08E-01	1.50E-07	6.43E-01
3576	GSM478153	GSE19274	Neuroblastoma	CNS	1.67E-17	4.94E-01	9.40E-09	6.98E-01
3577	GSM478154	GSE19274	Neuroblastoma	CNS	3.07E-19	5.19E-01	4.20E-10	7.54E-01
3578	GSM478155	GSE19274	Neuroblastoma	CNS	1.05E-04	2.41E-01	8.86E-05	4.96E-01
3579	GSM478156	GSE19274	Neuroblastoma	CNS	1.03E-16	4.82E-01	1.24E-08	6.92E-01
3580	GSM478157	GSE19274	Neuroblastoma	CNS	1.03E-07	3.19E-01	4.30E-06	5.70E-01
3581	GSM478158	GSE19274	Neuroblastoma	CNS	1.98E-17	4.93E-01	5.76E-09	7.07E-01
3582	GSM478159	GSE19274	Neuroblastoma	CNS	6.91E-13	4.20E-01	2.22E-08	6.81E-01
3583	GSM478160	GSE19274	Neuroblastoma	CNS	1.72E-21	5.49E-01	5.66E-10	7.49E-01
3584	GSM478161	GSE19274	Neuroblastoma	CNS	7.93E-18	4.99E-01	2.63E-09	7.21E-01
3585	GSM478162	GSE19274	Neuroblastoma	CNS	1.31E-20	5.38E-01	2.36E-09	7.23E-01
3586	GSM478163	GSE19274	Neuroblastoma	CNS	1.75E-12	4.13E-01	1.65E-07	6.41E-01
3587	GSM478164	GSE19274	Neuroblastoma	CNS	6.13E-16	4.70E-01	1.82E-07	6.39E-01
3588	GSM478165	GSE19274	Neuroblastoma	CNS	9.89E-19	5.12E-01	9.40E-09	6.98E-01
3589	GSM478166	GSE19274	Neuroblastoma	CNS	1.63E-13	4.31E-01	2.34E-07	6.34E-01
3590	GSM478167	GSE19274	Neuroblastoma	CNS	4.91E-15	4.56E-01	9.40E-09	6.98E-01
3591	GSM478168	GSE19274	Neuroblastoma	CNS	3.09E-19	5.19E-01	1.24E-08	6.92E-01
3592	GSM478169	GSE19274	Neuroblastoma	CNS	5.30E-27	6.18E-01	2.63E-12	8.38E-01
3593	GSM478170	GSE19274	Neuroblastoma	CNS	2.93E-17	4.90E-01	3.21E-08	6.74E-01
3594	GSM478171	GSE19274	Neuroblastoma	CNS	4.27E-13	4.24E-01	2.28E-06	5.85E-01
3595	GSM478172	GSE19274	Neuroblastoma	CNS	4.34E-12	4.06E-01	5.12E-08	6.65E-01
3596	GSM478173	GSE19274	Neuroblastoma	CNS	1.93E-20	5.35E-01	1.37E-08	6.90E-01
3597	GSM478174	GSE19274	Neuroblastoma	CNS	1.16E-14	4.50E-01	5.12E-08	6.65E-01
3598	GSM478175	GSE19274	Neuroblastoma	CNS	6.84E-13	4.20E-01	6.93E-06	5.59E-01
3599	GSM478176	GSE19274	Neuroblastoma	CNS	3.43E-17	4.89E-01	6.40E-09	7.05E-01
3600	GSM478177	GSE19274	Neuroblastoma	CNS	1.05E-16	4.82E-01	5.76E-09	7.07E-01
3601	GSM478178	GSE19274	Neuroblastoma	CNS	6.76E-13	4.21E-01	7.15E-07	6.10E-01
3602	GSM478179	GSE19274	Neuroblastoma	CNS	1.75E-12	4.13E-01	8.57E-07	6.07E-01
3603	GSM478180	GSE19274	Neuroblastoma	CNS	9.78E-11	3.81E-01	1.51E-06	5.94E-01
3604	GSM478181	GSE19274	Neuroblastoma	CNS	2.10E-16	4.77E-01	9.40E-09	6.98E-01
3605	GSM478182	GSE19274	Neuroblastoma	CNS	1.74E-19	5.22E-01	1.59E-09	7.30E-01
3606	GSM478183	GSE19274	Neuroblastoma	CNS	2.08E-21	5.48E-01	6.67E-08	6.60E-01
3607	GSM478184	GSE19274	Neuroblastoma	CNS	1.78E-15	4.63E-01	6.67E-08	6.60E-01
3608	GSM478185	GSE19274	Neuroblastoma	CNS	2.61E-13	4.28E-01	1.09E-06	6.01E-01
3609	GSM478186	GSE19274	Neuroblastoma	CNS	5.80E-09	3.46E-01	3.42E-06	5.76E-01
3610	GSM478187	GSE19274	Neuroblastoma	CNS	2.64E-10	3.73E-01	1.51E-06	5.94E-01
3611	GSM478188	GSE19274	Neuroblastoma	CNS	2.11E-16	4.77E-01	1.59E-09	7.30E-01
3612	GSM478189	GSE19274	Neuroblastoma	CNS	5.79E-15	4.55E-01	2.22E-08	6.81E-01
3613	GSM478190	GSE19274	Neuroblastoma	CNS	5.07E-12	4.05E-01	3.99E-07	6.23E-01
3614	GSM478191	GSE19274	Neuroblastoma	CNS	2.26E-13	4.29E-01	7.15E-07	6.10E-01
3615	GSM478192	GSE19274	Neuroblastoma	CNS	7.17E-17	4.84E-01	6.40E-09	7.05E-01
3616	GSM478193	GSE19274	Neuroblastoma	CNS	1.37E-17	4.95E-01	1.37E-08	6.90E-01
3617	GSM478194	GSE19274	Neuroblastoma	CNS	4.14E-17	4.88E-01	3.01E-07	6.29E-01
3618	GSM478195	GSE19274	Neuroblastoma	CNS	2.22E-13	4.29E-01	2.28E-06	5.85E-01
3619	GSM478196	GSE19274	Neuroblastoma	CNS	6.21E-16	4.70E-01	1.24E-08	6.92E-01
3620	GSM478197	GSE19274	Neuroblastoma	CNS	1.17E-17	4.96E-01	2.12E-09	7.25E-01
3621	GSM478198	GSE19274	Neuroblastoma	CNS	1.03E-16	4.82E-01	5.66E-10	7.49E-01

3622	GSM478199	GSE19274	Neuroblastoma	CNS	3.11E-16	4.75E-01	6.67E-08	6.60E-01
3623	GSM478200	GSE19274	Neuroblastoma	CNS	3.88E-08	3.28E-01	1.16E-04	4.89E-01
3624	GSM478201	GSE19274	Neuroblastoma	CNS	4.46E-14	4.41E-01	2.00E-08	6.83E-01
3625	GSM478202	GSE19274	Neuroblastoma	CNS	5.99E-21	5.42E-01	1.45E-10	7.72E-01
3626	GSM478203	GSE19274	Neuroblastoma	CNS	7.96E-12	4.02E-01	2.22E-08	6.81E-01
3627	GSM478204	GSE19274	Neuroblastoma	CNS	1.48E-16	4.80E-01	5.76E-09	7.07E-01
3628	GSM478205	GSE19274	Neuroblastoma	CNS	3.62E-20	5.32E-01	3.15E-11	7.98E-01
3629	GSM478206	GSE19274	Neuroblastoma	CNS	2.96E-15	4.60E-01	3.55E-08	6.72E-01
3630	GSM478207	GSE19274	Neuroblastoma	CNS	5.97E-10	3.66E-01	1.19E-06	5.99E-01
3631	GSM478208	GSE19274	Neuroblastoma	CNS	3.70E-07	3.06E-01	1.60E-03	4.12E-01
3632	GSM478209	GSE19274	Neuroblastoma	CNS	1.56E-09	3.58E-01	1.71E-05	5.38E-01
3633	GSM478210	GSE19274	Neuroblastoma	CNS	7.32E-11	3.84E-01	6.38E-06	5.61E-01
3634	GSM478211	GSE19274	Neuroblastoma	CNS	2.60E-18	5.06E-01	2.12E-09	7.25E-01
3635	GSM478212	GSE19274	Neuroblastoma	CNS	2.65E-11	3.92E-01	1.85E-05	5.36E-01
3636	GSM478213	GSE19274	Neuroblastoma	CNS	1.59E-08	3.37E-01	1.02E-05	5.50E-01
3637	GSM478214	GSE19274	Neuroblastoma	CNS	2.45E-15	4.61E-01	8.46E-09	7.00E-01
3638	GSM478215	GSE19274	Neuroblastoma	CNS	1.30E-25	6.01E-01	4.20E-10	7.54E-01
3639	GSM478216	GSE19274	Neuroblastoma	CNS	5.06E-17	4.87E-01	1.52E-08	6.89E-01
3640	GSM478217	GSE19274	Neuroblastoma	CNS	3.11E-19	5.19E-01	1.36E-07	6.45E-01
3641	GSM478218	GSE19274	Neuroblastoma	CNS	2.96E-15	4.60E-01	2.29E-05	5.30E-01
3642	GSM478219	GSE19274	Neuroblastoma	CNS	1.46E-18	5.09E-01	9.51E-10	7.40E-01
3643	GSM478220	GSE19274	Neuroblastoma	CNS	1.19E-19	5.24E-01	3.55E-08	6.72E-01
3644	GSM478221	GSE19274	Neuroblastoma	CNS	4.91E-15	4.56E-01	2.22E-08	6.81E-01
3645	GSM478222	GSE19274	Neuroblastoma	CNS	4.14E-15	4.57E-01	2.63E-09	7.21E-01
3646	GSM478223	GSE19274	Neuroblastoma	CNS	4.89E-22	5.57E-01	6.33E-10	7.47E-01
3647	GSM478224	GSE19274	Neuroblastoma	CNS	3.47E-15	4.58E-01	1.16E-07	6.49E-01
3648	GSM478225	GSE19274	Neuroblastoma	CNS	7.44E-23	5.67E-01	1.62E-10	7.70E-01
3649	GSM478226	GSE19274	Neuroblastoma	CNS	1.39E-17	4.95E-01	1.06E-09	7.38E-01
3650	GSM478227	GSE19274	Neuroblastoma	CNS	3.47E-15	4.58E-01	7.36E-08	6.58E-01
3651	GSM478228	GSE19274	Neuroblastoma	CNS	4.20E-15	4.57E-01	1.50E-07	6.43E-01
3652	GSM478229	GSE19274	Neuroblastoma	CNS	2.57E-21	5.47E-01	3.90E-09	7.14E-01
3653	GSM478230	GSE19274	Neuroblastoma	CNS	1.13E-22	5.65E-01	2.47E-10	7.63E-01
3654	GSM478231	GSE19274	Neuroblastoma	CNS	2.67E-14	4.44E-01	1.65E-07	6.41E-01
3655	GSM478232	GSE19274	Neuroblastoma	CNS	2.40E-17	4.92E-01	2.63E-09	7.21E-01
3656	GSM478233	GSE19274	Neuroblastoma	CNS	6.89E-15	4.54E-01	4.20E-10	7.54E-01
3657	GSM478234	GSE19274	Neuroblastoma	CNS	2.54E-16	4.76E-01	2.63E-09	7.21E-01
3658	GSM478235	GSE19274	Neuroblastoma	CNS	9.55E-18	4.97E-01	7.36E-08	6.58E-01
3659	GSM478236	GSE19274	Neuroblastoma	CNS	3.09E-13	4.26E-01	6.53E-07	6.12E-01
3660	GSM478237	GSE19274	Neuroblastoma	CNS	2.54E-19	5.20E-01	9.51E-10	7.40E-01
3661	GSM478238	GSE19274	Neuroblastoma	CNS	1.23E-16	4.81E-01	1.65E-07	6.41E-01
3662	GSM478239	GSE19274	Neuroblastoma	CNS	1.07E-12	4.17E-01	2.58E-07	6.32E-01
3663	GSM478240	GSE19274	Neuroblastoma	CNS	1.37E-13	4.32E-01	5.60E-07	6.16E-01
3664	GSM478241	GSE19274	Neuroblastoma	CNS	1.37E-13	4.32E-01	2.34E-07	6.34E-01
3665	GSM478242	GSE19274	Neuroblastoma	CNS	1.24E-16	4.81E-01	4.64E-08	6.67E-01
3666	GSM478243	GSE19274	Neuroblastoma	CNS	3.22E-21	5.46E-01	3.75E-10	7.56E-01
3667	GSM478244	GSE19274	Neuroblastoma	CNS	4.52E-10	3.69E-01	7.83E-07	6.09E-01
3668	GSM478245	GSE19274	Neuroblastoma	CNS	1.30E-20	5.38E-01	1.37E-08	6.90E-01
3669	GSM478246	GSE19274	Neuroblastoma	CNS	1.13E-17	4.96E-01	2.12E-09	7.25E-01
3670	GSM478247	GSE19274	Neuroblastoma	CNS	6.49E-20	5.28E-01	1.62E-10	7.70E-01
3671	GSM478248	GSE19274	Neuroblastoma	CNS	1.94E-11	3.95E-01	7.83E-07	6.09E-01
3672	GSM478249	GSE19274	Neuroblastoma	CNS	2.37E-12	4.11E-01	6.38E-06	5.61E-01
3673	GSM478250	GSE19274	Neuroblastoma	CNS	8.04E-18	4.98E-01	2.63E-09	7.21E-01
3674	GSM72395	GSE3218	Germ Cell Tumour	Germ Cell	2.76E-09	3.51E-01	4.02E-04	4.54E-01
3675	GSM72396	GSE3218	Germ Cell Tumour	Germ Cell	7.44E-09	3.42E-01	7.97E-06	5.56E-01
3676	GSM72397	GSE3218	Germ Cell Tumour	Germ Cell	2.06E-13	4.28E-01	2.13E-07	6.36E-01
3677	GSM72398	GSE3218	Germ Cell Tumour	Germ Cell	5.79E-03	1.80E-01	3.82E-03	3.83E-01
3678	GSM72399	GSE3218	Germ Cell Tumour	Germ Cell	1.58E-17	4.92E-01	2.22E-08	6.81E-01
3679	GSM72400	GSE3218	Germ Cell Tumour	Germ Cell	2.79E-18	5.03E-01	3.62E-06	5.74E-01
3680	GSM72401	GSE3218	Germ Cell Tumour	Germ Cell	1.00E-01	-1.20E-01	2.96E-01	1.79E-01
3681	GSM72402	GSE3218	Germ Cell Tumour	Germ Cell	4.79E-02	-1.38E-01	1.51E-01	-2.23E-01
3682	GSM72403	GSE3218	Germ Cell Tumour	Germ Cell	1.00E-01	1.20E-01	6.43E-03	3.65E-01
3683	GSM72404	GSE3218	Germ Cell Tumour	Germ Cell	1.17E-12	4.15E-01	8.46E-09	7.00E-01
3684	GSM72405	GSE3218	Germ Cell Tumour	Germ Cell	2.23E-05	2.59E-01	5.15E-04	4.47E-01
3685	GSM72406	GSE3218	Germ Cell Tumour	Germ Cell	7.13E-12	4.01E-01	9.40E-06	5.52E-01
3686	GSM72407	GSE3218	Germ Cell Tumour	Germ Cell	2.77E-02	-1.50E-01	2.61E-01	1.88E-01
3687	GSM72408	GSE3218	Germ Cell Tumour	Germ Cell	2.35E-01	9.52E-02	2.18E-02	3.17E-01
3688	GSM72409	GSE3218	Germ Cell Tumour	Germ Cell	7.07E-16	4.67E-01	1.51E-06	5.94E-01
3689	GSM72410	GSE3218	Germ Cell Tumour	Germ Cell	8.61E-07	2.96E-01	6.74E-05	5.03E-01
3690	GSM72411	GSE3218	Germ Cell Tumour	Germ Cell	1.00E-01	1.20E-01	5.17E-02	2.79E-01
3691	GSM72412	GSE3218	Germ Cell Tumour	Germ Cell	4.09E-15	4.55E-01	1.16E-07	6.49E-01
3692	GSM72413	GSE3218	Germ Cell Tumour	Germ Cell	7.96E-05	2.43E-01	6.43E-03	3.65E-01
3693	GSM72414	GSE3218	Germ Cell Tumour	Germ Cell	8.57E-02	1.24E-01	8.14E-02	2.57E-01
3694	GSM72415	GSE3218	Germ Cell Tumour	Germ Cell	1.24E-07	3.15E-01	8.65E-06	5.54E-01
3695	GSM72416	GSE3218	Germ Cell Tumour	Germ Cell	8.56E-03	-1.73E-01	4.90E-01	-1.37E-01

3696	GSM72417	GSE3218	Germ Cell Tumour	Germ Cell	4.69E-20	5.28E-01	3.75E-10	7.56E-01
3697	GSM72418	GSE3218	Germ Cell Tumour	Germ Cell	1.84E-16	4.76E-01	3.51E-09	7.16E-01
3698	GSM72419	GSE3218	Germ Cell Tumour	Germ Cell	3.35E-20	5.30E-01	2.36E-09	7.23E-01
3699	GSM72420	GSE3218	Germ Cell Tumour	Germ Cell	1.63E-03	2.00E-01	1.69E-02	3.28E-01
3700	GSM72421	GSE3218	Germ Cell Tumour	Germ Cell	1.83E-23	5.72E-01	2.29E-11	8.03E-01
3701	GSM72422	GSE3218	Germ Cell Tumour	Germ Cell	8.97E-13	4.17E-01	1.05E-07	6.50E-01
3702	GSM72423	GSE3218	Germ Cell Tumour	Germ Cell	1.57E-38	7.38E-01	6.66E-15	9.27E-01
3703	GSM72424	GSE3218	Germ Cell Tumour	Germ Cell	1.95E-11	3.93E-01	1.27E-05	5.45E-01
3704	GSM72425	GSE3218	Germ Cell Tumour	Germ Cell	6.21E-01	-5.46E-02	1.94E-01	2.08E-01
3705	GSM72426	GSE3218	Germ Cell Tumour	Germ Cell	1.96E-14	4.44E-01	1.65E-07	6.41E-01
3706	GSM72427	GSE3218	Germ Cell Tumour	Germ Cell	3.63E-03	1.88E-01	6.79E-03	3.63E-01
3707	GSM72428	GSE3218	Germ Cell Tumour	Germ Cell	7.60E-02	1.27E-01	8.03E-02	2.58E-01
3708	GSM72429	GSE3218	Germ Cell Tumour	Germ Cell	9.73E-04	2.08E-01	6.14E-04	4.41E-01
3709	GSM72430	GSE3218	Germ Cell Tumour	Germ Cell	1.21E-01	1.15E-01	3.77E-02	2.94E-01
3710	GSM72431	GSE3218	Germ Cell Tumour	Germ Cell	5.16E-03	1.82E-01	5.39E-02	2.77E-01
3711	GSM72432	GSE3218	Germ Cell Tumour	Germ Cell	8.61E-07	2.96E-01	8.89E-04	4.30E-01
3712	GSM72433	GSE3218	Germ Cell Tumour	Germ Cell	4.01E-02	-1.42E-01	1.82E-01	-2.12E-01
3713	GSM72434	GSE3218	Germ Cell Tumour	Germ Cell	1.97E-10	3.74E-01	1.71E-05	5.38E-01
3714	GSM72435	GSE3218	Germ Cell Tumour	Germ Cell	1.22E-41	7.68E-01	6.35E-16	9.60E-01
3715	GSM72436	GSE3218	Germ Cell Tumour	Germ Cell	3.47E-01	8.13E-02	3.27E-03	3.88E-01
3716	GSM72437	GSE3218	Germ Cell Tumour	Germ Cell	3.09E-09	3.50E-01	8.89E-04	4.30E-01
3717	GSM72438	GSE3218	Germ Cell Tumour	Germ Cell	3.38E-01	8.23E-02	2.42E-01	1.93E-01
3718	GSM72439	GSE3218	Germ Cell Tumour	Germ Cell	1.97E-03	1.97E-01	1.88E-03	4.07E-01
3719	GSM72440	GSE3218	Germ Cell Tumour	Germ Cell	1.44E-20	5.35E-01	2.63E-12	8.38E-01
3720	GSM72441	GSE3218	Germ Cell Tumour	Germ Cell	6.64E-01	5.06E-02	1.94E-01	2.08E-01
3721	GSM72442	GSE3218	Germ Cell Tumour	Germ Cell	4.25E-05	2.51E-01	1.71E-04	4.78E-01
3722	GSM72443	GSE3218	Germ Cell Tumour	Germ Cell	1.34E-11	-3.96E-01	1.33E-03	-4.18E-01
3723	GSM72444	GSE3218	Germ Cell Tumour	Germ Cell	9.67E-15	4.49E-01	4.64E-08	6.67E-01
3724	GSM72445	GSE3218	Germ Cell Tumour	Germ Cell	4.43E-01	-7.14E-02	2.76E-01	1.84E-01
3725	GSM72446	GSE3218	Germ Cell Tumour	Germ Cell	2.69E-03	1.92E-01	2.35E-03	3.99E-01
3726	GSM72447	GSE3218	Germ Cell Tumour	Germ Cell	2.80E-01	8.93E-02	8.14E-02	2.57E-01
3727	GSM72448	GSE3218	Germ Cell Tumour	Germ Cell	1.42E-34	6.98E-01	1.10E-14	9.20E-01
3728	GSM72449	GSE3218	Germ Cell Tumour	Germ Cell	2.72E-01	-9.03E-02	7.05E-02	2.64E-01
3729	GSM72450	GSE3218	Germ Cell Tumour	Germ Cell	2.49E-01	9.33E-02	9.35E-02	2.50E-01
3730	GSM72451	GSE3218	Germ Cell Tumour	Germ Cell	2.21E-09	3.53E-01	2.00E-05	5.34E-01
3731	GSM72452	GSE3218	Germ Cell Tumour	Germ Cell	1.57E-13	4.30E-01	3.01E-07	6.29E-01
3732	GSM72453	GSE3218	Germ Cell Tumour	Germ Cell	4.72E-15	4.54E-01	3.63E-07	6.25E-01
3733	GSM72454	GSE3218	Germ Cell Tumour	Germ Cell	9.27E-05	2.41E-01	3.51E-04	4.58E-01
3734	GSM72455	GSE3218	Germ Cell Tumour	Germ Cell	7.84E-07	2.97E-01	4.73E-05	5.12E-01
3735	GSM72456	GSE3218	Germ Cell Tumour	Germ Cell	5.37E-07	3.01E-01	1.16E-04	4.89E-01
3736	GSM72457	GSE3218	Germ Cell Tumour	Germ Cell	9.54E-03	1.71E-01	6.09E-03	3.67E-01
3737	GSM72458	GSE3218	Germ Cell Tumour	Germ Cell	1.26E-03	2.04E-01	1.98E-02	3.21E-01
3738	GSM72459	GSE3218	Germ Cell Tumour	Germ Cell	4.98E-08	3.24E-01	8.65E-06	5.54E-01
3739	GSM72460	GSE3218	Germ Cell Tumour	Germ Cell	2.83E-11	3.90E-01	7.15E-07	6.10E-01
3740	GSM72461	GSE3218	Germ Cell Tumour	Germ Cell	2.96E-01	-8.73E-02	1.88E-01	2.10E-01
3741	GSM72462	GSE3218	Germ Cell Tumour	Germ Cell	3.32E-07	3.06E-01	2.11E-05	5.32E-01
3742	GSM72463	GSE3218	Germ Cell Tumour	Germ Cell	2.91E-12	4.08E-01	2.88E-06	5.80E-01
3743	GSM72464	GSE3218	Germ Cell Tumour	Germ Cell	1.14E-05	2.67E-01	1.71E-05	5.38E-01
3744	GSM72465	GSE3218	Germ Cell Tumour	Germ Cell	1.35E-04	2.36E-01	1.27E-05	5.45E-01
3745	GSM72466	GSE3218	Germ Cell Tumour	Germ Cell	1.75E-10	3.75E-01	3.57E-05	5.19E-01
3746	GSM72467	GSE3218	Germ Cell Tumour	Germ Cell	2.44E-04	2.28E-01	4.02E-04	4.54E-01
3747	GSM72468	GSE3218	Germ Cell Tumour	Germ Cell	3.94E-01	7.64E-02	1.76E-01	2.14E-01
3748	GSM72469	GSE3218	Germ Cell Tumour	Germ Cell	1.60E-05	2.63E-01	3.13E-04	4.61E-01
3749	GSM72470	GSE3218	Germ Cell Tumour	Germ Cell	2.57E-01	-9.23E-02	1.78E-01	2.13E-01
3750	GSM72471	GSE3218	Germ Cell Tumour	Germ Cell	6.43E-28	6.26E-01	6.33E-10	7.47E-01
3751	GSM72472	GSE3218	Germ Cell Tumour	Germ Cell	1.04E-11	3.98E-01	8.65E-06	5.54E-01
3752	GSM72473	GSE3218	Germ Cell Tumour	Germ Cell	1.16E-04	2.38E-01	1.75E-04	4.77E-01
3753	GSM72474	GSE3218	Germ Cell Tumour	Germ Cell	1.83E-01	-1.03E-01	1.07E-01	2.43E-01
3754	GSM72475	GSE3218	Germ Cell Tumour	Germ Cell	1.08E-01	1.18E-01	1.27E-02	3.39E-01
3755	GSM72476	GSE3218	Germ Cell Tumour	Germ Cell	5.52E-08	3.23E-01	4.68E-06	5.69E-01
3756	GSM72477	GSE3218	Germ Cell Tumour	Germ Cell	2.57E-01	-9.23E-02	1.43E-01	2.26E-01
3757	GSM72478	GSE3218	Germ Cell Tumour	Germ Cell	4.95E-04	2.18E-01	2.02E-02	3.21E-01
3758	GSM72479	GSE3218	Germ Cell Tumour	Germ Cell	1.11E-22	5.63E-01	9.46E-11	7.80E-01
3759	GSM72480	GSE3218	Germ Cell Tumour	Germ Cell	4.88E-07	3.02E-01	2.73E-04	4.65E-01
3760	GSM72481	GSE3218	Germ Cell Tumour	Germ Cell	6.77E-08	3.21E-01	2.43E-04	4.68E-01
3761	GSM72482	GSE3218	Germ Cell Tumour	Germ Cell	1.70E-02	1.60E-01	2.49E-03	3.97E-01
3762	GSM72483	GSE3218	Germ Cell Tumour	Germ Cell	3.20E-11	3.89E-01	2.88E-06	5.80E-01
3763	GSM72484	GSE3218	Germ Cell Tumour	Germ Cell	1.46E-02	1.63E-01	1.38E-02	3.36E-01
3764	GSM72485	GSE3218	Germ Cell Tumour	Germ Cell	5.23E-02	1.36E-01	9.51E-03	3.50E-01
3765	GSM72486	GSE3218	Germ Cell Tumour	Germ Cell	5.36E-09	3.45E-01	6.74E-05	5.03E-01
3766	GSM72487	GSE3218	Germ Cell Tumour	Germ Cell	6.13E-24	5.78E-01	1.15E-11	8.14E-01
3767	GSM72488	GSE3218	Germ Cell Tumour	Germ Cell	5.46E-15	4.53E-01	3.01E-07	6.29E-01
3768	GSM72489	GSE3218	Germ Cell Tumour	Germ Cell	3.62E-11	3.88E-01	8.65E-06	5.54E-01
3769	GSM72490	GSE3218	Germ Cell Tumour	Germ Cell	7.50E-08	3.20E-01	1.67E-03	4.10E-01

3770	GSM72491	GSE3218	Germ Cell Tumour	Germ Cell	9.88E-18	4.95E-01	9.51E-10	7.40E-01
3771	GSM72492	GSE3218	Germ Cell Tumour	Germ Cell	1.18E-02	1.67E-01	4.12E-02	2.90E-01
3772	GSM72493	GSE3218	Germ Cell Tumour	Germ Cell	1.25E-18	5.08E-01	3.55E-11	7.96E-01
3773	GSM72494	GSE3218	Germ Cell Tumour	Germ Cell	9.23E-09	3.40E-01	4.68E-06	5.69E-01
3774	GSM498450	GSE19949	Renal	Kidney	5.78E-01	5.85E-02	1.53E-02	3.32E-01
3775	GSM498451	GSE19949	Renal	Kidney	1.27E-08	3.37E-01	2.58E-07	6.32E-01
3776	GSM498452	GSE19949	Renal	Kidney	8.57E-02	1.24E-01	1.98E-02	3.21E-01
3777	GSM498453	GSE19949	Renal	Kidney	6.28E-12	4.02E-01	1.09E-06	6.01E-01
3778	GSM498454	GSE19949	Renal	Kidney	1.97E-02	1.57E-01	2.26E-01	1.98E-01
3779	GSM498455	GSE19949	Renal	Kidney	4.83E-01	6.75E-02	6.26E-02	2.70E-01
3780	GSM498456	GSE19949	Renal	Kidney	3.62E-05	2.53E-01	6.25E-05	5.05E-01
3781	GSM498457	GSE19949	Renal	Kidney	4.62E-13	4.22E-01	8.51E-10	7.42E-01
3782	GSM498458	GSE19949	Renal	Kidney	2.14E-01	-9.82E-02	1.66E-02	3.29E-01
3783	GSM498459	GSE19949	Renal	Kidney	4.88E-07	3.02E-01	2.48E-05	5.29E-01
3784	GSM498460	GSE19949	Renal	Kidney	3.29E-08	3.28E-01	3.57E-05	5.19E-01
3785	GSM498461	GSE19949	Renal	Kidney	1.74E-03	1.99E-01	4.04E-03	3.81E-01
3786	GSM498462	GSE19949	Renal	Kidney	3.62E-05	2.53E-01	2.11E-04	4.72E-01
3787	GSM498463	GSE19949	Renal	Kidney	4.98E-08	3.24E-01	1.16E-04	4.89E-01
3788	GSM498464	GSE19949	Renal	Kidney	4.03E-07	3.04E-01	9.40E-06	5.52E-01
3789	GSM498465	GSE19949	Renal	Kidney	2.11E-04	2.30E-01	3.30E-05	5.21E-01
3790	GSM498466	GSE19949	Renal	Kidney	5.91E-11	3.84E-01	2.49E-06	5.83E-01
3791	GSM498467	GSE19949	Renal	Kidney	1.08E-01	1.18E-01	1.43E-01	2.26E-01
3792	GSM498468	GSE19949	Renal	Kidney	6.00E-01	5.65E-02	8.14E-02	2.57E-01
3793	GSM498469	GSE19949	Renal	Kidney	2.14E-01	-9.82E-02	2.86E-01	1.82E-01
3794	GSM498470	GSE19949	Renal	Kidney	3.47E-01	8.13E-02	7.53E-02	2.61E-01
3795	GSM498471	GSE19949	Renal	Kidney	3.56E-01	8.04E-02	7.24E-02	2.63E-01
3796	GSM498472	GSE19949	Renal	Kidney	1.96E-04	2.31E-01	1.12E-03	4.23E-01
3797	GSM498473	GSE19949	Renal	Kidney	1.67E-07	3.13E-01	2.29E-05	5.30E-01
3798	GSM498474	GSE19949	Renal	Kidney	1.08E-19	5.23E-01	1.62E-10	7.70E-01
3799	GSM498475	GSE19949	Renal	Kidney	3.62E-05	2.53E-01	2.21E-03	4.01E-01
3800	GSM498476	GSE19949	Renal	Kidney	2.21E-11	3.92E-01	9.51E-10	7.40E-01
3801	GSM498477	GSE19949	Renal	Kidney	1.47E-05	2.64E-01	2.49E-03	3.97E-01
3802	GSM498478	GSE19949	Renal	Kidney	4.05E-08	3.26E-01	1.71E-05	5.38E-01
3803	GSM498479	GSE19949	Renal	Kidney	4.62E-04	2.19E-01	4.82E-04	4.49E-01
3804	GSM498480	GSE19949	Renal	Kidney	1.35E-03	2.03E-01	3.09E-03	3.90E-01
3805	GSM498481	GSE19949	Renal	Kidney	2.53E-03	-1.93E-01	2.88E-01	-1.81E-01
3806	GSM498482	GSE19949	Renal	Kidney	3.23E-03	1.89E-01	5.25E-03	3.72E-01
3807	GSM498483	GSE19949	Renal	Kidney	5.23E-14	4.38E-01	4.20E-08	6.69E-01
3808	GSM498484	GSE19949	Renal	Kidney	1.27E-08	3.37E-01	3.95E-06	5.72E-01
3809	GSM498485	GSE19949	Renal	Kidney	7.97E-04	2.11E-01	3.76E-04	4.56E-01
3810	GSM498486	GSE19949	Renal	Kidney	3.31E-12	4.07E-01	9.97E-07	6.03E-01
3811	GSM498487	GSE19949	Renal	Kidney	1.67E-07	3.13E-01	6.25E-05	5.05E-01
3812	GSM498488	GSE19949	Renal	Kidney	3.93E-05	2.52E-01	2.00E-03	4.05E-01
3813	GSM498489	GSE19949	Renal	Kidney	1.06E-02	1.69E-01	2.91E-03	3.92E-01
3814	GSM498490	GSE19949	Renal	Kidney	1.47E-05	2.64E-01	3.76E-04	4.56E-01
3815	GSM498491	GSE19949	Renal	Kidney	7.36E-05	2.44E-01	6.25E-05	5.05E-01
3816	GSM498492	GSE19949	Renal	Kidney	1.04E-03	2.07E-01	8.86E-05	4.96E-01
3817	GSM498493	GSE19949	Renal	Kidney	4.88E-07	3.02E-01	3.30E-05	5.21E-01
3818	GSM498494	GSE19949	Renal	Kidney	8.56E-03	1.73E-01	4.04E-03	3.81E-01
3819	GSM498495	GSE19949	Renal	Kidney	1.12E-07	3.16E-01	8.65E-06	5.54E-01
3820	GSM498496	GSE19949	Renal	Kidney	2.97E-08	3.29E-01	1.71E-05	5.38E-01
3821	GSM498497	GSE19949	Renal	Kidney	1.16E-01	1.16E-01	1.53E-02	3.32E-01
3822	GSM498498	GSE19949	Renal	Kidney	1.60E-05	2.63E-01	8.65E-06	5.54E-01
3823	GSM498499	GSE19949	Renal	Kidney	2.62E-04	2.27E-01	1.12E-03	4.23E-01
3824	GSM498500	GSE19949	Renal	Kidney	3.62E-05	2.53E-01	4.73E-05	5.12E-01
3825	GSM498501	GSE19949	Renal	Kidney	9.73E-04	2.08E-01	7.01E-04	4.37E-01
3826	GSM498502	GSE19949	Renal	Kidney	1.84E-16	4.76E-01	5.18E-09	7.09E-01
3827	GSM498503	GSE19949	Renal	Kidney	2.69E-03	1.92E-01	3.76E-04	4.56E-01
3828	GSM498504	GSE19949	Renal	Kidney	1.00E-01	1.20E-01	5.25E-03	3.72E-01
3829	GSM498505	GSE19949	Renal	Kidney	3.75E-01	7.84E-02	1.07E-01	2.43E-01
3830	GSM498506	GSE19949	Renal	Kidney	6.81E-05	2.45E-01	9.54E-05	4.94E-01
3831	GSM498507	GSE19949	Renal	Kidney	1.37E-07	3.14E-01	5.80E-05	5.07E-01
3832	GSM498508	GSE19949	Renal	Kidney	9.11E-04	2.09E-01	1.12E-03	4.23E-01
3833	GSM498509	GSE19949	Renal	Kidney	4.01E-02	-1.42E-01	5.85E-02	2.73E-01
3834	GSM498510	GSE19949	Renal	Kidney	1.24E-05	2.66E-01	2.26E-04	4.70E-01
3835	GSM498511	GSE19949	Renal	Kidney	2.44E-04	2.28E-01	4.97E-03	3.74E-01
3836	GSM498512	GSE19949	Renal	Kidney	3.09E-13	4.25E-01	5.18E-09	7.09E-01
3837	GSM498513	GSE19949	Renal	Kidney	2.05E-05	2.60E-01	9.47E-04	4.28E-01
3838	GSM498514	GSE19949	Renal	Kidney	4.62E-04	2.19E-01	7.01E-04	4.37E-01
3839	GSM498515	GSE19949	Renal	Kidney	7.96E-05	2.43E-01	8.23E-05	4.98E-01
3840	GSM498516	GSE19949	Renal	Kidney	3.23E-03	1.89E-01	2.92E-04	4.63E-01
3841	GSM498517	GSE19949	Renal	Kidney	2.27E-04	-2.29E-01	1.26E-01	-2.33E-01
3842	GSM498518	GSE19949	Renal	Kidney	2.47E-09	3.52E-01	4.30E-06	5.70E-01
3843	GSM498519	GSE19949	Renal	Kidney	7.14E-07	2.98E-01	3.86E-05	5.17E-01

3844	GSM498520	GSE19949	Renal	Kidney	3.86E-03	1.87E-01	1.05E-03	4.25E-01
3845	GSM498521	GSE19949	Renal	Kidney	7.25E-03	1.76E-01	3.09E-03	3.90E-01
3846	GSM498522	GSE19949	Renal	Kidney	1.26E-09	3.58E-01	3.63E-07	6.25E-01
3847	GSM498523	GSE19949	Renal	Kidney	9.19E-12	3.99E-01	2.49E-06	5.83E-01
3848	GSM498524	GSE19949	Renal	Kidney	4.31E-04	2.20E-01	2.35E-03	3.99E-01
3849	GSM498525	GSE19949	Renal	Kidney	6.12E-08	3.22E-01	6.38E-06	5.61E-01
3850	GSM498526	GSE19949	Renal	Kidney	1.12E-09	3.59E-01	2.28E-06	5.85E-01
3851	GSM498527	GSE19949	Renal	Kidney	2.84E-05	2.56E-01	6.25E-05	5.05E-01
3852	GSM498528	GSE19949	Renal	Kidney	3.84E-02	1.43E-01	4.04E-03	3.81E-01
3853	GSM498529	GSE19949	Renal	Kidney	3.49E-04	-2.23E-01	4.96E-02	-2.81E-01
3854	GSM498530	GSE19949	Renal	Kidney	2.77E-02	-1.50E-01	1.67E-01	2.17E-01
3855	GSM498531	GSE19949	Renal	Kidney	6.86E-03	-1.77E-01	4.34E-01	-1.48E-01
3856	GSM498532	GSE19949	Renal	Kidney	1.61E-02	-1.61E-01	9.35E-02	2.50E-01
3857	GSM498533	GSE19949	Renal	Kidney	2.96E-01	8.73E-02	8.26E-03	3.55E-01
3858	GSM498534	GSE19949	Renal	Kidney	8.10E-03	-1.74E-01	4.15E-01	1.52E-01
3859	GSM498535	GSE19949	Renal	Kidney	1.18E-02	-1.67E-01	4.44E-01	1.46E-01
3860	GSM498536	GSE19949	Renal	Kidney	1.08E-01	-1.18E-01	9.35E-02	2.50E-01
3861	GSM498537	GSE19949	Renal	Kidney	5.94E-02	-1.33E-01	1.67E-01	2.17E-01
3862	GSM498538	GSE19949	Renal	Kidney	7.96E-05	-2.43E-01	4.25E-01	-1.50E-01
3863	GSM498539	GSE19949	Renal	Kidney	5.68E-04	2.16E-01	6.74E-05	5.03E-01
3864	GSM498540	GSE19949	Renal	Kidney	2.01E-01	1.00E-01	4.04E-03	3.81E-01
3865	GSM498541	GSE19949	Renal	Kidney	2.10E-03	-1.96E-01	4.80E-01	1.39E-01
3866	GSM498542	GSE19949	Renal	Kidney	8.52E-04	-2.10E-01	3.75E-01	-1.61E-01
3867	GSM498543	GSE19949	Renal	Kidney	4.59E-02	-1.39E-01	1.88E-01	2.10E-01
3868	GSM498544	GSE19949	Renal	Kidney	4.62E-04	-2.19E-01	5.10E-01	1.33E-01
3869	GSM498545	GSE19949	Renal	Kidney	3.49E-04	-2.23E-01	3.75E-01	-1.61E-01
3870	GSM498546	GSE19949	Renal	Kidney	1.31E-02	1.65E-01	1.06E-02	3.46E-01
3871	GSM498547	GSE19949	Renal	Kidney	1.39E-01	1.11E-01	6.43E-03	3.65E-01
3872	GSM498548	GSE19949	Renal	Kidney	1.18E-03	2.05E-01	2.00E-05	5.34E-01
3873	GSM498549	GSE19949	Renal	Kidney	1.26E-04	2.37E-01	1.38E-05	5.43E-01
3874	GSM498550	GSE19949	Renal	Kidney	6.18E-32	6.71E-01	1.05E-13	8.87E-01
3875	GSM498551	GSE19949	Renal	Kidney	1.24E-07	3.15E-01	2.11E-04	4.72E-01
3876	GSM498552	GSE19949	Renal	Kidney	4.44E-07	3.03E-01	3.57E-05	5.19E-01
3877	GSM498553	GSE19949	Renal	Kidney	9.67E-15	4.49E-01	2.12E-09	7.25E-01
3878	GSM498554	GSE19949	Renal	Kidney	2.37E-06	2.85E-01	3.86E-05	5.17E-01
3879	GSM498555	GSE19949	Renal	Kidney	5.75E-06	2.75E-01	2.48E-05	5.29E-01
3880	GSM498556	GSE19949	Renal	Kidney	4.81E-09	3.46E-01	1.16E-07	6.49E-01
3881	GSM498557	GSE19949	Renal	Kidney	4.87E-12	4.04E-01	1.65E-07	6.41E-01
3882	GSM498558	GSE19949	Renal	Kidney	1.44E-20	5.35E-01	2.21E-10	7.65E-01
3883	GSM498559	GSE19949	Renal	Kidney	1.58E-17	4.92E-01	1.42E-09	7.32E-01
3884	GSM498560	GSE19949	Renal	Kidney	1.25E-18	5.08E-01	7.62E-09	7.02E-01
3885	GSM498561	GSE19949	Renal	Kidney	1.27E-08	3.37E-01	2.00E-05	5.34E-01
3886	GSM498562	GSE19949	Renal	Kidney	5.19E-21	5.41E-01	8.51E-10	7.42E-01
3887	GSM904986	GSE36895	Renal	Kidney	3.32E-07	3.06E-01	1.88E-03	4.07E-01
3888	GSM904989	GSE36895	Renal	Kidney	3.10E-06	2.82E-01	1.51E-04	4.81E-01
3889	GSM904991	GSE36895	Renal	Kidney	1.04E-06	2.94E-01	7.64E-05	5.00E-01
3890	GSM904994	GSE36895	Renal	Kidney	1.24E-07	3.15E-01	1.27E-05	5.45E-01
3891	GSM904996	GSE36895	Renal	Kidney	2.41E-08	3.31E-01	1.16E-04	4.89E-01
3892	GSM904999	GSE36895	Renal	Kidney	4.60E-05	2.50E-01	2.49E-03	3.97E-01
3893	GSM905002	GSE36895	Renal	Kidney	2.21E-11	3.92E-01	1.17E-05	5.47E-01
3894	GSM905004	GSE36895	Renal	Kidney	8.24E-02	1.25E-01	1.92E-02	3.23E-01
3895	GSM905007	GSE36895	Renal	Kidney	1.37E-07	3.14E-01	4.30E-06	5.70E-01
3896	GSM905009	GSE36895	Renal	Kidney	1.46E-04	2.35E-01	8.86E-05	4.96E-01
3897	GSM905012	GSE36895	Renal	Kidney	4.25E-05	2.51E-01	6.56E-04	4.39E-01
3898	GSM905014	GSE36895	Renal	Kidney	1.52E-07	3.13E-01	4.30E-06	5.70E-01
3899	GSM905017	GSE36895	Renal	Kidney	5.27E-06	2.76E-01	8.23E-05	4.98E-01
3900	GSM905020	GSE36895	Renal	Kidney	1.82E-04	2.32E-01	1.88E-03	4.07E-01
3901	GSM905022	GSE36895	Renal	Kidney	8.30E-08	3.19E-01	4.38E-05	5.14E-01
3902	GSM905023	GSE36895	Renal	Kidney	4.31E-09	3.47E-01	1.09E-06	6.01E-01
3903	GSM905024	GSE36895	Renal	Kidney	1.52E-12	4.13E-01	2.13E-07	6.36E-01
3904	GSM905026	GSE36895	Renal	Kidney	3.09E-09	3.50E-01	1.09E-06	6.01E-01
3905	GSM905027	GSE36895	Renal	Kidney	1.37E-06	2.91E-01	4.38E-05	5.14E-01
3906	GSM905029	GSE36895	Renal	Kidney	9.18E-08	3.18E-01	6.56E-04	4.39E-01
3907	GSM905031	GSE36895	Renal	Kidney	9.54E-03	1.71E-01	1.09E-02	3.45E-01
3908	GSM905032	GSE36895	Renal	Kidney	7.21E-18	4.97E-01	1.59E-09	7.30E-01
3909	GSM905034	GSE36895	Renal	Kidney	9.46E-07	2.95E-01	3.57E-05	5.19E-01
3910	GSM905036	GSE36895	Renal	Kidney	1.97E-10	3.74E-01	1.09E-06	6.01E-01
3911	GSM905038	GSE36895	Renal	Kidney	4.42E-06	2.78E-01	6.56E-04	4.39E-01
3912	GSM905040	GSE36895	Renal	Kidney	1.36E-16	4.78E-01	9.51E-10	7.40E-01
3913	GSM905041	GSE36895	Renal	Kidney	1.06E-18	5.09E-01	6.67E-08	6.60E-01
3914	GSM905043	GSE36895	Renal	Kidney	4.55E-14	4.38E-01	1.50E-07	6.43E-01
3915	GSM905044	GSE36895	Renal	Kidney	6.30E-05	2.46E-01	7.43E-03	3.59E-01
3916	GSM655521	GSE26574	Renal	Kidney	3.15E-10	3.70E-01	1.58E-05	5.40E-01
3917	GSM655522	GSE26574	Renal	Kidney	3.96E-14	4.39E-01	3.31E-07	6.27E-01

3918	GSM655523	GSE26574	Renal	Kidney	1.95E-08	3.33E-01	1.08E-04	4.90E-01
3919	GSM655524	GSE26574	Renal	Kidney	6.28E-06	2.74E-01	2.21E-03	4.01E-01
3920	GSM655525	GSE26574	Renal	Kidney	5.28E-13	4.21E-01	2.13E-07	6.36E-01
3921	GSM655526	GSE26574	Renal	Kidney	3.27E-18	5.02E-01	1.12E-08	6.94E-01
3922	GSM655527	GSE26574	Renal	Kidney	1.98E-15	4.60E-01	6.04E-08	6.62E-01
3923	GSM655528	GSE26574	Renal	Kidney	9.65E-06	2.69E-01	8.86E-05	4.96E-01
3924	GSM655529	GSE26574	Renal	Kidney	2.88E-01	8.83E-02	4.96E-02	2.81E-01
3925	GSM655530	GSE26574	Renal	Kidney	1.71E-01	-1.05E-01	1.73E-01	2.15E-01
3926	GSM655531	GSE26574	Renal	Kidney	1.89E-01	1.02E-01	2.84E-02	3.06E-01
3927	GSM655532	GSE26574	Renal	Kidney	9.63E-02	1.21E-01	7.53E-02	2.61E-01
3928	GSM655533	GSE26574	Renal	Kidney	1.44E-01	-1.10E-01	3.04E-01	1.77E-01
3929	GSM655534	GSE26574	Renal	Kidney	5.46E-02	1.35E-01	7.83E-03	3.57E-01
3930	GSM655535	GSE26574	Renal	Kidney	3.86E-03	1.87E-01	2.92E-04	4.63E-01
3931	GSM655536	GSE26574	Renal	Kidney	1.79E-02	-1.59E-01	4.18E-01	1.51E-01
3932	GSM655537	GSE26574	Renal	Kidney	1.12E-01	-1.17E-01	4.73E-01	1.40E-01
3933	GSM655538	GSE26574	Renal	Kidney	4.63E-01	-6.94E-02	1.31E-01	2.31E-01
3934	GSM655539	GSE26574	Renal	Kidney	3.56E-01	-8.04E-02	7.83E-02	2.59E-01
3935	GSM655540	GSE26574	Renal	Kidney	2.08E-01	-9.92E-02	3.35E-02	2.99E-01
3936	GSM655541	GSE26574	Renal	Kidney	8.56E-03	-1.73E-01	3.04E-01	1.77E-01
3937	GSM655542	GSE26574	Renal	Kidney	1.44E-01	1.10E-01	2.29E-02	3.15E-01
3938	GSM655543	GSE26574	Renal	Kidney	1.34E-01	1.12E-01	1.38E-02	3.36E-01
3939	GSM655544	GSE26574	Renal	Kidney	5.79E-03	1.80E-01	4.12E-02	2.90E-01
3940	GSM655545	GSE26574	Renal	Kidney	2.11E-04	2.30E-01	1.60E-03	4.12E-01
3941	GSM655546	GSE26574	Renal	Kidney	8.24E-02	1.25E-01	6.09E-03	3.67E-01
3942	GSM655547	GSE26574	Renal	Kidney	5.98E-09	3.44E-01	1.38E-05	5.43E-01
3943	GSM655548	GSE26574	Renal	Kidney	1.60E-01	1.07E-01	1.83E-02	3.25E-01
3944	GSM655549	GSE26574	Renal	Kidney	1.11E-03	2.06E-01	6.25E-05	5.05E-01
3945	GSM655550	GSE26574	Renal	Kidney	2.49E-16	4.74E-01	6.67E-08	6.60E-01
3946	GSM655551	GSE26574	Renal	Kidney	4.83E-01	6.75E-02	2.18E-02	3.17E-01
3947	GSM655552	GSE26574	Renal	Kidney	2.35E-01	9.52E-02	5.76E-03	3.69E-01
3948	GSM655553	GSE26574	Renal	Kidney	7.01E-02	1.29E-01	2.91E-03	3.92E-01
3949	GSM655554	GSE26574	Renal	Kidney	2.14E-01	9.82E-02	9.02E-03	3.52E-01
3950	GSM655555	GSE26574	Renal	Kidney	3.04E-01	8.63E-02	4.62E-02	2.85E-01
3951	GSM655556	GSE26574	Renal	Kidney	2.52E-02	1.52E-01	5.76E-03	3.69E-01
3952	GSM655557	GSE26574	Renal	Kidney	4.79E-02	1.38E-01	4.44E-03	3.78E-01
3953	GSM655558	GSE26574	Renal	Kidney	7.01E-02	1.29E-01	1.04E-02	3.47E-01
3954	GSM655559	GSE26574	Renal	Kidney	3.84E-01	7.74E-02	1.67E-01	2.17E-01
3955	GSM655560	GSE26574	Renal	Kidney	1.88E-02	1.58E-01	1.67E-03	4.10E-01
3956	GSM655561	GSE26574	Renal	Kidney	9.65E-06	2.69E-01	4.02E-04	4.54E-01
3957	GSM655562	GSE26574	Renal	Kidney	3.93E-05	2.52E-01	6.25E-05	5.05E-01
3958	GSM655563	GSE26574	Renal	Kidney	4.60E-03	1.84E-01	3.60E-03	3.85E-01
3959	GSM655564	GSE26574	Renal	Kidney	3.06E-15	4.57E-01	2.13E-07	6.36E-01
3960	GSM655565	GSE26574	Renal	Kidney	1.58E-09	3.56E-01	1.09E-06	6.01E-01
3961	GSM655566	GSE26574	Renal	Kidney	2.73E-07	3.08E-01	6.38E-06	5.61E-01
3962	GSM655567	GSE26574	Renal	Kidney	1.58E-09	3.56E-01	4.68E-06	5.69E-01
3963	GSM655568	GSE26574	Renal	Kidney	8.57E-02	-1.24E-01	6.78E-02	2.66E-01
3964	GSM655569	GSE26574	Renal	Kidney	2.52E-17	4.89E-01	1.29E-10	7.74E-01
3965	GSM655570	GSE26574	Renal	Kidney	2.27E-04	2.29E-01	2.54E-04	4.67E-01
3966	GSM655571	GSE26574	Renal	Kidney	1.00E-04	2.40E-01	8.89E-04	4.30E-01
3967	GSM655572	GSE26574	Renal	Kidney	2.23E-05	2.59E-01	1.51E-04	4.81E-01
3968	GSM655573	GSE26574	Renal	Kidney	3.45E-09	3.49E-01	3.14E-06	5.78E-01
3969	GSM655574	GSE26574	Renal	Kidney	3.63E-03	1.88E-01	1.75E-04	4.77E-01
3970	GSM655575	GSE26574	Renal	Kidney	4.98E-08	3.24E-01	3.42E-06	5.76E-01
3971	GSM655576	GSE26574	Renal	Kidney	6.68E-11	3.83E-01	1.37E-08	6.90E-01
3972	GSM655577	GSE26574	Renal	Kidney	2.28E-01	-9.62E-02	3.94E-02	2.92E-01
3973	GSM559577	GSE22541	Renal	Kidney	3.93E-05	2.52E-01	2.92E-04	4.63E-01
3974	GSM559578	GSE22541	Renal	Kidney	1.21E-01	1.15E-01	3.21E-02	3.01E-01
3975	GSM559579	GSE22541	Renal	Kidney	3.63E-03	1.88E-01	1.74E-02	3.27E-01
3976	GSM559580	GSE22541	Renal	Kidney	6.97E-04	2.13E-01	6.74E-05	5.03E-01
3977	GSM559581	GSE22541	Renal	Kidney	1.88E-02	1.58E-01	7.16E-03	3.61E-01
3978	GSM559582	GSE22541	Renal	Kidney	7.54E-11	3.82E-01	9.56E-08	6.52E-01
3979	GSM559583	GSE22541	Renal	Kidney	3.50E-02	-1.45E-01	2.28E-01	1.97E-01
3980	GSM559584	GSE22541	Renal	Kidney	3.65E-08	3.27E-01	1.63E-04	4.79E-01
3981	GSM559585	GSE22541	Renal	Kidney	1.12E-02	1.68E-01	2.71E-02	3.08E-01
3982	GSM559586	GSE22541	Renal	Kidney	3.65E-08	3.27E-01	4.73E-05	5.12E-01
3983	GSM559587	GSE22541	Renal	Kidney	3.84E-01	-7.74E-02	9.94E-02	2.47E-01
3984	GSM559588	GSE22541	Renal	Kidney	1.16E-01	1.16E-01	7.43E-03	3.59E-01
3985	GSM559589	GSE22541	Renal	Kidney	3.67E-02	-1.44E-01	2.66E-01	1.87E-01
3986	GSM559590	GSE22541	Renal	Kidney	3.86E-09	3.48E-01	9.40E-06	5.52E-01
3987	GSM559591	GSE22541	Renal	Kidney	3.13E-01	8.53E-02	1.03E-01	2.45E-01
3988	GSM559592	GSE22541	Renal	Kidney	7.91E-14	4.35E-01	7.36E-08	6.58E-01
3989	GSM559593	GSE22541	Renal	Kidney	6.77E-08	3.21E-01	1.85E-05	5.36E-01
3990	GSM559594	GSE22541	Renal	Kidney	6.73E-02	1.30E-01	1.53E-02	3.32E-01
3991	GSM559595	GSE22541	Renal	Kidney	2.08E-01	9.92E-02	3.51E-02	2.97E-01

3992	GSM559596	GSE22541	Renal	Kidney	1.96E-04	2.31E-01	8.89E-04	4.30E-01
3993	GSM559597	GSE22541	Renal	Kidney	6.51E-04	2.14E-01	7.83E-03	3.57E-01
3994	GSM559598	GSE22541	Renal	Kidney	2.14E-16	4.75E-01	2.03E-11	8.05E-01
3995	GSM559599	GSE22541	Renal	Kidney	2.37E-28	6.31E-01	1.45E-10	7.72E-01
3996	GSM559600	GSE22541	Renal	Kidney	1.81E-06	2.88E-01	8.23E-05	4.98E-01
3997	GSM579349	GSE23629	Renal	Kidney	1.53E-02	1.62E-01	6.78E-02	2.66E-01
3998	GSM579351	GSE23629	Renal	Kidney	3.50E-02	1.45E-01	3.94E-02	2.92E-01
3999	GSM579353	GSE23629	Renal	Kidney	1.52E-07	3.13E-01	1.75E-04	4.77E-01
4000	GSM579355	GSE23629	Renal	Kidney	9.27E-05	2.41E-01	8.65E-06	5.54E-01
4001	GSM579357	GSE23629	Renal	Kidney	2.42E-01	-9.42E-02	8.35E-02	2.56E-01
4002	GSM579359	GSE23629	Renal	Kidney	3.34E-05	2.54E-01	4.44E-03	3.78E-01
4003	GSM579361	GSE23629	Renal	Kidney	6.08E-04	-2.15E-01	3.60E-01	-1.64E-01
4004	GSM579363	GSE23629	Renal	Kidney	5.89E-01	5.75E-02	6.79E-03	3.63E-01
4005	GSM579365	GSE23629	Renal	Kidney	2.80E-10	3.71E-01	5.39E-06	5.65E-01
4006	GSM579367	GSE23629	Renal	Kidney	3.25E-04	2.24E-01	4.82E-04	4.49E-01
4007	GSM579369	GSE23629	Renal	Kidney	4.44E-07	3.03E-01	3.76E-04	4.56E-01
4008	GSM579371	GSE23629	Renal	Kidney	3.21E-01	-8.43E-02	5.61E-02	2.75E-01
4009	GSM579373	GSE23629	Renal	Kidney	1.18E-03	2.05E-01	7.09E-05	5.02E-01
4010	GSM579375	GSE23629	Renal	Kidney	1.11E-14	4.48E-01	5.11E-07	6.18E-01
4011	GSM579377	GSE23629	Renal	Kidney	8.29E-09	3.41E-01	1.16E-07	6.49E-01
4012	GSM579379	GSE23629	Renal	Kidney	2.29E-02	-1.54E-01	3.35E-02	2.99E-01
4013	GSM146779	GSE6344	Renal	Kidney	1.00E-04	2.40E-01	1.16E-04	4.89E-01
4014	GSM146781	GSE6344	Renal	Kidney	6.51E-04	2.14E-01	6.56E-04	4.39E-01
4015	GSM146783	GSE6344	Renal	Kidney	2.79E-18	5.03E-01	4.64E-08	6.67E-01
4016	GSM146785	GSE6344	Renal	Kidney	3.65E-08	3.27E-01	2.11E-05	5.32E-01
4017	GSM146787	GSE6344	Renal	Kidney	5.91E-07	3.00E-01	1.12E-03	4.23E-01
4018	GSM146788	GSE6344	Renal	Kidney	9.46E-07	2.95E-01	5.80E-05	5.07E-01
4019	GSM146791	GSE6344	Renal	Kidney	8.50E-11	3.81E-01	7.97E-06	5.56E-01
4020	GSM146793	GSE6344	Renal	Kidney	4.62E-13	4.22E-01	5.86E-06	5.63E-01
4021	GSM146795	GSE6344	Renal	Kidney	1.41E-09	3.57E-01	6.25E-05	5.05E-01
4022	GSM146797	GSE6344	Renal	Kidney	3.84E-02	1.43E-01	1.50E-02	3.32E-01
4023	GSM11814	GSE781	Renal	Kidney	7.97E-04	2.11E-01	1.00E-02	3.48E-01
4024	GSM11830	GSE781	Renal	Kidney	3.42E-03	1.88E-01	2.18E-02	3.17E-01
4025	GSM12067	GSE781	Renal	Kidney	1.26E-03	2.04E-01	6.56E-04	4.39E-01
4026	GSM12079	GSE781	Renal	Kidney	6.16E-18	4.98E-01	2.21E-10	7.65E-01
4027	GSM12100	GSE781	Renal	Kidney	1.18E-02	1.67E-01	3.60E-03	3.85E-01
4028	GSM12105	GSE781	Renal	Kidney	3.10E-21	5.44E-01	1.42E-09	7.32E-01
4029	GSM12270	GSE781	Renal	Kidney	3.06E-33	6.85E-01	1.15E-11	8.14E-01
4030	GSM12283	GSE781	Renal	Kidney	1.44E-01	-1.10E-01	5.39E-02	2.77E-01
4031	GSM12298	GSE781	Renal	Kidney	1.60E-01	-1.07E-01	5.61E-02	2.75E-01
4032	GSM12300	GSE781	Renal	Kidney	7.30E-02	1.28E-01	7.83E-03	3.57E-01
4033	GSM12399	GSE781	Renal	Kidney	1.04E-06	2.94E-01	6.25E-05	5.05E-01
4034	GSM12444	GSE781	Renal	Kidney	1.44E-03	2.02E-01	4.02E-04	4.54E-01
4035	GSM362958	GSE14520	Liver	Liver	2.49E-01	9.33E-02	2.64E-03	3.95E-01
4036	GSM362959	GSE14520	Liver	Liver	7.45E-04	2.12E-01	1.25E-04	4.87E-01
4037	GSM362960	GSE14520	Liver	Liver	8.91E-02	1.23E-01	8.89E-04	4.30E-01
4038	GSM362964	GSE14520	Liver	Liver	4.09E-03	1.86E-01	8.57E-07	6.07E-01
4039	GSM362965	GSE14520	Liver	Liver	5.75E-06	2.75E-01	2.34E-07	6.34E-01
4040	GSM362966	GSE14520	Liver	Liver	4.87E-03	-1.83E-01	3.94E-02	2.92E-01
4041	GSM362970	GSE14520	Liver	Liver	2.11E-04	2.30E-01	3.14E-06	5.78E-01
4042	GSM362971	GSE14520	Liver	Liver	1.18E-03	2.05E-01	2.49E-06	5.83E-01
4043	GSM362972	GSE14520	Liver	Liver	7.97E-04	2.11E-01	2.49E-06	5.83E-01
4044	GSM362976	GSE14520	Liver	Liver	4.02E-04	2.21E-01	1.81E-06	5.90E-01
4045	GSM362977	GSE14520	Liver	Liver	1.54E-01	-1.08E-01	9.47E-04	4.28E-01
4046	GSM362978	GSE14520	Liver	Liver	1.08E-01	1.18E-01	8.89E-04	4.30E-01
4047	GSM362982	GSE14520	Liver	Liver	1.79E-02	-1.59E-01	1.60E-03	4.12E-01
4048	GSM362983	GSE14520	Liver	Liver	3.56E-01	-8.04E-02	4.04E-03	3.81E-01
4049	GSM362984	GSE14520	Liver	Liver	1.97E-03	1.97E-01	1.71E-05	5.38E-01
4050	GSM362986	GSE14520	Liver	Liver	2.69E-03	1.92E-01	1.51E-04	4.81E-01
4051	GSM362987	GSE14520	Liver	Liver	1.65E-01	1.06E-01	2.26E-04	4.70E-01
4052	GSM362988	GSE14520	Liver	Liver	1.21E-01	-1.15E-01	6.09E-03	3.67E-01
4053	GSM362992	GSE14520	Liver	Liver	3.19E-02	-1.47E-01	1.00E-02	3.48E-01
4054	GSM362993	GSE14520	Liver	Liver	9.54E-03	-1.71E-01	1.01E-01	2.46E-01
4055	GSM362994	GSE14520	Liver	Liver	3.19E-02	-1.47E-01	2.29E-02	3.15E-01
4056	GSM363008	GSE14520	Liver	Liver	3.03E-04	2.25E-01	1.05E-07	6.50E-01
4057	GSM363009	GSE14520	Liver	Liver	3.05E-02	1.48E-01	9.54E-05	4.94E-01
4058	GSM363010	GSE14520	Liver	Liver	4.63E-01	-6.94E-02	4.82E-02	2.83E-01
4059	GSM363011	GSE14520	Liver	Liver	6.19E-02	1.32E-01	1.25E-04	4.87E-01
4060	GSM363012	GSE14520	Liver	Liver	4.79E-02	1.38E-01	3.13E-04	4.61E-01
4061	GSM363013	GSE14520	Liver	Liver	1.11E-03	2.06E-01	7.36E-08	6.58E-01
4062	GSM363014	GSE14520	Liver	Liver	3.05E-02	-1.48E-01	2.40E-02	3.13E-01
4063	GSM363015	GSE14520	Liver	Liver	4.59E-02	1.39E-01	2.80E-03	3.93E-01
4064	GSM363016	GSE14520	Liver	Liver	1.12E-01	1.17E-01	6.74E-05	5.03E-01
4065	GSM363017	GSE14520	Liver	Liver	2.62E-05	2.57E-01	2.48E-05	5.29E-01

4066	GSM363029	GSE14520	Liver	Liver	8.29E-09	3.41E-01	3.90E-09	7.14E-01
4067	GSM363030	GSE14520	Liver	Liver	3.84E-02	-1.43E-01	1.15E-01	2.39E-01
4068	GSM363031	GSE14520	Liver	Liver	5.94E-02	1.33E-01	4.50E-04	4.50E-01
4069	GSM363032	GSE14520	Liver	Liver	7.30E-02	1.28E-01	1.67E-03	4.10E-01
4070	GSM363033	GSE14520	Liver	Liver	1.25E-01	1.14E-01	3.09E-03	3.90E-01
4071	GSM363034	GSE14520	Liver	Liver	2.14E-01	-9.82E-02	7.04E-03	3.61E-01
4072	GSM363035	GSE14520	Liver	Liver	3.05E-02	1.48E-01	6.74E-05	5.03E-01
4073	GSM363036	GSE14520	Liver	Liver	2.49E-01	-9.33E-02	1.88E-03	4.07E-01
4074	GSM363037	GSE14520	Liver	Liver	1.12E-01	1.17E-01	4.73E-05	5.12E-01
4075	GSM363038	GSE14520	Liver	Liver	3.47E-01	-8.13E-02	1.32E-02	3.38E-01
4076	GSM363039	GSE14520	Liver	Liver	1.65E-01	-1.06E-01	2.11E-04	4.72E-01
4077	GSM363048	GSE14520	Liver	Liver	7.92E-02	-1.26E-01	5.76E-03	3.69E-01
4078	GSM363049	GSE14520	Liver	Liver	5.69E-02	1.34E-01	2.26E-04	4.70E-01
4079	GSM363050	GSE14520	Liver	Liver	1.97E-03	-1.97E-01	8.45E-02	2.55E-01
4080	GSM363051	GSE14520	Liver	Liver	1.83E-01	1.03E-01	1.19E-02	3.42E-01
4081	GSM363052	GSE14520	Liver	Liver	2.80E-01	-8.93E-02	1.88E-03	4.07E-01
4082	GSM363053	GSE14520	Liver	Liver	1.04E-03	2.07E-01	3.05E-05	5.23E-01
4083	GSM363054	GSE14520	Liver	Liver	9.14E-20	5.24E-01	5.76E-09	7.07E-01
4084	GSM363055	GSE14520	Liver	Liver	4.79E-02	1.38E-01	1.67E-03	4.10E-01
4085	GSM363056	GSE14520	Liver	Liver	1.85E-03	-1.98E-01	3.20E-01	1.73E-01
4086	GSM363057	GSE14520	Liver	Liver	4.09E-03	1.86E-01	5.50E-04	4.45E-01
4087	GSM363069	GSE14520	Liver	Liver	9.04E-03	1.72E-01	2.29E-05	5.30E-01
4088	GSM363070	GSE14520	Liver	Liver	3.49E-04	-2.23E-01	1.43E-01	2.26E-01
4089	GSM363071	GSE14520	Liver	Liver	1.04E-01	-1.19E-01	1.06E-02	3.46E-01
4090	GSM363072	GSE14520	Liver	Liver	1.12E-02	1.68E-01	1.71E-05	5.38E-01
4091	GSM363073	GSE14520	Liver	Liver	1.12E-01	-1.17E-01	3.94E-02	2.92E-01
4092	GSM363074	GSE14520	Liver	Liver	1.63E-03	2.00E-01	9.54E-05	4.94E-01
4093	GSM363075	GSE14520	Liver	Liver	3.19E-02	1.47E-01	9.54E-05	4.94E-01
4094	GSM363076	GSE14520	Liver	Liver	2.14E-01	9.82E-02	9.89E-04	4.27E-01
4095	GSM363077	GSE14520	Liver	Liver	1.12E-01	1.17E-01	7.01E-04	4.37E-01
4096	GSM363078	GSE14520	Liver	Liver	3.67E-02	1.44E-01	2.09E-06	5.87E-01
4097	GSM363079	GSE14520	Liver	Liver	2.72E-01	9.03E-02	5.55E-03	3.70E-01
4098	GSM363080	GSE14520	Liver	Liver	1.34E-01	-1.12E-01	4.62E-02	2.85E-01
4099	GSM363081	GSE14520	Liver	Liver	4.39E-02	1.40E-01	2.48E-05	5.29E-01
4100	GSM363082	GSE14520	Liver	Liver	2.01E-01	-1.00E-01	4.04E-03	3.81E-01
4101	GSM363083	GSE14520	Liver	Liver	5.91E-07	3.00E-01	5.11E-07	6.18E-01
4102	GSM363084	GSE14520	Liver	Liver	1.79E-02	1.59E-01	6.74E-05	5.03E-01
4103	GSM363085	GSE14520	Liver	Liver	1.21E-01	1.15E-01	7.01E-04	4.37E-01
4104	GSM363086	GSE14520	Liver	Liver	2.49E-01	-9.33E-02	1.61E-02	3.30E-01
4105	GSM363087	GSE14520	Liver	Liver	2.72E-01	9.03E-02	6.93E-06	5.59E-01
4106	GSM363098	GSE14520	Liver	Liver	3.13E-01	-8.53E-02	2.00E-03	4.05E-01
4107	GSM363099	GSE14520	Liver	Liver	6.73E-02	1.30E-01	5.86E-06	5.63E-01
4108	GSM363100	GSE14520	Liver	Liver	4.94E-01	-6.65E-02	1.83E-02	3.25E-01
4109	GSM363101	GSE14520	Liver	Liver	9.04E-03	1.72E-01	9.54E-05	4.94E-01
4110	GSM363102	GSE14520	Liver	Liver	5.01E-02	-1.37E-01	2.02E-02	3.21E-01
4111	GSM363104	GSE14520	Liver	Liver	6.46E-02	1.31E-01	9.40E-06	5.52E-01
4112	GSM363105	GSE14520	Liver	Liver	1.34E-01	1.12E-01	3.13E-04	4.61E-01
4113	GSM363106	GSE14520	Liver	Liver	2.08E-01	-9.92E-02	3.76E-04	4.56E-01
4114	GSM363107	GSE14520	Liver	Liver	5.79E-03	1.80E-01	6.38E-06	5.61E-01
4115	GSM363108	GSE14520	Liver	Liver	2.29E-02	1.54E-01	2.00E-05	5.34E-01
4116	GSM363109	GSE14520	Liver	Liver	1.83E-01	1.03E-01	5.15E-04	4.47E-01
4117	GSM363115	GSE14520	Liver	Liver	5.47E-03	1.81E-01	5.09E-06	5.67E-01
4118	GSM363121	GSE14520	Liver	Liver	6.46E-02	1.31E-01	4.82E-04	4.49E-01
4119	GSM363122	GSE14520	Liver	Liver	2.28E-01	9.62E-02	7.01E-04	4.37E-01
4120	GSM363123	GSE14520	Liver	Liver	1.83E-01	-1.03E-01	1.63E-04	4.79E-01
4121	GSM363124	GSE14520	Liver	Liver	2.24E-03	1.95E-01	9.40E-06	5.52E-01
4122	GSM363125	GSE14520	Liver	Liver	3.63E-03	1.88E-01	3.30E-05	5.21E-01
4123	GSM363126	GSE14520	Liver	Liver	1.30E-01	-1.13E-01	5.15E-04	4.47E-01
4124	GSM363127	GSE14520	Liver	Liver	2.08E-01	-9.92E-02	1.88E-03	4.07E-01
4125	GSM363128	GSE14520	Liver	Liver	5.16E-03	-1.82E-01	6.78E-02	2.66E-01
4126	GSM363129	GSE14520	Liver	Liver	8.57E-02	-1.24E-01	2.00E-03	4.05E-01
4127	GSM363130	GSE14520	Liver	Liver	2.35E-01	9.52E-02	2.92E-04	4.63E-01
4128	GSM363142	GSE14520	Liver	Liver	4.39E-02	-1.40E-01	1.06E-02	3.46E-01
4129	GSM363143	GSE14520	Liver	Liver	2.62E-05	2.57E-01	1.05E-07	6.50E-01
4130	GSM363144	GSE14520	Liver	Liver	1.30E-01	-1.13E-01	2.49E-03	3.97E-01
4131	GSM363145	GSE14520	Liver	Liver	3.50E-02	1.45E-01	7.01E-04	4.37E-01
4132	GSM363146	GSE14520	Liver	Liver	2.62E-04	2.27E-01	1.51E-06	5.94E-01
4133	GSM363147	GSE14520	Liver	Liver	1.04E-01	1.19E-01	1.12E-03	4.23E-01
4134	GSM363148	GSE14520	Liver	Liver	1.65E-01	1.06E-01	2.68E-05	5.27E-01
4135	GSM363149	GSE14520	Liver	Liver	1.77E-01	1.04E-01	9.54E-05	4.94E-01
4136	GSM363150	GSE14520	Liver	Liver	7.45E-04	-2.12E-01	2.10E-01	2.03E-01
4137	GSM363151	GSE14520	Liver	Liver	2.21E-01	-9.72E-02	1.63E-04	4.79E-01
4138	GSM363152	GSE14520	Liver	Liver	1.35E-05	-2.65E-01	3.52E-01	1.66E-01
4139	GSM363164	GSE14520	Liver	Liver	2.27E-04	-2.29E-01	1.11E-01	2.41E-01

4140	GSM363166	GSE14520	Liver	Liver	1.60E-01	-1.07E-01	3.27E-03	3.88E-01
4141	GSM363168	GSE14520	Liver	Liver	2.44E-04	-2.28E-01	2.83E-01	1.82E-01
4142	GSM363169	GSE14520	Liver	Liver	2.64E-02	1.51E-01	8.86E-05	4.96E-01
4143	GSM363170	GSE14520	Liver	Liver	4.20E-02	1.41E-01	1.34E-04	4.85E-01
4144	GSM363172	GSE14520	Liver	Liver	2.53E-03	1.93E-01	9.54E-05	4.94E-01
4145	GSM363174	GSE14520	Liver	Liver	1.04E-01	1.19E-01	1.41E-03	4.16E-01
4146	GSM363176	GSE14520	Liver	Liver	7.96E-05	2.43E-01	5.60E-07	6.16E-01
4147	GSM363178	GSE14520	Liver	Liver	3.50E-02	1.45E-01	1.38E-05	5.43E-01
4148	GSM363180	GSE14520	Liver	Liver	1.63E-03	2.00E-01	1.75E-04	4.77E-01
4149	GSM363182	GSE14520	Liver	Liver	5.16E-03	-1.82E-01	9.51E-03	3.50E-01
4150	GSM363184	GSE14520	Liver	Liver	2.21E-11	-3.92E-01	2.75E-03	-3.94E-01
4151	GSM363186	GSE14520	Liver	Liver	1.00E-04	2.40E-01	3.99E-07	6.23E-01
4152	GSM363188	GSE14520	Liver	Liver	1.18E-03	2.05E-01	2.58E-07	6.32E-01
4153	GSM363190	GSE14520	Liver	Liver	2.18E-02	1.55E-01	1.41E-03	4.16E-01
4154	GSM363192	GSE14520	Liver	Liver	1.30E-01	-1.13E-01	2.49E-03	3.97E-01
4155	GSM363194	GSE14520	Liver	Liver	1.34E-01	1.12E-01	2.64E-03	3.95E-01
4156	GSM363196	GSE14520	Liver	Liver	2.29E-02	1.54E-01	1.88E-04	4.75E-01
4157	GSM363198	GSE14520	Liver	Liver	9.63E-02	-1.21E-01	2.49E-03	3.97E-01
4158	GSM363200	GSE14520	Liver	Liver	4.62E-04	2.19E-01	5.09E-06	5.67E-01
4159	GSM363202	GSE14520	Liver	Liver	2.91E-02	1.49E-01	3.86E-05	5.17E-01
4160	GSM363204	GSE14520	Liver	Liver	1.01E-02	-1.70E-01	4.12E-02	2.90E-01
4161	GSM363205	GSE14520	Liver	Liver	5.94E-02	1.33E-01	2.92E-04	4.63E-01
4162	GSM363207	GSE14520	Liver	Liver	6.19E-02	-1.32E-01	4.97E-03	3.74E-01
4163	GSM363209	GSE14520	Liver	Liver	2.08E-01	-9.92E-02	4.04E-03	3.81E-01
4164	GSM363211	GSE14520	Liver	Liver	1.79E-02	-1.59E-01	2.64E-03	3.95E-01
4165	GSM363213	GSE14520	Liver	Liver	1.53E-02	-1.62E-01	4.12E-02	2.90E-01
4166	GSM363215	GSE14520	Liver	Liver	2.38E-03	1.94E-01	2.00E-05	5.34E-01
4167	GSM363217	GSE14520	Liver	Liver	2.18E-02	-1.55E-01	7.05E-02	2.64E-01
4168	GSM363218	GSE14520	Liver	Liver	7.45E-04	-2.12E-01	3.35E-02	2.99E-01
4169	GSM363220	GSE14520	Liver	Liver	4.79E-02	1.38E-01	9.54E-05	4.94E-01
4170	GSM363222	GSE14520	Liver	Liver	1.74E-05	-2.62E-01	1.22E-01	-2.35E-01
4171	GSM363224	GSE14520	Liver	Liver	1.01E-02	1.70E-01	6.93E-06	5.59E-01
4172	GSM363226	GSE14520	Liver	Liver	1.00E-01	1.20E-01	3.13E-04	4.61E-01
4173	GSM363228	GSE14520	Liver	Liver	2.77E-02	-1.50E-01	4.30E-02	2.88E-01
4174	GSM363230	GSE14520	Liver	Liver	4.39E-02	-1.40E-01	5.39E-02	2.77E-01
4175	GSM363232	GSE14520	Liver	Liver	1.89E-01	1.02E-01	1.67E-03	4.10E-01
4176	GSM363235	GSE14520	Liver	Liver	9.73E-04	2.08E-01	1.63E-04	4.79E-01
4177	GSM363237	GSE14520	Liver	Liver	1.12E-02	1.68E-01	1.75E-04	4.77E-01
4178	GSM363239	GSE14520	Liver	Liver	2.96E-01	-8.73E-02	6.09E-03	3.67E-01
4179	GSM363241	GSE14520	Liver	Liver	2.42E-01	9.42E-02	1.75E-04	4.77E-01
4180	GSM363243	GSE14520	Liver	Liver	1.25E-02	1.66E-01	5.09E-06	5.67E-01
4181	GSM363245	GSE14520	Liver	Liver	8.59E-05	2.42E-01	2.28E-06	5.85E-01
4182	GSM363247	GSE14520	Liver	Liver	2.64E-01	-9.13E-02	5.50E-04	4.45E-01
4183	GSM363249	GSE14520	Liver	Liver	7.36E-05	-2.44E-01	4.12E-02	2.90E-01
4184	GSM363251	GSE14520	Liver	Liver	2.64E-01	-9.13E-02	6.43E-03	3.65E-01
4185	GSM363263	GSE14520	Liver	Liver	6.19E-02	-1.32E-01	4.31E-04	4.52E-01
4186	GSM363264	GSE14520	Liver	Liver	1.00E-01	-1.20E-01	6.43E-03	3.65E-01
4187	GSM363265	GSE14520	Liver	Liver	1.74E-03	1.99E-01	1.38E-05	5.43E-01
4188	GSM363266	GSE14520	Liver	Liver	1.04E-01	1.19E-01	7.27E-05	5.01E-01
4189	GSM363267	GSE14520	Liver	Liver	1.74E-03	1.99E-01	1.38E-05	5.43E-01
4190	GSM363268	GSE14520	Liver	Liver	4.01E-02	-1.42E-01	2.73E-04	4.65E-01
4191	GSM363269	GSE14520	Liver	Liver	4.33E-01	-7.24E-02	2.18E-02	3.17E-01
4192	GSM363270	GSE14520	Liver	Liver	7.60E-02	1.27E-01	5.50E-04	4.45E-01
4193	GSM363271	GSE14520	Liver	Liver	3.34E-05	2.54E-01	6.74E-05	5.03E-01
4194	GSM363272	GSE14520	Liver	Liver	9.04E-03	1.72E-01	1.34E-04	4.85E-01
4195	GSM363273	GSE14520	Liver	Liver	3.05E-02	1.48E-01	5.11E-05	5.10E-01
4196	GSM363274	GSE14520	Liver	Liver	7.97E-04	-2.11E-01	1.60E-03	4.12E-01
4197	GSM363275	GSE14520	Liver	Liver	1.46E-02	1.63E-01	5.11E-05	5.10E-01
4198	GSM363288	GSE14520	Liver	Liver	4.39E-02	1.40E-01	1.34E-04	4.85E-01
4199	GSM363289	GSE14520	Liver	Liver	4.39E-02	-1.40E-01	8.14E-02	2.57E-01
4200	GSM363290	GSE14520	Liver	Liver	4.39E-02	1.40E-01	1.75E-04	4.77E-01
4201	GSM363291	GSE14520	Liver	Liver	4.39E-02	1.40E-01	1.60E-03	4.12E-01
4202	GSM363292	GSE14520	Liver	Liver	7.60E-02	1.27E-01	2.26E-04	4.70E-01
4203	GSM363293	GSE14520	Liver	Liver	6.86E-03	1.77E-01	5.09E-06	5.67E-01
4204	GSM363294	GSE14520	Liver	Liver	9.65E-06	2.69E-01	2.58E-07	6.32E-01
4205	GSM363295	GSE14520	Liver	Liver	7.60E-02	1.27E-01	8.86E-05	4.96E-01
4206	GSM363296	GSE14520	Liver	Liver	3.19E-02	1.47E-01	1.60E-03	4.12E-01
4207	GSM363297	GSE14520	Liver	Liver	6.97E-04	2.13E-01	1.51E-04	4.81E-01
4208	GSM363298	GSE14520	Liver	Liver	1.04E-01	-1.19E-01	3.35E-02	2.99E-01
4209	GSM363309	GSE14520	Liver	Liver	1.25E-02	1.66E-01	2.48E-05	5.29E-01
4210	GSM363310	GSE14520	Liver	Liver	4.59E-02	1.39E-01	2.48E-05	5.29E-01
4211	GSM363311	GSE14520	Liver	Liver	6.48E-03	1.78E-01	9.54E-05	4.94E-01
4212	GSM363312	GSE14520	Liver	Liver	4.73E-01	-6.85E-02	1.25E-04	4.87E-01
4213	GSM363313	GSE14520	Liver	Liver	2.42E-01	-9.42E-02	8.89E-04	4.30E-01

4214	GSM363314	GSE14520	Liver	Liver	5.30E-04	2.17E-01	1.97E-04	4.74E-01
4215	GSM363315	GSE14520	Liver	Liver	3.23E-03	-1.89E-01	1.31E-01	2.31E-01
4216	GSM363316	GSE14520	Liver	Liver	1.46E-04	-2.35E-01	1.67E-01	2.17E-01
4217	GSM363317	GSE14520	Liver	Liver	1.12E-01	-1.17E-01	2.49E-03	3.97E-01
4218	GSM363326	GSE14520	Liver	Liver	1.31E-02	1.65E-01	2.92E-04	4.63E-01
4219	GSM363327	GSE14520	Liver	Liver	9.26E-02	1.22E-01	5.50E-04	4.45E-01
4220	GSM363328	GSE14520	Liver	Liver	1.54E-01	1.08E-01	1.08E-04	4.90E-01
4221	GSM363329	GSE14520	Liver	Liver	2.11E-04	-2.30E-01	2.40E-02	3.13E-01
4222	GSM363330	GSE14520	Liver	Liver	3.19E-02	1.47E-01	8.23E-05	4.98E-01
4223	GSM363331	GSE14520	Liver	Liver	2.38E-03	-1.94E-01	1.92E-02	3.23E-01
4224	GSM363332	GSE14520	Liver	Liver	1.46E-04	-2.35E-01	6.79E-03	3.63E-01
4225	GSM363333	GSE14520	Liver	Liver	4.87E-03	1.83E-01	1.27E-03	4.19E-01
4226	GSM363334	GSE14520	Liver	Liver	8.56E-03	1.73E-01	3.76E-04	4.56E-01
4227	GSM363335	GSE14520	Liver	Liver	1.85E-03	1.98E-01	5.60E-07	6.16E-01
4228	GSM363336	GSE14520	Liver	Liver	1.97E-03	1.97E-01	6.93E-06	5.59E-01
4229	GSM363337	GSE14520	Liver	Liver	1.21E-01	1.15E-01	4.53E-03	3.77E-01
4230	GSM363339	GSE14520	Liver	Liver	8.57E-02	-1.24E-01	1.88E-03	4.07E-01
4231	GSM363341	GSE14520	Liver	Liver	1.65E-01	1.06E-01	4.04E-03	3.81E-01
4232	GSM363343	GSE14520	Liver	Liver	3.49E-04	2.23E-01	7.83E-07	6.09E-01
4233	GSM363344	GSE14520	Liver	Liver	7.30E-02	-1.28E-01	1.83E-02	3.25E-01
4234	GSM363346	GSE14520	Liver	Liver	1.16E-01	-1.16E-01	1.09E-02	3.45E-01
4235	GSM363348	GSE14520	Liver	Liver	1.70E-02	-1.60E-01	4.30E-02	2.88E-01
4236	GSM363350	GSE14520	Liver	Liver	7.92E-02	1.26E-01	6.43E-03	3.65E-01
4237	GSM363352	GSE14520	Liver	Liver	2.84E-06	2.83E-01	2.34E-07	6.34E-01
4238	GSM363354	GSE14520	Liver	Liver	1.53E-02	-1.62E-01	1.76E-01	2.14E-01
4239	GSM363355	GSE14520	Liver	Liver	2.18E-02	-1.55E-01	1.00E-02	3.48E-01
4240	GSM363357	GSE14520	Liver	Liver	1.12E-01	-1.17E-01	2.64E-03	3.95E-01
4241	GSM363358	GSE14520	Liver	Liver	5.94E-02	1.33E-01	1.25E-04	4.87E-01
4242	GSM363360	GSE14520	Liver	Liver	9.54E-03	-1.71E-01	8.45E-02	2.55E-01
4243	GSM363362	GSE14520	Liver	Liver	2.49E-01	9.33E-02	2.00E-03	4.05E-01
4244	GSM363364	GSE14520	Liver	Liver	6.97E-04	2.13E-01	2.48E-05	5.29E-01
4245	GSM363366	GSE14520	Liver	Liver	2.80E-01	-8.93E-02	1.25E-04	4.87E-01
4246	GSM363368	GSE14520	Liver	Liver	5.23E-02	1.36E-01	8.89E-04	4.30E-01
4247	GSM363371	GSE14520	Liver	Liver	2.96E-01	8.73E-02	4.02E-04	4.54E-01
4248	GSM363376	GSE14520	Liver	Liver	3.45E-09	3.49E-01	5.12E-08	6.65E-01
4249	GSM363378	GSE14520	Liver	Liver	2.64E-01	-9.13E-02	1.27E-02	3.39E-01
4250	GSM363384	GSE14520	Liver	Liver	4.02E-04	2.21E-01	1.85E-05	5.36E-01
4251	GSM363386	GSE14520	Liver	Liver	2.69E-03	1.92E-01	1.85E-05	5.36E-01
4252	GSM363388	GSE14520	Liver	Liver	2.21E-11	3.92E-01	7.62E-09	7.02E-01
4253	GSM363391	GSE14520	Liver	Liver	6.97E-04	2.13E-01	1.38E-05	5.43E-01
4254	GSM363393	GSE14520	Liver	Liver	5.41E-23	5.66E-01	6.66E-15	9.27E-01
4255	GSM363400	GSE14520	Liver	Liver	1.00E-01	1.20E-01	2.49E-03	3.97E-01
4256	GSM363404	GSE14520	Liver	Liver	1.77E-01	1.04E-01	4.70E-03	3.76E-01
4257	GSM712532	GSE14520	Liver	Liver	1.12E-02	-1.68E-01	8.26E-03	3.55E-01
4258	GSM712534	GSE14520	Liver	Liver	7.36E-05	2.44E-01	1.65E-07	6.41E-01
4259	GSM712542	GSE14520	Liver	Liver	1.46E-02	-1.63E-01	9.47E-04	4.28E-01
4260	GSM362947	GSE14520	Liver	Liver	2.77E-02	1.50E-01	1.38E-05	5.43E-01
4261	GSM362948	GSE14520	Liver	Liver	4.79E-02	1.38E-01	7.01E-04	4.37E-01
4262	GSM362949	GSE14520	Liver	Liver	3.66E-01	7.94E-02	3.13E-04	4.61E-01
4263	GSM362950	GSE14520	Liver	Liver	1.97E-02	1.57E-01	2.43E-04	4.68E-01
4264	GSM362952	GSE14520	Liver	Liver	3.05E-02	1.48E-01	2.26E-04	4.70E-01
4265	GSM362954	GSE14520	Liver	Liver	1.85E-03	1.98E-01	8.23E-05	4.98E-01
4266	GSM362956	GSE14520	Liver	Liver	1.35E-04	-2.36E-01	4.28E-01	1.50E-01
4267	GSM363420	GSE14520	Liver	Liver	5.94E-02	-1.33E-01	2.18E-02	3.17E-01
4268	GSM363422	GSE14520	Liver	Liver	1.44E-03	2.02E-01	6.25E-05	5.05E-01
4269	GSM363424	GSE14520	Liver	Liver	9.63E-02	1.21E-01	5.51E-05	5.08E-01
4270	GSM363426	GSE14520	Liver	Liver	1.71E-01	1.05E-01	7.04E-03	3.61E-01
4271	GSM363428	GSE14520	Liver	Liver	1.12E-01	1.17E-01	4.53E-03	3.77E-01
4272	GSM363430	GSE14520	Liver	Liver	2.40E-02	-1.53E-01	4.62E-02	2.85E-01
4273	GSM363432	GSE14520	Liver	Liver	1.89E-01	-1.02E-01	3.07E-02	3.03E-01
4274	GSM363434	GSE14520	Liver	Liver	2.69E-03	-1.92E-01	3.87E-01	1.58E-01
4275	GSM363436	GSE14520	Liver	Liver	1.95E-01	-1.01E-01	2.40E-02	3.13E-01
4276	GSM363438	GSE14520	Liver	Liver	6.30E-05	2.46E-01	7.83E-07	6.09E-01
4277	GSM363440	GSE14520	Liver	Liver	1.30E-01	-1.13E-01	1.21E-02	3.41E-01
4278	GSM363442	GSE14520	Liver	Liver	3.66E-01	7.94E-02	5.45E-03	3.70E-01
4279	GSM363444	GSE14520	Liver	Liver	1.52E-07	3.13E-01	6.53E-07	6.12E-01
4280	GSM363446	GSE14520	Liver	Liver	7.30E-02	-1.28E-01	9.51E-03	3.50E-01
4281	GSM363448	GSE14520	Liver	Liver	3.30E-01	-8.33E-02	6.56E-04	4.39E-01
4282	GSM907025	GSE36924	Pancreatic Ductal Adenocarcinoma	Pancreatic	5.66E-01	-6.03E-02	2.77E-01	-1.86E-01
4283	GSM907026	GSE36924	Pancreatic Ductal Adenocarcinoma	Pancreatic	4.44E-13	-4.26E-01	1.06E-04	-4.98E-01
4284	GSM907027	GSE36924	Pancreatic Ductal Adenocarcinoma	Pancreatic	6.57E-17	-4.88E-01	1.39E-04	-4.90E-01
4285	GSM907028	GSE36924	Pancreatic Ductal Adenocarcinoma	Pancreatic	6.62E-06	-2.76E-01	2.75E-03	-4.00E-01
4286	GSM907029	GSE36924	Pancreatic Ductal Adenocarcinoma	Pancreatic	4.45E-03	-1.86E-01	6.99E-02	-2.68E-01
4287	GSM907030	GSE36924	Pancreatic Ductal Adenocarcinoma	Pancreatic	3.02E-03	-1.93E-01	2.59E-01	-1.91E-01

4288	GSM907031	GSE36924	Pancreatic Ductal Adenocarcinoma	Pancreatic	4.16E-01	-7.49E-02	4.18E-01	-1.54E-01
4289	GSM907032	GSE36924	Pancreatic Ductal Adenocarcinoma	Pancreatic	6.72E-02	-1.31E-01	1.53E-01	-2.25E-01
4290	GSM907033	GSE36924	Pancreatic Ductal Adenocarcinoma	Pancreatic	5.48E-03	-1.82E-01	1.69E-01	-2.19E-01
4291	GSM907034	GSE36924	Pancreatic Ductal Adenocarcinoma	Pancreatic	1.46E-03	-2.04E-01	1.53E-01	-2.25E-01
4292	GSM907035	GSE36924	Pancreatic Ductal Adenocarcinoma	Pancreatic	8.88E-20	-5.29E-01	4.05E-06	-5.80E-01
4293	GSM907036	GSE36924	Pancreatic Ductal Adenocarcinoma	Pancreatic	5.53E-02	-1.36E-01	1.87E-01	-2.13E-01
4294	GSM907037	GSE36924	Pancreatic Ductal Adenocarcinoma	Pancreatic	3.73E-02	-1.45E-01	1.57E-01	-2.24E-01
4295	GSM907038	GSE36924	Pancreatic Ductal Adenocarcinoma	Pancreatic	1.89E-01	-1.03E-01	1.29E-01	-2.36E-01
4296	GSM907039	GSE36924	Pancreatic Ductal Adenocarcinoma	Pancreatic	1.90E-02	-1.59E-01	2.42E-01	-1.96E-01
4297	GSM907040	GSE36924	Pancreatic Ductal Adenocarcinoma	Pancreatic	1.46E-01	-1.11E-01	4.18E-01	-1.54E-01
4298	GSM907041	GSE36924	Pancreatic Ductal Adenocarcinoma	Pancreatic	6.05E-04	-2.18E-01	1.43E-01	-2.30E-01
4299	GSM907042	GSE36924	Pancreatic Ductal Adenocarcinoma	Pancreatic	9.33E-05	-2.43E-01	1.35E-01	-2.33E-01
4300	GSM907043	GSE36924	Pancreatic Ductal Adenocarcinoma	Pancreatic	2.23E-01	-9.78E-02	4.68E-01	-1.43E-01
4301	GSM907044	GSE36924	Pancreatic Ductal Adenocarcinoma	Pancreatic	1.71E-01	1.06E-01	1.10E-01	2.45E-01
4302	GSM907045	GSE36924	Pancreatic Ductal Adenocarcinoma	Pancreatic	2.41E-03	-1.96E-01	8.53E-02	-2.58E-01
4303	GSM907046	GSE36924	Pancreatic Ductal Adenocarcinoma	Pancreatic	6.38E-14	-4.41E-01	4.59E-04	-4.56E-01
4304	GSM907047	GSE36924	Pancreatic Ductal Adenocarcinoma	Pancreatic	1.98E-01	-1.02E-01	1.50E-01	-2.27E-01
4305	GSM907048	GSE36924	Pancreatic Ductal Adenocarcinoma	Pancreatic	9.09E-02	-1.24E-01	3.77E-01	-1.63E-01
4306	GSM907049	GSE36924	Pancreatic Ductal Adenocarcinoma	Pancreatic	5.99E-01	5.72E-02	2.19E-01	2.03E-01
4307	GSM907050	GSE36924	Pancreatic Ductal Adenocarcinoma	Pancreatic	2.97E-01	-8.80E-02	5.44E-01	-1.28E-01
4308	GSM907051	GSE36924	Pancreatic Ductal Adenocarcinoma	Pancreatic	7.49E-02	-1.29E-01	1.69E-01	-2.19E-01
4309	GSM907052	GSE36924	Pancreatic Ductal Adenocarcinoma	Pancreatic	1.34E-01	-1.13E-01	1.50E-01	-2.27E-01
4310	GSM907053	GSE36924	Pancreatic Ductal Adenocarcinoma	Pancreatic	1.90E-01	-1.03E-01	1.53E-01	-2.25E-01
4311	GSM907054	GSE36924	Pancreatic Ductal Adenocarcinoma	Pancreatic	2.30E-01	-9.69E-02	3.77E-01	-1.63E-01
4312	GSM907055	GSE36924	Pancreatic Ductal Adenocarcinoma	Pancreatic	7.68E-02	1.28E-01	9.58E-02	2.52E-01
4313	GSM907056	GSE36924	Pancreatic Ductal Adenocarcinoma	Pancreatic	1.51E-08	-3.39E-01	6.11E-03	-3.72E-01
4314	GSM907057	GSE36924	Pancreatic Ductal Adenocarcinoma	Pancreatic	2.76E-02	1.51E-01	2.34E-02	3.19E-01
4315	GSM907058	GSE36924	Pancreatic Ductal Adenocarcinoma	Pancreatic	2.02E-02	-1.58E-01	1.07E-01	-2.46E-01
4316	GSM907059	GSE36924	Pancreatic Ductal Adenocarcinoma	Pancreatic	3.12E-01	-8.62E-02	5.12E-01	-1.35E-01
4317	GSM907060	GSE36924	Pancreatic Ductal Adenocarcinoma	Pancreatic	1.93E-09	-3.58E-01	1.33E-03	-4.23E-01
4318	GSM907061	GSE36924	Pancreatic Ductal Adenocarcinoma	Pancreatic	5.73E-01	-5.96E-02	3.07E-01	1.79E-01
4319	GSM907062	GSE36924	Pancreatic Ductal Adenocarcinoma	Pancreatic	3.21E-03	1.91E-01	1.63E-02	3.34E-01
4320	GSM907063	GSE36924	Pancreatic Ductal Adenocarcinoma	Pancreatic	1.17E-16	4.84E-01	2.02E-09	7.36E-01
4321	GSM907064	GSE36924	Pancreatic Ductal Adenocarcinoma	Pancreatic	3.84E-01	7.81E-02	3.38E-01	1.71E-01
4322	GSM907065	GSE36924	Pancreatic Ductal Adenocarcinoma	Pancreatic	6.40E-05	-2.48E-01	7.89E-02	-2.62E-01
4323	GSM907066	GSE36924	Pancreatic Ductal Adenocarcinoma	Pancreatic	6.01E-01	5.71E-02	3.77E-01	-1.63E-01
4324	GSM907067	GSE36924	Pancreatic Ductal Adenocarcinoma	Pancreatic	1.23E-03	2.07E-01	2.49E-02	3.16E-01
4325	GSM907068	GSE36924	Pancreatic Ductal Adenocarcinoma	Pancreatic	3.14E-01	-8.61E-02	2.44E-01	-1.95E-01
4326	GSM907069	GSE36924	Pancreatic Ductal Adenocarcinoma	Pancreatic	6.62E-07	3.01E-01	2.11E-03	4.08E-01
4327	GSM907070	GSE36924	Pancreatic Ductal Adenocarcinoma	Pancreatic	1.25E-01	-1.15E-01	2.44E-01	-1.95E-01
4328	GSM907071	GSE36924	Pancreatic Ductal Adenocarcinoma	Pancreatic	1.03E-01	1.20E-01	8.31E-02	2.60E-01
4329	GSM907072	GSE36924	Pancreatic Ductal Adenocarcinoma	Pancreatic	1.21E-05	-2.69E-01	9.22E-02	-2.54E-01
4330	GSM907073	GSE36924	Pancreatic Ductal Adenocarcinoma	Pancreatic	4.66E-03	1.85E-01	4.65E-02	2.88E-01
4331	GSM907074	GSE36924	Pancreatic Ductal Adenocarcinoma	Pancreatic	2.82E-03	1.94E-01	2.74E-02	3.12E-01
4332	GSM907075	GSE36924	Pancreatic Ductal Adenocarcinoma	Pancreatic	2.45E-05	-2.60E-01	1.06E-01	-2.47E-01
4333	GSM907076	GSE36924	Pancreatic Ductal Adenocarcinoma	Pancreatic	2.60E-03	-1.95E-01	3.51E-02	-3.01E-01
4334	GSM907077	GSE36924	Pancreatic Ductal Adenocarcinoma	Pancreatic	6.71E-01	-5.05E-02	4.54E-01	1.46E-01
4335	GSM907078	GSE36924	Pancreatic Ductal Adenocarcinoma	Pancreatic	8.28E-04	-2.13E-01	4.26E-02	-2.92E-01
4336	GSM907079	GSE36924	Pancreatic Ductal Adenocarcinoma	Pancreatic	2.53E-01	-9.36E-02	3.07E-01	-1.79E-01
4337	GSM907080	GSE36924	Pancreatic Ductal Adenocarcinoma	Pancreatic	1.07E-01	-1.19E-01	2.82E-01	-1.85E-01
4338	GSM907081	GSE36924	Pancreatic Ductal Adenocarcinoma	Pancreatic	4.77E-01	6.88E-02	4.95E-01	1.38E-01
4339	GSM907082	GSE36924	Pancreatic Ductal Adenocarcinoma	Pancreatic	4.96E-01	6.70E-02	2.77E-01	1.86E-01
4340	GSM907083	GSE36924	Pancreatic Ductal Adenocarcinoma	Pancreatic	3.70E-02	-1.45E-01	7.89E-02	-2.62E-01
4341	GSM907084	GSE36924	Pancreatic Ductal Adenocarcinoma	Pancreatic	1.16E-01	-1.17E-01	1.01E-01	-2.49E-01
4342	GSM907085	GSE36924	Pancreatic Ductal Adenocarcinoma	Pancreatic	1.85E-03	-2.01E-01	9.58E-02	-2.52E-01
4343	GSM907086	GSE36924	Pancreatic Ductal Adenocarcinoma	Pancreatic	3.77E-01	-7.89E-02	4.48E-01	-1.48E-01
4344	GSM907087	GSE36924	Pancreatic Ductal Adenocarcinoma	Pancreatic	1.88E-03	-2.00E-01	1.29E-01	-2.36E-01
4345	GSM907088	GSE36924	Pancreatic Ductal Adenocarcinoma	Pancreatic	4.59E-01	7.06E-02	1.69E-01	2.19E-01
4346	GSM907089	GSE36924	Pancreatic Ductal Adenocarcinoma	Pancreatic	5.15E-01	-6.51E-02	3.07E-01	-1.79E-01
4347	GSM907090	GSE36924	Pancreatic Ductal Adenocarcinoma	Pancreatic	1.10E-04	2.41E-01	1.44E-02	3.39E-01
4348	GSM907091	GSE36924	Pancreatic Ductal Adenocarcinoma	Pancreatic	2.10E-18	-5.10E-01	5.70E-08	-6.72E-01
4349	GSM907092	GSE36924	Pancreatic Ductal Adenocarcinoma	Pancreatic	7.21E-05	-2.47E-01	1.07E-01	-2.46E-01
4350	GSM907093	GSE36924	Pancreatic Ductal Adenocarcinoma	Pancreatic	3.69E-01	-7.98E-02	3.71E-01	-1.64E-01
4351	GSM907094	GSE36924	Pancreatic Ductal Adenocarcinoma	Pancreatic	5.29E-06	-2.79E-01	2.57E-02	-3.15E-01
4352	GSM907095	GSE36924	Pancreatic Ductal Adenocarcinoma	Pancreatic	4.87E-03	-1.84E-01	1.15E-01	-2.42E-01
4353	GSM907096	GSE36924	Pancreatic Ductal Adenocarcinoma	Pancreatic	2.15E-06	-2.89E-01	1.74E-02	-3.31E-01
4354	GSM907097	GSE36924	Pancreatic Ductal Adenocarcinoma	Pancreatic	3.01E-01	-8.75E-02	4.18E-01	-1.54E-01
4355	GSM907098	GSE36924	Pancreatic Ductal Adenocarcinoma	Pancreatic	4.19E-03	1.87E-01	7.58E-02	2.64E-01
4356	GSM907099	GSE36924	Pancreatic Ductal Adenocarcinoma	Pancreatic	1.35E-01	1.13E-01	1.66E-01	2.21E-01
4357	GSM907100	GSE36924	Pancreatic Ductal Adenocarcinoma	Pancreatic	3.39E-01	-8.31E-02	1.69E-01	-2.19E-01
4358	GSM907101	GSE36924	Pancreatic Ductal Adenocarcinoma	Pancreatic	1.02E-06	-2.97E-01	2.75E-03	-4.00E-01
4359	GSM907102	GSE36924	Pancreatic Ductal Adenocarcinoma	Pancreatic	2.81E-01	-9.00E-02	4.38E-01	-1.50E-01
4360	GSM907103	GSE36924	Pancreatic Ductal Adenocarcinoma	Pancreatic	1.02E-02	-1.71E-01	2.12E-01	-2.05E-01
4361	GSM907104	GSE36924	Pancreatic Ductal Adenocarcinoma	Pancreatic	9.13E-08	-3.22E-01	3.86E-03	-3.88E-01

4362	GSM907105	GSE36924	Pancreatic Ductal Adenocarcinoma	Pancreatic	5.10E-04	-2.20E-01	5.30E-02	-2.82E-01
4363	GSM907106	GSE36924	Pancreatic Ductal Adenocarcinoma	Pancreatic	3.33E-06	2.84E-01	8.25E-04	4.39E-01
4364	GSM907107	GSE36924	Pancreatic Ductal Adenocarcinoma	Pancreatic	2.39E-09	-3.56E-01	1.01E-03	-4.32E-01
4365	GSM907108	GSE36924	Pancreatic Ductal Adenocarcinoma	Pancreatic	1.98E-01	1.02E-01	3.38E-01	1.71E-01
4366	GSM907109	GSE36924	Pancreatic Ductal Adenocarcinoma	Pancreatic	9.81E-02	1.22E-01	1.50E-01	2.27E-01
4367	GSM907110	GSE36924	Pancreatic Ductal Adenocarcinoma	Pancreatic	5.75E-06	-2.78E-01	3.35E-02	-3.03E-01
4368	GSM907111	GSE36924	Pancreatic Ductal Adenocarcinoma	Pancreatic	5.26E-05	-2.51E-01	3.51E-02	-3.01E-01
4369	GSM907112	GSE36924	Pancreatic Ductal Adenocarcinoma	Pancreatic	4.59E-02	-1.40E-01	2.77E-01	-1.86E-01
4370	GSM907113	GSE36924	Pancreatic Ductal Adenocarcinoma	Pancreatic	3.74E-01	7.93E-02	1.95E-01	2.10E-01
4371	GSM907114	GSE36924	Pancreatic Ductal Adenocarcinoma	Pancreatic	1.64E-01	-1.07E-01	1.95E-01	2.10E-01
4372	GSM907115	GSE36924	Pancreatic Ductal Adenocarcinoma	Pancreatic	2.33E-11	-3.95E-01	2.88E-04	-4.70E-01
4373	PDAC_Survival_10	E-MEXP-278	Pancreatic Ductal Adenocarcinoma	Pancreatic	1.27E-02	1.65E-01	0.00E+00	0.00E+00
4374	PDAC_Survival_15	E-MEXP-278	Pancreatic Ductal Adenocarcinoma	Pancreatic	1.29E-08	3.37E-01	0.00E+00	0.00E+00
4375	PDAC_Survival_15	E-MEXP-278	Pancreatic Ductal Adenocarcinoma	Pancreatic	1.17E-07	3.16E-01	0.00E+00	0.00E+00
4376	PDAC_Survival_16	E-MEXP-278	Pancreatic Ductal Adenocarcinoma	Pancreatic	2.39E-04	2.28E-01	0.00E+00	0.00E+00
4377	PDAC_Survival_16	E-MEXP-278	Pancreatic Ductal Adenocarcinoma	Pancreatic	7.06E-19	5.11E-01	0.00E+00	0.00E+00
4378	PDAC_Survival_16	E-MEXP-278	Pancreatic Ductal Adenocarcinoma	Pancreatic	3.12E-04	2.24E-01	0.00E+00	0.00E+00
4379	PDAC_Survival_18	E-MEXP-278	Pancreatic Ductal Adenocarcinoma	Pancreatic	9.05E-14	4.33E-01	0.00E+00	0.00E+00
4380	PDAC_Survival_19	E-MEXP-278	Pancreatic Ductal Adenocarcinoma	Pancreatic	1.96E-11	-3.92E-01	0.00E+00	0.00E+00
4381	PDAC_Survival_19	E-MEXP-278	Pancreatic Ductal Adenocarcinoma	Pancreatic	1.24E-06	-2.91E-01	0.00E+00	0.00E+00
4382	PDAC_Survival_19	E-MEXP-278	Pancreatic Ductal Adenocarcinoma	Pancreatic	1.21E-09	-3.58E-01	0.00E+00	0.00E+00
4383	PDAC_Survival_20	E-MEXP-278	Pancreatic Ductal Adenocarcinoma	Pancreatic	4.49E-03	1.84E-01	0.00E+00	0.00E+00
4384	PDAC_Survival_21	E-MEXP-278	Pancreatic Ductal Adenocarcinoma	Pancreatic	1.93E-02	1.57E-01	0.00E+00	0.00E+00
4385	PDAC_Survival_21	E-MEXP-278	Pancreatic Ductal Adenocarcinoma	Pancreatic	6.93E-03	1.76E-01	0.00E+00	0.00E+00
4386	PDAC_Survival_23	E-MEXP-278	Pancreatic Ductal Adenocarcinoma	Pancreatic	8.96E-06	-2.69E-01	0.00E+00	0.00E+00
4387	PDAC_Survival_33	E-MEXP-278	Pancreatic Ductal Adenocarcinoma	Pancreatic	3.60E-03	1.87E-01	0.00E+00	0.00E+00
4388	PDAC_Survival_45	E-MEXP-278	Pancreatic Ductal Adenocarcinoma	Pancreatic	3.68E-02	-1.43E-01	0.00E+00	0.00E+00
4389	PDAC_Survival_47	E-MEXP-278	Pancreatic Ductal Adenocarcinoma	Pancreatic	1.88E-04	-2.31E-01	0.00E+00	0.00E+00
4390	PDAC_Survival_51	E-MEXP-278	Pancreatic Ductal Adenocarcinoma	Pancreatic	4.30E-02	1.40E-01	0.00E+00	0.00E+00
4391	PDAC_Survival_53	E-MEXP-278	Pancreatic Ductal Adenocarcinoma	Pancreatic	5.14E-09	-3.45E-01	0.00E+00	0.00E+00
4392	PDAC_Survival_62	E-MEXP-278	Pancreatic Ductal Adenocarcinoma	Pancreatic	4.71E-12	4.03E-01	0.00E+00	0.00E+00
4393	PDAC_Survival_63	E-MEXP-278	Pancreatic Ductal Adenocarcinoma	Pancreatic	4.62E-11	-3.85E-01	0.00E+00	0.00E+00
4394	PDAC_Survival_65	E-MEXP-278	Pancreatic Ductal Adenocarcinoma	Pancreatic	1.07E-16	-4.79E-01	0.00E+00	0.00E+00
4395	PDAC_Survival_70	E-MEXP-278	Pancreatic Ductal Adenocarcinoma	Pancreatic	5.27E-01	-6.32E-02	0.00E+00	0.00E+00
4396	PDAC_Survival_71	E-MEXP-278	Pancreatic Ductal Adenocarcinoma	Pancreatic	4.32E-01	7.23E-02	0.00E+00	0.00E+00
4397	PDAC_Survival_73	E-MEXP-278	Pancreatic Ductal Adenocarcinoma	Pancreatic	4.82E-06	2.76E-01	0.00E+00	0.00E+00
4398	PDAC_Survival_78	E-MEXP-278	Pancreatic Ductal Adenocarcinoma	Pancreatic	1.82E-15	4.60E-01	0.00E+00	0.00E+00
4399	PDAC_Survival_86	E-MEXP-278	Pancreatic Ductal Adenocarcinoma	Pancreatic	4.03E-10	3.67E-01	0.00E+00	0.00E+00
4400	PDAC_Survival_90	E-MEXP-278	Pancreatic Ductal Adenocarcinoma	Pancreatic	5.82E-02	1.33E-01	0.00E+00	0.00E+00
4401	PDAC_Survival_91	E-MEXP-278	Pancreatic Ductal Adenocarcinoma	Pancreatic	7.41E-02	1.27E-01	0.00E+00	0.00E+00
4402	PDAC_Survival_94	E-MEXP-278	Pancreatic Ductal Adenocarcinoma	Pancreatic	6.82E-02	1.29E-01	0.00E+00	0.00E+00
4403	GSM711904	GSE28735	Pancreatic Ductal Adenocarcinoma	Pancreatic	2.82E-02	-1.49E-01	0.00E+00	0.00E+00
4404	GSM711906	GSE28735	Pancreatic Ductal Adenocarcinoma	Pancreatic	1.24E-01	1.14E-01	0.00E+00	0.00E+00
4405	GSM711908	GSE28735	Pancreatic Ductal Adenocarcinoma	Pancreatic	7.31E-11	-3.81E-01	0.00E+00	0.00E+00
4406	GSM711910	GSE28735	Pancreatic Ductal Adenocarcinoma	Pancreatic	1.36E-25	5.97E-01	0.00E+00	0.00E+00
4407	GSM711912	GSE28735	Pancreatic Ductal Adenocarcinoma	Pancreatic	1.09E-01	-1.17E-01	0.00E+00	0.00E+00
4408	GSM711914	GSE28735	Pancreatic Ductal Adenocarcinoma	Pancreatic	2.03E-01	-9.96E-02	0.00E+00	0.00E+00
4409	GSM711916	GSE28735	Pancreatic Ductal Adenocarcinoma	Pancreatic	2.73E-06	2.82E-01	0.00E+00	0.00E+00
4410	GSM711918	GSE28735	Pancreatic Ductal Adenocarcinoma	Pancreatic	1.63E-02	-1.60E-01	0.00E+00	0.00E+00
4411	GSM711920	GSE28735	Pancreatic Ductal Adenocarcinoma	Pancreatic	6.11E-02	1.32E-01	0.00E+00	0.00E+00
4412	GSM711922	GSE28735	Pancreatic Ductal Adenocarcinoma	Pancreatic	3.55E-01	-8.02E-02	0.00E+00	0.00E+00
4413	GSM711924	GSE28735	Pancreatic Ductal Adenocarcinoma	Pancreatic	1.07E-02	1.68E-01	0.00E+00	0.00E+00
4414	GSM711926	GSE28735	Pancreatic Ductal Adenocarcinoma	Pancreatic	6.49E-03	-1.77E-01	0.00E+00	0.00E+00
4415	GSM711928	GSE28735	Pancreatic Ductal Adenocarcinoma	Pancreatic	1.61E-01	-1.07E-01	0.00E+00	0.00E+00
4416	GSM711930	GSE28735	Pancreatic Ductal Adenocarcinoma	Pancreatic	6.98E-05	2.44E-01	0.00E+00	0.00E+00
4417	GSM711932	GSE28735	Pancreatic Ductal Adenocarcinoma	Pancreatic	3.10E-01	8.53E-02	0.00E+00	0.00E+00
4418	GSM711934	GSE28735	Pancreatic Ductal Adenocarcinoma	Pancreatic	8.12E-04	2.10E-01	0.00E+00	0.00E+00
4419	GSM711936	GSE28735	Pancreatic Ductal Adenocarcinoma	Pancreatic	2.51E-01	-9.28E-02	0.00E+00	0.00E+00
4420	GSM711938	GSE28735	Pancreatic Ductal Adenocarcinoma	Pancreatic	1.64E-01	-1.06E-01	0.00E+00	0.00E+00
4421	GSM711940	GSE28735	Pancreatic Ductal Adenocarcinoma	Pancreatic	2.27E-06	2.84E-01	0.00E+00	0.00E+00
4422	GSM711942	GSE28735	Pancreatic Ductal Adenocarcinoma	Pancreatic	1.12E-01	-1.17E-01	0.00E+00	0.00E+00
4423	GSM711944	GSE28735	Pancreatic Ductal Adenocarcinoma	Pancreatic	4.86E-19	5.12E-01	0.00E+00	0.00E+00
4424	GSM711946	GSE28735	Pancreatic Ductal Adenocarcinoma	Pancreatic	5.14E-11	3.84E-01	0.00E+00	0.00E+00
4425	GSM711948	GSE28735	Pancreatic Ductal Adenocarcinoma	Pancreatic	1.08E-05	2.67E-01	0.00E+00	0.00E+00
4426	GSM711950	GSE28735	Pancreatic Ductal Adenocarcinoma	Pancreatic	2.57E-03	1.93E-01	0.00E+00	0.00E+00
4427	GSM711952	GSE28735	Pancreatic Ductal Adenocarcinoma	Pancreatic	1.58E-07	-3.12E-01	0.00E+00	0.00E+00
4428	GSM711954	GSE28735	Pancreatic Ductal Adenocarcinoma	Pancreatic	2.16E-01	-9.77E-02	0.00E+00	0.00E+00
4429	GSM711956	GSE28735	Pancreatic Ductal Adenocarcinoma	Pancreatic	4.32E-01	7.23E-02	0.00E+00	0.00E+00
4430	GSM711958	GSE28735	Pancreatic Ductal Adenocarcinoma	Pancreatic	2.95E-06	2.81E-01	0.00E+00	0.00E+00
4431	GSM711960	GSE28735	Pancreatic Ductal Adenocarcinoma	Pancreatic	2.45E-10	3.71E-01	0.00E+00	0.00E+00
4432	GSM711962	GSE28735	Pancreatic Ductal Adenocarcinoma	Pancreatic	9.89E-04	2.07E-01	0.00E+00	0.00E+00
4433	GSM711964	GSE28735	Pancreatic Ductal Adenocarcinoma	Pancreatic	1.26E-01	-1.13E-01	0.00E+00	0.00E+00
4434	GSM711966	GSE28735	Pancreatic Ductal Adenocarcinoma	Pancreatic	7.11E-04	2.12E-01	0.00E+00	0.00E+00
4435	GSM711968	GSE28735	Pancreatic Ductal Adenocarcinoma	Pancreatic	2.51E-02	-1.51E-01	0.00E+00	0.00E+00

4436	GSM711970	GSE28735	Pancreatic Ductal Adenocarcinoma	Pancreatic	2.57E-01	9.19E-02	0.00E+00	0.00E+00
4437	GSM711972	GSE28735	Pancreatic Ductal Adenocarcinoma	Pancreatic	4.04E-02	1.41E-01	0.00E+00	0.00E+00
4438	GSM711974	GSE28735	Pancreatic Ductal Adenocarcinoma	Pancreatic	3.56E-06	2.79E-01	0.00E+00	0.00E+00
4439	GSM711976	GSE28735	Pancreatic Ductal Adenocarcinoma	Pancreatic	7.03E-02	1.29E-01	0.00E+00	0.00E+00
4440	GSM711978	GSE28735	Pancreatic Ductal Adenocarcinoma	Pancreatic	2.12E-01	9.82E-02	0.00E+00	0.00E+00
4441	GSM711980	GSE28735	Pancreatic Ductal Adenocarcinoma	Pancreatic	1.77E-01	1.04E-01	0.00E+00	0.00E+00
4442	GSM711982	GSE28735	Pancreatic Ductal Adenocarcinoma	Pancreatic	7.78E-06	2.70E-01	0.00E+00	0.00E+00
4443	GSM711984	GSE28735	Pancreatic Ductal Adenocarcinoma	Pancreatic	3.51E-01	-8.07E-02	0.00E+00	0.00E+00
4444	GSM711986	GSE28735	Pancreatic Ductal Adenocarcinoma	Pancreatic	1.12E-03	-2.06E-01	0.00E+00	0.00E+00
4445	GSM711988	GSE28735	Pancreatic Ductal Adenocarcinoma	Pancreatic	2.52E-02	1.51E-01	0.00E+00	0.00E+00
4446	GSM711990	GSE28735	Pancreatic Ductal Adenocarcinoma	Pancreatic	3.33E-18	5.00E-01	0.00E+00	0.00E+00
4447	GSM711992	GSE28735	Pancreatic Ductal Adenocarcinoma	Pancreatic	3.39E-01	8.21E-02	0.00E+00	0.00E+00
4448	GSM449147	GSE17951	Prostate	Prostate	1.01E-25	6.00E-01	7.38E-12	8.22E-01
4449	GSM449148	GSE17951	Prostate	Prostate	1.01E-25	6.00E-01	1.04E-12	8.52E-01
4450	GSM449149	GSE17951	Prostate	Prostate	7.96E-05	2.43E-01	6.56E-04	4.39E-01
4451	GSM449150	GSE17951	Prostate	Prostate	1.16E-04	-2.38E-01	2.51E-02	-3.11E-01
4452	GSM449151	GSE17951	Prostate	Prostate	7.67E-03	-1.75E-01	1.35E-01	2.30E-01
4453	GSM449152	GSE17951	Prostate	Prostate	1.50E-06	2.90E-01	3.73E-06	5.74E-01
4454	GSM449153	GSE17951	Prostate	Prostate	5.78E-01	-5.85E-02	2.23E-01	1.99E-01
4455	GSM449154	GSE17951	Prostate	Prostate	3.62E-05	-2.53E-01	9.02E-03	-3.52E-01
4456	GSM449155	GSE17951	Prostate	Prostate	2.86E-03	-1.91E-01	2.30E-01	-1.97E-01
4457	GSM449156	GSE17951	Prostate	Prostate	2.59E-06	2.84E-01	3.57E-05	5.19E-01
4458	GSM449157	GSE17951	Prostate	Prostate	1.39E-01	-1.11E-01	1.73E-01	2.15E-01
4459	GSM449158	GSE17951	Prostate	Prostate	2.52E-02	1.52E-01	2.93E-02	3.05E-01
4460	GSM449159	GSE17951	Prostate	Prostate	1.96E-14	4.44E-01	8.12E-08	6.56E-01
4461	GSM449160	GSE17951	Prostate	Prostate	1.12E-02	-1.68E-01	1.57E-01	-2.21E-01
4462	GSM449161	GSE17951	Prostate	Prostate	1.45E-30	6.56E-01	3.93E-14	9.02E-01
4463	GSM449162	GSE17951	Prostate	Prostate	2.42E-01	-9.42E-02	1.35E-01	2.30E-01
4464	GSM449163	GSE17951	Prostate	Prostate	5.30E-04	2.17E-01	7.16E-03	3.61E-01
4465	GSM449164	GSE17951	Prostate	Prostate	1.53E-02	-1.62E-01	2.68E-01	1.86E-01
4466	GSM449165	GSE17951	Prostate	Prostate	1.70E-02	1.60E-01	3.27E-03	3.88E-01
4467	GSM449166	GSE17951	Prostate	Prostate	2.29E-02	-1.54E-01	8.45E-02	2.55E-01
4468	GSM449167	GSE17951	Prostate	Prostate	1.75E-10	3.75E-01	1.38E-05	5.43E-01
4469	GSM449168	GSE17951	Prostate	Prostate	1.39E-01	-1.11E-01	7.05E-02	2.64E-01
4470	GSM449169	GSE17951	Prostate	Prostate	3.01E-07	3.07E-01	1.38E-05	5.43E-01
4471	GSM449170	GSE17951	Prostate	Prostate	2.18E-02	1.55E-01	1.11E-02	3.44E-01
4472	GSM449171	GSE17951	Prostate	Prostate	2.52E-02	1.52E-01	2.26E-04	4.70E-01
4473	GSM449172	GSE17951	Prostate	Prostate	5.47E-03	1.81E-01	7.83E-03	3.57E-01
4474	GSM449173	GSE17951	Prostate	Prostate	5.68E-04	-2.16E-01	6.26E-02	-2.70E-01
4475	GSM449174	GSE17951	Prostate	Prostate	1.47E-05	2.64E-01	1.16E-04	4.89E-01
4476	GSM449175	GSE17951	Prostate	Prostate	1.18E-02	-1.67E-01	1.73E-01	-2.15E-01
4477	GSM449176	GSE17951	Prostate	Prostate	7.67E-03	-1.75E-01	2.23E-01	1.99E-01
4478	GSM449177	GSE17951	Prostate	Prostate	7.97E-04	-2.11E-01	1.94E-01	-2.08E-01
4479	GSM449178	GSE17951	Prostate	Prostate	3.84E-01	7.74E-02	4.30E-02	2.88E-01
4480	GSM449179	GSE17951	Prostate	Prostate	3.62E-11	3.88E-01	6.93E-06	5.59E-01
4481	GSM449180	GSE17951	Prostate	Prostate	1.53E-02	-1.62E-01	2.91E-01	-1.80E-01
4482	GSM449181	GSE17951	Prostate	Prostate	6.21E-01	5.46E-02	1.43E-01	2.26E-01
4483	GSM449182	GSE17951	Prostate	Prostate	4.73E-01	6.85E-02	7.34E-02	2.62E-01
4484	GSM449183	GSE17951	Prostate	Prostate	9.26E-02	-1.22E-01	1.48E-01	2.24E-01
4485	GSM449184	GSE17951	Prostate	Prostate	3.21E-01	8.43E-02	2.29E-02	3.15E-01
4486	GSM449185	GSE17951	Prostate	Prostate	1.65E-06	-2.89E-01	4.28E-03	-3.79E-01
4487	GSM449186	GSE17951	Prostate	Prostate	1.11E-03	-2.06E-01	5.61E-02	-2.75E-01
4488	GSM449187	GSE17951	Prostate	Prostate	1.12E-01	1.17E-01	2.97E-02	3.04E-01
4489	GSM449188	GSE17951	Prostate	Prostate	6.75E-01	4.96E-02	1.15E-01	2.39E-01
4490	GSM449189	GSE17951	Prostate	Prostate	2.40E-02	-1.53E-01	3.60E-01	-1.64E-01
4491	GSM449190	GSE17951	Prostate	Prostate	3.25E-04	-2.24E-01	9.00E-02	-2.52E-01
4492	GSM449191	GSE17951	Prostate	Prostate	1.97E-03	-1.97E-01	1.82E-01	-2.12E-01
4493	GSM449192	GSE17951	Prostate	Prostate	4.20E-02	-1.41E-01	2.49E-01	1.91E-01
4494	GSM449193	GSE17951	Prostate	Prostate	2.52E-02	1.52E-01	1.34E-02	3.37E-01
4495	GSM449194	GSE17951	Prostate	Prostate	8.10E-03	1.74E-01	3.67E-02	2.95E-01
4496	GSM449195	GSE17951	Prostate	Prostate	9.73E-04	-2.08E-01	1.76E-01	-2.14E-01
4497	GSM449196	GSE17951	Prostate	Prostate	1.21E-01	-1.15E-01	2.46E-01	-1.92E-01
4498	GSM449197	GSE17951	Prostate	Prostate	1.54E-01	1.08E-01	2.42E-01	1.93E-01
4499	GSM449198	GSE17951	Prostate	Prostate	1.46E-02	-1.63E-01	2.81E-01	-1.83E-01
4500	GSM449199	GSE17951	Prostate	Prostate	1.21E-01	1.15E-01	2.29E-02	3.15E-01
4501	GSM449200	GSE17951	Prostate	Prostate	3.56E-01	-8.04E-02	1.67E-01	2.17E-01
4502	GSM449201	GSE17951	Prostate	Prostate	3.75E-01	-7.84E-02	2.84E-02	3.06E-01
4503	GSM449202	GSE17951	Prostate	Prostate	1.38E-02	-1.64E-01	2.30E-01	-1.97E-01
4504	GSM449203	GSE17951	Prostate	Prostate	5.23E-02	-1.36E-01	2.91E-01	1.80E-01
4505	GSM449204	GSE17951	Prostate	Prostate	5.94E-02	1.33E-01	3.47E-03	3.86E-01
4506	GSM449205	GSE17951	Prostate	Prostate	2.10E-03	1.96E-01	5.50E-04	4.45E-01
4507	GSM449206	GSE17951	Prostate	Prostate	3.84E-02	1.43E-01	5.61E-02	2.75E-01
4508	GSM449207	GSE17951	Prostate	Prostate	1.30E-01	1.13E-01	4.12E-02	2.90E-01
4509	GSM449208	GSE17951	Prostate	Prostate	2.72E-01	9.03E-02	1.35E-01	2.30E-01

4510	GSM449209	GSE17951	Prostate	Prostate	6.19E-02	-1.32E-01	2.96E-01	-1.79E-01
4511	GSM449210	GSE17951	Prostate	Prostate	4.79E-02	-1.38E-01	2.53E-01	-1.90E-01
4512	GSM449211	GSE17951	Prostate	Prostate	1.81E-06	2.88E-01	2.49E-03	3.97E-01
4513	GSM449212	GSE17951	Prostate	Prostate	8.24E-02	1.25E-01	4.12E-02	2.90E-01
4514	GSM449213	GSE17951	Prostate	Prostate	5.68E-01	-5.95E-02	1.35E-01	2.30E-01
4515	GSM449214	GSE17951	Prostate	Prostate	3.62E-05	2.53E-01	2.43E-04	4.68E-01
4516	GSM449215	GSE17951	Prostate	Prostate	2.62E-04	-2.27E-01	1.38E-01	-2.28E-01
4517	GSM449216	GSE17951	Prostate	Prostate	1.83E-23	5.72E-01	1.06E-09	7.38E-01
4518	GSM449217	GSE17951	Prostate	Prostate	6.00E-14	4.37E-01	1.65E-07	6.41E-01
4519	GSM449218	GSE17951	Prostate	Prostate	2.64E-02	-1.51E-01	3.12E-01	1.75E-01
4520	GSM449219	GSE17951	Prostate	Prostate	6.08E-04	2.15E-01	7.16E-03	3.61E-01
4521	GSM449220	GSE17951	Prostate	Prostate	3.34E-02	-1.46E-01	3.60E-01	1.64E-01
4522	GSM449221	GSE17951	Prostate	Prostate	6.00E-01	-5.65E-02	3.20E-01	1.73E-01
4523	GSM449222	GSE17951	Prostate	Prostate	5.79E-03	-1.80E-01	1.34E-01	-2.30E-01
4524	GSM449223	GSE17951	Prostate	Prostate	1.12E-01	1.17E-01	1.31E-01	2.31E-01
4525	GSM449224	GSE17951	Prostate	Prostate	4.79E-02	-1.38E-01	1.73E-01	2.15E-01
4526	GSM449225	GSE17951	Prostate	Prostate	1.50E-06	-2.90E-01	1.83E-02	-3.25E-01
4527	GSM449226	GSE17951	Prostate	Prostate	3.19E-02	1.47E-01	1.31E-01	2.31E-01
4528	GSM449227	GSE17951	Prostate	Prostate	2.38E-03	-1.94E-01	1.38E-01	-2.28E-01
4529	GSM449228	GSE17951	Prostate	Prostate	2.28E-01	-9.62E-02	7.53E-02	2.61E-01
4530	GSM449229	GSE17951	Prostate	Prostate	7.97E-04	-2.11E-01	1.18E-01	-2.37E-01
4531	GSM449230	GSE17951	Prostate	Prostate	3.86E-03	-1.87E-01	9.70E-02	-2.48E-01
4532	GSM449231	GSE17951	Prostate	Prostate	1.53E-03	-2.01E-01	7.83E-02	-2.59E-01
4533	GSM449232	GSE17951	Prostate	Prostate	2.88E-01	-8.83E-02	6.09E-02	2.71E-01
4534	GSM449233	GSE17951	Prostate	Prostate	1.30E-01	1.13E-01	1.69E-02	3.28E-01
4535	GSM449234	GSE17951	Prostate	Prostate	1.60E-05	2.63E-01	3.57E-05	5.19E-01
4536	GSM449235	GSE17951	Prostate	Prostate	4.33E-01	-7.24E-02	1.22E-01	2.35E-01
4537	GSM449236	GSE17951	Prostate	Prostate	7.60E-02	-1.27E-01	3.94E-01	1.57E-01
4538	GSM449237	GSE17951	Prostate	Prostate	8.91E-02	-1.23E-01	2.30E-01	1.97E-01
4539	GSM449238	GSE17951	Prostate	Prostate	1.02E-07	3.17E-01	1.25E-04	4.87E-01
4540	GSM449239	GSE17951	Prostate	Prostate	8.01E-25	5.89E-01	2.29E-11	8.03E-01
4541	GSM449240	GSE17951	Prostate	Prostate	1.05E-05	2.68E-01	1.34E-04	4.85E-01
4542	GSM449241	GSE17951	Prostate	Prostate	1.00E-09	3.60E-01	6.53E-07	6.12E-01
4543	GSM449242	GSE17951	Prostate	Prostate	1.78E-30	6.55E-01	3.55E-11	7.96E-01
4544	GSM449243	GSE17951	Prostate	Prostate	1.16E-04	-2.38E-01	7.83E-02	-2.59E-01
4545	GSM449244	GSE17951	Prostate	Prostate	6.86E-03	-1.77E-01	1.06E-01	-2.43E-01
4546	GSM449245	GSE17951	Prostate	Prostate	2.57E-01	9.23E-02	2.49E-01	1.91E-01
4547	GSM449246	GSE17951	Prostate	Prostate	1.01E-02	1.70E-01	2.51E-02	3.11E-01
4548	GSM449247	GSE17951	Prostate	Prostate	2.28E-01	-9.62E-02	7.05E-02	2.64E-01
4549	GSM449248	GSE17951	Prostate	Prostate	2.24E-03	-1.95E-01	2.04E-01	-2.05E-01
4550	GSM449249	GSE17951	Prostate	Prostate	1.70E-02	-1.60E-01	4.64E-01	-1.42E-01
4551	GSM449250	GSE17951	Prostate	Prostate	7.92E-02	-1.26E-01	6.79E-01	-1.01E-01
4552	GSM449251	GSE17951	Prostate	Prostate	4.20E-02	-1.41E-01	1.73E-01	2.15E-01
4553	GSM449252	GSE17951	Prostate	Prostate	2.57E-01	9.23E-02	1.35E-01	2.30E-01
4554	GSM449253	GSE17951	Prostate	Prostate	1.82E-04	2.32E-01	2.43E-04	4.68E-01
4555	GSM449254	GSE17951	Prostate	Prostate	3.32E-07	3.06E-01	1.02E-05	5.50E-01
4556	GSM449255	GSE17951	Prostate	Prostate	4.13E-01	-7.44E-02	1.78E-01	2.13E-01
4557	GSM449256	GSE17951	Prostate	Prostate	1.95E-01	-1.01E-01	2.91E-01	1.80E-01
4558	GSM449257	GSE17951	Prostate	Prostate	4.01E-02	-1.42E-01	3.26E-01	-1.72E-01
4559	GSM449258	GSE17951	Prostate	Prostate	6.19E-02	-1.32E-01	2.91E-01	-1.80E-01
4560	GSM449259	GSE17951	Prostate	Prostate	2.96E-01	-8.73E-02	1.67E-01	2.17E-01
4561	GSM449260	GSE17951	Prostate	Prostate	3.05E-02	-1.48E-01	3.26E-01	-1.72E-01
4562	GSM449261	GSE17951	Prostate	Prostate	4.94E-01	6.65E-02	9.70E-02	2.48E-01
4563	GSM449262	GSE17951	Prostate	Prostate	4.43E-01	7.14E-02	6.09E-02	2.71E-01
4564	GSM449263	GSE17951	Prostate	Prostate	1.54E-01	-1.08E-01	4.82E-02	2.83E-01
4565	GSM449264	GSE17951	Prostate	Prostate	3.21E-01	8.43E-02	2.17E-01	2.01E-01
4566	GSM449265	GSE17951	Prostate	Prostate	1.53E-02	-1.62E-01	2.76E-01	-1.84E-01
4567	GSM449266	GSE17951	Prostate	Prostate	9.54E-03	1.71E-01	4.28E-03	3.79E-01
4568	GSM449267	GSE17951	Prostate	Prostate	1.71E-01	1.05E-01	4.82E-02	2.83E-01
4569	GSM449268	GSE17951	Prostate	Prostate	6.73E-02	-1.30E-01	3.58E-01	-1.65E-01
4570	GSM449269	GSE17951	Prostate	Prostate	2.07E-02	-1.56E-01	2.35E-01	-1.95E-01
4571	GSM449270	GSE17951	Prostate	Prostate	6.46E-02	1.31E-01	2.29E-02	3.15E-01
4572	GSM449271	GSE17951	Prostate	Prostate	2.24E-03	-1.95E-01	2.56E-01	1.90E-01
4573	GSM449272	GSE17951	Prostate	Prostate	5.27E-28	6.27E-01	5.72E-13	8.62E-01
4574	GSM449273	GSE17951	Prostate	Prostate	1.02E-07	3.17E-01	2.88E-06	5.80E-01
4575	GSM449274	GSE17951	Prostate	Prostate	5.64E-10	-3.65E-01	2.35E-03	-3.99E-01
4576	GSM449275	GSE17951	Prostate	Prostate	1.30E-01	1.13E-01	3.51E-02	2.97E-01
4577	GSM449276	GSE17951	Prostate	Prostate	9.63E-02	-1.21E-01	2.49E-01	1.91E-01
4578	GSM449277	GSE17951	Prostate	Prostate	2.01E-01	-1.00E-01	1.01E-01	2.46E-01
4579	GSM449278	GSE17951	Prostate	Prostate	5.91E-11	3.84E-01	2.49E-06	5.83E-01
4580	GSM449279	GSE17951	Prostate	Prostate	1.16E-01	-1.16E-01	1.78E-01	2.13E-01
4581	GSM449280	GSE17951	Prostate	Prostate	2.29E-02	1.54E-01	4.24E-02	2.89E-01
4582	GSM449281	GSE17951	Prostate	Prostate	8.56E-03	-1.73E-01	1.01E-01	2.46E-01
4583	GSM449282	GSE17951	Prostate	Prostate	7.30E-02	-1.28E-01	1.35E-01	2.30E-01

4584	GSM449283	GSE17951	Prostate	Prostate	4.33E-01	-7.24E-02	2.29E-02	3.15E-01
4585	GSM449284	GSE17951	Prostate	Prostate	8.91E-02	1.23E-01	3.21E-02	3.01E-01
4586	GSM449285	GSE17951	Prostate	Prostate	1.69E-04	-2.33E-01	1.34E-01	-2.30E-01
4587	GSM449286	GSE17951	Prostate	Prostate	2.72E-01	-9.03E-02	1.53E-01	2.22E-01
4588	GSM449287	GSE17951	Prostate	Prostate	4.63E-01	-6.94E-02	2.00E-01	2.06E-01
4589	GSM449288	GSE17951	Prostate	Prostate	2.21E-01	9.72E-02	1.51E-01	2.23E-01
4590	GSM449289	GSE17951	Prostate	Prostate	4.20E-02	1.41E-01	2.51E-02	3.11E-01
4591	GSM449290	GSE17951	Prostate	Prostate	7.60E-02	-1.27E-01	1.73E-01	2.15E-01
4592	GSM449291	GSE17951	Prostate	Prostate	7.92E-02	-1.26E-01	3.60E-01	1.64E-01
4593	GSM449292	GSE17951	Prostate	Prostate	5.30E-04	-2.17E-01	1.78E-01	2.13E-01
4594	GSM449293	GSE17951	Prostate	Prostate	5.46E-01	-6.15E-02	1.62E-01	2.19E-01
4595	GSM449294	GSE17951	Prostate	Prostate	1.14E-05	-2.67E-01	1.83E-02	-3.25E-01
4596	GSM449295	GSE17951	Prostate	Prostate	3.15E-10	3.70E-01	8.65E-06	5.54E-01
4597	GSM449296	GSE17951	Prostate	Prostate	1.00E-09	3.60E-01	1.02E-05	5.50E-01
4598	GSM449297	GSE17951	Prostate	Prostate	5.75E-06	2.75E-01	8.71E-03	3.53E-01
4599	GSM449298	GSE17951	Prostate	Prostate	8.62E-17	4.81E-01	1.52E-08	6.89E-01
4600	GSM449299	GSE17951	Prostate	Prostate	1.34E-01	1.12E-01	2.02E-02	3.21E-01
4601	GSM449300	GSE17951	Prostate	Prostate	2.44E-04	2.28E-01	1.70E-03	4.10E-01
4602	GSM160343	GSE6956	Prostate	Prostate	6.48E-03	-1.78E-01	2.88E-01	-1.81E-01
4603	GSM160344	GSE6956	Prostate	Prostate	1.41E-09	-3.57E-01	1.75E-04	-4.77E-01
4604	GSM160345	GSE6956	Prostate	Prostate	3.66E-01	7.94E-02	2.51E-02	3.11E-01
4605	GSM160346	GSE6956	Prostate	Prostate	3.30E-01	8.33E-02	1.69E-02	3.28E-01
4606	GSM160347	GSE6956	Prostate	Prostate	1.30E-01	-1.13E-01	2.91E-01	1.80E-01
4607	GSM160348	GSE6956	Prostate	Prostate	3.84E-02	-1.43E-01	1.01E-01	2.46E-01
4608	GSM160349	GSE6956	Prostate	Prostate	2.91E-12	-4.08E-01	1.88E-03	-4.07E-01
4609	GSM160350	GSE6956	Prostate	Prostate	3.62E-05	-2.53E-01	5.61E-02	-2.75E-01
4610	GSM160351	GSE6956	Prostate	Prostate	4.28E-12	4.05E-01	3.42E-06	5.76E-01
4611	GSM160352	GSE6956	Prostate	Prostate	8.86E-06	-2.70E-01	1.92E-02	-3.23E-01
4612	GSM160353	GSE6956	Prostate	Prostate	7.67E-03	1.75E-01	3.60E-03	3.85E-01
4613	GSM160354	GSE6956	Prostate	Prostate	6.84E-06	2.73E-01	1.51E-04	4.81E-01
4614	GSM160355	GSE6956	Prostate	Prostate	5.16E-03	1.82E-01	6.43E-03	3.65E-01
4615	GSM160356	GSE6956	Prostate	Prostate	1.21E-01	1.15E-01	2.02E-02	3.21E-01
4616	GSM160357	GSE6956	Prostate	Prostate	3.62E-05	2.53E-01	3.86E-05	5.17E-01
4617	GSM160358	GSE6956	Prostate	Prostate	7.45E-04	-2.12E-01	1.43E-01	-2.26E-01
4618	GSM160359	GSE6956	Prostate	Prostate	8.52E-04	-2.10E-01	3.21E-02	-3.01E-01
4619	GSM160360	GSE6956	Prostate	Prostate	1.95E-01	-1.01E-01	2.91E-01	1.80E-01
4620	GSM160361	GSE6956	Prostate	Prostate	8.91E-02	1.23E-01	2.97E-02	3.04E-01
4621	GSM160362	GSE6956	Prostate	Prostate	1.70E-02	-1.60E-01	1.78E-01	2.13E-01
4622	GSM160363	GSE6956	Prostate	Prostate	8.24E-02	-1.25E-01	2.91E-01	1.80E-01
4623	GSM160364	GSE6956	Prostate	Prostate	3.20E-11	3.89E-01	8.57E-07	6.07E-01
4624	GSM160365	GSE6956	Prostate	Prostate	1.04E-01	-1.19E-01	3.12E-01	1.75E-01
4625	GSM160366	GSE6956	Prostate	Prostate	7.92E-02	1.26E-01	9.35E-02	2.50E-01
4626	GSM160367	GSE6956	Prostate	Prostate	2.77E-02	-1.50E-01	2.68E-01	-1.86E-01
4627	GSM160368	GSE6956	Prostate	Prostate	2.08E-01	9.92E-02	7.16E-03	3.61E-01
4628	GSM160369	GSE6956	Prostate	Prostate	1.26E-03	-2.04E-01	2.30E-01	1.97E-01
4629	GSM160370	GSE6956	Prostate	Prostate	2.10E-03	1.96E-01	1.01E-03	4.26E-01
4630	GSM160371	GSE6956	Prostate	Prostate	1.85E-03	-1.98E-01	3.20E-01	1.73E-01
4631	GSM160372	GSE6956	Prostate	Prostate	1.11E-03	-2.06E-01	4.62E-02	-2.85E-01
4632	GSM160373	GSE6956	Prostate	Prostate	2.10E-03	1.96E-01	1.69E-02	3.28E-01
4633	GSM160374	GSE6956	Prostate	Prostate	1.31E-02	-1.65E-01	3.60E-01	1.64E-01
4634	GSM160375	GSE6956	Prostate	Prostate	2.72E-31	6.64E-01	6.43E-14	8.94E-01
4635	GSM160376	GSE6956	Prostate	Prostate	2.72E-01	-9.03E-02	2.96E-01	1.79E-01
4636	GSM160377	GSE6956	Prostate	Prostate	1.26E-03	-2.04E-01	1.01E-01	-2.46E-01
4637	GSM160378	GSE6956	Prostate	Prostate	6.11E-01	5.56E-02	1.53E-01	2.22E-01
4638	GSM160379	GSE6956	Prostate	Prostate	3.19E-02	-1.47E-01	1.92E-01	-2.09E-01
4639	GSM160380	GSE6956	Prostate	Prostate	3.49E-04	-2.23E-01	8.14E-02	-2.57E-01
4640	GSM160381	GSE6956	Prostate	Prostate	4.87E-03	-1.83E-01	1.94E-01	-2.08E-01
4641	GSM160382	GSE6956	Prostate	Prostate	1.88E-02	-1.58E-01	5.20E-01	-1.31E-01
4642	GSM160383	GSE6956	Prostate	Prostate	3.56E-01	-8.04E-02	1.35E-01	2.30E-01
4643	GSM160384	GSE6956	Prostate	Prostate	7.92E-02	1.26E-01	1.06E-02	3.46E-01
4644	GSM160385	GSE6956	Prostate	Prostate	4.31E-04	2.20E-01	3.35E-04	4.59E-01
4645	GSM160386	GSE6956	Prostate	Prostate	3.84E-01	7.74E-02	1.01E-01	2.46E-01
4646	GSM160387	GSE6956	Prostate	Prostate	2.21E-09	3.53E-01	1.65E-07	6.41E-01
4647	GSM160388	GSE6956	Prostate	Prostate	2.62E-04	2.27E-01	2.00E-03	4.05E-01
4648	GSM160389	GSE6956	Prostate	Prostate	3.34E-02	1.46E-01	3.67E-02	2.95E-01
4649	GSM160390	GSE6956	Prostate	Prostate	7.25E-03	1.76E-01	6.79E-03	3.63E-01
4650	GSM160391	GSE6956	Prostate	Prostate	1.06E-02	1.69E-01	1.60E-03	4.12E-01
4651	GSM160392	GSE6956	Prostate	Prostate	4.87E-03	-1.83E-01	1.82E-01	-2.12E-01
4652	GSM160393	GSE6956	Prostate	Prostate	1.61E-02	-1.61E-01	1.78E-01	-2.13E-01
4653	GSM160394	GSE6956	Prostate	Prostate	3.66E-01	-7.94E-02	2.91E-01	1.80E-01
4654	GSM160395	GSE6956	Prostate	Prostate	2.29E-02	-1.54E-01	2.23E-01	1.99E-01
4655	GSM160396	GSE6956	Prostate	Prostate	8.30E-08	3.19E-01	4.82E-04	4.49E-01
4656	GSM160397	GSE6956	Prostate	Prostate	1.30E-01	-1.13E-01	6.09E-02	2.71E-01
4657	GSM160398	GSE6956	Prostate	Prostate	6.13E-03	1.79E-01	5.03E-02	2.81E-01

4658	GSM160399	GSE6956	Prostate	Prostate	3.34E-05	-2.54E-01	5.85E-02	-2.73E-01
4659	GSM160400	GSE6956	Prostate	Prostate	1.21E-01	-1.15E-01	3.94E-01	1.57E-01
4660	GSM160401	GSE6956	Prostate	Prostate	6.13E-03	-1.79E-01	3.66E-01	-1.63E-01
4661	GSM160403	GSE6956	Prostate	Prostate	1.95E-01	1.01E-01	6.09E-02	2.71E-01
4662	GSM160405	GSE6956	Prostate	Prostate	1.44E-01	1.10E-01	4.30E-02	2.88E-01
4663	GSM160406	GSE6956	Prostate	Prostate	1.99E-38	7.37E-01	2.09E-14	9.11E-01
4664	GSM160408	GSE6956	Prostate	Prostate	1.08E-01	1.18E-01	1.57E-01	2.21E-01
4665	GSM160410	GSE6956	Prostate	Prostate	5.25E-01	6.35E-02	2.04E-01	2.05E-01
4666	GSM160412	GSE6956	Prostate	Prostate	1.35E-03	2.03E-01	1.61E-02	3.30E-01
4667	GSM160413	GSE6956	Prostate	Prostate	4.60E-05	2.50E-01	1.34E-04	4.85E-01
4668	GSM160414	GSE6956	Prostate	Prostate	1.98E-06	2.87E-01	4.31E-04	4.52E-01
4669	GSM160416	GSE6956	Prostate	Prostate	4.05E-08	3.26E-01	3.13E-04	4.61E-01
4670	GSM160417	GSE6956	Prostate	Prostate	3.84E-02	1.43E-01	2.80E-02	3.07E-01
4671	GSM203261	GSE8218	Prostate	Prostate	9.54E-03	-1.71E-01	1.15E-01	2.39E-01
4672	GSM203262	GSE8218	Prostate	Prostate	8.57E-02	1.24E-01	1.34E-02	3.37E-01
4673	GSM203263	GSE8218	Prostate	Prostate	5.39E-05	2.48E-01	3.13E-04	4.61E-01
4674	GSM203264	GSE8218	Prostate	Prostate	1.46E-02	-1.63E-01	5.20E-01	1.31E-01
4675	GSM203265	GSE8218	Prostate	Prostate	6.28E-06	-2.74E-01	2.59E-02	-3.10E-01
4676	GSM203266	GSE8218	Prostate	Prostate	6.28E-06	2.74E-01	1.27E-03	4.19E-01
4677	GSM203267	GSE8218	Prostate	Prostate	3.49E-04	-2.23E-01	1.07E-01	-2.43E-01
4678	GSM203268	GSE8218	Prostate	Prostate	5.52E-08	3.23E-01	1.49E-05	5.41E-01
4679	GSM203269	GSE8218	Prostate	Prostate	1.57E-13	4.30E-01	5.11E-07	6.18E-01
4680	GSM203270	GSE8218	Prostate	Prostate	2.08E-01	9.92E-02	2.29E-02	3.15E-01
4681	GSM203271	GSE8218	Prostate	Prostate	1.57E-13	4.30E-01	7.15E-07	6.10E-01
4682	GSM203272	GSE8218	Prostate	Prostate	1.89E-01	1.02E-01	2.29E-02	3.15E-01
4683	GSM203273	GSE8218	Prostate	Prostate	1.79E-02	-1.59E-01	1.78E-01	2.13E-01
4684	GSM203274	GSE8218	Prostate	Prostate	1.63E-03	-2.00E-01	5.24E-02	2.79E-01
4685	GSM203275	GSE8218	Prostate	Prostate	1.44E-01	1.10E-01	2.97E-02	3.04E-01
4686	GSM203276	GSE8218	Prostate	Prostate	6.48E-03	1.78E-01	5.88E-04	4.43E-01
4687	GSM203277	GSE8218	Prostate	Prostate	2.07E-02	-1.56E-01	1.01E-01	2.46E-01
4688	GSM203278	GSE8218	Prostate	Prostate	1.25E-01	-1.14E-01	2.30E-01	-1.97E-01
4689	GSM203279	GSE8218	Prostate	Prostate	4.02E-04	2.21E-01	8.89E-04	4.30E-01
4690	GSM203280	GSE8218	Prostate	Prostate	5.16E-03	1.82E-01	7.43E-03	3.59E-01
4691	GSM203281	GSE8218	Prostate	Prostate	1.12E-01	-1.17E-01	2.91E-01	1.80E-01
4692	GSM203282	GSE8218	Prostate	Prostate	1.49E-01	-1.09E-01	1.35E-01	2.30E-01
4693	GSM203284	GSE8218	Prostate	Prostate	2.01E-01	1.00E-01	2.40E-02	3.13E-01
4694	GSM203285	GSE8218	Prostate	Prostate	1.85E-17	4.91E-01	8.67E-08	6.54E-01
4695	GSM203286	GSE8218	Prostate	Prostate	1.96E-04	2.31E-01	5.50E-04	4.45E-01
4696	GSM203287	GSE8218	Prostate	Prostate	2.14E-01	-9.82E-02	9.00E-02	2.52E-01
4697	GSM203288	GSE8218	Prostate	Prostate	9.73E-04	2.08E-01	2.12E-03	4.03E-01
4698	GSM203289	GSE8218	Prostate	Prostate	2.42E-01	9.42E-02	2.02E-02	3.21E-01
4699	GSM203290	GSE8218	Prostate	Prostate	7.84E-07	2.97E-01	6.93E-06	5.59E-01
4700	GSM203291	GSE8218	Prostate	Prostate	2.77E-02	-1.50E-01	1.35E-01	2.30E-01
4701	GSM203292	GSE8218	Prostate	Prostate	2.84E-05	2.56E-01	1.05E-03	4.25E-01
4702	GSM203293	GSE8218	Prostate	Prostate	2.38E-03	-1.94E-01	1.78E-01	-2.13E-01
4703	GSM203294	GSE8218	Prostate	Prostate	5.46E-01	-6.15E-02	2.68E-01	1.86E-01
4704	GSM203295	GSE8218	Prostate	Prostate	3.66E-07	-3.05E-01	2.80E-03	-3.93E-01
4705	GSM203296	GSE8218	Prostate	Prostate	3.34E-02	-1.46E-01	9.70E-02	2.48E-01
4706	GSM203298	GSE8218	Prostate	Prostate	1.96E-04	2.31E-01	5.11E-05	5.10E-01
4707	GSM203299	GSE8218	Prostate	Prostate	1.95E-01	-1.01E-01	4.64E-01	1.42E-01
4708	GSM203300	GSE8218	Prostate	Prostate	1.71E-01	-1.05E-01	7.34E-02	2.62E-01
4709	GSM203301	GSE8218	Prostate	Prostate	6.13E-03	-1.79E-01	1.78E-01	-2.13E-01
4710	GSM203302	GSE8218	Prostate	Prostate	1.69E-04	2.33E-01	3.27E-03	3.88E-01
4711	GSM203303	GSE8218	Prostate	Prostate	5.25E-01	6.35E-02	2.30E-01	1.97E-01
4712	GSM203304	GSE8218	Prostate	Prostate	2.44E-04	2.28E-01	1.88E-03	4.07E-01
4713	GSM203305	GSE8218	Prostate	Prostate	2.01E-01	-1.00E-01	3.04E-01	1.77E-01
4714	GSM203306	GSE8218	Prostate	Prostate	4.98E-08	3.24E-01	1.03E-04	4.92E-01
4715	GSM203307	GSE8218	Prostate	Prostate	8.56E-03	-1.73E-01	2.17E-01	-2.01E-01
4716	GSM203308	GSE8218	Prostate	Prostate	1.39E-01	-1.11E-01	1.78E-01	2.13E-01
4717	GSM203309	GSE8218	Prostate	Prostate	7.84E-07	-2.97E-01	6.43E-03	-3.65E-01
4718	GSM203310	GSE8218	Prostate	Prostate	6.28E-06	-2.74E-01	4.28E-03	-3.79E-01
4719	GSM203311	GSE8218	Prostate	Prostate	7.67E-03	-1.75E-01	3.84E-01	1.59E-01
4720	GSM203312	GSE8218	Prostate	Prostate	9.11E-04	-2.09E-01	1.38E-01	-2.28E-01
4721	GSM203313	GSE8218	Prostate	Prostate	1.97E-02	-1.57E-01	2.15E-01	-2.01E-01
4722	GSM203314	GSE8218	Prostate	Prostate	2.62E-05	-2.57E-01	2.51E-02	-3.11E-01
4723	GSM203315	GSE8218	Prostate	Prostate	1.00E-01	-1.20E-01	9.70E-02	2.48E-01
4724	GSM203316	GSE8218	Prostate	Prostate	2.91E-02	1.49E-01	2.40E-02	3.13E-01
4725	GSM203317	GSE8218	Prostate	Prostate	3.67E-02	-1.44E-01	2.00E-01	2.06E-01
4726	GSM203318	GSE8218	Prostate	Prostate	3.34E-02	-1.46E-01	1.53E-01	2.22E-01
4727	GSM203319	GSE8218	Prostate	Prostate	3.19E-02	1.47E-01	1.70E-03	4.10E-01
4728	GSM203320	GSE8218	Prostate	Prostate	2.72E-01	-9.03E-02	2.02E-02	3.21E-01
4729	GSM203321	GSE8218	Prostate	Prostate	1.18E-02	-1.67E-01	7.24E-02	-2.63E-01
4730	GSM203322	GSE8218	Prostate	Prostate	3.34E-05	-2.54E-01	8.26E-03	-3.55E-01
4731	GSM203323	GSE8218	Prostate	Prostate	2.42E-01	-9.42E-02	2.91E-01	1.80E-01

4732	GSM203324	GSE8218	Prostate	Prostate	2.84E-05	-2.56E-01	1.61E-02	-3.30E-01
4733	GSM203325	GSE8218	Prostate	Prostate	4.73E-01	-6.85E-02	5.61E-02	2.75E-01
4734	GSM203326	GSE8218	Prostate	Prostate	2.27E-04	-2.29E-01	1.94E-01	-2.08E-01
4735	GSM203327	GSE8218	Prostate	Prostate	3.56E-01	-8.04E-02	2.00E-01	2.06E-01
4736	GSM203328	GSE8218	Prostate	Prostate	4.09E-03	1.86E-01	5.55E-03	3.70E-01
4737	GSM203329	GSE8218	Prostate	Prostate	2.21E-01	-9.72E-02	3.60E-01	-1.64E-01
4738	GSM203330	GSE8218	Prostate	Prostate	4.01E-02	-1.42E-01	3.20E-01	1.73E-01
4739	GSM203331	GSE8218	Prostate	Prostate	3.34E-02	1.46E-01	3.51E-02	2.97E-01
4740	GSM203332	GSE8218	Prostate	Prostate	2.88E-01	8.83E-02	2.30E-01	1.97E-01
4741	GSM203333	GSE8218	Prostate	Prostate	5.36E-09	-3.45E-01	3.27E-03	-3.88E-01
4742	GSM203334	GSE8218	Prostate	Prostate	3.04E-03	-1.90E-01	1.26E-01	-2.33E-01
4743	GSM203335	GSE8218	Prostate	Prostate	9.27E-05	2.41E-01	7.48E-04	4.35E-01
4744	GSM203336	GSE8218	Prostate	Prostate	1.34E-01	1.12E-01	2.00E-01	2.06E-01
4745	GSM203337	GSE8218	Prostate	Prostate	3.04E-03	-1.90E-01	2.76E-01	-1.84E-01
4746	GSM203338	GSE8218	Prostate	Prostate	8.29E-09	-3.41E-01	4.02E-04	-4.54E-01
4747	GSM203339	GSE8218	Prostate	Prostate	4.01E-02	-1.42E-01	1.57E-01	-2.21E-01
4748	GSM203340	GSE8218	Prostate	Prostate	1.27E-19	5.22E-01	3.75E-10	7.56E-01
4749	GSM203341	GSE8218	Prostate	Prostate	1.82E-04	-2.32E-01	8.26E-03	-3.55E-01
4750	GSM203342	GSE8218	Prostate	Prostate	1.71E-01	-1.05E-01	2.23E-01	1.99E-01
4751	GSM203343	GSE8218	Prostate	Prostate	2.08E-01	-9.92E-02	1.35E-01	2.30E-01
4752	GSM203344	GSE8218	Prostate	Prostate	3.47E-01	8.13E-02	1.15E-01	2.39E-01
4753	GSM203345	GSE8218	Prostate	Prostate	1.18E-03	2.05E-01	3.47E-03	3.86E-01
4754	GSM203346	GSE8218	Prostate	Prostate	4.47E-10	3.67E-01	4.68E-06	5.69E-01
4755	GSM203347	GSE8218	Prostate	Prostate	1.47E-05	-2.64E-01	6.79E-03	-3.63E-01
4756	GSM203348	GSE8218	Prostate	Prostate	4.03E-07	3.04E-01	9.40E-06	5.52E-01
4757	GSM203349	GSE8218	Prostate	Prostate	6.81E-05	2.45E-01	4.31E-04	4.52E-01
4758	GSM203350	GSE8218	Prostate	Prostate	1.65E-01	1.06E-01	7.05E-02	2.64E-01
4759	GSM203351	GSE8218	Prostate	Prostate	1.70E-02	1.60E-01	4.28E-03	3.79E-01
4760	GSM203352	GSE8218	Prostate	Prostate	3.32E-07	3.06E-01	5.60E-07	6.16E-01
4761	GSM203353	GSE8218	Prostate	Prostate	2.83E-11	3.90E-01	1.16E-07	6.49E-01
4762	GSM203356	GSE8218	Prostate	Prostate	3.86E-03	1.87E-01	7.16E-03	3.61E-01
4763	GSM203357	GSE8218	Prostate	Prostate	4.33E-01	-7.24E-02	1.48E-01	2.24E-01
4764	GSM203358	GSE8218	Prostate	Prostate	3.39E-06	2.81E-01	1.27E-03	4.19E-01
4765	GSM203359	GSE8218	Prostate	Prostate	3.19E-26	6.06E-01	5.48E-11	7.89E-01
4766	GSM203360	GSE8218	Prostate	Prostate	7.60E-02	-1.27E-01	2.17E-01	2.01E-01
4767	GSM203361	GSE8218	Prostate	Prostate	1.00E-01	1.20E-01	5.85E-02	2.73E-01
4768	GSM203362	GSE8218	Prostate	Prostate	1.85E-07	3.12E-01	7.54E-06	5.57E-01
4769	GSM203363	GSE8218	Prostate	Prostate	3.54E-10	3.69E-01	1.16E-07	6.49E-01
4770	GSM203364	GSE8218	Prostate	Prostate	4.62E-13	4.22E-01	1.06E-09	7.38E-01
4771	GSM203365	GSE8218	Prostate	Prostate	1.35E-03	2.03E-01	5.88E-04	4.43E-01
4772	GSM203366	GSE8218	Prostate	Prostate	4.42E-06	2.78E-01	3.73E-06	5.74E-01
4773	GSM203367	GSE8218	Prostate	Prostate	1.80E-13	4.29E-01	3.21E-08	6.74E-01
4774	GSM203368	GSE8218	Prostate	Prostate	2.88E-01	8.83E-02	1.15E-01	2.39E-01
4775	GSM203369	GSE8218	Prostate	Prostate	2.27E-04	-2.29E-01	4.62E-02	-2.85E-01
4776	GSM203370	GSE8218	Prostate	Prostate	1.88E-02	1.58E-01	1.83E-02	3.25E-01
4777	GSM203371	GSE8218	Prostate	Prostate	1.79E-26	6.09E-01	6.16E-11	7.87E-01
4778	GSM203372	GSE8218	Prostate	Prostate	5.57E-01	-6.05E-02	3.84E-01	1.59E-01
4779	GSM203373	GSE8218	Prostate	Prostate	4.42E-06	2.78E-01	2.26E-04	4.70E-01
4780	GSM203374	GSE8218	Prostate	Prostate	1.95E-01	-1.01E-01	2.10E-01	-2.03E-01
4781	GSM203375	GSE8218	Prostate	Prostate	1.74E-05	2.62E-01	7.01E-04	4.37E-01
4782	GSM203376	GSE8218	Prostate	Prostate	1.25E-06	-2.92E-01	9.47E-04	-4.28E-01
4783	GSM203377	GSE8218	Prostate	Prostate	1.08E-19	5.23E-01	2.36E-09	7.23E-01
4784	GSM203378	GSE8218	Prostate	Prostate	1.24E-05	2.66E-01	8.56E-03	3.54E-01
4785	GSM203379	GSE8218	Prostate	Prostate	2.72E-01	-9.03E-02	3.75E-01	1.61E-01
4786	GSM203380	GSE8218	Prostate	Prostate	1.97E-03	-1.97E-01	2.91E-01	-1.80E-01
4787	GSM203381	GSE8218	Prostate	Prostate	1.88E-02	-1.58E-01	4.28E-01	1.50E-01
4788	GSM203382	GSE8218	Prostate	Prostate	4.62E-04	-2.19E-01	7.53E-02	-2.61E-01
4789	GSM203383	GSE8218	Prostate	Prostate	1.70E-02	-1.60E-01	1.35E-01	-2.30E-01
4790	GSM203384	GSE8218	Prostate	Prostate	4.33E-01	7.24E-02	9.70E-02	2.48E-01
4791	GSM203385	GSE8218	Prostate	Prostate	1.04E-03	-2.07E-01	3.35E-02	-2.99E-01
4792	GSM203386	GSE8218	Prostate	Prostate	7.67E-03	1.75E-01	2.00E-03	4.05E-01
4793	GSM203387	GSE8218	Prostate	Prostate	5.64E-10	3.65E-01	1.02E-05	5.50E-01
4794	GSM203388	GSE8218	Prostate	Prostate	8.57E-02	-1.24E-01	8.45E-02	2.55E-01
4795	GSM203389	GSE8218	Prostate	Prostate	2.14E-01	-9.82E-02	5.03E-02	2.81E-01
4796	GSM203390	GSE8218	Prostate	Prostate	1.83E-01	-1.03E-01	4.38E-01	1.48E-01
4797	GSM203391	GSE8218	Prostate	Prostate	4.49E-08	3.25E-01	6.93E-06	5.59E-01
4798	GSM203392	GSE8218	Prostate	Prostate	1.16E-01	1.16E-01	1.07E-01	2.43E-01
4799	GSM203393	GSE8218	Prostate	Prostate	1.25E-02	-1.66E-01	4.54E-01	-1.44E-01
4800	GSM203394	GSE8218	Prostate	Prostate	9.63E-02	-1.21E-01	2.49E-01	1.91E-01
4801	GSM203395	GSE8218	Prostate	Prostate	1.83E-01	1.03E-01	2.97E-02	3.04E-01
4802	GSM203396	GSE8218	Prostate	Prostate	5.39E-05	-2.48E-01	1.53E-02	-3.32E-01
4803	GSM203397	GSE8218	Prostate	Prostate	7.25E-03	-1.76E-01	2.42E-01	-1.93E-01
4804	GSM203398	GSE8218	Prostate	Prostate	2.42E-01	-9.42E-02	4.12E-02	2.90E-01
4805	GSM203399	GSE8218	Prostate	Prostate	1.12E-02	-1.68E-01	2.10E-01	-2.03E-01

4806	GSM203400	GSE8218	Prostate	Prostate	8.10E-03	-1.74E-01	3.60E-01	-1.64E-01
4807	GSM203401	GSE8218	Prostate	Prostate	1.83E-01	-1.03E-01	2.91E-01	-1.80E-01
4808	GSM203402	GSE8218	Prostate	Prostate	5.04E-01	6.55E-02	4.82E-02	2.83E-01
4809	GSM203403	GSE8218	Prostate	Prostate	5.25E-01	-6.35E-02	5.85E-02	2.73E-01
4810	GSM203404	GSE8218	Prostate	Prostate	3.21E-01	8.43E-02	2.40E-02	3.13E-01
4811	GSM203405	GSE8218	Prostate	Prostate	5.69E-02	-1.34E-01	2.91E-01	1.80E-01
4812	GSM203406	GSE8218	Prostate	Prostate	1.30E-01	-1.13E-01	1.69E-02	3.28E-01
4813	GSM203407	GSE8218	Prostate	Prostate	3.75E-01	-7.84E-02	5.03E-02	2.81E-01
4814	GSM203408	GSE8218	Prostate	Prostate	4.47E-10	-3.67E-01	5.50E-04	-4.45E-01
4815	GSM617581	GSE25136	Prostate	Prostate	1.24E-05	2.66E-01	2.68E-05	5.27E-01
4816	GSM617582	GSE25136	Prostate	Prostate	2.10E-03	-1.96E-01	1.01E-01	2.46E-01
4817	GSM617583	GSE25136	Prostate	Prostate	3.67E-02	-1.44E-01	2.91E-01	1.80E-01
4818	GSM617584	GSE25136	Prostate	Prostate	2.21E-10	3.73E-01	5.09E-06	5.67E-01
4819	GSM617585	GSE25136	Prostate	Prostate	1.08E-01	-1.18E-01	4.28E-01	1.50E-01
4820	GSM617586	GSE25136	Prostate	Prostate	7.60E-02	-1.27E-01	1.26E-01	-2.33E-01
4821	GSM617587	GSE25136	Prostate	Prostate	2.64E-01	9.13E-02	1.31E-01	2.31E-01
4822	GSM617588	GSE25136	Prostate	Prostate	1.35E-05	2.65E-01	2.92E-04	4.63E-01
4823	GSM617589	GSE25136	Prostate	Prostate	1.18E-11	3.97E-01	4.68E-06	5.69E-01
4824	GSM617590	GSE25136	Prostate	Prostate	1.65E-01	1.06E-01	3.35E-02	2.99E-01
4825	GSM617591	GSE25136	Prostate	Prostate	1.89E-01	1.02E-01	2.42E-01	1.93E-01
4826	GSM617592	GSE25136	Prostate	Prostate	1.55E-10	3.76E-01	1.38E-05	5.43E-01
4827	GSM617593	GSE25136	Prostate	Prostate	2.21E-01	9.72E-02	2.02E-02	3.21E-01
4828	GSM617594	GSE25136	Prostate	Prostate	1.65E-01	-1.06E-01	2.30E-01	1.97E-01
4829	GSM617595	GSE25136	Prostate	Prostate	7.67E-03	1.75E-01	4.70E-03	3.76E-01
4830	GSM617596	GSE25136	Prostate	Prostate	5.30E-04	2.17E-01	1.15E-02	3.43E-01
4831	GSM617597	GSE25136	Prostate	Prostate	1.60E-01	-1.07E-01	2.02E-02	3.21E-01
4832	GSM617598	GSE25136	Prostate	Prostate	1.97E-02	1.57E-01	1.92E-02	3.23E-01
4833	GSM617599	GSE25136	Prostate	Prostate	1.95E-01	-1.01E-01	2.97E-02	3.04E-01
4834	GSM617600	GSE25136	Prostate	Prostate	5.91E-07	-3.00E-01	1.01E-03	-4.26E-01
4835	GSM617601	GSE25136	Prostate	Prostate	4.39E-02	-1.40E-01	3.60E-01	-1.64E-01
4836	GSM617602	GSE25136	Prostate	Prostate	1.82E-04	-2.32E-01	5.61E-02	-2.75E-01
4837	GSM617603	GSE25136	Prostate	Prostate	1.83E-01	1.03E-01	9.70E-02	2.48E-01
4838	GSM617604	GSE25136	Prostate	Prostate	8.10E-03	1.74E-01	7.48E-04	4.35E-01
4839	GSM617605	GSE25136	Prostate	Prostate	3.34E-02	1.46E-01	1.53E-02	3.32E-01
4840	GSM617606	GSE25136	Prostate	Prostate	3.30E-01	8.33E-02	1.67E-01	2.17E-01
4841	GSM617607	GSE25136	Prostate	Prostate	2.72E-01	9.03E-02	1.69E-02	3.28E-01
4842	GSM617608	GSE25136	Prostate	Prostate	7.30E-02	-1.28E-01	5.24E-02	2.79E-01
4843	GSM617609	GSE25136	Prostate	Prostate	1.53E-02	-1.62E-01	1.26E-01	2.33E-01
4844	GSM617610	GSE25136	Prostate	Prostate	2.84E-06	2.83E-01	4.50E-04	4.50E-01
4845	GSM617611	GSE25136	Prostate	Prostate	1.97E-02	1.57E-01	6.96E-02	2.65E-01
4846	GSM617612	GSE25136	Prostate	Prostate	9.46E-07	2.95E-01	1.00E-04	4.92E-01
4847	GSM617613	GSE25136	Prostate	Prostate	5.37E-07	-3.01E-01	8.26E-03	-3.55E-01
4848	GSM617614	GSE25136	Prostate	Prostate	3.34E-02	1.46E-01	2.93E-02	3.05E-01
4849	GSM617615	GSE25136	Prostate	Prostate	6.46E-02	1.31E-01	8.71E-03	3.53E-01
4850	GSM617616	GSE25136	Prostate	Prostate	5.01E-02	-1.37E-01	1.73E-01	2.15E-01
4851	GSM617617	GSE25136	Prostate	Prostate	7.27E-01	-4.46E-02	1.11E-01	2.41E-01
4852	GSM617618	GSE25136	Prostate	Prostate	3.19E-02	-1.47E-01	2.30E-01	1.97E-01
4853	GSM617619	GSE25136	Prostate	Prostate	6.30E-05	-2.46E-01	3.35E-02	-2.99E-01
4854	GSM617620	GSE25136	Prostate	Prostate	5.91E-07	3.00E-01	2.11E-04	4.72E-01
4855	GSM617621	GSE25136	Prostate	Prostate	1.38E-02	1.64E-01	1.09E-02	3.45E-01
4856	GSM617622	GSE25136	Prostate	Prostate	1.25E-01	1.14E-01	6.79E-03	3.63E-01
4857	GSM617623	GSE25136	Prostate	Prostate	8.10E-12	4.00E-01	2.34E-07	6.34E-01
4858	GSM617624	GSE25136	Prostate	Prostate	1.53E-03	-2.01E-01	1.48E-01	-2.24E-01
4859	GSM617625	GSE25136	Prostate	Prostate	1.08E-01	-1.18E-01	6.07E-01	1.15E-01
4860	GSM617626	GSE25136	Prostate	Prostate	2.52E-02	1.52E-01	1.46E-02	3.34E-01
4861	GSM617627	GSE25136	Prostate	Prostate	3.19E-02	-1.47E-01	1.26E-01	2.33E-01
4862	GSM617628	GSE25136	Prostate	Prostate	1.71E-01	1.05E-01	2.23E-01	1.99E-01
4863	GSM617629	GSE25136	Prostate	Prostate	1.97E-03	-1.97E-01	3.34E-01	-1.70E-01
4864	GSM617630	GSE25136	Prostate	Prostate	3.94E-01	7.64E-02	4.82E-02	2.83E-01
4865	GSM617631	GSE25136	Prostate	Prostate	2.27E-04	-2.29E-01	7.83E-02	-2.59E-01
4866	GSM617632	GSE25136	Prostate	Prostate	2.96E-01	-8.73E-02	1.11E-01	2.41E-01
4867	GSM617633	GSE25136	Prostate	Prostate	9.54E-03	-1.71E-01	8.67E-02	-2.54E-01
4868	GSM617634	GSE25136	Prostate	Prostate	5.01E-02	-1.37E-01	1.35E-01	2.30E-01
4869	GSM617635	GSE25136	Prostate	Prostate	5.36E-01	-6.25E-02	7.05E-02	2.64E-01
4870	GSM617636	GSE25136	Prostate	Prostate	2.18E-02	-1.55E-01	1.78E-01	2.13E-01
4871	GSM617637	GSE25136	Prostate	Prostate	1.00E-01	1.20E-01	2.29E-02	3.15E-01
4872	GSM617638	GSE25136	Prostate	Prostate	1.54E-01	-1.08E-01	3.67E-02	2.95E-01
4873	GSM617639	GSE25136	Prostate	Prostate	2.86E-03	1.91E-01	2.91E-03	3.92E-01
4874	GSM617640	GSE25136	Prostate	Prostate	4.94E-01	6.65E-02	1.11E-02	3.44E-01
4875	GSM617641	GSE25136	Prostate	Prostate	1.75E-10	3.75E-01	1.27E-05	5.45E-01
4876	GSM617642	GSE25136	Prostate	Prostate	1.21E-01	1.15E-01	5.24E-02	2.79E-01
4877	GSM617643	GSE25136	Prostate	Prostate	6.32E-01	5.36E-02	1.07E-01	2.43E-01
4878	GSM617644	GSE25136	Prostate	Prostate	2.21E-01	-9.72E-02	7.34E-02	2.62E-01
4879	GSM617645	GSE25136	Prostate	Prostate	3.38E-01	-8.23E-02	1.48E-01	2.24E-01

4880	GSM617646	GSE25136	Prostate	Prostate	5.94E-02	1.33E-01	1.92E-02	3.23E-01
4881	GSM617647	GSE25136	Prostate	Prostate	2.08E-01	9.92E-02	4.30E-02	2.88E-01
4882	GSM617648	GSE25136	Prostate	Prostate	1.49E-01	1.09E-01	2.29E-02	3.15E-01
4883	GSM617649	GSE25136	Prostate	Prostate	9.63E-02	1.21E-01	9.00E-02	2.52E-01
4884	GSM617650	GSE25136	Prostate	Prostate	1.00E-01	1.20E-01	1.53E-02	3.32E-01
4885	GSM617651	GSE25136	Prostate	Prostate	7.96E-05	2.43E-01	2.90E-05	5.25E-01
4886	GSM617652	GSE25136	Prostate	Prostate	3.66E-01	-7.94E-02	2.30E-01	1.97E-01
4887	GSM617653	GSE25136	Prostate	Prostate	2.52E-17	4.89E-01	1.06E-09	7.38E-01
4888	GSM617654	GSE25136	Prostate	Prostate	2.96E-01	8.73E-02	1.69E-02	3.28E-01
4889	GSM617655	GSE25136	Prostate	Prostate	1.61E-02	-1.61E-01	1.78E-01	2.13E-01
4890	GSM617656	GSE25136	Prostate	Prostate	6.97E-04	-2.13E-01	3.67E-02	-2.95E-01
4891	GSM617657	GSE25136	Prostate	Prostate	1.18E-03	-2.05E-01	9.35E-02	-2.50E-01
4892	GSM617658	GSE25136	Prostate	Prostate	4.59E-02	-1.39E-01	6.09E-02	2.71E-01
4893	GSM617659	GSE25136	Prostate	Prostate	1.18E-02	1.67E-01	1.69E-02	3.28E-01
4894	GSM701952	GSE28403	Prostate	Prostate	5.94E-02	1.33E-01	1.00E-02	3.48E-01
4895	GSM701953	GSE28403	Prostate	Prostate	4.13E-01	-7.44E-02	3.90E-01	-1.57E-01
4896	GSM701954	GSE28403	Prostate	Prostate	2.62E-04	2.27E-01	2.21E-03	4.01E-01
4897	GSM701955	GSE28403	Prostate	Prostate	9.63E-02	1.21E-01	2.12E-03	4.03E-01
4898	GSM701956	GSE28403	Prostate	Prostate	2.10E-03	1.96E-01	5.25E-03	3.72E-01
4899	GSM701957	GSE28403	Prostate	Prostate	6.00E-01	5.65E-02	5.85E-02	2.73E-01
4900	GSM701958	GSE28403	Prostate	Prostate	2.72E-01	-9.03E-02	1.11E-02	3.44E-01
4901	GSM701959	GSE28403	Prostate	Prostate	4.63E-01	-6.94E-02	9.70E-02	2.48E-01
4902	GSM701960	GSE28403	Prostate	Prostate	1.49E-01	1.09E-01	4.30E-02	2.88E-01
4903	GSM701961	GSE28403	Prostate	Prostate	1.53E-02	-1.62E-01	3.26E-01	-1.72E-01
4904	GSM701962	GSE28403	Prostate	Prostate	8.10E-03	1.74E-01	1.92E-02	3.23E-01
4905	GSM701963	GSE28403	Prostate	Prostate	6.97E-04	-2.13E-01	2.68E-01	-1.86E-01
4906	GSM701964	GSE28403	Prostate	Prostate	5.94E-02	1.33E-01	2.40E-02	3.13E-01
4907	GSM799468	GSE32269	Prostate	Prostate	1.70E-02	-1.60E-01	1.07E-01	-2.43E-01
4908	GSM799469	GSE32269	Prostate	Prostate	1.65E-01	-1.06E-01	2.30E-01	1.97E-01
4909	GSM799470	GSE32269	Prostate	Prostate	4.73E-01	6.85E-02	1.78E-01	2.13E-01
4910	GSM799471	GSE32269	Prostate	Prostate	3.30E-01	-8.33E-02	6.09E-02	2.71E-01
4911	GSM799472	GSE32269	Prostate	Prostate	5.25E-01	-6.35E-02	2.91E-01	1.80E-01
4912	GSM799473	GSE32269	Prostate	Prostate	4.01E-02	-1.42E-01	2.30E-01	-1.97E-01
4913	GSM799474	GSE32269	Prostate	Prostate	3.21E-01	8.43E-02	1.01E-01	2.46E-01
4914	GSM799475	GSE32269	Prostate	Prostate	3.04E-01	8.63E-02	1.15E-01	2.39E-01
4915	GSM799476	GSE32269	Prostate	Prostate	6.86E-03	-1.77E-01	1.38E-01	-2.28E-01
4916	GSM799477	GSE32269	Prostate	Prostate	4.02E-04	-2.21E-01	5.85E-02	-2.73E-01
4917	GSM799478	GSE32269	Prostate	Prostate	2.49E-01	9.33E-02	1.78E-01	2.13E-01
4918	GSM799479	GSE32269	Prostate	Prostate	1.21E-01	-1.15E-01	3.60E-01	1.64E-01
4919	GSM799480	GSE32269	Prostate	Prostate	4.43E-01	-7.14E-02	2.00E-01	2.06E-01
4920	GSM799481	GSE32269	Prostate	Prostate	2.49E-01	-9.33E-02	2.30E-01	1.97E-01
4921	GSM799482	GSE32269	Prostate	Prostate	1.49E-01	-1.09E-01	2.00E-01	2.06E-01
4922	GSM799483	GSE32269	Prostate	Prostate	5.69E-02	-1.34E-01	1.35E-01	2.30E-01
4923	GSM799484	GSE32269	Prostate	Prostate	1.38E-02	-1.64E-01	1.62E-01	-2.19E-01
4924	GSM799485	GSE32269	Prostate	Prostate	3.38E-01	-8.23E-02	1.62E-01	2.19E-01
4925	GSM799486	GSE32269	Prostate	Prostate	6.86E-01	-4.86E-02	1.35E-01	2.30E-01
4926	GSM799487	GSE32269	Prostate	Prostate	2.72E-01	9.03E-02	7.34E-02	2.62E-01
4927	GSM799488	GSE32269	Prostate	Prostate	3.84E-02	-1.43E-01	3.20E-01	1.73E-01
4928	GSM799489	GSE32269	Prostate	Prostate	1.34E-01	-1.12E-01	2.30E-01	1.97E-01
4929	GSM799490	GSE32269	Prostate	Prostate	2.53E-03	-1.93E-01	1.48E-01	-2.24E-01
4930	GSM799491	GSE32269	Prostate	Prostate	8.56E-03	1.73E-01	5.55E-03	3.70E-01
4931	GSM799492	GSE32269	Prostate	Prostate	2.21E-01	9.72E-02	3.51E-02	2.97E-01
4932	GSM799493	GSE32269	Prostate	Prostate	2.28E-01	9.62E-02	1.92E-02	3.23E-01
4933	GSM799494	GSE32269	Prostate	Prostate	9.54E-03	1.71E-01	2.00E-03	4.05E-01
4934	GSM799495	GSE32269	Prostate	Prostate	7.25E-03	-1.76E-01	9.70E-02	-2.48E-01
4935	GSM799496	GSE32269	Prostate	Prostate	1.53E-03	-2.01E-01	2.18E-02	-3.17E-01
4936	GSM799497	GSE32269	Prostate	Prostate	1.79E-02	-1.59E-01	1.31E-01	2.31E-01
4937	GSM799498	GSE32269	Prostate	Prostate	7.96E-05	2.43E-01	1.03E-04	4.92E-01
4938	GSM799499	GSE32269	Prostate	Prostate	9.26E-02	1.22E-01	8.71E-03	3.53E-01
4939	GSM799500	GSE32269	Prostate	Prostate	2.21E-01	9.72E-02	2.97E-02	3.04E-01
4940	GSM799501	GSE32269	Prostate	Prostate	2.07E-02	1.56E-01	6.79E-03	3.63E-01
4941	GSM799502	GSE32269	Prostate	Prostate	7.60E-02	1.27E-01	1.83E-02	3.25E-01
4942	GSM799503	GSE32269	Prostate	Prostate	1.82E-04	2.32E-01	1.12E-03	4.23E-01
4943	GSM799504	GSE32269	Prostate	Prostate	3.34E-02	1.46E-01	8.71E-03	3.53E-01
4944	GSM799505	GSE32269	Prostate	Prostate	8.57E-02	-1.24E-01	1.53E-01	2.22E-01
4945	GSM799506	GSE32269	Prostate	Prostate	1.25E-02	1.66E-01	4.28E-03	3.79E-01
4946	GSM799507	GSE32269	Prostate	Prostate	1.60E-01	-1.07E-01	6.09E-02	2.71E-01
4947	GSM799508	GSE32269	Prostate	Prostate	1.71E-01	-1.05E-01	1.78E-01	2.13E-01
4948	GSM799509	GSE32269	Prostate	Prostate	6.86E-03	1.77E-01	8.71E-03	3.53E-01
4949	GSM799510	GSE32269	Prostate	Prostate	1.63E-03	2.00E-01	3.47E-03	3.86E-01
4950	GSM799511	GSE32269	Prostate	Prostate	6.73E-02	1.30E-01	3.35E-04	4.59E-01
4951	GSM799512	GSE32269	Prostate	Prostate	3.30E-01	-8.33E-02	1.01E-01	2.46E-01
4952	GSM799513	GSE32269	Prostate	Prostate	1.00E-09	3.60E-01	4.30E-06	5.70E-01
4953	GSM799514	GSE32269	Prostate	Prostate	4.88E-07	3.02E-01	1.34E-04	4.85E-01

4954	GSM799515	GSE32269	Prostate	Prostate	2.84E-05	2.56E-01	1.50E-03	4.14E-01
4955	GSM799516	GSE32269	Prostate	Prostate	6.97E-04	2.13E-01	7.48E-04	4.35E-01
4956	GSM799517	GSE32269	Prostate	Prostate	2.62E-04	2.27E-01	2.35E-03	3.99E-01
4957	GSM799518	GSE32269	Prostate	Prostate	1.60E-01	1.07E-01	5.85E-02	2.73E-01
4958	GSM1131566	GSE46517	Malignant Melanoma	Skin	2.24E-12	4.10E-01	9.97E-07	6.03E-01
4959	GSM1131567	GSE46517	Malignant Melanoma	Skin	2.41E-08	3.31E-01	4.66E-07	6.20E-01
4960	GSM1131568	GSE46517	Malignant Melanoma	Skin	4.31E-09	3.47E-01	5.86E-06	5.63E-01
4961	GSM1131569	GSE46517	Malignant Melanoma	Skin	7.45E-04	2.12E-01	1.58E-02	3.30E-01
4962	GSM1131570	GSE46517	Malignant Melanoma	Skin	1.36E-16	4.78E-01	2.36E-09	7.23E-01
4963	GSM1131571	GSE46517	Malignant Melanoma	Skin	9.59E-11	3.80E-01	1.65E-06	5.92E-01
4964	GSM1131572	GSE46517	Malignant Melanoma	Skin	1.95E-08	3.33E-01	5.86E-06	5.63E-01
4965	GSM1131573	GSE46517	Malignant Melanoma	Skin	1.57E-13	4.30E-01	2.54E-04	4.67E-01
4966	GSM1131574	GSE46517	Malignant Melanoma	Skin	1.46E-04	2.35E-01	7.70E-03	3.58E-01
4967	GSM1131575	GSE46517	Malignant Melanoma	Skin	6.28E-06	2.74E-01	3.21E-03	3.89E-01
4968	GSM1131576	GSE46517	Malignant Melanoma	Skin	1.75E-08	3.34E-01	1.96E-03	4.05E-01
4969	GSM1131577	GSE46517	Malignant Melanoma	Skin	1.26E-04	2.37E-01	1.33E-03	4.18E-01
4970	GSM1131578	GSE46517	Malignant Melanoma	Skin	7.27E-15	4.51E-01	1.36E-07	6.45E-01
4971	GSM1131579	GSE46517	Malignant Melanoma	Skin	7.44E-09	3.42E-01	6.38E-06	5.61E-01
4972	GSM1131580	GSE46517	Malignant Melanoma	Skin	1.28E-15	4.63E-01	1.24E-08	6.92E-01
4973	GSM1131581	GSE46517	Malignant Melanoma	Skin	4.37E-21	5.42E-01	1.90E-09	7.27E-01
4974	GSM1131582	GSE46517	Malignant Melanoma	Skin	1.26E-03	2.04E-01	5.16E-03	3.72E-01
4975	GSM1131583	GSE46517	Malignant Melanoma	Skin	1.24E-05	2.66E-01	1.31E-04	4.85E-01
4976	GSM1131584	GSE46517	Malignant Melanoma	Skin	4.60E-05	2.50E-01	5.38E-04	4.45E-01
4977	GSM1131585	GSE46517	Malignant Melanoma	Skin	7.54E-11	3.82E-01	6.25E-05	5.05E-01
4978	GSM1131586	GSE46517	Malignant Melanoma	Skin	3.76E-12	4.06E-01	4.20E-08	6.69E-01
4979	GSM1131587	GSE46517	Malignant Melanoma	Skin	1.48E-15	4.62E-01	1.36E-07	6.45E-01
4980	GSM1131588	GSE46517	Malignant Melanoma	Skin	3.31E-12	4.07E-01	5.18E-09	7.09E-01
4981	GSM1131589	GSE46517	Malignant Melanoma	Skin	5.79E-03	1.80E-01	1.63E-04	4.79E-01
4982	GSM1131590	GSE46517	Malignant Melanoma	Skin	2.06E-13	4.28E-01	2.34E-07	6.34E-01
4983	GSM1131591	GSE46517	Malignant Melanoma	Skin	2.59E-06	2.84E-01	3.30E-05	5.21E-01
4984	GSM1131592	GSE46517	Malignant Melanoma	Skin	2.84E-06	2.83E-01	3.95E-06	5.72E-01
4985	GSM1131593	GSE46517	Malignant Melanoma	Skin	7.75E-23	5.64E-01	1.45E-10	7.72E-01
4986	GSM1131594	GSE46517	Malignant Melanoma	Skin	2.16E-06	2.86E-01	8.65E-06	5.54E-01
4987	GSM1131595	GSE46517	Malignant Melanoma	Skin	2.89E-16	4.73E-01	1.42E-09	7.32E-01
4988	GSM1131596	GSE46517	Malignant Melanoma	Skin	2.11E-04	2.30E-01	3.40E-03	3.87E-01
4989	GSM1131597	GSE46517	Malignant Melanoma	Skin	6.10E-16	4.68E-01	1.42E-09	7.32E-01
4990	GSM1131598	GSE46517	Malignant Melanoma	Skin	3.20E-11	3.89E-01	6.53E-07	6.12E-01
4991	GSM1131599	GSE46517	Malignant Melanoma	Skin	1.48E-26	6.10E-01	2.03E-11	8.05E-01
4992	GSM1131600	GSE46517	Malignant Melanoma	Skin	4.67E-17	4.85E-01	3.51E-09	7.16E-01
4993	GSM1131601	GSE46517	Malignant Melanoma	Skin	4.83E-06	2.77E-01	1.51E-06	5.94E-01
4994	GSM1131602	GSE46517	Malignant Melanoma	Skin	2.17E-08	3.32E-01	1.50E-07	6.43E-01
4995	GSM1131603	GSE46517	Malignant Melanoma	Skin	2.73E-07	3.08E-01	2.88E-06	5.80E-01
4996	GSM1131604	GSE46517	Malignant Melanoma	Skin	6.68E-11	3.83E-01	5.12E-08	6.65E-01
4997	GSM1131605	GSE46517	Malignant Melanoma	Skin	1.85E-17	4.91E-01	5.48E-11	7.89E-01
4998	GSM1131606	GSE46517	Malignant Melanoma	Skin	4.62E-04	2.19E-01	2.08E-03	4.03E-01
4999	GSM1131607	GSE46517	Malignant Melanoma	Skin	7.27E-15	4.51E-01	2.90E-08	6.76E-01
5000	GSM1131608	GSE46517	Malignant Melanoma	Skin	6.00E-14	4.37E-01	2.12E-09	7.25E-01
5001	GSM1131609	GSE46517	Malignant Melanoma	Skin	2.35E-01	9.52E-02	1.60E-01	2.20E-01
5002	GSM1131610	GSE46517	Malignant Melanoma	Skin	8.20E-16	4.66E-01	3.01E-07	6.29E-01
5003	GSM1131611	GSE46517	Malignant Melanoma	Skin	4.95E-04	2.18E-01	2.64E-03	3.95E-01
5004	GSM1131612	GSE46517	Malignant Melanoma	Skin	1.72E-11	3.94E-01	9.51E-10	7.40E-01
5005	GSM1131613	GSE46517	Malignant Melanoma	Skin	7.13E-12	4.01E-01	6.67E-08	6.60E-01
5006	GSM1131614	GSE46517	Malignant Melanoma	Skin	3.32E-07	3.06E-01	7.15E-07	6.10E-01
5007	GSM1131615	GSE46517	Malignant Melanoma	Skin	7.91E-14	4.35E-01	1.50E-07	6.43E-01
5008	GSM1131616	GSE46517	Malignant Melanoma	Skin	1.29E-14	4.47E-01	6.67E-08	6.60E-01
5009	GSM1131617	GSE46517	Malignant Melanoma	Skin	2.21E-09	3.53E-01	4.66E-07	6.20E-01
5010	GSM1131618	GSE46517	Malignant Melanoma	Skin	5.54E-20	5.27E-01	8.51E-10	7.42E-01
5011	GSM1131619	GSE46517	Malignant Melanoma	Skin	3.32E-07	3.06E-01	9.97E-07	6.03E-01
5012	GSM1131620	GSE46517	Malignant Melanoma	Skin	1.24E-07	3.15E-01	6.38E-06	5.61E-01
5013	GSM1131621	GSE46517	Malignant Melanoma	Skin	1.12E-07	3.16E-01	4.66E-07	6.20E-01
5014	GSM1131622	GSE46517	Malignant Melanoma	Skin	4.57E-22	5.55E-01	8.51E-10	7.42E-01
5015	GSM1131623	GSE46517	Malignant Melanoma	Skin	6.77E-08	3.21E-01	3.95E-06	5.72E-01
5016	GSM1131624	GSE46517	Malignant Melanoma	Skin	1.35E-17	4.93E-01	5.18E-09	7.09E-01
5017	GSM1131625	GSE46517	Malignant Melanoma	Skin	7.86E-13	4.18E-01	4.20E-08	6.69E-01
5018	GSM1131626	GSE46517	Malignant Melanoma	Skin	9.23E-09	3.40E-01	1.51E-06	5.94E-01
5019	GSM1131627	GSE46517	Malignant Melanoma	Skin	2.91E-12	4.08E-01	3.75E-10	7.56E-01
5020	GSM1131628	GSE46517	Malignant Melanoma	Skin	2.69E-22	5.58E-01	9.51E-10	7.40E-01
5021	GSM1131629	GSE46517	Malignant Melanoma	Skin	1.50E-06	2.90E-01	2.48E-05	5.29E-01
5022	GSM1131630	GSE46517	Malignant Melanoma	Skin	9.19E-12	3.99E-01	2.34E-07	6.34E-01
5023	GSM1131631	GSE46517	Malignant Melanoma	Skin	1.38E-10	3.77E-01	2.29E-05	5.30E-01
5024	GSM1131632	GSE46517	Malignant Melanoma	Skin	6.89E-14	4.36E-01	2.63E-08	6.78E-01
5025	GSM1131633	GSE46517	Malignant Melanoma	Skin	1.12E-02	1.68E-01	2.35E-03	3.99E-01
5026	GSM1131634	GSE46517	Malignant Melanoma	Skin	7.46E-06	2.72E-01	1.92E-06	5.89E-01
5027	GSM1131635	GSE46517	Malignant Melanoma	Skin	6.00E-14	4.37E-01	6.04E-08	6.62E-01

5028	GSM1131636	GSE46517	Malignant Melanoma	Skin	9.67E-15	4.49E-01	3.01E-07	6.29E-01
5029	GSM1131637	GSE46517	Malignant Melanoma	Skin	2.84E-06	2.83E-01	6.38E-06	5.61E-01
5030	GSM1131638	GSE46517	Malignant Melanoma	Skin	1.11E-14	4.48E-01	2.90E-08	6.76E-01
5031	GSM1131639	GSE46517	Malignant Melanoma	Skin	6.49E-07	2.99E-01	1.38E-05	5.43E-01
5032	GSM1131640	GSE46517	Malignant Melanoma	Skin	7.46E-06	2.72E-01	6.38E-06	5.61E-01
5033	GSM1131641	GSE46517	Malignant Melanoma	Skin	2.07E-02	1.56E-01	1.32E-02	3.38E-01
5034	GSM1131642	GSE46517	Malignant Melanoma	Skin	2.41E-08	3.31E-01	3.63E-07	6.25E-01
5035	GSM1131643	GSE46517	Malignant Melanoma	Skin	1.85E-03	1.98E-01	1.88E-03	4.07E-01
5036	GSM1131644	GSE46517	Malignant Melanoma	Skin	1.46E-04	2.35E-01	2.68E-05	5.27E-01
5037	GSM1131645	GSE46517	Malignant Melanoma	Skin	2.83E-11	3.90E-01	2.13E-07	6.36E-01
5038	GSM1131646	GSE46517	Malignant Melanoma	Skin	2.69E-03	1.92E-01	5.15E-04	4.47E-01
5039	GSM1131647	GSE46517	Malignant Melanoma	Skin	2.37E-06	2.85E-01	6.38E-06	5.61E-01
5040	GSM1131648	GSE46517	Malignant Melanoma	Skin	2.04E-07	3.11E-01	3.57E-05	5.19E-01
5041	GSM1131649	GSE46517	Malignant Melanoma	Skin	1.14E-06	2.93E-01	5.86E-06	5.63E-01
5042	GSM1131650	GSE46517	Malignant Melanoma	Skin	1.47E-05	2.64E-01	1.38E-05	5.43E-01
5043	GSM1131651	GSE46517	Malignant Melanoma	Skin	3.94E-01	7.64E-02	7.24E-02	2.63E-01
5044	GSM1131652	GSE46517	Malignant Melanoma	Skin	4.05E-06	2.79E-01	4.68E-06	5.69E-01
5045	GSM1131653	GSE46517	Malignant Melanoma	Skin	2.67E-08	3.30E-01	2.34E-07	6.34E-01
5046	GSM1131654	GSE46517	Malignant Melanoma	Skin	2.11E-04	2.30E-01	8.23E-05	4.98E-01
5047	GSM1131655	GSE46517	Malignant Melanoma	Skin	1.02E-07	3.17E-01	7.83E-07	6.09E-01
5048	GSM1131656	GSE46517	Malignant Melanoma	Skin	7.30E-02	1.28E-01	8.71E-03	3.53E-01
5049	GSM1131657	GSE46517	Malignant Melanoma	Skin	2.27E-04	2.29E-01	9.54E-05	4.94E-01
5050	GSM1131658	GSE46517	Malignant Melanoma	Skin	8.13E-06	2.71E-01	2.29E-05	5.30E-01
5051	GSM1131659	GSE46517	Malignant Melanoma	Skin	4.05E-06	2.79E-01	1.08E-04	4.90E-01
5052	GSM1131660	GSE46517	Malignant Melanoma	Skin	1.41E-09	3.57E-01	3.42E-06	5.76E-01
5053	GSM1131661	GSE46517	Malignant Melanoma	Skin	7.46E-06	2.72E-01	2.00E-05	5.34E-01
5054	GSM1131662	GSE46517	Malignant Melanoma	Skin	1.50E-06	2.90E-01	1.71E-05	5.38E-01
5055	GSM1131663	GSE46517	Malignant Melanoma	Skin	1.35E-05	2.65E-01	1.85E-05	5.36E-01
5056	GSM1131664	GSE46517	Malignant Melanoma	Skin	5.27E-06	2.76E-01	1.17E-05	5.47E-01
5057	GSM1131665	GSE46517	Malignant Melanoma	Skin	8.56E-03	1.73E-01	1.20E-03	4.21E-01
5058	GSM1131666	GSE46517	Malignant Melanoma	Skin	1.16E-01	1.16E-01	9.23E-02	2.50E-01
5059	GSM1131667	GSE46517	Malignant Melanoma	Skin	2.44E-04	2.28E-01	4.04E-03	3.81E-01
5060	GSM1131668	GSE46517	Malignant Melanoma	Skin	2.42E-01	9.42E-02	4.82E-02	2.83E-01
5061	GSM1131669	GSE46517	Malignant Melanoma	Skin	1.08E-10	3.79E-01	1.37E-08	6.90E-01
5062	A0.08	GSE3149	Ovarian Cancer	Ovarian	3.52E-05	-2.56E-01	3.81E-01	-1.59E-01
5063	A860	GSE3149	Ovarian Cancer	Ovarian	1.94E-02	-1.59E-01	4.20E-01	1.50E-01
5064	A872	GSE3149	Ovarian Cancer	Ovarian	2.20E-04	-2.32E-01	3.81E-01	-1.59E-01
5065	A922	GSE3149	Ovarian Cancer	Ovarian	3.14E-01	8.61E-02	1.94E-03	4.04E-01
5066	A1024	GSE3149	Ovarian Cancer	Ovarian	4.40E-05	-2.53E-01	3.61E-01	-1.63E-01
5067	A1447	GSE3149	Ovarian Cancer	Ovarian	2.14E-13	-4.32E-01	6.51E-03	-3.63E-01
5068	A1451	GSE3149	Ovarian Cancer	Ovarian	7.12E-02	1.30E-01	3.76E-02	2.93E-01
5069	A1504	GSE3149	Ovarian Cancer	Ovarian	3.84E-05	-2.55E-01	7.65E-02	-2.59E-01
5070	A1526	GSE3149	Ovarian Cancer	Ovarian	6.17E-03	-1.81E-01	4.05E-01	1.54E-01
5071	A1552	GSE3149	Ovarian Cancer	Ovarian	1.50E-01	-1.10E-01	5.24E-02	2.77E-01
5072	A1578	GSE3149	Ovarian Cancer	Ovarian	5.30E-12	-4.08E-01	6.98E-04	-4.36E-01
5073	A1590	GSE3149	Ovarian Cancer	Ovarian	7.05E-03	-1.78E-01	3.55E-01	1.64E-01
5074	A1615	GSE3149	Ovarian Cancer	Ovarian	2.41E-01	9.55E-02	5.60E-02	2.74E-01
5075	A1623	GSE3149	Ovarian Cancer	Ovarian	1.62E-17	-4.98E-01	4.35E-04	-4.50E-01
5076	A1665	GSE3149	Ovarian Cancer	Ovarian	9.33E-10	-3.65E-01	1.46E-02	-3.32E-01
5077	A1674	GSE3149	Ovarian Cancer	Ovarian	3.30E-02	-1.48E-01	2.49E-01	1.90E-01
5078	A1675	GSE3149	Ovarian Cancer	Ovarian	2.12E-02	-1.57E-01	3.49E-01	1.66E-01
5079	A1774	GSE3149	Ovarian Cancer	Ovarian	3.25E-03	-1.92E-01	5.86E-01	-1.18E-01
5080	A1784	GSE3149	Ovarian Cancer	Ovarian	2.48E-02	-1.54E-01	3.19E-01	1.73E-01
5081	A1834	GSE3149	Ovarian Cancer	Ovarian	2.98E-02	-1.50E-01	6.23E-02	2.69E-01
5082	A1846	GSE3149	Ovarian Cancer	Ovarian	8.56E-07	-2.99E-01	1.21E-01	-2.35E-01
5083	A1858	GSE3149	Ovarian Cancer	Ovarian	2.89E-01	-8.92E-02	2.00E-01	2.05E-01
5084	A1877	GSE3149	Ovarian Cancer	Ovarian	6.25E-03	1.80E-01	4.22E-03	3.78E-01
5085	A1913	GSE3149	Ovarian Cancer	Ovarian	8.32E-02	-1.26E-01	3.49E-01	1.66E-01
5086	A1929	GSE3149	Ovarian Cancer	Ovarian	1.38E-05	-2.68E-01	2.90E-01	-1.80E-01
5087	A2046	GSE3149	Ovarian Cancer	Ovarian	1.23E-07	3.19E-01	1.29E-06	5.95E-01
5088	A2063	GSE3149	Ovarian Cancer	Ovarian	5.69E-02	-1.35E-01	3.20E-02	3.00E-01
5089	A2064	GSE3149	Ovarian Cancer	Ovarian	1.25E-03	-2.07E-01	1.98E-01	-2.06E-01
5090	A2075	GSE3149	Ovarian Cancer	Ovarian	4.42E-02	-1.41E-01	3.84E-01	1.58E-01
5091	A2198	GSE3149	Ovarian Cancer	Ovarian	3.01E-01	8.77E-02	1.83E-02	3.23E-01
5092	A2204	GSE3149	Ovarian Cancer	Ovarian	8.16E-04	2.13E-01	7.88E-03	3.56E-01
5093	A2324	GSE3149	Ovarian Cancer	Ovarian	3.10E-02	1.49E-01	2.69E-03	3.93E-01
5094	A2419	GSE3149	Ovarian Cancer	Ovarian	1.19E-01	1.17E-01	6.48E-02	2.67E-01
5095	A2422	GSE3149	Ovarian Cancer	Ovarian	4.81E-02	-1.39E-01	3.46E-01	1.66E-01
5096	A2424	GSE3149	Ovarian Cancer	Ovarian	2.33E-01	9.65E-02	8.88E-02	2.51E-01
5097	A2465	GSE3149	Ovarian Cancer	Ovarian	1.37E-15	-4.68E-01	9.83E-05	-4.91E-01
5098	A2476	GSE3149	Ovarian Cancer	Ovarian	6.08E-01	-5.65E-02	4.74E-01	1.40E-01
5099	A2479	GSE3149	Ovarian Cancer	Ovarian	3.79E-03	-1.89E-01	5.39E-01	-1.27E-01
5100	A2505	GSE3149	Ovarian Cancer	Ovarian	5.42E-04	-2.19E-01	3.46E-01	1.66E-01
5101	A2542	GSE3149	Ovarian Cancer	Ovarian	1.29E-01	1.15E-01	3.76E-02	2.93E-01

5102	A2573	GSE3149	Ovarian Cancer	Ovarian	5.68E-01	6.02E-02	1.98E-01	2.06E-01
5103	A2673	GSE3149	Ovarian Cancer	Ovarian	3.48E-01	-8.23E-02	8.88E-02	2.51E-01
5104	A2739	GSE3149	Ovarian Cancer	Ovarian	8.04E-04	2.14E-01	2.11E-05	5.30E-01
5105	A2802	GSE3149	Ovarian Cancer	Ovarian	2.47E-03	-1.96E-01	5.96E-01	-1.16E-01
5106	A2849	GSE3149	Ovarian Cancer	Ovarian	8.91E-02	1.24E-01	6.17E-03	3.64E-01
5107	A2895	GSE3149	Ovarian Cancer	Ovarian	1.51E-05	-2.67E-01	1.40E-01	-2.27E-01
5108	A2967	GSE3149	Ovarian Cancer	Ovarian	1.56E-02	1.63E-01	1.59E-03	4.10E-01
5109	A2981	GSE3149	Ovarian Cancer	Ovarian	2.44E-01	-9.50E-02	1.79E-01	2.12E-01
5110	A2999	GSE3149	Ovarian Cancer	Ovarian	3.38E-01	8.33E-02	1.00E-01	2.45E-01
5111	A3018	GSE3149	Ovarian Cancer	Ovarian	1.48E-02	-1.64E-01	1.98E-01	-2.06E-01
5112	A3090	GSE3149	Ovarian Cancer	Ovarian	2.12E-02	-1.57E-01	3.27E-01	-1.71E-01
5113	A3102	GSE3149	Ovarian Cancer	Ovarian	8.25E-02	1.26E-01	1.34E-04	4.83E-01
5114	A3107	GSE3149	Ovarian Cancer	Ovarian	2.61E-04	-2.30E-01	3.61E-01	-1.63E-01
5115	A3142	GSE3149	Ovarian Cancer	Ovarian	1.01E-05	2.71E-01	4.47E-07	6.18E-01
5116	A3250	GSE3149	Ovarian Cancer	Ovarian	8.11E-02	-1.27E-01	3.27E-01	1.71E-01
5117	D1462	GSE3149	Ovarian Cancer	Ovarian	1.28E-01	1.15E-01	1.18E-01	2.36E-01
5118	D1805	GSE3149	Ovarian Cancer	Ovarian	1.69E-06	-2.92E-01	7.88E-03	-3.56E-01
5119	D1837	GSE3149	Ovarian Cancer	Ovarian	1.48E-03	-2.04E-01	5.83E-01	1.19E-01
5120	D1859	GSE3149	Ovarian Cancer	Ovarian	1.19E-13	-4.37E-01	6.73E-02	-2.65E-01
5121	D2098	GSE3149	Ovarian Cancer	Ovarian	5.54E-02	-1.36E-01	1.34E-01	2.29E-01
5122	D2147	GSE3149	Ovarian Cancer	Ovarian	1.58E-11	-3.99E-01	5.40E-04	-4.43E-01
5123	D2159	GSE3149	Ovarian Cancer	Ovarian	3.42E-03	-1.91E-01	2.51E-01	1.90E-01
5124	D2171	GSE3149	Ovarian Cancer	Ovarian	2.18E-02	-1.56E-01	2.80E-01	1.82E-01
5125	D2208	GSE3149	Ovarian Cancer	Ovarian	1.11E-01	1.19E-01	1.03E-01	2.44E-01
5126	D2247	GSE3149	Ovarian Cancer	Ovarian	4.67E-24	5.87E-01	7.78E-12	8.17E-01
5127	D2251	GSE3149	Ovarian Cancer	Ovarian	1.97E-05	-2.63E-01	2.63E-01	-1.87E-01
5128	D2287	GSE3149	Ovarian Cancer	Ovarian	3.88E-01	7.79E-02	7.46E-02	2.60E-01
5129	D2332	GSE3149	Ovarian Cancer	Ovarian	1.50E-01	-1.10E-01	2.56E-01	1.89E-01
5130	D2342	GSE3149	Ovarian Cancer	Ovarian	1.20E-24	5.94E-01	9.30E-11	7.77E-01
5131	D2358	GSE3149	Ovarian Cancer	Ovarian	1.26E-05	2.69E-01	2.05E-05	5.31E-01
5132	D2421	GSE3149	Ovarian Cancer	Ovarian	8.68E-12	4.04E-01	2.82E-08	6.74E-01
5133	D2432	GSE3149	Ovarian Cancer	Ovarian	4.67E-04	2.22E-01	1.28E-04	4.84E-01
5134	D2433	GSE3149	Ovarian Cancer	Ovarian	3.14E-01	-8.61E-02	1.37E-01	2.28E-01
5135	D2443	GSE3149	Ovarian Cancer	Ovarian	2.23E-08	3.36E-01	5.58E-06	5.62E-01
5136	D2480	GSE3149	Ovarian Cancer	Ovarian	1.32E-02	1.66E-01	5.16E-03	3.71E-01
5137	D2528	GSE3149	Ovarian Cancer	Ovarian	5.33E-31	-6.68E-01	1.27E-08	-6.89E-01
5138	D2557	GSE3149	Ovarian Cancer	Ovarian	7.58E-07	3.00E-01	5.58E-06	5.62E-01
5139	D2559	GSE3149	Ovarian Cancer	Ovarian	4.73E-01	-6.93E-02	1.06E-01	2.42E-01
5140	D2560	GSE3149	Ovarian Cancer	Ovarian	3.50E-02	-1.47E-01	2.22E-01	1.98E-01
5141	D2572	GSE3149	Ovarian Cancer	Ovarian	2.78E-03	-1.94E-01	2.80E-01	1.82E-01
5142	D2575	GSE3149	Ovarian Cancer	Ovarian	1.77E-18	5.12E-01	1.40E-09	7.30E-01
5143	D2576	GSE3149	Ovarian Cancer	Ovarian	3.65E-05	2.56E-01	1.50E-05	5.38E-01
5144	D2578	GSE3149	Ovarian Cancer	Ovarian	2.57E-28	6.38E-01	1.25E-13	8.81E-01
5145	D2581	GSE3149	Ovarian Cancer	Ovarian	2.81E-10	-3.75E-01	9.68E-03	-3.48E-01
5146	D2603	GSE3149	Ovarian Cancer	Ovarian	3.26E-02	1.48E-01	1.94E-03	4.04E-01
5147	D2610	GSE3149	Ovarian Cancer	Ovarian	5.98E-05	-2.50E-01	2.95E-01	-1.78E-01
5148	D2611	GSE3149	Ovarian Cancer	Ovarian	3.88E-01	7.79E-02	4.70E-02	2.83E-01
5149	D2612	GSE3149	Ovarian Cancer	Ovarian	1.15E-02	1.69E-01	3.38E-03	3.85E-01
5150	D2622	GSE3149	Ovarian Cancer	Ovarian	7.27E-04	2.15E-01	2.28E-04	4.68E-01
5151	D2629	GSE3149	Ovarian Cancer	Ovarian	1.54E-01	-1.10E-01	2.80E-01	1.82E-01
5152	D2640	GSE3149	Ovarian Cancer	Ovarian	3.52E-01	8.18E-02	3.76E-02	2.93E-01
5153	D2648	GSE3149	Ovarian Cancer	Ovarian	9.43E-12	-4.03E-01	3.05E-04	-4.60E-01
5154	D2651	GSE3149	Ovarian Cancer	Ovarian	4.55E-02	-1.41E-01	7.55E-02	2.60E-01
5155	D2668	GSE3149	Ovarian Cancer	Ovarian	2.18E-02	1.56E-01	9.60E-05	4.91E-01
5156	D2679	GSE3149	Ovarian Cancer	Ovarian	8.28E-04	-2.13E-01	3.55E-01	-1.64E-01
5157	D2689	GSE3149	Ovarian Cancer	Ovarian	7.80E-06	2.74E-01	2.72E-06	5.78E-01
5158	D2691	GSE3149	Ovarian Cancer	Ovarian	1.79E-04	-2.35E-01	9.10E-02	-2.50E-01
5159	D2700	GSE3149	Ovarian Cancer	Ovarian	5.53E-03	-1.82E-01	4.03E-02	2.90E-01
5160	D2711	GSE3149	Ovarian Cancer	Ovarian	6.38E-08	-3.26E-01	6.28E-03	-3.64E-01
5161	D2726	GSE3149	Ovarian Cancer	Ovarian	1.04E-01	-1.20E-01	3.46E-01	1.66E-01
5162	D2727	GSE3149	Ovarian Cancer	Ovarian	5.50E-04	-2.19E-01	3.87E-01	-1.57E-01
5163	D2732	GSE3149	Ovarian Cancer	Ovarian	9.53E-09	-3.44E-01	1.83E-02	-3.23E-01
5164	D2733	GSE3149	Ovarian Cancer	Ovarian	2.45E-02	1.54E-01	1.50E-03	4.12E-01
5165	D2734	GSE3149	Ovarian Cancer	Ovarian	2.28E-03	-1.97E-01	4.20E-01	1.50E-01
5166	D2736	GSE3149	Ovarian Cancer	Ovarian	2.75E-01	9.09E-02	1.38E-01	2.27E-01
5167	D2738	GSE3149	Ovarian Cancer	Ovarian	3.12E-01	-8.64E-02	3.93E-01	1.56E-01
5168	D2749	GSE3149	Ovarian Cancer	Ovarian	9.69E-12	-4.03E-01	1.20E-02	-3.40E-01
5169	D2775	GSE3149	Ovarian Cancer	Ovarian	7.16E-04	-2.15E-01	3.46E-01	1.66E-01
5170	D2776	GSE3149	Ovarian Cancer	Ovarian	1.23E-03	-2.07E-01	5.19E-01	-1.31E-01
5171	D2791	GSE3149	Ovarian Cancer	Ovarian	4.53E-06	-2.81E-01	1.40E-01	-2.27E-01
5172	D2792	GSE3149	Ovarian Cancer	Ovarian	4.29E-02	-1.42E-01	1.80E-01	2.11E-01
5173	M1025	GSE3149	Ovarian Cancer	Ovarian	1.86E-08	3.38E-01	9.66E-07	6.01E-01
5174	M1054	GSE3149	Ovarian Cancer	Ovarian	3.06E-07	-3.10E-01	2.61E-01	-1.87E-01
5175	M1055	GSE3149	Ovarian Cancer	Ovarian	2.09E-09	3.58E-01	1.35E-07	6.43E-01

5176	M120	GSE3149	Ovarian Cancer	Ovarian	3.90E-02	1.44E-01	1.20E-02	3.40E-01
5177	M1241	GSE3149	Ovarian Cancer	Ovarian	1.28E-03	-2.06E-01	5.96E-01	-1.16E-01
5178	M1390	GSE3149	Ovarian Cancer	Ovarian	1.86E-03	-2.01E-01	4.29E-01	-1.49E-01
5179	M1503	GSE3149	Ovarian Cancer	Ovarian	3.00E-04	-2.28E-01	1.84E-01	-2.10E-01
5180	M1572	GSE3149	Ovarian Cancer	Ovarian	1.68E-04	-2.36E-01	1.05E-01	-2.43E-01
5181	M17	GSE3149	Ovarian Cancer	Ovarian	2.09E-07	-3.14E-01	6.39E-02	-2.68E-01
5182	M1777	GSE3149	Ovarian Cancer	Ovarian	1.46E-07	3.18E-01	5.58E-06	5.62E-01
5183	M1891	GSE3149	Ovarian Cancer	Ovarian	4.46E-04	2.22E-01	3.98E-04	4.52E-01
5184	M2070	GSE3149	Ovarian Cancer	Ovarian	2.31E-06	-2.88E-01	3.81E-01	-1.59E-01
5185	M2097	GSE3149	Ovarian Cancer	Ovarian	1.36E-01	-1.13E-01	2.02E-01	2.04E-01
5186	M2184	GSE3149	Ovarian Cancer	Ovarian	6.51E-05	-2.48E-01	1.05E-01	-2.43E-01
5187	M2437	GSE3149	Ovarian Cancer	Ovarian	1.24E-10	-3.82E-01	7.75E-03	-3.56E-01
5188	M2515	GSE3149	Ovarian Cancer	Ovarian	7.18E-02	-1.30E-01	1.38E-01	2.27E-01
5189	M2729	GSE3149	Ovarian Cancer	Ovarian	4.96E-15	-4.59E-01	7.44E-04	-4.34E-01
5190	M2807	GSE3149	Ovarian Cancer	Ovarian	3.56E-03	1.90E-01	9.17E-04	4.27E-01
5191	M3035	GSE3149	Ovarian Cancer	Ovarian	1.38E-09	-3.61E-01	4.57E-02	-2.84E-01
5192	M3142	GSE3149	Ovarian Cancer	Ovarian	3.30E-09	-3.54E-01	1.83E-02	-3.23E-01
5193	M337	GSE3149	Ovarian Cancer	Ovarian	6.81E-02	1.31E-01	1.98E-03	4.03E-01
5194	M3484	GSE3149	Ovarian Cancer	Ovarian	1.65E-03	2.03E-01	1.90E-03	4.04E-01
5195	M3514	GSE3149	Ovarian Cancer	Ovarian	1.33E-01	-1.14E-01	3.46E-01	1.66E-01
5196	M359	GSE3149	Ovarian Cancer	Ovarian	7.48E-04	2.15E-01	2.49E-03	3.96E-01
5197	M3627	GSE3149	Ovarian Cancer	Ovarian	6.80E-11	-3.87E-01	1.20E-02	-3.40E-01
5198	M4161	GSE3149	Ovarian Cancer	Ovarian	6.94E-02	-1.31E-01	3.58E-01	1.64E-01
5199	M4171	GSE3149	Ovarian Cancer	Ovarian	2.69E-20	-5.37E-01	8.16E-06	-5.53E-01
5200	M444	GSE3149	Ovarian Cancer	Ovarian	2.48E-05	-2.61E-01	4.64E-01	-1.42E-01
5201	M485	GSE3149	Ovarian Cancer	Ovarian	1.15E-05	-2.70E-01	6.39E-02	-2.68E-01
5202	M503	GSE3149	Ovarian Cancer	Ovarian	2.24E-01	9.78E-02	3.30E-02	2.98E-01
5203	M5668	GSE3149	Ovarian Cancer	Ovarian	1.42E-09	-3.61E-01	8.30E-03	-3.54E-01
5204	M5775	GSE3149	Ovarian Cancer	Ovarian	8.89E-04	-2.12E-01	6.50E-01	1.06E-01
5205	M6199	GSE3149	Ovarian Cancer	Ovarian	2.51E-12	-4.14E-01	9.56E-04	-4.26E-01
5206	M806	GSE3149	Ovarian Cancer	Ovarian	3.47E-03	-1.90E-01	2.36E-01	-1.94E-01
5207	M810	GSE3149	Ovarian Cancer	Ovarian	1.37E-02	1.66E-01	3.13E-03	3.88E-01
5208	GSM249714	GSE9891	Ovarian Cancer	Ovarian	3.68E-02	-1.45E-01	6.53E-01	-1.05E-01
5209	GSM249715	GSE9891	Ovarian Cancer	Ovarian	1.19E-13	-4.37E-01	5.07E-03	-3.71E-01
5210	GSM249716	GSE9891	Ovarian Cancer	Ovarian	6.84E-05	-2.48E-01	1.86E-01	-2.10E-01
5211	GSM249717	GSE9891	Ovarian Cancer	Ovarian	1.82E-01	-1.05E-01	1.24E-01	2.34E-01
5212	GSM249718	GSE9891	Ovarian Cancer	Ovarian	4.53E-06	-2.81E-01	2.68E-01	-1.85E-01
5213	GSM249719	GSE9891	Ovarian Cancer	Ovarian	3.55E-04	-2.26E-01	1.86E-01	-2.10E-01
5214	GSM249720	GSE9891	Ovarian Cancer	Ovarian	4.51E-01	7.14E-02	1.56E-01	2.20E-01
5215	GSM249721	GSE9891	Ovarian Cancer	Ovarian	5.98E-05	-2.50E-01	2.95E-01	-1.78E-01
5216	GSM249722	GSE9891	Ovarian Cancer	Ovarian	1.90E-11	-3.98E-01	9.56E-04	-4.26E-01
5217	GSM249723	GSE9891	Ovarian Cancer	Ovarian	5.34E-04	-2.20E-01	4.08E-01	-1.53E-01
5218	GSM249724	GSE9891	Ovarian Cancer	Ovarian	1.55E-07	-3.17E-01	1.21E-01	-2.35E-01
5219	GSM249725	GSE9891	Ovarian Cancer	Ovarian	9.74E-06	-2.72E-01	1.63E-01	-2.18E-01
5220	GSM249726	GSE9891	Ovarian Cancer	Ovarian	6.28E-04	2.17E-01	1.22E-03	4.18E-01
5221	GSM249727	GSE9891	Ovarian Cancer	Ovarian	8.30E-08	-3.23E-01	4.09E-02	-2.89E-01
5222	GSM249728	GSE9891	Ovarian Cancer	Ovarian	6.62E-05	-2.48E-01	2.90E-01	-1.80E-01
5223	GSM249729	GSE9891	Ovarian Cancer	Ovarian	2.40E-06	-2.88E-01	1.06E-01	-2.42E-01
5224	GSM249730	GSE9891	Ovarian Cancer	Ovarian	2.40E-05	-2.61E-01	2.25E-01	-1.97E-01
5225	GSM249731	GSE9891	Ovarian Cancer	Ovarian	2.96E-05	-2.58E-01	3.61E-01	-1.63E-01
5226	GSM249732	GSE9891	Ovarian Cancer	Ovarian	1.16E-03	-2.08E-01	2.80E-01	1.82E-01
5227	GSM249733	GSE9891	Ovarian Cancer	Ovarian	5.98E-01	5.74E-02	2.27E-01	1.97E-01
5228	GSM249734	GSE9891	Ovarian Cancer	Ovarian	1.31E-06	-2.95E-01	1.06E-01	-2.42E-01
5229	GSM249735	GSE9891	Ovarian Cancer	Ovarian	1.06E-03	2.10E-01	4.63E-02	2.83E-01
5230	GSM249736	GSE9891	Ovarian Cancer	Ovarian	6.52E-08	-3.26E-01	1.98E-03	-4.03E-01
5231	GSM249737	GSE9891	Ovarian Cancer	Ovarian	2.26E-01	9.76E-02	2.51E-01	1.90E-01
5232	GSM249738	GSE9891	Ovarian Cancer	Ovarian	4.53E-10	-3.71E-01	1.29E-02	-3.37E-01
5233	GSM249739	GSE9891	Ovarian Cancer	Ovarian	2.78E-02	-1.52E-01	5.83E-01	1.19E-01
5234	GSM249740	GSE9891	Ovarian Cancer	Ovarian	3.60E-12	4.11E-01	8.79E-10	7.38E-01
5235	GSM249741	GSE9891	Ovarian Cancer	Ovarian	1.28E-01	-1.15E-01	2.51E-01	1.90E-01
5236	GSM249742	GSE9891	Ovarian Cancer	Ovarian	4.98E-06	2.80E-01	7.01E-05	5.00E-01
5237	GSM249743	GSE9891	Ovarian Cancer	Ovarian	3.01E-01	8.77E-02	1.75E-01	2.13E-01
5238	GSM249744	GSE9891	Ovarian Cancer	Ovarian	4.04E-06	-2.82E-01	1.86E-01	-2.10E-01
5239	GSM249745	GSE9891	Ovarian Cancer	Ovarian	8.61E-02	-1.25E-01	3.49E-01	1.66E-01
5240	GSM249746	GSE9891	Ovarian Cancer	Ovarian	4.13E-12	-4.10E-01	3.97E-02	-2.90E-01
5241	GSM249747	GSE9891	Ovarian Cancer	Ovarian	5.18E-09	3.50E-01	1.35E-07	6.43E-01
5242	GSM249748	GSE9891	Ovarian Cancer	Ovarian	2.08E-03	1.99E-01	3.05E-04	4.60E-01
5243	GSM249749	GSE9891	Ovarian Cancer	Ovarian	4.46E-02	-1.41E-01	7.27E-02	2.62E-01
5244	GSM249750	GSE9891	Ovarian Cancer	Ovarian	1.46E-04	2.38E-01	7.01E-05	5.00E-01
5245	GSM249751	GSE9891	Ovarian Cancer	Ovarian	2.55E-01	9.35E-02	1.52E-01	2.22E-01
5246	GSM249752	GSE9891	Ovarian Cancer	Ovarian	1.92E-01	1.03E-01	9.10E-02	2.50E-01
5247	GSM249753	GSE9891	Ovarian Cancer	Ovarian	2.49E-01	-9.44E-02	2.25E-01	1.97E-01
5248	GSM249754	GSE9891	Ovarian Cancer	Ovarian	5.26E-03	1.83E-01	1.04E-05	5.47E-01
5249	GSM249755	GSE9891	Ovarian Cancer	Ovarian	7.31E-03	1.77E-01	6.51E-03	3.63E-01

5250	GSM249756	GSE9891	Ovarian Cancer	Ovarian	2.75E-01	-9.09E-02	2.90E-01	1.80E-01
5251	GSM249757	GSE9891	Ovarian Cancer	Ovarian	2.21E-01	9.83E-02	8.66E-02	2.53E-01
5252	GSM249758	GSE9891	Ovarian Cancer	Ovarian	3.29E-03	-1.91E-01	5.43E-01	1.26E-01
5253	GSM249759	GSE9891	Ovarian Cancer	Ovarian	1.18E-04	2.41E-01	1.37E-04	4.82E-01
5254	GSM249760	GSE9891	Ovarian Cancer	Ovarian	3.06E-05	-2.58E-01	3.33E-01	1.70E-01
5255	GSM249761	GSE9891	Ovarian Cancer	Ovarian	4.51E-02	1.41E-01	1.16E-02	3.41E-01
5256	GSM249762	GSE9891	Ovarian Cancer	Ovarian	1.14E-04	2.41E-01	7.74E-06	5.54E-01
5257	GSM249763	GSE9891	Ovarian Cancer	Ovarian	1.60E-03	-2.03E-01	5.43E-01	1.26E-01
5258	GSM249764	GSE9891	Ovarian Cancer	Ovarian	5.31E-05	2.51E-01	9.17E-04	4.27E-01
5259	GSM249765	GSE9891	Ovarian Cancer	Ovarian	5.39E-03	-1.83E-01	2.49E-01	1.90E-01
5260	GSM249766	GSE9891	Ovarian Cancer	Ovarian	2.23E-05	-2.62E-01	2.06E-01	-2.03E-01
5261	GSM249767	GSE9891	Ovarian Cancer	Ovarian	1.80E-01	1.05E-01	2.27E-02	3.14E-01
5262	GSM249768	GSE9891	Ovarian Cancer	Ovarian	4.59E-02	1.40E-01	2.59E-03	3.94E-01
5263	GSM249769	GSE9891	Ovarian Cancer	Ovarian	9.05E-08	-3.22E-01	6.31E-02	-2.69E-01
5264	GSM249770	GSE9891	Ovarian Cancer	Ovarian	4.77E-10	-3.71E-01	1.37E-01	-2.28E-01
5265	GSM249771	GSE9891	Ovarian Cancer	Ovarian	3.19E-04	2.27E-01	2.33E-04	4.67E-01
5266	GSM249773	GSE9891	Ovarian Cancer	Ovarian	6.46E-02	-1.32E-01	3.14E-01	1.74E-01
5267	GSM249774	GSE9891	Ovarian Cancer	Ovarian	9.97E-04	-2.10E-01	2.36E-01	-1.94E-01
5268	GSM249775	GSE9891	Ovarian Cancer	Ovarian	1.78E-08	-3.38E-01	3.07E-02	-3.02E-01
5269	GSM249776	GSE9891	Ovarian Cancer	Ovarian	1.94E-06	-2.90E-01	1.40E-01	-2.27E-01
5270	GSM249777	GSE9891	Ovarian Cancer	Ovarian	5.09E-02	-1.38E-01	5.43E-01	1.26E-01
5271	GSM249778	GSE9891	Ovarian Cancer	Ovarian	3.23E-05	-2.57E-01	4.61E-01	-1.42E-01
5272	GSM249779	GSE9891	Ovarian Cancer	Ovarian	2.95E-16	-4.79E-01	1.27E-03	-4.17E-01
5273	GSM249780	GSE9891	Ovarian Cancer	Ovarian	1.26E-05	-2.69E-01	3.66E-01	-1.62E-01
5274	GSM249781	GSE9891	Ovarian Cancer	Ovarian	1.20E-03	-2.08E-01	3.90E-01	-1.57E-01
5275	GSM249782	GSE9891	Ovarian Cancer	Ovarian	7.08E-05	2.47E-01	4.01E-06	5.70E-01
5276	GSM249783	GSE9891	Ovarian Cancer	Ovarian	7.37E-09	-3.46E-01	3.92E-02	-2.91E-01
5277	GSM249784	GSE9891	Ovarian Cancer	Ovarian	1.95E-01	1.02E-01	5.53E-02	2.75E-01
5278	GSM249785	GSE9891	Ovarian Cancer	Ovarian	2.65E-04	-2.30E-01	6.56E-02	-2.67E-01
5279	GSM249786	GSE9891	Ovarian Cancer	Ovarian	7.44E-05	-2.47E-01	6.65E-02	-2.66E-01
5280	GSM249788	GSE9891	Ovarian Cancer	Ovarian	1.66E-07	-3.16E-01	5.25E-03	-3.70E-01
5281	GSM249789	GSE9891	Ovarian Cancer	Ovarian	3.67E-06	-2.83E-01	1.79E-01	-2.12E-01
5282	GSM249790	GSE9891	Ovarian Cancer	Ovarian	2.31E-05	-2.61E-01	1.59E-01	-2.19E-01
5283	GSM249791	GSE9891	Ovarian Cancer	Ovarian	9.86E-05	-2.43E-01	5.56E-01	-1.24E-01
5284	GSM249792	GSE9891	Ovarian Cancer	Ovarian	1.44E-03	-2.05E-01	5.00E-01	1.35E-01
5285	GSM249793	GSE9891	Ovarian Cancer	Ovarian	2.65E-06	-2.87E-01	2.36E-01	-1.94E-01
5286	GSM249794	GSE9891	Ovarian Cancer	Ovarian	1.12E-08	-3.42E-01	3.30E-02	-2.98E-01
5287	GSM249795	GSE9891	Ovarian Cancer	Ovarian	1.64E-01	1.08E-01	9.90E-02	2.46E-01
5288	GSM249796	GSE9891	Ovarian Cancer	Ovarian	2.17E-04	-2.32E-01	2.85E-01	-1.81E-01
5289	GSM249797	GSE9891	Ovarian Cancer	Ovarian	1.01E-01	1.21E-01	3.81E-02	2.92E-01
5290	GSM249798	GSE9891	Ovarian Cancer	Ovarian	6.25E-03	1.80E-01	4.15E-03	3.78E-01
5291	GSM249799	GSE9891	Ovarian Cancer	Ovarian	5.50E-04	-2.19E-01	4.23E-01	-1.50E-01
5292	GSM249800	GSE9891	Ovarian Cancer	Ovarian	3.46E-05	-2.56E-01	8.99E-02	-2.51E-01
5293	GSM249801	GSE9891	Ovarian Cancer	Ovarian	2.18E-02	-1.56E-01	2.90E-01	1.80E-01
5294	GSM249802	GSE9891	Ovarian Cancer	Ovarian	2.50E-15	-4.64E-01	1.62E-03	-4.10E-01
5295	GSM249803	GSE9891	Ovarian Cancer	Ovarian	3.26E-02	1.48E-01	2.68E-02	3.07E-01
5296	GSM249804	GSE9891	Ovarian Cancer	Ovarian	1.50E-03	-2.04E-01	1.80E-01	-2.11E-01
5297	GSM249805	GSE9891	Ovarian Cancer	Ovarian	6.81E-02	1.31E-01	1.49E-02	3.31E-01
5298	GSM249806	GSE9891	Ovarian Cancer	Ovarian	2.18E-08	3.36E-01	4.61E-07	6.17E-01
5299	GSM249807	GSE9891	Ovarian Cancer	Ovarian	9.49E-03	-1.73E-01	1.79E-01	2.12E-01
5300	GSM249808	GSE9891	Ovarian Cancer	Ovarian	6.32E-03	-1.80E-01	3.99E-01	-1.55E-01
5301	GSM249809	GSE9891	Ovarian Cancer	Ovarian	2.43E-02	1.54E-01	1.50E-03	4.12E-01
5302	GSM249810	GSE9891	Ovarian Cancer	Ovarian	3.33E-02	1.48E-01	7.74E-02	2.58E-01
5303	GSM249811	GSE9891	Ovarian Cancer	Ovarian	2.56E-02	-1.53E-01	1.77E-01	2.13E-01
5304	GSM249812	GSE9891	Ovarian Cancer	Ovarian	1.08E-02	-1.70E-01	4.70E-01	1.40E-01
5305	GSM249813	GSE9891	Ovarian Cancer	Ovarian	7.76E-03	1.76E-01	3.19E-03	3.87E-01
5306	GSM249814	GSE9891	Ovarian Cancer	Ovarian	4.19E-09	3.52E-01	3.80E-06	5.71E-01
5307	GSM249815	GSE9891	Ovarian Cancer	Ovarian	1.34E-02	1.66E-01	1.16E-02	3.41E-01
5308	GSM249816	GSE9891	Ovarian Cancer	Ovarian	4.09E-02	1.43E-01	1.24E-02	3.38E-01
5309	GSM249817	GSE9891	Ovarian Cancer	Ovarian	2.49E-01	9.44E-02	3.76E-02	2.93E-01
5310	GSM249818	GSE9891	Ovarian Cancer	Ovarian	1.62E-08	3.39E-01	7.53E-06	5.55E-01
5311	GSM249819	GSE9891	Ovarian Cancer	Ovarian	5.90E-06	-2.78E-01	4.36E-01	-1.47E-01
5312	GSM249820	GSE9891	Ovarian Cancer	Ovarian	2.27E-01	-9.74E-02	3.81E-02	2.92E-01
5313	GSM249821	GSE9891	Ovarian Cancer	Ovarian	7.28E-07	3.01E-01	5.23E-05	5.07E-01
5314	GSM249822	GSE9891	Ovarian Cancer	Ovarian	5.80E-02	-1.35E-01	3.52E-01	1.65E-01
5315	GSM249823	GSE9891	Ovarian Cancer	Ovarian	6.99E-14	4.40E-01	2.73E-08	6.74E-01
5316	GSM249824	GSE9891	Ovarian Cancer	Ovarian	5.89E-01	-5.82E-02	3.22E-01	1.72E-01
5317	GSM249825	GSE9891	Ovarian Cancer	Ovarian	1.71E-01	-1.06E-01	7.74E-02	2.58E-01
5318	GSM249826	GSE9891	Ovarian Cancer	Ovarian	6.75E-04	-2.16E-01	5.03E-01	1.34E-01
5319	GSM249827	GSE9891	Ovarian Cancer	Ovarian	3.33E-02	1.48E-01	7.36E-02	2.61E-01
5320	GSM249828	GSE9891	Ovarian Cancer	Ovarian	1.56E-01	1.09E-01	2.20E-02	3.16E-01
5321	GSM249829	GSE9891	Ovarian Cancer	Ovarian	1.52E-03	2.04E-01	7.13E-04	4.35E-01
5322	GSM249830	GSE9891	Ovarian Cancer	Ovarian	2.45E-02	1.54E-01	7.27E-02	2.62E-01
5323	GSM249831	GSE9891	Ovarian Cancer	Ovarian	2.12E-02	1.57E-01	6.39E-03	3.63E-01

5324	GSM249832	GSE9891	Ovarian Cancer	Ovarian	3.05E-12	-4.12E-01	4.30E-03	-3.77E-01
5325	GSM249833	GSE9891	Ovarian Cancer	Ovarian	2.12E-05	-2.63E-01	1.77E-01	-2.13E-01
5326	GSM249834	GSE9891	Ovarian Cancer	Ovarian	1.19E-02	1.68E-01	2.54E-03	3.95E-01
5327	GSM249835	GSE9891	Ovarian Cancer	Ovarian	1.54E-01	1.10E-01	6.28E-03	3.64E-01
5328	GSM249836	GSE9891	Ovarian Cancer	Ovarian	1.29E-08	3.41E-01	2.72E-06	5.78E-01
5329	GSM249837	GSE9891	Ovarian Cancer	Ovarian	1.66E-06	-2.92E-01	1.05E-01	-2.43E-01
5330	GSM249838	GSE9891	Ovarian Cancer	Ovarian	5.54E-02	1.36E-01	9.52E-03	3.49E-01
5331	GSM249839	GSE9891	Ovarian Cancer	Ovarian	1.54E-03	-2.04E-01	5.83E-01	1.19E-01
5332	GSM249840	GSE9891	Ovarian Cancer	Ovarian	1.25E-07	3.19E-01	1.37E-06	5.94E-01
5333	GSM249841	GSE9891	Ovarian Cancer	Ovarian	7.91E-09	-3.46E-01	7.65E-02	-2.59E-01
5334	GSM249842	GSE9891	Ovarian Cancer	Ovarian	1.45E-01	-1.11E-01	2.61E-02	3.09E-01
5335	GSM249843	GSE9891	Ovarian Cancer	Ovarian	5.56E-09	3.49E-01	9.57E-08	6.50E-01
5336	GSM249844	GSE9891	Ovarian Cancer	Ovarian	9.46E-08	-3.22E-01	6.56E-02	-2.67E-01
5337	GSM249845	GSE9891	Ovarian Cancer	Ovarian	6.88E-03	1.79E-01	3.05E-04	4.60E-01
5338	GSM249846	GSE9891	Ovarian Cancer	Ovarian	1.56E-05	2.66E-01	1.77E-04	4.75E-01
5339	GSM249847	GSE9891	Ovarian Cancer	Ovarian	3.52E-13	-4.29E-01	6.74E-03	-3.61E-01
5340	GSM249848	GSE9891	Ovarian Cancer	Ovarian	4.11E-05	2.54E-01	3.98E-05	5.14E-01
5341	GSM249849	GSE9891	Ovarian Cancer	Ovarian	8.18E-02	1.27E-01	1.15E-01	2.37E-01
5342	GSM249850	GSE9891	Ovarian Cancer	Ovarian	6.71E-09	-3.47E-01	4.15E-03	-3.78E-01
5343	GSM249851	GSE9891	Ovarian Cancer	Ovarian	2.18E-01	-9.87E-02	3.84E-01	1.58E-01
5344	GSM249852	GSE9891	Ovarian Cancer	Ovarian	8.32E-02	-1.26E-01	3.14E-01	1.74E-01
5345	GSM249853	GSE9891	Ovarian Cancer	Ovarian	6.58E-02	1.32E-01	9.56E-04	4.26E-01
5346	GSM249854	GSE9891	Ovarian Cancer	Ovarian	2.23E-10	3.77E-01	6.13E-08	6.58E-01
5347	GSM249855	GSE9891	Ovarian Cancer	Ovarian	9.47E-07	2.98E-01	2.16E-05	5.30E-01
5348	GSM249856	GSE9891	Ovarian Cancer	Ovarian	3.72E-09	-3.53E-01	1.51E-02	-3.31E-01
5349	GSM249857	GSE9891	Ovarian Cancer	Ovarian	2.96E-01	-8.83E-02	3.14E-01	1.74E-01
5350	GSM249858	GSE9891	Ovarian Cancer	Ovarian	1.69E-06	2.92E-01	2.72E-05	5.24E-01
5351	GSM249859	GSE9891	Ovarian Cancer	Ovarian	3.04E-02	1.50E-01	7.75E-03	3.56E-01
5352	GSM249860	GSE9891	Ovarian Cancer	Ovarian	1.39E-04	2.39E-01	3.05E-04	4.60E-01
5353	GSM249861	GSE9891	Ovarian Cancer	Ovarian	1.60E-10	-3.80E-01	8.16E-03	-3.54E-01
5354	GSM249862	GSE9891	Ovarian Cancer	Ovarian	1.90E-02	-1.59E-01	5.93E-01	-1.17E-01
5355	GSM249863	GSE9891	Ovarian Cancer	Ovarian	2.66E-05	2.60E-01	1.85E-04	4.74E-01
5356	GSM249864	GSE9891	Ovarian Cancer	Ovarian	2.60E-03	1.95E-01	7.59E-04	4.33E-01
5357	GSM249865	GSE9891	Ovarian Cancer	Ovarian	7.81E-04	2.14E-01	1.17E-03	4.20E-01
5358	GSM249866	GSE9891	Ovarian Cancer	Ovarian	3.01E-05	-2.58E-01	3.24E-01	-1.71E-01
5359	GSM249867	GSE9891	Ovarian Cancer	Ovarian	1.81E-03	2.01E-01	9.17E-04	4.27E-01
5360	GSM249868	GSE9891	Ovarian Cancer	Ovarian	2.08E-05	2.63E-01	3.88E-05	5.15E-01
5361	GSM249869	GSE9891	Ovarian Cancer	Ovarian	2.78E-03	-1.94E-01	5.53E-01	-1.24E-01
5362	GSM249870	GSE9891	Ovarian Cancer	Ovarian	2.35E-10	-3.77E-01	1.89E-02	-3.22E-01
5363	GSM249871	GSE9891	Ovarian Cancer	Ovarian	2.02E-06	2.90E-01	4.07E-04	4.51E-01
5364	GSM249872	GSE9891	Ovarian Cancer	Ovarian	1.20E-04	2.40E-01	5.40E-04	4.43E-01
5365	GSM249873	GSE9891	Ovarian Cancer	Ovarian	1.74E-01	-1.06E-01	4.20E-01	1.50E-01
5366	GSM249874	GSE9891	Ovarian Cancer	Ovarian	4.34E-01	-7.32E-02	2.56E-01	1.89E-01
5367	GSM249875	GSE9891	Ovarian Cancer	Ovarian	5.85E-02	-1.35E-01	3.84E-01	1.58E-01
5368	GSM249876	GSE9891	Ovarian Cancer	Ovarian	9.46E-08	3.22E-01	2.00E-05	5.31E-01
5369	GSM249877	GSE9891	Ovarian Cancer	Ovarian	3.42E-01	8.29E-02	5.24E-02	2.77E-01
5370	GSM249878	GSE9891	Ovarian Cancer	Ovarian	2.39E-08	-3.35E-01	2.66E-01	-1.86E-01
5371	GSM249879	GSE9891	Ovarian Cancer	Ovarian	4.25E-02	1.42E-01	6.23E-02	2.69E-01
5372	GSM249880	GSE9891	Ovarian Cancer	Ovarian	2.76E-14	-4.47E-01	1.12E-05	-5.45E-01
5373	GSM249881	GSE9891	Ovarian Cancer	Ovarian	1.56E-02	-1.63E-01	6.50E-01	1.06E-01
5374	GSM249882	GSE9891	Ovarian Cancer	Ovarian	1.65E-05	-2.66E-01	3.27E-01	-1.71E-01
5375	GSM249883	GSE9891	Ovarian Cancer	Ovarian	2.96E-05	-2.58E-01	2.08E-01	-2.03E-01
5376	GSM249884	GSE9891	Ovarian Cancer	Ovarian	3.91E-05	-2.55E-01	2.58E-01	-1.88E-01
5377	GSM249885	GSE9891	Ovarian Cancer	Ovarian	4.64E-02	1.40E-01	1.49E-02	3.31E-01
5378	GSM249886	GSE9891	Ovarian Cancer	Ovarian	8.22E-19	-5.16E-01	9.36E-04	-4.27E-01
5379	GSM249887	GSE9891	Ovarian Cancer	Ovarian	5.14E-02	-1.38E-01	1.54E-01	2.21E-01
5380	GSM249888	GSE9891	Ovarian Cancer	Ovarian	7.81E-04	2.14E-01	9.36E-04	4.27E-01
5381	GSM249889	GSE9891	Ovarian Cancer	Ovarian	1.10E-01	-1.19E-01	2.51E-01	1.90E-01
5382	GSM249890	GSE9891	Ovarian Cancer	Ovarian	2.06E-06	2.90E-01	9.60E-05	4.91E-01
5383	GSM249891	GSE9891	Ovarian Cancer	Ovarian	1.10E-02	-1.70E-01	2.49E-01	-1.90E-01
5384	GSM249892	GSE9891	Ovarian Cancer	Ovarian	2.71E-05	-2.60E-01	3.58E-01	-1.64E-01
5385	GSM249893	GSE9891	Ovarian Cancer	Ovarian	6.01E-06	-2.77E-01	3.76E-02	-2.93E-01
5386	GSM249894	GSE9891	Ovarian Cancer	Ovarian	2.20E-04	-2.32E-01	1.22E-01	-2.34E-01
5387	GSM249895	GSE9891	Ovarian Cancer	Ovarian	4.94E-03	1.84E-01	1.17E-03	4.20E-01
5388	GSM249896	GSE9891	Ovarian Cancer	Ovarian	6.94E-02	1.31E-01	9.52E-03	3.49E-01
5389	GSM249897	GSE9891	Ovarian Cancer	Ovarian	3.60E-03	-1.90E-01	4.36E-01	-1.47E-01
5390	GSM249898	GSE9891	Ovarian Cancer	Ovarian	5.58E-04	-2.19E-01	1.63E-01	-2.18E-01
5391	GSM249899	GSE9891	Ovarian Cancer	Ovarian	4.55E-02	-1.41E-01	5.13E-01	1.32E-01
5392	GSM249900	GSE9891	Ovarian Cancer	Ovarian	2.03E-02	-1.58E-01	4.20E-01	1.50E-01
5393	GSM249901	GSE9891	Ovarian Cancer	Ovarian	1.85E-33	-6.95E-01	8.95E-11	-7.77E-01
5394	GSM249902	GSE9891	Ovarian Cancer	Ovarian	2.07E-02	-1.58E-01	3.14E-01	1.74E-01
5395	GSM249903	GSE9891	Ovarian Cancer	Ovarian	2.01E-01	-1.01E-01	1.32E-01	2.30E-01
5396	GSM249904	GSE9891	Ovarian Cancer	Ovarian	1.65E-04	-2.36E-01	4.77E-01	-1.39E-01
5397	GSM249905	GSE9891	Ovarian Cancer	Ovarian	1.64E-12	-4.17E-01	1.31E-04	-4.83E-01

5398	GSM249906	GSE9891	Ovarian Cancer	Ovarian	6.48E-03	1.80E-01	1.42E-02	3.33E-01
5399	GSM249907	GSE9891	Ovarian Cancer	Ovarian	4.59E-02	1.40E-01	5.38E-02	2.76E-01
5400	GSM249908	GSE9891	Ovarian Cancer	Ovarian	2.28E-02	-1.56E-01	7.86E-01	-7.94E-02
5401	GSM249909	GSE9891	Ovarian Cancer	Ovarian	1.73E-02	1.61E-01	4.22E-03	3.78E-01
5402	GSM249910	GSE9891	Ovarian Cancer	Ovarian	8.41E-06	-2.74E-01	4.63E-02	-2.83E-01
5403	GSM249911	GSE9891	Ovarian Cancer	Ovarian	3.79E-03	1.89E-01	1.77E-04	4.75E-01
5404	GSM249912	GSE9891	Ovarian Cancer	Ovarian	2.53E-04	-2.30E-01	4.58E-01	-1.43E-01
5405	GSM249913	GSE9891	Ovarian Cancer	Ovarian	3.91E-08	3.31E-01	7.74E-06	5.54E-01
5406	GSM249914	GSE9891	Ovarian Cancer	Ovarian	1.39E-04	2.39E-01	5.40E-04	4.43E-01
5407	GSM249915	GSE9891	Ovarian Cancer	Ovarian	2.38E-02	1.55E-01	4.76E-02	2.82E-01
5408	GSM249916	GSE9891	Ovarian Cancer	Ovarian	1.06E-01	1.20E-01	4.07E-03	3.79E-01
5409	GSM249917	GSE9891	Ovarian Cancer	Ovarian	2.26E-02	1.56E-01	7.54E-05	4.98E-01
5410	GSM249918	GSE9891	Ovarian Cancer	Ovarian	2.74E-03	-1.94E-01	2.33E-01	-1.95E-01
5411	GSM249919	GSE9891	Ovarian Cancer	Ovarian	8.91E-02	-1.24E-01	2.51E-01	1.90E-01
5412	GSM249920	GSE9891	Ovarian Cancer	Ovarian	1.74E-03	2.02E-01	1.90E-03	4.04E-01
5413	GSM249921	GSE9891	Ovarian Cancer	Ovarian	1.90E-06	-2.90E-01	1.51E-01	-2.22E-01
5414	GSM249922	GSE9891	Ovarian Cancer	Ovarian	1.16E-01	-1.18E-01	3.38E-01	-1.68E-01
5415	GSM249923	GSE9891	Ovarian Cancer	Ovarian	3.69E-01	7.99E-02	6.23E-02	2.69E-01
5416	GSM249924	GSE9891	Ovarian Cancer	Ovarian	4.09E-02	1.43E-01	1.18E-01	2.36E-01
5417	GSM249925	GSE9891	Ovarian Cancer	Ovarian	6.96E-04	2.16E-01	4.89E-03	3.73E-01
5418	GSM249926	GSE9891	Ovarian Cancer	Ovarian	4.58E-08	-3.29E-01	6.56E-02	-2.67E-01
5419	GSM249927	GSE9891	Ovarian Cancer	Ovarian	1.76E-01	-1.05E-01	1.54E-01	2.21E-01
5420	GSM249928	GSE9891	Ovarian Cancer	Ovarian	8.48E-09	-3.45E-01	4.30E-03	-3.77E-01
5421	GSM249929	GSE9891	Ovarian Cancer	Ovarian	8.14E-03	-1.76E-01	6.03E-01	-1.15E-01
5422	GSM249930	GSE9891	Ovarian Cancer	Ovarian	1.07E-04	-2.42E-01	3.66E-01	-1.62E-01
5423	GSM249931	GSE9891	Ovarian Cancer	Ovarian	2.13E-08	3.36E-01	9.12E-07	6.03E-01
5424	GSM249932	GSE9891	Ovarian Cancer	Ovarian	1.05E-02	-1.71E-01	5.43E-01	1.26E-01
5425	GSM249933	GSE9891	Ovarian Cancer	Ovarian	6.40E-02	-1.33E-01	4.20E-01	1.50E-01
5426	GSM249934	GSE9891	Ovarian Cancer	Ovarian	4.53E-06	-2.81E-01	2.61E-01	-1.87E-01
5427	GSM249935	GSE9891	Ovarian Cancer	Ovarian	2.38E-01	9.59E-02	3.20E-02	3.00E-01
5428	GSM249936	GSE9891	Ovarian Cancer	Ovarian	4.20E-03	-1.87E-01	2.00E-01	-2.05E-01
5429	GSM249937	GSE9891	Ovarian Cancer	Ovarian	9.71E-03	-1.72E-01	5.00E-01	1.35E-01
5430	GSM249938	GSE9891	Ovarian Cancer	Ovarian	1.48E-04	-2.38E-01	1.00E-01	-2.45E-01
5431	GSM249939	GSE9891	Ovarian Cancer	Ovarian	8.08E-05	-2.46E-01	1.37E-01	-2.28E-01
5432	GSM249940	GSE9891	Ovarian Cancer	Ovarian	1.47E-06	2.93E-01	1.81E-04	4.74E-01
5433	GSM249941	GSE9891	Ovarian Cancer	Ovarian	3.12E-07	-3.10E-01	1.56E-02	-3.30E-01
5434	GSM249942	GSE9891	Ovarian Cancer	Ovarian	1.40E-03	-2.05E-01	2.25E-01	-1.97E-01
5435	GSM249943	GSE9891	Ovarian Cancer	Ovarian	2.07E-04	-2.33E-01	7.55E-02	-2.60E-01
5436	GSM249944	GSE9891	Ovarian Cancer	Ovarian	2.78E-03	-1.94E-01	2.68E-01	1.85E-01
5437	GSM249945	GSE9891	Ovarian Cancer	Ovarian	1.57E-01	1.09E-01	4.51E-02	2.84E-01
5438	GSM249946	GSE9891	Ovarian Cancer	Ovarian	2.26E-01	9.76E-02	1.18E-01	2.36E-01
5439	GSM249947	GSE9891	Ovarian Cancer	Ovarian	5.92E-04	2.18E-01	5.28E-04	4.44E-01
5440	GSM249948	GSE9891	Ovarian Cancer	Ovarian	2.23E-01	-9.81E-02	4.51E-02	2.84E-01
5441	GSM249949	GSE9891	Ovarian Cancer	Ovarian	4.26E-05	-2.54E-01	2.61E-02	-3.09E-01
5442	GSM249950	GSE9891	Ovarian Cancer	Ovarian	6.85E-04	2.16E-01	1.77E-04	4.75E-01
5443	GSM249951	GSE9891	Ovarian Cancer	Ovarian	4.51E-02	-1.41E-01	4.58E-01	1.43E-01
5444	GSM249953	GSE9891	Ovarian Cancer	Ovarian	8.18E-02	-1.27E-01	2.22E-01	1.98E-01
5445	GSM249954	GSE9891	Ovarian Cancer	Ovarian	6.73E-06	-2.76E-01	2.06E-01	-2.03E-01
5446	GSM249955	GSE9891	Ovarian Cancer	Ovarian	3.66E-04	-2.25E-01	1.98E-01	-2.06E-01
5447	GSM249956	GSE9891	Ovarian Cancer	Ovarian	8.76E-02	1.25E-01	7.88E-03	3.56E-01
5448	GSM249957	GSE9891	Ovarian Cancer	Ovarian	1.43E-01	-1.12E-01	1.82E-01	2.11E-01
5449	GSM249958	GSE9891	Ovarian Cancer	Ovarian	6.97E-03	-1.78E-01	3.61E-01	1.63E-01
5450	GSM249959	GSE9891	Ovarian Cancer	Ovarian	6.20E-14	-4.41E-01	4.18E-05	-5.13E-01
5451	GSM249960	GSE9891	Ovarian Cancer	Ovarian	6.84E-05	-2.48E-01	4.70E-01	-1.40E-01
5452	GSM249961	GSE9891	Ovarian Cancer	Ovarian	2.47E-03	-1.96E-01	5.13E-01	-1.32E-01
5453	GSM249962	GSE9891	Ovarian Cancer	Ovarian	3.42E-01	8.29E-02	2.17E-02	3.16E-01
5454	GSM249963	GSE9891	Ovarian Cancer	Ovarian	2.78E-03	1.94E-01	3.13E-03	3.88E-01
5455	GSM249964	GSE9891	Ovarian Cancer	Ovarian	1.07E-05	2.71E-01	3.98E-05	5.14E-01
5456	GSM249965	GSE9891	Ovarian Cancer	Ovarian	4.72E-02	1.40E-01	2.45E-03	3.96E-01
5457	GSM249966	GSE9891	Ovarian Cancer	Ovarian	1.72E-09	-3.60E-01	1.24E-01	-2.34E-01
5458	GSM249967	GSE9891	Ovarian Cancer	Ovarian	9.22E-06	-2.73E-01	1.35E-01	-2.29E-01
5459	GSM249968	GSE9891	Ovarian Cancer	Ovarian	3.77E-07	-3.08E-01	4.70E-02	-2.83E-01
5460	GSM249969	GSE9891	Ovarian Cancer	Ovarian	2.92E-01	-8.87E-02	9.90E-02	2.46E-01
5461	GSM249970	GSE9891	Ovarian Cancer	Ovarian	6.62E-13	4.24E-01	2.21E-09	7.21E-01
5462	GSM249971	GSE9891	Ovarian Cancer	Ovarian	5.20E-03	-1.84E-01	3.30E-01	-1.70E-01
5463	GSM249972	GSE9891	Ovarian Cancer	Ovarian	8.30E-08	-3.23E-01	1.44E-01	-2.25E-01
5464	GSM249973	GSE9891	Ovarian Cancer	Ovarian	6.60E-10	-3.68E-01	4.03E-02	-2.90E-01
5465	GSM249974	GSE9891	Ovarian Cancer	Ovarian	3.01E-05	-2.58E-01	4.36E-01	-1.47E-01
5466	GSM249975	GSE9891	Ovarian Cancer	Ovarian	7.64E-02	-1.28E-01	2.80E-01	1.82E-01
5467	GSM249976	GSE9891	Ovarian Cancer	Ovarian	1.43E-05	-2.67E-01	4.02E-01	-1.54E-01
5468	GSM249977	GSE9891	Ovarian Cancer	Ovarian	2.33E-01	9.65E-02	1.04E-01	2.43E-01
5469	GSM249978	GSE9891	Ovarian Cancer	Ovarian	6.80E-03	1.79E-01	9.36E-04	4.27E-01
5470	GSM249979	GSE9891	Ovarian Cancer	Ovarian	2.40E-02	1.55E-01	3.11E-02	3.01E-01
5471	GSM249980	GSE9891	Ovarian Cancer	Ovarian	1.51E-02	-1.64E-01	2.68E-01	1.85E-01

5472	GSM249981	GSE9891	Ovarian Cancer	Ovarian	2.08E-08	-3.37E-01	7.74E-02	-2.58E-01
5473	GSM249982	GSE9891	Ovarian Cancer	Ovarian	9.15E-04	2.12E-01	6.98E-04	4.36E-01
5474	GSM249983	GSE9891	Ovarian Cancer	Ovarian	6.28E-04	-2.17E-01	1.24E-01	-2.34E-01
5475	GSM249984	GSE9891	Ovarian Cancer	Ovarian	4.95E-02	1.39E-01	1.01E-01	2.44E-01
5476	GSM249985	GSE9891	Ovarian Cancer	Ovarian	3.54E-07	-3.08E-01	9.21E-02	-2.50E-01
5477	GSM249986	GSE9891	Ovarian Cancer	Ovarian	4.08E-04	-2.24E-01	2.58E-01	-1.88E-01
5478	GSM249987	GSE9891	Ovarian Cancer	Ovarian	1.45E-09	-3.61E-01	4.07E-03	-3.79E-01
5479	GSM249988	GSE9891	Ovarian Cancer	Ovarian	2.47E-03	1.96E-01	3.92E-03	3.80E-01
5480	GSM249989	GSE9891	Ovarian Cancer	Ovarian	1.09E-01	1.19E-01	1.32E-01	2.30E-01
5481	GSM249990	GSE9891	Ovarian Cancer	Ovarian	1.71E-01	1.06E-01	4.45E-02	2.85E-01
5482	GSM249991	GSE9891	Ovarian Cancer	Ovarian	2.44E-03	-1.96E-01	7.10E-01	-9.46E-02
5483	GSM249992	GSE9891	Ovarian Cancer	Ovarian	2.91E-01	8.90E-02	7.46E-02	2.60E-01
5484	GSM249993	GSE9891	Ovarian Cancer	Ovarian	3.51E-03	-1.90E-01	6.10E-01	-1.14E-01
5485	GSM249994	GSE9891	Ovarian Cancer	Ovarian	6.18E-02	-1.34E-01	5.31E-02	2.77E-01
5486	GSM249995	GSE9891	Ovarian Cancer	Ovarian	1.12E-08	-3.42E-01	1.02E-02	-3.46E-01
5487	GSM249996	GSE9891	Ovarian Cancer	Ovarian	1.38E-13	-4.35E-01	7.44E-04	-4.34E-01
5488	GSM249997	GSE9891	Ovarian Cancer	Ovarian	5.04E-07	3.05E-01	2.94E-05	5.22E-01
5489	GSM249998	GSE9891	Ovarian Cancer	Ovarian	5.97E-11	-3.88E-01	1.86E-02	-3.23E-01
5490	GSM249999	GSE9891	Ovarian Cancer	Ovarian	2.87E-01	-8.94E-02	6.73E-01	1.02E-01
5491	GSM250000	GSE9891	Ovarian Cancer	Ovarian	1.70E-01	1.06E-01	8.66E-02	2.53E-01
5492	GSM250001	GSE9891	Ovarian Cancer	Ovarian	3.17E-03	1.92E-01	7.88E-03	3.56E-01
5493	GSM102426	ExpO	Ovarian Cancer	Ovarian	1.11E-16	-4.85E-01	2.72E-06	-5.78E-01
5494	GSM102445	ExpO	Ovarian Cancer	Ovarian	8.18E-02	1.27E-01	3.86E-02	2.91E-01
5495	GSM102446	ExpO	Ovarian Cancer	Ovarian	1.85E-11	3.98E-01	1.23E-08	6.90E-01
5496	GSM102478	ExpO	Ovarian Cancer	Ovarian	8.88E-10	-3.65E-01	9.85E-03	-3.47E-01
5497	GSM102483	ExpO	Ovarian Cancer	Ovarian	1.03E-02	1.71E-01	9.56E-04	4.26E-01
5498	GSM102489	ExpO	Ovarian Cancer	Ovarian	8.39E-02	1.26E-01	2.59E-03	3.94E-01
5499	GSM102491	ExpO	Ovarian Cancer	Ovarian	1.73E-01	-1.06E-01	1.98E-01	2.06E-01
5500	GSM102504	ExpO	Ovarian Cancer	Ovarian	1.43E-01	1.12E-01	1.83E-02	3.23E-01
5501	GSM102528	ExpO	Ovarian Cancer	Ovarian	1.12E-01	1.18E-01	3.35E-02	2.98E-01
5502	GSM102538	ExpO	Ovarian Cancer	Ovarian	1.92E-07	3.15E-01	1.37E-06	5.94E-01
5503	GSM102546	ExpO	Ovarian Cancer	Ovarian	1.28E-03	-2.06E-01	3.14E-01	-1.74E-01
5504	GSM102552	ExpO	Ovarian Cancer	Ovarian	5.16E-12	-4.08E-01	1.53E-03	-4.11E-01
5505	GSM102557	ExpO	Ovarian Cancer	Ovarian	1.53E-04	-2.37E-01	3.27E-01	-1.71E-01
5506	GSM117592	ExpO	Ovarian Cancer	Ovarian	3.65E-03	-1.90E-01	4.26E-01	-1.49E-01
5507	GSM117648	ExpO	Ovarian Cancer	Ovarian	2.64E-01	-9.24E-02	1.77E-01	2.13E-01
5508	GSM117663	ExpO	Ovarian Cancer	Ovarian	1.99E-09	-3.58E-01	1.25E-03	-4.18E-01
5509	GSM117668	ExpO	Ovarian Cancer	Ovarian	5.29E-02	-1.37E-01	3.27E-01	-1.71E-01
5510	GSM117670	ExpO	Ovarian Cancer	Ovarian	4.45E-07	-3.06E-01	1.63E-01	-2.18E-01
5511	GSM117696	ExpO	Ovarian Cancer	Ovarian	4.09E-02	-1.43E-01	2.83E-01	1.82E-01
5512	GSM117698	ExpO	Ovarian Cancer	Ovarian	6.75E-04	2.16E-01	5.16E-03	3.71E-01
5513	GSM117716	ExpO	Ovarian Cancer	Ovarian	4.95E-01	6.71E-02	1.51E-02	3.31E-01
5514	GSM117717	ExpO	Ovarian Cancer	Ovarian	1.13E-01	1.18E-01	2.11E-02	3.17E-01
5515	GSM117718	ExpO	Ovarian Cancer	Ovarian	4.31E-03	-1.87E-01	4.45E-01	1.45E-01
5516	GSM117744	ExpO	Ovarian Cancer	Ovarian	1.51E-08	3.40E-01	1.46E-05	5.39E-01
5517	GSM117768	ExpO	Ovarian Cancer	Ovarian	3.19E-01	8.55E-02	6.56E-02	2.67E-01
5518	GSM137904	ExpO	Ovarian Cancer	Ovarian	1.30E-05	-2.68E-01	1.03E-01	-2.44E-01
5519	GSM137905	ExpO	Ovarian Cancer	Ovarian	7.31E-02	1.29E-01	3.11E-02	3.01E-01
5520	GSM137915	ExpO	Ovarian Cancer	Ovarian	7.90E-07	3.00E-01	1.46E-05	5.39E-01
5521	GSM137917	ExpO	Ovarian Cancer	Ovarian	8.84E-17	-4.87E-01	9.83E-05	-4.91E-01
5522	GSM137923	ExpO	Ovarian Cancer	Ovarian	2.45E-06	2.88E-01	2.00E-05	5.31E-01
5523	GSM137957	ExpO	Ovarian Cancer	Ovarian	2.70E-11	-3.95E-01	2.72E-02	-3.07E-01
5524	GSM137965	ExpO	Ovarian Cancer	Ovarian	1.56E-20	-5.40E-01	5.58E-06	-5.62E-01
5525	GSM137969	ExpO	Ovarian Cancer	Ovarian	2.80E-02	-1.51E-01	1.40E-01	2.27E-01
5526	GSM137982	ExpO	Ovarian Cancer	Ovarian	1.74E-19	5.26E-01	2.08E-11	8.01E-01
5527	GSM137983	ExpO	Ovarian Cancer	Ovarian	4.36E-03	-1.87E-01	3.66E-01	-1.62E-01
5528	GSM138026	ExpO	Ovarian Cancer	Ovarian	2.00E-01	1.02E-01	1.80E-02	3.24E-01
5529	GSM152568	ExpO	Ovarian Cancer	Ovarian	1.40E-03	2.05E-01	6.83E-04	4.36E-01
5530	GSM152574	ExpO	Ovarian Cancer	Ovarian	1.11E-04	-2.42E-01	2.00E-01	-2.05E-01
5531	GSM152577	ExpO	Ovarian Cancer	Ovarian	9.36E-02	1.23E-01	1.34E-01	2.29E-01
5532	GSM152578	ExpO	Ovarian Cancer	Ovarian	1.93E-01	1.03E-01	1.32E-01	2.30E-01
5533	GSM152581	ExpO	Ovarian Cancer	Ovarian	4.42E-02	-1.41E-01	3.46E-01	1.66E-01
5534	GSM152589	ExpO	Ovarian Cancer	Ovarian	3.95E-16	4.77E-01	1.19E-08	6.90E-01
5535	GSM152596	ExpO	Ovarian Cancer	Ovarian	8.33E-03	-1.75E-01	5.83E-01	1.19E-01
5536	GSM152599	ExpO	Ovarian Cancer	Ovarian	4.95E-04	-2.21E-01	4.23E-01	-1.50E-01
5537	GSM152603	ExpO	Ovarian Cancer	Ovarian	2.80E-02	-1.51E-01	3.99E-01	1.55E-01
5538	GSM152607	ExpO	Ovarian Cancer	Ovarian	2.19E-05	2.62E-01	9.60E-05	4.91E-01
5539	GSM152623	ExpO	Ovarian Cancer	Ovarian	5.82E-07	3.03E-01	7.95E-06	5.54E-01
5540	GSM152634	ExpO	Ovarian Cancer	Ovarian	5.15E-01	6.52E-02	2.22E-01	1.98E-01
5541	GSM152639	ExpO	Ovarian Cancer	Ovarian	4.94E-09	3.50E-01	1.45E-09	7.29E-01
5542	GSM152646	ExpO	Ovarian Cancer	Ovarian	2.41E-13	-4.31E-01	3.25E-03	-3.87E-01
5543	GSM152648	ExpO	Ovarian Cancer	Ovarian	2.40E-02	-1.55E-01	1.98E-01	2.06E-01
5544	GSM152653	ExpO	Ovarian Cancer	Ovarian	2.04E-01	-1.01E-01	7.06E-01	9.52E-02
5545	GSM152654	ExpO	Ovarian Cancer	Ovarian	2.10E-03	1.99E-01	2.54E-03	3.95E-01

5546	GSM152657	ExpO	Ovarian Cancer	Ovarian	2.64E-03	-1.95E-01	5.06E-01	-1.33E-01
5547	GSM152659	ExpO	Ovarian Cancer	Ovarian	8.09E-13	-4.22E-01	1.89E-02	-3.22E-01
5548	GSM152662	ExpO	Ovarian Cancer	Ovarian	3.43E-02	1.47E-01	2.45E-03	3.96E-01
5549	GSM152668	ExpO	Ovarian Cancer	Ovarian	6.43E-10	-3.68E-01	1.22E-02	-3.39E-01
5550	GSM152672	ExpO	Ovarian Cancer	Ovarian	3.48E-28	6.36E-01	6.23E-13	8.57E-01
5551	GSM152673	ExpO	Ovarian Cancer	Ovarian	1.17E-02	-1.69E-01	5.39E-01	1.27E-01
5552	GSM152676	ExpO	Ovarian Cancer	Ovarian	1.24E-05	-2.69E-01	6.56E-02	-2.67E-01
5553	GSM152679	ExpO	Ovarian Cancer	Ovarian	7.06E-02	-1.30E-01	1.79E-01	2.12E-01
5554	GSM152683	ExpO	Ovarian Cancer	Ovarian	4.88E-05	2.52E-01	2.38E-04	4.67E-01
5555	GSM152688	ExpO	Ovarian Cancer	Ovarian	2.74E-03	-1.94E-01	6.27E-01	1.10E-01
5556	GSM152693	ExpO	Ovarian Cancer	Ovarian	5.47E-07	-3.04E-01	1.43E-01	-2.25E-01
5557	GSM152700	ExpO	Ovarian Cancer	Ovarian	1.62E-20	-5.40E-01	1.10E-05	-5.46E-01
5558	GSM152706	ExpO	Ovarian Cancer	Ovarian	5.27E-10	3.70E-01	2.93E-07	6.27E-01
5559	GSM152709	ExpO	Ovarian Cancer	Ovarian	5.09E-02	-1.38E-01	5.59E-01	1.23E-01
5560	GSM152721	ExpO	Ovarian Cancer	Ovarian	1.72E-23	-5.79E-01	9.57E-08	-6.50E-01
5561	GSM152722	ExpO	Ovarian Cancer	Ovarian	1.50E-14	-4.52E-01	2.44E-04	-4.66E-01
5562	GSM152724	ExpO	Ovarian Cancer	Ovarian	2.09E-02	1.57E-01	1.75E-02	3.25E-01
5563	GSM152733	ExpO	Ovarian Cancer	Ovarian	1.11E-05	-2.70E-01	6.65E-02	-2.66E-01
5564	GSM152735	ExpO	Ovarian Cancer	Ovarian	4.83E-09	3.50E-01	3.90E-06	5.70E-01
5565	GSM152739	ExpO	Ovarian Cancer	Ovarian	3.52E-15	4.62E-01	1.40E-09	7.30E-01
5566	GSM152746	ExpO	Ovarian Cancer	Ovarian	1.60E-09	-3.60E-01	4.76E-02	-2.82E-01
5567	GSM152747	ExpO	Ovarian Cancer	Ovarian	2.57E-04	-2.30E-01	1.98E-01	-2.06E-01
5568	GSM152749	ExpO	Ovarian Cancer	Ovarian	1.48E-03	-2.04E-01	3.55E-01	-1.64E-01
5569	GSM152752	ExpO	Ovarian Cancer	Ovarian	7.85E-03	-1.76E-01	3.30E-01	-1.70E-01
5570	GSM152754	ExpO	Ovarian Cancer	Ovarian	7.00E-02	-1.31E-01	7.36E-02	2.61E-01
5571	GSM152769	ExpO	Ovarian Cancer	Ovarian	7.97E-02	1.27E-01	4.00E-03	3.80E-01
5572	GSM152784	ExpO	Ovarian Cancer	Ovarian	2.59E-09	-3.56E-01	3.30E-02	-2.98E-01
5573	GSM152792	ExpO	Ovarian Cancer	Ovarian	2.01E-07	-3.14E-01	1.05E-01	-2.43E-01
5574	GSM152794	ExpO	Ovarian Cancer	Ovarian	1.42E-01	1.12E-01	2.61E-02	3.09E-01
5575	GSM152795	ExpO	Ovarian Cancer	Ovarian	3.31E-12	-4.11E-01	3.25E-02	-2.99E-01
5576	GSM152800	ExpO	Ovarian Cancer	Ovarian	9.52E-02	-1.23E-01	2.22E-01	1.98E-01
5577	GSM179787	ExpO	Ovarian Cancer	Ovarian	2.11E-01	-9.98E-02	6.23E-02	2.69E-01
5578	GSM179798	ExpO	Ovarian Cancer	Ovarian	2.96E-01	8.83E-02	2.83E-01	1.82E-01
5579	GSM179799	ExpO	Ovarian Cancer	Ovarian	2.59E-09	-3.56E-01	3.11E-02	-3.01E-01
5580	GSM179807	ExpO	Ovarian Cancer	Ovarian	1.91E-03	-2.00E-01	3.14E-01	-1.74E-01
5581	GSM179808	ExpO	Ovarian Cancer	Ovarian	3.57E-01	8.12E-02	6.48E-02	2.67E-01
5582	GSM179812	ExpO	Ovarian Cancer	Ovarian	2.65E-06	2.87E-01	2.87E-06	5.77E-01
5583	GSM179813	ExpO	Ovarian Cancer	Ovarian	2.70E-01	-9.16E-02	2.95E-01	1.78E-01
5584	GSM179817	ExpO	Ovarian Cancer	Ovarian	7.93E-04	2.14E-01	2.49E-03	3.96E-01
5585	GSM179822	ExpO	Ovarian Cancer	Ovarian	5.22E-05	-2.51E-01	1.54E-01	-2.21E-01
5586	GSM179823	ExpO	Ovarian Cancer	Ovarian	7.95E-05	-2.46E-01	2.33E-01	-1.95E-01
5587	GSM179830	ExpO	Ovarian Cancer	Ovarian	5.11E-16	-4.75E-01	1.07E-05	-5.47E-01
5588	GSM179836	ExpO	Ovarian Cancer	Ovarian	6.97E-01	-4.81E-02	6.65E-02	2.66E-01
5589	GSM179850	ExpO	Ovarian Cancer	Ovarian	1.24E-11	-4.01E-01	4.70E-02	-2.83E-01
5590	GSM179852	ExpO	Ovarian Cancer	Ovarian	3.64E-18	5.07E-01	8.95E-11	7.77E-01
5591	GSM179866	ExpO	Ovarian Cancer	Ovarian	3.81E-09	-3.52E-01	9.68E-03	-3.48E-01
5592	GSM179870	ExpO	Ovarian Cancer	Ovarian	1.75E-13	-4.34E-01	3.19E-04	-4.58E-01
5593	GSM179878	ExpO	Ovarian Cancer	Ovarian	4.46E-02	-1.41E-01	3.93E-01	1.56E-01
5594	GSM179890	ExpO	Ovarian Cancer	Ovarian	1.67E-03	-2.02E-01	6.47E-01	1.07E-01
5595	GSM179906	ExpO	Ovarian Cancer	Ovarian	8.39E-07	2.99E-01	5.36E-05	5.07E-01
5596	GSM179942	ExpO	Ovarian Cancer	Ovarian	6.17E-03	1.81E-01	2.94E-05	5.22E-01
5597	GSM179943	ExpO	Ovarian Cancer	Ovarian	1.34E-02	1.66E-01	3.11E-02	3.01E-01
5598	GSM38064	ExpO	Ovarian Cancer	Ovarian	1.77E-07	-3.16E-01	2.72E-02	-3.07E-01
5599	GSM38065	ExpO	Ovarian Cancer	Ovarian	8.33E-03	1.75E-01	1.75E-01	2.13E-01
5600	GSM38070	ExpO	Ovarian Cancer	Ovarian	4.20E-04	2.23E-01	7.13E-04	4.35E-01
5601	GSM38071	ExpO	Ovarian Cancer	Ovarian	4.14E-04	2.23E-01	2.05E-05	5.31E-01
5602	GSM38088	ExpO	Ovarian Cancer	Ovarian	1.61E-01	-1.08E-01	2.58E-01	1.88E-01
5603	GSM38093	ExpO	Ovarian Cancer	Ovarian	2.92E-08	3.33E-01	2.87E-06	5.77E-01
5604	GSM38095	ExpO	Ovarian Cancer	Ovarian	1.21E-20	-5.42E-01	2.72E-06	-5.78E-01
5605	GSM46814	ExpO	Ovarian Cancer	Ovarian	1.30E-05	2.68E-01	5.28E-04	4.44E-01
5606	GSM46815	ExpO	Ovarian Cancer	Ovarian	1.70E-01	-1.06E-01	1.41E-01	2.26E-01
5607	GSM46818	ExpO	Ovarian Cancer	Ovarian	9.38E-16	4.71E-01	2.37E-10	7.61E-01
5608	GSM46828	ExpO	Ovarian Cancer	Ovarian	1.07E-02	1.71E-01	2.01E-03	4.03E-01
5609	GSM46830	ExpO	Ovarian Cancer	Ovarian	6.90E-19	-5.18E-01	2.11E-05	-5.30E-01
5610	GSM46831	ExpO	Ovarian Cancer	Ovarian	2.67E-03	-1.95E-01	6.23E-01	-1.11E-01
5611	GSM46834	ExpO	Ovarian Cancer	Ovarian	6.72E-03	-1.79E-01	2.06E-01	-2.03E-01
5612	GSM46839	ExpO	Ovarian Cancer	Ovarian	6.88E-03	-1.79E-01	3.55E-01	-1.64E-01
5613	GSM46853	ExpO	Ovarian Cancer	Ovarian	3.33E-02	-1.48E-01	2.29E-01	1.96E-01
5614	GSM46886	ExpO	Ovarian Cancer	Ovarian	5.36E-07	-3.04E-01	2.64E-02	-3.08E-01
5615	GSM46888	ExpO	Ovarian Cancer	Ovarian	2.50E-02	1.54E-01	2.17E-02	3.16E-01
5616	GSM46897	ExpO	Ovarian Cancer	Ovarian	4.45E-07	-3.06E-01	1.86E-01	-2.10E-01
5617	GSM46898	ExpO	Ovarian Cancer	Ovarian	2.31E-04	-2.32E-01	5.43E-01	-1.26E-01
5618	GSM46902	ExpO	Ovarian Cancer	Ovarian	5.34E-08	3.27E-01	5.74E-06	5.61E-01
5619	GSM46906	ExpO	Ovarian Cancer	Ovarian	4.81E-02	1.39E-01	1.46E-02	3.32E-01

5620	GSM46910	ExpO	Ovarian Cancer	Ovarian	3.40E-06	-2.84E-01	1.04E-01	-2.43E-01
5621	GSM46918	ExpO	Ovarian Cancer	Ovarian	9.52E-02	-1.23E-01	3.46E-01	1.66E-01
5622	GSM46925	ExpO	Ovarian Cancer	Ovarian	2.77E-16	-4.79E-01	1.77E-04	-4.75E-01
5623	GSM46935	ExpO	Ovarian Cancer	Ovarian	9.16E-03	1.73E-01	8.02E-03	3.55E-01
5624	GSM46948	ExpO	Ovarian Cancer	Ovarian	1.18E-01	-1.17E-01	6.56E-02	2.67E-01
5625	GSM46950	ExpO	Ovarian Cancer	Ovarian	8.56E-07	2.99E-01	3.80E-06	5.71E-01
5626	GSM46966	ExpO	Ovarian Cancer	Ovarian	2.26E-02	1.56E-01	6.31E-02	2.69E-01
5627	GSM53029	ExpO	Ovarian Cancer	Ovarian	2.61E-02	-1.53E-01	3.49E-01	1.66E-01
5628	GSM53036	ExpO	Ovarian Cancer	Ovarian	5.14E-02	1.38E-01	1.83E-02	3.23E-01
5629	GSM53040	ExpO	Ovarian Cancer	Ovarian	1.93E-01	1.03E-01	1.00E-01	2.45E-01
5630	GSM53049	ExpO	Ovarian Cancer	Ovarian	7.64E-14	4.40E-01	1.40E-07	6.42E-01
5631	GSM53054	ExpO	Ovarian Cancer	Ovarian	1.84E-02	-1.60E-01	3.46E-01	1.66E-01
5632	GSM53056	ExpO	Ovarian Cancer	Ovarian	2.47E-22	5.65E-01	5.67E-11	7.85E-01
5633	GSM53063	ExpO	Ovarian Cancer	Ovarian	5.43E-09	-3.49E-01	8.16E-03	-3.54E-01
5634	GSM53066	ExpO	Ovarian Cancer	Ovarian	2.78E-02	-1.52E-01	4.32E-01	1.48E-01
5635	GSM53068	ExpO	Ovarian Cancer	Ovarian	5.50E-05	-2.51E-01	1.06E-01	-2.42E-01
5636	GSM53069	ExpO	Ovarian Cancer	Ovarian	1.20E-07	-3.19E-01	1.34E-01	-2.29E-01
5637	GSM53077	ExpO	Ovarian Cancer	Ovarian	1.38E-03	2.05E-01	2.33E-04	4.67E-01
5638	GSM53082	ExpO	Ovarian Cancer	Ovarian	8.50E-05	2.45E-01	6.83E-04	4.36E-01
5639	GSM53098	ExpO	Ovarian Cancer	Ovarian	5.43E-02	-1.37E-01	2.58E-01	1.88E-01
5640	GSM53100	ExpO	Ovarian Cancer	Ovarian	8.32E-02	1.26E-01	9.17E-04	4.27E-01
5641	GSM53104	ExpO	Ovarian Cancer	Ovarian	5.97E-11	-3.88E-01	1.53E-03	-4.11E-01
5642	GSM53105	ExpO	Ovarian Cancer	Ovarian	2.02E-13	-4.33E-01	4.25E-04	-4.50E-01
5643	GSM53118	ExpO	Ovarian Cancer	Ovarian	7.81E-04	-2.14E-01	9.10E-02	-2.50E-01
5644	GSM53121	ExpO	Ovarian Cancer	Ovarian	4.34E-01	-7.32E-02	3.52E-01	1.65E-01
5645	GSM53124	ExpO	Ovarian Cancer	Ovarian	2.04E-01	1.01E-01	5.31E-02	2.77E-01
5646	GSM53125	ExpO	Ovarian Cancer	Ovarian	6.72E-03	1.79E-01	5.28E-04	4.44E-01
5647	GSM53144	ExpO	Ovarian Cancer	Ovarian	4.40E-05	-2.53E-01	1.17E-01	-2.37E-01
5648	GSM53150	ExpO	Ovarian Cancer	Ovarian	2.18E-07	-3.13E-01	2.77E-02	-3.06E-01
5649	GSM53151	ExpO	Ovarian Cancer	Ovarian	4.53E-06	-2.81E-01	5.45E-02	-2.76E-01
5650	GSM53163	ExpO	Ovarian Cancer	Ovarian	1.65E-02	-1.62E-01	2.25E-01	1.97E-01
5651	GSM53165	ExpO	Ovarian Cancer	Ovarian	5.68E-06	-2.78E-01	1.22E-01	-2.34E-01
5652	GSM53166	ExpO	Ovarian Cancer	Ovarian	1.44E-01	1.11E-01	5.24E-02	2.77E-01
5653	GSM53173	ExpO	Ovarian Cancer	Ovarian	2.23E-02	-1.56E-01	3.46E-01	1.66E-01
5654	GSM53175	ExpO	Ovarian Cancer	Ovarian	5.96E-02	-1.34E-01	3.81E-02	2.92E-01
5655	GSM53177	ExpO	Ovarian Cancer	Ovarian	1.02E-08	-3.43E-01	4.98E-03	-3.72E-01
5656	GSM53180	ExpO	Ovarian Cancer	Ovarian	3.25E-01	8.48E-02	1.89E-02	3.22E-01
5657	GSM53184	ExpO	Ovarian Cancer	Ovarian	2.20E-04	-2.32E-01	3.30E-01	-1.70E-01
5658	GSM53185	ExpO	Ovarian Cancer	Ovarian	3.17E-02	-1.49E-01	9.90E-02	2.46E-01
5659	GSM53186	ExpO	Ovarian Cancer	Ovarian	6.19E-04	2.18E-01	3.98E-04	4.52E-01
5660	GSM76489	ExpO	Ovarian Cancer	Ovarian	4.56E-05	-2.53E-01	1.21E-01	-2.35E-01
5661	GSM76500	ExpO	Ovarian Cancer	Ovarian	1.78E-23	5.79E-01	3.45E-11	7.93E-01
5662	GSM76502	ExpO	Ovarian Cancer	Ovarian	3.38E-01	8.33E-02	1.80E-01	2.11E-01
5663	GSM76504	ExpO	Ovarian Cancer	Ovarian	1.36E-06	-2.94E-01	1.05E-01	-2.43E-01
5664	GSM76509	ExpO	Ovarian Cancer	Ovarian	5.67E-18	-5.04E-01	3.98E-05	-5.14E-01
5665	GSM76510	ExpO	Ovarian Cancer	Ovarian	4.33E-04	-2.23E-01	4.77E-01	-1.39E-01
5666	GSM76511	ExpO	Ovarian Cancer	Ovarian	3.60E-23	-5.75E-01	1.37E-06	-5.94E-01
5667	GSM76533	ExpO	Ovarian Cancer	Ovarian	2.57E-04	-2.30E-01	5.68E-02	-2.74E-01
5668	GSM76537	ExpO	Ovarian Cancer	Ovarian	5.02E-12	4.08E-01	5.27E-09	7.05E-01
5669	GSM76539	ExpO	Ovarian Cancer	Ovarian	1.99E-03	2.00E-01	4.89E-03	3.73E-01
5670	GSM76540	ExpO	Ovarian Cancer	Ovarian	1.22E-19	-5.28E-01	1.40E-07	-6.42E-01
5671	GSM76542	ExpO	Ovarian Cancer	Ovarian	7.11E-10	3.67E-01	6.33E-08	6.58E-01
5672	GSM76547	ExpO	Ovarian Cancer	Ovarian	4.59E-03	1.86E-01	1.34E-04	4.83E-01
5673	GSM76554	ExpO	Ovarian Cancer	Ovarian	4.78E-08	-3.29E-01	1.96E-01	-2.06E-01
5674	GSM76567	ExpO	Ovarian Cancer	Ovarian	1.05E-07	-3.21E-01	1.96E-01	-2.06E-01
5675	GSM76581	ExpO	Ovarian Cancer	Ovarian	1.65E-04	-2.36E-01	2.04E-01	-2.04E-01
5676	GSM76596	ExpO	Ovarian Cancer	Ovarian	8.12E-08	-3.23E-01	6.23E-02	-2.69E-01
5677	GSM76599	ExpO	Ovarian Cancer	Ovarian	1.16E-09	-3.63E-01	4.70E-02	-2.83E-01
5678	GSM76601	ExpO	Ovarian Cancer	Ovarian	1.56E-05	2.66E-01	7.36E-05	4.98E-01
5679	GSM76604	ExpO	Ovarian Cancer	Ovarian	4.25E-01	7.40E-02	2.02E-01	2.04E-01
5680	GSM76616	ExpO	Ovarian Cancer	Ovarian	1.74E-01	1.06E-01	4.38E-02	2.86E-01
5681	GSM76623	ExpO	Ovarian Cancer	Ovarian	7.95E-05	2.46E-01	8.98E-04	4.28E-01
5682	GSM76633	ExpO	Ovarian Cancer	Ovarian	4.13E-11	3.91E-01	7.95E-06	5.54E-01
5683	GSM76636	ExpO	Ovarian Cancer	Ovarian	1.96E-01	1.02E-01	2.27E-02	3.14E-01
5684	GSM76645	ExpO	Ovarian Cancer	Ovarian	8.39E-02	-1.26E-01	1.19E-01	2.36E-01
5685	GSM76646	ExpO	Ovarian Cancer	Ovarian	4.15E-03	1.87E-01	7.13E-04	4.35E-01
5686	GSM88956	ExpO	Ovarian Cancer	Ovarian	1.82E-10	-3.79E-01	1.89E-02	-3.22E-01
5687	GSM88957	ExpO	Ovarian Cancer	Ovarian	2.06E-01	-1.01E-01	1.80E-01	2.11E-01
5688	GSM88973	ExpO	Ovarian Cancer	Ovarian	8.68E-02	1.25E-01	1.41E-01	2.26E-01
5689	GSM88974	ExpO	Ovarian Cancer	Ovarian	1.37E-01	-1.13E-01	1.73E-01	2.14E-01
5690	GSM88984	ExpO	Ovarian Cancer	Ovarian	3.90E-09	-3.52E-01	3.11E-02	-3.01E-01
5691	GSM88992	ExpO	Ovarian Cancer	Ovarian	3.60E-02	1.46E-01	3.97E-02	2.90E-01
5692	GSM89001	ExpO	Ovarian Cancer	Ovarian	2.44E-12	-4.14E-01	5.25E-03	-3.70E-01
5693	GSM89012	ExpO	Ovarian Cancer	Ovarian	4.71E-09	-3.50E-01	2.17E-02	-3.16E-01

5694	GSM89020	ExpO	Ovarian Cancer	Ovarian	8.39E-02	-1.26E-01	2.83E-01	1.82E-01
5695	GSM89024	ExpO	Ovarian Cancer	Ovarian	8.95E-03	1.74E-01	2.14E-02	3.17E-01
5696	GSM89025	ExpO	Ovarian Cancer	Ovarian	1.15E-08	-3.42E-01	4.70E-02	-2.83E-01
5697	GSM89028	ExpO	Ovarian Cancer	Ovarian	1.94E-02	1.59E-01	6.17E-03	3.64E-01
5698	GSM89036	ExpO	Ovarian Cancer	Ovarian	7.82E-23	-5.71E-01	2.79E-06	-5.78E-01
5699	GSM89057	ExpO	Ovarian Cancer	Ovarian	7.64E-02	1.28E-01	1.24E-02	3.38E-01
5700	GSM89070	ExpO	Ovarian Cancer	Ovarian	6.37E-04	-2.17E-01	2.29E-01	-1.96E-01
5701	GSM89079	ExpO	Ovarian Cancer	Ovarian	2.28E-02	-1.56E-01	5.43E-01	1.26E-01
5702	GSM89081	ExpO	Ovarian Cancer	Ovarian	1.28E-01	1.15E-01	3.49E-01	1.66E-01
5703	GSM89082	ExpO	Ovarian Cancer	Ovarian	2.33E-01	-9.65E-02	3.46E-01	1.66E-01
5704	GSM89091	ExpO	Ovarian Cancer	Ovarian	1.03E-15	4.70E-01	2.28E-10	7.61E-01
5705	GSM89093	ExpO	Ovarian Cancer	Ovarian	1.03E-15	4.70E-01	3.55E-10	7.54E-01
5706	GSM89097	ExpO	Ovarian Cancer	Ovarian	8.68E-02	1.25E-01	2.49E-01	1.90E-01
5707	A11	TCGA	Ovarian Cancer	Ovarian	2.89E-02	1.51E-01	9.52E-03	3.49E-01
5708	D04	TCGA	Ovarian Cancer	Ovarian	4.00E-01	-7.66E-02	2.27E-02	3.14E-01
5709	D05	TCGA	Ovarian Cancer	Ovarian	4.97E-01	6.69E-02	4.63E-02	2.83E-01
5710	D06	TCGA	Ovarian Cancer	Ovarian	7.52E-06	2.75E-01	2.03E-07	6.34E-01
5711	D07	TCGA	Ovarian Cancer	Ovarian	5.58E-13	4.25E-01	1.84E-08	6.82E-01
5712	D08	TCGA	Ovarian Cancer	Ovarian	2.49E-04	-2.31E-01	2.06E-01	-2.03E-01
5713	D10	TCGA	Ovarian Cancer	Ovarian	1.30E-01	-1.14E-01	4.42E-01	1.46E-01
5714	D11	TCGA	Ovarian Cancer	Ovarian	1.51E-01	-1.10E-01	2.31E-01	1.96E-01
5715	E01	TCGA	Ovarian Cancer	Ovarian	1.01E-03	-2.10E-01	3.27E-01	-1.71E-01
5716	E02	TCGA	Ovarian Cancer	Ovarian	4.53E-04	-2.22E-01	2.88E-01	-1.80E-01
5717	E04	TCGA	Ovarian Cancer	Ovarian	2.74E-03	-1.94E-01	2.29E-01	-1.96E-01
5718	E05	TCGA	Ovarian Cancer	Ovarian	3.46E-01	8.25E-02	5.31E-02	2.77E-01
5719	E06	TCGA	Ovarian Cancer	Ovarian	7.66E-06	-2.75E-01	1.01E-01	-2.44E-01
5720	E07	TCGA	Ovarian Cancer	Ovarian	1.32E-04	-2.39E-01	3.93E-01	-1.56E-01
5721	E08	TCGA	Ovarian Cancer	Ovarian	1.09E-01	-1.19E-01	3.14E-01	1.74E-01
5722	E10	TCGA	Ovarian Cancer	Ovarian	3.06E-07	-3.10E-01	1.77E-01	-2.13E-01
5723	E11	TCGA	Ovarian Cancer	Ovarian	3.09E-03	-1.92E-01	1.35E-01	-2.29E-01
5724	F01	TCGA	Ovarian Cancer	Ovarian	6.12E-02	1.34E-01	2.59E-03	3.94E-01
5725	F02	TCGA	Ovarian Cancer	Ovarian	9.15E-04	-2.12E-01	2.58E-01	-1.88E-01
5726	F04	TCGA	Ovarian Cancer	Ovarian	1.46E-04	2.38E-01	9.83E-05	4.91E-01
5727	F05	TCGA	Ovarian Cancer	Ovarian	3.50E-01	-8.20E-02	1.38E-01	2.27E-01
5728	F06	TCGA	Ovarian Cancer	Ovarian	1.78E-02	-1.61E-01	5.26E-01	1.30E-01
5729	F07	TCGA	Ovarian Cancer	Ovarian	5.27E-06	-2.79E-01	1.41E-01	-2.26E-01
5730	F08	TCGA	Ovarian Cancer	Ovarian	4.46E-02	-1.41E-01	6.30E-01	1.10E-01
5731	F10	TCGA	Ovarian Cancer	Ovarian	1.99E-01	1.02E-01	2.24E-02	3.15E-01
5732	F11	TCGA	Ovarian Cancer	Ovarian	1.38E-08	3.40E-01	3.48E-09	7.13E-01
5733	G01	TCGA	Ovarian Cancer	Ovarian	5.19E-02	-1.38E-01	3.46E-01	1.66E-01
5734	G02	TCGA	Ovarian Cancer	Ovarian	2.40E-01	9.57E-02	2.14E-02	3.17E-01
5735	G04	TCGA	Ovarian Cancer	Ovarian	1.89E-01	1.03E-01	8.56E-02	2.53E-01
5736	G05	TCGA	Ovarian Cancer	Ovarian	4.00E-10	-3.72E-01	9.10E-02	-2.50E-01
5737	G06	TCGA	Ovarian Cancer	Ovarian	9.05E-06	-2.73E-01	2.88E-01	-1.80E-01
5738	G07	TCGA	Ovarian Cancer	Ovarian	7.17E-11	-3.87E-01	3.98E-05	-5.14E-01
5739	G10	TCGA	Ovarian Cancer	Ovarian	2.06E-12	-4.15E-01	2.64E-03	-3.94E-01
5740	G11	TCGA	Ovarian Cancer	Ovarian	2.29E-01	-9.72E-02	2.61E-01	1.87E-01
5741	H01	TCGA	Ovarian Cancer	Ovarian	5.10E-04	-2.20E-01	4.80E-01	-1.38E-01
5742	H08	TCGA	Ovarian Cancer	Ovarian	1.54E-01	1.10E-01	1.83E-02	3.23E-01
5743	H09	TCGA	Ovarian Cancer	Ovarian	8.95E-03	-1.74E-01	2.54E-01	1.89E-01
5744	H10	TCGA	Ovarian Cancer	Ovarian	1.32E-02	-1.66E-01	3.24E-01	1.71E-01
5745	A12.A01	TCGA	Ovarian Cancer	Ovarian	1.96E-02	-1.59E-01	1.43E-01	2.25E-01
5746	A12.A02	TCGA	Ovarian Cancer	Ovarian	5.96E-02	-1.34E-01	2.95E-01	1.78E-01
5747	A12.A03	TCGA	Ovarian Cancer	Ovarian	1.12E-03	-2.09E-01	2.88E-01	-1.80E-01
5748	A12.A04	TCGA	Ovarian Cancer	Ovarian	3.58E-05	2.56E-01	1.31E-04	4.83E-01
5749	A12.A05	TCGA	Ovarian Cancer	Ovarian	9.47E-07	-2.98E-01	4.70E-02	-2.83E-01
5750	A12.A06	TCGA	Ovarian Cancer	Ovarian	1.65E-05	2.66E-01	1.77E-04	4.75E-01
5751	A12.A07	TCGA	Ovarian Cancer	Ovarian	4.64E-02	-1.40E-01	6.33E-01	-1.09E-01
5752	A12.A08	TCGA	Ovarian Cancer	Ovarian	1.78E-08	-3.38E-01	1.51E-01	-2.22E-01
5753	A12.A09	TCGA	Ovarian Cancer	Ovarian	1.19E-02	-1.68E-01	2.93E-01	1.79E-01
5754	A12.A10	TCGA	Ovarian Cancer	Ovarian	2.02E-03	-1.99E-01	5.43E-01	1.26E-01
5755	A12.A11	TCGA	Ovarian Cancer	Ovarian	4.64E-01	-7.01E-02	6.39E-02	2.68E-01
5756	A12.B01	TCGA	Ovarian Cancer	Ovarian	3.23E-01	8.51E-02	1.46E-02	3.32E-01
5757	A12.B02	TCGA	Ovarian Cancer	Ovarian	1.31E-16	-4.84E-01	7.54E-05	-4.98E-01
5758	A12.B03	TCGA	Ovarian Cancer	Ovarian	4.60E-01	7.06E-02	1.82E-01	2.11E-01
5759	A12.B04	TCGA	Ovarian Cancer	Ovarian	1.73E-01	-1.06E-01	1.35E-01	2.29E-01
5760	A12.B05	TCGA	Ovarian Cancer	Ovarian	5.47E-06	-2.79E-01	6.93E-01	-9.78E-02
5761	A12.B06	TCGA	Ovarian Cancer	Ovarian	7.58E-03	-1.77E-01	3.52E-01	-1.65E-01
5762	A12.B07	TCGA	Ovarian Cancer	Ovarian	1.18E-02	-1.69E-01	5.03E-01	-1.34E-01
5763	A12.B08	TCGA	Ovarian Cancer	Ovarian	9.74E-06	-2.72E-01	1.49E-02	-3.31E-01
5764	A12.B09	TCGA	Ovarian Cancer	Ovarian	1.25E-06	-2.95E-01	2.72E-02	-3.07E-01
5765	A12.B10	TCGA	Ovarian Cancer	Ovarian	4.27E-01	-7.38E-02	3.46E-01	1.66E-01
5766	A12.B11	TCGA	Ovarian Cancer	Ovarian	4.81E-02	-1.39E-01	3.66E-01	1.62E-01
5767	A12.B12	TCGA	Ovarian Cancer	Ovarian	1.05E-01	-1.20E-01	2.33E-01	1.95E-01

5768	A12.C01	TCGA	Ovarian Cancer	Ovarian	1.01E-06	-2.97E-01	3.30E-02	-2.98E-01
5769	A12.C02	TCGA	Ovarian Cancer	Ovarian	5.95E-03	-1.81E-01	4.23E-01	-1.50E-01
5770	A12.C03	TCGA	Ovarian Cancer	Ovarian	1.09E-03	2.09E-01	1.25E-03	4.18E-01
5771	A12.C04	TCGA	Ovarian Cancer	Ovarian	1.09E-01	-1.19E-01	3.14E-01	1.74E-01
5772	A12.C05	TCGA	Ovarian Cancer	Ovarian	3.77E-01	7.90E-02	6.39E-03	3.63E-01
5773	A12.C06	TCGA	Ovarian Cancer	Ovarian	2.82E-01	9.00E-02	6.39E-03	3.63E-01
5774	A12.C07	TCGA	Ovarian Cancer	Ovarian	1.85E-01	1.04E-01	8.66E-02	2.53E-01
5775	A12.C08	TCGA	Ovarian Cancer	Ovarian	5.25E-01	6.43E-02	1.80E-02	3.24E-01
5776	A12.C09	TCGA	Ovarian Cancer	Ovarian	3.05E-03	-1.93E-01	2.04E-01	-2.04E-01
5777	A12.C10	TCGA	Ovarian Cancer	Ovarian	1.51E-01	-1.10E-01	1.73E-01	2.14E-01
5778	A12.C11	TCGA	Ovarian Cancer	Ovarian	1.76E-06	2.91E-01	3.98E-05	5.14E-01
5779	A12.C12	TCGA	Ovarian Cancer	Ovarian	9.15E-04	-2.12E-01	7.80E-01	-8.06E-02
5780	A12.D01	TCGA	Ovarian Cancer	Ovarian	1.11E-04	-2.42E-01	3.63E-01	-1.63E-01
5781	A12.D02	TCGA	Ovarian Cancer	Ovarian	5.14E-02	1.38E-01	9.60E-05	4.91E-01
5782	A12.D03	TCGA	Ovarian Cancer	Ovarian	7.44E-02	-1.29E-01	5.19E-01	-1.31E-01
5783	A12.D04	TCGA	Ovarian Cancer	Ovarian	2.57E-01	-9.33E-02	1.88E-01	2.09E-01
5784	A12.D05	TCGA	Ovarian Cancer	Ovarian	9.10E-07	-2.98E-01	6.40E-01	-1.08E-01
5785	A12.D06	TCGA	Ovarian Cancer	Ovarian	2.66E-02	1.52E-01	3.70E-02	2.93E-01
5786	A12.D07	TCGA	Ovarian Cancer	Ovarian	2.65E-01	9.22E-02	2.63E-01	1.87E-01
5787	A12.D08	TCGA	Ovarian Cancer	Ovarian	1.09E-02	1.70E-01	9.17E-04	4.27E-01
5788	A12.D09	TCGA	Ovarian Cancer	Ovarian	1.38E-02	1.66E-01	9.17E-04	4.27E-01
5789	A12.D10	TCGA	Ovarian Cancer	Ovarian	4.82E-03	-1.85E-01	4.36E-01	-1.47E-01
5790	A12.D11	TCGA	Ovarian Cancer	Ovarian	6.85E-07	-3.02E-01	3.81E-01	-1.59E-01
5791	A12.D12	TCGA	Ovarian Cancer	Ovarian	1.55E-02	1.63E-01	2.57E-02	3.09E-01
5792	APEEK_A01_46413	TCGA	Ovarian Cancer	Ovarian	3.35E-10	3.74E-01	3.11E-07	6.25E-01
5793	APEEK_A02_46413	TCGA	Ovarian Cancer	Ovarian	1.34E-02	-1.66E-01	6.03E-01	-1.15E-01
5794	APEEK_A03_46412	TCGA	Ovarian Cancer	Ovarian	2.02E-06	-2.90E-01	3.55E-01	-1.64E-01
5795	APEEK_A04_46413	TCGA	Ovarian Cancer	Ovarian	1.62E-02	1.63E-01	1.22E-03	4.18E-01
5796	APEEK_A05_46414	TCGA	Ovarian Cancer	Ovarian	3.68E-02	-1.45E-01	6.06E-01	1.14E-01
5797	APEEK_A06_46415	TCGA	Ovarian Cancer	Ovarian	2.13E-15	4.65E-01	9.11E-10	7.37E-01
5798	APEEK_A07_46414	TCGA	Ovarian Cancer	Ovarian	9.98E-09	-3.44E-01	1.15E-01	-2.37E-01
5799	APEEK_A08_46414	TCGA	Ovarian Cancer	Ovarian	1.26E-04	-2.40E-01	4.32E-01	-1.48E-01
5800	APEEK_A09_46414	TCGA	Ovarian Cancer	Ovarian	5.42E-04	-2.19E-01	2.31E-01	-1.96E-01
5801	APEEK_A10_46414	TCGA	Ovarian Cancer	Ovarian	2.04E-01	-1.01E-01	1.56E-01	2.20E-01
5802	APEEK_A11_46413	TCGA	Ovarian Cancer	Ovarian	3.66E-04	-2.25E-01	1.01E-01	-2.44E-01
5803	APEEK_A12_46413	TCGA	Ovarian Cancer	Ovarian	1.11E-05	-2.70E-01	1.18E-01	-2.36E-01
5804	APEEK_B01_46407	TCGA	Ovarian Cancer	Ovarian	1.97E-03	-2.00E-01	5.96E-01	-1.16E-01
5805	APEEK_B02_46407	TCGA	Ovarian Cancer	Ovarian	1.56E-01	-1.09E-01	4.45E-02	2.85E-01
5806	APEEK_B03_46407	TCGA	Ovarian Cancer	Ovarian	2.38E-01	-9.59E-02	2.66E-01	1.86E-01
5807	APEEK_B04_46407	TCGA	Ovarian Cancer	Ovarian	1.38E-01	-1.13E-01	1.73E-01	2.14E-01
5808	APEEK_B05_46406	TCGA	Ovarian Cancer	Ovarian	3.84E-04	-2.24E-01	6.93E-01	-9.78E-02
5809	APEEK_B06_46406	TCGA	Ovarian Cancer	Ovarian	1.21E-05	-2.69E-01	3.97E-02	-2.90E-01
5810	APEEK_B07_46407	TCGA	Ovarian Cancer	Ovarian	1.23E-01	1.16E-01	5.40E-04	4.43E-01
5811	APEEK_B08_46406	TCGA	Ovarian Cancer	Ovarian	4.40E-01	-7.25E-02	2.25E-01	1.97E-01
5812	APEEK_B10_46408	TCGA	Ovarian Cancer	Ovarian	7.11E-06	-2.76E-01	1.82E-01	-2.11E-01
5813	APEEK_B11_46409	TCGA	Ovarian Cancer	Ovarian	1.36E-01	1.13E-01	8.77E-02	2.52E-01
5814	CELLS_A01_45197	TCGA	Ovarian Cancer	Ovarian	1.76E-02	-1.61E-01	4.42E-01	-1.46E-01
5815	CELLS_A02_45197	TCGA	Ovarian Cancer	Ovarian	7.81E-04	2.14E-01	5.28E-04	4.44E-01
5816	CELLS_A03_45197	TCGA	Ovarian Cancer	Ovarian	9.13E-02	1.24E-01	1.20E-02	3.40E-01
5817	CELLS_A04_45197	TCGA	Ovarian Cancer	Ovarian	5.00E-02	-1.39E-01	3.61E-01	-1.63E-01
5818	CELLS_A05_45196	TCGA	Ovarian Cancer	Ovarian	9.27E-03	1.73E-01	5.36E-05	5.07E-01
5819	CELLS_A06_45196	TCGA	Ovarian Cancer	Ovarian	9.37E-03	-1.73E-01	4.74E-01	-1.40E-01
5820	CELLS_A07_45195	TCGA	Ovarian Cancer	Ovarian	6.18E-02	-1.34E-01	4.26E-01	1.49E-01
5821	CELLS_A08_45195	TCGA	Ovarian Cancer	Ovarian	1.90E-09	-3.59E-01	5.53E-02	-2.75E-01
5822	CELLS_A09_45197	TCGA	Ovarian Cancer	Ovarian	1.28E-02	-1.67E-01	4.67E-01	1.41E-01
5823	CELLS_A10_45196	TCGA	Ovarian Cancer	Ovarian	1.96E-13	-4.33E-01	1.22E-03	-4.18E-01
5824	CELLS_A11_45196	TCGA	Ovarian Cancer	Ovarian	3.79E-02	1.45E-01	9.68E-03	3.48E-01
5825	CELLS_B01_45199	TCGA	Ovarian Cancer	Ovarian	4.59E-02	-1.40E-01	9.90E-02	2.46E-01
5826	CELLS_B02_45198	TCGA	Ovarian Cancer	Ovarian	4.04E-03	-1.88E-01	4.20E-01	1.50E-01
5827	CELLS_B03_45199	TCGA	Ovarian Cancer	Ovarian	4.51E-02	-1.41E-01	1.18E-01	2.36E-01
5828	CELLS_B04_45199	TCGA	Ovarian Cancer	Ovarian	8.08E-05	-2.46E-01	2.93E-01	-1.79E-01
5829	CELLS_B05_45198	TCGA	Ovarian Cancer	Ovarian	2.84E-01	8.98E-02	1.44E-02	3.33E-01
5830	CELLS_B06_45198	TCGA	Ovarian Cancer	Ovarian	2.98E-01	-8.81E-02	1.84E-01	2.10E-01
5831	CELLS_B07_45198	TCGA	Ovarian Cancer	Ovarian	2.66E-05	-2.60E-01	1.05E-01	-2.43E-01
5832	CELLS_B08_45198	TCGA	Ovarian Cancer	Ovarian	5.80E-01	-5.91E-02	3.52E-01	1.65E-01
5833	CELLS_B09_45200	TCGA	Ovarian Cancer	Ovarian	4.60E-04	-2.22E-01	3.33E-01	1.70E-01
5834	CELLS_B10_45200	TCGA	Ovarian Cancer	Ovarian	1.84E-03	-2.01E-01	3.52E-01	-1.65E-01
5835	CELLS_B11_45201	TCGA	Ovarian Cancer	Ovarian	3.23E-05	-2.57E-01	3.30E-01	-1.70E-01
5836	CELLS_B12_45200	TCGA	Ovarian Cancer	Ovarian	5.90E-02	-1.35E-01	4.67E-01	1.41E-01
5837	CELLS_C01_45199	TCGA	Ovarian Cancer	Ovarian	6.01E-02	-1.34E-01	2.83E-01	-1.82E-01
5838	CELLS_C02_45199	TCGA	Ovarian Cancer	Ovarian	9.60E-02	1.23E-01	3.11E-02	3.01E-01
5839	CELLS_C03_45200	TCGA	Ovarian Cancer	Ovarian	3.60E-02	-1.46E-01	2.88E-01	1.80E-01
5840	CELLS_C04_45200	TCGA	Ovarian Cancer	Ovarian	2.82E-01	-9.00E-02	7.36E-02	2.61E-01
5841	CELLS_C05_45202	TCGA	Ovarian Cancer	Ovarian	4.21E-01	7.45E-02	3.16E-02	3.00E-01

5842	CELLS_C06_45202	TCGA	Ovarian Cancer	Ovarian	3.77E-07	-3.08E-01	2.81E-02	-3.05E-01
5843	CELLS_C07_45202	TCGA	Ovarian Cancer	Ovarian	4.88E-03	-1.85E-01	4.32E-01	-1.48E-01
5844	CELLS_C08_45202	TCGA	Ovarian Cancer	Ovarian	7.05E-03	1.78E-01	2.20E-02	3.16E-01
5845	CELLS_C09_45201	TCGA	Ovarian Cancer	Ovarian	2.86E-02	-1.51E-01	2.02E-01	2.04E-01
5846	CELLS_C10_45201	TCGA	Ovarian Cancer	Ovarian	2.30E-02	-1.55E-01	5.39E-01	-1.27E-01
5847	CELLS_C11_45201	TCGA	Ovarian Cancer	Ovarian	1.16E-01	1.18E-01	2.14E-02	3.17E-01
5848	CELLS_D01_45203	TCGA	Ovarian Cancer	Ovarian	3.94E-02	1.44E-01	1.01E-04	4.90E-01
5849	CELLS_D02_45203	TCGA	Ovarian Cancer	Ovarian	2.79E-01	9.05E-02	1.17E-01	2.37E-01
5850	CELLS_D03_45204	TCGA	Ovarian Cancer	Ovarian	1.70E-01	-1.06E-01	1.32E-01	2.30E-01
5851	CELLS_D04_45204	TCGA	Ovarian Cancer	Ovarian	3.91E-08	-3.31E-01	9.10E-02	-2.50E-01
5852	CELLS_D05_45202	TCGA	Ovarian Cancer	Ovarian	4.15E-03	1.87E-01	8.98E-04	4.28E-01
5853	CELLS_D06_45203	TCGA	Ovarian Cancer	Ovarian	6.62E-05	2.48E-01	5.36E-05	5.07E-01
5854	CELLS_D07_45203	TCGA	Ovarian Cancer	Ovarian	2.44E-01	-9.50E-02	6.56E-02	2.67E-01
5855	CELLS_D08_45203	TCGA	Ovarian Cancer	Ovarian	5.53E-03	-1.82E-01	2.22E-01	1.98E-01
5856	CELLS_D09_45205	TCGA	Ovarian Cancer	Ovarian	1.79E-04	-2.35E-01	3.87E-01	-1.57E-01
5857	CELLS_D10_45205	TCGA	Ovarian Cancer	Ovarian	2.27E-05	-2.62E-01	1.59E-01	-2.19E-01
5858	CELLS_D11_45205	TCGA	Ovarian Cancer	Ovarian	8.64E-05	-2.45E-01	1.84E-01	-2.10E-01
5859	CELLS_D12_45205	TCGA	Ovarian Cancer	Ovarian	1.28E-03	-2.06E-01	2.49E-01	-1.90E-01
5860	CELLS_E01_45205	TCGA	Ovarian Cancer	Ovarian	4.74E-04	2.21E-01	5.10E-05	5.08E-01
5861	FEAST_E01_51629	TCGA	Ovarian Cancer	Ovarian	8.52E-16	-4.71E-01	4.16E-04	-4.51E-01
5862	FEAST_E03_51634	TCGA	Ovarian Cancer	Ovarian	8.64E-04	-2.13E-01	2.27E-01	1.97E-01
5863	FEAST_E08_51634	TCGA	Ovarian Cancer	Ovarian	6.48E-03	-1.80E-01	1.06E-01	-2.42E-01
5864	FEAST_E09_51643	TCGA	Ovarian Cancer	Ovarian	6.32E-03	-1.80E-01	7.86E-01	-7.94E-02
5865	FEAST_E10_51630	TCGA	Ovarian Cancer	Ovarian	7.57E-02	1.29E-01	1.77E-02	3.24E-01
5866	FEAST_E11_51634	TCGA	Ovarian Cancer	Ovarian	3.50E-02	-1.47E-01	1.98E-01	2.06E-01
5867	FEAST_F02_51630	TCGA	Ovarian Cancer	Ovarian	5.81E-03	-1.82E-01	5.26E-01	1.30E-01
5868	FEAST_F03_51635	TCGA	Ovarian Cancer	Ovarian	1.62E-02	1.63E-01	1.20E-03	4.19E-01
5869	FEAST_F05_51639	TCGA	Ovarian Cancer	Ovarian	1.69E-06	-2.92E-01	1.19E-01	-2.36E-01
5870	FEAST_F08_51647	TCGA	Ovarian Cancer	Ovarian	3.47E-02	-1.47E-01	3.96E-01	1.56E-01
5871	FEAST_F09_51631	TCGA	Ovarian Cancer	Ovarian	5.38E-02	1.37E-01	4.15E-03	3.78E-01
5872	FEAST_F10_51644	TCGA	Ovarian Cancer	Ovarian	5.78E-05	2.50E-01	3.05E-04	4.60E-01
5873	FEAST_F11_51630	TCGA	Ovarian Cancer	Ovarian	2.14E-06	-2.89E-01	2.77E-02	-3.06E-01
5874	FEAST_G02_51632	TCGA	Ovarian Cancer	Ovarian	6.34E-02	1.33E-01	1.83E-02	3.23E-01
5875	FEAST_G03_51641	TCGA	Ovarian Cancer	Ovarian	1.15E-02	1.69E-01	2.49E-03	3.96E-01
5876	FEAST_G04_51640	TCGA	Ovarian Cancer	Ovarian	1.60E-02	1.63E-01	6.17E-03	3.64E-01
5877	FEAST_G05_51644	TCGA	Ovarian Cancer	Ovarian	1.59E-01	-1.08E-01	2.51E-01	1.90E-01
5878	FEAST_G07_51637	TCGA	Ovarian Cancer	Ovarian	8.28E-04	-2.13E-01	4.23E-01	-1.50E-01
5879	FEAST_G08_51643	TCGA	Ovarian Cancer	Ovarian	2.14E-01	9.94E-02	9.21E-02	2.50E-01
5880	FEAST_G09_51635	TCGA	Ovarian Cancer	Ovarian	3.34E-03	-1.91E-01	4.61E-01	1.42E-01
5881	FEAST_G10_51645	TCGA	Ovarian Cancer	Ovarian	7.74E-07	-3.00E-01	2.66E-01	-1.86E-01
5882	FEAST_G11_51633	TCGA	Ovarian Cancer	Ovarian	2.60E-03	-1.95E-01	6.10E-01	-1.14E-01
5883	FEAST_H01_51630	TCGA	Ovarian Cancer	Ovarian	7.16E-04	-2.15E-01	2.08E-01	-2.03E-01
5884	FEAST_H02_51629	TCGA	Ovarian Cancer	Ovarian	5.40E-05	-2.51E-01	2.08E-01	-2.03E-01
5885	FEAST_H03_51628	TCGA	Ovarian Cancer	Ovarian	2.38E-01	9.59E-02	3.76E-02	2.93E-01
5886	FEAST_H05_51636	TCGA	Ovarian Cancer	Ovarian	2.74E-03	1.94E-01	1.20E-03	4.19E-01
5887	FEAST_H07_51635	TCGA	Ovarian Cancer	Ovarian	9.70E-05	2.43E-01	5.63E-05	5.05E-01
5888	FEAST_H08_51637	TCGA	Ovarian Cancer	Ovarian	6.24E-06	2.77E-01	7.53E-06	5.55E-01
5889	FEAST_H09_51636	TCGA	Ovarian Cancer	Ovarian	4.24E-11	-3.91E-01	6.39E-03	-3.63E-01
5890	GHYLL_A01_47188	TCGA	Ovarian Cancer	Ovarian	5.31E-05	-2.51E-01	1.41E-01	-2.26E-01
5891	GHYLL_A02_47188	TCGA	Ovarian Cancer	Ovarian	1.35E-01	-1.13E-01	2.36E-01	1.94E-01
5892	GHYLL_A03_47189	TCGA	Ovarian Cancer	Ovarian	1.97E-05	-2.63E-01	3.99E-01	-1.55E-01
5893	GHYLL_A04_47189	TCGA	Ovarian Cancer	Ovarian	1.20E-04	-2.40E-01	1.77E-01	-2.13E-01
5894	GHYLL_A05_47187	TCGA	Ovarian Cancer	Ovarian	3.89E-06	2.82E-01	1.33E-06	5.94E-01
5895	GHYLL_A06_47187	TCGA	Ovarian Cancer	Ovarian	2.31E-03	-1.97E-01	4.32E-01	-1.48E-01
5896	GHYLL_A07_47187	TCGA	Ovarian Cancer	Ovarian	6.73E-06	2.76E-01	4.34E-07	6.18E-01
5897	GHYLL_A08_47187	TCGA	Ovarian Cancer	Ovarian	2.13E-08	-3.36E-01	1.08E-01	-2.41E-01
5898	GHYLL_A09_47187	TCGA	Ovarian Cancer	Ovarian	8.33E-03	-1.75E-01	3.84E-01	1.58E-01
5899	GHYLL_A10_47188	TCGA	Ovarian Cancer	Ovarian	1.46E-04	-2.38E-01	3.35E-02	-2.98E-01
5900	GHYLL_A11_47188	TCGA	Ovarian Cancer	Ovarian	1.20E-01	-1.16E-01	2.14E-01	2.01E-01
5901	GHYLL_A12_47188	TCGA	Ovarian Cancer	Ovarian	4.81E-02	-1.39E-01	8.77E-02	2.52E-01
5902	GHYLL_B01_47206	TCGA	Ovarian Cancer	Ovarian	1.09E-02	-1.70E-01	1.80E-01	2.11E-01
5903	GHYLL_B02_47205	TCGA	Ovarian Cancer	Ovarian	3.67E-06	-2.83E-01	2.36E-01	-1.94E-01
5904	GHYLL_B03_47205	TCGA	Ovarian Cancer	Ovarian	3.50E-01	-8.20E-02	1.17E-01	2.37E-01
5905	GHYLL_B04_47205	TCGA	Ovarian Cancer	Ovarian	3.50E-12	-4.11E-01	2.01E-03	-4.03E-01
5906	GHYLL_B05_47205	TCGA	Ovarian Cancer	Ovarian	3.15E-06	-2.85E-01	8.88E-02	-2.51E-01
5907	GHYLL_B06_47205	TCGA	Ovarian Cancer	Ovarian	1.09E-05	-2.71E-01	4.38E-02	-2.86E-01
5908	GHYLL_B07_47204	TCGA	Ovarian Cancer	Ovarian	8.25E-06	-2.74E-01	1.04E-01	-2.43E-01
5909	GHYLL_B08_47204	TCGA	Ovarian Cancer	Ovarian	2.31E-04	-2.32E-01	3.55E-01	-1.64E-01
5910	GHYLL_B09_47204	TCGA	Ovarian Cancer	Ovarian	2.91E-04	-2.28E-01	5.43E-01	1.26E-01
5911	GHYLL_B10_47204	TCGA	Ovarian Cancer	Ovarian	1.77E-05	-2.65E-01	3.69E-01	-1.61E-01
5912	GHYLL_B11_47204	TCGA	Ovarian Cancer	Ovarian	1.16E-01	-1.18E-01	5.06E-01	1.33E-01
5913	GHYLL_B12_47203	TCGA	Ovarian Cancer	Ovarian	3.85E-07	-3.08E-01	1.19E-01	-2.36E-01
5914	GHYLL_C01_47203	TCGA	Ovarian Cancer	Ovarian	3.28E-05	-2.57E-01	4.76E-02	-2.82E-01
5915	GHYLL_C02_47203	TCGA	Ovarian Cancer	Ovarian	7.28E-08	-3.24E-01	2.31E-02	-3.14E-01

5916	GHYLL_C03_47203	TCGA	Ovarian Cancer	Ovarian	5.33E-02	-1.37E-01	4.61E-01	1.42E-01
5917	GHYLL_C04_47203	TCGA	Ovarian Cancer	Ovarian	8.74E-07	2.99E-01	3.88E-05	5.15E-01
5918	GHYLL_C05_47202	TCGA	Ovarian Cancer	Ovarian	4.70E-03	1.85E-01	1.94E-03	4.04E-01
5919	GHYLL_C06_47202	TCGA	Ovarian Cancer	Ovarian	2.08E-01	1.00E-01	1.77E-01	2.13E-01
5920	GHYLL_C07_47202	TCGA	Ovarian Cancer	Ovarian	5.58E-04	2.19E-01	3.05E-04	4.60E-01
5921	GHYLL_C08_47202	TCGA	Ovarian Cancer	Ovarian	4.25E-02	-1.42E-01	3.46E-01	1.66E-01
5922	GHYLL_C09_47201	TCGA	Ovarian Cancer	Ovarian	6.10E-01	-5.63E-02	3.66E-01	1.62E-01
5923	GHYLL_C10_47202	TCGA	Ovarian Cancer	Ovarian	4.80E-05	-2.52E-01	2.61E-01	-1.87E-01
5924	GHYLL_C11_47201	TCGA	Ovarian Cancer	Ovarian	4.09E-02	-1.43E-01	6.50E-01	1.06E-01
5925	GHYLL_C12_47201	TCGA	Ovarian Cancer	Ovarian	9.22E-06	-2.73E-01	7.74E-02	-2.58E-01
5926	GHYLL_D01_47201	TCGA	Ovarian Cancer	Ovarian	1.32E-03	2.06E-01	2.59E-03	3.94E-01
5927	GHYLL_D02_47201	TCGA	Ovarian Cancer	Ovarian	1.74E-04	2.35E-01	1.20E-03	4.19E-01
5928	GHYLL_D03_47200	TCGA	Ovarian Cancer	Ovarian	8.56E-07	2.99E-01	5.49E-05	5.06E-01
5929	GHYLL_D04_47200	TCGA	Ovarian Cancer	Ovarian	6.26E-13	4.24E-01	1.40E-07	6.42E-01
5930	GHYLL_D05_47200	TCGA	Ovarian Cancer	Ovarian	2.64E-03	1.95E-01	3.19E-04	4.58E-01
5931	GHYLL_D06_47200	TCGA	Ovarian Cancer	Ovarian	1.80E-01	1.05E-01	1.18E-02	3.40E-01
5932	GHYLL_D07_47199	TCGA	Ovarian Cancer	Ovarian	8.16E-04	-2.13E-01	3.93E-01	-1.56E-01
5933	GHYLL_D08_47200	TCGA	Ovarian Cancer	Ovarian	1.66E-07	-3.16E-01	1.34E-01	-2.29E-01
5934	GHYLL_D09_47199	TCGA	Ovarian Cancer	Ovarian	1.65E-05	2.66E-01	1.81E-04	4.74E-01
5935	GHYLL_D10_47199	TCGA	Ovarian Cancer	Ovarian	1.51E-01	-1.10E-01	6.17E-03	3.64E-01
5936	GHYLL_D11_47199	TCGA	Ovarian Cancer	Ovarian	2.87E-01	8.94E-02	2.17E-02	3.16E-01
5937	LENOS_A01_49116	TCGA	Ovarian Cancer	Ovarian	3.88E-01	7.79E-02	5.53E-02	2.75E-01
5938	LENOS_A02_49116	TCGA	Ovarian Cancer	Ovarian	1.47E-01	1.11E-01	5.38E-02	2.76E-01
5939	LENOS_A03_49116	TCGA	Ovarian Cancer	Ovarian	6.81E-02	-1.31E-01	1.98E-01	2.06E-01
5940	LENOS_A04_49116	TCGA	Ovarian Cancer	Ovarian	4.08E-01	7.58E-02	1.56E-01	2.20E-01
5941	LENOS_A05_49117	TCGA	Ovarian Cancer	Ovarian	8.91E-02	1.24E-01	5.60E-02	2.74E-01
5942	LENOS_A06_49117	TCGA	Ovarian Cancer	Ovarian	7.37E-09	-3.46E-01	2.77E-02	-3.06E-01
5943	LENOS_A07_49117	TCGA	Ovarian Cancer	Ovarian	1.66E-06	-2.92E-01	1.89E-02	-3.22E-01
5944	LENOS_A08_49117	TCGA	Ovarian Cancer	Ovarian	6.26E-13	4.24E-01	3.36E-09	7.14E-01
5945	LENOS_A09_49118	TCGA	Ovarian Cancer	Ovarian	1.70E-01	1.06E-01	6.23E-02	2.69E-01
5946	LENOS_A10_49117	TCGA	Ovarian Cancer	Ovarian	2.11E-01	-9.98E-02	1.66E-01	2.17E-01
5947	LENOS_A11_49118	TCGA	Ovarian Cancer	Ovarian	6.64E-03	-1.79E-01	3.49E-01	-1.66E-01
5948	LENOS_A12_49118	TCGA	Ovarian Cancer	Ovarian	1.15E-03	2.08E-01	1.94E-03	4.04E-01
5949	LENOS_B01_49110	TCGA	Ovarian Cancer	Ovarian	1.69E-03	-2.02E-01	2.95E-01	-1.78E-01
5950	LENOS_B02_49110	TCGA	Ovarian Cancer	Ovarian	1.20E-03	-2.08E-01	1.61E-01	-2.18E-01
5951	LENOS_B03_49109	TCGA	Ovarian Cancer	Ovarian	2.28E-03	-1.97E-01	4.77E-01	-1.39E-01
5952	LENOS_B04_49110	TCGA	Ovarian Cancer	Ovarian	2.18E-02	-1.56E-01	5.26E-01	1.30E-01
5953	LENOS_B05_49111	TCGA	Ovarian Cancer	Ovarian	2.50E-02	-1.54E-01	5.43E-01	1.26E-01
5954	LENOS_B06_49111	TCGA	Ovarian Cancer	Ovarian	1.34E-01	1.13E-01	1.64E-01	2.17E-01
5955	LENOS_B07_49110	TCGA	Ovarian Cancer	Ovarian	2.92E-02	-1.50E-01	4.80E-01	-1.38E-01
5956	LENOS_B08_49110	TCGA	Ovarian Cancer	Ovarian	5.19E-02	-1.38E-01	3.16E-01	-1.73E-01
5957	LENOS_B09_49111	TCGA	Ovarian Cancer	Ovarian	9.02E-04	2.12E-01	6.28E-03	3.64E-01
5958	LENOS_B10_49112	TCGA	Ovarian Cancer	Ovarian	1.28E-02	1.67E-01	3.13E-03	3.88E-01
5959	LENOS_B11_49111	TCGA	Ovarian Cancer	Ovarian	4.53E-03	1.86E-01	9.52E-03	3.49E-01
5960	LENOS_B12_49111	TCGA	Ovarian Cancer	Ovarian	7.64E-02	-1.28E-01	2.27E-01	1.97E-01
5961	LENOS_C01_49112	TCGA	Ovarian Cancer	Ovarian	6.40E-09	-3.48E-01	2.77E-02	-3.06E-01
5962	LENOS_C02_49112	TCGA	Ovarian Cancer	Ovarian	1.17E-12	-4.19E-01	5.40E-04	-4.43E-01
5963	LENOS_C03_49112	TCGA	Ovarian Cancer	Ovarian	2.44E-12	-4.14E-01	9.17E-04	-4.27E-01
5964	LENOS_C04_49112	TCGA	Ovarian Cancer	Ovarian	5.26E-04	-2.20E-01	5.23E-01	-1.30E-01
5965	LENOS_C05_49113	TCGA	Ovarian Cancer	Ovarian	4.20E-03	1.87E-01	9.17E-04	4.27E-01
5966	LENOS_C06_49113	TCGA	Ovarian Cancer	Ovarian	2.05E-03	-1.99E-01	2.80E-01	1.82E-01
5967	LENOS_C07_49113	TCGA	Ovarian Cancer	Ovarian	3.74E-06	2.83E-01	2.05E-05	5.31E-01
5968	LENOS_C08_49113	TCGA	Ovarian Cancer	Ovarian	1.02E-02	-1.71E-01	2.00E-01	-2.05E-01
5969	LENOS_C09_49114	TCGA	Ovarian Cancer	Ovarian	3.33E-07	-3.09E-01	7.65E-02	-2.59E-01
5970	LENOS_C10_49114	TCGA	Ovarian Cancer	Ovarian	2.50E-02	1.54E-01	2.68E-02	3.07E-01
5971	LENOS_C11_49114	TCGA	Ovarian Cancer	Ovarian	3.21E-03	1.92E-01	2.27E-02	3.14E-01
5972	LENOS_C12_49113	TCGA	Ovarian Cancer	Ovarian	2.23E-01	9.81E-02	6.23E-02	2.69E-01
5973	LENOS_D01_49115	TCGA	Ovarian Cancer	Ovarian	1.05E-02	-1.71E-01	6.70E-01	1.02E-01
5974	LENOS_D02_49115	TCGA	Ovarian Cancer	Ovarian	1.59E-01	1.08E-01	1.01E-01	2.44E-01
5975	LENOS_D03_49114	TCGA	Ovarian Cancer	Ovarian	6.80E-03	1.79E-01	6.17E-03	3.64E-01
5976	LENOS_D04_49114	TCGA	Ovarian Cancer	Ovarian	2.28E-02	-1.56E-01	4.26E-01	1.49E-01
5977	LENOS_D05_49116	TCGA	Ovarian Cancer	Ovarian	1.11E-09	3.63E-01	1.88E-06	5.87E-01
5978	LENOS_D06_49115	TCGA	Ovarian Cancer	Ovarian	1.26E-02	-1.67E-01	4.51E-02	2.84E-01
5979	LENOS_D07_49115	TCGA	Ovarian Cancer	Ovarian	2.66E-09	-3.56E-01	9.21E-02	-2.50E-01
5980	LENOS_D08_49115	TCGA	Ovarian Cancer	Ovarian	1.00E-01	-1.21E-01	3.14E-01	1.74E-01
5981	LENOS_D09_49123	TCGA	Ovarian Cancer	Ovarian	1.25E-01	-1.15E-01	2.83E-01	1.82E-01
5982	LENOS_D10_49123	TCGA	Ovarian Cancer	Ovarian	3.12E-07	-3.10E-01	1.96E-01	-2.06E-01
5983	LENOS_E01_49122	TCGA	Ovarian Cancer	Ovarian	7.12E-08	-3.25E-01	2.31E-02	-3.14E-01
5984	LENOS_E02_49122	TCGA	Ovarian Cancer	Ovarian	4.57E-01	-7.08E-02	1.20E-02	3.40E-01
5985	LENOS_E03_49123	TCGA	Ovarian Cancer	Ovarian	6.28E-10	-3.68E-01	7.88E-03	-3.56E-01
5986	LENOS_E04_49123	TCGA	Ovarian Cancer	Ovarian	1.00E-01	-1.21E-01	1.34E-01	2.29E-01
5987	LENOS_E05_49125	TCGA	Ovarian Cancer	Ovarian	6.52E-08	3.26E-01	6.59E-07	6.10E-01
5988	LENOS_E06_49125	TCGA	Ovarian Cancer	Ovarian	1.07E-02	-1.71E-01	3.22E-01	-1.72E-01
5989	LENOS_E07_49125	TCGA	Ovarian Cancer	Ovarian	2.27E-06	-2.89E-01	7.10E-01	-9.46E-02

5990	LENOS_E08_49125	TCGA	Ovarian Cancer	Ovarian	2.21E-01	9.83E-02	1.46E-02	3.32E-01
5991	LENOS_E09_49124	TCGA	Ovarian Cancer	Ovarian	9.37E-03	-1.73E-01	2.85E-01	1.81E-01
5992	LENOS_E10_49124	TCGA	Ovarian Cancer	Ovarian	9.71E-03	-1.72E-01	5.00E-01	1.35E-01
5993	LENOS_E11_49124	TCGA	Ovarian Cancer	Ovarian	2.68E-01	9.18E-02	1.59E-03	4.10E-01
5994	LENOS_E12_49124	TCGA	Ovarian Cancer	Ovarian	8.64E-04	-2.13E-01	5.96E-01	1.16E-01
5995	LENOS_F01_49126	TCGA	Ovarian Cancer	Ovarian	5.56E-09	-3.49E-01	5.60E-02	-2.74E-01
5996	LENOS_F02_49126	TCGA	Ovarian Cancer	Ovarian	2.37E-03	1.97E-01	1.17E-03	4.20E-01
5997	LENOS_F03_49127	TCGA	Ovarian Cancer	Ovarian	3.01E-01	-8.77E-02	2.66E-01	1.86E-01
5998	LENOS_F04_49127	TCGA	Ovarian Cancer	Ovarian	1.01E-03	-2.10E-01	4.02E-01	-1.54E-01
5999	LENOS_F05_49126	TCGA	Ovarian Cancer	Ovarian	1.05E-02	1.71E-01	5.64E-04	4.42E-01
6000	LENOS_F06_49125	TCGA	Ovarian Cancer	Ovarian	3.13E-03	-1.92E-01	2.29E-01	-1.96E-01
6001	LENOS_F07_49126	TCGA	Ovarian Cancer	Ovarian	3.80E-10	-3.73E-01	1.56E-02	-3.30E-01
6002	LENOS_F08_49126	TCGA	Ovarian Cancer	Ovarian	1.65E-01	1.07E-01	1.18E-01	2.36E-01
6003	LENOS_F09_49128	TCGA	Ovarian Cancer	Ovarian	6.06E-02	-1.34E-01	2.02E-01	2.04E-01
6004	LENOS_F10_49128	TCGA	Ovarian Cancer	Ovarian	2.47E-01	-9.46E-02	1.19E-01	2.36E-01
6005	LENOS_F11_49128	TCGA	Ovarian Cancer	Ovarian	3.85E-07	-3.08E-01	1.83E-02	-3.23E-01
6006	LENOS_F12_49128	TCGA	Ovarian Cancer	Ovarian	1.62E-06	-2.92E-01	1.59E-01	-2.19E-01
6007	LENOS_G01_49127	TCGA	Ovarian Cancer	Ovarian	2.02E-13	-4.33E-01	4.98E-03	-3.72E-01
6008	LENOS_G02_49127	TCGA	Ovarian Cancer	Ovarian	1.47E-01	1.11E-01	3.81E-02	2.92E-01
6009	LENOS_G03_49128	TCGA	Ovarian Cancer	Ovarian	3.46E-01	8.25E-02	1.05E-01	2.43E-01
6010	LENOS_G04_49127	TCGA	Ovarian Cancer	Ovarian	2.72E-01	-9.13E-02	9.90E-02	2.46E-01
6011	LENOS_G05_49119	TCGA	Ovarian Cancer	Ovarian	3.71E-01	-7.97E-02	2.58E-01	1.88E-01
6012	LENOS_G06_49120	TCGA	Ovarian Cancer	Ovarian	2.48E-02	-1.54E-01	2.38E-01	1.94E-01
6013	LENOS_G07_49119	TCGA	Ovarian Cancer	Ovarian	1.93E-01	1.03E-01	1.05E-01	2.43E-01
6014	LENOS_G08_49119	TCGA	Ovarian Cancer	Ovarian	1.35E-02	-1.66E-01	9.90E-02	2.46E-01
6015	LENOS_G09_49119	TCGA	Ovarian Cancer	Ovarian	5.54E-01	-6.15E-02	1.24E-01	2.34E-01
6016	LENOS_G10_49119	TCGA	Ovarian Cancer	Ovarian	2.07E-04	-2.33E-01	1.82E-01	-2.11E-01
6017	LENOS_G11_49118	TCGA	Ovarian Cancer	Ovarian	5.09E-02	-1.38E-01	5.83E-01	1.19E-01
6018	LENOS_G12_49118	TCGA	Ovarian Cancer	Ovarian	4.25E-02	-1.42E-01	3.46E-01	1.66E-01
6019	LENOS_H01_49121	TCGA	Ovarian Cancer	Ovarian	6.98E-12	-4.06E-01	3.40E-02	-2.97E-01
6020	LENOS_H02_49121	TCGA	Ovarian Cancer	Ovarian	2.37E-03	1.97E-01	5.28E-04	4.44E-01
6021	LENOS_H03_49121	TCGA	Ovarian Cancer	Ovarian	1.45E-01	-1.11E-01	1.59E-01	2.19E-01
6022	LENOS_H04_49121	TCGA	Ovarian Cancer	Ovarian	1.52E-09	-3.61E-01	4.70E-02	-2.83E-01
6023	LENOS_H05_49120	TCGA	Ovarian Cancer	Ovarian	8.24E-10	-3.66E-01	1.51E-02	-3.31E-01
6024	LENOS_H06_49120	TCGA	Ovarian Cancer	Ovarian	1.63E-04	-2.36E-01	2.98E-01	-1.78E-01
6025	LENOS_H07_49120	TCGA	Ovarian Cancer	Ovarian	1.29E-02	1.67E-01	1.31E-04	4.83E-01
6026	LENOS_H08_49120	TCGA	Ovarian Cancer	Ovarian	1.30E-03	2.06E-01	3.13E-03	3.88E-01
6027	LENOS_H09_49122	TCGA	Ovarian Cancer	Ovarian	1.38E-03	2.05E-01	2.54E-03	3.95E-01
6028	LENOS_H10_49122	TCGA	Ovarian Cancer	Ovarian	1.91E-01	1.03E-01	1.18E-02	3.40E-01
6029	LENOS_H11_49122	TCGA	Ovarian Cancer	Ovarian	2.23E-02	-1.56E-01	6.27E-01	1.10E-01
6030	PSHAW_A01_4337	TCGA	Ovarian Cancer	Ovarian	2.26E-02	1.56E-01	3.92E-03	3.80E-01
6031	PSHAW_A02_4337	TCGA	Ovarian Cancer	Ovarian	7.91E-02	-1.27E-01	3.96E-01	-1.56E-01
6032	PSHAW_A03_4337	TCGA	Ovarian Cancer	Ovarian	5.17E-06	-2.79E-01	1.41E-01	-2.26E-01
6033	PSHAW_A04_4337	TCGA	Ovarian Cancer	Ovarian	1.37E-04	-2.39E-01	1.22E-01	-2.34E-01
6034	PSHAW_A05_4337	TCGA	Ovarian Cancer	Ovarian	8.46E-35	-7.09E-01	1.50E-09	-7.28E-01
6035	PSHAW_A07_4337	TCGA	Ovarian Cancer	Ovarian	1.64E-01	1.08E-01	3.97E-02	2.90E-01
6036	PSHAW_A08_4337	TCGA	Ovarian Cancer	Ovarian	2.31E-03	-1.97E-01	6.23E-01	1.11E-01
6037	PSHAW_A09_4337	TCGA	Ovarian Cancer	Ovarian	2.03E-02	-1.58E-01	5.19E-01	1.31E-01
6038	PSHAW_A10_4337	TCGA	Ovarian Cancer	Ovarian	6.42E-01	5.32E-02	5.38E-02	2.76E-01
6039	PSHAW_A11_4337	TCGA	Ovarian Cancer	Ovarian	8.24E-03	-1.75E-01	2.06E-01	-2.03E-01
6040	PSHAW_A12_4337	TCGA	Ovarian Cancer	Ovarian	1.58E-01	1.09E-01	1.20E-02	3.40E-01
6041	PSHAW_B01_4337	TCGA	Ovarian Cancer	Ovarian	8.04E-03	-1.76E-01	2.08E-01	2.03E-01
6042	PSHAW_B02_4337	TCGA	Ovarian Cancer	Ovarian	7.84E-02	1.28E-01	5.24E-02	2.77E-01
6043	PSHAW_B03_4337	TCGA	Ovarian Cancer	Ovarian	4.35E-11	-3.91E-01	8.02E-03	-3.55E-01
6044	PSHAW_B04_4337	TCGA	Ovarian Cancer	Ovarian	1.72E-03	2.02E-01	6.83E-04	4.36E-01
6045	PSHAW_B06_4337	TCGA	Ovarian Cancer	Ovarian	4.17E-02	1.43E-01	5.60E-02	2.74E-01
6046	PSHAW_B07_4337	TCGA	Ovarian Cancer	Ovarian	7.40E-03	1.77E-01	1.89E-02	3.22E-01
6047	PSHAW_B08_4337	TCGA	Ovarian Cancer	Ovarian	1.04E-02	-1.71E-01	4.20E-01	1.50E-01
6048	PSHAW_B09_4337	TCGA	Ovarian Cancer	Ovarian	1.87E-01	-1.04E-01	4.39E-01	1.47E-01
6049	PSHAW_B10_4337	TCGA	Ovarian Cancer	Ovarian	4.20E-06	-2.82E-01	1.19E-01	-2.36E-01
6050	PSHAW_B11_4337	TCGA	Ovarian Cancer	Ovarian	4.29E-02	1.42E-01	9.52E-03	3.49E-01
6051	PSHAW_B12_4337	TCGA	Ovarian Cancer	Ovarian	3.98E-02	-1.44E-01	2.63E-01	1.87E-01
6052	PSHAW_C01_4337	TCGA	Ovarian Cancer	Ovarian	2.55E-06	-2.87E-01	2.04E-01	-2.04E-01
6053	PSHAW_C02_4337	TCGA	Ovarian Cancer	Ovarian	2.04E-01	1.01E-01	1.18E-01	2.36E-01
6054	PSHAW_C03_4337	TCGA	Ovarian Cancer	Ovarian	2.69E-02	-1.52E-01	5.83E-01	1.19E-01
6055	PSHAW_C04_4336	TCGA	Ovarian Cancer	Ovarian	3.18E-01	-8.57E-02	1.80E-01	2.11E-01
6056	PSHAW_C05_4336	TCGA	Ovarian Cancer	Ovarian	2.21E-01	9.83E-02	1.51E-02	3.31E-01
6057	PSHAW_C06_4337	TCGA	Ovarian Cancer	Ovarian	2.73E-08	-3.34E-01	6.48E-02	-2.67E-01
6058	PSHAW_C07_4337	TCGA	Ovarian Cancer	Ovarian	6.40E-02	-1.33E-01	1.00E-01	2.45E-01
6059	PSHAW_C08_4336	TCGA	Ovarian Cancer	Ovarian	5.59E-02	1.36E-01	3.70E-02	2.93E-01
6060	PSHAW_C09_4336	TCGA	Ovarian Cancer	Ovarian	1.21E-03	-2.07E-01	1.77E-01	-2.13E-01
6061	PSHAW_C10_4336	TCGA	Ovarian Cancer	Ovarian	6.19E-07	-3.03E-01	3.99E-01	-1.55E-01
6062	PSHAW_C11_4336	TCGA	Ovarian Cancer	Ovarian	1.71E-05	2.65E-01	2.05E-05	5.31E-01
6063	PSHAW_C12_4336	TCGA	Ovarian Cancer	Ovarian	1.50E-03	2.04E-01	1.50E-05	5.38E-01

6064	PSHAW_D01_4336	TCGA	Ovarian Cancer	Ovarian	3.53E-02	1.46E-01	3.19E-04	4.58E-01
6065	PSHAW_D02_4336	TCGA	Ovarian Cancer	Ovarian	1.38E-03	-2.05E-01	6.53E-01	1.05E-01
6066	PSHAW_D03_4336	TCGA	Ovarian Cancer	Ovarian	3.20E-02	-1.48E-01	1.34E-01	2.29E-01
6067	SEDUM_A01_4588	TCGA	Ovarian Cancer	Ovarian	2.58E-12	-4.13E-01	2.05E-03	-4.02E-01
6068	SEDUM_A02_4588	TCGA	Ovarian Cancer	Ovarian	2.38E-04	2.31E-01	1.90E-03	4.04E-01
6069	SEDUM_A03_4588	TCGA	Ovarian Cancer	Ovarian	3.71E-05	-2.56E-01	1.56E-01	-2.20E-01
6070	SEDUM_A04_4588	TCGA	Ovarian Cancer	Ovarian	7.91E-02	-1.27E-01	2.88E-01	1.80E-01
6071	SEDUM_A05_4588	TCGA	Ovarian Cancer	Ovarian	9.37E-03	-1.73E-01	5.00E-01	1.35E-01
6072	SEDUM_A06_4588	TCGA	Ovarian Cancer	Ovarian	2.82E-03	1.94E-01	4.35E-04	4.50E-01
6073	SEDUM_A07_4588	TCGA	Ovarian Cancer	Ovarian	5.46E-03	1.83E-01	3.13E-03	3.88E-01
6074	SEDUM_A08_4588	TCGA	Ovarian Cancer	Ovarian	3.43E-02	-1.47E-01	2.80E-01	1.82E-01
6075	SEDUM_A09_4588	TCGA	Ovarian Cancer	Ovarian	1.32E-09	-3.62E-01	2.31E-02	-3.14E-01
6076	SEDUM_A10_4587	TCGA	Ovarian Cancer	Ovarian	6.06E-02	1.34E-01	1.20E-03	4.19E-01
6077	SEDUM_A11_4587	TCGA	Ovarian Cancer	Ovarian	2.99E-08	-3.33E-01	4.83E-02	-2.81E-01
6078	SEDUM_A12_4587	TCGA	Ovarian Cancer	Ovarian	3.34E-05	-2.57E-01	3.55E-01	-1.64E-01
6079	SEDUM_B01_4586	TCGA	Ovarian Cancer	Ovarian	8.04E-02	1.27E-01	1.75E-02	3.25E-01
6080	SEDUM_B02_4586	TCGA	Ovarian Cancer	Ovarian	1.84E-01	-1.04E-01	4.83E-02	2.81E-01
6081	SEDUM_B03_4586	TCGA	Ovarian Cancer	Ovarian	8.30E-08	-3.23E-01	1.56E-01	-2.20E-01
6082	SEDUM_B04_4586	TCGA	Ovarian Cancer	Ovarian	2.13E-01	-9.96E-02	1.32E-01	2.30E-01
6083	SEDUM_B05_4586	TCGA	Ovarian Cancer	Ovarian	9.44E-02	-1.23E-01	3.84E-01	1.58E-01
6084	SEDUM_B06_4586	TCGA	Ovarian Cancer	Ovarian	7.06E-02	1.30E-01	1.40E-01	2.27E-01
6085	SEDUM_B07_4586	TCGA	Ovarian Cancer	Ovarian	1.02E-08	-3.43E-01	1.00E-02	-3.47E-01
6086	SEDUM_B08_4586	TCGA	Ovarian Cancer	Ovarian	1.48E-10	-3.81E-01	3.12E-04	-4.59E-01
6087	SEDUM_B09_4586	TCGA	Ovarian Cancer	Ovarian	6.87E-02	-1.31E-01	3.90E-01	1.57E-01
6088	SEDUM_B10_4586	TCGA	Ovarian Cancer	Ovarian	9.83E-04	-2.11E-01	3.84E-01	1.58E-01
6089	SEDUM_B11_4586	TCGA	Ovarian Cancer	Ovarian	3.20E-02	-1.48E-01	4.32E-01	1.48E-01
6090	SEDUM_B12_4586	TCGA	Ovarian Cancer	Ovarian	5.69E-02	1.35E-01	3.92E-03	3.80E-01
6091	SEDUM_C01_4586	TCGA	Ovarian Cancer	Ovarian	2.40E-03	1.97E-01	9.52E-03	3.49E-01
6092	SEDUM_C02_4586	TCGA	Ovarian Cancer	Ovarian	1.33E-06	2.94E-01	2.79E-05	5.23E-01
6093	SEDUM_C04_4586	TCGA	Ovarian Cancer	Ovarian	1.10E-07	-3.20E-01	6.73E-02	-2.65E-01
6094	SEDUM_C05_4586	TCGA	Ovarian Cancer	Ovarian	3.12E-07	3.10E-01	2.64E-06	5.79E-01
6095	SEDUM_C06_4586	TCGA	Ovarian Cancer	Ovarian	1.94E-03	-2.00E-01	3.27E-01	-1.71E-01
6096	SEDUM_C07_4586	TCGA	Ovarian Cancer	Ovarian	2.08E-05	-2.63E-01	1.82E-01	-2.11E-01
6097	SEDUM_C08_4586	TCGA	Ovarian Cancer	Ovarian	3.43E-02	1.47E-01	1.44E-02	3.33E-01
6098	SEDUM_C09_4586	TCGA	Ovarian Cancer	Ovarian	9.06E-02	1.24E-01	1.53E-03	4.11E-01
6099	SEDUM_C10_4586	TCGA	Ovarian Cancer	Ovarian	4.88E-03	-1.85E-01	1.86E-01	2.10E-01
6100	SEDUM_C11_4586	TCGA	Ovarian Cancer	Ovarian	3.94E-02	-1.44E-01	3.84E-01	1.58E-01
6101	SEDUM_C12_4586	TCGA	Ovarian Cancer	Ovarian	3.10E-04	-2.27E-01	1.80E-01	-2.11E-01
6102	SEDUM_D01_4586	TCGA	Ovarian Cancer	Ovarian	2.51E-01	9.42E-02	5.24E-02	2.77E-01
6103	SEDUM_D02_4586	TCGA	Ovarian Cancer	Ovarian	2.08E-03	-1.99E-01	4.39E-01	-1.47E-01
6104	SEDUM_D03_4586	TCGA	Ovarian Cancer	Ovarian	2.54E-01	-9.37E-02	3.22E-01	1.72E-01
6105	SEDUM_D04_4586	TCGA	Ovarian Cancer	Ovarian	2.57E-04	-2.30E-01	7.20E-01	-9.27E-02
6106	SEDUM_D05_4586	TCGA	Ovarian Cancer	Ovarian	3.21E-01	-8.53E-02	7.27E-02	2.62E-01
6107	SEDUM_D06_4586	TCGA	Ovarian Cancer	Ovarian	3.23E-02	-1.48E-01	4.63E-02	2.83E-01
6108	SEDUM_D07_4586	TCGA	Ovarian Cancer	Ovarian	4.09E-02	-1.43E-01	1.75E-01	2.13E-01
6109	SEDUM_D08_4586	TCGA	Ovarian Cancer	Ovarian	1.12E-01	1.18E-01	6.17E-03	3.64E-01
6110	SEDUM_D09_4587	TCGA	Ovarian Cancer	Ovarian	2.49E-01	9.44E-02	5.31E-02	2.77E-01
6111	SEDUM_D10_4587	TCGA	Ovarian Cancer	Ovarian	9.70E-05	2.43E-01	2.98E-04	4.60E-01
6112	SEDUM_D11_4587	TCGA	Ovarian Cancer	Ovarian	3.68E-02	1.45E-01	8.66E-02	2.53E-01
6113	ovarial15104-01	Rubys Data	Ovarian Cancer	Ovarian	8.77E-04	2.12E-01	1.56E-03	4.11E-01
6114	ovarial1971-99	Rubys Data	Ovarian Cancer	Ovarian	6.87E-09	-3.47E-01	1.80E-02	-3.24E-01
6115	ovarial2392-99	Rubys Data	Ovarian Cancer	Ovarian	2.00E-04	-2.34E-01	2.88E-01	-1.80E-01
6116	ovarial32736-99	Rubys Data	Ovarian Cancer	Ovarian	4.31E-03	-1.87E-01	2.66E-01	1.86E-01
6117	ovarial3803-02	Rubys Data	Ovarian Cancer	Ovarian	4.25E-12	-4.10E-01	1.98E-03	-4.03E-01
6118	ovarial9787-03	Rubys Data	Ovarian Cancer	Ovarian	6.87E-02	1.31E-01	8.56E-02	2.53E-01
6119	ovarial_12216-01	Rubys Data	Ovarian Cancer	Ovarian	1.68E-04	-2.36E-01	1.37E-01	-2.28E-01
6120	ovarial_14508-02	Rubys Data	Ovarian Cancer	Ovarian	8.76E-02	-1.25E-01	1.98E-01	2.06E-01
6121	ovarial_1462-01	Rubys Data	Ovarian Cancer	Ovarian	1.99E-03	-2.00E-01	3.22E-01	-1.72E-01
6122	ovarial_17118-01	Rubys Data	Ovarian Cancer	Ovarian	2.82E-04	2.29E-01	3.05E-04	4.60E-01
6123	ovarial_19826-01	Rubys Data	Ovarian Cancer	Ovarian	1.28E-06	-2.95E-01	5.45E-02	-2.76E-01
6124	ovarial_27475-01	Rubys Data	Ovarian Cancer	Ovarian	1.50E-03	-2.04E-01	3.24E-01	-1.71E-01
6125	ovarial_29502-01	Rubys Data	Ovarian Cancer	Ovarian	1.02E-01	-1.21E-01	2.80E-01	1.82E-01
6126	ovarial_3206-04	Rubys Data	Ovarian Cancer	Ovarian	1.41E-04	-2.38E-01	1.96E-01	-2.06E-01
6127	ovarial_34577-98	Rubys Data	Ovarian Cancer	Ovarian	3.95E-04	-2.24E-01	3.49E-01	-1.66E-01
6128	ovarial_3617-04	Rubys Data	Ovarian Cancer	Ovarian	3.44E-01	-8.27E-02	1.06E-01	2.42E-01
6129	ovarial_3921-03	Rubys Data	Ovarian Cancer	Ovarian	7.95E-05	-2.46E-01	7.65E-02	-2.59E-01
6130	ovarial_4115-02	Rubys Data	Ovarian Cancer	Ovarian	1.46E-02	-1.65E-01	5.83E-01	1.19E-01
6131	ovarial_4424-99	Rubys Data	Ovarian Cancer	Ovarian	1.46E-02	1.65E-01	5.52E-04	4.43E-01
6132	ovarial_54402-98	Rubys Data	Ovarian Cancer	Ovarian	4.56E-13	4.27E-01	1.23E-08	6.90E-01
6133	ovarial_7025-04	Rubys Data	Ovarian Cancer	Ovarian	6.12E-02	1.34E-01	2.38E-04	4.67E-01
6134	ovarial_7890-02	Rubys Data	Ovarian Cancer	Ovarian	1.48E-03	-2.04E-01	4.83E-01	1.38E-01
6135	ovarial_8556-02	Rubys Data	Ovarian Cancer	Ovarian	2.12E-05	-2.63E-01	2.88E-01	-1.80E-01
6136	ovarial_8567-04	Rubys Data	Ovarian Cancer	Ovarian	4.42E-03	-1.86E-01	3.19E-01	-1.73E-01
6137	ovarial_9136-04	Rubys Data	Ovarian Cancer	Ovarian	2.62E-01	9.26E-02	6.62E-03	3.62E-01

6138	ovarial_9896-01	Rubys Data	Ovarian Cancer	Ovarian	1.82E-02	-1.60E-01	5.00E-01	1.35E-01
6139	GSM139377	GSE6008	Ovarian Cancer	Ovarian	2.14E-02	-1.57E-01	1.24E-01	2.34E-01
6140	GSM139378	GSE6008	Ovarian Cancer	Ovarian	9.60E-03	-1.73E-01	3.61E-01	-1.63E-01
6141	GSM139379	GSE6008	Ovarian Cancer	Ovarian	9.83E-04	-2.11E-01	3.22E-01	-1.72E-01
6142	GSM139380	GSE6008	Ovarian Cancer	Ovarian	2.14E-09	-3.58E-01	2.72E-02	-3.07E-01
6143	GSM139381	GSE6008	Ovarian Cancer	Ovarian	4.44E-06	-2.81E-01	8.99E-02	-2.51E-01
6144	GSM139382	GSE6008	Ovarian Cancer	Ovarian	4.97E-01	6.69E-02	1.21E-01	2.35E-01
6145	GSM139383	GSE6008	Ovarian Cancer	Ovarian	4.84E-01	-6.82E-02	1.43E-01	2.25E-01
6146	GSM139384	GSE6008	Ovarian Cancer	Ovarian	2.28E-07	-3.13E-01	8.02E-03	-3.55E-01
6147	GSM139385	GSE6008	Ovarian Cancer	Ovarian	2.97E-03	-1.93E-01	1.51E-01	-2.22E-01
6148	GSM139386	GSE6008	Ovarian Cancer	Ovarian	1.04E-01	-1.20E-01	3.30E-01	1.70E-01
6149	GSM139387	GSE6008	Ovarian Cancer	Ovarian	2.33E-02	1.55E-01	6.39E-03	3.63E-01
6150	GSM139388	GSE6008	Ovarian Cancer	Ovarian	2.30E-02	1.55E-01	8.02E-03	3.55E-01
6151	GSM139389	GSE6008	Ovarian Cancer	Ovarian	7.38E-02	-1.29E-01	3.46E-01	1.66E-01
6152	GSM139390	GSE6008	Ovarian Cancer	Ovarian	2.11E-01	-9.98E-02	4.58E-01	1.43E-01
6153	GSM139391	GSE6008	Ovarian Cancer	Ovarian	7.82E-05	2.46E-01	2.05E-03	4.02E-01
6154	GSM139392	GSE6008	Ovarian Cancer	Ovarian	2.79E-09	-3.55E-01	1.86E-02	-3.23E-01
6155	GSM139393	GSE6008	Ovarian Cancer	Ovarian	2.13E-03	-1.98E-01	2.58E-01	-1.88E-01
6156	GSM139394	GSE6008	Ovarian Cancer	Ovarian	1.82E-04	-2.35E-01	2.93E-01	-1.79E-01
6157	GSM139395	GSE6008	Ovarian Cancer	Ovarian	1.32E-03	-2.06E-01	2.54E-01	-1.89E-01
6158	GSM139396	GSE6008	Ovarian Cancer	Ovarian	3.64E-02	-1.46E-01	3.58E-01	1.64E-01
6159	GSM139397	GSE6008	Ovarian Cancer	Ovarian	8.53E-03	1.75E-01	3.25E-03	3.87E-01
6160	GSM139398	GSE6008	Ovarian Cancer	Ovarian	2.07E-01	1.00E-01	1.15E-01	2.37E-01
6161	GSM139399	GSE6008	Ovarian Cancer	Ovarian	2.27E-17	4.95E-01	8.62E-11	7.78E-01
6162	GSM139400	GSE6008	Ovarian Cancer	Ovarian	1.53E-05	-2.66E-01	1.18E-01	-2.36E-01
6163	GSM139401	GSE6008	Ovarian Cancer	Ovarian	3.29E-04	-2.27E-01	4.63E-02	-2.83E-01
6164	GSM139402	GSE6008	Ovarian Cancer	Ovarian	1.94E-03	-2.00E-01	4.77E-01	1.39E-01
6165	GSM139403	GSE6008	Ovarian Cancer	Ovarian	6.87E-09	-3.47E-01	2.81E-02	-3.05E-01
6166	GSM139404	GSE6008	Ovarian Cancer	Ovarian	1.47E-01	1.11E-01	2.05E-03	4.02E-01
6167	GSM139405	GSE6008	Ovarian Cancer	Ovarian	1.35E-01	-1.13E-01	2.80E-01	1.82E-01
6168	GSM139406	GSE6008	Ovarian Cancer	Ovarian	6.58E-02	1.32E-01	7.36E-02	2.61E-01
6169	GSM139407	GSE6008	Ovarian Cancer	Ovarian	2.70E-04	-2.29E-01	1.37E-01	-2.28E-01
6170	GSM139408	GSE6008	Ovarian Cancer	Ovarian	7.69E-05	-2.46E-01	3.81E-01	-1.59E-01
6171	GSM139409	GSE6008	Ovarian Cancer	Ovarian	1.09E-01	-1.19E-01	8.77E-02	2.52E-01
6172	GSM139410	GSE6008	Ovarian Cancer	Ovarian	1.69E-02	-1.62E-01	4.29E-01	1.49E-01
6173	GSM139411	GSE6008	Ovarian Cancer	Ovarian	1.50E-03	-2.04E-01	3.46E-01	1.66E-01
6174	GSM139412	GSE6008	Ovarian Cancer	Ovarian	1.26E-01	1.15E-01	1.51E-01	2.22E-01
6175	GSM139413	GSE6008	Ovarian Cancer	Ovarian	6.24E-08	3.26E-01	4.30E-08	6.65E-01
6176	GSM139414	GSE6008	Ovarian Cancer	Ovarian	5.39E-03	-1.83E-01	3.49E-01	1.66E-01
6177	GSM139415	GSE6008	Ovarian Cancer	Ovarian	1.16E-04	-2.41E-01	4.42E-01	-1.46E-01
6178	GSM139416	GSE6008	Ovarian Cancer	Ovarian	5.00E-01	6.67E-02	7.46E-02	2.60E-01
6179	GSM139417	GSE6008	Ovarian Cancer	Ovarian	1.68E-05	2.65E-01	5.63E-05	5.05E-01
6180	GSM139418	GSE6008	Ovarian Cancer	Ovarian	7.77E-08	-3.24E-01	2.72E-02	-3.07E-01
6181	GSM139419	GSE6008	Ovarian Cancer	Ovarian	9.68E-02	1.22E-01	3.86E-02	2.91E-01
6182	GSM139420	GSE6008	Ovarian Cancer	Ovarian	1.60E-09	-3.60E-01	6.73E-02	-2.65E-01
6183	GSM139421	GSE6008	Ovarian Cancer	Ovarian	8.48E-08	-3.23E-01	5.75E-02	-2.73E-01
6184	GSM139422	GSE6008	Ovarian Cancer	Ovarian	1.94E-08	-3.37E-01	5.53E-02	-2.75E-01
6185	GSM139423	GSE6008	Ovarian Cancer	Ovarian	8.89E-09	-3.45E-01	2.68E-02	-3.07E-01
6186	GSM139424	GSE6008	Ovarian Cancer	Ovarian	1.52E-10	3.81E-01	6.59E-07	6.10E-01
6187	GSM139425	GSE6008	Ovarian Cancer	Ovarian	5.56E-09	-3.49E-01	9.21E-02	-2.50E-01
6188	GSM139426	GSE6008	Ovarian Cancer	Ovarian	1.23E-08	-3.42E-01	3.16E-02	-3.00E-01
6189	GSM139427	GSE6008	Ovarian Cancer	Ovarian	3.04E-13	-4.30E-01	1.81E-04	-4.74E-01
6190	GSM139428	GSE6008	Ovarian Cancer	Ovarian	4.95E-18	-5.05E-01	4.08E-05	-5.14E-01
6191	GSM139429	GSE6008	Ovarian Cancer	Ovarian	6.46E-21	-5.46E-01	2.05E-05	-5.31E-01
6192	GSM139430	GSE6008	Ovarian Cancer	Ovarian	6.72E-03	-1.79E-01	6.37E-01	1.09E-01
6193	GSM139431	GSE6008	Ovarian Cancer	Ovarian	1.05E-11	-4.02E-01	1.20E-03	-4.19E-01
6194	GSM139432	GSE6008	Ovarian Cancer	Ovarian	1.57E-26	-6.17E-01	6.59E-07	-6.10E-01
6195	GSM139433	GSE6008	Ovarian Cancer	Ovarian	8.08E-05	-2.46E-01	1.00E-01	-2.45E-01
6196	GSM139434	GSE6008	Ovarian Cancer	Ovarian	7.97E-02	-1.27E-01	1.40E-01	2.27E-01
6197	GSM139435	GSE6008	Ovarian Cancer	Ovarian	1.79E-06	-2.91E-01	2.68E-02	-3.07E-01
6198	GSM139436	GSE6008	Ovarian Cancer	Ovarian	1.03E-01	1.21E-01	1.80E-02	3.24E-01
6199	GSM139437	GSE6008	Ovarian Cancer	Ovarian	1.06E-01	-1.20E-01	2.22E-01	1.98E-01
6200	GSM139438	GSE6008	Ovarian Cancer	Ovarian	3.01E-02	-1.50E-01	1.18E-01	2.36E-01
6201	GSM139439	GSE6008	Ovarian Cancer	Ovarian	1.26E-01	1.15E-01	3.25E-02	2.99E-01
6202	GSM139440	GSE6008	Ovarian Cancer	Ovarian	1.73E-01	1.06E-01	4.38E-02	2.86E-01
6203	GSM139441	GSE6008	Ovarian Cancer	Ovarian	2.42E-04	-2.31E-01	2.08E-01	-2.03E-01
6204	GSM139442	GSE6008	Ovarian Cancer	Ovarian	3.54E-01	8.16E-02	3.25E-02	2.99E-01
6205	GSM139443	GSE6008	Ovarian Cancer	Ovarian	4.64E-02	1.40E-01	7.55E-02	2.60E-01
6206	GSM139444	GSE6008	Ovarian Cancer	Ovarian	6.24E-06	-2.77E-01	4.29E-01	-1.49E-01
6207	GSM139445	GSE6008	Ovarian Cancer	Ovarian	3.54E-06	2.84E-01	3.80E-06	5.71E-01
6208	GSM139446	GSE6008	Ovarian Cancer	Ovarian	9.36E-02	1.23E-01	2.81E-02	3.05E-01
6209	GSM139447	GSE6008	Ovarian Cancer	Ovarian	7.85E-03	-1.76E-01	2.08E-01	-2.03E-01
6210	GSM139448	GSE6008	Ovarian Cancer	Ovarian	5.26E-04	-2.20E-01	2.25E-01	-1.97E-01
6211	GSM139449	GSE6008	Ovarian Cancer	Ovarian	2.70E-01	9.16E-02	3.61E-01	1.63E-01

6212	GSM139450	GSE6008	Ovarian Cancer	Ovarian	2.89E-02	-1.51E-01	4.20E-01	1.50E-01
6213	GSM139451	GSE6008	Ovarian Cancer	Ovarian	5.33E-02	1.37E-01	7.27E-02	2.62E-01
6214	GSM139452	GSE6008	Ovarian Cancer	Ovarian	4.55E-02	-1.41E-01	5.03E-01	1.34E-01
6215	GSM139453	GSE6008	Ovarian Cancer	Ovarian	1.14E-02	1.69E-01	1.77E-04	4.75E-01
6216	GSM139454	GSE6008	Ovarian Cancer	Ovarian	1.84E-02	-1.60E-01	4.08E-01	1.53E-01
6217	GSM139455	GSE6008	Ovarian Cancer	Ovarian	1.53E-05	-2.66E-01	9.10E-02	-2.50E-01
6218	GSM139456	GSE6008	Ovarian Cancer	Ovarian	9.06E-02	1.24E-01	1.37E-01	2.28E-01
6219	GSM139457	GSE6008	Ovarian Cancer	Ovarian	5.88E-03	1.81E-01	1.02E-02	3.46E-01
6220	GSM139458	GSE6008	Ovarian Cancer	Ovarian	5.73E-03	1.82E-01	1.85E-04	4.74E-01
6221	GSM139459	GSE6008	Ovarian Cancer	Ovarian	4.42E-10	3.71E-01	1.40E-07	6.42E-01
6222	GSM139460	GSE6008	Ovarian Cancer	Ovarian	2.98E-02	-1.50E-01	2.02E-01	2.04E-01
6223	GSM139461	GSE6008	Ovarian Cancer	Ovarian	1.91E-01	1.03E-01	1.73E-01	2.14E-01
6224	GSM139462	GSE6008	Ovarian Cancer	Ovarian	1.18E-02	1.69E-01	1.94E-03	4.04E-01
6225	GSM139463	GSE6008	Ovarian Cancer	Ovarian	2.53E-04	2.30E-01	1.20E-03	4.19E-01
6226	GSM139464	GSE6008	Ovarian Cancer	Ovarian	2.97E-06	-2.85E-01	3.86E-02	-2.91E-01
6227	GSM139465	GSE6008	Ovarian Cancer	Ovarian	9.44E-02	1.23E-01	5.45E-02	2.76E-01
6228	GSM139466	GSE6008	Ovarian Cancer	Ovarian	2.45E-02	1.54E-01	2.68E-02	3.07E-01
6229	GSM139467	GSE6008	Ovarian Cancer	Ovarian	2.12E-05	-2.63E-01	3.61E-01	-1.63E-01
6230	GSM139468	GSE6008	Ovarian Cancer	Ovarian	3.59E-01	8.10E-02	1.19E-01	2.36E-01
6231	GSM139469	GSE6008	Ovarian Cancer	Ovarian	2.87E-01	8.94E-02	2.61E-01	1.87E-01
6232	GSM139470	GSE6008	Ovarian Cancer	Ovarian	3.52E-05	-2.56E-01	5.49E-01	1.25E-01
6233	GSM139471	GSE6008	Ovarian Cancer	Ovarian	1.78E-02	1.61E-01	4.15E-03	3.78E-01
6234	GSM139472	GSE6008	Ovarian Cancer	Ovarian	1.01E-01	1.21E-01	3.90E-01	1.57E-01
6235	GSM139473	GSE6008	Ovarian Cancer	Ovarian	3.24E-04	-2.27E-01	2.58E-01	-1.88E-01
6236	GSM139474	GSE6008	Ovarian Cancer	Ovarian	3.05E-04	2.28E-01	8.98E-04	4.28E-01
6237	GSM139475	GSE6008	Ovarian Cancer	Ovarian	1.57E-01	-1.09E-01	1.41E-01	2.26E-01
6238	1A	EMEX935	Ovarian Cancer	Ovarian	1.83E-01	-1.04E-01	5.83E-01	1.19E-01
6239	1B	EMEX935	Ovarian Cancer	Ovarian	1.69E-07	-3.16E-01	2.34E-02	-3.13E-01
6240	1C	EMEX935	Ovarian Cancer	Ovarian	4.11E-05	-2.54E-01	1.77E-01	-2.13E-01
6241	2A	EMEX935	Ovarian Cancer	Ovarian	2.01E-02	-1.58E-01	3.99E-01	-1.55E-01
6242	2B	EMEX935	Ovarian Cancer	Ovarian	3.98E-02	-1.44E-01	3.46E-01	1.66E-01
6243	2C	EMEX935	Ovarian Cancer	Ovarian	9.06E-02	1.24E-01	3.13E-03	3.88E-01
6244	3A	EMEX935	Ovarian Cancer	Ovarian	9.15E-04	-2.12E-01	2.06E-01	-2.03E-01
6245	3B	EMEX935	Ovarian Cancer	Ovarian	1.16E-01	-1.18E-01	1.61E-01	2.18E-01
6246	3C	EMEX935	Ovarian Cancer	Ovarian	2.33E-02	-1.55E-01	4.42E-01	1.46E-01
6247	4A	EMEX935	Ovarian Cancer	Ovarian	7.24E-01	4.55E-02	4.42E-01	1.46E-01
6248	4B	EMEX935	Ovarian Cancer	Ovarian	5.96E-01	5.76E-02	4.29E-01	1.49E-01
6249	4C	EMEX935	Ovarian Cancer	Ovarian	1.99E-02	-1.58E-01	3.14E-01	-1.74E-01
6250	5A	EMEX935	Ovarian Cancer	Ovarian	9.54E-05	-2.44E-01	4.02E-01	-1.54E-01
6251	5B	EMEX935	Ovarian Cancer	Ovarian	4.72E-05	-2.53E-01	3.61E-01	-1.63E-01
6252	5C	EMEX935	Ovarian Cancer	Ovarian	2.33E-07	-3.13E-01	1.59E-01	-2.19E-01
6253	6A	EMEX935	Ovarian Cancer	Ovarian	2.49E-01	9.44E-02	2.29E-01	1.96E-01
6254	6B	EMEX935	Ovarian Cancer	Ovarian	4.13E-02	1.43E-01	7.75E-03	3.56E-01
6255	6C	EMEX935	Ovarian Cancer	Ovarian	2.29E-01	9.72E-02	7.74E-02	2.58E-01
6256	7A	EMEX935	Ovarian Cancer	Ovarian	2.10E-04	-2.33E-01	3.22E-01	-1.72E-01
6257	7B	EMEX935	Ovarian Cancer	Ovarian	4.86E-02	1.39E-01	5.24E-02	2.77E-01
6258	7C	EMEX935	Ovarian Cancer	Ovarian	3.23E-05	-2.57E-01	2.06E-01	-2.03E-01
6259	8A	EMEX935	Ovarian Cancer	Ovarian	2.57E-01	9.33E-02	1.20E-02	3.40E-01
6260	8B	EMEX935	Ovarian Cancer	Ovarian	2.84E-01	-8.98E-02	1.20E-02	3.40E-01
6261	8C	EMEX935	Ovarian Cancer	Ovarian	1.61E-01	1.08E-01	1.98E-03	4.03E-01
6262	9A	EMEX935	Ovarian Cancer	Ovarian	3.38E-01	8.33E-02	5.07E-03	3.71E-01
6263	9B	EMEX935	Ovarian Cancer	Ovarian	5.24E-02	1.37E-01	3.19E-04	4.58E-01
6264	9C	EMEX935	Ovarian Cancer	Ovarian	3.97E-05	2.55E-01	7.36E-05	4.98E-01
6265	GSM277800	GSE10971	Ovarian Cancer	Ovarian	6.40E-02	-1.33E-01	2.02E-01	2.04E-01
6266	GSM277801	GSE10971	Ovarian Cancer	Ovarian	2.40E-19	5.24E-01	5.91E-10	7.45E-01
6267	GSM277802	GSE10971	Ovarian Cancer	Ovarian	3.70E-03	-1.89E-01	2.54E-01	1.89E-01
6268	GSM277803	GSE10971	Ovarian Cancer	Ovarian	4.28E-06	-2.81E-01	3.11E-01	-1.75E-01
6269	GSM277804	GSE10971	Ovarian Cancer	Ovarian	2.94E-01	-8.85E-02	2.10E-01	2.02E-01
6270	GSM277805	GSE10971	Ovarian Cancer	Ovarian	1.03E-07	3.21E-01	5.43E-06	5.63E-01
6271	GSM277806	GSE10971	Ovarian Cancer	Ovarian	1.01E-03	-2.10E-01	1.63E-01	-2.18E-01
6272	GSM277807	GSE10971	Ovarian Cancer	Ovarian	5.90E-02	-1.35E-01	2.83E-01	1.82E-01
6273	GSM277808	GSE10971	Ovarian Cancer	Ovarian	6.87E-09	-3.47E-01	2.54E-03	-3.95E-01
6274	GSM277809	GSE10971	Ovarian Cancer	Ovarian	5.18E-09	-3.50E-01	4.09E-02	-2.89E-01
6275	GSM277810	GSE10971	Ovarian Cancer	Ovarian	1.76E-04	-2.35E-01	5.49E-01	1.25E-01
6276	GSM277811	GSE10971	Ovarian Cancer	Ovarian	1.50E-04	-2.37E-01	2.00E-01	-2.05E-01
6277	GSM277812	GSE10971	Ovarian Cancer	Ovarian	2.21E-01	9.83E-02	1.38E-01	2.27E-01
6278	GSM350579	GSE14001	Ovarian Cancer	Ovarian	3.17E-11	-3.94E-01	7.75E-03	-3.56E-01
6279	GSM350582	GSE14001	Ovarian Cancer	Ovarian	2.40E-05	-2.61E-01	1.06E-01	-2.42E-01
6280	GSM350584	GSE14001	Ovarian Cancer	Ovarian	3.12E-01	8.64E-02	8.99E-02	2.51E-01
6281	GSM350586	GSE14001	Ovarian Cancer	Ovarian	2.80E-02	-1.51E-01	4.80E-01	-1.38E-01
6282	GSM350588	GSE14001	Ovarian Cancer	Ovarian	3.43E-11	-3.93E-01	2.49E-03	-3.96E-01
6283	GSM350600	GSE14001	Ovarian Cancer	Ovarian	7.06E-02	-1.30E-01	3.81E-01	1.59E-01
6284	GSM350603	GSE14001	Ovarian Cancer	Ovarian	9.21E-02	-1.24E-01	5.63E-01	1.23E-01
6285	GSM350605	GSE14001	Ovarian Cancer	Ovarian	2.07E-01	1.00E-01	1.20E-02	3.40E-01

6286	GSM350606	GSE14001	Ovarian Cancer	Ovarian	1.71E-05	-2.65E-01	1.40E-01	-2.27E-01
6287	GSM350607	GSE14001	Ovarian Cancer	Ovarian	4.44E-06	2.81E-01	7.74E-06	5.54E-01
6288	GSM350609	GSE14001	Ovarian Cancer	Ovarian	3.86E-02	-1.44E-01	5.13E-01	1.32E-01
6289	GSM350610	GSE14001	Ovarian Cancer	Ovarian	4.40E-05	-2.53E-01	1.51E-01	-2.22E-01
6290	GSM350612	GSE14001	Ovarian Cancer	Ovarian	1.78E-01	1.05E-01	1.79E-01	2.12E-01
6291	GSM350613	GSE14001	Ovarian Cancer	Ovarian	2.51E-01	-9.42E-02	1.83E-02	3.23E-01
6292	GSM350614	GSE14001	Ovarian Cancer	Ovarian	4.64E-03	-1.85E-01	3.14E-01	1.74E-01
6293	GSM350615	GSE14001	Ovarian Cancer	Ovarian	9.39E-06	-2.72E-01	2.04E-01	-2.04E-01
6294	GSM350616	GSE14001	Ovarian Cancer	Ovarian	1.31E-01	-1.14E-01	4.64E-01	1.42E-01
6295	GSM350617	GSE14001	Ovarian Cancer	Ovarian	5.96E-02	1.34E-01	1.73E-01	2.14E-01
6296	GSM350618	GSE14001	Ovarian Cancer	Ovarian	5.09E-02	1.38E-01	1.21E-01	2.35E-01
6297	GSM350619	GSE14001	Ovarian Cancer	Ovarian	3.40E-06	-2.84E-01	5.53E-02	-2.75E-01
6298	GSM359984	GSE14407	Ovarian Cancer	Ovarian	5.38E-02	-1.37E-01	1.04E-01	2.43E-01
6299	GSM360039	GSE14407	Ovarian Cancer	Ovarian	4.01E-02	-1.44E-01	2.22E-01	1.98E-01
6300	GSM360040	GSE14407	Ovarian Cancer	Ovarian	1.72E-03	-2.02E-01	6.00E-01	1.16E-01
6301	GSM360041	GSE14407	Ovarian Cancer	Ovarian	1.19E-02	-1.68E-01	1.98E-01	2.06E-01
6302	GSM360042	GSE14407	Ovarian Cancer	Ovarian	8.83E-02	1.25E-01	5.24E-02	2.77E-01
6303	GSM360043	GSE14407	Ovarian Cancer	Ovarian	2.06E-01	-1.01E-01	6.56E-02	2.67E-01
6304	GSM360044	GSE14407	Ovarian Cancer	Ovarian	3.05E-04	-2.28E-01	3.61E-01	-1.63E-01
6305	GSM360045	GSE14407	Ovarian Cancer	Ovarian	1.86E-03	-2.01E-01	1.98E-01	2.06E-01
6306	GSM360046	GSE14407	Ovarian Cancer	Ovarian	3.03E-01	8.74E-02	9.68E-03	3.48E-01
6307	GSM360047	GSE14407	Ovarian Cancer	Ovarian	1.18E-01	-1.17E-01	3.46E-01	1.66E-01
6308	GSM360048	GSE14407	Ovarian Cancer	Ovarian	4.53E-04	-2.22E-01	2.58E-01	-1.88E-01
6309	GSM360049	GSE14407	Ovarian Cancer	Ovarian	2.51E-01	9.42E-02	3.25E-03	3.87E-01
6310	GSM368661	GSE14764	Ovarian Cancer	Ovarian	1.21E-02	-1.68E-01	2.22E-01	1.98E-01
6311	GSM368662	GSE14764	Ovarian Cancer	Ovarian	1.03E-01	-1.21E-01	2.17E-02	3.16E-01
6312	GSM368663	GSE14764	Ovarian Cancer	Ovarian	1.42E-03	2.05E-01	5.74E-06	5.61E-01
6313	GSM368664	GSE14764	Ovarian Cancer	Ovarian	1.18E-04	-2.41E-01	4.63E-02	-2.83E-01
6314	GSM368665	GSE14764	Ovarian Cancer	Ovarian	2.66E-02	-1.52E-01	1.34E-01	2.29E-01
6315	GSM368666	GSE14764	Ovarian Cancer	Ovarian	4.21E-01	7.45E-02	5.68E-02	2.74E-01
6316	GSM368667	GSE14764	Ovarian Cancer	Ovarian	7.14E-03	1.78E-01	1.90E-03	4.04E-01
6317	GSM368668	GSE14764	Ovarian Cancer	Ovarian	1.63E-02	1.62E-01	4.89E-03	3.73E-01
6318	GSM368669	GSE14764	Ovarian Cancer	Ovarian	8.09E-09	-3.45E-01	7.55E-02	-2.60E-01
6319	GSM368670	GSE14764	Ovarian Cancer	Ovarian	1.44E-01	1.11E-01	1.46E-02	3.32E-01
6320	GSM368671	GSE14764	Ovarian Cancer	Ovarian	1.60E-02	-1.63E-01	1.98E-01	2.06E-01
6321	GSM368672	GSE14764	Ovarian Cancer	Ovarian	9.13E-02	1.24E-01	1.32E-01	2.30E-01
6322	GSM368673	GSE14764	Ovarian Cancer	Ovarian	2.76E-18	5.09E-01	8.49E-09	6.97E-01
6323	GSM368674	GSE14764	Ovarian Cancer	Ovarian	1.90E-02	1.59E-01	2.87E-05	5.23E-01
6324	GSM368675	GSE14764	Ovarian Cancer	Ovarian	4.47E-01	7.19E-02	8.99E-02	2.51E-01
6325	GSM368676	GSE14764	Ovarian Cancer	Ovarian	1.39E-06	-2.94E-01	2.58E-01	-1.88E-01
6326	GSM368677	GSE14764	Ovarian Cancer	Ovarian	1.56E-01	1.09E-01	1.41E-01	2.26E-01
6327	GSM368678	GSE14764	Ovarian Cancer	Ovarian	3.01E-02	1.50E-01	1.17E-03	4.20E-01
6328	GSM368679	GSE14764	Ovarian Cancer	Ovarian	2.97E-06	-2.85E-01	2.51E-01	-1.90E-01
6329	GSM368680	GSE14764	Ovarian Cancer	Ovarian	8.25E-06	-2.74E-01	8.77E-02	-2.52E-01
6330	GSM368681	GSE14764	Ovarian Cancer	Ovarian	3.27E-08	-3.32E-01	2.68E-02	-3.07E-01
6331	GSM368682	GSE14764	Ovarian Cancer	Ovarian	1.69E-01	1.07E-01	6.17E-03	3.64E-01
6332	GSM368683	GSE14764	Ovarian Cancer	Ovarian	1.58E-03	2.03E-01	8.02E-03	3.55E-01
6333	GSM368684	GSE14764	Ovarian Cancer	Ovarian	5.03E-14	-4.43E-01	1.31E-04	-4.83E-01
6334	GSM368685	GSE14764	Ovarian Cancer	Ovarian	5.31E-05	-2.51E-01	2.29E-01	-1.96E-01
6335	GSM368686	GSE14764	Ovarian Cancer	Ovarian	1.71E-01	-1.06E-01	4.45E-01	-1.45E-01
6336	GSM368687	GSE14764	Ovarian Cancer	Ovarian	1.65E-23	5.80E-01	1.03E-12	8.49E-01
6337	GSM368688	GSE14764	Ovarian Cancer	Ovarian	5.45E-16	-4.74E-01	1.58E-05	-5.37E-01
6338	GSM368689	GSE14764	Ovarian Cancer	Ovarian	2.95E-10	-3.75E-01	5.16E-03	-3.71E-01
6339	GSM368690	GSE14764	Ovarian Cancer	Ovarian	4.86E-01	-6.80E-02	9.85E-03	3.47E-01
6340	GSM368691	GSE14764	Ovarian Cancer	Ovarian	4.40E-05	-2.53E-01	1.51E-01	-2.22E-01
6341	GSM368692	GSE14764	Ovarian Cancer	Ovarian	2.08E-03	-1.99E-01	5.53E-01	-1.24E-01
6342	GSM368693	GSE14764	Ovarian Cancer	Ovarian	6.85E-07	3.02E-01	4.18E-05	5.13E-01
6343	GSM368694	GSE14764	Ovarian Cancer	Ovarian	4.47E-03	1.86E-01	9.83E-05	4.91E-01
6344	GSM368695	GSE14764	Ovarian Cancer	Ovarian	1.28E-07	-3.19E-01	1.22E-01	-2.34E-01
6345	GSM368696	GSE14764	Ovarian Cancer	Ovarian	6.34E-02	-1.33E-01	1.01E-01	2.44E-01
6346	GSM368697	GSE14764	Ovarian Cancer	Ovarian	4.97E-13	-4.26E-01	2.54E-03	-3.95E-01
6347	GSM368698	GSE14764	Ovarian Cancer	Ovarian	3.10E-02	-1.49E-01	5.89E-01	1.17E-01
6348	GSM368699	GSE14764	Ovarian Cancer	Ovarian	9.44E-02	1.23E-01	2.31E-02	3.14E-01
6349	GSM368700	GSE14764	Ovarian Cancer	Ovarian	1.03E-03	-2.10E-01	7.36E-02	-2.61E-01
6350	GSM368701	GSE14764	Ovarian Cancer	Ovarian	4.88E-04	-2.21E-01	2.63E-01	-1.87E-01
6351	GSM368702	GSE14764	Ovarian Cancer	Ovarian	6.19E-05	-2.49E-01	2.56E-01	-1.89E-01
6352	GSM368703	GSE14764	Ovarian Cancer	Ovarian	9.60E-02	-1.23E-01	2.88E-01	1.80E-01
6353	GSM368704	GSE14764	Ovarian Cancer	Ovarian	1.34E-07	3.18E-01	2.72E-06	5.78E-01
6354	GSM368705	GSE14764	Ovarian Cancer	Ovarian	4.96E-15	4.59E-01	3.68E-10	7.53E-01
6355	GSM368706	GSE14764	Ovarian Cancer	Ovarian	1.62E-02	1.63E-01	4.15E-03	3.78E-01
6356	GSM368707	GSE14764	Ovarian Cancer	Ovarian	4.00E-01	-7.66E-02	1.34E-01	2.29E-01
6357	GSM368708	GSE14764	Ovarian Cancer	Ovarian	1.26E-01	1.15E-01	2.25E-01	1.97E-01
6358	GSM368709	GSE14764	Ovarian Cancer	Ovarian	3.77E-01	-7.90E-02	2.04E-01	2.04E-01
6359	GSM368710	GSE14764	Ovarian Cancer	Ovarian	2.57E-03	-1.95E-01	4.58E-01	1.43E-01

6360	GSM368711	GSE14764	Ovarian Cancer	Ovarian	2.18E-08	-3.36E-01	3.97E-02	-2.90E-01
6361	GSM368712	GSE14764	Ovarian Cancer	Ovarian	1.64E-09	-3.60E-01	2.20E-02	-3.16E-01
6362	GSM368713	GSE14764	Ovarian Cancer	Ovarian	9.49E-03	-1.73E-01	3.84E-01	1.58E-01
6363	GSM368714	GSE14764	Ovarian Cancer	Ovarian	6.84E-05	-2.48E-01	1.04E-01	-2.43E-01
6364	GSM368715	GSE14764	Ovarian Cancer	Ovarian	1.16E-01	-1.18E-01	1.77E-01	2.13E-01
6365	GSM368716	GSE14764	Ovarian Cancer	Ovarian	4.28E-06	-2.81E-01	2.36E-01	-1.94E-01
6366	GSM368717	GSE14764	Ovarian Cancer	Ovarian	5.05E-02	1.38E-01	6.39E-03	3.63E-01
6367	GSM368718	GSE14764	Ovarian Cancer	Ovarian	2.25E-18	5.10E-01	2.14E-09	7.22E-01
6368	GSM368719	GSE14764	Ovarian Cancer	Ovarian	3.54E-06	2.84E-01	4.03E-08	6.67E-01
6369	GSM368720	GSE14764	Ovarian Cancer	Ovarian	2.66E-02	-1.52E-01	5.03E-01	-1.34E-01
6370	GSM368721	GSE14764	Ovarian Cancer	Ovarian	7.60E-08	-3.24E-01	2.77E-02	-3.06E-01
6371	GSM368722	GSE14764	Ovarian Cancer	Ovarian	1.71E-04	-2.36E-01	1.82E-01	-2.11E-01
6372	GSM368723	GSE14764	Ovarian Cancer	Ovarian	8.39E-02	-1.26E-01	2.85E-01	1.81E-01
6373	GSM368724	GSE14764	Ovarian Cancer	Ovarian	6.33E-01	-5.41E-02	5.45E-02	2.76E-01
6374	GSM368725	GSE14764	Ovarian Cancer	Ovarian	2.41E-09	-3.56E-01	3.13E-03	-3.88E-01
6375	GSM368726	GSE14764	Ovarian Cancer	Ovarian	3.96E-06	-2.82E-01	1.41E-01	-2.26E-01
6376	GSM368727	GSE14764	Ovarian Cancer	Ovarian	7.64E-02	-1.28E-01	3.49E-01	1.66E-01
6377	GSM368728	GSE14764	Ovarian Cancer	Ovarian	8.08E-05	-2.46E-01	4.70E-02	-2.83E-01
6378	GSM368729	GSE14764	Ovarian Cancer	Ovarian	2.39E-08	3.35E-01	4.17E-08	6.66E-01
6379	GSM368730	GSE14764	Ovarian Cancer	Ovarian	2.72E-01	-9.13E-02	2.29E-01	1.96E-01
6380	GSM368731	GSE14764	Ovarian Cancer	Ovarian	4.25E-01	7.40E-02	4.63E-02	2.83E-01
6381	GSM368732	GSE14764	Ovarian Cancer	Ovarian	4.94E-03	1.84E-01	9.17E-04	4.27E-01
6382	GSM368733	GSE14764	Ovarian Cancer	Ovarian	2.70E-07	-3.11E-01	2.64E-02	-3.08E-01
6383	GSM368734	GSE14764	Ovarian Cancer	Ovarian	4.18E-13	-4.27E-01	4.22E-03	-3.78E-01
6384	GSM368735	GSE14764	Ovarian Cancer	Ovarian	6.46E-11	-3.88E-01	9.68E-03	-3.48E-01
6385	GSM368736	GSE14764	Ovarian Cancer	Ovarian	3.74E-03	1.89E-01	2.01E-03	4.03E-01
6386	GSM368737	GSE14764	Ovarian Cancer	Ovarian	1.85E-04	-2.35E-01	2.90E-01	-1.80E-01
6387	GSM368738	GSE14764	Ovarian Cancer	Ovarian	2.34E-03	-1.97E-01	4.74E-01	-1.40E-01
6388	GSM368739	GSE14764	Ovarian Cancer	Ovarian	1.26E-02	-1.67E-01	1.18E-01	2.36E-01
6389	GSM368740	GSE14764	Ovarian Cancer	Ovarian	4.15E-03	1.87E-01	3.26E-04	4.58E-01
6390	GSM461348	GSE18520	Ovarian Cancer	Ovarian	1.21E-03	-2.07E-01	4.42E-01	1.46E-01
6391	GSM461349	GSE18520	Ovarian Cancer	Ovarian	1.60E-04	-2.37E-01	2.04E-01	-2.04E-01
6392	GSM461350	GSE18520	Ovarian Cancer	Ovarian	4.72E-02	-1.40E-01	4.90E-01	1.37E-01
6393	GSM461351	GSE18520	Ovarian Cancer	Ovarian	2.33E-02	-1.55E-01	3.30E-01	-1.70E-01
6394	GSM461352	GSE18520	Ovarian Cancer	Ovarian	2.06E-06	-2.90E-01	3.96E-01	-1.56E-01
6395	GSM461353	GSE18520	Ovarian Cancer	Ovarian	1.62E-01	1.08E-01	3.20E-02	3.00E-01
6396	GSM461354	GSE18520	Ovarian Cancer	Ovarian	4.51E-01	-7.14E-02	8.66E-02	2.53E-01
6397	GSM461355	GSE18520	Ovarian Cancer	Ovarian	2.19E-03	-1.98E-01	4.70E-01	1.40E-01
6398	GSM461356	GSE18520	Ovarian Cancer	Ovarian	7.94E-08	-3.24E-01	1.35E-01	-2.29E-01
6399	GSM461357	GSE18520	Ovarian Cancer	Ovarian	1.00E-01	1.21E-01	1.77E-02	3.24E-01
6400	GSM461358	GSE18520	Ovarian Cancer	Ovarian	2.98E-02	-1.50E-01	6.77E-01	1.01E-01
6401	GSM461359	GSE18520	Ovarian Cancer	Ovarian	1.65E-05	-2.66E-01	4.36E-01	-1.47E-01
6402	GSM461360	GSE18520	Ovarian Cancer	Ovarian	4.18E-07	-3.07E-01	1.54E-02	-3.30E-01
6403	GSM461361	GSE18520	Ovarian Cancer	Ovarian	6.01E-06	-2.77E-01	2.00E-01	-2.05E-01
6404	GSM461362	GSE18520	Ovarian Cancer	Ovarian	1.58E-03	2.03E-01	3.69E-06	5.71E-01
6405	GSM461363	GSE18520	Ovarian Cancer	Ovarian	3.98E-02	-1.44E-01	3.11E-01	-1.75E-01
6406	GSM461364	GSE18520	Ovarian Cancer	Ovarian	3.32E-01	8.40E-02	1.32E-01	2.30E-01
6407	GSM461365	GSE18520	Ovarian Cancer	Ovarian	6.63E-02	1.32E-01	2.57E-02	3.09E-01
6408	GSM461366	GSE18520	Ovarian Cancer	Ovarian	4.38E-08	-3.29E-01	1.24E-01	-2.34E-01
6409	GSM461367	GSE18520	Ovarian Cancer	Ovarian	7.06E-04	-2.16E-01	6.43E-01	-1.07E-01
6410	GSM461368	GSE18520	Ovarian Cancer	Ovarian	5.19E-02	-1.38E-01	4.51E-02	2.84E-01
6411	GSM461369	GSE18520	Ovarian Cancer	Ovarian	4.95E-02	1.39E-01	1.20E-03	4.19E-01
6412	GSM461370	GSE18520	Ovarian Cancer	Ovarian	2.10E-01	1.00E-01	3.30E-02	2.98E-01
6413	GSM461371	GSE18520	Ovarian Cancer	Ovarian	3.75E-01	7.92E-02	3.63E-01	1.63E-01
6414	GSM461372	GSE18520	Ovarian Cancer	Ovarian	3.83E-01	-7.84E-02	1.32E-01	2.30E-01
6415	GSM461373	GSE18520	Ovarian Cancer	Ovarian	6.01E-04	-2.18E-01	2.22E-01	1.98E-01
6416	GSM461374	GSE18520	Ovarian Cancer	Ovarian	8.25E-02	-1.26E-01	1.35E-01	2.29E-01
6417	GSM461375	GSE18520	Ovarian Cancer	Ovarian	2.01E-05	-2.63E-01	3.22E-01	-1.72E-01
6418	GSM461376	GSE18520	Ovarian Cancer	Ovarian	2.55E-06	-2.87E-01	2.25E-01	-1.97E-01
6419	GSM461377	GSE18520	Ovarian Cancer	Ovarian	1.47E-12	-4.18E-01	5.16E-03	-3.71E-01
6420	GSM461378	GSE18520	Ovarian Cancer	Ovarian	2.64E-01	9.24E-02	1.18E-02	3.40E-01
6421	GSM461379	GSE18520	Ovarian Cancer	Ovarian	2.91E-01	-8.90E-02	2.90E-01	1.80E-01
6422	GSM461380	GSE18520	Ovarian Cancer	Ovarian	2.64E-02	-1.53E-01	4.20E-01	1.50E-01
6423	GSM461381	GSE18520	Ovarian Cancer	Ovarian	1.63E-02	-1.62E-01	2.04E-01	2.04E-01
6424	GSM461382	GSE18520	Ovarian Cancer	Ovarian	4.38E-02	-1.42E-01	1.06E-01	2.42E-01
6425	GSM461383	GSE18520	Ovarian Cancer	Ovarian	1.94E-05	2.64E-01	1.41E-06	5.93E-01
6426	GSM461384	GSE18520	Ovarian Cancer	Ovarian	1.17E-01	1.17E-01	1.26E-02	3.38E-01
6427	GSM461385	GSE18520	Ovarian Cancer	Ovarian	9.33E-13	4.21E-01	9.28E-08	6.50E-01
6428	GSM461386	GSE18520	Ovarian Cancer	Ovarian	4.64E-02	-1.40E-01	4.39E-01	1.47E-01
6429	GSM461387	GSE18520	Ovarian Cancer	Ovarian	2.75E-06	-2.86E-01	1.79E-01	-2.12E-01
6430	GSM461388	GSE18520	Ovarian Cancer	Ovarian	2.13E-03	1.98E-01	3.05E-04	4.60E-01
6431	GSM461389	GSE18520	Ovarian Cancer	Ovarian	3.70E-03	1.89E-01	3.12E-04	4.59E-01
6432	GSM461390	GSE18520	Ovarian Cancer	Ovarian	1.39E-04	-2.39E-01	5.63E-01	-1.23E-01
6433	GSM461391	GSE18520	Ovarian Cancer	Ovarian	4.21E-02	-1.42E-01	3.46E-01	1.66E-01

6434	GSM461392	GSE18520	Ovarian Cancer	Ovarian	5.64E-02	-1.36E-01	4.61E-01	-1.42E-01
6435	GSM461393	GSE18520	Ovarian Cancer	Ovarian	1.42E-01	1.12E-01	1.75E-02	3.25E-01
6436	GSM461394	GSE18520	Ovarian Cancer	Ovarian	5.33E-02	-1.37E-01	6.70E-01	1.02E-01
6437	GSM461395	GSE18520	Ovarian Cancer	Ovarian	2.62E-01	-9.26E-02	2.22E-01	1.98E-01
6438	GSM461396	GSE18520	Ovarian Cancer	Ovarian	2.04E-01	1.01E-01	1.17E-01	2.37E-01
6439	GSM461397	GSE18520	Ovarian Cancer	Ovarian	5.58E-07	-3.04E-01	5.53E-02	-2.75E-01
6440	GSM461398	GSE18520	Ovarian Cancer	Ovarian	1.19E-13	-4.37E-01	7.44E-04	-4.34E-01
6441	GSM461399	GSE18520	Ovarian Cancer	Ovarian	1.64E-01	1.08E-01	3.08E-03	3.89E-01
6442	GSM461400	GSE18520	Ovarian Cancer	Ovarian	3.47E-07	3.09E-01	3.78E-05	5.16E-01
6443	GSM480502_R473_	GSE19352	Ovarian Cancer	Ovarian	8.82E-13	-4.22E-01	1.51E-02	-3.31E-01
6444	GSM480503_R473_	GSE19352	Ovarian Cancer	Ovarian	4.26E-04	-2.23E-01	4.23E-01	-1.50E-01
6445	GSM480504_R473_	GSE19352	Ovarian Cancer	Ovarian	2.88E-10	-3.75E-01	5.68E-02	-2.74E-01
6446	GSM480505_R473_	GSE19352	Ovarian Cancer	Ovarian	1.28E-03	-2.06E-01	6.27E-01	1.10E-01
6447	GSM480506_R473_	GSE19352	Ovarian Cancer	Ovarian	1.03E-05	-2.71E-01	7.65E-02	-2.59E-01
6448	GSM480507_R473_	GSE19352	Ovarian Cancer	Ovarian	7.50E-16	4.72E-01	8.79E-10	7.38E-01
6449	GSM480508_R473_	GSE19352	Ovarian Cancer	Ovarian	4.30E-13	-4.27E-01	1.56E-03	-4.11E-01
6450	GSM480509_R473_	GSE19352	Ovarian Cancer	Ovarian	5.33E-02	-1.37E-01	4.77E-01	1.39E-01
6451	GSM480510_R473_	GSE19352	Ovarian Cancer	Ovarian	1.99E-02	-1.58E-01	1.54E-01	-2.21E-01
6452	GSM480511_R473_	GSE19352	Ovarian Cancer	Ovarian	9.77E-18	5.01E-01	2.37E-10	7.61E-01
6453	GSM480512_R473_	GSE19352	Ovarian Cancer	Ovarian	7.00E-02	1.31E-01	2.68E-02	3.07E-01
6454	GSM480513_R473_	GSE19352	Ovarian Cancer	Ovarian	5.73E-03	1.82E-01	5.40E-04	4.43E-01
6455	GSM480514_R473_	GSE19352	Ovarian Cancer	Ovarian	1.60E-03	2.03E-01	7.54E-05	4.98E-01
6456	GSM480515_R473_	GSE19352	Ovarian Cancer	Ovarian	3.28E-05	-2.57E-01	4.05E-01	1.54E-01
6457	GSM480516_R473_	GSE19352	Ovarian Cancer	Ovarian	6.98E-06	2.76E-01	3.98E-05	5.14E-01
6458	GSM480517_R473_	GSE19352	Ovarian Cancer	Ovarian	1.50E-01	1.10E-01	1.21E-01	2.35E-01
6459	GSM480518_R473_	GSE19352	Ovarian Cancer	Ovarian	1.22E-04	-2.40E-01	4.77E-01	-1.39E-01
6460	GSM480519_R473_	GSE19352	Ovarian Cancer	Ovarian	5.79E-06	-2.78E-01	3.96E-01	-1.56E-01
6461	GSM480520_R473_	GSE19352	Ovarian Cancer	Ovarian	7.93E-04	-2.14E-01	2.63E-01	-1.87E-01
6462	GSM480521_R473_	GSE19352	Ovarian Cancer	Ovarian	1.25E-06	-2.95E-01	7.55E-02	-2.60E-01
6463	E1085_1145	E-MEX1085	Ovarian Cancer	Ovarian	1.76E-11	-3.98E-01	4.70E-02	-2.83E-01
6464	E1085_1323	E-MEX1085	Ovarian Cancer	Ovarian	1.01E-03	-2.10E-01	3.87E-01	-1.57E-01
6465	E1085_1348	E-MEX1085	Ovarian Cancer	Ovarian	5.66E-03	-1.82E-01	5.86E-01	-1.18E-01
6466	E1085_1398	E-MEX1085	Ovarian Cancer	Ovarian	4.88E-04	2.21E-01	1.85E-04	4.74E-01
6467	E1085_1484	E-MEX1085	Ovarian Cancer	Ovarian	8.61E-02	-1.25E-01	6.33E-01	-1.09E-01
6468	E1085_1489	E-MEX1085	Ovarian Cancer	Ovarian	6.75E-02	1.31E-01	1.44E-02	3.33E-01
6469	E1085_1498	E-MEX1085	Ovarian Cancer	Ovarian	4.42E-10	-3.71E-01	8.02E-03	-3.55E-01
6470	E1085_1583	E-MEX1085	Ovarian Cancer	Ovarian	2.91E-01	-8.90E-02	5.24E-02	2.77E-01
6471	E1085_1586	E-MEX1085	Ovarian Cancer	Ovarian	2.50E-02	1.54E-01	3.08E-03	3.89E-01
6472	E1085_1588	E-MEX1085	Ovarian Cancer	Ovarian	1.20E-11	4.01E-01	1.93E-06	5.86E-01
6473	E1085_1594	E-MEX1085	Ovarian Cancer	Ovarian	5.71E-08	-3.27E-01	3.35E-02	-2.98E-01
6474	E1085_1706	E-MEX1085	Ovarian Cancer	Ovarian	2.81E-05	-2.59E-01	2.88E-01	-1.80E-01
6475	E1085_1726	E-MEX1085	Ovarian Cancer	Ovarian	1.43E-01	-1.12E-01	2.08E-01	2.03E-01
6476	E1085_1744	E-MEX1085	Ovarian Cancer	Ovarian	1.10E-02	1.70E-01	4.07E-04	4.51E-01
6477	E1085_1886	E-MEX1085	Ovarian Cancer	Ovarian	1.09E-03	-2.09E-01	4.23E-01	-1.50E-01
6478	E1085_1972	E-MEX1085	Ovarian Cancer	Ovarian	4.33E-05	-2.54E-01	2.29E-01	-1.96E-01
6479	E1085_1996	E-MEX1085	Ovarian Cancer	Ovarian	5.33E-03	-1.83E-01	1.77E-01	-2.13E-01
6480	E1085_32	E-MEX1085	Ovarian Cancer	Ovarian	4.08E-04	-2.24E-01	7.36E-01	-8.95E-02
6481	E1085_3532	E-MEX1085	Ovarian Cancer	Ovarian	1.31E-06	-2.95E-01	2.77E-02	-3.06E-01
6482	E1085_3719	E-MEX1085	Ovarian Cancer	Ovarian	8.54E-02	-1.26E-01	7.36E-02	2.61E-01
6483	E1085_3730	E-MEX1085	Ovarian Cancer	Ovarian	2.04E-05	-2.63E-01	2.06E-01	-2.03E-01
6484	E1085_3745	E-MEX1085	Ovarian Cancer	Ovarian	4.42E-03	-1.86E-01	3.61E-01	1.63E-01
6485	E1085_57824	E-MEX1085	Ovarian Cancer	Ovarian	2.95E-02	-1.50E-01	3.16E-01	1.73E-01
6486	E1085_57846	E-MEX1085	Ovarian Cancer	Ovarian	4.01E-04	2.24E-01	2.59E-03	3.94E-01
6487	E1085_57852	E-MEX1085	Ovarian Cancer	Ovarian	2.36E-09	3.57E-01	4.47E-07	6.18E-01
6488	E1085_57857	E-MEX1085	Ovarian Cancer	Ovarian	6.98E-06	-2.76E-01	1.22E-01	-2.34E-01
6489	E1085_58002	E-MEX1085	Ovarian Cancer	Ovarian	1.54E-01	-1.10E-01	1.86E-01	2.10E-01
6490	E1085_58090	E-MEX1085	Ovarian Cancer	Ovarian	1.06E-15	4.70E-01	1.35E-09	7.30E-01
6491	E1085_58159	E-MEX1085	Ovarian Cancer	Ovarian	1.94E-05	-2.64E-01	6.23E-02	-2.69E-01
6492	E1085_58193	E-MEX1085	Ovarian Cancer	Ovarian	1.74E-08	-3.38E-01	2.68E-02	-3.07E-01
6493	E1085_58218	E-MEX1085	Ovarian Cancer	Ovarian	3.83E-08	-3.31E-01	1.63E-01	-2.18E-01
6494	E1085_58253	E-MEX1085	Ovarian Cancer	Ovarian	2.74E-03	1.94E-01	9.17E-04	4.27E-01
6495	E1085_58256	E-MEX1085	Ovarian Cancer	Ovarian	9.02E-04	-2.12E-01	2.58E-01	-1.88E-01
6496	E1085_58297	E-MEX1085	Ovarian Cancer	Ovarian	3.01E-15	-4.63E-01	4.25E-04	-4.50E-01
6497	E1085_58318	E-MEX1085	Ovarian Cancer	Ovarian	8.56E-06	-2.73E-01	8.88E-02	-2.51E-01
6498	E1085_58419	E-MEX1085	Ovarian Cancer	Ovarian	4.84E-11	3.90E-01	2.03E-07	6.34E-01
6499	E1085_58425	E-MEX1085	Ovarian Cancer	Ovarian	3.95E-04	2.24E-01	7.36E-05	4.98E-01
6500	E1085_58492	E-MEX1085	Ovarian Cancer	Ovarian	4.39E-04	-2.23E-01	2.61E-01	-1.87E-01
6501	E1085_58499	E-MEX1085	Ovarian Cancer	Ovarian	4.42E-01	7.23E-02	4.98E-03	3.72E-01
6502	E1085_58500	E-MEX1085	Ovarian Cancer	Ovarian	1.17E-12	-4.19E-01	1.51E-02	-3.31E-01
6503	E1085_73	E-MEX1085	Ovarian Cancer	Ovarian	4.20E-14	4.44E-01	2.79E-06	5.78E-01
6504	E1085_972	E-MEX1085	Ovarian Cancer	Ovarian	1.31E-02	-1.67E-01	4.58E-01	-1.43E-01
6505	E1085_977	E-MEX1085	Ovarian Cancer	Ovarian	3.01E-03	1.93E-01	2.01E-03	4.03E-01
6506	GSM389785	GSE15578	Ovarian Cancer	Ovarian	9.83E-04	-2.11E-01	5.56E-01	1.24E-01
6507	GSM389786	GSE15578	Ovarian Cancer	Ovarian	2.04E-01	-1.01E-01	5.33E-01	1.28E-01

6508	GSM389788	GSE15578	Ovarian Cancer	Ovarian	2.06E-06	-2.90E-01	5.39E-01	-1.27E-01
6509	GSM389793	GSE15578	Ovarian Cancer	Ovarian	4.21E-01	7.45E-02	5.49E-01	1.25E-01
6510	GSM306158	GSE12172	Ovarian Cancer	Ovarian	4.82E-03	-1.85E-01	3.55E-01	-1.64E-01
6511	GSM306159	GSE12172	Ovarian Cancer	Ovarian	3.72E-04	-2.25E-01	2.49E-01	-1.90E-01
6512	GSM306160	GSE12172	Ovarian Cancer	Ovarian	5.58E-07	3.04E-01	1.99E-06	5.85E-01
6513	GSM306161	GSE12172	Ovarian Cancer	Ovarian	4.72E-05	-2.53E-01	2.93E-01	-1.79E-01
6514	GSM306162	GSE12172	Ovarian Cancer	Ovarian	1.97E-04	-2.34E-01	5.00E-01	1.35E-01
6515	GSM306163	GSE12172	Ovarian Cancer	Ovarian	2.16E-03	-1.98E-01	5.23E-01	-1.30E-01
6516	GSM306164	GSE12172	Ovarian Cancer	Ovarian	1.16E-01	1.18E-01	1.20E-02	3.40E-01
6517	GSM306165	GSE12172	Ovarian Cancer	Ovarian	1.94E-04	-2.34E-01	5.00E-01	1.35E-01
6518	GSM306166	GSE12172	Ovarian Cancer	Ovarian	6.75E-02	1.31E-01	1.22E-02	3.39E-01
6519	GSM306167	GSE12172	Ovarian Cancer	Ovarian	1.15E-07	-3.20E-01	9.36E-04	-4.27E-01
6520	GSM306168	GSE12172	Ovarian Cancer	Ovarian	1.58E-04	-2.37E-01	2.66E-01	-1.86E-01
6521	GSM306169	GSE12172	Ovarian Cancer	Ovarian	9.97E-04	-2.10E-01	2.29E-01	-1.96E-01
6522	GSM306170	GSE12172	Ovarian Cancer	Ovarian	1.66E-07	-3.16E-01	1.41E-01	-2.26E-01
6523	GSM306171	GSE12172	Ovarian Cancer	Ovarian	1.78E-01	-1.05E-01	8.99E-02	2.51E-01
6524	GSM306172	GSE12172	Ovarian Cancer	Ovarian	2.66E-05	-2.60E-01	2.38E-01	-1.94E-01
6525	GSM306173	GSE12172	Ovarian Cancer	Ovarian	1.65E-03	-2.03E-01	7.32E-01	-9.02E-02
6526	GSM306174	GSE12172	Ovarian Cancer	Ovarian	3.66E-08	-3.31E-01	5.68E-02	-2.74E-01
6527	GSM306175	GSE12172	Ovarian Cancer	Ovarian	1.59E-06	-2.92E-01	2.12E-01	-2.01E-01
6528	GSM306176	GSE12172	Ovarian Cancer	Ovarian	1.23E-01	-1.16E-01	2.58E-01	1.88E-01
6529	GSM306177	GSE12172	Ovarian Cancer	Ovarian	1.58E-03	-2.03E-01	3.69E-01	-1.61E-01
6530	GSM306178	GSE12172	Ovarian Cancer	Ovarian	1.36E-01	1.13E-01	1.51E-02	3.31E-01
6531	GSM306179	GSE12172	Ovarian Cancer	Ovarian	2.00E-04	-2.34E-01	3.90E-01	-1.57E-01
6532	GSM306180	GSE12172	Ovarian Cancer	Ovarian	3.94E-03	1.88E-01	5.40E-04	4.43E-01
6533	GSM306181	GSE12172	Ovarian Cancer	Ovarian	2.70E-04	2.29E-01	2.45E-03	3.96E-01
6534	GSM306182	GSE12172	Ovarian Cancer	Ovarian	1.99E-03	-2.00E-01	7.13E-01	9.40E-02
6535	GSM306183	GSE12172	Ovarian Cancer	Ovarian	1.15E-01	-1.18E-01	1.54E-01	2.21E-01
6536	GSM306184	GSE12172	Ovarian Cancer	Ovarian	4.06E-01	7.60E-02	1.35E-01	2.29E-01
6537	GSM306185	GSE12172	Ovarian Cancer	Ovarian	8.64E-03	1.74E-01	2.59E-03	3.94E-01
6538	GSM306186	GSE12172	Ovarian Cancer	Ovarian	2.70E-01	9.16E-02	1.18E-01	2.36E-01
6539	GSM306187	GSE12172	Ovarian Cancer	Ovarian	2.19E-05	2.62E-01	1.46E-05	5.39E-01
6540	GSM306188	GSE12172	Ovarian Cancer	Ovarian	2.84E-01	-8.98E-02	2.33E-01	1.95E-01
6541	GSM306189	GSE12172	Ovarian Cancer	Ovarian	7.22E-03	-1.78E-01	3.49E-01	1.66E-01
6542	GSM306190	GSE12172	Ovarian Cancer	Ovarian	9.56E-06	-2.72E-01	3.66E-01	-1.62E-01
6543	GSM306191	GSE12172	Ovarian Cancer	Ovarian	1.51E-13	-4.35E-01	1.54E-02	-3.30E-01
6544	GSM306192	GSE12172	Ovarian Cancer	Ovarian	5.69E-02	-1.35E-01	2.51E-01	1.90E-01
6545	GSM306193	GSE12172	Ovarian Cancer	Ovarian	1.43E-05	-2.67E-01	1.24E-01	-2.34E-01
6546	GSM306194	GSE12172	Ovarian Cancer	Ovarian	2.24E-04	-2.32E-01	3.66E-01	-1.62E-01
6547	GSM306195	GSE12172	Ovarian Cancer	Ovarian	1.24E-12	-4.19E-01	1.98E-03	-4.03E-01
6548	GSM306196	GSE12172	Ovarian Cancer	Ovarian	1.67E-02	-1.62E-01	5.66E-01	-1.22E-01
6549	GSM306197	GSE12172	Ovarian Cancer	Ovarian	7.06E-02	1.30E-01	8.16E-03	3.54E-01
6550	GSM306198	GSE12172	Ovarian Cancer	Ovarian	2.89E-01	-8.92E-02	1.61E-01	2.18E-01
6551	GSM306199	GSE12172	Ovarian Cancer	Ovarian	1.86E-02	-1.60E-01	3.99E-01	-1.55E-01
6552	GSM306200	GSE12172	Ovarian Cancer	Ovarian	1.28E-03	2.06E-01	1.26E-02	3.38E-01
6553	GSM306201	GSE12172	Ovarian Cancer	Ovarian	9.15E-04	-2.12E-01	4.58E-01	-1.43E-01
6554	GSM306202	GSE12172	Ovarian Cancer	Ovarian	1.86E-03	2.01E-01	3.34E-04	4.57E-01
6555	GSM306203	GSE12172	Ovarian Cancer	Ovarian	2.54E-01	9.37E-02	1.37E-01	2.28E-01
6556	GSM306204	GSE12172	Ovarian Cancer	Ovarian	1.71E-04	2.36E-01	3.05E-04	4.60E-01
6557	GSM306205	GSE12172	Ovarian Cancer	Ovarian	3.54E-07	3.08E-01	5.29E-06	5.63E-01
6558	GSM306206	GSE12172	Ovarian Cancer	Ovarian	1.19E-05	2.69E-01	1.37E-04	4.82E-01
6559	GSM306207	GSE12172	Ovarian Cancer	Ovarian	3.80E-12	-4.10E-01	2.94E-05	-5.22E-01
6560	GSM306208	GSE12172	Ovarian Cancer	Ovarian	3.21E-01	8.53E-02	1.21E-01	2.35E-01
6561	GSM306209	GSE12172	Ovarian Cancer	Ovarian	3.36E-02	-1.47E-01	5.46E-01	1.26E-01
6562	GSM306210	GSE12172	Ovarian Cancer	Ovarian	6.98E-06	-2.76E-01	3.66E-01	-1.62E-01
6563	GSM306211	GSE12172	Ovarian Cancer	Ovarian	9.49E-03	1.73E-01	1.94E-03	4.04E-01
6564	GSM306212	GSE12172	Ovarian Cancer	Ovarian	2.99E-08	3.33E-01	7.33E-06	5.56E-01
6565	GSM306213	GSE12172	Ovarian Cancer	Ovarian	1.46E-07	3.18E-01	7.95E-06	5.54E-01
6566	GSM306214	GSE12172	Ovarian Cancer	Ovarian	1.65E-02	1.62E-01	1.53E-03	4.11E-01
6567	GSM306215	GSE12172	Ovarian Cancer	Ovarian	5.79E-06	-2.78E-01	1.06E-01	-2.42E-01
6568	GSM306216	GSE12172	Ovarian Cancer	Ovarian	7.85E-10	-3.66E-01	4.09E-02	-2.89E-01
6569	GSM306217	GSE12172	Ovarian Cancer	Ovarian	1.13E-03	-2.08E-01	5.23E-01	-1.30E-01
6570	GSM306218	GSE12172	Ovarian Cancer	Ovarian	6.29E-05	-2.49E-01	6.90E-01	-9.84E-02
6571	GSM306219	GSE12172	Ovarian Cancer	Ovarian	2.72E-02	-1.52E-01	3.84E-01	-1.58E-01
6572	GSM306220	GSE12172	Ovarian Cancer	Ovarian	1.18E-01	-1.17E-01	6.39E-02	2.68E-01
6573	GSM306221	GSE12172	Ovarian Cancer	Ovarian	1.82E-02	1.60E-01	4.00E-03	3.80E-01
6574	GSM306222	GSE12172	Ovarian Cancer	Ovarian	9.29E-02	1.23E-01	4.45E-02	2.85E-01
6575	GSM306223	GSE12172	Ovarian Cancer	Ovarian	1.76E-01	-1.05E-01	5.19E-01	1.31E-01
6576	GSM306224	GSE12172	Ovarian Cancer	Ovarian	2.23E-10	-3.77E-01	1.59E-03	-4.10E-01
6577	GSM306225	GSE12172	Ovarian Cancer	Ovarian	1.89E-03	-2.00E-01	6.65E-02	-2.66E-01
6578	GSM306226	GSE12172	Ovarian Cancer	Ovarian	2.25E-03	-1.98E-01	1.40E-01	-2.27E-01
6579	GSM306227	GSE12172	Ovarian Cancer	Ovarian	8.04E-04	-2.14E-01	6.73E-01	-1.02E-01
6580	GSM306228	GSE12172	Ovarian Cancer	Ovarian	1.63E-04	-2.36E-01	5.03E-01	1.34E-01
6581	GSM306229	GSE12172	Ovarian Cancer	Ovarian	1.31E-01	1.14E-01	3.40E-02	2.97E-01

6582	GSM306230	GSE12172	Ovarian Cancer	Ovarian	4.81E-04	-2.21E-01	4.32E-01	-1.48E-01
6583	GSM306231	GSE12172	Ovarian Cancer	Ovarian	2.17E-04	-2.32E-01	4.83E-01	-1.38E-01
6584	GSM306232	GSE12172	Ovarian Cancer	Ovarian	4.04E-05	2.55E-01	1.81E-04	4.74E-01
6585	GSM306233	GSE12172	Ovarian Cancer	Ovarian	2.25E-03	-1.98E-01	4.08E-01	-1.53E-01
6586	GSM306234	GSE12172	Ovarian Cancer	Ovarian	6.66E-04	-2.16E-01	3.99E-01	-1.55E-01
6587	GSM306235	GSE12172	Ovarian Cancer	Ovarian	2.26E-02	-1.56E-01	4.20E-01	1.50E-01
6588	GSM306236	GSE12172	Ovarian Cancer	Ovarian	6.31E-07	3.02E-01	3.90E-06	5.70E-01
6589	GSM306237	GSE12172	Ovarian Cancer	Ovarian	1.72E-03	-2.02E-01	3.33E-01	1.70E-01
6590	GSM306238	GSE12172	Ovarian Cancer	Ovarian	4.88E-04	2.21E-01	8.98E-04	4.28E-01
6591	GSM306239	GSE12172	Ovarian Cancer	Ovarian	4.72E-05	-2.53E-01	2.54E-01	-1.89E-01
6592	GSM306240	GSE12172	Ovarian Cancer	Ovarian	7.27E-04	2.15E-01	5.29E-06	5.63E-01
6593	GSM306241	GSE12172	Ovarian Cancer	Ovarian	8.04E-10	-3.66E-01	2.81E-02	-3.05E-01
6594	GSM306242	GSE12172	Ovarian Cancer	Ovarian	3.06E-08	-3.33E-01	1.83E-02	-3.23E-01
6595	GSM306243	GSE12172	Ovarian Cancer	Ovarian	1.93E-01	1.03E-01	1.01E-01	2.44E-01
6596	GSM306244	GSE12172	Ovarian Cancer	Ovarian	3.54E-01	8.16E-02	4.57E-02	2.84E-01
6597	GSM306245	GSE12172	Ovarian Cancer	Ovarian	2.99E-01	-8.79E-02	1.32E-01	2.30E-01
6598	GSM306246	GSE12172	Ovarian Cancer	Ovarian	2.54E-01	9.37E-02	7.46E-02	2.60E-01
6599	GSM306247	GSE12172	Ovarian Cancer	Ovarian	1.84E-02	-1.60E-01	1.73E-01	2.14E-01
6600	GSM107072	GSE1456	Breast Cancer	Breast	2.08E-01	9.92E-02	4.63E-02	2.83E-01
6601	GSM107073	GSE1456	Breast Cancer	Breast	4.49E-08	-3.25E-01	2.54E-03	-3.95E-01
6602	GSM107074	GSE1456	Breast Cancer	Breast	1.71E-14	-4.45E-01	9.12E-07	-6.03E-01
6603	GSM107075	GSE1456	Breast Cancer	Breast	1.65E-01	1.06E-01	1.03E-01	2.44E-01
6604	GSM107076	GSE1456	Breast Cancer	Breast	4.09E-03	1.86E-01	2.45E-03	3.96E-01
6605	GSM107077	GSE1456	Breast Cancer	Breast	1.18E-11	-3.97E-01	1.77E-04	-4.75E-01
6606	GSM107078	GSE1456	Breast Cancer	Breast	5.01E-02	-1.37E-01	1.96E-01	2.06E-01
6607	GSM107079	GSE1456	Breast Cancer	Breast	1.30E-01	-1.13E-01	2.49E-01	1.90E-01
6608	GSM107080	GSE1456	Breast Cancer	Breast	9.73E-04	2.08E-01	4.00E-03	3.80E-01
6609	GSM107081	GSE1456	Breast Cancer	Breast	8.56E-03	-1.73E-01	4.29E-01	-1.49E-01
6610	GSM107082	GSE1456	Breast Cancer	Breast	1.16E-01	1.16E-01	8.56E-02	2.53E-01
6611	GSM107083	GSE1456	Breast Cancer	Breast	2.21E-01	-9.72E-02	3.11E-01	1.75E-01
6612	GSM107084	GSE1456	Breast Cancer	Breast	1.50E-06	-2.90E-01	1.51E-02	-3.31E-01
6613	GSM107085	GSE1456	Breast Cancer	Breast	8.91E-02	-1.23E-01	1.51E-01	2.22E-01
6614	GSM107086	GSE1456	Breast Cancer	Breast	5.46E-02	-1.35E-01	5.03E-01	-1.34E-01
6615	GSM107087	GSE1456	Breast Cancer	Breast	1.53E-03	-2.01E-01	2.51E-01	-1.90E-01
6616	GSM107088	GSE1456	Breast Cancer	Breast	1.37E-07	-3.14E-01	6.62E-03	-3.62E-01
6617	GSM107089	GSE1456	Breast Cancer	Breast	4.94E-01	6.65E-02	2.33E-01	1.95E-01
6618	GSM107090	GSE1456	Breast Cancer	Breast	4.03E-01	-7.54E-02	6.39E-02	2.68E-01
6619	GSM107091	GSE1456	Breast Cancer	Breast	3.10E-06	-2.82E-01	2.17E-02	-3.16E-01
6620	GSM107092	GSE1456	Breast Cancer	Breast	3.66E-07	-3.05E-01	4.22E-03	-3.78E-01
6621	GSM107093	GSE1456	Breast Cancer	Breast	5.79E-03	-1.80E-01	2.33E-01	-1.95E-01
6622	GSM107094	GSE1456	Breast Cancer	Breast	2.50E-11	-3.91E-01	7.28E-04	-4.34E-01
6623	GSM107095	GSE1456	Breast Cancer	Breast	2.53E-03	-1.93E-01	4.26E-01	-1.49E-01
6624	GSM107096	GSE1456	Breast Cancer	Breast	1.01E-02	1.70E-01	3.70E-02	2.93E-01
6625	GSM107097	GSE1456	Breast Cancer	Breast	1.58E-08	-3.35E-01	7.75E-03	-3.56E-01
6626	GSM107098	GSE1456	Breast Cancer	Breast	5.01E-02	1.37E-01	6.39E-02	2.68E-01
6627	GSM107099	GSE1456	Breast Cancer	Breast	5.39E-05	-2.48E-01	3.81E-02	-2.92E-01
6628	GSM107100	GSE1456	Breast Cancer	Breast	2.88E-01	8.83E-02	2.72E-02	3.07E-01
6629	GSM107101	GSE1456	Breast Cancer	Breast	6.84E-06	2.73E-01	1.28E-04	4.84E-01
6630	GSM107102	GSE1456	Breast Cancer	Breast	1.49E-01	1.09E-01	1.00E-01	2.45E-01
6631	GSM107103	GSE1456	Breast Cancer	Breast	1.77E-01	1.04E-01	1.73E-01	2.14E-01
6632	GSM107104	GSE1456	Breast Cancer	Breast	2.29E-02	-1.54E-01	2.10E-01	-2.02E-01
6633	GSM107105	GSE1456	Breast Cancer	Breast	4.39E-02	-1.40E-01	5.43E-01	-1.26E-01
6634	GSM107106	GSE1456	Breast Cancer	Breast	2.18E-02	1.55E-01	2.14E-02	3.17E-01
6635	GSM107107	GSE1456	Breast Cancer	Breast	1.63E-03	-2.00E-01	2.83E-01	-1.82E-01
6636	GSM107108	GSE1456	Breast Cancer	Breast	2.28E-01	-9.62E-02	5.09E-01	1.33E-01
6637	GSM107109	GSE1456	Breast Cancer	Breast	3.47E-01	-8.13E-02	1.37E-01	2.28E-01
6638	GSM107110	GSE1456	Breast Cancer	Breast	3.19E-02	-1.47E-01	3.19E-01	-1.73E-01
6639	GSM107111	GSE1456	Breast Cancer	Breast	5.83E-05	2.47E-01	9.83E-05	4.91E-01
6640	GSM107112	GSE1456	Breast Cancer	Breast	1.16E-01	-1.16E-01	3.46E-01	1.66E-01
6641	GSM107113	GSE1456	Breast Cancer	Breast	8.59E-05	-2.42E-01	4.51E-02	-2.84E-01
6642	GSM107114	GSE1456	Breast Cancer	Breast	2.84E-05	-2.56E-01	6.31E-02	-2.69E-01
6643	GSM107115	GSE1456	Breast Cancer	Breast	4.44E-07	3.03E-01	1.98E-03	4.03E-01
6644	GSM107117	GSE1456	Breast Cancer	Breast	1.12E-01	-1.17E-01	1.34E-01	2.29E-01
6645	GSM107118	GSE1456	Breast Cancer	Breast	2.64E-01	9.13E-02	2.02E-01	2.04E-01
6646	GSM107119	GSE1456	Breast Cancer	Breast	6.73E-02	-1.30E-01	5.03E-01	1.34E-01
6647	GSM107120	GSE1456	Breast Cancer	Breast	7.96E-05	-2.43E-01	2.49E-01	1.90E-01
6648	GSM107121	GSE1456	Breast Cancer	Breast	2.27E-04	2.29E-01	1.37E-04	4.82E-01
6649	GSM107122	GSE1456	Breast Cancer	Breast	8.50E-11	3.81E-01	3.02E-07	6.26E-01
6650	GSM107123	GSE1456	Breast Cancer	Breast	1.08E-04	-2.39E-01	4.61E-01	-1.42E-01
6651	GSM107124	GSE1456	Breast Cancer	Breast	1.25E-18	5.08E-01	5.70E-10	7.45E-01
6652	GSM107125	GSE1456	Breast Cancer	Breast	9.27E-05	-2.41E-01	7.74E-02	-2.58E-01
6653	GSM107126	GSE1456	Breast Cancer	Breast	1.96E-04	-2.31E-01	2.63E-01	-1.87E-01
6654	GSM107127	GSE1456	Breast Cancer	Breast	5.01E-02	-1.37E-01	4.20E-01	1.50E-01
6655	GSM107128	GSE1456	Breast Cancer	Breast	1.79E-02	-1.59E-01	8.66E-02	-2.53E-01

6656	GSM107129	GSE1456	Breast Cancer	Breast	1.69E-04	2.33E-01	4.15E-03	3.78E-01
6657	GSM107130	GSE1456	Breast Cancer	Breast	7.96E-05	-2.43E-01	1.61E-01	-2.18E-01
6658	GSM107131	GSE1456	Breast Cancer	Breast	7.45E-04	2.12E-01	7.75E-03	3.56E-01
6659	GSM107132	GSE1456	Breast Cancer	Breast	5.01E-02	-1.37E-01	3.84E-01	1.58E-01
6660	GSM107133	GSE1456	Breast Cancer	Breast	1.16E-04	-2.38E-01	1.51E-02	-3.31E-01
6661	GSM107134	GSE1456	Breast Cancer	Breast	6.48E-03	-1.78E-01	2.02E-01	-2.04E-01
6662	GSM107135	GSE1456	Breast Cancer	Breast	1.89E-05	2.61E-01	2.38E-04	4.67E-01
6663	GSM107136	GSE1456	Breast Cancer	Breast	3.03E-04	2.25E-01	3.85E-03	3.81E-01
6664	GSM107137	GSE1456	Breast Cancer	Breast	6.86E-03	-1.77E-01	2.00E-01	-2.05E-01
6665	GSM107138	GSE1456	Breast Cancer	Breast	1.38E-10	-3.77E-01	9.36E-04	-4.27E-01
6666	GSM107139	GSE1456	Breast Cancer	Breast	2.96E-01	8.73E-02	1.98E-01	2.06E-01
6667	GSM107140	GSE1456	Breast Cancer	Breast	2.18E-02	1.55E-01	4.57E-02	2.84E-01
6668	GSM107141	GSE1456	Breast Cancer	Breast	1.04E-03	2.07E-01	1.22E-02	3.39E-01
6669	GSM107142	GSE1456	Breast Cancer	Breast	1.88E-02	1.58E-01	2.11E-02	3.17E-01
6670	GSM107143	GSE1456	Breast Cancer	Breast	9.63E-02	-1.21E-01	2.51E-01	1.90E-01
6671	GSM107144	GSE1456	Breast Cancer	Breast	1.31E-02	-1.65E-01	4.58E-01	1.43E-01
6672	GSM107145	GSE1456	Breast Cancer	Breast	1.08E-01	1.18E-01	1.17E-01	2.37E-01
6673	GSM107146	GSE1456	Breast Cancer	Breast	1.54E-01	1.08E-01	1.57E-01	2.20E-01
6674	GSM107147	GSE1456	Breast Cancer	Breast	1.61E-02	-1.61E-01	3.11E-01	1.75E-01
6675	GSM107148	GSE1456	Breast Cancer	Breast	1.38E-02	-1.64E-01	1.32E-01	2.30E-01
6676	GSM107149	GSE1456	Breast Cancer	Breast	5.26E-18	-4.99E-01	7.19E-05	-4.99E-01
6677	GSM107150	GSE1456	Breast Cancer	Breast	1.97E-03	-1.97E-01	3.11E-01	1.75E-01
6678	GSM107151	GSE1456	Breast Cancer	Breast	2.14E-01	-9.82E-02	1.56E-01	2.20E-01
6679	GSM107152	GSE1456	Breast Cancer	Breast	1.47E-05	-2.64E-01	1.77E-02	-3.24E-01
6680	GSM107153	GSE1456	Breast Cancer	Breast	2.07E-02	1.56E-01	8.45E-02	2.54E-01
6681	GSM107154	GSE1456	Breast Cancer	Breast	9.65E-06	-2.69E-01	1.46E-02	-3.32E-01
6682	GSM107155	GSE1456	Breast Cancer	Breast	9.63E-02	1.21E-01	1.96E-01	2.06E-01
6683	GSM107156	GSE1456	Breast Cancer	Breast	9.27E-05	2.41E-01	5.17E-04	4.44E-01
6684	GSM107157	GSE1456	Breast Cancer	Breast	5.46E-02	-1.35E-01	1.32E-01	2.30E-01
6685	GSM107158	GSE1456	Breast Cancer	Breast	1.26E-03	-2.04E-01	1.98E-01	-2.06E-01
6686	GSM107159	GSE1456	Breast Cancer	Breast	1.96E-04	2.31E-01	4.89E-03	3.73E-01
6687	GSM107160	GSE1456	Breast Cancer	Breast	6.73E-02	1.30E-01	3.19E-03	3.87E-01
6688	GSM107161	GSE1456	Breast Cancer	Breast	5.46E-02	1.35E-01	7.74E-02	2.58E-01
6689	GSM107162	GSE1456	Breast Cancer	Breast	6.19E-02	1.32E-01	3.11E-02	3.01E-01
6690	GSM107163	GSE1456	Breast Cancer	Breast	1.26E-03	-2.04E-01	1.35E-01	-2.29E-01
6691	GSM107164	GSE1456	Breast Cancer	Breast	1.12E-07	3.16E-01	4.16E-04	4.51E-01
6692	GSM107165	GSE1456	Breast Cancer	Breast	4.60E-05	2.50E-01	4.07E-04	4.51E-01
6693	GSM107166	GSE1456	Breast Cancer	Breast	1.04E-06	2.94E-01	2.82E-08	6.74E-01
6694	GSM107167	GSE1456	Breast Cancer	Breast	4.31E-04	2.20E-01	3.85E-03	3.81E-01
6695	GSM107168	GSE1456	Breast Cancer	Breast	3.67E-02	-1.44E-01	2.04E-01	2.04E-01
6696	GSM107169	GSE1456	Breast Cancer	Breast	3.86E-03	1.87E-01	3.08E-03	3.89E-01
6697	GSM107170	GSE1456	Breast Cancer	Breast	7.14E-07	2.98E-01	1.69E-04	4.76E-01
6698	GSM107171	GSE1456	Breast Cancer	Breast	1.08E-01	1.18E-01	7.27E-02	2.62E-01
6699	GSM107172	GSE1456	Breast Cancer	Breast	1.58E-08	3.35E-01	1.07E-05	5.47E-01
6700	GSM107173	GSE1456	Breast Cancer	Breast	3.75E-01	-7.84E-02	1.73E-01	2.14E-01
6701	GSM107174	GSE1456	Breast Cancer	Breast	5.94E-02	1.33E-01	5.24E-02	2.77E-01
6702	GSM107175	GSE1456	Breast Cancer	Breast	8.13E-06	2.71E-01	9.83E-05	4.91E-01
6703	GSM107176	GSE1456	Breast Cancer	Breast	5.04E-01	6.55E-02	9.10E-02	2.50E-01
6704	GSM107177	GSE1456	Breast Cancer	Breast	1.06E-02	-1.69E-01	2.83E-01	-1.82E-01
6705	GSM107178	GSE1456	Breast Cancer	Breast	7.46E-06	-2.72E-01	2.36E-01	-1.94E-01
6706	GSM107179	GSE1456	Breast Cancer	Breast	8.24E-02	-1.25E-01	1.77E-01	-2.13E-01
6707	GSM107180	GSE1456	Breast Cancer	Breast	1.08E-19	-5.23E-01	1.33E-06	-5.94E-01
6708	GSM107181	GSE1456	Breast Cancer	Breast	2.28E-01	-9.62E-02	7.36E-02	2.61E-01
6709	GSM107182	GSE1456	Breast Cancer	Breast	9.73E-04	2.08E-01	8.79E-04	4.29E-01
6710	GSM107183	GSE1456	Breast Cancer	Breast	1.35E-03	2.03E-01	7.13E-04	4.35E-01
6711	GSM107184	GSE1456	Breast Cancer	Breast	6.73E-02	1.30E-01	6.15E-02	2.70E-01
6712	GSM107185	GSE1456	Breast Cancer	Breast	2.77E-02	1.50E-01	1.77E-02	3.24E-01
6713	GSM107186	GSE1456	Breast Cancer	Breast	6.86E-44	7.89E-01	2.31E-17	1.00E+00
6714	GSM107187	GSE1456	Breast Cancer	Breast	1.77E-01	1.04E-01	3.81E-02	2.92E-01
6715	GSM107188	GSE1456	Breast Cancer	Breast	2.28E-01	9.62E-02	6.31E-02	2.69E-01
6716	GSM107189	GSE1456	Breast Cancer	Breast	1.46E-02	1.63E-01	6.98E-04	4.36E-01
6717	GSM107190	GSE1456	Breast Cancer	Breast	7.25E-03	1.76E-01	2.40E-03	3.97E-01
6718	GSM107191	GSE1456	Breast Cancer	Breast	1.18E-02	-1.67E-01	2.06E-01	-2.03E-01
6719	GSM107192	GSE1456	Breast Cancer	Breast	4.88E-07	-3.02E-01	3.92E-03	-3.80E-01
6720	GSM107193	GSE1456	Breast Cancer	Breast	4.42E-06	-2.78E-01	2.14E-02	-3.17E-01
6721	GSM107194	GSE1456	Breast Cancer	Breast	4.25E-05	-2.51E-01	7.55E-02	-2.60E-01
6722	GSM107195	GSE1456	Breast Cancer	Breast	4.69E-20	-5.28E-01	5.43E-06	-5.63E-01
6723	GSM107196	GSE1456	Breast Cancer	Breast	4.34E-03	-1.85E-01	1.37E-01	-2.28E-01
6724	GSM107197	GSE1456	Breast Cancer	Breast	3.94E-01	-7.64E-02	5.00E-01	1.35E-01
6725	GSM107198	GSE1456	Breast Cancer	Breast	4.63E-11	-3.86E-01	2.38E-04	-4.67E-01
6726	GSM107199	GSE1456	Breast Cancer	Breast	1.60E-05	-2.63E-01	1.46E-02	-3.32E-01
6727	GSM107200	GSE1456	Breast Cancer	Breast	2.52E-02	-1.52E-01	1.96E-01	2.06E-01
6728	GSM107201	GSE1456	Breast Cancer	Breast	2.59E-06	-2.84E-01	6.23E-02	-2.69E-01
6729	GSM107202	GSE1456	Breast Cancer	Breast	5.36E-01	6.25E-02	4.38E-02	2.86E-01

6730	GSM107203	GSE1456	Breast Cancer	Breast	2.07E-02	-1.56E-01	4.58E-01	1.43E-01
6731	GSM107204	GSE1456	Breast Cancer	Breast	4.98E-08	-3.24E-01	1.20E-03	-4.19E-01
6732	GSM107205	GSE1456	Breast Cancer	Breast	4.31E-09	-3.47E-01	6.62E-03	-3.62E-01
6733	GSM107206	GSE1456	Breast Cancer	Breast	3.93E-05	-2.52E-01	1.05E-01	-2.43E-01
6734	GSM107207	GSE1456	Breast Cancer	Breast	1.25E-02	1.66E-01	1.18E-02	3.40E-01
6735	GSM107208	GSE1456	Breast Cancer	Breast	3.04E-01	8.63E-02	2.45E-03	3.96E-01
6736	GSM107209	GSE1456	Breast Cancer	Breast	4.34E-03	1.85E-01	3.98E-04	4.52E-01
6737	GSM107210	GSE1456	Breast Cancer	Breast	1.57E-04	-2.34E-01	4.45E-02	-2.85E-01
6738	GSM107211	GSE1456	Breast Cancer	Breast	1.85E-03	-1.98E-01	2.88E-01	-1.80E-01
6739	GSM107212	GSE1456	Breast Cancer	Breast	1.12E-01	-1.17E-01	2.49E-01	1.90E-01
6740	GSM107213	GSE1456	Breast Cancer	Breast	2.88E-01	-8.83E-02	4.61E-01	1.42E-01
6741	GSM107214	GSE1456	Breast Cancer	Breast	1.54E-01	1.08E-01	8.98E-04	4.28E-01
6742	GSM107215	GSE1456	Breast Cancer	Breast	4.02E-04	2.21E-01	8.98E-04	4.28E-01
6743	GSM107216	GSE1456	Breast Cancer	Breast	8.52E-04	-2.10E-01	1.56E-01	-2.20E-01
6744	GSM107217	GSE1456	Breast Cancer	Breast	1.08E-01	-1.18E-01	1.56E-01	2.20E-01
6745	GSM107218	GSE1456	Breast Cancer	Breast	3.19E-02	-1.47E-01	5.06E-01	-1.33E-01
6746	GSM107219	GSE1456	Breast Cancer	Breast	2.88E-01	-8.83E-02	3.46E-01	1.66E-01
6747	GSM107220	GSE1456	Breast Cancer	Breast	1.70E-02	-1.60E-01	9.90E-02	2.46E-01
6748	GSM107221	GSE1456	Breast Cancer	Breast	5.47E-03	1.81E-01	2.28E-04	4.68E-01
6749	GSM107222	GSE1456	Breast Cancer	Breast	1.88E-02	-1.58E-01	2.80E-01	1.82E-01
6750	GSM107223	GSE1456	Breast Cancer	Breast	1.31E-02	-1.65E-01	2.33E-01	1.95E-01
6751	GSM107224	GSE1456	Breast Cancer	Breast	7.60E-02	1.27E-01	6.39E-02	2.68E-01
6752	GSM107225	GSE1456	Breast Cancer	Breast	8.59E-05	2.42E-01	7.01E-05	5.00E-01
6753	GSM107226	GSE1456	Breast Cancer	Breast	8.13E-06	-2.71E-01	2.17E-02	-3.16E-01
6754	GSM107227	GSE1456	Breast Cancer	Breast	8.13E-06	-2.71E-01	1.51E-02	-3.31E-01
6755	GSM107228	GSE1456	Breast Cancer	Breast	4.60E-03	-1.84E-01	1.98E-01	-2.06E-01
6756	GSM107229	GSE1456	Breast Cancer	Breast	2.23E-05	-2.59E-01	1.46E-02	-3.32E-01
6757	GSM107230	GSE1456	Breast Cancer	Breast	5.79E-03	-1.80E-01	1.96E-01	2.06E-01
6758	GSM107231	GSE1456	Breast Cancer	Breast	1.65E-06	-2.89E-01	2.68E-02	-3.07E-01
6759	GSM26804	GSE1561	Breast Cancer	Breast	3.04E-01	-8.63E-02	1.75E-01	2.13E-01
6760	GSM26867	GSE1561	Breast Cancer	Breast	4.87E-03	-1.83E-01	8.66E-02	-2.53E-01
6761	GSM26868	GSE1561	Breast Cancer	Breast	7.46E-06	-2.72E-01	1.54E-02	-3.30E-01
6762	GSM26869	GSE1561	Breast Cancer	Breast	4.73E-01	6.85E-02	1.17E-01	2.37E-01
6763	GSM26870	GSE1561	Breast Cancer	Breast	1.00E-01	1.20E-01	5.24E-02	2.77E-01
6764	GSM26871	GSE1561	Breast Cancer	Breast	1.97E-03	1.97E-01	3.25E-03	3.87E-01
6765	GSM26872	GSE1561	Breast Cancer	Breast	2.53E-03	1.93E-01	6.28E-03	3.64E-01
6766	GSM26873	GSE1561	Breast Cancer	Breast	8.56E-03	1.73E-01	9.36E-03	3.49E-01
6767	GSM26874	GSE1561	Breast Cancer	Breast	8.91E-02	-1.23E-01	1.52E-01	2.22E-01
6768	GSM26875	GSE1561	Breast Cancer	Breast	2.36E-13	-4.27E-01	1.07E-05	-5.47E-01
6769	GSM26876	GSE1561	Breast Cancer	Breast	1.39E-01	-1.11E-01	6.31E-02	2.69E-01
6770	GSM26877	GSE1561	Breast Cancer	Breast	5.94E-02	1.33E-01	1.44E-02	3.33E-01
6771	GSM26878	GSE1561	Breast Cancer	Breast	2.42E-05	-2.58E-01	1.59E-01	-2.19E-01
6772	GSM26879	GSE1561	Breast Cancer	Breast	3.94E-01	-7.64E-02	1.34E-01	2.29E-01
6773	GSM26880	GSE1561	Breast Cancer	Breast	4.53E-01	-7.04E-02	1.15E-01	2.37E-01
6774	GSM26881	GSE1561	Breast Cancer	Breast	2.53E-03	-1.93E-01	1.75E-01	-2.13E-01
6775	GSM26882	GSE1561	Breast Cancer	Breast	3.09E-09	3.50E-01	6.33E-08	6.58E-01
6776	GSM26883	GSE1561	Breast Cancer	Breast	1.14E-05	-2.67E-01	7.74E-02	-2.58E-01
6777	GSM26884	GSE1561	Breast Cancer	Breast	2.14E-01	9.82E-02	3.11E-02	3.01E-01
6778	GSM26885	GSE1561	Breast Cancer	Breast	1.79E-02	1.59E-01	6.06E-03	3.65E-01
6779	GSM26886	GSE1561	Breast Cancer	Breast	2.35E-01	9.52E-02	1.14E-01	2.38E-01
6780	GSM26887	GSE1561	Breast Cancer	Breast	5.53E-12	-4.03E-01	1.20E-02	-3.40E-01
6781	GSM26888	GSE1561	Breast Cancer	Breast	3.21E-22	5.57E-01	1.46E-10	7.69E-01
6782	GSM26889	GSE1561	Breast Cancer	Breast	1.71E-01	1.05E-01	1.14E-01	2.38E-01
6783	GSM26890	GSE1561	Breast Cancer	Breast	1.04E-01	-1.19E-01	2.51E-01	1.90E-01
6784	GSM26891	GSE1561	Breast Cancer	Breast	1.48E-14	-4.46E-01	7.53E-06	-5.55E-01
6785	GSM26892	GSE1561	Breast Cancer	Breast	5.23E-02	-1.36E-01	2.27E-01	-1.97E-01
6786	GSM26893	GSE1561	Breast Cancer	Breast	5.23E-02	-1.36E-01	3.93E-01	-1.56E-01
6787	GSM26894	GSE1561	Breast Cancer	Breast	8.10E-03	1.74E-01	1.98E-03	4.03E-01
6788	GSM26895	GSE1561	Breast Cancer	Breast	2.14E-01	-9.82E-02	3.72E-01	1.61E-01
6789	GSM26896	GSE1561	Breast Cancer	Breast	1.60E-01	1.07E-01	1.75E-02	3.25E-01
6790	GSM26897	GSE1561	Breast Cancer	Breast	3.25E-04	-2.24E-01	6.48E-02	-2.67E-01
6791	GSM26898	GSE1561	Breast Cancer	Breast	2.14E-01	-9.82E-02	1.77E-01	2.13E-01
6792	GSM26899	GSE1561	Breast Cancer	Breast	1.11E-03	-2.06E-01	4.45E-02	-2.85E-01
6793	GSM26900	GSE1561	Breast Cancer	Breast	3.04E-03	1.90E-01	9.36E-03	3.49E-01
6794	GSM26901	GSE1561	Breast Cancer	Breast	8.13E-06	-2.71E-01	1.46E-02	-3.32E-01
6795	GSM26902	GSE1561	Breast Cancer	Breast	1.60E-01	-1.07E-01	2.22E-01	1.98E-01
6796	GSM26903	GSE1561	Breast Cancer	Breast	6.08E-04	-2.15E-01	2.27E-01	-1.97E-01
6797	GSM26904	GSE1561	Breast Cancer	Breast	4.01E-02	-1.42E-01	2.22E-01	1.98E-01
6798	GSM26905	GSE1561	Breast Cancer	Breast	6.51E-04	2.14E-01	8.98E-04	4.28E-01
6799	GSM26906	GSE1561	Breast Cancer	Breast	8.59E-05	2.42E-01	2.44E-04	4.66E-01
6800	GSM26907	GSE1561	Breast Cancer	Breast	9.46E-07	-2.95E-01	1.83E-02	-3.23E-01
6801	GSM26908	GSE1561	Breast Cancer	Breast	5.15E-01	-6.45E-02	2.02E-01	2.04E-01
6802	GSM26909	GSE1561	Breast Cancer	Breast	7.67E-03	1.75E-01	9.52E-03	3.49E-01
6803	GSM26910	GSE1561	Breast Cancer	Breast	1.11E-03	-2.06E-01	3.63E-01	-1.63E-01

6804	GSM26911	GSE1561	Breast Cancer	Breast	2.62E-04	-2.27E-01	7.36E-02	-2.61E-01
6805	GSM26912	GSE1561	Breast Cancer	Breast	1.79E-02	-1.59E-01	4.32E-01	1.48E-01
6806	GSM26913	GSE1561	Breast Cancer	Breast	4.33E-01	7.24E-02	3.84E-01	1.58E-01
6807	GSM26914	GSE1561	Breast Cancer	Breast	8.86E-06	-2.70E-01	2.68E-02	-3.07E-01
6808	GSM36777	GSE2034	Breast Cancer	Breast	1.08E-01	-1.18E-01	9.90E-02	2.46E-01
6809	GSM36778	GSE2034	Breast Cancer	Breast	1.04E-03	-2.07E-01	1.21E-01	-2.35E-01
6810	GSM36779	GSE2034	Breast Cancer	Breast	2.52E-17	-4.89E-01	3.02E-07	-6.26E-01
6811	GSM36780	GSE2034	Breast Cancer	Breast	1.97E-03	-1.97E-01	3.52E-01	-1.65E-01
6812	GSM36781	GSE2034	Breast Cancer	Breast	2.42E-01	-9.42E-02	2.10E-01	2.02E-01
6813	GSM36782	GSE2034	Breast Cancer	Breast	1.44E-03	2.02E-01	1.20E-02	3.40E-01
6814	GSM36783	GSE2034	Breast Cancer	Breast	5.23E-11	-3.85E-01	5.35E-03	-3.70E-01
6815	GSM36784	GSE2034	Breast Cancer	Breast	2.42E-01	-9.42E-02	3.11E-01	1.75E-01
6816	GSM36785	GSE2034	Breast Cancer	Breast	1.74E-03	-1.99E-01	1.37E-01	-2.28E-01
6817	GSM36786	GSE2034	Breast Cancer	Breast	2.88E-01	8.83E-02	4.45E-02	2.85E-01
6818	GSM36787	GSE2034	Breast Cancer	Breast	1.46E-04	-2.35E-01	3.27E-01	-1.71E-01
6819	GSM36788	GSE2034	Breast Cancer	Breast	9.04E-03	1.72E-01	1.80E-02	3.24E-01
6820	GSM36789	GSE2034	Breast Cancer	Breast	9.73E-04	-2.08E-01	3.30E-01	-1.70E-01
6821	GSM36790	GSE2034	Breast Cancer	Breast	1.95E-01	1.01E-01	1.32E-01	2.30E-01
6822	GSM36791	GSE2034	Breast Cancer	Breast	2.01E-01	-1.00E-01	4.20E-01	1.50E-01
6823	GSM36792	GSE2034	Breast Cancer	Breast	3.66E-07	-3.05E-01	7.88E-03	-3.56E-01
6824	GSM36793	GSE2034	Breast Cancer	Breast	4.53E-01	-7.04E-02	1.57E-01	2.20E-01
6825	GSM36794	GSE2034	Breast Cancer	Breast	1.44E-20	5.35E-01	7.15E-14	8.89E-01
6826	GSM36795	GSE2034	Breast Cancer	Breast	1.98E-06	-2.87E-01	2.45E-03	-3.96E-01
6827	GSM36796	GSE2034	Breast Cancer	Breast	6.08E-04	-2.15E-01	2.90E-01	-1.80E-01
6828	GSM36797	GSE2034	Breast Cancer	Breast	2.18E-02	1.55E-01	4.38E-02	2.86E-01
6829	GSM36798	GSE2034	Breast Cancer	Breast	2.52E-02	1.52E-01	4.00E-03	3.80E-01
6830	GSM36799	GSE2034	Breast Cancer	Breast	6.97E-04	-2.13E-01	2.90E-01	-1.80E-01
6831	GSM36800	GSE2034	Breast Cancer	Breast	4.98E-05	-2.49E-01	8.56E-02	-2.53E-01
6832	GSM36801	GSE2034	Breast Cancer	Breast	4.13E-01	7.44E-02	2.20E-02	3.16E-01
6833	GSM36802	GSE2034	Breast Cancer	Breast	2.29E-02	-1.54E-01	1.79E-01	-2.12E-01
6834	GSM36803	GSE2034	Breast Cancer	Breast	1.74E-05	-2.62E-01	3.25E-02	-2.99E-01
6835	GSM36804	GSE2034	Breast Cancer	Breast	5.69E-02	-1.34E-01	4.20E-01	1.50E-01
6836	GSM36805	GSE2034	Breast Cancer	Breast	3.86E-09	-3.48E-01	3.16E-02	-3.00E-01
6837	GSM36806	GSE2034	Breast Cancer	Breast	2.80E-01	-8.93E-02	1.51E-01	2.22E-01
6838	GSM36807	GSE2034	Breast Cancer	Breast	1.04E-01	1.19E-01	1.04E-01	2.43E-01
6839	GSM36808	GSE2034	Breast Cancer	Breast	2.07E-02	1.56E-01	6.15E-02	2.70E-01
6840	GSM36809	GSE2034	Breast Cancer	Breast	3.54E-15	4.56E-01	3.36E-09	7.14E-01
6841	GSM36810	GSE2034	Breast Cancer	Breast	1.46E-04	-2.35E-01	1.17E-01	-2.37E-01
6842	GSM36811	GSE2034	Breast Cancer	Breast	9.73E-04	2.08E-01	1.29E-06	5.95E-01
6843	GSM36812	GSE2034	Breast Cancer	Breast	1.79E-02	1.59E-01	7.36E-02	2.61E-01
6844	GSM36813	GSE2034	Breast Cancer	Breast	2.40E-02	1.53E-01	2.11E-02	3.17E-01
6845	GSM36814	GSE2034	Breast Cancer	Breast	4.79E-02	1.38E-01	4.98E-03	3.72E-01
6846	GSM36815	GSE2034	Breast Cancer	Breast	2.64E-02	-1.51E-01	1.14E-01	2.38E-01
6847	GSM36816	GSE2034	Breast Cancer	Breast	3.54E-10	3.69E-01	2.82E-08	6.74E-01
6848	GSM36817	GSE2034	Breast Cancer	Breast	4.34E-03	1.85E-01	2.59E-03	3.94E-01
6849	GSM36818	GSE2034	Breast Cancer	Breast	3.49E-04	2.23E-01	5.17E-04	4.44E-01
6850	GSM36819	GSE2034	Breast Cancer	Breast	2.38E-03	-1.94E-01	1.75E-01	-2.13E-01
6851	GSM36820	GSE2034	Breast Cancer	Breast	1.16E-04	-2.38E-01	2.29E-01	-1.96E-01
6852	GSM36821	GSE2034	Breast Cancer	Breast	6.13E-03	-1.79E-01	1.17E-01	-2.37E-01
6853	GSM36822	GSE2034	Breast Cancer	Breast	2.42E-05	-2.58E-01	2.61E-02	-3.09E-01
6854	GSM36823	GSE2034	Breast Cancer	Breast	1.24E-05	-2.66E-01	3.25E-02	-2.99E-01
6855	GSM36824	GSE2034	Breast Cancer	Breast	2.77E-02	1.50E-01	6.39E-03	3.63E-01
6856	GSM36825	GSE2034	Breast Cancer	Breast	5.46E-02	1.35E-01	8.45E-02	2.54E-01
6857	GSM36826	GSE2034	Breast Cancer	Breast	1.16E-04	2.38E-01	4.89E-03	3.73E-01
6858	GSM36827	GSE2034	Breast Cancer	Breast	2.24E-03	1.95E-01	1.59E-02	3.29E-01
6859	GSM36828	GSE2034	Breast Cancer	Breast	2.29E-02	1.54E-01	2.17E-02	3.16E-01
6860	GSM36829	GSE2034	Breast Cancer	Breast	1.83E-01	1.03E-01	6.15E-02	2.70E-01
6861	GSM36830	GSE2034	Breast Cancer	Breast	1.97E-02	-1.57E-01	2.22E-01	1.98E-01
6862	GSM36831	GSE2034	Breast Cancer	Breast	2.77E-02	1.50E-01	1.44E-02	3.33E-01
6863	GSM36832	GSE2034	Breast Cancer	Breast	1.04E-01	1.19E-01	8.66E-02	2.53E-01
6864	GSM36833	GSE2034	Breast Cancer	Breast	3.50E-02	-1.45E-01	4.58E-01	1.43E-01
6865	GSM36834	GSE2034	Breast Cancer	Breast	6.12E-08	-3.22E-01	9.52E-03	-3.49E-01
6866	GSM36835	GSE2034	Breast Cancer	Breast	2.21E-01	9.72E-02	6.15E-02	2.70E-01
6867	GSM36836	GSE2034	Breast Cancer	Breast	9.65E-06	-2.69E-01	3.81E-02	-2.92E-01
6868	GSM36837	GSE2034	Breast Cancer	Breast	4.44E-07	-3.03E-01	9.10E-02	-2.50E-01
6869	GSM36838	GSE2034	Breast Cancer	Breast	1.46E-04	-2.35E-01	1.22E-02	-3.39E-01
6870	GSM36839	GSE2034	Breast Cancer	Breast	6.28E-06	-2.74E-01	1.77E-02	-3.24E-01
6871	GSM36840	GSE2034	Breast Cancer	Breast	2.49E-01	9.33E-02	1.96E-01	2.06E-01
6872	GSM36841	GSE2034	Breast Cancer	Breast	2.86E-03	-1.91E-01	2.51E-01	-1.90E-01
6873	GSM36842	GSE2034	Breast Cancer	Breast	6.81E-05	-2.45E-01	8.45E-02	-2.54E-01
6874	GSM36843	GSE2034	Breast Cancer	Breast	9.11E-04	-2.09E-01	3.86E-02	-2.91E-01
6875	GSM36844	GSE2034	Breast Cancer	Breast	1.65E-01	-1.06E-01	1.73E-01	2.14E-01
6876	GSM36845	GSE2034	Breast Cancer	Breast	1.17E-12	-4.15E-01	3.88E-05	-5.15E-01
6877	GSM36846	GSE2034	Breast Cancer	Breast	1.70E-02	1.60E-01	4.38E-02	2.86E-01

6878	GSM36847	GSE2034	Breast Cancer	Breast	7.97E-04	-2.11E-01	4.07E-03	-3.79E-01
6879	GSM36848	GSE2034	Breast Cancer	Breast	2.84E-05	2.56E-01	2.79E-05	5.23E-01
6880	GSM36849	GSE2034	Breast Cancer	Breast	2.37E-06	-2.85E-01	1.46E-02	-3.32E-01
6881	GSM36850	GSE2034	Breast Cancer	Breast	7.01E-02	1.29E-01	9.36E-03	3.49E-01
6882	GSM36851	GSE2034	Breast Cancer	Breast	2.53E-03	1.93E-01	1.56E-03	4.11E-01
6883	GSM36852	GSE2034	Breast Cancer	Breast	2.28E-01	9.62E-02	3.58E-01	1.64E-01
6884	GSM36853	GSE2034	Breast Cancer	Breast	1.54E-01	-1.08E-01	2.64E-02	3.08E-01
6885	GSM36854	GSE2034	Breast Cancer	Breast	1.52E-11	3.95E-01	2.03E-07	6.34E-01
6886	GSM36855	GSE2034	Breast Cancer	Breast	5.91E-07	3.00E-01	4.07E-04	4.51E-01
6887	GSM36856	GSE2034	Breast Cancer	Breast	1.27E-08	-3.37E-01	4.30E-03	-3.77E-01
6888	GSM36857	GSE2034	Breast Cancer	Breast	3.56E-01	-8.04E-02	2.22E-01	1.98E-01
6889	GSM36858	GSE2034	Breast Cancer	Breast	3.49E-04	-2.23E-01	1.57E-01	-2.20E-01
6890	GSM36859	GSE2034	Breast Cancer	Breast	3.56E-01	8.04E-02	1.34E-01	2.29E-01
6891	GSM36860	GSE2034	Breast Cancer	Breast	1.79E-02	1.59E-01	3.85E-03	3.81E-01
6892	GSM36861	GSE2034	Breast Cancer	Breast	3.01E-07	-3.07E-01	2.68E-02	-3.07E-01
6893	GSM36862	GSE2034	Breast Cancer	Breast	2.53E-03	1.93E-01	1.83E-02	3.23E-01
6894	GSM36863	GSE2034	Breast Cancer	Breast	6.19E-02	1.32E-01	8.16E-03	3.54E-01
6895	GSM36864	GSE2034	Breast Cancer	Breast	5.15E-01	6.45E-02	1.51E-01	2.22E-01
6896	GSM36865	GSE2034	Breast Cancer	Breast	1.63E-03	2.00E-01	3.76E-02	2.93E-01
6897	GSM36866	GSE2034	Breast Cancer	Breast	3.36E-16	-4.72E-01	2.72E-06	-5.78E-01
6898	GSM36867	GSE2034	Breast Cancer	Breast	1.42E-08	-3.36E-01	4.98E-03	-3.72E-01
6899	GSM36868	GSE2034	Breast Cancer	Breast	7.30E-02	-1.28E-01	1.51E-01	2.22E-01
6900	GSM36869	GSE2034	Breast Cancer	Breast	2.72E-01	9.03E-02	8.45E-02	2.54E-01
6901	GSM36870	GSE2034	Breast Cancer	Breast	4.87E-03	-1.83E-01	4.38E-02	2.86E-01
6902	GSM36871	GSE2034	Breast Cancer	Breast	5.01E-02	1.37E-01	1.16E-02	3.41E-01
6903	GSM36872	GSE2034	Breast Cancer	Breast	3.94E-01	-7.64E-02	6.31E-02	2.69E-01
6904	GSM36873	GSE2034	Breast Cancer	Breast	1.69E-04	-2.33E-01	1.59E-01	-2.19E-01
6905	GSM36874	GSE2034	Breast Cancer	Breast	1.97E-03	-1.97E-01	2.51E-01	-1.90E-01
6906	GSM36875	GSE2034	Breast Cancer	Breast	8.56E-03	1.73E-01	1.34E-04	4.83E-01
6907	GSM36876	GSE2034	Breast Cancer	Breast	1.83E-01	-1.03E-01	1.73E-01	2.14E-01
6908	GSM36877	GSE2034	Breast Cancer	Breast	3.03E-04	-2.25E-01	1.75E-01	-2.13E-01
6909	GSM36878	GSE2034	Breast Cancer	Breast	7.44E-09	-3.42E-01	4.15E-03	-3.78E-01
6910	GSM36879	GSE2034	Breast Cancer	Breast	8.52E-04	-2.10E-01	1.96E-01	2.06E-01
6911	GSM36880	GSE2034	Breast Cancer	Breast	1.74E-03	1.99E-01	2.49E-03	3.96E-01
6912	GSM36881	GSE2034	Breast Cancer	Breast	8.91E-02	-1.23E-01	2.22E-01	1.98E-01
6913	GSM36882	GSE2034	Breast Cancer	Breast	2.88E-01	-8.83E-02	5.09E-01	1.33E-01
6914	GSM36883	GSE2034	Breast Cancer	Breast	3.34E-02	-1.46E-01	2.49E-01	1.90E-01
6915	GSM36884	GSE2034	Breast Cancer	Breast	5.23E-02	-1.36E-01	2.49E-01	1.90E-01
6916	GSM36885	GSE2034	Breast Cancer	Breast	2.42E-01	9.42E-02	1.18E-01	2.36E-01
6917	GSM36886	GSE2034	Breast Cancer	Breast	6.81E-05	2.45E-01	2.87E-05	5.23E-01
6918	GSM36887	GSE2034	Breast Cancer	Breast	5.23E-02	-1.36E-01	2.54E-01	-1.89E-01
6919	GSM36888	GSE2034	Breast Cancer	Breast	1.12E-01	-1.17E-01	8.45E-02	2.54E-01
6920	GSM36889	GSE2034	Breast Cancer	Breast	4.72E-15	4.54E-01	2.73E-08	6.74E-01
6921	GSM36890	GSE2034	Breast Cancer	Breast	2.01E-01	1.00E-01	1.20E-02	3.40E-01
6922	GSM36891	GSE2034	Breast Cancer	Breast	8.10E-03	-1.74E-01	4.58E-01	1.43E-01
6923	GSM36892	GSE2034	Breast Cancer	Breast	1.95E-01	-1.01E-01	1.56E-01	2.20E-01
6924	GSM36893	GSE2034	Breast Cancer	Breast	1.12E-01	-1.17E-01	6.15E-02	2.70E-01
6925	GSM36894	GSE2034	Breast Cancer	Breast	4.34E-03	1.85E-01	6.06E-03	3.65E-01
6926	GSM36895	GSE2034	Breast Cancer	Breast	1.17E-12	-4.15E-01	5.23E-05	-5.07E-01
6927	GSM36896	GSE2034	Breast Cancer	Breast	4.60E-05	-2.50E-01	3.20E-02	-3.00E-01
6928	GSM36897	GSE2034	Breast Cancer	Breast	1.30E-01	1.13E-01	7.27E-02	2.62E-01
6929	GSM36898	GSE2034	Breast Cancer	Breast	4.13E-01	-7.44E-02	5.39E-01	1.27E-01
6930	GSM36899	GSE2034	Breast Cancer	Breast	7.86E-13	-4.18E-01	1.33E-06	-5.94E-01
6931	GSM36900	GSE2034	Breast Cancer	Breast	1.63E-03	-2.00E-01	2.25E-01	1.97E-01
6932	GSM36901	GSE2034	Breast Cancer	Breast	6.81E-05	-2.45E-01	3.99E-01	-1.55E-01
6933	GSM36902	GSE2034	Breast Cancer	Breast	1.17E-12	4.15E-01	7.93E-09	6.98E-01
6934	GSM36903	GSE2034	Breast Cancer	Breast	3.04E-03	1.90E-01	7.13E-04	4.35E-01
6935	GSM36904	GSE2034	Breast Cancer	Breast	1.65E-06	-2.89E-01	9.10E-02	-2.50E-01
6936	GSM36905	GSE2034	Breast Cancer	Breast	2.44E-24	5.83E-01	4.25E-14	8.97E-01
6937	GSM36906	GSE2034	Breast Cancer	Breast	5.89E-01	5.75E-02	1.15E-01	2.37E-01
6938	GSM36907	GSE2034	Breast Cancer	Breast	3.56E-01	-8.04E-02	2.22E-01	1.98E-01
6939	GSM36908	GSE2034	Breast Cancer	Breast	3.74E-04	-2.22E-01	5.38E-02	-2.76E-01
6940	GSM36909	GSE2034	Breast Cancer	Breast	3.21E-22	5.57E-01	4.63E-12	8.25E-01
6941	GSM36910	GSE2034	Breast Cancer	Breast	1.89E-01	1.02E-01	1.16E-02	3.41E-01
6942	GSM36911	GSE2034	Breast Cancer	Breast	2.27E-04	-2.29E-01	4.63E-02	-2.83E-01
6943	GSM36912	GSE2034	Breast Cancer	Breast	3.13E-01	8.53E-02	4.63E-02	2.83E-01
6944	GSM36913	GSE2034	Breast Cancer	Breast	7.92E-02	1.26E-01	5.31E-02	2.77E-01
6945	GSM36914	GSE2034	Breast Cancer	Breast	1.49E-01	1.09E-01	1.44E-02	3.33E-01
6946	GSM36915	GSE2034	Breast Cancer	Breast	1.65E-01	-1.06E-01	1.77E-01	2.13E-01
6947	GSM36916	GSE2034	Breast Cancer	Breast	6.81E-05	2.45E-01	1.28E-04	4.84E-01
6948	GSM36917	GSE2034	Breast Cancer	Breast	8.10E-03	-1.74E-01	3.16E-02	3.00E-01
6949	GSM36918	GSE2034	Breast Cancer	Breast	1.08E-04	2.39E-01	1.81E-04	4.74E-01
6950	GSM36919	GSE2034	Breast Cancer	Breast	2.28E-01	-9.62E-02	8.45E-02	2.54E-01
6951	GSM36920	GSE2034	Breast Cancer	Breast	2.14E-01	9.82E-02	5.24E-02	2.77E-01

6952	GSM36921	GSE2034	Breast Cancer	Breast	6.28E-06	-2.74E-01	2.64E-02	-3.08E-01
6953	GSM36922	GSE2034	Breast Cancer	Breast	1.24E-07	-3.15E-01	1.17E-03	-4.20E-01
6954	GSM36923	GSE2034	Breast Cancer	Breast	7.01E-02	1.29E-01	3.16E-02	3.00E-01
6955	GSM36924	GSE2034	Breast Cancer	Breast	3.62E-05	-2.53E-01	1.22E-01	-2.34E-01
6956	GSM36925	GSE2034	Breast Cancer	Breast	3.84E-02	-1.43E-01	3.22E-01	-1.72E-01
6957	GSM36926	GSE2034	Breast Cancer	Breast	3.67E-02	-1.44E-01	1.32E-01	2.30E-01
6958	GSM36927	GSE2034	Breast Cancer	Breast	9.63E-02	-1.21E-01	1.86E-01	2.10E-01
6959	GSM36928	GSE2034	Breast Cancer	Breast	1.16E-04	-2.38E-01	1.04E-01	-2.43E-01
6960	GSM36929	GSE2034	Breast Cancer	Breast	3.05E-02	-1.48E-01	3.16E-01	-1.73E-01
6961	GSM36930	GSE2034	Breast Cancer	Breast	4.55E-14	-4.38E-01	2.79E-05	-5.23E-01
6962	GSM36931	GSE2034	Breast Cancer	Breast	7.36E-05	2.44E-01	5.28E-04	4.44E-01
6963	GSM36932	GSE2034	Breast Cancer	Breast	6.77E-08	3.21E-01	1.07E-05	5.47E-01
6964	GSM36933	GSE2034	Breast Cancer	Breast	2.42E-01	-9.42E-02	1.98E-01	2.06E-01
6965	GSM36934	GSE2034	Breast Cancer	Breast	2.01E-01	-1.00E-01	3.81E-01	1.59E-01
6966	GSM36935	GSE2034	Breast Cancer	Breast	4.79E-02	1.38E-01	1.77E-02	3.24E-01
6967	GSM36936	GSE2034	Breast Cancer	Breast	4.44E-07	3.03E-01	9.37E-05	4.92E-01
6968	GSM36937	GSE2034	Breast Cancer	Breast	2.80E-01	-8.93E-02	2.22E-01	1.98E-01
6969	GSM36938	GSE2034	Breast Cancer	Breast	1.34E-01	1.12E-01	6.15E-02	2.70E-01
6970	GSM36939	GSE2034	Breast Cancer	Breast	4.98E-05	-2.49E-01	4.70E-02	-2.83E-01
6971	GSM36940	GSE2034	Breast Cancer	Breast	4.13E-01	7.44E-02	6.15E-02	2.70E-01
6972	GSM36941	GSE2034	Breast Cancer	Breast	2.18E-02	-1.55E-01	1.34E-01	2.29E-01
6973	GSM36942	GSE2034	Breast Cancer	Breast	4.60E-03	1.84E-01	2.49E-03	3.96E-01
6974	GSM36943	GSE2034	Breast Cancer	Breast	1.53E-03	-2.01E-01	2.58E-01	-1.88E-01
6975	GSM36944	GSE2034	Breast Cancer	Breast	1.74E-05	2.62E-01	1.04E-05	5.47E-01
6976	GSM36945	GSE2034	Breast Cancer	Breast	3.86E-03	-1.87E-01	7.46E-02	-2.60E-01
6977	GSM36946	GSE2034	Breast Cancer	Breast	9.73E-04	-2.08E-01	1.04E-01	-2.43E-01
6978	GSM36947	GSE2034	Breast Cancer	Breast	4.87E-12	-4.04E-01	1.34E-04	-4.83E-01
6979	GSM36948	GSE2034	Breast Cancer	Breast	2.91E-12	-4.08E-01	9.85E-03	-3.47E-01
6980	GSM36949	GSE2034	Breast Cancer	Breast	9.04E-03	1.72E-01	5.31E-02	2.77E-01
6981	GSM36950	GSE2034	Breast Cancer	Breast	7.92E-02	-1.26E-01	2.49E-01	1.90E-01
6982	GSM36951	GSE2034	Breast Cancer	Breast	1.57E-04	-2.34E-01	4.63E-02	-2.83E-01
6983	GSM36952	GSE2034	Breast Cancer	Breast	4.87E-03	1.83E-01	5.52E-04	4.43E-01
6984	GSM36953	GSE2034	Breast Cancer	Breast	5.79E-03	-1.80E-01	2.02E-01	2.04E-01
6985	GSM36954	GSE2034	Breast Cancer	Breast	1.27E-08	-3.37E-01	9.36E-04	-4.27E-01
6986	GSM36955	GSE2034	Breast Cancer	Breast	9.04E-03	1.72E-01	7.61E-03	3.57E-01
6987	GSM36956	GSE2034	Breast Cancer	Breast	4.03E-01	7.54E-02	4.45E-02	2.85E-01
6988	GSM36957	GSE2034	Breast Cancer	Breast	8.24E-02	-1.25E-01	1.51E-01	2.22E-01
6989	GSM36958	GSE2034	Breast Cancer	Breast	2.53E-03	1.93E-01	7.61E-03	3.57E-01
6990	GSM36959	GSE2034	Breast Cancer	Breast	3.32E-07	3.06E-01	9.60E-05	4.91E-01
6991	GSM36960	GSE2034	Breast Cancer	Breast	8.10E-03	1.74E-01	1.00E-02	3.47E-01
6992	GSM36961	GSE2034	Breast Cancer	Breast	5.23E-02	-1.36E-01	1.51E-01	2.22E-01
6993	GSM36962	GSE2034	Breast Cancer	Breast	1.89E-01	1.02E-01	6.39E-02	2.68E-01
6994	GSM36963	GSE2034	Breast Cancer	Breast	1.12E-01	-1.17E-01	3.11E-01	1.75E-01
6995	GSM36964	GSE2034	Breast Cancer	Breast	2.52E-02	-1.52E-01	2.80E-01	1.82E-01
6996	GSM36965	GSE2034	Breast Cancer	Breast	4.87E-03	1.83E-01	8.79E-04	4.29E-01
6997	GSM36966	GSE2034	Breast Cancer	Breast	1.53E-02	1.62E-01	9.36E-03	3.49E-01
6998	GSM36967	GSE2034	Breast Cancer	Breast	5.57E-01	6.05E-02	7.75E-03	3.56E-01
6999	GSM36968	GSE2034	Breast Cancer	Breast	2.47E-09	-3.52E-01	1.56E-02	-3.30E-01
7000	GSM36969	GSE2034	Breast Cancer	Breast	7.60E-02	1.27E-01	1.73E-01	2.14E-01
7001	GSM36970	GSE2034	Breast Cancer	Breast	8.24E-02	-1.25E-01	3.11E-01	1.75E-01
7002	GSM36971	GSE2034	Breast Cancer	Breast	3.13E-01	-8.53E-02	1.73E-01	2.14E-01
7003	GSM36972	GSE2034	Breast Cancer	Breast	2.01E-01	1.00E-01	1.44E-02	3.33E-01
7004	GSM36973	GSE2034	Breast Cancer	Breast	1.60E-05	-2.63E-01	7.88E-03	-3.56E-01
7005	GSM36974	GSE2034	Breast Cancer	Breast	9.08E-14	-4.34E-01	1.93E-06	-5.86E-01
7006	GSM36975	GSE2034	Breast Cancer	Breast	1.57E-04	-2.34E-01	5.45E-02	-2.76E-01
7007	GSM36976	GSE2034	Breast Cancer	Breast	1.39E-01	1.11E-01	3.07E-02	3.02E-01
7008	GSM36977	GSE2034	Breast Cancer	Breast	3.21E-01	8.43E-02	9.90E-02	2.46E-01
7009	GSM36978	GSE2034	Breast Cancer	Breast	4.87E-03	1.83E-01	5.24E-02	2.77E-01
7010	GSM36979	GSE2034	Breast Cancer	Breast	4.20E-02	-1.41E-01	3.46E-01	1.66E-01
7011	GSM36980	GSE2034	Breast Cancer	Breast	7.36E-05	-2.44E-01	1.98E-01	-2.06E-01
7012	GSM36981	GSE2034	Breast Cancer	Breast	1.12E-01	1.17E-01	1.73E-01	2.14E-01
7013	GSM36982	GSE2034	Breast Cancer	Breast	1.44E-01	1.10E-01	1.96E-01	2.06E-01
7014	GSM36983	GSE2034	Breast Cancer	Breast	3.63E-03	1.88E-01	7.27E-02	2.62E-01
7015	GSM36984	GSE2034	Breast Cancer	Breast	1.30E-21	-5.49E-01	7.93E-09	-6.98E-01
7016	GSM36985	GSE2034	Breast Cancer	Breast	1.89E-05	-2.61E-01	2.68E-02	-3.07E-01
7017	GSM36986	GSE2034	Breast Cancer	Breast	6.48E-03	-1.78E-01	2.56E-01	-1.89E-01
7018	GSM36987	GSE2034	Breast Cancer	Breast	8.52E-04	-2.10E-01	7.55E-02	-2.60E-01
7019	GSM36988	GSE2034	Breast Cancer	Breast	7.60E-02	-1.27E-01	1.14E-01	2.38E-01
7020	GSM36989	GSE2034	Breast Cancer	Breast	1.46E-02	-1.63E-01	3.81E-01	1.59E-01
7021	GSM36990	GSE2034	Breast Cancer	Breast	9.65E-06	-2.69E-01	6.28E-03	-3.64E-01
7022	GSM36991	GSE2034	Breast Cancer	Breast	2.14E-01	9.82E-02	2.57E-02	3.09E-01
7023	GSM36992	GSE2034	Breast Cancer	Breast	1.74E-03	-1.99E-01	2.93E-01	-1.79E-01
7024	GSM36993	GSE2034	Breast Cancer	Breast	3.39E-06	2.81E-01	9.83E-05	4.91E-01
7025	GSM36994	GSE2034	Breast Cancer	Breast	1.83E-01	1.03E-01	1.19E-01	2.36E-01

7026	GSM36995	GSE2034	Breast Cancer	Breast	1.79E-02	-1.59E-01	2.36E-01	-1.94E-01
7027	GSM36996	GSE2034	Breast Cancer	Breast	6.73E-02	1.30E-01	6.28E-03	3.64E-01
7028	GSM36997	GSE2034	Breast Cancer	Breast	8.10E-03	-1.74E-01	2.02E-01	-2.04E-01
7029	GSM36998	GSE2034	Breast Cancer	Breast	2.14E-01	-9.82E-02	3.46E-01	1.66E-01
7030	GSM36999	GSE2034	Breast Cancer	Breast	3.71E-06	-2.80E-01	2.68E-02	-3.07E-01
7031	GSM37000	GSE2034	Breast Cancer	Breast	4.98E-05	2.49E-01	5.28E-04	4.44E-01
7032	GSM37001	GSE2034	Breast Cancer	Breast	5.46E-02	-1.35E-01	4.20E-01	1.50E-01
7033	GSM37002	GSE2034	Breast Cancer	Breast	2.57E-01	9.23E-02	1.21E-01	2.35E-01
7034	GSM37003	GSE2034	Breast Cancer	Breast	5.69E-02	-1.34E-01	3.96E-01	-1.56E-01
7035	GSM37004	GSE2034	Breast Cancer	Breast	1.28E-15	-4.63E-01	1.37E-06	-5.94E-01
7036	GSM37005	GSE2034	Breast Cancer	Breast	4.39E-02	-1.40E-01	4.32E-01	1.48E-01
7037	GSM37006	GSE2034	Breast Cancer	Breast	6.46E-02	-1.31E-01	3.49E-01	1.66E-01
7038	GSM37007	GSE2034	Breast Cancer	Breast	2.23E-05	-2.59E-01	1.03E-01	-2.44E-01
7039	GSM37008	GSE2034	Breast Cancer	Breast	7.25E-03	1.76E-01	1.16E-02	3.41E-01
7040	GSM37009	GSE2034	Breast Cancer	Breast	3.86E-03	1.87E-01	2.64E-02	3.08E-01
7041	GSM37010	GSE2034	Breast Cancer	Breast	4.98E-08	-3.24E-01	1.51E-02	-3.31E-01
7042	GSM37011	GSE2034	Breast Cancer	Breast	1.89E-05	-2.61E-01	2.24E-02	-3.15E-01
7043	GSM37012	GSE2034	Breast Cancer	Breast	2.21E-09	-3.53E-01	6.62E-03	-3.62E-01
7044	GSM37013	GSE2034	Breast Cancer	Breast	3.84E-02	-1.43E-01	2.88E-01	-1.80E-01
7045	GSM37014	GSE2034	Breast Cancer	Breast	4.87E-03	-1.83E-01	3.81E-01	1.59E-01
7046	GSM37015	GSE2034	Breast Cancer	Breast	2.21E-01	-9.72E-02	1.14E-01	2.38E-01
7047	GSM37016	GSE2034	Breast Cancer	Breast	9.11E-04	-2.09E-01	1.56E-01	-2.20E-01
7048	GSM37017	GSE2034	Breast Cancer	Breast	6.73E-02	-1.30E-01	1.73E-01	2.14E-01
7049	GSM37018	GSE2034	Breast Cancer	Breast	1.18E-02	-1.67E-01	2.83E-01	1.82E-01
7050	GSM37019	GSE2034	Breast Cancer	Breast	2.80E-01	-8.93E-02	3.11E-02	3.01E-01
7051	GSM37020	GSE2034	Breast Cancer	Breast	2.42E-05	-2.58E-01	2.31E-01	-1.96E-01
7052	GSM37021	GSE2034	Breast Cancer	Breast	2.62E-04	-2.27E-01	2.02E-01	-2.04E-01
7053	GSM37022	GSE2034	Breast Cancer	Breast	3.50E-02	1.45E-01	1.44E-02	3.33E-01
7054	GSM37023	GSE2034	Breast Cancer	Breast	2.14E-01	-9.82E-02	2.56E-01	-1.89E-01
7055	GSM37024	GSE2034	Breast Cancer	Breast	5.57E-01	-6.05E-02	3.84E-01	1.58E-01
7056	GSM37025	GSE2034	Breast Cancer	Breast	9.63E-02	-1.21E-01	2.49E-01	1.90E-01
7057	GSM37026	GSE2034	Breast Cancer	Breast	3.45E-09	-3.49E-01	6.98E-04	-4.36E-01
7058	GSM37027	GSE2034	Breast Cancer	Breast	6.11E-01	5.56E-02	2.66E-01	1.86E-01
7059	GSM37028	GSE2034	Breast Cancer	Breast	6.97E-04	-2.13E-01	3.30E-01	-1.70E-01
7060	GSM37029	GSE2034	Breast Cancer	Breast	5.91E-07	-3.00E-01	1.46E-02	-3.32E-01
7061	GSM37030	GSE2034	Breast Cancer	Breast	3.31E-12	-4.07E-01	9.36E-04	-4.27E-01
7062	GSM37031	GSE2034	Breast Cancer	Breast	1.53E-02	-1.62E-01	1.51E-01	-2.22E-01
7063	GSM37032	GSE2034	Breast Cancer	Breast	1.89E-01	1.02E-01	1.15E-01	2.37E-01
7064	GSM37033	GSE2034	Breast Cancer	Breast	5.69E-02	-1.34E-01	2.54E-01	-1.89E-01
7065	GSM37034	GSE2034	Breast Cancer	Breast	7.30E-02	1.28E-01	1.86E-02	3.23E-01
7066	GSM37035	GSE2034	Breast Cancer	Breast	4.83E-06	-2.77E-01	3.25E-02	-2.99E-01
7067	GSM37036	GSE2034	Breast Cancer	Breast	3.94E-01	7.64E-02	1.96E-01	2.06E-01
7068	GSM37037	GSE2034	Breast Cancer	Breast	2.82E-04	-2.26E-01	6.39E-02	-2.68E-01
7069	GSM37038	GSE2034	Breast Cancer	Breast	1.24E-05	-2.66E-01	7.88E-03	-3.56E-01
7070	GSM37039	GSE2034	Breast Cancer	Breast	1.21E-01	-1.15E-01	3.81E-01	1.59E-01
7071	GSM37040	GSE2034	Breast Cancer	Breast	3.25E-04	2.24E-01	1.22E-03	4.18E-01
7072	GSM37041	GSE2034	Breast Cancer	Breast	3.04E-01	8.63E-02	1.73E-01	2.14E-01
7073	GSM37042	GSE2034	Breast Cancer	Breast	4.05E-06	-2.79E-01	3.19E-01	-1.73E-01
7074	GSM37043	GSE2034	Breast Cancer	Breast	1.00E-04	2.40E-01	7.13E-04	4.35E-01
7075	GSM37044	GSE2034	Breast Cancer	Breast	1.52E-12	-4.13E-01	7.28E-04	-4.34E-01
7076	GSM37045	GSE2034	Breast Cancer	Breast	1.16E-01	-1.16E-01	3.14E-01	1.74E-01
7077	GSM37046	GSE2034	Breast Cancer	Breast	1.04E-03	2.07E-01	6.17E-03	3.64E-01
7078	GSM37047	GSE2034	Breast Cancer	Breast	5.46E-02	1.35E-01	7.36E-02	2.61E-01
7079	GSM37048	GSE2034	Breast Cancer	Breast	1.83E-01	-1.03E-01	3.49E-01	1.66E-01
7080	GSM37049	GSE2034	Breast Cancer	Breast	1.25E-02	1.66E-01	2.61E-02	3.09E-01
7081	GSM37050	GSE2034	Breast Cancer	Breast	1.28E-33	6.88E-01	8.12E-16	9.52E-01
7082	GSM37051	GSE2034	Breast Cancer	Breast	4.31E-09	3.47E-01	3.98E-05	5.14E-01
7083	GSM37052	GSE2034	Breast Cancer	Breast	4.31E-04	2.20E-01	2.98E-04	4.60E-01
7084	GSM37053	GSE2034	Breast Cancer	Breast	1.60E-01	1.07E-01	2.14E-02	3.17E-01
7085	GSM37054	GSE2034	Breast Cancer	Breast	4.87E-12	-4.04E-01	6.28E-03	-3.64E-01
7086	GSM37055	GSE2034	Breast Cancer	Breast	6.33E-10	-3.64E-01	2.59E-03	-3.94E-01
7087	GSM37056	GSE2034	Breast Cancer	Breast	1.83E-01	1.03E-01	1.26E-02	3.38E-01
7088	GSM37057	GSE2034	Breast Cancer	Breast	5.46E-02	-1.35E-01	3.46E-01	1.66E-01
7089	GSM37058	GSE2034	Breast Cancer	Breast	1.08E-04	2.39E-01	3.98E-04	4.52E-01
7090	GSM37059	GSE2034	Breast Cancer	Breast	5.79E-03	1.80E-01	6.28E-03	3.64E-01
7091	GSM37060	GSE2034	Breast Cancer	Breast	3.05E-02	-1.48E-01	5.00E-01	1.35E-01
7092	GSM37061	GSE2034	Breast Cancer	Breast	2.24E-03	-1.95E-01	6.48E-02	-2.67E-01
7093	GSM37062	GSE2034	Breast Cancer	Breast	2.77E-02	-1.50E-01	3.11E-01	1.75E-01
7094	GSM79114	GSE3494	Breast Cancer	Breast	1.26E-03	2.04E-01	6.83E-04	4.36E-01
7095	GSM79115	GSE3494	Breast Cancer	Breast	2.14E-01	9.82E-02	3.20E-02	3.00E-01
7096	GSM79116	GSE3494	Breast Cancer	Breast	1.65E-06	-2.89E-01	9.17E-04	-4.27E-01
7097	GSM79117	GSE3494	Breast Cancer	Breast	3.13E-01	-8.53E-02	1.32E-01	2.30E-01
7098	GSM79118	GSE3494	Breast Cancer	Breast	3.84E-01	7.74E-02	1.75E-01	2.13E-01
7099	GSM79119	GSE3494	Breast Cancer	Breast	3.34E-02	-1.46E-01	3.24E-01	-1.71E-01

7100	GSM79120	GSE3494	Breast Cancer	Breast	1.25E-01	-1.14E-01	2.22E-01	1.98E-01
7101	GSM79121	GSE3494	Breast Cancer	Breast	1.75E-08	-3.34E-01	2.05E-05	-5.31E-01
7102	GSM79122	GSE3494	Breast Cancer	Breast	2.91E-02	1.49E-01	2.40E-03	3.97E-01
7103	GSM79123	GSE3494	Breast Cancer	Breast	3.45E-09	-3.49E-01	9.60E-05	-4.91E-01
7104	GSM79124	GSE3494	Breast Cancer	Breast	1.35E-05	-2.65E-01	2.24E-02	-3.15E-01
7105	GSM79125	GSE3494	Breast Cancer	Breast	6.77E-08	-3.21E-01	5.07E-03	-3.71E-01
7106	GSM79126	GSE3494	Breast Cancer	Breast	1.24E-05	-2.66E-01	1.57E-01	-2.20E-01
7107	GSM79127	GSE3494	Breast Cancer	Breast	6.81E-05	-2.45E-01	4.51E-02	-2.84E-01
7108	GSM79128	GSE3494	Breast Cancer	Breast	4.42E-06	-2.78E-01	4.45E-02	-2.85E-01
7109	GSM79129	GSE3494	Breast Cancer	Breast	3.71E-06	-2.80E-01	1.80E-02	-3.24E-01
7110	GSM79130	GSE3494	Breast Cancer	Breast	3.50E-02	1.45E-01	3.08E-03	3.89E-01
7111	GSM79131	GSE3494	Breast Cancer	Breast	6.86E-03	-1.77E-01	2.83E-01	-1.82E-01
7112	GSM79132	GSE3494	Breast Cancer	Breast	8.10E-03	-1.74E-01	1.51E-01	2.22E-01
7113	GSM79133	GSE3494	Breast Cancer	Breast	6.77E-08	-3.21E-01	4.07E-03	-3.79E-01
7114	GSM79134	GSE3494	Breast Cancer	Breast	4.20E-02	-1.41E-01	2.02E-01	-2.04E-01
7115	GSM79135	GSE3494	Breast Cancer	Breast	1.74E-03	-1.99E-01	1.96E-01	2.06E-01
7116	GSM79136	GSE3494	Breast Cancer	Breast	2.88E-01	8.83E-02	6.15E-02	2.70E-01
7117	GSM79137	GSE3494	Breast Cancer	Breast	1.65E-01	1.06E-01	8.56E-02	2.53E-01
7118	GSM79138	GSE3494	Breast Cancer	Breast	1.39E-01	1.11E-01	6.31E-02	2.69E-01
7119	GSM79139	GSE3494	Breast Cancer	Breast	3.63E-03	-1.88E-01	8.56E-02	-2.53E-01
7120	GSM79140	GSE3494	Breast Cancer	Breast	1.37E-13	-4.31E-01	7.53E-06	-5.55E-01
7121	GSM79141	GSE3494	Breast Cancer	Breast	1.85E-07	3.12E-01	2.79E-05	5.23E-01
7122	GSM79142	GSE3494	Breast Cancer	Breast	2.23E-05	2.59E-01	2.98E-04	4.60E-01
7123	GSM79143	GSE3494	Breast Cancer	Breast	1.16E-04	-2.38E-01	4.26E-01	-1.49E-01
7124	GSM79144	GSE3494	Breast Cancer	Breast	8.10E-03	-1.74E-01	3.84E-01	-1.58E-01
7125	GSM79145	GSE3494	Breast Cancer	Breast	1.06E-02	1.69E-01	1.75E-02	3.25E-01
7126	GSM79146	GSE3494	Breast Cancer	Breast	2.01E-01	-1.00E-01	3.87E-01	1.57E-01
7127	GSM79147	GSE3494	Breast Cancer	Breast	1.61E-02	1.61E-01	7.75E-03	3.56E-01
7128	GSM79148	GSE3494	Breast Cancer	Breast	1.44E-03	-2.02E-01	1.32E-01	2.30E-01
7129	GSM79149	GSE3494	Breast Cancer	Breast	4.60E-05	-2.50E-01	6.39E-02	-2.68E-01
7130	GSM79150	GSE3494	Breast Cancer	Breast	7.92E-02	1.26E-01	2.00E-01	2.05E-01
7131	GSM79151	GSE3494	Breast Cancer	Breast	3.86E-03	1.87E-01	5.40E-04	4.43E-01
7132	GSM79152	GSE3494	Breast Cancer	Breast	1.01E-02	-1.70E-01	6.23E-01	-1.11E-01
7133	GSM79153	GSE3494	Breast Cancer	Breast	3.56E-01	-8.04E-02	2.25E-01	1.97E-01
7134	GSM79154	GSE3494	Breast Cancer	Breast	1.04E-03	2.07E-01	7.61E-03	3.57E-01
7135	GSM79155	GSE3494	Breast Cancer	Breast	1.97E-12	-4.11E-01	4.16E-04	-4.51E-01
7136	GSM79156	GSE3494	Breast Cancer	Breast	9.23E-09	3.40E-01	2.64E-06	5.79E-01
7137	GSM79157	GSE3494	Breast Cancer	Breast	6.73E-02	1.30E-01	7.88E-03	3.56E-01
7138	GSM79158	GSE3494	Breast Cancer	Breast	3.20E-11	3.89E-01	1.07E-05	5.47E-01
7139	GSM79159	GSE3494	Breast Cancer	Breast	4.83E-06	-2.77E-01	3.86E-02	-2.91E-01
7140	GSM79160	GSE3494	Breast Cancer	Breast	1.01E-02	1.70E-01	4.45E-02	2.85E-01
7141	GSM79161	GSE3494	Breast Cancer	Breast	5.69E-02	1.34E-01	3.98E-04	4.52E-01
7142	GSM79162	GSE3494	Breast Cancer	Breast	1.21E-01	-1.15E-01	2.25E-01	1.97E-01
7143	GSM79163	GSE3494	Breast Cancer	Breast	2.42E-05	-2.58E-01	5.38E-02	-2.76E-01
7144	GSM79164	GSE3494	Breast Cancer	Breast	4.31E-04	-2.20E-01	1.01E-01	-2.44E-01
7145	GSM79165	GSE3494	Breast Cancer	Breast	3.04E-01	8.63E-02	5.53E-02	2.75E-01
7146	GSM79166	GSE3494	Breast Cancer	Breast	9.73E-04	2.08E-01	1.46E-02	3.32E-01
7147	GSM79167	GSE3494	Breast Cancer	Breast	4.25E-05	-2.51E-01	1.50E-03	-4.12E-01
7148	GSM79168	GSE3494	Breast Cancer	Breast	2.64E-02	1.51E-01	2.61E-02	3.09E-01
7149	GSM79169	GSE3494	Breast Cancer	Breast	1.85E-03	1.98E-01	8.79E-04	4.29E-01
7150	GSM79170	GSE3494	Breast Cancer	Breast	6.73E-02	1.30E-01	3.20E-02	3.00E-01
7151	GSM79171	GSE3494	Breast Cancer	Breast	3.62E-11	-3.88E-01	3.88E-05	-5.15E-01
7152	GSM79172	GSE3494	Breast Cancer	Breast	1.61E-02	-1.61E-01	2.51E-01	-1.90E-01
7153	GSM79173	GSE3494	Breast Cancer	Breast	5.75E-06	2.75E-01	9.83E-05	4.91E-01
7154	GSM79174	GSE3494	Breast Cancer	Breast	1.54E-01	1.08E-01	9.90E-02	2.46E-01
7155	GSM79175	GSE3494	Breast Cancer	Breast	2.86E-03	1.91E-01	1.44E-02	3.33E-01
7156	GSM79176	GSE3494	Breast Cancer	Breast	3.21E-01	8.43E-02	9.90E-02	2.46E-01
7157	GSM79177	GSE3494	Breast Cancer	Breast	2.49E-01	9.33E-02	1.96E-01	2.06E-01
7158	GSM79178	GSE3494	Breast Cancer	Breast	1.46E-04	2.35E-01	1.15E-03	4.20E-01
7159	GSM79179	GSE3494	Breast Cancer	Breast	3.19E-02	-1.47E-01	3.14E-01	-1.74E-01
7160	GSM79180	GSE3494	Breast Cancer	Breast	2.18E-02	1.55E-01	5.24E-02	2.77E-01
7161	GSM79181	GSE3494	Breast Cancer	Breast	1.89E-05	-2.61E-01	1.00E-01	-2.45E-01
7162	GSM79182	GSE3494	Breast Cancer	Breast	1.42E-08	3.36E-01	9.60E-05	4.91E-01
7163	GSM79183	GSE3494	Breast Cancer	Breast	4.59E-02	1.39E-01	3.35E-02	2.98E-01
7164	GSM79184	GSE3494	Breast Cancer	Breast	1.04E-01	1.19E-01	2.12E-01	2.01E-01
7165	GSM79185	GSE3494	Breast Cancer	Breast	5.69E-02	-1.34E-01	3.19E-01	-1.73E-01
7166	GSM79186	GSE3494	Breast Cancer	Breast	9.04E-03	1.72E-01	3.08E-03	3.89E-01
7167	GSM79187	GSE3494	Breast Cancer	Breast	1.65E-01	1.06E-01	1.14E-01	2.38E-01
7168	GSM79188	GSE3494	Breast Cancer	Breast	1.52E-12	-4.13E-01	9.76E-04	-4.25E-01
7169	GSM79189	GSE3494	Breast Cancer	Breast	3.74E-04	-2.22E-01	7.46E-02	-2.60E-01
7170	GSM79190	GSE3494	Breast Cancer	Breast	2.25E-07	3.10E-01	6.13E-08	6.58E-01
7171	GSM79191	GSE3494	Breast Cancer	Breast	8.94E-10	-3.61E-01	1.46E-02	-3.32E-01
7172	GSM79192	GSE3494	Breast Cancer	Breast	1.18E-02	-1.67E-01	1.98E-01	-2.06E-01
7173	GSM79193	GSE3494	Breast Cancer	Breast	1.85E-07	-3.12E-01	2.54E-03	-3.95E-01

7174	GSM79194	GSE3494	Breast Cancer	Breast	1.54E-01	1.08E-01	6.39E-02	2.68E-01
7175	GSM79195	GSE3494	Breast Cancer	Breast	8.62E-17	4.81E-01	1.30E-11	8.09E-01
7176	GSM79196	GSE3494	Breast Cancer	Breast	1.16E-04	-2.38E-01	1.37E-01	-2.28E-01
7177	GSM79197	GSE3494	Breast Cancer	Breast	2.62E-05	-2.57E-01	1.84E-01	-2.10E-01
7178	GSM79198	GSE3494	Breast Cancer	Breast	1.21E-01	-1.15E-01	1.52E-01	2.22E-01
7179	GSM79199	GSE3494	Breast Cancer	Breast	6.08E-04	-2.15E-01	1.79E-01	-2.12E-01
7180	GSM79200	GSE3494	Breast Cancer	Breast	7.92E-02	1.26E-01	6.15E-02	2.70E-01
7181	GSM79201	GSE3494	Breast Cancer	Breast	5.52E-08	3.23E-01	7.95E-06	5.54E-01
7182	GSM79202	GSE3494	Breast Cancer	Breast	6.35E-17	4.83E-01	1.78E-08	6.83E-01
7183	GSM79203	GSE3494	Breast Cancer	Breast	4.39E-02	1.40E-01	8.45E-02	2.54E-01
7184	GSM79204	GSE3494	Breast Cancer	Breast	8.59E-05	-2.42E-01	1.46E-02	-3.32E-01
7185	GSM79205	GSE3494	Breast Cancer	Breast	4.10E-11	3.87E-01	3.02E-07	6.26E-01
7186	GSM79206	GSE3494	Breast Cancer	Breast	5.39E-05	2.48E-01	1.47E-03	4.13E-01
7187	GSM79207	GSE3494	Breast Cancer	Breast	3.66E-01	-7.94E-02	4.39E-01	1.47E-01
7188	GSM79208	GSE3494	Breast Cancer	Breast	4.34E-03	1.85E-01	7.75E-03	3.56E-01
7189	GSM79209	GSE3494	Breast Cancer	Breast	3.21E-01	8.43E-02	3.84E-01	1.58E-01
7190	GSM79210	GSE3494	Breast Cancer	Breast	2.77E-02	-1.50E-01	3.52E-01	-1.65E-01
7191	GSM79211	GSE3494	Breast Cancer	Breast	2.60E-25	-5.95E-01	4.82E-12	-8.25E-01
7192	GSM79212	GSE3494	Breast Cancer	Breast	1.35E-05	-2.65E-01	1.77E-02	-3.24E-01
7193	GSM79213	GSE3494	Breast Cancer	Breast	4.98E-05	-2.49E-01	4.45E-02	-2.85E-01
7194	GSM79214	GSE3494	Breast Cancer	Breast	2.25E-07	-3.10E-01	6.28E-03	-3.64E-01
7195	GSM79215	GSE3494	Breast Cancer	Breast	2.52E-02	1.52E-01	1.32E-01	2.30E-01
7196	GSM79216	GSE3494	Breast Cancer	Breast	4.01E-02	1.42E-01	6.06E-03	3.65E-01
7197	GSM79217	GSE3494	Breast Cancer	Breast	9.27E-05	2.41E-01	7.01E-05	5.00E-01
7198	GSM79218	GSE3494	Breast Cancer	Breast	2.84E-06	-2.83E-01	3.25E-03	-3.87E-01
7199	GSM79219	GSE3494	Breast Cancer	Breast	6.97E-04	-2.13E-01	4.57E-02	-2.84E-01
7200	GSM79220	GSE3494	Breast Cancer	Breast	3.34E-05	2.54E-01	1.98E-03	4.03E-01
7201	GSM79221	GSE3494	Breast Cancer	Breast	3.50E-02	1.45E-01	3.16E-02	3.00E-01
7202	GSM79222	GSE3494	Breast Cancer	Breast	1.39E-01	1.11E-01	2.68E-02	3.07E-01
7203	GSM79223	GSE3494	Breast Cancer	Breast	4.31E-09	-3.47E-01	2.01E-03	-4.03E-01
7204	GSM79224	GSE3494	Breast Cancer	Breast	1.44E-01	-1.10E-01	3.11E-01	1.75E-01
7205	GSM79225	GSE3494	Breast Cancer	Breast	5.94E-02	-1.33E-01	3.85E-03	3.81E-01
7206	GSM79226	GSE3494	Breast Cancer	Breast	2.11E-04	-2.30E-01	2.22E-01	1.98E-01
7207	GSM79227	GSE3494	Breast Cancer	Breast	1.89E-01	1.02E-01	4.38E-02	2.86E-01
7208	GSM79228	GSE3494	Breast Cancer	Breast	2.96E-01	-8.73E-02	1.14E-01	2.38E-01
7209	GSM79229	GSE3494	Breast Cancer	Breast	2.21E-11	-3.92E-01	3.19E-04	-4.58E-01
7210	GSM79230	GSE3494	Breast Cancer	Breast	1.08E-10	-3.79E-01	4.07E-04	-4.51E-01
7211	GSM79231	GSE3494	Breast Cancer	Breast	6.08E-04	-2.15E-01	1.80E-01	-2.11E-01
7212	GSM79232	GSE3494	Breast Cancer	Breast	3.21E-01	-8.43E-02	2.80E-01	1.82E-01
7213	GSM79233	GSE3494	Breast Cancer	Breast	2.96E-01	8.73E-02	1.32E-01	2.30E-01
7214	GSM79234	GSE3494	Breast Cancer	Breast	1.70E-02	1.60E-01	1.75E-02	3.25E-01
7215	GSM79235	GSE3494	Breast Cancer	Breast	5.04E-01	6.55E-02	1.82E-01	2.11E-01
7216	GSM79236	GSE3494	Breast Cancer	Breast	7.30E-02	-1.28E-01	2.83E-01	1.82E-01
7217	GSM79237	GSE3494	Breast Cancer	Breast	4.59E-02	-1.39E-01	2.04E-01	2.04E-01
7218	GSM79238	GSE3494	Breast Cancer	Breast	5.23E-02	-1.36E-01	1.73E-01	2.14E-01
7219	GSM79239	GSE3494	Breast Cancer	Breast	3.04E-03	1.90E-01	7.28E-04	4.34E-01
7220	GSM79240	GSE3494	Breast Cancer	Breast	7.45E-04	2.12E-01	9.36E-03	3.49E-01
7221	GSM79241	GSE3494	Breast Cancer	Breast	8.91E-02	1.23E-01	4.38E-02	2.86E-01
7222	GSM79242	GSE3494	Breast Cancer	Breast	1.46E-02	1.63E-01	2.33E-04	4.67E-01
7223	GSM79243	GSE3494	Breast Cancer	Breast	8.61E-07	-2.96E-01	3.11E-02	-3.01E-01
7224	GSM79244	GSE3494	Breast Cancer	Breast	2.86E-03	-1.91E-01	1.34E-01	-2.29E-01
7225	GSM79245	GSE3494	Breast Cancer	Breast	1.34E-01	-1.12E-01	2.22E-01	1.98E-01
7226	GSM79246	GSE3494	Breast Cancer	Breast	7.84E-07	-2.97E-01	3.16E-02	-3.00E-01
7227	GSM79247	GSE3494	Breast Cancer	Breast	1.30E-01	1.13E-01	2.22E-01	1.98E-01
7228	GSM79248	GSE3494	Breast Cancer	Breast	2.52E-02	-1.52E-01	2.49E-01	1.90E-01
7229	GSM79249	GSE3494	Breast Cancer	Breast	6.33E-10	-3.64E-01	5.52E-04	-4.43E-01
7230	GSM79250	GSE3494	Breast Cancer	Breast	5.23E-02	1.36E-01	4.38E-02	2.86E-01
7231	GSM79251	GSE3494	Breast Cancer	Breast	9.54E-03	-1.71E-01	2.83E-01	1.82E-01
7232	GSM79252	GSE3494	Breast Cancer	Breast	2.64E-02	1.51E-01	8.45E-02	2.54E-01
7233	GSM79253	GSE3494	Breast Cancer	Breast	4.60E-03	1.84E-01	1.15E-03	4.20E-01
7234	GSM79254	GSE3494	Breast Cancer	Breast	4.59E-02	1.39E-01	3.07E-02	3.02E-01
7235	GSM79255	GSE3494	Breast Cancer	Breast	8.59E-05	2.42E-01	1.90E-03	4.04E-01
7236	GSM79256	GSE3494	Breast Cancer	Breast	1.04E-01	-1.19E-01	3.70E-02	2.93E-01
7237	GSM79257	GSE3494	Breast Cancer	Breast	4.01E-02	-1.42E-01	2.51E-01	-1.90E-01
7238	GSM79258	GSE3494	Breast Cancer	Breast	3.29E-08	-3.28E-01	2.68E-02	-3.07E-01
7239	GSM79259	GSE3494	Breast Cancer	Breast	2.57E-01	-9.23E-02	9.90E-02	2.46E-01
7240	GSM79260	GSE3494	Breast Cancer	Breast	6.08E-04	2.15E-01	3.08E-03	3.89E-01
7241	GSM79261	GSE3494	Breast Cancer	Breast	5.91E-07	3.00E-01	4.34E-07	6.18E-01
7242	GSM79262	GSE3494	Breast Cancer	Breast	6.81E-05	2.45E-01	7.53E-06	5.55E-01
7243	GSM79263	GSE3494	Breast Cancer	Breast	2.70E-13	4.26E-01	1.96E-07	6.35E-01
7244	GSM79264	GSE3494	Breast Cancer	Breast	1.04E-01	1.19E-01	4.76E-02	2.82E-01
7245	GSM79265	GSE3494	Breast Cancer	Breast	3.25E-04	-2.24E-01	2.24E-02	-3.15E-01
7246	GSM79266	GSE3494	Breast Cancer	Breast	6.28E-06	-2.74E-01	6.65E-02	-2.66E-01
7247	GSM79267	GSE3494	Breast Cancer	Breast	1.98E-06	-2.87E-01	1.77E-02	-3.24E-01

7248	GSM79268	GSE3494	Breast Cancer	Breast	6.32E-01	5.36E-02	2.80E-01	1.82E-01
7249	GSM79269	GSE3494	Breast Cancer	Breast	5.91E-11	-3.84E-01	1.73E-04	-4.76E-01
7250	GSM79270	GSE3494	Breast Cancer	Breast	2.18E-02	1.55E-01	4.83E-02	2.81E-01
7251	GSM79271	GSE3494	Breast Cancer	Breast	3.21E-01	8.43E-02	2.38E-01	1.94E-01
7252	GSM79272	GSE3494	Breast Cancer	Breast	1.60E-05	2.63E-01	6.98E-04	4.36E-01
7253	GSM79273	GSE3494	Breast Cancer	Breast	2.91E-02	-1.49E-01	3.11E-01	-1.75E-01
7254	GSM79274	GSE3494	Breast Cancer	Breast	2.23E-05	-2.59E-01	7.46E-02	-2.60E-01
7255	GSM79275	GSE3494	Breast Cancer	Breast	4.53E-01	7.04E-02	2.51E-01	1.90E-01
7256	GSM79276	GSE3494	Breast Cancer	Breast	5.75E-06	-2.75E-01	2.06E-01	-2.03E-01
7257	GSM79277	GSE3494	Breast Cancer	Breast	2.73E-07	-3.08E-01	1.24E-02	-3.38E-01
7258	GSM79278	GSE3494	Breast Cancer	Breast	1.30E-01	-1.13E-01	3.81E-01	1.59E-01
7259	GSM79279	GSE3494	Breast Cancer	Breast	2.01E-01	-1.00E-01	3.86E-02	2.91E-01
7260	GSM79280	GSE3494	Breast Cancer	Breast	1.25E-01	-1.14E-01	2.54E-01	1.89E-01
7261	GSM79281	GSE3494	Breast Cancer	Breast	8.24E-02	1.25E-01	4.38E-02	2.86E-01
7262	GSM79282	GSE3494	Breast Cancer	Breast	1.34E-01	1.12E-01	1.32E-01	2.30E-01
7263	GSM79283	GSE3494	Breast Cancer	Breast	1.83E-01	1.03E-01	1.22E-01	2.34E-01
7264	GSM79284	GSE3494	Breast Cancer	Breast	8.56E-03	-1.73E-01	1.37E-01	-2.28E-01
7265	GSM79285	GSE3494	Breast Cancer	Breast	1.25E-02	-1.66E-01	1.75E-01	-2.13E-01
7266	GSM79286	GSE3494	Breast Cancer	Breast	4.79E-02	-1.38E-01	3.93E-01	-1.56E-01
7267	GSM79287	GSE3494	Breast Cancer	Breast	2.64E-02	1.51E-01	2.57E-02	3.09E-01
7268	GSM79288	GSE3494	Breast Cancer	Breast	7.01E-02	-1.29E-01	3.63E-01	1.63E-01
7269	GSM79289	GSE3494	Breast Cancer	Breast	4.59E-02	-1.39E-01	3.46E-01	1.66E-01
7270	GSM79290	GSE3494	Breast Cancer	Breast	2.64E-02	1.51E-01	2.11E-02	3.17E-01
7271	GSM79291	GSE3494	Breast Cancer	Breast	1.60E-01	-1.07E-01	3.81E-01	1.59E-01
7272	GSM79292	GSE3494	Breast Cancer	Breast	7.25E-03	-1.76E-01	5.19E-01	1.31E-01
7273	GSM79293	GSE3494	Breast Cancer	Breast	5.23E-02	-1.36E-01	2.49E-01	1.90E-01
7274	GSM79294	GSE3494	Breast Cancer	Breast	2.84E-06	-2.83E-01	1.06E-01	-2.42E-01
7275	GSM79295	GSE3494	Breast Cancer	Breast	2.91E-02	-1.49E-01	1.51E-01	2.22E-01
7276	GSM79296	GSE3494	Breast Cancer	Breast	6.86E-03	-1.77E-01	2.85E-01	-1.81E-01
7277	GSM79297	GSE3494	Breast Cancer	Breast	1.46E-02	-1.63E-01	3.84E-01	-1.58E-01
7278	GSM79298	GSE3494	Breast Cancer	Breast	5.23E-02	-1.36E-01	2.80E-01	1.82E-01
7279	GSM79299	GSE3494	Breast Cancer	Breast	3.38E-01	-8.23E-02	3.92E-02	2.91E-01
7280	GSM79300	GSE3494	Breast Cancer	Breast	5.23E-02	1.36E-01	2.57E-02	3.09E-01
7281	GSM79301	GSE3494	Breast Cancer	Breast	4.88E-07	-3.02E-01	5.25E-03	-3.70E-01
7282	GSM79302	GSE3494	Breast Cancer	Breast	3.34E-02	1.46E-01	2.20E-02	3.16E-01
7283	GSM79303	GSE3494	Breast Cancer	Breast	1.39E-01	-1.11E-01	3.84E-01	1.58E-01
7284	GSM79304	GSE3494	Breast Cancer	Breast	4.87E-12	-4.04E-01	2.54E-03	-3.95E-01
7285	GSM79305	GSE3494	Breast Cancer	Breast	5.69E-02	-1.34E-01	4.29E-01	1.49E-01
7286	GSM79306	GSE3494	Breast Cancer	Breast	8.57E-02	1.24E-01	1.51E-01	2.22E-01
7287	GSM79307	GSE3494	Breast Cancer	Breast	1.39E-01	-1.11E-01	1.73E-01	2.14E-01
7288	GSM79308	GSE3494	Breast Cancer	Breast	9.23E-09	3.40E-01	7.53E-06	5.55E-01
7289	GSM79309	GSE3494	Breast Cancer	Breast	3.10E-06	2.82E-01	5.23E-05	5.07E-01
7290	GSM79310	GSE3494	Breast Cancer	Breast	7.92E-02	-1.26E-01	5.43E-01	-1.26E-01
7291	GSM79311	GSE3494	Breast Cancer	Breast	2.11E-04	2.30E-01	3.11E-02	3.01E-01
7292	GSM79312	GSE3494	Breast Cancer	Breast	2.07E-02	1.56E-01	2.57E-02	3.09E-01
7293	GSM79313	GSE3494	Breast Cancer	Breast	3.34E-02	1.46E-01	1.56E-03	4.11E-01
7294	GSM79314	GSE3494	Breast Cancer	Breast	1.04E-01	1.19E-01	2.81E-02	3.05E-01
7295	GSM79315	GSE3494	Breast Cancer	Breast	3.34E-02	1.46E-01	3.70E-02	2.93E-01
7296	GSM79316	GSE3494	Breast Cancer	Breast	6.13E-03	1.79E-01	1.47E-03	4.13E-01
7297	GSM79317	GSE3494	Breast Cancer	Breast	2.42E-01	-9.42E-02	3.46E-01	1.66E-01
7298	GSM79318	GSE3494	Breast Cancer	Breast	7.30E-02	-1.28E-01	3.11E-01	1.75E-01
7299	GSM79319	GSE3494	Breast Cancer	Breast	5.79E-03	1.80E-01	7.55E-02	2.60E-01
7300	GSM79320	GSE3494	Breast Cancer	Breast	5.64E-10	-3.65E-01	1.46E-05	-5.39E-01
7301	GSM79321	GSE3494	Breast Cancer	Breast	1.37E-06	-2.91E-01	1.02E-02	-3.46E-01
7302	GSM79322	GSE3494	Breast Cancer	Breast	6.73E-02	1.30E-01	2.24E-02	3.15E-01
7303	GSM79323	GSE3494	Breast Cancer	Breast	2.44E-04	-2.28E-01	1.34E-01	-2.29E-01
7304	GSM79324	GSE3494	Breast Cancer	Breast	3.93E-05	2.52E-01	1.73E-04	4.76E-01
7305	GSM79325	GSE3494	Breast Cancer	Breast	3.05E-02	1.48E-01	2.57E-02	3.09E-01
7306	GSM79326	GSE3494	Breast Cancer	Breast	2.84E-06	-2.83E-01	1.46E-02	-3.32E-01
7307	GSM79327	GSE3494	Breast Cancer	Breast	9.04E-03	1.72E-01	4.07E-03	3.79E-01
7308	GSM79328	GSE3494	Breast Cancer	Breast	3.74E-04	-2.22E-01	1.80E-01	-2.11E-01
7309	GSM79329	GSE3494	Breast Cancer	Breast	5.94E-02	1.33E-01	3.11E-02	3.01E-01
7310	GSM79330	GSE3494	Breast Cancer	Breast	1.17E-12	-4.15E-01	7.28E-04	-4.34E-01
7311	GSM79331	GSE3494	Breast Cancer	Breast	3.75E-01	7.84E-02	3.11E-01	1.75E-01
7312	GSM79332	GSE3494	Breast Cancer	Breast	4.81E-09	-3.46E-01	9.83E-05	-4.91E-01
7313	GSM79333	GSE3494	Breast Cancer	Breast	3.05E-02	-1.48E-01	4.61E-01	-1.42E-01
7314	GSM79334	GSE3494	Breast Cancer	Breast	4.95E-04	-2.18E-01	5.53E-02	-2.75E-01
7315	GSM79335	GSE3494	Breast Cancer	Breast	3.38E-01	-8.23E-02	8.45E-02	2.54E-01
7316	GSM79336	GSE3494	Breast Cancer	Breast	2.10E-03	-1.96E-01	8.56E-02	-2.53E-01
7317	GSM79337	GSE3494	Breast Cancer	Breast	2.01E-01	1.00E-01	6.31E-02	2.69E-01
7318	GSM79338	GSE3494	Breast Cancer	Breast	1.54E-01	1.08E-01	5.45E-02	2.76E-01
7319	GSM79339	GSE3494	Breast Cancer	Breast	2.52E-02	1.52E-01	5.68E-02	2.74E-01
7320	GSM79340	GSE3494	Breast Cancer	Breast	1.08E-04	-2.39E-01	6.23E-02	-2.69E-01
7321	GSM79341	GSE3494	Breast Cancer	Breast	7.45E-04	-2.12E-01	3.55E-01	-1.64E-01

7322	GSM79342	GSE3494	Breast Cancer	Breast	4.23E-01	7.34E-02	2.51E-01	1.90E-01
7323	GSM79343	GSE3494	Breast Cancer	Breast	1.80E-13	-4.29E-01	4.08E-05	-5.14E-01
7324	GSM79344	GSE3494	Breast Cancer	Breast	1.89E-01	1.02E-01	3.08E-03	3.89E-01
7325	GSM79345	GSE3494	Breast Cancer	Breast	7.14E-07	2.98E-01	7.01E-05	5.00E-01
7326	GSM79346	GSE3494	Breast Cancer	Breast	7.25E-03	1.76E-01	1.77E-02	3.24E-01
7327	GSM79347	GSE3494	Breast Cancer	Breast	1.37E-06	-2.91E-01	1.21E-01	-2.35E-01
7328	GSM79348	GSE3494	Breast Cancer	Breast	1.00E-01	-1.20E-01	6.30E-01	1.10E-01
7329	GSM79349	GSE3494	Breast Cancer	Breast	1.74E-05	-2.62E-01	7.36E-02	-2.61E-01
7330	GSM79350	GSE3494	Breast Cancer	Breast	1.74E-03	1.99E-01	2.45E-03	3.96E-01
7331	GSM79351	GSE3494	Breast Cancer	Breast	1.85E-03	1.98E-01	9.68E-03	3.48E-01
7332	GSM79352	GSE3494	Breast Cancer	Breast	3.94E-01	-7.64E-02	1.37E-01	2.28E-01
7333	GSM79353	GSE3494	Breast Cancer	Breast	2.21E-09	-3.53E-01	4.15E-03	-3.78E-01
7334	GSM79354	GSE3494	Breast Cancer	Breast	7.11E-10	-3.63E-01	7.88E-03	-3.56E-01
7335	GSM79355	GSE3494	Breast Cancer	Breast	5.05E-38	-7.33E-01	6.23E-13	-8.57E-01
7336	GSM79356	GSE3494	Breast Cancer	Breast	5.46E-02	-1.35E-01	5.89E-01	-1.17E-01
7337	GSM79357	GSE3494	Breast Cancer	Breast	4.25E-05	2.51E-01	8.98E-04	4.28E-01
7338	GSM79358	GSE3494	Breast Cancer	Breast	4.43E-01	7.14E-02	1.38E-01	2.27E-01
7339	GSM79359	GSE3494	Breast Cancer	Breast	4.63E-01	6.94E-02	8.45E-02	2.54E-01
7340	GSM79360	GSE3494	Breast Cancer	Breast	4.03E-01	-7.54E-02	4.45E-02	2.85E-01
7341	GSM79361	GSE3494	Breast Cancer	Breast	2.64E-01	9.13E-02	4.45E-02	2.85E-01
7342	GSM79362	GSE3494	Breast Cancer	Breast	4.87E-12	-4.04E-01	1.20E-03	-4.19E-01
7343	GSM79363	GSE3494	Breast Cancer	Breast	2.37E-06	-2.85E-01	1.49E-02	-3.31E-01
7344	GSM79364	GSE3494	Breast Cancer	Breast	5.16E-03	1.82E-01	3.86E-02	2.91E-01
7345	GSM85473	GSE3744	Breast Cancer	Breast	1.61E-02	-1.61E-01	2.00E-01	2.05E-01
7346	GSM85474	GSE3744	Breast Cancer	Breast	2.96E-01	8.73E-02	3.11E-02	3.01E-01
7347	GSM85475	GSE3744	Breast Cancer	Breast	5.23E-02	-1.36E-01	3.14E-01	1.74E-01
7348	GSM85476	GSE3744	Breast Cancer	Breast	1.12E-02	1.68E-01	9.36E-03	3.49E-01
7349	GSM85477	GSE3744	Breast Cancer	Breast	8.24E-02	1.25E-01	3.07E-02	3.02E-01
7350	GSM85478	GSE3744	Breast Cancer	Breast	7.92E-02	-1.26E-01	2.70E-01	1.85E-01
7351	GSM85479	GSE3744	Breast Cancer	Breast	2.08E-01	-9.92E-02	2.29E-01	1.96E-01
7352	GSM85480	GSE3744	Breast Cancer	Breast	4.20E-02	1.41E-01	4.98E-03	3.72E-01
7353	GSM85481	GSE3744	Breast Cancer	Breast	1.06E-02	-1.69E-01	3.16E-01	-1.73E-01
7354	GSM85482	GSE3744	Breast Cancer	Breast	5.27E-06	-2.76E-01	1.83E-02	-3.23E-01
7355	GSM85483	GSE3744	Breast Cancer	Breast	1.14E-05	2.67E-01	1.34E-04	4.83E-01
7356	GSM85484	GSE3744	Breast Cancer	Breast	1.08E-01	1.18E-01	5.24E-02	2.77E-01
7357	GSM85485	GSE3744	Breast Cancer	Breast	1.24E-05	-2.66E-01	1.49E-02	-3.31E-01
7358	GSM85486	GSE3744	Breast Cancer	Breast	1.60E-05	2.63E-01	1.31E-04	4.83E-01
7359	GSM85487	GSE3744	Breast Cancer	Breast	1.88E-02	-1.58E-01	6.53E-01	-1.05E-01
7360	GSM85488	GSE3744	Breast Cancer	Breast	2.96E-01	-8.73E-02	1.52E-01	2.22E-01
7361	GSM85489	GSE3744	Breast Cancer	Breast	9.54E-03	-1.71E-01	1.34E-01	-2.29E-01
7362	GSM85490	GSE3744	Breast Cancer	Breast	5.23E-02	-1.36E-01	4.45E-01	1.45E-01
7363	GSM85491	GSE3744	Breast Cancer	Breast	3.19E-02	1.47E-01	1.46E-02	3.32E-01
7364	GSM85492	GSE3744	Breast Cancer	Breast	3.34E-02	-1.46E-01	4.42E-01	1.46E-01
7365	GSM85493	GSE3744	Breast Cancer	Breast	1.18E-03	-2.05E-01	8.45E-02	-2.54E-01
7366	GSM85494	GSE3744	Breast Cancer	Breast	6.08E-04	-2.15E-01	8.66E-02	-2.53E-01
7367	GSM85495	GSE3744	Breast Cancer	Breast	4.03E-01	-7.54E-02	3.87E-01	1.57E-01
7368	GSM85496	GSE3744	Breast Cancer	Breast	2.84E-06	-2.83E-01	3.92E-03	-3.80E-01
7369	GSM85497	GSE3744	Breast Cancer	Breast	3.66E-01	-7.94E-02	8.56E-02	2.53E-01
7370	GSM85498	GSE3744	Breast Cancer	Breast	2.82E-04	-2.26E-01	2.61E-01	-1.87E-01
7371	GSM85499	GSE3744	Breast Cancer	Breast	1.18E-03	-2.05E-01	7.55E-02	-2.60E-01
7372	GSM85500	GSE3744	Breast Cancer	Breast	1.37E-07	-3.14E-01	9.52E-03	-3.49E-01
7373	GSM85501	GSE3744	Breast Cancer	Breast	1.35E-03	-2.03E-01	1.57E-01	-2.20E-01
7374	GSM85502	GSE3744	Breast Cancer	Breast	3.34E-05	-2.54E-01	3.92E-02	-2.91E-01
7375	GSM85503	GSE3744	Breast Cancer	Breast	4.05E-08	3.26E-01	2.05E-05	5.31E-01
7376	GSM85504	GSE3744	Breast Cancer	Breast	5.27E-06	-2.76E-01	3.81E-02	-2.92E-01
7377	GSM85505	GSE3744	Breast Cancer	Breast	3.19E-02	-1.47E-01	4.58E-01	1.43E-01
7378	GSM85506	GSE3744	Breast Cancer	Breast	5.23E-02	1.36E-01	3.11E-02	3.01E-01
7379	GSM85507	GSE3744	Breast Cancer	Breast	3.25E-04	-2.24E-01	3.11E-02	-3.01E-01
7380	GSM85508	GSE3744	Breast Cancer	Breast	2.35E-01	-9.52E-02	4.51E-02	2.84E-01
7381	GSM85509	GSE3744	Breast Cancer	Breast	2.53E-03	-1.93E-01	8.77E-02	-2.52E-01
7382	GSM85510	GSE3744	Breast Cancer	Breast	2.42E-01	-9.42E-02	4.58E-01	1.43E-01
7383	GSM85511	GSE3744	Breast Cancer	Breast	1.35E-03	-2.03E-01	2.83E-01	-1.82E-01
7384	GSM85512	GSE3744	Breast Cancer	Breast	1.74E-05	-2.62E-01	4.03E-02	-2.90E-01
7385	GSM85513	GSE3744	Breast Cancer	Breast	3.04E-01	8.63E-02	4.38E-02	2.86E-01
7386	GSM85514	GSE3744	Breast Cancer	Breast	1.89E-01	1.02E-01	4.38E-02	2.86E-01
7387	GSM85515	GSE3744	Breast Cancer	Breast	5.01E-02	1.37E-01	4.98E-03	3.72E-01
7388	GSM85516	GSE3744	Breast Cancer	Breast	1.49E-01	-1.09E-01	2.83E-01	1.82E-01
7389	GSM85517	GSE3744	Breast Cancer	Breast	8.59E-05	2.42E-01	1.47E-03	4.13E-01
7390	GSM85518	GSE3744	Breast Cancer	Breast	3.42E-03	1.88E-01	2.59E-03	3.94E-01
7391	GSM85519	GSE3744	Breast Cancer	Breast	3.71E-06	2.80E-01	2.00E-05	5.31E-01
7392	GSM119938	GSE4922	Breast Cancer	Breast	3.38E-01	8.23E-02	1.14E-01	2.38E-01
7393	GSM119939	GSE4922	Breast Cancer	Breast	1.54E-01	-1.08E-01	2.80E-01	1.82E-01
7394	GSM119940	GSE4922	Breast Cancer	Breast	3.93E-05	-2.52E-01	4.70E-02	-2.83E-01
7395	GSM119941	GSE4922	Breast Cancer	Breast	3.10E-06	-2.82E-01	6.62E-03	-3.62E-01

7396	GSM119942	GSE4922	Breast Cancer	Breast	3.67E-02	-1.44E-01	3.11E-02	-3.01E-01
7397	GSM119943	GSE4922	Breast Cancer	Breast	4.39E-02	-1.40E-01	2.61E-01	-1.87E-01
7398	GSM119944	GSE4922	Breast Cancer	Breast	1.38E-02	-1.64E-01	4.61E-01	1.42E-01
7399	GSM119945	GSE4922	Breast Cancer	Breast	3.50E-02	-1.45E-01	2.80E-01	1.82E-01
7400	GSM119946	GSE4922	Breast Cancer	Breast	6.84E-06	2.73E-01	3.98E-04	4.52E-01
7401	GSM119947	GSE4922	Breast Cancer	Breast	1.35E-05	-2.65E-01	6.39E-02	-2.68E-01
7402	GSM119948	GSE4922	Breast Cancer	Breast	1.26E-03	-2.04E-01	1.96E-01	2.06E-01
7403	GSM119949	GSE4922	Breast Cancer	Breast	2.01E-01	1.00E-01	1.96E-01	2.06E-01
7404	GSM119950	GSE4922	Breast Cancer	Breast	2.21E-01	-9.72E-02	1.14E-01	2.38E-01
7405	GSM119951	GSE4922	Breast Cancer	Breast	1.12E-01	-1.17E-01	6.23E-02	2.69E-01
7406	GSM119952	GSE4922	Breast Cancer	Breast	4.02E-04	-2.21E-01	3.58E-01	-1.64E-01
7407	GSM119953	GSE4922	Breast Cancer	Breast	1.95E-11	-3.93E-01	7.28E-04	-4.34E-01
7408	GSM119954	GSE4922	Breast Cancer	Breast	1.60E-01	1.07E-01	6.15E-02	2.70E-01
7409	GSM119955	GSE4922	Breast Cancer	Breast	6.77E-08	-3.21E-01	6.39E-03	-3.63E-01
7410	GSM119956	GSE4922	Breast Cancer	Breast	1.97E-03	1.97E-01	9.60E-05	4.91E-01
7411	GSM119957	GSE4922	Breast Cancer	Breast	3.65E-08	-3.27E-01	3.38E-03	-3.85E-01
7412	GSM119958	GSE4922	Breast Cancer	Breast	2.35E-01	9.52E-02	7.55E-02	2.60E-01
7413	GSM119959	GSE4922	Breast Cancer	Breast	2.10E-27	6.20E-01	5.98E-13	8.57E-01
7414	GSM119960	GSE4922	Breast Cancer	Breast	1.18E-03	2.05E-01	1.90E-03	4.04E-01
7415	GSM119961	GSE4922	Breast Cancer	Breast	4.62E-04	-2.19E-01	2.51E-01	-1.90E-01
7416	GSM119962	GSE4922	Breast Cancer	Breast	1.53E-03	-2.01E-01	2.51E-01	-1.90E-01
7417	GSM119963	GSE4922	Breast Cancer	Breast	1.63E-03	2.00E-01	1.94E-03	4.04E-01
7418	GSM119964	GSE4922	Breast Cancer	Breast	2.62E-04	-2.27E-01	8.56E-02	-2.53E-01
7419	GSM119965	GSE4922	Breast Cancer	Breast	1.60E-01	-1.07E-01	5.83E-01	1.19E-01
7420	GSM119966	GSE4922	Breast Cancer	Breast	1.21E-01	1.15E-01	7.65E-02	2.59E-01
7421	GSM119967	GSE4922	Breast Cancer	Breast	3.38E-01	-8.23E-02	2.95E-01	1.78E-01
7422	GSM119968	GSE4922	Breast Cancer	Breast	3.30E-01	8.33E-02	1.96E-01	2.06E-01
7423	GSM119969	GSE4922	Breast Cancer	Breast	2.02E-20	5.33E-01	3.31E-11	7.94E-01
7424	GSM119970	GSE4922	Breast Cancer	Breast	3.62E-05	2.53E-01	2.28E-04	4.68E-01
7425	GSM119971	GSE4922	Breast Cancer	Breast	1.53E-03	-2.01E-01	1.54E-01	-2.21E-01
7426	GSM119972	GSE4922	Breast Cancer	Breast	1.85E-07	-3.12E-01	3.92E-02	-2.91E-01
7427	GSM119973	GSE4922	Breast Cancer	Breast	2.41E-08	-3.31E-01	1.77E-02	-3.24E-01
7428	GSM119974	GSE4922	Breast Cancer	Breast	9.04E-03	-1.72E-01	3.49E-01	-1.66E-01
7429	GSM119975	GSE4922	Breast Cancer	Breast	2.53E-03	-1.93E-01	3.90E-01	-1.57E-01
7430	GSM119976	GSE4922	Breast Cancer	Breast	1.79E-02	1.59E-01	4.00E-03	3.80E-01
7431	GSM119977	GSE4922	Breast Cancer	Breast	1.25E-01	-1.14E-01	3.90E-01	-1.57E-01
7432	GSM152338	GSE6596	Breast Cancer	Breast	2.23E-05	-2.59E-01	1.18E-02	-3.40E-01
7433	GSM152339	GSE6596	Breast Cancer	Breast	1.39E-01	1.11E-01	1.75E-02	3.25E-01
7434	GSM152340	GSE6596	Breast Cancer	Breast	1.12E-01	1.17E-01	2.57E-02	3.09E-01
7435	GSM152341	GSE6596	Breast Cancer	Breast	3.04E-03	-1.90E-01	3.14E-01	-1.74E-01
7436	GSM152342	GSE6596	Breast Cancer	Breast	4.44E-07	-3.03E-01	6.56E-02	-2.67E-01
7437	GSM152343	GSE6596	Breast Cancer	Breast	4.87E-03	-1.83E-01	2.85E-01	-1.81E-01
7438	GSM152344	GSE6596	Breast Cancer	Breast	1.58E-09	-3.56E-01	6.51E-03	-3.63E-01
7439	GSM152345	GSE6596	Breast Cancer	Breast	2.88E-01	8.83E-02	1.14E-01	2.38E-01
7440	GSM152346	GSE6596	Breast Cancer	Breast	1.39E-01	-1.11E-01	2.49E-01	-1.90E-01
7441	GSM152347	GSE6596	Breast Cancer	Breast	3.67E-02	-1.44E-01	6.15E-02	2.70E-01
7442	GSM152348	GSE6596	Breast Cancer	Breast	3.38E-01	8.23E-02	5.24E-02	2.77E-01
7443	GSM152349	GSE6596	Breast Cancer	Breast	1.49E-01	-1.09E-01	1.54E-01	2.21E-01
7444	GSM152350	GSE6596	Breast Cancer	Breast	2.29E-02	-1.54E-01	4.26E-01	-1.49E-01
7445	GSM152351	GSE6596	Breast Cancer	Breast	1.49E-01	1.09E-01	2.22E-01	1.98E-01
7446	GSM152352	GSE6596	Breast Cancer	Breast	1.26E-04	-2.37E-01	2.88E-01	-1.80E-01
7447	GSM152353	GSE6596	Breast Cancer	Breast	7.36E-05	-2.44E-01	8.56E-02	-2.53E-01
7448	GSM152354	GSE6596	Breast Cancer	Breast	3.71E-06	-2.80E-01	3.05E-04	-4.60E-01
7449	GSM152355	GSE6596	Breast Cancer	Breast	6.19E-02	1.32E-01	7.65E-02	2.59E-01
7450	GSM152356	GSE6596	Breast Cancer	Breast	6.48E-03	1.78E-01	3.11E-02	3.01E-01
7451	GSM152357	GSE6596	Breast Cancer	Breast	6.12E-08	3.22E-01	7.19E-05	4.99E-01
7452	GSM152358	GSE6596	Breast Cancer	Breast	5.01E-02	1.37E-01	1.16E-02	3.41E-01
7453	GSM152359	GSE6596	Breast Cancer	Breast	6.86E-03	-1.77E-01	4.45E-02	-2.85E-01
7454	GSM152360	GSE6596	Breast Cancer	Breast	2.73E-07	3.08E-01	7.53E-06	5.55E-01
7455	GSM152361	GSE6596	Breast Cancer	Breast	1.67E-07	3.13E-01	7.36E-05	4.98E-01
7456	091604_WN405650	ETABM158	Breast Cancer	Breast	4.02E-04	2.21E-01	5.35E-03	3.70E-01
7457	091604_WN405650	ETABM158	Breast Cancer	Breast	1.01E-02	-1.70E-01	3.49E-01	-1.66E-01
7458	091604_WN405650	ETABM158	Breast Cancer	Breast	3.30E-01	8.33E-02	1.14E-01	2.38E-01
7459	091604_WN405650	ETABM158	Breast Cancer	Breast	3.96E-14	-4.39E-01	3.88E-05	-5.15E-01
7460	091604_WN405650	ETABM158	Breast Cancer	Breast	5.01E-02	1.37E-01	4.63E-02	2.83E-01
7461	091604_WN405650	ETABM158	Breast Cancer	Breast	1.95E-08	-3.33E-01	5.25E-03	-3.70E-01
7462	091604_WN405650	ETABM158	Breast Cancer	Breast	4.02E-04	2.21E-01	2.05E-03	4.02E-01
7463	091604_WN405650	ETABM158	Breast Cancer	Breast	3.49E-04	-2.23E-01	5.60E-02	-2.74E-01
7464	091604_WN405650	ETABM158	Breast Cancer	Breast	3.86E-09	-3.48E-01	9.17E-04	-4.27E-01
7465	091604_WN405650	ETABM158	Breast Cancer	Breast	7.30E-02	-1.28E-01	2.22E-01	1.98E-01
7466	091604_WN405650	ETABM158	Breast Cancer	Breast	1.21E-01	1.15E-01	2.11E-02	3.17E-01
7467	091604_WN405650	ETABM158	Breast Cancer	Breast	7.60E-02	-1.27E-01	9.90E-02	2.46E-01
7468	091604_WN405650	ETABM158	Breast Cancer	Breast	5.57E-01	-6.05E-02	3.52E-01	1.65E-01
7469	091604_WN405650	ETABM158	Breast Cancer	Breast	2.42E-01	-9.42E-02	1.79E-01	-2.12E-01

7470	091604_WN405650	ETABM158	Breast Cancer	Breast	5.16E-03	-1.82E-01	2.02E-01	-2.04E-01
7471	091604_WN405650	ETABM158	Breast Cancer	Breast	7.30E-02	-1.28E-01	2.80E-01	1.82E-01
7472	091604_WN405650	ETABM158	Breast Cancer	Breast	4.34E-03	-1.85E-01	2.64E-02	-3.08E-01
7473	091604_WN405650	ETABM158	Breast Cancer	Breast	2.25E-07	3.10E-01	5.17E-04	4.44E-01
7474	091604_WN405650	ETABM158	Breast Cancer	Breast	4.83E-06	2.77E-01	9.37E-05	4.92E-01
7475	091604_WN405650	ETABM158	Breast Cancer	Breast	1.60E-01	-1.07E-01	6.73E-01	1.02E-01
7476	091604_WN405650	ETABM158	Breast Cancer	Breast	2.01E-01	-1.00E-01	3.11E-02	3.01E-01
7477	091604_WN405650	ETABM158	Breast Cancer	Breast	2.84E-05	2.56E-01	1.03E-04	4.90E-01
7478	091604_WN405650	ETABM158	Breast Cancer	Breast	5.79E-03	-1.80E-01	2.31E-01	-1.96E-01
7479	091604_WN405650	ETABM158	Breast Cancer	Breast	5.46E-01	6.15E-02	2.04E-01	2.04E-01
7480	091604_WN405650	ETABM158	Breast Cancer	Breast	1.47E-05	-2.64E-01	1.52E-01	2.22E-01
7481	091604_WN405650	ETABM158	Breast Cancer	Breast	2.44E-04	2.28E-01	4.89E-03	3.73E-01
7482	091604_WN405650	ETABM158	Breast Cancer	Breast	1.14E-08	-3.38E-01	5.36E-05	-5.07E-01
7483	091604_WN405650	ETABM158	Breast Cancer	Breast	5.68E-04	-2.16E-01	7.36E-02	-2.61E-01
7484	091604_WN405650	ETABM158	Breast Cancer	Breast	5.25E-01	-6.35E-02	2.22E-01	1.98E-01
7485	091604_WN405650	ETABM158	Breast Cancer	Breast	1.60E-01	1.07E-01	1.73E-01	2.14E-01
7486	LBL_POP_W40560!	ETABM158	Breast Cancer	Breast	3.23E-03	1.89E-01	6.39E-03	3.63E-01
7487	LBL_POP_W40560!	ETABM158	Breast Cancer	Breast	1.88E-02	-1.58E-01	1.17E-01	2.37E-01
7488	LBL_POP_W40560!	ETABM158	Breast Cancer	Breast	2.18E-02	1.55E-01	6.23E-02	2.69E-01
7489	LBL_POP_W40560!	ETABM158	Breast Cancer	Breast	4.39E-02	1.40E-01	1.83E-02	3.23E-01
7490	LBL_POP_W40560!	ETABM158	Breast Cancer	Breast	2.21E-01	-9.72E-02	4.64E-01	-1.42E-01
7491	LBL_POP_W40560!	ETABM158	Breast Cancer	Breast	7.84E-07	-2.97E-01	8.77E-02	-2.52E-01
7492	LBL_POP_W40560!	ETABM158	Breast Cancer	Breast	3.30E-01	-8.33E-02	3.11E-01	1.75E-01
7493	LBL_POP_W40560!	ETABM158	Breast Cancer	Breast	4.23E-01	-7.34E-02	1.18E-01	2.36E-01
7494	LBL_POP_W40560!	ETABM158	Breast Cancer	Breast	1.21E-01	-1.15E-01	1.75E-01	-2.13E-01
7495	LBL_POP_W40560!	ETABM158	Breast Cancer	Breast	1.00E-01	1.20E-01	2.56E-01	1.89E-01
7496	LBL_POP_W40560!	ETABM158	Breast Cancer	Breast	5.91E-07	-3.00E-01	1.50E-03	-4.12E-01
7497	LBL_POP_W40560!	ETABM158	Breast Cancer	Breast	7.92E-02	-1.26E-01	5.83E-01	1.19E-01
7498	LBL_POP_W40560!	ETABM158	Breast Cancer	Breast	9.63E-02	-1.21E-01	1.73E-01	2.14E-01
7499	LBL_POP_W40560!	ETABM158	Breast Cancer	Breast	3.10E-06	-2.82E-01	9.10E-02	-2.50E-01
7500	LBL_POP_W40560!	ETABM158	Breast Cancer	Breast	5.01E-02	-1.37E-01	4.29E-01	1.49E-01
7501	LBL_POP_W40560!	ETABM158	Breast Cancer	Breast	1.24E-05	-2.66E-01	2.17E-02	-3.16E-01
7502	LBL_POP_W40560!	ETABM158	Breast Cancer	Breast	8.57E-02	-1.24E-01	2.22E-01	1.98E-01
7503	LBL_POP_W40560!	ETABM158	Breast Cancer	Breast	1.25E-02	-1.66E-01	1.52E-01	-2.22E-01
7504	LBL_POP_W40560!	ETABM158	Breast Cancer	Breast	3.50E-02	-1.45E-01	3.16E-01	-1.73E-01
7505	LBL_POP_W40560!	ETABM158	Breast Cancer	Breast	2.29E-02	-1.54E-01	1.79E-01	-2.12E-01
7506	LBL_POP_W40560!	ETABM158	Breast Cancer	Breast	3.09E-09	3.50E-01	4.47E-07	6.18E-01
7507	LBL_POP_W40560!	ETABM158	Breast Cancer	Breast	1.18E-02	1.67E-01	4.00E-03	3.80E-01
7508	LBL_POP_W40560!	ETABM158	Breast Cancer	Breast	1.58E-08	-3.35E-01	5.36E-05	-5.07E-01
7509	LBL_POP_W40560!	ETABM158	Breast Cancer	Breast	8.57E-02	1.24E-01	7.36E-02	2.61E-01
7510	LBL_POP_W40560!	ETABM158	Breast Cancer	Breast	3.38E-01	8.23E-02	2.72E-02	3.07E-01
7511	LBL_POP_W40560!	ETABM158	Breast Cancer	Breast	1.31E-02	-1.65E-01	2.51E-01	-1.90E-01
7512	LBL_POP_W40560!	ETABM158	Breast Cancer	Breast	8.10E-03	-1.74E-01	3.84E-01	-1.58E-01
7513	LBL_POP_W40560!	ETABM158	Breast Cancer	Breast	9.63E-02	-1.21E-01	3.81E-01	1.59E-01
7514	LBL_POP_W40560!	ETABM158	Breast Cancer	Breast	8.24E-02	-1.25E-01	3.81E-01	1.59E-01
7515	LBL_POP_W40560!	ETABM158	Breast Cancer	Breast	2.73E-07	3.08E-01	4.08E-05	5.14E-01
7516	LBL_POP_W40560!	ETABM158	Breast Cancer	Breast	1.46E-02	-1.63E-01	2.27E-01	-1.97E-01
7517	LBL_POP_W40560!	ETABM158	Breast Cancer	Breast	2.21E-01	9.72E-02	2.90E-01	1.80E-01
7518	LBL_POP_W40560!	ETABM158	Breast Cancer	Breast	6.51E-04	2.14E-01	4.07E-04	4.51E-01
7519	LBL_POP_W40560!	ETABM158	Breast Cancer	Breast	2.62E-04	2.27E-01	1.50E-05	5.38E-01
7520	LBL_POP_W40560!	ETABM158	Breast Cancer	Breast	4.39E-02	1.40E-01	1.42E-02	3.33E-01
7521	LBL_POP_W40560!	ETABM158	Breast Cancer	Breast	1.46E-02	1.63E-01	9.36E-03	3.49E-01
7522	LBL_POP_W40560!	ETABM158	Breast Cancer	Breast	1.12E-01	-1.17E-01	3.35E-02	2.98E-01
7523	LBL_POP_W40560!	ETABM158	Breast Cancer	Breast	1.14E-05	-2.67E-01	6.56E-02	-2.67E-01
7524	LBL_POP_W40560!	ETABM158	Breast Cancer	Breast	6.00E-01	5.65E-02	1.96E-01	2.06E-01
7525	LBL_POP_W40560!	ETABM158	Breast Cancer	Breast	5.79E-03	-1.80E-01	2.25E-01	-1.97E-01
7526	LBL_POP_W40560!	ETABM158	Breast Cancer	Breast	3.19E-02	1.47E-01	1.51E-02	3.31E-01
7527	LBL_POP_W40560!	ETABM158	Breast Cancer	Breast	9.46E-07	-2.95E-01	1.54E-02	-3.30E-01
7528	LBL_POP_W40560!	ETABM158	Breast Cancer	Breast	4.94E-01	-6.65E-02	5.69E-01	1.21E-01
7529	LBL_POP_W40560!	ETABM158	Breast Cancer	Breast	3.04E-01	-8.63E-02	4.58E-01	1.43E-01
7530	LBL_POP_W40560!	ETABM158	Breast Cancer	Breast	1.63E-03	2.00E-01	1.90E-03	4.04E-01
7531	LBL_POP_W40560!	ETABM158	Breast Cancer	Breast	6.97E-04	-2.13E-01	1.96E-01	2.06E-01
7532	LBL_POP_W40560!	ETABM158	Breast Cancer	Breast	1.44E-01	1.10E-01	3.11E-02	3.01E-01
7533	LBL_POP_W40560!	ETABM158	Breast Cancer	Breast	2.96E-01	-8.73E-02	2.54E-01	1.89E-01
7534	LBL_POP_W40560!	ETABM158	Breast Cancer	Breast	1.34E-01	1.12E-01	2.11E-02	3.17E-01
7535	LBL_POP_W40560!	ETABM158	Breast Cancer	Breast	1.39E-01	-1.11E-01	8.45E-02	2.54E-01
7536	LBL_POP_W40560!	ETABM158	Breast Cancer	Breast	1.77E-01	-1.04E-01	2.49E-01	1.90E-01
7537	LBL_POP_W40560!	ETABM158	Breast Cancer	Breast	2.14E-01	-9.82E-02	5.39E-01	1.27E-01
7538	LBL_POP_W40560!	ETABM158	Breast Cancer	Breast	2.21E-09	-3.53E-01	1.22E-03	-4.18E-01
7539	LBL_POP_W40560!	ETABM158	Breast Cancer	Breast	1.65E-01	-1.06E-01	2.49E-01	1.90E-01
7540	LBL_POP_W40560!	ETABM158	Breast Cancer	Breast	1.60E-01	-1.07E-01	1.51E-01	2.22E-01
7541	LBL_POP_W40560!	ETABM158	Breast Cancer	Breast	2.23E-05	-2.59E-01	3.25E-02	-2.99E-01
7542	LBL_POP_W40560!	ETABM158	Breast Cancer	Breast	6.28E-06	-2.74E-01	5.38E-02	-2.76E-01
7543	LBL_POP_W40560!	ETABM158	Breast Cancer	Breast	8.91E-02	1.23E-01	4.57E-02	2.84E-01

7544	LBL_POP_W40560!	ETABM158	Breast Cancer	Breast	1.97E-02	1.57E-01	8.66E-02	2.53E-01
7545	LBL_POP_W40560!	ETABM158	Breast Cancer	Breast	7.84E-07	-2.97E-01	1.94E-03	-4.04E-01
7546	LBL_POP_W40560!	ETABM158	Breast Cancer	Breast	3.75E-01	-7.84E-02	3.93E-01	-1.56E-01
7547	LBL_POP_W40560!	ETABM158	Breast Cancer	Breast	5.37E-07	-3.01E-01	1.20E-02	-3.40E-01
7548	LBL_POP_W40560!	ETABM158	Breast Cancer	Breast	2.73E-07	3.08E-01	1.46E-05	5.39E-01
7549	LBL_POP_W40560!	ETABM158	Breast Cancer	Breast	2.25E-07	3.10E-01	1.34E-04	4.83E-01
7550	LBL_POP_W40560!	ETABM158	Breast Cancer	Breast	1.12E-02	-1.68E-01	3.52E-01	-1.65E-01
7551	LBL_POP_W40560!	ETABM158	Breast Cancer	Breast	6.30E-05	-2.46E-01	3.24E-01	-1.71E-01
7552	LBL_POP_W40560!	ETABM158	Breast Cancer	Breast	1.34E-01	1.12E-01	3.76E-02	2.93E-01
7553	LBL_POP_W40560!	ETABM158	Breast Cancer	Breast	9.26E-02	-1.22E-01	3.11E-01	1.75E-01
7554	LBL_POP_W40560!	ETABM158	Breast Cancer	Breast	2.38E-03	1.94E-01	3.13E-03	3.88E-01
7555	LBL_POP_W40560!	ETABM158	Breast Cancer	Breast	1.02E-12	-4.16E-01	5.63E-05	-5.05E-01
7556	LBL_POP_W40560!	ETABM158	Breast Cancer	Breast	4.05E-08	-3.26E-01	3.12E-04	-4.59E-01
7557	LBL_POP_W40560!	ETABM158	Breast Cancer	Breast	1.16E-04	-2.38E-01	5.16E-03	-3.71E-01
7558	LBL_POP_W40560!	ETABM158	Breast Cancer	Breast	6.13E-03	-1.79E-01	3.96E-01	-1.56E-01
7559	LBL_POP_W40560!	ETABM158	Breast Cancer	Breast	1.35E-04	2.36E-01	2.01E-03	4.03E-01
7560	LBL_POP_W40560!	ETABM158	Breast Cancer	Breast	8.91E-02	-1.23E-01	6.16E-01	1.12E-01
7561	LBL_POP_W40560!	ETABM158	Breast Cancer	Breast	6.19E-02	1.32E-01	1.82E-01	2.11E-01
7562	LBL_POP_W40560!	ETABM158	Breast Cancer	Breast	4.42E-06	-2.78E-01	4.03E-02	-2.90E-01
7563	LBL_POP_W40560!	ETABM158	Breast Cancer	Breast	1.39E-01	1.11E-01	5.53E-02	2.75E-01
7564	LBL_POP_W40560!	ETABM158	Breast Cancer	Breast	3.74E-04	2.22E-01	4.25E-04	4.50E-01
7565	LBL_POP_W40560!	ETABM158	Breast Cancer	Breast	2.35E-01	9.52E-02	1.35E-01	2.29E-01
7566	LBL_POP_W40560!	ETABM158	Breast Cancer	Breast	2.38E-03	-1.94E-01	1.03E-01	-2.44E-01
7567	LBL_POP_W40560!	ETABM158	Breast Cancer	Breast	4.73E-01	6.85E-02	2.93E-01	1.79E-01
7568	LBL_POP_W40560!	ETABM158	Breast Cancer	Breast	4.94E-01	6.65E-02	2.49E-01	1.90E-01
7569	LBL_POP_W40560!	ETABM158	Breast Cancer	Breast	1.74E-03	-1.99E-01	1.17E-01	-2.37E-01
7570	LBL_POP5_400615	ETABM158	Breast Cancer	Breast	3.29E-08	-3.28E-01	2.59E-03	-3.94E-01
7571	LBL_POP5_400615	ETABM158	Breast Cancer	Breast	4.98E-08	-3.24E-01	6.62E-03	-3.62E-01
7572	LBL_POP5_400615	ETABM158	Breast Cancer	Breast	2.57E-01	-9.23E-02	2.49E-01	1.90E-01
7573	LBL_POP5_400615	ETABM158	Breast Cancer	Breast	2.96E-01	8.73E-02	1.15E-01	2.37E-01
7574	LBL_POP5_400615	ETABM158	Breast Cancer	Breast	1.95E-01	1.01E-01	6.23E-02	2.69E-01
7575	LBL_POP5_400615	ETABM158	Breast Cancer	Breast	1.17E-12	-4.15E-01	2.33E-04	-4.67E-01
7576	LBL_POP5_400615	ETABM158	Breast Cancer	Breast	6.13E-03	1.79E-01	3.08E-03	3.89E-01
7577	LBL_POP5_400615	ETABM158	Breast Cancer	Breast	5.68E-04	-2.16E-01	8.56E-02	-2.53E-01
7578	LBL_POP5_400615	ETABM158	Breast Cancer	Breast	2.91E-02	-1.49E-01	2.80E-01	1.82E-01
7579	LBL_POP5_400615	ETABM158	Breast Cancer	Breast	2.29E-02	1.54E-01	1.42E-02	3.33E-01
7580	LBL_POP5_400615	ETABM158	Breast Cancer	Breast	6.13E-03	1.79E-01	7.75E-03	3.56E-01
7581	LBL_POP5_400615	ETABM158	Breast Cancer	Breast	2.96E-01	8.73E-02	2.85E-01	1.81E-01
7582	LBL_POP5_400615	ETABM158	Breast Cancer	Breast	1.12E-02	1.68E-01	1.42E-02	3.33E-01
7583	LBL_POP5_400615	ETABM158	Breast Cancer	Breast	4.31E-04	2.20E-01	7.44E-04	4.34E-01
7584	LBL_POP5_400615	ETABM158	Breast Cancer	Breast	3.06E-15	-4.57E-01	1.54E-05	-5.38E-01
7585	LBL_POP5_400615	ETABM158	Breast Cancer	Breast	9.46E-07	-2.95E-01	2.20E-02	-3.16E-01
7586	GSM124994	GSE5460	Breast Cancer	Breast	1.74E-03	-1.99E-01	1.98E-01	-2.06E-01
7587	GSM124995	GSE5460	Breast Cancer	Breast	5.39E-05	2.48E-01	3.08E-03	3.89E-01
7588	GSM124996	GSE5460	Breast Cancer	Breast	7.46E-06	-2.72E-01	9.68E-03	-3.48E-01
7589	GSM124997	GSE5460	Breast Cancer	Breast	8.52E-04	2.10E-01	4.00E-03	3.80E-01
7590	GSM124998	GSE5460	Breast Cancer	Breast	4.31E-04	-2.20E-01	2.00E-01	-2.05E-01
7591	GSM124999	GSE5460	Breast Cancer	Breast	5.64E-10	-3.65E-01	5.25E-03	-3.70E-01
7592	GSM125000	GSE5460	Breast Cancer	Breast	3.93E-05	2.52E-01	5.28E-04	4.44E-01
7593	GSM125001	GSE5460	Breast Cancer	Breast	1.29E-14	-4.47E-01	3.90E-06	-5.70E-01
7594	GSM125002	GSE5460	Breast Cancer	Breast	2.57E-01	9.23E-02	9.90E-02	2.46E-01
7595	GSM125003	GSE5460	Breast Cancer	Breast	7.01E-02	-1.29E-01	2.83E-01	1.82E-01
7596	GSM125004	GSE5460	Breast Cancer	Breast	9.26E-02	1.22E-01	1.18E-01	2.36E-01
7597	GSM125005	GSE5460	Breast Cancer	Breast	4.87E-03	-1.83E-01	1.80E-01	-2.11E-01
7598	GSM125006	GSE5460	Breast Cancer	Breast	6.49E-07	2.99E-01	5.76E-04	4.41E-01
7599	GSM125007	GSE5460	Breast Cancer	Breast	6.86E-03	-1.77E-01	3.30E-01	-1.70E-01
7600	GSM125008	GSE5460	Breast Cancer	Breast	8.52E-04	-2.10E-01	2.02E-01	-2.04E-01
7601	GSM125009	GSE5460	Breast Cancer	Breast	3.42E-03	1.88E-01	5.16E-03	3.71E-01
7602	GSM125010	GSE5460	Breast Cancer	Breast	9.26E-02	1.22E-01	6.23E-02	2.69E-01
7603	GSM125011	GSE5460	Breast Cancer	Breast	2.88E-01	-8.83E-02	1.96E-01	2.06E-01
7604	GSM125012	GSE5460	Breast Cancer	Breast	1.65E-06	2.89E-01	7.01E-05	5.00E-01
7605	GSM125013	GSE5460	Breast Cancer	Breast	2.91E-02	-1.49E-01	2.25E-01	1.97E-01
7606	GSM125014	GSE5460	Breast Cancer	Breast	7.44E-09	3.42E-01	3.88E-05	5.15E-01
7607	GSM125015	GSE5460	Breast Cancer	Breast	2.70E-13	-4.26E-01	2.87E-05	-5.23E-01
7608	GSM125016	GSE5460	Breast Cancer	Breast	1.46E-02	-1.63E-01	1.75E-01	-2.13E-01
7609	GSM125017	GSE5460	Breast Cancer	Breast	2.28E-01	9.62E-02	4.38E-02	2.86E-01
7610	GSM125018	GSE5460	Breast Cancer	Breast	3.38E-01	8.23E-02	2.22E-01	1.98E-01
7611	GSM125019	GSE5460	Breast Cancer	Breast	2.44E-04	2.28E-01	3.32E-03	3.86E-01
7612	GSM125020	GSE5460	Breast Cancer	Breast	7.17E-01	4.56E-02	3.16E-01	1.73E-01
7613	GSM125021	GSE5460	Breast Cancer	Breast	1.35E-05	-2.65E-01	1.35E-01	-2.29E-01
7614	GSM125022	GSE5460	Breast Cancer	Breast	1.25E-01	-1.14E-01	3.11E-02	3.01E-01
7615	GSM125023	GSE5460	Breast Cancer	Breast	4.20E-02	-1.41E-01	4.11E-01	1.52E-01
7616	GSM125024	GSE5460	Breast Cancer	Breast	1.39E-01	-1.11E-01	3.58E-01	1.64E-01
7617	GSM125025	GSE5460	Breast Cancer	Breast	3.34E-02	-1.46E-01	5.89E-01	-1.17E-01

7618	GSM125026	GSE5460	Breast Cancer	Breast	1.04E-01	1.19E-01	3.07E-02	3.02E-01
7619	GSM125027	GSE5460	Breast Cancer	Breast	1.60E-01	-1.07E-01	3.84E-01	1.58E-01
7620	GSM125028	GSE5460	Breast Cancer	Breast	1.26E-03	-2.04E-01	5.19E-01	-1.31E-01
7621	GSM125029	GSE5460	Breast Cancer	Breast	9.46E-07	2.95E-01	1.43E-05	5.40E-01
7622	GSM125030	GSE5460	Breast Cancer	Breast	5.46E-02	-1.35E-01	1.96E-01	2.06E-01
7623	GSM125031	GSE5460	Breast Cancer	Breast	4.09E-03	-1.86E-01	3.11E-01	1.75E-01
7624	GSM125032	GSE5460	Breast Cancer	Breast	1.83E-01	1.03E-01	1.15E-01	2.37E-01
7625	GSM125033	GSE5460	Breast Cancer	Breast	4.59E-02	-1.39E-01	2.27E-01	1.97E-01
7626	GSM125034	GSE5460	Breast Cancer	Breast	1.31E-02	1.65E-01	4.98E-03	3.72E-01
7627	GSM125035	GSE5460	Breast Cancer	Breast	1.46E-02	-1.63E-01	3.24E-01	-1.71E-01
7628	GSM125036	GSE5460	Breast Cancer	Breast	1.89E-01	-1.02E-01	1.03E-01	2.44E-01
7629	GSM125037	GSE5460	Breast Cancer	Breast	6.19E-02	-1.32E-01	3.16E-01	1.73E-01
7630	GSM125038	GSE5460	Breast Cancer	Breast	1.95E-01	1.01E-01	1.15E-01	2.37E-01
7631	GSM125039	GSE5460	Breast Cancer	Breast	4.87E-03	1.83E-01	3.85E-03	3.81E-01
7632	GSM125040	GSE5460	Breast Cancer	Breast	2.42E-05	-2.58E-01	1.49E-02	-3.31E-01
7633	GSM125041	GSE5460	Breast Cancer	Breast	1.61E-02	1.61E-01	1.47E-03	4.13E-01
7634	GSM125042	GSE5460	Breast Cancer	Breast	2.86E-03	-1.91E-01	2.29E-01	-1.96E-01
7635	GSM125043	GSE5460	Breast Cancer	Breast	4.09E-03	-1.86E-01	1.96E-01	2.06E-01
7636	GSM125044	GSE5460	Breast Cancer	Breast	9.73E-04	-2.08E-01	1.04E-01	-2.43E-01
7637	GSM125045	GSE5460	Breast Cancer	Breast	5.69E-02	1.34E-01	4.38E-02	2.86E-01
7638	GSM125046	GSE5460	Breast Cancer	Breast	9.04E-03	1.72E-01	1.53E-03	4.11E-01
7639	GSM125047	GSE5460	Breast Cancer	Breast	1.06E-02	-1.69E-01	2.31E-01	-1.96E-01
7640	GSM125048	GSE5460	Breast Cancer	Breast	6.84E-06	-2.73E-01	8.88E-02	-2.51E-01
7641	GSM125049	GSE5460	Breast Cancer	Breast	2.16E-06	-2.86E-01	3.16E-02	-3.00E-01
7642	GSM125050	GSE5460	Breast Cancer	Breast	1.21E-01	1.15E-01	7.46E-02	2.60E-01
7643	GSM125051	GSE5460	Breast Cancer	Breast	5.23E-02	-1.36E-01	2.80E-01	1.82E-01
7644	GSM125052	GSE5460	Breast Cancer	Breast	7.45E-04	-2.12E-01	1.37E-01	-2.28E-01
7645	GSM125053	GSE5460	Breast Cancer	Breast	7.36E-05	2.44E-01	3.90E-06	5.70E-01
7646	GSM125054	GSE5460	Breast Cancer	Breast	2.64E-02	1.51E-01	4.38E-02	2.86E-01
7647	GSM125055	GSE5460	Breast Cancer	Breast	3.56E-01	-8.04E-02	2.22E-01	1.98E-01
7648	GSM125056	GSE5460	Breast Cancer	Breast	3.23E-03	1.89E-01	1.86E-02	3.23E-01
7649	GSM125057	GSE5460	Breast Cancer	Breast	7.14E-07	-2.98E-01	1.18E-02	-3.40E-01
7650	GSM125058	GSE5460	Breast Cancer	Breast	8.13E-06	-2.71E-01	7.74E-02	-2.58E-01
7651	GSM125059	GSE5460	Breast Cancer	Breast	1.88E-02	1.58E-01	1.18E-02	3.40E-01
7652	GSM125060	GSE5460	Breast Cancer	Breast	1.77E-01	-1.04E-01	4.20E-01	1.50E-01
7653	GSM125061	GSE5460	Breast Cancer	Breast	2.57E-01	-9.23E-02	1.32E-01	2.30E-01
7654	GSM125062	GSE5460	Breast Cancer	Breast	3.63E-03	-1.88E-01	2.58E-01	-1.88E-01
7655	GSM125063	GSE5460	Breast Cancer	Breast	2.56E-12	-4.09E-01	2.44E-04	-4.66E-01
7656	GSM125064	GSE5460	Breast Cancer	Breast	1.97E-02	-1.57E-01	3.16E-01	-1.73E-01
7657	GSM125065	GSE5460	Breast Cancer	Breast	1.00E-01	1.20E-01	3.08E-03	3.89E-01
7658	GSM125066	GSE5460	Breast Cancer	Breast	2.24E-03	-1.95E-01	2.00E-01	-2.05E-01
7659	GSM125067	GSE5460	Breast Cancer	Breast	3.56E-01	8.04E-02	2.22E-01	1.98E-01
7660	GSM125068	GSE5460	Breast Cancer	Breast	4.20E-02	-1.41E-01	3.84E-01	1.58E-01
7661	GSM125069	GSE5460	Breast Cancer	Breast	1.53E-03	-2.01E-01	1.03E-01	-2.44E-01
7662	GSM125070	GSE5460	Breast Cancer	Breast	2.96E-01	8.73E-02	9.90E-02	2.46E-01
7663	GSM125071	GSE5460	Breast Cancer	Breast	9.27E-05	2.41E-01	1.17E-03	4.20E-01
7664	GSM125072	GSE5460	Breast Cancer	Breast	3.04E-01	-8.63E-02	1.77E-01	2.13E-01
7665	GSM125073	GSE5460	Breast Cancer	Breast	2.11E-04	-2.30E-01	2.00E-01	-2.05E-01
7666	GSM125074	GSE5460	Breast Cancer	Breast	4.95E-04	2.18E-01	1.47E-03	4.13E-01
7667	GSM125075	GSE5460	Breast Cancer	Breast	4.53E-01	-7.04E-02	1.96E-01	2.06E-01
7668	GSM125076	GSE5460	Breast Cancer	Breast	1.06E-02	1.69E-01	6.15E-02	2.70E-01
7669	GSM125077	GSE5460	Breast Cancer	Breast	3.03E-04	2.25E-01	5.28E-04	4.44E-01
7670	GSM125078	GSE5460	Breast Cancer	Breast	5.46E-02	1.35E-01	4.38E-02	2.86E-01
7671	GSM125079	GSE5460	Breast Cancer	Breast	3.34E-02	-1.46E-01	2.80E-01	1.82E-01
7672	GSM125080	GSE5460	Breast Cancer	Breast	2.44E-04	-2.28E-01	1.98E-01	-2.06E-01
7673	GSM125081	GSE5460	Breast Cancer	Breast	1.85E-07	-3.12E-01	4.07E-04	-4.51E-01
7674	GSM125082	GSE5460	Breast Cancer	Breast	1.26E-04	-2.37E-01	1.18E-02	-3.40E-01
7675	GSM125083	GSE5460	Breast Cancer	Breast	1.04E-01	1.19E-01	8.56E-02	2.53E-01
7676	GSM125084	GSE5460	Breast Cancer	Breast	2.49E-01	-9.33E-02	2.51E-01	1.90E-01
7677	GSM125085	GSE5460	Breast Cancer	Breast	1.12E-01	1.17E-01	9.36E-03	3.49E-01
7678	GSM125086	GSE5460	Breast Cancer	Breast	3.84E-01	-7.74E-02	8.77E-02	2.52E-01
7679	GSM125087	GSE5460	Breast Cancer	Breast	3.66E-07	3.05E-01	5.23E-05	5.07E-01
7680	GSM125088	GSE5460	Breast Cancer	Breast	1.44E-01	1.10E-01	5.31E-02	2.77E-01
7681	GSM125089	GSE5460	Breast Cancer	Breast	2.84E-05	-2.56E-01	2.77E-02	-3.06E-01
7682	GSM125090	GSE5460	Breast Cancer	Breast	2.23E-05	-2.59E-01	6.23E-02	-2.69E-01
7683	GSM125091	GSE5460	Breast Cancer	Breast	1.57E-04	2.34E-01	6.83E-04	4.36E-01
7684	GSM125092	GSE5460	Breast Cancer	Breast	1.67E-07	-3.13E-01	1.50E-03	-4.12E-01
7685	GSM125093	GSE5460	Breast Cancer	Breast	1.44E-01	1.10E-01	3.85E-03	3.81E-01
7686	GSM125094	GSE5460	Breast Cancer	Breast	8.56E-03	-1.73E-01	3.49E-01	1.66E-01
7687	GSM125095	GSE5460	Breast Cancer	Breast	2.14E-01	9.82E-02	1.32E-01	2.30E-01
7688	GSM125096	GSE5460	Breast Cancer	Breast	7.60E-02	-1.27E-01	3.81E-01	1.59E-01
7689	GSM125097	GSE5460	Breast Cancer	Breast	4.59E-02	1.39E-01	4.98E-03	3.72E-01
7690	GSM125098	GSE5460	Breast Cancer	Breast	7.97E-04	2.11E-01	7.75E-03	3.56E-01
7691	GSM125099	GSE5460	Breast Cancer	Breast	4.34E-03	-1.85E-01	2.90E-01	-1.80E-01

7692	GSM125100	GSE5460	Breast Cancer	Breast	1.12E-02	-1.68E-01	2.49E-01	-1.90E-01
7693	GSM125101	GSE5460	Breast Cancer	Breast	1.21E-01	1.15E-01	2.14E-02	3.17E-01
7694	GSM125102	GSE5460	Breast Cancer	Breast	4.63E-01	6.94E-02	2.22E-01	1.98E-01
7695	GSM125103	GSE5460	Breast Cancer	Breast	3.09E-09	-3.50E-01	1.50E-03	-4.12E-01
7696	GSM125104	GSE5460	Breast Cancer	Breast	7.92E-02	-1.26E-01	4.23E-01	1.50E-01
7697	GSM125105	GSE5460	Breast Cancer	Breast	5.23E-02	1.36E-01	8.56E-02	2.53E-01
7698	GSM125106	GSE5460	Breast Cancer	Breast	4.09E-03	1.86E-01	3.13E-03	3.88E-01
7699	GSM125107	GSE5460	Breast Cancer	Breast	2.17E-08	-3.32E-01	3.12E-04	-4.59E-01
7700	GSM125108	GSE5460	Breast Cancer	Breast	3.19E-02	-1.47E-01	4.58E-01	1.43E-01
7701	GSM125109	GSE5460	Breast Cancer	Breast	2.29E-02	1.54E-01	3.16E-02	3.00E-01
7702	GSM125110	GSE5460	Breast Cancer	Breast	6.51E-04	-2.14E-01	2.51E-01	-1.90E-01
7703	GSM125111	GSE5460	Breast Cancer	Breast	1.65E-01	-1.06E-01	1.32E-01	2.30E-01
7704	GSM125112	GSE5460	Breast Cancer	Breast	7.01E-02	1.29E-01	2.11E-02	3.17E-01
7705	GSM125113	GSE5460	Breast Cancer	Breast	1.35E-05	-2.65E-01	9.85E-03	-3.47E-01
7706	GSM125114	GSE5460	Breast Cancer	Breast	7.97E-10	-3.62E-01	2.54E-03	-3.95E-01
7707	GSM125115	GSE5460	Breast Cancer	Breast	8.29E-09	-3.41E-01	6.28E-03	-3.64E-01
7708	GSM125116	GSE5460	Breast Cancer	Breast	3.49E-04	-2.23E-01	1.80E-01	-2.11E-01
7709	GSM125117	GSE5460	Breast Cancer	Breast	3.21E-01	-8.43E-02	1.73E-01	2.14E-01
7710	GSM125118	GSE5460	Breast Cancer	Breast	1.04E-01	-1.19E-01	3.81E-01	1.59E-01
7711	GSM125121	GSE5460	Breast Cancer	Breast	1.98E-06	2.87E-01	3.98E-04	4.52E-01
7712	GSM125122	GSE5460	Breast Cancer	Breast	1.71E-01	1.05E-01	1.32E-01	2.30E-01
7713	GSM134587	GSE5764	Breast Cancer	Breast	1.77E-01	1.04E-01	6.30E-01	1.10E-01
7714	GSM134591	GSE5764	Breast Cancer	Breast	4.43E-01	-7.14E-02	3.84E-01	1.58E-01
7715	GSM134689	GSE5764	Breast Cancer	Breast	5.79E-03	-1.80E-01	2.02E-01	-2.04E-01
7716	GSM134692	GSE5764	Breast Cancer	Breast	1.85E-03	-1.98E-01	2.83E-01	-1.82E-01
7717	GSM134695	GSE5764	Breast Cancer	Breast	3.68E-21	5.43E-01	2.09E-13	8.73E-01
7718	GSM134698	GSE5764	Breast Cancer	Breast	1.50E-19	5.21E-01	1.41E-10	7.70E-01
7719	GSM134701	GSE5764	Breast Cancer	Breast	4.03E-01	7.54E-02	2.33E-01	1.95E-01
7720	GSM134704	GSE5764	Breast Cancer	Breast	2.05E-05	-2.60E-01	1.54E-02	-3.30E-01
7721	GSM134707	GSE5764	Breast Cancer	Breast	6.81E-05	-2.45E-01	5.60E-02	-2.74E-01
7722	GSM134710	GSE5764	Breast Cancer	Breast	6.51E-04	-2.14E-01	1.79E-01	-2.12E-01
7723	GSM491175	GSE19615	Breast Cancer	Breast	1.18E-03	-2.05E-01	2.51E-01	-1.90E-01
7724	GSM491176	GSE19615	Breast Cancer	Breast	3.34E-05	2.54E-01	3.08E-03	3.89E-01
7725	GSM491177	GSE19615	Breast Cancer	Breast	5.68E-04	2.16E-01	2.49E-03	3.96E-01
7726	GSM491178	GSE19615	Breast Cancer	Breast	7.01E-02	-1.29E-01	4.61E-01	-1.42E-01
7727	GSM491179	GSE19615	Breast Cancer	Breast	1.26E-03	-2.04E-01	2.54E-01	-1.89E-01
7728	GSM491180	GSE19615	Breast Cancer	Breast	2.80E-10	-3.71E-01	8.16E-03	-3.54E-01
7729	GSM491181	GSE19615	Breast Cancer	Breast	1.89E-05	2.61E-01	6.98E-04	4.36E-01
7730	GSM491182	GSE19615	Breast Cancer	Breast	7.91E-14	-4.35E-01	2.87E-06	-5.77E-01
7731	GSM491183	GSE19615	Breast Cancer	Breast	1.71E-01	1.05E-01	9.90E-02	2.46E-01
7732	GSM491184	GSE19615	Breast Cancer	Breast	6.73E-02	1.30E-01	8.56E-02	2.53E-01
7733	GSM491185	GSE19615	Breast Cancer	Breast	6.49E-07	2.99E-01	1.89E-04	4.73E-01
7734	GSM491186	GSE19615	Breast Cancer	Breast	6.67E-09	-3.43E-01	1.22E-03	-4.18E-01
7735	GSM491187	GSE19615	Breast Cancer	Breast	3.25E-04	-2.24E-01	2.33E-01	-1.95E-01
7736	GSM491188	GSE19615	Breast Cancer	Breast	2.53E-03	1.93E-01	8.16E-03	3.54E-01
7737	GSM491189	GSE19615	Breast Cancer	Breast	1.16E-01	1.16E-01	4.45E-02	2.85E-01
7738	GSM491190	GSE19615	Breast Cancer	Breast	2.57E-01	-9.23E-02	1.14E-01	2.38E-01
7739	GSM491191	GSE19615	Breast Cancer	Breast	8.61E-07	2.96E-01	3.78E-05	5.16E-01
7740	GSM491192	GSE19615	Breast Cancer	Breast	2.18E-02	-1.55E-01	1.75E-01	2.13E-01
7741	GSM491193	GSE19615	Breast Cancer	Breast	1.58E-08	3.35E-01	1.07E-05	5.47E-01
7742	GSM491194	GSE19615	Breast Cancer	Breast	3.53E-13	-4.24E-01	1.50E-05	-5.38E-01
7743	GSM491195	GSE19615	Breast Cancer	Breast	1.46E-02	-1.63E-01	1.75E-01	-2.13E-01
7744	GSM491196	GSE19615	Breast Cancer	Breast	2.14E-01	9.82E-02	3.07E-02	3.02E-01
7745	GSM491197	GSE19615	Breast Cancer	Breast	2.35E-01	9.52E-02	9.90E-02	2.46E-01
7746	GSM491198	GSE19615	Breast Cancer	Breast	1.26E-04	2.37E-01	2.05E-03	4.02E-01
7747	GSM491199	GSE19615	Breast Cancer	Breast	6.32E-01	5.36E-02	2.83E-01	1.82E-01
7748	GSM491200	GSE19615	Breast Cancer	Breast	1.74E-05	-2.62E-01	1.77E-01	-2.13E-01
7749	GSM491201	GSE19615	Breast Cancer	Breast	2.08E-01	-9.92E-02	3.55E-01	1.64E-01
7750	GSM491202	GSE19615	Breast Cancer	Breast	4.20E-02	-1.41E-01	5.83E-01	1.19E-01
7751	GSM491203	GSE19615	Breast Cancer	Breast	1.65E-01	1.06E-01	1.75E-01	2.13E-01
7752	GSM491204	GSE19615	Breast Cancer	Breast	1.30E-01	1.13E-01	1.42E-02	3.33E-01
7753	GSM491205	GSE19615	Breast Cancer	Breast	2.80E-01	-8.93E-02	3.14E-01	1.74E-01
7754	GSM491206	GSE19615	Breast Cancer	Breast	2.53E-03	-1.93E-01	5.43E-01	1.26E-01
7755	GSM491207	GSE19615	Breast Cancer	Breast	5.37E-07	3.01E-01	7.33E-06	5.56E-01
7756	GSM491208	GSE19615	Breast Cancer	Breast	1.00E-01	-1.20E-01	1.96E-01	2.06E-01
7757	GSM491209	GSE19615	Breast Cancer	Breast	1.53E-02	-1.62E-01	3.11E-01	1.75E-01
7758	GSM491210	GSE19615	Breast Cancer	Breast	2.21E-01	9.72E-02	8.56E-02	2.53E-01
7759	GSM491211	GSE19615	Breast Cancer	Breast	5.01E-02	-1.37E-01	2.27E-01	1.97E-01
7760	GSM491212	GSE19615	Breast Cancer	Breast	2.29E-02	1.54E-01	4.98E-03	3.72E-01
7761	GSM491213	GSE19615	Breast Cancer	Breast	2.29E-02	-1.54E-01	3.49E-01	1.66E-01
7762	GSM491214	GSE19615	Breast Cancer	Breast	2.21E-01	-9.72E-02	1.00E-01	2.45E-01
7763	GSM491215	GSE19615	Breast Cancer	Breast	1.30E-01	-1.13E-01	2.54E-01	1.89E-01
7764	GSM491216	GSE19615	Breast Cancer	Breast	1.71E-01	1.05E-01	1.15E-01	2.37E-01
7765	GSM491217	GSE19615	Breast Cancer	Breast	4.25E-05	-2.51E-01	1.49E-02	-3.31E-01

7766	GSM491218	GSE19615	Breast Cancer	Breast	1.70E-02	1.60E-01	8.79E-04	4.29E-01
7767	GSM491219	GSE19615	Breast Cancer	Breast	1.44E-03	-2.02E-01	1.04E-01	-2.43E-01
7768	GSM491220	GSE19615	Breast Cancer	Breast	5.01E-02	1.37E-01	3.07E-02	3.02E-01
7769	GSM491221	GSE19615	Breast Cancer	Breast	3.63E-03	1.88E-01	1.53E-03	4.11E-01
7770	GSM491222	GSE19615	Breast Cancer	Breast	1.31E-02	-1.65E-01	2.31E-01	-1.96E-01
7771	GSM491223	GSE19615	Breast Cancer	Breast	3.62E-05	-2.53E-01	1.15E-01	-2.37E-01
7772	GSM491224	GSE19615	Breast Cancer	Breast	4.42E-06	-2.78E-01	3.16E-02	-3.00E-01
7773	GSM491225	GSE19615	Breast Cancer	Breast	1.95E-01	-1.01E-01	4.20E-01	1.50E-01
7774	GSM491226	GSE19615	Breast Cancer	Breast	4.01E-02	-1.42E-01	2.22E-01	1.98E-01
7775	GSM491227	GSE19615	Breast Cancer	Breast	1.04E-03	-2.07E-01	1.37E-01	-2.28E-01
7776	GSM491228	GSE19615	Breast Cancer	Breast	1.00E-04	2.40E-01	7.53E-06	5.55E-01
7777	GSM491229	GSE19615	Breast Cancer	Breast	1.38E-02	1.64E-01	2.68E-02	3.07E-01
7778	GSM491230	GSE19615	Breast Cancer	Breast	3.38E-01	8.23E-02	1.73E-01	2.14E-01
7779	GSM491231	GSE19615	Breast Cancer	Breast	1.53E-03	2.01E-01	2.24E-02	3.15E-01
7780	GSM491232	GSE19615	Breast Cancer	Breast	1.98E-06	-2.87E-01	1.77E-02	-3.24E-01
7781	GSM491233	GSE19615	Breast Cancer	Breast	2.23E-05	-2.59E-01	7.74E-02	-2.58E-01
7782	GSM491234	GSE19615	Breast Cancer	Breast	1.46E-02	1.63E-01	1.51E-02	3.31E-01
7783	GSM491235	GSE19615	Breast Cancer	Breast	2.42E-01	-9.42E-02	1.52E-01	2.22E-01
7784	GSM491236	GSE19615	Breast Cancer	Breast	3.86E-03	-1.87E-01	2.58E-01	-1.88E-01
7785	GSM491237	GSE19615	Breast Cancer	Breast	2.56E-12	-4.09E-01	4.25E-04	-4.50E-01
7786	GSM491238	GSE19615	Breast Cancer	Breast	1.53E-02	-1.62E-01	3.87E-01	-1.57E-01
7787	GSM491239	GSE19615	Breast Cancer	Breast	7.92E-02	1.26E-01	6.06E-03	3.65E-01
7788	GSM491240	GSE19615	Breast Cancer	Breast	3.30E-01	8.33E-02	2.00E-01	2.05E-01
7789	GSM491241	GSE19615	Breast Cancer	Breast	7.60E-02	-1.27E-01	3.14E-01	1.74E-01
7790	GSM491242	GSE19615	Breast Cancer	Breast	5.47E-03	-1.81E-01	2.58E-01	-1.88E-01
7791	GSM491243	GSE19615	Breast Cancer	Breast	3.04E-01	8.63E-02	7.27E-02	2.62E-01
7792	GSM491244	GSE19615	Breast Cancer	Breast	3.62E-05	2.53E-01	7.13E-04	4.35E-01
7793	GSM491245	GSE19615	Breast Cancer	Breast	1.35E-04	-2.36E-01	3.52E-01	-1.65E-01
7794	GSM491246	GSE19615	Breast Cancer	Breast	7.96E-05	2.43E-01	8.79E-04	4.29E-01
7795	GSM491247	GSE19615	Breast Cancer	Breast	7.25E-03	1.76E-01	4.38E-02	2.86E-01
7796	GSM491248	GSE19615	Breast Cancer	Breast	3.50E-02	-1.45E-01	2.49E-01	1.90E-01
7797	GSM491249	GSE19615	Breast Cancer	Breast	1.96E-04	-2.31E-01	1.98E-01	-2.06E-01
7798	GSM491250	GSE19615	Breast Cancer	Breast	1.98E-06	-2.87E-01	6.98E-04	-4.36E-01
7799	GSM491251	GSE19615	Breast Cancer	Breast	1.35E-04	-2.36E-01	9.52E-03	-3.49E-01
7800	GSM491252	GSE19615	Breast Cancer	Breast	1.39E-01	1.11E-01	6.23E-02	2.69E-01
7801	GSM491253	GSE19615	Breast Cancer	Breast	2.57E-01	-9.23E-02	2.51E-01	1.90E-01
7802	GSM491254	GSE19615	Breast Cancer	Breast	6.19E-02	1.32E-01	9.36E-03	3.49E-01
7803	GSM491255	GSE19615	Breast Cancer	Breast	4.33E-01	-7.24E-02	6.15E-02	2.70E-01
7804	GSM491256	GSE19615	Breast Cancer	Breast	3.32E-07	3.06E-01	2.79E-05	5.23E-01
7805	GSM491257	GSE19615	Breast Cancer	Breast	2.62E-05	-2.57E-01	1.89E-02	-3.22E-01
7806	GSM491258	GSE19615	Breast Cancer	Breast	9.27E-05	2.41E-01	6.83E-04	4.36E-01
7807	GSM491259	GSE19615	Breast Cancer	Breast	1.24E-07	-3.15E-01	1.50E-03	-4.12E-01
7808	GSM491260	GSE19615	Breast Cancer	Breast	1.44E-01	1.10E-01	3.85E-03	3.81E-01
7809	GSM491261	GSE19615	Breast Cancer	Breast	1.31E-02	-1.65E-01	2.25E-01	1.97E-01
7810	GSM491262	GSE19615	Breast Cancer	Breast	8.91E-02	1.23E-01	1.32E-01	2.30E-01
7811	GSM491263	GSE19615	Breast Cancer	Breast	5.94E-02	-1.33E-01	2.80E-01	1.82E-01
7812	GSM491264	GSE19615	Breast Cancer	Breast	6.19E-02	-1.32E-01	1.75E-01	2.13E-01
7813	GSM491265	GSE19615	Breast Cancer	Breast	2.77E-02	1.50E-01	3.13E-03	3.88E-01
7814	GSM491266	GSE19615	Breast Cancer	Breast	3.74E-04	2.22E-01	7.75E-03	3.56E-01
7815	GSM491267	GSE19615	Breast Cancer	Breast	3.86E-03	-1.87E-01	3.16E-01	1.73E-01
7816	GSM491268	GSE19615	Breast Cancer	Breast	5.47E-03	-1.81E-01	2.49E-01	-1.90E-01
7817	GSM491269	GSE19615	Breast Cancer	Breast	5.46E-02	1.35E-01	3.11E-02	3.01E-01
7818	GSM491270	GSE19615	Breast Cancer	Breast	7.30E-02	-1.28E-01	3.84E-01	1.58E-01
7819	GSM491271	GSE19615	Breast Cancer	Breast	3.05E-02	1.48E-01	8.56E-02	2.53E-01
7820	GSM491272	GSE19615	Breast Cancer	Breast	3.19E-02	1.47E-01	9.85E-03	3.47E-01
7821	GSM491273	GSE19615	Breast Cancer	Breast	1.58E-08	-3.35E-01	5.28E-04	-4.44E-01
7822	GSM491274	GSE19615	Breast Cancer	Breast	2.40E-02	-1.53E-01	3.81E-01	1.59E-01
7823	GSM491275	GSE19615	Breast Cancer	Breast	3.05E-02	1.48E-01	3.16E-02	3.00E-01
7824	GSM491276	GSE19615	Breast Cancer	Breast	5.69E-02	1.34E-01	9.36E-03	3.49E-01
7825	GSM491277	GSE19615	Breast Cancer	Breast	8.94E-10	-3.61E-01	2.01E-03	-4.03E-01
7826	GSM491278	GSE19615	Breast Cancer	Breast	3.67E-02	1.44E-01	1.16E-02	3.41E-01
7827	GSM491279	GSE19615	Breast Cancer	Breast	1.14E-08	-3.38E-01	5.07E-03	-3.71E-01
7828	GSM491280	GSE19615	Breast Cancer	Breast	1.37E-06	-2.91E-01	2.20E-02	-3.16E-01
7829	GSM491281	GSE19615	Breast Cancer	Breast	8.13E-06	-2.71E-01	4.51E-02	-2.84E-01
7830	GSM491282	GSE19615	Breast Cancer	Breast	1.89E-05	-2.61E-01	5.07E-03	-3.71E-01
7831	GSM491283	GSE19615	Breast Cancer	Breast	1.38E-02	-1.64E-01	2.58E-01	-1.88E-01
7832	GSM491284	GSE19615	Breast Cancer	Breast	1.63E-03	2.00E-01	1.50E-03	4.12E-01
7833	GSM491285	GSE19615	Breast Cancer	Breast	9.04E-03	1.72E-01	1.15E-03	4.20E-01
7834	GSM491286	GSE19615	Breast Cancer	Breast	4.59E-02	-1.39E-01	3.90E-01	-1.57E-01
7835	GSM491287	GSE19615	Breast Cancer	Breast	1.89E-05	-2.61E-01	8.02E-03	-3.55E-01
7836	GSM491288	GSE19615	Breast Cancer	Breast	5.75E-06	-2.75E-01	2.17E-02	-3.16E-01
7837	GSM491289	GSE19615	Breast Cancer	Breast	1.14E-05	-2.67E-01	5.07E-03	-3.71E-01
7838	GSM282373	GSE11121	Breast Cancer	Breast	4.02E-04	-2.21E-01	2.20E-02	-3.16E-01
7839	GSM282374	GSE11121	Breast Cancer	Breast	9.51E-16	-4.65E-01	6.40E-07	-6.10E-01

7840	GSM282375	GSE11121	Breast Cancer	Breast	4.13E-01	7.44E-02	8.45E-02	2.54E-01
7841	GSM282376	GSE11121	Breast Cancer	Breast	1.53E-02	-1.62E-01	3.93E-01	-1.56E-01
7842	GSM282377	GSE11121	Breast Cancer	Breast	2.82E-04	-2.26E-01	1.03E-01	-2.44E-01
7843	GSM282378	GSE11121	Breast Cancer	Breast	6.00E-01	5.65E-02	1.00E-01	2.45E-01
7844	GSM282379	GSE11121	Breast Cancer	Breast	2.53E-03	-1.93E-01	1.37E-01	-2.28E-01
7845	GSM282380	GSE11121	Breast Cancer	Breast	4.31E-04	2.20E-01	2.01E-03	4.03E-01
7846	GSM282381	GSE11121	Breast Cancer	Breast	1.38E-10	-3.77E-01	3.20E-02	-3.00E-01
7847	GSM282382	GSE11121	Breast Cancer	Breast	4.59E-02	-1.39E-01	4.23E-01	1.50E-01
7848	GSM282383	GSE11121	Breast Cancer	Breast	3.93E-05	2.52E-01	3.98E-04	4.52E-01
7849	GSM282384	GSE11121	Breast Cancer	Breast	2.28E-01	-9.62E-02	2.80E-01	1.82E-01
7850	GSM282385	GSE11121	Breast Cancer	Breast	1.65E-01	-1.06E-01	1.15E-01	2.37E-01
7851	GSM282386	GSE11121	Breast Cancer	Breast	2.62E-05	-2.57E-01	1.17E-01	-2.37E-01
7852	GSM282387	GSE11121	Breast Cancer	Breast	2.42E-01	-9.42E-02	1.96E-01	2.06E-01
7853	GSM282388	GSE11121	Breast Cancer	Breast	1.04E-01	-1.19E-01	3.81E-01	1.59E-01
7854	GSM282389	GSE11121	Breast Cancer	Breast	5.46E-02	1.35E-01	1.16E-02	3.41E-01
7855	GSM282390	GSE11121	Breast Cancer	Breast	2.62E-04	2.27E-01	1.53E-03	4.11E-01
7856	GSM282391	GSE11121	Breast Cancer	Breast	1.77E-01	1.04E-01	7.46E-02	2.60E-01
7857	GSM282392	GSE11121	Breast Cancer	Breast	1.39E-01	-1.11E-01	2.49E-01	1.90E-01
7858	GSM282393	GSE11121	Breast Cancer	Breast	1.39E-01	1.11E-01	5.38E-02	2.76E-01
7859	GSM282394	GSE11121	Breast Cancer	Breast	7.13E-12	-4.01E-01	5.28E-04	-4.44E-01
7860	GSM282395	GSE11121	Breast Cancer	Breast	3.74E-04	-2.22E-01	5.31E-02	-2.77E-01
7861	GSM282396	GSE11121	Breast Cancer	Breast	1.96E-04	-2.31E-01	1.21E-01	-2.35E-01
7862	GSM282397	GSE11121	Breast Cancer	Breast	1.24E-05	-2.66E-01	2.20E-02	-3.16E-01
7863	GSM282398	GSE11121	Breast Cancer	Breast	7.38E-01	-4.37E-02	3.19E-01	1.73E-01
7864	GSM282399	GSE11121	Breast Cancer	Breast	7.96E-05	-2.43E-01	1.50E-03	-4.12E-01
7865	GSM282400	GSE11121	Breast Cancer	Breast	5.79E-03	1.80E-01	2.40E-03	3.97E-01
7866	GSM282401	GSE11121	Breast Cancer	Breast	2.52E-02	1.52E-01	6.39E-03	3.63E-01
7867	GSM282402	GSE11121	Breast Cancer	Breast	6.73E-02	1.30E-01	1.44E-02	3.33E-01
7868	GSM282403	GSE11121	Breast Cancer	Breast	3.32E-07	-3.06E-01	6.39E-03	-3.63E-01
7869	GSM282404	GSE11121	Breast Cancer	Breast	5.36E-09	-3.45E-01	4.15E-03	-3.78E-01
7870	GSM282405	GSE11121	Breast Cancer	Breast	2.38E-03	-1.94E-01	3.58E-01	-1.64E-01
7871	GSM282406	GSE11121	Breast Cancer	Breast	7.46E-06	2.72E-01	1.53E-03	4.11E-01
7872	GSM282407	GSE11121	Breast Cancer	Breast	3.34E-02	-1.46E-01	3.19E-01	-1.73E-01
7873	GSM282408	GSE11121	Breast Cancer	Breast	1.53E-02	-1.62E-01	3.14E-01	-1.74E-01
7874	GSM282409	GSE11121	Breast Cancer	Breast	3.84E-01	7.74E-02	2.64E-02	3.08E-01
7875	GSM282410	GSE11121	Breast Cancer	Breast	6.77E-08	-3.21E-01	6.39E-02	-2.68E-01
7876	GSM282411	GSE11121	Breast Cancer	Breast	6.48E-03	1.78E-01	6.98E-04	4.36E-01
7877	GSM282412	GSE11121	Breast Cancer	Breast	5.01E-02	1.37E-01	6.06E-03	3.65E-01
7878	GSM282413	GSE11121	Breast Cancer	Breast	1.46E-02	1.63E-01	6.15E-02	2.70E-01
7879	GSM282414	GSE11121	Breast Cancer	Breast	3.03E-04	-2.25E-01	3.96E-01	-1.56E-01
7880	GSM282415	GSE11121	Breast Cancer	Breast	1.88E-02	-1.58E-01	3.58E-01	-1.64E-01
7881	GSM282416	GSE11121	Breast Cancer	Breast	4.95E-04	-2.18E-01	1.51E-01	2.22E-01
7882	GSM282417	GSE11121	Breast Cancer	Breast	2.25E-07	-3.10E-01	4.57E-02	-2.84E-01
7883	GSM282418	GSE11121	Breast Cancer	Breast	2.67E-08	-3.30E-01	6.28E-03	-3.64E-01
7884	GSM282419	GSE11121	Breast Cancer	Breast	4.44E-07	-3.03E-01	6.28E-03	-3.64E-01
7885	GSM282420	GSE11121	Breast Cancer	Breast	7.44E-09	3.42E-01	1.83E-06	5.87E-01
7886	GSM282421	GSE11121	Breast Cancer	Breast	1.44E-01	1.10E-01	3.70E-02	2.93E-01
7887	GSM282422	GSE11121	Breast Cancer	Breast	1.16E-01	1.16E-01	8.45E-02	2.54E-01
7888	GSM282423	GSE11121	Breast Cancer	Breast	1.16E-01	1.16E-01	8.45E-02	2.54E-01
7889	GSM282424	GSE11121	Breast Cancer	Breast	5.91E-07	-3.00E-01	4.07E-03	-3.79E-01
7890	GSM282425	GSE11121	Breast Cancer	Breast	5.23E-02	1.36E-01	8.45E-02	2.54E-01
7891	GSM282426	GSE11121	Breast Cancer	Breast	1.60E-01	-1.07E-01	2.80E-01	1.82E-01
7892	GSM282427	GSE11121	Breast Cancer	Breast	4.83E-01	6.75E-02	1.38E-01	2.27E-01
7893	GSM282428	GSE11121	Breast Cancer	Breast	4.49E-08	-3.25E-01	3.81E-02	-2.92E-01
7894	GSM282429	GSE11121	Breast Cancer	Breast	9.46E-07	2.95E-01	3.01E-05	5.21E-01
7895	GSM282430	GSE11121	Breast Cancer	Breast	1.00E-04	-2.40E-01	9.10E-02	-2.50E-01
7896	GSM282431	GSE11121	Breast Cancer	Breast	3.23E-03	-1.89E-01	1.75E-01	-2.13E-01
7897	GSM282432	GSE11121	Breast Cancer	Breast	1.54E-01	1.08E-01	1.51E-01	2.22E-01
7898	GSM282433	GSE11121	Breast Cancer	Breast	2.57E-01	-9.23E-02	1.14E-01	2.38E-01
7899	GSM282434	GSE11121	Breast Cancer	Breast	3.34E-05	2.54E-01	3.78E-05	5.16E-01
7900	GSM282435	GSE11121	Breast Cancer	Breast	7.36E-05	-2.44E-01	6.23E-02	-2.69E-01
7901	GSM282436	GSE11121	Breast Cancer	Breast	3.34E-05	-2.54E-01	2.27E-02	-3.14E-01
7902	GSM282437	GSE11121	Breast Cancer	Breast	4.09E-03	1.86E-01	6.23E-02	2.69E-01
7903	GSM282438	GSE11121	Breast Cancer	Breast	3.86E-09	3.48E-01	2.93E-07	6.27E-01
7904	GSM282439	GSE11121	Breast Cancer	Breast	1.39E-01	-1.11E-01	2.49E-01	1.90E-01
7905	GSM282440	GSE11121	Breast Cancer	Breast	1.60E-01	-1.07E-01	6.15E-02	2.70E-01
7906	GSM282441	GSE11121	Breast Cancer	Breast	3.71E-06	-2.80E-01	9.52E-03	-3.49E-01
7907	GSM282442	GSE11121	Breast Cancer	Breast	1.75E-10	-3.75E-01	1.17E-03	-4.20E-01
7908	GSM282443	GSE11121	Breast Cancer	Breast	4.98E-05	-2.49E-01	1.22E-02	-3.39E-01
7909	GSM282444	GSE11121	Breast Cancer	Breast	2.47E-09	-3.52E-01	4.07E-04	-4.51E-01
7910	GSM282445	GSE11121	Breast Cancer	Breast	5.68E-04	2.16E-01	1.15E-03	4.20E-01
7911	GSM282446	GSE11121	Breast Cancer	Breast	2.52E-02	-1.52E-01	2.85E-01	-1.81E-01
7912	GSM282447	GSE11121	Breast Cancer	Breast	2.18E-02	1.55E-01	9.68E-03	3.48E-01
7913	GSM282448	GSE11121	Breast Cancer	Breast	2.05E-05	-2.60E-01	3.86E-02	-2.91E-01

7914	GSM282449	GSE11121	Breast Cancer	Breast	2.50E-11	-3.91E-01	1.37E-04	-4.82E-01
7915	GSM282450	GSE11121	Breast Cancer	Breast	2.24E-03	-1.95E-01	2.06E-01	-2.03E-01
7916	GSM282451	GSE11121	Breast Cancer	Breast	4.79E-02	-1.38E-01	3.81E-01	1.59E-01
7917	GSM282452	GSE11121	Breast Cancer	Breast	6.00E-14	-4.37E-01	3.88E-05	-5.15E-01
7918	GSM282453	GSE11121	Breast Cancer	Breast	4.79E-02	-1.38E-01	3.52E-01	-1.65E-01
7919	GSM282454	GSE11121	Breast Cancer	Breast	5.94E-02	-1.33E-01	1.15E-01	2.37E-01
7920	GSM282455	GSE11121	Breast Cancer	Breast	1.63E-03	-2.00E-01	2.64E-02	-3.08E-01
7921	GSM282456	GSE11121	Breast Cancer	Breast	1.14E-06	2.93E-01	2.72E-05	5.24E-01
7922	GSM282457	GSE11121	Breast Cancer	Breast	8.24E-02	1.25E-01	3.16E-02	3.00E-01
7923	GSM282458	GSE11121	Breast Cancer	Breast	2.72E-01	9.03E-02	8.66E-02	2.53E-01
7924	GSM282459	GSE11121	Breast Cancer	Breast	9.11E-04	2.09E-01	3.92E-03	3.80E-01
7925	GSM282460	GSE11121	Breast Cancer	Breast	1.53E-03	2.01E-01	1.50E-03	4.12E-01
7926	GSM282461	GSE11121	Breast Cancer	Breast	2.05E-05	-2.60E-01	1.03E-01	-2.44E-01
7927	GSM282462	GSE11121	Breast Cancer	Breast	7.01E-02	-1.29E-01	9.90E-02	2.46E-01
7928	GSM282463	GSE11121	Breast Cancer	Breast	1.75E-10	-3.75E-01	5.40E-04	-4.43E-01
7929	GSM282464	GSE11121	Breast Cancer	Breast	4.01E-02	-1.42E-01	3.55E-01	-1.64E-01
7930	GSM282465	GSE11121	Breast Cancer	Breast	1.60E-05	2.63E-01	1.15E-03	4.20E-01
7931	GSM282466	GSE11121	Breast Cancer	Breast	1.95E-01	1.01E-01	7.65E-02	2.59E-01
7932	GSM282467	GSE11121	Breast Cancer	Breast	2.72E-01	9.03E-02	2.63E-01	1.87E-01
7933	GSM282468	GSE11121	Breast Cancer	Breast	1.49E-01	1.09E-01	3.81E-01	1.59E-01
7934	GSM282469	GSE11121	Breast Cancer	Breast	4.01E-02	-1.42E-01	1.57E-01	2.20E-01
7935	GSM282470	GSE11121	Breast Cancer	Breast	1.49E-01	-1.09E-01	1.59E-01	2.19E-01
7936	GSM282471	GSE11121	Breast Cancer	Breast	3.74E-04	2.22E-01	9.36E-04	4.27E-01
7937	GSM282472	GSE11121	Breast Cancer	Breast	5.69E-02	-1.34E-01	1.32E-01	2.30E-01
7938	GSM282473	GSE11121	Breast Cancer	Breast	3.49E-04	-2.23E-01	1.63E-01	-2.18E-01
7939	GSM282474	GSE11121	Breast Cancer	Breast	7.97E-04	2.11E-01	6.06E-03	3.65E-01
7940	GSM282475	GSE11121	Breast Cancer	Breast	3.84E-02	1.43E-01	4.38E-02	2.86E-01
7941	GSM282476	GSE11121	Breast Cancer	Breast	2.64E-02	-1.51E-01	6.83E-01	-9.97E-02
7942	GSM282477	GSE11121	Breast Cancer	Breast	1.14E-06	-2.93E-01	9.10E-02	-2.50E-01
7943	GSM282478	GSE11121	Breast Cancer	Breast	2.64E-01	9.13E-02	1.15E-01	2.37E-01
7944	GSM282479	GSE11121	Breast Cancer	Breast	8.52E-04	-2.10E-01	2.58E-01	-1.88E-01
7945	GSM282480	GSE11121	Breast Cancer	Breast	1.46E-04	-2.35E-01	7.36E-02	-2.61E-01
7946	GSM282481	GSE11121	Breast Cancer	Breast	3.04E-03	-1.90E-01	2.90E-01	-1.80E-01
7947	GSM282482	GSE11121	Breast Cancer	Breast	4.98E-08	3.24E-01	1.37E-06	5.94E-01
7948	GSM282483	GSE11121	Breast Cancer	Breast	1.85E-03	1.98E-01	1.94E-03	4.04E-01
7949	GSM282484	GSE11121	Breast Cancer	Breast	1.70E-02	-1.60E-01	2.51E-01	-1.90E-01
7950	GSM282485	GSE11121	Breast Cancer	Breast	1.57E-04	-2.34E-01	1.98E-01	-2.06E-01
7951	GSM282486	GSE11121	Breast Cancer	Breast	1.26E-04	2.37E-01	3.78E-05	5.16E-01
7952	GSM282487	GSE11121	Breast Cancer	Breast	1.88E-02	-1.58E-01	3.49E-01	-1.66E-01
7953	GSM282488	GSE11121	Breast Cancer	Breast	4.31E-09	-3.47E-01	3.13E-03	-3.88E-01
7954	GSM282489	GSE11121	Breast Cancer	Breast	1.88E-02	-1.58E-01	3.46E-01	1.66E-01
7955	GSM282490	GSE11121	Breast Cancer	Breast	2.38E-03	-1.94E-01	2.25E-01	1.97E-01
7956	GSM282491	GSE11121	Breast Cancer	Breast	1.18E-02	-1.67E-01	1.14E-01	2.38E-01
7957	GSM282492	GSE11121	Breast Cancer	Breast	1.54E-01	1.08E-01	9.52E-03	3.49E-01
7958	GSM282493	GSE11121	Breast Cancer	Breast	1.21E-01	1.15E-01	4.38E-02	2.86E-01
7959	GSM282494	GSE11121	Breast Cancer	Breast	7.86E-13	-4.18E-01	2.11E-05	-5.30E-01
7960	GSM282495	GSE11121	Breast Cancer	Breast	2.80E-10	-3.71E-01	7.13E-04	-4.35E-01
7961	GSM282496	GSE11121	Breast Cancer	Breast	3.05E-02	1.48E-01	1.46E-02	3.32E-01
7962	GSM282497	GSE11121	Breast Cancer	Breast	1.53E-02	-1.62E-01	4.70E-01	-1.40E-01
7963	GSM282498	GSE11121	Breast Cancer	Breast	1.65E-01	1.06E-01	1.73E-01	2.14E-01
7964	GSM282499	GSE11121	Breast Cancer	Breast	5.15E-01	6.45E-02	2.90E-01	1.80E-01
7965	GSM282500	GSE11121	Breast Cancer	Breast	1.14E-08	3.38E-01	2.00E-05	5.31E-01
7966	GSM282501	GSE11121	Breast Cancer	Breast	3.62E-05	-2.53E-01	1.34E-01	-2.29E-01
7967	GSM282502	GSE11121	Breast Cancer	Breast	7.30E-02	1.28E-01	6.15E-02	2.70E-01
7968	GSM282503	GSE11121	Breast Cancer	Breast	1.49E-01	1.09E-01	1.46E-02	3.32E-01
7969	GSM282504	GSE11121	Breast Cancer	Breast	2.49E-10	-3.72E-01	1.20E-02	-3.40E-01
7970	GSM282505	GSE11121	Breast Cancer	Breast	4.13E-01	-7.44E-02	3.90E-01	1.57E-01
7971	GSM282506	GSE11121	Breast Cancer	Breast	4.60E-03	-1.84E-01	2.27E-01	-1.97E-01
7972	GSM282507	GSE11121	Breast Cancer	Breast	6.96E-01	4.76E-02	2.80E-01	1.82E-01
7973	GSM282508	GSE11121	Breast Cancer	Breast	2.82E-04	2.26E-01	3.85E-03	3.81E-01
7974	GSM282509	GSE11121	Breast Cancer	Breast	3.42E-03	-1.88E-01	2.49E-01	1.90E-01
7975	GSM282510	GSE11121	Breast Cancer	Breast	8.52E-04	-2.10E-01	8.88E-02	-2.51E-01
7976	GSM282511	GSE11121	Breast Cancer	Breast	1.83E-01	1.03E-01	8.88E-02	2.51E-01
7977	GSM282512	GSE11121	Breast Cancer	Breast	3.84E-02	1.43E-01	3.81E-02	2.92E-01
7978	GSM282513	GSE11121	Breast Cancer	Breast	4.34E-03	1.85E-01	1.94E-03	4.04E-01
7979	GSM282514	GSE11121	Breast Cancer	Breast	2.38E-03	-1.94E-01	1.98E-01	-2.06E-01
7980	GSM282515	GSE11121	Breast Cancer	Breast	4.79E-02	-1.38E-01	2.06E-01	-2.03E-01
7981	GSM282516	GSE11121	Breast Cancer	Breast	2.07E-02	1.56E-01	1.42E-02	3.33E-01
7982	GSM282517	GSE11121	Breast Cancer	Breast	1.74E-05	2.62E-01	1.69E-04	4.76E-01
7983	GSM282518	GSE11121	Breast Cancer	Breast	7.36E-05	-2.44E-01	2.49E-01	1.90E-01
7984	GSM282519	GSE11121	Breast Cancer	Breast	1.97E-03	-1.97E-01	1.84E-01	-2.10E-01
7985	GSM282520	GSE11121	Breast Cancer	Breast	4.87E-03	-1.83E-01	3.61E-01	-1.63E-01
7986	GSM282521	GSE11121	Breast Cancer	Breast	1.35E-03	2.03E-01	4.89E-03	3.73E-01
7987	GSM282522	GSE11121	Breast Cancer	Breast	3.63E-03	-1.88E-01	6.23E-02	-2.69E-01

7988	GSM282523	GSE11121	Breast Cancer	Breast	3.62E-11	3.88E-01	1.50E-05	5.38E-01
7989	GSM282524	GSE11121	Breast Cancer	Breast	2.72E-01	-9.03E-02	3.11E-01	1.75E-01
7990	GSM282525	GSE11121	Breast Cancer	Breast	1.25E-06	-2.92E-01	1.83E-02	-3.23E-01
7991	GSM282526	GSE11121	Breast Cancer	Breast	2.80E-01	8.93E-02	3.11E-02	3.01E-01
7992	GSM282527	GSE11121	Breast Cancer	Breast	8.10E-03	1.74E-01	1.18E-02	3.40E-01
7993	GSM282528	GSE11121	Breast Cancer	Breast	8.59E-05	2.42E-01	7.75E-03	3.56E-01
7994	GSM282529	GSE11121	Breast Cancer	Breast	2.52E-02	-1.52E-01	3.81E-01	1.59E-01
7995	GSM282530	GSE11121	Breast Cancer	Breast	9.63E-02	1.21E-01	5.24E-02	2.77E-01
7996	GSM282531	GSE11121	Breast Cancer	Breast	3.04E-03	1.90E-01	3.92E-03	3.80E-01
7997	GSM282532	GSE11121	Breast Cancer	Breast	3.04E-03	1.90E-01	6.06E-03	3.65E-01
7998	GSM282533	GSE11121	Breast Cancer	Breast	2.69E-03	-1.92E-01	1.77E-01	-2.13E-01
7999	GSM282534	GSE11121	Breast Cancer	Breast	1.14E-05	2.67E-01	4.07E-04	4.51E-01
8000	GSM282535	GSE11121	Breast Cancer	Breast	3.21E-37	7.25E-01	2.53E-16	9.68E-01
8001	GSM282536	GSE11121	Breast Cancer	Breast	9.54E-03	1.71E-01	3.92E-03	3.80E-01
8002	GSM282537	GSE11121	Breast Cancer	Breast	6.00E-14	-4.37E-01	1.33E-06	-5.94E-01
8003	GSM282538	GSE11121	Breast Cancer	Breast	3.54E-10	-3.69E-01	5.52E-04	-4.43E-01
8004	GSM282539	GSE11121	Breast Cancer	Breast	7.11E-10	-3.63E-01	1.85E-04	-4.74E-01
8005	GSM282540	GSE11121	Breast Cancer	Breast	5.75E-06	2.75E-01	2.87E-05	5.23E-01
8006	GSM282541	GSE11121	Breast Cancer	Breast	7.01E-02	1.29E-01	1.16E-02	3.41E-01
8007	GSM282542	GSE11121	Breast Cancer	Breast	4.33E-01	7.24E-02	3.07E-02	3.02E-01
8008	GSM282543	GSE11121	Breast Cancer	Breast	1.97E-10	-3.74E-01	2.87E-05	-5.23E-01
8009	GSM282544	GSE11121	Breast Cancer	Breast	2.28E-01	-9.62E-02	6.23E-02	2.69E-01
8010	GSM282545	GSE11121	Breast Cancer	Breast	4.39E-02	-1.40E-01	2.06E-01	-2.03E-01
8011	GSM282546	GSE11121	Breast Cancer	Breast	2.07E-02	1.56E-01	3.85E-03	3.81E-01
8012	GSM282547	GSE11121	Breast Cancer	Breast	1.46E-04	2.35E-01	5.23E-05	5.07E-01
8013	GSM282548	GSE11121	Breast Cancer	Breast	3.05E-02	-1.48E-01	3.93E-01	-1.56E-01
8014	GSM282549	GSE11121	Breast Cancer	Breast	5.37E-07	-3.01E-01	1.77E-02	-3.24E-01
8015	GSM282550	GSE11121	Breast Cancer	Breast	2.96E-01	8.73E-02	2.68E-02	3.07E-01
8016	GSM282551	GSE11121	Breast Cancer	Breast	2.40E-02	1.53E-01	1.24E-02	3.38E-01
8017	GSM282552	GSE11121	Breast Cancer	Breast	2.49E-01	-9.33E-02	3.24E-01	1.71E-01
8018	GSM282553	GSE11121	Breast Cancer	Breast	5.94E-02	1.33E-01	5.53E-02	2.75E-01
8019	GSM282554	GSE11121	Breast Cancer	Breast	2.49E-01	9.33E-02	6.23E-02	2.69E-01
8020	GSM282555	GSE11121	Breast Cancer	Breast	2.29E-02	-1.54E-01	3.81E-01	-1.59E-01
8021	GSM282556	GSE11121	Breast Cancer	Breast	2.08E-01	-9.92E-02	1.51E-01	2.22E-01
8022	GSM282557	GSE11121	Breast Cancer	Breast	2.80E-01	-8.93E-02	1.51E-01	2.22E-01
8023	GSM282558	GSE11121	Breast Cancer	Breast	9.65E-06	-2.69E-01	1.00E-01	-2.45E-01
8024	GSM282559	GSE11121	Breast Cancer	Breast	1.18E-03	2.05E-01	2.49E-03	3.96E-01
8025	GSM282560	GSE11121	Breast Cancer	Breast	5.36E-01	-6.25E-02	8.77E-02	2.52E-01
8026	GSM282561	GSE11121	Breast Cancer	Breast	3.19E-02	-1.47E-01	1.98E-01	-2.06E-01
8027	GSM282562	GSE11121	Breast Cancer	Breast	4.98E-05	-2.49E-01	5.31E-02	-2.77E-01
8028	GSM282563	GSE11121	Breast Cancer	Breast	6.51E-04	2.14E-01	1.53E-03	4.11E-01
8029	GSM282564	GSE11121	Breast Cancer	Breast	1.89E-05	2.61E-01	5.40E-04	4.43E-01
8030	GSM282565	GSE11121	Breast Cancer	Breast	1.54E-01	1.08E-01	5.31E-02	2.77E-01
8031	GSM282566	GSE11121	Breast Cancer	Breast	1.95E-01	-1.01E-01	2.00E-01	2.05E-01
8032	GSM282567	GSE11121	Breast Cancer	Breast	1.47E-05	-2.64E-01	6.23E-02	-2.69E-01
8033	GSM282568	GSE11121	Breast Cancer	Breast	8.30E-08	-3.19E-01	4.98E-03	-3.72E-01
8034	GSM282569	GSE11121	Breast Cancer	Breast	4.03E-01	7.54E-02	1.15E-01	2.37E-01
8035	GSM282570	GSE11121	Breast Cancer	Breast	6.97E-04	-2.13E-01	6.28E-03	-3.64E-01
8036	GSM282571	GSE11121	Breast Cancer	Breast	6.28E-06	2.74E-01	1.33E-06	5.94E-01
8037	GSM282572	GSE11121	Breast Cancer	Breast	3.32E-07	-3.06E-01	3.11E-02	-3.01E-01
8038	GSM540108	GSE21653	Breast Cancer	Breast	7.97E-10	-3.62E-01	1.22E-03	-4.18E-01
8039	GSM540109	GSE21653	Breast Cancer	Breast	1.71E-15	-4.61E-01	3.02E-07	-6.26E-01
8040	GSM540110	GSE21653	Breast Cancer	Breast	1.50E-06	-2.90E-01	5.28E-04	-4.44E-01
8041	GSM540111	GSE21653	Breast Cancer	Breast	3.34E-02	-1.46E-01	8.45E-02	2.54E-01
8042	GSM540112	GSE21653	Breast Cancer	Breast	5.39E-05	-2.48E-01	2.68E-02	-3.07E-01
8043	GSM540113	GSE21653	Breast Cancer	Breast	1.74E-05	2.62E-01	9.37E-05	4.92E-01
8044	GSM540114	GSE21653	Breast Cancer	Breast	5.94E-02	-1.33E-01	9.90E-02	2.46E-01
8045	GSM540115	GSE21653	Breast Cancer	Breast	1.35E-04	2.36E-01	1.69E-04	4.76E-01
8046	GSM540116	GSE21653	Breast Cancer	Breast	5.39E-05	-2.48E-01	1.49E-02	-3.31E-01
8047	GSM540117	GSE21653	Breast Cancer	Breast	1.79E-02	-1.59E-01	1.54E-01	-2.21E-01
8048	GSM540118	GSE21653	Breast Cancer	Breast	3.04E-03	1.90E-01	4.98E-03	3.72E-01
8049	GSM540119	GSE21653	Breast Cancer	Breast	1.00E-01	-1.20E-01	2.49E-01	1.90E-01
8050	GSM540120	GSE21653	Breast Cancer	Breast	5.01E-02	1.37E-01	3.70E-02	2.93E-01
8051	GSM540121	GSE21653	Breast Cancer	Breast	3.23E-03	1.89E-01	3.85E-03	3.81E-01
8052	GSM540122	GSE21653	Breast Cancer	Breast	1.25E-02	-1.66E-01	2.00E-01	-2.05E-01
8053	GSM540123	GSE21653	Breast Cancer	Breast	3.05E-02	-1.48E-01	1.34E-01	2.29E-01
8054	GSM540124	GSE21653	Breast Cancer	Breast	3.23E-03	-1.89E-01	4.64E-01	-1.42E-01
8055	GSM540125	GSE21653	Breast Cancer	Breast	4.20E-02	1.41E-01	9.85E-03	3.47E-01
8056	GSM540126	GSE21653	Breast Cancer	Breast	2.62E-05	-2.57E-01	3.11E-02	-3.01E-01
8057	GSM540127	GSE21653	Breast Cancer	Breast	1.08E-01	1.18E-01	4.38E-02	2.86E-01
8058	GSM540128	GSE21653	Breast Cancer	Breast	6.48E-03	-1.78E-01	3.49E-01	-1.66E-01
8059	GSM540129	GSE21653	Breast Cancer	Breast	5.69E-02	1.34E-01	3.12E-04	4.59E-01
8060	GSM540130	GSE21653	Breast Cancer	Breast	4.87E-12	4.04E-01	7.93E-09	6.98E-01
8061	GSM540131	GSE21653	Breast Cancer	Breast	8.86E-06	-2.70E-01	4.57E-02	-2.84E-01

8062	GSM540132	GSE21653	Breast Cancer	Breast	2.23E-05	2.59E-01	9.83E-05	4.91E-01
8063	GSM540133	GSE21653	Breast Cancer	Breast	4.13E-01	-7.44E-02	1.98E-01	2.06E-01
8064	GSM540134	GSE21653	Breast Cancer	Breast	3.84E-02	-1.43E-01	3.84E-01	-1.58E-01
8065	GSM540135	GSE21653	Breast Cancer	Breast	1.35E-03	-2.03E-01	1.34E-01	-2.29E-01
8066	GSM540136	GSE21653	Breast Cancer	Breast	4.63E-01	6.94E-02	8.77E-02	2.52E-01
8067	GSM540137	GSE21653	Breast Cancer	Breast	2.42E-01	-9.42E-02	1.14E-01	2.38E-01
8068	GSM540138	GSE21653	Breast Cancer	Breast	7.67E-03	-1.75E-01	5.93E-01	1.17E-01
8069	GSM540139	GSE21653	Breast Cancer	Breast	3.29E-08	-3.28E-01	3.19E-03	-3.87E-01
8070	GSM540140	GSE21653	Breast Cancer	Breast	2.91E-02	1.49E-01	3.85E-03	3.81E-01
8071	GSM540141	GSE21653	Breast Cancer	Breast	6.73E-02	-1.30E-01	5.86E-01	1.18E-01
8072	GSM540142	GSE21653	Breast Cancer	Breast	7.97E-10	-3.62E-01	6.17E-03	-3.64E-01
8073	GSM540143	GSE21653	Breast Cancer	Breast	2.86E-03	1.91E-01	3.08E-03	3.89E-01
8074	GSM540144	GSE21653	Breast Cancer	Breast	2.62E-05	-2.57E-01	6.56E-02	-2.67E-01
8075	GSM540145	GSE21653	Breast Cancer	Breast	8.10E-03	-1.74E-01	3.81E-01	1.59E-01
8076	GSM540146	GSE21653	Breast Cancer	Breast	2.52E-02	1.52E-01	3.07E-02	3.02E-01
8077	GSM540147	GSE21653	Breast Cancer	Breast	5.47E-03	-1.81E-01	2.83E-01	-1.82E-01
8078	GSM540148	GSE21653	Breast Cancer	Breast	7.40E-17	-4.82E-01	3.90E-06	-5.70E-01
8079	GSM540149	GSE21653	Breast Cancer	Breast	1.27E-08	-3.37E-01	3.32E-03	-3.86E-01
8080	GSM540150	GSE21653	Breast Cancer	Breast	1.18E-03	2.05E-01	1.17E-03	4.20E-01
8081	GSM540151	GSE21653	Breast Cancer	Breast	3.38E-01	8.23E-02	1.73E-01	2.14E-01
8082	GSM540152	GSE21653	Breast Cancer	Breast	4.95E-04	-2.18E-01	6.39E-02	-2.68E-01
8083	GSM540153	GSE21653	Breast Cancer	Breast	1.74E-03	1.99E-01	3.98E-04	4.52E-01
8084	GSM540154	GSE21653	Breast Cancer	Breast	3.56E-01	8.04E-02	4.57E-02	2.84E-01
8085	GSM540155	GSE21653	Breast Cancer	Breast	4.02E-04	2.21E-01	6.98E-04	4.36E-01
8086	GSM540156	GSE21653	Breast Cancer	Breast	1.31E-02	1.65E-01	6.83E-04	4.36E-01
8087	GSM540157	GSE21653	Breast Cancer	Breast	7.67E-03	1.75E-01	1.90E-03	4.04E-01
8088	GSM540158	GSE21653	Breast Cancer	Breast	3.04E-03	1.90E-01	3.85E-03	3.81E-01
8089	GSM540159	GSE21653	Breast Cancer	Breast	2.80E-01	-8.93E-02	9.90E-02	2.46E-01
8090	GSM540160	GSE21653	Breast Cancer	Breast	6.19E-02	-1.32E-01	7.27E-02	2.62E-01
8091	GSM540161	GSE21653	Breast Cancer	Breast	1.18E-02	-1.67E-01	4.45E-02	-2.85E-01
8092	GSM540162	GSE21653	Breast Cancer	Breast	1.25E-01	-1.14E-01	8.66E-02	2.53E-01
8093	GSM540163	GSE21653	Breast Cancer	Breast	1.12E-02	1.68E-01	3.76E-02	2.93E-01
8094	GSM540164	GSE21653	Breast Cancer	Breast	8.91E-02	1.23E-01	4.38E-02	2.86E-01
8095	GSM540165	GSE21653	Breast Cancer	Breast	4.59E-02	-1.39E-01	2.54E-01	-1.89E-01
8096	GSM540166	GSE21653	Breast Cancer	Breast	2.17E-08	-3.32E-01	3.13E-03	-3.88E-01
8097	GSM540167	GSE21653	Breast Cancer	Breast	1.44E-03	2.02E-01	3.26E-04	4.58E-01
8098	GSM540168	GSE21653	Breast Cancer	Breast	1.31E-02	-1.65E-01	2.33E-01	-1.95E-01
8099	GSM540169	GSE21653	Breast Cancer	Breast	2.14E-01	-9.82E-02	1.96E-01	2.06E-01
8100	GSM540170	GSE21653	Breast Cancer	Breast	2.01E-01	1.00E-01	3.11E-01	1.75E-01
8101	GSM540171	GSE21653	Breast Cancer	Breast	3.23E-03	-1.89E-01	8.56E-02	-2.53E-01
8102	GSM540172	GSE21653	Breast Cancer	Breast	2.41E-08	-3.31E-01	5.38E-02	-2.76E-01
8103	GSM540173	GSE21653	Breast Cancer	Breast	2.53E-03	-1.93E-01	1.51E-01	2.22E-01
8104	GSM540174	GSE21653	Breast Cancer	Breast	1.01E-02	-1.70E-01	3.14E-01	-1.74E-01
8105	GSM540175	GSE21653	Breast Cancer	Breast	8.24E-02	-1.25E-01	1.51E-01	2.22E-01
8106	GSM540176	GSE21653	Breast Cancer	Breast	2.88E-01	-8.83E-02	1.14E-01	2.38E-01
8107	GSM540177	GSE21653	Breast Cancer	Breast	2.96E-01	8.73E-02	1.96E-01	2.06E-01
8108	GSM540178	GSE21653	Breast Cancer	Breast	2.18E-02	-1.55E-01	2.54E-01	1.89E-01
8109	GSM540179	GSE21653	Breast Cancer	Breast	8.10E-03	-1.74E-01	1.79E-01	-2.12E-01
8110	GSM540180	GSE21653	Breast Cancer	Breast	1.38E-02	1.64E-01	2.14E-02	3.17E-01
8111	GSM540181	GSE21653	Breast Cancer	Breast	4.02E-04	2.21E-01	3.32E-03	3.86E-01
8112	GSM540182	GSE21653	Breast Cancer	Breast	1.18E-02	-1.67E-01	5.89E-01	1.17E-01
8113	GSM540183	GSE21653	Breast Cancer	Breast	2.69E-03	-1.92E-01	8.45E-02	2.54E-01
8114	GSM540184	GSE21653	Breast Cancer	Breast	5.57E-01	6.05E-02	2.25E-01	1.97E-01
8115	GSM540185	GSE21653	Breast Cancer	Breast	9.26E-02	1.22E-01	1.52E-01	2.22E-01
8116	GSM540186	GSE21653	Breast Cancer	Breast	1.31E-02	1.65E-01	2.61E-02	3.09E-01
8117	GSM540187	GSE21653	Breast Cancer	Breast	4.42E-06	2.78E-01	1.07E-05	5.47E-01
8118	GSM540188	GSE21653	Breast Cancer	Breast	9.63E-02	-1.21E-01	3.11E-01	1.75E-01
8119	GSM540189	GSE21653	Breast Cancer	Breast	3.84E-01	7.74E-02	6.56E-02	2.67E-01
8120	GSM540190	GSE21653	Breast Cancer	Breast	8.10E-03	1.74E-01	9.85E-03	3.47E-01
8121	GSM540191	GSE21653	Breast Cancer	Breast	9.26E-02	-1.22E-01	3.81E-01	1.59E-01
8122	GSM540192	GSE21653	Breast Cancer	Breast	4.31E-04	2.20E-01	2.45E-03	3.96E-01
8123	GSM540193	GSE21653	Breast Cancer	Breast	2.25E-07	-3.10E-01	1.18E-02	-3.40E-01
8124	GSM540194	GSE21653	Breast Cancer	Breast	4.23E-01	7.34E-02	4.38E-02	2.86E-01
8125	GSM540195	GSE21653	Breast Cancer	Breast	2.86E-03	1.91E-01	7.28E-04	4.34E-01
8126	GSM540196	GSE21653	Breast Cancer	Breast	3.62E-05	-2.53E-01	1.54E-01	-2.21E-01
8127	GSM540197	GSE21653	Breast Cancer	Breast	2.97E-08	-3.29E-01	1.49E-02	-3.31E-01
8128	GSM540198	GSE21653	Breast Cancer	Breast	4.59E-02	-1.39E-01	3.55E-01	-1.64E-01
8129	GSM540199	GSE21653	Breast Cancer	Breast	5.78E-01	-5.85E-02	2.11E-02	3.17E-01
8130	GSM540200	GSE21653	Breast Cancer	Breast	4.53E-01	-7.04E-02	5.00E-01	1.35E-01
8131	GSM540201	GSE21653	Breast Cancer	Breast	9.11E-04	-2.09E-01	1.03E-01	-2.44E-01
8132	GSM540202	GSE21653	Breast Cancer	Breast	1.30E-01	1.13E-01	1.14E-01	2.38E-01
8133	GSM540203	GSE21653	Breast Cancer	Breast	5.01E-02	1.37E-01	9.52E-03	3.49E-01
8134	GSM540204	GSE21653	Breast Cancer	Breast	1.38E-02	-1.64E-01	2.95E-01	-1.78E-01
8135	GSM540205	GSE21653	Breast Cancer	Breast	1.12E-01	1.17E-01	3.07E-02	3.02E-01

8136	GSM540206	GSE21653	Breast Cancer	Breast	1.89E-01	-1.02E-01	1.17E-01	2.37E-01
8137	GSM540207	GSE21653	Breast Cancer	Breast	1.70E-02	-1.60E-01	4.39E-01	-1.47E-01
8138	GSM540208	GSE21653	Breast Cancer	Breast	1.24E-05	2.66E-01	2.11E-05	5.30E-01
8139	GSM540209	GSE21653	Breast Cancer	Breast	4.20E-02	1.41E-01	3.70E-02	2.93E-01
8140	GSM540210	GSE21653	Breast Cancer	Breast	1.49E-01	-1.09E-01	2.49E-01	1.90E-01
8141	GSM540211	GSE21653	Breast Cancer	Breast	4.13E-01	7.44E-02	1.51E-01	2.22E-01
8142	GSM540212	GSE21653	Breast Cancer	Breast	1.08E-01	-1.18E-01	3.11E-01	1.75E-01
8143	GSM540213	GSE21653	Breast Cancer	Breast	1.97E-02	-1.57E-01	4.61E-01	-1.42E-01
8144	GSM540214	GSE21653	Breast Cancer	Breast	7.30E-02	1.28E-01	3.07E-02	3.02E-01
8145	GSM540215	GSE21653	Breast Cancer	Breast	4.79E-02	1.38E-01	6.39E-03	3.63E-01
8146	GSM540216	GSE21653	Breast Cancer	Breast	9.63E-02	1.21E-01	7.27E-02	2.62E-01
8147	GSM540217	GSE21653	Breast Cancer	Breast	3.74E-04	2.22E-01	1.47E-03	4.13E-01
8148	GSM540218	GSE21653	Breast Cancer	Breast	1.08E-01	1.18E-01	3.11E-02	3.01E-01
8149	GSM540219	GSE21653	Breast Cancer	Breast	1.35E-03	2.03E-01	9.85E-03	3.47E-01
8150	GSM540220	GSE21653	Breast Cancer	Breast	8.57E-02	-1.24E-01	5.03E-01	1.34E-01
8151	GSM540221	GSE21653	Breast Cancer	Breast	2.91E-02	-1.49E-01	2.83E-01	-1.82E-01
8152	GSM540222	GSE21653	Breast Cancer	Breast	1.26E-09	-3.58E-01	1.31E-04	-4.83E-01
8153	GSM540223	GSE21653	Breast Cancer	Breast	8.10E-03	1.74E-01	9.60E-05	4.91E-01
8154	GSM540224	GSE21653	Breast Cancer	Breast	2.91E-02	-1.49E-01	2.25E-01	1.97E-01
8155	GSM540225	GSE21653	Breast Cancer	Breast	1.04E-03	-2.07E-01	3.61E-01	-1.63E-01
8156	GSM540226	GSE21653	Breast Cancer	Breast	2.14E-01	-9.82E-02	2.49E-01	1.90E-01
8157	GSM540227	GSE21653	Breast Cancer	Breast	1.58E-08	-3.35E-01	6.62E-03	-3.62E-01
8158	GSM540228	GSE21653	Breast Cancer	Breast	6.86E-03	-1.77E-01	1.40E-01	-2.27E-01
8159	GSM540229	GSE21653	Breast Cancer	Breast	1.47E-05	-2.64E-01	8.88E-02	-2.51E-01
8160	GSM540230	GSE21653	Breast Cancer	Breast	9.26E-02	-1.22E-01	2.80E-01	1.82E-01
8161	GSM540231	GSE21653	Breast Cancer	Breast	1.12E-09	-3.59E-01	9.76E-04	-4.25E-01
8162	GSM540232	GSE21653	Breast Cancer	Breast	4.31E-09	3.47E-01	1.83E-06	5.87E-01
8163	GSM540233	GSE21653	Breast Cancer	Breast	1.25E-01	1.14E-01	1.96E-01	2.06E-01
8164	GSM540234	GSE21653	Breast Cancer	Breast	1.47E-05	-2.64E-01	3.11E-02	-3.01E-01
8165	GSM540235	GSE21653	Breast Cancer	Breast	1.05E-05	2.68E-01	1.46E-05	5.39E-01
8166	GSM540236	GSE21653	Breast Cancer	Breast	2.77E-02	1.50E-01	5.24E-02	2.77E-01
8167	GSM540237	GSE21653	Breast Cancer	Breast	2.25E-07	3.10E-01	1.04E-05	5.47E-01
8168	GSM540238	GSE21653	Breast Cancer	Breast	2.17E-08	3.32E-01	2.79E-05	5.23E-01
8169	GSM540239	GSE21653	Breast Cancer	Breast	2.07E-02	1.56E-01	5.24E-02	2.77E-01
8170	GSM540240	GSE21653	Breast Cancer	Breast	4.59E-02	1.39E-01	3.07E-02	3.02E-01
8171	GSM540241	GSE21653	Breast Cancer	Breast	7.60E-02	-1.27E-01	5.00E-01	1.35E-01
8172	GSM540242	GSE21653	Breast Cancer	Breast	1.34E-01	1.12E-01	2.27E-02	3.14E-01
8173	GSM540243	GSE21653	Breast Cancer	Breast	1.08E-04	-2.39E-01	3.11E-02	-3.01E-01
8174	GSM540244	GSE21653	Breast Cancer	Breast	2.64E-01	9.13E-02	9.90E-02	2.46E-01
8175	GSM540245	GSE21653	Breast Cancer	Breast	1.00E-01	1.20E-01	1.50E-03	4.12E-01
8176	GSM540246	GSE21653	Breast Cancer	Breast	1.08E-04	-2.39E-01	2.24E-02	-3.15E-01
8177	GSM540247	GSE21653	Breast Cancer	Breast	5.39E-05	-2.48E-01	1.00E-01	-2.45E-01
8178	GSM540248	GSE21653	Breast Cancer	Breast	1.06E-02	-1.69E-01	5.86E-01	-1.18E-01
8179	GSM540249	GSE21653	Breast Cancer	Breast	2.69E-03	1.92E-01	3.85E-03	3.81E-01
8180	GSM540250	GSE21653	Breast Cancer	Breast	2.18E-02	-1.55E-01	3.19E-01	-1.73E-01
8181	GSM540251	GSE21653	Breast Cancer	Breast	4.09E-03	-1.86E-01	4.29E-01	-1.49E-01
8182	GSM540252	GSE21653	Breast Cancer	Breast	3.10E-06	2.82E-01	6.83E-04	4.36E-01
8183	GSM540253	GSE21653	Breast Cancer	Breast	1.65E-01	1.06E-01	6.39E-02	2.68E-01
8184	GSM540254	GSE21653	Breast Cancer	Breast	1.31E-02	1.65E-01	3.07E-02	3.02E-01
8185	GSM540255	GSE21653	Breast Cancer	Breast	4.01E-02	1.42E-01	2.57E-02	3.09E-01
8186	GSM540256	GSE21653	Breast Cancer	Breast	6.30E-05	2.46E-01	7.13E-04	4.35E-01
8187	GSM540257	GSE21653	Breast Cancer	Breast	4.83E-01	-6.75E-02	1.98E-01	2.06E-01
8188	GSM540258	GSE21653	Breast Cancer	Breast	2.38E-03	-1.94E-01	1.15E-01	-2.37E-01
8189	GSM540259	GSE21653	Breast Cancer	Breast	5.02E-10	3.66E-01	6.21E-07	6.11E-01
8190	GSM540260	GSE21653	Breast Cancer	Breast	6.97E-04	2.13E-01	3.98E-04	4.52E-01
8191	GSM540261	GSE21653	Breast Cancer	Breast	1.06E-02	1.69E-01	2.59E-03	3.94E-01
8192	GSM540262	GSE21653	Breast Cancer	Breast	1.38E-02	-1.64E-01	3.19E-01	-1.73E-01
8193	GSM540263	GSE21653	Breast Cancer	Breast	8.24E-02	1.25E-01	1.05E-01	2.43E-01
8194	GSM540264	GSE21653	Breast Cancer	Breast	1.77E-01	-1.04E-01	3.19E-01	1.73E-01
8195	GSM540265	GSE21653	Breast Cancer	Breast	3.94E-01	7.64E-02	1.04E-01	2.43E-01
8196	GSM540266	GSE21653	Breast Cancer	Breast	5.36E-09	-3.45E-01	5.28E-04	-4.44E-01
8197	GSM540267	GSE21653	Breast Cancer	Breast	2.35E-01	-9.52E-02	4.70E-01	1.40E-01
8198	GSM540268	GSE21653	Breast Cancer	Breast	2.80E-01	-8.93E-02	2.22E-01	1.98E-01
8199	GSM540269	GSE21653	Breast Cancer	Breast	3.50E-02	-1.45E-01	4.39E-01	-1.47E-01
8200	GSM540270	GSE21653	Breast Cancer	Breast	1.71E-01	1.05E-01	2.49E-01	1.90E-01
8201	GSM540271	GSE21653	Breast Cancer	Breast	4.01E-02	1.42E-01	1.01E-01	2.44E-01
8202	GSM540272	GSE21653	Breast Cancer	Breast	4.59E-02	1.39E-01	1.01E-01	2.44E-01
8203	GSM540273	GSE21653	Breast Cancer	Breast	3.23E-03	1.89E-01	4.89E-03	3.73E-01
8204	GSM540274	GSE21653	Breast Cancer	Breast	1.11E-03	2.06E-01	2.98E-04	4.60E-01
8205	GSM540275	GSE21653	Breast Cancer	Breast	1.85E-03	1.98E-01	9.68E-03	3.48E-01
8206	GSM540276	GSE21653	Breast Cancer	Breast	2.49E-01	-9.33E-02	6.06E-01	1.14E-01
8207	GSM540277	GSE21653	Breast Cancer	Breast	8.57E-02	-1.24E-01	2.04E-01	2.04E-01
8208	GSM540278	GSE21653	Breast Cancer	Breast	2.24E-03	-1.95E-01	7.36E-02	-2.61E-01
8209	GSM540279	GSE21653	Breast Cancer	Breast	7.97E-04	-2.11E-01	1.04E-01	-2.43E-01

8210	GSM540280	GSE21653	Breast Cancer	Breast	2.27E-04	-2.29E-01	8.99E-02	-2.51E-01
8211	GSM540281	GSE21653	Breast Cancer	Breast	1.53E-02	1.62E-01	3.70E-02	2.93E-01
8212	GSM540282	GSE21653	Breast Cancer	Breast	1.01E-02	-1.70E-01	6.27E-01	1.10E-01
8213	GSM540283	GSE21653	Breast Cancer	Breast	4.88E-07	-3.02E-01	7.94E-02	-2.57E-01
8214	GSM540284	GSE21653	Breast Cancer	Breast	1.18E-03	-2.05E-01	1.15E-01	-2.37E-01
8215	GSM540285	GSE21653	Breast Cancer	Breast	3.75E-01	7.84E-02	1.15E-01	2.37E-01
8216	GSM540286	GSE21653	Breast Cancer	Breast	5.39E-05	-2.48E-01	7.36E-02	-2.61E-01
8217	GSM540287	GSE21653	Breast Cancer	Breast	3.63E-03	-1.88E-01	3.19E-01	-1.73E-01
8218	GSM540288	GSE21653	Breast Cancer	Breast	3.84E-01	-7.74E-02	1.57E-01	2.20E-01
8219	GSM540289	GSE21653	Breast Cancer	Breast	3.65E-08	-3.27E-01	1.53E-03	-4.11E-01
8220	GSM540290	GSE21653	Breast Cancer	Breast	3.06E-15	4.57E-01	9.57E-08	6.50E-01
8221	GSM540291	GSE21653	Breast Cancer	Breast	3.34E-05	-2.54E-01	5.38E-02	-2.76E-01
8222	GSM540292	GSE21653	Breast Cancer	Breast	2.86E-03	1.91E-01	6.39E-03	3.63E-01
8223	GSM540293	GSE21653	Breast Cancer	Breast	1.57E-04	2.34E-01	8.98E-04	4.28E-01
8224	GSM540294	GSE21653	Breast Cancer	Breast	1.97E-03	1.97E-01	1.25E-03	4.18E-01
8225	GSM540295	GSE21653	Breast Cancer	Breast	8.59E-05	-2.42E-01	3.92E-02	-2.91E-01
8226	GSM540296	GSE21653	Breast Cancer	Breast	1.34E-01	1.12E-01	7.27E-02	2.62E-01
8227	GSM540297	GSE21653	Breast Cancer	Breast	5.68E-04	-2.16E-01	1.52E-01	-2.22E-01
8228	GSM540298	GSE21653	Breast Cancer	Breast	2.82E-04	-2.26E-01	7.65E-02	-2.59E-01
8229	GSM540299	GSE21653	Breast Cancer	Breast	7.96E-05	-2.43E-01	1.00E-01	-2.45E-01
8230	GSM540300	GSE21653	Breast Cancer	Breast	2.11E-04	2.30E-01	8.79E-04	4.29E-01
8231	GSM540301	GSE21653	Breast Cancer	Breast	1.49E-01	1.09E-01	6.15E-02	2.70E-01
8232	GSM540302	GSE21653	Breast Cancer	Breast	3.04E-01	-8.63E-02	6.31E-02	2.69E-01
8233	GSM540303	GSE21653	Breast Cancer	Breast	2.07E-02	-1.56E-01	5.31E-02	-2.77E-01
8234	GSM540304	GSE21653	Breast Cancer	Breast	1.63E-03	-2.00E-01	3.22E-01	-1.72E-01
8235	GSM540305	GSE21653	Breast Cancer	Breast	1.97E-12	-4.11E-01	3.12E-04	-4.59E-01
8236	GSM540306	GSE21653	Breast Cancer	Breast	1.21E-01	1.15E-01	1.20E-02	3.40E-01
8237	GSM540307	GSE21653	Breast Cancer	Breast	1.49E-01	-1.09E-01	1.15E-01	2.37E-01
8238	GSM540308	GSE21653	Breast Cancer	Breast	4.62E-04	2.19E-01	5.52E-04	4.43E-01
8239	GSM540309	GSE21653	Breast Cancer	Breast	3.54E-10	-3.69E-01	6.28E-03	-3.64E-01
8240	GSM540310	GSE21653	Breast Cancer	Breast	9.63E-02	1.21E-01	2.14E-02	3.17E-01
8241	GSM540311	GSE21653	Breast Cancer	Breast	1.49E-01	-1.09E-01	3.11E-01	1.75E-01
8242	GSM540312	GSE21653	Breast Cancer	Breast	1.60E-01	1.07E-01	1.34E-01	2.29E-01
8243	GSM540313	GSE21653	Breast Cancer	Breast	1.34E-01	1.12E-01	4.38E-02	2.86E-01
8244	GSM540314	GSE21653	Breast Cancer	Breast	1.18E-02	1.67E-01	8.79E-04	4.29E-01
8245	GSM540315	GSE21653	Breast Cancer	Breast	4.13E-01	-7.44E-02	6.15E-02	2.70E-01
8246	GSM540316	GSE21653	Breast Cancer	Breast	2.82E-04	-2.26E-01	2.56E-01	-1.89E-01
8247	GSM540317	GSE21653	Breast Cancer	Breast	1.35E-03	-2.03E-01	1.98E-01	-2.06E-01
8248	GSM540318	GSE21653	Breast Cancer	Breast	1.01E-02	-1.70E-01	2.56E-01	-1.89E-01
8249	GSM540319	GSE21653	Breast Cancer	Breast	2.40E-02	-1.53E-01	3.14E-01	-1.74E-01
8250	GSM540320	GSE21653	Breast Cancer	Breast	1.26E-09	-3.58E-01	9.17E-04	-4.27E-01
8251	GSM540321	GSE21653	Breast Cancer	Breast	1.16E-01	-1.16E-01	1.98E-01	-2.06E-01
8252	GSM540322	GSE21653	Breast Cancer	Breast	1.12E-02	-1.68E-01	3.55E-01	-1.64E-01
8253	GSM540323	GSE21653	Breast Cancer	Breast	2.27E-04	-2.29E-01	6.31E-02	-2.69E-01
8254	GSM540324	GSE21653	Breast Cancer	Breast	4.01E-02	-1.42E-01	3.55E-01	-1.64E-01
8255	GSM540325	GSE21653	Breast Cancer	Breast	4.83E-01	-6.75E-02	1.34E-01	2.29E-01
8256	GSM540326	GSE21653	Breast Cancer	Breast	6.08E-04	-2.15E-01	1.77E-01	-2.13E-01
8257	GSM540327	GSE21653	Breast Cancer	Breast	7.30E-02	1.28E-01	4.51E-02	2.84E-01
8258	GSM540328	GSE21653	Breast Cancer	Breast	3.19E-02	-1.47E-01	2.27E-01	-1.97E-01
8259	GSM540329	GSE21653	Breast Cancer	Breast	3.04E-01	-8.63E-02	1.14E-01	2.38E-01
8260	GSM540330	GSE21653	Breast Cancer	Breast	7.36E-05	-2.44E-01	1.46E-02	-3.32E-01
8261	GSM540331	GSE21653	Breast Cancer	Breast	3.10E-06	2.82E-01	1.31E-04	4.83E-01
8262	GSM540332	GSE21653	Breast Cancer	Breast	1.21E-01	-1.15E-01	2.80E-01	1.82E-01
8263	GSM540333	GSE21653	Breast Cancer	Breast	1.47E-05	-2.64E-01	3.11E-02	-3.01E-01
8264	GSM540334	GSE21653	Breast Cancer	Breast	2.14E-01	-9.82E-02	3.81E-01	1.59E-01
8265	GSM540335	GSE21653	Breast Cancer	Breast	3.30E-01	-8.33E-02	5.09E-01	1.33E-01
8266	GSM540336	GSE21653	Breast Cancer	Breast	4.87E-03	-1.83E-01	3.49E-01	-1.66E-01
8267	GSM540337	GSE21653	Breast Cancer	Breast	3.10E-06	-2.82E-01	1.34E-01	-2.29E-01
8268	GSM540338	GSE21653	Breast Cancer	Breast	2.29E-02	1.54E-01	4.51E-02	2.84E-01
8269	GSM540339	GSE21653	Breast Cancer	Breast	6.97E-04	-2.13E-01	3.19E-01	-1.73E-01
8270	GSM540340	GSE21653	Breast Cancer	Breast	6.48E-03	-1.78E-01	2.80E-01	1.82E-01
8271	GSM540341	GSE21653	Breast Cancer	Breast	4.87E-12	-4.04E-01	5.40E-04	-4.43E-01
8272	GSM540342	GSE21653	Breast Cancer	Breast	4.05E-08	-3.26E-01	7.75E-03	-3.56E-01
8273	GSM540343	GSE21653	Breast Cancer	Breast	4.56E-25	-5.92E-01	2.09E-07	-6.34E-01
8274	GSM540344	GSE21653	Breast Cancer	Breast	3.84E-02	-1.43E-01	8.45E-02	2.54E-01
8275	GSM540345	GSE21653	Breast Cancer	Breast	1.97E-03	1.97E-01	2.45E-03	3.96E-01
8276	GSM540346	GSE21653	Breast Cancer	Breast	1.01E-02	-1.70E-01	4.77E-01	-1.39E-01
8277	GSM540347	GSE21653	Breast Cancer	Breast	2.35E-01	9.52E-02	8.66E-02	2.53E-01
8278	GSM540348	GSE21653	Breast Cancer	Breast	3.30E-01	-8.33E-02	2.83E-01	1.82E-01
8279	GSM540349	GSE21653	Breast Cancer	Breast	2.70E-13	-4.26E-01	2.87E-05	-5.23E-01
8280	GSM540350	GSE21653	Breast Cancer	Breast	1.08E-10	-3.79E-01	1.00E-02	-3.47E-01
8281	GSM540351	GSE21653	Breast Cancer	Breast	3.47E-01	-8.13E-02	2.22E-01	1.98E-01
8282	GSM540352	GSE21653	Breast Cancer	Breast	1.44E-01	-1.10E-01	1.51E-01	2.22E-01
8283	GSM540353	GSE21653	Breast Cancer	Breast	3.32E-07	-3.06E-01	1.02E-02	-3.46E-01

8284	GSM540354	GSE21653	Breast Cancer	Breast	4.94E-01	-6.65E-02	3.87E-01	1.57E-01
8285	GSM540355	GSE21653	Breast Cancer	Breast	2.49E-10	3.72E-01	4.21E-07	6.19E-01
8286	GSM540356	GSE21653	Breast Cancer	Breast	2.14E-01	-9.82E-02	6.15E-02	2.70E-01
8287	GSM540357	GSE21653	Breast Cancer	Breast	5.46E-02	1.35E-01	1.15E-01	2.37E-01
8288	GSM540358	GSE21653	Breast Cancer	Breast	4.83E-06	-2.77E-01	6.39E-02	-2.68E-01
8289	GSM540359	GSE21653	Breast Cancer	Breast	2.38E-03	-1.94E-01	4.26E-01	-1.49E-01
8290	GSM540360	GSE21653	Breast Cancer	Breast	5.39E-05	2.48E-01	1.50E-05	5.38E-01
8291	GSM540361	GSE21653	Breast Cancer	Breast	1.77E-01	1.04E-01	1.20E-02	3.40E-01
8292	GSM540362	GSE21653	Breast Cancer	Breast	2.77E-02	1.50E-01	3.16E-02	3.00E-01
8293	GSM540363	GSE21653	Breast Cancer	Breast	3.13E-25	-5.94E-01	5.91E-10	-7.45E-01
8294	GSM540364	GSE21653	Breast Cancer	Breast	1.00E-01	1.20E-01	9.36E-03	3.49E-01
8295	GSM540365	GSE21653	Breast Cancer	Breast	1.18E-03	2.05E-01	2.28E-04	4.68E-01
8296	GSM540366	GSE21653	Breast Cancer	Breast	1.25E-02	-1.66E-01	1.01E-01	-2.44E-01
8297	GSM540367	GSE21653	Breast Cancer	Breast	7.92E-02	1.26E-01	3.07E-02	3.02E-01
8298	GSM540368	GSE21653	Breast Cancer	Breast	2.57E-01	-9.23E-02	2.85E-01	1.81E-01
8299	GSM540369	GSE21653	Breast Cancer	Breast	7.30E-02	-1.28E-01	2.00E-01	2.05E-01
8300	GSM540370	GSE21653	Breast Cancer	Breast	9.63E-02	-1.21E-01	4.23E-01	-1.50E-01
8301	GSM540371	GSE21653	Breast Cancer	Breast	2.91E-02	-1.49E-01	3.19E-01	-1.73E-01
8302	GSM540372	GSE21653	Breast Cancer	Breast	2.80E-01	-8.93E-02	8.45E-02	2.54E-01
8303	GSM540373	GSE21653	Breast Cancer	Breast	6.13E-03	1.79E-01	9.85E-03	3.47E-01
8304	GSM782523	GSE31519	Breast Cancer	Breast	7.60E-02	-1.27E-01	2.80E-01	1.82E-01
8305	GSM782524	GSE31519	Breast Cancer	Breast	3.04E-03	-1.90E-01	4.58E-01	1.43E-01
8306	GSM782525	GSE31519	Breast Cancer	Breast	7.30E-02	1.28E-01	3.92E-03	3.80E-01
8307	GSM782526	GSE31519	Breast Cancer	Breast	2.29E-02	-1.54E-01	6.23E-02	2.69E-01
8308	GSM782527	GSE31519	Breast Cancer	Breast	1.30E-01	-1.13E-01	1.51E-01	2.22E-01
8309	GSM782528	GSE31519	Breast Cancer	Breast	4.49E-08	3.25E-01	3.21E-07	6.25E-01
8310	GSM782529	GSE31519	Breast Cancer	Breast	4.03E-01	-7.54E-02	2.57E-02	3.09E-01
8311	GSM782530	GSE31519	Breast Cancer	Breast	4.98E-08	-3.24E-01	5.25E-03	-3.70E-01
8312	GSM782531	GSE31519	Breast Cancer	Breast	2.57E-01	-9.23E-02	2.22E-01	1.98E-01
8313	GSM782532	GSE31519	Breast Cancer	Breast	4.03E-07	3.04E-01	7.74E-06	5.54E-01
8314	GSM782533	GSE31519	Breast Cancer	Breast	9.18E-08	-3.18E-01	9.36E-04	-4.27E-01
8315	GSM782534	GSE31519	Breast Cancer	Breast	1.18E-02	1.67E-01	2.11E-02	3.17E-01
8316	GSM782535	GSE31519	Breast Cancer	Breast	1.89E-05	2.61E-01	1.90E-03	4.04E-01
8317	GSM782536	GSE31519	Breast Cancer	Breast	3.05E-02	1.48E-01	6.31E-02	2.69E-01
8318	GSM782537	GSE31519	Breast Cancer	Breast	4.34E-03	-1.85E-01	3.49E-01	-1.66E-01
8319	GSM782538	GSE31519	Breast Cancer	Breast	3.42E-03	-1.88E-01	1.96E-01	2.06E-01
8320	GSM782539	GSE31519	Breast Cancer	Breast	1.61E-02	1.61E-01	1.47E-03	4.13E-01
8321	GSM782540	GSE31519	Breast Cancer	Breast	1.16E-01	-1.16E-01	4.29E-01	-1.49E-01
8322	GSM782541	GSE31519	Breast Cancer	Breast	1.53E-02	1.62E-01	5.17E-04	4.44E-01
8323	GSM782542	GSE31519	Breast Cancer	Breast	7.36E-05	-2.44E-01	7.65E-02	-2.59E-01
8324	GSM782543	GSE31519	Breast Cancer	Breast	1.25E-06	-2.92E-01	1.77E-02	-3.24E-01
8325	GSM782544	GSE31519	Breast Cancer	Breast	1.44E-03	-2.02E-01	4.58E-01	1.43E-01
8326	GSM782545	GSE31519	Breast Cancer	Breast	1.74E-03	-1.99E-01	1.77E-01	-2.13E-01
8327	GSM782546	GSE31519	Breast Cancer	Breast	6.16E-21	5.40E-01	5.67E-11	7.85E-01
8328	GSM782547	GSE31519	Breast Cancer	Breast	6.86E-03	-1.77E-01	4.70E-01	-1.40E-01
8329	GSM782548	GSE31519	Breast Cancer	Breast	7.01E-02	-1.29E-01	3.11E-01	1.75E-01
8330	GSM782549	GSE31519	Breast Cancer	Breast	3.01E-07	-3.07E-01	1.57E-01	-2.20E-01
8331	GSM782550	GSE31519	Breast Cancer	Breast	1.89E-01	1.02E-01	1.96E-01	2.06E-01
8332	GSM782551	GSE31519	Breast Cancer	Breast	6.46E-02	-1.31E-01	3.14E-01	-1.74E-01
8333	GSM782552	GSE31519	Breast Cancer	Breast	1.77E-01	1.04E-01	1.32E-01	2.30E-01
8334	GSM782553	GSE31519	Breast Cancer	Breast	3.21E-01	8.43E-02	1.77E-02	3.24E-01
8335	GSM782554	GSE31519	Breast Cancer	Breast	1.18E-03	-2.05E-01	6.39E-03	-3.63E-01
8336	GSM782555	GSE31519	Breast Cancer	Breast	2.21E-09	3.53E-01	1.29E-06	5.95E-01
8337	GSM782556	GSE31519	Breast Cancer	Breast	1.46E-04	2.35E-01	2.40E-03	3.97E-01
8338	GSM782557	GSE31519	Breast Cancer	Breast	7.27E-01	4.46E-02	2.80E-01	1.82E-01
8339	GSM782558	GSE31519	Breast Cancer	Breast	1.05E-05	-2.68E-01	7.74E-02	-2.58E-01
8340	GSM782559	GSE31519	Breast Cancer	Breast	1.30E-01	-1.13E-01	2.83E-01	1.82E-01
8341	GSM782560	GSE31519	Breast Cancer	Breast	1.53E-02	-1.62E-01	4.61E-01	1.42E-01
8342	GSM782561	GSE31519	Breast Cancer	Breast	1.35E-03	-2.03E-01	2.54E-01	-1.89E-01
8343	GSM782562	GSE31519	Breast Cancer	Breast	3.66E-01	-7.94E-02	2.00E-01	2.05E-01
8344	GSM782563	GSE31519	Breast Cancer	Breast	3.13E-01	-8.53E-02	3.46E-01	1.66E-01
8345	GSM782564	GSE31519	Breast Cancer	Breast	2.57E-01	9.23E-02	3.46E-01	1.66E-01
8346	GSM782565	GSE31519	Breast Cancer	Breast	4.87E-12	4.04E-01	2.09E-07	6.34E-01
8347	GSM782566	GSE31519	Breast Cancer	Breast	1.35E-04	-2.36E-01	1.38E-01	-2.27E-01
8348	GSM782567	GSE31519	Breast Cancer	Breast	3.34E-02	1.46E-01	1.16E-02	3.41E-01
8349	GSM782568	GSE31519	Breast Cancer	Breast	2.21E-01	9.72E-02	5.60E-02	2.74E-01
8350	GSM782569	GSE31519	Breast Cancer	Breast	8.56E-03	-1.73E-01	2.51E-01	-1.90E-01
8351	GSM782570	GSE31519	Breast Cancer	Breast	1.65E-06	-2.89E-01	7.46E-02	-2.60E-01
8352	GSM782571	GSE31519	Breast Cancer	Breast	3.20E-11	-3.89E-01	6.40E-07	-6.10E-01
8353	GSM782572	GSE31519	Breast Cancer	Breast	7.13E-12	-4.01E-01	3.34E-04	-4.57E-01
8354	GSM782573	GSE31519	Breast Cancer	Breast	1.35E-04	-2.36E-01	1.40E-01	-2.27E-01
8355	GSM782574	GSE31519	Breast Cancer	Breast	3.74E-04	-2.22E-01	2.61E-02	-3.09E-01
8356	GSM782575	GSE31519	Breast Cancer	Breast	1.85E-03	-1.98E-01	1.56E-01	-2.20E-01
8357	GSM782576	GSE31519	Breast Cancer	Breast	2.21E-09	3.53E-01	2.05E-05	5.31E-01

8358	GSM782577	GSE31519	Breast Cancer	Breast	2.64E-01	9.13E-02	8.56E-02	2.53E-01
8359	GSM782578	GSE31519	Breast Cancer	Breast	5.94E-02	-1.33E-01	5.83E-01	1.19E-01
8360	GSM782579	GSE31519	Breast Cancer	Breast	1.52E-12	4.13E-01	9.28E-08	6.50E-01
8361	GSM782580	GSE31519	Breast Cancer	Breast	1.57E-04	-2.34E-01	2.68E-02	-3.07E-01
8362	GSM782581	GSE31519	Breast Cancer	Breast	6.13E-03	-1.79E-01	2.25E-01	-1.97E-01
8363	GSM782582	GSE31519	Breast Cancer	Breast	4.02E-04	2.21E-01	7.28E-04	4.34E-01
8364	GSM782583	GSE31519	Breast Cancer	Breast	1.72E-18	-5.06E-01	1.44E-07	-6.41E-01
8365	GSM782584	GSE31519	Breast Cancer	Breast	4.39E-02	-1.40E-01	3.46E-01	1.66E-01
8366	GSM782585	GSE31519	Breast Cancer	Breast	3.62E-05	-2.53E-01	2.77E-02	-3.06E-01
8367	GSM782586	GSE31519	Breast Cancer	Breast	4.20E-02	-1.41E-01	1.52E-01	2.22E-01
8368	GSM782587	GSE31519	Breast Cancer	Breast	1.31E-02	1.65E-01	9.52E-03	3.49E-01
8369	GSM782588	GSE31519	Breast Cancer	Breast	2.35E-01	9.52E-02	1.77E-02	3.24E-01
8370	GSM782589	GSE31519	Breast Cancer	Breast	4.01E-02	1.42E-01	1.90E-03	4.04E-01
8371	GSM308422	GSE12276	Breast Cancer	Breast	4.44E-07	-3.03E-01	5.16E-03	-3.71E-01
8372	GSM308423	GSE12276	Breast Cancer	Breast	3.04E-01	8.63E-02	9.90E-02	2.46E-01
8373	GSM308425	GSE12276	Breast Cancer	Breast	8.24E-02	1.25E-01	1.77E-01	2.13E-01
8374	GSM308426	GSE12276	Breast Cancer	Breast	1.34E-01	1.12E-01	9.90E-02	2.46E-01
8375	GSM308429	GSE12276	Breast Cancer	Breast	1.18E-02	1.67E-01	3.76E-02	2.93E-01
8376	GSM308430	GSE12276	Breast Cancer	Breast	6.19E-02	-1.32E-01	4.70E-01	-1.40E-01
8377	GSM308433	GSE12276	Breast Cancer	Breast	5.46E-02	-1.35E-01	1.51E-01	2.22E-01
8378	GSM308434	GSE12276	Breast Cancer	Breast	1.30E-01	1.13E-01	3.11E-02	3.01E-01
8379	GSM308458	GSE12276	Breast Cancer	Breast	1.05E-05	-2.68E-01	9.68E-03	-3.48E-01
8380	GSM308414	GSE12276	Breast Cancer	Breast	1.72E-11	-3.94E-01	1.56E-02	-3.30E-01
8381	GSM308416	GSE12276	Breast Cancer	Breast	4.79E-02	1.38E-01	1.77E-01	2.13E-01
8382	GSM308418	GSE12276	Breast Cancer	Breast	1.88E-02	-1.58E-01	4.26E-01	-1.49E-01
8383	GSM308420	GSE12276	Breast Cancer	Breast	1.06E-02	-1.69E-01	1.18E-01	-2.36E-01
8384	GSM308421	GSE12276	Breast Cancer	Breast	6.46E-02	-1.31E-01	5.03E-01	1.34E-01
8385	GSM308435	GSE12276	Breast Cancer	Breast	1.36E-16	-4.78E-01	3.02E-07	-6.26E-01
8386	GSM308436	GSE12276	Breast Cancer	Breast	4.62E-04	2.19E-01	4.15E-03	3.78E-01
8387	GSM308438	GSE12276	Breast Cancer	Breast	1.70E-02	1.60E-01	1.94E-03	4.04E-01
8388	GSM308439	GSE12276	Breast Cancer	Breast	4.20E-02	1.41E-01	8.88E-02	2.51E-01
8389	GSM308441	GSE12276	Breast Cancer	Breast	1.12E-07	3.16E-01	2.93E-07	6.27E-01
8390	GSM308442	GSE12276	Breast Cancer	Breast	1.12E-01	1.17E-01	2.83E-01	1.82E-01
8391	GSM308444	GSE12276	Breast Cancer	Breast	1.50E-06	-2.90E-01	2.33E-01	-1.95E-01
8392	GSM308446	GSE12276	Breast Cancer	Breast	1.71E-01	1.05E-01	3.70E-02	2.93E-01
8393	GSM308456	GSE12276	Breast Cancer	Breast	6.48E-03	1.78E-01	1.56E-03	4.11E-01
8394	GSM308457	GSE12276	Breast Cancer	Breast	5.94E-02	-1.33E-01	7.27E-02	2.62E-01
8395	GSM308413	GSE12276	Breast Cancer	Breast	1.05E-05	2.68E-01	4.30E-03	3.77E-01
8396	GSM308415	GSE12276	Breast Cancer	Breast	1.65E-01	1.06E-01	3.07E-02	3.02E-01
8397	GSM308417	GSE12276	Breast Cancer	Breast	6.73E-02	-1.30E-01	2.51E-01	1.90E-01
8398	GSM308419	GSE12276	Breast Cancer	Breast	1.53E-03	-2.01E-01	4.36E-01	-1.47E-01
8399	GSM308424	GSE12276	Breast Cancer	Breast	3.05E-02	-1.48E-01	4.64E-01	-1.42E-01
8400	GSM308427	GSE12276	Breast Cancer	Breast	1.95E-08	-3.33E-01	6.56E-02	-2.67E-01
8401	GSM308428	GSE12276	Breast Cancer	Breast	1.95E-01	-1.01E-01	3.16E-01	1.73E-01
8402	GSM308431	GSE12276	Breast Cancer	Breast	1.83E-01	1.03E-01	4.45E-02	2.85E-01
8403	GSM308432	GSE12276	Breast Cancer	Breast	3.84E-01	-7.74E-02	6.15E-02	2.70E-01
8404	GSM308437	GSE12276	Breast Cancer	Breast	2.05E-05	-2.60E-01	4.70E-02	-2.83E-01
8405	GSM308440	GSE12276	Breast Cancer	Breast	1.61E-02	-1.61E-01	3.84E-01	-1.58E-01
8406	GSM308443	GSE12276	Breast Cancer	Breast	9.46E-07	2.95E-01	5.23E-05	5.07E-01
8407	GSM308445	GSE12276	Breast Cancer	Breast	2.29E-02	-1.54E-01	3.16E-01	1.73E-01
8408	GSM308447	GSE12276	Breast Cancer	Breast	7.30E-02	1.28E-01	1.73E-01	2.14E-01
8409	GSM308448	GSE12276	Breast Cancer	Breast	1.89E-01	1.02E-01	9.90E-02	2.46E-01
8410	GSM308449	GSE12276	Breast Cancer	Breast	3.50E-02	1.45E-01	3.70E-02	2.93E-01
8411	GSM308450	GSE12276	Breast Cancer	Breast	5.46E-02	-1.35E-01	8.45E-02	2.54E-01
8412	GSM308451	GSE12276	Breast Cancer	Breast	4.05E-08	-3.26E-01	4.76E-02	-2.82E-01
8413	GSM308452	GSE12276	Breast Cancer	Breast	1.60E-01	1.07E-01	2.11E-02	3.17E-01
8414	GSM308453	GSE12276	Breast Cancer	Breast	1.21E-01	-1.15E-01	2.83E-01	1.82E-01
8415	GSM308454	GSE12276	Breast Cancer	Breast	1.63E-03	-2.00E-01	3.22E-01	-1.72E-01
8416	GSM308455	GSE12276	Breast Cancer	Breast	6.08E-04	-2.15E-01	2.29E-01	-1.96E-01
8417	GSM308459	GSE12276	Breast Cancer	Breast	3.66E-07	3.05E-01	1.04E-05	5.47E-01
8418	GSM308460	GSE12276	Breast Cancer	Breast	2.21E-09	-3.53E-01	1.51E-02	-3.31E-01
8419	GSM308256	GSE12276	Breast Cancer	Breast	1.11E-03	2.06E-01	3.13E-03	3.88E-01
8420	GSM308257	GSE12276	Breast Cancer	Breast	1.34E-01	1.12E-01	1.46E-02	3.32E-01
8421	GSM308258	GSE12276	Breast Cancer	Breast	5.78E-01	5.85E-02	1.61E-01	2.18E-01
8422	GSM308259	GSE12276	Breast Cancer	Breast	4.02E-04	-2.21E-01	6.31E-02	-2.69E-01
8423	GSM308260	GSE12276	Breast Cancer	Breast	3.93E-05	-2.52E-01	3.61E-01	-1.63E-01
8424	GSM308261	GSE12276	Breast Cancer	Breast	5.79E-03	1.80E-01	2.68E-02	3.07E-01
8425	GSM308262	GSE12276	Breast Cancer	Breast	1.60E-01	1.07E-01	4.57E-02	2.84E-01
8426	GSM308263	GSE12276	Breast Cancer	Breast	8.91E-02	1.23E-01	4.45E-02	2.85E-01
8427	GSM308264	GSE12276	Breast Cancer	Breast	6.46E-02	-1.31E-01	1.54E-01	2.21E-01
8428	GSM308265	GSE12276	Breast Cancer	Breast	3.41E-19	5.16E-01	3.68E-10	7.53E-01
8429	GSM308266	GSE12276	Breast Cancer	Breast	1.46E-04	2.35E-01	6.06E-03	3.65E-01
8430	GSM308267	GSE12276	Breast Cancer	Breast	9.27E-05	-2.41E-01	2.61E-02	-3.09E-01
8431	GSM308268	GSE12276	Breast Cancer	Breast	1.18E-03	2.05E-01	9.36E-04	4.27E-01

8432	GSM308269	GSE12276	Breast Cancer	Breast	3.84E-01	7.74E-02	2.25E-01	1.97E-01
8433	GSM308270	GSE12276	Breast Cancer	Breast	3.50E-02	1.45E-01	4.38E-02	2.86E-01
8434	GSM308271	GSE12276	Breast Cancer	Breast	8.59E-05	-2.42E-01	4.02E-01	-1.54E-01
8435	GSM308272	GSE12276	Breast Cancer	Breast	1.70E-02	-1.60E-01	1.56E-01	-2.20E-01
8436	GSM308273	GSE12276	Breast Cancer	Breast	1.25E-06	-2.92E-01	1.83E-02	-3.23E-01
8437	GSM308274	GSE12276	Breast Cancer	Breast	1.08E-04	-2.39E-01	2.02E-01	-2.04E-01
8438	GSM308275	GSE12276	Breast Cancer	Breast	9.73E-04	-2.08E-01	1.77E-01	-2.13E-01
8439	GSM308276	GSE12276	Breast Cancer	Breast	1.89E-05	-2.61E-01	1.18E-02	-3.40E-01
8440	GSM308277	GSE12276	Breast Cancer	Breast	2.62E-04	-2.27E-01	1.75E-01	-2.13E-01
8441	GSM308278	GSE12276	Breast Cancer	Breast	3.45E-09	-3.49E-01	4.07E-04	-4.51E-01
8442	GSM308279	GSE12276	Breast Cancer	Breast	2.52E-02	-1.52E-01	1.75E-01	-2.13E-01
8443	GSM308280	GSE12276	Breast Cancer	Breast	2.91E-12	-4.08E-01	2.11E-05	-5.30E-01
8444	GSM308281	GSE12276	Breast Cancer	Breast	8.13E-06	-2.71E-01	3.20E-02	-3.00E-01
8445	GSM308282	GSE12276	Breast Cancer	Breast	3.42E-03	-1.88E-01	1.98E-01	-2.06E-01
8446	GSM308283	GSE12276	Breast Cancer	Breast	8.57E-02	1.24E-01	6.39E-02	2.68E-01
8447	GSM308284	GSE12276	Breast Cancer	Breast	1.61E-02	1.61E-01	4.00E-03	3.80E-01
8448	GSM308285	GSE12276	Breast Cancer	Breast	1.95E-08	3.33E-01	3.78E-05	5.16E-01
8449	GSM308286	GSE12276	Breast Cancer	Breast	8.52E-04	-2.10E-01	3.20E-02	-3.00E-01
8450	GSM308287	GSE12276	Breast Cancer	Breast	2.91E-02	1.49E-01	4.51E-02	2.84E-01
8451	GSM308288	GSE12276	Breast Cancer	Breast	3.08E-05	-2.55E-01	1.00E-01	-2.45E-01
8452	GSM308289	GSE12276	Breast Cancer	Breast	5.68E-01	-5.95E-02	3.11E-01	1.75E-01
8453	GSM308290	GSE12276	Breast Cancer	Breast	2.88E-01	8.83E-02	8.77E-02	2.52E-01
8454	GSM308291	GSE12276	Breast Cancer	Breast	8.29E-09	-3.41E-01	7.13E-04	-4.35E-01
8455	GSM308292	GSE12276	Breast Cancer	Breast	2.91E-02	1.49E-01	8.02E-03	3.55E-01
8456	GSM308293	GSE12276	Breast Cancer	Breast	1.63E-03	-2.00E-01	1.75E-01	-2.13E-01
8457	GSM308294	GSE12276	Breast Cancer	Breast	3.34E-05	-2.54E-01	8.77E-02	-2.52E-01
8458	GSM308295	GSE12276	Breast Cancer	Breast	3.08E-05	-2.55E-01	3.25E-02	-2.99E-01
8459	GSM308296	GSE12276	Breast Cancer	Breast	1.89E-01	1.02E-01	1.75E-02	3.25E-01
8460	GSM308297	GSE12276	Breast Cancer	Breast	5.16E-03	1.82E-01	7.88E-03	3.56E-01
8461	GSM308298	GSE12276	Breast Cancer	Breast	1.18E-03	-2.05E-01	1.18E-01	-2.36E-01
8462	GSM308299	GSE12276	Breast Cancer	Breast	3.63E-03	1.88E-01	1.75E-02	3.25E-01
8463	GSM308300	GSE12276	Breast Cancer	Breast	1.16E-01	1.16E-01	1.51E-02	3.31E-01
8464	GSM308301	GSE12276	Breast Cancer	Breast	1.24E-05	2.66E-01	6.83E-04	4.36E-01
8465	GSM308302	GSE12276	Breast Cancer	Breast	3.42E-03	-1.88E-01	8.77E-02	-2.52E-01
8466	GSM308303	GSE12276	Breast Cancer	Breast	5.16E-03	1.82E-01	9.68E-03	3.48E-01
8467	GSM308304	GSE12276	Breast Cancer	Breast	1.25E-01	-1.14E-01	2.80E-01	1.82E-01
8468	GSM308305	GSE12276	Breast Cancer	Breast	8.24E-02	1.25E-01	1.03E-01	2.44E-01
8469	GSM308306	GSE12276	Breast Cancer	Breast	2.96E-01	8.73E-02	8.56E-02	2.53E-01
8470	GSM308307	GSE12276	Breast Cancer	Breast	5.16E-03	1.82E-01	3.16E-02	3.00E-01
8471	GSM308308	GSE12276	Breast Cancer	Breast	1.25E-02	1.66E-01	2.57E-02	3.09E-01
8472	GSM308309	GSE12276	Breast Cancer	Breast	9.11E-04	-2.09E-01	2.10E-01	-2.02E-01
8473	GSM308310	GSE12276	Breast Cancer	Breast	1.05E-05	-2.68E-01	1.22E-02	-3.39E-01
8474	GSM308311	GSE12276	Breast Cancer	Breast	2.86E-03	-1.91E-01	1.17E-01	-2.37E-01
8475	GSM308312	GSE12276	Breast Cancer	Breast	1.34E-01	1.12E-01	4.45E-02	2.85E-01
8476	GSM308313	GSE12276	Breast Cancer	Breast	6.28E-06	-2.74E-01	4.57E-02	-2.84E-01
8477	GSM308314	GSE12276	Breast Cancer	Breast	4.20E-02	1.41E-01	2.17E-02	3.16E-01
8478	GSM308315	GSE12276	Breast Cancer	Breast	6.16E-30	6.49E-01	7.92E-15	9.21E-01
8479	GSM308316	GSE12276	Breast Cancer	Breast	1.83E-01	1.03E-01	2.11E-02	3.17E-01
8480	GSM308318	GSE12276	Breast Cancer	Breast	4.53E-16	4.70E-01	1.40E-09	7.30E-01
8481	GSM308319	GSE12276	Breast Cancer	Breast	3.38E-01	8.23E-02	5.53E-02	2.75E-01
8482	GSM308320	GSE12276	Breast Cancer	Breast	2.64E-02	-1.51E-01	3.63E-01	-1.63E-01
8483	GSM308321	GSE12276	Breast Cancer	Breast	1.18E-03	-2.05E-01	1.00E-01	-2.45E-01
8484	GSM308322	GSE12276	Breast Cancer	Breast	5.69E-02	-1.34E-01	8.56E-02	2.53E-01
8485	GSM308323	GSE12276	Breast Cancer	Breast	2.08E-01	9.92E-02	2.00E-01	2.05E-01
8486	GSM308324	GSE12276	Breast Cancer	Breast	6.73E-02	-1.30E-01	2.83E-01	-1.82E-01
8487	GSM308325	GSE12276	Breast Cancer	Breast	2.91E-02	1.49E-01	3.11E-02	3.01E-01
8488	GSM308326	GSE12276	Breast Cancer	Breast	3.04E-03	1.90E-01	4.00E-03	3.80E-01
8489	GSM308327	GSE12276	Breast Cancer	Breast	7.25E-03	-1.76E-01	1.38E-01	-2.27E-01
8490	GSM308328	GSE12276	Breast Cancer	Breast	2.38E-03	-1.94E-01	6.10E-01	1.14E-01
8491	GSM308329	GSE12276	Breast Cancer	Breast	1.49E-01	1.09E-01	1.75E-01	2.13E-01
8492	GSM308330	GSE12276	Breast Cancer	Breast	1.60E-01	-1.07E-01	6.23E-02	2.69E-01
8493	GSM308331	GSE12276	Breast Cancer	Breast	1.79E-02	-1.59E-01	1.73E-01	2.14E-01
8494	GSM308332	GSE12276	Breast Cancer	Breast	4.95E-04	2.18E-01	3.92E-03	3.80E-01
8495	GSM308333	GSE12276	Breast Cancer	Breast	7.45E-04	2.12E-01	2.40E-03	3.97E-01
8496	GSM308334	GSE12276	Breast Cancer	Breast	9.04E-03	-1.72E-01	3.27E-01	-1.71E-01
8497	GSM308335	GSE12276	Breast Cancer	Breast	6.48E-03	-1.78E-01	2.51E-01	-1.90E-01
8498	GSM308336	GSE12276	Breast Cancer	Breast	2.62E-04	2.27E-01	6.83E-04	4.36E-01
8499	GSM308337	GSE12276	Breast Cancer	Breast	1.96E-04	2.31E-01	3.98E-04	4.52E-01
8500	GSM308338	GSE12276	Breast Cancer	Breast	6.08E-04	-2.15E-01	1.35E-01	-2.29E-01
8501	GSM308339	GSE12276	Breast Cancer	Breast	3.93E-05	2.52E-01	1.77E-04	4.75E-01
8502	GSM308340	GSE12276	Breast Cancer	Breast	4.09E-03	1.86E-01	2.01E-03	4.03E-01
8503	GSM308341	GSE12276	Breast Cancer	Breast	2.44E-04	-2.28E-01	1.59E-01	-2.19E-01
8504	GSM308342	GSE12276	Breast Cancer	Breast	3.84E-01	-7.74E-02	1.73E-01	2.14E-01
8505	GSM308343	GSE12276	Breast Cancer	Breast	5.79E-03	-1.80E-01	2.54E-01	1.89E-01

8506	GSM308344	GSE12276	Breast Cancer	Breast	3.71E-06	2.80E-01	3.90E-06	5.70E-01
8507	GSM308345	GSE12276	Breast Cancer	Breast	4.13E-01	7.44E-02	2.17E-02	3.16E-01
8508	GSM308346	GSE12276	Breast Cancer	Breast	2.96E-01	-8.73E-02	4.26E-01	1.49E-01
8509	GSM308347	GSE12276	Breast Cancer	Breast	3.74E-04	-2.22E-01	7.55E-02	-2.60E-01
8510	GSM308348	GSE12276	Breast Cancer	Breast	1.63E-03	-2.00E-01	3.58E-01	-1.64E-01
8511	GSM308349	GSE12276	Breast Cancer	Breast	3.34E-02	-1.46E-01	1.34E-01	-2.29E-01
8512	GSM308350	GSE12276	Breast Cancer	Breast	1.35E-03	-2.03E-01	1.73E-01	2.14E-01
8513	GSM308351	GSE12276	Breast Cancer	Breast	1.54E-01	-1.08E-01	3.58E-01	-1.64E-01
8514	GSM308352	GSE12276	Breast Cancer	Breast	5.46E-02	1.35E-01	1.32E-01	2.30E-01
8515	GSM308353	GSE12276	Breast Cancer	Breast	2.88E-01	8.83E-02	1.14E-01	2.38E-01
8516	GSM308354	GSE12276	Breast Cancer	Breast	1.44E-01	-1.10E-01	8.45E-02	2.54E-01
8517	GSM308355	GSE12276	Breast Cancer	Breast	6.19E-02	-1.32E-01	4.20E-01	1.50E-01
8518	GSM308356	GSE12276	Breast Cancer	Breast	1.83E-01	-1.03E-01	2.22E-01	1.98E-01
8519	GSM308357	GSE12276	Breast Cancer	Breast	2.08E-01	-9.92E-02	1.05E-01	2.43E-01
8520	GSM308358	GSE12276	Breast Cancer	Breast	2.83E-11	-3.90E-01	1.46E-05	-5.39E-01
8521	GSM308359	GSE12276	Breast Cancer	Breast	1.25E-01	1.14E-01	8.66E-02	2.53E-01
8522	GSM308360	GSE12276	Breast Cancer	Breast	2.01E-01	1.00E-01	2.22E-01	1.98E-01
8523	GSM308361	GSE12276	Breast Cancer	Breast	1.39E-01	1.11E-01	8.45E-02	2.54E-01
8524	GSM308362	GSE12276	Breast Cancer	Breast	9.73E-04	-2.08E-01	1.41E-01	-2.26E-01
8525	GSM308363	GSE12276	Breast Cancer	Breast	4.44E-07	3.03E-01	2.87E-05	5.23E-01
8526	GSM308364	GSE12276	Breast Cancer	Breast	4.95E-04	-2.18E-01	5.09E-01	-1.33E-01
8527	GSM308365	GSE12276	Breast Cancer	Breast	7.36E-05	-2.44E-01	1.15E-01	-2.37E-01
8528	GSM308366	GSE12276	Breast Cancer	Breast	1.08E-01	1.18E-01	3.70E-02	2.93E-01
8529	GSM308367	GSE12276	Breast Cancer	Breast	9.54E-03	1.71E-01	2.14E-02	3.17E-01
8530	GSM308368	GSE12276	Breast Cancer	Breast	7.46E-06	-2.72E-01	1.49E-02	-3.31E-01
8531	GSM308369	GSE12276	Breast Cancer	Breast	3.09E-09	-3.50E-01	1.56E-03	-4.11E-01
8532	GSM308370	GSE12276	Breast Cancer	Breast	1.88E-02	1.58E-01	1.20E-02	3.40E-01
8533	GSM308371	GSE12276	Breast Cancer	Breast	3.38E-01	8.23E-02	1.01E-01	2.44E-01
8534	GSM308372	GSE12276	Breast Cancer	Breast	6.81E-05	-2.45E-01	1.03E-01	-2.44E-01
8535	GSM308373	GSE12276	Breast Cancer	Breast	5.36E-09	-3.45E-01	4.07E-04	-4.51E-01
8536	GSM308374	GSE12276	Breast Cancer	Breast	9.26E-02	-1.22E-01	1.51E-01	2.22E-01
8537	GSM308375	GSE12276	Breast Cancer	Breast	1.85E-07	-3.12E-01	2.49E-03	-3.96E-01
8538	GSM308376	GSE12276	Breast Cancer	Breast	1.44E-01	-1.10E-01	3.52E-01	-1.65E-01
8539	GSM308377	GSE12276	Breast Cancer	Breast	1.49E-01	-1.09E-01	3.14E-01	1.74E-01
8540	GSM308378	GSE12276	Breast Cancer	Breast	1.75E-08	-3.34E-01	2.01E-03	-4.03E-01
8541	GSM308379	GSE12276	Breast Cancer	Breast	2.23E-05	-2.59E-01	5.38E-02	-2.76E-01
8542	GSM308380	GSE12276	Breast Cancer	Breast	1.31E-02	-1.65E-01	2.80E-01	1.82E-01
8543	GSM308381	GSE12276	Breast Cancer	Breast	5.91E-07	-3.00E-01	3.76E-02	-2.93E-01
8544	GSM308382	GSE12276	Breast Cancer	Breast	6.51E-04	2.14E-01	1.34E-04	4.83E-01
8545	GSM308383	GSE12276	Breast Cancer	Breast	3.25E-04	2.24E-01	2.28E-04	4.68E-01
8546	GSM308384	GSE12276	Breast Cancer	Breast	4.03E-01	-7.54E-02	2.61E-01	1.87E-01
8547	GSM308385	GSE12276	Breast Cancer	Breast	7.67E-03	1.75E-01	3.07E-02	3.02E-01
8548	GSM308386	GSE12276	Breast Cancer	Breast	9.06E-19	-5.10E-01	9.66E-07	-6.01E-01
8549	GSM308387	GSE12276	Breast Cancer	Breast	7.60E-02	-1.27E-01	3.11E-01	1.75E-01
8550	GSM308388	GSE12276	Breast Cancer	Breast	4.87E-03	1.83E-01	1.44E-02	3.33E-01
8551	GSM308389	GSE12276	Breast Cancer	Breast	1.34E-11	-3.96E-01	9.36E-04	-4.27E-01
8552	GSM308390	GSE12276	Breast Cancer	Breast	4.34E-03	-1.85E-01	6.30E-01	-1.10E-01
8553	GSM308391	GSE12276	Breast Cancer	Breast	1.00E-01	1.20E-01	1.56E-01	2.20E-01
8554	GSM308392	GSE12276	Breast Cancer	Breast	3.39E-06	-2.81E-01	1.00E-02	-3.47E-01
8555	GSM308393	GSE12276	Breast Cancer	Breast	8.24E-02	1.25E-01	9.36E-03	3.49E-01
8556	GSM308394	GSE12276	Breast Cancer	Breast	1.25E-02	-1.66E-01	2.88E-01	-1.80E-01
8557	GSM308395	GSE12276	Breast Cancer	Breast	1.18E-03	2.05E-01	5.16E-03	3.71E-01
8558	GSM308396	GSE12276	Breast Cancer	Breast	9.63E-02	-1.21E-01	5.06E-01	1.33E-01
8559	GSM308397	GSE12276	Breast Cancer	Breast	2.62E-04	-2.27E-01	1.21E-01	-2.35E-01
8560	GSM308398	GSE12276	Breast Cancer	Breast	4.79E-02	-1.38E-01	3.46E-01	1.66E-01
8561	GSM308399	GSE12276	Breast Cancer	Breast	1.18E-02	1.67E-01	1.49E-02	3.31E-01
8562	GSM308400	GSE12276	Breast Cancer	Breast	2.49E-01	9.33E-02	1.98E-01	2.06E-01
8563	GSM308401	GSE12276	Breast Cancer	Breast	9.46E-07	2.95E-01	4.08E-05	5.14E-01
8564	GSM308402	GSE12276	Breast Cancer	Breast	1.38E-02	-1.64E-01	3.11E-01	1.75E-01
8565	GSM308403	GSE12276	Breast Cancer	Breast	4.31E-09	-3.47E-01	5.40E-04	-4.43E-01
8566	GSM308404	GSE12276	Breast Cancer	Breast	1.96E-04	-2.31E-01	3.14E-01	-1.74E-01
8567	GSM308405	GSE12276	Breast Cancer	Breast	1.14E-08	-3.38E-01	1.73E-04	-4.76E-01
8568	GSM308406	GSE12276	Breast Cancer	Breast	6.73E-02	-1.30E-01	1.51E-01	2.22E-01
8569	GSM308407	GSE12276	Breast Cancer	Breast	2.99E-14	-4.41E-01	1.50E-05	-5.38E-01
8570	GSM308408	GSE12276	Breast Cancer	Breast	5.98E-09	-3.44E-01	1.26E-02	-3.38E-01
8571	GSM308409	GSE12276	Breast Cancer	Breast	3.31E-12	-4.07E-01	2.79E-06	-5.78E-01
8572	GSM308410	GSE12276	Breast Cancer	Breast	1.65E-01	1.06E-01	3.70E-02	2.93E-01
8573	GSM308411	GSE12276	Breast Cancer	Breast	2.42E-05	-2.58E-01	6.39E-02	-2.68E-01
8574	GSM308412	GSE12276	Breast Cancer	Breast	4.34E-03	1.85E-01	1.46E-02	3.32E-01
8575	GSM125123	GSE20181	Breast Cancer	Breast	1.61E-02	-1.61E-01	1.98E-01	2.06E-01
8576	GSM125124	GSE20181	Breast Cancer	Breast	4.62E-13	4.22E-01	7.93E-09	6.98E-01
8577	GSM125125	GSE20181	Breast Cancer	Breast	8.13E-06	-2.71E-01	4.45E-02	-2.85E-01
8578	GSM125126	GSE20181	Breast Cancer	Breast	5.91E-07	-3.00E-01	6.17E-03	-3.64E-01
8579	GSM125127	GSE20181	Breast Cancer	Breast	8.56E-03	1.73E-01	6.39E-03	3.63E-01

8580	GSM125128	GSE20181	Breast Cancer	Breast	1.37E-06	-2.91E-01	3.76E-02	-2.93E-01
8581	GSM125129	GSE20181	Breast Cancer	Breast	2.42E-01	-9.42E-02	3.11E-01	1.75E-01
8582	GSM125130	GSE20181	Breast Cancer	Breast	5.69E-02	1.34E-01	4.07E-03	3.79E-01
8583	GSM125131	GSE20181	Breast Cancer	Breast	1.18E-03	-2.05E-01	2.51E-01	-1.90E-01
8584	GSM125132	GSE20181	Breast Cancer	Breast	1.16E-04	-2.38E-01	1.32E-01	2.30E-01
8585	GSM125133	GSE20181	Breast Cancer	Breast	7.84E-07	-2.97E-01	4.45E-02	-2.85E-01
8586	GSM125134	GSE20181	Breast Cancer	Breast	1.54E-01	1.08E-01	3.11E-02	3.01E-01
8587	GSM125135	GSE20181	Breast Cancer	Breast	9.73E-04	2.08E-01	1.56E-03	4.11E-01
8588	GSM125136	GSE20181	Breast Cancer	Breast	1.16E-04	-2.38E-01	3.25E-02	-2.99E-01
8589	GSM125137	GSE20181	Breast Cancer	Breast	5.64E-10	-3.65E-01	8.98E-04	-4.28E-01
8590	GSM125138	GSE20181	Breast Cancer	Breast	4.98E-08	3.24E-01	7.95E-06	5.54E-01
8591	GSM125139	GSE20181	Breast Cancer	Breast	7.92E-02	-1.26E-01	5.24E-02	2.77E-01
8592	GSM125140	GSE20181	Breast Cancer	Breast	1.65E-01	1.06E-01	1.42E-02	3.33E-01
8593	GSM125141	GSE20181	Breast Cancer	Breast	1.00E-04	-2.40E-01	3.30E-02	-2.98E-01
8594	GSM125142	GSE20181	Breast Cancer	Breast	4.59E-02	-1.39E-01	2.54E-01	-1.89E-01
8595	GSM125143	GSE20181	Breast Cancer	Breast	2.64E-01	-9.13E-02	1.57E-01	2.20E-01
8596	GSM125144	GSE20181	Breast Cancer	Breast	5.27E-06	2.76E-01	2.64E-06	5.79E-01
8597	GSM125145	GSE20181	Breast Cancer	Breast	4.01E-02	1.42E-01	1.18E-02	3.40E-01
8598	GSM125146	GSE20181	Breast Cancer	Breast	2.28E-01	-9.62E-02	3.81E-01	1.59E-01
8599	GSM125147	GSE20181	Breast Cancer	Breast	2.23E-05	-2.59E-01	3.76E-02	-2.93E-01
8600	GSM125148	GSE20181	Breast Cancer	Breast	1.18E-03	-2.05E-01	1.82E-01	-2.11E-01
8601	GSM125149	GSE20181	Breast Cancer	Breast	3.98E-10	-3.68E-01	2.49E-03	-3.96E-01
8602	GSM125150	GSE20181	Breast Cancer	Breast	1.74E-03	-1.99E-01	5.38E-02	-2.76E-01
8603	GSM125151	GSE20181	Breast Cancer	Breast	7.92E-02	1.26E-01	4.45E-02	2.85E-01
8604	GSM125152	GSE20181	Breast Cancer	Breast	2.10E-03	1.96E-01	3.85E-03	3.81E-01
8605	GSM125153	GSE20181	Breast Cancer	Breast	4.63E-01	6.94E-02	1.04E-01	2.43E-01
8606	GSM125154	GSE20181	Breast Cancer	Breast	9.04E-03	1.72E-01	4.98E-03	3.72E-01
8607	GSM125155	GSE20181	Breast Cancer	Breast	1.81E-06	-2.88E-01	4.00E-03	-3.80E-01
8608	GSM125156	GSE20181	Breast Cancer	Breast	7.67E-03	-1.75E-01	1.96E-01	2.06E-01
8609	GSM125157	GSE20181	Breast Cancer	Breast	9.11E-04	-2.09E-01	8.56E-02	-2.53E-01
8610	GSM125158	GSE20181	Breast Cancer	Breast	4.13E-01	7.44E-02	2.61E-02	3.09E-01
8611	GSM125159	GSE20181	Breast Cancer	Breast	5.52E-08	-3.23E-01	2.01E-03	-4.03E-01
8612	GSM125160	GSE20181	Breast Cancer	Breast	3.63E-03	-1.88E-01	2.56E-01	-1.89E-01
8613	GSM125161	GSE20181	Breast Cancer	Breast	1.71E-15	-4.61E-01	1.73E-04	-4.76E-01
8614	GSM125162	GSE20181	Breast Cancer	Breast	2.24E-12	-4.10E-01	1.73E-04	-4.76E-01
8615	GSM125163	GSE20181	Breast Cancer	Breast	2.44E-04	-2.28E-01	7.74E-02	-2.58E-01
8616	GSM125164	GSE20181	Breast Cancer	Breast	1.61E-02	-1.61E-01	2.88E-01	-1.80E-01
8617	GSM125165	GSE20181	Breast Cancer	Breast	1.89E-05	-2.61E-01	6.48E-02	-2.67E-01
8618	GSM125166	GSE20181	Breast Cancer	Breast	6.13E-03	-1.79E-01	2.49E-01	1.90E-01
8619	GSM125167	GSE20181	Breast Cancer	Breast	4.28E-12	-4.05E-01	8.98E-04	-4.28E-01
8620	GSM125168	GSE20181	Breast Cancer	Breast	1.30E-01	-1.13E-01	1.00E-01	2.45E-01
8621	GSM125169	GSE20181	Breast Cancer	Breast	7.54E-11	-3.82E-01	5.23E-05	-5.07E-01
8622	GSM125170	GSE20181	Breast Cancer	Breast	2.29E-02	-1.54E-01	9.90E-02	2.46E-01
8623	GSM125171	GSE20181	Breast Cancer	Breast	3.56E-01	8.04E-02	1.51E-02	3.31E-01
8624	GSM125172	GSE20181	Breast Cancer	Breast	6.73E-02	-1.30E-01	3.14E-01	1.74E-01
8625	GSM125173	GSE20181	Breast Cancer	Breast	2.24E-03	-1.95E-01	2.83E-01	1.82E-01
8626	GSM125174	GSE20181	Breast Cancer	Breast	2.42E-05	2.58E-01	3.98E-04	4.52E-01
8627	GSM125175	GSE20181	Breast Cancer	Breast	1.46E-04	-2.35E-01	2.49E-01	-1.90E-01
8628	GSM125176	GSE20181	Breast Cancer	Breast	2.57E-01	9.23E-02	2.57E-02	3.09E-01
8629	GSM125177	GSE20181	Breast Cancer	Breast	1.18E-02	1.67E-01	3.11E-02	3.01E-01
8630	GSM125178	GSE20181	Breast Cancer	Breast	7.30E-02	1.28E-01	5.45E-02	2.76E-01
8631	GSM125179	GSE20181	Breast Cancer	Breast	1.01E-02	1.70E-01	1.90E-03	4.04E-01
8632	GSM125180	GSE20181	Breast Cancer	Breast	1.04E-06	2.94E-01	2.00E-05	5.31E-01
8633	GSM125181	GSE20181	Breast Cancer	Breast	6.67E-09	-3.43E-01	5.25E-03	-3.70E-01
8634	GSM125182	GSE20181	Breast Cancer	Breast	6.51E-04	-2.14E-01	8.56E-02	-2.53E-01
8635	GSM125183	GSE20181	Breast Cancer	Breast	7.30E-02	1.28E-01	6.15E-02	2.70E-01
8636	GSM125184	GSE20181	Breast Cancer	Breast	2.73E-07	3.08E-01	2.79E-06	5.78E-01
8637	GSM125185	GSE20181	Breast Cancer	Breast	2.64E-02	1.51E-01	2.57E-02	3.09E-01
8638	GSM125186	GSE20181	Breast Cancer	Breast	1.31E-02	1.65E-01	2.57E-02	3.09E-01
8639	GSM125187	GSE20181	Breast Cancer	Breast	1.97E-03	-1.97E-01	1.37E-01	-2.28E-01
8640	GSM125188	GSE20181	Breast Cancer	Breast	4.98E-08	-3.24E-01	4.98E-03	-3.72E-01
8641	GSM125189	GSE20181	Breast Cancer	Breast	5.69E-02	-1.34E-01	2.66E-01	-1.86E-01
8642	GSM125190	GSE20181	Breast Cancer	Breast	1.12E-02	-1.68E-01	1.77E-01	-2.13E-01
8643	GSM125191	GSE20181	Breast Cancer	Breast	1.16E-01	-1.16E-01	2.00E-01	2.05E-01
8644	GSM125192	GSE20181	Breast Cancer	Breast	4.03E-01	7.54E-02	1.14E-01	2.38E-01
8645	GSM125193	GSE20181	Breast Cancer	Breast	3.08E-05	-2.55E-01	9.68E-03	-3.48E-01
8646	GSM125194	GSE20181	Breast Cancer	Breast	9.04E-03	-1.72E-01	2.27E-01	-1.97E-01
8647	GSM125195	GSE20181	Breast Cancer	Breast	2.42E-01	-9.42E-02	5.00E-01	1.35E-01
8648	GSM125196	GSE20181	Breast Cancer	Breast	6.46E-02	-1.31E-01	1.32E-01	2.30E-01
8649	GSM125197	GSE20181	Breast Cancer	Breast	1.35E-03	-2.03E-01	2.20E-02	-3.16E-01
8650	GSM125198	GSE20181	Breast Cancer	Breast	9.26E-02	-1.22E-01	3.46E-01	1.66E-01
8651	GSM125199	GSE20181	Breast Cancer	Breast	3.66E-07	-3.05E-01	1.00E-02	-3.47E-01
8652	GSM125200	GSE20181	Breast Cancer	Breast	3.21E-01	8.43E-02	1.96E-01	2.06E-01
8653	GSM125201	GSE20181	Breast Cancer	Breast	1.21E-01	-1.15E-01	3.81E-01	1.59E-01

8654	GSM125202	GSE20181	Breast Cancer	Breast	5.94E-02	-1.33E-01	1.51E-01	2.22E-01
8655	GSM125203	GSE20181	Breast Cancer	Breast	7.67E-03	-1.75E-01	4.20E-01	1.50E-01
8656	GSM125204	GSE20181	Breast Cancer	Breast	2.52E-02	-1.52E-01	6.23E-01	1.11E-01
8657	GSM125205	GSE20181	Breast Cancer	Breast	2.57E-01	-9.23E-02	2.49E-01	1.90E-01
8658	GSM125206	GSE20181	Breast Cancer	Breast	1.89E-01	-1.02E-01	6.77E-01	-1.01E-01
8659	GSM125207	GSE20181	Breast Cancer	Breast	6.28E-06	2.74E-01	5.07E-03	3.71E-01
8660	GSM125208	GSE20181	Breast Cancer	Breast	6.73E-02	1.30E-01	8.77E-02	2.52E-01
8661	GSM125209	GSE20181	Breast Cancer	Breast	2.07E-02	-1.56E-01	1.98E-01	-2.06E-01
8662	GSM125210	GSE20181	Breast Cancer	Breast	4.60E-05	2.50E-01	1.17E-03	4.20E-01
8663	GSM125211	GSE20181	Breast Cancer	Breast	2.84E-05	-2.56E-01	1.56E-01	-2.20E-01
8664	GSM125212	GSE20181	Breast Cancer	Breast	8.13E-06	-2.71E-01	1.51E-02	-3.31E-01
8665	GSM125213	GSE20181	Breast Cancer	Breast	2.29E-02	-1.54E-01	3.11E-01	-1.75E-01
8666	GSM125214	GSE20181	Breast Cancer	Breast	1.88E-02	-1.58E-01	1.75E-01	2.13E-01
8667	GSM125215	GSE20181	Breast Cancer	Breast	2.96E-01	-8.73E-02	3.58E-01	1.64E-01
8668	GSM125216	GSE20181	Breast Cancer	Breast	8.52E-04	2.10E-01	1.77E-02	3.24E-01
8669	GSM125217	GSE20181	Breast Cancer	Breast	9.59E-11	-3.80E-01	9.17E-04	-4.27E-01
8670	GSM125218	GSE20181	Breast Cancer	Breast	6.33E-10	-3.64E-01	9.60E-05	-4.91E-01
8671	GSM125219	GSE20181	Breast Cancer	Breast	2.14E-01	9.82E-02	6.31E-02	2.69E-01
8672	GSM125220	GSE20181	Breast Cancer	Breast	5.98E-09	-3.44E-01	3.92E-03	-3.80E-01
8673	GSM125221	GSE20181	Breast Cancer	Breast	8.30E-08	-3.19E-01	1.22E-02	-3.39E-01
8674	GSM125222	GSE20181	Breast Cancer	Breast	7.96E-05	-2.43E-01	3.25E-02	-2.99E-01
8675	GSM125223	GSE20181	Breast Cancer	Breast	2.25E-07	-3.10E-01	3.25E-02	-2.99E-01
8676	GSM125224	GSE20181	Breast Cancer	Breast	4.79E-02	-1.38E-01	3.19E-01	1.73E-01
8677	GSM125225	GSE20181	Breast Cancer	Breast	3.66E-07	-3.05E-01	1.94E-03	-4.04E-01
8678	GSM125226	GSE20181	Breast Cancer	Breast	2.73E-07	-3.08E-01	1.94E-03	-4.04E-01
8679	GSM125227	GSE20181	Breast Cancer	Breast	1.61E-02	-1.61E-01	3.11E-01	-1.75E-01
8680	GSM125228	GSE20181	Breast Cancer	Breast	1.97E-02	-1.57E-01	2.88E-01	-1.80E-01
8681	GSM125229	GSE20181	Breast Cancer	Breast	1.38E-10	-3.77E-01	1.94E-03	-4.04E-01
8682	GSM125230	GSE20181	Breast Cancer	Breast	4.79E-02	-1.38E-01	2.83E-01	1.82E-01
8683	GSM125231	GSE20181	Breast Cancer	Breast	4.25E-05	2.51E-01	6.98E-04	4.36E-01
8684	GSM125232	GSE20181	Breast Cancer	Breast	5.41E-23	5.66E-01	7.78E-12	8.17E-01
8685	GSM125233	GSE20181	Breast Cancer	Breast	2.88E-01	8.83E-02	4.45E-02	2.85E-01
8686	GSM125234	GSE20181	Breast Cancer	Breast	2.37E-06	2.85E-01	2.98E-04	4.60E-01
8687	GSM125235	GSE20181	Breast Cancer	Breast	1.03E-08	-3.39E-01	1.20E-02	-3.40E-01
8688	GSM125236	GSE20181	Breast Cancer	Breast	2.14E-01	-9.82E-02	1.96E-01	2.06E-01
8689	GSM125237	GSE20181	Breast Cancer	Breast	2.11E-04	-2.30E-01	1.00E-01	-2.45E-01
8690	GSM125238	GSE20181	Breast Cancer	Breast	1.27E-08	-3.37E-01	1.54E-02	-3.30E-01
8691	GSM506267	GSE20181	Breast Cancer	Breast	6.46E-02	-1.31E-01	3.81E-01	1.59E-01
8692	GSM506268	GSE20181	Breast Cancer	Breast	1.01E-02	-1.70E-01	2.25E-01	-1.97E-01
8693	GSM506269	GSE20181	Breast Cancer	Breast	5.68E-01	-5.95E-02	1.32E-01	2.30E-01
8694	GSM506270	GSE20181	Breast Cancer	Breast	4.13E-01	7.44E-02	8.45E-02	2.54E-01
8695	GSM506271	GSE20181	Breast Cancer	Breast	1.97E-03	-1.97E-01	3.58E-01	-1.64E-01
8696	GSM506272	GSE20181	Breast Cancer	Breast	2.83E-11	3.90E-01	2.03E-07	6.34E-01
8697	GSM506273	GSE20181	Breast Cancer	Breast	6.08E-04	-2.15E-01	1.15E-01	-2.37E-01
8698	GSM506274	GSE20181	Breast Cancer	Breast	5.16E-03	-1.82E-01	3.61E-01	-1.63E-01
8699	GSM506275	GSE20181	Breast Cancer	Breast	4.33E-01	7.24E-02	6.17E-03	3.64E-01
8700	GSM506276	GSE20181	Breast Cancer	Breast	2.64E-01	9.13E-02	3.11E-01	1.75E-01
8701	GSM506277	GSE20181	Breast Cancer	Breast	8.56E-03	-1.73E-01	1.98E-01	2.06E-01
8702	GSM506278	GSE20181	Breast Cancer	Breast	4.87E-03	-1.83E-01	3.11E-01	1.75E-01
8703	GSM506279	GSE20181	Breast Cancer	Breast	1.65E-01	1.06E-01	6.06E-03	3.65E-01
8704	GSM506280	GSE20181	Breast Cancer	Breast	1.08E-19	5.23E-01	1.25E-11	8.10E-01
8705	GSM506281	GSE20181	Breast Cancer	Breast	3.39E-06	-2.81E-01	1.83E-02	-3.23E-01
8706	GSM506282	GSE20181	Breast Cancer	Breast	6.97E-04	2.13E-01	6.98E-04	4.36E-01
8707	GSM506283	GSE20181	Breast Cancer	Breast	5.46E-02	1.35E-01	4.45E-02	2.85E-01
8708	GSM506284	GSE20181	Breast Cancer	Breast	1.01E-02	-1.70E-01	3.58E-01	-1.64E-01
8709	GSM506285	GSE20181	Breast Cancer	Breast	2.17E-08	-3.32E-01	3.13E-03	-3.88E-01
8710	GSM506286	GSE20181	Breast Cancer	Breast	1.04E-01	1.19E-01	1.14E-01	2.38E-01
8711	GSM506287	GSE20181	Breast Cancer	Breast	4.95E-04	2.18E-01	8.79E-04	4.29E-01
8712	GSM506288	GSE20181	Breast Cancer	Breast	1.11E-03	2.06E-01	6.06E-03	3.65E-01
8713	GSM506289	GSE20181	Breast Cancer	Breast	4.63E-01	-6.94E-02	2.49E-01	1.90E-01
8714	GSM506290	GSE20181	Breast Cancer	Breast	2.14E-01	9.82E-02	9.90E-02	2.46E-01
8715	GSM506291	GSE20181	Breast Cancer	Breast	1.61E-02	1.61E-01	2.61E-02	3.09E-01
8716	GSM506292	GSE20181	Breast Cancer	Breast	1.30E-01	-1.13E-01	4.20E-01	1.50E-01
8717	GSM506293	GSE20181	Breast Cancer	Breast	1.70E-02	-1.60E-01	5.03E-01	-1.34E-01
8718	GSM506294	GSE20181	Breast Cancer	Breast	3.03E-04	2.25E-01	8.79E-04	4.29E-01
8719	GSM506295	GSE20181	Breast Cancer	Breast	1.04E-01	1.19E-01	6.15E-02	2.70E-01
8720	GSM506296	GSE20181	Breast Cancer	Breast	1.85E-03	1.98E-01	6.62E-03	3.62E-01
8721	GSM506297	GSE20181	Breast Cancer	Breast	9.67E-15	4.49E-01	3.60E-09	7.12E-01
8722	GSM506298	GSE20181	Breast Cancer	Breast	7.86E-13	-4.18E-01	3.80E-06	-5.71E-01
8723	GSM506299	GSE20181	Breast Cancer	Breast	4.28E-12	4.05E-01	3.80E-06	5.71E-01
8724	GSM506300	GSE20181	Breast Cancer	Breast	5.04E-01	-6.55E-02	7.46E-02	2.60E-01
8725	GSM506301	GSE20181	Breast Cancer	Breast	7.67E-03	-1.75E-01	1.15E-01	-2.37E-01
8726	GSM506302	GSE20181	Breast Cancer	Breast	5.36E-01	-6.25E-02	2.58E-01	1.88E-01
8727	GSM506303	GSE20181	Breast Cancer	Breast	4.81E-09	-3.46E-01	5.40E-04	-4.43E-01

8728	GSM506304	GSE20181	Breast Cancer	Breast	7.01E-02	-1.29E-01	3.16E-01	1.73E-01
8729	GSM506305	GSE20181	Breast Cancer	Breast	2.64E-01	-9.13E-02	3.76E-02	2.93E-01
8730	GSM506306	GSE20181	Breast Cancer	Breast	2.73E-07	3.08E-01	7.33E-06	5.56E-01
8731	GSM506307	GSE20181	Breast Cancer	Breast	5.83E-05	2.47E-01	1.01E-04	4.90E-01
8732	GSM506308	GSE20181	Breast Cancer	Breast	8.94E-10	3.61E-01	4.21E-07	6.19E-01
8733	GSM506309	GSE20181	Breast Cancer	Breast	7.92E-02	1.26E-01	3.07E-02	3.02E-01
8734	GSM506310	GSE20181	Breast Cancer	Breast	3.63E-03	1.88E-01	7.61E-03	3.57E-01
8735	GSM506311	GSE20181	Breast Cancer	Breast	3.30E-01	8.33E-02	1.35E-01	2.29E-01
8736	GSM506312	GSE20181	Breast Cancer	Breast	2.82E-04	-2.26E-01	1.96E-01	2.06E-01
8737	GSM506313	GSE20181	Breast Cancer	Breast	7.96E-05	-2.43E-01	5.31E-02	-2.77E-01
8738	GSM506314	GSE20181	Breast Cancer	Breast	1.83E-01	1.03E-01	4.51E-02	2.84E-01
8739	GSM506315	GSE20181	Breast Cancer	Breast	3.66E-07	3.05E-01	3.69E-06	5.71E-01
8740	GSM506316	GSE20181	Breast Cancer	Breast	1.34E-11	3.96E-01	1.35E-07	6.43E-01
8741	GSM506317	GSE20181	Breast Cancer	Breast	6.19E-02	-1.32E-01	1.96E-01	2.06E-01
8742	GSM506318	GSE20181	Breast Cancer	Breast	1.89E-01	1.02E-01	3.07E-02	3.02E-01
8743	GSM506319	GSE20181	Breast Cancer	Breast	3.93E-05	2.52E-01	1.34E-04	4.83E-01
8744	GSM506320	GSE20181	Breast Cancer	Breast	5.36E-09	3.45E-01	1.88E-06	5.87E-01
8745	GSM506321	GSE20181	Breast Cancer	Breast	3.25E-04	-2.24E-01	2.08E-01	-2.03E-01
8746	GSM506322	GSE20181	Breast Cancer	Breast	3.21E-01	8.43E-02	8.56E-02	2.53E-01
8747	GSM506323	GSE20181	Breast Cancer	Breast	5.23E-11	3.85E-01	4.03E-08	6.67E-01
8748	GSM506324	GSE20181	Breast Cancer	Breast	2.84E-05	-2.56E-01	6.28E-03	-3.64E-01
8749	GSM506325	GSE20181	Breast Cancer	Breast	1.75E-08	3.34E-01	5.29E-06	5.63E-01
8750	GSM506326	GSE20181	Breast Cancer	Breast	1.65E-01	-1.06E-01	2.22E-01	1.98E-01
8751	GSM655624	GSE26639	Breast Cancer	Breast	6.67E-09	-3.43E-01	1.53E-03	-4.11E-01
8752	GSM655627	GSE26639	Breast Cancer	Breast	3.67E-02	-1.44E-01	2.49E-01	1.90E-01
8753	GSM655628	GSE26639	Breast Cancer	Breast	2.10E-03	-1.96E-01	7.46E-02	-2.60E-01
8754	GSM655630	GSE26639	Breast Cancer	Breast	7.97E-04	-2.11E-01	4.64E-01	-1.42E-01
8755	GSM655633	GSE26639	Breast Cancer	Breast	2.84E-05	-2.56E-01	2.24E-02	-3.15E-01
8756	GSM655642	GSE26639	Breast Cancer	Breast	3.05E-02	1.48E-01	6.17E-03	3.64E-01
8757	GSM655644	GSE26639	Breast Cancer	Breast	1.71E-01	-1.05E-01	7.27E-02	2.62E-01
8758	GSM655650	GSE26639	Breast Cancer	Breast	3.42E-03	-1.88E-01	2.90E-01	-1.80E-01
8759	GSM655653	GSE26639	Breast Cancer	Breast	5.27E-06	-2.76E-01	8.02E-03	-3.55E-01
8760	GSM655657	GSE26639	Breast Cancer	Breast	1.01E-02	1.70E-01	1.83E-02	3.23E-01
8761	GSM655658	GSE26639	Breast Cancer	Breast	1.77E-01	-1.04E-01	1.51E-01	2.22E-01
8762	GSM655659	GSE26639	Breast Cancer	Breast	4.83E-01	-6.75E-02	2.22E-01	1.98E-01
8763	GSM655661	GSE26639	Breast Cancer	Breast	1.04E-11	-3.98E-01	1.34E-04	-4.83E-01
8764	GSM655662	GSE26639	Breast Cancer	Breast	2.67E-08	-3.30E-01	2.01E-03	-4.03E-01
8765	GSM655664	GSE26639	Breast Cancer	Breast	1.65E-06	-2.89E-01	6.28E-03	-3.64E-01
8766	GSM655666	GSE26639	Breast Cancer	Breast	1.18E-03	-2.05E-01	1.98E-01	2.06E-01
8767	GSM655670	GSE26639	Breast Cancer	Breast	1.53E-02	1.62E-01	3.07E-02	3.02E-01
8768	GSM655672	GSE26639	Breast Cancer	Breast	5.16E-03	1.82E-01	6.98E-04	4.36E-01
8769	GSM655676	GSE26639	Breast Cancer	Breast	2.62E-05	-2.57E-01	7.55E-02	-2.60E-01
8770	GSM655677	GSE26639	Breast Cancer	Breast	2.28E-01	9.62E-02	2.83E-01	1.82E-01
8771	GSM655679	GSE26639	Breast Cancer	Breast	1.18E-03	2.05E-01	6.62E-03	3.62E-01
8772	GSM655680	GSE26639	Breast Cancer	Breast	1.25E-02	-1.66E-01	1.98E-01	-2.06E-01
8773	GSM655683	GSE26639	Breast Cancer	Breast	3.04E-01	8.63E-02	3.07E-02	3.02E-01
8774	GSM655686	GSE26639	Breast Cancer	Breast	2.40E-02	1.53E-01	4.45E-02	2.85E-01
8775	GSM655692	GSE26639	Breast Cancer	Breast	3.39E-06	2.81E-01	6.83E-04	4.36E-01
8776	GSM655693	GSE26639	Breast Cancer	Breast	6.46E-02	-1.31E-01	1.51E-01	2.22E-01
8777	GSM655695	GSE26639	Breast Cancer	Breast	9.27E-05	2.41E-01	7.61E-03	3.57E-01
8778	GSM655697	GSE26639	Breast Cancer	Breast	4.02E-04	-2.21E-01	3.86E-02	-2.91E-01
8779	GSM655704	GSE26639	Breast Cancer	Breast	1.63E-03	-2.00E-01	1.35E-01	-2.29E-01
8780	GSM655706	GSE26639	Breast Cancer	Breast	2.24E-03	-1.95E-01	1.06E-01	-2.42E-01
8781	GSM655707	GSE26639	Breast Cancer	Breast	1.95E-01	1.01E-01	1.35E-01	2.29E-01
8782	GSM655709	GSE26639	Breast Cancer	Breast	5.16E-03	1.82E-01	4.89E-03	3.73E-01
8783	GSM655710	GSE26639	Breast Cancer	Breast	2.77E-02	1.50E-01	4.51E-02	2.84E-01
8784	GSM655711	GSE26639	Breast Cancer	Breast	9.54E-03	1.71E-01	2.57E-02	3.09E-01
8785	GSM655712	GSE26639	Breast Cancer	Breast	2.40E-02	-1.53E-01	2.25E-01	-1.97E-01
8786	GSM655715	GSE26639	Breast Cancer	Breast	4.02E-04	2.21E-01	7.01E-05	5.00E-01
8787	GSM655716	GSE26639	Breast Cancer	Breast	4.59E-02	1.39E-01	8.56E-02	2.53E-01
8788	GSM655717	GSE26639	Breast Cancer	Breast	7.97E-10	-3.62E-01	1.98E-03	-4.03E-01
8789	GSM655718	GSE26639	Breast Cancer	Breast	1.31E-02	1.65E-01	3.11E-02	3.01E-01
8790	GSM655720	GSE26639	Breast Cancer	Breast	1.06E-02	-1.69E-01	2.80E-01	1.82E-01
8791	GSM655721	GSE26639	Breast Cancer	Breast	6.86E-03	-1.77E-01	5.39E-01	-1.27E-01
8792	GSM655723	GSE26639	Breast Cancer	Breast	2.96E-01	8.73E-02	5.31E-02	2.77E-01
8793	GSM655724	GSE26639	Breast Cancer	Breast	3.23E-03	-1.89E-01	1.37E-01	-2.28E-01
8794	GSM655725	GSE26639	Breast Cancer	Breast	7.97E-10	-3.62E-01	8.30E-03	-3.54E-01
8795	GSM655726	GSE26639	Breast Cancer	Breast	7.54E-11	-3.82E-01	3.26E-04	-4.58E-01
8796	GSM655727	GSE26639	Breast Cancer	Breast	1.12E-02	-1.68E-01	3.96E-01	-1.56E-01
8797	GSM655728	GSE26639	Breast Cancer	Breast	1.00E-01	1.20E-01	5.31E-02	2.77E-01
8798	GSM655729	GSE26639	Breast Cancer	Breast	5.37E-07	-3.01E-01	1.26E-02	-3.38E-01
8799	GSM655730	GSE26639	Breast Cancer	Breast	6.08E-04	2.15E-01	3.12E-04	4.59E-01
8800	GSM655731	GSE26639	Breast Cancer	Breast	3.76E-12	-4.06E-01	5.07E-03	-3.71E-01
8801	GSM655732	GSE26639	Breast Cancer	Breast	4.83E-06	2.77E-01	1.73E-04	4.76E-01

8802	GSM655733	GSE26639	Breast Cancer	Breast	2.10E-03	-1.96E-01	1.52E-01	-2.22E-01
8803	GSM655734	GSE26639	Breast Cancer	Breast	2.14E-01	9.82E-02	4.51E-02	2.84E-01
8804	GSM655735	GSE26639	Breast Cancer	Breast	2.86E-03	-1.91E-01	3.14E-01	-1.74E-01
8805	GSM655736	GSE26639	Breast Cancer	Breast	1.63E-03	2.00E-01	4.89E-03	3.73E-01
8806	GSM655737	GSE26639	Breast Cancer	Breast	1.01E-02	-1.70E-01	3.11E-01	1.75E-01
8807	GSM655738	GSE26639	Breast Cancer	Breast	1.53E-02	-1.62E-01	4.26E-01	-1.49E-01
8808	GSM655739	GSE26639	Breast Cancer	Breast	3.62E-05	-2.53E-01	3.92E-02	-2.91E-01
8809	GSM655740	GSE26639	Breast Cancer	Breast	4.34E-03	-1.85E-01	2.49E-01	1.90E-01
8810	GSM655741	GSE26639	Breast Cancer	Breast	5.75E-06	-2.75E-01	7.74E-02	-2.58E-01
8811	GSM655742	GSE26639	Breast Cancer	Breast	2.07E-02	1.56E-01	3.07E-02	3.02E-01
8812	GSM655743	GSE26639	Breast Cancer	Breast	2.91E-02	-1.49E-01	5.31E-02	-2.77E-01
8813	GSM655744	GSE26639	Breast Cancer	Breast	3.93E-05	-2.52E-01	1.15E-01	-2.37E-01
8814	GSM655745	GSE26639	Breast Cancer	Breast	6.08E-04	2.15E-01	6.06E-03	3.65E-01
8815	GSM655746	GSE26639	Breast Cancer	Breast	1.44E-03	-2.02E-01	2.00E-01	-2.05E-01
8816	GSM655747	GSE26639	Breast Cancer	Breast	1.30E-01	-1.13E-01	1.32E-01	2.30E-01
8817	GSM655748	GSE26639	Breast Cancer	Breast	1.96E-04	2.31E-01	5.28E-04	4.44E-01
8818	GSM655749	GSE26639	Breast Cancer	Breast	1.04E-01	1.19E-01	8.56E-02	2.53E-01
8819	GSM655750	GSE26639	Breast Cancer	Breast	1.61E-02	1.61E-01	8.56E-02	2.53E-01
8820	GSM655751	GSE26639	Breast Cancer	Breast	4.03E-01	-7.54E-02	2.49E-01	1.90E-01
8821	GSM655752	GSE26639	Breast Cancer	Breast	3.93E-05	-2.52E-01	1.01E-01	-2.44E-01
8822	GSM655753	GSE26639	Breast Cancer	Breast	1.52E-07	3.13E-01	2.38E-04	4.67E-01
8823	GSM655754	GSE26639	Breast Cancer	Breast	1.34E-01	-1.12E-01	4.23E-01	1.50E-01
8824	GSM655755	GSE26639	Breast Cancer	Breast	3.93E-05	-2.52E-01	5.60E-02	-2.74E-01
8825	GSM655756	GSE26639	Breast Cancer	Breast	2.23E-05	2.59E-01	1.98E-03	4.03E-01
8826	GSM655757	GSE26639	Breast Cancer	Breast	2.77E-02	1.50E-01	6.83E-04	4.36E-01
8827	GSM655758	GSE26639	Breast Cancer	Breast	1.06E-02	-1.69E-01	2.29E-01	-1.96E-01
8828	GSM655759	GSE26639	Breast Cancer	Breast	2.38E-03	1.94E-01	9.52E-03	3.49E-01
8829	GSM655760	GSE26639	Breast Cancer	Breast	1.14E-05	2.67E-01	7.01E-05	5.00E-01
8830	GSM655761	GSE26639	Breast Cancer	Breast	2.41E-08	-3.31E-01	4.98E-03	-3.72E-01
8831	GSM655762	GSE26639	Breast Cancer	Breast	1.08E-01	1.18E-01	3.07E-02	3.02E-01
8832	GSM655763	GSE26639	Breast Cancer	Breast	1.70E-02	-1.60E-01	3.49E-01	-1.66E-01
8833	GSM655764	GSE26639	Breast Cancer	Breast	6.51E-04	-2.14E-01	1.57E-01	-2.20E-01
8834	GSM655765	GSE26639	Breast Cancer	Breast	1.01E-02	-1.70E-01	6.15E-02	2.70E-01
8835	GSM655766	GSE26639	Breast Cancer	Breast	4.20E-02	-1.41E-01	1.54E-01	-2.21E-01
8836	GSM655767	GSE26639	Breast Cancer	Breast	1.49E-01	-1.09E-01	2.22E-01	1.98E-01
8837	GSM655768	GSE26639	Breast Cancer	Breast	2.96E-01	8.73E-02	1.73E-01	2.14E-01
8838	GSM655769	GSE26639	Breast Cancer	Breast	7.57E-30	6.48E-01	7.92E-15	9.21E-01
8839	GSM655770	GSE26639	Breast Cancer	Breast	9.54E-03	-1.71E-01	7.74E-02	-2.58E-01
8840	GSM655771	GSE26639	Breast Cancer	Breast	3.67E-02	1.44E-01	5.31E-02	2.77E-01
8841	GSM655772	GSE26639	Breast Cancer	Breast	2.29E-02	1.54E-01	6.15E-02	2.70E-01
8842	GSM655773	GSE26639	Breast Cancer	Breast	4.59E-02	1.39E-01	6.39E-03	3.63E-01
8843	GSM655774	GSE26639	Breast Cancer	Breast	4.73E-01	6.85E-02	1.01E-01	2.44E-01
8844	GSM655775	GSE26639	Breast Cancer	Breast	5.78E-01	5.85E-02	1.01E-01	2.44E-01
8845	GSM655776	GSE26639	Breast Cancer	Breast	1.39E-01	-1.11E-01	2.80E-01	1.82E-01
8846	GSM655777	GSE26639	Breast Cancer	Breast	1.21E-01	-1.15E-01	2.51E-01	1.90E-01
8847	GSM655778	GSE26639	Breast Cancer	Breast	5.79E-03	1.80E-01	6.62E-03	3.62E-01
8848	GSM655779	GSE26639	Breast Cancer	Breast	2.64E-02	1.51E-01	3.11E-02	3.01E-01
8849	GSM655780	GSE26639	Breast Cancer	Breast	8.91E-02	-1.23E-01	3.90E-01	-1.57E-01
8850	GSM655781	GSE26639	Breast Cancer	Breast	6.08E-04	-2.15E-01	2.31E-01	-1.96E-01
8851	GSM655782	GSE26639	Breast Cancer	Breast	1.63E-03	-2.00E-01	5.19E-01	-1.31E-01
8852	GSM655783	GSE26639	Breast Cancer	Breast	3.66E-01	-7.94E-02	2.06E-01	2.03E-01
8853	GSM655784	GSE26639	Breast Cancer	Breast	1.00E-01	-1.20E-01	1.98E-01	2.06E-01
8854	GSM655785	GSE26639	Breast Cancer	Breast	9.51E-16	-4.65E-01	2.44E-04	-4.66E-01
8855	GSM655786	GSE26639	Breast Cancer	Breast	1.97E-03	-1.97E-01	4.64E-01	1.42E-01
8856	GSM655787	GSE26639	Breast Cancer	Breast	2.38E-03	-1.94E-01	7.55E-02	-2.60E-01
8857	GSM655788	GSE26639	Breast Cancer	Breast	5.94E-02	-1.33E-01	4.20E-01	1.50E-01
8858	GSM655789	GSE26639	Breast Cancer	Breast	2.18E-02	1.55E-01	1.17E-01	2.37E-01
8859	GSM655626	GSE26639	Breast Cancer	Breast	1.14E-05	-2.67E-01	1.22E-02	-3.39E-01
8860	GSM655629	GSE26639	Breast Cancer	Breast	1.75E-08	-3.34E-01	3.13E-03	-3.88E-01
8861	GSM655631	GSE26639	Breast Cancer	Breast	1.06E-02	1.69E-01	1.51E-02	3.31E-01
8862	GSM655632	GSE26639	Breast Cancer	Breast	3.68E-21	5.43E-01	9.30E-11	7.77E-01
8863	GSM655634	GSE26639	Breast Cancer	Breast	1.46E-02	1.63E-01	9.68E-03	3.48E-01
8864	GSM655635	GSE26639	Breast Cancer	Breast	1.44E-01	-1.10E-01	1.32E-01	2.30E-01
8865	GSM655638	GSE26639	Breast Cancer	Breast	4.44E-07	3.03E-01	1.43E-05	5.40E-01
8866	GSM655639	GSE26639	Breast Cancer	Breast	1.54E-01	1.08E-01	1.96E-01	2.06E-01
8867	GSM655641	GSE26639	Breast Cancer	Breast	7.30E-02	1.28E-01	1.32E-01	2.30E-01
8868	GSM655643	GSE26639	Breast Cancer	Breast	4.79E-02	1.38E-01	1.51E-01	2.22E-01
8869	GSM655645	GSE26639	Breast Cancer	Breast	5.37E-07	-3.01E-01	5.16E-03	-3.71E-01
8870	GSM655646	GSE26639	Breast Cancer	Breast	3.04E-01	8.63E-02	2.49E-01	1.90E-01
8871	GSM655649	GSE26639	Breast Cancer	Breast	3.67E-02	1.44E-01	9.52E-03	3.49E-01
8872	GSM655651	GSE26639	Breast Cancer	Breast	3.29E-08	3.28E-01	2.93E-07	6.27E-01
8873	GSM655654	GSE26639	Breast Cancer	Breast	4.39E-02	-1.40E-01	3.16E-01	-1.73E-01
8874	GSM655655	GSE26639	Breast Cancer	Breast	8.61E-07	-2.96E-01	5.07E-03	-3.71E-01
8875	GSM655660	GSE26639	Breast Cancer	Breast	4.53E-01	7.04E-02	8.66E-02	2.53E-01

8876	GSM655663	GSE26639	Breast Cancer	Breast	5.94E-02	-1.33E-01	8.56E-02	2.53E-01
8877	GSM655668	GSE26639	Breast Cancer	Breast	3.15E-10	-3.70E-01	1.50E-03	-4.12E-01
8878	GSM655669	GSE26639	Breast Cancer	Breast	4.25E-05	-2.51E-01	4.51E-02	-2.84E-01
8879	GSM655671	GSE26639	Breast Cancer	Breast	3.93E-05	-2.52E-01	6.23E-02	-2.69E-01
8880	GSM655673	GSE26639	Breast Cancer	Breast	3.50E-02	-1.45E-01	4.20E-01	1.50E-01
8881	GSM655674	GSE26639	Breast Cancer	Breast	5.46E-02	1.35E-01	4.38E-02	2.86E-01
8882	GSM655678	GSE26639	Breast Cancer	Breast	1.04E-01	1.19E-01	5.31E-02	2.77E-01
8883	GSM655681	GSE26639	Breast Cancer	Breast	5.23E-02	1.36E-01	3.08E-03	3.89E-01
8884	GSM655684	GSE26639	Breast Cancer	Breast	4.98E-08	-3.24E-01	4.07E-03	-3.79E-01
8885	GSM655687	GSE26639	Breast Cancer	Breast	1.46E-02	-1.63E-01	1.51E-01	2.22E-01
8886	GSM655688	GSE26639	Breast Cancer	Breast	5.15E-01	6.45E-02	1.37E-01	2.28E-01
8887	GSM655690	GSE26639	Breast Cancer	Breast	9.04E-03	1.72E-01	5.64E-04	4.42E-01
8888	GSM655694	GSE26639	Breast Cancer	Breast	1.65E-01	-1.06E-01	4.64E-01	-1.42E-01
8889	GSM655696	GSE26639	Breast Cancer	Breast	1.57E-13	4.30E-01	4.47E-07	6.18E-01
8890	GSM655698	GSE26639	Breast Cancer	Breast	1.12E-01	-1.17E-01	2.22E-01	1.98E-01
8891	GSM655700	GSE26639	Breast Cancer	Breast	3.30E-01	-8.33E-02	1.98E-01	2.06E-01
8892	GSM655701	GSE26639	Breast Cancer	Breast	5.30E-04	-2.17E-01	3.25E-02	-2.99E-01
8893	GSM655702	GSE26639	Breast Cancer	Breast	3.23E-03	-1.89E-01	4.23E-01	-1.50E-01
8894	GSM655703	GSE26639	Breast Cancer	Breast	2.64E-01	9.13E-02	1.51E-01	2.22E-01
8895	GSM655719	GSE26639	Breast Cancer	Breast	3.74E-04	-2.22E-01	1.15E-01	-2.37E-01
8896	GSM655722	GSE26639	Breast Cancer	Breast	6.30E-05	2.46E-01	1.34E-04	4.83E-01
8897	GSM655790	GSE26639	Breast Cancer	Breast	4.59E-02	1.39E-01	2.72E-02	3.07E-01
8898	GSM655791	GSE26639	Breast Cancer	Breast	3.19E-02	-1.47E-01	4.23E-01	1.50E-01
8899	GSM655792	GSE26639	Breast Cancer	Breast	3.74E-04	2.22E-01	2.28E-04	4.68E-01
8900	GSM655793	GSE26639	Breast Cancer	Breast	5.79E-03	-1.80E-01	2.80E-01	1.82E-01
8901	GSM655794	GSE26639	Breast Cancer	Breast	4.20E-02	1.41E-01	1.80E-02	3.24E-01
8902	GSM655795	GSE26639	Breast Cancer	Breast	7.60E-02	1.27E-01	1.18E-02	3.40E-01
8903	GSM655796	GSE26639	Breast Cancer	Breast	2.59E-06	-2.84E-01	2.27E-01	-1.97E-01
8904	GSM655797	GSE26639	Breast Cancer	Breast	7.96E-05	-2.43E-01	7.84E-02	-2.58E-01
8905	GSM655798	GSE26639	Breast Cancer	Breast	8.52E-04	2.10E-01	6.06E-03	3.65E-01
8906	GSM655799	GSE26639	Breast Cancer	Breast	1.00E-04	-2.40E-01	1.77E-01	-2.13E-01
8907	GSM655800	GSE26639	Breast Cancer	Breast	1.36E-16	-4.78E-01	2.05E-05	-5.31E-01
8908	GSM655801	GSE26639	Breast Cancer	Breast	5.15E-01	-6.45E-02	2.80E-01	1.82E-01
8909	GSM655802	GSE26639	Breast Cancer	Breast	3.84E-01	-7.74E-02	1.56E-01	2.20E-01
8910	GSM655803	GSE26639	Breast Cancer	Breast	1.24E-07	-3.15E-01	2.61E-02	-3.09E-01
8911	GSM655804	GSE26639	Breast Cancer	Breast	1.95E-01	1.01E-01	5.03E-01	1.34E-01
8912	GSM655805	GSE26639	Breast Cancer	Breast	8.10E-03	-1.74E-01	2.51E-01	-1.90E-01
8913	GSM655806	GSE26639	Breast Cancer	Breast	9.23E-09	-3.40E-01	5.16E-03	-3.71E-01
8914	GSM655807	GSE26639	Breast Cancer	Breast	3.96E-14	4.39E-01	1.78E-08	6.83E-01
8915	GSM655808	GSE26639	Breast Cancer	Breast	7.01E-02	-1.29E-01	4.29E-01	-1.49E-01
8916	GSM655809	GSE26639	Breast Cancer	Breast	2.36E-13	-4.27E-01	2.05E-05	-5.31E-01
8917	GSM655810	GSE26639	Breast Cancer	Breast	4.01E-17	-4.86E-01	1.93E-06	-5.86E-01
8918	GSM655811	GSE26639	Breast Cancer	Breast	1.44E-01	-1.10E-01	3.11E-01	1.75E-01
8919	GSM655812	GSE26639	Breast Cancer	Breast	4.43E-01	7.14E-02	1.52E-01	2.22E-01
8920	GSM655813	GSE26639	Breast Cancer	Breast	7.01E-02	-1.29E-01	4.58E-01	1.43E-01
8921	GSM655637	GSE26639	Breast Cancer	Breast	7.97E-10	-3.62E-01	5.76E-04	-4.41E-01
8922	GSM655648	GSE26639	Breast Cancer	Breast	3.74E-04	-2.22E-01	1.15E-01	-2.37E-01
8923	GSM655689	GSE26639	Breast Cancer	Breast	4.59E-02	-1.39E-01	3.55E-01	-1.64E-01
8924	GSM655691	GSE26639	Breast Cancer	Breast	1.88E-02	-1.58E-01	1.52E-01	-2.22E-01
8925	GSM655699	GSE26639	Breast Cancer	Breast	3.01E-07	-3.07E-01	8.56E-02	-2.53E-01
8926	GSM655705	GSE26639	Breast Cancer	Breast	6.32E-01	-5.36E-02	3.99E-01	1.55E-01
8927	GSM655714	GSE26639	Breast Cancer	Breast	1.37E-06	-2.91E-01	8.99E-02	-2.51E-01
8928	GSM655814	GSE26639	Breast Cancer	Breast	1.85E-07	3.12E-01	1.88E-06	5.87E-01
8929	GSM655815	GSE26639	Breast Cancer	Breast	2.64E-02	-1.51E-01	1.41E-01	2.26E-01
8930	GSM655816	GSE26639	Breast Cancer	Breast	8.52E-04	-2.10E-01	1.22E-01	-2.34E-01
8931	GSM655817	GSE26639	Breast Cancer	Breast	3.74E-35	7.04E-01	7.15E-14	8.89E-01
8932	GSM655818	GSE26639	Breast Cancer	Breast	1.18E-02	-1.67E-01	1.77E-01	-2.13E-01
8933	GSM655819	GSE26639	Breast Cancer	Breast	1.21E-01	-1.15E-01	1.96E-01	2.06E-01
8934	GSM655820	GSE26639	Breast Cancer	Breast	1.08E-01	-1.18E-01	3.90E-01	-1.57E-01
8935	GSM655821	GSE26639	Breast Cancer	Breast	4.98E-05	-2.49E-01	1.24E-01	-2.34E-01
8936	GSM655822	GSE26639	Breast Cancer	Breast	1.06E-02	-1.69E-01	1.75E-01	2.13E-01
8937	GSM655823	GSE26639	Breast Cancer	Breast	3.50E-02	1.45E-01	3.16E-02	3.00E-01
8938	GSM655824	GSE26639	Breast Cancer	Breast	9.26E-02	1.22E-01	2.57E-02	3.09E-01
8939	GSM655825	GSE26639	Breast Cancer	Breast	6.64E-01	-5.06E-02	2.22E-01	1.98E-01
8940	GSM655625	GSE26639	Breast Cancer	Breast	5.23E-02	-1.36E-01	3.19E-01	1.73E-01
8941	GSM655636	GSE26639	Breast Cancer	Breast	1.69E-24	5.85E-01	4.63E-12	8.25E-01
8942	GSM655640	GSE26639	Breast Cancer	Breast	3.84E-01	7.74E-02	2.22E-01	1.98E-01
8943	GSM655647	GSE26639	Breast Cancer	Breast	2.10E-03	1.96E-01	1.90E-03	4.04E-01
8944	GSM655652	GSE26639	Breast Cancer	Breast	8.24E-02	1.25E-01	7.27E-02	2.62E-01
8945	GSM655656	GSE26639	Breast Cancer	Breast	6.08E-04	2.15E-01	2.05E-03	4.02E-01
8946	GSM655665	GSE26639	Breast Cancer	Breast	2.72E-01	9.03E-02	1.96E-01	2.06E-01
8947	GSM655667	GSE26639	Breast Cancer	Breast	8.56E-03	1.73E-01	9.36E-03	3.49E-01
8948	GSM655675	GSE26639	Breast Cancer	Breast	1.30E-01	1.13E-01	1.83E-02	3.23E-01
8949	GSM655682	GSE26639	Breast Cancer	Breast	1.60E-01	1.07E-01	3.76E-02	2.93E-01

8950	GSM655685	GSE26639	Breast Cancer	Breast	3.74E-04	-2.22E-01	3.16E-01	-1.73E-01
8951	GSM655708	GSE26639	Breast Cancer	Breast	4.03E-01	7.54E-02	1.59E-01	2.19E-01
8952	GSM655713	GSE26639	Breast Cancer	Breast	1.97E-03	-1.97E-01	2.31E-01	-1.96E-01
8953	GSM655826	GSE26639	Breast Cancer	Breast	7.01E-02	-1.29E-01	2.49E-01	1.90E-01
8954	GSM655827	GSE26639	Breast Cancer	Breast	2.38E-03	-1.94E-01	3.16E-01	-1.73E-01
8955	GSM655828	GSE26639	Breast Cancer	Breast	7.30E-02	-1.28E-01	3.20E-02	3.00E-01
8956	GSM655829	GSE26639	Breast Cancer	Breast	1.18E-03	2.05E-01	1.77E-02	3.24E-01
8957	GSM655830	GSE26639	Breast Cancer	Breast	5.46E-02	-1.35E-01	2.80E-01	1.82E-01
8958	GSM655831	GSE26639	Breast Cancer	Breast	2.42E-05	-2.58E-01	2.95E-01	-1.78E-01
8959	GSM655832	GSE26639	Breast Cancer	Breast	5.37E-07	-3.01E-01	1.24E-02	-3.38E-01
8960	GSM655833	GSE26639	Breast Cancer	Breast	1.18E-03	-2.05E-01	8.99E-02	-2.51E-01
8961	GSM655834	GSE26639	Breast Cancer	Breast	5.46E-02	-1.35E-01	3.46E-01	1.66E-01
8962	GSM655835	GSE26639	Breast Cancer	Breast	1.58E-09	-3.56E-01	3.19E-04	-4.58E-01
8963	GSM655836	GSE26639	Breast Cancer	Breast	2.96E-01	8.73E-02	1.40E-01	2.27E-01
8964	GSM655837	GSE26639	Breast Cancer	Breast	2.48E-07	3.09E-01	9.83E-05	4.91E-01
8965	GSM655838	GSE26639	Breast Cancer	Breast	6.73E-02	-1.30E-01	2.49E-01	1.90E-01
8966	GSM655839	GSE26639	Breast Cancer	Breast	1.95E-08	-3.33E-01	2.61E-02	-3.09E-01
8967	GSM655840	GSE26639	Breast Cancer	Breast	2.28E-01	-9.62E-02	3.46E-01	1.66E-01
8968	GSM655841	GSE26639	Breast Cancer	Breast	1.04E-01	1.19E-01	6.28E-03	3.64E-01
8969	GSM655842	GSE26639	Breast Cancer	Breast	1.74E-03	-1.99E-01	2.93E-01	-1.79E-01
8970	GSM655843	GSE26639	Breast Cancer	Breast	2.48E-07	-3.09E-01	2.33E-04	-4.67E-01
8971	GSM655844	GSE26639	Breast Cancer	Breast	1.24E-05	2.66E-01	1.37E-04	4.82E-01
8972	GSM655845	GSE26639	Breast Cancer	Breast	2.76E-09	-3.51E-01	1.22E-03	-4.18E-01
8973	GSM655846	GSE26639	Breast Cancer	Breast	5.23E-02	-1.36E-01	2.54E-01	-1.89E-01
8974	GSM655847	GSE26639	Breast Cancer	Breast	4.39E-02	-1.40E-01	2.51E-01	1.90E-01
8975	GSM655848	GSE26639	Breast Cancer	Breast	5.46E-02	-1.35E-01	2.25E-01	1.97E-01
8976	GSM655849	GSE26639	Breast Cancer	Breast	8.13E-06	-2.71E-01	6.51E-03	-3.63E-01
8977	GSM232194	GSE9195	Breast Cancer	Breast	2.69E-03	-1.92E-01	1.40E-01	-2.27E-01
8978	GSM232195	GSE9195	Breast Cancer	Breast	1.54E-01	1.08E-01	3.16E-02	3.00E-01
8979	GSM232196	GSE9195	Breast Cancer	Breast	4.60E-05	-2.50E-01	1.77E-01	-2.13E-01
8980	GSM232197	GSE9195	Breast Cancer	Breast	1.31E-02	1.65E-01	9.85E-03	3.47E-01
8981	GSM232198	GSE9195	Breast Cancer	Breast	4.03E-07	-3.04E-01	4.00E-03	-3.80E-01
8982	GSM232199	GSE9195	Breast Cancer	Breast	1.63E-03	2.00E-01	5.64E-04	4.42E-01
8983	GSM232200	GSE9195	Breast Cancer	Breast	6.08E-04	-2.15E-01	4.51E-02	-2.84E-01
8984	GSM232201	GSE9195	Breast Cancer	Breast	1.05E-05	-2.68E-01	6.48E-02	-2.67E-01
8985	GSM232202	GSE9195	Breast Cancer	Breast	2.50E-11	-3.91E-01	2.59E-03	-3.94E-01
8986	GSM232203	GSE9195	Breast Cancer	Breast	1.83E-01	1.03E-01	5.31E-02	2.77E-01
8987	GSM232204	GSE9195	Breast Cancer	Breast	6.46E-02	-1.31E-01	1.14E-01	2.38E-01
8988	GSM232205	GSE9195	Breast Cancer	Breast	4.59E-02	-1.39E-01	3.81E-01	1.59E-01
8989	GSM232206	GSE9195	Breast Cancer	Breast	1.97E-02	-1.57E-01	3.11E-01	1.75E-01
8990	GSM232207	GSE9195	Breast Cancer	Breast	1.57E-04	2.34E-01	2.28E-04	4.68E-01
8991	GSM232208	GSE9195	Breast Cancer	Breast	4.53E-01	7.04E-02	5.31E-02	2.77E-01
8992	GSM232209	GSE9195	Breast Cancer	Breast	1.30E-01	1.13E-01	5.31E-02	2.77E-01
8993	GSM232210	GSE9195	Breast Cancer	Breast	2.35E-01	9.52E-02	4.38E-02	2.86E-01
8994	GSM232211	GSE9195	Breast Cancer	Breast	1.04E-01	-1.19E-01	3.14E-01	1.74E-01
8995	GSM232212	GSE9195	Breast Cancer	Breast	4.39E-02	1.40E-01	1.75E-02	3.25E-01
8996	GSM232213	GSE9195	Breast Cancer	Breast	4.79E-02	1.38E-01	5.24E-02	2.77E-01
8997	GSM232214	GSE9195	Breast Cancer	Breast	7.60E-02	-1.27E-01	3.81E-01	1.59E-01
8998	GSM232215	GSE9195	Breast Cancer	Breast	2.10E-03	-1.96E-01	3.16E-01	-1.73E-01
8999	GSM232216	GSE9195	Breast Cancer	Breast	8.50E-11	3.81E-01	1.83E-06	5.87E-01
9000	GSM232217	GSE9195	Breast Cancer	Breast	9.27E-05	-2.41E-01	2.27E-02	-3.14E-01
9001	GSM232218	GSE9195	Breast Cancer	Breast	3.38E-01	8.23E-02	5.43E-01	1.26E-01
9002	GSM232219	GSE9195	Breast Cancer	Breast	2.96E-01	8.73E-02	1.96E-01	2.06E-01
9003	GSM232220	GSE9195	Breast Cancer	Breast	2.38E-03	1.94E-01	9.90E-02	2.46E-01
9004	GSM232221	GSE9195	Breast Cancer	Breast	9.65E-06	-2.69E-01	2.45E-03	-3.96E-01
9005	GSM232222	GSE9195	Breast Cancer	Breast	2.52E-02	-1.52E-01	4.74E-01	-1.40E-01
9006	GSM232223	GSE9195	Breast Cancer	Breast	7.97E-04	2.11E-01	2.24E-02	3.15E-01
9007	GSM232224	GSE9195	Breast Cancer	Breast	3.86E-03	1.87E-01	2.14E-02	3.17E-01
9008	GSM232225	GSE9195	Breast Cancer	Breast	7.67E-03	-1.75E-01	2.29E-01	-1.96E-01
9009	GSM232226	GSE9195	Breast Cancer	Breast	3.54E-10	-3.69E-01	7.28E-04	-4.34E-01
9010	GSM232227	GSE9195	Breast Cancer	Breast	1.49E-01	-1.09E-01	5.24E-02	2.77E-01
9011	GSM232228	GSE9195	Breast Cancer	Breast	1.25E-06	-2.92E-01	4.15E-03	-3.78E-01
9012	GSM232229	GSE9195	Breast Cancer	Breast	4.03E-07	-3.04E-01	2.49E-03	-3.96E-01
9013	GSM232230	GSE9195	Breast Cancer	Breast	3.04E-01	-8.63E-02	1.14E-01	2.38E-01
9014	GSM232231	GSE9195	Breast Cancer	Breast	1.24E-05	2.66E-01	3.05E-04	4.60E-01
9015	GSM232232	GSE9195	Breast Cancer	Breast	8.52E-04	2.10E-01	4.89E-03	3.73E-01
9016	GSM232233	GSE9195	Breast Cancer	Breast	1.88E-02	1.58E-01	6.98E-04	4.36E-01
9017	GSM232234	GSE9195	Breast Cancer	Breast	1.97E-02	1.57E-01	4.38E-02	2.86E-01
9018	GSM232235	GSE9195	Breast Cancer	Breast	9.27E-05	2.41E-01	3.05E-04	4.60E-01
9019	GSM232236	GSE9195	Breast Cancer	Breast	2.53E-03	-1.93E-01	2.63E-01	-1.87E-01
9020	GSM232237	GSE9195	Breast Cancer	Breast	6.08E-04	-2.15E-01	5.45E-02	-2.76E-01
9021	GSM232238	GSE9195	Breast Cancer	Breast	3.34E-05	-2.54E-01	1.46E-02	-3.32E-01
9022	GSM232239	GSE9195	Breast Cancer	Breast	2.42E-05	-2.58E-01	2.14E-02	-3.17E-01
9023	GSM232240	GSE9195	Breast Cancer	Breast	3.34E-05	-2.54E-01	3.11E-02	-3.01E-01

9024	GSM232241	GSE9195	Breast Cancer	Breast	1.53E-03	-2.01E-01	2.58E-01	-1.88E-01
9025	GSM232242	GSE9195	Breast Cancer	Breast	1.11E-03	2.06E-01	6.62E-03	3.62E-01
9026	GSM232243	GSE9195	Breast Cancer	Breast	1.69E-04	-2.33E-01	1.15E-01	-2.37E-01
9027	GSM232244	GSE9195	Breast Cancer	Breast	3.94E-01	-7.64E-02	2.25E-01	1.97E-01
9028	GSM232245	GSE9195	Breast Cancer	Breast	1.16E-01	1.16E-01	2.80E-01	1.82E-01
9029	GSM232246	GSE9195	Breast Cancer	Breast	9.19E-12	-3.99E-01	2.38E-04	-4.67E-01
9030	GSM232247	GSE9195	Breast Cancer	Breast	7.84E-07	2.97E-01	2.64E-06	5.79E-01
9031	GSM232248	GSE9195	Breast Cancer	Breast	2.07E-02	1.56E-01	1.77E-02	3.24E-01
9032	GSM232249	GSE9195	Breast Cancer	Breast	4.60E-03	-1.84E-01	4.20E-01	1.50E-01
9033	GSM232250	GSE9195	Breast Cancer	Breast	1.75E-08	-3.34E-01	1.49E-02	-3.31E-01
9034	GSM232251	GSE9195	Breast Cancer	Breast	1.82E-04	-2.32E-01	3.25E-02	-2.99E-01
9035	GSM232252	GSE9195	Breast Cancer	Breast	1.24E-05	-2.66E-01	8.77E-02	-2.52E-01
9036	GSM232253	GSE9195	Breast Cancer	Breast	3.71E-06	-2.80E-01	3.16E-02	-3.00E-01
9037	GSM232254	GSE9195	Breast Cancer	Breast	4.01E-02	-1.42E-01	3.11E-01	1.75E-01
9038	GSM232255	GSE9195	Breast Cancer	Breast	2.35E-01	9.52E-02	1.96E-01	2.06E-01
9039	GSM232256	GSE9195	Breast Cancer	Breast	1.85E-03	-1.98E-01	2.63E-01	-1.87E-01
9040	GSM232257	GSE9195	Breast Cancer	Breast	1.08E-01	1.18E-01	8.45E-02	2.54E-01
9041	GSM232258	GSE9195	Breast Cancer	Breast	1.71E-01	-1.05E-01	1.15E-01	2.37E-01
9042	GSM232259	GSE9195	Breast Cancer	Breast	5.23E-02	-1.36E-01	2.49E-01	1.90E-01
9043	GSM232260	GSE9195	Breast Cancer	Breast	7.44E-09	3.42E-01	2.11E-05	5.30E-01
9044	GSM232261	GSE9195	Breast Cancer	Breast	6.30E-05	-2.46E-01	6.48E-02	-2.67E-01
9045	GSM232262	GSE9195	Breast Cancer	Breast	2.49E-01	-9.33E-02	1.14E-01	2.38E-01
9046	GSM232263	GSE9195	Breast Cancer	Breast	7.60E-02	-1.27E-01	4.02E-01	-1.54E-01
9047	GSM232264	GSE9195	Breast Cancer	Breast	6.73E-02	-1.30E-01	9.90E-02	2.46E-01
9048	GSM232265	GSE9195	Breast Cancer	Breast	4.60E-03	-1.84E-01	3.11E-01	1.75E-01
9049	GSM232266	GSE9195	Breast Cancer	Breast	2.59E-06	-2.84E-01	4.70E-02	-2.83E-01
9050	GSM232267	GSE9195	Breast Cancer	Breast	2.64E-01	9.13E-02	4.98E-03	3.72E-01
9051	GSM232268	GSE9195	Breast Cancer	Breast	5.46E-02	1.35E-01	1.35E-01	2.29E-01
9052	GSM232269	GSE9195	Breast Cancer	Breast	1.21E-01	-1.15E-01	1.59E-01	2.19E-01
9053	GSM232270	GSE9195	Breast Cancer	Breast	7.92E-02	-1.26E-01	1.14E-01	2.38E-01
9054	GSM120649	GSE5327	Breast Cancer	Breast	1.44E-03	2.02E-01	4.98E-03	3.72E-01
9055	GSM120651	GSE5327	Breast Cancer	Breast	4.39E-02	1.40E-01	3.20E-02	3.00E-01
9056	GSM120652	GSE5327	Breast Cancer	Breast	1.57E-13	-4.30E-01	5.64E-04	-4.42E-01
9057	GSM120653	GSE5327	Breast Cancer	Breast	3.47E-01	8.13E-02	3.49E-01	1.66E-01
9058	GSM120654	GSE5327	Breast Cancer	Breast	2.72E-01	9.03E-02	1.77E-01	2.13E-01
9059	GSM120655	GSE5327	Breast Cancer	Breast	5.01E-02	-1.37E-01	3.19E-01	-1.73E-01
9060	GSM120656	GSE5327	Breast Cancer	Breast	1.60E-01	1.07E-01	2.57E-02	3.09E-01
9061	GSM120657	GSE5327	Breast Cancer	Breast	4.53E-01	7.04E-02	1.24E-01	2.34E-01
9062	GSM120658	GSE5327	Breast Cancer	Breast	1.25E-02	-1.66E-01	7.29E-01	9.08E-02
9063	GSM120659	GSE5327	Breast Cancer	Breast	2.84E-06	-2.83E-01	3.81E-02	-2.92E-01
9064	GSM120660	GSE5327	Breast Cancer	Breast	2.29E-02	1.54E-01	7.88E-03	3.56E-01
9065	GSM120661	GSE5327	Breast Cancer	Breast	2.28E-01	9.62E-02	9.85E-03	3.47E-01
9066	GSM120662	GSE5327	Breast Cancer	Breast	1.39E-01	-1.11E-01	4.57E-02	2.84E-01
9067	GSM120663	GSE5327	Breast Cancer	Breast	1.60E-05	2.63E-01	7.19E-05	4.99E-01
9068	GSM120664	GSE5327	Breast Cancer	Breast	5.91E-07	-3.00E-01	4.45E-02	-2.85E-01
9069	GSM120665	GSE5327	Breast Cancer	Breast	3.63E-03	-1.88E-01	1.77E-01	-2.13E-01
9070	GSM120666	GSE5327	Breast Cancer	Breast	2.18E-02	-1.55E-01	5.89E-01	-1.17E-01
9071	GSM120667	GSE5327	Breast Cancer	Breast	8.91E-02	-1.23E-01	1.32E-01	2.30E-01
9072	GSM120668	GSE5327	Breast Cancer	Breast	3.74E-04	-2.22E-01	3.55E-01	-1.64E-01
9073	GSM120669	GSE5327	Breast Cancer	Breast	9.04E-03	1.72E-01	1.77E-02	3.24E-01
9074	GSM120670	GSE5327	Breast Cancer	Breast	2.53E-03	-1.93E-01	2.61E-02	-3.09E-01
9075	GSM120671	GSE5327	Breast Cancer	Breast	2.86E-03	-1.91E-01	1.54E-01	-2.21E-01
9076	GSM120672	GSE5327	Breast Cancer	Breast	1.06E-02	1.69E-01	1.44E-02	3.33E-01
9077	GSM120673	GSE5327	Breast Cancer	Breast	2.86E-03	-1.91E-01	2.88E-01	-1.80E-01
9078	GSM120674	GSE5327	Breast Cancer	Breast	7.84E-07	-2.97E-01	2.17E-02	-3.16E-01
9079	GSM120675	GSE5327	Breast Cancer	Breast	2.40E-02	1.53E-01	3.76E-02	2.93E-01
9080	GSM120676	GSE5327	Breast Cancer	Breast	7.96E-05	-2.43E-01	8.56E-02	-2.53E-01
9081	GSM120677	GSE5327	Breast Cancer	Breast	6.46E-02	-1.31E-01	2.33E-01	1.95E-01
9082	GSM120678	GSE5327	Breast Cancer	Breast	2.52E-02	1.52E-01	3.13E-03	3.88E-01
9083	GSM120679	GSE5327	Breast Cancer	Breast	1.30E-01	-1.13E-01	1.73E-01	2.14E-01
9084	GSM120680	GSE5327	Breast Cancer	Breast	7.92E-02	-1.26E-01	6.73E-01	1.02E-01
9085	GSM120681	GSE5327	Breast Cancer	Breast	1.50E-19	-5.21E-01	2.87E-06	-5.77E-01
9086	GSM120682	GSE5327	Breast Cancer	Breast	1.83E-01	1.03E-01	2.59E-03	3.94E-01
9087	GSM120683	GSE5327	Breast Cancer	Breast	6.73E-02	1.30E-01	3.85E-03	3.81E-01
9088	GSM120684	GSE5327	Breast Cancer	Breast	6.08E-04	-2.15E-01	6.06E-01	1.14E-01
9089	GSM120685	GSE5327	Breast Cancer	Breast	8.56E-03	-1.73E-01	1.75E-01	-2.13E-01
9090	GSM120686	GSE5327	Breast Cancer	Breast	5.68E-04	-2.16E-01	6.48E-02	-2.67E-01
9091	GSM120687	GSE5327	Breast Cancer	Breast	2.88E-01	8.83E-02	1.98E-01	2.06E-01
9092	GSM120688	GSE5327	Breast Cancer	Breast	1.97E-02	1.57E-01	2.45E-03	3.96E-01
9093	GSM120689	GSE5327	Breast Cancer	Breast	9.46E-07	2.95E-01	2.40E-03	3.97E-01
9094	GSM120690	GSE5327	Breast Cancer	Breast	1.35E-03	2.03E-01	9.85E-03	3.47E-01
9095	GSM120691	GSE5327	Breast Cancer	Breast	6.10E-16	4.68E-01	8.99E-08	6.51E-01
9096	GSM120692	GSE5327	Breast Cancer	Breast	2.28E-01	-9.62E-02	2.27E-01	-1.97E-01
9097	GSM120693	GSE5327	Breast Cancer	Breast	1.08E-01	-1.18E-01	2.85E-01	-1.81E-01

9098	GSM120694	GSE5327	Breast Cancer	Breast	4.60E-05	-2.50E-01	7.74E-02	-2.58E-01
9099	GSM120695	GSE5327	Breast Cancer	Breast	2.28E-01	9.62E-02	1.03E-01	2.44E-01
9100	GSM120696	GSE5327	Breast Cancer	Breast	3.13E-01	8.53E-02	2.54E-01	1.89E-01
9101	GSM120697	GSE5327	Breast Cancer	Breast	2.77E-02	1.50E-01	2.57E-02	3.09E-01
9102	GSM120698	GSE5327	Breast Cancer	Breast	1.21E-01	-1.15E-01	4.45E-01	1.45E-01
9103	GSM120699	GSE5327	Breast Cancer	Breast	1.16E-04	-2.38E-01	1.15E-01	-2.37E-01
9104	GSM120700	GSE5327	Breast Cancer	Breast	1.98E-06	-2.87E-01	6.48E-02	-2.67E-01
9105	GSM120701	GSE5327	Breast Cancer	Breast	1.08E-01	-1.18E-01	2.49E-01	1.90E-01
9106	GSM120702	GSE5327	Breast Cancer	Breast	5.36E-09	3.45E-01	1.07E-05	5.47E-01
9107	GSM120703	GSE5327	Breast Cancer	Breast	6.86E-03	1.77E-01	6.06E-03	3.65E-01
9108	GSM120704	GSE5327	Breast Cancer	Breast	3.47E-01	8.13E-02	1.98E-01	2.06E-01
9109	GSM120705	GSE5327	Breast Cancer	Breast	1.79E-02	-1.59E-01	3.81E-01	1.59E-01
9110	GSM120706	GSE5327	Breast Cancer	Breast	6.86E-03	1.77E-01	2.68E-02	3.07E-01
9111	GSM120707	GSE5327	Breast Cancer	Breast	2.59E-06	-2.84E-01	3.16E-02	-3.00E-01
9112	GSM150794	GSE6532	Breast Cancer	Breast	2.40E-02	-1.53E-01	5.09E-01	-1.33E-01
9113	GSM150795	GSE6532	Breast Cancer	Breast	6.43E-01	5.26E-02	1.16E-02	3.41E-01
9114	GSM150796	GSE6532	Breast Cancer	Breast	3.74E-04	-2.22E-01	1.18E-01	-2.36E-01
9115	GSM150797	GSE6532	Breast Cancer	Breast	6.30E-15	4.52E-01	6.33E-08	6.58E-01
9116	GSM150798	GSE6532	Breast Cancer	Breast	4.43E-01	7.14E-02	1.41E-01	2.26E-01
9117	GSM150799	GSE6532	Breast Cancer	Breast	2.91E-02	-1.49E-01	1.96E-01	2.06E-01
9118	GSM150800	GSE6532	Breast Cancer	Breast	1.65E-01	1.06E-01	3.81E-02	2.92E-01
9119	GSM150801	GSE6532	Breast Cancer	Breast	6.67E-09	3.43E-01	2.64E-06	5.79E-01
9120	GSM65820	GSE6532	Breast Cancer	Breast	2.79E-18	-5.03E-01	1.40E-07	-6.42E-01
9121	GSM65821	GSE6532	Breast Cancer	Breast	9.63E-02	-1.21E-01	3.16E-01	-1.73E-01
9122	GSM65822	GSE6532	Breast Cancer	Breast	5.46E-02	1.35E-01	5.38E-02	2.76E-01
9123	GSM65823	GSE6532	Breast Cancer	Breast	3.71E-06	-2.80E-01	1.18E-02	-3.40E-01
9124	GSM65824	GSE6532	Breast Cancer	Breast	2.35E-01	-9.52E-02	1.98E-01	-2.06E-01
9125	GSM65825	GSE6532	Breast Cancer	Breast	1.03E-08	-3.39E-01	4.00E-03	-3.80E-01
9126	GSM65826	GSE6532	Breast Cancer	Breast	1.01E-02	-1.70E-01	2.49E-01	1.90E-01
9127	GSM65827	GSE6532	Breast Cancer	Breast	4.73E-01	-6.85E-02	3.46E-01	1.66E-01
9128	GSM65828	GSE6532	Breast Cancer	Breast	1.65E-06	-2.89E-01	2.72E-02	-3.07E-01
9129	GSM65829	GSE6532	Breast Cancer	Breast	3.30E-01	8.33E-02	1.17E-01	2.37E-01
9130	GSM65830	GSE6532	Breast Cancer	Breast	2.10E-03	-1.96E-01	3.84E-01	-1.58E-01
9131	GSM65831	GSE6532	Breast Cancer	Breast	8.30E-08	-3.19E-01	1.22E-03	-4.18E-01
9132	GSM65832	GSE6532	Breast Cancer	Breast	5.79E-03	-1.80E-01	4.58E-01	1.43E-01
9133	GSM65833	GSE6532	Breast Cancer	Breast	2.91E-02	-1.49E-01	4.32E-01	-1.48E-01
9134	GSM65834	GSE6532	Breast Cancer	Breast	3.42E-03	-1.88E-01	2.25E-01	1.97E-01
9135	GSM65835	GSE6532	Breast Cancer	Breast	2.73E-07	-3.08E-01	2.24E-02	-3.15E-01
9136	GSM65836	GSE6532	Breast Cancer	Breast	1.83E-01	1.03E-01	1.77E-01	2.13E-01
9137	GSM65837	GSE6532	Breast Cancer	Breast	4.60E-03	-1.84E-01	1.56E-01	2.20E-01
9138	GSM65838	GSE6532	Breast Cancer	Breast	2.80E-01	-8.93E-02	3.99E-01	1.55E-01
9139	GSM65839	GSE6532	Breast Cancer	Breast	6.86E-03	-1.77E-01	2.29E-01	-1.96E-01
9140	GSM65840	GSE6532	Breast Cancer	Breast	4.43E-01	7.14E-02	1.51E-01	2.22E-01
9141	GSM65841	GSE6532	Breast Cancer	Breast	2.21E-01	9.72E-02	3.16E-02	3.00E-01
9142	GSM65842	GSE6532	Breast Cancer	Breast	1.06E-02	1.69E-01	2.85E-02	3.05E-01
9143	GSM65843	GSE6532	Breast Cancer	Breast	5.46E-02	1.35E-01	2.20E-02	3.16E-01
9144	GSM65844	GSE6532	Breast Cancer	Breast	1.50E-06	-2.90E-01	2.45E-03	-3.96E-01
9145	GSM65845	GSE6532	Breast Cancer	Breast	1.21E-01	-1.15E-01	3.76E-02	2.93E-01
9146	GSM65846	GSE6532	Breast Cancer	Breast	4.25E-05	-2.51E-01	4.76E-02	-2.82E-01
9147	GSM65847	GSE6532	Breast Cancer	Breast	7.60E-02	-1.27E-01	4.58E-01	1.43E-01
9148	GSM65848	GSE6532	Breast Cancer	Breast	1.12E-01	-1.17E-01	2.22E-01	1.98E-01
9149	GSM65849	GSE6532	Breast Cancer	Breast	1.70E-02	-1.60E-01	1.98E-01	-2.06E-01
9150	GSM65850	GSE6532	Breast Cancer	Breast	4.95E-04	2.18E-01	3.85E-03	3.81E-01
9151	GSM65851	GSE6532	Breast Cancer	Breast	3.03E-04	-2.25E-01	1.34E-01	-2.29E-01
9152	GSM65852	GSE6532	Breast Cancer	Breast	8.24E-02	-1.25E-01	3.52E-01	-1.65E-01
9153	GSM65853	GSE6532	Breast Cancer	Breast	4.13E-01	7.44E-02	2.51E-01	1.90E-01
9154	GSM65854	GSE6532	Breast Cancer	Breast	6.19E-02	1.32E-01	3.70E-02	2.93E-01
9155	GSM65855	GSE6532	Breast Cancer	Breast	1.31E-02	-1.65E-01	2.22E-01	1.98E-01
9156	GSM65856	GSE6532	Breast Cancer	Breast	9.19E-12	-3.99E-01	5.36E-05	-5.07E-01
9157	GSM65857	GSE6532	Breast Cancer	Breast	2.18E-02	-1.55E-01	2.02E-01	-2.04E-01
9158	GSM65858	GSE6532	Breast Cancer	Breast	3.25E-04	2.24E-01	1.94E-03	4.04E-01
9159	GSM65859	GSE6532	Breast Cancer	Breast	2.52E-02	1.52E-01	2.11E-02	3.17E-01
9160	GSM65860	GSE6532	Breast Cancer	Breast	9.18E-08	-3.18E-01	1.54E-02	-3.30E-01
9161	GSM65861	GSE6532	Breast Cancer	Breast	5.68E-01	5.95E-02	1.34E-01	2.29E-01
9162	GSM65862	GSE6532	Breast Cancer	Breast	1.61E-02	-1.61E-01	2.49E-01	1.90E-01
9163	GSM65863	GSE6532	Breast Cancer	Breast	2.84E-05	2.56E-01	1.56E-03	4.11E-01
9164	GSM65864	GSE6532	Breast Cancer	Breast	3.13E-01	-8.53E-02	6.39E-02	2.68E-01
9165	GSM65865	GSE6532	Breast Cancer	Breast	3.86E-09	3.48E-01	7.74E-06	5.54E-01
9166	GSM65866	GSE6532	Breast Cancer	Breast	1.14E-06	-2.93E-01	7.65E-02	-2.59E-01
9167	GSM65867	GSE6532	Breast Cancer	Breast	2.38E-03	-1.94E-01	1.37E-01	-2.28E-01
9168	GSM65868	GSE6532	Breast Cancer	Breast	1.85E-07	-3.12E-01	3.92E-03	-3.80E-01
9169	GSM65869	GSE6532	Breast Cancer	Breast	4.95E-04	-2.18E-01	7.65E-02	-2.59E-01
9170	GSM65870	GSE6532	Breast Cancer	Breast	1.16E-01	1.16E-01	3.70E-02	2.93E-01
9171	GSM65871	GSE6532	Breast Cancer	Breast	3.67E-02	1.44E-01	5.24E-02	2.77E-01

9172	GSM65872	GSE6532	Breast Cancer	Breast	5.47E-03	1.81E-01	1.22E-02	3.39E-01
9173	GSM65873	GSE6532	Breast Cancer	Breast	2.14E-01	9.82E-02	2.49E-01	1.90E-01
9174	GSM65874	GSE6532	Breast Cancer	Breast	6.19E-02	1.32E-01	1.20E-03	4.19E-01
9175	GSM65875	GSE6532	Breast Cancer	Breast	6.03E-13	-4.20E-01	7.53E-06	-5.55E-01
9176	GSM65876	GSE6532	Breast Cancer	Breast	1.73E-27	6.21E-01	7.15E-14	8.89E-01
9177	GSM65877	GSE6532	Breast Cancer	Breast	8.57E-02	-1.24E-01	4.20E-01	1.50E-01
9178	GSM65878	GSE6532	Breast Cancer	Breast	1.74E-03	1.99E-01	3.07E-02	3.02E-01
9179	GSM65879	GSE6532	Breast Cancer	Breast	2.84E-05	2.56E-01	1.53E-03	4.11E-01
9180	GSM65880	GSE6532	Breast Cancer	Breast	2.64E-01	9.13E-02	1.17E-01	2.37E-01
9181	GSM150802	GSE6532	Breast Cancer	Breast	6.28E-06	-2.74E-01	9.85E-03	-3.47E-01
9182	GSM150803	GSE6532	Breast Cancer	Breast	7.25E-03	-1.76E-01	6.06E-01	-1.14E-01
9183	GSM150804	GSE6532	Breast Cancer	Breast	1.26E-03	-2.04E-01	1.17E-01	-2.37E-01
9184	GSM150805	GSE6532	Breast Cancer	Breast	4.01E-02	-1.42E-01	1.18E-01	2.36E-01
9185	GSM65752	GSE6532	Breast Cancer	Breast	4.73E-01	6.85E-02	9.90E-02	2.46E-01
9186	GSM65753	GSE6532	Breast Cancer	Breast	7.97E-04	-2.11E-01	9.10E-02	-2.50E-01
9187	GSM65754	GSE6532	Breast Cancer	Breast	8.10E-03	-1.74E-01	2.56E-01	-1.89E-01
9188	GSM65755	GSE6532	Breast Cancer	Breast	9.19E-12	-3.99E-01	3.80E-06	-5.71E-01
9189	GSM65756	GSE6532	Breast Cancer	Breast	7.97E-10	-3.62E-01	5.28E-04	-4.44E-01
9190	GSM65757	GSE6532	Breast Cancer	Breast	3.45E-09	-3.49E-01	8.98E-04	-4.28E-01
9191	GSM65758	GSE6532	Breast Cancer	Breast	7.60E-02	1.27E-01	3.92E-03	3.80E-01
9192	GSM65760	GSE6532	Breast Cancer	Breast	5.30E-04	-2.17E-01	7.36E-02	-2.61E-01
9193	GSM65761	GSE6532	Breast Cancer	Breast	1.00E-04	-2.40E-01	1.03E-01	-2.44E-01
9194	GSM65762	GSE6532	Breast Cancer	Breast	1.89E-01	-1.02E-01	2.51E-01	1.90E-01
9195	GSM65763	GSE6532	Breast Cancer	Breast	1.18E-03	2.05E-01	1.98E-03	4.03E-01
9196	GSM65764	GSE6532	Breast Cancer	Breast	2.64E-02	1.51E-01	2.57E-02	3.09E-01
9197	GSM65765	GSE6532	Breast Cancer	Breast	2.27E-04	-2.29E-01	2.04E-01	-2.04E-01
9198	GSM65766	GSE6532	Breast Cancer	Breast	8.30E-08	3.19E-01	3.69E-06	5.71E-01
9199	GSM65767	GSE6532	Breast Cancer	Breast	2.84E-06	-2.83E-01	4.98E-03	-3.72E-01
9200	GSM65768	GSE6532	Breast Cancer	Breast	4.73E-01	6.85E-02	1.00E-01	2.45E-01
9201	GSM65769	GSE6532	Breast Cancer	Breast	1.69E-04	2.33E-01	2.33E-04	4.67E-01
9202	GSM65770	GSE6532	Breast Cancer	Breast	5.79E-03	1.80E-01	2.40E-03	3.97E-01
9203	GSM65771	GSE6532	Breast Cancer	Breast	3.67E-02	1.44E-01	3.16E-02	3.00E-01
9204	GSM65772	GSE6532	Breast Cancer	Breast	3.62E-05	2.53E-01	5.58E-06	5.62E-01
9205	GSM65773	GSE6532	Breast Cancer	Breast	9.04E-03	-1.72E-01	3.49E-01	1.66E-01
9206	GSM65774	GSE6532	Breast Cancer	Breast	2.84E-06	-2.83E-01	4.45E-02	-2.85E-01
9207	GSM65775	GSE6532	Breast Cancer	Breast	2.25E-07	3.10E-01	3.78E-05	5.16E-01
9208	GSM65776	GSE6532	Breast Cancer	Breast	1.53E-02	1.62E-01	4.38E-02	2.86E-01
9209	GSM65779	GSE6532	Breast Cancer	Breast	8.91E-02	1.23E-01	3.76E-02	2.93E-01
9210	GSM65780	GSE6532	Breast Cancer	Breast	6.46E-02	-1.31E-01	1.96E-01	2.06E-01
9211	GSM65781	GSE6532	Breast Cancer	Breast	5.46E-15	-4.53E-01	2.11E-05	-5.30E-01
9212	GSM65782	GSE6532	Breast Cancer	Breast	6.46E-02	-1.31E-01	2.25E-01	-1.97E-01
9213	GSM65783	GSE6532	Breast Cancer	Breast	1.44E-01	1.10E-01	2.57E-02	3.09E-01
9214	GSM65784	GSE6532	Breast Cancer	Breast	3.62E-05	-2.53E-01	1.44E-02	-3.33E-01
9215	GSM65785	GSE6532	Breast Cancer	Breast	2.07E-02	-1.56E-01	2.49E-01	1.90E-01
9216	GSM65786	GSE6532	Breast Cancer	Breast	4.87E-12	-4.04E-01	3.88E-05	-5.15E-01
9217	GSM65787	GSE6532	Breast Cancer	Breast	2.35E-01	9.52E-02	1.96E-01	2.06E-01
9218	GSM65788	GSE6532	Breast Cancer	Breast	1.71E-01	1.05E-01	1.14E-01	2.38E-01
9219	GSM65789	GSE6532	Breast Cancer	Breast	1.18E-03	2.05E-01	1.31E-04	4.83E-01
9220	GSM65790	GSE6532	Breast Cancer	Breast	4.28E-12	4.05E-01	1.96E-07	6.35E-01
9221	GSM65791	GSE6532	Breast Cancer	Breast	2.72E-01	-9.03E-02	4.20E-01	1.50E-01
9222	GSM65792	GSE6532	Breast Cancer	Breast	1.60E-01	1.07E-01	3.40E-02	2.97E-01
9223	GSM65793	GSE6532	Breast Cancer	Breast	2.57E-01	-9.23E-02	3.96E-01	1.56E-01
9224	GSM65794	GSE6532	Breast Cancer	Breast	7.92E-02	-1.26E-01	5.45E-02	2.76E-01
9225	GSM65795	GSE6532	Breast Cancer	Breast	4.20E-02	-1.41E-01	3.93E-01	-1.56E-01
9226	GSM65796	GSE6532	Breast Cancer	Breast	2.28E-01	9.62E-02	1.73E-01	2.14E-01
9227	GSM65797	GSE6532	Breast Cancer	Breast	4.73E-01	6.85E-02	2.22E-01	1.98E-01
9228	GSM65798	GSE6532	Breast Cancer	Breast	5.68E-04	-2.16E-01	8.56E-02	-2.53E-01
9229	GSM65799	GSE6532	Breast Cancer	Breast	9.04E-03	-1.72E-01	2.95E-01	-1.78E-01
9230	GSM65800	GSE6532	Breast Cancer	Breast	5.69E-02	1.34E-01	4.38E-02	2.86E-01
9231	GSM65801	GSE6532	Breast Cancer	Breast	7.25E-03	-1.76E-01	3.11E-01	1.75E-01
9232	GSM65802	GSE6532	Breast Cancer	Breast	5.23E-02	-1.36E-01	4.26E-01	1.49E-01
9233	GSM65803	GSE6532	Breast Cancer	Breast	3.04E-01	8.63E-02	5.38E-02	2.76E-01
9234	GSM65804	GSE6532	Breast Cancer	Breast	1.44E-01	1.10E-01	3.20E-02	3.00E-01
9235	GSM65805	GSE6532	Breast Cancer	Breast	5.69E-02	-1.34E-01	5.00E-01	1.35E-01
9236	GSM65806	GSE6532	Breast Cancer	Breast	5.25E-01	-6.35E-02	3.46E-01	1.66E-01
9237	GSM65807	GSE6532	Breast Cancer	Breast	1.65E-06	2.89E-01	7.53E-06	5.55E-01
9238	GSM65808	GSE6532	Breast Cancer	Breast	1.83E-01	1.03E-01	3.07E-02	3.02E-01
9239	GSM65810	GSE6532	Breast Cancer	Breast	1.49E-01	-1.09E-01	4.26E-01	1.49E-01
9240	GSM65811	GSE6532	Breast Cancer	Breast	1.53E-02	-1.62E-01	5.16E-01	-1.31E-01
9241	GSM65812	GSE6532	Breast Cancer	Breast	5.69E-02	1.34E-01	1.46E-02	3.32E-01
9242	GSM65813	GSE6532	Breast Cancer	Breast	1.53E-02	-1.62E-01	1.51E-01	-2.22E-01
9243	GSM65814	GSE6532	Breast Cancer	Breast	8.24E-02	1.25E-01	7.84E-02	2.58E-01
9244	GSM65815	GSE6532	Breast Cancer	Breast	9.23E-09	-3.40E-01	1.54E-02	-3.30E-01
9245	GSM65816	GSE6532	Breast Cancer	Breast	3.42E-03	-1.88E-01	3.55E-01	-1.64E-01

9246	GSM65817	GSE6532	Breast Cancer	Breast	7.97E-04	2.11E-01	9.52E-03	3.49E-01
9247	GSM65818	GSE6532	Breast Cancer	Breast	2.01E-01	-1.00E-01	2.31E-01	1.96E-01
9248	GSM65819	GSE6532	Breast Cancer	Breast	2.96E-01	-8.73E-02	4.58E-01	1.43E-01
9249	GSM150943	GSE6532	Breast Cancer	Breast	5.46E-02	-1.35E-01	3.16E-01	-1.73E-01
9250	GSM150944	GSE6532	Breast Cancer	Breast	6.49E-07	2.99E-01	7.33E-06	5.56E-01
9251	GSM150945	GSE6532	Breast Cancer	Breast	2.29E-02	-1.54E-01	2.85E-01	-1.81E-01
9252	GSM150946	GSE6532	Breast Cancer	Breast	6.03E-13	-4.20E-01	7.36E-05	-4.98E-01
9253	GSM150947	GSE6532	Breast Cancer	Breast	1.25E-06	-2.92E-01	2.27E-02	-3.14E-01
9254	GSM150948	GSE6532	Breast Cancer	Breast	5.98E-09	-3.44E-01	9.85E-03	-3.47E-01
9255	GSM150949	GSE6532	Breast Cancer	Breast	1.16E-01	1.16E-01	9.90E-02	2.46E-01
9256	GSM150950	GSE6532	Breast Cancer	Breast	3.25E-04	-2.24E-01	1.54E-01	-2.21E-01
9257	GSM150951	GSE6532	Breast Cancer	Breast	4.23E-01	7.34E-02	5.39E-01	1.27E-01
9258	GSM150952	GSE6532	Breast Cancer	Breast	7.92E-02	-1.26E-01	1.54E-01	2.21E-01
9259	GSM150953	GSE6532	Breast Cancer	Breast	3.29E-08	-3.28E-01	1.81E-04	-4.74E-01
9260	GSM150954	GSE6532	Breast Cancer	Breast	4.49E-08	3.25E-01	1.07E-05	5.47E-01
9261	GSM150955	GSE6532	Breast Cancer	Breast	1.44E-03	-2.02E-01	3.66E-01	-1.62E-01
9262	GSM150956	GSE6532	Breast Cancer	Breast	2.88E-01	8.83E-02	1.42E-02	3.33E-01
9263	GSM150957	GSE6532	Breast Cancer	Breast	3.13E-01	8.53E-02	7.65E-02	2.59E-01
9264	GSM150958	GSE6532	Breast Cancer	Breast	4.02E-04	2.21E-01	2.40E-03	3.97E-01
9265	GSM150959	GSE6532	Breast Cancer	Breast	3.15E-10	-3.70E-01	5.28E-04	-4.44E-01
9266	GSM150960	GSE6532	Breast Cancer	Breast	4.05E-08	-3.26E-01	7.28E-04	-4.34E-01
9267	GSM150961	GSE6532	Breast Cancer	Breast	2.57E-01	-9.23E-02	2.83E-01	1.82E-01
9268	GSM150962	GSE6532	Breast Cancer	Breast	6.30E-05	-2.46E-01	9.85E-03	-3.47E-01
9269	GSM150963	GSE6532	Breast Cancer	Breast	7.67E-03	1.75E-01	1.18E-02	3.40E-01
9270	GSM150964	GSE6532	Breast Cancer	Breast	2.77E-02	-1.50E-01	2.27E-01	-1.97E-01
9271	GSM150965	GSE6532	Breast Cancer	Breast	4.39E-02	-1.40E-01	3.11E-01	1.75E-01
9272	GSM150966	GSE6532	Breast Cancer	Breast	7.92E-02	1.26E-01	4.98E-03	3.72E-01
9273	GSM150967	GSE6532	Breast Cancer	Breast	4.23E-01	7.34E-02	4.70E-02	2.83E-01
9274	GSM150968	GSE6532	Breast Cancer	Breast	1.63E-03	2.00E-01	4.00E-03	3.80E-01
9275	GSM150969	GSE6532	Breast Cancer	Breast	8.24E-02	-1.25E-01	2.51E-01	1.90E-01
9276	GSM150970	GSE6532	Breast Cancer	Breast	2.38E-03	1.94E-01	3.85E-03	3.81E-01
9277	GSM150971	GSE6532	Breast Cancer	Breast	7.45E-04	-2.12E-01	3.11E-01	-1.75E-01
9278	GSM150972	GSE6532	Breast Cancer	Breast	1.54E-01	-1.08E-01	5.39E-01	1.27E-01
9279	GSM150973	GSE6532	Breast Cancer	Breast	1.08E-10	-3.79E-01	2.05E-05	-5.31E-01
9280	GSM150974	GSE6532	Breast Cancer	Breast	6.89E-14	-4.36E-01	5.23E-05	-5.07E-01
9281	GSM150975	GSE6532	Breast Cancer	Breast	4.98E-05	-2.49E-01	6.48E-02	-2.67E-01
9282	GSM150976	GSE6532	Breast Cancer	Breast	1.65E-01	-1.06E-01	3.22E-01	1.72E-01
9283	GSM150977	GSE6532	Breast Cancer	Breast	2.67E-08	-3.30E-01	2.33E-04	-4.67E-01
9284	GSM150978	GSE6532	Breast Cancer	Breast	3.42E-03	1.88E-01	9.17E-04	4.27E-01
9285	GSM150979	GSE6532	Breast Cancer	Breast	1.70E-02	1.60E-01	2.57E-02	3.09E-01
9286	GSM150980	GSE6532	Breast Cancer	Breast	2.77E-02	-1.50E-01	3.87E-01	-1.57E-01
9287	GSM150981	GSE6532	Breast Cancer	Breast	1.61E-02	-1.61E-01	8.45E-02	2.54E-01
9288	GSM150982	GSE6532	Breast Cancer	Breast	1.97E-02	-1.57E-01	2.25E-01	-1.97E-01
9289	GSM150983	GSE6532	Breast Cancer	Breast	4.63E-11	-3.86E-01	9.17E-04	-4.27E-01
9290	GSM150984	GSE6532	Breast Cancer	Breast	7.97E-01	-3.77E-02	1.80E-01	2.11E-01
9291	GSM150985	GSE6532	Breast Cancer	Breast	7.97E-04	-2.11E-01	2.06E-01	-2.03E-01
9292	GSM150986	GSE6532	Breast Cancer	Breast	4.39E-02	1.40E-01	1.75E-02	3.25E-01
9293	GSM150987	GSE6532	Breast Cancer	Breast	1.04E-01	1.19E-01	7.36E-02	2.61E-01
9294	GSM150988	GSE6532	Breast Cancer	Breast	1.00E-04	-2.40E-01	1.51E-02	-3.31E-01
9295	GSM150989	GSE6532	Breast Cancer	Breast	8.91E-02	1.23E-01	4.38E-02	2.86E-01
9296	GSM150990	GSE6532	Breast Cancer	Breast	3.84E-01	-7.74E-02	4.20E-01	1.50E-01
9297	GSM150991	GSE6532	Breast Cancer	Breast	1.49E-01	-1.09E-01	4.45E-02	2.85E-01
9298	GSM150992	GSE6532	Breast Cancer	Breast	1.83E-01	1.03E-01	2.01E-03	4.03E-01
9299	GSM150993	GSE6532	Breast Cancer	Breast	2.40E-02	-1.53E-01	3.11E-01	1.75E-01
9300	GSM150994	GSE6532	Breast Cancer	Breast	1.12E-07	-3.16E-01	2.31E-02	-3.14E-01
9301	GSM150995	GSE6532	Breast Cancer	Breast	4.60E-03	1.84E-01	3.08E-03	3.89E-01
9302	GSM150996	GSE6532	Breast Cancer	Breast	2.08E-01	-9.92E-02	2.25E-01	-1.97E-01
9303	GSM150997	GSE6532	Breast Cancer	Breast	1.44E-03	2.02E-01	5.17E-04	4.44E-01
9304	GSM150998	GSE6532	Breast Cancer	Breast	1.08E-04	-2.39E-01	6.65E-02	-2.66E-01
9305	GSM150999	GSE6532	Breast Cancer	Breast	1.60E-05	2.63E-01	7.28E-04	4.34E-01
9306	GSM151000	GSE6532	Breast Cancer	Breast	1.61E-02	1.61E-01	6.15E-02	2.70E-01
9307	GSM151001	GSE6532	Breast Cancer	Breast	6.30E-05	-2.46E-01	1.06E-01	-2.42E-01
9308	GSM151002	GSE6532	Breast Cancer	Breast	7.01E-02	-1.29E-01	3.61E-01	1.63E-01
9309	GSM151003	GSE6532	Breast Cancer	Breast	2.57E-01	9.23E-02	2.95E-01	1.78E-01
9310	GSM151004	GSE6532	Breast Cancer	Breast	3.67E-02	-1.44E-01	4.58E-01	1.43E-01
9311	GSM151005	GSE6532	Breast Cancer	Breast	3.54E-10	3.69E-01	2.73E-08	6.74E-01
9312	GSM151006	GSE6532	Breast Cancer	Breast	5.30E-04	2.17E-01	9.56E-04	4.26E-01
9313	GSM151007	GSE6532	Breast Cancer	Breast	4.60E-03	-1.84E-01	1.17E-01	-2.37E-01
9314	GSM151008	GSE6532	Breast Cancer	Breast	1.53E-03	-2.01E-01	3.24E-01	-1.71E-01
9315	GSM151009	GSE6532	Breast Cancer	Breast	5.25E-01	-6.35E-02	1.73E-01	2.14E-01
9316	GSM151010	GSE6532	Breast Cancer	Breast	1.75E-08	3.34E-01	1.29E-06	5.95E-01
9317	GSM151011	GSE6532	Breast Cancer	Breast	4.33E-01	-7.24E-02	2.00E-01	2.05E-01
9318	GSM65340	GSE6532	Breast Cancer	Breast	1.06E-02	1.69E-01	8.02E-03	3.55E-01
9319	GSM65341	GSE6532	Breast Cancer	Breast	6.28E-06	-2.74E-01	1.17E-01	-2.37E-01

9320	GSM65342	GSE6532	Breast Cancer	Breast	1.60E-01	-1.07E-01	1.32E-01	2.30E-01
9321	GSM65343	GSE6532	Breast Cancer	Breast	1.21E-01	-1.15E-01	2.25E-01	1.97E-01
9322	GSM65344	GSE6532	Breast Cancer	Breast	2.82E-04	-2.26E-01	8.77E-02	-2.52E-01
9323	GSM65345	GSE6532	Breast Cancer	Breast	3.94E-01	7.64E-02	1.51E-01	2.22E-01
9324	GSM65346	GSE6532	Breast Cancer	Breast	2.44E-04	2.28E-01	1.56E-03	4.11E-01
9325	GSM65347	GSE6532	Breast Cancer	Breast	3.84E-01	7.74E-02	1.17E-01	2.37E-01
9326	GSM65348	GSE6532	Breast Cancer	Breast	9.08E-14	-4.34E-01	2.44E-04	-4.66E-01
9327	GSM65349	GSE6532	Breast Cancer	Breast	3.03E-04	2.25E-01	1.20E-03	4.19E-01
9328	GSM65350	GSE6532	Breast Cancer	Breast	2.88E-01	8.83E-02	4.63E-02	2.83E-01
9329	GSM65351	GSE6532	Breast Cancer	Breast	7.92E-02	-1.26E-01	1.52E-01	2.22E-01
9330	GSM65352	GSE6532	Breast Cancer	Breast	3.05E-02	1.48E-01	2.05E-03	4.02E-01
9331	GSM65353	GSE6532	Breast Cancer	Breast	8.30E-08	-3.19E-01	6.56E-02	-2.67E-01
9332	GSM65354	GSE6532	Breast Cancer	Breast	1.26E-03	-2.04E-01	1.56E-01	-2.20E-01
9333	GSM65355	GSE6532	Breast Cancer	Breast	1.89E-01	1.02E-01	1.17E-01	2.37E-01
9334	GSM65356	GSE6532	Breast Cancer	Breast	2.18E-02	1.55E-01	3.07E-02	3.02E-01
9335	GSM65357	GSE6532	Breast Cancer	Breast	1.82E-04	2.32E-01	1.47E-03	4.13E-01
9336	GSM65358	GSE6532	Breast Cancer	Breast	3.03E-04	-2.25E-01	6.39E-02	-2.68E-01
9337	GSM65359	GSE6532	Breast Cancer	Breast	2.62E-04	-2.27E-01	1.56E-01	-2.20E-01
9338	GSM65360	GSE6532	Breast Cancer	Breast	1.18E-03	-2.05E-01	1.37E-01	-2.28E-01
9339	GSM65361	GSE6532	Breast Cancer	Breast	1.38E-02	-1.64E-01	6.15E-02	2.70E-01
9340	GSM65362	GSE6532	Breast Cancer	Breast	1.53E-02	1.62E-01	2.27E-02	3.14E-01
9341	GSM65363	GSE6532	Breast Cancer	Breast	2.27E-04	2.29E-01	1.50E-03	4.12E-01
9342	GSM65364	GSE6532	Breast Cancer	Breast	1.53E-02	-1.62E-01	2.51E-01	-1.90E-01
9343	GSM65365	GSE6532	Breast Cancer	Breast	1.44E-03	-2.02E-01	6.67E-01	1.03E-01
9344	GSM65366	GSE6532	Breast Cancer	Breast	5.37E-07	-3.01E-01	1.77E-04	-4.75E-01
9345	GSM65367	GSE6532	Breast Cancer	Breast	4.63E-01	6.94E-02	7.74E-02	2.58E-01
9346	GSM65368	GSE6532	Breast Cancer	Breast	4.01E-02	-1.42E-01	5.00E-01	1.35E-01
9347	GSM65369	GSE6532	Breast Cancer	Breast	3.67E-02	-1.44E-01	3.11E-01	1.75E-01
9348	GSM65370	GSE6532	Breast Cancer	Breast	1.44E-01	-1.10E-01	1.51E-01	2.22E-01
9349	GSM65371	GSE6532	Breast Cancer	Breast	1.65E-06	-2.89E-01	1.20E-02	-3.40E-01
9350	GSM65372	GSE6532	Breast Cancer	Breast	1.27E-08	-3.37E-01	4.98E-03	-3.72E-01
9351	GSM65373	GSE6532	Breast Cancer	Breast	4.49E-08	-3.25E-01	4.15E-03	-3.78E-01
9352	GSM65374	GSE6532	Breast Cancer	Breast	2.37E-06	2.85E-01	2.11E-05	5.30E-01
9353	GSM65375	GSE6532	Breast Cancer	Breast	2.52E-02	-1.52E-01	4.38E-02	2.86E-01
9354	GSM65376	GSE6532	Breast Cancer	Breast	9.26E-02	1.22E-01	2.14E-02	3.17E-01
9355	GSM65377	GSE6532	Breast Cancer	Breast	5.46E-02	-1.35E-01	1.17E-01	-2.37E-01
9356	GSM65378	GSE6532	Breast Cancer	Breast	2.35E-01	-9.52E-02	2.80E-01	1.82E-01
9357	GSM65379	GSE6532	Breast Cancer	Breast	4.59E-02	-1.39E-01	5.59E-01	-1.23E-01
9358	GSM151012	GSE6532	Breast Cancer	Breast	1.89E-01	1.02E-01	7.27E-02	2.62E-01
9359	GSM151013	GSE6532	Breast Cancer	Breast	5.68E-04	-2.16E-01	1.17E-01	-2.37E-01
9360	GSM151014	GSE6532	Breast Cancer	Breast	3.32E-07	-3.06E-01	1.83E-02	-3.23E-01
9361	GSM151015	GSE6532	Breast Cancer	Breast	1.61E-02	-1.61E-01	1.32E-01	2.30E-01
9362	GSM151016	GSE6532	Breast Cancer	Breast	3.74E-04	-2.22E-01	2.54E-01	-1.89E-01
9363	GSM151017	GSE6532	Breast Cancer	Breast	8.56E-03	1.73E-01	1.54E-01	2.21E-01
9364	GSM151018	GSE6532	Breast Cancer	Breast	2.82E-04	2.26E-01	6.98E-04	4.36E-01
9365	GSM151019	GSE6532	Breast Cancer	Breast	1.08E-10	-3.79E-01	1.46E-02	-3.32E-01
9366	GSM151020	GSE6532	Breast Cancer	Breast	4.25E-05	-2.51E-01	1.21E-01	-2.35E-01
9367	GSM151021	GSE6532	Breast Cancer	Breast	1.35E-03	2.03E-01	2.14E-02	3.17E-01
9368	GSM151022	GSE6532	Breast Cancer	Breast	5.83E-05	2.47E-01	9.68E-03	3.48E-01
9369	GSM151023	GSE6532	Breast Cancer	Breast	4.95E-04	-2.18E-01	2.45E-03	-3.96E-01
9370	GSM151024	GSE6532	Breast Cancer	Breast	1.57E-04	2.34E-01	8.79E-04	4.29E-01
9371	GSM151025	GSE6532	Breast Cancer	Breast	9.23E-09	-3.40E-01	6.98E-04	-4.36E-01
9372	GSM151026	GSE6532	Breast Cancer	Breast	1.16E-01	-1.16E-01	3.84E-01	-1.58E-01
9373	GSM151027	GSE6532	Breast Cancer	Breast	8.52E-04	-2.10E-01	2.25E-01	-1.97E-01
9374	GSM151028	GSE6532	Breast Cancer	Breast	1.60E-01	-1.07E-01	2.83E-01	1.82E-01
9375	GSM151029	GSE6532	Breast Cancer	Breast	3.15E-10	-3.70E-01	6.51E-03	-3.63E-01
9376	GSM151030	GSE6532	Breast Cancer	Breast	1.37E-06	-2.91E-01	8.66E-02	-2.53E-01
9377	GSM151031	GSE6532	Breast Cancer	Breast	1.65E-06	-2.89E-01	8.02E-03	-3.55E-01
9378	GSM151032	GSE6532	Breast Cancer	Breast	2.09E-19	5.19E-01	4.82E-12	8.25E-01
9379	GSM151033	GSE6532	Breast Cancer	Breast	4.28E-12	4.05E-01	3.02E-07	6.26E-01
9380	GSM151034	GSE6532	Breast Cancer	Breast	4.44E-07	3.03E-01	2.28E-04	4.68E-01
9381	GSM151035	GSE6532	Breast Cancer	Breast	5.79E-03	1.80E-01	9.90E-02	2.46E-01
9382	GSM151036	GSE6532	Breast Cancer	Breast	1.89E-05	2.61E-01	7.01E-05	5.00E-01
9383	GSM151037	GSE6532	Breast Cancer	Breast	2.24E-03	-1.95E-01	1.51E-01	2.22E-01
9384	GSM151038	GSE6532	Breast Cancer	Breast	1.01E-02	-1.70E-01	5.86E-01	1.18E-01
9385	GSM151039	GSE6532	Breast Cancer	Breast	2.35E-01	-9.52E-02	1.32E-01	2.30E-01
9386	GSM151040	GSE6532	Breast Cancer	Breast	6.08E-04	2.15E-01	1.73E-04	4.76E-01
9387	GSM151041	GSE6532	Breast Cancer	Breast	1.35E-04	2.36E-01	4.89E-03	3.73E-01
9388	GSM151042	GSE6532	Breast Cancer	Breast	7.96E-05	-2.43E-01	6.31E-02	-2.69E-01
9389	GSM151043	GSE6532	Breast Cancer	Breast	1.70E-02	1.60E-01	1.14E-01	2.38E-01
9390	GSM151044	GSE6532	Breast Cancer	Breast	1.34E-01	-1.12E-01	3.11E-01	1.75E-01
9391	GSM151045	GSE6532	Breast Cancer	Breast	1.89E-01	1.02E-01	7.27E-02	2.62E-01
9392	GSM151046	GSE6532	Breast Cancer	Breast	1.04E-03	-2.07E-01	1.01E-01	-2.44E-01
9393	GSM151047	GSE6532	Breast Cancer	Breast	1.31E-02	1.65E-01	1.83E-02	3.23E-01

9394	GSM151048	GSE6532	Breast Cancer	Breast	1.60E-01	-1.07E-01	1.84E-01	2.10E-01
9395	GSM151049	GSE6532	Breast Cancer	Breast	5.23E-02	-1.36E-01	3.63E-01	1.63E-01
9396	GSM151050	GSE6532	Breast Cancer	Breast	5.69E-02	-1.34E-01	3.81E-01	1.59E-01
9397	GSM151051	GSE6532	Breast Cancer	Breast	2.08E-01	-9.92E-02	3.14E-01	1.74E-01
9398	GSM151052	GSE6532	Breast Cancer	Breast	8.13E-06	-2.71E-01	1.02E-02	-3.46E-01
9399	GSM151053	GSE6532	Breast Cancer	Breast	6.84E-06	2.73E-01	2.45E-03	3.96E-01
9400	GSM151054	GSE6532	Breast Cancer	Breast	4.60E-03	1.84E-01	6.06E-03	3.65E-01
9401	GSM151055	GSE6532	Breast Cancer	Breast	7.45E-04	2.12E-01	6.83E-04	4.36E-01
9402	GSM151056	GSE6532	Breast Cancer	Breast	1.16E-04	-2.38E-01	5.31E-02	-2.77E-01
9403	GSM151057	GSE6532	Breast Cancer	Breast	1.25E-02	-1.66E-01	8.45E-02	2.54E-01
9404	GSM151058	GSE6532	Breast Cancer	Breast	7.01E-02	1.29E-01	1.15E-01	2.37E-01
9405	GSM151059	GSE6532	Breast Cancer	Breast	4.20E-02	1.41E-01	1.77E-02	3.24E-01
9406	GSM151060	GSE6532	Breast Cancer	Breast	4.79E-02	1.38E-01	3.20E-02	3.00E-01
9407	GSM151061	GSE6532	Breast Cancer	Breast	3.42E-03	-1.88E-01	4.70E-01	-1.40E-01
9408	GSM151062	GSE6532	Breast Cancer	Breast	4.79E-02	1.38E-01	6.23E-02	2.69E-01
9409	GSM151063	GSE6532	Breast Cancer	Breast	2.84E-05	2.56E-01	1.73E-04	4.76E-01
9410	GSM151064	GSE6532	Breast Cancer	Breast	1.69E-04	2.33E-01	5.40E-04	4.43E-01
9411	GSM151065	GSE6532	Breast Cancer	Breast	3.56E-01	8.04E-02	6.23E-02	2.69E-01
9412	GSM151066	GSE6532	Breast Cancer	Breast	4.49E-08	-3.25E-01	4.09E-02	-2.89E-01
9413	GSM151067	GSE6532	Breast Cancer	Breast	1.27E-36	-7.19E-01	8.09E-12	-8.17E-01
9414	GSM151068	GSE6532	Breast Cancer	Breast	1.98E-09	-3.54E-01	3.32E-03	-3.86E-01
9415	GSM65316	GSE6532	Breast Cancer	Breast	2.73E-07	-3.08E-01	1.83E-02	-3.23E-01
9416	GSM65317	GSE6532	Breast Cancer	Breast	1.55E-10	-3.76E-01	4.25E-04	-4.50E-01
9417	GSM65318	GSE6532	Breast Cancer	Breast	1.39E-01	1.11E-01	1.83E-02	3.23E-01
9418	GSM65319	GSE6532	Breast Cancer	Breast	2.35E-01	-9.52E-02	3.46E-01	1.66E-01
9419	GSM65320	GSE6532	Breast Cancer	Breast	2.91E-02	1.49E-01	1.04E-02	3.45E-01
9420	GSM65321	GSE6532	Breast Cancer	Breast	1.83E-01	1.03E-01	2.61E-02	3.09E-01
9421	GSM65322	GSE6532	Breast Cancer	Breast	2.49E-01	9.33E-02	2.04E-01	2.04E-01
9422	GSM65323	GSE6532	Breast Cancer	Breast	2.86E-03	-1.91E-01	2.25E-01	-1.97E-01
9423	GSM65324	GSE6532	Breast Cancer	Breast	2.47E-09	-3.52E-01	2.01E-03	-4.03E-01
9424	GSM65325	GSE6532	Breast Cancer	Breast	1.98E-09	-3.54E-01	3.30E-02	-2.98E-01
9425	GSM65326	GSE6532	Breast Cancer	Breast	2.01E-01	1.00E-01	9.90E-02	2.46E-01
9426	GSM65327	GSE6532	Breast Cancer	Breast	4.62E-04	-2.19E-01	8.66E-02	-2.53E-01
9427	GSM65328	GSE6532	Breast Cancer	Breast	9.11E-04	-2.09E-01	1.34E-01	-2.29E-01
9428	GSM65329	GSE6532	Breast Cancer	Breast	7.11E-10	3.63E-01	2.72E-05	5.24E-01
9429	GSM65330	GSE6532	Breast Cancer	Breast	7.45E-04	-2.12E-01	2.58E-01	-1.88E-01
9430	GSM65331	GSE6532	Breast Cancer	Breast	2.07E-02	-1.56E-01	3.24E-01	-1.71E-01
9431	GSM65332	GSE6532	Breast Cancer	Breast	5.69E-02	1.34E-01	4.45E-02	2.85E-01
9432	GSM65333	GSE6532	Breast Cancer	Breast	1.11E-03	-2.06E-01	2.61E-01	-1.87E-01
9433	GSM65334	GSE6532	Breast Cancer	Breast	1.60E-01	-1.07E-01	1.96E-01	2.06E-01
9434	GSM65335	GSE6532	Breast Cancer	Breast	7.92E-02	-1.26E-01	1.73E-01	2.14E-01
9435	GSM65336	GSE6532	Breast Cancer	Breast	4.39E-02	1.40E-01	7.61E-03	3.57E-01
9436	GSM65337	GSE6532	Breast Cancer	Breast	3.31E-12	-4.07E-01	2.49E-04	-4.65E-01
9437	GSM65338	GSE6532	Breast Cancer	Breast	2.44E-04	-2.28E-01	1.18E-01	-2.36E-01
9438	GSM65339	GSE6532	Breast Cancer	Breast	1.37E-06	2.91E-01	5.17E-04	4.44E-01
9439	GSM151259	GSE6532	Breast Cancer	Breast	2.88E-01	8.83E-02	1.04E-01	2.43E-01
9440	GSM151260	GSE6532	Breast Cancer	Breast	8.13E-06	-2.71E-01	1.18E-01	-2.36E-01
9441	GSM151261	GSE6532	Breast Cancer	Breast	3.19E-02	1.47E-01	1.77E-02	3.24E-01
9442	GSM151262	GSE6532	Breast Cancer	Breast	4.20E-02	1.41E-01	3.16E-02	3.00E-01
9443	GSM151263	GSE6532	Breast Cancer	Breast	2.49E-01	9.33E-02	1.96E-01	2.06E-01
9444	GSM151264	GSE6532	Breast Cancer	Breast	2.53E-03	1.93E-01	2.57E-02	3.09E-01
9445	GSM151265	GSE6532	Breast Cancer	Breast	1.60E-01	-1.07E-01	2.11E-02	3.17E-01
9446	GSM151266	GSE6532	Breast Cancer	Breast	6.97E-04	-2.13E-01	2.61E-01	-1.87E-01
9447	GSM151267	GSE6532	Breast Cancer	Breast	5.46E-02	1.35E-01	1.42E-02	3.33E-01
9448	GSM151268	GSE6532	Breast Cancer	Breast	3.54E-10	-3.69E-01	4.25E-04	-4.50E-01
9449	GSM151269	GSE6532	Breast Cancer	Breast	2.27E-04	2.29E-01	3.85E-03	3.81E-01
9450	GSM151270	GSE6532	Breast Cancer	Breast	2.94E-17	4.88E-01	3.68E-10	7.53E-01
9451	GSM151271	GSE6532	Breast Cancer	Breast	1.95E-01	-1.01E-01	4.61E-01	-1.42E-01
9452	GSM151272	GSE6532	Breast Cancer	Breast	4.62E-04	-2.19E-01	4.45E-02	-2.85E-01
9453	GSM151273	GSE6532	Breast Cancer	Breast	4.83E-06	2.77E-01	1.47E-03	4.13E-01
9454	GSM151274	GSE6532	Breast Cancer	Breast	3.19E-02	1.47E-01	9.90E-02	2.46E-01
9455	GSM151275	GSE6532	Breast Cancer	Breast	1.83E-01	1.03E-01	5.24E-02	2.77E-01
9456	GSM151276	GSE6532	Breast Cancer	Breast	6.48E-03	1.78E-01	6.06E-03	3.65E-01
9457	GSM151277	GSE6532	Breast Cancer	Breast	3.63E-03	-1.88E-01	2.02E-01	-2.04E-01
9458	GSM151278	GSE6532	Breast Cancer	Breast	2.24E-03	1.95E-01	1.90E-03	4.04E-01
9459	GSM151279	GSE6532	Breast Cancer	Breast	2.57E-01	9.23E-02	3.11E-02	3.01E-01
9460	GSM151280	GSE6532	Breast Cancer	Breast	1.44E-03	2.02E-01	7.88E-03	3.56E-01
9461	GSM151281	GSE6532	Breast Cancer	Breast	2.77E-02	-1.50E-01	3.46E-01	1.66E-01
9462	GSM151282	GSE6532	Breast Cancer	Breast	9.54E-03	-1.71E-01	2.29E-01	-1.96E-01
9463	GSM151283	GSE6532	Breast Cancer	Breast	2.42E-05	-2.58E-01	8.56E-02	-2.53E-01
9464	GSM151284	GSE6532	Breast Cancer	Breast	1.05E-05	-2.68E-01	3.16E-02	-3.00E-01
9465	GSM151285	GSE6532	Breast Cancer	Breast	7.25E-03	-1.76E-01	2.00E-01	-2.05E-01
9466	GSM151286	GSE6532	Breast Cancer	Breast	2.64E-02	-1.51E-01	5.46E-01	1.26E-01
9467	GSM151287	GSE6532	Breast Cancer	Breast	5.37E-07	3.01E-01	2.38E-04	4.67E-01

9468	GSM151288	GSE6532	Breast Cancer	Breast	9.26E-02	-1.22E-01	3.81E-01	1.59E-01
9469	GSM151289	GSE6532	Breast Cancer	Breast	8.52E-04	-2.10E-01	3.14E-01	1.74E-01
9470	GSM151290	GSE6532	Breast Cancer	Breast	4.25E-05	2.51E-01	1.53E-03	4.11E-01
9471	GSM151291	GSE6532	Breast Cancer	Breast	6.73E-02	1.30E-01	1.52E-01	2.22E-01
9472	GSM151292	GSE6532	Breast Cancer	Breast	2.29E-02	1.54E-01	5.24E-02	2.77E-01
9473	GSM151293	GSE6532	Breast Cancer	Breast	2.04E-07	-3.11E-01	4.98E-03	-3.72E-01
9474	GSM151294	GSE6532	Breast Cancer	Breast	4.20E-02	1.41E-01	3.81E-02	2.92E-01
9475	GSM151295	GSE6532	Breast Cancer	Breast	1.44E-01	1.10E-01	2.11E-02	3.17E-01
9476	GSM151296	GSE6532	Breast Cancer	Breast	4.47E-10	-3.67E-01	4.00E-03	-3.80E-01
9477	GSM151297	GSE6532	Breast Cancer	Breast	1.11E-03	2.06E-01	1.17E-03	4.20E-01
9478	GSM151298	GSE6532	Breast Cancer	Breast	4.79E-02	1.38E-01	4.00E-03	3.80E-01
9479	GSM151299	GSE6532	Breast Cancer	Breast	4.34E-03	1.85E-01	5.25E-03	3.70E-01
9480	GSM151300	GSE6532	Breast Cancer	Breast	1.25E-01	1.14E-01	3.25E-03	3.87E-01
9481	GSM151301	GSE6532	Breast Cancer	Breast	1.30E-01	1.13E-01	4.45E-02	2.85E-01
9482	GSM151302	GSE6532	Breast Cancer	Breast	1.00E-04	2.40E-01	5.49E-05	5.06E-01
9483	GSM151303	GSE6532	Breast Cancer	Breast	3.21E-01	-8.43E-02	4.61E-01	1.42E-01
9484	GSM151304	GSE6532	Breast Cancer	Breast	1.18E-02	1.67E-01	2.17E-02	3.16E-01
9485	GSM151305	GSE6532	Breast Cancer	Breast	4.25E-05	-2.51E-01	8.56E-02	-2.53E-01
9486	GSM151306	GSE6532	Breast Cancer	Breast	1.79E-02	-1.59E-01	5.93E-01	-1.17E-01
9487	GSM151307	GSE6532	Breast Cancer	Breast	1.05E-05	2.68E-01	4.07E-04	4.51E-01
9488	GSM151308	GSE6532	Breast Cancer	Breast	2.82E-04	-2.26E-01	2.25E-01	-1.97E-01
9489	GSM151309	GSE6532	Breast Cancer	Breast	5.01E-02	1.37E-01	5.16E-03	3.71E-01
9490	GSM151310	GSE6532	Breast Cancer	Breast	2.57E-01	-9.23E-02	6.31E-02	2.69E-01
9491	GSM151311	GSE6532	Breast Cancer	Breast	1.25E-01	1.14E-01	1.46E-02	3.32E-01
9492	GSM151312	GSE6532	Breast Cancer	Breast	3.30E-01	8.33E-02	4.45E-02	2.85E-01
9493	GSM151313	GSE6532	Breast Cancer	Breast	4.23E-01	7.34E-02	4.38E-02	2.86E-01
9494	GSM151314	GSE6532	Breast Cancer	Breast	1.88E-02	-1.58E-01	1.21E-01	-2.35E-01
9495	GSM151315	GSE6532	Breast Cancer	Breast	3.04E-03	-1.90E-01	4.61E-01	-1.42E-01
9496	GSM151316	GSE6532	Breast Cancer	Breast	5.64E-10	-3.65E-01	4.25E-04	-4.50E-01
9497	GSM151317	GSE6532	Breast Cancer	Breast	1.05E-05	-2.68E-01	1.35E-01	-2.29E-01
9498	GSM151318	GSE6532	Breast Cancer	Breast	1.26E-04	-2.37E-01	7.46E-02	-2.60E-01
9499	GSM151319	GSE6532	Breast Cancer	Breast	3.05E-02	-1.48E-01	3.11E-01	1.75E-01
9500	GSM151320	GSE6532	Breast Cancer	Breast	3.86E-09	-3.48E-01	2.49E-03	-3.96E-01
9501	GSM151321	GSE6532	Breast Cancer	Breast	7.60E-02	-1.27E-01	2.54E-01	1.89E-01
9502	GSM151322	GSE6532	Breast Cancer	Breast	1.60E-01	-1.07E-01	5.24E-02	2.77E-01
9503	GSM151323	GSE6532	Breast Cancer	Breast	1.48E-15	-4.62E-01	5.40E-04	-4.43E-01
9504	GSM151324	GSE6532	Breast Cancer	Breast	6.97E-04	2.13E-01	7.75E-03	3.56E-01
9505	GSM151325	GSE6532	Breast Cancer	Breast	3.50E-02	-1.45E-01	2.22E-01	1.98E-01
9506	GSM151326	GSE6532	Breast Cancer	Breast	2.21E-10	-3.73E-01	7.13E-04	-4.35E-01
9507	GSM151327	GSE6532	Breast Cancer	Breast	6.12E-08	-3.22E-01	7.88E-03	-3.56E-01
9508	GSM151328	GSE6532	Breast Cancer	Breast	2.23E-05	-2.59E-01	1.00E-02	-3.47E-01
9509	GSM151329	GSE6532	Breast Cancer	Breast	4.33E-01	-7.24E-02	2.80E-01	1.82E-01
9510	GSM151330	GSE6532	Breast Cancer	Breast	3.86E-03	1.87E-01	2.33E-04	4.67E-01
9511	GSM151331	GSE6532	Breast Cancer	Breast	1.60E-01	1.07E-01	1.32E-01	2.30E-01
9512	GSM151332	GSE6532	Breast Cancer	Breast	7.25E-03	-1.76E-01	3.46E-01	1.66E-01
9513	GSM151333	GSE6532	Breast Cancer	Breast	1.46E-02	1.63E-01	2.40E-03	3.97E-01
9514	GSM151334	GSE6532	Breast Cancer	Breast	3.54E-10	-3.69E-01	3.12E-04	-4.59E-01
9515	GSM151335	GSE6532	Breast Cancer	Breast	4.88E-07	-3.02E-01	2.24E-02	-3.15E-01
9516	GSM151336	GSE6532	Breast Cancer	Breast	7.13E-12	-4.01E-01	3.19E-04	-4.58E-01
9517	GSM151337	GSE6532	Breast Cancer	Breast	3.93E-05	-2.52E-01	1.00E-01	-2.45E-01
9518	GSM151338	GSE6532	Breast Cancer	Breast	1.12E-02	-1.68E-01	6.15E-02	2.70E-01
9519	GSM151339	GSE6532	Breast Cancer	Breast	1.47E-05	-2.64E-01	4.63E-02	-2.83E-01
9520	GSM151340	GSE6532	Breast Cancer	Breast	9.26E-02	-1.22E-01	5.03E-01	1.34E-01
9521	GSM151341	GSE6532	Breast Cancer	Breast	1.08E-04	2.39E-01	8.98E-04	4.28E-01
9522	GSM151342	GSE6532	Breast Cancer	Breast	2.06E-13	-4.28E-01	2.16E-05	-5.30E-01
9523	GSM151343	GSE6532	Breast Cancer	Breast	5.83E-05	-2.47E-01	6.31E-02	-2.69E-01
9524	GSM151344	GSE6532	Breast Cancer	Breast	2.40E-02	-1.53E-01	3.46E-01	1.66E-01
9525	GSM151345	GSE6532	Breast Cancer	Breast	2.77E-02	-1.50E-01	2.00E-01	2.05E-01
9526	GSM177885	GSE7390	Breast Cancer	Breast	6.32E-01	5.36E-02	2.00E-01	2.05E-01
9527	GSM177886	GSE7390	Breast Cancer	Breast	1.85E-03	-1.98E-01	3.14E-01	-1.74E-01
9528	GSM177887	GSE7390	Breast Cancer	Breast	7.84E-07	2.97E-01	7.53E-06	5.55E-01
9529	GSM177888	GSE7390	Breast Cancer	Breast	3.47E-01	-8.13E-02	3.84E-01	1.58E-01
9530	GSM177889	GSE7390	Breast Cancer	Breast	6.08E-04	-2.15E-01	6.39E-03	-3.63E-01
9531	GSM177890	GSE7390	Breast Cancer	Breast	4.01E-02	1.42E-01	4.38E-02	2.86E-01
9532	GSM177891	GSE7390	Breast Cancer	Breast	1.34E-01	1.12E-01	9.90E-02	2.46E-01
9533	GSM177892	GSE7390	Breast Cancer	Breast	1.14E-06	2.93E-01	7.19E-05	4.99E-01
9534	GSM177893	GSE7390	Breast Cancer	Breast	1.85E-17	-4.91E-01	1.37E-06	-5.94E-01
9535	GSM177894	GSE7390	Breast Cancer	Breast	5.47E-03	1.81E-01	1.53E-03	4.11E-01
9536	GSM177895	GSE7390	Breast Cancer	Breast	2.14E-01	-9.82E-02	9.90E-02	2.46E-01
9537	GSM177896	GSE7390	Breast Cancer	Breast	1.04E-01	1.19E-01	9.90E-02	2.46E-01
9538	GSM177897	GSE7390	Breast Cancer	Breast	6.13E-03	1.79E-01	1.81E-04	4.74E-01
9539	GSM177898	GSE7390	Breast Cancer	Breast	3.04E-01	-8.63E-02	9.90E-02	2.46E-01
9540	GSM177899	GSE7390	Breast Cancer	Breast	1.34E-01	1.12E-01	1.51E-01	2.22E-01
9541	GSM177900	GSE7390	Breast Cancer	Breast	1.79E-02	-1.59E-01	1.01E-01	-2.44E-01

9542	GSM177901	GSE7390	Breast Cancer	Breast	7.30E-02	1.28E-01	1.34E-01	2.29E-01
9543	GSM177902	GSE7390	Breast Cancer	Breast	4.49E-08	-3.25E-01	4.15E-03	-3.78E-01
9544	GSM177903	GSE7390	Breast Cancer	Breast	6.73E-02	1.30E-01	1.77E-02	3.24E-01
9545	GSM177904	GSE7390	Breast Cancer	Breast	7.60E-02	-1.27E-01	2.49E-01	1.90E-01
9546	GSM177905	GSE7390	Breast Cancer	Breast	1.12E-01	-1.17E-01	7.46E-02	2.60E-01
9547	GSM177906	GSE7390	Breast Cancer	Breast	3.27E-18	-5.02E-01	9.66E-07	-6.01E-01
9548	GSM177907	GSE7390	Breast Cancer	Breast	9.26E-02	1.22E-01	4.45E-02	2.85E-01
9549	GSM177908	GSE7390	Breast Cancer	Breast	4.60E-03	-1.84E-01	1.96E-01	2.06E-01
9550	GSM177909	GSE7390	Breast Cancer	Breast	7.67E-03	1.75E-01	8.02E-03	3.55E-01
9551	GSM177910	GSE7390	Breast Cancer	Breast	6.46E-02	-1.31E-01	4.26E-01	-1.49E-01
9552	GSM177911	GSE7390	Breast Cancer	Breast	5.30E-04	2.17E-01	1.53E-03	4.11E-01
9553	GSM177912	GSE7390	Breast Cancer	Breast	2.64E-01	-9.13E-02	4.70E-01	1.40E-01
9554	GSM177913	GSE7390	Breast Cancer	Breast	1.71E-01	1.05E-01	8.45E-02	2.54E-01
9555	GSM177914	GSE7390	Breast Cancer	Breast	5.01E-02	1.37E-01	3.25E-02	2.99E-01
9556	GSM177915	GSE7390	Breast Cancer	Breast	1.95E-01	1.01E-01	2.22E-01	1.98E-01
9557	GSM177916	GSE7390	Breast Cancer	Breast	9.54E-03	-1.71E-01	3.49E-01	-1.66E-01
9558	GSM177917	GSE7390	Breast Cancer	Breast	3.45E-09	-3.49E-01	1.18E-02	-3.40E-01
9559	GSM177918	GSE7390	Breast Cancer	Breast	8.57E-02	-1.24E-01	2.54E-01	1.89E-01
9560	GSM177919	GSE7390	Breast Cancer	Breast	1.96E-14	-4.44E-01	7.53E-06	-5.55E-01
9561	GSM177920	GSE7390	Breast Cancer	Breast	4.60E-03	-1.84E-01	3.14E-01	1.74E-01
9562	GSM177921	GSE7390	Breast Cancer	Breast	1.60E-01	-1.07E-01	5.00E-01	1.35E-01
9563	GSM177922	GSE7390	Breast Cancer	Breast	1.74E-05	-2.62E-01	1.15E-01	-2.37E-01
9564	GSM177923	GSE7390	Breast Cancer	Breast	3.42E-03	1.88E-01	2.01E-03	4.03E-01
9565	GSM177924	GSE7390	Breast Cancer	Breast	6.86E-03	-1.77E-01	4.23E-01	1.50E-01
9566	GSM177925	GSE7390	Breast Cancer	Breast	4.03E-07	3.04E-01	1.10E-05	5.46E-01
9567	GSM177926	GSE7390	Breast Cancer	Breast	1.02E-07	-3.17E-01	3.40E-02	-2.97E-01
9568	GSM177927	GSE7390	Breast Cancer	Breast	3.67E-02	-1.44E-01	1.14E-01	2.38E-01
9569	GSM177928	GSE7390	Breast Cancer	Breast	3.42E-03	-1.88E-01	4.39E-01	-1.47E-01
9570	GSM177929	GSE7390	Breast Cancer	Breast	1.11E-03	-2.06E-01	1.82E-01	-2.11E-01
9571	GSM177930	GSE7390	Breast Cancer	Breast	1.54E-01	1.08E-01	1.98E-01	2.06E-01
9572	GSM177931	GSE7390	Breast Cancer	Breast	6.51E-04	-2.14E-01	2.95E-01	-1.78E-01
9573	GSM177932	GSE7390	Breast Cancer	Breast	3.34E-05	-2.54E-01	1.80E-02	-3.24E-01
9574	GSM177933	GSE7390	Breast Cancer	Breast	1.05E-05	-2.68E-01	1.22E-02	-3.39E-01
9575	GSM177934	GSE7390	Breast Cancer	Breast	1.22E-10	-3.78E-01	1.59E-03	-4.10E-01
9576	GSM177935	GSE7390	Breast Cancer	Breast	1.44E-03	2.02E-01	3.70E-02	2.93E-01
9577	GSM177936	GSE7390	Breast Cancer	Breast	1.54E-01	-1.08E-01	1.17E-01	2.37E-01
9578	GSM177937	GSE7390	Breast Cancer	Breast	2.69E-03	1.92E-01	1.18E-02	3.40E-01
9579	GSM177938	GSE7390	Breast Cancer	Breast	8.57E-02	-1.24E-01	3.16E-01	-1.73E-01
9580	GSM177939	GSE7390	Breast Cancer	Breast	2.21E-01	-9.72E-02	1.15E-01	2.37E-01
9581	GSM177940	GSE7390	Breast Cancer	Breast	4.42E-06	-2.78E-01	3.13E-03	-3.88E-01
9582	GSM177941	GSE7390	Breast Cancer	Breast	6.28E-12	-4.02E-01	1.77E-04	-4.75E-01
9583	GSM177942	GSE7390	Breast Cancer	Breast	1.63E-03	-2.00E-01	2.02E-01	-2.04E-01
9584	GSM177943	GSE7390	Breast Cancer	Breast	4.23E-01	7.34E-02	1.51E-01	2.22E-01
9585	GSM177944	GSE7390	Breast Cancer	Breast	7.27E-15	4.51E-01	8.49E-09	6.97E-01
9586	GSM177945	GSE7390	Breast Cancer	Breast	5.01E-02	-1.37E-01	1.00E-01	-2.45E-01
9587	GSM177946	GSE7390	Breast Cancer	Breast	3.66E-07	-3.05E-01	6.28E-03	-3.64E-01
9588	GSM177947	GSE7390	Breast Cancer	Breast	9.26E-02	1.22E-01	8.56E-02	2.53E-01
9589	GSM177948	GSE7390	Breast Cancer	Breast	2.96E-01	-8.73E-02	3.11E-01	1.75E-01
9590	GSM177949	GSE7390	Breast Cancer	Breast	2.52E-02	-1.52E-01	3.81E-01	1.59E-01
9591	GSM177950	GSE7390	Breast Cancer	Breast	5.91E-07	-3.00E-01	6.48E-02	-2.67E-01
9592	GSM177951	GSE7390	Breast Cancer	Breast	6.46E-02	1.31E-01	4.00E-03	3.80E-01
9593	GSM177952	GSE7390	Breast Cancer	Breast	2.69E-03	1.92E-01	1.22E-02	3.39E-01
9594	GSM177953	GSE7390	Breast Cancer	Breast	1.53E-03	-2.01E-01	8.66E-02	-2.53E-01
9595	GSM177954	GSE7390	Breast Cancer	Breast	4.01E-02	1.42E-01	2.14E-02	3.17E-01
9596	GSM177955	GSE7390	Breast Cancer	Breast	1.18E-02	-1.67E-01	4.23E-01	-1.50E-01
9597	GSM177956	GSE7390	Breast Cancer	Breast	2.36E-13	4.27E-01	8.99E-08	6.51E-01
9598	GSM177957	GSE7390	Breast Cancer	Breast	5.46E-02	1.35E-01	1.51E-01	2.22E-01
9599	GSM177958	GSE7390	Breast Cancer	Breast	1.79E-02	1.59E-01	6.23E-02	2.69E-01
9600	GSM177959	GSE7390	Breast Cancer	Breast	1.34E-01	1.12E-01	4.38E-02	2.86E-01
9601	GSM177960	GSE7390	Breast Cancer	Breast	2.01E-01	1.00E-01	1.98E-01	2.06E-01
9602	GSM177961	GSE7390	Breast Cancer	Breast	4.73E-01	6.85E-02	7.27E-02	2.62E-01
9603	GSM177962	GSE7390	Breast Cancer	Breast	2.84E-05	-2.56E-01	1.03E-01	-2.44E-01
9604	GSM177963	GSE7390	Breast Cancer	Breast	1.16E-01	1.16E-01	1.44E-02	3.33E-01
9605	GSM177964	GSE7390	Breast Cancer	Breast	1.95E-01	-1.01E-01	3.16E-01	1.73E-01
9606	GSM177965	GSE7390	Breast Cancer	Breast	1.12E-01	-1.17E-01	4.20E-01	1.50E-01
9607	GSM177966	GSE7390	Breast Cancer	Breast	9.73E-04	2.08E-01	7.75E-03	3.56E-01
9608	GSM177967	GSE7390	Breast Cancer	Breast	1.44E-03	-2.02E-01	3.61E-01	-1.63E-01
9609	GSM177968	GSE7390	Breast Cancer	Breast	9.63E-02	-1.21E-01	3.81E-01	1.59E-01
9610	GSM177969	GSE7390	Breast Cancer	Breast	3.23E-03	-1.89E-01	3.11E-01	-1.75E-01
9611	GSM177970	GSE7390	Breast Cancer	Breast	3.13E-01	-8.53E-02	6.10E-01	1.14E-01
9612	GSM177971	GSE7390	Breast Cancer	Breast	2.57E-01	-9.23E-02	2.22E-01	1.98E-01
9613	GSM177972	GSE7390	Breast Cancer	Breast	5.23E-02	-1.36E-01	3.16E-01	-1.73E-01
9614	GSM177973	GSE7390	Breast Cancer	Breast	9.23E-09	-3.40E-01	5.52E-04	-4.43E-01
9615	GSM177974	GSE7390	Breast Cancer	Breast	6.86E-03	-1.77E-01	1.54E-01	-2.21E-01

9616	GSM177975	GSE7390	Breast Cancer	Breast	2.14E-01	9.82E-02	6.23E-02	2.69E-01
9617	GSM177976	GSE7390	Breast Cancer	Breast	2.08E-01	-9.92E-02	3.22E-01	1.72E-01
9618	GSM177977	GSE7390	Breast Cancer	Breast	1.18E-02	-1.67E-01	2.83E-01	-1.82E-01
9619	GSM177978	GSE7390	Breast Cancer	Breast	1.34E-01	1.12E-01	9.21E-02	2.50E-01
9620	GSM177979	GSE7390	Breast Cancer	Breast	5.25E-01	6.35E-02	1.52E-01	2.22E-01
9621	GSM177980	GSE7390	Breast Cancer	Breast	1.97E-02	-1.57E-01	6.23E-02	2.69E-01
9622	GSM177981	GSE7390	Breast Cancer	Breast	1.14E-06	-2.93E-01	1.22E-02	-3.39E-01
9623	GSM177982	GSE7390	Breast Cancer	Breast	6.51E-04	-2.14E-01	2.25E-01	-1.97E-01
9624	GSM177983	GSE7390	Breast Cancer	Breast	2.86E-03	-1.91E-01	1.01E-01	-2.44E-01
9625	GSM177984	GSE7390	Breast Cancer	Breast	4.02E-04	-2.21E-01	2.51E-01	-1.90E-01
9626	GSM177985	GSE7390	Breast Cancer	Breast	3.04E-03	1.90E-01	1.46E-02	3.32E-01
9627	GSM177986	GSE7390	Breast Cancer	Breast	2.28E-01	9.62E-02	2.57E-02	3.09E-01
9628	GSM177987	GSE7390	Breast Cancer	Breast	2.08E-01	-9.92E-02	3.84E-01	1.58E-01
9629	GSM177988	GSE7390	Breast Cancer	Breast	9.59E-11	3.80E-01	3.21E-07	6.25E-01
9630	GSM177989	GSE7390	Breast Cancer	Breast	2.64E-01	-9.13E-02	4.23E-01	1.50E-01
9631	GSM177990	GSE7390	Breast Cancer	Breast	2.42E-01	-9.42E-02	4.20E-01	1.50E-01
9632	GSM177991	GSE7390	Breast Cancer	Breast	5.46E-01	6.15E-02	6.56E-02	2.67E-01
9633	GSM177992	GSE7390	Breast Cancer	Breast	6.86E-03	-1.77E-01	2.54E-01	-1.89E-01
9634	GSM177993	GSE7390	Breast Cancer	Breast	1.79E-02	1.59E-01	9.52E-03	3.49E-01
9635	GSM177994	GSE7390	Breast Cancer	Breast	1.85E-03	1.98E-01	2.61E-02	3.09E-01
9636	GSM177995	GSE7390	Breast Cancer	Breast	4.01E-17	-4.86E-01	6.40E-07	-6.10E-01
9637	GSM177996	GSE7390	Breast Cancer	Breast	3.38E-01	8.23E-02	1.15E-01	2.37E-01
9638	GSM177997	GSE7390	Breast Cancer	Breast	1.60E-01	1.07E-01	1.75E-02	3.25E-01
9639	GSM177998	GSE7390	Breast Cancer	Breast	1.39E-01	1.11E-01	3.11E-02	3.01E-01
9640	GSM177999	GSE7390	Breast Cancer	Breast	6.81E-05	-2.45E-01	8.77E-02	-2.52E-01
9641	GSM178000	GSE7390	Breast Cancer	Breast	5.01E-02	1.37E-01	6.15E-02	2.70E-01
9642	GSM178001	GSE7390	Breast Cancer	Breast	3.66E-01	-7.94E-02	8.56E-02	2.53E-01
9643	GSM178002	GSE7390	Breast Cancer	Breast	1.34E-01	1.12E-01	4.38E-02	2.86E-01
9644	GSM178003	GSE7390	Breast Cancer	Breast	4.59E-02	-1.39E-01	6.23E-02	2.69E-01
9645	GSM178004	GSE7390	Breast Cancer	Breast	2.42E-01	9.42E-02	8.66E-02	2.53E-01
9646	GSM178005	GSE7390	Breast Cancer	Breast	7.92E-02	1.26E-01	3.86E-02	2.91E-01
9647	GSM178006	GSE7390	Breast Cancer	Breast	1.54E-01	-1.08E-01	3.81E-01	1.59E-01
9648	GSM178007	GSE7390	Breast Cancer	Breast	4.49E-08	3.25E-01	1.83E-06	5.87E-01
9649	GSM178008	GSE7390	Breast Cancer	Breast	2.29E-02	-1.54E-01	3.16E-01	-1.73E-01
9650	GSM178009	GSE7390	Breast Cancer	Breast	1.16E-04	2.38E-01	8.98E-04	4.28E-01
9651	GSM178010	GSE7390	Breast Cancer	Breast	3.23E-03	-1.89E-01	3.61E-01	-1.63E-01
9652	GSM178011	GSE7390	Breast Cancer	Breast	6.97E-04	2.13E-01	1.94E-03	4.04E-01
9653	GSM178012	GSE7390	Breast Cancer	Breast	1.79E-02	-1.59E-01	2.49E-01	1.90E-01
9654	GSM178013	GSE7390	Breast Cancer	Breast	2.57E-01	9.23E-02	3.70E-02	2.93E-01
9655	GSM178014	GSE7390	Breast Cancer	Breast	1.18E-02	-1.67E-01	1.51E-01	2.22E-01
9656	GSM178015	GSE7390	Breast Cancer	Breast	1.95E-01	1.01E-01	2.51E-01	1.90E-01
9657	GSM178016	GSE7390	Breast Cancer	Breast	1.12E-02	1.68E-01	3.85E-03	3.81E-01
9658	GSM178017	GSE7390	Breast Cancer	Breast	9.08E-14	-4.34E-01	2.44E-04	-4.66E-01
9659	GSM178018	GSE7390	Breast Cancer	Breast	2.76E-09	-3.51E-01	2.38E-04	-4.67E-01
9660	GSM178019	GSE7390	Breast Cancer	Breast	1.25E-02	-1.66E-01	2.51E-01	-1.90E-01
9661	GSM178020	GSE7390	Breast Cancer	Breast	3.34E-02	-1.46E-01	4.20E-01	1.50E-01
9662	GSM178021	GSE7390	Breast Cancer	Breast	3.56E-01	-8.04E-02	4.58E-01	1.43E-01
9663	GSM178022	GSE7390	Breast Cancer	Breast	1.39E-01	-1.11E-01	2.54E-01	1.89E-01
9664	GSM178023	GSE7390	Breast Cancer	Breast	5.69E-02	-1.34E-01	3.72E-01	1.61E-01
9665	GSM178024	GSE7390	Breast Cancer	Breast	1.24E-05	-2.66E-01	1.04E-01	-2.43E-01
9666	GSM178025	GSE7390	Breast Cancer	Breast	1.26E-03	-2.04E-01	4.45E-02	-2.85E-01
9667	GSM178026	GSE7390	Breast Cancer	Breast	2.62E-04	-2.27E-01	1.19E-01	-2.36E-01
9668	GSM178027	GSE7390	Breast Cancer	Breast	4.98E-05	2.49E-01	7.01E-05	5.00E-01
9669	GSM178028	GSE7390	Breast Cancer	Breast	1.01E-02	-1.70E-01	3.84E-01	1.58E-01
9670	GSM178029	GSE7390	Breast Cancer	Breast	1.95E-01	1.01E-01	6.48E-02	2.67E-01
9671	GSM178030	GSE7390	Breast Cancer	Breast	2.42E-01	-9.42E-02	1.32E-01	2.30E-01
9672	GSM178031	GSE7390	Breast Cancer	Breast	1.98E-09	-3.54E-01	1.94E-03	-4.04E-01
9673	GSM178032	GSE7390	Breast Cancer	Breast	5.69E-02	1.34E-01	4.38E-02	2.86E-01
9674	GSM178033	GSE7390	Breast Cancer	Breast	6.21E-01	5.46E-02	1.96E-01	2.06E-01
9675	GSM178034	GSE7390	Breast Cancer	Breast	5.16E-03	1.82E-01	3.08E-03	3.89E-01
9676	GSM178035	GSE7390	Breast Cancer	Breast	1.89E-01	-1.02E-01	1.73E-01	2.14E-01
9677	GSM178036	GSE7390	Breast Cancer	Breast	1.95E-01	1.01E-01	1.52E-01	2.22E-01
9678	GSM178037	GSE7390	Breast Cancer	Breast	3.49E-04	-2.23E-01	1.34E-01	-2.29E-01
9679	GSM178038	GSE7390	Breast Cancer	Breast	2.06E-13	-4.28E-01	5.23E-05	-5.07E-01
9680	GSM178039	GSE7390	Breast Cancer	Breast	3.21E-01	8.43E-02	2.02E-01	2.04E-01
9681	GSM178040	GSE7390	Breast Cancer	Breast	1.88E-02	-1.58E-01	1.98E-01	-2.06E-01
9682	GSM178041	GSE7390	Breast Cancer	Breast	1.35E-03	2.03E-01	8.98E-04	4.28E-01
9683	GSM178042	GSE7390	Breast Cancer	Breast	6.19E-02	-1.32E-01	1.14E-01	2.38E-01
9684	GSM178043	GSE7390	Breast Cancer	Breast	1.65E-01	1.06E-01	4.38E-02	2.86E-01
9685	GSM178044	GSE7390	Breast Cancer	Breast	7.92E-02	-1.26E-01	2.80E-01	1.82E-01
9686	GSM178045	GSE7390	Breast Cancer	Breast	3.71E-06	2.80E-01	9.60E-05	4.91E-01
9687	GSM178046	GSE7390	Breast Cancer	Breast	9.26E-02	1.22E-01	4.38E-02	2.86E-01
9688	GSM178047	GSE7390	Breast Cancer	Breast	1.49E-01	-1.09E-01	2.56E-01	-1.89E-01
9689	GSM178048	GSE7390	Breast Cancer	Breast	1.22E-10	-3.78E-01	1.77E-04	-4.75E-01

9690	GSM178049	GSE7390	Breast Cancer	Breast	7.36E-05	-2.44E-01	1.46E-02	-3.32E-01
9691	GSM178050	GSE7390	Breast Cancer	Breast	2.76E-09	-3.51E-01	4.16E-04	-4.51E-01
9692	GSM178051	GSE7390	Breast Cancer	Breast	1.97E-02	1.57E-01	5.31E-02	2.77E-01
9693	GSM178052	GSE7390	Breast Cancer	Breast	2.29E-15	-4.59E-01	1.12E-05	-5.45E-01
9694	GSM178053	GSE7390	Breast Cancer	Breast	1.25E-06	-2.92E-01	2.27E-02	-3.14E-01
9695	GSM178054	GSE7390	Breast Cancer	Breast	1.70E-02	1.60E-01	2.49E-03	3.96E-01
9696	GSM178055	GSE7390	Breast Cancer	Breast	2.03E-24	5.84E-01	2.40E-14	9.05E-01
9697	GSM178056	GSE7390	Breast Cancer	Breast	3.47E-01	8.13E-02	3.11E-01	1.75E-01
9698	GSM178057	GSE7390	Breast Cancer	Breast	1.46E-04	-2.35E-01	4.76E-02	-2.82E-01
9699	GSM178058	GSE7390	Breast Cancer	Breast	7.01E-02	-1.29E-01	3.11E-01	1.75E-01
9700	GSM178059	GSE7390	Breast Cancer	Breast	2.27E-04	2.29E-01	3.05E-04	4.60E-01
9701	GSM178060	GSE7390	Breast Cancer	Breast	3.23E-03	-1.89E-01	2.10E-01	-2.02E-01
9702	GSM178061	GSE7390	Breast Cancer	Breast	3.67E-02	-1.44E-01	8.45E-02	2.54E-01
9703	GSM178062	GSE7390	Breast Cancer	Breast	6.73E-02	1.30E-01	3.76E-02	2.93E-01
9704	GSM178063	GSE7390	Breast Cancer	Breast	2.21E-01	9.72E-02	5.31E-02	2.77E-01
9705	GSM178064	GSE7390	Breast Cancer	Breast	1.52E-07	-3.13E-01	1.86E-02	-3.23E-01
9706	GSM178065	GSE7390	Breast Cancer	Breast	1.65E-01	-1.06E-01	5.24E-02	2.77E-01
9707	GSM178066	GSE7390	Breast Cancer	Breast	1.11E-03	-2.06E-01	2.00E-01	-2.05E-01
9708	GSM178067	GSE7390	Breast Cancer	Breast	3.34E-02	1.46E-01	2.61E-02	3.09E-01
9709	GSM178068	GSE7390	Breast Cancer	Breast	7.67E-03	-1.75E-01	2.33E-01	-1.95E-01
9710	GSM178069	GSE7390	Breast Cancer	Breast	1.70E-02	-1.60E-01	1.82E-01	2.11E-01
9711	GSM178070	GSE7390	Breast Cancer	Breast	1.97E-10	-3.74E-01	5.28E-04	-4.44E-01
9712	GSM178071	GSE7390	Breast Cancer	Breast	2.40E-02	-1.53E-01	1.51E-01	2.22E-01
9713	GSM178072	GSE7390	Breast Cancer	Breast	1.16E-01	1.16E-01	6.15E-02	2.70E-01
9714	GSM178073	GSE7390	Breast Cancer	Breast	1.60E-01	1.07E-01	7.74E-02	2.58E-01
9715	GSM178074	GSE7390	Breast Cancer	Breast	2.40E-02	-1.53E-01	2.90E-01	-1.80E-01
9716	GSM178075	GSE7390	Breast Cancer	Breast	3.04E-01	8.63E-02	7.46E-02	2.60E-01
9717	GSM178076	GSE7390	Breast Cancer	Breast	3.86E-09	3.48E-01	5.29E-06	5.63E-01
9718	GSM178077	GSE7390	Breast Cancer	Breast	2.64E-02	1.51E-01	8.45E-02	2.54E-01
9719	GSM178078	GSE7390	Breast Cancer	Breast	3.84E-02	-1.43E-01	2.61E-01	-1.87E-01
9720	GSM178079	GSE7390	Breast Cancer	Breast	9.11E-04	-2.09E-01	3.61E-01	-1.63E-01
9721	GSM178080	GSE7390	Breast Cancer	Breast	1.25E-01	-1.14E-01	1.73E-01	2.14E-01
9722	GSM178081	GSE7390	Breast Cancer	Breast	3.21E-01	8.43E-02	7.36E-02	2.61E-01
9723	GSM178082	GSE7390	Breast Cancer	Breast	1.24E-07	3.15E-01	3.78E-05	5.16E-01
9724	19893_AB0177847(	HESS	Breast Cancer	Breast	4.59E-02	-1.39E-01	3.81E-01	1.59E-01
9725	19893_AB0177850	HESS	Breast Cancer	Breast	5.75E-06	-2.75E-01	1.17E-03	-4.20E-01
9726	19893_AB0177851(	HESS	Breast Cancer	Breast	5.37E-07	-3.01E-01	4.22E-03	-3.78E-01
9727	19893_AB0177918	HESS	Breast Cancer	Breast	4.01E-02	-1.42E-01	2.49E-01	1.90E-01
9728	19893_AB0177918	HESS	Breast Cancer	Breast	8.13E-06	-2.71E-01	2.04E-01	-2.04E-01
9729	19893_AB0186019	HESS	Breast Cancer	Breast	4.98E-05	2.49E-01	9.56E-04	4.26E-01
9730	19893_AB0186031	HESS	Breast Cancer	Breast	2.62E-04	-2.27E-01	4.45E-02	-2.85E-01
9731	19893_AB0191318	HESS	Breast Cancer	Breast	1.53E-03	-2.01E-01	1.80E-01	-2.11E-01
9732	19893_AB0191326	HESS	Breast Cancer	Breast	2.57E-01	9.23E-02	2.61E-02	3.09E-01
9733	19893_AB0191330(	HESS	Breast Cancer	Breast	8.10E-03	-1.74E-01	1.35E-01	-2.29E-01
9734	19893_AB0192309(	HESS	Breast Cancer	Breast	3.71E-06	2.80E-01	2.87E-05	5.23E-01
9735	19893_AB0194385	HESS	Breast Cancer	Breast	9.54E-03	-1.71E-01	2.49E-01	1.90E-01
9736	19893_AB0198330	HESS	Breast Cancer	Breast	3.05E-02	-1.48E-01	1.14E-01	2.38E-01
9737	19893_AB0198344	HESS	Breast Cancer	Breast	2.64E-02	-1.51E-01	2.80E-01	1.82E-01
9738	19893_AB0198344	HESS	Breast Cancer	Breast	4.62E-04	-2.19E-01	2.51E-01	-1.90E-01
9739	19893_AB0198347	HESS	Breast Cancer	Breast	3.86E-03	1.87E-01	6.39E-03	3.63E-01
9740	19893_AB0198386	HESS	Breast Cancer	Breast	9.04E-03	-1.72E-01	1.75E-01	2.13E-01
9741	19893_AB0198388	HESS	Breast Cancer	Breast	1.58E-08	3.35E-01	2.28E-04	4.68E-01
9742	19893_AB0198389	HESS	Breast Cancer	Breast	6.97E-04	-2.13E-01	3.16E-02	-3.00E-01
9743	19893_AB0198390	HESS	Breast Cancer	Breast	2.59E-06	-2.84E-01	6.98E-04	-4.36E-01
9744	19893_AB0198813	HESS	Breast Cancer	Breast	9.27E-05	-2.41E-01	4.51E-02	-2.84E-01
9745	19893_AB0198840(	HESS	Breast Cancer	Breast	1.02E-07	-3.17E-01	5.07E-03	-3.71E-01
9746	19893_AB0198862	HESS	Breast Cancer	Breast	2.42E-01	-9.42E-02	1.96E-01	2.06E-01
9747	19893_AB0198863	HESS	Breast Cancer	Breast	7.36E-05	2.44E-01	1.46E-02	3.32E-01
9748	19893_AB0198864	HESS	Breast Cancer	Breast	4.88E-07	-3.02E-01	1.77E-02	-3.24E-01
9749	19893_AB0198866	HESS	Breast Cancer	Breast	1.50E-06	-2.90E-01	1.94E-03	-4.04E-01
9750	19893_AB0198870	HESS	Breast Cancer	Breast	3.56E-01	8.04E-02	1.52E-01	2.22E-01
9751	19893_AB0198870	HESS	Breast Cancer	Breast	3.50E-02	1.45E-01	3.70E-02	2.93E-01
9752	19893_AB0198871	HESS	Breast Cancer	Breast	5.39E-05	2.48E-01	1.17E-03	4.20E-01
9753	19893_AB0198871	HESS	Breast Cancer	Breast	2.59E-06	-2.84E-01	7.46E-02	-2.60E-01
9754	19893_AB0198872	HESS	Breast Cancer	Breast	7.45E-04	2.12E-01	4.07E-03	3.79E-01
9755	19893_AB0198874	HESS	Breast Cancer	Breast	7.60E-02	1.27E-01	5.45E-02	2.76E-01
9756	19893_AB0198874	HESS	Breast Cancer	Breast	1.97E-03	1.97E-01	3.35E-02	2.98E-01
9757	19893_AB0198874	HESS	Breast Cancer	Breast	4.53E-01	7.04E-02	3.46E-01	1.66E-01
9758	19893_AB0198878	HESS	Breast Cancer	Breast	5.01E-02	-1.37E-01	3.81E-01	1.59E-01
9759	19893_AB0201453(	HESS	Breast Cancer	Breast	1.82E-04	-2.32E-01	1.19E-01	-2.36E-01
9760	19893_AB0201458	HESS	Breast Cancer	Breast	4.13E-01	7.44E-02	8.66E-02	2.53E-01
9761	20537_AB0191320	HESS	Breast Cancer	Breast	1.35E-03	2.03E-01	2.45E-03	3.96E-01
9762	20537_AB0191324	HESS	Breast Cancer	Breast	1.00E-09	-3.60E-01	2.87E-05	-5.23E-01
9763	20537_AB0198391	HESS	Breast Cancer	Breast	5.25E-01	-6.35E-02	1.19E-01	2.36E-01

9764	20558_AB0171171	HESS	Breast Cancer	Breast	2.40E-02	-1.53E-01	3.81E-01	1.59E-01
9765	20558_AB0171216	HESS	Breast Cancer	Breast	3.67E-02	-1.44E-01	1.51E-01	2.22E-01
9766	20558_AB0172470	HESS	Breast Cancer	Breast	3.34E-02	1.46E-01	5.25E-03	3.70E-01
9767	20979_AB0191319	HESS	Breast Cancer	Breast	9.04E-03	-1.72E-01	3.58E-01	-1.64E-01
9768	20979_AB0191368	HESS	Breast Cancer	Breast	8.94E-10	3.61E-01	3.80E-06	5.71E-01
9769	20979_AB0194378	HESS	Breast Cancer	Breast	2.41E-08	-3.31E-01	1.56E-03	-4.11E-01
9770	20979_AB0198866	HESS	Breast Cancer	Breast	9.46E-07	-2.95E-01	6.39E-02	-2.68E-01
9771	20979_AB0198874	HESS	Breast Cancer	Breast	1.60E-05	-2.63E-01	4.45E-02	-2.85E-01
9772	23678_AB0123300	HESS	Breast Cancer	Breast	1.12E-01	1.17E-01	1.32E-01	2.30E-01
9773	23678_AB0123304	HESS	Breast Cancer	Breast	5.16E-03	-1.82E-01	2.29E-01	-1.96E-01
9774	23678_AB0129974	HESS	Breast Cancer	Breast	4.02E-04	-2.21E-01	7.74E-02	-2.58E-01
9775	23678_AB0129977	HESS	Breast Cancer	Breast	6.13E-03	-1.79E-01	2.49E-01	1.90E-01
9776	23678_AB0154214	HESS	Breast Cancer	Breast	1.65E-01	-1.06E-01	6.23E-02	2.69E-01
9777	23678_AB0154215	HESS	Breast Cancer	Breast	1.97E-03	-1.97E-01	2.90E-01	-1.80E-01
9778	23678_AB0154216	HESS	Breast Cancer	Breast	2.73E-07	-3.08E-01	9.68E-03	-3.48E-01
9779	23678_AB0154222	HESS	Breast Cancer	Breast	4.03E-01	7.54E-02	1.84E-01	2.10E-01
9780	23678_AB0154223	HESS	Breast Cancer	Breast	1.74E-05	-2.62E-01	1.82E-01	-2.11E-01
9781	23678_AB0154224	HESS	Breast Cancer	Breast	3.86E-03	-1.87E-01	3.14E-01	-1.74E-01
9782	23678_AB0156210	HESS	Breast Cancer	Breast	1.63E-03	2.00E-01	1.16E-02	3.41E-01
9783	23678_AB0156211	HESS	Breast Cancer	Breast	4.59E-02	1.39E-01	8.77E-02	2.52E-01
9784	23678_AB0156212	HESS	Breast Cancer	Breast	8.56E-03	-1.73E-01	3.81E-01	1.59E-01
9785	23678_AB0156213	HESS	Breast Cancer	Breast	1.50E-06	2.90E-01	1.73E-04	4.76E-01
9786	23678_AB0156215	HESS	Breast Cancer	Breast	5.83E-05	-2.47E-01	3.81E-02	-2.92E-01
9787	23678_AB0156215	HESS	Breast Cancer	Breast	5.47E-03	1.81E-01	1.90E-03	4.04E-01
9788	23678_AB0156221	HESS	Breast Cancer	Breast	1.12E-07	-3.16E-01	1.17E-03	-4.20E-01
9789	24817_AB0226070	HESS	Breast Cancer	Breast	1.01E-02	-1.70E-01	1.98E-01	-2.06E-01
9790	24817_AB0226097	HESS	Breast Cancer	Breast	1.31E-02	1.65E-01	1.51E-02	3.31E-01
9791	24817_AB0226148	HESS	Breast Cancer	Breast	1.30E-21	5.49E-01	3.45E-11	7.93E-01
9792	24817_AB0226150	HESS	Breast Cancer	Breast	1.16E-04	2.38E-01	6.98E-04	4.36E-01
9793	24817_AB0226150	HESS	Breast Cancer	Breast	1.18E-03	-2.05E-01	5.45E-02	-2.76E-01
9794	24817_AB0226151	HESS	Breast Cancer	Breast	3.67E-02	-1.44E-01	2.80E-01	1.82E-01
9795	24817_AB0226260	HESS	Breast Cancer	Breast	3.42E-03	1.88E-01	1.94E-03	4.04E-01
9796	24817_AB0226261	HESS	Breast Cancer	Breast	9.11E-04	-2.09E-01	1.86E-02	-3.23E-01
9797	24817_AB0226265	HESS	Breast Cancer	Breast	7.46E-06	-2.72E-01	2.93E-01	-1.79E-01
9798	24817_AB0226336	HESS	Breast Cancer	Breast	3.08E-05	-2.55E-01	7.55E-02	-2.60E-01
9799	24817_AB0226337	HESS	Breast Cancer	Breast	4.49E-08	-3.25E-01	2.31E-02	-3.14E-01
9800	24817_AB0226338	HESS	Breast Cancer	Breast	2.28E-01	9.62E-02	7.27E-02	2.62E-01
9801	24817_AB0226339	HESS	Breast Cancer	Breast	4.10E-11	-3.87E-01	3.88E-05	-5.15E-01
9802	24817_AB0226339	HESS	Breast Cancer	Breast	1.54E-01	1.08E-01	1.52E-01	2.22E-01
9803	24817_AB0226340	HESS	Breast Cancer	Breast	1.44E-01	1.10E-01	3.86E-02	2.91E-01
9804	24817_AB0226340	HESS	Breast Cancer	Breast	5.37E-07	-3.01E-01	2.77E-02	-3.06E-01
9805	24817_AB0226341	HESS	Breast Cancer	Breast	2.07E-02	-1.56E-01	2.49E-01	1.90E-01
9806	28998_AB0207726	HESS	Breast Cancer	Breast	8.24E-02	-1.25E-01	1.51E-01	2.22E-01
9807	28998_AB0208647	HESS	Breast Cancer	Breast	9.63E-02	1.21E-01	5.24E-02	2.77E-01
9808	28998_AB0208649	HESS	Breast Cancer	Breast	3.13E-01	-8.53E-02	4.02E-01	1.54E-01
9809	28998_AB0208650	HESS	Breast Cancer	Breast	1.26E-04	-2.37E-01	1.08E-01	-2.41E-01
9810	28998_AB0208869	HESS	Breast Cancer	Breast	1.65E-06	-2.89E-01	2.14E-02	-3.17E-01
9811	28998_AB0209018	HESS	Breast Cancer	Breast	1.47E-05	-2.64E-01	1.86E-02	-3.23E-01
9812	28998_AB0209023	HESS	Breast Cancer	Breast	9.63E-02	1.21E-01	6.15E-02	2.70E-01
9813	28998_AB0209066	HESS	Breast Cancer	Breast	4.60E-03	-1.84E-01	3.16E-01	-1.73E-01
9814	28998_AB0209071	HESS	Breast Cancer	Breast	1.95E-11	3.93E-01	1.96E-08	6.81E-01
9815	28998_AB0209109	HESS	Breast Cancer	Breast	3.67E-02	1.44E-01	7.36E-02	2.61E-01
9816	29099_AB0208868	HESS	Breast Cancer	Breast	1.37E-07	-3.14E-01	2.24E-02	-3.15E-01
9817	29539_AB0172300	HESS	Breast Cancer	Breast	1.61E-02	1.61E-01	2.11E-02	3.17E-01
9818	29539_AB0172302	HESS	Breast Cancer	Breast	4.10E-11	-3.87E-01	1.10E-05	-5.46E-01
9819	29539_AB0172303	HESS	Breast Cancer	Breast	4.31E-04	-2.20E-01	8.56E-02	-2.53E-01
9820	29539_AB0172303	HESS	Breast Cancer	Breast	2.29E-02	-1.54E-01	2.49E-01	1.90E-01
9821	29539_AB0172303	HESS	Breast Cancer	Breast	4.63E-01	-6.94E-02	2.83E-01	1.82E-01
9822	29539_AB0172303	HESS	Breast Cancer	Breast	8.13E-06	2.71E-01	3.05E-04	4.60E-01
9823	29539_AB0172304	HESS	Breast Cancer	Breast	7.67E-03	-1.75E-01	1.51E-01	2.22E-01
9824	29539_AB0172304	HESS	Breast Cancer	Breast	9.26E-02	-1.22E-01	2.88E-01	-1.80E-01
9825	29539_AB0172304	HESS	Breast Cancer	Breast	7.13E-12	-4.01E-01	3.12E-04	-4.59E-01
9826	29539_AB0172304	HESS	Breast Cancer	Breast	3.04E-03	-1.90E-01	1.15E-01	-2.37E-01
9827	29539_AB0172305	HESS	Breast Cancer	Breast	9.65E-06	2.69E-01	5.28E-04	4.44E-01
9828	29539_AB0183349	HESS	Breast Cancer	Breast	1.16E-01	-1.16E-01	1.51E-01	2.22E-01
9829	29539_AB0183350	HESS	Breast Cancer	Breast	8.94E-10	3.61E-01	2.79E-06	5.78E-01
9830	29539_AB0183351	HESS	Breast Cancer	Breast	2.25E-07	-3.10E-01	2.27E-02	-3.14E-01
9831	29539_AB0183352	HESS	Breast Cancer	Breast	4.95E-04	2.18E-01	3.19E-03	3.87E-01
9832	29539_AB0183352	HESS	Breast Cancer	Breast	7.97E-04	2.11E-01	7.13E-04	4.35E-01
9833	29539_AB0183353	HESS	Breast Cancer	Breast	3.39E-06	-2.81E-01	3.81E-02	-2.92E-01
9834	29539_AB0183354	HESS	Breast Cancer	Breast	2.40E-02	-1.53E-01	2.49E-01	1.90E-01
9835	29539_AB0183369	HESS	Breast Cancer	Breast	2.10E-03	1.96E-01	2.59E-03	3.94E-01
9836	29539_AB0183371	HESS	Breast Cancer	Breast	1.18E-02	1.67E-01	4.57E-02	2.84E-01
9837	29539_AB0183372	HESS	Breast Cancer	Breast	1.54E-01	-1.08E-01	3.84E-01	1.58E-01

9838	29539_AB01833732	HESS	Breast Cancer	Breast	1.74E-03	-1.99E-01	1.17E-01	-2.37E-01
9839	29539_AB01833741	HESS	Breast Cancer	Breast	7.14E-07	-2.98E-01	2.61E-02	-3.09E-01
9840	29539_AB01833747	HESS	Breast Cancer	Breast	3.19E-02	1.47E-01	6.15E-02	2.70E-01
9841	29539_AB01833749	HESS	Breast Cancer	Breast	1.65E-01	1.06E-01	2.88E-01	1.80E-01
9842	29539_AB01833754	HESS	Breast Cancer	Breast	2.84E-06	-2.83E-01	1.44E-02	-3.33E-01
9843	29539_AB01833756	HESS	Breast Cancer	Breast	2.64E-02	1.51E-01	5.24E-02	2.77E-01
9844	29539_AB01833758	HESS	Breast Cancer	Breast	2.40E-02	-1.53E-01	1.54E-01	-2.21E-01
9845	29539_AB01833759	HESS	Breast Cancer	Breast	6.84E-06	-2.73E-01	2.36E-01	-1.94E-01
9846	29539_AB01833769	HESS	Breast Cancer	Breast	1.27E-19	5.22E-01	3.45E-11	7.93E-01
9847	29539_AB01833780	HESS	Breast Cancer	Breast	1.18E-11	-3.97E-01	4.25E-04	-4.50E-01
9848	29539_AB01833820	HESS	Breast Cancer	Breast	2.17E-08	-3.32E-01	1.59E-03	-4.10E-01
9849	29539_AB01833821	HESS	Breast Cancer	Breast	2.28E-01	-9.62E-02	4.77E-01	1.39E-01
9850	29539_AB01833829	HESS	Breast Cancer	Breast	2.35E-01	9.52E-02	2.36E-01	1.94E-01
9851	29539_AB01833832	HESS	Breast Cancer	Breast	6.48E-03	-1.78E-01	2.27E-01	-1.97E-01
9852	29539_AB01833840	HESS	Breast Cancer	Breast	6.08E-04	-2.15E-01	3.11E-01	-1.75E-01
9853	29539_AB01833841	HESS	Breast Cancer	Breast	1.75E-08	-3.34E-01	9.52E-03	-3.49E-01
9854	29539_AB01833876	HESS	Breast Cancer	Breast	4.60E-03	-1.84E-01	1.98E-01	-2.06E-01
9855	29539_AB01833931	HESS	Breast Cancer	Breast	3.86E-03	-1.87E-01	1.14E-01	2.38E-01
9856	29539_AB01833935	HESS	Breast Cancer	Breast	3.34E-05	-2.54E-01	5.60E-02	-2.74E-01
9857	GSM570498	GSE23177	Breast Cancer	Breast	3.38E-01	8.23E-02	3.16E-02	3.00E-01
9858	GSM570499	GSE23177	Breast Cancer	Breast	5.64E-10	-3.65E-01	6.98E-04	-4.36E-01
9859	GSM570500	GSE23177	Breast Cancer	Breast	5.30E-04	-2.17E-01	1.00E-01	-2.45E-01
9860	GSM570501	GSE23177	Breast Cancer	Breast	8.61E-07	-2.96E-01	5.31E-02	-2.77E-01
9861	GSM570502	GSE23177	Breast Cancer	Breast	1.65E-01	-1.06E-01	3.81E-01	1.59E-01
9862	GSM570503	GSE23177	Breast Cancer	Breast	2.49E-01	-9.33E-02	2.02E-01	2.04E-01
9863	GSM570504	GSE23177	Breast Cancer	Breast	2.41E-08	-3.31E-01	7.28E-04	-4.34E-01
9864	GSM570505	GSE23177	Breast Cancer	Breast	4.39E-02	1.40E-01	1.51E-01	2.22E-01
9865	GSM570506	GSE23177	Breast Cancer	Breast	4.02E-04	-2.21E-01	1.01E-01	-2.44E-01
9866	GSM570507	GSE23177	Breast Cancer	Breast	6.48E-03	1.78E-01	9.52E-03	3.49E-01
9867	GSM570508	GSE23177	Breast Cancer	Breast	1.08E-01	-1.18E-01	1.37E-01	2.28E-01
9868	GSM570509	GSE23177	Breast Cancer	Breast	1.38E-02	1.64E-01	3.07E-02	3.02E-01
9869	GSM570510	GSE23177	Breast Cancer	Breast	9.65E-06	2.69E-01	1.06E-04	4.89E-01
9870	GSM570511	GSE23177	Breast Cancer	Breast	1.08E-01	1.18E-01	1.01E-01	2.44E-01
9871	GSM570512	GSE23177	Breast Cancer	Breast	1.44E-01	-1.10E-01	1.52E-01	2.22E-01
9872	GSM570513	GSE23177	Breast Cancer	Breast	8.91E-02	-1.23E-01	1.98E-01	2.06E-01
9873	GSM570514	GSE23177	Breast Cancer	Breast	1.04E-01	-1.19E-01	1.96E-01	2.06E-01
9874	GSM570515	GSE23177	Breast Cancer	Breast	2.07E-02	-1.56E-01	2.54E-01	-1.89E-01
9875	GSM570516	GSE23177	Breast Cancer	Breast	7.92E-02	-1.26E-01	1.54E-01	-2.21E-01
9876	GSM570517	GSE23177	Breast Cancer	Breast	8.57E-02	1.24E-01	6.15E-02	2.70E-01
9877	GSM570518	GSE23177	Breast Cancer	Breast	3.50E-02	-1.45E-01	2.83E-01	1.82E-01
9878	GSM570519	GSE23177	Breast Cancer	Breast	3.19E-02	-1.47E-01	2.51E-01	-1.90E-01
9879	GSM570520	GSE23177	Breast Cancer	Breast	4.20E-02	1.41E-01	1.42E-02	3.33E-01
9880	GSM570521	GSE23177	Breast Cancer	Breast	2.29E-02	-1.54E-01	2.49E-01	1.90E-01
9881	GSM570522	GSE23177	Breast Cancer	Breast	1.01E-02	1.70E-01	3.07E-02	3.02E-01
9882	GSM570523	GSE23177	Breast Cancer	Breast	1.04E-01	1.19E-01	2.61E-02	3.09E-01
9883	GSM570524	GSE23177	Breast Cancer	Breast	1.44E-01	-1.10E-01	1.15E-01	2.37E-01
9884	GSM570525	GSE23177	Breast Cancer	Breast	1.89E-01	-1.02E-01	2.85E-01	1.81E-01
9885	GSM570526	GSE23177	Breast Cancer	Breast	8.10E-03	1.74E-01	1.80E-02	3.24E-01
9886	GSM570527	GSE23177	Breast Cancer	Breast	5.01E-02	-1.37E-01	1.96E-01	2.06E-01
9887	GSM570528	GSE23177	Breast Cancer	Breast	5.69E-02	-1.34E-01	2.83E-01	1.82E-01
9888	GSM570529	GSE23177	Breast Cancer	Breast	1.77E-01	1.04E-01	1.14E-01	2.38E-01
9889	GSM570530	GSE23177	Breast Cancer	Breast	9.11E-04	-2.09E-01	1.51E-01	2.22E-01
9890	GSM570531	GSE23177	Breast Cancer	Breast	2.72E-01	9.03E-02	2.88E-01	1.80E-01
9891	GSM570532	GSE23177	Breast Cancer	Breast	7.11E-10	-3.63E-01	7.13E-04	-4.35E-01
9892	GSM570533	GSE23177	Breast Cancer	Breast	1.97E-03	1.97E-01	2.57E-02	3.09E-01
9893	GSM570534	GSE23177	Breast Cancer	Breast	3.31E-12	-4.07E-01	7.36E-05	-4.98E-01
9894	GSM570535	GSE23177	Breast Cancer	Breast	5.69E-02	-1.34E-01	1.75E-01	-2.13E-01
9895	GSM570536	GSE23177	Breast Cancer	Breast	2.77E-02	-1.50E-01	1.96E-01	2.06E-01
9896	GSM570537	GSE23177	Breast Cancer	Breast	7.30E-02	-1.28E-01	5.39E-01	1.27E-01
9897	GSM570538	GSE23177	Breast Cancer	Breast	5.68E-04	-2.16E-01	2.27E-01	-1.97E-01
9898	GSM570539	GSE23177	Breast Cancer	Breast	1.25E-01	1.14E-01	6.15E-02	2.70E-01
9899	GSM570540	GSE23177	Breast Cancer	Breast	7.36E-05	-2.44E-01	3.97E-02	-2.90E-01
9900	GSM570541	GSE23177	Breast Cancer	Breast	5.57E-01	6.05E-02	2.63E-01	1.87E-01
9901	GSM570542	GSE23177	Breast Cancer	Breast	3.84E-01	7.74E-02	1.22E-01	2.34E-01
9902	GSM570543	GSE23177	Breast Cancer	Breast	7.92E-02	1.26E-01	1.80E-02	3.24E-01
9903	GSM570544	GSE23177	Breast Cancer	Breast	1.00E-01	-1.20E-01	3.63E-01	-1.63E-01
9904	GSM570545	GSE23177	Breast Cancer	Breast	4.33E-01	-7.24E-02	7.55E-02	2.60E-01
9905	GSM570546	GSE23177	Breast Cancer	Breast	8.52E-04	-2.10E-01	1.96E-01	2.06E-01
9906	GSM570547	GSE23177	Breast Cancer	Breast	2.52E-02	-1.52E-01	4.61E-01	1.42E-01
9907	GSM570548	GSE23177	Breast Cancer	Breast	1.98E-09	-3.54E-01	6.98E-04	-4.36E-01
9908	GSM570549	GSE23177	Breast Cancer	Breast	3.45E-09	-3.49E-01	2.54E-03	-3.95E-01
9909	GSM570550	GSE23177	Breast Cancer	Breast	6.84E-06	2.73E-01	3.05E-04	4.60E-01
9910	GSM570551	GSE23177	Breast Cancer	Breast	1.63E-03	-2.00E-01	1.77E-01	-2.13E-01
9911	GSM570552	GSE23177	Breast Cancer	Breast	2.69E-03	1.92E-01	7.75E-03	3.56E-01

9912	GSM570553	GSE23177	Breast Cancer	Breast	2.40E-02	-1.53E-01	2.31E-01	-1.96E-01
9913	GSM570554	GSE23177	Breast Cancer	Breast	1.08E-04	-2.39E-01	1.59E-01	-2.19E-01
9914	GSM570555	GSE23177	Breast Cancer	Breast	6.86E-03	1.77E-01	9.36E-03	3.49E-01
9915	GSM570556	GSE23177	Breast Cancer	Breast	1.79E-02	-1.59E-01	2.51E-01	-1.90E-01
9916	GSM570557	GSE23177	Breast Cancer	Breast	5.53E-12	4.03E-01	1.40E-07	6.42E-01
9917	GSM570558	GSE23177	Breast Cancer	Breast	2.42E-05	2.58E-01	7.95E-06	5.54E-01
9918	GSM570559	GSE23177	Breast Cancer	Breast	8.86E-06	-2.70E-01	3.20E-02	-3.00E-01
9919	GSM570560	GSE23177	Breast Cancer	Breast	4.95E-04	-2.18E-01	2.29E-01	-1.96E-01
9920	GSM570561	GSE23177	Breast Cancer	Breast	7.50E-08	-3.20E-01	1.22E-01	-2.34E-01
9921	GSM570562	GSE23177	Breast Cancer	Breast	1.04E-06	-2.94E-01	7.94E-02	-2.57E-01
9922	GSM570563	GSE23177	Breast Cancer	Breast	1.89E-01	1.02E-01	1.52E-01	2.22E-01
9923	GSM570564	GSE23177	Breast Cancer	Breast	2.62E-05	-2.57E-01	2.17E-02	-3.16E-01
9924	GSM570565	GSE23177	Breast Cancer	Breast	1.27E-08	3.37E-01	3.69E-06	5.71E-01
9925	GSM570566	GSE23177	Breast Cancer	Breast	2.07E-02	-1.56E-01	5.06E-01	-1.33E-01
9926	GSM570567	GSE23177	Breast Cancer	Breast	2.84E-06	-2.83E-01	6.28E-03	-3.64E-01
9927	GSM570568	GSE23177	Breast Cancer	Breast	4.39E-02	-1.40E-01	1.52E-01	-2.22E-01
9928	GSM570569	GSE23177	Breast Cancer	Breast	1.53E-03	-2.01E-01	7.46E-02	-2.60E-01
9929	GSM570570	GSE23177	Breast Cancer	Breast	8.91E-02	1.23E-01	3.92E-02	2.91E-01
9930	GSM570571	GSE23177	Breast Cancer	Breast	2.05E-05	-2.60E-01	4.98E-03	-3.72E-01
9931	GSM570572	GSE23177	Breast Cancer	Breast	2.07E-02	1.56E-01	2.61E-02	3.09E-01
9932	GSM570573	GSE23177	Breast Cancer	Breast	2.42E-01	-9.42E-02	3.61E-01	1.63E-01
9933	GSM570574	GSE23177	Breast Cancer	Breast	1.71E-01	1.05E-01	6.48E-02	2.67E-01
9934	GSM570575	GSE23177	Breast Cancer	Breast	1.65E-01	-1.06E-01	6.15E-02	2.70E-01
9935	GSM570576	GSE23177	Breast Cancer	Breast	2.86E-03	-1.91E-01	3.87E-01	-1.57E-01
9936	GSM570577	GSE23177	Breast Cancer	Breast	9.73E-04	-2.08E-01	2.25E-01	-1.97E-01
9937	GSM570578	GSE23177	Breast Cancer	Breast	1.25E-06	-2.92E-01	3.86E-02	-2.91E-01
9938	GSM570579	GSE23177	Breast Cancer	Breast	1.14E-05	2.67E-01	1.25E-03	4.18E-01
9939	GSM570580	GSE23177	Breast Cancer	Breast	3.67E-02	1.44E-01	6.15E-02	2.70E-01
9940	GSM570581	GSE23177	Breast Cancer	Breast	1.25E-02	-1.66E-01	6.23E-01	1.11E-01
9941	GSM570582	GSE23177	Breast Cancer	Breast	2.29E-02	1.54E-01	3.98E-04	4.52E-01
9942	GSM570583	GSE23177	Breast Cancer	Breast	2.40E-02	-1.53E-01	2.49E-01	1.90E-01
9943	GSM570584	GSE23177	Breast Cancer	Breast	1.31E-02	-1.65E-01	4.45E-02	-2.85E-01
9944	GSM570585	GSE23177	Breast Cancer	Breast	2.38E-03	-1.94E-01	1.57E-01	-2.20E-01
9945	GSM570586	GSE23177	Breast Cancer	Breast	7.84E-07	2.97E-01	2.72E-05	5.24E-01
9946	GSM570587	GSE23177	Breast Cancer	Breast	2.40E-02	1.53E-01	4.45E-02	2.85E-01
9947	GSM570588	GSE23177	Breast Cancer	Breast	1.16E-01	1.16E-01	6.15E-02	2.70E-01
9948	GSM570589	GSE23177	Breast Cancer	Breast	4.59E-02	1.39E-01	3.07E-02	3.02E-01
9949	GSM570590	GSE23177	Breast Cancer	Breast	9.26E-02	1.22E-01	1.00E-02	3.47E-01
9950	GSM570591	GSE23177	Breast Cancer	Breast	2.01E-01	-1.00E-01	8.45E-02	2.54E-01
9951	GSM570592	GSE23177	Breast Cancer	Breast	1.04E-03	-2.07E-01	4.26E-01	-1.49E-01
9952	GSM570593	GSE23177	Breast Cancer	Breast	2.88E-01	8.83E-02	3.07E-02	3.02E-01
9953	GSM570594	GSE23177	Breast Cancer	Breast	3.05E-02	1.48E-01	2.11E-02	3.17E-01
9954	GSM570595	GSE23177	Breast Cancer	Breast	3.74E-04	-2.22E-01	1.75E-01	-2.13E-01
9955	GSM570596	GSE23177	Breast Cancer	Breast	9.63E-02	1.21E-01	3.07E-02	3.02E-01
9956	GSM570597	GSE23177	Breast Cancer	Breast	2.96E-01	-8.73E-02	5.39E-01	1.27E-01
9957	GSM570598	GSE23177	Breast Cancer	Breast	3.56E-01	-8.04E-02	1.01E-01	2.44E-01
9958	GSM570599	GSE23177	Breast Cancer	Breast	2.28E-01	-9.62E-02	1.77E-01	2.13E-01
9959	GSM570600	GSE23177	Breast Cancer	Breast	3.75E-01	-7.84E-02	1.51E-01	2.22E-01
9960	GSM570601	GSE23177	Breast Cancer	Breast	1.95E-01	-1.01E-01	2.49E-01	1.90E-01
9961	GSM570602	GSE23177	Breast Cancer	Breast	1.34E-01	1.12E-01	4.00E-03	3.80E-01
9962	GSM570603	GSE23177	Breast Cancer	Breast	2.57E-01	-9.23E-02	1.14E-01	2.38E-01
9963	GSM570604	GSE23177	Breast Cancer	Breast	6.19E-02	1.32E-01	1.06E-01	2.42E-01
9964	GSM570605	GSE23177	Breast Cancer	Breast	3.19E-02	-1.47E-01	2.83E-01	-1.82E-01
9965	GSM570606	GSE23177	Breast Cancer	Breast	2.67E-08	-3.30E-01	1.98E-03	-4.03E-01
9966	GSM570607	GSE23177	Breast Cancer	Breast	1.77E-01	1.04E-01	4.38E-02	2.86E-01
9967	GSM570608	GSE23177	Breast Cancer	Breast	1.38E-02	-1.64E-01	2.27E-01	-1.97E-01
9968	GSM570609	GSE23177	Breast Cancer	Breast	1.58E-08	-3.35E-01	3.12E-04	-4.59E-01
9969	GSM570610	GSE23177	Breast Cancer	Breast	2.42E-05	-2.58E-01	4.51E-02	-2.84E-01
9970	GSM570611	GSE23177	Breast Cancer	Breast	5.30E-04	-2.17E-01	1.18E-01	-2.36E-01
9971	GSM570612	GSE23177	Breast Cancer	Breast	3.50E-02	1.45E-01	5.31E-02	2.77E-01
9972	GSM570613	GSE23177	Breast Cancer	Breast	3.67E-02	1.44E-01	1.24E-02	3.38E-01
9973	GSM578633	GSE23593	Breast Cancer	Breast	1.53E-02	-1.62E-01	2.80E-01	1.82E-01
9974	GSM578634	GSE23593	Breast Cancer	Breast	1.21E-01	-1.15E-01	3.46E-01	1.66E-01
9975	GSM578635	GSE23593	Breast Cancer	Breast	5.01E-02	-1.37E-01	2.83E-01	-1.82E-01
9976	GSM578636	GSE23593	Breast Cancer	Breast	8.57E-02	-1.24E-01	1.51E-01	2.22E-01
9977	GSM578637	GSE23593	Breast Cancer	Breast	4.28E-12	-4.05E-01	3.01E-05	-5.21E-01
9978	GSM578638	GSE23593	Breast Cancer	Breast	1.16E-01	-1.16E-01	1.96E-01	2.06E-01
9979	GSM578639	GSE23593	Breast Cancer	Breast	7.36E-05	2.44E-01	1.15E-03	4.20E-01
9980	GSM578640	GSE23593	Breast Cancer	Breast	3.63E-03	1.88E-01	1.47E-03	4.13E-01
9981	GSM578641	GSE23593	Breast Cancer	Breast	2.80E-10	3.71E-01	5.29E-06	5.63E-01
9982	GSM578642	GSE23593	Breast Cancer	Breast	1.04E-01	-1.19E-01	2.51E-01	1.90E-01
9983	GSM578643	GSE23593	Breast Cancer	Breast	3.34E-02	-1.46E-01	3.11E-01	1.75E-01
9984	GSM578644	GSE23593	Breast Cancer	Breast	5.46E-02	-1.35E-01	5.00E-01	1.35E-01
9985	GSM578645	GSE23593	Breast Cancer	Breast	6.30E-05	-2.46E-01	7.65E-02	-2.59E-01

9986	GSM578646	GSE23593	Breast Cancer	Breast	3.34E-05	-2.54E-01	1.21E-01	-2.35E-01
9987	GSM578647	GSE23593	Breast Cancer	Breast	1.60E-01	1.07E-01	1.96E-01	2.06E-01
9988	GSM578648	GSE23593	Breast Cancer	Breast	1.17E-12	-4.15E-01	1.22E-03	-4.18E-01
9989	GSM578649	GSE23593	Breast Cancer	Breast	1.26E-04	-2.37E-01	5.53E-02	-2.75E-01
9990	GSM578650	GSE23593	Breast Cancer	Breast	2.07E-02	1.56E-01	2.57E-02	3.09E-01
9991	GSM578651	GSE23593	Breast Cancer	Breast	2.91E-02	1.49E-01	4.45E-02	2.85E-01
9992	GSM578652	GSE23593	Breast Cancer	Breast	3.05E-02	1.48E-01	3.16E-02	3.00E-01
9993	GSM578653	GSE23593	Breast Cancer	Breast	2.49E-01	-9.33E-02	3.11E-01	1.75E-01
9994	GSM578654	GSE23593	Breast Cancer	Breast	5.28E-13	4.21E-01	2.14E-09	7.22E-01
9995	GSM578655	GSE23593	Breast Cancer	Breast	1.18E-11	3.97E-01	2.14E-09	7.22E-01
9996	GSM578656	GSE23593	Breast Cancer	Breast	4.81E-09	3.46E-01	6.40E-07	6.10E-01
9997	GSM578657	GSE23593	Breast Cancer	Breast	5.68E-04	2.16E-01	2.33E-04	4.67E-01
9998	GSM578658	GSE23593	Breast Cancer	Breast	1.95E-01	1.01E-01	1.04E-01	2.43E-01
9999	GSM578659	GSE23593	Breast Cancer	Breast	4.03E-07	-3.04E-01	1.89E-02	-3.22E-01
10000	GSM578660	GSE23593	Breast Cancer	Breast	7.97E-04	-2.11E-01	2.36E-01	-1.94E-01
10001	GSM578661	GSE23593	Breast Cancer	Breast	2.21E-10	-3.73E-01	2.64E-03	-3.94E-01
10002	GSM578662	GSE23593	Breast Cancer	Breast	1.02E-12	-4.16E-01	2.49E-04	-4.65E-01
10003	GSM578663	GSE23593	Breast Cancer	Breast	2.21E-11	-3.92E-01	1.85E-04	-4.74E-01
10004	GSM578664	GSE23593	Breast Cancer	Breast	9.23E-09	-3.40E-01	1.56E-03	-4.11E-01
10005	GSM578665	GSE23593	Breast Cancer	Breast	3.98E-10	-3.68E-01	2.01E-03	-4.03E-01
10006	GSM578666	GSE23593	Breast Cancer	Breast	5.68E-04	-2.16E-01	7.65E-02	-2.59E-01
10007	GSM578667	GSE23593	Breast Cancer	Breast	2.88E-01	8.83E-02	7.74E-02	2.58E-01
10008	GSM578668	GSE23593	Breast Cancer	Breast	5.68E-04	-2.16E-01	3.66E-01	-1.62E-01
10009	GSM578669	GSE23593	Breast Cancer	Breast	9.11E-04	2.09E-01	1.28E-04	4.84E-01
10010	GSM578670	GSE23593	Breast Cancer	Breast	1.46E-02	1.63E-01	3.13E-03	3.88E-01
10011	GSM578671	GSE23593	Breast Cancer	Breast	3.30E-01	8.33E-02	7.46E-02	2.60E-01
10012	GSM578672	GSE23593	Breast Cancer	Breast	1.12E-01	-1.17E-01	4.58E-01	1.43E-01
10013	GSM578673	GSE23593	Breast Cancer	Breast	2.64E-01	9.13E-02	1.77E-01	2.13E-01
10014	GSM578674	GSE23593	Breast Cancer	Breast	1.49E-01	-1.09E-01	4.23E-01	1.50E-01
10015	GSM578675	GSE23593	Breast Cancer	Breast	1.10E-15	-4.64E-01	3.88E-05	-5.15E-01
10016	GSM578676	GSE23593	Breast Cancer	Breast	1.19E-13	-4.32E-01	2.87E-05	-5.23E-01
10017	GSM578677	GSE23593	Breast Cancer	Breast	7.46E-06	2.72E-01	4.08E-05	5.14E-01
10018	GSM578678	GSE23593	Breast Cancer	Breast	1.38E-10	3.77E-01	4.47E-07	6.18E-01
10019	GSM578679	GSE23593	Breast Cancer	Breast	1.63E-03	2.00E-01	1.17E-03	4.20E-01
10020	GSM578680	GSE23593	Breast Cancer	Breast	3.08E-05	2.55E-01	5.23E-05	5.07E-01
10021	GSM578681	GSE23593	Breast Cancer	Breast	9.11E-04	-2.09E-01	3.24E-01	-1.71E-01
10022	GSM578682	GSE23593	Breast Cancer	Breast	2.28E-01	9.62E-02	6.56E-02	2.67E-01
10023	GSM590840	GSE23988	Breast Cancer	Breast	1.85E-07	3.12E-01	3.05E-04	4.60E-01
10024	GSM590841	GSE23988	Breast Cancer	Breast	5.68E-04	2.16E-01	4.07E-03	3.79E-01
10025	GSM590842	GSE23988	Breast Cancer	Breast	5.83E-05	2.47E-01	7.13E-04	4.35E-01
10026	GSM590843	GSE23988	Breast Cancer	Breast	1.11E-03	-2.06E-01	2.06E-01	-2.03E-01
10027	GSM590844	GSE23988	Breast Cancer	Breast	3.23E-03	-1.89E-01	2.49E-01	1.90E-01
10028	GSM590845	GSE23988	Breast Cancer	Breast	1.04E-01	1.19E-01	1.96E-01	2.06E-01
10029	GSM590846	GSE23988	Breast Cancer	Breast	3.84E-01	-7.74E-02	1.59E-01	2.19E-01
10030	GSM590847	GSE23988	Breast Cancer	Breast	1.34E-11	-3.96E-01	6.98E-04	-4.36E-01
10031	GSM590848	GSE23988	Breast Cancer	Breast	4.23E-01	7.34E-02	2.08E-01	2.03E-01
10032	GSM590849	GSE23988	Breast Cancer	Breast	9.04E-03	1.72E-01	1.51E-01	2.22E-01
10033	GSM590850	GSE23988	Breast Cancer	Breast	3.21E-01	8.43E-02	3.11E-01	1.75E-01
10034	GSM590851	GSE23988	Breast Cancer	Breast	5.79E-03	-1.80E-01	1.17E-01	-2.37E-01
10035	GSM590852	GSE23988	Breast Cancer	Breast	1.38E-02	1.64E-01	5.24E-02	2.77E-01
10036	GSM590853	GSE23988	Breast Cancer	Breast	2.37E-06	2.85E-01	5.36E-05	5.07E-01
10037	GSM590854	GSE23988	Breast Cancer	Breast	5.98E-09	-3.44E-01	2.54E-03	-3.95E-01
10038	GSM590855	GSE23988	Breast Cancer	Breast	9.63E-02	1.21E-01	2.49E-01	1.90E-01
10039	GSM590856	GSE23988	Breast Cancer	Breast	2.21E-10	-3.73E-01	5.40E-04	-4.43E-01
10040	GSM590857	GSE23988	Breast Cancer	Breast	1.46E-02	-1.63E-01	1.61E-01	-2.18E-01
10041	GSM590858	GSE23988	Breast Cancer	Breast	1.30E-01	-1.13E-01	2.80E-01	1.82E-01
10042	GSM590859	GSE23988	Breast Cancer	Breast	1.08E-04	-2.39E-01	8.66E-02	-2.53E-01
10043	GSM590860	GSE23988	Breast Cancer	Breast	2.38E-03	1.94E-01	1.75E-02	3.25E-01
10044	GSM590861	GSE23988	Breast Cancer	Breast	3.62E-05	2.53E-01	1.73E-04	4.76E-01
10045	GSM590862	GSE23988	Breast Cancer	Breast	3.67E-02	-1.44E-01	2.27E-01	-1.97E-01
10046	GSM590863	GSE23988	Breast Cancer	Breast	1.53E-02	-1.62E-01	1.98E-01	-2.06E-01
10047	GSM590864	GSE23988	Breast Cancer	Breast	7.84E-07	-2.97E-01	1.54E-01	-2.21E-01
10048	GSM590865	GSE23988	Breast Cancer	Breast	3.63E-03	-1.88E-01	1.79E-01	-2.12E-01
10049	GSM590866	GSE23988	Breast Cancer	Breast	1.26E-03	-2.04E-01	7.36E-02	-2.61E-01
10050	GSM590867	GSE23988	Breast Cancer	Breast	1.65E-01	-1.06E-01	1.54E-01	2.21E-01
10051	GSM590868	GSE23988	Breast Cancer	Breast	4.87E-03	-1.83E-01	1.57E-01	-2.20E-01
10052	GSM590869	GSE23988	Breast Cancer	Breast	7.97E-04	2.11E-01	1.53E-03	4.11E-01
10053	GSM590870	GSE23988	Breast Cancer	Breast	6.19E-02	1.32E-01	1.77E-02	3.24E-01
10054	GSM590871	GSE23988	Breast Cancer	Breast	5.52E-08	3.23E-01	2.33E-04	4.67E-01
10055	GSM590872	GSE23988	Breast Cancer	Breast	1.79E-02	-1.59E-01	1.51E-01	2.22E-01
10056	GSM590873	GSE23988	Breast Cancer	Breast	2.72E-01	9.03E-02	3.55E-01	1.64E-01
10057	GSM590874	GSE23988	Breast Cancer	Breast	8.52E-04	-2.10E-01	2.22E-01	1.98E-01
10058	GSM590875	GSE23988	Breast Cancer	Breast	2.53E-03	1.93E-01	6.39E-03	3.63E-01
10059	GSM590876	GSE23988	Breast Cancer	Breast	4.05E-06	-2.79E-01	1.89E-02	-3.22E-01

10060	GSM590877	GSE23988	Breast Cancer	Breast	2.38E-03	-1.94E-01	1.96E-01	2.06E-01
10061	GSM590878	GSE23988	Breast Cancer	Breast	1.25E-02	-1.66E-01	3.27E-01	-1.71E-01
10062	GSM590879	GSE23988	Breast Cancer	Breast	8.24E-02	-1.25E-01	1.52E-01	2.22E-01
10063	GSM590880	GSE23988	Breast Cancer	Breast	4.02E-04	2.21E-01	7.13E-04	4.35E-01
10064	GSM590881	GSE23988	Breast Cancer	Breast	7.97E-04	-2.11E-01	2.22E-01	1.98E-01
10065	GSM590882	GSE23988	Breast Cancer	Breast	7.92E-02	-1.26E-01	3.81E-01	1.59E-01
10066	GSM590883	GSE23988	Breast Cancer	Breast	3.67E-02	-1.44E-01	4.70E-01	-1.40E-01
10067	GSM590884	GSE23988	Breast Cancer	Breast	1.16E-04	2.38E-01	9.83E-05	4.91E-01
10068	GSM590885	GSE23988	Breast Cancer	Breast	8.24E-02	1.25E-01	1.52E-01	2.22E-01
10069	GSM590886	GSE23988	Breast Cancer	Breast	6.48E-03	1.78E-01	7.19E-05	4.99E-01
10070	GSM590887	GSE23988	Breast Cancer	Breast	2.84E-05	-2.56E-01	1.18E-02	-3.40E-01
10071	GSM590888	GSE23988	Breast Cancer	Breast	1.02E-07	-3.17E-01	6.17E-03	-3.64E-01
10072	GSM590889	GSE23988	Breast Cancer	Breast	1.83E-01	-1.03E-01	2.22E-01	1.98E-01
10073	GSM590890	GSE23988	Breast Cancer	Breast	8.56E-03	-1.73E-01	1.79E-01	-2.12E-01
10074	GSM590891	GSE23988	Breast Cancer	Breast	8.29E-09	-3.41E-01	1.22E-02	-3.39E-01
10075	GSM590892	GSE23988	Breast Cancer	Breast	7.45E-04	-2.12E-01	3.01E-01	-1.77E-01
10076	GSM590893	GSE23988	Breast Cancer	Breast	1.82E-04	-2.32E-01	1.98E-01	-2.06E-01
10077	GSM590894	GSE23988	Breast Cancer	Breast	1.35E-04	2.36E-01	1.81E-04	4.74E-01
10078	GSM590895	GSE23988	Breast Cancer	Breast	9.65E-06	-2.69E-01	2.33E-01	-1.95E-01
10079	GSM590896	GSE23988	Breast Cancer	Breast	1.49E-01	-1.09E-01	4.20E-01	1.50E-01
10080	GSM590897	GSE23988	Breast Cancer	Breast	2.21E-09	-3.53E-01	6.39E-03	-3.63E-01
10081	GSM590898	GSE23988	Breast Cancer	Breast	8.10E-03	-1.74E-01	3.84E-01	-1.58E-01
10082	GSM590899	GSE23988	Breast Cancer	Breast	2.21E-01	9.72E-02	2.68E-02	3.07E-01
10083	GSM590900	GSE23988	Breast Cancer	Breast	1.54E-01	-1.08E-01	2.49E-01	1.90E-01
10084	GSM615096	GSE25066	Breast Cancer	Breast	1.30E-01	-1.13E-01	1.96E-01	2.06E-01
10085	GSM615097	GSE25066	Breast Cancer	Breast	2.77E-02	1.50E-01	2.11E-02	3.17E-01
10086	GSM615098	GSE25066	Breast Cancer	Breast	5.94E-02	1.33E-01	1.75E-02	3.25E-01
10087	GSM615099	GSE25066	Breast Cancer	Breast	1.97E-03	1.97E-01	7.88E-03	3.56E-01
10088	GSM615100	GSE25066	Breast Cancer	Breast	1.08E-04	2.39E-01	6.17E-03	3.64E-01
10089	GSM615101	GSE25066	Breast Cancer	Breast	2.77E-02	1.50E-01	1.75E-02	3.25E-01
10090	GSM615102	GSE25066	Breast Cancer	Breast	1.12E-01	1.17E-01	1.77E-02	3.24E-01
10091	GSM615103	GSE25066	Breast Cancer	Breast	3.03E-04	-2.25E-01	1.63E-01	-2.18E-01
10092	GSM615104	GSE25066	Breast Cancer	Breast	9.63E-02	1.21E-01	9.85E-03	3.47E-01
10093	GSM615105	GSE25066	Breast Cancer	Breast	1.44E-03	-2.02E-01	1.15E-01	-2.37E-01
10094	GSM615106	GSE25066	Breast Cancer	Breast	5.79E-03	1.80E-01	2.05E-03	4.02E-01
10095	GSM615107	GSE25066	Breast Cancer	Breast	2.57E-01	9.23E-02	3.76E-02	2.93E-01
10096	GSM615108	GSE25066	Breast Cancer	Breast	6.77E-08	-3.21E-01	2.64E-03	-3.94E-01
10097	GSM615109	GSE25066	Breast Cancer	Breast	1.25E-01	1.14E-01	9.36E-03	3.49E-01
10098	GSM615110	GSE25066	Breast Cancer	Breast	1.25E-02	1.66E-01	1.20E-03	4.19E-01
10099	GSM615111	GSE25066	Breast Cancer	Breast	9.63E-02	-1.21E-01	3.90E-01	1.57E-01
10100	GSM615112	GSE25066	Breast Cancer	Breast	1.89E-01	1.02E-01	7.74E-02	2.58E-01
10101	GSM615113	GSE25066	Breast Cancer	Breast	1.16E-01	-1.16E-01	2.22E-01	1.98E-01
10102	GSM615114	GSE25066	Breast Cancer	Breast	9.63E-02	-1.21E-01	5.83E-01	1.19E-01
10103	GSM615115	GSE25066	Breast Cancer	Breast	9.63E-02	1.21E-01	4.45E-02	2.85E-01
10104	GSM615116	GSE25066	Breast Cancer	Breast	6.86E-03	1.77E-01	3.19E-04	4.58E-01
10105	GSM615117	GSE25066	Breast Cancer	Breast	4.59E-02	1.39E-01	3.70E-02	2.93E-01
10106	GSM615118	GSE25066	Breast Cancer	Breast	4.05E-08	-3.26E-01	3.20E-02	-3.00E-01
10107	GSM615119	GSE25066	Breast Cancer	Breast	1.65E-06	2.89E-01	7.74E-06	5.54E-01
10108	GSM615120	GSE25066	Breast Cancer	Breast	1.61E-02	-1.61E-01	2.83E-01	-1.82E-01
10109	GSM615121	GSE25066	Breast Cancer	Breast	1.35E-05	-2.65E-01	6.23E-02	-2.69E-01
10110	GSM615122	GSE25066	Breast Cancer	Breast	5.83E-05	-2.47E-01	1.75E-01	-2.13E-01
10111	GSM615123	GSE25066	Breast Cancer	Breast	1.71E-01	1.05E-01	2.61E-02	3.09E-01
10112	GSM615124	GSE25066	Breast Cancer	Breast	2.01E-01	-1.00E-01	1.96E-01	2.06E-01
10113	GSM615125	GSE25066	Breast Cancer	Breast	2.42E-01	-9.42E-02	3.87E-01	1.57E-01
10114	GSM615126	GSE25066	Breast Cancer	Breast	9.54E-03	-1.71E-01	4.36E-01	-1.47E-01
10115	GSM615127	GSE25066	Breast Cancer	Breast	2.80E-01	8.93E-02	1.14E-01	2.38E-01
10116	GSM615128	GSE25066	Breast Cancer	Breast	1.35E-04	2.36E-01	2.28E-04	4.68E-01
10117	GSM615129	GSE25066	Breast Cancer	Breast	1.67E-07	3.13E-01	1.43E-05	5.40E-01
10118	GSM615130	GSE25066	Breast Cancer	Breast	3.04E-03	1.90E-01	1.90E-03	4.04E-01
10119	GSM615131	GSE25066	Breast Cancer	Breast	6.86E-03	1.77E-01	1.98E-03	4.03E-01
10120	GSM615132	GSE25066	Breast Cancer	Breast	6.13E-03	-1.79E-01	3.81E-01	1.59E-01
10121	GSM615133	GSE25066	Breast Cancer	Breast	1.95E-01	1.01E-01	5.53E-02	2.75E-01
10122	GSM615134	GSE25066	Breast Cancer	Breast	5.94E-02	1.33E-01	4.38E-02	2.86E-01
10123	GSM615135	GSE25066	Breast Cancer	Breast	1.49E-01	-1.09E-01	1.73E-01	2.14E-01
10124	GSM615136	GSE25066	Breast Cancer	Breast	3.34E-02	-1.46E-01	4.58E-01	1.43E-01
10125	GSM615137	GSE25066	Breast Cancer	Breast	4.02E-04	2.21E-01	1.98E-03	4.03E-01
10126	GSM615138	GSE25066	Breast Cancer	Breast	1.11E-03	2.06E-01	2.33E-04	4.67E-01
10127	GSM615139	GSE25066	Breast Cancer	Breast	6.84E-06	-2.73E-01	5.45E-02	-2.76E-01
10128	GSM615140	GSE25066	Breast Cancer	Breast	3.50E-02	1.45E-01	5.38E-02	2.76E-01
10129	GSM615141	GSE25066	Breast Cancer	Breast	1.39E-01	1.11E-01	5.45E-02	2.76E-01
10130	GSM615142	GSE25066	Breast Cancer	Breast	2.28E-01	-9.62E-02	2.27E-01	1.97E-01
10131	GSM615143	GSE25066	Breast Cancer	Breast	1.38E-02	1.64E-01	3.92E-03	3.80E-01
10132	GSM615144	GSE25066	Breast Cancer	Breast	5.23E-02	-1.36E-01	3.11E-01	1.75E-01
10133	GSM615145	GSE25066	Breast Cancer	Breast	2.53E-03	-1.93E-01	2.56E-01	-1.89E-01

10134	GSM615146	GSE25066	Breast Cancer	Breast	1.46E-02	1.63E-01	2.44E-04	4.66E-01
10135	GSM615147	GSE25066	Breast Cancer	Breast	3.66E-01	-7.94E-02	1.51E-01	2.22E-01
10136	GSM615148	GSE25066	Breast Cancer	Breast	2.27E-04	2.29E-01	3.26E-04	4.58E-01
10137	GSM615149	GSE25066	Breast Cancer	Breast	1.44E-01	1.10E-01	6.15E-02	2.70E-01
10138	GSM615150	GSE25066	Breast Cancer	Breast	1.70E-02	1.60E-01	3.70E-02	2.93E-01
10139	GSM615151	GSE25066	Breast Cancer	Breast	1.39E-01	1.11E-01	3.76E-02	2.93E-01
10140	GSM615152	GSE25066	Breast Cancer	Breast	8.24E-02	1.25E-01	2.49E-03	3.96E-01
10141	GSM615153	GSE25066	Breast Cancer	Breast	4.63E-01	6.94E-02	2.85E-01	1.81E-01
10142	GSM615154	GSE25066	Breast Cancer	Breast	6.51E-04	2.14E-01	1.50E-03	4.12E-01
10143	GSM615155	GSE25066	Breast Cancer	Breast	1.21E-01	1.15E-01	3.86E-02	2.91E-01
10144	GSM615156	GSE25066	Breast Cancer	Breast	3.67E-02	1.44E-01	4.07E-03	3.79E-01
10145	GSM615157	GSE25066	Breast Cancer	Breast	4.87E-03	1.83E-01	1.42E-02	3.33E-01
10146	GSM615158	GSE25066	Breast Cancer	Breast	2.69E-03	-1.92E-01	2.80E-01	1.82E-01
10147	GSM615159	GSE25066	Breast Cancer	Breast	8.56E-03	1.73E-01	1.16E-02	3.41E-01
10148	GSM615160	GSE25066	Breast Cancer	Breast	1.44E-03	2.02E-01	4.07E-04	4.51E-01
10149	GSM615161	GSE25066	Breast Cancer	Breast	7.60E-02	-1.27E-01	2.85E-01	-1.81E-01
10150	GSM615162	GSE25066	Breast Cancer	Breast	4.73E-01	6.85E-02	1.51E-01	2.22E-01
10151	GSM615163	GSE25066	Breast Cancer	Breast	1.97E-02	-1.57E-01	2.49E-01	-1.90E-01
10152	GSM615164	GSE25066	Breast Cancer	Breast	1.49E-01	1.09E-01	6.39E-02	2.68E-01
10153	GSM615165	GSE25066	Breast Cancer	Breast	1.95E-01	1.01E-01	4.57E-02	2.84E-01
10154	GSM615166	GSE25066	Breast Cancer	Breast	8.24E-02	1.25E-01	6.15E-02	2.70E-01
10155	GSM615167	GSE25066	Breast Cancer	Breast	1.89E-01	1.02E-01	1.89E-02	3.22E-01
10156	GSM615168	GSE25066	Breast Cancer	Breast	2.38E-03	1.94E-01	7.44E-04	4.34E-01
10157	GSM615169	GSE25066	Breast Cancer	Breast	1.34E-11	3.96E-01	1.35E-07	6.43E-01
10158	GSM615170	GSE25066	Breast Cancer	Breast	2.49E-01	-9.33E-02	1.52E-01	2.22E-01
10159	GSM615171	GSE25066	Breast Cancer	Breast	1.48E-15	4.62E-01	3.25E-09	7.14E-01
10160	GSM615172	GSE25066	Breast Cancer	Breast	5.46E-01	6.15E-02	2.22E-01	1.98E-01
10161	GSM615173	GSE25066	Breast Cancer	Breast	2.49E-01	-9.33E-02	2.49E-01	1.90E-01
10162	GSM615174	GSE25066	Breast Cancer	Breast	1.39E-01	-1.11E-01	3.16E-01	1.73E-01
10163	GSM615175	GSE25066	Breast Cancer	Breast	2.49E-10	3.72E-01	4.21E-07	6.19E-01
10164	GSM615176	GSE25066	Breast Cancer	Breast	1.70E-02	1.60E-01	3.11E-02	3.01E-01
10165	GSM615177	GSE25066	Breast Cancer	Breast	3.63E-03	1.88E-01	3.85E-03	3.81E-01
10166	GSM615178	GSE25066	Breast Cancer	Breast	4.87E-03	1.83E-01	7.61E-03	3.57E-01
10167	GSM615179	GSE25066	Breast Cancer	Breast	1.04E-13	-4.33E-01	9.12E-07	-6.03E-01
10168	GSM615180	GSE25066	Breast Cancer	Breast	3.54E-10	-3.69E-01	2.45E-03	-3.96E-01
10169	GSM615181	GSE25066	Breast Cancer	Breast	8.57E-02	1.24E-01	1.00E-01	2.45E-01
10170	GSM615182	GSE25066	Breast Cancer	Breast	2.56E-12	-4.09E-01	3.19E-03	-3.87E-01
10171	GSM615183	GSE25066	Breast Cancer	Breast	1.44E-01	-1.10E-01	3.11E-01	1.75E-01
10172	GSM615184	GSE25066	Breast Cancer	Breast	3.71E-06	2.80E-01	1.04E-05	5.47E-01
10173	GSM615185	GSE25066	Breast Cancer	Breast	2.52E-02	1.52E-01	1.06E-01	2.42E-01
10174	GSM615186	GSE25066	Breast Cancer	Breast	4.44E-07	-3.03E-01	1.52E-01	-2.22E-01
10175	GSM615187	GSE25066	Breast Cancer	Breast	9.59E-11	-3.80E-01	1.46E-05	-5.39E-01
10176	GSM615188	GSE25066	Breast Cancer	Breast	3.34E-05	-2.54E-01	3.11E-02	-3.01E-01
10177	GSM615189	GSE25066	Breast Cancer	Breast	1.08E-01	-1.18E-01	3.11E-01	1.75E-01
10178	GSM615190	GSE25066	Breast Cancer	Breast	4.88E-07	3.02E-01	1.34E-04	4.83E-01
10179	GSM615191	GSE25066	Breast Cancer	Breast	1.44E-01	1.10E-01	4.45E-02	2.85E-01
10180	GSM615192	GSE25066	Breast Cancer	Breast	3.86E-03	-1.87E-01	2.25E-01	-1.97E-01
10181	GSM615193	GSE25066	Breast Cancer	Breast	2.06E-13	4.28E-01	8.79E-10	7.38E-01
10182	GSM615194	GSE25066	Breast Cancer	Breast	6.48E-03	-1.78E-01	3.84E-01	1.58E-01
10183	GSM615195	GSE25066	Breast Cancer	Breast	1.16E-01	1.16E-01	7.36E-02	2.61E-01
10184	GSM615196	GSE25066	Breast Cancer	Breast	1.52E-07	-3.13E-01	9.17E-04	-4.27E-01
10185	GSM615197	GSE25066	Breast Cancer	Breast	7.96E-05	-2.43E-01	2.29E-01	-1.96E-01
10186	GSM615198	GSE25066	Breast Cancer	Breast	7.46E-06	-2.72E-01	4.03E-02	-2.90E-01
10187	GSM615199	GSE25066	Breast Cancer	Breast	4.02E-04	-2.21E-01	1.05E-01	-2.43E-01
10188	GSM615200	GSE25066	Breast Cancer	Breast	4.31E-09	-3.47E-01	9.52E-03	-3.49E-01
10189	GSM615201	GSE25066	Breast Cancer	Breast	4.83E-06	-2.77E-01	4.63E-02	-2.83E-01
10190	GSM615202	GSE25066	Breast Cancer	Breast	5.75E-06	-2.75E-01	1.80E-02	-3.24E-01
10191	GSM615203	GSE25066	Breast Cancer	Breast	5.75E-06	-2.75E-01	1.44E-02	-3.33E-01
10192	GSM615204	GSE25066	Breast Cancer	Breast	3.49E-04	-2.23E-01	1.00E-01	-2.45E-01
10193	GSM615205	GSE25066	Breast Cancer	Breast	1.75E-10	-3.75E-01	1.34E-04	-4.83E-01
10194	GSM615206	GSE25066	Breast Cancer	Breast	1.85E-07	-3.12E-01	1.20E-02	-3.40E-01
10195	GSM615207	GSE25066	Breast Cancer	Breast	8.29E-09	-3.41E-01	1.18E-02	-3.40E-01
10196	GSM615208	GSE25066	Breast Cancer	Breast	5.91E-11	-3.84E-01	2.79E-05	-5.23E-01
10197	GSM615209	GSE25066	Breast Cancer	Breast	1.54E-01	1.08E-01	3.16E-02	3.00E-01
10198	GSM615210	GSE25066	Breast Cancer	Breast	3.62E-11	-3.88E-01	4.07E-04	-4.51E-01
10199	GSM615211	GSE25066	Breast Cancer	Breast	1.04E-01	-1.19E-01	3.90E-01	1.57E-01
10200	GSM615212	GSE25066	Breast Cancer	Breast	4.20E-02	-1.41E-01	4.23E-01	-1.50E-01
10201	GSM615213	GSE25066	Breast Cancer	Breast	3.66E-01	7.94E-02	3.81E-01	1.59E-01
10202	GSM615214	GSE25066	Breast Cancer	Breast	4.05E-06	-2.79E-01	1.00E-01	-2.45E-01
10203	GSM615215	GSE25066	Breast Cancer	Breast	2.56E-12	-4.09E-01	5.25E-03	-3.70E-01
10204	GSM615216	GSE25066	Breast Cancer	Breast	1.58E-09	-3.56E-01	1.25E-03	-4.18E-01
10205	GSM615217	GSE25066	Breast Cancer	Breast	1.44E-03	-2.02E-01	3.11E-01	-1.75E-01
10206	GSM615218	GSE25066	Breast Cancer	Breast	1.38E-10	3.77E-01	4.61E-07	6.17E-01
10207	GSM615219	GSE25066	Breast Cancer	Breast	1.11E-03	-2.06E-01	5.38E-02	-2.76E-01

10208	GSM615220	GSE25066	Breast Cancer	Breast	7.67E-03	-1.75E-01	3.84E-01	-1.58E-01
10209	GSM615221	GSE25066	Breast Cancer	Breast	3.86E-09	-3.48E-01	1.94E-03	-4.04E-01
10210	GSM615222	GSE25066	Breast Cancer	Breast	1.11E-03	2.06E-01	2.49E-03	3.96E-01
10211	GSM615223	GSE25066	Breast Cancer	Breast	1.61E-02	1.61E-01	3.70E-02	2.93E-01
10212	GSM615224	GSE25066	Breast Cancer	Breast	1.65E-01	-1.06E-01	1.96E-01	2.06E-01
10213	GSM615225	GSE25066	Breast Cancer	Breast	9.26E-02	-1.22E-01	2.51E-01	1.90E-01
10214	GSM615226	GSE25066	Breast Cancer	Breast	6.35E-17	4.83E-01	3.48E-09	7.13E-01
10215	GSM615227	GSE25066	Breast Cancer	Breast	1.27E-08	-3.37E-01	3.25E-03	-3.87E-01
10216	GSM615228	GSE25066	Breast Cancer	Breast	3.29E-08	-3.28E-01	5.31E-02	-2.77E-01
10217	GSM615229	GSE25066	Breast Cancer	Breast	6.73E-02	1.30E-01	3.16E-02	3.00E-01
10218	GSM615230	GSE25066	Breast Cancer	Breast	1.82E-04	-2.32E-01	1.98E-01	-2.06E-01
10219	GSM615231	GSE25066	Breast Cancer	Breast	1.11E-03	-2.06E-01	7.46E-02	-2.60E-01
10220	GSM615232	GSE25066	Breast Cancer	Breast	8.52E-04	-2.10E-01	2.49E-01	1.90E-01
10221	GSM615233	GSE25066	Breast Cancer	Breast	5.98E-09	3.44E-01	1.07E-05	5.47E-01
10222	GSM615234	GSE25066	Breast Cancer	Breast	3.08E-05	-2.55E-01	2.02E-01	-2.04E-01
10223	GSM615235	GSE25066	Breast Cancer	Breast	1.96E-14	-4.44E-01	5.43E-06	-5.63E-01
10224	GSM615236	GSE25066	Breast Cancer	Breast	2.21E-11	-3.92E-01	4.07E-04	-4.51E-01
10225	GSM615237	GSE25066	Breast Cancer	Breast	4.05E-06	-2.79E-01	1.49E-02	-3.31E-01
10226	GSM615238	GSE25066	Breast Cancer	Breast	2.01E-01	-1.00E-01	4.45E-02	2.85E-01
10227	GSM615239	GSE25066	Breast Cancer	Breast	3.94E-01	7.64E-02	1.38E-01	2.27E-01
10228	GSM615240	GSE25066	Breast Cancer	Breast	1.30E-01	-1.13E-01	1.32E-01	2.30E-01
10229	GSM615241	GSE25066	Breast Cancer	Breast	3.31E-12	-4.07E-01	1.73E-04	-4.76E-01
10230	GSM615242	GSE25066	Breast Cancer	Breast	1.77E-01	1.04E-01	3.92E-02	2.91E-01
10231	GSM615243	GSE25066	Breast Cancer	Breast	1.14E-08	-3.38E-01	9.52E-03	-3.49E-01
10232	GSM615244	GSE25066	Breast Cancer	Breast	1.11E-03	2.06E-01	2.40E-03	3.97E-01
10233	GSM615245	GSE25066	Breast Cancer	Breast	6.19E-02	-1.32E-01	1.15E-01	-2.37E-01
10234	GSM615246	GSE25066	Breast Cancer	Breast	7.84E-07	-2.97E-01	1.44E-02	-3.33E-01
10235	GSM615247	GSE25066	Breast Cancer	Breast	4.03E-01	-7.54E-02	1.15E-01	2.37E-01
10236	GSM615248	GSE25066	Breast Cancer	Breast	2.64E-02	1.51E-01	1.49E-02	3.31E-01
10237	GSM615249	GSE25066	Breast Cancer	Breast	1.54E-01	-1.08E-01	6.15E-02	2.70E-01
10238	GSM615250	GSE25066	Breast Cancer	Breast	7.36E-05	-2.44E-01	2.58E-01	-1.88E-01
10239	GSM615251	GSE25066	Breast Cancer	Breast	1.60E-05	2.63E-01	5.52E-04	4.43E-01
10240	GSM615252	GSE25066	Breast Cancer	Breast	1.97E-03	-1.97E-01	8.88E-02	-2.51E-01
10241	GSM615253	GSE25066	Breast Cancer	Breast	1.04E-13	-4.33E-01	1.31E-04	-4.83E-01
10242	GSM615254	GSE25066	Breast Cancer	Breast	4.60E-05	-2.50E-01	1.15E-01	-2.37E-01
10243	GSM615255	GSE25066	Breast Cancer	Breast	4.39E-02	1.40E-01	3.76E-02	2.93E-01
10244	GSM615256	GSE25066	Breast Cancer	Breast	3.49E-04	-2.23E-01	2.17E-02	-3.16E-01
10245	GSM615257	GSE25066	Breast Cancer	Breast	4.31E-04	2.20E-01	3.85E-03	3.81E-01
10246	GSM615258	GSE25066	Breast Cancer	Breast	2.24E-03	1.95E-01	2.49E-03	3.96E-01
10247	GSM615259	GSE25066	Breast Cancer	Breast	3.71E-06	-2.80E-01	1.44E-02	-3.33E-01
10248	GSM615260	GSE25066	Breast Cancer	Breast	1.04E-01	-1.19E-01	3.81E-01	1.59E-01
10249	GSM615261	GSE25066	Breast Cancer	Breast	1.85E-07	-3.12E-01	1.49E-02	-3.31E-01
10250	GSM615262	GSE25066	Breast Cancer	Breast	7.67E-03	1.75E-01	1.26E-02	3.38E-01
10251	GSM615263	GSE25066	Breast Cancer	Breast	2.38E-03	-1.94E-01	2.58E-01	-1.88E-01
10252	GSM615264	GSE25066	Breast Cancer	Breast	4.73E-01	6.85E-02	1.01E-01	2.44E-01
10253	GSM615265	GSE25066	Breast Cancer	Breast	2.41E-08	-3.31E-01	1.56E-03	-4.11E-01
10254	GSM615266	GSE25066	Breast Cancer	Breast	3.38E-01	8.23E-02	2.29E-01	1.96E-01
10255	GSM615267	GSE25066	Breast Cancer	Breast	2.37E-06	-2.85E-01	1.51E-02	-3.31E-01
10256	GSM615268	GSE25066	Breast Cancer	Breast	1.53E-03	2.01E-01	6.06E-03	3.65E-01
10257	GSM615269	GSE25066	Breast Cancer	Breast	3.19E-02	1.47E-01	5.16E-03	3.71E-01
10258	GSM615270	GSE25066	Breast Cancer	Breast	3.67E-02	1.44E-01	1.17E-01	2.37E-01
10259	GSM615271	GSE25066	Breast Cancer	Breast	7.50E-08	-3.20E-01	1.80E-02	-3.24E-01
10260	GSM615272	GSE25066	Breast Cancer	Breast	2.16E-06	2.86E-01	4.07E-04	4.51E-01
10261	GSM615273	GSE25066	Breast Cancer	Breast	3.45E-09	-3.49E-01	5.31E-02	-2.77E-01
10262	GSM615274	GSE25066	Breast Cancer	Breast	2.73E-07	-3.08E-01	2.77E-02	-3.06E-01
10263	GSM615275	GSE25066	Breast Cancer	Breast	2.29E-02	-1.54E-01	3.81E-01	1.59E-01
10264	GSM615276	GSE25066	Breast Cancer	Breast	1.30E-01	1.13E-01	1.75E-01	2.13E-01
10265	GSM615277	GSE25066	Breast Cancer	Breast	1.35E-03	-2.03E-01	5.38E-02	-2.76E-01
10266	GSM615278	GSE25066	Breast Cancer	Breast	1.00E-04	-2.40E-01	8.56E-02	-2.53E-01
10267	GSM615279	GSE25066	Breast Cancer	Breast	1.35E-05	-2.65E-01	1.44E-02	-3.33E-01
10268	GSM615280	GSE25066	Breast Cancer	Breast	1.58E-16	-4.77E-01	2.79E-06	-5.78E-01
10269	GSM615281	GSE25066	Breast Cancer	Breast	5.75E-06	-2.75E-01	4.00E-03	-3.80E-01
10270	GSM615282	GSE25066	Breast Cancer	Breast	2.24E-03	-1.95E-01	1.19E-01	-2.36E-01
10271	GSM615283	GSE25066	Breast Cancer	Breast	3.39E-06	-2.81E-01	7.46E-02	-2.60E-01
10272	GSM615284	GSE25066	Breast Cancer	Breast	1.08E-01	1.18E-01	7.61E-03	3.57E-01
10273	GSM615285	GSE25066	Breast Cancer	Breast	1.14E-05	-2.67E-01	3.92E-03	-3.80E-01
10274	GSM615286	GSE25066	Breast Cancer	Breast	3.19E-02	-1.47E-01	4.29E-01	1.49E-01
10275	GSM615287	GSE25066	Breast Cancer	Breast	1.52E-12	4.13E-01	2.20E-10	7.62E-01
10276	GSM615288	GSE25066	Breast Cancer	Breast	5.46E-01	-6.15E-02	2.04E-01	2.04E-01
10277	GSM615289	GSE25066	Breast Cancer	Breast	1.85E-17	-4.91E-01	3.90E-06	-5.70E-01
10278	GSM615290	GSE25066	Breast Cancer	Breast	9.26E-02	1.22E-01	4.03E-02	2.90E-01
10279	GSM615291	GSE25066	Breast Cancer	Breast	1.89E-05	2.61E-01	3.05E-04	4.60E-01
10280	GSM615292	GSE25066	Breast Cancer	Breast	3.25E-04	-2.24E-01	1.40E-01	-2.27E-01
10281	GSM615293	GSE25066	Breast Cancer	Breast	7.36E-05	-2.44E-01	7.55E-02	-2.60E-01

10282	GSM615294	GSE25066	Breast Cancer	Breast	7.97E-04	-2.11E-01	3.84E-01	-1.58E-01
10283	GSM615295	GSE25066	Breast Cancer	Breast	4.55E-14	4.38E-01	1.27E-08	6.89E-01
10284	GSM615296	GSE25066	Breast Cancer	Breast	3.43E-17	-4.87E-01	3.90E-06	-5.70E-01
10285	GSM615297	GSE25066	Breast Cancer	Breast	3.19E-02	-1.47E-01	5.89E-01	-1.17E-01
10286	GSM615298	GSE25066	Breast Cancer	Breast	1.38E-02	1.64E-01	3.11E-02	3.01E-01
10287	GSM615299	GSE25066	Breast Cancer	Breast	1.06E-02	1.69E-01	2.11E-02	3.17E-01
10288	GSM615300	GSE25066	Breast Cancer	Breast	5.83E-05	2.47E-01	1.98E-03	4.03E-01
10289	GSM615301	GSE25066	Breast Cancer	Breast	2.69E-03	1.92E-01	8.02E-03	3.55E-01
10290	GSM615302	GSE25066	Breast Cancer	Breast	3.49E-04	-2.23E-01	1.22E-01	-2.34E-01
10291	GSM615303	GSE25066	Breast Cancer	Breast	2.62E-04	2.27E-01	2.49E-04	4.65E-01
10292	GSM615304	GSE25066	Breast Cancer	Breast	3.39E-06	-2.81E-01	9.52E-03	-3.49E-01
10293	GSM615305	GSE25066	Breast Cancer	Breast	6.73E-02	1.30E-01	4.00E-03	3.80E-01
10294	GSM615306	GSE25066	Breast Cancer	Breast	2.23E-05	2.59E-01	6.39E-03	3.63E-01
10295	GSM615307	GSE25066	Breast Cancer	Breast	5.69E-02	1.34E-01	3.12E-04	4.59E-01
10296	GSM615308	GSE25066	Breast Cancer	Breast	5.75E-06	-2.75E-01	1.20E-02	-3.40E-01
10297	GSM615309	GSE25066	Breast Cancer	Breast	2.05E-05	-2.60E-01	3.86E-02	-2.91E-01
10298	GSM615310	GSE25066	Breast Cancer	Breast	1.53E-02	1.62E-01	2.27E-02	3.14E-01
10299	GSM615311	GSE25066	Breast Cancer	Breast	1.67E-07	-3.13E-01	2.14E-02	-3.17E-01
10300	GSM615312	GSE25066	Breast Cancer	Breast	2.42E-01	9.42E-02	8.66E-02	2.53E-01
10301	GSM615313	GSE25066	Breast Cancer	Breast	7.25E-03	1.76E-01	2.11E-02	3.17E-01
10302	GSM615314	GSE25066	Breast Cancer	Breast	3.94E-01	7.64E-02	8.45E-02	2.54E-01
10303	GSM615315	GSE25066	Breast Cancer	Breast	2.40E-02	-1.53E-01	1.73E-01	2.14E-01
10304	GSM615316	GSE25066	Breast Cancer	Breast	8.57E-02	-1.24E-01	2.51E-01	-1.90E-01
10305	GSM615317	GSE25066	Breast Cancer	Breast	1.81E-06	-2.88E-01	2.49E-03	-3.96E-01
10306	GSM615318	GSE25066	Breast Cancer	Breast	1.25E-02	1.66E-01	1.24E-02	3.38E-01
10307	GSM615319	GSE25066	Breast Cancer	Breast	2.07E-02	1.56E-01	9.17E-04	4.27E-01
10308	GSM615320	GSE25066	Breast Cancer	Breast	4.34E-03	1.85E-01	4.07E-03	3.79E-01
10309	GSM615321	GSE25066	Breast Cancer	Breast	1.88E-02	1.58E-01	6.17E-03	3.64E-01
10310	GSM615322	GSE25066	Breast Cancer	Breast	7.92E-02	1.26E-01	7.55E-02	2.60E-01
10311	GSM615323	GSE25066	Breast Cancer	Breast	6.35E-17	4.83E-01	8.62E-11	7.78E-01
10312	GSM615324	GSE25066	Breast Cancer	Breast	4.01E-02	-1.42E-01	2.51E-01	1.90E-01
10313	GSM615325	GSE25066	Breast Cancer	Breast	1.16E-01	-1.16E-01	1.73E-01	2.14E-01
10314	GSM615326	GSE25066	Breast Cancer	Breast	8.91E-02	1.23E-01	7.46E-02	2.60E-01
10315	GSM615327	GSE25066	Breast Cancer	Breast	1.42E-08	3.36E-01	2.72E-06	5.78E-01
10316	GSM615328	GSE25066	Breast Cancer	Breast	1.79E-02	-1.59E-01	2.25E-01	-1.97E-01
10317	GSM615329	GSE25066	Breast Cancer	Breast	6.51E-04	-2.14E-01	1.34E-01	-2.29E-01
10318	GSM615330	GSE25066	Breast Cancer	Breast	4.87E-03	1.83E-01	3.76E-02	2.93E-01
10319	GSM615331	GSE25066	Breast Cancer	Breast	2.57E-01	-9.23E-02	8.45E-02	2.54E-01
10320	GSM615332	GSE25066	Breast Cancer	Breast	7.30E-02	-1.28E-01	1.52E-01	2.22E-01
10321	GSM615333	GSE25066	Breast Cancer	Breast	9.46E-07	2.95E-01	5.36E-05	5.07E-01
10322	GSM615334	GSE25066	Breast Cancer	Breast	2.14E-01	-9.82E-02	1.51E-01	2.22E-01
10323	GSM615335	GSE25066	Breast Cancer	Breast	6.19E-02	1.32E-01	3.19E-03	3.87E-01
10324	GSM615336	GSE25066	Breast Cancer	Breast	7.46E-06	-2.72E-01	2.61E-02	-3.09E-01
10325	GSM615337	GSE25066	Breast Cancer	Breast	3.93E-05	-2.52E-01	9.52E-03	-3.49E-01
10326	GSM615338	GSE25066	Breast Cancer	Breast	2.14E-01	9.82E-02	7.46E-02	2.60E-01
10327	GSM615339	GSE25066	Breast Cancer	Breast	1.12E-02	-1.68E-01	3.11E-02	-3.01E-01
10328	GSM615340	GSE25066	Breast Cancer	Breast	1.44E-01	-1.10E-01	1.75E-01	-2.13E-01
10329	GSM615341	GSE25066	Breast Cancer	Breast	1.89E-05	-2.61E-01	3.19E-03	-3.87E-01
10330	GSM615342	GSE25066	Breast Cancer	Breast	6.19E-02	-1.32E-01	2.49E-01	1.90E-01
10331	GSM615343	GSE25066	Breast Cancer	Breast	1.71E-01	1.05E-01	1.77E-01	2.13E-01
10332	GSM615344	GSE25066	Breast Cancer	Breast	1.97E-03	1.97E-01	2.61E-02	3.09E-01
10333	GSM615345	GSE25066	Breast Cancer	Breast	8.10E-03	-1.74E-01	1.75E-01	-2.13E-01
10334	GSM615346	GSE25066	Breast Cancer	Breast	1.24E-07	3.15E-01	4.01E-06	5.70E-01
10335	GSM615347	GSE25066	Breast Cancer	Breast	7.25E-03	-1.76E-01	2.51E-01	1.90E-01
10336	GSM615348	GSE25066	Breast Cancer	Breast	1.44E-01	-1.10E-01	2.80E-01	1.82E-01
10337	GSM615349	GSE25066	Breast Cancer	Breast	4.98E-08	-3.24E-01	9.36E-03	-3.49E-01
10338	GSM615350	GSE25066	Breast Cancer	Breast	4.49E-08	-3.25E-01	3.92E-03	-3.80E-01
10339	GSM615351	GSE25066	Breast Cancer	Breast	1.52E-11	-3.95E-01	3.05E-04	-4.60E-01
10340	GSM615352	GSE25066	Breast Cancer	Breast	9.54E-03	-1.71E-01	1.75E-01	-2.13E-01
10341	GSM615353	GSE25066	Breast Cancer	Breast	4.81E-09	-3.46E-01	2.33E-04	-4.67E-01
10342	GSM615354	GSE25066	Breast Cancer	Breast	4.60E-03	-1.84E-01	1.54E-01	-2.21E-01
10343	GSM615355	GSE25066	Breast Cancer	Breast	5.69E-02	-1.34E-01	3.11E-01	1.75E-01
10344	GSM615356	GSE25066	Breast Cancer	Breast	2.91E-02	-1.49E-01	1.51E-01	2.22E-01
10345	GSM615357	GSE25066	Breast Cancer	Breast	4.39E-02	-1.40E-01	1.14E-01	2.38E-01
10346	GSM615358	GSE25066	Breast Cancer	Breast	1.70E-02	-1.60E-01	2.54E-01	-1.89E-01
10347	GSM615359	GSE25066	Breast Cancer	Breast	2.21E-01	9.72E-02	1.32E-01	2.30E-01
10348	GSM615360	GSE25066	Breast Cancer	Breast	4.79E-02	-1.38E-01	1.52E-01	-2.22E-01
10349	GSM615361	GSE25066	Breast Cancer	Breast	5.04E-01	-6.55E-02	3.11E-01	1.75E-01
10350	GSM615362	GSE25066	Breast Cancer	Breast	6.33E-10	-3.64E-01	2.45E-03	-3.96E-01
10351	GSM615363	GSE25066	Breast Cancer	Breast	2.07E-02	1.56E-01	7.75E-03	3.56E-01
10352	GSM615364	GSE25066	Breast Cancer	Breast	4.39E-02	1.40E-01	1.46E-02	3.32E-01
10353	GSM615365	GSE25066	Breast Cancer	Breast	1.35E-04	-2.36E-01	1.83E-02	-3.23E-01
10354	GSM615366	GSE25066	Breast Cancer	Breast	1.08E-04	-2.39E-01	1.34E-01	-2.29E-01
10355	GSM615367	GSE25066	Breast Cancer	Breast	1.14E-06	-2.93E-01	3.30E-02	-2.98E-01

10356	GSM615368	GSE25066	Breast Cancer	Breast	5.01E-02	-1.37E-01	3.46E-01	1.66E-01
10357	GSM615369	GSE25066	Breast Cancer	Breast	2.88E-01	8.83E-02	2.17E-02	3.16E-01
10358	GSM615370	GSE25066	Breast Cancer	Breast	1.96E-04	-2.31E-01	8.56E-02	-2.53E-01
10359	GSM615371	GSE25066	Breast Cancer	Breast	2.64E-02	1.51E-01	5.24E-02	2.77E-01
10360	GSM615372	GSE25066	Breast Cancer	Breast	7.67E-03	-1.75E-01	3.16E-01	-1.73E-01
10361	GSM615373	GSE25066	Breast Cancer	Breast	1.08E-04	2.39E-01	1.22E-03	4.18E-01
10362	GSM615374	GSE25066	Breast Cancer	Breast	2.23E-05	-2.59E-01	1.20E-02	-3.40E-01
10363	GSM615375	GSE25066	Breast Cancer	Breast	1.44E-03	-2.02E-01	1.84E-01	-2.10E-01
10364	GSM615376	GSE25066	Breast Cancer	Breast	3.67E-02	-1.44E-01	2.51E-01	1.90E-01
10365	GSM615377	GSE25066	Breast Cancer	Breast	4.87E-03	-1.83E-01	5.31E-02	-2.77E-01
10366	GSM615378	GSE25066	Breast Cancer	Breast	6.46E-02	-1.31E-01	1.00E-01	2.45E-01
10367	GSM615379	GSE25066	Breast Cancer	Breast	1.34E-01	1.12E-01	2.17E-02	3.16E-01
10368	GSM615380	GSE25066	Breast Cancer	Breast	2.77E-02	-1.50E-01	4.29E-01	1.49E-01
10369	GSM615381	GSE25066	Breast Cancer	Breast	1.34E-01	-1.12E-01	2.22E-01	1.98E-01
10370	GSM615382	GSE25066	Breast Cancer	Breast	7.14E-07	2.98E-01	3.26E-04	4.58E-01
10371	GSM615383	GSE25066	Breast Cancer	Breast	1.12E-07	-3.16E-01	1.77E-02	-3.24E-01
10372	GSM615384	GSE25066	Breast Cancer	Breast	2.57E-01	9.23E-02	1.52E-01	2.22E-01
10373	GSM615385	GSE25066	Breast Cancer	Breast	6.48E-03	1.78E-01	6.17E-03	3.64E-01
10374	GSM615386	GSE25066	Breast Cancer	Breast	2.27E-04	2.29E-01	7.88E-03	3.56E-01
10375	GSM615387	GSE25066	Breast Cancer	Breast	7.25E-03	-1.76E-01	2.04E-01	-2.04E-01
10376	GSM615388	GSE25066	Breast Cancer	Breast	2.42E-05	-2.58E-01	1.83E-02	-3.23E-01
10377	GSM615389	GSE25066	Breast Cancer	Breast	2.64E-01	-9.13E-02	1.37E-01	2.28E-01
10378	GSM615390	GSE25066	Breast Cancer	Breast	2.42E-01	-9.42E-02	1.96E-01	2.06E-01
10379	GSM615391	GSE25066	Breast Cancer	Breast	1.12E-01	1.17E-01	5.45E-02	2.76E-01
10380	GSM615392	GSE25066	Breast Cancer	Breast	1.46E-04	-2.35E-01	4.57E-02	-2.84E-01
10381	GSM615393	GSE25066	Breast Cancer	Breast	2.35E-01	-9.52E-02	1.35E-01	2.29E-01
10382	GSM615394	GSE25066	Breast Cancer	Breast	2.88E-01	-8.83E-02	2.56E-01	1.89E-01
10383	GSM615395	GSE25066	Breast Cancer	Breast	2.07E-02	1.56E-01	1.80E-02	3.24E-01
10384	GSM615396	GSE25066	Breast Cancer	Breast	2.29E-02	-1.54E-01	1.79E-01	-2.12E-01
10385	GSM615397	GSE25066	Breast Cancer	Breast	2.77E-02	1.50E-01	5.38E-02	2.76E-01
10386	GSM615398	GSE25066	Breast Cancer	Breast	2.77E-02	1.50E-01	2.20E-02	3.16E-01
10387	GSM615399	GSE25066	Breast Cancer	Breast	1.53E-02	1.62E-01	7.27E-02	2.62E-01
10388	GSM615400	GSE25066	Breast Cancer	Breast	4.31E-09	-3.47E-01	1.51E-02	-3.31E-01
10389	GSM615401	GSE25066	Breast Cancer	Breast	5.23E-11	-3.85E-01	1.17E-03	-4.20E-01
10390	GSM615402	GSE25066	Breast Cancer	Breast	5.53E-12	-4.03E-01	1.50E-03	-4.12E-01
10391	GSM615403	GSE25066	Breast Cancer	Breast	1.44E-01	1.10E-01	8.88E-02	2.51E-01
10392	GSM615404	GSE25066	Breast Cancer	Breast	3.62E-11	-3.88E-01	1.94E-03	-4.04E-01
10393	GSM615405	GSE25066	Breast Cancer	Breast	1.85E-07	-3.12E-01	1.53E-03	-4.11E-01
10394	GSM615631	GSE25066	Breast Cancer	Breast	1.38E-02	1.64E-01	5.07E-03	3.71E-01
10395	GSM615632	GSE25066	Breast Cancer	Breast	1.18E-02	1.67E-01	1.20E-03	4.19E-01
10396	GSM615633	GSE25066	Breast Cancer	Breast	1.08E-04	-2.39E-01	1.00E-01	-2.45E-01
10397	GSM615634	GSE25066	Breast Cancer	Breast	8.59E-05	-2.42E-01	2.22E-01	1.98E-01
10398	GSM615635	GSE25066	Breast Cancer	Breast	1.04E-01	-1.19E-01	2.25E-01	-1.97E-01
10399	GSM615636	GSE25066	Breast Cancer	Breast	1.04E-01	-1.19E-01	4.58E-01	1.43E-01
10400	GSM615637	GSE25066	Breast Cancer	Breast	2.88E-01	-8.83E-02	1.79E-01	2.12E-01
10401	GSM615638	GSE25066	Breast Cancer	Breast	5.46E-02	1.35E-01	1.98E-01	2.06E-01
10402	GSM615639	GSE25066	Breast Cancer	Breast	9.63E-02	-1.21E-01	1.98E-01	2.06E-01
10403	GSM615640	GSE25066	Breast Cancer	Breast	8.57E-02	1.24E-01	1.35E-01	2.29E-01
10404	GSM615641	GSE25066	Breast Cancer	Breast	1.57E-04	-2.34E-01	6.31E-02	-2.69E-01
10405	GSM615642	GSE25066	Breast Cancer	Breast	7.11E-10	-3.63E-01	9.36E-04	-4.27E-01
10406	GSM615643	GSE25066	Breast Cancer	Breast	2.82E-04	2.26E-01	5.40E-04	4.43E-01
10407	GSM615644	GSE25066	Breast Cancer	Breast	4.34E-03	-1.85E-01	3.81E-01	1.59E-01
10408	GSM615645	GSE25066	Breast Cancer	Breast	3.53E-13	-4.24E-01	7.19E-05	-4.99E-01
10409	GSM615646	GSE25066	Breast Cancer	Breast	1.16E-04	-2.38E-01	6.39E-02	-2.68E-01
10410	GSM615647	GSE25066	Breast Cancer	Breast	2.37E-06	-2.85E-01	1.44E-02	-3.33E-01
10411	GSM615648	GSE25066	Breast Cancer	Breast	3.34E-02	-1.46E-01	2.54E-01	-1.89E-01
10412	GSM615649	GSE25066	Breast Cancer	Breast	1.97E-03	-1.97E-01	1.19E-01	-2.36E-01
10413	GSM615650	GSE25066	Breast Cancer	Breast	2.86E-03	1.91E-01	2.54E-03	3.95E-01
10414	GSM615651	GSE25066	Breast Cancer	Breast	6.67E-09	-3.43E-01	1.00E-01	-2.45E-01
10415	GSM615652	GSE25066	Breast Cancer	Breast	5.30E-04	-2.17E-01	8.77E-02	-2.52E-01
10416	GSM615653	GSE25066	Breast Cancer	Breast	5.39E-05	-2.48E-01	8.56E-02	-2.53E-01
10417	GSM615654	GSE25066	Breast Cancer	Breast	1.35E-05	-2.65E-01	3.11E-02	-3.01E-01
10418	GSM615655	GSE25066	Breast Cancer	Breast	1.16E-04	-2.38E-01	1.19E-01	-2.36E-01
10419	GSM615656	GSE25066	Breast Cancer	Breast	1.08E-01	1.18E-01	1.38E-01	2.27E-01
10420	GSM615657	GSE25066	Breast Cancer	Breast	6.28E-06	2.74E-01	9.76E-04	4.25E-01
10421	GSM615658	GSE25066	Breast Cancer	Breast	6.19E-02	1.32E-01	1.18E-02	3.40E-01
10422	GSM615659	GSE25066	Breast Cancer	Breast	1.04E-03	-2.07E-01	8.56E-02	-2.53E-01
10423	GSM615660	GSE25066	Breast Cancer	Breast	1.00E-04	-2.40E-01	1.18E-01	-2.36E-01
10424	GSM615661	GSE25066	Breast Cancer	Breast	5.57E-01	-6.05E-02	3.55E-01	1.64E-01
10425	GSM615662	GSE25066	Breast Cancer	Breast	1.25E-01	-1.14E-01	3.81E-01	1.59E-01
10426	GSM615663	GSE25066	Breast Cancer	Breast	1.79E-02	1.59E-01	4.70E-02	2.83E-01
10427	GSM615664	GSE25066	Breast Cancer	Breast	1.27E-08	-3.37E-01	5.07E-03	-3.71E-01
10428	GSM615665	GSE25066	Breast Cancer	Breast	9.27E-05	-2.41E-01	1.21E-01	-2.35E-01
10429	GSM615666	GSE25066	Breast Cancer	Breast	3.34E-02	-1.46E-01	2.29E-01	-1.96E-01

10430	GSM615667	GSE25066	Breast Cancer	Breast	2.40E-02	1.53E-01	8.02E-03	3.55E-01
10431	GSM615668	GSE25066	Breast Cancer	Breast	1.06E-02	-1.69E-01	3.87E-01	-1.57E-01
10432	GSM615669	GSE25066	Breast Cancer	Breast	2.23E-05	-2.59E-01	1.38E-01	-2.27E-01
10433	GSM615670	GSE25066	Breast Cancer	Breast	4.87E-03	-1.83E-01	2.58E-01	-1.88E-01
10434	GSM615671	GSE25066	Breast Cancer	Breast	6.48E-03	1.78E-01	7.13E-04	4.35E-01
10435	GSM615672	GSE25066	Breast Cancer	Breast	2.49E-01	9.33E-02	1.54E-01	2.21E-01
10436	GSM615673	GSE25066	Breast Cancer	Breast	1.26E-09	-3.58E-01	2.45E-03	-3.96E-01
10437	GSM615674	GSE25066	Breast Cancer	Breast	3.50E-02	1.45E-01	1.20E-03	4.19E-01
10438	GSM615675	GSE25066	Breast Cancer	Breast	1.60E-05	-2.63E-01	2.64E-02	-3.08E-01
10439	GSM615676	GSE25066	Breast Cancer	Breast	4.39E-02	-1.40E-01	2.22E-01	1.98E-01
10440	GSM615677	GSE25066	Breast Cancer	Breast	1.35E-03	2.03E-01	1.15E-03	4.20E-01
10441	GSM615678	GSE25066	Breast Cancer	Breast	3.93E-05	-2.52E-01	3.76E-02	-2.93E-01
10442	GSM615679	GSE25066	Breast Cancer	Breast	8.94E-10	-3.61E-01	4.15E-03	-3.78E-01
10443	GSM615680	GSE25066	Breast Cancer	Breast	2.53E-03	-1.93E-01	8.77E-02	-2.52E-01
10444	GSM615681	GSE25066	Breast Cancer	Breast	4.59E-02	-1.39E-01	5.00E-01	1.35E-01
10445	GSM615682	GSE25066	Breast Cancer	Breast	5.91E-07	-3.00E-01	8.88E-02	-2.51E-01
10446	GSM615683	GSE25066	Breast Cancer	Breast	6.68E-11	-3.83E-01	3.92E-03	-3.80E-01
10447	GSM615684	GSE25066	Breast Cancer	Breast	9.27E-05	-2.41E-01	2.51E-01	-1.90E-01
10448	GSM615685	GSE25066	Breast Cancer	Breast	1.98E-06	-2.87E-01	6.17E-03	-3.64E-01
10449	GSM615686	GSE25066	Breast Cancer	Breast	1.49E-01	-1.09E-01	3.11E-01	1.75E-01
10450	GSM615687	GSE25066	Breast Cancer	Breast	4.73E-01	-6.85E-02	1.77E-01	2.13E-01
10451	GSM615688	GSE25066	Breast Cancer	Breast	8.61E-07	-2.96E-01	1.56E-03	-4.11E-01
10452	GSM615689	GSE25066	Breast Cancer	Breast	3.04E-03	-1.90E-01	1.00E-01	-2.45E-01
10453	GSM615690	GSE25066	Breast Cancer	Breast	9.73E-04	-2.08E-01	8.56E-02	-2.53E-01
10454	GSM615691	GSE25066	Breast Cancer	Breast	4.79E-02	-1.38E-01	2.58E-01	1.88E-01
10455	GSM615692	GSE25066	Breast Cancer	Breast	6.19E-02	1.32E-01	1.75E-02	3.25E-01
10456	GSM615693	GSE25066	Breast Cancer	Breast	8.56E-03	-1.73E-01	3.11E-01	1.75E-01
10457	GSM615694	GSE25066	Breast Cancer	Breast	8.24E-02	-1.25E-01	2.54E-01	1.89E-01
10458	GSM615695	GSE25066	Breast Cancer	Breast	3.29E-08	-3.28E-01	2.68E-02	-3.07E-01
10459	GSM615696	GSE25066	Breast Cancer	Breast	1.63E-03	2.00E-01	2.28E-04	4.68E-01
10460	GSM615697	GSE25066	Breast Cancer	Breast	1.89E-05	-2.61E-01	1.17E-03	-4.20E-01
10461	GSM615698	GSE25066	Breast Cancer	Breast	1.25E-02	-1.66E-01	2.00E-01	-2.05E-01
10462	GSM615699	GSE25066	Breast Cancer	Breast	3.84E-02	-1.43E-01	4.26E-01	1.49E-01
10463	GSM615700	GSE25066	Breast Cancer	Breast	1.37E-06	-2.91E-01	1.44E-02	-3.33E-01
10464	GSM615701	GSE25066	Breast Cancer	Breast	1.46E-02	-1.63E-01	2.85E-01	-1.81E-01
10465	GSM615702	GSE25066	Breast Cancer	Breast	2.84E-05	-2.56E-01	3.76E-02	-2.93E-01
10466	GSM615703	GSE25066	Breast Cancer	Breast	3.49E-04	-2.23E-01	2.24E-02	-3.15E-01
10467	GSM615704	GSE25066	Breast Cancer	Breast	1.16E-01	1.16E-01	6.23E-02	2.69E-01
10468	GSM615705	GSE25066	Breast Cancer	Breast	1.46E-02	-1.63E-01	1.35E-01	-2.29E-01
10469	GSM615706	GSE25066	Breast Cancer	Breast	7.60E-02	1.27E-01	1.26E-02	3.38E-01
10470	GSM615707	GSE25066	Breast Cancer	Breast	1.97E-03	-1.97E-01	1.75E-01	-2.13E-01
10471	GSM615708	GSE25066	Breast Cancer	Breast	5.68E-04	-2.16E-01	5.38E-02	-2.76E-01
10472	GSM615709	GSE25066	Breast Cancer	Breast	2.73E-07	-3.08E-01	3.92E-03	-3.80E-01
10473	GSM615710	GSE25066	Breast Cancer	Breast	1.60E-01	-1.07E-01	1.35E-01	2.29E-01
10474	GSM615711	GSE25066	Breast Cancer	Breast	6.08E-04	-2.15E-01	1.44E-02	-3.33E-01
10475	GSM615712	GSE25066	Breast Cancer	Breast	1.31E-02	-1.65E-01	1.34E-01	-2.29E-01
10476	GSM615713	GSE25066	Breast Cancer	Breast	1.26E-03	-2.04E-01	1.20E-02	-3.40E-01
10477	GSM615714	GSE25066	Breast Cancer	Breast	9.54E-03	1.71E-01	9.90E-02	2.46E-01
10478	GSM615715	GSE25066	Breast Cancer	Breast	1.89E-01	1.02E-01	1.17E-01	2.37E-01
10479	GSM615716	GSE25066	Breast Cancer	Breast	1.00E-01	-1.20E-01	2.85E-01	1.81E-01
10480	GSM615717	GSE25066	Breast Cancer	Breast	2.77E-02	1.50E-01	1.94E-03	4.04E-01
10481	GSM615718	GSE25066	Breast Cancer	Breast	3.39E-06	-2.81E-01	2.20E-02	-3.16E-01
10482	GSM615719	GSE25066	Breast Cancer	Breast	3.04E-01	-8.63E-02	1.14E-01	2.38E-01
10483	GSM615720	GSE25066	Breast Cancer	Breast	2.44E-04	-2.28E-01	8.02E-03	-3.55E-01
10484	GSM615721	GSE25066	Breast Cancer	Breast	1.74E-05	-2.62E-01	2.45E-03	-3.96E-01
10485	GSM615722	GSE25066	Breast Cancer	Breast	2.64E-02	-1.51E-01	3.19E-01	-1.73E-01
10486	GSM615723	GSE25066	Breast Cancer	Breast	2.21E-09	-3.53E-01	5.28E-04	-4.44E-01
10487	GSM615724	GSE25066	Breast Cancer	Breast	8.57E-02	1.24E-01	3.70E-02	2.93E-01
10488	GSM615725	GSE25066	Breast Cancer	Breast	7.36E-05	-2.44E-01	4.45E-02	-2.85E-01
10489	GSM615726	GSE25066	Breast Cancer	Breast	5.53E-12	-4.03E-01	5.52E-04	-4.43E-01
10490	GSM615727	GSE25066	Breast Cancer	Breast	1.54E-01	1.08E-01	1.32E-01	2.30E-01
10491	GSM615728	GSE25066	Breast Cancer	Breast	7.92E-02	-1.26E-01	2.22E-01	1.98E-01
10492	GSM615729	GSE25066	Breast Cancer	Breast	8.91E-02	1.23E-01	1.96E-01	2.06E-01
10493	GSM615730	GSE25066	Breast Cancer	Breast	2.91E-02	1.49E-01	1.54E-01	2.21E-01
10494	GSM615731	GSE25066	Breast Cancer	Breast	2.91E-02	-1.49E-01	1.14E-01	2.38E-01
10495	GSM615732	GSE25066	Breast Cancer	Breast	1.44E-01	1.10E-01	2.51E-01	1.90E-01
10496	GSM615733	GSE25066	Breast Cancer	Breast	1.21E-01	-1.15E-01	3.90E-01	1.57E-01
10497	GSM615734	GSE25066	Breast Cancer	Breast	1.82E-04	-2.32E-01	1.57E-01	-2.20E-01
10498	GSM615735	GSE25066	Breast Cancer	Breast	6.51E-04	-2.14E-01	2.14E-02	-3.17E-01
10499	GSM615736	GSE25066	Breast Cancer	Breast	7.46E-06	-2.72E-01	6.56E-02	-2.67E-01
10500	GSM615737	GSE25066	Breast Cancer	Breast	2.08E-01	-9.92E-02	8.66E-02	2.53E-01
10501	GSM615738	GSE25066	Breast Cancer	Breast	1.24E-07	-3.15E-01	1.46E-02	-3.32E-01
10502	GSM615739	GSE25066	Breast Cancer	Breast	4.23E-01	7.34E-02	3.11E-01	1.75E-01
10503	GSM615740	GSE25066	Breast Cancer	Breast	2.28E-01	9.62E-02	1.32E-01	2.30E-01

10504	GSM615741	GSE25066	Breast Cancer	Breast	3.21E-01	8.43E-02	1.80E-01	2.11E-01
10505	GSM615742	GSE25066	Breast Cancer	Breast	1.77E-09	3.55E-01	3.21E-07	6.25E-01
10506	GSM615743	GSE25066	Breast Cancer	Breast	2.84E-05	-2.56E-01	4.45E-02	-2.85E-01
10507	GSM615744	GSE25066	Breast Cancer	Breast	5.27E-06	2.76E-01	1.31E-04	4.83E-01
10508	GSM615745	GSE25066	Breast Cancer	Breast	7.30E-02	-1.28E-01	2.22E-01	1.98E-01
10509	GSM615746	GSE25066	Breast Cancer	Breast	2.28E-01	-9.62E-02	2.49E-01	1.90E-01
10510	GSM615747	GSE25066	Breast Cancer	Breast	4.43E-01	-7.14E-02	1.73E-01	2.14E-01
10511	GSM615748	GSE25066	Breast Cancer	Breast	6.19E-02	1.32E-01	1.46E-02	3.32E-01
10512	GSM615749	GSE25066	Breast Cancer	Breast	9.18E-08	-3.18E-01	1.46E-02	-3.32E-01
10513	GSM615750	GSE25066	Breast Cancer	Breast	6.49E-07	-2.99E-01	6.17E-03	-3.64E-01
10514	GSM615751	GSE25066	Breast Cancer	Breast	2.86E-03	-1.91E-01	1.35E-01	-2.29E-01
10515	GSM615752	GSE25066	Breast Cancer	Breast	6.97E-04	-2.13E-01	7.55E-02	-2.60E-01
10516	GSM615753	GSE25066	Breast Cancer	Breast	9.18E-08	-3.18E-01	1.44E-02	-3.33E-01
10517	GSM615754	GSE25066	Breast Cancer	Breast	1.81E-06	-2.88E-01	3.86E-02	-2.91E-01
10518	GSM615755	GSE25066	Breast Cancer	Breast	8.59E-05	-2.42E-01	4.00E-03	-3.80E-01
10519	GSM615756	GSE25066	Breast Cancer	Breast	2.62E-04	-2.27E-01	5.60E-02	-2.74E-01
10520	GSM615757	GSE25066	Breast Cancer	Breast	3.21E-01	-8.43E-02	7.27E-02	2.62E-01
10521	GSM615758	GSE25066	Breast Cancer	Breast	1.39E-01	-1.11E-01	2.49E-01	1.90E-01
10522	GSM615759	GSE25066	Breast Cancer	Breast	1.82E-04	-2.32E-01	4.45E-02	-2.85E-01
10523	GSM615760	GSE25066	Breast Cancer	Breast	4.25E-05	-2.51E-01	8.99E-02	-2.51E-01
10524	GSM615761	GSE25066	Breast Cancer	Breast	1.46E-04	2.35E-01	2.49E-03	3.96E-01
10525	GSM615762	GSE25066	Breast Cancer	Breast	6.43E-01	-5.26E-02	7.36E-02	2.61E-01
10526	GSM615763	GSE25066	Breast Cancer	Breast	3.38E-01	-8.23E-02	8.45E-02	2.54E-01
10527	GSM615764	GSE25066	Breast Cancer	Breast	2.38E-03	1.94E-01	5.25E-03	3.70E-01
10528	GSM615765	GSE25066	Breast Cancer	Breast	4.05E-06	2.79E-01	9.83E-05	4.91E-01
10529	GSM615766	GSE25066	Breast Cancer	Breast	1.35E-05	2.65E-01	5.36E-05	5.07E-01
10530	GSM615767	GSE25066	Breast Cancer	Breast	1.04E-06	2.94E-01	2.33E-04	4.67E-01
10531	GSM615768	GSE25066	Breast Cancer	Breast	3.84E-01	-7.74E-02	3.87E-01	1.57E-01
10532	GSM615769	GSE25066	Breast Cancer	Breast	1.12E-07	3.16E-01	1.29E-06	5.95E-01
10533	GSM615770	GSE25066	Breast Cancer	Breast	2.64E-02	1.51E-01	1.16E-02	3.41E-01
10534	GSM615771	GSE25066	Breast Cancer	Breast	9.26E-02	1.22E-01	1.77E-02	3.24E-01
10535	GSM615772	GSE25066	Breast Cancer	Breast	2.11E-04	2.30E-01	8.98E-04	4.28E-01
10536	GSM615773	GSE25066	Breast Cancer	Breast	6.33E-10	3.64E-01	6.13E-08	6.58E-01
10537	GSM615774	GSE25066	Breast Cancer	Breast	2.44E-04	-2.28E-01	1.77E-01	-2.13E-01
10538	GSM615775	GSE25066	Breast Cancer	Breast	4.79E-02	-1.38E-01	2.49E-01	1.90E-01
10539	GSM615776	GSE25066	Breast Cancer	Breast	1.35E-03	2.03E-01	4.89E-03	3.73E-01
10540	GSM615777	GSE25066	Breast Cancer	Breast	1.53E-02	1.62E-01	6.06E-03	3.65E-01
10541	GSM615778	GSE25066	Breast Cancer	Breast	2.14E-01	-9.82E-02	2.51E-01	1.90E-01
10542	GSM615779	GSE25066	Breast Cancer	Breast	5.01E-02	1.37E-01	7.28E-04	4.34E-01
10543	GSM615780	GSE25066	Breast Cancer	Breast	7.72E-22	5.52E-01	1.75E-12	8.41E-01
10544	GSM615781	GSE25066	Breast Cancer	Breast	1.01E-02	1.70E-01	4.98E-03	3.72E-01
10545	GSM615782	GSE25066	Breast Cancer	Breast	3.10E-21	5.44E-01	2.28E-10	7.61E-01
10546	GSM615783	GSE25066	Breast Cancer	Breast	4.03E-01	7.54E-02	3.70E-02	2.93E-01
10547	GSM615784	GSE25066	Breast Cancer	Breast	9.18E-08	3.18E-01	1.43E-05	5.40E-01
10548	GSM615785	GSE25066	Breast Cancer	Breast	3.74E-04	-2.22E-01	1.03E-01	-2.44E-01
10549	GSM615786	GSE25066	Breast Cancer	Breast	9.04E-03	1.72E-01	2.64E-03	3.94E-01
10550	GSM615787	GSE25066	Breast Cancer	Breast	1.61E-02	1.61E-01	7.59E-04	4.33E-01
10551	GSM615788	GSE25066	Breast Cancer	Breast	2.64E-02	-1.51E-01	3.84E-01	-1.58E-01
10552	GSM615789	GSE25066	Breast Cancer	Breast	1.38E-10	-3.77E-01	2.45E-03	-3.96E-01
10553	GSM615790	GSE25066	Breast Cancer	Breast	2.77E-02	-1.50E-01	2.49E-01	1.90E-01
10554	GSM615791	GSE25066	Breast Cancer	Breast	7.13E-12	-4.01E-01	6.98E-04	-4.36E-01
10555	GSM615792	GSE25066	Breast Cancer	Breast	3.06E-15	-4.57E-01	1.34E-04	-4.83E-01
10556	GSM615793	GSE25066	Breast Cancer	Breast	2.04E-07	-3.11E-01	4.07E-04	-4.51E-01
10557	GSM615794	GSE25066	Breast Cancer	Breast	5.91E-11	3.84E-01	1.54E-05	5.38E-01
10558	GSM615795	GSE25066	Breast Cancer	Breast	3.30E-01	8.33E-02	6.39E-02	2.68E-01
10559	GSM615796	GSE25066	Breast Cancer	Breast	1.05E-05	-2.68E-01	5.53E-02	-2.75E-01
10560	GSM615797	GSE25066	Breast Cancer	Breast	6.30E-05	-2.46E-01	7.75E-03	-3.56E-01
10561	GSM615798	GSE25066	Breast Cancer	Breast	1.11E-03	-2.06E-01	4.70E-01	-1.40E-01
10562	GSM615799	GSE25066	Breast Cancer	Breast	2.73E-07	-3.08E-01	2.61E-02	-3.09E-01
10563	GSM615800	GSE25066	Breast Cancer	Breast	4.62E-04	2.19E-01	3.20E-02	3.00E-01
10564	GSM615801	GSE25066	Breast Cancer	Breast	3.19E-02	-1.47E-01	1.35E-01	-2.29E-01
10565	GSM615802	GSE25066	Breast Cancer	Breast	4.25E-05	-2.51E-01	1.98E-01	-2.06E-01
10566	GSM615803	GSE25066	Breast Cancer	Breast	6.12E-08	-3.22E-01	9.10E-02	-2.50E-01
10567	GSM615804	GSE25066	Breast Cancer	Breast	3.01E-07	3.07E-01	1.85E-04	4.74E-01
10568	GSM615805	GSE25066	Breast Cancer	Breast	2.28E-01	9.62E-02	5.07E-03	3.71E-01
10569	GSM615806	GSE25066	Breast Cancer	Breast	1.77E-01	-1.04E-01	1.98E-01	2.06E-01
10570	GSM615807	GSE25066	Breast Cancer	Breast	1.97E-02	-1.57E-01	3.81E-01	1.59E-01
10571	GSM615808	GSE25066	Breast Cancer	Breast	1.42E-08	-3.36E-01	2.54E-03	-3.95E-01
10572	GSM615809	GSE25066	Breast Cancer	Breast	7.14E-07	-2.98E-01	1.83E-02	-3.23E-01
10573	GSM615810	GSE25066	Breast Cancer	Breast	5.91E-07	-3.00E-01	1.51E-02	-3.31E-01
10574	GSM615811	GSE25066	Breast Cancer	Breast	2.28E-01	-9.62E-02	2.27E-01	1.97E-01
10575	GSM615812	GSE25066	Breast Cancer	Breast	8.91E-02	-1.23E-01	2.49E-01	1.90E-01
10576	GSM615813	GSE25066	Breast Cancer	Breast	9.08E-14	4.34E-01	4.30E-08	6.65E-01
10577	GSM615814	GSE25066	Breast Cancer	Breast	9.65E-06	-2.69E-01	6.23E-02	-2.69E-01

10578	GSM615815	GSE25066	Breast Cancer	Breast	2.56E-12	4.09E-01	8.16E-06	5.53E-01
10579	GSM615816	GSE25066	Breast Cancer	Breast	4.02E-19	5.15E-01	2.37E-10	7.61E-01
10580	GSM615817	GSE25066	Breast Cancer	Breast	5.64E-10	3.65E-01	7.33E-06	5.56E-01
10581	GSM615818	GSE25066	Breast Cancer	Breast	2.53E-03	-1.93E-01	6.48E-02	-2.67E-01
10582	GSM615819	GSE25066	Breast Cancer	Breast	5.23E-02	1.36E-01	2.11E-02	3.17E-01
10583	GSM615820	GSE25066	Breast Cancer	Breast	7.96E-05	2.43E-01	1.49E-02	3.31E-01
10584	GSM615821	GSE25066	Breast Cancer	Breast	8.56E-03	1.73E-01	2.11E-02	3.17E-01
10585	GSM615822	GSE25066	Breast Cancer	Breast	4.79E-02	-1.38E-01	4.20E-01	1.50E-01
10586	GSM615823	GSE25066	Breast Cancer	Breast	1.83E-01	1.03E-01	3.55E-01	1.64E-01
10587	GSM615824	GSE25066	Breast Cancer	Breast	9.59E-11	3.80E-01	2.72E-06	5.78E-01
10588	GSM615825	GSE25066	Breast Cancer	Breast	1.16E-01	-1.16E-01	1.51E-01	2.22E-01
10589	GSM615826	GSE25066	Breast Cancer	Breast	1.77E-01	1.04E-01	1.80E-01	2.11E-01
10590	GSM615827	GSE25066	Breast Cancer	Breast	7.37E-24	5.77E-01	1.30E-11	8.09E-01
10591	GSM615828	GSE25066	Breast Cancer	Breast	9.11E-04	2.09E-01	2.49E-03	3.96E-01
10592	GSM358341	GSE14333	Colorectal Cancer	Colorectal	1.22E-26	-6.11E-01	2.21E-09	-7.21E-01
10593	GSM358342	GSE14333	Colorectal Cancer	Colorectal	1.83E-01	1.03E-01	1.46E-02	3.32E-01
10594	GSM358343	GSE14333	Colorectal Cancer	Colorectal	6.35E-17	-4.83E-01	8.16E-06	-5.53E-01
10595	GSM358344	GSE14333	Colorectal Cancer	Colorectal	6.16E-21	-5.40E-01	2.79E-06	-5.78E-01
10596	GSM358345	GSE14333	Colorectal Cancer	Colorectal	5.23E-11	-3.85E-01	4.07E-03	-3.79E-01
10597	GSM358346	GSE14333	Colorectal Cancer	Colorectal	3.19E-26	-6.06E-01	9.88E-08	-6.49E-01
10598	GSM358347	GSE14333	Colorectal Cancer	Colorectal	7.27E-15	-4.51E-01	5.49E-05	-5.06E-01
10599	GSM358348	GSE14333	Colorectal Cancer	Colorectal	3.95E-36	-7.14E-01	9.30E-11	-7.77E-01
10600	GSM358349	GSE14333	Colorectal Cancer	Colorectal	5.44E-22	-5.54E-01	4.30E-08	-6.65E-01
10601	GSM358350	GSE14333	Colorectal Cancer	Colorectal	1.77E-34	-6.97E-01	3.48E-09	-7.13E-01
10602	GSM358351	GSE14333	Colorectal Cancer	Colorectal	3.29E-08	-3.28E-01	2.59E-03	-3.94E-01
10603	GSM358352	GSE14333	Colorectal Cancer	Colorectal	1.47E-25	-5.98E-01	2.91E-08	-6.73E-01
10604	GSM358353	GSE14333	Colorectal Cancer	Colorectal	9.55E-28	-6.24E-01	1.46E-10	-7.69E-01
10605	GSM358354	GSE14333	Colorectal Cancer	Colorectal	4.95E-36	-7.13E-01	2.16E-11	-8.01E-01
10606	GSM358355	GSE14333	Colorectal Cancer	Colorectal	2.99E-35	-7.05E-01	5.91E-10	-7.45E-01
10607	GSM358356	GSE14333	Colorectal Cancer	Colorectal	1.24E-38	-7.39E-01	5.89E-11	-7.84E-01
10608	GSM358357	GSE14333	Colorectal Cancer	Colorectal	1.89E-39	-7.47E-01	2.25E-11	-8.00E-01
10609	GSM358358	GSE14333	Colorectal Cancer	Colorectal	6.48E-23	-5.65E-01	1.40E-07	-6.42E-01
10610	GSM358359	GSE14333	Colorectal Cancer	Colorectal	1.63E-03	-2.00E-01	5.53E-02	-2.75E-01
10611	GSM358360	GSE14333	Colorectal Cancer	Colorectal	8.32E-26	-6.01E-01	3.01E-08	-6.72E-01
10612	GSM358361	GSE14333	Colorectal Cancer	Colorectal	5.23E-41	-7.62E-01	2.97E-12	-8.32E-01
10613	GSM358362	GSE14333	Colorectal Cancer	Colorectal	2.53E-41	-7.65E-01	3.77E-13	-8.64E-01
10614	GSM358363	GSE14333	Colorectal Cancer	Colorectal	5.60E-27	-6.15E-01	1.23E-08	-6.90E-01
10615	GSM358364	GSE14333	Colorectal Cancer	Colorectal	2.53E-03	-1.93E-01	3.14E-01	1.74E-01
10616	GSM358365	GSE14333	Colorectal Cancer	Colorectal	6.12E-08	-3.22E-01	8.02E-03	-3.55E-01
10617	GSM358366	GSE14333	Colorectal Cancer	Colorectal	9.74E-36	-7.10E-01	2.97E-12	-8.32E-01
10618	GSM358367	GSE14333	Colorectal Cancer	Colorectal	2.21E-34	-6.96E-01	3.58E-11	-7.92E-01
10619	GSM358368	GSE14333	Colorectal Cancer	Colorectal	1.03E-20	-5.37E-01	7.95E-06	-5.54E-01
10620	GSM358369	GSE14333	Colorectal Cancer	Colorectal	2.40E-39	-7.46E-01	6.13E-10	-7.44E-01
10621	GSM358370	GSE14333	Colorectal Cancer	Colorectal	2.36E-51	-8.54E-01	7.70E-17	-9.84E-01
10622	GSM358371	GSE14333	Colorectal Cancer	Colorectal	1.99E-41	-7.66E-01	5.01E-12	-8.24E-01
10623	GSM358372	GSE14333	Colorectal Cancer	Colorectal	5.10E-31	-6.61E-01	1.45E-09	-7.29E-01
10624	GSM358373	GSE14333	Colorectal Cancer	Colorectal	7.79E-39	-7.41E-01	3.45E-11	-7.93E-01
10625	GSM358374	GSE14333	Colorectal Cancer	Colorectal	1.98E-15	-4.60E-01	1.81E-04	-4.74E-01
10626	GSM358375	GSE14333	Colorectal Cancer	Colorectal	3.15E-36	-7.15E-01	2.37E-10	-7.61E-01
10627	GSM358376	GSE14333	Colorectal Cancer	Colorectal	1.72E-32	-6.77E-01	1.45E-09	-7.29E-01
10628	GSM358377	GSE14333	Colorectal Cancer	Colorectal	1.73E-27	-6.21E-01	4.44E-08	-6.65E-01
10629	GSM358378	GSE14333	Colorectal Cancer	Colorectal	3.36E-46	-8.10E-01	2.51E-14	-9.04E-01
10630	GSM358379	GSE14333	Colorectal Cancer	Colorectal	7.78E-36	-7.11E-01	9.11E-10	-7.37E-01
10631	GSM358380	GSE14333	Colorectal Cancer	Colorectal	5.05E-38	-7.33E-01	8.09E-12	-8.17E-01
10632	GSM358381	GSE14333	Colorectal Cancer	Colorectal	1.27E-36	-7.19E-01	1.46E-10	-7.69E-01
10633	GSM358382	GSE14333	Colorectal Cancer	Colorectal	2.20E-23	-5.71E-01	2.87E-06	-5.77E-01
10634	GSM358383	GSE14333	Colorectal Cancer	Colorectal	5.46E-15	-4.53E-01	3.01E-05	-5.21E-01
10635	GSM358384	GSE14333	Colorectal Cancer	Colorectal	8.50E-11	-3.81E-01	4.25E-04	-4.50E-01
10636	GSM358385	GSE14333	Colorectal Cancer	Colorectal	1.74E-05	-2.62E-01	1.20E-02	-3.40E-01
10637	GSM358386	GSE14333	Colorectal Cancer	Colorectal	2.49E-10	-3.72E-01	5.64E-04	-4.42E-01
10638	GSM358387	GSE14333	Colorectal Cancer	Colorectal	2.89E-16	-4.73E-01	1.31E-04	-4.83E-01
10639	GSM358388	GSE14333	Colorectal Cancer	Colorectal	1.34E-11	-3.96E-01	4.35E-04	-4.50E-01
10640	GSM358389	GSE14333	Colorectal Cancer	Colorectal	5.91E-11	-3.84E-01	2.05E-03	-4.02E-01
10641	GSM358390	GSE14333	Colorectal Cancer	Colorectal	2.89E-16	-4.73E-01	4.08E-05	-5.14E-01
10642	GSM358391	GSE14333	Colorectal Cancer	Colorectal	2.40E-20	-5.32E-01	3.11E-07	-6.25E-01
10643	GSM358392	GSE14333	Colorectal Cancer	Colorectal	1.04E-06	-2.94E-01	7.88E-03	-3.56E-01
10644	GSM358393	GSE14333	Colorectal Cancer	Colorectal	5.19E-21	-5.41E-01	6.40E-07	-6.10E-01
10645	GSM358394	GSE14333	Colorectal Cancer	Colorectal	1.71E-15	-4.61E-01	2.09E-07	-6.34E-01
10646	GSM358395	GSE14333	Colorectal Cancer	Colorectal	2.04E-07	-3.11E-01	3.19E-03	-3.87E-01
10647	GSM358396	GSE14333	Colorectal Cancer	Colorectal	5.26E-16	-4.69E-01	2.72E-06	-5.78E-01
10648	GSM358397	GSE14333	Colorectal Cancer	Colorectal	3.83E-22	-5.56E-01	8.21E-09	-6.97E-01
10649	GSM358398	GSE14333	Colorectal Cancer	Colorectal	5.44E-22	-5.54E-01	1.44E-07	-6.41E-01
10650	GSM358399	GSE14333	Colorectal Cancer	Colorectal	8.01E-25	-5.89E-01	3.82E-10	-7.52E-01
10651	GSM358400	GSE14333	Colorectal Cancer	Colorectal	1.28E-15	-4.63E-01	2.94E-05	-5.22E-01

10652	GSM358401	GSE14333	Colorectal Cancer	Colorectal	9.74E-36	-7.10E-01	1.75E-12	-8.41E-01
10653	GSM358402	GSE14333	Colorectal Cancer	Colorectal	1.47E-25	-5.98E-01	6.33E-08	-6.58E-01
10654	GSM358403	GSE14333	Colorectal Cancer	Colorectal	2.65E-15	-4.58E-01	4.08E-05	-5.14E-01
10655	GSM358404	GSE14333	Colorectal Cancer	Colorectal	1.11E-14	-4.48E-01	3.88E-05	-5.15E-01
10656	GSM358405	GSE14333	Colorectal Cancer	Colorectal	1.04E-11	-3.98E-01	4.07E-04	-4.51E-01
10657	GSM358406	GSE14333	Colorectal Cancer	Colorectal	7.30E-21	-5.39E-01	2.09E-07	-6.34E-01
10658	GSM358407	GSE14333	Colorectal Cancer	Colorectal	3.78E-23	-5.68E-01	9.88E-08	-6.49E-01
10659	GSM358408	GSE14333	Colorectal Cancer	Colorectal	8.32E-26	-6.01E-01	2.91E-08	-6.73E-01
10660	GSM358409	GSE14333	Colorectal Cancer	Colorectal	2.04E-07	-3.11E-01	4.15E-03	-3.78E-01
10661	GSM358410	GSE14333	Colorectal Cancer	Colorectal	3.54E-28	-6.29E-01	9.11E-10	-7.37E-01
10662	GSM358411	GSE14333	Colorectal Cancer	Colorectal	3.62E-11	-3.88E-01	1.98E-03	-4.03E-01
10663	GSM358412	GSE14333	Colorectal Cancer	Colorectal	1.83E-23	-5.72E-01	3.48E-09	-7.13E-01
10664	GSM358413	GSE14333	Colorectal Cancer	Colorectal	3.66E-07	-3.05E-01	1.56E-03	-4.11E-01
10665	GSM358414	GSE14333	Colorectal Cancer	Colorectal	7.57E-30	-6.48E-01	1.23E-08	-6.90E-01
10666	GSM358415	GSE14333	Colorectal Cancer	Colorectal	2.69E-03	-1.92E-01	4.26E-01	-1.49E-01
10667	GSM358416	GSE14333	Colorectal Cancer	Colorectal	7.11E-10	-3.63E-01	4.15E-03	-3.78E-01
10668	GSM358417	GSE14333	Colorectal Cancer	Colorectal	2.59E-06	-2.84E-01	1.22E-02	-3.39E-01
10669	GSM358418	GSE14333	Colorectal Cancer	Colorectal	6.21E-36	-7.12E-01	1.75E-12	-8.41E-01
10670	GSM358419	GSE14333	Colorectal Cancer	Colorectal	6.49E-07	-2.99E-01	1.18E-02	-3.40E-01
10671	GSM358420	GSE14333	Colorectal Cancer	Colorectal	1.42E-27	-6.22E-01	4.44E-08	-6.65E-01
10672	GSM358421	GSE14333	Colorectal Cancer	Colorectal	4.52E-23	-5.67E-01	1.90E-08	-6.81E-01
10673	GSM358422	GSE14333	Colorectal Cancer	Colorectal	5.01E-30	-6.50E-01	3.58E-11	-7.92E-01
10674	GSM358423	GSE14333	Colorectal Cancer	Colorectal	1.16E-27	-6.23E-01	1.46E-10	-7.69E-01
10675	GSM358424	GSE14333	Colorectal Cancer	Colorectal	4.42E-06	-2.78E-01	2.24E-02	-3.15E-01
10676	GSM358425	GSE14333	Colorectal Cancer	Colorectal	1.97E-02	-1.57E-01	8.88E-02	-2.51E-01
10677	GSM358426	GSE14333	Colorectal Cancer	Colorectal	2.94E-17	-4.88E-01	2.11E-05	-5.30E-01
10678	GSM358427	GSE14333	Colorectal Cancer	Colorectal	2.49E-16	-4.74E-01	2.72E-06	-5.78E-01
10679	GSM358428	GSE14333	Colorectal Cancer	Colorectal	2.58E-29	-6.42E-01	3.82E-10	-7.52E-01
10680	GSM358429	GSE14333	Colorectal Cancer	Colorectal	1.12E-32	-6.79E-01	9.45E-10	-7.37E-01
10681	GSM358430	GSE14333	Colorectal Cancer	Colorectal	5.85E-33	-6.82E-01	5.67E-11	-7.85E-01
10682	GSM358431	GSE14333	Colorectal Cancer	Colorectal	2.61E-21	-5.45E-01	2.79E-06	-5.78E-01
10683	GSM358432	GSE14333	Colorectal Cancer	Colorectal	2.52E-17	-4.89E-01	2.05E-05	-5.31E-01
10684	GSM358433	GSE14333	Colorectal Cancer	Colorectal	2.40E-20	-5.32E-01	5.74E-06	-5.61E-01
10685	GSM358434	GSE14333	Colorectal Cancer	Colorectal	1.72E-32	-6.77E-01	8.09E-12	-8.17E-01
10686	GSM358435	GSE14333	Colorectal Cancer	Colorectal	1.89E-22	-5.60E-01	1.90E-08	-6.81E-01
10687	GSM358436	GSE14333	Colorectal Cancer	Colorectal	1.17E-16	-4.79E-01	3.01E-05	-5.21E-01
10688	GSM358437	GSE14333	Colorectal Cancer	Colorectal	1.14E-29	-6.46E-01	1.40E-07	-6.42E-01
10689	GSM358438	GSE14333	Colorectal Cancer	Colorectal	4.53E-01	-7.04E-02	3.93E-01	-1.56E-01
10690	GSM358439	GSE14333	Colorectal Cancer	Colorectal	2.89E-16	-4.73E-01	1.34E-04	-4.83E-01
10691	GSM358440	GSE14333	Colorectal Cancer	Colorectal	6.35E-17	-4.83E-01	2.11E-05	-5.30E-01
10692	GSM358441	GSE14333	Colorectal Cancer	Colorectal	1.34E-11	-3.96E-01	3.38E-03	-3.85E-01
10693	GSM358442	GSE14333	Colorectal Cancer	Colorectal	5.19E-21	-5.41E-01	3.90E-06	-5.70E-01
10694	GSM358443	GSE14333	Colorectal Cancer	Colorectal	1.22E-25	-5.99E-01	2.82E-08	-6.74E-01
10695	GSM358444	GSE14333	Colorectal Cancer	Colorectal	6.21E-36	-7.12E-01	2.21E-09	-7.21E-01
10696	GSM358445	GSE14333	Colorectal Cancer	Colorectal	8.03E-38	-7.31E-01	9.30E-11	-7.77E-01
10697	GSM358446	GSE14333	Colorectal Cancer	Colorectal	6.16E-18	-4.98E-01	1.99E-06	-5.85E-01
10698	GSM358447	GSE14333	Colorectal Cancer	Colorectal	5.36E-09	-3.45E-01	6.98E-04	-4.36E-01
10699	GSM358448	GSE14333	Colorectal Cancer	Colorectal	1.56E-45	-8.04E-01	8.12E-16	-9.52E-01
10700	GSM358449	GSE14333	Colorectal Cancer	Colorectal	2.39E-35	-7.06E-01	5.67E-11	-7.85E-01
10701	GSM358450	GSE14333	Colorectal Cancer	Colorectal	5.57E-19	-5.13E-01	6.59E-07	-6.10E-01
10702	GSM358451	GSE14333	Colorectal Cancer	Colorectal	1.57E-38	-7.38E-01	3.58E-11	-7.92E-01
10703	GSM358452	GSE14333	Colorectal Cancer	Colorectal	3.26E-32	-6.74E-01	1.96E-08	-6.81E-01
10704	GSM358453	GSE14333	Colorectal Cancer	Colorectal	4.95E-36	-7.13E-01	3.48E-09	-7.13E-01
10705	GSM358454	GSE14333	Colorectal Cancer	Colorectal	5.01E-30	-6.50E-01	2.29E-09	-7.21E-01
10706	GSM358455	GSE14333	Colorectal Cancer	Colorectal	4.92E-59	-9.17E-01	2.53E-16	-9.68E-01
10707	GSM358456	GSE14333	Colorectal Cancer	Colorectal	6.87E-26	-6.02E-01	3.90E-06	-5.70E-01
10708	GSM358457	GSE14333	Colorectal Cancer	Colorectal	3.53E-24	-5.81E-01	2.15E-07	-6.33E-01
10709	GSM358458	GSE14333	Colorectal Cancer	Colorectal	2.40E-20	-5.32E-01	1.37E-06	-5.94E-01
10710	GSM358459	GSE14333	Colorectal Cancer	Colorectal	1.22E-35	-7.09E-01	1.75E-12	-8.41E-01
10711	GSM358460	GSE14333	Colorectal Cancer	Colorectal	1.22E-41	-7.68E-01	3.77E-13	-8.64E-01
10712	GSM358461	GSE14333	Colorectal Cancer	Colorectal	4.71E-33	-6.83E-01	3.82E-10	-7.52E-01
10713	GSM358462	GSE14333	Colorectal Cancer	Colorectal	6.86E-01	4.86E-02	3.46E-01	1.66E-01
10714	GSM358463	GSE14333	Colorectal Cancer	Colorectal	6.80E-27	-6.14E-01	2.91E-08	-6.73E-01
10715	GSM358464	GSE14333	Colorectal Cancer	Colorectal	1.14E-34	-6.99E-01	2.37E-10	-7.61E-01
10716	GSM358465	GSE14333	Colorectal Cancer	Colorectal	5.51E-25	-5.91E-01	9.39E-07	-6.02E-01
10717	GSM358466	GSE14333	Colorectal Cancer	Colorectal	5.10E-24	-5.79E-01	6.54E-08	-6.57E-01
10718	GSM358467	GSE14333	Colorectal Cancer	Colorectal	2.79E-18	-5.03E-01	4.01E-06	-5.70E-01
10719	GSM358468	GSE14333	Colorectal Cancer	Colorectal	2.09E-19	-5.19E-01	5.58E-06	-5.62E-01
10720	GSM358469	GSE14333	Colorectal Cancer	Colorectal	6.37E-38	-7.32E-01	1.35E-11	-8.08E-01
10721	GSM358470	GSE14333	Colorectal Cancer	Colorectal	8.62E-17	-4.81E-01	2.05E-05	-5.31E-01
10722	GSM358471	GSE14333	Colorectal Cancer	Colorectal	2.64E-26	-6.07E-01	3.48E-09	-7.13E-01
10723	GSM358472	GSE14333	Colorectal Cancer	Colorectal	8.86E-06	-2.70E-01	1.49E-02	-3.31E-01
10724	GSM358473	GSE14333	Colorectal Cancer	Colorectal	3.29E-47	-8.18E-01	3.77E-13	-8.64E-01
10725	GSM358474	GSE14333	Colorectal Cancer	Colorectal	1.45E-30	-6.56E-01	9.11E-10	-7.37E-01

10726	GSM358475	GSE14333	Colorectal Cancer	Colorectal	8.86E-06	-2.70E-01	1.54E-02	-3.30E-01
10727	GSM358476	GSE14333	Colorectal Cancer	Colorectal	2.02E-18	-5.05E-01	9.66E-07	-6.01E-01
10728	GSM358477	GSE14333	Colorectal Cancer	Colorectal	3.54E-10	-3.69E-01	1.37E-04	-4.82E-01
10729	GSM358478	GSE14333	Colorectal Cancer	Colorectal	2.64E-02	-1.51E-01	3.19E-01	-1.73E-01
10730	GSM358479	GSE14333	Colorectal Cancer	Colorectal	1.48E-15	-4.62E-01	1.33E-06	-5.94E-01
10731	GSM358480	GSE14333	Colorectal Cancer	Colorectal	5.10E-24	-5.79E-01	3.68E-10	-7.53E-01
10732	GSM358481	GSE14333	Colorectal Cancer	Colorectal	7.70E-19	-5.11E-01	5.90E-06	-5.61E-01
10733	GSM358482	GSE14333	Colorectal Cancer	Colorectal	8.26E-27	-6.13E-01	5.27E-09	-7.05E-01
10734	GSM358483	GSE14333	Colorectal Cancer	Colorectal	5.08E-37	-7.23E-01	1.45E-09	-7.29E-01
10735	GSM358484	GSE14333	Colorectal Cancer	Colorectal	1.89E-22	-5.60E-01	1.23E-08	-6.90E-01
10736	GSM358485	GSE14333	Colorectal Cancer	Colorectal	8.39E-15	-4.50E-01	2.72E-06	-5.78E-01
10737	GSM358486	GSE14333	Colorectal Cancer	Colorectal	3.79E-27	-6.17E-01	1.46E-10	-7.69E-01
10738	GSM358487	GSE14333	Colorectal Cancer	Colorectal	2.89E-16	-4.73E-01	1.07E-05	-5.47E-01
10739	GSM358488	GSE14333	Colorectal Cancer	Colorectal	2.14E-16	-4.75E-01	3.90E-06	-5.70E-01
10740	GSM358489	GSE14333	Colorectal Cancer	Colorectal	9.23E-09	-3.40E-01	2.38E-04	-4.67E-01
10741	GSM358490	GSE14333	Colorectal Cancer	Colorectal	5.10E-24	-5.79E-01	6.33E-08	-6.58E-01
10742	GSM358491	GSE14333	Colorectal Cancer	Colorectal	1.96E-04	-2.31E-01	1.01E-01	-2.44E-01
10743	GSM358492	GSE14333	Colorectal Cancer	Colorectal	5.94E-02	-1.33E-01	2.27E-01	-1.97E-01
10744	GSM358493	GSE14333	Colorectal Cancer	Colorectal	2.37E-06	2.85E-01	1.20E-03	4.19E-01
10745	GSM358494	GSE14333	Colorectal Cancer	Colorectal	1.22E-20	-5.36E-01	1.93E-06	-5.86E-01
10746	GSM358495	GSE14333	Colorectal Cancer	Colorectal	3.19E-26	-6.06E-01	6.54E-08	-6.57E-01
10747	GSM358496	GSE14333	Colorectal Cancer	Colorectal	3.54E-10	-3.69E-01	1.59E-03	-4.10E-01
10748	GSM358497	GSE14333	Colorectal Cancer	Colorectal	1.71E-14	-4.45E-01	3.90E-06	-5.70E-01
10749	GSM358498	GSE14333	Colorectal Cancer	Colorectal	2.63E-23	-5.70E-01	1.45E-09	-7.29E-01
10750	GSM358499	GSE14333	Colorectal Cancer	Colorectal	8.97E-13	-4.17E-01	4.16E-04	-4.51E-01
10751	GSM358500	GSE14333	Colorectal Cancer	Colorectal	3.49E-04	-2.23E-01	3.81E-02	-2.92E-01
10752	GSM358501	GSE14333	Colorectal Cancer	Colorectal	6.51E-04	-2.14E-01	3.35E-02	-2.98E-01
10753	GSM358502	GSE14333	Colorectal Cancer	Colorectal	1.10E-15	-4.64E-01	4.08E-05	-5.14E-01
10754	GSM358503	GSE14333	Colorectal Cancer	Colorectal	1.35E-17	-4.93E-01	6.40E-07	-6.10E-01
10755	GSM358504	GSE14333	Colorectal Cancer	Colorectal	1.30E-21	-5.49E-01	1.40E-07	-6.42E-01
10756	GSM358505	GSE14333	Colorectal Cancer	Colorectal	1.98E-15	-4.60E-01	5.36E-05	-5.07E-01
10757	GSM358506	GSE14333	Colorectal Cancer	Colorectal	4.02E-19	-5.15E-01	1.44E-07	-6.41E-01
10758	GSM358507	GSE14333	Colorectal Cancer	Colorectal	1.37E-06	-2.91E-01	1.20E-02	-3.40E-01
10759	GSM358508	GSE14333	Colorectal Cancer	Colorectal	5.00E-32	-6.72E-01	2.29E-09	-7.21E-01
10760	GSM358509	GSE14333	Colorectal Cancer	Colorectal	5.83E-05	-2.47E-01	5.07E-03	-3.71E-01
10761	GSM358510	GSE14333	Colorectal Cancer	Colorectal	7.01E-02	1.29E-01	3.11E-02	3.01E-01
10762	GSM358511	GSE14333	Colorectal Cancer	Colorectal	1.22E-35	-7.09E-01	9.30E-11	-7.77E-01
10763	GSM358512	GSE14333	Colorectal Cancer	Colorectal	6.55E-19	-5.12E-01	7.74E-06	-5.54E-01
10764	GSM358513	GSE14333	Colorectal Cancer	Colorectal	1.02E-12	-4.16E-01	5.52E-04	-4.43E-01
10765	GSM358514	GSE14333	Colorectal Cancer	Colorectal	1.52E-12	-4.13E-01	5.52E-04	-4.43E-01
10766	GSM358515	GSE14333	Colorectal Cancer	Colorectal	2.49E-16	-4.74E-01	4.01E-06	-5.70E-01
10767	GSM358516	GSE14333	Colorectal Cancer	Colorectal	2.61E-21	-5.45E-01	6.59E-07	-6.10E-01
10768	GSM358517	GSE14333	Colorectal Cancer	Colorectal	1.85E-17	-4.91E-01	9.39E-07	-6.02E-01
10769	GSM358518	GSE14333	Colorectal Cancer	Colorectal	1.12E-32	-6.79E-01	2.37E-10	-7.61E-01
10770	GSM358519	GSE14333	Colorectal Cancer	Colorectal	1.53E-23	-5.73E-01	1.90E-08	-6.81E-01
10771	GSM358520	GSE14333	Colorectal Cancer	Colorectal	1.85E-07	-3.12E-01	2.59E-03	-3.94E-01
10772	GSM358521	GSE14333	Colorectal Cancer	Colorectal	2.63E-23	-5.70E-01	3.02E-07	-6.26E-01
10773	GSM358522	GSE14333	Colorectal Cancer	Colorectal	4.01E-17	-4.86E-01	5.74E-06	-5.61E-01
10774	GSM358523	GSE14333	Colorectal Cancer	Colorectal	9.65E-25	-5.88E-01	1.90E-08	-6.81E-01
10775	GSM358524	GSE14333	Colorectal Cancer	Colorectal	2.49E-16	-4.74E-01	2.72E-06	-5.78E-01
10776	GSM358525	GSE14333	Colorectal Cancer	Colorectal	1.46E-04	-2.35E-01	1.80E-02	-3.24E-01
10777	GSM358526	GSE14333	Colorectal Cancer	Colorectal	1.95E-11	-3.93E-01	5.76E-04	-4.41E-01
10778	GSM358527	GSE14333	Colorectal Cancer	Colorectal	1.85E-17	-4.91E-01	4.47E-07	-6.18E-01
10779	GSM358528	GSE14333	Colorectal Cancer	Colorectal	1.48E-14	-4.46E-01	3.88E-05	-5.15E-01
10780	GSM358529	GSE14333	Colorectal Cancer	Colorectal	5.16E-03	-1.82E-01	2.25E-01	-1.97E-01
10781	GSM358530	GSE14333	Colorectal Cancer	Colorectal	2.62E-05	-2.57E-01	1.26E-02	-3.38E-01
10782	GSM358531	GSE14333	Colorectal Cancer	Colorectal	1.53E-35	-7.08E-01	1.35E-11	-8.08E-01
10783	GSM358532	GSE14333	Colorectal Cancer	Colorectal	4.39E-02	-1.40E-01	2.56E-01	1.89E-01
10784	GSM358533	GSE14333	Colorectal Cancer	Colorectal	9.51E-16	-4.65E-01	1.58E-05	-5.37E-01
10785	GSM358534	GSE14333	Colorectal Cancer	Colorectal	1.85E-17	-4.91E-01	7.74E-06	-5.54E-01
10786	GSM358535	GSE14333	Colorectal Cancer	Colorectal	4.83E-06	-2.77E-01	6.62E-03	-3.62E-01
10787	GSM358536	GSE14333	Colorectal Cancer	Colorectal	1.26E-09	-3.58E-01	1.27E-03	-4.17E-01
10788	GSM358537	GSE14333	Colorectal Cancer	Colorectal	4.14E-31	-6.62E-01	2.21E-09	-7.21E-01
10789	GSM358538	GSE14333	Colorectal Cancer	Colorectal	6.19E-02	-1.32E-01	5.03E-01	-1.34E-01
10790	GSM358539	GSE14333	Colorectal Cancer	Colorectal	3.53E-24	-5.81E-01	9.88E-08	-6.49E-01
10791	GSM358540	GSE14333	Colorectal Cancer	Colorectal	1.96E-44	-7.94E-01	7.15E-14	-8.89E-01
10792	GSM358541	GSE14333	Colorectal Cancer	Colorectal	2.21E-09	3.53E-01	3.80E-06	5.71E-01
10793	GSM358542	GSE14333	Colorectal Cancer	Colorectal	4.74E-29	-6.39E-01	1.44E-07	-6.41E-01
10794	GSM358543	GSE14333	Colorectal Cancer	Colorectal	3.34E-45	-8.01E-01	7.47E-14	-8.88E-01
10795	GSM358544	GSE14333	Colorectal Cancer	Colorectal	1.53E-35	-7.08E-01	1.35E-11	-8.08E-01
10796	GSM358545	GSE14333	Colorectal Cancer	Colorectal	1.28E-37	-7.29E-01	5.89E-11	-7.84E-01
10797	GSM358546	GSE14333	Colorectal Cancer	Colorectal	1.96E-14	-4.44E-01	8.16E-03	-3.54E-01
10798	GSM358547	GSE14333	Colorectal Cancer	Colorectal	8.32E-26	-6.01E-01	1.90E-08	-6.81E-01
10799	GSM358548	GSE14333	Colorectal Cancer	Colorectal	1.59E-33	-6.88E-01	4.44E-08	-6.65E-01

10800	GSM358549	GSE14333	Colorectal Cancer	Colorectal	4.28E-34	-6.93E-01	5.91E-10	-7.45E-01
10801	GSM358550	GSE14333	Colorectal Cancer	Colorectal	6.39E-37	-7.22E-01	1.35E-11	-8.08E-01
10802	GSM358551	GSE14333	Colorectal Cancer	Colorectal	6.80E-27	-6.14E-01	4.30E-08	-6.65E-01
10803	GSM358552	GSE14333	Colorectal Cancer	Colorectal	6.21E-36	-7.12E-01	5.91E-10	-7.45E-01
10804	GSM358553	GSE14333	Colorectal Cancer	Colorectal	2.21E-10	-3.73E-01	2.27E-02	-3.14E-01
10805	GSM358554	GSE14333	Colorectal Cancer	Colorectal	2.02E-18	-5.05E-01	1.03E-04	-4.90E-01
10806	GSM358555	GSE14333	Colorectal Cancer	Colorectal	2.52E-17	-4.89E-01	3.98E-05	-5.14E-01
10807	GSM358556	GSE14333	Colorectal Cancer	Colorectal	4.68E-35	-7.03E-01	3.58E-11	-7.92E-01
10808	GSM358557	GSE14333	Colorectal Cancer	Colorectal	1.78E-30	-6.55E-01	9.11E-10	-7.37E-01
10809	GSM358558	GSE14333	Colorectal Cancer	Colorectal	3.44E-14	-4.40E-01	2.16E-05	-5.30E-01
10810	GSM358559	GSE14333	Colorectal Cancer	Colorectal	3.06E-33	-6.85E-01	7.93E-09	-6.98E-01
10811	GSM358560	GSE14333	Colorectal Cancer	Colorectal	1.74E-40	-7.57E-01	1.68E-12	-8.41E-01
10812	GSM358561	GSE14333	Colorectal Cancer	Colorectal	6.48E-22	-5.53E-01	7.95E-06	-5.54E-01
10813	GSM358562	GSE14333	Colorectal Cancer	Colorectal	5.05E-38	-7.33E-01	9.30E-11	-7.77E-01
10814	GSM358563	GSE14333	Colorectal Cancer	Colorectal	1.72E-18	-5.06E-01	9.39E-07	-6.02E-01
10815	GSM358564	GSE14333	Colorectal Cancer	Colorectal	5.60E-27	-6.15E-01	5.91E-10	-7.45E-01
10816	GSM358565	GSE14333	Colorectal Cancer	Colorectal	4.71E-33	-6.83E-01	9.30E-11	-7.77E-01
10817	GSM358566	GSE14333	Colorectal Cancer	Colorectal	1.27E-36	-7.19E-01	3.82E-10	-7.52E-01
10818	GSM358567	GSE14333	Colorectal Cancer	Colorectal	1.19E-13	-4.32E-01	1.85E-04	-4.74E-01
10819	GSM358568	GSE14333	Colorectal Cancer	Colorectal	4.53E-16	-4.70E-01	7.95E-06	-5.54E-01
10820	GSM358569	GSE14333	Colorectal Cancer	Colorectal	8.03E-38	-7.31E-01	2.16E-11	-8.01E-01
10821	GSM358570	GSE14333	Colorectal Cancer	Colorectal	1.24E-07	-3.15E-01	2.44E-04	-4.66E-01
10822	GSM358571	GSE14333	Colorectal Cancer	Colorectal	5.10E-31	-6.61E-01	1.23E-08	-6.90E-01
10823	GSM358572	GSE14333	Colorectal Cancer	Colorectal	8.84E-24	-5.76E-01	1.90E-08	-6.81E-01
10824	GSM358573	GSE14333	Colorectal Cancer	Colorectal	1.41E-09	-3.57E-01	2.01E-03	-4.03E-01
10825	GSM358574	GSE14333	Colorectal Cancer	Colorectal	1.32E-22	-5.62E-01	4.75E-07	-6.17E-01
10826	GSM358575	GSE14333	Colorectal Cancer	Colorectal	3.20E-11	-3.89E-01	3.12E-04	-4.59E-01
10827	GSM358576	GSE14333	Colorectal Cancer	Colorectal	1.10E-21	-5.50E-01	3.11E-07	-6.25E-01
10828	GSM358577	GSE14333	Colorectal Cancer	Colorectal	1.33E-12	-4.14E-01	7.36E-05	-4.98E-01
10829	GSM358578	GSE14333	Colorectal Cancer	Colorectal	2.60E-14	-4.42E-01	5.36E-05	-5.07E-01
10830	GSM358579	GSE14333	Colorectal Cancer	Colorectal	7.13E-12	-4.01E-01	3.19E-04	-4.58E-01
10831	GSM358580	GSE14333	Colorectal Cancer	Colorectal	9.51E-16	-4.65E-01	1.99E-06	-5.85E-01
10832	GSM358581	GSE14333	Colorectal Cancer	Colorectal	8.66E-21	-5.38E-01	1.44E-07	-6.41E-01
10833	GSM358582	GSE14333	Colorectal Cancer	Colorectal	7.13E-12	-4.01E-01	3.19E-04	-4.58E-01
10834	GSM358583	GSE14333	Colorectal Cancer	Colorectal	1.11E-14	-4.48E-01	2.72E-06	-5.78E-01
10835	GSM358584	GSE14333	Colorectal Cancer	Colorectal	4.04E-37	-7.24E-01	9.11E-10	-7.37E-01
10836	GSM358585	GSE14333	Colorectal Cancer	Colorectal	1.06E-28	-6.35E-01	1.44E-07	-6.41E-01
10837	GSM358586	GSE14333	Colorectal Cancer	Colorectal	2.83E-11	-3.90E-01	5.40E-04	-4.43E-01
10838	GSM358587	GSE14333	Colorectal Cancer	Colorectal	1.53E-35	7.08E-01	4.62E-15	9.28E-01
10839	GSM358588	GSE14333	Colorectal Cancer	Colorectal	9.12E-35	-7.00E-01	3.68E-10	-7.53E-01
10840	GSM358589	GSE14333	Colorectal Cancer	Colorectal	1.30E-21	-5.49E-01	2.72E-06	-5.78E-01
10841	GSM358590	GSE14333	Colorectal Cancer	Colorectal	1.70E-02	-1.60E-01	1.75E-01	-2.13E-01
10842	GSM358591	GSE14333	Colorectal Cancer	Colorectal	1.55E-10	-3.76E-01	3.44E-03	-3.85E-01
10843	GSM358592	GSE14333	Colorectal Cancer	Colorectal	3.16E-29	-6.41E-01	1.96E-08	-6.81E-01
10844	GSM358593	GSE14333	Colorectal Cancer	Colorectal	5.33E-34	-6.92E-01	3.58E-11	-7.92E-01
10845	GSM358594	GSE14333	Colorectal Cancer	Colorectal	1.26E-03	-2.04E-01	4.29E-01	-1.49E-01
10846	GSM358595	GSE14333	Colorectal Cancer	Colorectal	1.72E-32	-6.77E-01	1.32E-08	-6.88E-01
10847	GSM358596	GSE14333	Colorectal Cancer	Colorectal	5.28E-13	-4.21E-01	5.40E-04	-4.43E-01
10848	GSM358597	GSE14333	Colorectal Cancer	Colorectal	9.65E-25	-5.88E-01	6.33E-08	-6.58E-01
10849	GSM358598	GSE14333	Colorectal Cancer	Colorectal	4.42E-06	-2.78E-01	6.56E-02	-2.67E-01
10850	GSM358599	GSE14333	Colorectal Cancer	Colorectal	4.74E-29	-6.39E-01	8.21E-09	-6.97E-01
10851	GSM358600	GSE14333	Colorectal Cancer	Colorectal	1.83E-23	-5.72E-01	2.22E-07	-6.32E-01
10852	GSM358601	GSE14333	Colorectal Cancer	Colorectal	3.44E-14	-4.40E-01	1.40E-04	-4.81E-01
10853	GSM358602	GSE14333	Colorectal Cancer	Colorectal	6.64E-34	-6.91E-01	5.67E-11	-7.85E-01
10854	GSM358603	GSE14333	Colorectal Cancer	Colorectal	4.28E-12	-4.05E-01	1.81E-04	-4.74E-01
10855	GSM358604	GSE14333	Colorectal Cancer	Colorectal	3.09E-09	-3.50E-01	1.20E-03	-4.19E-01
10856	GSM358605	GSE14333	Colorectal Cancer	Colorectal	1.18E-30	-6.57E-01	1.52E-10	-7.68E-01
10857	GSM358606	GSE14333	Colorectal Cancer	Colorectal	1.16E-04	-2.38E-01	7.55E-02	-2.60E-01
10858	GSM358607	GSE14333	Colorectal Cancer	Colorectal	2.38E-18	-5.04E-01	1.85E-04	-4.74E-01
10859	GSM358608	GSE14333	Colorectal Cancer	Colorectal	1.58E-17	-4.92E-01	4.08E-05	-5.14E-01
10860	GSM358609	GSE14333	Colorectal Cancer	Colorectal	8.27E-34	-6.90E-01	5.91E-10	-7.45E-01
10861	GSM358610	GSE14333	Colorectal Cancer	Colorectal	6.16E-30	-6.49E-01	3.68E-10	-7.53E-01
10862	GSM358611	GSE14333	Colorectal Cancer	Colorectal	5.36E-09	-3.45E-01	1.85E-04	-4.74E-01
10863	GSM358612	GSE14333	Colorectal Cancer	Colorectal	1.18E-44	-7.96E-01	1.07E-12	-8.48E-01
10864	GSM358613	GSE14333	Colorectal Cancer	Colorectal	2.21E-34	-6.96E-01	3.25E-09	-7.14E-01
10865	GSM358614	GSE14333	Colorectal Cancer	Colorectal	1.02E-12	-4.16E-01	9.56E-04	-4.26E-01
10866	GSM358615	GSE14333	Colorectal Cancer	Colorectal	2.67E-52	-8.62E-01	2.31E-17	-1.00E+00
10867	GSM358616	GSE14333	Colorectal Cancer	Colorectal	7.75E-23	-5.64E-01	9.57E-08	-6.50E-01
10868	GSM358617	GSE14333	Colorectal Cancer	Colorectal	4.09E-15	-4.55E-01	5.36E-05	-5.07E-01
10869	GSM358618	GSE14333	Colorectal Cancer	Colorectal	4.60E-27	-6.16E-01	8.21E-09	-6.97E-01
10870	GSM358619	GSE14333	Colorectal Cancer	Colorectal	6.66E-41	-7.61E-01	2.97E-12	-8.32E-01
10871	GSM358620	GSE14333	Colorectal Cancer	Colorectal	1.05E-42	-7.78E-01	1.82E-12	-8.40E-01
10872	GSM358621	GSE14333	Colorectal Cancer	Colorectal	1.14E-34	6.99E-01	7.47E-14	8.88E-01
10873	GSM358622	GSE14333	Colorectal Cancer	Colorectal	2.36E-51	-8.54E-01	8.12E-16	-9.52E-01

10874	GSM358623	GSE14333	Colorectal Cancer	Colorectal	3.36E-46	-8.10E-01	2.18E-13	-8.72E-01
10875	GSM358624	GSE14333	Colorectal Cancer	Colorectal	1.18E-39	-7.49E-01	3.60E-09	-7.12E-01
10876	GSM358625	GSE14333	Colorectal Cancer	Colorectal	2.40E-02	-1.53E-01	2.38E-01	-1.94E-01
10877	GSM358626	GSE14333	Colorectal Cancer	Colorectal	8.03E-38	-7.31E-01	2.46E-10	-7.60E-01
10878	GSM358627	GSE14333	Colorectal Cancer	Colorectal	9.51E-16	-4.65E-01	1.50E-05	-5.38E-01
10879	GSM358628	GSE14333	Colorectal Cancer	Colorectal	1.97E-10	-3.74E-01	3.38E-03	-3.85E-01
10880	GSM358629	GSE14333	Colorectal Cancer	Colorectal	6.16E-30	-6.49E-01	2.21E-09	-7.21E-01
10881	GSM358630	GSE14333	Colorectal Cancer	Colorectal	1.65E-06	-2.89E-01	1.51E-02	-3.31E-01
10882	GSM324715	GSE12945	Colorectal Cancer	Colorectal	2.63E-23	-5.70E-01	9.95E-07	-6.01E-01
10883	GSM324716	GSE12945	Colorectal Cancer	Colorectal	1.78E-25	-5.97E-01	6.54E-08	-6.57E-01
10884	GSM324717	GSE12945	Colorectal Cancer	Colorectal	1.35E-17	-4.93E-01	5.90E-06	-5.61E-01
10885	GSM324718	GSE12945	Colorectal Cancer	Colorectal	5.41E-23	-5.66E-01	4.24E-06	-5.68E-01
10886	GSM324719	GSE12945	Colorectal Cancer	Colorectal	1.40E-29	-6.45E-01	2.91E-08	-6.73E-01
10887	GSM324720	GSE12945	Colorectal Cancer	Colorectal	1.42E-27	-6.22E-01	3.01E-08	-6.72E-01
10888	GSM324721	GSE12945	Colorectal Cancer	Colorectal	1.00E-26	-6.12E-01	3.82E-10	-7.52E-01
10889	GSM324722	GSE12945	Colorectal Cancer	Colorectal	2.40E-20	-5.32E-01	1.33E-06	-5.94E-01
10890	GSM324723	GSE12945	Colorectal Cancer	Colorectal	4.60E-27	-6.16E-01	1.44E-07	-6.41E-01
10891	GSM324724	GSE12945	Colorectal Cancer	Colorectal	2.61E-21	-5.45E-01	1.93E-06	-5.86E-01
10892	GSM324725	GSE12945	Colorectal Cancer	Colorectal	7.74E-20	-5.25E-01	1.10E-05	-5.46E-01
10893	GSM324726	GSE12945	Colorectal Cancer	Colorectal	1.14E-29	-6.46E-01	1.52E-10	-7.68E-01
10894	GSM324727	GSE12945	Colorectal Cancer	Colorectal	1.06E-23	-5.75E-01	6.59E-07	-6.10E-01
10895	GSM324728	GSE12945	Colorectal Cancer	Colorectal	1.58E-22	-5.61E-01	2.15E-07	-6.33E-01
10896	GSM324729	GSE12945	Colorectal Cancer	Colorectal	2.10E-29	-6.43E-01	1.96E-08	-6.81E-01
10897	GSM324730	GSE12945	Colorectal Cancer	Colorectal	3.54E-28	-6.29E-01	1.96E-08	-6.81E-01
10898	GSM324731	GSE12945	Colorectal Cancer	Colorectal	7.72E-22	-5.52E-01	1.49E-07	-6.41E-01
10899	GSM324732	GSE12945	Colorectal Cancer	Colorectal	4.60E-05	-2.50E-01	1.88E-01	-2.09E-01
10900	GSM324733	GSE12945	Colorectal Cancer	Colorectal	2.51E-36	-7.16E-01	5.67E-11	-7.85E-01
10901	GSM324734	GSE12945	Colorectal Cancer	Colorectal	6.21E-36	-7.12E-01	5.89E-11	-7.84E-01
10902	GSM324735	GSE12945	Colorectal Cancer	Colorectal	1.16E-27	-6.23E-01	1.40E-09	-7.30E-01
10903	GSM324736	GSE12945	Colorectal Cancer	Colorectal	1.27E-23	-5.74E-01	1.23E-08	-6.90E-01
10904	GSM324737	GSE12945	Colorectal Cancer	Colorectal	1.58E-16	-4.77E-01	7.74E-06	-5.54E-01
10905	GSM324738	GSE12945	Colorectal Cancer	Colorectal	2.63E-23	-5.70E-01	3.90E-06	-5.70E-01
10906	GSM324739	GSE12945	Colorectal Cancer	Colorectal	6.89E-14	-4.36E-01	6.59E-07	-6.10E-01
10907	GSM324740	GSE12945	Colorectal Cancer	Colorectal	1.89E-22	-5.60E-01	7.95E-06	-5.54E-01
10908	GSM324741	GSE12945	Colorectal Cancer	Colorectal	2.76E-09	-3.51E-01	2.01E-03	-4.03E-01
10909	GSM324742	GSE12945	Colorectal Cancer	Colorectal	1.27E-36	-7.19E-01	3.58E-11	-7.92E-01
10910	GSM324743	GSE12945	Colorectal Cancer	Colorectal	1.00E-16	-4.80E-01	7.73E-05	-4.97E-01
10911	GSM324744	GSE12945	Colorectal Cancer	Colorectal	6.16E-18	-4.98E-01	4.01E-06	-5.70E-01
10912	GSM324745	GSE12945	Colorectal Cancer	Colorectal	3.36E-16	-4.72E-01	1.16E-05	-5.45E-01
10913	GSM324746	GSE12945	Colorectal Cancer	Colorectal	1.50E-19	-5.21E-01	2.15E-07	-6.33E-01
10914	GSM324747	GSE12945	Colorectal Cancer	Colorectal	1.84E-21	-5.47E-01	8.39E-06	-5.52E-01
10915	GSM324748	GSE12945	Colorectal Cancer	Colorectal	1.58E-22	-5.61E-01	1.40E-07	-6.42E-01
10916	GSM324749	GSE12945	Colorectal Cancer	Colorectal	5.01E-30	-6.50E-01	2.29E-09	-7.21E-01
10917	GSM324750	GSE12945	Colorectal Cancer	Colorectal	3.86E-09	-3.48E-01	4.30E-03	-3.77E-01
10918	GSM324751	GSE12945	Colorectal Cancer	Colorectal	4.28E-34	-6.93E-01	3.82E-10	-7.52E-01
10919	GSM324752	GSE12945	Colorectal Cancer	Colorectal	3.87E-26	-6.05E-01	8.21E-09	-6.97E-01
10920	GSM324753	GSE12945	Colorectal Cancer	Colorectal	3.19E-26	-6.06E-01	1.23E-08	-6.90E-01
10921	GSM324754	GSE12945	Colorectal Cancer	Colorectal	1.32E-22	-5.62E-01	1.37E-06	-5.94E-01
10922	GSM324755	GSE12945	Colorectal Cancer	Colorectal	1.73E-12	-4.12E-01	1.37E-04	-4.82E-01
10923	GSM324756	GSE12945	Colorectal Cancer	Colorectal	1.58E-22	-5.61E-01	4.61E-07	-6.17E-01
10924	GSM324757	GSE12945	Colorectal Cancer	Colorectal	2.89E-16	-4.73E-01	3.98E-05	-5.14E-01
10925	GSM324758	GSE12945	Colorectal Cancer	Colorectal	2.64E-26	-6.07E-01	6.33E-08	-6.58E-01
10926	GSM324759	GSE12945	Colorectal Cancer	Colorectal	6.87E-26	-6.02E-01	6.40E-07	-6.10E-01
10927	GSM324760	GSE12945	Colorectal Cancer	Colorectal	7.84E-28	-6.25E-01	3.60E-09	-7.12E-01
10928	GSM324761	GSE12945	Colorectal Cancer	Colorectal	1.42E-27	-6.22E-01	2.21E-09	-7.21E-01
10929	GSM324762	GSE12945	Colorectal Cancer	Colorectal	7.30E-21	-5.39E-01	2.15E-07	-6.33E-01
10930	GSM324763	GSE12945	Colorectal Cancer	Colorectal	3.78E-23	-5.68E-01	9.57E-08	-6.50E-01
10931	GSM324764	GSE12945	Colorectal Cancer	Colorectal	4.09E-15	-4.55E-01	1.81E-04	-4.74E-01
10932	GSM324765	GSE12945	Colorectal Cancer	Colorectal	4.37E-21	-5.42E-01	4.61E-07	-6.17E-01
10933	GSM324766	GSE12945	Colorectal Cancer	Colorectal	1.44E-20	-5.35E-01	1.90E-08	-6.81E-01
10934	GSM324767	GSE12945	Colorectal Cancer	Colorectal	7.37E-24	-5.77E-01	2.05E-06	-5.85E-01
10935	GSM324768	GSE12945	Colorectal Cancer	Colorectal	2.63E-23	-5.70E-01	9.12E-07	-6.03E-01
10936	GSM324769	GSE12945	Colorectal Cancer	Colorectal	6.43E-28	-6.26E-01	2.29E-09	-7.21E-01
10937	GSM324770	GSE12945	Colorectal Cancer	Colorectal	1.30E-21	-5.49E-01	2.15E-07	-6.33E-01
10938	GSM324771	GSE12945	Colorectal Cancer	Colorectal	1.85E-07	3.12E-01	3.78E-05	5.16E-01
10939	GSM324772	GSE12945	Colorectal Cancer	Colorectal	2.53E-03	-1.93E-01	1.24E-01	-2.34E-01
10940	GSM324773	GSE12945	Colorectal Cancer	Colorectal	6.00E-14	-4.37E-01	5.58E-06	-5.62E-01
10941	GSM324774	GSE12945	Colorectal Cancer	Colorectal	1.45E-30	-6.56E-01	9.11E-10	-7.37E-01
10942	GSM324775	GSE12945	Colorectal Cancer	Colorectal	7.84E-07	2.97E-01	9.56E-04	4.26E-01
10943	GSM324776	GSE12945	Colorectal Cancer	Colorectal	1.40E-24	-5.86E-01	3.01E-08	-6.72E-01
10944	GSM277231	GSE10961	Colorectal Cancer	Colorectal	6.43E-28	-6.26E-01	3.60E-09	-7.12E-01
10945	GSM277236	GSE10961	Colorectal Cancer	Colorectal	3.76E-12	-4.06E-01	6.65E-02	-2.66E-01
10946	GSM277238	GSE10961	Colorectal Cancer	Colorectal	2.59E-06	-2.84E-01	1.84E-01	-2.10E-01
10947	GSM277239	GSE10961	Colorectal Cancer	Colorectal	1.28E-15	-4.63E-01	1.92E-02	-3.21E-01

10948	GSM277246	GSE10961	Colorectal Cancer	Colorectal	1.42E-34	-6.98E-01	1.35E-11	-8.08E-01
10949	GSM277248	GSE10961	Colorectal Cancer	Colorectal	5.33E-34	-6.92E-01	2.25E-11	-8.00E-01
10950	GSM277253	GSE10961	Colorectal Cancer	Colorectal	1.08E-40	-7.59E-01	1.07E-12	-8.48E-01
10951	GSM277256	GSE10961	Colorectal Cancer	Colorectal	5.81E-29	-6.38E-01	9.30E-11	-7.77E-01
10952	GSM277466	GSE10961	Colorectal Cancer	Colorectal	1.96E-14	-4.44E-01	1.93E-06	-5.86E-01
10953	GSM277469	GSE10961	Colorectal Cancer	Colorectal	1.89E-39	-7.47E-01	1.35E-11	-8.08E-01
10954	GSM277477	GSE10961	Colorectal Cancer	Colorectal	1.00E-26	-6.12E-01	2.91E-08	-6.73E-01
10955	GSM277478	GSE10961	Colorectal Cancer	Colorectal	6.16E-30	-6.49E-01	9.30E-11	-7.77E-01
10956	GSM277479	GSE10961	Colorectal Cancer	Colorectal	5.00E-32	-6.72E-01	1.32E-08	-6.88E-01
10957	GSM277481	GSE10961	Colorectal Cancer	Colorectal	4.43E-01	-7.14E-02	1.35E-01	-2.29E-01
10958	GSM277494	GSE10961	Colorectal Cancer	Colorectal	3.83E-18	-5.01E-01	6.54E-08	-6.57E-01
10959	GSM277646	GSE10961	Colorectal Cancer	Colorectal	2.21E-09	-3.53E-01	9.21E-02	-2.50E-01
10960	GSM277647	GSE10961	Colorectal Cancer	Colorectal	9.26E-02	1.22E-01	7.36E-02	2.61E-01
10961	GSM277648	GSE10961	Colorectal Cancer	Colorectal	1.48E-14	-4.46E-01	1.25E-03	-4.18E-01
10962	GSM400180	GSE15960	Colorectal Cancer	Colorectal	2.90E-28	-6.30E-01	9.45E-10	-7.37E-01
10963	GSM400181	GSE15960	Colorectal Cancer	Colorectal	9.23E-09	-3.40E-01	2.09E-03	-4.01E-01
10964	GSM400182	GSE15960	Colorectal Cancer	Colorectal	5.60E-27	-6.15E-01	2.82E-08	-6.74E-01
10965	GSM400183	GSE15960	Colorectal Cancer	Colorectal	2.52E-17	-4.89E-01	7.74E-06	-5.54E-01
10966	GSM400184	GSE15960	Colorectal Cancer	Colorectal	9.26E-23	-5.63E-01	1.33E-06	-5.94E-01
10967	GSM400185	GSE15960	Colorectal Cancer	Colorectal	7.74E-20	-5.25E-01	2.79E-06	-5.78E-01
10968	GSM437093	GSE17536	Colorectal Cancer	Colorectal	1.01E-25	-6.00E-01	9.66E-07	-6.01E-01
10969	GSM437094	GSE17536	Colorectal Cancer	Colorectal	5.08E-37	-7.23E-01	2.25E-11	-8.00E-01
10970	GSM437095	GSE17536	Colorectal Cancer	Colorectal	5.05E-38	-7.33E-01	1.30E-11	-8.09E-01
10971	GSM437096	GSE17536	Colorectal Cancer	Colorectal	6.16E-39	-7.42E-01	1.07E-12	-8.48E-01
10972	GSM437097	GSE17536	Colorectal Cancer	Colorectal	2.72E-31	-6.64E-01	5.67E-11	-7.85E-01
10973	GSM437098	GSE17536	Colorectal Cancer	Colorectal	1.17E-16	-4.79E-01	1.50E-05	-5.38E-01
10974	GSM437099	GSE17536	Colorectal Cancer	Colorectal	2.59E-45	-8.02E-01	2.51E-14	-9.04E-01
10975	GSM437100	GSE17536	Colorectal Cancer	Colorectal	6.67E-09	-3.43E-01	2.05E-03	-4.02E-01
10976	GSM437101	GSE17536	Colorectal Cancer	Colorectal	1.35E-42	-7.77E-01	1.68E-12	-8.41E-01
10977	GSM437102	GSE17536	Colorectal Cancer	Colorectal	5.79E-03	-1.80E-01	4.20E-01	1.50E-01
10978	GSM437103	GSE17536	Colorectal Cancer	Colorectal	8.66E-21	-5.38E-01	9.66E-07	-6.01E-01
10979	GSM437104	GSE17536	Colorectal Cancer	Colorectal	2.01E-46	-8.12E-01	2.51E-14	-9.04E-01
10980	GSM437105	GSE17536	Colorectal Cancer	Colorectal	2.58E-29	-6.42E-01	5.27E-09	-7.05E-01
10981	GSM437106	GSE17536	Colorectal Cancer	Colorectal	1.78E-25	-5.97E-01	5.27E-09	-7.05E-01
10982	GSM437107	GSE17536	Colorectal Cancer	Colorectal	2.51E-36	-7.16E-01	9.30E-11	-7.77E-01
10983	GSM437108	GSE17536	Colorectal Cancer	Colorectal	1.22E-41	-7.68E-01	1.07E-12	-8.48E-01
10984	GSM437109	GSE17536	Colorectal Cancer	Colorectal	3.15E-10	-3.70E-01	3.32E-03	-3.86E-01
10985	GSM437110	GSE17536	Colorectal Cancer	Colorectal	4.74E-29	-6.39E-01	8.21E-09	-6.97E-01
10986	GSM437111	GSE17536	Colorectal Cancer	Colorectal	9.59E-11	-3.80E-01	5.16E-03	-3.71E-01
10987	GSM437112	GSE17536	Colorectal Cancer	Colorectal	1.79E-26	-6.09E-01	2.37E-10	-7.61E-01
10988	GSM437113	GSE17536	Colorectal Cancer	Colorectal	4.52E-23	-5.67E-01	9.66E-07	-6.01E-01
10989	GSM437114	GSE17536	Colorectal Cancer	Colorectal	1.40E-29	-6.45E-01	2.21E-09	-7.21E-01
10990	GSM437115	GSE17536	Colorectal Cancer	Colorectal	9.29E-30	-6.47E-01	1.27E-08	-6.89E-01
10991	GSM437116	GSE17536	Colorectal Cancer	Colorectal	5.23E-14	-4.38E-01	9.36E-04	-4.27E-01
10992	GSM437117	GSE17536	Colorectal Cancer	Colorectal	4.28E-12	-4.05E-01	7.28E-04	-4.34E-01
10993	GSM437118	GSE17536	Colorectal Cancer	Colorectal	9.46E-07	-2.95E-01	2.68E-02	-3.07E-01
10994	GSM437119	GSE17536	Colorectal Cancer	Colorectal	4.63E-01	-6.94E-02	3.22E-01	-1.72E-01
10995	GSM437120	GSE17536	Colorectal Cancer	Colorectal	1.10E-21	-5.50E-01	4.13E-06	-5.69E-01
10996	GSM437121	GSE17536	Colorectal Cancer	Colorectal	1.78E-25	-5.97E-01	8.21E-09	-6.97E-01
10997	GSM437122	GSE17536	Colorectal Cancer	Colorectal	9.06E-19	-5.10E-01	2.87E-05	-5.23E-01
10998	GSM437123	GSE17536	Colorectal Cancer	Colorectal	1.06E-18	-5.09E-01	2.87E-06	-5.77E-01
10999	GSM437124	GSE17536	Colorectal Cancer	Colorectal	5.26E-18	-4.99E-01	6.33E-08	-6.58E-01
11000	GSM437125	GSE17536	Colorectal Cancer	Colorectal	1.10E-15	-4.64E-01	2.72E-06	-5.78E-01
11001	GSM437126	GSE17536	Colorectal Cancer	Colorectal	2.19E-30	-6.54E-01	5.67E-11	-7.85E-01
11002	GSM437127	GSE17536	Colorectal Cancer	Colorectal	7.72E-22	-5.52E-01	3.11E-07	-6.25E-01
11003	GSM437128	GSE17536	Colorectal Cancer	Colorectal	1.39E-32	-6.78E-01	4.44E-08	-6.65E-01
11004	GSM437129	GSE17536	Colorectal Cancer	Colorectal	2.51E-36	-7.16E-01	8.09E-12	-8.17E-01
11005	GSM437130	GSE17536	Colorectal Cancer	Colorectal	3.53E-13	-4.24E-01	1.37E-04	-4.82E-01
11006	GSM437131	GSE17536	Colorectal Cancer	Colorectal	7.37E-24	-5.77E-01	1.27E-08	-6.89E-01
11007	GSM437132	GSE17536	Colorectal Cancer	Colorectal	3.16E-29	-6.41E-01	8.21E-09	-6.97E-01
11008	GSM437133	GSE17536	Colorectal Cancer	Colorectal	6.08E-04	-2.15E-01	2.56E-01	-1.89E-01
11009	GSM437134	GSE17536	Colorectal Cancer	Colorectal	2.02E-18	-5.05E-01	1.12E-05	-5.45E-01
11010	GSM437135	GSE17536	Colorectal Cancer	Colorectal	8.10E-03	-1.74E-01	6.48E-02	-2.67E-01
11011	GSM437136	GSE17536	Colorectal Cancer	Colorectal	3.53E-24	-5.81E-01	4.75E-07	-6.17E-01
11012	GSM437137	GSE17536	Colorectal Cancer	Colorectal	8.66E-21	-5.38E-01	6.40E-07	-6.10E-01
11013	GSM437138	GSE17536	Colorectal Cancer	Colorectal	3.78E-23	-5.68E-01	1.93E-06	-5.86E-01
11014	GSM437139	GSE17536	Colorectal Cancer	Colorectal	1.71E-20	-5.34E-01	4.34E-07	-6.18E-01
11015	GSM437140	GSE17536	Colorectal Cancer	Colorectal	2.55E-37	-7.26E-01	2.97E-12	-8.32E-01
11016	GSM437141	GSE17536	Colorectal Cancer	Colorectal	1.39E-32	-6.78E-01	9.45E-10	-7.37E-01
11017	GSM437142	GSE17536	Colorectal Cancer	Colorectal	1.94E-28	-6.32E-01	8.95E-11	-7.77E-01
11018	GSM437143	GSE17536	Colorectal Cancer	Colorectal	1.18E-30	-6.57E-01	1.46E-10	-7.69E-01
11019	GSM437144	GSE17536	Colorectal Cancer	Colorectal	8.50E-11	-3.81E-01	3.44E-03	-3.85E-01
11020	GSM437145	GSE17536	Colorectal Cancer	Colorectal	7.44E-09	-3.42E-01	1.20E-03	-4.19E-01
11021	GSM437146	GSE17536	Colorectal Cancer	Colorectal	4.95E-36	-7.13E-01	2.37E-10	-7.61E-01

11022	GSM437147	GSE17536	Colorectal Cancer	Colorectal	1.69E-24	-5.85E-01	1.27E-08	-6.89E-01
11023	GSM437148	GSE17536	Colorectal Cancer	Colorectal	2.46E-33	-6.86E-01	2.97E-12	-8.32E-01
11024	GSM437149	GSE17536	Colorectal Cancer	Colorectal	5.54E-20	-5.27E-01	1.33E-06	-5.94E-01
11025	GSM437150	GSE17536	Colorectal Cancer	Colorectal	7.26E-33	-6.81E-01	3.82E-10	-7.52E-01
11026	GSM437151	GSE17536	Colorectal Cancer	Colorectal	1.78E-31	-6.66E-01	9.45E-10	-7.37E-01
11027	GSM437152	GSE17536	Colorectal Cancer	Colorectal	1.85E-07	-3.12E-01	9.68E-03	-3.48E-01
11028	GSM437153	GSE17536	Colorectal Cancer	Colorectal	3.58E-40	-7.54E-01	1.25E-11	-8.10E-01
11029	GSM437154	GSE17536	Colorectal Cancer	Colorectal	9.65E-25	-5.88E-01	1.90E-08	-6.81E-01
11030	GSM437155	GSE17536	Colorectal Cancer	Colorectal	1.12E-07	-3.16E-01	4.00E-03	-3.80E-01
11031	GSM437156	GSE17536	Colorectal Cancer	Colorectal	1.55E-21	-5.48E-01	4.30E-08	-6.65E-01
11032	GSM437157	GSE17536	Colorectal Cancer	Colorectal	1.12E-07	-3.16E-01	1.22E-02	-3.39E-01
11033	GSM437158	GSE17536	Colorectal Cancer	Colorectal	2.76E-34	-6.95E-01	5.27E-09	-7.05E-01
11034	GSM437159	GSE17536	Colorectal Cancer	Colorectal	2.90E-19	-5.17E-01	9.66E-07	-6.01E-01
11035	GSM437160	GSE17536	Colorectal Cancer	Colorectal	9.28E-40	-7.50E-01	7.47E-14	-8.88E-01
11036	GSM437161	GSE17536	Colorectal Cancer	Colorectal	4.81E-09	-3.46E-01	4.16E-04	-4.51E-01
11037	GSM437162	GSE17536	Colorectal Cancer	Colorectal	3.83E-18	-5.01E-01	4.08E-05	-5.14E-01
11038	GSM437163	GSE17536	Colorectal Cancer	Colorectal	6.12E-08	-3.22E-01	4.15E-03	-3.78E-01
11039	GSM437164	GSE17536	Colorectal Cancer	Colorectal	6.80E-27	-6.14E-01	5.91E-10	-7.45E-01
11040	GSM437165	GSE17536	Colorectal Cancer	Colorectal	1.35E-42	-7.77E-01	4.82E-12	-8.25E-01
11041	GSM437166	GSE17536	Colorectal Cancer	Colorectal	2.70E-13	-4.26E-01	9.76E-04	-4.25E-01
11042	GSM437167	GSE17536	Colorectal Cancer	Colorectal	1.06E-28	-6.35E-01	3.68E-10	-7.53E-01
11043	GSM437168	GSE17536	Colorectal Cancer	Colorectal	4.54E-40	-7.53E-01	6.23E-13	-8.57E-01
11044	GSM437169	GSE17536	Colorectal Cancer	Colorectal	6.80E-27	-6.14E-01	9.11E-10	-7.37E-01
11045	GSM437170	GSE17536	Colorectal Cancer	Colorectal	9.06E-19	-5.10E-01	9.66E-07	-6.01E-01
11046	GSM437171	GSE17536	Colorectal Cancer	Colorectal	9.46E-07	-2.95E-01	9.85E-03	-3.47E-01
11047	GSM437172	GSE17536	Colorectal Cancer	Colorectal	2.60E-25	-5.95E-01	2.09E-07	-6.34E-01
11048	GSM437173	GSE17536	Colorectal Cancer	Colorectal	5.28E-13	-4.21E-01	2.38E-04	-4.67E-01
11049	GSM437174	GSE17536	Colorectal Cancer	Colorectal	3.15E-10	-3.70E-01	2.38E-04	-4.67E-01
11050	GSM437175	GSE17536	Colorectal Cancer	Colorectal	1.54E-01	-1.08E-01	3.61E-01	1.63E-01
11051	GSM437176	GSE17536	Colorectal Cancer	Colorectal	3.36E-16	-4.72E-01	1.93E-06	-5.86E-01
11052	GSM437177	GSE17536	Colorectal Cancer	Colorectal	1.58E-22	-5.61E-01	5.27E-09	-7.05E-01
11053	GSM437178	GSE17536	Colorectal Cancer	Colorectal	9.19E-12	-3.99E-01	3.12E-04	-4.59E-01
11054	GSM437179	GSE17536	Colorectal Cancer	Colorectal	1.42E-27	-6.22E-01	3.48E-09	-7.13E-01
11055	GSM437180	GSE17536	Colorectal Cancer	Colorectal	1.84E-16	-4.76E-01	6.40E-07	-6.10E-01
11056	GSM437181	GSE17536	Colorectal Cancer	Colorectal	8.86E-06	-2.70E-01	1.35E-01	-2.29E-01
11057	GSM437182	GSE17536	Colorectal Cancer	Colorectal	5.26E-18	-4.99E-01	3.88E-05	-5.15E-01
11058	GSM437183	GSE17536	Colorectal Cancer	Colorectal	5.60E-27	-6.15E-01	1.90E-08	-6.81E-01
11059	GSM437184	GSE17536	Colorectal Cancer	Colorectal	1.78E-25	-5.97E-01	3.48E-09	-7.13E-01
11060	GSM437185	GSE17536	Colorectal Cancer	Colorectal	3.74E-35	-7.04E-01	1.46E-10	-7.69E-01
11061	GSM437186	GSE17536	Colorectal Cancer	Colorectal	8.70E-29	-6.36E-01	2.91E-08	-6.73E-01
11062	GSM437187	GSE17536	Colorectal Cancer	Colorectal	1.83E-23	-5.72E-01	1.23E-08	-6.90E-01
11063	GSM437188	GSE17536	Colorectal Cancer	Colorectal	2.79E-18	-5.03E-01	3.02E-07	-6.26E-01
11064	GSM437189	GSE17536	Colorectal Cancer	Colorectal	2.60E-14	-4.42E-01	1.37E-04	-4.82E-01
11065	GSM437190	GSE17536	Colorectal Cancer	Colorectal	3.32E-07	-3.06E-01	4.15E-03	-3.78E-01
11066	GSM437191	GSE17536	Colorectal Cancer	Colorectal	8.44E-18	-4.96E-01	2.79E-06	-5.78E-01
11067	GSM437192	GSE17536	Colorectal Cancer	Colorectal	1.02E-12	-4.16E-01	3.34E-04	-4.57E-01
11068	GSM437193	GSE17536	Colorectal Cancer	Colorectal	1.01E-25	-6.00E-01	3.68E-10	-7.53E-01
11069	GSM437194	GSE17536	Colorectal Cancer	Colorectal	7.79E-39	-7.41E-01	1.35E-11	-8.08E-01
11070	GSM437195	GSE17536	Colorectal Cancer	Colorectal	5.19E-21	-5.41E-01	6.33E-08	-6.58E-01
11071	GSM437196	GSE17536	Colorectal Cancer	Colorectal	7.70E-19	-5.11E-01	9.57E-08	-6.50E-01
11072	GSM437197	GSE17536	Colorectal Cancer	Colorectal	1.03E-20	-5.37E-01	2.95E-06	-5.77E-01
11073	GSM437198	GSE17536	Colorectal Cancer	Colorectal	3.42E-03	-1.88E-01	2.83E-01	-1.82E-01
11074	GSM437199	GSE17536	Colorectal Cancer	Colorectal	1.11E-03	-2.06E-01	6.65E-02	-2.66E-01
11075	GSM437200	GSE17536	Colorectal Cancer	Colorectal	6.89E-14	-4.36E-01	1.37E-04	-4.82E-01
11076	GSM437201	GSE17536	Colorectal Cancer	Colorectal	2.40E-02	1.53E-01	2.61E-02	3.09E-01
11077	GSM437202	GSE17536	Colorectal Cancer	Colorectal	7.72E-22	-5.52E-01	1.40E-07	-6.42E-01
11078	GSM437203	GSE17536	Colorectal Cancer	Colorectal	5.36E-09	-3.45E-01	1.59E-03	-4.10E-01
11079	GSM437204	GSE17536	Colorectal Cancer	Colorectal	1.24E-38	7.39E-01	4.65E-16	9.60E-01
11080	GSM437205	GSE17536	Colorectal Cancer	Colorectal	3.44E-14	-4.40E-01	5.49E-05	-5.06E-01
11081	GSM437206	GSE17536	Colorectal Cancer	Colorectal	4.69E-20	-5.28E-01	3.11E-07	-6.25E-01
11082	GSM437207	GSE17536	Colorectal Cancer	Colorectal	1.10E-15	-4.64E-01	2.05E-05	-5.31E-01
11083	GSM437208	GSE17536	Colorectal Cancer	Colorectal	1.40E-29	-6.45E-01	5.27E-09	-7.05E-01
11084	GSM437209	GSE17536	Colorectal Cancer	Colorectal	2.42E-05	-2.58E-01	2.68E-02	-3.07E-01
11085	GSM437210	GSE17536	Colorectal Cancer	Colorectal	3.41E-19	-5.16E-01	1.40E-07	-6.42E-01
11086	GSM437211	GSE17536	Colorectal Cancer	Colorectal	2.09E-19	-5.19E-01	1.93E-06	-5.86E-01
11087	GSM437212	GSE17536	Colorectal Cancer	Colorectal	4.01E-17	-4.86E-01	7.95E-06	-5.54E-01
11088	GSM437213	GSE17536	Colorectal Cancer	Colorectal	3.79E-27	-6.17E-01	2.09E-07	-6.34E-01
11089	GSM437214	GSE17536	Colorectal Cancer	Colorectal	8.10E-12	-4.00E-01	7.28E-04	-4.34E-01
11090	GSM437215	GSE17536	Colorectal Cancer	Colorectal	6.55E-20	-5.26E-01	6.59E-07	-6.10E-01
11091	GSM437216	GSE17536	Colorectal Cancer	Colorectal	9.28E-40	-7.50E-01	1.35E-11	-8.08E-01
11092	GSM437217	GSE17536	Colorectal Cancer	Colorectal	1.22E-25	-5.99E-01	8.21E-09	-6.97E-01
11093	GSM437218	GSE17536	Colorectal Cancer	Colorectal	2.13E-32	-6.76E-01	9.45E-10	-7.37E-01
11094	GSM437219	GSE17536	Colorectal Cancer	Colorectal	2.51E-38	-7.36E-01	5.67E-11	-7.85E-01
11095	GSM437220	GSE17536	Colorectal Cancer	Colorectal	6.48E-22	-5.53E-01	3.21E-07	-6.25E-01

11096	GSM437221	GSE17536	Colorectal Cancer	Colorectal	8.01E-25	-5.89E-01	6.33E-08	-6.58E-01
11097	GSM437222	GSE17536	Colorectal Cancer	Colorectal	6.30E-05	-2.46E-01	1.20E-02	-3.40E-01
11098	GSM437223	GSE17536	Colorectal Cancer	Colorectal	3.62E-05	-2.53E-01	1.80E-02	-3.24E-01
11099	GSM437224	GSE17536	Colorectal Cancer	Colorectal	3.27E-18	-5.02E-01	2.09E-07	-6.34E-01
11100	GSM437225	GSE17536	Colorectal Cancer	Colorectal	3.29E-08	-3.28E-01	3.19E-03	-3.87E-01
11101	GSM437226	GSE17536	Colorectal Cancer	Colorectal	6.49E-07	2.99E-01	9.83E-05	4.91E-01
11102	GSM437227	GSE17536	Colorectal Cancer	Colorectal	2.90E-19	-5.17E-01	9.39E-07	-6.02E-01
11103	GSM437228	GSE17536	Colorectal Cancer	Colorectal	1.42E-34	-6.98E-01	3.82E-10	-7.52E-01
11104	GSM437229	GSE17536	Colorectal Cancer	Colorectal	1.69E-24	-5.85E-01	2.09E-07	-6.34E-01
11105	GSM437230	GSE17536	Colorectal Cancer	Colorectal	4.86E-39	-7.43E-01	2.37E-10	-7.61E-01
11106	GSM437231	GSE17536	Colorectal Cancer	Colorectal	1.36E-16	-4.78E-01	3.90E-06	-5.70E-01
11107	GSM437232	GSE17536	Colorectal Cancer	Colorectal	1.49E-39	-7.48E-01	4.63E-12	-8.25E-01
11108	GSM437233	GSE17536	Colorectal Cancer	Colorectal	1.00E-16	-4.80E-01	4.16E-04	-4.51E-01
11109	GSM437234	GSE17536	Colorectal Cancer	Colorectal	2.21E-10	-3.73E-01	1.03E-04	-4.90E-01
11110	GSM437235	GSE17536	Colorectal Cancer	Colorectal	8.29E-09	-3.41E-01	2.44E-04	-4.66E-01
11111	GSM437236	GSE17536	Colorectal Cancer	Colorectal	4.28E-34	-6.93E-01	2.28E-10	-7.61E-01
11112	GSM437237	GSE17536	Colorectal Cancer	Colorectal	1.06E-28	-6.35E-01	3.48E-09	-7.13E-01
11113	GSM437238	GSE17536	Colorectal Cancer	Colorectal	1.42E-27	-6.22E-01	1.96E-08	-6.81E-01
11114	GSM437239	GSE17536	Colorectal Cancer	Colorectal	5.28E-13	-4.21E-01	3.88E-05	-5.15E-01
11115	GSM437240	GSE17536	Colorectal Cancer	Colorectal	2.44E-24	-5.83E-01	1.40E-07	-6.42E-01
11116	GSM437241	GSE17536	Colorectal Cancer	Colorectal	3.96E-14	-4.39E-01	1.50E-05	-5.38E-01
11117	GSM437242	GSE17536	Colorectal Cancer	Colorectal	1.19E-13	-4.32E-01	1.77E-04	-4.75E-01
11118	GSM437243	GSE17536	Colorectal Cancer	Colorectal	3.43E-17	-4.87E-01	3.11E-07	-6.25E-01
11119	GSM437244	GSE17536	Colorectal Cancer	Colorectal	2.94E-24	-5.82E-01	1.44E-07	-6.41E-01
11120	GSM437245	GSE17536	Colorectal Cancer	Colorectal	4.01E-17	-4.86E-01	2.09E-07	-6.34E-01
11121	GSM437246	GSE17536	Colorectal Cancer	Colorectal	2.64E-32	-6.75E-01	1.27E-08	-6.89E-01
11122	GSM437247	GSE17536	Colorectal Cancer	Colorectal	1.97E-12	-4.11E-01	1.31E-04	-4.83E-01
11123	GSM437248	GSE17536	Colorectal Cancer	Colorectal	8.47E-41	-7.60E-01	9.30E-11	-7.77E-01
11124	GSM437249	GSE17536	Colorectal Cancer	Colorectal	1.47E-25	-5.98E-01	3.02E-07	-6.26E-01
11125	GSM437250	GSE17536	Colorectal Cancer	Colorectal	3.19E-02	-1.47E-01	2.25E-01	-1.97E-01
11126	GSM437251	GSE17536	Colorectal Cancer	Colorectal	8.10E-12	-4.00E-01	1.98E-03	-4.03E-01
11127	GSM437252	GSE17536	Colorectal Cancer	Colorectal	2.21E-34	-6.96E-01	3.60E-09	-7.12E-01
11128	GSM437253	GSE17536	Colorectal Cancer	Colorectal	3.03E-39	-7.45E-01	1.68E-12	-8.41E-01
11129	GSM437254	GSE17536	Colorectal Cancer	Colorectal	1.04E-03	-2.07E-01	3.93E-01	-1.56E-01
11130	GSM437255	GSE17536	Colorectal Cancer	Colorectal	1.01E-36	-7.20E-01	5.64E-09	-7.04E-01
11131	GSM437256	GSE17536	Colorectal Cancer	Colorectal	7.11E-29	-6.37E-01	6.54E-08	-6.57E-01
11132	GSM437257	GSE17536	Colorectal Cancer	Colorectal	1.98E-06	-2.87E-01	6.56E-02	-2.67E-01
11133	GSM437258	GSE17536	Colorectal Cancer	Colorectal	2.76E-34	-6.95E-01	9.11E-10	-7.37E-01
11134	GSM437259	GSE17536	Colorectal Cancer	Colorectal	8.84E-24	-5.76E-01	6.75E-08	-6.57E-01
11135	GSM437260	GSE17536	Colorectal Cancer	Colorectal	1.58E-16	-4.77E-01	5.49E-05	-5.06E-01
11136	GSM437261	GSE17536	Colorectal Cancer	Colorectal	8.03E-38	-7.31E-01	4.82E-12	-8.25E-01
11137	GSM437262	GSE17536	Colorectal Cancer	Colorectal	7.91E-14	-4.35E-01	1.81E-04	-4.74E-01
11138	GSM437263	GSE17536	Colorectal Cancer	Colorectal	4.47E-10	-3.67E-01	5.52E-04	-4.43E-01
11139	GSM437264	GSE17536	Colorectal Cancer	Colorectal	1.22E-35	-7.09E-01	1.75E-12	-8.41E-01
11140	GSM437265	GSE17536	Colorectal Cancer	Colorectal	3.93E-05	-2.52E-01	1.03E-01	-2.44E-01
11141	GSM437266	GSE17536	Colorectal Cancer	Colorectal	2.02E-20	-5.33E-01	5.63E-05	-5.05E-01
11142	GSM437267	GSE17536	Colorectal Cancer	Colorectal	3.68E-21	-5.43E-01	4.01E-06	-5.70E-01
11143	GSM437268	GSE17536	Colorectal Cancer	Colorectal	2.02E-37	-7.27E-01	9.30E-11	-7.77E-01
11144	GSM437269	GSE17536	Colorectal Cancer	Colorectal	6.64E-34	-6.91E-01	2.28E-10	-7.61E-01
11145	GSM437270	GSE17537	Colorectal Cancer	Colorectal	9.51E-16	-4.65E-01	4.01E-06	-5.70E-01
11146	GSM437271	GSE17537	Colorectal Cancer	Colorectal	6.43E-28	-6.26E-01	2.21E-09	-7.21E-01
11147	GSM437272	GSE17537	Colorectal Cancer	Colorectal	1.38E-10	-3.77E-01	4.16E-04	-4.51E-01
11148	GSM437273	GSE17537	Colorectal Cancer	Colorectal	2.21E-34	-6.96E-01	5.89E-11	-7.84E-01
11149	GSM437274	GSE17537	Colorectal Cancer	Colorectal	6.48E-23	-5.65E-01	2.09E-07	-6.34E-01
11150	GSM437275	GSE17537	Colorectal Cancer	Colorectal	7.32E-40	-7.51E-01	2.16E-11	-8.01E-01
11151	GSM437276	GSE17537	Colorectal Cancer	Colorectal	1.50E-06	-2.90E-01	4.07E-03	-3.79E-01
11152	GSM437277	GSE17537	Colorectal Cancer	Colorectal	9.29E-30	-6.47E-01	2.29E-09	-7.21E-01
11153	GSM437278	GSE17537	Colorectal Cancer	Colorectal	7.65E-32	-6.70E-01	5.67E-11	-7.85E-01
11154	GSM437279	GSE17537	Colorectal Cancer	Colorectal	1.48E-15	-4.62E-01	5.36E-05	-5.07E-01
11155	GSM437280	GSE17537	Colorectal Cancer	Colorectal	1.08E-10	-3.79E-01	1.17E-03	-4.20E-01
11156	GSM437281	GSE17537	Colorectal Cancer	Colorectal	2.24E-03	-1.95E-01	6.48E-02	-2.67E-01
11157	GSM437282	GSE17537	Colorectal Cancer	Colorectal	2.84E-20	-5.31E-01	1.90E-08	-6.81E-01
11158	GSM437283	GSE17537	Colorectal Cancer	Colorectal	2.46E-33	-6.86E-01	1.45E-09	-7.29E-01
11159	GSM437284	GSE17537	Colorectal Cancer	Colorectal	5.16E-03	-1.82E-01	3.01E-01	-1.77E-01
11160	GSM437285	GSE17537	Colorectal Cancer	Colorectal	4.04E-32	-6.73E-01	5.89E-11	-7.84E-01
11161	GSM437286	GSE17537	Colorectal Cancer	Colorectal	1.52E-07	-3.13E-01	9.21E-02	-2.50E-01
11162	GSM437287	GSE17537	Colorectal Cancer	Colorectal	9.74E-36	-7.10E-01	2.25E-11	-8.00E-01
11163	GSM437288	GSE17537	Colorectal Cancer	Colorectal	3.54E-28	-6.29E-01	6.33E-08	-6.58E-01
11164	GSM437289	GSE17537	Colorectal Cancer	Colorectal	1.99E-38	-7.37E-01	8.09E-12	-8.17E-01
11165	GSM437290	GSE17537	Colorectal Cancer	Colorectal	4.68E-35	-7.03E-01	2.37E-10	-7.61E-01
11166	GSM437291	GSE17537	Colorectal Cancer	Colorectal	2.39E-35	-7.06E-01	2.97E-12	-8.32E-01
11167	GSM437292	GSE17537	Colorectal Cancer	Colorectal	1.77E-34	-6.97E-01	9.11E-10	-7.37E-01
11168	GSM437293	GSE17537	Colorectal Cancer	Colorectal	6.28E-12	-4.02E-01	7.13E-04	-4.35E-01
11169	GSM437294	GSE17537	Colorectal Cancer	Colorectal	2.16E-17	-4.90E-01	4.01E-06	-5.70E-01

11170	GSM437295	GSE17537	Colorectal Cancer	Colorectal	4.08E-30	-6.51E-01	3.68E-10	-7.53E-01
11171	GSM437296	GSE17537	Colorectal Cancer	Colorectal	1.77E-34	-6.97E-01	2.29E-09	-7.21E-01
11172	GSM437297	GSE17537	Colorectal Cancer	Colorectal	1.22E-10	-3.78E-01	7.36E-05	-4.98E-01
11173	GSM437298	GSE17537	Colorectal Cancer	Colorectal	8.44E-49	-8.32E-01	1.31E-13	-8.80E-01
11174	GSM437299	GSE17537	Colorectal Cancer	Colorectal	2.60E-25	-5.95E-01	1.96E-08	-6.81E-01
11175	GSM437300	GSE17537	Colorectal Cancer	Colorectal	4.25E-24	-5.80E-01	1.40E-07	-6.42E-01
11176	GSM437301	GSE17537	Colorectal Cancer	Colorectal	9.54E-03	-1.71E-01	3.22E-01	-1.72E-01
11177	GSM437302	GSE17537	Colorectal Cancer	Colorectal	5.81E-29	-6.38E-01	8.49E-09	-6.97E-01
11178	GSM437303	GSE17537	Colorectal Cancer	Colorectal	7.79E-39	-7.41E-01	2.16E-11	-8.01E-01
11179	GSM437304	GSE17537	Colorectal Cancer	Colorectal	1.19E-13	-4.32E-01	7.36E-05	-4.98E-01
11180	GSM437305	GSE17537	Colorectal Cancer	Colorectal	2.49E-10	-3.72E-01	3.25E-02	-2.99E-01
11181	GSM437306	GSE17537	Colorectal Cancer	Colorectal	5.57E-01	-6.05E-02	5.03E-01	-1.34E-01
11182	GSM437307	GSE17537	Colorectal Cancer	Colorectal	1.18E-02	-1.67E-01	3.22E-01	-1.72E-01
11183	GSM437308	GSE17537	Colorectal Cancer	Colorectal	5.57E-19	-5.13E-01	2.09E-07	-6.34E-01
11184	GSM437309	GSE17537	Colorectal Cancer	Colorectal	8.01E-25	-5.89E-01	4.47E-07	-6.18E-01
11185	GSM437310	GSE17537	Colorectal Cancer	Colorectal	2.51E-36	-7.16E-01	4.82E-12	-8.25E-01
11186	GSM437311	GSE17537	Colorectal Cancer	Colorectal	9.26E-23	-5.63E-01	1.41E-06	-5.93E-01
11187	GSM437312	GSE17537	Colorectal Cancer	Colorectal	6.90E-48	-8.24E-01	1.68E-12	-8.41E-01
11188	GSM437313	GSE17537	Colorectal Cancer	Colorectal	8.39E-15	-4.50E-01	3.98E-05	-5.14E-01
11189	GSM437314	GSE17537	Colorectal Cancer	Colorectal	1.58E-16	-4.77E-01	1.07E-05	-5.47E-01
11190	GSM437315	GSE17537	Colorectal Cancer	Colorectal	3.53E-13	4.24E-01	7.53E-06	5.55E-01
11191	GSM437316	GSE17537	Colorectal Cancer	Colorectal	3.42E-03	-1.88E-01	1.75E-01	-2.13E-01
11192	GSM437317	GSE17537	Colorectal Cancer	Colorectal	4.62E-13	-4.22E-01	5.52E-04	-4.43E-01
11193	GSM437318	GSE17537	Colorectal Cancer	Colorectal	5.91E-07	-3.00E-01	1.46E-02	-3.32E-01
11194	GSM437319	GSE17537	Colorectal Cancer	Colorectal	1.18E-11	-3.97E-01	1.31E-04	-4.83E-01
11195	GSM437320	GSE17537	Colorectal Cancer	Colorectal	2.94E-24	-5.82E-01	4.30E-08	-6.65E-01
11196	GSM437321	GSE17537	Colorectal Cancer	Colorectal	2.39E-35	-7.06E-01	5.89E-11	-7.84E-01
11197	GSM437322	GSE17537	Colorectal Cancer	Colorectal	6.43E-28	-6.26E-01	5.64E-09	-7.04E-01
11198	GSM437323	GSE17537	Colorectal Cancer	Colorectal	4.32E-28	-6.28E-01	8.21E-09	-6.97E-01
11199	GSM437324	GSE17537	Colorectal Cancer	Colorectal	8.03E-37	-7.21E-01	8.09E-12	-8.17E-01
11200	GSM452148	GSE18088	Colorectal Cancer	Colorectal	6.97E-04	-2.13E-01	1.19E-01	-2.36E-01
11201	GSM452149	GSE18088	Colorectal Cancer	Colorectal	7.74E-20	-5.25E-01	2.09E-07	-6.34E-01
11202	GSM452150	GSE18088	Colorectal Cancer	Colorectal	2.99E-14	-4.41E-01	2.94E-05	-5.22E-01
11203	GSM452151	GSE18088	Colorectal Cancer	Colorectal	5.76E-40	-7.52E-01	2.97E-12	-8.32E-01
11204	GSM452152	GSE18088	Colorectal Cancer	Colorectal	1.78E-31	-6.66E-01	1.49E-07	-6.41E-01
11205	GSM452153	GSE18088	Colorectal Cancer	Colorectal	1.22E-35	-7.09E-01	8.09E-12	-8.17E-01
11206	GSM452154	GSE18088	Colorectal Cancer	Colorectal	1.53E-23	-5.73E-01	2.15E-07	-6.33E-01
11207	GSM452155	GSE18088	Colorectal Cancer	Colorectal	1.83E-23	-5.72E-01	4.47E-07	-6.18E-01
11208	GSM452156	GSE18088	Colorectal Cancer	Colorectal	4.28E-12	-4.05E-01	2.49E-03	-3.96E-01
11209	GSM452157	GSE18088	Colorectal Cancer	Colorectal	4.74E-29	-6.39E-01	5.70E-10	-7.45E-01
11210	GSM452158	GSE18088	Colorectal Cancer	Colorectal	6.89E-14	-4.36E-01	4.25E-04	-4.50E-01
11211	GSM452159	GSE18088	Colorectal Cancer	Colorectal	2.20E-23	-5.71E-01	5.27E-09	-7.05E-01
11212	GSM452160	GSE18088	Colorectal Cancer	Colorectal	1.08E-10	-3.79E-01	5.64E-04	-4.42E-01
11213	GSM452161	GSE18088	Colorectal Cancer	Colorectal	7.86E-13	-4.18E-01	9.56E-04	-4.26E-01
11214	GSM452162	GSE18088	Colorectal Cancer	Colorectal	1.36E-16	-4.78E-01	3.12E-04	-4.59E-01
11215	GSM452163	GSE18088	Colorectal Cancer	Colorectal	1.22E-20	-5.36E-01	1.93E-06	-5.86E-01
11216	GSM452164	GSE18088	Colorectal Cancer	Colorectal	1.06E-18	-5.09E-01	1.93E-06	-5.86E-01
11217	GSM452165	GSE18088	Colorectal Cancer	Colorectal	4.81E-09	-3.46E-01	3.32E-03	-3.86E-01
11218	GSM452166	GSE18088	Colorectal Cancer	Colorectal	1.49E-39	-7.48E-01	2.20E-10	-7.62E-01
11219	GSM452167	GSE18088	Colorectal Cancer	Colorectal	8.44E-18	-4.96E-01	2.94E-05	-5.22E-01
11220	GSM452168	GSE18088	Colorectal Cancer	Colorectal	3.27E-18	-5.02E-01	2.91E-08	-6.73E-01
11221	GSM452169	GSE18088	Colorectal Cancer	Colorectal	1.16E-24	-5.87E-01	1.27E-08	-6.89E-01
11222	GSM452170	GSE18088	Colorectal Cancer	Colorectal	2.39E-43	-7.84E-01	1.07E-12	-8.48E-01
11223	GSM452171	GSE18088	Colorectal Cancer	Colorectal	1.25E-18	-5.08E-01	5.74E-06	-5.61E-01
11224	GSM452172	GSE18088	Colorectal Cancer	Colorectal	6.39E-37	-7.22E-01	2.16E-11	-8.01E-01
11225	GSM452173	GSE18088	Colorectal Cancer	Colorectal	5.53E-12	-4.03E-01	9.56E-04	-4.26E-01
11226	GSM452174	GSE18088	Colorectal Cancer	Colorectal	1.37E-07	-3.14E-01	2.64E-02	-3.08E-01
11227	GSM452175	GSE18088	Colorectal Cancer	Colorectal	2.90E-28	-6.30E-01	5.91E-10	-7.45E-01
11228	GSM452176	GSE18088	Colorectal Cancer	Colorectal	1.05E-42	-7.78E-01	2.09E-13	-8.73E-01
11229	GSM452177	GSE18088	Colorectal Cancer	Colorectal	6.35E-17	-4.83E-01	2.87E-05	-5.23E-01
11230	GSM452178	GSE18088	Colorectal Cancer	Colorectal	3.19E-26	-6.06E-01	1.49E-07	-6.41E-01
11231	GSM452179	GSE18088	Colorectal Cancer	Colorectal	2.60E-14	-4.42E-01	4.16E-04	-4.51E-01
11232	GSM452180	GSE18088	Colorectal Cancer	Colorectal	2.26E-14	-4.43E-01	1.54E-05	-5.38E-01
11233	GSM452181	GSE18088	Colorectal Cancer	Colorectal	4.42E-06	-2.78E-01	1.61E-01	-2.18E-01
11234	GSM452182	GSE18088	Colorectal Cancer	Colorectal	3.68E-21	-5.43E-01	1.41E-06	-5.93E-01
11235	GSM452183	GSE18088	Colorectal Cancer	Colorectal	6.48E-22	-5.53E-01	2.95E-06	-5.77E-01
11236	GSM452184	GSE18088	Colorectal Cancer	Colorectal	7.74E-20	-5.25E-01	1.33E-06	-5.94E-01
11237	GSM452185	GSE18088	Colorectal Cancer	Colorectal	1.59E-28	-6.33E-01	5.27E-09	-7.05E-01
11238	GSM452186	GSE18088	Colorectal Cancer	Colorectal	3.66E-07	-3.05E-01	2.24E-02	-3.15E-01
11239	GSM452187	GSE18088	Colorectal Cancer	Colorectal	3.79E-27	-6.17E-01	4.44E-08	-6.65E-01
11240	GSM452188	GSE18088	Colorectal Cancer	Colorectal	4.37E-21	-5.42E-01	2.21E-09	-7.21E-01
11241	GSM452189	GSE18088	Colorectal Cancer	Colorectal	1.10E-15	-4.64E-01	3.90E-06	-5.70E-01
11242	GSM452190	GSE18088	Colorectal Cancer	Colorectal	5.91E-07	3.00E-01	5.40E-04	4.43E-01
11243	GSM452191	GSE18088	Colorectal Cancer	Colorectal	9.18E-08	-3.18E-01	4.25E-04	-4.50E-01

11244	GSM452192	GSE18088	Colorectal Cancer	Colorectal	1.80E-13	-4.29E-01	3.98E-05	-5.14E-01
11245	GSM452193	GSE18088	Colorectal Cancer	Colorectal	1.48E-26	-6.10E-01	2.29E-09	-7.21E-01
11246	GSM452194	GSE18088	Colorectal Cancer	Colorectal	5.27E-28	-6.27E-01	6.54E-08	-6.57E-01
11247	GSM452195	GSE18088	Colorectal Cancer	Colorectal	1.37E-13	-4.31E-01	2.33E-04	-4.67E-01
11248	GSM452196	GSE18088	Colorectal Cancer	Colorectal	7.11E-10	-3.63E-01	3.25E-03	-3.87E-01
11249	GSM452197	GSE18088	Colorectal Cancer	Colorectal	4.49E-18	-5.00E-01	1.07E-05	-5.47E-01
11250	GSM452198	GSE18088	Colorectal Cancer	Colorectal	5.81E-29	-6.38E-01	2.16E-11	-8.01E-01
11251	GSM452199	GSE18088	Colorectal Cancer	Colorectal	5.27E-06	-2.76E-01	1.04E-02	-3.45E-01
11252	GSM452200	GSE18088	Colorectal Cancer	Colorectal	5.05E-38	-7.33E-01	2.09E-13	-8.73E-01
11253	GSM452552	GSE18105	Colorectal Cancer	Colorectal	6.33E-10	-3.64E-01	1.46E-02	-3.32E-01
11254	GSM452553	GSE18105	Colorectal Cancer	Colorectal	2.20E-42	-7.75E-01	3.77E-13	-8.64E-01
11255	GSM452554	GSE18105	Colorectal Cancer	Colorectal	1.48E-14	-4.46E-01	4.25E-04	-4.50E-01
11256	GSM452555	GSE18105	Colorectal Cancer	Colorectal	1.37E-06	-2.91E-01	2.38E-02	-3.12E-01
11257	GSM452556	GSE18105	Colorectal Cancer	Colorectal	5.02E-10	-3.66E-01	1.56E-03	-4.11E-01
11258	GSM452557	GSE18105	Colorectal Cancer	Colorectal	9.51E-16	-4.65E-01	1.01E-04	-4.90E-01
11259	GSM452558	GSE18105	Colorectal Cancer	Colorectal	9.19E-12	-3.99E-01	6.98E-04	-4.36E-01
11260	GSM452559	GSE18105	Colorectal Cancer	Colorectal	1.18E-11	-3.97E-01	1.81E-04	-4.74E-01
11261	GSM452560	GSE18105	Colorectal Cancer	Colorectal	3.09E-13	-4.25E-01	1.37E-04	-4.82E-01
11262	GSM452561	GSE18105	Colorectal Cancer	Colorectal	3.31E-12	-4.07E-01	7.44E-04	-4.34E-01
11263	GSM452562	GSE18105	Colorectal Cancer	Colorectal	1.72E-29	-6.44E-01	1.23E-08	-6.90E-01
11264	GSM452563	GSE18105	Colorectal Cancer	Colorectal	5.44E-22	-5.54E-01	9.57E-08	-6.50E-01
11265	GSM452564	GSE18105	Colorectal Cancer	Colorectal	4.44E-07	-3.03E-01	1.80E-02	-3.24E-01
11266	GSM452565	GSE18105	Colorectal Cancer	Colorectal	3.17E-38	-7.35E-01	3.77E-13	-8.64E-01
11267	GSM452566	GSE18105	Colorectal Cancer	Colorectal	1.30E-21	-5.49E-01	2.91E-08	-6.73E-01
11268	GSM452567	GSE18105	Colorectal Cancer	Colorectal	7.07E-16	-4.67E-01	5.63E-05	-5.05E-01
11269	GSM452568	GSE18105	Colorectal Cancer	Colorectal	4.67E-17	-4.85E-01	3.34E-04	-4.57E-01
11270	GSM452569	GSE18105	Colorectal Cancer	Colorectal	3.27E-18	-5.02E-01	1.41E-06	-5.93E-01
11271	GSM452570	GSE18105	Colorectal Cancer	Colorectal	7.75E-23	-5.64E-01	2.15E-07	-6.33E-01
11272	GSM452571	GSE18105	Colorectal Cancer	Colorectal	6.67E-09	-3.43E-01	1.25E-03	-4.18E-01
11273	GSM452572	GSE18105	Colorectal Cancer	Colorectal	1.10E-21	-5.50E-01	2.05E-06	-5.85E-01
11274	GSM452573	GSE18105	Colorectal Cancer	Colorectal	1.98E-09	-3.54E-01	6.39E-03	-3.63E-01
11275	GSM452574	GSE18105	Colorectal Cancer	Colorectal	7.86E-13	-4.18E-01	3.98E-05	-5.14E-01
11276	GSM452575	GSE18105	Colorectal Cancer	Colorectal	5.60E-27	-6.15E-01	3.68E-10	-7.53E-01
11277	GSM452576	GSE18105	Colorectal Cancer	Colorectal	2.47E-09	-3.52E-01	1.34E-04	-4.83E-01
11278	GSM452577	GSE18105	Colorectal Cancer	Colorectal	1.16E-24	-5.87E-01	6.75E-08	-6.57E-01
11279	GSM452578	GSE18105	Colorectal Cancer	Colorectal	2.63E-23	-5.70E-01	5.74E-06	-5.61E-01
11280	GSM452579	GSE18105	Colorectal Cancer	Colorectal	2.16E-17	-4.90E-01	1.54E-05	-5.38E-01
11281	GSM452580	GSE18105	Colorectal Cancer	Colorectal	1.05E-42	-7.78E-01	2.18E-13	-8.72E-01
11282	GSM452581	GSE18105	Colorectal Cancer	Colorectal	4.31E-09	-3.47E-01	3.12E-04	-4.59E-01
11283	GSM452582	GSE18105	Colorectal Cancer	Colorectal	3.15E-23	-5.69E-01	4.44E-08	-6.65E-01
11284	GSM452583	GSE18105	Colorectal Cancer	Colorectal	8.32E-26	-6.01E-01	3.68E-10	-7.53E-01
11285	GSM452584	GSE18105	Colorectal Cancer	Colorectal	1.39E-32	-6.78E-01	1.46E-10	-7.69E-01
11286	GSM452585	GSE18105	Colorectal Cancer	Colorectal	1.30E-28	-6.34E-01	1.27E-08	-6.89E-01
11287	GSM452586	GSE18105	Colorectal Cancer	Colorectal	7.37E-24	-5.77E-01	4.47E-07	-6.18E-01
11288	GSM452587	GSE18105	Colorectal Cancer	Colorectal	2.84E-20	-5.31E-01	2.15E-07	-6.33E-01
11289	GSM452588	GSE18105	Colorectal Cancer	Colorectal	2.39E-35	-7.06E-01	1.45E-09	-7.29E-01
11290	GSM452589	GSE18105	Colorectal Cancer	Colorectal	8.62E-17	-4.81E-01	2.16E-05	-5.30E-01
11291	GSM452590	GSE18105	Colorectal Cancer	Colorectal	3.78E-23	-5.68E-01	2.79E-06	-5.78E-01
11292	GSM452591	GSE18105	Colorectal Cancer	Colorectal	1.10E-15	-4.64E-01	3.98E-05	-5.14E-01
11293	GSM452592	GSE18105	Colorectal Cancer	Colorectal	4.98E-05	-2.49E-01	2.64E-02	-3.08E-01
11294	GSM452593	GSE18105	Colorectal Cancer	Colorectal	2.19E-21	-5.46E-01	1.40E-07	-6.42E-01
11295	GSM452594	GSE18105	Colorectal Cancer	Colorectal	2.84E-20	-5.31E-01	3.90E-06	-5.70E-01
11296	GSM452595	GSE18105	Colorectal Cancer	Colorectal	1.16E-17	-4.94E-01	2.79E-06	-5.78E-01
11297	GSM452596	GSE18105	Colorectal Cancer	Colorectal	8.84E-24	-5.76E-01	3.68E-10	-7.53E-01
11298	GSM452597	GSE18105	Colorectal Cancer	Colorectal	1.58E-17	-4.92E-01	5.49E-05	-5.06E-01
11299	GSM452598	GSE18105	Colorectal Cancer	Colorectal	7.27E-15	-4.51E-01	5.40E-04	-4.43E-01
11300	GSM452599	GSE18105	Colorectal Cancer	Colorectal	2.38E-18	-5.04E-01	5.74E-06	-5.61E-01
11301	GSM452600	GSE18105	Colorectal Cancer	Colorectal	1.84E-21	-5.47E-01	2.16E-05	-5.30E-01
11302	GSM452601	GSE18105	Colorectal Cancer	Colorectal	3.39E-06	-2.81E-01	7.36E-02	-2.61E-01
11303	GSM452602	GSE18105	Colorectal Cancer	Colorectal	6.16E-21	-5.40E-01	2.87E-06	-5.77E-01
11304	GSM452603	GSE18105	Colorectal Cancer	Colorectal	2.60E-14	-4.42E-01	4.16E-04	-4.51E-01
11305	GSM452604	GSE18105	Colorectal Cancer	Colorectal	2.20E-31	-6.65E-01	3.68E-10	-7.53E-01
11306	GSM452605	GSE18105	Colorectal Cancer	Colorectal	1.08E-19	-5.23E-01	5.74E-06	-5.61E-01
11307	GSM452606	GSE18105	Colorectal Cancer	Colorectal	1.38E-10	-3.77E-01	2.64E-03	-3.94E-01
11308	GSM452607	GSE18105	Colorectal Cancer	Colorectal	9.51E-16	-4.65E-01	3.90E-06	-5.70E-01
11309	GSM452608	GSE18105	Colorectal Cancer	Colorectal	1.78E-25	-5.97E-01	1.90E-08	-6.81E-01
11310	GSM452609	GSE18105	Colorectal Cancer	Colorectal	6.16E-21	-5.40E-01	1.40E-07	-6.42E-01
11311	GSM452610	GSE18105	Colorectal Cancer	Colorectal	4.52E-23	-5.67E-01	4.47E-07	-6.18E-01
11312	GSM452611	GSE18105	Colorectal Cancer	Colorectal	5.81E-29	-6.38E-01	3.48E-09	-7.13E-01
11313	GSM452612	GSE18105	Colorectal Cancer	Colorectal	3.32E-30	-6.52E-01	1.46E-10	-7.69E-01
11314	GSM452613	GSE18105	Colorectal Cancer	Colorectal	2.20E-23	-5.71E-01	9.57E-08	-6.50E-01
11315	GSM452614	GSE18105	Colorectal Cancer	Colorectal	3.27E-18	-5.02E-01	2.15E-07	-6.33E-01
11316	GSM452615	GSE18105	Colorectal Cancer	Colorectal	1.80E-13	-4.29E-01	7.74E-06	-5.54E-01
11317	GSM452616	GSE18105	Colorectal Cancer	Colorectal	2.60E-14	-4.42E-01	1.34E-04	-4.83E-01

11318	GSM452617	GSE18105	Colorectal Cancer	Colorectal	1.48E-14	-4.46E-01	2.44E-04	-4.66E-01
11319	GSM452618	GSE18105	Colorectal Cancer	Colorectal	9.65E-25	-5.88E-01	9.57E-08	-6.50E-01
11320	GSM452619	GSE18105	Colorectal Cancer	Colorectal	1.02E-12	-4.16E-01	1.50E-05	-5.38E-01
11321	GSM452620	GSE18105	Colorectal Cancer	Colorectal	1.79E-26	-6.09E-01	3.68E-10	-7.53E-01
11322	GSM452621	GSE18105	Colorectal Cancer	Colorectal	5.45E-17	-4.84E-01	3.98E-05	-5.14E-01
11323	GSM452622	GSE18105	Colorectal Cancer	Colorectal	9.88E-18	-4.95E-01	4.61E-07	-6.17E-01
11324	GSM452623	GSE18105	Colorectal Cancer	Colorectal	3.27E-18	-5.02E-01	1.10E-05	-5.46E-01
11325	GSM452624	GSE18105	Colorectal Cancer	Colorectal	3.53E-13	-4.24E-01	2.69E-03	-3.93E-01
11326	GSM452625	GSE18105	Colorectal Cancer	Colorectal	6.89E-14	-4.36E-01	3.12E-04	-4.59E-01
11327	GSM452626	GSE18105	Colorectal Cancer	Colorectal	6.16E-18	-4.98E-01	1.10E-05	-5.46E-01
11328	GSM452627	GSE18105	Colorectal Cancer	Colorectal	4.37E-21	-5.42E-01	6.59E-07	-6.10E-01
11329	GSM452628	GSE18105	Colorectal Cancer	Colorectal	1.29E-14	-4.47E-01	1.99E-06	-5.85E-01
11330	GSM523248	GSE20916	Colorectal Cancer	Colorectal	7.13E-12	-4.01E-01	1.98E-03	-4.03E-01
11331	GSM523249	GSE20916	Colorectal Cancer	Colorectal	3.06E-15	-4.57E-01	2.55E-04	-4.65E-01
11332	GSM523250	GSE20916	Colorectal Cancer	Colorectal	4.62E-13	-4.22E-01	9.56E-04	-4.26E-01
11333	GSM523253	GSE20916	Colorectal Cancer	Colorectal	5.23E-11	-3.85E-01	1.53E-03	-4.11E-01
11334	GSM523257	GSE20916	Colorectal Cancer	Colorectal	8.26E-27	-6.13E-01	3.11E-07	-6.25E-01
11335	GSM523258	GSE20916	Colorectal Cancer	Colorectal	6.18E-32	-6.71E-01	2.37E-10	-7.61E-01
11336	GSM523262	GSE20916	Colorectal Cancer	Colorectal	1.06E-28	-6.35E-01	6.54E-08	-6.57E-01
11337	GSM523263	GSE20916	Colorectal Cancer	Colorectal	1.45E-30	-6.56E-01	2.21E-09	-7.21E-01
11338	GSM523275	GSE20916	Colorectal Cancer	Colorectal	1.22E-10	-3.78E-01	1.20E-03	-4.19E-01
11339	GSM523278	GSE20916	Colorectal Cancer	Colorectal	1.11E-22	-5.63E-01	1.93E-06	-5.86E-01
11340	GSM523283	GSE20916	Colorectal Cancer	Colorectal	9.23E-09	-3.40E-01	7.28E-04	-4.34E-01
11341	GSM523284	GSE20916	Colorectal Cancer	Colorectal	6.43E-01	5.26E-02	5.89E-01	1.17E-01
11342	GSM523285	GSE20916	Colorectal Cancer	Colorectal	8.24E-02	-1.25E-01	4.61E-01	1.42E-01
11343	GSM523287	GSE20916	Colorectal Cancer	Colorectal	1.53E-23	-5.73E-01	3.02E-07	-6.26E-01
11344	GSM523288	GSE20916	Colorectal Cancer	Colorectal	6.43E-28	-6.26E-01	2.15E-07	-6.33E-01
11345	GSM523292	GSE20916	Colorectal Cancer	Colorectal	1.98E-15	-4.60E-01	1.37E-06	-5.94E-01
11346	GSM523293	GSE20916	Colorectal Cancer	Colorectal	1.44E-20	-5.35E-01	9.88E-08	-6.49E-01
11347	GSM523294	GSE20916	Colorectal Cancer	Colorectal	2.02E-20	-5.33E-01	3.11E-07	-6.25E-01
11348	GSM523295	GSE20916	Colorectal Cancer	Colorectal	7.65E-32	-6.70E-01	5.91E-10	-7.45E-01
11349	GSM523296	GSE20916	Colorectal Cancer	Colorectal	3.98E-10	-3.68E-01	4.08E-05	-5.14E-01
11350	GSM523298	GSE20916	Colorectal Cancer	Colorectal	9.08E-14	-4.34E-01	1.10E-05	-5.46E-01
11351	GSM523300	GSE20916	Colorectal Cancer	Colorectal	1.48E-26	-6.10E-01	5.27E-09	-7.05E-01
11352	GSM523301	GSE20916	Colorectal Cancer	Colorectal	2.61E-21	-5.45E-01	9.88E-08	-6.49E-01
11353	GSM523302	GSE20916	Colorectal Cancer	Colorectal	7.84E-28	-6.25E-01	8.49E-09	-6.97E-01
11354	GSM523303	GSE20916	Colorectal Cancer	Colorectal	1.18E-03	-2.05E-01	5.60E-02	-2.74E-01
11355	GSM523305	GSE20916	Colorectal Cancer	Colorectal	5.75E-06	-2.75E-01	5.64E-04	-4.42E-01
11356	GSM523306	GSE20916	Colorectal Cancer	Colorectal	1.03E-08	-3.39E-01	4.15E-03	-3.78E-01
11357	GSM523307	GSE20916	Colorectal Cancer	Colorectal	5.51E-25	-5.91E-01	1.23E-08	-6.90E-01
11358	GSM523308	GSE20916	Colorectal Cancer	Colorectal	5.08E-37	-7.23E-01	9.11E-10	-7.37E-01
11359	GSM523309	GSE20916	Colorectal Cancer	Colorectal	8.70E-29	-6.36E-01	1.27E-08	-6.89E-01
11360	GSM523312	GSE20916	Colorectal Cancer	Colorectal	1.97E-03	-1.97E-01	2.17E-02	-3.16E-01
11361	GSM523313	GSE20916	Colorectal Cancer	Colorectal	6.87E-26	-6.02E-01	6.33E-08	-6.58E-01
11362	GSM523315	GSE20916	Colorectal Cancer	Colorectal	3.10E-21	-5.44E-01	6.40E-07	-6.10E-01
11363	GSM523316	GSE20916	Colorectal Cancer	Colorectal	7.84E-07	-2.97E-01	1.77E-02	-3.24E-01
11364	GSM523317	GSE20916	Colorectal Cancer	Colorectal	2.41E-08	-3.31E-01	2.54E-03	-3.95E-01
11365	GSM523318	GSE20916	Colorectal Cancer	Colorectal	1.97E-12	-4.11E-01	5.40E-04	-4.43E-01
11366	GSM523319	GSE20916	Colorectal Cancer	Colorectal	2.15E-25	-5.96E-01	1.27E-08	-6.89E-01
11367	GSM523320	GSE20916	Colorectal Cancer	Colorectal	3.53E-24	-5.81E-01	6.40E-07	-6.10E-01
11368	GSM523321	GSE20916	Colorectal Cancer	Colorectal	6.80E-27	-6.14E-01	2.91E-08	-6.73E-01
11369	GSM523322	GSE20916	Colorectal Cancer	Colorectal	2.99E-35	-7.05E-01	2.21E-09	-7.21E-01
11370	GSM523323	GSE20916	Colorectal Cancer	Colorectal	3.08E-05	2.55E-01	6.17E-03	3.64E-01
11371	GSM523325	GSE20916	Colorectal Cancer	Colorectal	2.23E-05	-2.59E-01	7.88E-03	-3.56E-01
11372	GSM523326	GSE20916	Colorectal Cancer	Colorectal	1.58E-17	-4.92E-01	1.12E-05	-5.45E-01
11373	GSM523327	GSE20916	Colorectal Cancer	Colorectal	2.20E-23	-5.71E-01	3.11E-07	-6.25E-01
11374	GSM523328	GSE20916	Colorectal Cancer	Colorectal	1.83E-23	-5.72E-01	4.61E-07	-6.17E-01
11375	GSM523329	GSE20916	Colorectal Cancer	Colorectal	2.39E-35	-7.06E-01	8.95E-11	-7.77E-01
11376	GSM523331	GSE20916	Colorectal Cancer	Colorectal	4.09E-15	-4.55E-01	2.16E-05	-5.30E-01
11377	GSM523332	GSE20916	Colorectal Cancer	Colorectal	1.52E-11	-3.95E-01	2.87E-05	-5.23E-01
11378	GSM523333	GSE20916	Colorectal Cancer	Colorectal	3.54E-28	-6.29E-01	8.49E-09	-6.97E-01
11379	GSM523334	GSE20916	Colorectal Cancer	Colorectal	1.39E-32	-6.78E-01	8.21E-09	-6.97E-01
11380	GSM523335	GSE20916	Colorectal Cancer	Colorectal	5.45E-17	-4.84E-01	1.44E-07	-6.41E-01
11381	GSM523336	GSE20916	Colorectal Cancer	Colorectal	1.48E-26	-6.10E-01	4.44E-08	-6.65E-01
11382	GSM523337	GSE20916	Colorectal Cancer	Colorectal	2.21E-11	-3.92E-01	1.22E-03	-4.18E-01
11383	GSM523339	GSE20916	Colorectal Cancer	Colorectal	4.63E-01	6.94E-02	3.90E-01	1.57E-01
11384	GSM523340	GSE20916	Colorectal Cancer	Colorectal	4.08E-30	-6.51E-01	1.23E-08	-6.90E-01
11385	GSM523341	GSE20916	Colorectal Cancer	Colorectal	4.52E-23	-5.67E-01	6.54E-08	-6.57E-01
11386	GSM523342	GSE20916	Colorectal Cancer	Colorectal	4.04E-37	-7.24E-01	9.45E-10	-7.37E-01
11387	GSM523344	GSE20916	Colorectal Cancer	Colorectal	4.23E-01	7.34E-02	3.19E-01	1.73E-01
11388	GSM523345	GSE20916	Colorectal Cancer	Colorectal	1.19E-13	-4.32E-01	5.49E-05	-5.06E-01
11389	GSM523346	GSE20916	Colorectal Cancer	Colorectal	1.01E-25	-6.00E-01	2.15E-07	-6.33E-01
11390	GSM523347	GSE20916	Colorectal Cancer	Colorectal	2.10E-27	-6.20E-01	1.44E-07	-6.41E-01
11391	GSM523348	GSE20916	Colorectal Cancer	Colorectal	6.64E-25	-5.90E-01	1.27E-08	-6.89E-01

11392	GSM523350	GSE20916	Colorectal Cancer	Colorectal	6.08E-04	-2.15E-01	1.63E-01	-2.18E-01
11393	GSM523351	GSE20916	Colorectal Cancer	Colorectal	7.50E-08	-3.20E-01	5.52E-04	-4.43E-01
11394	GSM523352	GSE20916	Colorectal Cancer	Colorectal	4.67E-17	-4.85E-01	2.72E-06	-5.78E-01
11395	GSM523353	GSE20916	Colorectal Cancer	Colorectal	3.17E-38	-7.35E-01	9.11E-10	-7.37E-01
11396	GSM523354	GSE20916	Colorectal Cancer	Colorectal	6.43E-28	-6.26E-01	3.60E-09	-7.12E-01
11397	GSM523355	GSE20916	Colorectal Cancer	Colorectal	2.10E-27	-6.20E-01	3.21E-07	-6.25E-01
11398	GSM523356	GSE20916	Colorectal Cancer	Colorectal	3.21E-37	-7.25E-01	1.40E-09	-7.30E-01
11399	GSM523357	GSE20916	Colorectal Cancer	Colorectal	1.60E-05	-2.63E-01	1.04E-02	-3.45E-01
11400	GSM523359	GSE20916	Colorectal Cancer	Colorectal	7.46E-06	-2.72E-01	1.89E-02	-3.22E-01
11401	GSM523361	GSE20916	Colorectal Cancer	Colorectal	1.48E-26	-6.10E-01	2.03E-08	-6.80E-01
11402	GSM523362	GSE20916	Colorectal Cancer	Colorectal	4.95E-36	-7.13E-01	6.13E-10	-7.44E-01
11403	GSM523363	GSE20916	Colorectal Cancer	Colorectal	6.43E-28	-6.26E-01	9.57E-08	-6.50E-01
11404	GSM523366	GSE20916	Colorectal Cancer	Colorectal	6.11E-01	5.56E-02	2.00E-01	2.05E-01
11405	GSM523367	GSE20916	Colorectal Cancer	Colorectal	1.00E-26	-6.12E-01	6.54E-08	-6.57E-01
11406	GSM523368	GSE20916	Colorectal Cancer	Colorectal	1.77E-09	-3.55E-01	4.15E-03	-3.78E-01
11407	GSM523370	GSE20916	Colorectal Cancer	Colorectal	6.51E-04	-2.14E-01	1.75E-01	-2.13E-01
11408	GSM523371	GSE20916	Colorectal Cancer	Colorectal	1.16E-27	-6.23E-01	1.49E-07	-6.41E-01
11409	GSM523372	GSE20916	Colorectal Cancer	Colorectal	3.97E-20	-5.29E-01	6.59E-07	-6.10E-01
11410	GSM523374	GSE20916	Colorectal Cancer	Colorectal	2.19E-21	-5.46E-01	9.39E-07	-6.02E-01
11411	GSM523375	GSE20916	Colorectal Cancer	Colorectal	1.03E-33	-6.89E-01	1.50E-09	-7.28E-01
11412	GSM523376	GSE20916	Colorectal Cancer	Colorectal	3.87E-29	-6.40E-01	8.49E-09	-6.97E-01
11413	GSM523378	GSE20916	Colorectal Cancer	Colorectal	3.23E-03	-1.89E-01	2.02E-01	-2.04E-01
11414	GSM523379	GSE20916	Colorectal Cancer	Colorectal	7.30E-21	-5.39E-01	5.90E-06	-5.61E-01
11415	GSM523380	GSE20916	Colorectal Cancer	Colorectal	1.77E-19	-5.20E-01	6.54E-08	-6.57E-01
11416	GSM523381	GSE20916	Colorectal Cancer	Colorectal	9.06E-19	-5.10E-01	9.57E-08	-6.50E-01
11417	GSM523383	GSE20916	Colorectal Cancer	Colorectal	1.71E-15	-4.61E-01	2.38E-04	-4.67E-01
11418	GSM523384	GSE20916	Colorectal Cancer	Colorectal	1.58E-22	-5.61E-01	2.09E-07	-6.34E-01
11419	GSM523385	GSE20916	Colorectal Cancer	Colorectal	1.58E-22	-5.61E-01	3.11E-07	-6.25E-01
11420	GSM523386	GSE20916	Colorectal Cancer	Colorectal	1.59E-36	-7.18E-01	6.13E-10	-7.44E-01
11421	GSM588828	GSE23878	Colorectal Cancer	Colorectal	1.08E-40	-7.59E-01	1.82E-12	-8.40E-01
11422	GSM588829	GSE23878	Colorectal Cancer	Colorectal	9.12E-35	-7.00E-01	2.97E-12	-8.32E-01
11423	GSM588830	GSE23878	Colorectal Cancer	Colorectal	4.56E-25	-5.92E-01	2.21E-09	-7.21E-01
11424	GSM588831	GSE23878	Colorectal Cancer	Colorectal	8.81E-44	-7.88E-01	3.77E-13	-8.64E-01
11425	GSM588832	GSE23878	Colorectal Cancer	Colorectal	2.01E-46	-8.12E-01	8.28E-15	-9.20E-01
11426	GSM588833	GSE23878	Colorectal Cancer	Colorectal	5.10E-31	-6.61E-01	3.68E-10	-7.53E-01
11427	GSM588834	GSE23878	Colorectal Cancer	Colorectal	1.36E-16	-4.78E-01	3.90E-06	-5.70E-01
11428	GSM588835	GSE23878	Colorectal Cancer	Colorectal	6.43E-28	-6.26E-01	8.49E-09	-6.97E-01
11429	GSM588836	GSE23878	Colorectal Cancer	Colorectal	1.06E-23	-5.75E-01	9.57E-08	-6.50E-01
11430	GSM588837	GSE23878	Colorectal Cancer	Colorectal	1.14E-29	-6.46E-01	2.21E-09	-7.21E-01
11431	GSM588838	GSE23878	Colorectal Cancer	Colorectal	3.16E-29	-6.41E-01	3.48E-09	-7.13E-01
11432	GSM588839	GSE23878	Colorectal Cancer	Colorectal	4.56E-25	-5.92E-01	1.46E-10	-7.69E-01
11433	GSM588840	GSE23878	Colorectal Cancer	Colorectal	4.86E-39	-7.43E-01	1.35E-11	-8.08E-01
11434	GSM588841	GSE23878	Colorectal Cancer	Colorectal	9.65E-25	-5.88E-01	5.91E-10	-7.45E-01
11435	GSM588842	GSE23878	Colorectal Cancer	Colorectal	9.06E-19	-5.10E-01	1.99E-06	-5.85E-01
11436	GSM588843	GSE23878	Colorectal Cancer	Colorectal	1.53E-35	-7.08E-01	5.89E-11	-7.84E-01
11437	GSM588844	GSE23878	Colorectal Cancer	Colorectal	4.67E-17	-4.85E-01	1.99E-06	-5.85E-01
11438	GSM588845	GSE23878	Colorectal Cancer	Colorectal	1.01E-25	-6.00E-01	1.40E-07	-6.42E-01
11439	GSM588846	GSE23878	Colorectal Cancer	Colorectal	7.11E-10	-3.63E-01	1.08E-01	-2.41E-01
11440	GSM588847	GSE23878	Colorectal Cancer	Colorectal	6.13E-03	-1.79E-01	8.99E-02	-2.51E-01
11441	GSM588848	GSE23878	Colorectal Cancer	Colorectal	1.65E-06	-2.89E-01	7.46E-02	-2.60E-01
11442	GSM588849	GSE23878	Colorectal Cancer	Colorectal	2.69E-22	-5.58E-01	2.09E-07	-6.34E-01
11443	GSM588850	GSE23878	Colorectal Cancer	Colorectal	8.26E-27	-6.13E-01	4.44E-08	-6.65E-01
11444	GSM588851	GSE23878	Colorectal Cancer	Colorectal	1.37E-06	-2.91E-01	8.30E-03	-3.54E-01
11445	GSM588852	GSE23878	Colorectal Cancer	Colorectal	4.98E-08	-3.24E-01	1.51E-02	-3.31E-01
11446	GSM588853	GSE23878	Colorectal Cancer	Colorectal	1.22E-35	-7.09E-01	1.07E-12	-8.48E-01
11447	GSM588854	GSE23878	Colorectal Cancer	Colorectal	1.25E-18	-5.08E-01	4.01E-06	-5.70E-01
11448	GSM588855	GSE23878	Colorectal Cancer	Colorectal	1.98E-09	-3.54E-01	9.76E-04	-4.25E-01
11449	GSM588856	GSE23878	Colorectal Cancer	Colorectal	3.06E-15	-4.57E-01	1.99E-06	-5.85E-01
11450	GSM588857	GSE23878	Colorectal Cancer	Colorectal	2.60E-25	-5.95E-01	5.91E-10	-7.45E-01
11451	GSM588858	GSE23878	Colorectal Cancer	Colorectal	1.71E-15	-4.61E-01	2.79E-05	-5.23E-01
11452	GSM588859	GSE23878	Colorectal Cancer	Colorectal	4.49E-18	-5.00E-01	5.90E-06	-5.61E-01
11453	GSM588860	GSE23878	Colorectal Cancer	Colorectal	9.29E-30	-6.47E-01	5.45E-09	-7.05E-01
11454	GSM588861	GSE23878	Colorectal Cancer	Colorectal	6.29E-31	-6.60E-01	3.31E-11	-7.94E-01
11455	GSM588862	GSE23878	Colorectal Cancer	Colorectal	3.54E-15	-4.56E-01	5.74E-06	-5.61E-01
11456	GSM604484	GSE24514	Colorectal Cancer	Colorectal	1.14E-34	-6.99E-01	9.30E-11	-7.77E-01
11457	GSM604485	GSE24514	Colorectal Cancer	Colorectal	1.72E-32	-6.77E-01	1.96E-08	-6.81E-01
11458	GSM604486	GSE24514	Colorectal Cancer	Colorectal	2.99E-35	-7.05E-01	3.58E-11	-7.92E-01
11459	GSM604487	GSE24514	Colorectal Cancer	Colorectal	5.33E-34	-6.92E-01	2.28E-10	-7.61E-01
11460	GSM604488	GSE24514	Colorectal Cancer	Colorectal	3.74E-35	-7.04E-01	2.16E-11	-8.01E-01
11461	GSM604489	GSE24514	Colorectal Cancer	Colorectal	3.79E-27	-6.17E-01	5.27E-09	-7.05E-01
11462	GSM604490	GSE24514	Colorectal Cancer	Colorectal	2.03E-24	-5.84E-01	2.29E-09	-7.21E-01
11463	GSM604491	GSE24514	Colorectal Cancer	Colorectal	9.65E-25	-5.88E-01	2.82E-08	-6.74E-01
11464	GSM604492	GSE24514	Colorectal Cancer	Colorectal	7.75E-23	-5.64E-01	1.44E-07	-6.41E-01
11465	GSM604493	GSE24514	Colorectal Cancer	Colorectal	1.27E-23	-5.74E-01	4.30E-08	-6.65E-01

11466	GSM604494	GSE24514	Colorectal Cancer	Colorectal	3.84E-39	-7.44E-01	3.58E-11	-7.92E-01
11467	GSM604495	GSE24514	Colorectal Cancer	Colorectal	2.94E-17	-4.88E-01	5.49E-05	-5.06E-01
11468	GSM604496	GSE24514	Colorectal Cancer	Colorectal	1.71E-01	1.05E-01	3.61E-01	1.63E-01
11469	GSM604497	GSE24514	Colorectal Cancer	Colorectal	2.70E-30	-6.53E-01	9.11E-10	-7.37E-01
11470	GSM604498	GSE24514	Colorectal Cancer	Colorectal	1.10E-21	-5.50E-01	2.82E-08	-6.74E-01
11471	GSM604499	GSE24514	Colorectal Cancer	Colorectal	1.28E-15	-4.63E-01	7.95E-06	-5.54E-01
11472	GSM604500	GSE24514	Colorectal Cancer	Colorectal	1.00E-26	-6.12E-01	3.21E-07	-6.25E-01
11473	GSM604501	GSE24514	Colorectal Cancer	Colorectal	3.32E-30	-6.52E-01	3.36E-09	-7.14E-01
11474	GSM604502	GSE24514	Colorectal Cancer	Colorectal	1.05E-42	-7.78E-01	1.31E-13	-8.80E-01
11475	GSM604503	GSE24514	Colorectal Cancer	Colorectal	1.27E-19	-5.22E-01	1.63E-05	-5.37E-01
11476	GSM604504	GSE24514	Colorectal Cancer	Colorectal	3.87E-26	-6.05E-01	1.45E-09	-7.29E-01
11477	GSM604505	GSE24514	Colorectal Cancer	Colorectal	2.13E-32	-6.76E-01	5.64E-09	-7.04E-01
11478	GSM604506	GSE24514	Colorectal Cancer	Colorectal	6.48E-03	1.78E-01	1.89E-02	3.22E-01
11479	GSM604507	GSE24514	Colorectal Cancer	Colorectal	4.49E-18	-5.00E-01	2.79E-06	-5.78E-01
11480	GSM604508	GSE24514	Colorectal Cancer	Colorectal	1.85E-17	-4.91E-01	1.58E-05	-5.37E-01
11481	GSM604509	GSE24514	Colorectal Cancer	Colorectal	9.12E-35	-7.00E-01	8.09E-12	-8.17E-01
11482	GSM604510	GSE24514	Colorectal Cancer	Colorectal	1.89E-22	-5.60E-01	2.09E-07	-6.34E-01
11483	GSM604511	GSE24514	Colorectal Cancer	Colorectal	2.69E-22	-5.58E-01	9.57E-08	-6.50E-01
11484	GSM604512	GSE24514	Colorectal Cancer	Colorectal	1.05E-05	-2.68E-01	1.38E-01	-2.27E-01
11485	GSM604513	GSE24514	Colorectal Cancer	Colorectal	1.26E-09	-3.58E-01	6.62E-03	-3.62E-01
11486	GSM604514	GSE24514	Colorectal Cancer	Colorectal	1.06E-18	-5.09E-01	2.05E-05	-5.31E-01
11487	GSM604515	GSE24514	Colorectal Cancer	Colorectal	5.41E-23	-5.66E-01	5.45E-09	-7.05E-01
11488	GSM604516	GSE24514	Colorectal Cancer	Colorectal	4.95E-04	-2.18E-01	9.21E-02	-2.50E-01
11489	GSM604517	GSE24514	Colorectal Cancer	Colorectal	1.16E-24	-5.87E-01	2.15E-07	-6.33E-01
11490	GSM782671	GSE31595	Colorectal Cancer	Colorectal	5.98E-09	-3.44E-01	1.17E-03	-4.20E-01
11491	GSM782674	GSE31595	Colorectal Cancer	Colorectal	2.15E-25	-5.96E-01	2.21E-09	-7.21E-01
11492	GSM784850	GSE31595	Colorectal Cancer	Colorectal	8.20E-16	-4.66E-01	7.36E-05	-4.98E-01
11493	GSM784851	GSE31595	Colorectal Cancer	Colorectal	2.60E-14	-4.42E-01	5.76E-04	-4.41E-01
11494	GSM784852	GSE31595	Colorectal Cancer	Colorectal	7.37E-24	-5.77E-01	8.21E-09	-6.97E-01
11495	GSM784853	GSE31595	Colorectal Cancer	Colorectal	2.70E-13	-4.26E-01	5.36E-05	-5.07E-01
11496	GSM784854	GSE31595	Colorectal Cancer	Colorectal	1.84E-16	-4.76E-01	7.54E-05	-4.98E-01
11497	GSM784855	GSE31595	Colorectal Cancer	Colorectal	1.37E-07	-3.14E-01	1.49E-02	-3.31E-01
11498	GSM784856	GSE31595	Colorectal Cancer	Colorectal	4.49E-08	-3.25E-01	2.01E-03	-4.03E-01
11499	GSM784857	GSE31595	Colorectal Cancer	Colorectal	5.87E-42	-7.71E-01	4.63E-12	-8.25E-01
11500	GSM784858	GSE31595	Colorectal Cancer	Colorectal	2.94E-17	-4.88E-01	1.54E-05	-5.38E-01
11501	GSM784860	GSE31595	Colorectal Cancer	Colorectal	1.57E-04	-2.34E-01	3.76E-02	-2.93E-01
11502	GSM784862	GSE31595	Colorectal Cancer	Colorectal	4.60E-27	-6.16E-01	1.96E-08	-6.81E-01
11503	GSM784865	GSE31595	Colorectal Cancer	Colorectal	1.98E-09	-3.54E-01	3.13E-03	-3.88E-01
11504	GSM784867	GSE31595	Colorectal Cancer	Colorectal	2.49E-10	-3.72E-01	3.19E-04	-4.58E-01
11505	GSM784871	GSE31595	Colorectal Cancer	Colorectal	5.23E-41	-7.62E-01	7.47E-14	-8.88E-01
11506	GSM784873	GSE31595	Colorectal Cancer	Colorectal	1.44E-31	-6.67E-01	5.01E-12	-8.24E-01
11507	GSM784875	GSE31595	Colorectal Cancer	Colorectal	4.60E-05	-2.50E-01	8.99E-02	-2.51E-01
11508	GSM784878	GSE31595	Colorectal Cancer	Colorectal	1.40E-29	-6.45E-01	2.29E-09	-7.21E-01
11509	GSM784881	GSE31595	Colorectal Cancer	Colorectal	3.11E-27	-6.18E-01	5.91E-10	-7.45E-01
11510	GSM784884	GSE31595	Colorectal Cancer	Colorectal	3.32E-30	-6.52E-01	1.45E-09	-7.29E-01
11511	GSM784885	GSE31595	Colorectal Cancer	Colorectal	4.54E-40	-7.53E-01	1.35E-11	-8.08E-01
11512	GSM784886	GSE31595	Colorectal Cancer	Colorectal	3.54E-28	-6.29E-01	4.03E-08	-6.67E-01
11513	GSM784887	GSE31595	Colorectal Cancer	Colorectal	2.46E-19	-5.18E-01	1.93E-06	-5.86E-01
11514	GSM784888	GSE31595	Colorectal Cancer	Colorectal	1.98E-15	-4.60E-01	1.93E-06	-5.86E-01
11515	GSM784889	GSE31595	Colorectal Cancer	Colorectal	3.90E-16	-4.71E-01	2.16E-05	-5.30E-01
11516	GSM784890	GSE31595	Colorectal Cancer	Colorectal	7.40E-17	-4.82E-01	1.16E-05	-5.45E-01
11517	GSM784891	GSE31595	Colorectal Cancer	Colorectal	3.45E-09	-3.49E-01	5.25E-03	-3.70E-01
11518	GSM784892	GSE31595	Colorectal Cancer	Colorectal	2.19E-30	-6.54E-01	1.23E-08	-6.90E-01
11519	GSM784893	GSE31595	Colorectal Cancer	Colorectal	7.27E-15	-4.51E-01	7.74E-06	-5.54E-01
11520	GSM784894	GSE31595	Colorectal Cancer	Colorectal	8.97E-13	-4.17E-01	3.90E-06	-5.70E-01
11521	GSM784901	GSE31595	Colorectal Cancer	Colorectal	7.30E-21	-5.39E-01	1.40E-07	-6.42E-01
11522	GSM784902	GSE31595	Colorectal Cancer	Colorectal	5.87E-42	-7.71E-01	1.07E-12	-8.48E-01
11523	GSM784903	GSE31595	Colorectal Cancer	Colorectal	3.95E-36	-7.14E-01	3.45E-11	-7.93E-01
11524	GSM784904	GSE31595	Colorectal Cancer	Colorectal	4.37E-21	-5.42E-01	2.16E-05	-5.30E-01
11525	GSM784905	GSE31595	Colorectal Cancer	Colorectal	8.52E-04	-2.10E-01	5.49E-01	-1.25E-01
11526	GSM784906	GSE31595	Colorectal Cancer	Colorectal	2.16E-17	-4.90E-01	1.50E-05	-5.38E-01
11527	GSM92240	GSE4045	Colorectal Cancer	Colorectal	4.02E-19	-5.15E-01	5.58E-06	-5.62E-01
11528	GSM92241	GSE4045	Colorectal Cancer	Colorectal	2.52E-17	-4.89E-01	2.05E-05	-5.31E-01
11529	GSM92242	GSE4045	Colorectal Cancer	Colorectal	3.01E-07	-3.07E-01	2.72E-02	-3.07E-01
11530	GSM92243	GSE4045	Colorectal Cancer	Colorectal	1.72E-32	-6.77E-01	1.45E-09	-7.29E-01
11531	GSM92244	GSE4045	Colorectal Cancer	Colorectal	1.97E-12	-4.11E-01	7.54E-05	-4.98E-01
11532	GSM92245	GSE4045	Colorectal Cancer	Colorectal	7.46E-06	-2.72E-01	1.34E-01	-2.29E-01
11533	GSM92246	GSE4045	Colorectal Cancer	Colorectal	2.40E-02	1.53E-01	8.66E-02	2.53E-01
11534	GSM92247	GSE4045	Colorectal Cancer	Colorectal	1.35E-17	-4.93E-01	2.72E-06	-5.78E-01
11535	GSM92248	GSE4045	Colorectal Cancer	Colorectal	1.98E-33	-6.87E-01	8.09E-12	-8.17E-01
11536	GSM92249	GSE4045	Colorectal Cancer	Colorectal	3.06E-33	-6.85E-01	3.58E-11	-7.92E-01
11537	GSM92250	GSE4045	Colorectal Cancer	Colorectal	1.28E-15	-4.63E-01	1.34E-04	-4.83E-01
11538	GSM92251	GSE4045	Colorectal Cancer	Colorectal	2.10E-29	-6.43E-01	1.40E-09	-7.30E-01
11539	GSM92252	GSE4045	Colorectal Cancer	Colorectal	1.32E-22	-5.62E-01	3.11E-07	-6.25E-01

11540	GSM92253	GSE4045	Colorectal Cancer	Colorectal	5.05E-38	-7.33E-01	2.18E-13	-8.72E-01
11541	GSM92254	GSE4045	Colorectal Cancer	Colorectal	4.62E-13	-4.22E-01	9.56E-04	-4.26E-01
11542	GSM92255	GSE4045	Colorectal Cancer	Colorectal	1.59E-36	-7.18E-01	2.16E-11	-8.01E-01
11543	GSM92256	GSE4045	Colorectal Cancer	Colorectal	1.18E-44	-7.96E-01	1.68E-12	-8.41E-01
11544	GSM92257	GSE4045	Colorectal Cancer	Colorectal	1.30E-21	-5.49E-01	2.91E-08	-6.73E-01
11545	GSM92258	GSE4045	Colorectal Cancer	Colorectal	4.87E-12	-4.04E-01	3.25E-03	-3.87E-01
11546	GSM92259	GSE4045	Colorectal Cancer	Colorectal	3.22E-41	-7.64E-01	1.82E-12	-8.40E-01
11547	GSM92260	GSE4045	Colorectal Cancer	Colorectal	3.96E-14	-4.39E-01	7.28E-04	-4.34E-01
11548	GSM92261	GSE4045	Colorectal Cancer	Colorectal	1.72E-42	-7.76E-01	2.97E-12	-8.32E-01
11549	GSM92262	GSE4045	Colorectal Cancer	Colorectal	2.63E-23	-5.70E-01	1.23E-08	-6.90E-01
11550	GSM92263	GSE4045	Colorectal Cancer	Colorectal	1.08E-19	-5.23E-01	4.61E-07	-6.17E-01
11551	GSM92264	GSE4045	Colorectal Cancer	Colorectal	3.06E-15	-4.57E-01	1.34E-04	-4.83E-01
11552	GSM92265	GSE4045	Colorectal Cancer	Colorectal	4.62E-13	-4.22E-01	5.52E-04	-4.43E-01
11553	GSM92266	GSE4045	Colorectal Cancer	Colorectal	3.36E-16	-4.72E-01	7.54E-05	-4.98E-01
11554	GSM92267	GSE4045	Colorectal Cancer	Colorectal	1.73E-27	-6.21E-01	3.21E-07	-6.25E-01
11555	GSM92268	GSE4045	Colorectal Cancer	Colorectal	2.25E-22	-5.59E-01	9.11E-10	-7.37E-01
11556	GSM92269	GSE4045	Colorectal Cancer	Colorectal	6.55E-20	-5.26E-01	6.33E-08	-6.58E-01
11557	GSM92270	GSE4045	Colorectal Cancer	Colorectal	1.11E-22	-5.63E-01	1.99E-06	-5.85E-01
11558	GSM92271	GSE4045	Colorectal Cancer	Colorectal	4.71E-33	-6.83E-01	1.35E-11	-8.08E-01
11559	GSM92272	GSE4045	Colorectal Cancer	Colorectal	4.95E-04	-2.18E-01	5.53E-02	-2.75E-01
11560	GSM92273	GSE4045	Colorectal Cancer	Colorectal	4.69E-20	-5.28E-01	4.47E-07	-6.18E-01
11561	GSM92274	GSE4045	Colorectal Cancer	Colorectal	2.80E-10	-3.71E-01	6.62E-03	-3.62E-01
11562	GSM92275	GSE4045	Colorectal Cancer	Colorectal	1.52E-07	-3.13E-01	2.77E-02	-3.06E-01
11563	GSM92276	GSE4045	Colorectal Cancer	Colorectal	1.16E-17	-4.94E-01	1.33E-06	-5.94E-01
11564	GSM136578	GSE5851	Colorectal Cancer	Colorectal	1.00E-09	-3.60E-01	1.20E-03	-4.19E-01
11565	GSM136579	GSE5851	Colorectal Cancer	Colorectal	2.44E-24	-5.83E-01	3.48E-09	-7.13E-01
11566	GSM136580	GSE5851	Colorectal Cancer	Colorectal	4.71E-33	-6.83E-01	2.37E-09	-7.20E-01
11567	GSM136581	GSE5851	Colorectal Cancer	Colorectal	1.01E-36	-7.20E-01	4.82E-12	-8.25E-01
11568	GSM136582	GSE5851	Colorectal Cancer	Colorectal	1.34E-11	-3.96E-01	1.01E-04	-4.90E-01
11569	GSM136583	GSE5851	Colorectal Cancer	Colorectal	3.80E-33	-6.84E-01	1.82E-12	-8.40E-01
11570	GSM136584	GSE5851	Colorectal Cancer	Colorectal	3.98E-10	-3.68E-01	4.22E-03	-3.78E-01
11571	GSM136585	GSE5851	Colorectal Cancer	Colorectal	3.11E-27	-6.18E-01	3.36E-09	-7.14E-01
11572	GSM136586	GSE5851	Colorectal Cancer	Colorectal	2.76E-09	-3.51E-01	7.36E-05	-4.98E-01
11573	GSM136587	GSE5851	Colorectal Cancer	Colorectal	2.37E-06	-2.85E-01	5.36E-05	-5.07E-01
11574	GSM136588	GSE5851	Colorectal Cancer	Colorectal	8.20E-16	-4.66E-01	4.30E-08	-6.65E-01
11575	GSM136589	GSE5851	Colorectal Cancer	Colorectal	1.28E-33	-6.88E-01	3.58E-11	-7.92E-01
11576	GSM136590	GSE5851	Colorectal Cancer	Colorectal	4.52E-23	-5.67E-01	4.08E-05	-5.14E-01
11577	GSM136591	GSE5851	Colorectal Cancer	Colorectal	2.64E-26	-6.07E-01	9.11E-10	-7.37E-01
11578	GSM136592	GSE5851	Colorectal Cancer	Colorectal	4.02E-04	-2.21E-01	4.07E-03	-3.79E-01
11579	GSM136593	GSE5851	Colorectal Cancer	Colorectal	2.29E-15	-4.59E-01	4.47E-07	-6.18E-01
11580	GSM136594	GSE5851	Colorectal Cancer	Colorectal	9.06E-19	-5.10E-01	4.18E-05	-5.13E-01
11581	GSM136595	GSE5851	Colorectal Cancer	Colorectal	1.47E-25	-5.98E-01	5.91E-10	-7.45E-01
11582	GSM136596	GSE5851	Colorectal Cancer	Colorectal	5.54E-45	-7.99E-01	1.31E-13	-8.80E-01
11583	GSM136597	GSE5851	Colorectal Cancer	Colorectal	4.83E-06	-2.77E-01	1.56E-03	-4.11E-01
11584	GSM136598	GSE5851	Colorectal Cancer	Colorectal	2.20E-31	-6.65E-01	8.09E-12	-8.17E-01
11585	GSM136599	GSE5851	Colorectal Cancer	Colorectal	8.32E-26	-6.01E-01	5.45E-09	-7.05E-01
11586	GSM136600	GSE5851	Colorectal Cancer	Colorectal	3.35E-20	-5.30E-01	8.21E-09	-6.97E-01
11587	GSM136601	GSE5851	Colorectal Cancer	Colorectal	1.19E-13	-4.32E-01	1.99E-06	-5.85E-01
11588	GSM136602	GSE5851	Colorectal Cancer	Colorectal	5.84E-35	-7.02E-01	1.96E-08	-6.81E-01
11589	GSM136603	GSE5851	Colorectal Cancer	Colorectal	2.67E-08	-3.30E-01	1.06E-01	-2.42E-01
11590	GSM136604	GSE5851	Colorectal Cancer	Colorectal	1.12E-09	-3.59E-01	1.50E-05	-5.38E-01
11591	GSM136605	GSE5851	Colorectal Cancer	Colorectal	1.08E-19	-5.23E-01	3.09E-05	-5.21E-01
11592	GSM136606	GSE5851	Colorectal Cancer	Colorectal	8.03E-38	-7.31E-01	5.89E-11	-7.84E-01
11593	GSM136607	GSE5851	Colorectal Cancer	Colorectal	2.60E-14	-4.42E-01	7.95E-06	-5.54E-01
11594	GSM136608	GSE5851	Colorectal Cancer	Colorectal	2.37E-06	-2.85E-01	5.52E-04	-4.43E-01
11595	GSM136609	GSE5851	Colorectal Cancer	Colorectal	7.57E-30	-6.48E-01	1.52E-10	-7.68E-01
11596	GSM136610	GSE5851	Colorectal Cancer	Colorectal	1.16E-27	-6.23E-01	2.21E-09	-7.21E-01
11597	GSM136611	GSE5851	Colorectal Cancer	Colorectal	4.32E-28	-6.28E-01	2.37E-10	-7.61E-01
11598	GSM136612	GSE5851	Colorectal Cancer	Colorectal	3.27E-18	-5.02E-01	2.72E-06	-5.78E-01
11599	GSM136613	GSE5851	Colorectal Cancer	Colorectal	1.71E-20	-5.34E-01	1.99E-06	-5.85E-01
11600	GSM136614	GSE5851	Colorectal Cancer	Colorectal	1.97E-12	-4.11E-01	7.28E-04	-4.34E-01
11601	GSM136615	GSE5851	Colorectal Cancer	Colorectal	1.97E-12	-4.11E-01	5.68E-02	-2.74E-01
11602	GSM136616	GSE5851	Colorectal Cancer	Colorectal	1.17E-12	-4.15E-01	1.37E-06	-5.94E-01
11603	GSM136617	GSE5851	Colorectal Cancer	Colorectal	9.65E-25	-5.88E-01	2.15E-07	-6.33E-01
11604	GSM136618	GSE5851	Colorectal Cancer	Colorectal	6.49E-07	-2.99E-01	1.51E-02	-3.31E-01
11605	GSM136619	GSE5851	Colorectal Cancer	Colorectal	9.28E-40	-7.50E-01	9.30E-11	-7.77E-01
11606	GSM136620	GSE5851	Colorectal Cancer	Colorectal	4.00E-38	-7.34E-01	3.77E-13	-8.64E-01
11607	GSM136621	GSE5851	Colorectal Cancer	Colorectal	1.18E-11	-3.97E-01	4.08E-05	-5.14E-01
11608	GSM136622	GSE5851	Colorectal Cancer	Colorectal	3.13E-25	-5.94E-01	9.88E-08	-6.49E-01
11609	GSM136623	GSE5851	Colorectal Cancer	Colorectal	8.47E-41	-7.60E-01	2.56E-15	-9.37E-01
11610	GSM136624	GSE5851	Colorectal Cancer	Colorectal	1.78E-31	-6.66E-01	3.82E-10	-7.52E-01
11611	GSM136625	GSE5851	Colorectal Cancer	Colorectal	4.44E-07	-3.03E-01	3.33E-01	-1.70E-01
11612	GSM136626	GSE5851	Colorectal Cancer	Colorectal	6.16E-39	-7.42E-01	3.58E-11	-7.92E-01
11613	GSM136627	GSE5851	Colorectal Cancer	Colorectal	3.62E-05	-2.53E-01	3.30E-01	1.70E-01

11614	GSM136628	GSE5851	Colorectal Cancer	Colorectal	4.98E-49	-8.34E-01	7.15E-14	-8.89E-01
11615	GSM136629	GSE5851	Colorectal Cancer	Colorectal	4.71E-33	-6.83E-01	2.46E-10	-7.60E-01
11616	GSM136630	GSE5851	Colorectal Cancer	Colorectal	1.00E-16	4.80E-01	1.46E-05	5.39E-01
11617	GSM136631	GSE5851	Colorectal Cancer	Colorectal	1.51E-47	-8.21E-01	2.51E-14	-9.04E-01
11618	GSM136632	GSE5851	Colorectal Cancer	Colorectal	1.00E-26	-6.12E-01	4.44E-08	-6.65E-01
11619	GSM136633	GSE5851	Colorectal Cancer	Colorectal	1.58E-22	-5.61E-01	9.11E-10	-7.37E-01
11620	GSM136634	GSE5851	Colorectal Cancer	Colorectal	6.37E-38	-7.32E-01	2.37E-10	-7.61E-01
11621	GSM136635	GSE5851	Colorectal Cancer	Colorectal	4.98E-08	-3.24E-01	8.88E-02	-2.51E-01
11622	GSM136636	GSE5851	Colorectal Cancer	Colorectal	2.94E-17	-4.88E-01	7.44E-04	-4.34E-01
11623	GSM136637	GSE5851	Colorectal Cancer	Colorectal	6.86E-44	-7.89E-01	7.47E-14	-8.88E-01
11624	GSM136638	GSE5851	Colorectal Cancer	Colorectal	3.23E-03	-1.89E-01	4.67E-01	1.41E-01
11625	GSM136639	GSE5851	Colorectal Cancer	Colorectal	3.15E-23	-5.69E-01	2.21E-09	-7.21E-01
11626	GSM136640	GSE5851	Colorectal Cancer	Colorectal	5.57E-19	-5.13E-01	5.74E-06	-5.61E-01
11627	GSM136641	GSE5851	Colorectal Cancer	Colorectal	7.75E-31	-6.59E-01	2.37E-10	-7.61E-01
11628	GSM136642	GSE5851	Colorectal Cancer	Colorectal	7.25E-03	-1.76E-01	1.19E-01	2.36E-01
11629	GSM136643	GSE5851	Colorectal Cancer	Colorectal	7.07E-16	-4.67E-01	5.76E-04	-4.41E-01
11630	GSM136644	GSE5851	Colorectal Cancer	Colorectal	1.72E-18	-5.06E-01	1.01E-04	-4.90E-01
11631	GSM136645	GSE5851	Colorectal Cancer	Colorectal	4.04E-13	-4.23E-01	2.79E-06	-5.78E-01
11632	GSM136646	GSE5851	Colorectal Cancer	Colorectal	1.98E-06	-2.87E-01	4.80E-01	-1.38E-01
11633	GSM136647	GSE5851	Colorectal Cancer	Colorectal	1.53E-23	-5.73E-01	2.87E-06	-5.77E-01
11634	GSM136648	GSE5851	Colorectal Cancer	Colorectal	2.15E-25	-5.96E-01	3.48E-09	-7.13E-01
11635	GSM136649	GSE5851	Colorectal Cancer	Colorectal	1.11E-14	-4.48E-01	1.92E-02	-3.21E-01
11636	GSM136650	GSE5851	Colorectal Cancer	Colorectal	7.75E-31	-6.59E-01	2.97E-12	-8.32E-01
11637	GSM136651	GSE5851	Colorectal Cancer	Colorectal	1.91E-35	-7.07E-01	1.45E-09	-7.29E-01
11638	GSM136652	GSE5851	Colorectal Cancer	Colorectal	1.74E-03	1.99E-01	2.87E-05	5.23E-01
11639	GSM136653	GSE5851	Colorectal Cancer	Colorectal	4.02E-19	-5.15E-01	1.33E-06	-5.94E-01
11640	GSM136654	GSE5851	Colorectal Cancer	Colorectal	1.53E-35	-7.08E-01	1.07E-12	-8.48E-01
11641	GSM136655	GSE5851	Colorectal Cancer	Colorectal	1.40E-24	-5.86E-01	8.21E-09	-6.97E-01
11642	GSM136656	GSE5851	Colorectal Cancer	Colorectal	4.55E-14	-4.38E-01	1.54E-02	-3.30E-01
11643	GSM136657	GSE5851	Colorectal Cancer	Colorectal	4.44E-07	-3.03E-01	1.98E-03	-4.03E-01
11644	GSM215083	GSE8671	Colorectal Cancer	Colorectal	1.22E-26	-6.11E-01	1.45E-09	-7.29E-01
11645	GSM215084	GSE8671	Colorectal Cancer	Colorectal	1.22E-25	-5.99E-01	1.23E-08	-6.90E-01
11646	GSM215085	GSE8671	Colorectal Cancer	Colorectal	6.87E-26	-6.02E-01	3.48E-09	-7.13E-01
11647	GSM215086	GSE8671	Colorectal Cancer	Colorectal	1.27E-36	-7.19E-01	1.07E-12	-8.48E-01
11648	GSM215087	GSE8671	Colorectal Cancer	Colorectal	6.64E-25	-5.90E-01	6.33E-08	-6.58E-01
11649	GSM215088	GSE8671	Colorectal Cancer	Colorectal	5.68E-26	-6.03E-01	1.27E-08	-6.89E-01
11650	GSM215089	GSE8671	Colorectal Cancer	Colorectal	7.24E-46	-8.07E-01	1.75E-12	-8.41E-01
11651	GSM215090	GSE8671	Colorectal Cancer	Colorectal	2.82E-40	-7.55E-01	1.30E-11	-8.09E-01
11652	GSM215091	GSE8671	Colorectal Cancer	Colorectal	1.22E-26	-6.11E-01	1.90E-08	-6.81E-01
11653	GSM215092	GSE8671	Colorectal Cancer	Colorectal	7.37E-24	-5.77E-01	9.57E-08	-6.50E-01
11654	GSM215093	GSE8671	Colorectal Cancer	Colorectal	9.55E-31	-6.58E-01	1.50E-09	-7.28E-01
11655	GSM215094	GSE8671	Colorectal Cancer	Colorectal	2.49E-16	-4.74E-01	3.98E-05	-5.14E-01
11656	GSM215095	GSE8671	Colorectal Cancer	Colorectal	3.79E-27	-6.17E-01	6.33E-08	-6.58E-01
11657	GSM215096	GSE8671	Colorectal Cancer	Colorectal	3.97E-20	-5.29E-01	1.10E-05	-5.46E-01
11658	GSM215097	GSE8671	Colorectal Cancer	Colorectal	7.30E-35	-7.01E-01	2.16E-11	-8.01E-01
11659	GSM215098	GSE8671	Colorectal Cancer	Colorectal	5.44E-22	-5.54E-01	5.58E-06	-5.62E-01
11660	GSM215099	GSE8671	Colorectal Cancer	Colorectal	5.01E-30	-6.50E-01	2.21E-09	-7.21E-01
11661	GSM215100	GSE8671	Colorectal Cancer	Colorectal	3.74E-35	-7.04E-01	2.97E-12	-8.32E-01
11662	GSM215101	GSE8671	Colorectal Cancer	Colorectal	6.80E-27	-6.14E-01	3.60E-09	-7.12E-01
11663	GSM215102	GSE8671	Colorectal Cancer	Colorectal	6.86E-44	-7.89E-01	2.51E-14	-9.04E-01
11664	GSM215103	GSE8671	Colorectal Cancer	Colorectal	2.63E-23	-5.70E-01	6.79E-07	-6.09E-01
11665	GSM215104	GSE8671	Colorectal Cancer	Colorectal	6.18E-32	-6.71E-01	1.46E-10	-7.69E-01
11666	GSM215105	GSE8671	Colorectal Cancer	Colorectal	6.29E-31	-6.60E-01	4.44E-08	-6.65E-01
11667	GSM215106	GSE8671	Colorectal Cancer	Colorectal	4.08E-30	-6.51E-01	1.27E-08	-6.89E-01
11668	GSM215107	GSE8671	Colorectal Cancer	Colorectal	2.25E-22	-5.59E-01	2.15E-07	-6.33E-01
11669	GSM215108	GSE8671	Colorectal Cancer	Colorectal	4.11E-41	-7.63E-01	1.07E-12	-8.48E-01
11670	GSM215109	GSE8671	Colorectal Cancer	Colorectal	4.60E-27	-6.16E-01	5.27E-09	-7.05E-01
11671	GSM215110	GSE8671	Colorectal Cancer	Colorectal	3.15E-36	-7.15E-01	1.07E-12	-8.48E-01
11672	GSM215111	GSE8671	Colorectal Cancer	Colorectal	1.22E-35	-7.09E-01	2.37E-10	-7.61E-01
11673	GSM215112	GSE8671	Colorectal Cancer	Colorectal	7.74E-20	-5.25E-01	2.05E-06	-5.85E-01
11674	GSM215113	GSE8671	Colorectal Cancer	Colorectal	1.42E-34	-6.98E-01	2.16E-11	-8.01E-01
11675	GSM215114	GSE8671	Colorectal Cancer	Colorectal	5.68E-26	-6.03E-01	3.48E-09	-7.13E-01
11676	GSM237914	GSE9348	Colorectal Cancer	Colorectal	5.01E-30	-6.50E-01	3.68E-10	-7.53E-01
11677	GSM237915	GSE9348	Colorectal Cancer	Colorectal	1.16E-27	-6.23E-01	3.48E-09	-7.13E-01
11678	GSM237916	GSE9348	Colorectal Cancer	Colorectal	4.55E-14	-4.38E-01	4.16E-04	-4.51E-01
11679	GSM237917	GSE9348	Colorectal Cancer	Colorectal	3.80E-33	-6.84E-01	3.58E-11	-7.92E-01
11680	GSM237918	GSE9348	Colorectal Cancer	Colorectal	2.16E-06	-2.86E-01	2.64E-02	-3.08E-01
11681	GSM237919	GSE9348	Colorectal Cancer	Colorectal	5.26E-18	-4.99E-01	5.74E-06	-5.61E-01
11682	GSM237920	GSE9348	Colorectal Cancer	Colorectal	1.11E-14	-4.48E-01	1.37E-04	-4.82E-01
11683	GSM237921	GSE9348	Colorectal Cancer	Colorectal	3.80E-33	-6.84E-01	9.30E-11	-7.77E-01
11684	GSM237922	GSE9348	Colorectal Cancer	Colorectal	3.27E-18	-5.02E-01	7.95E-06	-5.54E-01
11685	GSM237923	GSE9348	Colorectal Cancer	Colorectal	2.02E-37	-7.27E-01	3.45E-11	-7.93E-01
11686	GSM237924	GSE9348	Colorectal Cancer	Colorectal	4.25E-24	-5.80E-01	5.70E-10	-7.45E-01
11687	GSM237925	GSE9348	Colorectal Cancer	Colorectal	2.21E-34	-6.96E-01	3.82E-10	-7.52E-01

11688	GSM237926	GSE9348	Colorectal Cancer	Colorectal	2.99E-14	-4.41E-01	8.39E-06	-5.52E-01
11689	GSM237927	GSE9348	Colorectal Cancer	Colorectal	1.81E-06	-2.88E-01	6.28E-03	-3.64E-01
11690	GSM237928	GSE9348	Colorectal Cancer	Colorectal	9.26E-02	-1.22E-01	3.90E-01	1.57E-01
11691	GSM237929	GSE9348	Colorectal Cancer	Colorectal	3.50E-02	-1.45E-01	5.03E-01	-1.34E-01
11692	GSM237930	GSE9348	Colorectal Cancer	Colorectal	6.48E-22	-5.53E-01	6.54E-08	-6.57E-01
11693	GSM237931	GSE9348	Colorectal Cancer	Colorectal	1.97E-12	-4.11E-01	2.54E-03	-3.95E-01
11694	GSM237932	GSE9348	Colorectal Cancer	Colorectal	2.60E-25	-5.95E-01	6.33E-08	-6.58E-01
11695	GSM237933	GSE9348	Colorectal Cancer	Colorectal	3.25E-04	-2.24E-01	6.39E-02	-2.68E-01
11696	GSM237934	GSE9348	Colorectal Cancer	Colorectal	1.14E-29	-6.46E-01	3.48E-09	-7.13E-01
11697	GSM237935	GSE9348	Colorectal Cancer	Colorectal	8.94E-10	-3.61E-01	4.07E-04	-4.51E-01
11698	GSM237936	GSE9348	Colorectal Cancer	Colorectal	1.03E-08	-3.39E-01	1.56E-03	-4.11E-01
11699	GSM237937	GSE9348	Colorectal Cancer	Colorectal	1.25E-18	-5.08E-01	8.16E-06	-5.53E-01
11700	GSM237938	GSE9348	Colorectal Cancer	Colorectal	8.61E-07	-2.96E-01	9.85E-03	-3.47E-01
11701	GSM237939	GSE9348	Colorectal Cancer	Colorectal	4.25E-24	-5.80E-01	4.47E-07	-6.18E-01
11702	GSM237940	GSE9348	Colorectal Cancer	Colorectal	2.19E-30	-6.54E-01	2.46E-10	-7.60E-01
11703	GSM237941	GSE9348	Colorectal Cancer	Colorectal	8.10E-12	-4.00E-01	9.17E-04	-4.27E-01
11704	GSM237942	GSE9348	Colorectal Cancer	Colorectal	2.49E-10	-3.72E-01	4.35E-04	-4.50E-01
11705	GSM237943	GSE9348	Colorectal Cancer	Colorectal	5.76E-40	-7.52E-01	2.18E-13	-8.72E-01
11706	GSM237944	GSE9348	Colorectal Cancer	Colorectal	7.30E-35	-7.01E-01	1.35E-11	-8.08E-01
11707	GSM237945	GSE9348	Colorectal Cancer	Colorectal	4.55E-14	-4.38E-01	5.25E-03	-3.70E-01
11708	GSM237946	GSE9348	Colorectal Cancer	Colorectal	1.14E-34	-6.99E-01	1.52E-10	-7.68E-01
11709	GSM237947	GSE9348	Colorectal Cancer	Colorectal	8.32E-26	-6.01E-01	2.82E-08	-6.74E-01
11710	GSM237948	GSE9348	Colorectal Cancer	Colorectal	1.99E-41	-7.66E-01	6.23E-13	-8.57E-01
11711	GSM237949	GSE9348	Colorectal Cancer	Colorectal	7.97E-10	-3.62E-01	2.38E-04	-4.67E-01
11712	GSM237950	GSE9348	Colorectal Cancer	Colorectal	1.58E-22	-5.61E-01	1.49E-07	-6.41E-01
11713	GSM237951	GSE9348	Colorectal Cancer	Colorectal	8.47E-41	-7.60E-01	5.89E-11	-7.84E-01
11714	GSM237952	GSE9348	Colorectal Cancer	Colorectal	5.02E-10	-3.66E-01	1.81E-04	-4.74E-01
11715	GSM237953	GSE9348	Colorectal Cancer	Colorectal	4.32E-28	-6.28E-01	3.48E-09	-7.13E-01
11716	GSM237954	GSE9348	Colorectal Cancer	Colorectal	1.02E-12	-4.16E-01	4.35E-04	-4.50E-01
11717	GSM237955	GSE9348	Colorectal Cancer	Colorectal	1.16E-24	-5.87E-01	9.57E-08	-6.50E-01
11718	GSM237956	GSE9348	Colorectal Cancer	Colorectal	1.37E-07	-3.14E-01	1.01E-01	-2.44E-01
11719	GSM237957	GSE9348	Colorectal Cancer	Colorectal	9.46E-32	-6.69E-01	2.16E-11	-8.01E-01
11720	GSM237958	GSE9348	Colorectal Cancer	Colorectal	4.63E-11	-3.86E-01	1.81E-04	-4.74E-01
11721	GSM237959	GSE9348	Colorectal Cancer	Colorectal	1.89E-05	-2.61E-01	1.40E-01	-2.27E-01
11722	GSM237960	GSE9348	Colorectal Cancer	Colorectal	7.84E-07	-2.97E-01	1.56E-03	-4.11E-01
11723	GSM237961	GSE9348	Colorectal Cancer	Colorectal	9.29E-30	-6.47E-01	3.68E-10	-7.53E-01
11724	GSM237962	GSE9348	Colorectal Cancer	Colorectal	6.16E-21	-5.40E-01	4.47E-07	-6.18E-01
11725	GSM237963	GSE9348	Colorectal Cancer	Colorectal	4.60E-03	-1.84E-01	2.40E-01	-1.93E-01
11726	GSM237964	GSE9348	Colorectal Cancer	Colorectal	1.85E-17	-4.91E-01	1.10E-05	-5.46E-01
11727	GSM237965	GSE9348	Colorectal Cancer	Colorectal	3.63E-03	-1.88E-01	1.40E-01	-2.27E-01
11728	GSM237966	GSE9348	Colorectal Cancer	Colorectal	6.67E-09	-3.43E-01	2.59E-03	-3.94E-01
11729	GSM237967	GSE9348	Colorectal Cancer	Colorectal	6.64E-25	-5.90E-01	4.30E-08	-6.65E-01
11730	GSM237968	GSE9348	Colorectal Cancer	Colorectal	1.01E-02	-1.70E-01	1.18E-01	-2.36E-01
11731	GSM237969	GSE9348	Colorectal Cancer	Colorectal	2.59E-06	-2.84E-01	9.21E-02	-2.50E-01
11732	GSM237970	GSE9348	Colorectal Cancer	Colorectal	1.00E-16	-4.80E-01	1.99E-06	-5.85E-01
11733	GSM237971	GSE9348	Colorectal Cancer	Colorectal	2.17E-26	-6.08E-01	2.09E-07	-6.34E-01
11734	GSM237972	GSE9348	Colorectal Cancer	Colorectal	2.91E-12	-4.08E-01	3.12E-04	-4.59E-01
11735	GSM237973	GSE9348	Colorectal Cancer	Colorectal	1.06E-23	-5.75E-01	1.90E-08	-6.81E-01
11736	GSM237974	GSE9348	Colorectal Cancer	Colorectal	9.12E-35	-7.00E-01	2.37E-10	-7.61E-01
11737	GSM237975	GSE9348	Colorectal Cancer	Colorectal	1.08E-19	-5.23E-01	1.37E-06	-5.94E-01
11738	GSM237976	GSE9348	Colorectal Cancer	Colorectal	2.83E-11	-3.90E-01	1.94E-03	-4.04E-01
11739	GSM237977	GSE9348	Colorectal Cancer	Colorectal	8.47E-41	-7.60E-01	7.15E-14	-8.89E-01
11740	GSM237978	GSE9348	Colorectal Cancer	Colorectal	3.78E-25	-5.93E-01	9.88E-08	-6.49E-01
11741	GSM237979	GSE9348	Colorectal Cancer	Colorectal	1.20E-46	-8.13E-01	7.92E-15	-9.21E-01
11742	GSM237980	GSE9348	Colorectal Cancer	Colorectal	5.23E-41	-7.62E-01	7.15E-14	-8.89E-01
11743	GSM237981	GSE9348	Colorectal Cancer	Colorectal	7.26E-33	-6.81E-01	6.13E-10	-7.44E-01
11744	GSM237982	GSE9348	Colorectal Cancer	Colorectal	2.64E-02	-1.51E-01	2.31E-01	-1.96E-01
11745	GSM237983	GSE9348	Colorectal Cancer	Colorectal	1.48E-15	-4.62E-01	7.73E-05	-4.97E-01
11746	GSM820048	GSE33113	Colorectal Cancer	Colorectal	2.19E-21	-5.46E-01	8.21E-09	-6.97E-01
11747	GSM820049	GSE33113	Colorectal Cancer	Colorectal	9.55E-28	-6.24E-01	1.45E-09	-7.29E-01
11748	GSM820050	GSE33113	Colorectal Cancer	Colorectal	2.25E-22	-5.59E-01	4.30E-08	-6.65E-01
11749	GSM820051	GSE33113	Colorectal Cancer	Colorectal	2.20E-31	-6.65E-01	2.16E-11	-8.01E-01
11750	GSM820052	GSE33113	Colorectal Cancer	Colorectal	9.12E-35	-7.00E-01	8.09E-12	-8.17E-01
11751	GSM820053	GSE33113	Colorectal Cancer	Colorectal	3.21E-22	-5.57E-01	9.39E-07	-6.02E-01
11752	GSM820054	GSE33113	Colorectal Cancer	Colorectal	1.50E-19	-5.21E-01	1.07E-05	-5.47E-01
11753	GSM820055	GSE33113	Colorectal Cancer	Colorectal	3.27E-18	-5.02E-01	3.11E-07	-6.25E-01
11754	GSM820056	GSE33113	Colorectal Cancer	Colorectal	4.74E-29	-6.39E-01	5.67E-11	-7.85E-01
11755	GSM820057	GSE33113	Colorectal Cancer	Colorectal	6.89E-13	-4.19E-01	7.36E-05	-4.98E-01
11756	GSM820058	GSE33113	Colorectal Cancer	Colorectal	1.42E-27	-6.22E-01	8.49E-09	-6.97E-01
11757	GSM820059	GSE33113	Colorectal Cancer	Colorectal	8.57E-02	-1.24E-01	2.31E-01	-1.96E-01
11758	GSM820060	GSE33113	Colorectal Cancer	Colorectal	9.88E-18	-4.95E-01	4.47E-07	-6.18E-01
11759	GSM820061	GSE33113	Colorectal Cancer	Colorectal	1.02E-07	-3.17E-01	8.02E-03	-3.55E-01
11760	GSM820062	GSE33113	Colorectal Cancer	Colorectal	2.39E-35	-7.06E-01	1.46E-10	-7.69E-01
11761	GSM820063	GSE33113	Colorectal Cancer	Colorectal	5.05E-38	-7.33E-01	7.47E-14	-8.88E-01

11762	GSM820064	GSE33113	Colorectal Cancer	Colorectal	1.45E-30	-6.56E-01	9.11E-10	-7.37E-01
11763	GSM820065	GSE33113	Colorectal Cancer	Colorectal	4.25E-24	-5.80E-01	1.44E-07	-6.41E-01
11764	GSM820066	GSE33113	Colorectal Cancer	Colorectal	1.22E-35	-7.09E-01	2.16E-11	-8.01E-01
11765	GSM820067	GSE33113	Colorectal Cancer	Colorectal	4.01E-17	-4.86E-01	9.39E-07	-6.02E-01
11766	GSM820068	GSE33113	Colorectal Cancer	Colorectal	5.01E-30	-6.50E-01	1.40E-09	-7.30E-01
11767	GSM820069	GSE33113	Colorectal Cancer	Colorectal	6.94E-51	-8.50E-01	2.40E-14	-9.05E-01
11768	GSM820070	GSE33113	Colorectal Cancer	Colorectal	5.02E-43	-7.81E-01	6.23E-13	-8.57E-01
11769	GSM820071	GSE33113	Colorectal Cancer	Colorectal	2.97E-08	-3.29E-01	2.72E-02	-3.07E-01
11770	GSM820072	GSE33113	Colorectal Cancer	Colorectal	3.86E-03	1.87E-01	5.38E-02	2.76E-01
11771	GSM820073	GSE33113	Colorectal Cancer	Colorectal	2.21E-34	-6.96E-01	5.45E-09	-7.05E-01
11772	GSM820074	GSE33113	Colorectal Cancer	Colorectal	1.73E-12	-4.12E-01	7.44E-04	-4.34E-01
11773	GSM820075	GSE33113	Colorectal Cancer	Colorectal	2.83E-11	-3.90E-01	4.07E-04	-4.51E-01
11774	GSM820076	GSE33113	Colorectal Cancer	Colorectal	4.03E-01	7.54E-02	1.17E-01	2.37E-01
11775	GSM820077	GSE33113	Colorectal Cancer	Colorectal	1.42E-08	-3.36E-01	4.22E-03	-3.78E-01
11776	GSM820078	GSE33113	Colorectal Cancer	Colorectal	4.69E-26	-6.04E-01	8.21E-09	-6.97E-01
11777	GSM820079	GSE33113	Colorectal Cancer	Colorectal	1.71E-53	-8.72E-01	7.47E-14	-8.88E-01
11778	GSM820080	GSE33113	Colorectal Cancer	Colorectal	1.00E-26	-6.12E-01	5.27E-09	-7.05E-01
11779	GSM820081	GSE33113	Colorectal Cancer	Colorectal	6.10E-16	-4.68E-01	2.94E-05	-5.22E-01
11780	GSM820082	GSE33113	Colorectal Cancer	Colorectal	1.01E-36	-7.20E-01	2.28E-10	-7.61E-01
11781	GSM820083	GSE33113	Colorectal Cancer	Colorectal	8.57E-02	-1.24E-01	5.39E-01	-1.27E-01
11782	GSM820084	GSE33113	Colorectal Cancer	Colorectal	7.72E-22	-5.52E-01	9.57E-08	-6.50E-01
11783	GSM820085	GSE33113	Colorectal Cancer	Colorectal	1.08E-19	-5.23E-01	5.49E-05	-5.06E-01
11784	GSM820086	GSE33113	Colorectal Cancer	Colorectal	5.84E-35	-7.02E-01	5.91E-10	-7.45E-01
11785	GSM820087	GSE33113	Colorectal Cancer	Colorectal	4.49E-18	-5.00E-01	1.93E-06	-5.86E-01
11786	GSM820088	GSE33113	Colorectal Cancer	Colorectal	3.53E-13	-4.24E-01	1.98E-03	-4.03E-01
11787	GSM820089	GSE33113	Colorectal Cancer	Colorectal	1.17E-31	-6.68E-01	9.30E-11	-7.77E-01
11788	GSM820090	GSE33113	Colorectal Cancer	Colorectal	6.13E-03	-1.79E-01	2.06E-01	-2.03E-01
11789	GSM820091	GSE33113	Colorectal Cancer	Colorectal	4.09E-15	-4.55E-01	1.07E-05	-5.47E-01
11790	GSM820092	GSE33113	Colorectal Cancer	Colorectal	7.11E-29	-6.37E-01	2.29E-09	-7.21E-01
11791	GSM820093	GSE33113	Colorectal Cancer	Colorectal	3.26E-32	-6.74E-01	5.27E-09	-7.05E-01
11792	GSM820094	GSE33113	Colorectal Cancer	Colorectal	6.89E-14	-4.36E-01	3.26E-04	-4.58E-01
11793	GSM820095	GSE33113	Colorectal Cancer	Colorectal	1.18E-30	-6.57E-01	1.23E-08	-6.90E-01
11794	GSM820096	GSE33113	Colorectal Cancer	Colorectal	4.55E-14	-4.38E-01	1.07E-05	-5.47E-01
11795	GSM820097	GSE33113	Colorectal Cancer	Colorectal	6.89E-13	-4.19E-01	4.16E-04	-4.51E-01
11796	GSM820098	GSE33113	Colorectal Cancer	Colorectal	3.43E-17	-4.87E-01	5.74E-06	-5.61E-01
11797	GSM820099	GSE33113	Colorectal Cancer	Colorectal	9.18E-08	-3.18E-01	4.00E-03	-3.80E-01
11798	GSM820100	GSE33113	Colorectal Cancer	Colorectal	1.95E-08	-3.33E-01	4.98E-03	-3.72E-01
11799	GSM820101	GSE33113	Colorectal Cancer	Colorectal	2.39E-35	-7.06E-01	1.07E-12	-8.48E-01
11800	GSM820102	GSE33113	Colorectal Cancer	Colorectal	4.55E-14	-4.38E-01	7.73E-05	-4.97E-01
11801	GSM820103	GSE33113	Colorectal Cancer	Colorectal	2.20E-31	-6.65E-01	3.48E-09	-7.13E-01
11802	GSM820104	GSE33113	Colorectal Cancer	Colorectal	1.83E-01	-1.03E-01	5.00E-01	1.35E-01
11803	GSM820105	GSE33113	Colorectal Cancer	Colorectal	9.12E-35	-7.00E-01	2.97E-12	-8.32E-01
11804	GSM820106	GSE33113	Colorectal Cancer	Colorectal	8.81E-44	-7.88E-01	2.40E-14	-9.05E-01
11805	GSM820107	GSE33113	Colorectal Cancer	Colorectal	7.72E-22	-5.52E-01	1.41E-06	-5.93E-01
11806	GSM820108	GSE33113	Colorectal Cancer	Colorectal	3.10E-21	-5.44E-01	2.79E-06	-5.78E-01
11807	GSM820109	GSE33113	Colorectal Cancer	Colorectal	4.33E-01	-7.24E-02	1.00E-01	2.45E-01
11808	GSM820110	GSE33113	Colorectal Cancer	Colorectal	6.80E-27	-6.14E-01	5.27E-09	-7.05E-01
11809	GSM820111	GSE33113	Colorectal Cancer	Colorectal	1.91E-35	-7.07E-01	3.82E-10	-7.52E-01
11810	GSM820112	GSE33113	Colorectal Cancer	Colorectal	2.01E-01	-1.00E-01	3.49E-01	-1.66E-01
11811	GSM820113	GSE33113	Colorectal Cancer	Colorectal	8.26E-27	-6.13E-01	5.27E-09	-7.05E-01
11812	GSM820114	GSE33113	Colorectal Cancer	Colorectal	2.20E-31	-6.65E-01	1.46E-10	-7.69E-01
11813	GSM820115	GSE33113	Colorectal Cancer	Colorectal	1.01E-37	-7.30E-01	9.30E-11	-7.77E-01
11814	GSM820116	GSE33113	Colorectal Cancer	Colorectal	2.79E-18	-5.03E-01	1.50E-05	-5.38E-01
11815	GSM820117	GSE33113	Colorectal Cancer	Colorectal	8.50E-11	-3.81E-01	5.64E-04	-4.42E-01
11816	GSM820118	GSE33113	Colorectal Cancer	Colorectal	1.83E-23	-5.72E-01	1.90E-08	-6.81E-01
11817	GSM820119	GSE33113	Colorectal Cancer	Colorectal	7.21E-18	-4.97E-01	2.87E-05	-5.23E-01
11818	GSM820120	GSE33113	Colorectal Cancer	Colorectal	1.45E-43	-7.86E-01	2.97E-12	-8.32E-01
11819	GSM820121	GSE33113	Colorectal Cancer	Colorectal	2.01E-01	-1.00E-01	4.20E-01	1.50E-01
11820	GSM820122	GSE33113	Colorectal Cancer	Colorectal	1.10E-15	-4.64E-01	1.50E-05	-5.38E-01
11821	GSM820123	GSE33113	Colorectal Cancer	Colorectal	1.34E-11	-3.96E-01	7.54E-05	-4.98E-01
11822	GSM820124	GSE33113	Colorectal Cancer	Colorectal	5.26E-18	-4.99E-01	1.93E-06	-5.86E-01
11823	GSM820125	GSE33113	Colorectal Cancer	Colorectal	1.27E-19	-5.22E-01	2.72E-06	-5.78E-01
11824	GSM820126	GSE33113	Colorectal Cancer	Colorectal	1.63E-03	-2.00E-01	1.75E-01	-2.13E-01
11825	GSM820127	GSE33113	Colorectal Cancer	Colorectal	1.02E-07	-3.17E-01	6.39E-03	-3.63E-01
11826	GSM820128	GSE33113	Colorectal Cancer	Colorectal	9.14E-20	-5.24E-01	1.40E-07	-6.42E-01
11827	GSM820129	GSE33113	Colorectal Cancer	Colorectal	2.15E-25	-5.96E-01	5.45E-09	-7.05E-01
11828	GSM820130	GSE33113	Colorectal Cancer	Colorectal	1.77E-19	-5.20E-01	1.41E-06	-5.93E-01
11829	GSM820131	GSE33113	Colorectal Cancer	Colorectal	2.94E-17	-4.88E-01	4.13E-06	-5.69E-01
11830	GSM820132	GSE33113	Colorectal Cancer	Colorectal	1.14E-34	-6.99E-01	1.52E-10	-7.68E-01
11831	GSM820133	GSE33113	Colorectal Cancer	Colorectal	2.21E-10	-3.73E-01	4.98E-03	-3.72E-01
11832	GSM820134	GSE33113	Colorectal Cancer	Colorectal	1.89E-39	-7.47E-01	5.01E-12	-8.24E-01
11833	GSM820135	GSE33113	Colorectal Cancer	Colorectal	5.36E-09	-3.45E-01	3.26E-04	-4.58E-01
11834	GSM820136	GSE33113	Colorectal Cancer	Colorectal	2.14E-16	-4.75E-01	8.16E-06	-5.53E-01
11835	GSM820137	GSE33113	Colorectal Cancer	Colorectal	6.37E-38	-7.32E-01	8.09E-12	-8.17E-01

11836	GSM656544	GSE26682	Colorectal Cancer	Colorectal	9.01E-33	-6.80E-01	1.46E-10	-7.69E-01
11837	GSM656547	GSE26682	Colorectal Cancer	Colorectal	1.72E-11	-3.94E-01	5.49E-05	-5.06E-01
11838	GSM656554	GSE26682	Colorectal Cancer	Colorectal	4.68E-35	-7.03E-01	1.31E-13	-8.80E-01
11839	GSM656562	GSE26682	Colorectal Cancer	Colorectal	2.21E-01	-9.72E-02	6.60E-01	-1.04E-01
11840	GSM656563	GSE26682	Colorectal Cancer	Colorectal	2.02E-20	-5.33E-01	4.24E-06	-5.68E-01
11841	GSM656565	GSE26682	Colorectal Cancer	Colorectal	1.18E-44	-7.96E-01	6.23E-13	-8.57E-01
11842	GSM656566	GSE26682	Colorectal Cancer	Colorectal	1.04E-13	-4.33E-01	1.07E-05	-5.47E-01
11843	GSM656571	GSE26682	Colorectal Cancer	Colorectal	7.40E-17	-4.82E-01	1.50E-05	-5.38E-01
11844	GSM656572	GSE26682	Colorectal Cancer	Colorectal	4.10E-11	-3.87E-01	7.28E-04	-4.34E-01
11845	GSM656576	GSE26682	Colorectal Cancer	Colorectal	7.26E-33	-6.81E-01	2.37E-10	-7.61E-01
11846	GSM656577	GSE26682	Colorectal Cancer	Colorectal	3.75E-01	-7.84E-02	3.70E-02	2.93E-01
11847	GSM656578	GSE26682	Colorectal Cancer	Colorectal	2.21E-11	-3.92E-01	7.19E-05	-4.99E-01
11848	GSM656579	GSE26682	Colorectal Cancer	Colorectal	5.61E-46	-8.08E-01	7.15E-14	-8.89E-01
11849	GSM656581	GSE26682	Colorectal Cancer	Colorectal	3.36E-16	-4.72E-01	5.74E-06	-5.61E-01
11850	GSM656588	GSE26682	Colorectal Cancer	Colorectal	2.02E-37	-7.27E-01	5.67E-11	-7.85E-01
11851	GSM656589	GSE26682	Colorectal Cancer	Colorectal	1.78E-30	-6.55E-01	5.70E-10	-7.45E-01
11852	GSM656591	GSE26682	Colorectal Cancer	Colorectal	1.02E-12	-4.16E-01	7.13E-04	-4.35E-01
11853	GSM656593	GSE26682	Colorectal Cancer	Colorectal	9.20E-22	-5.51E-01	1.27E-08	-6.89E-01
11854	GSM656594	GSE26682	Colorectal Cancer	Colorectal	5.02E-10	-3.66E-01	4.35E-04	-4.50E-01
11855	GSM656597	GSE26682	Colorectal Cancer	Colorectal	1.57E-38	-7.38E-01	1.31E-13	-8.80E-01
11856	GSM656599	GSE26682	Colorectal Cancer	Colorectal	3.10E-06	-2.82E-01	3.35E-02	-2.98E-01
11857	GSM656600	GSE26682	Colorectal Cancer	Colorectal	1.58E-16	-4.77E-01	1.07E-05	-5.47E-01
11858	GSM656604	GSE26682	Colorectal Cancer	Colorectal	1.12E-32	-6.79E-01	1.75E-12	-8.41E-01
11859	GSM656605	GSE26682	Colorectal Cancer	Colorectal	9.04E-03	-1.72E-01	5.93E-01	-1.17E-01
11860	GSM656606	GSE26682	Colorectal Cancer	Colorectal	6.00E-14	-4.37E-01	3.98E-05	-5.14E-01
11861	GSM656608	GSE26682	Colorectal Cancer	Colorectal	2.59E-06	-2.84E-01	2.31E-02	-3.14E-01
11862	GSM656611	GSE26682	Colorectal Cancer	Colorectal	8.39E-15	-4.50E-01	7.44E-04	-4.34E-01
11863	GSM656614	GSE26682	Colorectal Cancer	Colorectal	2.55E-37	-7.26E-01	4.82E-12	-8.25E-01
11864	GSM656615	GSE26682	Colorectal Cancer	Colorectal	3.54E-28	-6.29E-01	2.37E-10	-7.61E-01
11865	GSM656616	GSE26682	Colorectal Cancer	Colorectal	2.36E-13	-4.27E-01	2.05E-05	-5.31E-01
11866	GSM656620	GSE26682	Colorectal Cancer	Colorectal	6.16E-21	-5.40E-01	4.61E-07	-6.17E-01
11867	GSM656621	GSE26682	Colorectal Cancer	Colorectal	2.56E-12	-4.09E-01	9.17E-04	-4.27E-01
11868	GSM656622	GSE26682	Colorectal Cancer	Colorectal	1.85E-03	-1.98E-01	4.90E-01	-1.37E-01
11869	GSM656623	GSE26682	Colorectal Cancer	Colorectal	1.72E-11	-3.94E-01	7.28E-04	-4.34E-01
11870	GSM656624	GSE26682	Colorectal Cancer	Colorectal	2.86E-03	-1.91E-01	7.46E-02	-2.60E-01
11871	GSM656626	GSE26682	Colorectal Cancer	Colorectal	1.27E-08	-3.37E-01	1.00E-02	-3.47E-01
11872	GSM656628	GSE26682	Colorectal Cancer	Colorectal	1.24E-07	-3.15E-01	1.54E-02	-3.30E-01
11873	GSM656629	GSE26682	Colorectal Cancer	Colorectal	1.98E-33	-6.87E-01	9.11E-10	-7.37E-01
11874	GSM656630	GSE26682	Colorectal Cancer	Colorectal	9.27E-05	-2.41E-01	8.88E-02	-2.51E-01
11875	GSM656632	GSE26682	Colorectal Cancer	Colorectal	5.30E-04	-2.17E-01	1.18E-01	-2.36E-01
11876	GSM656633	GSE26682	Colorectal Cancer	Colorectal	5.44E-22	-5.54E-01	6.79E-07	-6.09E-01
11877	GSM656637	GSE26682	Colorectal Cancer	Colorectal	1.55E-10	-3.76E-01	3.26E-04	-4.58E-01
11878	GSM656638	GSE26682	Colorectal Cancer	Colorectal	1.06E-23	-5.75E-01	2.15E-07	-6.33E-01
11879	GSM656641	GSE26682	Colorectal Cancer	Colorectal	1.47E-25	-5.98E-01	1.27E-08	-6.89E-01
11880	GSM656643	GSE26682	Colorectal Cancer	Colorectal	3.68E-21	-5.43E-01	1.93E-06	-5.86E-01
11881	GSM656645	GSE26682	Colorectal Cancer	Colorectal	2.29E-15	-4.59E-01	4.25E-04	-4.50E-01
11882	GSM656647	GSE26682	Colorectal Cancer	Colorectal	1.52E-12	-4.13E-01	7.44E-04	-4.34E-01
11883	GSM656648	GSE26682	Colorectal Cancer	Colorectal	2.46E-19	-5.18E-01	5.58E-06	-5.62E-01
11884	GSM656649	GSE26682	Colorectal Cancer	Colorectal	4.49E-18	-5.00E-01	1.12E-05	-5.45E-01
11885	GSM656650	GSE26682	Colorectal Cancer	Colorectal	2.72E-31	-6.64E-01	1.45E-09	-7.29E-01
11886	GSM656651	GSE26682	Colorectal Cancer	Colorectal	5.64E-10	-3.65E-01	2.49E-03	-3.96E-01
11887	GSM656652	GSE26682	Colorectal Cancer	Colorectal	2.29E-15	-4.59E-01	2.16E-05	-5.30E-01
11888	GSM656653	GSE26682	Colorectal Cancer	Colorectal	2.60E-46	-8.11E-01	1.31E-13	-8.80E-01
11889	GSM656654	GSE26682	Colorectal Cancer	Colorectal	3.16E-29	-6.41E-01	3.68E-10	-7.53E-01
11890	GSM656655	GSE26682	Colorectal Cancer	Colorectal	2.70E-30	-6.53E-01	1.27E-08	-6.89E-01
11891	GSM656656	GSE26682	Colorectal Cancer	Colorectal	1.17E-16	-4.79E-01	1.40E-07	-6.42E-01
11892	GSM656658	GSE26682	Colorectal Cancer	Colorectal	9.20E-22	-5.51E-01	2.15E-07	-6.33E-01
11893	GSM656659	GSE26682	Colorectal Cancer	Colorectal	2.63E-23	-5.70E-01	5.27E-09	-7.05E-01
11894	GSM656660	GSE26682	Colorectal Cancer	Colorectal	1.08E-19	-5.23E-01	1.10E-05	-5.46E-01
11895	GSM656661	GSE26682	Colorectal Cancer	Colorectal	1.57E-13	-4.30E-01	1.27E-03	-4.17E-01
11896	GSM656664	GSE26682	Colorectal Cancer	Colorectal	3.09E-13	-4.25E-01	1.03E-04	-4.90E-01
11897	GSM656665	GSE26682	Colorectal Cancer	Colorectal	9.23E-09	-3.40E-01	2.64E-03	-3.94E-01
11898	GSM656666	GSE26682	Colorectal Cancer	Colorectal	5.84E-35	-7.02E-01	2.37E-10	-7.61E-01
11899	GSM656667	GSE26682	Colorectal Cancer	Colorectal	1.55E-21	-5.48E-01	1.40E-07	-6.42E-01
11900	GSM656668	GSE26682	Colorectal Cancer	Colorectal	1.55E-21	-5.48E-01	1.40E-09	-7.30E-01
11901	GSM656670	GSE26682	Colorectal Cancer	Colorectal	8.66E-21	-5.38E-01	7.95E-06	-5.54E-01
11902	GSM656671	GSE26682	Colorectal Cancer	Colorectal	8.01E-25	-5.89E-01	4.47E-07	-6.18E-01
11903	GSM656674	GSE26682	Colorectal Cancer	Colorectal	3.78E-23	-5.68E-01	2.21E-09	-7.21E-01
11904	GSM656677	GSE26682	Colorectal Cancer	Colorectal	1.39E-01	1.11E-01	5.24E-02	2.77E-01
11905	GSM656680	GSE26682	Colorectal Cancer	Colorectal	6.97E-04	-2.13E-01	3.52E-01	-1.65E-01
11906	GSM656681	GSE26682	Colorectal Cancer	Colorectal	1.98E-09	-3.54E-01	4.07E-03	-3.79E-01
11907	GSM656682	GSE26682	Colorectal Cancer	Colorectal	7.70E-19	-5.11E-01	2.91E-08	-6.73E-01
11908	GSM656684	GSE26682	Colorectal Cancer	Colorectal	1.18E-39	-7.49E-01	4.63E-12	-8.25E-01
11909	GSM656685	GSE26682	Colorectal Cancer	Colorectal	7.79E-39	-7.41E-01	3.68E-10	-7.53E-01

11910	GSM656686	GSE26682	Colorectal Cancer	Colorectal	5.94E-02	1.33E-01	1.00E-02	3.47E-01
11911	GSM656689	GSE26682	Colorectal Cancer	Colorectal	1.77E-19	-5.20E-01	1.50E-05	-5.38E-01
11912	GSM656697	GSE26682	Colorectal Cancer	Colorectal	9.11E-04	-2.09E-01	1.38E-01	-2.27E-01
11913	GSM656708	GSE26682	Colorectal Cancer	Colorectal	3.53E-24	-5.81E-01	1.90E-08	-6.81E-01
11914	GSM656709	GSE26682	Colorectal Cancer	Colorectal	3.86E-09	-3.48E-01	2.77E-02	-3.06E-01
11915	GSM656712	GSE26682	Colorectal Cancer	Colorectal	1.10E-21	-5.50E-01	1.02E-07	-6.48E-01
11916	GSM656713	GSE26682	Colorectal Cancer	Colorectal	1.04E-11	-3.98E-01	5.36E-05	-5.07E-01
11917	GSM656716	GSE26682	Colorectal Cancer	Colorectal	6.16E-21	-5.40E-01	4.13E-06	-5.69E-01
11918	GSM656717	GSE26682	Colorectal Cancer	Colorectal	8.39E-15	-4.50E-01	1.77E-04	-4.75E-01
11919	GSM656718	GSE26682	Colorectal Cancer	Colorectal	9.19E-45	-7.97E-01	2.18E-13	-8.72E-01
11920	GSM656719	GSE26682	Colorectal Cancer	Colorectal	1.40E-24	-5.86E-01	4.44E-08	-6.65E-01
11921	GSM656722	GSE26682	Colorectal Cancer	Colorectal	4.94E-01	-6.65E-02	3.14E-01	1.74E-01
11922	GSM656723	GSE26682	Colorectal Cancer	Colorectal	2.84E-05	-2.56E-01	4.03E-02	-2.90E-01
11923	GSM656724	GSE26682	Colorectal Cancer	Colorectal	1.72E-18	-5.06E-01	4.47E-07	-6.18E-01
11924	GSM656725	GSE26682	Colorectal Cancer	Colorectal	7.50E-42	-7.70E-01	8.09E-12	-8.17E-01
11925	GSM656727	GSE26682	Colorectal Cancer	Colorectal	2.29E-15	-4.59E-01	6.74E-03	-3.61E-01
11926	GSM656728	GSE26682	Colorectal Cancer	Colorectal	3.78E-25	-5.93E-01	1.90E-08	-6.81E-01
11927	GSM656729	GSE26682	Colorectal Cancer	Colorectal	4.72E-15	-4.54E-01	3.88E-05	-5.15E-01
11928	GSM656730	GSE26682	Colorectal Cancer	Colorectal	1.01E-36	-7.20E-01	2.16E-11	-8.01E-01
11929	GSM656733	GSE26682	Colorectal Cancer	Colorectal	4.71E-33	-6.83E-01	3.48E-09	-7.13E-01
11930	GSM656734	GSE26682	Colorectal Cancer	Colorectal	7.75E-23	-5.64E-01	1.49E-07	-6.41E-01
11931	GSM656738	GSE26682	Colorectal Cancer	Colorectal	1.49E-39	-7.48E-01	5.89E-11	-7.84E-01
11932	GSM656740	GSE26682	Colorectal Cancer	Colorectal	3.53E-13	-4.24E-01	1.81E-04	-4.74E-01
11933	GSM656741	GSE26682	Colorectal Cancer	Colorectal	1.16E-01	-1.16E-01	1.77E-01	-2.13E-01
11934	GSM656743	GSE26682	Colorectal Cancer	Colorectal	5.08E-37	-7.23E-01	6.13E-10	-7.44E-01
11935	GSM656744	GSE26682	Colorectal Cancer	Colorectal	2.90E-19	-5.17E-01	1.93E-06	-5.86E-01
11936	GSM656745	GSE26682	Colorectal Cancer	Colorectal	3.78E-25	-5.93E-01	1.45E-09	-7.29E-01
11937	GSM656746	GSE26682	Colorectal Cancer	Colorectal	2.61E-21	-5.45E-01	4.47E-07	-6.18E-01
11938	GSM656747	GSE26682	Colorectal Cancer	Colorectal	6.30E-15	-4.52E-01	4.18E-05	-5.13E-01
11939	GSM656748	GSE26682	Colorectal Cancer	Colorectal	5.44E-22	-5.54E-01	9.88E-08	-6.49E-01
11940	GSM656750	GSE26682	Colorectal Cancer	Colorectal	1.04E-11	-3.98E-01	2.59E-03	-3.94E-01
11941	GSM656751	GSE26682	Colorectal Cancer	Colorectal	4.56E-25	-5.92E-01	2.91E-08	-6.73E-01
11942	GSM656752	GSE26682	Colorectal Cancer	Colorectal	4.08E-30	-6.51E-01	3.82E-10	-7.52E-01
11943	GSM656753	GSE26682	Colorectal Cancer	Colorectal	8.66E-21	-5.38E-01	1.93E-06	-5.86E-01
11944	GSM656754	GSE26682	Colorectal Cancer	Colorectal	1.26E-09	-3.58E-01	7.73E-05	-4.97E-01
11945	GSM656755	GSE26682	Colorectal Cancer	Colorectal	1.37E-13	-4.31E-01	4.07E-04	-4.51E-01
11946	GSM656756	GSE26682	Colorectal Cancer	Colorectal	4.62E-13	-4.22E-01	7.44E-04	-4.34E-01
11947	GSM656757	GSE26682	Colorectal Cancer	Colorectal	2.49E-16	-4.74E-01	2.16E-05	-5.30E-01
11948	GSM656758	GSE26682	Colorectal Cancer	Colorectal	9.26E-23	-5.63E-01	4.47E-07	-6.18E-01
11949	GSM656759	GSE26682	Colorectal Cancer	Colorectal	7.65E-32	-6.70E-01	2.21E-09	-7.21E-01
11950	GSM656760	GSE26682	Colorectal Cancer	Colorectal	7.91E-14	-4.35E-01	7.13E-04	-4.35E-01
11951	GSM656761	GSE26682	Colorectal Cancer	Colorectal	3.44E-14	-4.40E-01	2.33E-04	-4.67E-01
11952	GSM656763	GSE26682	Colorectal Cancer	Colorectal	7.40E-17	-4.82E-01	4.18E-05	-5.13E-01
11953	GSM656764	GSE26682	Colorectal Cancer	Colorectal	1.74E-03	-1.99E-01	2.14E-01	-2.01E-01
11954	GSM656765	GSE26682	Colorectal Cancer	Colorectal	1.25E-06	-2.92E-01	7.88E-03	-3.56E-01
11955	GSM656766	GSE26682	Colorectal Cancer	Colorectal	6.48E-23	-5.65E-01	3.11E-07	-6.25E-01
11956	GSM656767	GSE26682	Colorectal Cancer	Colorectal	2.06E-13	-4.28E-01	2.38E-04	-4.67E-01
11957	GSM656771	GSE26682	Colorectal Cancer	Colorectal	3.44E-34	-6.94E-01	3.58E-11	-7.92E-01
11958	GSM656773	GSE26682	Colorectal Cancer	Colorectal	6.18E-32	-6.71E-01	5.45E-09	-7.05E-01
11959	GSM656774	GSE26682	Colorectal Cancer	Colorectal	6.39E-37	-7.22E-01	2.16E-11	-8.01E-01
11960	GSM656778	GSE26682	Colorectal Cancer	Colorectal	6.43E-28	-6.26E-01	3.68E-10	-7.53E-01
11961	GSM656779	GSE26682	Colorectal Cancer	Colorectal	6.16E-18	-4.98E-01	5.49E-05	-5.06E-01
11962	GSM656780	GSE26682	Colorectal Cancer	Colorectal	8.84E-24	-5.76E-01	9.66E-07	-6.01E-01
11963	GSM656781	GSE26682	Colorectal Cancer	Colorectal	9.65E-25	-5.88E-01	7.73E-05	-4.97E-01
11964	GSM656786	GSE26682	Colorectal Cancer	Colorectal	7.75E-23	-5.64E-01	6.40E-07	-6.10E-01
11965	GSM656788	GSE26682	Colorectal Cancer	Colorectal	2.10E-27	-6.20E-01	8.21E-09	-6.97E-01
11966	GSM656789	GSE26682	Colorectal Cancer	Colorectal	4.49E-08	-3.25E-01	9.68E-03	-3.48E-01
11967	GSM656790	GSE26682	Colorectal Cancer	Colorectal	7.84E-28	-6.25E-01	2.91E-08	-6.73E-01
11968	GSM656791	GSE26682	Colorectal Cancer	Colorectal	6.43E-28	-6.26E-01	4.30E-08	-6.65E-01
11969	GSM656792	GSE26682	Colorectal Cancer	Colorectal	2.01E-45	-8.03E-01	1.75E-12	-8.41E-01
11970	GSM656793	GSE26682	Colorectal Cancer	Colorectal	3.54E-15	-4.56E-01	5.36E-05	-5.07E-01
11971	GSM656796	GSE26682	Colorectal Cancer	Colorectal	4.67E-17	-4.85E-01	3.90E-06	-5.70E-01
11972	GSM656797	GSE26682	Colorectal Cancer	Colorectal	1.71E-20	-5.34E-01	6.40E-07	-6.10E-01
11973	GSM656799	GSE26682	Colorectal Cancer	Colorectal	1.71E-15	-4.61E-01	1.08E-04	-4.88E-01
11974	GSM656801	GSE26682	Colorectal Cancer	Colorectal	2.02E-37	-7.27E-01	8.09E-12	-8.17E-01
11975	GSM656805	GSE26682	Colorectal Cancer	Colorectal	5.60E-27	-6.15E-01	4.30E-08	-6.65E-01
11976	GSM656807	GSE26682	Colorectal Cancer	Colorectal	4.32E-28	-6.28E-01	2.21E-09	-7.21E-01
11977	GSM656808	GSE26682	Colorectal Cancer	Colorectal	2.14E-16	-4.75E-01	1.43E-04	-4.81E-01
11978	GSM656810	GSE26682	Colorectal Cancer	Colorectal	3.24E-44	-7.92E-01	2.97E-12	-8.32E-01
11979	GSM656812	GSE26682	Colorectal Cancer	Colorectal	3.09E-09	-3.50E-01	2.59E-03	-3.94E-01
11980	GSM656813	GSE26682	Colorectal Cancer	Colorectal	3.79E-27	-6.17E-01	8.21E-09	-6.97E-01
11981	GSM656814	GSE26682	Colorectal Cancer	Colorectal	5.87E-42	-7.71E-01	2.18E-13	-8.72E-01
11982	GSM656815	GSE26682	Colorectal Cancer	Colorectal	4.34E-46	-8.09E-01	5.01E-12	-8.24E-01
11983	GSM656817	GSE26682	Colorectal Cancer	Colorectal	1.61E-37	-7.28E-01	8.09E-12	-8.17E-01

11984	GSM656818	GSE26682	Colorectal Cancer	Colorectal	2.56E-27	-6.19E-01	1.96E-08	-6.81E-01
11985	GSM656821	GSE26682	Colorectal Cancer	Colorectal	2.46E-33	-6.86E-01	9.30E-11	-7.77E-01
11986	GSM656823	GSE26682	Colorectal Cancer	Colorectal	4.87E-12	-4.04E-01	1.40E-04	-4.81E-01
11987	GSM656826	GSE26682	Colorectal Cancer	Colorectal	4.47E-10	-3.67E-01	5.40E-04	-4.43E-01
11988	GSM656828	GSE26682	Colorectal Cancer	Colorectal	1.06E-23	-5.75E-01	1.23E-08	-6.90E-01
11989	GSM656829	GSE26682	Colorectal Cancer	Colorectal	1.08E-19	-5.23E-01	2.94E-05	-5.22E-01
11990	GSM656835	GSE26682	Colorectal Cancer	Colorectal	1.28E-37	-7.29E-01	2.16E-11	-8.01E-01
11991	GSM656542	GSE26682	Colorectal Cancer	Colorectal	1.17E-12	-4.15E-01	1.17E-03	-4.20E-01
11992	GSM656543	GSE26682	Colorectal Cancer	Colorectal	5.10E-24	-5.79E-01	9.88E-08	-6.49E-01
11993	GSM656545	GSE26682	Colorectal Cancer	Colorectal	2.60E-14	-4.42E-01	2.11E-05	-5.30E-01
11994	GSM656546	GSE26682	Colorectal Cancer	Colorectal	3.36E-16	-4.72E-01	1.07E-05	-5.47E-01
11995	GSM656548	GSE26682	Colorectal Cancer	Colorectal	1.84E-16	-4.76E-01	1.63E-05	-5.37E-01
11996	GSM656549	GSE26682	Colorectal Cancer	Colorectal	6.48E-03	-1.78E-01	1.37E-01	-2.28E-01
11997	GSM656550	GSE26682	Colorectal Cancer	Colorectal	1.18E-11	-3.97E-01	5.52E-04	-4.43E-01
11998	GSM656551	GSE26682	Colorectal Cancer	Colorectal	3.27E-18	-5.02E-01	1.33E-06	-5.94E-01
11999	GSM656552	GSE26682	Colorectal Cancer	Colorectal	3.31E-12	-4.07E-01	2.55E-04	-4.65E-01
12000	GSM656553	GSE26682	Colorectal Cancer	Colorectal	1.40E-24	-5.86E-01	1.40E-07	-6.42E-01
12001	GSM656555	GSE26682	Colorectal Cancer	Colorectal	8.81E-44	-7.88E-01	3.58E-11	-7.92E-01
12002	GSM656556	GSE26682	Colorectal Cancer	Colorectal	1.25E-06	-2.92E-01	9.17E-04	-4.27E-01
12003	GSM656557	GSE26682	Colorectal Cancer	Colorectal	2.99E-35	-7.05E-01	2.21E-09	-7.21E-01
12004	GSM656558	GSE26682	Colorectal Cancer	Colorectal	4.32E-28	-6.28E-01	1.27E-08	-6.89E-01
12005	GSM656559	GSE26682	Colorectal Cancer	Colorectal	1.27E-19	-5.22E-01	1.93E-06	-5.86E-01
12006	GSM656560	GSE26682	Colorectal Cancer	Colorectal	4.03E-07	-3.04E-01	3.35E-02	-2.98E-01
12007	GSM656561	GSE26682	Colorectal Cancer	Colorectal	1.05E-05	-2.68E-01	7.84E-02	-2.58E-01
12008	GSM656564	GSE26682	Colorectal Cancer	Colorectal	1.72E-49	-8.38E-01	2.51E-14	-9.04E-01
12009	GSM656567	GSE26682	Colorectal Cancer	Colorectal	1.98E-09	-3.54E-01	5.07E-03	-3.71E-01
12010	GSM656568	GSE26682	Colorectal Cancer	Colorectal	7.13E-12	-4.01E-01	1.59E-03	-4.10E-01
12011	GSM656569	GSE26682	Colorectal Cancer	Colorectal	2.04E-07	-3.11E-01	9.68E-03	-3.48E-01
12012	GSM656570	GSE26682	Colorectal Cancer	Colorectal	1.27E-19	-5.22E-01	1.10E-05	-5.46E-01
12013	GSM656573	GSE26682	Colorectal Cancer	Colorectal	2.90E-28	-6.30E-01	1.27E-08	-6.89E-01
12014	GSM656574	GSE26682	Colorectal Cancer	Colorectal	3.29E-08	-3.28E-01	9.68E-03	-3.48E-01
12015	GSM656575	GSE26682	Colorectal Cancer	Colorectal	1.29E-14	-4.47E-01	1.85E-04	-4.74E-01
12016	GSM656580	GSE26682	Colorectal Cancer	Colorectal	2.09E-19	-5.19E-01	1.50E-05	-5.38E-01
12017	GSM656582	GSE26682	Colorectal Cancer	Colorectal	1.46E-04	-2.35E-01	2.81E-02	-3.05E-01
12018	GSM656583	GSE26682	Colorectal Cancer	Colorectal	1.85E-17	-4.91E-01	5.43E-06	-5.63E-01
12019	GSM656584	GSE26682	Colorectal Cancer	Colorectal	3.24E-44	-7.92E-01	3.77E-13	-8.64E-01
12020	GSM656585	GSE26682	Colorectal Cancer	Colorectal	3.78E-25	-5.93E-01	9.95E-07	-6.01E-01
12021	GSM656586	GSE26682	Colorectal Cancer	Colorectal	9.51E-16	-4.65E-01	3.88E-05	-5.15E-01
12022	GSM656587	GSE26682	Colorectal Cancer	Colorectal	1.40E-29	-6.45E-01	3.48E-09	-7.13E-01
12023	GSM656590	GSE26682	Colorectal Cancer	Colorectal	2.70E-13	-4.26E-01	6.40E-07	-6.10E-01
12024	GSM656592	GSE26682	Colorectal Cancer	Colorectal	1.06E-18	-5.09E-01	7.74E-06	-5.54E-01
12025	GSM656595	GSE26682	Colorectal Cancer	Colorectal	5.23E-11	-3.85E-01	5.52E-04	-4.43E-01
12026	GSM656596	GSE26682	Colorectal Cancer	Colorectal	1.58E-16	-4.77E-01	2.87E-05	-5.23E-01
12027	GSM656598	GSE26682	Colorectal Cancer	Colorectal	2.40E-20	-5.32E-01	1.33E-06	-5.94E-01
12028	GSM656601	GSE26682	Colorectal Cancer	Colorectal	6.30E-15	-4.52E-01	3.26E-04	-4.58E-01
12029	GSM656602	GSE26682	Colorectal Cancer	Colorectal	1.27E-19	-5.22E-01	3.90E-06	-5.70E-01
12030	GSM656603	GSE26682	Colorectal Cancer	Colorectal	8.01E-25	-5.89E-01	2.09E-07	-6.34E-01
12031	GSM656607	GSE26682	Colorectal Cancer	Colorectal	2.36E-13	-4.27E-01	5.49E-05	-5.06E-01
12032	GSM656609	GSE26682	Colorectal Cancer	Colorectal	1.32E-22	-5.62E-01	9.95E-07	-6.01E-01
12033	GSM656610	GSE26682	Colorectal Cancer	Colorectal	2.56E-27	-6.19E-01	1.32E-08	-6.88E-01
12034	GSM656612	GSE26682	Colorectal Cancer	Colorectal	7.70E-19	-5.11E-01	4.61E-07	-6.17E-01
12035	GSM656613	GSE26682	Colorectal Cancer	Colorectal	2.21E-09	-3.53E-01	9.36E-04	-4.27E-01
12036	GSM656617	GSE26682	Colorectal Cancer	Colorectal	1.37E-07	-3.14E-01	7.55E-02	-2.60E-01
12037	GSM656618	GSE26682	Colorectal Cancer	Colorectal	3.90E-16	-4.71E-01	2.55E-04	-4.65E-01
12038	GSM656619	GSE26682	Colorectal Cancer	Colorectal	8.24E-02	-1.25E-01	5.46E-01	1.26E-01
12039	GSM656625	GSE26682	Colorectal Cancer	Colorectal	6.12E-08	-3.22E-01	2.17E-02	-3.16E-01
12040	GSM656627	GSE26682	Colorectal Cancer	Colorectal	7.07E-16	-4.67E-01	2.87E-05	-5.23E-01
12041	GSM656631	GSE26682	Colorectal Cancer	Colorectal	1.39E-32	-6.78E-01	1.46E-10	-7.69E-01
12042	GSM656634	GSE26682	Colorectal Cancer	Colorectal	7.84E-28	-6.25E-01	2.21E-09	-7.21E-01
12043	GSM656635	GSE26682	Colorectal Cancer	Colorectal	1.44E-20	-5.35E-01	2.91E-08	-6.73E-01
12044	GSM656636	GSE26682	Colorectal Cancer	Colorectal	2.82E-40	-7.55E-01	1.75E-12	-8.41E-01
12045	GSM656639	GSE26682	Colorectal Cancer	Colorectal	2.60E-14	-4.42E-01	1.34E-04	-4.83E-01
12046	GSM656640	GSE26682	Colorectal Cancer	Colorectal	3.62E-05	-2.53E-01	2.72E-02	-3.07E-01
12047	GSM656642	GSE26682	Colorectal Cancer	Colorectal	2.15E-25	-5.96E-01	1.90E-08	-6.81E-01
12048	GSM656644	GSE26682	Colorectal Cancer	Colorectal	2.90E-28	-6.30E-01	1.45E-09	-7.29E-01
12049	GSM656646	GSE26682	Colorectal Cancer	Colorectal	9.46E-32	-6.69E-01	3.82E-10	-7.52E-01
12050	GSM656657	GSE26682	Colorectal Cancer	Colorectal	1.42E-27	-6.22E-01	4.47E-07	-6.18E-01
12051	GSM656662	GSE26682	Colorectal Cancer	Colorectal	4.72E-15	-4.54E-01	2.94E-05	-5.22E-01
12052	GSM656663	GSE26682	Colorectal Cancer	Colorectal	1.30E-28	-6.34E-01	6.79E-07	-6.09E-01
12053	GSM656669	GSE26682	Colorectal Cancer	Colorectal	3.68E-21	-5.43E-01	4.61E-07	-6.17E-01
12054	GSM656672	GSE26682	Colorectal Cancer	Colorectal	3.44E-34	-6.94E-01	5.67E-11	-7.85E-01
12055	GSM656673	GSE26682	Colorectal Cancer	Colorectal	6.16E-30	-6.49E-01	1.02E-07	-6.48E-01
12056	GSM656675	GSE26682	Colorectal Cancer	Colorectal	2.25E-07	-3.10E-01	2.77E-02	-3.06E-01
12057	GSM656676	GSE26682	Colorectal Cancer	Colorectal	4.25E-05	-2.51E-01	7.74E-02	-2.58E-01

12058	GSM656678	GSE26682	Colorectal Cancer	Colorectal	5.81E-29	-6.38E-01	1.45E-09	-7.29E-01
12059	GSM656679	GSE26682	Colorectal Cancer	Colorectal	1.58E-09	-3.56E-01	9.83E-05	-4.91E-01
12060	GSM656683	GSE26682	Colorectal Cancer	Colorectal	2.69E-22	-5.58E-01	6.33E-08	-6.58E-01
12061	GSM656687	GSE26682	Colorectal Cancer	Colorectal	1.17E-31	-6.68E-01	1.90E-08	-6.81E-01
12062	GSM656688	GSE26682	Colorectal Cancer	Colorectal	4.11E-41	-7.63E-01	2.25E-11	-8.00E-01
12063	GSM656690	GSE26682	Colorectal Cancer	Colorectal	5.26E-18	-4.99E-01	5.43E-06	-5.63E-01
12064	GSM656691	GSE26682	Colorectal Cancer	Colorectal	4.05E-08	-3.26E-01	8.16E-03	-3.54E-01
12065	GSM656692	GSE26682	Colorectal Cancer	Colorectal	4.53E-16	-4.70E-01	1.33E-06	-5.94E-01
12066	GSM656693	GSE26682	Colorectal Cancer	Colorectal	2.21E-09	-3.53E-01	1.20E-03	-4.19E-01
12067	GSM656694	GSE26682	Colorectal Cancer	Colorectal	1.40E-29	-6.45E-01	2.21E-09	-7.21E-01
12068	GSM656695	GSE26682	Colorectal Cancer	Colorectal	1.77E-19	-5.20E-01	9.95E-07	-6.01E-01
12069	GSM656696	GSE26682	Colorectal Cancer	Colorectal	4.69E-20	-5.28E-01	9.39E-07	-6.02E-01
12070	GSM656698	GSE26682	Colorectal Cancer	Colorectal	1.58E-22	-5.61E-01	9.66E-07	-6.01E-01
12071	GSM656699	GSE26682	Colorectal Cancer	Colorectal	1.26E-09	-3.58E-01	5.64E-04	-4.42E-01
12072	GSM656700	GSE26682	Colorectal Cancer	Colorectal	2.99E-35	-7.05E-01	1.50E-09	-7.28E-01
12073	GSM656701	GSE26682	Colorectal Cancer	Colorectal	1.69E-24	-5.85E-01	2.29E-09	-7.21E-01
12074	GSM656702	GSE26682	Colorectal Cancer	Colorectal	6.13E-24	-5.78E-01	6.33E-08	-6.58E-01
12075	GSM656703	GSE26682	Colorectal Cancer	Colorectal	3.27E-18	-5.02E-01	1.88E-06	-5.87E-01
12076	GSM656704	GSE26682	Colorectal Cancer	Colorectal	1.47E-05	-2.64E-01	2.68E-02	-3.07E-01
12077	GSM656705	GSE26682	Colorectal Cancer	Colorectal	1.27E-08	-3.37E-01	9.17E-04	-4.27E-01
12078	GSM656706	GSE26682	Colorectal Cancer	Colorectal	3.15E-10	-3.70E-01	8.02E-03	-3.55E-01
12079	GSM656707	GSE26682	Colorectal Cancer	Colorectal	3.03E-39	-7.45E-01	2.29E-09	-7.21E-01
12080	GSM656710	GSE26682	Colorectal Cancer	Colorectal	4.53E-16	-4.70E-01	1.07E-05	-5.47E-01
12081	GSM656711	GSE26682	Colorectal Cancer	Colorectal	3.15E-10	-3.70E-01	4.07E-03	-3.79E-01
12082	GSM656714	GSE26682	Colorectal Cancer	Colorectal	6.55E-19	-5.12E-01	5.43E-06	-5.63E-01
12083	GSM656715	GSE26682	Colorectal Cancer	Colorectal	4.53E-16	-4.70E-01	2.72E-06	-5.78E-01
12084	GSM656720	GSE26682	Colorectal Cancer	Colorectal	2.19E-30	-6.54E-01	5.67E-11	-7.85E-01
12085	GSM656721	GSE26682	Colorectal Cancer	Colorectal	1.10E-21	-5.50E-01	6.54E-08	-6.57E-01
12086	GSM656726	GSE26682	Colorectal Cancer	Colorectal	8.10E-12	-4.00E-01	1.81E-04	-4.74E-01
12087	GSM656731	GSE26682	Colorectal Cancer	Colorectal	1.04E-11	-3.98E-01	4.07E-03	-3.79E-01
12088	GSM656732	GSE26682	Colorectal Cancer	Colorectal	4.52E-23	-5.67E-01	1.93E-06	-5.86E-01
12089	GSM656735	GSE26682	Colorectal Cancer	Colorectal	3.36E-16	-4.72E-01	2.72E-06	-5.78E-01
12090	GSM656736	GSE26682	Colorectal Cancer	Colorectal	4.56E-25	-5.92E-01	1.23E-08	-6.90E-01
12091	GSM656737	GSE26682	Colorectal Cancer	Colorectal	5.01E-02	-1.37E-01	3.16E-01	-1.73E-01
12092	GSM656739	GSE26682	Colorectal Cancer	Colorectal	1.27E-23	-5.74E-01	5.45E-09	-7.05E-01
12093	GSM656742	GSE26682	Colorectal Cancer	Colorectal	5.64E-10	-3.65E-01	4.00E-03	-3.80E-01
12094	GSM656749	GSE26682	Colorectal Cancer	Colorectal	4.10E-11	-3.87E-01	3.13E-03	-3.88E-01
12095	GSM656762	GSE26682	Colorectal Cancer	Colorectal	3.49E-04	-2.23E-01	1.38E-01	-2.27E-01
12096	GSM656768	GSE26682	Colorectal Cancer	Colorectal	3.15E-36	-7.15E-01	3.58E-11	-7.92E-01
12097	GSM656769	GSE26682	Colorectal Cancer	Colorectal	1.40E-24	-5.86E-01	2.09E-07	-6.34E-01
12098	GSM656770	GSE26682	Colorectal Cancer	Colorectal	6.35E-17	-4.83E-01	3.88E-05	-5.15E-01
12099	GSM656772	GSE26682	Colorectal Cancer	Colorectal	9.35E-46	-8.06E-01	3.77E-13	-8.64E-01
12100	GSM656775	GSE26682	Colorectal Cancer	Colorectal	1.19E-13	-4.32E-01	5.07E-03	-3.71E-01
12101	GSM656776	GSE26682	Colorectal Cancer	Colorectal	2.38E-18	-5.04E-01	6.79E-07	-6.09E-01
12102	GSM656777	GSE26682	Colorectal Cancer	Colorectal	1.46E-04	-2.35E-01	6.48E-02	-2.67E-01
12103	GSM656782	GSE26682	Colorectal Cancer	Colorectal	2.84E-20	-5.31E-01	1.33E-06	-5.94E-01
12104	GSM656783	GSE26682	Colorectal Cancer	Colorectal	1.73E-12	-4.12E-01	2.01E-03	-4.03E-01
12105	GSM656784	GSE26682	Colorectal Cancer	Colorectal	3.29E-47	-8.18E-01	2.40E-14	-9.05E-01
12106	GSM656785	GSE26682	Colorectal Cancer	Colorectal	1.37E-40	-7.58E-01	1.52E-10	-7.68E-01
12107	GSM656787	GSE26682	Colorectal Cancer	Colorectal	1.72E-42	-7.76E-01	2.97E-12	-8.32E-01
12108	GSM656794	GSE26682	Colorectal Cancer	Colorectal	7.74E-20	-5.25E-01	2.09E-07	-6.34E-01
12109	GSM656795	GSE26682	Colorectal Cancer	Colorectal	2.59E-45	-8.02E-01	6.23E-13	-8.57E-01
12110	GSM656798	GSE26682	Colorectal Cancer	Colorectal	2.97E-08	-3.29E-01	9.56E-04	-4.26E-01
12111	GSM656800	GSE26682	Colorectal Cancer	Colorectal	1.56E-41	-7.67E-01	4.63E-12	-8.25E-01
12112	GSM656802	GSE26682	Colorectal Cancer	Colorectal	9.14E-20	-5.24E-01	5.77E-05	-5.05E-01
12113	GSM656803	GSE26682	Colorectal Cancer	Colorectal	1.59E-36	-7.18E-01	1.46E-10	-7.69E-01
12114	GSM656804	GSE26682	Colorectal Cancer	Colorectal	1.05E-42	-7.78E-01	6.23E-13	-8.57E-01
12115	GSM656806	GSE26682	Colorectal Cancer	Colorectal	8.66E-21	-5.38E-01	1.58E-05	-5.37E-01
12116	GSM656809	GSE26682	Colorectal Cancer	Colorectal	4.37E-21	-5.42E-01	2.09E-07	-6.34E-01
12117	GSM656811	GSE26682	Colorectal Cancer	Colorectal	1.35E-17	-4.93E-01	5.49E-05	-5.06E-01
12118	GSM656816	GSE26682	Colorectal Cancer	Colorectal	3.39E-06	-2.81E-01	3.16E-02	-3.00E-01
12119	GSM656819	GSE26682	Colorectal Cancer	Colorectal	2.91E-12	-4.08E-01	1.25E-03	-4.18E-01
12120	GSM656820	GSE26682	Colorectal Cancer	Colorectal	1.53E-35	-7.08E-01	1.07E-12	-8.48E-01
12121	GSM656822	GSE26682	Colorectal Cancer	Colorectal	1.08E-04	-2.39E-01	2.61E-02	-3.09E-01
12122	GSM656824	GSE26682	Colorectal Cancer	Colorectal	4.60E-03	1.84E-01	4.89E-03	3.73E-01
12123	GSM656825	GSE26682	Colorectal Cancer	Colorectal	2.07E-02	1.56E-01	9.68E-03	3.48E-01
12124	GSM656827	GSE26682	Colorectal Cancer	Colorectal	3.53E-13	-4.24E-01	5.40E-04	-4.43E-01
12125	GSM656830	GSE26682	Colorectal Cancer	Colorectal	2.39E-43	-7.84E-01	6.23E-13	-8.57E-01
12126	GSM656831	GSE26682	Colorectal Cancer	Colorectal	1.39E-32	-6.78E-01	1.46E-10	-7.69E-01
12127	GSM656832	GSE26682	Colorectal Cancer	Colorectal	1.89E-22	-5.60E-01	3.11E-07	-6.25E-01
12128	GSM656833	GSE26682	Colorectal Cancer	Colorectal	6.10E-16	-4.68E-01	9.60E-05	-4.91E-01
12129	GSM656834	GSE26682	Colorectal Cancer	Colorectal	1.14E-29	-6.46E-01	1.50E-09	-7.28E-01
12130	GSM656836	GSE26682	Colorectal Cancer	Colorectal	9.55E-31	-6.58E-01	1.46E-10	-7.69E-01
12131	GSM656837	GSE26682	Colorectal Cancer	Colorectal	7.70E-19	-5.11E-01	2.79E-06	-5.78E-01

12132	GSM656838	GSE26682	Colorectal Cancer	Colorectal	1.58E-22	-5.61E-01	8.16E-06	-5.53E-01
12133	GSM656839	GSE26682	Colorectal Cancer	Colorectal	3.11E-27	-6.18E-01	2.82E-08	-6.74E-01
12134	GSM656840	GSE26682	Colorectal Cancer	Colorectal	1.72E-29	-6.44E-01	8.21E-09	-6.97E-01
12135	GSM656841	GSE26682	Colorectal Cancer	Colorectal	9.35E-46	-8.06E-01	1.25E-11	-8.10E-01
12136	GSM656842	GSE26682	Colorectal Cancer	Colorectal	2.19E-30	-6.54E-01	3.48E-09	-7.13E-01
12137	GSM656843	GSE26682	Colorectal Cancer	Colorectal	6.00E-14	-4.37E-01	1.20E-03	-4.19E-01
12138	GSM656844	GSE26682	Colorectal Cancer	Colorectal	1.89E-39	-7.47E-01	5.67E-11	-7.85E-01
12139	GSM656845	GSE26682	Colorectal Cancer	Colorectal	3.16E-29	-6.41E-01	1.45E-09	-7.29E-01
12140	GSM656846	GSE26682	Colorectal Cancer	Colorectal	2.19E-30	-6.54E-01	5.91E-10	-7.45E-01
12141	GSM656847	GSE26682	Colorectal Cancer	Colorectal	2.77E-02	-1.50E-01	1.01E-01	2.44E-01
12142	GSM656848	GSE26682	Colorectal Cancer	Colorectal	9.29E-30	-6.47E-01	2.37E-10	-7.61E-01
12143	GSM656849	GSE26682	Colorectal Cancer	Colorectal	7.97E-10	-3.62E-01	3.13E-03	-3.88E-01
12144	GSM656850	GSE26682	Colorectal Cancer	Colorectal	2.21E-10	-3.73E-01	1.77E-04	-4.75E-01
12145	GSM656851	GSE26682	Colorectal Cancer	Colorectal	1.65E-01	-1.06E-01	1.75E-01	-2.13E-01
12146	GSM656852	GSE26682	Colorectal Cancer	Colorectal	2.58E-29	-6.42E-01	2.37E-10	-7.61E-01
12147	GSM656853	GSE26682	Colorectal Cancer	Colorectal	7.72E-22	-5.52E-01	2.72E-06	-5.78E-01
12148	GSM656854	GSE26682	Colorectal Cancer	Colorectal	2.84E-20	-5.31E-01	1.44E-07	-6.41E-01
12149	GSM656855	GSE26682	Colorectal Cancer	Colorectal	1.18E-30	-6.57E-01	3.82E-10	-7.52E-01
12150	GSM656856	GSE26682	Colorectal Cancer	Colorectal	2.65E-15	-4.58E-01	1.85E-04	-4.74E-01
12151	GSM656857	GSE26682	Colorectal Cancer	Colorectal	4.86E-39	-7.43E-01	1.30E-11	-8.09E-01
12152	GSM656858	GSE26682	Colorectal Cancer	Colorectal	1.10E-15	-4.64E-01	9.83E-05	-4.91E-01
12153	GSM656859	GSE26682	Colorectal Cancer	Colorectal	1.17E-31	-6.68E-01	1.75E-12	-8.41E-01
12154	GSM656860	GSE26682	Colorectal Cancer	Colorectal	1.44E-03	-2.02E-01	2.72E-02	-3.07E-01
12155	GSM656861	GSE26682	Colorectal Cancer	Colorectal	8.66E-21	-5.38E-01	9.39E-07	-6.02E-01
12156	GSM656862	GSE26682	Colorectal Cancer	Colorectal	5.10E-31	-6.61E-01	5.91E-10	-7.45E-01
12157	GSM656863	GSE26682	Colorectal Cancer	Colorectal	1.77E-09	-3.55E-01	4.22E-03	-3.78E-01
12158	GSM656864	GSE26682	Colorectal Cancer	Colorectal	7.86E-13	-4.18E-01	2.38E-04	-4.67E-01
12159	GSM656865	GSE26682	Colorectal Cancer	Colorectal	2.00E-36	-7.17E-01	1.35E-11	-8.08E-01
12160	GSM656866	GSE26682	Colorectal Cancer	Colorectal	6.16E-30	-6.49E-01	2.21E-09	-7.21E-01
12161	GSM656867	GSE26682	Colorectal Cancer	Colorectal	6.39E-37	-7.22E-01	5.91E-10	-7.45E-01
12162	GSM656868	GSE26682	Colorectal Cancer	Colorectal	1.45E-30	-6.56E-01	5.45E-09	-7.05E-01
12163	GSM656869	GSE26682	Colorectal Cancer	Colorectal	5.54E-45	-7.99E-01	2.51E-14	-9.04E-01
12164	GSM656870	GSE26682	Colorectal Cancer	Colorectal	1.37E-40	-7.58E-01	7.47E-14	-8.88E-01
12165	GSM656871	GSE26682	Colorectal Cancer	Colorectal	6.97E-04	-2.13E-01	1.37E-01	-2.28E-01
12166	GSM656872	GSE26682	Colorectal Cancer	Colorectal	6.16E-21	-5.40E-01	9.83E-05	-4.91E-01
12167	GSM327282	GSE13067	Colorectal Cancer	Colorectal	4.56E-25	-5.92E-01	1.40E-07	-6.42E-01
12168	GSM327283	GSE13067	Colorectal Cancer	Colorectal	1.12E-32	-6.79E-01	1.52E-10	-7.68E-01
12169	GSM327284	GSE13067	Colorectal Cancer	Colorectal	4.54E-40	-7.53E-01	3.58E-11	-7.92E-01
12170	GSM327285	GSE13067	Colorectal Cancer	Colorectal	7.84E-28	-6.25E-01	5.91E-10	-7.45E-01
12171	GSM327286	GSE13067	Colorectal Cancer	Colorectal	1.16E-27	-6.23E-01	1.27E-08	-6.89E-01
12172	GSM327287	GSE13067	Colorectal Cancer	Colorectal	1.56E-45	-8.04E-01	7.47E-14	-8.88E-01
12173	GSM327288	GSE13067	Colorectal Cancer	Colorectal	2.48E-07	-3.09E-01	8.77E-02	-2.52E-01
12174	GSM327289	GSE13067	Colorectal Cancer	Colorectal	4.28E-12	-4.05E-01	9.76E-04	-4.25E-01
12175	GSM327290	GSE13067	Colorectal Cancer	Colorectal	5.68E-26	-6.03E-01	2.21E-09	-7.21E-01
12176	GSM327291	GSE13067	Colorectal Cancer	Colorectal	1.04E-11	-3.98E-01	9.83E-05	-4.91E-01
12177	GSM327292	GSE13067	Colorectal Cancer	Colorectal	6.48E-23	-5.65E-01	4.47E-07	-6.18E-01
12178	GSM327293	GSE13067	Colorectal Cancer	Colorectal	1.48E-14	-4.46E-01	3.98E-05	-5.14E-01
12179	GSM327294	GSE13067	Colorectal Cancer	Colorectal	2.20E-31	-6.65E-01	1.52E-10	-7.68E-01
12180	GSM327295	GSE13067	Colorectal Cancer	Colorectal	3.44E-34	-6.94E-01	1.30E-11	-8.09E-01
12181	GSM327296	GSE13067	Colorectal Cancer	Colorectal	6.43E-28	-6.26E-01	8.49E-09	-6.97E-01
12182	GSM327297	GSE13067	Colorectal Cancer	Colorectal	9.65E-06	-2.69E-01	1.22E-02	-3.39E-01
12183	GSM327298	GSE13067	Colorectal Cancer	Colorectal	9.46E-07	-2.95E-01	5.07E-03	-3.71E-01
12184	GSM327299	GSE13067	Colorectal Cancer	Colorectal	1.20E-46	-8.13E-01	7.92E-15	-9.21E-01
12185	GSM327300	GSE13067	Colorectal Cancer	Colorectal	6.13E-24	-5.78E-01	9.88E-08	-6.49E-01
12186	GSM327301	GSE13067	Colorectal Cancer	Colorectal	2.14E-16	-4.75E-01	1.99E-06	-5.85E-01
12187	GSM327302	GSE13067	Colorectal Cancer	Colorectal	7.72E-22	-5.52E-01	9.39E-07	-6.02E-01
12188	GSM327303	GSE13067	Colorectal Cancer	Colorectal	8.62E-17	-4.81E-01	1.10E-05	-5.46E-01
12189	GSM327304	GSE13067	Colorectal Cancer	Colorectal	1.27E-23	-5.74E-01	1.23E-08	-6.90E-01
12190	GSM327305	GSE13067	Colorectal Cancer	Colorectal	1.94E-28	-6.32E-01	8.21E-09	-6.97E-01
12191	GSM327306	GSE13067	Colorectal Cancer	Colorectal	3.11E-27	-6.18E-01	4.44E-08	-6.65E-01
12192	GSM327307	GSE13067	Colorectal Cancer	Colorectal	3.87E-26	-6.05E-01	2.21E-09	-7.21E-01
12193	GSM327308	GSE13067	Colorectal Cancer	Colorectal	1.18E-30	-6.57E-01	6.13E-10	-7.44E-01
12194	GSM327309	GSE13067	Colorectal Cancer	Colorectal	2.56E-27	-6.19E-01	1.96E-08	-6.81E-01
12195	GSM327310	GSE13067	Colorectal Cancer	Colorectal	5.01E-30	-6.50E-01	1.32E-08	-6.88E-01
12196	GSM327311	GSE13067	Colorectal Cancer	Colorectal	2.03E-24	-5.84E-01	4.30E-08	-6.65E-01
12197	GSM327312	GSE13067	Colorectal Cancer	Colorectal	8.44E-49	-8.32E-01	7.15E-14	-8.89E-01
12198	GSM327313	GSE13067	Colorectal Cancer	Colorectal	3.27E-18	-5.02E-01	1.37E-06	-5.94E-01
12199	GSM327314	GSE13067	Colorectal Cancer	Colorectal	1.79E-26	-6.09E-01	5.27E-09	-7.05E-01
12200	GSM327315	GSE13067	Colorectal Cancer	Colorectal	2.17E-08	-3.32E-01	4.98E-03	-3.72E-01
12201	GSM327316	GSE13067	Colorectal Cancer	Colorectal	2.99E-14	-4.41E-01	3.12E-04	-4.59E-01
12202	GSM327317	GSE13067	Colorectal Cancer	Colorectal	2.44E-04	-2.28E-01	1.18E-01	-2.36E-01
12203	GSM327318	GSE13067	Colorectal Cancer	Colorectal	8.84E-24	-5.76E-01	2.22E-07	-6.32E-01
12204	GSM327319	GSE13067	Colorectal Cancer	Colorectal	2.20E-31	-6.65E-01	2.21E-09	-7.21E-01
12205	GSM327320	GSE13067	Colorectal Cancer	Colorectal	2.16E-17	-4.90E-01	7.19E-05	-4.99E-01

12206	GSM327321	GSE13067	Colorectal Cancer	Colorectal	6.16E-18	-4.98E-01	1.07E-05	-5.47E-01
12207	GSM327322	GSE13067	Colorectal Cancer	Colorectal	1.12E-09	-3.59E-01	3.19E-04	-4.58E-01
12208	GSM327323	GSE13067	Colorectal Cancer	Colorectal	1.77E-34	-6.97E-01	5.01E-12	-8.24E-01
12209	GSM327324	GSE13067	Colorectal Cancer	Colorectal	4.55E-14	-4.38E-01	9.83E-05	-4.91E-01
12210	GSM327325	GSE13067	Colorectal Cancer	Colorectal	5.23E-11	-3.85E-01	9.56E-04	-4.26E-01
12211	GSM327326	GSE13067	Colorectal Cancer	Colorectal	3.15E-23	-5.69E-01	1.40E-07	-6.42E-01
12212	GSM327327	GSE13067	Colorectal Cancer	Colorectal	2.21E-11	-3.92E-01	7.36E-05	-4.98E-01
12213	GSM327328	GSE13067	Colorectal Cancer	Colorectal	1.12E-09	-3.59E-01	5.52E-04	-4.43E-01
12214	GSM327329	GSE13067	Colorectal Cancer	Colorectal	3.09E-51	-8.53E-01	8.28E-15	-9.20E-01
12215	GSM327330	GSE13067	Colorectal Cancer	Colorectal	6.89E-14	-4.36E-01	1.01E-04	-4.90E-01
12216	GSM327331	GSE13067	Colorectal Cancer	Colorectal	3.76E-12	-4.06E-01	1.03E-04	-4.90E-01
12217	GSM327332	GSE13067	Colorectal Cancer	Colorectal	1.30E-28	-6.34E-01	6.33E-08	-6.58E-01
12218	GSM327333	GSE13067	Colorectal Cancer	Colorectal	7.45E-04	-2.12E-01	1.19E-01	-2.36E-01
12219	GSM327334	GSE13067	Colorectal Cancer	Colorectal	1.18E-30	-6.57E-01	3.60E-09	-7.12E-01
12220	GSM327335	GSE13067	Colorectal Cancer	Colorectal	4.28E-34	-6.93E-01	1.52E-10	-7.68E-01
12221	GSM327336	GSE13067	Colorectal Cancer	Colorectal	1.89E-22	-5.60E-01	1.44E-07	-6.41E-01
12222	GSM327337	GSE13067	Colorectal Cancer	Colorectal	7.65E-32	-6.70E-01	3.68E-10	-7.53E-01
12223	GSM327338	GSE13067	Colorectal Cancer	Colorectal	2.10E-27	-6.20E-01	3.48E-09	-7.13E-01
12224	GSM327339	GSE13067	Colorectal Cancer	Colorectal	1.37E-40	7.58E-01	2.56E-15	9.37E-01
12225	GSM327340	GSE13067	Colorectal Cancer	Colorectal	2.56E-27	-6.19E-01	3.01E-08	-6.72E-01
12226	GSM327341	GSE13067	Colorectal Cancer	Colorectal	2.29E-15	-4.59E-01	2.87E-06	-5.77E-01
12227	GSM327342	GSE13067	Colorectal Cancer	Colorectal	2.00E-36	-7.17E-01	1.46E-10	-7.69E-01
12228	GSM327343	GSE13067	Colorectal Cancer	Colorectal	1.03E-20	-5.37E-01	1.49E-07	-6.41E-01
12229	GSM327344	GSE13067	Colorectal Cancer	Colorectal	2.49E-10	-3.72E-01	4.16E-04	-4.51E-01
12230	GSM327345	GSE13067	Colorectal Cancer	Colorectal	1.11E-03	-2.06E-01	2.64E-02	-3.08E-01
12231	GSM327346	GSE13067	Colorectal Cancer	Colorectal	2.00E-36	-7.17E-01	8.09E-12	-8.17E-01
12232	GSM327347	GSE13067	Colorectal Cancer	Colorectal	1.58E-22	-5.61E-01	1.40E-09	-7.30E-01
12233	GSM327348	GSE13067	Colorectal Cancer	Colorectal	2.37E-06	-2.85E-01	5.07E-03	-3.71E-01
12234	GSM327349	GSE13067	Colorectal Cancer	Colorectal	7.50E-42	-7.70E-01	8.09E-12	-8.17E-01
12235	GSM327350	GSE13067	Colorectal Cancer	Colorectal	7.30E-35	-7.01E-01	1.46E-10	-7.69E-01
12236	GSM327351	GSE13067	Colorectal Cancer	Colorectal	1.48E-14	4.46E-01	9.57E-08	6.50E-01
12237	GSM327352	GSE13067	Colorectal Cancer	Colorectal	2.40E-20	-5.32E-01	2.22E-05	-5.29E-01
12238	GSM327353	GSE13067	Colorectal Cancer	Colorectal	1.37E-13	-4.31E-01	9.17E-04	-4.27E-01
12239	GSM327354	GSE13067	Colorectal Cancer	Colorectal	2.46E-19	-5.18E-01	1.07E-05	-5.47E-01
12240	GSM327355	GSE13067	Colorectal Cancer	Colorectal	6.16E-18	-4.98E-01	9.39E-07	-6.02E-01
12241	GSM335510	GSE13294	Colorectal Cancer	Colorectal	3.43E-17	-4.87E-01	1.33E-06	-5.94E-01
12242	GSM335511	GSE13294	Colorectal Cancer	Colorectal	9.01E-33	-6.80E-01	6.13E-10	-7.44E-01
12243	GSM335512	GSE13294	Colorectal Cancer	Colorectal	8.24E-02	-1.25E-01	3.49E-01	1.66E-01
12244	GSM335513	GSE13294	Colorectal Cancer	Colorectal	2.10E-29	-6.43E-01	5.91E-10	-7.45E-01
12245	GSM335514	GSE13294	Colorectal Cancer	Colorectal	7.84E-07	-2.97E-01	5.38E-02	-2.76E-01
12246	GSM335515	GSE13294	Colorectal Cancer	Colorectal	1.37E-07	-3.14E-01	3.25E-03	-3.87E-01
12247	GSM335516	GSE13294	Colorectal Cancer	Colorectal	2.06E-13	-4.28E-01	1.62E-03	-4.10E-01
12248	GSM335517	GSE13294	Colorectal Cancer	Colorectal	8.01E-25	-5.89E-01	1.33E-06	-5.94E-01
12249	GSM335518	GSE13294	Colorectal Cancer	Colorectal	6.89E-13	-4.19E-01	1.54E-05	-5.38E-01
12250	GSM335519	GSE13294	Colorectal Cancer	Colorectal	4.81E-09	-3.46E-01	3.38E-03	-3.85E-01
12251	GSM335520	GSE13294	Colorectal Cancer	Colorectal	3.76E-12	-4.06E-01	3.26E-04	-4.58E-01
12252	GSM335521	GSE13294	Colorectal Cancer	Colorectal	5.28E-13	-4.21E-01	3.12E-04	-4.59E-01
12253	GSM335522	GSE13294	Colorectal Cancer	Colorectal	2.60E-25	-5.95E-01	3.02E-07	-6.26E-01
12254	GSM335523	GSE13294	Colorectal Cancer	Colorectal	2.44E-24	-5.83E-01	2.82E-08	-6.74E-01
12255	GSM335524	GSE13294	Colorectal Cancer	Colorectal	1.02E-07	-3.17E-01	3.25E-03	-3.87E-01
12256	GSM335525	GSE13294	Colorectal Cancer	Colorectal	8.62E-17	-4.81E-01	3.01E-05	-5.21E-01
12257	GSM335526	GSE13294	Colorectal Cancer	Colorectal	3.83E-18	-5.01E-01	6.40E-07	-6.10E-01
12258	GSM335527	GSE13294	Colorectal Cancer	Colorectal	1.75E-08	-3.34E-01	3.13E-03	-3.88E-01
12259	GSM335528	GSE13294	Colorectal Cancer	Colorectal	8.01E-25	-5.89E-01	2.22E-07	-6.32E-01
12260	GSM335529	GSE13294	Colorectal Cancer	Colorectal	1.10E-21	-5.50E-01	2.72E-06	-5.78E-01
12261	GSM335530	GSE13294	Colorectal Cancer	Colorectal	1.06E-23	-5.75E-01	5.27E-09	-7.05E-01
12262	GSM335531	GSE13294	Colorectal Cancer	Colorectal	7.74E-20	-5.25E-01	9.66E-07	-6.01E-01
12263	GSM335532	GSE13294	Colorectal Cancer	Colorectal	1.95E-11	-3.93E-01	4.08E-05	-5.14E-01
12264	GSM335533	GSE13294	Colorectal Cancer	Colorectal	1.72E-11	-3.94E-01	7.28E-04	-4.34E-01
12265	GSM335534	GSE13294	Colorectal Cancer	Colorectal	4.67E-17	-4.85E-01	2.79E-06	-5.78E-01
12266	GSM335535	GSE13294	Colorectal Cancer	Colorectal	5.37E-07	-3.01E-01	6.62E-03	-3.62E-01
12267	GSM335536	GSE13294	Colorectal Cancer	Colorectal	1.17E-12	-4.15E-01	3.88E-05	-5.15E-01
12268	GSM335537	GSE13294	Colorectal Cancer	Colorectal	3.19E-26	-6.06E-01	6.33E-08	-6.58E-01
12269	GSM335538	GSE13294	Colorectal Cancer	Colorectal	1.00E-01	-1.20E-01	3.30E-01	-1.70E-01
12270	GSM335539	GSE13294	Colorectal Cancer	Colorectal	8.30E-08	-3.19E-01	1.46E-02	-3.32E-01
12271	GSM335540	GSE13294	Colorectal Cancer	Colorectal	8.01E-25	-5.89E-01	6.33E-08	-6.58E-01
12272	GSM335541	GSE13294	Colorectal Cancer	Colorectal	1.48E-15	-4.62E-01	5.49E-05	-5.06E-01
12273	GSM335542	GSE13294	Colorectal Cancer	Colorectal	3.44E-14	-4.40E-01	5.63E-05	-5.05E-01
12274	GSM335543	GSE13294	Colorectal Cancer	Colorectal	4.49E-08	-3.25E-01	5.25E-03	-3.70E-01
12275	GSM335544	GSE13294	Colorectal Cancer	Colorectal	3.36E-16	-4.72E-01	1.33E-06	-5.94E-01
12276	GSM335545	GSE13294	Colorectal Cancer	Colorectal	9.63E-02	-1.21E-01	2.83E-01	-1.82E-01
12277	GSM335546	GSE13294	Colorectal Cancer	Colorectal	2.50E-11	-3.91E-01	1.01E-04	-4.90E-01
12278	GSM335547	GSE13294	Colorectal Cancer	Colorectal	2.25E-22	-5.59E-01	2.79E-06	-5.78E-01
12279	GSM335548	GSE13294	Colorectal Cancer	Colorectal	7.70E-19	-5.11E-01	7.74E-06	-5.54E-01

12280	GSM335549	GSE13294	Colorectal Cancer	Colorectal	4.32E-28	-6.28E-01	3.11E-07	-6.25E-01
12281	GSM335550	GSE13294	Colorectal Cancer	Colorectal	3.24E-44	-7.92E-01	3.58E-11	-7.92E-01
12282	GSM335551	GSE13294	Colorectal Cancer	Colorectal	2.37E-06	-2.85E-01	2.27E-02	-3.14E-01
12283	GSM335552	GSE13294	Colorectal Cancer	Colorectal	7.30E-02	-1.28E-01	1.18E-01	2.36E-01
12284	GSM335553	GSE13294	Colorectal Cancer	Colorectal	2.10E-29	-6.43E-01	9.45E-10	-7.37E-01
12285	GSM335554	GSE13294	Colorectal Cancer	Colorectal	8.84E-24	-5.76E-01	3.02E-07	-6.26E-01
12286	GSM335555	GSE13294	Colorectal Cancer	Colorectal	2.64E-32	-6.75E-01	5.91E-10	-7.45E-01
12287	GSM335556	GSE13294	Colorectal Cancer	Colorectal	2.03E-24	-5.84E-01	1.23E-08	-6.90E-01
12288	GSM335557	GSE13294	Colorectal Cancer	Colorectal	3.97E-20	-5.29E-01	3.02E-07	-6.26E-01
12289	GSM335558	GSE13294	Colorectal Cancer	Colorectal	7.50E-42	-7.70E-01	5.01E-12	-8.24E-01
12290	GSM335559	GSE13294	Colorectal Cancer	Colorectal	1.01E-25	-6.00E-01	3.02E-07	-6.26E-01
12291	GSM335560	GSE13294	Colorectal Cancer	Colorectal	5.81E-29	-6.38E-01	1.46E-10	-7.69E-01
12292	GSM335561	GSE13294	Colorectal Cancer	Colorectal	2.51E-38	-7.36E-01	2.16E-11	-8.01E-01
12293	GSM335562	GSE13294	Colorectal Cancer	Colorectal	2.90E-28	-6.30E-01	2.82E-08	-6.74E-01
12294	GSM335563	GSE13294	Colorectal Cancer	Colorectal	1.96E-04	-2.31E-01	1.83E-02	-3.23E-01
12295	GSM335564	GSE13294	Colorectal Cancer	Colorectal	5.27E-06	-2.76E-01	3.30E-02	-2.98E-01
12296	GSM335565	GSE13294	Colorectal Cancer	Colorectal	4.01E-17	-4.86E-01	5.74E-06	-5.61E-01
12297	GSM335566	GSE13294	Colorectal Cancer	Colorectal	2.64E-32	-6.75E-01	9.11E-10	-7.37E-01
12298	GSM335567	GSE13294	Colorectal Cancer	Colorectal	5.31E-48	-8.25E-01	7.15E-14	-8.89E-01
12299	GSM335568	GSE13294	Colorectal Cancer	Colorectal	1.57E-13	-4.30E-01	2.49E-04	-4.65E-01
12300	GSM335569	GSE13294	Colorectal Cancer	Colorectal	6.18E-32	-6.71E-01	9.45E-10	-7.37E-01
12301	GSM335570	GSE13294	Colorectal Cancer	Colorectal	8.70E-29	-6.36E-01	9.45E-10	-7.37E-01
12302	GSM335571	GSE13294	Colorectal Cancer	Colorectal	5.00E-32	-6.72E-01	5.91E-10	-7.45E-01
12303	GSM335572	GSE13294	Colorectal Cancer	Colorectal	4.16E-44	-7.91E-01	1.07E-12	-8.48E-01
12304	GSM335573	GSE13294	Colorectal Cancer	Colorectal	1.42E-27	-6.22E-01	1.90E-08	-6.81E-01
12305	GSM335574	GSE13294	Colorectal Cancer	Colorectal	1.79E-02	1.59E-01	3.81E-02	2.92E-01
12306	GSM335575	GSE13294	Colorectal Cancer	Colorectal	8.30E-08	-3.19E-01	1.54E-02	-3.30E-01
12307	GSM335576	GSE13294	Colorectal Cancer	Colorectal	3.44E-14	-4.40E-01	5.36E-05	-5.07E-01
12308	GSM335577	GSE13294	Colorectal Cancer	Colorectal	4.72E-15	-4.54E-01	7.36E-05	-4.98E-01
12309	GSM335578	GSE13294	Colorectal Cancer	Colorectal	9.26E-23	-5.63E-01	2.15E-07	-6.33E-01
12310	GSM335579	GSE13294	Colorectal Cancer	Colorectal	5.02E-43	-7.81E-01	5.01E-12	-8.24E-01
12311	GSM335580	GSE13294	Colorectal Cancer	Colorectal	1.98E-06	-2.87E-01	4.76E-02	-2.82E-01
12312	GSM335581	GSE13294	Colorectal Cancer	Colorectal	3.16E-29	-6.41E-01	2.15E-07	-6.33E-01
12313	GSM335582	GSE13294	Colorectal Cancer	Colorectal	4.14E-31	-6.62E-01	9.11E-10	-7.37E-01
12314	GSM335583	GSE13294	Colorectal Cancer	Colorectal	1.06E-23	-5.75E-01	6.99E-07	-6.08E-01
12315	GSM335584	GSE13294	Colorectal Cancer	Colorectal	4.60E-42	-7.72E-01	1.07E-12	-8.48E-01
12316	GSM335585	GSE13294	Colorectal Cancer	Colorectal	5.10E-31	-6.61E-01	5.67E-11	-7.85E-01
12317	GSM335586	GSE13294	Colorectal Cancer	Colorectal	5.23E-41	-7.62E-01	1.52E-10	-7.68E-01
12318	GSM335587	GSE13294	Colorectal Cancer	Colorectal	1.06E-23	-5.75E-01	1.90E-08	-6.81E-01
12319	GSM335588	GSE13294	Colorectal Cancer	Colorectal	5.02E-10	-3.66E-01	1.17E-03	-4.20E-01
12320	GSM335589	GSE13294	Colorectal Cancer	Colorectal	9.55E-31	-6.58E-01	9.11E-10	-7.37E-01
12321	GSM335590	GSE13294	Colorectal Cancer	Colorectal	1.47E-18	-5.07E-01	1.45E-06	-5.92E-01
12322	GSM335591	GSE13294	Colorectal Cancer	Colorectal	4.25E-24	-5.80E-01	6.40E-07	-6.10E-01
12323	GSM335592	GSE13294	Colorectal Cancer	Colorectal	1.83E-23	-5.72E-01	4.47E-07	-6.18E-01
12324	GSM335593	GSE13294	Colorectal Cancer	Colorectal	3.87E-29	-6.40E-01	9.57E-08	-6.50E-01
12325	GSM335594	GSE13294	Colorectal Cancer	Colorectal	1.78E-30	-6.55E-01	3.01E-08	-6.72E-01
12326	GSM335595	GSE13294	Colorectal Cancer	Colorectal	3.87E-26	-6.05E-01	1.90E-08	-6.81E-01
12327	GSM335596	GSE13294	Colorectal Cancer	Colorectal	7.57E-30	-6.48E-01	2.82E-08	-6.74E-01
12328	GSM335597	GSE13294	Colorectal Cancer	Colorectal	2.21E-34	-6.96E-01	3.68E-10	-7.53E-01
12329	GSM335598	GSE13294	Colorectal Cancer	Colorectal	1.16E-17	-4.94E-01	4.01E-06	-5.70E-01
12330	GSM335599	GSE13294	Colorectal Cancer	Colorectal	1.42E-34	-6.98E-01	3.58E-11	-7.92E-01
12331	GSM335600	GSE13294	Colorectal Cancer	Colorectal	1.79E-26	-6.09E-01	1.44E-07	-6.41E-01
12332	GSM335601	GSE13294	Colorectal Cancer	Colorectal	7.79E-39	-7.41E-01	3.58E-11	-7.92E-01
12333	GSM335602	GSE13294	Colorectal Cancer	Colorectal	8.03E-38	-7.31E-01	8.09E-12	-8.17E-01
12334	GSM335603	GSE13294	Colorectal Cancer	Colorectal	4.56E-25	-5.92E-01	5.27E-09	-7.05E-01
12335	GSM335604	GSE13294	Colorectal Cancer	Colorectal	9.08E-14	-4.34E-01	1.31E-04	-4.83E-01
12336	GSM335605	GSE13294	Colorectal Cancer	Colorectal	3.26E-32	-6.74E-01	9.45E-10	-7.37E-01
12337	GSM335606	GSE13294	Colorectal Cancer	Colorectal	1.75E-10	-3.75E-01	5.88E-04	-4.41E-01
12338	GSM335607	GSE13294	Colorectal Cancer	Colorectal	1.57E-38	-7.38E-01	8.09E-12	-8.17E-01
12339	GSM335608	GSE13294	Colorectal Cancer	Colorectal	1.55E-10	-3.76E-01	2.49E-03	-3.96E-01
12340	GSM335609	GSE13294	Colorectal Cancer	Colorectal	4.68E-35	-7.03E-01	5.91E-10	-7.45E-01
12341	GSM335610	GSE13294	Colorectal Cancer	Colorectal	1.04E-13	-4.33E-01	2.11E-05	-5.30E-01
12342	GSM335611	GSE13294	Colorectal Cancer	Colorectal	7.96E-52	-8.58E-01	1.31E-13	-8.80E-01
12343	GSM335612	GSE13294	Colorectal Cancer	Colorectal	2.56E-12	-4.09E-01	4.54E-04	-4.48E-01
12344	GSM335613	GSE13294	Colorectal Cancer	Colorectal	6.48E-23	-5.65E-01	2.79E-06	-5.78E-01
12345	GSM335614	GSE13294	Colorectal Cancer	Colorectal	1.72E-11	-3.94E-01	5.40E-04	-4.43E-01
12346	GSM335615	GSE13294	Colorectal Cancer	Colorectal	5.81E-29	-6.38E-01	3.68E-10	-7.53E-01
12347	GSM335616	GSE13294	Colorectal Cancer	Colorectal	2.76E-34	-6.95E-01	2.37E-10	-7.61E-01
12348	GSM335617	GSE13294	Colorectal Cancer	Colorectal	2.96E-01	8.73E-02	7.55E-02	2.60E-01
12349	GSM335618	GSE13294	Colorectal Cancer	Colorectal	1.40E-29	-6.45E-01	3.48E-09	-7.13E-01
12350	GSM335619	GSE13294	Colorectal Cancer	Colorectal	8.44E-18	-4.96E-01	3.21E-07	-6.25E-01
12351	GSM335620	GSE13294	Colorectal Cancer	Colorectal	4.63E-11	-3.86E-01	7.36E-05	-4.98E-01
12352	GSM335621	GSE13294	Colorectal Cancer	Colorectal	5.51E-25	-5.91E-01	8.21E-09	-6.97E-01
12353	GSM335622	GSE13294	Colorectal Cancer	Colorectal	1.27E-19	-5.22E-01	4.61E-07	-6.17E-01

12354	GSM335623	GSE13294	Colorectal Cancer	Colorectal	4.72E-15	-4.54E-01	7.36E-05	-4.98E-01
12355	GSM335624	GSE13294	Colorectal Cancer	Colorectal	6.37E-38	-7.32E-01	2.16E-11	-8.01E-01
12356	GSM335625	GSE13294	Colorectal Cancer	Colorectal	2.10E-29	-6.43E-01	2.37E-10	-7.61E-01
12357	GSM335626	GSE13294	Colorectal Cancer	Colorectal	3.17E-38	-7.35E-01	9.30E-11	-7.77E-01
12358	GSM335627	GSE13294	Colorectal Cancer	Colorectal	8.27E-34	-6.90E-01	1.35E-11	-8.08E-01
12359	GSM335628	GSE13294	Colorectal Cancer	Colorectal	1.94E-28	-6.32E-01	3.68E-10	-7.53E-01
12360	GSM335629	GSE13294	Colorectal Cancer	Colorectal	7.97E-04	-2.11E-01	7.55E-02	-2.60E-01
12361	GSM335630	GSE13294	Colorectal Cancer	Colorectal	7.21E-18	-4.97E-01	3.11E-07	-6.25E-01
12362	GSM335631	GSE13294	Colorectal Cancer	Colorectal	3.32E-30	-6.52E-01	9.11E-10	-7.37E-01
12363	GSM335632	GSE13294	Colorectal Cancer	Colorectal	1.16E-27	-6.23E-01	1.23E-08	-6.90E-01
12364	GSM335633	GSE13294	Colorectal Cancer	Colorectal	6.03E-13	-4.20E-01	3.88E-05	-5.15E-01
12365	GSM335634	GSE13294	Colorectal Cancer	Colorectal	1.25E-18	-5.08E-01	4.47E-07	-6.18E-01
12366	GSM335635	GSE13294	Colorectal Cancer	Colorectal	9.88E-18	-4.95E-01	4.08E-05	-5.14E-01
12367	GSM335636	GSE13294	Colorectal Cancer	Colorectal	7.74E-20	-5.25E-01	8.16E-06	-5.53E-01
12368	GSM335637	GSE13294	Colorectal Cancer	Colorectal	1.63E-03	2.00E-01	6.28E-03	3.64E-01
12369	GSM335638	GSE13294	Colorectal Cancer	Colorectal	3.32E-30	-6.52E-01	1.46E-10	-7.69E-01
12370	GSM335639	GSE13294	Colorectal Cancer	Colorectal	1.77E-34	-6.97E-01	5.67E-11	-7.85E-01
12371	GSM335640	GSE13294	Colorectal Cancer	Colorectal	5.98E-09	-3.44E-01	6.51E-03	-3.63E-01
12372	GSM335641	GSE13294	Colorectal Cancer	Colorectal	2.84E-20	-5.31E-01	2.09E-07	-6.34E-01
12373	GSM335642	GSE13294	Colorectal Cancer	Colorectal	1.22E-20	-5.36E-01	6.33E-08	-6.58E-01
12374	GSM335643	GSE13294	Colorectal Cancer	Colorectal	4.60E-42	-7.72E-01	1.75E-12	-8.41E-01
12375	GSM335644	GSE13294	Colorectal Cancer	Colorectal	4.62E-13	-4.22E-01	2.44E-04	-4.66E-01
12376	GSM335645	GSE13294	Colorectal Cancer	Colorectal	1.98E-09	-3.54E-01	1.07E-05	-5.47E-01
12377	GSM335646	GSE13294	Colorectal Cancer	Colorectal	1.49E-39	-7.48E-01	2.16E-11	-8.01E-01
12378	GSM335647	GSE13294	Colorectal Cancer	Colorectal	2.15E-25	-5.96E-01	9.11E-10	-7.37E-01
12379	GSM335648	GSE13294	Colorectal Cancer	Colorectal	4.54E-40	-7.53E-01	5.67E-11	-7.85E-01
12380	GSM335649	GSE13294	Colorectal Cancer	Colorectal	1.98E-06	-2.87E-01	2.64E-02	-3.08E-01
12381	GSM335650	GSE13294	Colorectal Cancer	Colorectal	4.52E-23	-5.67E-01	3.48E-09	-7.13E-01
12382	GSM335651	GSE13294	Colorectal Cancer	Colorectal	3.06E-33	-6.85E-01	9.30E-11	-7.77E-01
12383	GSM335652	GSE13294	Colorectal Cancer	Colorectal	1.75E-08	-3.34E-01	8.16E-03	-3.54E-01
12384	GSM335653	GSE13294	Colorectal Cancer	Colorectal	1.50E-06	-2.90E-01	1.17E-03	-4.20E-01
12385	GSM335654	GSE13294	Colorectal Cancer	Colorectal	4.39E-02	-1.40E-01	2.66E-01	-1.86E-01
12386	GSM335655	GSE13294	Colorectal Cancer	Colorectal	3.27E-18	-5.02E-01	5.58E-06	-5.62E-01
12387	GSM335656	GSE13294	Colorectal Cancer	Colorectal	5.23E-14	-4.38E-01	9.36E-04	-4.27E-01
12388	GSM335657	GSE13294	Colorectal Cancer	Colorectal	2.02E-18	-5.05E-01	3.80E-06	-5.71E-01
12389	GSM335658	GSE13294	Colorectal Cancer	Colorectal	4.95E-04	-2.18E-01	3.97E-02	-2.90E-01
12390	GSM335659	GSE13294	Colorectal Cancer	Colorectal	3.98E-10	-3.68E-01	9.83E-05	-4.91E-01
12391	GSM335660	GSE13294	Colorectal Cancer	Colorectal	5.36E-09	-3.45E-01	4.98E-03	-3.72E-01
12392	GSM335661	GSE13294	Colorectal Cancer	Colorectal	1.73E-27	-6.21E-01	2.82E-08	-6.74E-01
12393	GSM335662	GSE13294	Colorectal Cancer	Colorectal	2.70E-13	-4.26E-01	4.25E-04	-4.50E-01
12394	GSM335663	GSE13294	Colorectal Cancer	Colorectal	1.73E-27	-6.21E-01	5.91E-10	-7.45E-01
12395	GSM335664	GSE13294	Colorectal Cancer	Colorectal	3.56E-01	-8.04E-02	8.66E-02	2.53E-01
12396	GSM400174	GSE15960	Colorectal Cancer	Colorectal.Ader	4.01E-17	-4.86E-01	8.16E-06	-5.53E-01
12397	GSM400175	GSE15960	Colorectal Cancer	Colorectal.Ader	8.94E-10	-3.61E-01	4.16E-04	-4.51E-01
12398	GSM400176	GSE15960	Colorectal Cancer	Colorectal.Ader	6.28E-12	-4.02E-01	7.13E-04	-4.35E-01
12399	GSM400177	GSE15960	Colorectal Cancer	Colorectal.Ader	1.35E-17	-4.93E-01	2.79E-06	-5.78E-01
12400	GSM400178	GSE15960	Colorectal Cancer	Colorectal.Ader	7.75E-31	-6.59E-01	2.37E-10	-7.61E-01
12401	GSM400179	GSE15960	Colorectal Cancer	Colorectal.Ader	1.50E-19	-5.21E-01	2.11E-05	-5.30E-01
12402	GSM523244	GSE20916	Colorectal Cancer	Colorectal.Ader	2.90E-28	-6.30E-01	6.33E-08	-6.58E-01
12403	GSM523247	GSE20916	Colorectal Cancer	Colorectal.Ader	1.58E-22	-5.61E-01	9.39E-07	-6.02E-01
12404	GSM523251	GSE20916	Colorectal Cancer	Colorectal.Ader	1.84E-21	-5.47E-01	9.37E-05	-4.92E-01
12405	GSM523252	GSE20916	Colorectal Cancer	Colorectal.Ader	4.56E-25	-5.92E-01	4.61E-07	-6.17E-01
12406	GSM523254	GSE20916	Colorectal Cancer	Colorectal.Ader	3.58E-40	-7.54E-01	6.23E-13	-8.57E-01
12407	GSM523256	GSE20916	Colorectal Cancer	Colorectal.Ader	6.66E-41	-7.61E-01	8.09E-12	-8.17E-01
12408	GSM523259	GSE20916	Colorectal Cancer	Colorectal.Ader	7.50E-42	-7.70E-01	2.18E-13	-8.72E-01
12409	GSM523261	GSE20916	Colorectal Cancer	Colorectal.Ader	2.20E-42	-7.75E-01	3.77E-13	-8.64E-01
12410	GSM523277	GSE20916	Colorectal Cancer	Colorectal.Ader	3.60E-42	-7.73E-01	6.23E-13	-8.57E-01
12411	GSM523281	GSE20916	Colorectal Cancer	Colorectal.Ader	2.17E-26	-6.08E-01	4.47E-07	-6.18E-01
12412	GSM786491	GSE31684	Bladder	Bladder	7.84E-07	2.97E-01	1.92E-06	5.89E-01
12413	GSM786492	GSE31684	Bladder	Bladder	4.37E-21	5.42E-01	5.72E-13	8.62E-01
12414	GSM786493	GSE31684	Bladder	Bladder	9.59E-11	-3.80E-01	1.00E-02	-3.48E-01
12415	GSM786494	GSE31684	Bladder	Bladder	3.62E-11	3.88E-01	5.11E-07	6.18E-01
12416	GSM786495	GSE31684	Bladder	Bladder	3.79E-27	-6.17E-01	7.15E-07	-6.10E-01
12417	GSM786496	GSE31684	Bladder	Bladder	9.06E-19	-5.10E-01	4.66E-07	-6.20E-01
12418	GSM786497	GSE31684	Bladder	Bladder	1.25E-02	1.66E-01	7.05E-02	2.64E-01
12419	GSM786498	GSE31684	Bladder	Bladder	3.45E-09	-3.49E-01	6.25E-05	-5.05E-01
12420	GSM786499	GSE31684	Bladder	Bladder	1.71E-14	-4.45E-01	5.15E-04	-4.47E-01
12421	GSM786500	GSE31684	Bladder	Bladder	1.44E-01	1.10E-01	2.10E-01	2.03E-01
12422	GSM786501	GSE31684	Bladder	Bladder	1.44E-20	-5.35E-01	1.51E-06	-5.94E-01
12423	GSM786502	GSE31684	Bladder	Bladder	5.46E-15	-4.53E-01	3.13E-04	-4.61E-01
12424	GSM786503	GSE31684	Bladder	Bladder	2.84E-05	-2.56E-01	1.62E-01	-2.19E-01
12425	GSM786504	GSE31684	Bladder	Bladder	1.31E-02	1.65E-01	3.94E-02	2.92E-01
12426	GSM786505	GSE31684	Bladder	Bladder	2.91E-02	1.49E-01	7.83E-03	3.57E-01
12427	GSM786506	GSE31684	Bladder	Bladder	3.32E-07	-3.06E-01	2.11E-04	-4.72E-01

12428	GSM786507	GSE31684	Bladder	Bladder	5.46E-15	-4.53E-01	2.11E-04	-4.72E-01
12429	GSM786508	GSE31684	Bladder	Bladder	1.38E-02	-1.64E-01	2.10E-01	-2.03E-01
12430	GSM786509	GSE31684	Bladder	Bladder	5.47E-03	1.81E-01	1.53E-02	3.32E-01
12431	GSM786510	GSE31684	Bladder	Bladder	8.84E-24	-5.76E-01	1.17E-05	-5.47E-01
12432	GSM786511	GSE31684	Bladder	Bladder	7.44E-09	3.42E-01	4.38E-05	5.14E-01
12433	GSM786512	GSE31684	Bladder	Bladder	9.54E-03	-1.71E-01	2.71E-02	-3.08E-01
12434	GSM786513	GSE31684	Bladder	Bladder	2.96E-01	8.73E-02	1.78E-01	2.13E-01
12435	GSM786514	GSE31684	Bladder	Bladder	4.43E-01	-7.14E-02	3.81E-01	-1.59E-01
12436	GSM786515	GSE31684	Bladder	Bladder	2.37E-06	-2.85E-01	7.43E-03	-3.59E-01
12437	GSM786516	GSE31684	Bladder	Bladder	1.04E-06	2.94E-01	1.88E-03	4.07E-01
12438	GSM786517	GSE31684	Bladder	Bladder	2.19E-30	-6.54E-01	1.28E-09	-7.34E-01
12439	GSM786518	GSE31684	Bladder	Bladder	1.81E-06	2.88E-01	1.16E-04	4.89E-01
12440	GSM786519	GSE31684	Bladder	Bladder	1.00E-01	1.20E-01	2.02E-02	3.21E-01
12441	GSM786520	GSE31684	Bladder	Bladder	5.83E-05	2.47E-01	8.33E-04	4.32E-01
12442	GSM786521	GSE31684	Bladder	Bladder	6.68E-11	3.83E-01	6.53E-07	6.12E-01
12443	GSM786522	GSE31684	Bladder	Bladder	4.31E-04	2.20E-01	1.88E-03	4.07E-01
12444	GSM786523	GSE31684	Bladder	Bladder	4.62E-04	-2.19E-01	8.35E-02	-2.56E-01
12445	GSM786524	GSE31684	Bladder	Bladder	1.81E-06	2.88E-01	7.48E-04	4.35E-01
12446	GSM786525	GSE31684	Bladder	Bladder	1.80E-13	-4.29E-01	6.25E-05	-5.05E-01
12447	GSM786526	GSE31684	Bladder	Bladder	3.04E-01	8.63E-02	2.42E-01	1.93E-01
12448	GSM786527	GSE31684	Bladder	Bladder	4.05E-08	-3.26E-01	1.15E-02	-3.43E-01
12449	GSM786528	GSE31684	Bladder	Bladder	7.67E-03	-1.75E-01	2.68E-01	-1.86E-01
12450	GSM786529	GSE31684	Bladder	Bladder	6.08E-04	-2.15E-01	2.96E-01	-1.79E-01
12451	GSM786530	GSE31684	Bladder	Bladder	2.18E-02	1.55E-01	4.28E-03	3.79E-01
12452	GSM786531	GSE31684	Bladder	Bladder	8.91E-02	1.23E-01	2.59E-02	3.10E-01
12453	GSM786532	GSE31684	Bladder	Bladder	3.38E-01	8.23E-02	1.78E-01	2.13E-01
12454	GSM786533	GSE31684	Bladder	Bladder	1.98E-09	-3.54E-01	4.12E-02	-2.90E-01
12455	GSM786534	GSE31684	Bladder	Bladder	1.52E-11	-3.95E-01	4.82E-04	-4.49E-01
12456	GSM786535	GSE31684	Bladder	Bladder	5.64E-10	-3.65E-01	1.96E-03	-4.05E-01
12457	GSM786536	GSE31684	Bladder	Bladder	7.01E-02	-1.29E-01	3.04E-01	-1.77E-01
12458	GSM786537	GSE31684	Bladder	Bladder	2.37E-28	6.31E-01	1.30E-11	8.12E-01
12459	GSM786538	GSE31684	Bladder	Bladder	1.96E-04	2.31E-01	5.55E-03	3.70E-01
12460	GSM786539	GSE31684	Bladder	Bladder	1.17E-12	-4.15E-01	2.00E-03	-4.05E-01
12461	GSM786540	GSE31684	Bladder	Bladder	2.56E-12	-4.09E-01	1.33E-03	-4.18E-01
12462	GSM786541	GSE31684	Bladder	Bladder	1.40E-24	-5.86E-01	8.65E-06	-5.54E-01
12463	GSM786542	GSE31684	Bladder	Bladder	1.37E-13	-4.31E-01	1.16E-04	-4.89E-01
12464	GSM786543	GSE31684	Bladder	Bladder	7.97E-10	3.62E-01	1.34E-04	4.85E-01
12465	GSM786544	GSE31684	Bladder	Bladder	8.86E-06	2.70E-01	7.48E-04	4.35E-01
12466	GSM786545	GSE31684	Bladder	Bladder	3.67E-02	1.44E-01	5.03E-02	2.81E-01
12467	GSM786546	GSE31684	Bladder	Bladder	3.25E-04	-2.24E-01	2.59E-02	-3.10E-01
12468	GSM786547	GSE31684	Bladder	Bladder	2.52E-02	-1.52E-01	8.45E-02	2.55E-01
12469	GSM786548	GSE31684	Bladder	Bladder	1.72E-11	-3.94E-01	1.67E-03	-4.10E-01
12470	GSM786549	GSE31684	Bladder	Bladder	1.25E-02	1.66E-01	5.03E-02	2.81E-01
12471	GSM786550	GSE31684	Bladder	Bladder	4.03E-07	3.04E-01	7.81E-04	4.34E-01
12472	GSM786551	GSE31684	Bladder	Bladder	4.63E-01	6.94E-02	2.23E-01	1.99E-01
12473	GSM786552	GSE31684	Bladder	Bladder	1.12E-07	-3.16E-01	6.96E-02	-2.65E-01
12474	GSM786553	GSE31684	Bladder	Bladder	2.05E-05	-2.60E-01	8.35E-02	-2.56E-01
12475	GSM786554	GSE31684	Bladder	Bladder	6.16E-21	-5.40E-01	4.68E-06	-5.69E-01
12476	GSM786555	GSE31684	Bladder	Bladder	3.68E-21	-5.43E-01	1.27E-05	-5.45E-01
12477	GSM786556	GSE31684	Bladder	Bladder	2.25E-22	-5.59E-01	2.90E-05	-5.25E-01
12478	GSM786557	GSE31684	Bladder	Bladder	3.50E-02	1.45E-01	8.35E-02	2.56E-01
12479	GSM786558	GSE31684	Bladder	Bladder	1.58E-16	-4.77E-01	3.76E-04	-4.56E-01
12480	GSM786559	GSE31684	Bladder	Bladder	2.79E-18	-5.03E-01	1.27E-05	-5.45E-01
12481	GSM786560	GSE31684	Bladder	Bladder	1.95E-11	3.93E-01	1.38E-05	5.43E-01
12482	GSM786561	GSE31684	Bladder	Bladder	1.77E-09	3.55E-01	9.40E-06	5.52E-01
12483	GSM786562	GSE31684	Bladder	Bladder	3.54E-10	-3.69E-01	2.75E-03	-3.94E-01
12484	GSM786563	GSE31684	Bladder	Bladder	2.59E-06	-2.84E-01	1.07E-01	-2.43E-01
12485	GSM786564	GSE31684	Bladder	Bladder	4.83E-06	-2.77E-01	2.64E-03	-3.95E-01
12486	GSM786565	GSE31684	Bladder	Bladder	2.25E-22	-5.59E-01	4.68E-06	-5.69E-01
12487	GSM786566	GSE31684	Bladder	Bladder	7.84E-07	-2.97E-01	2.47E-02	-3.12E-01
12488	GSM786567	GSE31684	Bladder	Bladder	8.32E-26	-6.01E-01	1.27E-05	-5.45E-01
12489	GSM786568	GSE31684	Bladder	Bladder	8.57E-02	1.24E-01	1.60E-03	4.12E-01
12490	GSM786569	GSE31684	Bladder	Bladder	5.16E-03	1.82E-01	3.27E-03	3.88E-01
12491	GSM786570	GSE31684	Bladder	Bladder	3.83E-18	-5.01E-01	5.86E-06	-5.63E-01
12492	GSM786571	GSE31684	Bladder	Bladder	3.21E-01	8.43E-02	2.97E-02	3.04E-01
12493	GSM786572	GSE31684	Bladder	Bladder	4.28E-12	-4.05E-01	6.67E-03	-3.63E-01
12494	GSM786573	GSE31684	Bladder	Bladder	8.52E-04	-2.10E-01	2.30E-01	-1.97E-01
12495	GSM786574	GSE31684	Bladder	Bladder	1.28E-15	-4.63E-01	1.97E-04	-4.74E-01
12496	GSM786575	GSE31684	Bladder	Bladder	1.84E-21	5.47E-01	4.87E-11	7.91E-01
12497	GSM786576	GSE31684	Bladder	Bladder	2.02E-18	-5.05E-01	6.38E-06	-5.61E-01
12498	GSM786577	GSE31684	Bladder	Bladder	1.55E-21	5.48E-01	9.40E-09	6.98E-01
12499	GSM786578	GSE31684	Bladder	Bladder	3.39E-06	-2.81E-01	7.43E-03	-3.59E-01
12500	GSM786579	GSE31684	Bladder	Bladder	1.14E-06	-2.93E-01	3.77E-02	-2.94E-01
12501	GSM786580	GSE31684	Bladder	Bladder	2.62E-05	2.57E-01	8.65E-06	5.54E-01

12502	GSM786581	GSE31684	Bladder	Bladder	3.93E-05	-2.52E-01	1.48E-01	-2.24E-01
12503	GSM786582	GSE31684	Bladder	Bladder	1.50E-06	2.90E-01	1.20E-03	4.21E-01
12504	GSM786583	GSE31684	Bladder	Bladder	3.03E-04	2.25E-01	4.02E-04	4.54E-01
12505	GSM180994	GSE7476	Bladder	Bladder	8.94E-10	-3.61E-01	4.04E-03	-3.81E-01
12506	GSM180995	GSE7476	Bladder	Bladder	6.48E-23	-5.65E-01	4.30E-06	-5.70E-01
12507	GSM180996	GSE7476	Bladder	Bladder	6.35E-17	-4.83E-01	2.28E-06	-5.85E-01
12508	GSM180997	GSE7476	Bladder	Bladder	3.86E-09	-3.48E-01	3.51E-04	-4.58E-01
12509	GSM180998	GSE7476	Bladder	Bladder	2.59E-06	-2.84E-01	1.83E-02	-3.25E-01
12510	GSM180999	GSE7476	Bladder	Bladder	1.50E-06	-2.90E-01	3.21E-02	-3.01E-01
12511	GSM181000	GSE7476	Bladder	Bladder	2.11E-04	-2.30E-01	5.61E-02	-2.75E-01
12512	GSM181001	GSE7476	Bladder	Bladder	5.39E-05	-2.48E-01	1.03E-01	-2.45E-01
12513	GSM181002	GSE7476	Bladder	Bladder	2.44E-04	-2.28E-01	7.83E-02	-2.59E-01
12514	GSM119491	GSE5287	Bladder	Bladder	4.59E-02	-1.39E-01	4.18E-01	-1.51E-01
12515	GSM119492	GSE5287	Bladder	Bladder	1.57E-04	2.34E-01	1.50E-03	4.14E-01
12516	GSM119493	GSE5287	Bladder	Bladder	5.10E-24	-5.79E-01	4.24E-07	-6.22E-01
12517	GSM119494	GSE5287	Bladder	Bladder	3.43E-17	4.87E-01	7.36E-08	6.58E-01
12518	GSM119495	GSE5287	Bladder	Bladder	1.11E-14	-4.48E-01	5.11E-05	-5.10E-01
12519	GSM119497	GSE5287	Bladder	Bladder	2.49E-01	-9.33E-02	3.75E-01	1.61E-01
12520	GSM119498	GSE5287	Bladder	Bladder	9.51E-16	-4.65E-01	6.56E-04	-4.39E-01
12521	GSM119499	GSE5287	Bladder	Bladder	4.33E-01	7.24E-02	2.84E-02	3.06E-01
12522	GSM119500	GSE5287	Bladder	Bladder	5.27E-06	-2.76E-01	1.98E-02	-3.21E-01
12523	GSM119501	GSE5287	Bladder	Bladder	2.44E-24	5.83E-01	9.46E-11	7.80E-01
12524	GSM119502	GSE5287	Bladder	Bladder	4.39E-02	-1.40E-01	1.22E-01	2.35E-01
12525	GSM119711	GSE5287	Bladder	Bladder	1.01E-02	-1.70E-01	1.34E-01	-2.30E-01
12526	GSM119712	GSE5287	Bladder	Bladder	4.02E-04	2.21E-01	2.49E-03	3.97E-01
12527	GSM119713	GSE5287	Bladder	Bladder	4.60E-03	1.84E-01	1.50E-03	4.14E-01
12528	GSM119714	GSE5287	Bladder	Bladder	5.91E-07	-3.00E-01	3.07E-02	-3.03E-01
12529	GSM119716	GSE5287	Bladder	Bladder	1.18E-02	-1.67E-01	1.67E-01	-2.17E-01
12530	GSM119720	GSE5287	Bladder	Bladder	4.02E-04	-2.21E-01	1.25E-01	-2.34E-01
12531	GSM119721	GSE5287	Bladder	Bladder	1.96E-04	-2.31E-01	1.38E-01	-2.28E-01
12532	GSM119722	GSE5287	Bladder	Bladder	5.64E-10	-3.65E-01	4.12E-02	-2.90E-01
12533	GSM119723	GSE5287	Bladder	Bladder	1.44E-01	-1.10E-01	5.03E-02	2.81E-01
12534	GSM119724	GSE5287	Bladder	Bladder	4.79E-02	-1.38E-01	1.94E-01	-2.08E-01
12535	GSM119725	GSE5287	Bladder	Bladder	1.88E-02	1.58E-01	4.12E-02	2.90E-01
12536	GSM119726	GSE5287	Bladder	Bladder	2.42E-05	-2.58E-01	2.47E-02	-3.12E-01
12537	GSM119727	GSE5287	Bladder	Bladder	3.74E-04	-2.22E-01	1.57E-01	-2.21E-01
12538	GSM119728	GSE5287	Bladder	Bladder	2.21E-09	-3.53E-01	8.89E-04	-4.30E-01
12539	GSM119729	GSE5287	Bladder	Bladder	2.19E-30	-6.54E-01	8.46E-09	-7.00E-01
12540	GSM119730	GSE5287	Bladder	Bladder	2.44E-04	2.28E-01	6.79E-03	3.63E-01
12541	GSM119731	GSE5287	Bladder	Bladder	1.14E-05	-2.67E-01	5.76E-03	-3.69E-01
12542	GSM119732	GSE5287	Bladder	Bladder	2.83E-11	-3.90E-01	6.74E-05	-5.03E-01
12543	GSM119733	GSE5287	Bladder	Bladder	1.38E-02	1.64E-01	3.82E-03	3.83E-01
12544	GSM466947	GSE18842	Lung	Lung	4.13E-01	7.44E-02	6.78E-02	2.66E-01
12545	GSM466949	GSE18842	Lung	Lung	8.30E-08	-3.19E-01	1.46E-02	-3.34E-01
12546	GSM466951	GSE18842	Lung	Lung	1.71E-01	1.05E-01	1.18E-01	2.37E-01
12547	GSM466952	GSE18842	Lung	Lung	3.49E-04	-2.23E-01	3.94E-01	1.57E-01
12548	GSM466954	GSE18842	Lung	Lung	8.91E-02	-1.23E-01	2.88E-01	-1.81E-01
12549	GSM466956	GSE18842	Lung	Lung	3.94E-01	-7.64E-02	5.38E-01	-1.28E-01
12550	GSM466958	GSE18842	Lung	Lung	2.07E-02	-1.56E-01	4.18E-01	-1.51E-01
12551	GSM466960	GSE18842	Lung	Lung	2.24E-03	-1.95E-01	1.82E-01	-2.12E-01
12552	GSM466962	GSE18842	Lung	Lung	3.47E-01	-8.13E-02	1.35E-01	2.30E-01
12553	GSM466963	GSE18842	Lung	Lung	3.66E-01	-7.94E-02	2.35E-01	1.95E-01
12554	GSM466965	GSE18842	Lung	Lung	2.28E-01	-9.62E-02	3.12E-01	1.75E-01
12555	GSM466967	GSE18842	Lung	Lung	1.02E-07	-3.17E-01	9.51E-03	-3.50E-01
12556	GSM466969	GSE18842	Lung	Lung	1.05E-05	-2.68E-01	9.51E-03	-3.50E-01
12557	GSM466971	GSE18842	Lung	Lung	2.77E-02	1.50E-01	3.77E-02	2.94E-01
12558	GSM466973	GSE18842	Lung	Lung	2.01E-01	-1.00E-01	4.38E-01	-1.48E-01
12559	GSM466975	GSE18842	Lung	Lung	1.35E-05	-2.65E-01	1.32E-02	-3.38E-01
12560	GSM466977	GSE18842	Lung	Lung	4.60E-03	-1.84E-01	2.91E-01	-1.80E-01
12561	GSM466980	GSE18842	Lung	Lung	2.64E-01	-9.13E-02	2.00E-01	2.06E-01
12562	GSM466982	GSE18842	Lung	Lung	1.53E-03	-2.01E-01	1.48E-01	-2.24E-01
12563	GSM466983	GSE18842	Lung	Lung	3.67E-02	1.44E-01	3.07E-02	3.03E-01
12564	GSM466985	GSE18842	Lung	Lung	1.97E-03	-1.97E-01	3.01E-01	-1.78E-01
12565	GSM466987	GSE18842	Lung	Lung	2.25E-07	-3.10E-01	2.80E-02	-3.07E-01
12566	GSM466989	GSE18842	Lung	Lung	1.35E-03	-2.03E-01	1.10E-01	-2.41E-01
12567	GSM466991	GSE18842	Lung	Lung	2.41E-08	-3.31E-01	1.77E-03	-4.09E-01
12568	GSM466993	GSE18842	Lung	Lung	1.73E-27	-6.21E-01	1.24E-07	-6.47E-01
12569	GSM466994	GSE18842	Lung	Lung	6.21E-01	-5.46E-02	5.65E-01	1.23E-01
12570	GSM466996	GSE18842	Lung	Lung	6.19E-02	-1.32E-01	5.20E-01	-1.31E-01
12571	GSM466998	GSE18842	Lung	Lung	3.96E-14	-4.39E-01	2.11E-04	-4.72E-01
12572	GSM467004	GSE18842	Lung	Lung	1.74E-03	1.99E-01	1.46E-02	3.34E-01
12573	GSM467006	GSE18842	Lung	Lung	1.02E-12	4.16E-01	1.50E-07	6.43E-01
12574	GSM467008	GSE18842	Lung	Lung	6.12E-08	3.22E-01	1.75E-04	4.77E-01
12575	GSM467010	GSE18842	Lung	Lung	2.56E-12	4.09E-01	1.38E-05	5.43E-01

12576	GSM467012	GSE18842	Lung	Lung	1.06E-02	-1.69E-01	3.94E-01	1.57E-01
12577	GSM467014	GSE18842	Lung	Lung	1.04E-11	-3.98E-01	1.04E-02	-3.47E-01
12578	GSM467016	GSE18842	Lung	Lung	5.27E-06	-2.76E-01	4.43E-02	-2.87E-01
12579	GSM467018	GSE18842	Lung	Lung	1.83E-01	1.03E-01	5.85E-02	2.73E-01
12580	GSM467021	GSE18842	Lung	Lung	7.84E-07	2.97E-01	6.74E-05	5.03E-01
12581	GSM467023	GSE18842	Lung	Lung	3.94E-01	7.64E-02	2.42E-01	1.93E-01
12582	GSM467024	GSE18842	Lung	Lung	1.44E-01	1.10E-01	1.60E-01	2.20E-01
12583	GSM467026	GSE18842	Lung	Lung	3.21E-01	8.43E-02	2.30E-01	1.97E-01
12584	GSM467028	GSE18842	Lung	Lung	1.01E-02	-1.70E-01	2.30E-01	-1.97E-01
12585	GSM467029	GSE18842	Lung	Lung	3.08E-05	-2.55E-01	4.24E-02	-2.89E-01
12586	GSM467030	GSE18842	Lung	Lung	1.27E-08	-3.37E-01	3.09E-03	-3.90E-01
12587	GSM467032	GSE18842	Lung	Lung	1.14E-06	-2.93E-01	1.41E-03	-4.16E-01
12588	GSM467034	GSE18842	Lung	Lung	1.97E-02	-1.57E-01	2.53E-01	-1.90E-01
12589	GSM467036	GSE18842	Lung	Lung	2.44E-24	-5.83E-01	4.20E-08	-6.69E-01
12590	GSM258551	GSE10245	non-small cell lung cancer Tumour	Lung	1.16E-04	2.38E-01	5.24E-02	2.79E-01
12591	GSM258552	GSE10245	non-small cell lung cancer Tumour	Lung	1.25E-01	1.14E-01	7.53E-02	2.61E-01
12592	GSM258553	GSE10245	non-small cell lung cancer Tumour	Lung	3.47E-01	8.13E-02	2.49E-01	1.91E-01
12593	GSM258554	GSE10245	non-small cell lung cancer Tumour	Lung	4.03E-01	7.54E-02	2.00E-01	2.06E-01
12594	GSM258555	GSE10245	non-small cell lung cancer Tumour	Lung	8.52E-04	-2.10E-01	1.82E-01	-2.12E-01
12595	GSM258556	GSE10245	non-small cell lung cancer Tumour	Lung	1.97E-03	-1.97E-01	7.83E-02	-2.59E-01
12596	GSM258557	GSE10245	non-small cell lung cancer Tumour	Lung	1.97E-10	3.74E-01	2.48E-05	5.29E-01
12597	GSM258558	GSE10245	non-small cell lung cancer Tumour	Lung	6.00E-01	5.65E-02	7.83E-02	2.59E-01
12598	GSM258559	GSE10245	non-small cell lung cancer Tumour	Lung	1.44E-01	-1.10E-01	5.96E-01	-1.17E-01
12599	GSM258560	GSE10245	non-small cell lung cancer Tumour	Lung	2.52E-02	-1.52E-01	5.24E-02	2.79E-01
12600	GSM258561	GSE10245	non-small cell lung cancer Tumour	Lung	1.96E-04	-2.31E-01	9.35E-02	-2.50E-01
12601	GSM258562	GSE10245	non-small cell lung cancer Tumour	Lung	3.21E-01	-8.43E-02	2.83E-01	1.82E-01
12602	GSM258563	GSE10245	non-small cell lung cancer Tumour	Lung	3.86E-09	3.48E-01	1.27E-05	5.45E-01
12603	GSM258564	GSE10245	non-small cell lung cancer Tumour	Lung	1.25E-02	-1.66E-01	4.75E-02	-2.83E-01
12604	GSM258565	GSE10245	non-small cell lung cancer Tumour	Lung	1.00E-01	1.20E-01	2.51E-02	3.11E-01
12605	GSM258566	GSE10245	non-small cell lung cancer Tumour	Lung	1.30E-01	-1.13E-01	3.20E-01	1.73E-01
12606	GSM258567	GSE10245	non-small cell lung cancer Tumour	Lung	3.03E-04	2.25E-01	3.27E-03	3.88E-01
12607	GSM258568	GSE10245	non-small cell lung cancer Tumour	Lung	4.05E-08	-3.26E-01	2.73E-04	-4.65E-01
12608	GSM258569	GSE10245	non-small cell lung cancer Tumour	Lung	2.21E-09	-3.53E-01	1.63E-04	-4.79E-01
12609	GSM258570	GSE10245	non-small cell lung cancer Tumour	Lung	1.65E-01	-1.06E-01	3.51E-02	2.97E-01
12610	GSM258571	GSE10245	non-small cell lung cancer Tumour	Lung	4.60E-03	-1.84E-01	4.34E-01	-1.48E-01
12611	GSM258572	GSE10245	non-small cell lung cancer Tumour	Lung	9.73E-04	2.08E-01	3.07E-02	3.03E-01
12612	GSM258573	GSE10245	non-small cell lung cancer Tumour	Lung	2.52E-02	-1.52E-01	3.49E-01	-1.67E-01
12613	GSM258574	GSE10245	non-small cell lung cancer Tumour	Lung	1.35E-03	2.03E-01	6.14E-04	4.41E-01
12614	GSM258575	GSE10245	non-small cell lung cancer Tumour	Lung	2.28E-01	-9.62E-02	5.10E-01	1.33E-01
12615	GSM258576	GSE10245	non-small cell lung cancer Tumour	Lung	2.23E-05	-2.59E-01	9.23E-02	-2.50E-01
12616	GSM258577	GSE10245	non-small cell lung cancer Tumour	Lung	6.84E-06	2.73E-01	3.51E-04	4.58E-01
12617	GSM258578	GSE10245	non-small cell lung cancer Tumour	Lung	8.91E-02	-1.23E-01	2.42E-01	1.93E-01
12618	GSM258579	GSE10245	non-small cell lung cancer Tumour	Lung	1.38E-10	3.77E-01	6.25E-05	5.05E-01
12619	GSM258580	GSE10245	non-small cell lung cancer Tumour	Lung	6.81E-05	2.45E-01	1.88E-03	4.07E-01
12620	GSM258581	GSE10245	non-small cell lung cancer Tumour	Lung	1.88E-02	-1.58E-01	3.12E-01	-1.75E-01
12621	GSM258582	GSE10245	non-small cell lung cancer Tumour	Lung	5.30E-04	-2.17E-01	6.78E-02	-2.66E-01
12622	GSM258583	GSE10245	non-small cell lung cancer Tumour	Lung	3.45E-09	3.49E-01	1.16E-04	4.89E-01
12623	GSM258584	GSE10245	non-small cell lung cancer Tumour	Lung	1.05E-05	2.68E-01	9.54E-05	4.94E-01
12624	GSM258585	GSE10245	non-small cell lung cancer Tumour	Lung	3.45E-09	3.49E-01	9.54E-05	4.94E-01
12625	GSM258586	GSE10245	non-small cell lung cancer Tumour	Lung	7.46E-06	2.72E-01	4.31E-04	4.52E-01
12626	GSM258587	GSE10245	non-small cell lung cancer Tumour	Lung	2.05E-05	-2.60E-01	1.03E-01	-2.45E-01
12627	GSM258588	GSE10245	non-small cell lung cancer Tumour	Lung	4.79E-02	1.38E-01	1.61E-02	3.30E-01
12628	GSM258589	GSE10245	non-small cell lung cancer Tumour	Lung	3.93E-05	-2.52E-01	9.00E-02	-2.52E-01
12629	GSM258590	GSE10245	non-small cell lung cancer Tumour	Lung	2.91E-02	1.49E-01	3.47E-03	3.86E-01
12630	GSM258591	GSE10245	non-small cell lung cancer Tumour	Lung	1.01E-02	-1.70E-01	7.05E-02	-2.64E-01
12631	GSM258592	GSE10245	non-small cell lung cancer Tumour	Lung	1.18E-03	-2.05E-01	2.21E-01	-1.99E-01
12632	GSM258593	GSE10245	non-small cell lung cancer Tumour	Lung	1.65E-06	-2.89E-01	4.62E-02	-2.85E-01
12633	GSM258594	GSE10245	non-small cell lung cancer Tumour	Lung	6.21E-01	-5.46E-02	2.76E-01	1.84E-01
12634	GSM258595	GSE10245	non-small cell lung cancer Tumour	Lung	2.77E-02	-1.50E-01	2.42E-01	-1.93E-01
12635	GSM258596	GSE10245	non-small cell lung cancer Tumour	Lung	2.27E-04	2.29E-01	9.47E-04	4.28E-01
12636	GSM258597	GSE10245	non-small cell lung cancer Tumour	Lung	9.11E-04	-2.09E-01	6.51E-02	-2.68E-01
12637	GSM258598	GSE10245	non-small cell lung cancer Tumour	Lung	3.62E-11	-3.88E-01	6.14E-04	-4.41E-01
12638	GSM258599	GSE10245	non-small cell lung cancer Tumour	Lung	4.23E-01	-7.34E-02	3.26E-01	1.72E-01
12639	GSM258600	GSE10245	non-small cell lung cancer Tumour	Lung	1.83E-01	-1.03E-01	4.80E-01	1.39E-01
12640	GSM258601	GSE10245	non-small cell lung cancer Tumour	Lung	3.74E-04	-2.22E-01	1.10E-01	-2.41E-01
12641	GSM258602	GSE10245	non-small cell lung cancer Tumour	Lung	5.46E-02	-1.35E-01	4.54E-01	1.44E-01
12642	GSM258603	GSE10245	non-small cell lung cancer Tumour	Lung	4.31E-04	2.20E-01	1.27E-03	4.19E-01
12643	GSM258604	GSE10245	non-small cell lung cancer Tumour	Lung	3.34E-02	-1.46E-01	4.50E-01	-1.45E-01
12644	GSM258605	GSE10245	non-small cell lung cancer Tumour	Lung	1.08E-01	-1.18E-01	1.48E-01	2.24E-01
12645	GSM258606	GSE10245	non-small cell lung cancer Tumour	Lung	4.09E-03	-1.86E-01	4.87E-01	-1.38E-01
12646	GSM258607	GSE10245	non-small cell lung cancer Tumour	Lung	5.79E-03	-1.80E-01	4.31E-01	-1.49E-01
12647	GSM258608	GSE10245	non-small cell lung cancer Tumour	Lung	8.30E-08	3.19E-01	5.15E-04	4.47E-01
12648	GSM370913	GSE14814	primary lung cancer	Lung	5.26E-16	-4.69E-01	7.54E-06	-5.57E-01
12649	GSM370914	GSE14814	primary lung cancer	Lung	4.10E-11	-3.87E-01	1.77E-03	-4.09E-01

12650	GSM370915	GSE14814	primary lung cancer	Lung	1.77E-01	1.04E-01	4.04E-03	3.81E-01
12651	GSM370916	GSE14814	primary lung cancer	Lung	3.13E-01	-8.53E-02	5.10E-01	-1.33E-01
12652	GSM370917	GSE14814	primary lung cancer	Lung	6.08E-04	2.15E-01	3.21E-02	3.01E-01
12653	GSM370918	GSE14814	primary lung cancer	Lung	3.50E-02	-1.45E-01	5.10E-01	-1.33E-01
12654	GSM370919	GSE14814	primary lung cancer	Lung	3.67E-02	-1.44E-01	2.17E-01	-2.01E-01
12655	GSM370920	GSE14814	primary lung cancer	Lung	2.64E-01	-9.13E-02	3.84E-01	1.59E-01
12656	GSM370921	GSE14814	primary lung cancer	Lung	2.27E-04	-2.29E-01	1.06E-02	-3.46E-01
12657	GSM370922	GSE14814	primary lung cancer	Lung	1.21E-01	1.15E-01	9.94E-02	2.47E-01
12658	GSM370923	GSE14814	primary lung cancer	Lung	1.58E-09	3.56E-01	1.38E-05	5.43E-01
12659	GSM370924	GSE14814	primary lung cancer	Lung	4.39E-02	-1.40E-01	2.23E-01	-1.99E-01
12660	GSM370925	GSE14814	primary lung cancer	Lung	3.30E-01	-8.33E-02	1.57E-01	2.21E-01
12661	GSM370926	GSE14814	primary lung cancer	Lung	1.95E-01	1.01E-01	1.14E-01	2.39E-01
12662	GSM370927	GSE14814	primary lung cancer	Lung	1.50E-06	2.90E-01	5.15E-04	4.47E-01
12663	GSM370928	GSE14814	primary lung cancer	Lung	2.08E-01	-9.92E-02	1.73E-01	-2.15E-01
12664	GSM370929	GSE14814	primary lung cancer	Lung	4.63E-01	6.94E-02	8.14E-02	2.57E-01
12665	GSM370930	GSE14814	primary lung cancer	Lung	3.84E-01	7.74E-02	1.78E-01	2.13E-01
12666	GSM370931	GSE14814	primary lung cancer	Lung	1.35E-03	-2.03E-01	6.79E-03	-3.63E-01
12667	GSM370932	GSE14814	primary lung cancer	Lung	1.00E-09	3.60E-01	8.86E-05	4.96E-01
12668	GSM370933	GSE14814	primary lung cancer	Lung	3.34E-02	1.46E-01	1.57E-01	2.21E-01
12669	GSM370934	GSE14814	primary lung cancer	Lung	4.03E-01	7.54E-02	1.53E-01	2.22E-01
12670	GSM370935	GSE14814	primary lung cancer	Lung	2.64E-01	9.13E-02	2.28E-01	1.97E-01
12671	GSM370936	GSE14814	primary lung cancer	Lung	2.91E-02	1.49E-01	2.29E-02	3.15E-01
12672	GSM370937	GSE14814	primary lung cancer	Lung	2.64E-01	9.13E-02	3.27E-03	3.88E-01
12673	GSM370938	GSE14814	primary lung cancer	Lung	1.88E-02	-1.58E-01	3.90E-01	-1.57E-01
12674	GSM370939	GSE14814	primary lung cancer	Lung	1.04E-01	1.19E-01	2.97E-02	3.04E-01
12675	GSM370940	GSE14814	primary lung cancer	Lung	2.72E-01	-9.03E-02	5.24E-02	2.79E-01
12676	GSM370941	GSE14814	primary lung cancer	Lung	2.88E-01	-8.83E-02	2.91E-01	1.80E-01
12677	GSM370942	GSE14814	primary lung cancer	Lung	3.04E-01	8.63E-02	6.78E-02	2.66E-01
12678	GSM370943	GSE14814	primary lung cancer	Lung	1.26E-03	2.04E-01	1.63E-04	4.79E-01
12679	GSM370944	GSE14814	primary lung cancer	Lung	7.45E-04	2.12E-01	6.01E-02	2.72E-01
12680	GSM370945	GSE14814	primary lung cancer	Lung	5.30E-04	-2.17E-01	2.28E-01	-1.97E-01
12681	GSM370946	GSE14814	primary lung cancer	Lung	6.89E-13	4.19E-01	1.82E-07	6.39E-01
12682	GSM370947	GSE14814	primary lung cancer	Lung	1.00E-04	2.40E-01	9.89E-04	4.27E-01
12683	GSM370948	GSE14814	primary lung cancer	Lung	5.68E-01	5.95E-02	2.00E-01	2.06E-01
12684	GSM370949	GSE14814	primary lung cancer	Lung	1.85E-03	-1.98E-01	1.31E-01	2.31E-01
12685	GSM370950	GSE14814	primary lung cancer	Lung	7.45E-04	-2.12E-01	1.88E-01	-2.10E-01
12686	GSM370951	GSE14814	primary lung cancer	Lung	4.63E-01	-6.94E-02	5.48E-01	1.26E-01
12687	GSM370952	GSE14814	primary lung cancer	Lung	2.64E-02	1.51E-01	5.39E-02	2.77E-01
12688	GSM370953	GSE14814	primary lung cancer	Lung	1.79E-02	-1.59E-01	1.78E-01	2.13E-01
12689	GSM370954	GSE14814	primary lung cancer	Lung	4.63E-01	6.94E-02	8.14E-02	2.57E-01
12690	GSM370955	GSE14814	primary lung cancer	Lung	1.12E-01	-1.17E-01	5.07E-01	-1.34E-01
12691	GSM370956	GSE14814	primary lung cancer	Lung	3.38E-01	-8.23E-02	5.58E-01	1.24E-01
12692	GSM370957	GSE14814	primary lung cancer	Lung	4.04E-13	4.23E-01	1.58E-05	5.40E-01
12693	GSM370958	GSE14814	primary lung cancer	Lung	1.26E-03	2.04E-01	4.73E-05	5.12E-01
12694	GSM370959	GSE14814	primary lung cancer	Lung	7.46E-06	2.72E-01	1.75E-04	4.77E-01
12695	GSM370960	GSE14814	primary lung cancer	Lung	1.70E-02	-1.60E-01	5.48E-01	-1.26E-01
12696	GSM370961	GSE14814	primary lung cancer	Lung	7.01E-02	-1.29E-01	4.73E-01	1.40E-01
12697	GSM370962	GSE14814	primary lung cancer	Lung	1.83E-01	-1.03E-01	3.20E-01	1.73E-01
12698	GSM370963	GSE14814	primary lung cancer	Lung	7.11E-10	3.63E-01	9.40E-06	5.52E-01
12699	GSM370964	GSE14814	primary lung cancer	Lung	7.01E-02	-1.29E-01	1.15E-01	2.39E-01
12700	GSM370965	GSE14814	primary lung cancer	Lung	2.53E-03	-1.93E-01	5.58E-01	1.24E-01
12701	GSM370966	GSE14814	primary lung cancer	Lung	3.54E-10	-3.69E-01	1.32E-02	-3.38E-01
12702	GSM370967	GSE14814	primary lung cancer	Lung	1.96E-04	-2.31E-01	5.39E-02	-2.77E-01
12703	GSM370968	GSE14814	primary lung cancer	Lung	6.13E-03	-1.79E-01	6.26E-02	-2.70E-01
12704	GSM370969	GSE14814	primary lung cancer	Lung	7.27E-01	-4.46E-02	1.03E-01	2.45E-01
12705	GSM370970	GSE14814	primary lung cancer	Lung	2.01E-01	-1.00E-01	2.56E-01	1.90E-01
12706	GSM370971	GSE14814	primary lung cancer	Lung	6.81E-05	-2.45E-01	1.74E-02	-3.27E-01
12707	GSM370972	GSE14814	primary lung cancer	Lung	2.21E-01	-9.72E-02	3.66E-01	1.63E-01
12708	GSM370973	GSE14814	primary lung cancer	Lung	3.03E-04	-2.25E-01	8.14E-02	-2.57E-01
12709	GSM370974	GSE14814	primary lung cancer	Lung	3.66E-01	-7.94E-02	1.53E-01	2.22E-01
12710	GSM370975	GSE14814	primary lung cancer	Lung	8.24E-02	1.25E-01	4.30E-02	2.88E-01
12711	GSM370976	GSE14814	primary lung cancer	Lung	4.33E-01	7.24E-02	8.14E-02	2.57E-01
12712	GSM370977	GSE14814	primary lung cancer	Lung	1.60E-05	-2.63E-01	6.78E-02	-2.66E-01
12713	GSM370978	GSE14814	primary lung cancer	Lung	1.57E-04	2.34E-01	2.35E-03	3.99E-01
12714	GSM370979	GSE14814	primary lung cancer	Lung	4.98E-08	-3.24E-01	7.83E-03	-3.57E-01
12715	GSM370980	GSE14814	primary lung cancer	Lung	3.54E-10	-3.69E-01	1.32E-02	-3.38E-01
12716	GSM370981	GSE14814	primary lung cancer	Lung	2.11E-04	-2.30E-01	1.22E-01	-2.35E-01
12717	GSM370982	GSE14814	primary lung cancer	Lung	5.01E-02	-1.37E-01	5.27E-01	1.30E-01
12718	GSM370983	GSE14814	primary lung cancer	Lung	1.65E-01	-1.06E-01	4.38E-01	1.48E-01
12719	GSM370984	GSE14814	primary lung cancer	Lung	3.03E-04	-2.25E-01	1.76E-01	-2.14E-01
12720	GSM370985	GSE14814	primary lung cancer	Lung	4.79E-02	1.38E-01	3.61E-02	2.96E-01
12721	GSM370986	GSE14814	primary lung cancer	Lung	2.60E-14	4.42E-01	3.42E-06	5.76E-01
12722	GSM370987	GSE14814	primary lung cancer	Lung	2.91E-02	1.49E-01	2.59E-02	3.10E-01
12723	GSM370988	GSE14814	primary lung cancer	Lung	1.71E-15	-4.61E-01	8.33E-04	-4.32E-01

12724	GSM370989	GSE14814	primary lung cancer	Lung	1.74E-03	1.99E-01	5.39E-02	2.77E-01
12725	GSM370990	GSE14814	primary lung cancer	Lung	4.53E-01	-7.04E-02	2.56E-01	1.90E-01
12726	GSM370991	GSE14814	primary lung cancer	Lung	8.59E-05	2.42E-01	5.55E-03	3.70E-01
12727	GSM370992	GSE14814	primary lung cancer	Lung	4.79E-02	1.38E-01	2.29E-02	3.15E-01
12728	GSM370993	GSE14814	primary lung cancer	Lung	3.19E-02	-1.47E-01	3.75E-01	1.61E-01
12729	GSM370994	GSE14814	primary lung cancer	Lung	2.49E-01	-9.33E-02	3.43E-01	-1.68E-01
12730	GSM370995	GSE14814	primary lung cancer	Lung	1.65E-01	1.06E-01	1.67E-01	2.17E-01
12731	GSM370996	GSE14814	primary lung cancer	Lung	1.46E-02	1.63E-01	1.27E-02	3.39E-01
12732	GSM370997	GSE14814	primary lung cancer	Lung	1.97E-03	-1.97E-01	1.62E-01	-2.19E-01
12733	GSM370998	GSE14814	primary lung cancer	Lung	1.04E-03	-2.07E-01	2.17E-01	-2.01E-01
12734	GSM370999	GSE14814	primary lung cancer	Lung	3.86E-03	-1.87E-01	1.73E-01	-2.15E-01
12735	GSM371000	GSE14814	primary lung cancer	Lung	1.60E-01	1.07E-01	1.46E-01	2.25E-01
12736	GSM371001	GSE14814	primary lung cancer	Lung	1.16E-01	1.16E-01	1.32E-02	3.38E-01
12737	GSM371002	GSE14814	primary lung cancer	Lung	1.22E-10	3.78E-01	2.68E-05	5.27E-01
12738	GSM257694	GSE10445	Lung Tumour	Lung	2.73E-07	-3.08E-01	4.43E-02	-2.87E-01
12739	GSM257769	GSE10445	Lung Tumour	Lung	2.96E-01	8.73E-02	2.49E-01	1.91E-01
12740	GSM257772	GSE10445	Lung Tumour	Lung	6.49E-07	2.99E-01	1.02E-05	5.50E-01
12741	GSM257774	GSE10445	Lung Tumour	Lung	6.54E-01	-5.16E-02	4.00E-01	1.55E-01
12742	GSM257775	GSE10445	Lung Tumour	Lung	2.25E-07	3.10E-01	1.63E-04	4.79E-01
12743	GSM257790	GSE10445	Lung Tumour	Lung	3.93E-05	2.52E-01	4.02E-04	4.54E-01
12744	GSM257791	GSE10445	Lung Tumour	Lung	1.18E-02	1.67E-01	7.34E-02	2.62E-01
12745	GSM257792	GSE10445	Lung Tumour	Lung	7.97E-04	2.11E-01	2.49E-03	3.97E-01
12746	GSM257806	GSE10445	Lung Tumour	Lung	6.51E-04	-2.14E-01	1.29E-01	-2.32E-01
12747	GSM257807	GSE10445	Lung Tumour	Lung	7.92E-02	-1.26E-01	1.67E-01	-2.17E-01
12748	GSM257809	GSE10445	Lung Tumour	Lung	1.85E-03	-1.98E-01	3.90E-01	-1.57E-01
12749	GSM257811	GSE10445	Lung Tumour	Lung	3.45E-09	-3.49E-01	2.35E-03	-3.99E-01
12750	GSM257957	GSE10445	Lung Tumour	Lung	1.89E-01	-1.02E-01	2.23E-01	-1.99E-01
12751	GSM257975	GSE10445	Lung Tumour	Lung	9.26E-02	1.22E-01	3.21E-02	3.01E-01
12752	GSM257978	GSE10445	Lung Tumour	Lung	2.52E-02	-1.52E-01	2.18E-02	-3.17E-01
12753	GSM257979	GSE10445	Lung Tumour	Lung	1.08E-10	3.79E-01	2.36E-09	7.23E-01
12754	GSM257980	GSE10445	Lung Tumour	Lung	1.44E-01	-1.10E-01	3.49E-01	-1.67E-01
12755	GSM257995	GSE10445	Lung Tumour	Lung	4.79E-02	-1.38E-01	5.00E-01	-1.35E-01
12756	GSM257997	GSE10445	Lung Tumour	Lung	2.41E-08	3.31E-01	1.25E-04	4.87E-01
12757	GSM258036	GSE10445	Lung Tumour	Lung	1.65E-06	2.89E-01	4.82E-04	4.49E-01
12758	GSM258037	GSE10445	Lung Tumour	Lung	3.86E-09	-3.48E-01	9.89E-04	-4.27E-01
12759	GSM258038	GSE10445	Lung Tumour	Lung	2.01E-01	-1.00E-01	3.94E-01	1.57E-01
12760	GSM258043	GSE10445	Lung Tumour	Lung	2.35E-01	9.52E-02	1.03E-01	2.45E-01
12761	GSM258044	GSE10445	Lung Tumour	Lung	4.79E-02	-1.38E-01	2.23E-01	-1.99E-01
12762	GSM258045	GSE10445	Lung Tumour	Lung	5.46E-02	-1.35E-01	2.83E-01	-1.82E-01
12763	GSM258046	GSE10445	Lung Tumour	Lung	1.44E-01	1.10E-01	6.51E-02	2.68E-01
12764	GSM258047	GSE10445	Lung Tumour	Lung	2.52E-02	-1.52E-01	5.20E-01	1.31E-01
12765	GSM258048	GSE10445	Lung Tumour	Lung	4.62E-13	-4.22E-01	2.75E-03	-3.94E-01
12766	GSM258052	GSE10445	Lung Tumour	Lung	6.89E-14	4.36E-01	4.68E-06	5.69E-01
12767	GSM258054	GSE10445	Lung Tumour	Lung	2.14E-01	-9.82E-02	4.30E-02	2.88E-01
12768	GSM258057	GSE10445	Lung Tumour	Lung	2.38E-03	1.94E-01	1.15E-02	3.43E-01
12769	GSM258058	GSE10445	Lung Tumour	Lung	2.37E-06	2.85E-01	5.15E-04	4.47E-01
12770	GSM258059	GSE10445	Lung Tumour	Lung	1.25E-01	1.14E-01	2.84E-02	3.06E-01
12771	GSM258060	GSE10445	Lung Tumour	Lung	7.11E-10	3.63E-01	5.09E-06	5.67E-01
12772	GSM258061	GSE10445	Lung Tumour	Lung	2.52E-02	1.52E-01	1.07E-01	2.43E-01
12773	GSM258062	GSE10445	Lung Tumour	Lung	9.27E-05	2.41E-01	2.92E-04	4.63E-01
12774	GSM258069	GSE10445	Lung Tumour	Lung	5.04E-01	6.55E-02	5.61E-02	2.75E-01
12775	GSM258071	GSE10445	Lung Tumour	Lung	2.23E-05	-2.59E-01	7.34E-02	-2.62E-01
12776	GSM258072	GSE10445	Lung Tumour	Lung	2.84E-06	-2.83E-01	1.27E-02	-3.39E-01
12777	GSM258074	GSE10445	Lung Tumour	Lung	4.33E-01	7.24E-02	3.84E-01	1.59E-01
12778	GSM258076	GSE10445	Lung Tumour	Lung	2.80E-01	8.93E-02	1.10E-01	2.41E-01
12779	GSM258895	GSE10445	Lung Tumour	Lung	6.30E-05	-2.46E-01	1.57E-01	-2.21E-01
12780	GSM258896	GSE10445	Lung Tumour	Lung	1.12E-01	-1.17E-01	4.73E-01	1.40E-01
12781	GSM258897	GSE10445	Lung Tumour	Lung	3.45E-09	-3.49E-01	9.51E-03	-3.50E-01
12782	GSM258898	GSE10445	Lung Tumour	Lung	2.52E-02	1.52E-01	4.62E-02	2.85E-01
12783	GSM258899	GSE10445	Lung Tumour	Lung	3.93E-05	2.52E-01	1.05E-03	4.25E-01
12784	GSM258900	GSE10445	Lung Tumour	Lung	1.95E-01	-1.01E-01	4.38E-01	1.48E-01
12785	GSM258901	GSE10445	Lung Tumour	Lung	5.75E-06	-2.75E-01	4.24E-02	-2.89E-01
12786	GSM258902	GSE10445	Lung Tumour	Lung	1.08E-01	-1.18E-01	3.52E-01	-1.66E-01
12787	GSM258903	GSE10445	Lung Tumour	Lung	1.55E-10	-3.76E-01	7.83E-03	-3.57E-01
12788	GSM258904	GSE10445	Lung Tumour	Lung	7.30E-02	1.28E-01	7.24E-02	2.63E-01
12789	GSM258905	GSE10445	Lung Tumour	Lung	1.71E-01	1.05E-01	1.53E-01	2.22E-01
12790	GSM259073	GSE10445	Lung Tumour	Lung	1.53E-02	1.62E-01	3.35E-02	2.99E-01
12791	GSM259399	GSE10445	Lung Tumour	Lung	4.60E-05	-2.50E-01	8.35E-02	-2.56E-01
12792	GSM259400	GSE10445	Lung Tumour	Lung	1.01E-02	-1.70E-01	3.18E-01	-1.74E-01
12793	GSM259401	GSE10445	Lung Tumour	Lung	1.35E-05	-2.65E-01	9.51E-03	-3.50E-01
12794	GSM259402	GSE10445	Lung Tumour	Lung	5.91E-07	-3.00E-01	4.75E-02	-2.83E-01
12795	GSM259403	GSE10445	Lung Tumour	Lung	3.31E-12	4.07E-01	7.54E-06	5.57E-01
12796	GSM259405	GSE10445	Lung Tumour	Lung	3.45E-09	-3.49E-01	5.75E-04	-4.43E-01
12797	GSM259410	GSE10445	Lung Tumour	Lung	8.59E-05	2.42E-01	1.60E-03	4.12E-01

12798	GSM259417	GSE10445	Lung Tumour	Lung	5.98E-09	-3.44E-01	2.47E-02	-3.12E-01
12799	GSM259422	GSE10445	Lung Tumour	Lung	1.04E-01	1.19E-01	6.51E-02	2.68E-01
12800	GSM259424	GSE10445	Lung Tumour	Lung	1.67E-07	3.13E-01	1.27E-05	5.45E-01
12801	GSM259425	GSE10445	Lung Tumour	Lung	4.47E-10	-3.67E-01	1.70E-03	-4.10E-01
12802	GSM259427	GSE10445	Lung Tumour	Lung	3.21E-01	8.43E-02	1.73E-01	2.15E-01
12803	GSM260378	GSE10445	Lung Tumour	Lung	4.87E-03	-1.83E-01	5.96E-01	-1.17E-01
12804	GSM260379	GSE10445	Lung Tumour	Lung	4.73E-01	-6.85E-02	3.60E-01	1.64E-01
12805	GSM264313	GSE10445	Lung Tumour	Lung	5.25E-01	6.35E-02	5.58E-01	1.24E-01
12806	GSM264314	GSE10445	Lung Tumour	Lung	7.60E-02	1.27E-01	1.38E-01	2.28E-01
12807	GSM264315	GSE10445	Lung Tumour	Lung	3.19E-02	-1.47E-01	7.53E-02	-2.61E-01
12808	GSM264316	GSE10445	Lung Tumour	Lung	1.00E-01	-1.20E-01	3.90E-01	-1.57E-01
12809	GSM264317	GSE10445	Lung Tumour	Lung	1.26E-04	2.37E-01	8.89E-04	4.30E-01
12810	GSM318073	GSE12667	Lung Tumour	Lung	3.67E-02	-1.44E-01	4.06E-01	1.54E-01
12811	GSM318074	GSE12667	Lung Tumour	Lung	2.77E-02	1.50E-01	4.53E-03	3.77E-01
12812	GSM318075	GSE12667	Lung Tumour	Lung	6.97E-04	-2.13E-01	1.01E-01	-2.46E-01
12813	GSM318076	GSE12667	Lung Tumour	Lung	4.59E-02	1.39E-01	4.97E-03	3.74E-01
12814	GSM318077	GSE12667	Lung Tumour	Lung	7.54E-11	-3.82E-01	1.67E-03	-4.10E-01
12815	GSM318078	GSE12667	Lung Tumour	Lung	8.86E-06	-2.70E-01	1.98E-01	-2.07E-01
12816	GSM318079	GSE12667	Lung Tumour	Lung	6.84E-06	-2.73E-01	1.21E-02	-3.41E-01
12817	GSM318080	GSE12667	Lung Tumour	Lung	1.35E-03	2.03E-01	1.74E-02	3.27E-01
12818	GSM318081	GSE12667	Lung Tumour	Lung	1.25E-01	1.14E-01	6.09E-03	3.67E-01
12819	GSM318082	GSE12667	Lung Tumour	Lung	1.11E-03	2.06E-01	1.88E-03	4.07E-01
12820	GSM318083	GSE12667	Lung Tumour	Lung	1.27E-23	5.74E-01	2.12E-09	7.25E-01
12821	GSM318084	GSE12667	Lung Tumour	Lung	8.30E-08	3.19E-01	1.25E-04	4.87E-01
12822	GSM318085	GSE12667	Lung Tumour	Lung	1.42E-08	3.36E-01	6.25E-05	5.05E-01
12823	GSM318086	GSE12667	Lung Tumour	Lung	3.47E-01	8.13E-02	4.64E-01	1.42E-01
12824	GSM318087	GSE12667	Lung Tumour	Lung	4.83E-01	-6.75E-02	1.35E-01	2.30E-01
12825	GSM318088	GSE12667	Lung Tumour	Lung	1.79E-02	-1.59E-01	1.26E-01	-2.33E-01
12826	GSM318089	GSE12667	Lung Tumour	Lung	4.31E-09	3.47E-01	1.85E-05	5.36E-01
12827	GSM318090	GSE12667	Lung Tumour	Lung	1.65E-01	-1.06E-01	3.12E-01	-1.75E-01
12828	GSM318091	GSE12667	Lung Tumour	Lung	2.14E-01	-9.82E-02	3.60E-01	-1.64E-01
12829	GSM318092	GSE12667	Lung Tumour	Lung	2.91E-02	-1.49E-01	3.75E-01	-1.61E-01
12830	GSM318093	GSE12667	Lung Tumour	Lung	5.89E-01	5.75E-02	9.00E-02	2.52E-01
12831	GSM318094	GSE12667	Lung Tumour	Lung	7.30E-02	-1.28E-01	3.26E-01	-1.72E-01
12832	GSM318095	GSE12667	Lung Tumour	Lung	3.13E-01	-8.53E-02	1.01E-01	2.46E-01
12833	GSM318096	GSE12667	Lung Tumour	Lung	2.64E-01	-9.13E-02	3.58E-01	1.65E-01
12834	GSM318097	GSE12667	Lung Tumour	Lung	3.05E-02	-1.48E-01	2.21E-01	-1.99E-01
12835	GSM318098	GSE12667	Lung Tumour	Lung	2.96E-01	8.73E-02	1.03E-01	2.45E-01
12836	GSM318099	GSE12667	Lung Tumour	Lung	3.38E-01	8.23E-02	2.18E-02	3.17E-01
12837	GSM318100	GSE12667	Lung Tumour	Lung	2.23E-05	2.59E-01	8.71E-03	3.53E-01
12838	GSM318101	GSE12667	Lung Tumour	Lung	2.10E-03	1.96E-01	3.76E-04	4.56E-01
12839	GSM318102	GSE12667	Lung Tumour	Lung	4.63E-01	6.94E-02	1.01E-01	2.46E-01
12840	GSM318103	GSE12667	Lung Tumour	Lung	3.93E-05	-2.52E-01	2.21E-01	-1.99E-01
12841	GSM318104	GSE12667	Lung Tumour	Lung	4.28E-12	-4.05E-01	1.77E-03	-4.09E-01
12842	GSM318105	GSE12667	Lung Tumour	Lung	1.25E-01	-1.14E-01	4.73E-01	1.40E-01
12843	GSM318106	GSE12667	Lung Tumour	Lung	5.78E-01	-5.85E-02	7.34E-02	2.62E-01
12844	GSM318107	GSE12667	Lung Tumour	Lung	3.42E-03	1.88E-01	1.21E-02	3.41E-01
12845	GSM318108	GSE12667	Lung Tumour	Lung	1.25E-02	-1.66E-01	2.49E-01	-1.91E-01
12846	GSM318109	GSE12667	Lung Tumour	Lung	7.96E-05	2.43E-01	3.47E-03	3.86E-01
12847	GSM318110	GSE12667	Lung Tumour	Lung	1.24E-05	-2.66E-01	1.25E-01	-2.34E-01
12848	GSM318111	GSE12667	Lung Tumour	Lung	5.46E-02	-1.35E-01	2.23E-01	1.99E-01
12849	GSM318112	GSE12667	Lung Tumour	Lung	1.79E-02	-1.59E-01	3.12E-01	-1.75E-01
12850	GSM318113	GSE12667	Lung Tumour	Lung	7.45E-04	-2.12E-01	9.58E-02	-2.49E-01
12851	GSM318114	GSE12667	Lung Tumour	Lung	2.07E-02	-1.56E-01	4.70E-01	-1.41E-01
12852	GSM318115	GSE12667	Lung Tumour	Lung	1.03E-08	3.39E-01	8.89E-04	4.30E-01
12853	GSM318116	GSE12667	Lung Tumour	Lung	5.57E-01	6.05E-02	3.60E-01	1.64E-01
12854	GSM318117	GSE12667	Lung Tumour	Lung	1.26E-04	-2.37E-01	9.58E-02	-2.49E-01
12855	GSM318118	GSE12667	Lung Tumour	Lung	4.02E-19	-5.15E-01	4.50E-04	-4.50E-01
12856	GSM318119	GSE12667	Lung Tumour	Lung	1.55E-10	-3.76E-01	2.91E-03	-3.92E-01
12857	GSM318120	GSE12667	Lung Tumour	Lung	4.94E-01	6.65E-02	1.31E-01	2.31E-01
12858	GSM318121	GSE12667	Lung Tumour	Lung	1.44E-03	2.02E-01	4.28E-03	3.79E-01
12859	GSM318122	GSE12667	Lung Tumour	Lung	5.15E-01	6.45E-02	3.94E-01	1.57E-01
12860	GSM318123	GSE12667	Lung Tumour	Lung	2.08E-01	-9.92E-02	3.84E-01	-1.59E-01
12861	GSM318124	GSE12667	Lung Tumour	Lung	5.04E-01	6.55E-02	2.53E-01	1.90E-01
12862	GSM318125	GSE12667	Lung Tumour	Lung	9.67E-15	-4.49E-01	3.06E-04	-4.62E-01
12863	GSM318126	GSE12667	Lung Tumour	Lung	1.21E-01	-1.15E-01	3.75E-01	1.61E-01
12864	GSM318127	GSE12667	Lung Tumour	Lung	3.13E-01	8.53E-02	1.43E-01	2.26E-01
12865	GSM318128	GSE12667	Lung Tumour	Lung	5.23E-02	-1.36E-01	2.68E-01	-1.86E-01
12866	GSM318129	GSE12667	Lung Tumour	Lung	1.89E-05	-2.61E-01	1.37E-01	-2.29E-01
12867	GSM318130	GSE12667	Lung Tumour	Lung	6.86E-01	-4.86E-02	2.76E-01	1.84E-01
12868	GSM318131	GSE12667	Lung Tumour	Lung	3.63E-03	-1.88E-01	3.52E-01	-1.66E-01
12869	GSM318132	GSE12667	Lung Tumour	Lung	9.54E-03	1.71E-01	1.27E-02	3.39E-01
12870	GSM318133	GSE12667	Lung Tumour	Lung	2.67E-08	3.30E-01	4.82E-04	4.49E-01
12871	GSM318134	GSE12667	Lung Tumour	Lung	2.35E-01	9.52E-02	1.48E-01	2.24E-01

12872	GSM318135	GSE12667	Lung Tumour	Lung	2.49E-01	9.33E-02	1.76E-01	2.14E-01
12873	GSM318136	GSE12667	Lung Tumour	Lung	1.31E-02	-1.65E-01	3.84E-01	-1.59E-01
12874	GSM318137	GSE12667	Lung Tumour	Lung	4.79E-02	-1.38E-01	6.96E-01	-9.77E-02
12875	GSM318138	GSE12667	Lung Tumour	Lung	6.03E-13	4.20E-01	3.31E-07	6.27E-01
12876	GSM318139	GSE12667	Lung Tumour	Lung	1.53E-03	2.01E-01	2.71E-02	3.08E-01
12877	GSM318140	GSE12667	Lung Tumour	Lung	3.62E-05	2.53E-01	1.38E-02	3.36E-01
12878	GSM318141	GSE12667	Lung Tumour	Lung	2.96E-01	-8.73E-02	2.91E-01	1.80E-01
12879	GSM318142	GSE12667	Lung Tumour	Lung	2.44E-04	2.28E-01	3.77E-02	2.94E-01
12880	GSM318143	GSE12667	Lung Tumour	Lung	2.80E-10	-3.71E-01	7.04E-03	-3.61E-01
12881	GSM318144	GSE12667	Lung Tumour	Lung	8.59E-05	2.42E-01	3.27E-03	3.88E-01
12882	GSM318145	GSE12667	Lung Tumour	Lung	1.98E-06	-2.87E-01	2.84E-02	-3.06E-01
12883	GSM318146	GSE12667	Lung Tumour	Lung	7.25E-03	-1.76E-01	2.28E-01	-1.97E-01
12884	GSM318147	GSE12667	Lung Tumour	Lung	1.55E-10	3.76E-01	2.68E-05	5.27E-01
12885	GSM686287	GSE27716	Lung Tumour	Lung	1.69E-04	2.33E-01	8.71E-03	3.53E-01
12886	GSM686288	GSE27716	Lung Tumour	Lung	1.25E-01	-1.14E-01	4.77E-01	1.40E-01
12887	GSM686289	GSE27716	Lung Tumour	Lung	1.00E-01	-1.20E-01	3.10E-01	-1.76E-01
12888	GSM686290	GSE27716	Lung Tumour	Lung	1.14E-29	6.46E-01	6.33E-10	7.47E-01
12889	GSM686291	GSE27716	Lung Tumour	Lung	2.64E-01	-9.13E-02	3.52E-01	1.66E-01
12890	GSM686292	GSE27716	Lung Tumour	Lung	6.21E-01	-5.46E-02	4.25E-01	1.50E-01
12891	GSM686293	GSE27716	Lung Tumour	Lung	5.23E-02	1.36E-01	1.94E-01	2.08E-01
12892	GSM686294	GSE27716	Lung Tumour	Lung	1.12E-02	-1.68E-01	5.93E-01	-1.17E-01
12893	GSM686295	GSE27716	Lung Tumour	Lung	5.23E-02	-1.36E-01	3.20E-01	1.73E-01
12894	GSM686296	GSE27716	Lung Tumour	Lung	1.70E-02	-1.60E-01	2.10E-01	-2.03E-01
12895	GSM686297	GSE27716	Lung Tumour	Lung	8.91E-02	-1.23E-01	1.98E-01	2.07E-01
12896	GSM686298	GSE27716	Lung Tumour	Lung	2.91E-02	-1.49E-01	3.12E-01	-1.75E-01
12897	GSM686299	GSE27716	Lung Tumour	Lung	4.60E-03	-1.84E-01	4.06E-01	-1.54E-01
12898	GSM686300	GSE27716	Lung Tumour	Lung	4.33E-01	7.24E-02	2.18E-02	3.17E-01
12899	GSM686301	GSE27716	Lung Tumour	Lung	5.46E-01	6.15E-02	1.03E-01	2.45E-01
12900	GSM686302	GSE27716	Lung Tumour	Lung	3.13E-01	8.53E-02	1.15E-01	2.39E-01
12901	GSM686303	GSE27716	Lung Tumour	Lung	2.14E-01	9.82E-02	4.62E-02	2.85E-01
12902	GSM686304	GSE27716	Lung Tumour	Lung	2.80E-01	-8.93E-02	8.14E-02	2.57E-01
12903	GSM686305	GSE27716	Lung Tumour	Lung	2.10E-03	-1.96E-01	2.23E-01	-1.99E-01
12904	GSM686306	GSE27716	Lung Tumour	Lung	1.04E-01	-1.19E-01	5.65E-01	-1.23E-01
12905	GSM686307	GSE27716	Lung Tumour	Lung	1.49E-01	1.09E-01	1.78E-01	2.13E-01
12906	GSM686308	GSE27716	Lung Tumour	Lung	1.11E-03	-2.06E-01	3.49E-01	-1.67E-01
12907	GSM686309	GSE27716	Lung Tumour	Lung	3.56E-01	8.04E-02	1.31E-01	2.31E-01
12908	GSM686310	GSE27716	Lung Tumour	Lung	4.60E-03	-1.84E-01	1.22E-01	-2.35E-01
12909	GSM686311	GSE27716	Lung Tumour	Lung	9.27E-05	-2.41E-01	1.11E-01	-2.41E-01
12910	GSM686312	GSE27716	Lung Tumour	Lung	1.97E-02	-1.57E-01	2.91E-01	1.80E-01
12911	GSM686313	GSE27716	Lung Tumour	Lung	3.86E-03	-1.87E-01	1.14E-01	-2.39E-01
12912	GSM686314	GSE27716	Lung Tumour	Lung	2.14E-01	-9.82E-02	1.86E-01	2.10E-01
12913	GSM686315	GSE27716	Lung Tumour	Lung	9.11E-04	-2.09E-01	2.21E-01	-1.99E-01
12914	GSM686316	GSE27716	Lung Tumour	Lung	7.92E-02	-1.26E-01	3.60E-01	1.64E-01
12915	GSM686317	GSE27716	Lung Tumour	Lung	3.04E-01	8.63E-02	9.00E-02	2.52E-01
12916	GSM686318	GSE27716	Lung Tumour	Lung	2.29E-02	-1.54E-01	6.45E-01	1.08E-01
12917	GSM686319	GSE27716	Lung Tumour	Lung	4.01E-02	1.42E-01	3.77E-02	2.94E-01
12918	GSM686320	GSE27716	Lung Tumour	Lung	1.12E-02	-1.68E-01	1.11E-01	2.41E-01
12919	GSM686321	GSE27716	Lung Tumour	Lung	3.67E-02	-1.44E-01	6.09E-02	2.71E-01
12920	GSM686322	GSE27716	Lung Tumour	Lung	2.96E-01	-8.73E-02	3.66E-01	1.63E-01
12921	GSM686323	GSE27716	Lung Tumour	Lung	1.46E-02	-1.63E-01	2.83E-01	1.82E-01
12922	GSM686324	GSE27716	Lung Tumour	Lung	6.96E-01	4.76E-02	5.85E-02	2.73E-01
12923	GSM686325	GSE27716	Lung Tumour	Lung	1.39E-01	-1.11E-01	1.73E-01	-2.15E-01
12924	GSM686326	GSE27716	Lung Tumour	Lung	1.46E-02	1.63E-01	1.83E-02	3.25E-01
12925	GSM707333	GSE28582	Non-small cell lung cancer	Lung	1.21E-01	-1.15E-01	1.01E-01	-2.46E-01
12926	GSM707334	GSE28582	Non-small cell lung cancer	Lung	5.16E-03	-1.82E-01	3.43E-01	-1.68E-01
12927	GSM707335	GSE28582	Non-small cell lung cancer	Lung	6.77E-08	3.21E-01	2.09E-06	5.87E-01
12928	GSM707336	GSE28582	Non-small cell lung cancer	Lung	1.97E-03	1.97E-01	7.53E-02	2.61E-01
12929	GSM707337	GSE28582	Non-small cell lung cancer	Lung	1.65E-01	-1.06E-01	1.78E-01	-2.13E-01
12930	GSM707338	GSE28582	Non-small cell lung cancer	Lung	4.59E-02	-1.39E-01	2.76E-01	-1.84E-01
12931	GSM707339	GSE28582	Non-small cell lung cancer	Lung	2.82E-04	-2.26E-01	1.92E-01	-2.09E-01
12932	GSM707340	GSE28582	Non-small cell lung cancer	Lung	4.87E-03	-1.83E-01	3.04E-01	-1.77E-01
12933	GSM707341	GSE28582	Non-small cell lung cancer	Lung	5.68E-01	-5.95E-02	2.23E-01	1.99E-01
12934	GSM707342	GSE28582	Non-small cell lung cancer	Lung	2.29E-02	-1.54E-01	4.18E-01	1.51E-01
12935	GSM707343	GSE28582	Non-small cell lung cancer	Lung	5.68E-04	-2.16E-01	3.43E-01	-1.68E-01
12936	GSM707344	GSE28582	Non-small cell lung cancer	Lung	2.80E-01	-8.93E-02	6.20E-01	1.12E-01
12937	GSM707345	GSE28582	Non-small cell lung cancer	Lung	1.08E-01	-1.18E-01	3.84E-01	-1.59E-01
12938	GSM707346	GSE28582	Non-small cell lung cancer	Lung	5.46E-02	1.35E-01	1.92E-02	3.23E-01
12939	GSM707347	GSE28582	Non-small cell lung cancer	Lung	5.04E-01	-6.55E-02	2.81E-01	1.83E-01
12940	GSM707348	GSE28582	Non-small cell lung cancer	Lung	2.05E-05	-2.60E-01	3.61E-02	-2.96E-01
12941	GSM707349	GSE28582	Non-small cell lung cancer	Lung	1.49E-01	-1.09E-01	4.28E-01	1.50E-01
12942	GSM707350	GSE28582	Non-small cell lung cancer	Lung	9.04E-03	-1.72E-01	1.98E-01	-2.07E-01
12943	GSM707351	GSE28582	Non-small cell lung cancer	Lung	6.97E-04	-2.13E-01	2.88E-01	-1.81E-01
12944	GSM707352	GSE28582	Non-small cell lung cancer	Lung	5.94E-02	1.33E-01	1.67E-01	2.17E-01
12945	GSM707353	GSE28582	Non-small cell lung cancer	Lung	4.63E-01	-6.94E-02	5.38E-01	-1.28E-01

12946	GSM707354	GSE28582	Non-small cell lung cancer	Lung	1.16E-01	1.16E-01	1.74E-02	3.27E-01
12947	GSM707355	GSE28582	Non-small cell lung cancer	Lung	6.46E-02	1.31E-01	5.24E-02	2.79E-01
12948	GSM707356	GSE28582	Non-small cell lung cancer	Lung	4.79E-02	1.38E-01	1.73E-01	2.15E-01
12949	GSM707357	GSE28582	Non-small cell lung cancer	Lung	1.50E-06	-2.90E-01	1.15E-02	-3.43E-01
12950	GSM707358	GSE28582	Non-small cell lung cancer	Lung	3.05E-02	1.48E-01	5.24E-02	2.79E-01
12951	GSM707359	GSE28582	Non-small cell lung cancer	Lung	6.13E-03	-1.79E-01	2.96E-01	-1.79E-01
12952	GSM707360	GSE28582	Non-small cell lung cancer	Lung	3.62E-05	2.53E-01	1.27E-02	3.39E-01
12953	GSM707361	GSE28582	Non-small cell lung cancer	Lung	9.63E-02	-1.21E-01	1.29E-01	2.32E-01
12954	GSM707362	GSE28582	Non-small cell lung cancer	Lung	4.31E-04	2.20E-01	6.79E-03	3.63E-01
12955	GSM707363	GSE28582	Non-small cell lung cancer	Lung	1.34E-01	-1.12E-01	5.58E-01	1.24E-01
12956	GSM707364	GSE28582	Non-small cell lung cancer	Lung	9.04E-03	-1.72E-01	3.43E-01	-1.68E-01
12957	GSM707365	GSE28582	Non-small cell lung cancer	Lung	1.39E-01	1.11E-01	4.96E-02	2.81E-01
12958	GSM707366	GSE28582	Non-small cell lung cancer	Lung	4.53E-01	7.04E-02	2.23E-01	1.99E-01
12959	GSM707367	GSE28582	Non-small cell lung cancer	Lung	7.97E-04	2.11E-01	1.66E-02	3.29E-01
12960	GSM707368	GSE28582	Non-small cell lung cancer	Lung	8.91E-02	-1.23E-01	9.70E-02	2.48E-01
12961	GSM707369	GSE28582	Non-small cell lung cancer	Lung	2.25E-07	3.10E-01	7.83E-07	6.09E-01
12962	GSM707370	GSE28582	Non-small cell lung cancer	Lung	1.83E-01	1.03E-01	3.20E-01	1.73E-01
12963	GSM707371	GSE28582	Non-small cell lung cancer	Lung	6.73E-02	1.30E-01	8.67E-02	2.54E-01
12964	GSM707372	GSE28582	Non-small cell lung cancer	Lung	7.60E-02	-1.27E-01	2.76E-01	-1.84E-01
12965	GSM707373	GSE28582	Non-small cell lung cancer	Lung	1.44E-01	1.10E-01	6.51E-02	2.68E-01
12966	GSM707374	GSE28582	Non-small cell lung cancer	Lung	3.94E-01	7.64E-02	1.53E-01	2.22E-01
12967	GSM707375	GSE28582	Non-small cell lung cancer	Lung	2.57E-01	-9.23E-02	1.31E-01	2.31E-01
12968	GSM707376	GSE28582	Non-small cell lung cancer	Lung	1.26E-03	-2.04E-01	1.71E-01	-2.16E-01
12969	GSM707377	GSE28582	Non-small cell lung cancer	Lung	2.35E-01	9.52E-02	1.65E-01	2.18E-01
12970	GSM707378	GSE28582	Non-small cell lung cancer	Lung	3.54E-10	3.69E-01	1.65E-07	6.41E-01
12971	GSM707379	GSE28582	Non-small cell lung cancer	Lung	4.60E-05	2.50E-01	4.31E-04	4.52E-01
12972	GSM707380	GSE28582	Non-small cell lung cancer	Lung	4.43E-01	7.14E-02	1.18E-01	2.37E-01
12973	GSM707381	GSE28582	Non-small cell lung cancer	Lung	8.97E-13	-4.17E-01	1.05E-03	-4.25E-01
12974	GSM707382	GSE28582	Non-small cell lung cancer	Lung	4.59E-02	1.39E-01	1.62E-01	2.19E-01
12975	GSM707383	GSE28582	Non-small cell lung cancer	Lung	7.46E-06	-2.72E-01	7.43E-03	-3.59E-01
12976	GSM707384	GSE28582	Non-small cell lung cancer	Lung	5.15E-01	-6.45E-02	1.34E-02	3.37E-01
12977	GSM707385	GSE28582	Non-small cell lung cancer	Lung	2.49E-01	-9.33E-02	2.91E-01	1.80E-01
12978	GSM707386	GSE28582	Non-small cell lung cancer	Lung	4.39E-02	-1.40E-01	2.91E-01	-1.80E-01
12979	GSM707387	GSE28582	Non-small cell lung cancer	Lung	3.63E-03	1.88E-01	2.80E-02	3.07E-01
12980	GSM707388	GSE28582	Non-small cell lung cancer	Lung	6.19E-02	1.32E-01	3.21E-02	3.01E-01
12981	GSM707389	GSE28582	Non-small cell lung cancer	Lung	2.28E-01	-9.62E-02	1.38E-01	2.28E-01
12982	GSM707390	GSE28582	Non-small cell lung cancer	Lung	2.40E-02	-1.53E-01	5.20E-01	-1.31E-01
12983	GSM707391	GSE28582	Non-small cell lung cancer	Lung	5.69E-02	1.34E-01	3.94E-02	2.92E-01
12984	GSM707392	GSE28582	Non-small cell lung cancer	Lung	5.69E-02	1.34E-01	6.78E-02	2.66E-01
12985	GSM707393	GSE28582	Non-small cell lung cancer	Lung	2.72E-01	9.03E-02	3.26E-01	1.72E-01
12986	GSM707394	GSE28582	Non-small cell lung cancer	Lung	6.48E-03	1.78E-01	6.69E-02	2.67E-01
12987	GSM707395	GSE28582	Non-small cell lung cancer	Lung	8.10E-03	-1.74E-01	5.82E-01	-1.19E-01
12988	GSM707396	GSE28582	Non-small cell lung cancer	Lung	5.69E-02	-1.34E-01	4.80E-01	-1.39E-01
12989	GSM707397	GSE28582	Non-small cell lung cancer	Lung	1.02E-07	3.17E-01	1.63E-04	4.79E-01
12990	GSM707398	GSE28582	Non-small cell lung cancer	Lung	1.60E-01	-1.07E-01	2.30E-01	1.97E-01
12991	GSM707399	GSE28582	Non-small cell lung cancer	Lung	3.19E-02	1.47E-01	1.26E-01	2.33E-01
12992	GSM707400	GSE28582	Non-small cell lung cancer	Lung	3.50E-02	1.45E-01	4.12E-02	2.90E-01
12993	GSM707401	GSE28582	Non-small cell lung cancer	Lung	8.50E-11	-3.81E-01	2.11E-04	-4.72E-01
12994	GSM707402	GSE28582	Non-small cell lung cancer	Lung	1.01E-02	1.70E-01	4.97E-03	3.74E-01
12995	GSM707403	GSE28582	Non-small cell lung cancer	Lung	7.45E-04	2.12E-01	3.60E-03	3.85E-01
12996	GSM707404	GSE28582	Non-small cell lung cancer	Lung	2.80E-01	-8.93E-02	3.20E-01	1.73E-01
12997	GSM707405	GSE28582	Non-small cell lung cancer	Lung	1.97E-02	1.57E-01	1.48E-01	2.24E-01
12998	GSM707406	GSE28582	Non-small cell lung cancer	Lung	2.62E-05	2.57E-01	1.06E-02	3.46E-01
12999	GSM707407	GSE28582	Non-small cell lung cancer	Lung	2.21E-01	9.72E-02	2.97E-02	3.04E-01
13000	GSM707408	GSE28582	Non-small cell lung cancer	Lung	1.81E-06	-2.88E-01	2.36E-02	-3.14E-01
13001	GSM707409	GSE28582	Non-small cell lung cancer	Lung	1.24E-07	-3.15E-01	2.08E-03	-4.03E-01
13002	GSM707410	GSE28582	Non-small cell lung cancer	Lung	1.08E-01	-1.18E-01	3.04E-01	-1.77E-01
13003	GSM707411	GSE28582	Non-small cell lung cancer	Lung	8.56E-03	-1.73E-01	3.84E-01	-1.59E-01
13004	GSM707412	GSE28582	Non-small cell lung cancer	Lung	2.42E-01	-9.42E-02	2.02E-02	3.21E-01
13005	GSM707413	GSE28582	Non-small cell lung cancer	Lung	1.44E-01	1.10E-01	5.03E-02	2.81E-01
13006	GSM707414	GSE28582	Non-small cell lung cancer	Lung	3.08E-05	2.55E-01	8.33E-04	4.32E-01
13007	GSM707415	GSE28582	Non-small cell lung cancer	Lung	3.10E-06	-2.82E-01	3.67E-02	-2.95E-01
13008	GSM707416	GSE28582	Non-small cell lung cancer	Lung	3.47E-01	-8.13E-02	6.48E-01	1.07E-01
13009	GSM707417	GSE28582	Non-small cell lung cancer	Lung	1.25E-01	-1.14E-01	3.60E-01	1.64E-01
13010	GSM707418	GSE28582	Non-small cell lung cancer	Lung	7.07E-01	4.66E-02	1.53E-01	2.22E-01
13011	GSM707419	GSE28582	Non-small cell lung cancer	Lung	1.61E-02	-1.61E-01	3.87E-01	-1.58E-01
13012	GSM707420	GSE28582	Non-small cell lung cancer	Lung	1.83E-01	-1.03E-01	4.64E-01	-1.42E-01
13013	GSM707421	GSE28582	Non-small cell lung cancer	Lung	1.00E-04	-2.40E-01	1.98E-01	-2.07E-01
13014	GSM707422	GSE28582	Non-small cell lung cancer	Lung	3.75E-01	7.84E-02	4.97E-03	3.74E-01
13015	GSM707423	GSE28582	Non-small cell lung cancer	Lung	4.09E-03	-1.86E-01	1.92E-01	-2.09E-01
13016	GSM707424	GSE28582	Non-small cell lung cancer	Lung	4.13E-01	7.44E-02	4.62E-02	2.85E-01
13017	GSM707425	GSE28582	Non-small cell lung cancer	Lung	1.12E-02	-1.68E-01	1.73E-01	-2.15E-01
13018	GSM707426	GSE28582	Non-small cell lung cancer	Lung	4.01E-02	-1.42E-01	5.00E-01	1.35E-01
13019	GSM707427	GSE28582	Non-small cell lung cancer	Lung	3.34E-02	1.46E-01	5.25E-03	3.72E-01

13020	GSM707428	GSE28582	Non-small cell lung cancer	Lung	3.38E-01	8.23E-02	2.61E-01	1.88E-01
13021	GSM707429	GSE28582	Non-small cell lung cancer	Lung	1.25E-01	-1.14E-01	1.43E-01	2.26E-01
13022	GSM707430	GSE28582	Non-small cell lung cancer	Lung	9.63E-02	-1.21E-01	2.35E-01	-1.95E-01
13023	GSM707431	GSE28582	Non-small cell lung cancer	Lung	4.13E-01	-7.44E-02	4.38E-01	-1.48E-01
13024	GSM707432	GSE28582	Non-small cell lung cancer	Lung	1.14E-06	2.93E-01	6.25E-05	5.05E-01
13025	GSM387757	GSE15460	Gastric	Gastric	1.18E-02	1.67E-01	2.59E-03	3.94E-01
13026	GSM387758	GSE15460	Gastric	Gastric	4.32E-28	-6.28E-01	3.01E-08	-6.72E-01
13027	GSM387759	GSE15460	Gastric	Gastric	1.61E-02	-1.61E-01	4.77E-01	-1.39E-01
13028	GSM387760	GSE15460	Gastric	Gastric	6.16E-18	-4.98E-01	1.12E-05	-5.45E-01
13029	GSM387761	GSE15460	Gastric	Gastric	5.30E-04	2.17E-01	9.56E-04	4.26E-01
13030	GSM387762	GSE15460	Gastric	Gastric	3.54E-28	-6.29E-01	3.11E-07	-6.25E-01
13031	GSM387763	GSE15460	Gastric	Gastric	8.44E-18	-4.96E-01	1.12E-05	-5.45E-01
13032	GSM387764	GSE15460	Gastric	Gastric	6.28E-12	4.02E-01	1.31E-04	4.83E-01
13033	GSM387765	GSE15460	Gastric	Gastric	3.84E-02	-1.43E-01	2.88E-01	-1.80E-01
13034	GSM387766	GSE15460	Gastric	Gastric	1.95E-01	-1.01E-01	1.37E-01	2.28E-01
13035	GSM387767	GSE15460	Gastric	Gastric	1.42E-08	-3.36E-01	1.49E-02	-3.31E-01
13036	GSM387768	GSE15460	Gastric	Gastric	9.27E-05	-2.41E-01	1.51E-01	2.22E-01
13037	GSM387769	GSE15460	Gastric	Gastric	1.53E-03	-2.01E-01	2.56E-01	-1.89E-01
13038	GSM387770	GSE15460	Gastric	Gastric	6.16E-30	-6.49E-01	5.27E-09	-7.05E-01
13039	GSM387771	GSE15460	Gastric	Gastric	1.65E-01	-1.06E-01	3.33E-01	1.70E-01
13040	GSM387772	GSE15460	Gastric	Gastric	4.68E-35	-7.03E-01	9.30E-11	-7.77E-01
13041	GSM387773	GSE15460	Gastric	Gastric	3.27E-18	-5.02E-01	1.77E-04	-4.75E-01
13042	GSM387774	GSE15460	Gastric	Gastric	3.34E-05	-2.54E-01	6.56E-02	-2.67E-01
13043	GSM387775	GSE15460	Gastric	Gastric	5.16E-03	1.82E-01	1.90E-03	4.04E-01
13044	GSM387776	GSE15460	Gastric	Gastric	1.60E-05	-2.63E-01	1.29E-02	-3.37E-01
13045	GSM387777	GSE15460	Gastric	Gastric	1.19E-13	-4.32E-01	1.43E-04	-4.81E-01
13046	GSM387778	GSE15460	Gastric	Gastric	4.47E-10	-3.67E-01	1.89E-02	-3.22E-01
13047	GSM387779	GSE15460	Gastric	Gastric	4.95E-04	-2.18E-01	1.41E-01	-2.26E-01
13048	GSM387780	GSE15460	Gastric	Gastric	2.37E-06	-2.85E-01	1.95E-02	-3.21E-01
13049	GSM387781	GSE15460	Gastric	Gastric	5.91E-07	-3.00E-01	4.51E-02	-2.84E-01
13050	GSM387782	GSE15460	Gastric	Gastric	2.01E-01	1.00E-01	3.20E-02	3.00E-01
13051	GSM387783	GSE15460	Gastric	Gastric	1.65E-01	1.06E-01	3.99E-01	1.55E-01
13052	GSM387784	GSE15460	Gastric	Gastric	9.63E-02	1.21E-01	1.05E-01	2.43E-01
13053	GSM387785	GSE15460	Gastric	Gastric	4.73E-19	-5.14E-01	3.02E-07	-6.26E-01
13054	GSM387786	GSE15460	Gastric	Gastric	5.91E-07	-3.00E-01	2.49E-03	-3.96E-01
13055	GSM387787	GSE15460	Gastric	Gastric	1.85E-07	-3.12E-01	3.38E-03	-3.85E-01
13056	GSM387788	GSE15460	Gastric	Gastric	4.03E-01	-7.54E-02	1.15E-01	2.37E-01
13057	GSM387789	GSE15460	Gastric	Gastric	1.95E-01	1.01E-01	1.38E-01	2.27E-01
13058	GSM387790	GSE15460	Gastric	Gastric	2.40E-39	-7.46E-01	3.77E-13	-8.64E-01
13059	GSM387791	GSE15460	Gastric	Gastric	2.25E-07	-3.10E-01	9.32E-02	-2.49E-01
13060	GSM387792	GSE15460	Gastric	Gastric	4.47E-10	-3.67E-01	1.85E-04	-4.74E-01
13061	GSM387793	GSE15460	Gastric	Gastric	1.14E-29	6.46E-01	4.25E-14	8.97E-01
13062	GSM387794	GSE15460	Gastric	Gastric	6.67E-09	3.43E-01	1.37E-06	5.94E-01
13063	GSM387795	GSE15460	Gastric	Gastric	5.10E-24	5.79E-01	1.41E-10	7.70E-01
13064	GSM387796	GSE15460	Gastric	Gastric	8.03E-38	-7.31E-01	9.30E-11	-7.77E-01
13065	GSM387797	GSE15460	Gastric	Gastric	3.26E-32	-6.74E-01	1.40E-09	-7.30E-01
13066	GSM387798	GSE15460	Gastric	Gastric	7.13E-12	-4.01E-01	1.17E-03	-4.20E-01
13067	GSM387799	GSE15460	Gastric	Gastric	3.32E-07	-3.06E-01	7.55E-02	-2.60E-01
13068	GSM387800	GSE15460	Gastric	Gastric	5.00E-32	-6.72E-01	2.29E-09	-7.21E-01
13069	GSM387801	GSE15460	Gastric	Gastric	1.06E-23	-5.75E-01	9.57E-08	-6.50E-01
13070	GSM387802	GSE15460	Gastric	Gastric	7.07E-16	-4.67E-01	3.34E-04	-4.57E-01
13071	GSM387803	GSE15460	Gastric	Gastric	3.82E-49	-8.35E-01	8.12E-16	-9.52E-01
13072	GSM387804	GSE15460	Gastric	Gastric	1.16E-04	2.38E-01	1.98E-03	4.03E-01
13073	GSM387805	GSE15460	Gastric	Gastric	7.50E-42	-7.70E-01	7.15E-14	-8.89E-01
13074	GSM387806	GSE15460	Gastric	Gastric	1.73E-12	-4.12E-01	2.54E-03	-3.95E-01
13075	GSM387807	GSE15460	Gastric	Gastric	2.10E-27	-6.20E-01	1.45E-09	-7.29E-01
13076	GSM387808	GSE15460	Gastric	Gastric	3.68E-21	-5.43E-01	2.79E-06	-5.78E-01
13077	GSM387809	GSE15460	Gastric	Gastric	3.97E-20	-5.29E-01	2.82E-08	-6.74E-01
13078	GSM387810	GSE15460	Gastric	Gastric	3.44E-14	-4.40E-01	4.07E-04	-4.51E-01
13079	GSM387811	GSE15460	Gastric	Gastric	5.64E-10	-3.65E-01	4.07E-03	-3.79E-01
13080	GSM387812	GSE15460	Gastric	Gastric	1.55E-10	-3.76E-01	2.44E-04	-4.66E-01
13081	GSM387813	GSE15460	Gastric	Gastric	1.57E-38	-7.38E-01	2.16E-11	-8.01E-01
13082	GSM387814	GSE15460	Gastric	Gastric	8.52E-04	2.10E-01	6.51E-03	3.63E-01
13083	GSM387815	GSE15460	Gastric	Gastric	5.81E-29	-6.38E-01	5.70E-10	-7.45E-01
13084	GSM387816	GSE15460	Gastric	Gastric	1.26E-04	2.37E-01	1.20E-03	4.19E-01
13085	GSM387817	GSE15460	Gastric	Gastric	7.72E-22	-5.52E-01	6.40E-07	-6.10E-01
13086	GSM387818	GSE15460	Gastric	Gastric	2.70E-30	-6.53E-01	5.91E-10	-7.45E-01
13087	GSM387819	GSE15460	Gastric	Gastric	3.63E-03	1.88E-01	9.52E-03	3.49E-01
13088	GSM387820	GSE15460	Gastric	Gastric	5.54E-20	-5.27E-01	1.99E-06	-5.85E-01
13089	GSM387821	GSE15460	Gastric	Gastric	7.97E-10	-3.62E-01	5.07E-03	-3.71E-01
13090	GSM387822	GSE15460	Gastric	Gastric	2.49E-16	-4.74E-01	2.11E-05	-5.30E-01
13091	GSM387823	GSE15460	Gastric	Gastric	2.21E-01	9.72E-02	4.58E-01	-1.43E-01
13092	GSM387824	GSE15460	Gastric	Gastric	9.46E-32	6.69E-01	7.15E-14	8.89E-01
13093	GSM387825	GSE15460	Gastric	Gastric	3.86E-09	-3.48E-01	1.26E-02	-3.38E-01

13094	GSM387826	GSE15460	Gastric	Gastric	6.13E-24	5.78E-01	5.46E-11	7.85E-01
13095	GSM387827	GSE15460	Gastric	Gastric	2.25E-07	3.10E-01	4.01E-06	5.70E-01
13096	GSM387828	GSE15460	Gastric	Gastric	1.98E-09	-3.54E-01	1.59E-03	-4.10E-01
13097	GSM387829	GSE15460	Gastric	Gastric	3.63E-03	-1.88E-01	5.43E-01	1.26E-01
13098	GSM387830	GSE15460	Gastric	Gastric	4.20E-02	-1.41E-01	4.29E-01	-1.49E-01
13099	GSM387831	GSE15460	Gastric	Gastric	3.13E-25	-5.94E-01	1.99E-06	-5.85E-01
13100	GSM387832	GSE15460	Gastric	Gastric	6.55E-19	5.12E-01	7.67E-09	6.98E-01
13101	GSM387833	GSE15460	Gastric	Gastric	3.45E-09	-3.49E-01	4.22E-03	-3.78E-01
13102	GSM387834	GSE15460	Gastric	Gastric	2.62E-05	2.57E-01	2.01E-03	4.03E-01
13103	GSM387835	GSE15460	Gastric	Gastric	3.38E-01	-8.23E-02	3.61E-01	1.63E-01
13104	GSM387836	GSE15460	Gastric	Gastric	5.39E-05	-2.48E-01	1.59E-01	-2.19E-01
13105	GSM387837	GSE15460	Gastric	Gastric	4.33E-01	7.24E-02	2.25E-01	1.97E-01
13106	GSM387838	GSE15460	Gastric	Gastric	6.37E-38	7.32E-01	2.53E-16	9.68E-01
13107	GSM387839	GSE15460	Gastric	Gastric	6.13E-24	-5.78E-01	1.40E-07	-6.42E-01
13108	GSM387840	GSE15460	Gastric	Gastric	4.04E-32	6.73E-01	2.40E-14	9.05E-01
13109	GSM387841	GSE15460	Gastric	Gastric	2.52E-17	4.89E-01	1.40E-09	7.30E-01
13110	GSM387842	GSE15460	Gastric	Gastric	7.25E-03	-1.76E-01	2.54E-01	1.89E-01
13111	GSM387843	GSE15460	Gastric	Gastric	4.98E-05	-2.49E-01	8.66E-02	-2.53E-01
13112	GSM387844	GSE15460	Gastric	Gastric	2.76E-09	-3.51E-01	6.62E-03	-3.62E-01
13113	GSM387845	GSE15460	Gastric	Gastric	3.10E-06	-2.82E-01	9.68E-03	-3.48E-01
13114	GSM387846	GSE15460	Gastric	Gastric	1.11E-14	-4.48E-01	1.81E-04	-4.74E-01
13115	GSM387847	GSE15460	Gastric	Gastric	7.65E-32	6.70E-01	7.15E-14	8.89E-01
13116	GSM387848	GSE15460	Gastric	Gastric	6.08E-04	-2.15E-01	4.64E-01	-1.42E-01
13117	GSM387849	GSE15460	Gastric	Gastric	5.78E-01	5.85E-02	1.04E-01	2.43E-01
13118	GSM387850	GSE15460	Gastric	Gastric	2.52E-17	4.89E-01	1.84E-08	6.82E-01
13119	GSM387851	GSE15460	Gastric	Gastric	3.04E-01	8.63E-02	3.61E-01	1.63E-01
13120	GSM387852	GSE15460	Gastric	Gastric	4.94E-01	-6.65E-02	3.96E-01	1.56E-01
13121	GSM387853	GSE15460	Gastric	Gastric	8.26E-27	6.13E-01	1.30E-11	8.09E-01
13122	GSM387854	GSE15460	Gastric	Gastric	9.19E-12	3.99E-01	9.12E-07	6.03E-01
13123	GSM387855	GSE15460	Gastric	Gastric	3.17E-38	-7.35E-01	3.58E-11	-7.92E-01
13124	GSM387856	GSE15460	Gastric	Gastric	4.73E-01	-6.85E-02	3.33E-01	-1.70E-01
13125	GSM387857	GSE15460	Gastric	Gastric	2.46E-19	5.18E-01	1.35E-07	6.43E-01
13126	GSM387858	GSE15460	Gastric	Gastric	3.50E-02	-1.45E-01	1.24E-01	-2.34E-01
13127	GSM387859	GSE15460	Gastric	Gastric	4.00E-38	7.34E-01	4.62E-15	9.28E-01
13128	GSM387860	GSE15460	Gastric	Gastric	1.55E-10	-3.76E-01	3.19E-04	-4.58E-01
13129	GSM387861	GSE15460	Gastric	Gastric	1.01E-25	-6.00E-01	6.54E-08	-6.57E-01
13130	GSM387862	GSE15460	Gastric	Gastric	1.37E-06	2.91E-01	7.75E-03	3.56E-01
13131	GSM387863	GSE15460	Gastric	Gastric	1.42E-34	6.98E-01	4.25E-14	8.97E-01
13132	GSM387864	GSE15460	Gastric	Gastric	2.06E-13	-4.28E-01	7.54E-05	-4.98E-01
13133	GSM387865	GSE15460	Gastric	Gastric	1.89E-05	-2.61E-01	3.13E-03	-3.88E-01
13134	GSM387866	GSE15460	Gastric	Gastric	4.98E-05	2.49E-01	5.16E-03	3.71E-01
13135	GSM387867	GSE15460	Gastric	Gastric	3.96E-14	-4.39E-01	1.77E-04	-4.75E-01
13136	GSM387868	GSE15460	Gastric	Gastric	2.46E-19	5.18E-01	1.40E-09	7.30E-01
13137	GSM387869	GSE15460	Gastric	Gastric	4.23E-01	-7.34E-02	3.19E-01	1.73E-01
13138	GSM387870	GSE15460	Gastric	Gastric	1.05E-05	-2.68E-01	3.92E-02	-2.91E-01
13139	GSM387871	GSE15460	Gastric	Gastric	1.27E-23	-5.74E-01	3.11E-07	-6.25E-01
13140	GSM387872	GSE15460	Gastric	Gastric	2.90E-19	-5.17E-01	4.01E-06	-5.70E-01
13141	GSM387873	GSE15460	Gastric	Gastric	8.66E-21	-5.38E-01	5.74E-06	-5.61E-01
13142	GSM387874	GSE15460	Gastric	Gastric	1.21E-01	-1.15E-01	3.90E-01	-1.57E-01
13143	GSM387875	GSE15460	Gastric	Gastric	6.28E-06	2.74E-01	1.28E-04	4.84E-01
13144	GSM387876	GSE15460	Gastric	Gastric	1.58E-09	3.56E-01	2.05E-05	5.31E-01
13145	GSM387877	GSE15460	Gastric	Gastric	1.12E-02	-1.68E-01	3.01E-01	-1.77E-01
13146	GSM387878	GSE15460	Gastric	Gastric	5.47E-03	-1.81E-01	5.86E-01	-1.18E-01
13147	GSM387879	GSE15460	Gastric	Gastric	5.19E-21	-5.41E-01	1.93E-06	-5.86E-01
13148	GSM387880	GSE15460	Gastric	Gastric	3.49E-04	2.23E-01	9.85E-03	3.47E-01
13149	GSM387881	GSE15460	Gastric	Gastric	4.13E-01	7.44E-02	5.93E-01	1.17E-01
13150	GSM387882	GSE15460	Gastric	Gastric	3.83E-18	-5.01E-01	1.37E-06	-5.94E-01
13151	GSM387883	GSE15460	Gastric	Gastric	8.84E-24	-5.76E-01	4.13E-06	-5.69E-01
13152	GSM387884	GSE15460	Gastric	Gastric	1.40E-29	-6.45E-01	2.91E-08	-6.73E-01
13153	GSM387885	GSE15460	Gastric	Gastric	2.99E-14	-4.41E-01	3.19E-04	-4.58E-01
13154	GSM387886	GSE15460	Gastric	Gastric	9.88E-18	-4.95E-01	3.01E-05	-5.21E-01
13155	GSM387887	GSE15460	Gastric	Gastric	3.39E-06	-2.81E-01	5.16E-03	-3.71E-01
13156	GSM387888	GSE15460	Gastric	Gastric	1.21E-01	1.15E-01	1.21E-01	2.35E-01
13157	GSM387889	GSE15460	Gastric	Gastric	1.04E-06	-2.94E-01	2.20E-02	-3.16E-01
13158	GSM387890	GSE15460	Gastric	Gastric	1.28E-33	-6.88E-01	1.46E-10	-7.69E-01
13159	GSM387891	GSE15460	Gastric	Gastric	3.83E-18	-5.01E-01	2.38E-04	-4.67E-01
13160	GSM387892	GSE15460	Gastric	Gastric	3.15E-10	-3.70E-01	7.76E-04	-4.32E-01
13161	GSM387893	GSE15460	Gastric	Gastric	1.69E-04	-2.33E-01	1.52E-01	-2.22E-01
13162	GSM387894	GSE15460	Gastric	Gastric	2.64E-32	-6.75E-01	1.23E-08	-6.90E-01
13163	GSM387895	GSE15460	Gastric	Gastric	5.26E-18	-4.99E-01	1.37E-04	-4.82E-01
13164	GSM387896	GSE15460	Gastric	Gastric	1.85E-17	-4.91E-01	2.15E-07	-6.33E-01
13165	GSM387897	GSE15460	Gastric	Gastric	3.83E-22	-5.56E-01	9.39E-07	-6.02E-01
13166	GSM387898	GSE15460	Gastric	Gastric	6.03E-13	4.20E-01	2.09E-07	6.34E-01
13167	GSM387899	GSE15460	Gastric	Gastric	1.38E-02	1.64E-01	1.83E-02	3.23E-01

13168	GSM387900	GSE15460	Gastric	Gastric	8.70E-29	-6.36E-01	6.54E-08	-6.57E-01
13169	GSM387901	GSE15460	Gastric	Gastric	4.04E-37	-7.24E-01	1.07E-12	-8.48E-01
13170	GSM387902	GSE15460	Gastric	Gastric	1.38E-10	-3.77E-01	3.32E-03	-3.86E-01
13171	GSM387903	GSE15460	Gastric	Gastric	9.57E-42	-7.69E-01	1.31E-13	-8.80E-01
13172	GSM387904	GSE15460	Gastric	Gastric	2.19E-21	-5.46E-01	4.30E-08	-6.65E-01
13173	GSM387905	GSE15460	Gastric	Gastric	2.18E-02	1.55E-01	4.45E-02	2.85E-01
13174	GSM387906	GSE15460	Gastric	Gastric	3.03E-04	-2.25E-01	1.54E-02	-3.30E-01
13175	GSM387907	GSE15460	Gastric	Gastric	3.80E-33	-6.84E-01	2.28E-10	-7.61E-01
13176	GSM387908	GSE15460	Gastric	Gastric	9.65E-06	-2.69E-01	7.46E-02	-2.60E-01
13177	GSM387909	GSE15460	Gastric	Gastric	2.29E-02	1.54E-01	1.59E-01	2.19E-01
13178	GSM387910	GSE15460	Gastric	Gastric	2.49E-16	-4.74E-01	5.58E-06	-5.62E-01
13179	GSM387911	GSE15460	Gastric	Gastric	1.34E-01	-1.12E-01	5.63E-01	-1.23E-01
13180	GSM387912	GSE15460	Gastric	Gastric	2.46E-19	-5.18E-01	3.90E-06	-5.70E-01
13181	GSM387913	GSE15460	Gastric	Gastric	8.03E-37	-7.21E-01	9.30E-11	-7.77E-01
13182	GSM387914	GSE15460	Gastric	Gastric	2.11E-04	-2.30E-01	6.39E-02	-2.68E-01
13183	GSM387915	GSE15460	Gastric	Gastric	1.88E-02	-1.58E-01	3.90E-01	-1.57E-01
13184	GSM387916	GSE15460	Gastric	Gastric	3.80E-33	-6.84E-01	2.21E-09	-7.21E-01
13185	GSM387917	GSE15460	Gastric	Gastric	8.70E-29	-6.36E-01	1.49E-07	-6.41E-01
13186	GSM387918	GSE15460	Gastric	Gastric	1.50E-19	-5.21E-01	7.53E-06	-5.55E-01
13187	GSM387919	GSE15460	Gastric	Gastric	2.10E-03	-1.96E-01	1.37E-01	-2.28E-01
13188	GSM387920	GSE15460	Gastric	Gastric	1.73E-27	-6.21E-01	1.45E-09	-7.29E-01
13189	GSM387921	GSE15460	Gastric	Gastric	4.72E-15	-4.54E-01	5.74E-06	-5.61E-01
13190	GSM387922	GSE15460	Gastric	Gastric	5.81E-29	-6.38E-01	2.21E-09	-7.21E-01
13191	GSM387923	GSE15460	Gastric	Gastric	2.79E-18	-5.03E-01	6.59E-07	-6.10E-01
13192	GSM387924	GSE15460	Gastric	Gastric	6.10E-16	-4.68E-01	1.37E-04	-4.82E-01
13193	GSM387925	GSE15460	Gastric	Gastric	8.56E-03	-1.73E-01	1.80E-01	-2.11E-01
13194	GSM387926	GSE15460	Gastric	Gastric	2.94E-24	-5.82E-01	1.96E-08	-6.81E-01
13195	GSM387927	GSE15460	Gastric	Gastric	4.95E-04	-2.18E-01	7.94E-02	-2.57E-01
13196	GSM387928	GSE15460	Gastric	Gastric	1.33E-12	-4.14E-01	1.17E-03	-4.20E-01
13197	GSM387929	GSE15460	Gastric	Gastric	2.07E-02	1.56E-01	4.57E-02	2.84E-01
13198	GSM387930	GSE15460	Gastric	Gastric	1.48E-26	-6.10E-01	3.60E-09	-7.12E-01
13199	GSM387931	GSE15460	Gastric	Gastric	7.79E-39	7.41E-01	1.41E-14	9.12E-01
13200	GSM387932	GSE15460	Gastric	Gastric	4.57E-22	-5.55E-01	9.88E-08	-6.49E-01
13201	GSM387933	GSE15460	Gastric	Gastric	8.59E-05	-2.42E-01	1.22E-02	-3.39E-01
13202	GSM387934	GSE15460	Gastric	Gastric	2.91E-12	-4.08E-01	1.98E-03	-4.03E-01
13203	GSM387935	GSE15460	Gastric	Gastric	4.56E-25	-5.92E-01	2.91E-08	-6.73E-01
13204	GSM387936	GSE15460	Gastric	Gastric	1.59E-36	7.18E-01	4.25E-14	8.97E-01
13205	GSM387937	GSE15460	Gastric	Gastric	1.35E-04	2.36E-01	7.36E-05	4.98E-01
13206	GSM387938	GSE15460	Gastric	Gastric	4.56E-25	-5.92E-01	9.66E-07	-6.01E-01
13207	GSM387939	GSE15460	Gastric	Gastric	9.74E-36	-7.10E-01	1.40E-09	-7.30E-01
13208	GSM387940	GSE15460	Gastric	Gastric	2.80E-01	8.93E-02	3.25E-02	2.99E-01
13209	GSM387941	GSE15460	Gastric	Gastric	1.10E-21	-5.50E-01	1.45E-06	-5.92E-01
13210	GSM387942	GSE15460	Gastric	Gastric	4.63E-01	-6.94E-02	4.36E-01	1.47E-01
13211	GSM387943	GSE15460	Gastric	Gastric	9.27E-05	2.41E-01	1.01E-04	4.90E-01
13212	GSM387944	GSE15460	Gastric	Gastric	4.04E-32	-6.73E-01	2.28E-10	-7.61E-01
13213	GSM387945	GSE15460	Gastric	Gastric	6.49E-07	-2.99E-01	6.28E-03	-3.64E-01
13214	GSM387946	GSE15460	Gastric	Gastric	6.73E-02	-1.30E-01	5.09E-01	-1.33E-01
13215	GSM387947	GSE15460	Gastric	Gastric	5.46E-02	-1.35E-01	5.06E-01	-1.33E-01
13216	GSM387948	GSE15460	Gastric	Gastric	6.35E-17	-4.83E-01	2.09E-07	-6.34E-01
13217	GSM387949	GSE15460	Gastric	Gastric	1.52E-44	-7.95E-01	6.23E-13	-8.57E-01
13218	GSM387950	GSE15460	Gastric	Gastric	1.29E-14	4.47E-01	3.02E-07	6.26E-01
13219	GSM387951	GSE15460	Gastric	Gastric	3.95E-36	-7.14E-01	3.58E-11	-7.92E-01
13220	GSM387952	GSE15460	Gastric	Gastric	9.46E-32	-6.69E-01	3.68E-10	-7.53E-01
13221	GSM387953	GSE15460	Gastric	Gastric	2.84E-06	-2.83E-01	1.01E-01	-2.44E-01
13222	GSM387954	GSE15460	Gastric	Gastric	3.74E-04	-2.22E-01	5.68E-02	-2.74E-01
13223	GSM387955	GSE15460	Gastric	Gastric	2.48E-07	-3.09E-01	1.56E-03	-4.11E-01
13224	GSM387956	GSE15460	Gastric	Gastric	9.19E-12	-3.99E-01	1.85E-04	-4.74E-01
13225	GSM387957	GSE15460	Gastric	Gastric	3.65E-08	-3.27E-01	1.86E-02	-3.23E-01
13226	GSM387958	GSE15460	Gastric	Gastric	6.16E-18	-4.98E-01	6.75E-08	-6.57E-01
13227	GSM387959	GSE15460	Gastric	Gastric	5.16E-03	1.82E-01	2.14E-02	3.17E-01
13228	GSM387960	GSE15460	Gastric	Gastric	1.59E-33	6.88E-01	1.25E-13	8.81E-01
13229	GSM387961	GSE15460	Gastric	Gastric	2.72E-01	-9.03E-02	2.54E-01	1.89E-01
13230	GSM387962	GSE15460	Gastric	Gastric	1.35E-03	-2.03E-01	3.27E-01	-1.71E-01
13231	GSM387963	GSE15460	Gastric	Gastric	9.19E-12	-3.99E-01	4.25E-04	-4.50E-01
13232	GSM387964	GSE15460	Gastric	Gastric	2.52E-17	-4.89E-01	3.88E-05	-5.15E-01
13233	GSM387965	GSE15460	Gastric	Gastric	7.13E-12	-4.01E-01	7.54E-05	-4.98E-01
13234	GSM387966	GSE15460	Gastric	Gastric	5.34E-44	-7.90E-01	1.07E-12	-8.48E-01
13235	GSM387967	GSE15460	Gastric	Gastric	2.18E-02	1.55E-01	3.70E-02	2.93E-01
13236	GSM387968	GSE15460	Gastric	Gastric	2.94E-24	-5.82E-01	9.57E-08	-6.50E-01
13237	GSM387969	GSE15460	Gastric	Gastric	3.32E-07	3.06E-01	1.10E-05	5.46E-01
13238	GSM387970	GSE15460	Gastric	Gastric	5.27E-06	-2.76E-01	2.81E-02	-3.05E-01
13239	GSM387971	GSE15460	Gastric	Gastric	3.87E-26	-6.05E-01	2.29E-09	-7.21E-01
13240	GSM387972	GSE15460	Gastric	Gastric	1.46E-02	-1.63E-01	1.86E-01	-2.10E-01
13241	GSM387973	GSE15460	Gastric	Gastric	1.38E-10	-3.77E-01	6.28E-03	-3.64E-01

13242	GSM387974	GSE15460	Gastric	Gastric	8.61E-07	-2.96E-01	1.51E-02	-3.31E-01
13243	GSM387975	GSE15460	Gastric	Gastric	4.28E-12	-4.05E-01	1.40E-04	-4.81E-01
13244	GSM387976	GSE15460	Gastric	Gastric	2.59E-06	-2.84E-01	1.26E-02	-3.38E-01
13245	GSM387977	GSE15460	Gastric	Gastric	1.25E-18	-5.08E-01	7.74E-06	-5.54E-01
13246	GSM387978	GSE15460	Gastric	Gastric	5.02E-10	3.66E-01	4.08E-05	5.14E-01
13247	GSM387979	GSE15460	Gastric	Gastric	1.34E-11	-3.96E-01	9.56E-04	-4.26E-01
13248	GSM387980	GSE15460	Gastric	Gastric	2.10E-03	-1.96E-01	3.38E-01	-1.68E-01
13249	GSM387981	GSE15460	Gastric	Gastric	3.09E-13	-4.25E-01	5.64E-04	-4.42E-01
13250	GSM387982	GSE15460	Gastric	Gastric	2.65E-15	-4.58E-01	3.26E-04	-4.58E-01
13251	GSM387983	GSE15460	Gastric	Gastric	3.84E-01	-7.74E-02	7.58E-01	-8.51E-02
13252	GSM387984	GSE15460	Gastric	Gastric	3.79E-27	-6.17E-01	5.27E-09	-7.05E-01
13253	GSM387985	GSE15460	Gastric	Gastric	1.94E-28	-6.32E-01	1.90E-08	-6.81E-01
13254	GSM387986	GSE15460	Gastric	Gastric	7.07E-16	-4.67E-01	5.74E-06	-5.61E-01
13255	GSM387987	GSE15460	Gastric	Gastric	4.73E-19	-5.14E-01	9.39E-07	-6.02E-01

Table S4B. EMT score (generic cell line EMT signature) of Broad-Novartis cell line data (Cancer Cell Line Encyclopedia; CCLE)

Index	CCLE name	Cell line primary name	Site Primary	Histology	Hist Subtype1	Generic.EMT.CL.pv	Generic.EMT.CL.Ksscore	EGFR.Mutation_Amino Acid/Type
1	DMS53_LUNG	DMS 53	lung	carcinoma	small_cell_carcinoma	1.54E-03	-2.86E-01	WT
2	SW1116_LARGE_INTESTINE	SW1116	large_intestine	carcinoma	adenocarcinoma	3.56E-13	-6.01E-01	MUT_Intron
3	NCIH1694_LUNG	NCI-H1694	lung	carcinoma	small_cell_carcinoma	1.84E-07	4.42E-01	WT
4	P3HR1_HAEMATOPOIETIC_AND_LYMPHOID_TISSUE	P3HR-1	haematopoietic_&_lymphoid_tissue	lymphoid_neoplasm	Burkitt_lymphoma	1.08E-02	2.39E-01	WT
5	HUT78_HAEMATOPOIETIC_AND_LYMPHOID_TISSUE	HuT 78	haematopoietic_&_lymphoid_tissue	lymphoid_neoplasm	mycosisfungoides-Sezary_syndrome	1.36E-04	3.35E-01	WT
6	UMUC3_URINARY_TRACT	UM-UC-3	urinary_tract	carcinoma	transitional_cell_carcinoma	2.83E-19	7.34E-01	WT
7	HOS_BONE	HOS	bone	osteosarcoma	---	3.93E-18	7.11E-01	WT
8	HUNS1_HAEMATOPOIETIC_AND_LYMPHOID_TISSUE	HuNS1	haematopoietic_&_lymphoid_tissue	lymphoid_neoplasm	plasma_cell_myeloma	4.10E-02	2.01E-01	WT
9	AML193_HAEMATOPOIETIC_AND_LYMPHOID_TISSUE	AML-193	haematopoietic_&_lymphoid_tissue	haematopoietic_neoplasm	acute_myeloid_leukaemia	7.78E-02	1.79E-01	WT
10	RVH421_SKIN	RVH-421	skin	malignant_melanoma	---	8.25E-15	6.39E-01	WT
11	NCIH1184_LUNG	NCI-H1184	lung	carcinoma	small_cell_carcinoma	9.23E-09	4.83E-01	WT
12	HCC2157_BREAST	HCC2157	breast	carcinoma	ductal_carcinoma	1.80E-03	-2.82E-01	WT
13	TC71_BONE	TC-71	bone	Ewings_sarcoma-peripheral_primitive_neuro_ectodermal_tumour	---	1.51E-10	5.34E-01	WT
14	NCIH2227_LUNG	NCI-H2227	lung	carcinoma	small_cell_carcinoma	2.93E-08	4.68E-01	WT
15	COLO205_LARGE_INTESTINE	COLO 205	large_intestine	carcinoma	adenocarcinoma	1.71E-13	-6.09E-01	WT
16	SNU449_LIVER	SNU-449	liver	carcinoma	hepato_cellular_carcinoma	7.29E-11	5.43E-01	WT
17	NCIH28_PLEURA	NCI-H28	pleura	mesothelioma	---	1.84E-07	4.42E-01	WT
18	OV56_OVARY	OV56	ovary	carcinoma	---	6.67E-10	5.16E-01	WT
19	JHOS4_OVARY	JHOS-4	ovary	carcinoma	adenocarcinoma	2.52E-03	-2.75E-01	WT
20	KYSE450_OESOPHAGUS	KYSE-450	oesophagus	carcinoma	squamous_cell_carcinoma	5.70E-01	8.42E-02	MUT_p.S768I/Missense
21	RMUGS_OVARY	RMUG-S	ovary	carcinoma	adenocarcinoma	5.10E-06	-3.92E-01	WT
22	KLE_ENDOMETRIUM	KLE	endometrium	carcinoma	adenocarcinoma	4.24E-04	3.13E-01	WT
23	HS895T_SKIN	Hs 895.T	skin	malignant_melanoma	---	2.33E-22	7.93E-01	WT
24	LN229_CENTRAL_NERVOUS_SYSTEM	LN-229	central_nervous_system	glioma	astrocytoma_GradeIV	6.63E-17	6.85E-01	WT
25	P31FUJ_HAEMATOPOIETIC_AND_LYMPHOID_TISSUE	P31/FUJ	haematopoietic_&_lymphoid_tissue	haematopoietic_neoplasm	acute_myeloid_leukaemia	6.58E-03	2.52E-01	WT
26	RKN_OVARY	RKN	ovary	leiomyo_sarcoma	---	2.83E-19	7.34E-01	WT
27	PATU8988S_PANCREAS	PA-TU-8988S	pancreas	carcinoma	ductal_carcinoma	2.97E-14	-6.27E-01	WT
28	NH6_AUTONOMIC_GANGLIA	NH-6	autonomic_ganglia	neuroblastoma	---	3.13E-19	7.33E-01	WT
29	SF126_CENTRAL_NERVOUS_SYSTEM	SF126	central_nervous_system	glioma	astrocytoma	2.15E-21	7.75E-01	WT
30	RERFLCAD2_LUNG	RERF-LC-Ad2	lung	carcinoma	adenocarcinoma	4.33E-02	-1.99E-01	WT
31	OUMS23_LARGE_INTESTINE	OUMS-23	large_intestine	carcinoma	adenocarcinoma	8.77E-02	-1.75E-01	WT
32	SNGM_ENDOMETRIUM	SNG-M	endometrium	carcinoma	adenocarcinoma	4.56E-01	9.95E-02	WT
33	OUMS27_BONE	OUMS-27	bone	chondrosarcoma	---	1.25E-20	7.60E-01	WT
34	NCIH2347_LUNG	NCI-H2347	lung	carcinoma	adenocarcinoma	1.12E-02	-2.38E-01	WT
35	SW1990_PANCREAS	SW 1990	pancreas	carcinoma	ductal_carcinoma	7.90E-07	4.21E-01	WT
36	HS940T_SKIN	Hs 940.T	skin	malignant_melanoma	---	1.12E-17	7.02E-01	WT
37	HS611T_HAEMATOPOIETIC_AND_LYMPHOID_TISSUE	Hs 611.T	haematopoietic_&_lymphoid_tissue	lymphoid_neoplasm	Hodgkin_lymphoma	1.84E-05	3.71E-01	WT
38	TOLEDO_HAEMATOPOIETIC_AND_LYMPHOID_TISSUE	Toledo	haematopoietic_&_lymphoid_tissue	lymphoid_neoplasm	diffuse_large_B-cell_lymphoma	3.76E-03	2.65E-01	WT
39	RERFGC1B_STOMACH	RERF-GC-1B	stomach	carcinoma	---	2.10E-01	-1.40E-01	WT
40	HT1080_SOFT_TISSUE	HT-1080	soft_tissue	fibrosarcoma	---	1.82E-16	6.76E-01	WT
41	NCIH2087_LUNG	NCI-H2087	lung	carcinoma	adenocarcinoma	2.47E-02	-2.16E-01	WT
42	COV318_OVARY	COV318	ovary	carcinoma	---	6.66E-07	4.23E-01	WT
43	NCIH2085_LUNG	NCI-H2085	lung	carcinoma	adenocarcinoma	3.02E-02	2.10E-01	WT
44	NCIH510_LUNG	NCI-H510	lung	carcinoma	small_cell_carcinoma	5.00E-01	-9.35E-02	WT
45	CAKI1_KIDNEY	Caki-1	kidney	carcinoma	clear_cell_renal_cell_carcinoma	8.33E-12	5.67E-01	WT
46	NCIH716_LARGE_INTESTINE	NCI-H716	large_intestine	carcinoma	adenocarcinoma	6.99E-06	3.87E-01	WT
47	NCIH2066_LUNG	NCI-H2066	lung	carcinoma	small_cell_carcinoma	6.36E-03	2.53E-01	WT
48	NCIH1341_LUNG	NCI-H1341	lung	carcinoma	small_cell_carcinoma	1.00E-05	3.81E-01	WT
49	NCIH2029_LUNG	NCI-H2029	lung	carcinoma	small_cell_carcinoma	2.27E-02	-2.19E-01	WT

50	C3A_LIVER	C3A	liver	carcinoma	hepato_cellular_carcinoma	4.50E-01	-1.00E-01	WT
51	SW480_LARGE_INTESTINE	SW480	large_intestine	carcinoma	adenocarcinoma	1.73E-02	2.26E-01	WT
52	HS746T_STOMACH	Hs 746T	stomach	carcinoma	---	6.37E-15	6.42E-01	WT
53	LU65_LUNG	LU65	lung	carcinoma	non-small_cell_carcinoma	2.02E-01	1.42E-01	WT
54	HS739T_BREAST	Hs 739.T	breast	carcinoma	---	1.57E-21	7.77E-01	WT
55	SNUC2A_LARGE_INTESTINE	SNU-C2A	large_intestine	carcinoma	adenocarcinoma	5.00E-07	-4.28E-01	MUT_p.R165Q/Missense
56	HEC151_ENDOMETRIUM	HEC-151	endometrium	carcinoma	adenocarcinoma	1.56E-01	1.53E-01	WT
57	MSTO211H_PLEURA	MSTO-211H	pleura	mesothelioma	---	7.75E-20	7.45E-01	WT
58	RAJI_HAEMATOPOIETIC_AND_LYMPHOID_TISSUE	Raji	haematopoietic_&_lymphoid_tissue	lymphoid_neoplasm	Burkitt_lymphoma	1.48E-02	2.30E-01	WT
59	SKMEL1_SKIN	SK-MEL-1	skin	malignant_melanoma	---	2.13E-08	4.72E-01	WT
60	SUPT1_HAEMATOPOIETIC_AND_LYMPHOID_TISSUE	SUP-T1	haematopoietic_&_lymphoid_tissue	lymphoid_neoplasm	acute_lymphoblastic_T-cell_leukaemia	5.37E-06	3.91E-01	WT
61	JHH7_LIVER	JHH-7	liver	carcinoma	hepato_cellular_carcinoma	2.10E-01	1.40E-01	WT
62	HUT102_HAEMATOPOIETIC_AND_LYMPHOID_TISSUE	HuT 102	haematopoietic_&_lymphoid_tissue	lymphoid_neoplasm	mycosisfungoides-Sezary_syndrome	4.67E-05	3.55E-01	WT
63	5637_URINARY_TRACT	5637	urinary_tract	carcinoma	---	4.10E-02	2.01E-01	WT
64	HCC38_BREAST	HCC38	breast	carcinoma	ductal_carcinoma	2.18E-01	1.39E-01	WT
65	DU4475_BREAST	DU4475	breast	carcinoma	---	3.36E-01	-1.17E-01	WT
66	NCIH1573_LUNG	NCI-H1573	lung	carcinoma	adenocarcinoma	9.23E-09	-4.83E-01	WT
67	NCIH508_LARGE_INTESTINE	NCI-H508	large_intestine	carcinoma	adenocarcinoma	1.97E-17	-6.96E-01	WT
68	NCIH1651_LUNG	NCI-H1651	lung	carcinoma	adenocarcinoma	2.20E-07	4.40E-01	WT
69	SKUT1_SOFT_TISSUE	SK-UT-1	soft_tissue	leiomyo_sarcoma	---	1.20E-15	6.58E-01	WT
70	MDAMB175VII_BREAST	MDA-MB-175-VII	breast	carcinoma	ductal_carcinoma	2.21E-18	-7.16E-01	WT
71	SUPB15_HAEMATOPOIETIC_AND_LYMPHOID_TISSUE	SUP-B15	haematopoietic_&_lymphoid_tissue	lymphoid_neoplasm	acute_lymphoblastic_B-cell_leukaemia	1.24E-04	3.37E-01	WT
72	BECKER_CENTRAL_NERVOUS_SYSTEM	Becker	central_nervous_system	glioma	astrocytoma_GradeIV	1.82E-16	6.76E-01	WT
73	NCIH226_LUNG	NCI-H226	lung	carcinoma	squamous_cell_carcinoma	2.59E-11	5.54E-01	WT
74	NCIH1666_LUNG	NCI-H1666	lung	carcinoma	bronchiolo_alveolar_adenocarcinoma	5.02E-04	-3.10E-01	WT
75	SW1783_CENTRAL_NERVOUS_SYSTEM	SW 1783	central_nervous_system	glioma	astrocytoma_GradeIII	1.28E-19	7.41E-01	WT
76	ISTMES1_PLEURA	IST-MES1	pleura	mesothelioma	---	2.57E-13	6.05E-01	WT
77	NCIH1734_LUNG	NCI-H1734	lung	carcinoma	adenocarcinoma	4.99E-03	2.59E-01	WT
78	NCIH2342_LUNG	NCI-H2342	lung	carcinoma	adenocarcinoma	1.17E-06	-4.15E-01	WT
79	EBC1_LUNG	EBC-1	lung	carcinoma	squamous_cell_carcinoma	5.06E-01	9.27E-02	WT
80	YMB1_BREAST	YMB-1	breast	carcinoma	---	4.16E-12	-5.75E-01	WT
81	HS936T_SKIN	Hs 936.T	skin	malignant_melanoma	---	7.66E-10	5.14E-01	WT
82	T173_BONE	T1-73	bone	osteosarcoma	---	9.16E-21	7.63E-01	WT
83	NCIH322_LUNG	NCI-H322	lung	carcinoma	adenocarcinoma	3.17E-05	-3.61E-01	WT
84	HS618T_LUNG	Hs 618.T	lung	carcinoma	adenocarcinoma	4.91E-22	7.87E-01	WT
85	HS870T_BONE	Hs 870.T	bone	osteosarcoma	---	1.73E-23	8.13E-01	WT
86	HS706T_BONE	Hs 706.T	bone	giant_cell_tumour	---	2.15E-21	7.75E-01	WT
87	PANC1_PANCREAS	PANC-1	pancreas	carcinoma	ductal_carcinoma	5.82E-08	4.58E-01	WT
88	NCIH2291_LUNG	NCI-H2291	lung	carcinoma	adenocarcinoma	6.23E-02	-1.87E-01	MUT_p.V592F/Missense
89	T47D_BREAST	T-47D	breast	carcinoma	ductal_carcinoma	1.17E-05	-3.78E-01	WT
90	MDAMB231_BREAST	MDA-MB-231	breast	carcinoma	---	6.67E-10	5.16E-01	WT
91	OV90_OVARY	OV-90	ovary	carcinoma	mixed_carcinoma	1.87E-01	1.45E-01	WT
92	RS411_HAEMATOPOIETIC_AND_LYMPHOID_TISSUE	RS4;11	haematopoietic_&_lymphoid_tissue	lymphoid_neoplasm	acute_lymphoblastic_B-cell_leukaemia	1.48E-02	2.30E-01	MUT_p.L815F/Missense
93	OVTOKO_OVARY	OVTOKO	ovary	carcinoma	clear_cell_carcinoma	1.74E-07	4.43E-01	WT
94	OVCAR4_OVARY	OVCAR-4	ovary	carcinoma	---	6.08E-02	1.88E-01	WT
95	HLF_LIVER	HLF	liver	carcinoma	hepato_cellular_carcinoma	1.23E-17	7.01E-01	WT
96	HCT15_LARGE_INTESTINE	HCT-15	large_intestine	carcinoma	adenocarcinoma	4.67E-05	-3.55E-01	WT
97	SW1463_LARGE_INTESTINE	SW1463	large_intestine	carcinoma	adenocarcinoma	5.90E-16	-6.65E-01	WT
98	SF295_CENTRAL_NERVOUS_SYSTEM	SF-295	central_nervous_system	glioma	astrocytoma_GradeIV	8.44E-18	7.04E-01	WT
99	WM115_SKIN	WM-115	skin	malignant_melanoma	---	1.26E-16	6.79E-01	WT
100	JHH4_LIVER	JHH-4	liver	carcinoma	hepato_cellular_carcinoma	2.34E-07	4.39E-01	WT
101	OE33_OESOPHAGUS	OE33	oesophagus	other	metaplasia	3.32E-08	-4.66E-01	WT

102 CCK81_LARGE_INTESTINE	CCK-81	large_intestine	carcinoma	adenocarcinoma	9.35E-13	-5.91E-01	MUT_p.Y1069C/Missense
103 JHH1_LIVER	JHH-1	liver	carcinoma	hepato_cellular_carcinoma	7.24E-02	-1.82E-01	WT
104 SJSA1_BONE	SJSA-1	bone	osteosarcoma	---	5.65E-19	7.28E-01	WT
105 NCIH2228_LUNG	NCI-H2228	lung	carcinoma	adenocarcinoma	4.04E-05	3.57E-01	WT
106 RERFLCAI_LUNG	RERF-LC-AI	lung	carcinoma	squamous_cell_carcinoma	7.05E-16	6.63E-01	WT
107 SKMES1_LUNG	SK-MES-1	lung	carcinoma	squamous_cell_carcinoma	9.91E-07	4.18E-01	WT
108 NCIH520_LUNG	NCI-H520	lung	carcinoma	squamous_cell_carcinoma	2.99E-01	1.23E-01	WT
109 JHH5_LIVER	JHH-5	liver	carcinoma	hepato_cellular_carcinoma	1.53E-01	-1.54E-01	WT
110 FU97_STOMACH	FU97	stomach	carcinoma	diffuse_adenocarcinoma	5.07E-02	1.94E-01	WT
111 COLO800_SKIN	COLO-800	skin	malignant_melanoma	---	3.56E-12	5.77E-01	WT
112 NCIH1963_LUNG	NCI-H1963	lung	carcinoma	small_cell_carcinoma	2.34E-03	2.76E-01	WT
113 RL952_ENDOMETRIUM	RL95-2	endometrium	carcinoma	mixed_adeno-squamous_carcinoma	3.15E-04	-3.19E-01	WT
114 J82_URINARY_TRACT	J82	urinary_tract	carcinoma	transitional_cell_carcinoma	5.35E-15	6.44E-01	WT
115 HCC15_LUNG	HCC-15	lung	carcinoma	squamous_cell_carcinoma	4.50E-03	2.61E-01	WT
116 M059K_CENTRAL_NERVOUS_SYSTEM	M059K	central_nervous_system	glioma	astrocytoma_GradeIV	6.07E-22	7.85E-01	WT
117 CALU6_LUNG	Calu-6	lung	carcinoma	undifferentiated_carcinoma	5.82E-14	6.20E-01	WT
118 SKLU1_LUNG	SK-LU-1	lung	carcinoma	adenocarcinoma	2.41E-12	5.81E-01	WT
119 SW900_LUNG	SW 900	lung	carcinoma	squamous_cell_carcinoma	2.43E-01	1.34E-01	WT
120 UACC62_SKIN	UACC-62	skin	malignant_melanoma	---	3.52E-14	6.25E-01	WT
121 MFE280_ENDOMETRIUM	MFE-280	endometrium	carcinoma	adenocarcinoma	2.27E-02	2.19E-01	WT
122 MFE319_ENDOMETRIUM	MFE-319	endometrium	carcinoma	adenocarcinoma	4.62E-01	9.86E-02	MUT_Intron
123 MFE296_ENDOMETRIUM	MFE-296	endometrium	carcinoma	adenocarcinoma	4.69E-11	5.48E-01	WT
124 GOS3_CENTRAL_NERVOUS_SYSTEM	GOS-3	central_nervous_system	glioma	astrocytoma	1.49E-17	6.99E-01	MUT_p.D256G/Missense
125 GI1_CENTRAL_NERVOUS_SYSTEM	GI-1	central_nervous_system	glioma	gliosarcoma	1.41E-21	7.78E-01	WT
126 PC14_LUNG	PC-14	lung	carcinoma	non-small_cell_carcinoma	3.38E-03	-2.68E-01	MUT_p.ELREA746del/In_Frame_ Deletion
127 KYSE410_OESOPHAGUS	KYSE-410	oesophagus	carcinoma	squamous_cell_carcinoma	1.27E-02	-2.35E-01	WT
128 A204_SOFT_TISSUE	A-204	soft_tissue	rhabdomyosarcoma	---	2.66E-15	6.51E-01	WT
129 PATU8902_PANCREAS	PA-TU-8902	pancreas	carcinoma	ductal_carcinoma	4.12E-06	-3.95E-01	WT
130 NCIH810_LUNG	NCI-H810	lung	carcinoma	large_cell_carcinoma	4.14E-01	1.05E-01	WT
131 RERFLCMS_LUNG	RERF-LC-MS	lung	carcinoma	non-small_cell_carcinoma	1.42E-11	5.61E-01	WT
132 L1236_HAEMATOPOIETIC_AND_LYMPHOID_TISSUE	L-1236	haematopoietic_&_lymphoid_tissue	lymphoid_neoplasm	Hodgkin_lymphoma	9.26E-04	2.97E-01	WT
133 DANG_PANCREAS	DAN-G	pancreas	carcinoma	---	1.98E-01	-1.43E-01	WT
134 NCIH460_LUNG	NCI-H460	lung	carcinoma	large_cell_carcinoma	5.44E-11	5.46E-01	WT
135 KYSE140_OESOPHAGUS	KYSE-140	oesophagus	carcinoma	squamous_cell_carcinoma	7.60E-02	1.80E-01	WT
136 KYSE180_OESOPHAGUS	KYSE-180	oesophagus	carcinoma	squamous_cell_carcinoma	5.51E-01	8.67E-02	WT
137 HUPT3_PANCREAS	HUP-T3	pancreas	carcinoma	---	8.37E-02	-1.77E-01	WT
138 EFE184_ENDOMETRIUM	EFE-184	endometrium	carcinoma	---	4.34E-03	2.62E-01	WT
139 GCIY_STOMACH	GCIY	stomach	carcinoma	adenocarcinoma	6.43E-04	3.04E-01	WT
140 SH10TC_STOMACH	SH-10-TC	stomach	carcinoma	---	1.01E-12	5.90E-01	WT
141 CAOV4_OVARY	Caov-4	ovary	carcinoma	adenocarcinoma	6.55E-02	-1.85E-01	WT
142 HCC2218_BREAST	HCC2218	breast	carcinoma	ductal_carcinoma	1.19E-12	-5.88E-01	MUT_Intron
143 KMS21BM_HAEMATOPOIETIC_AND_LYMPHOID_TISSUE	KMS-21BM	haematopoietic_&_lymphoid_tissue	lymphoid_neoplasm	plasma_cell_myeloma	3.78E-02	2.03E-01	WT
144 KMS26_HAEMATOPOIETIC_AND_LYMPHOID_TISSUE	KMS-26	haematopoietic_&_lymphoid_tissue	lymphoid_neoplasm	plasma_cell_myeloma	1.54E-03	2.86E-01	WT
145 KMS27_HAEMATOPOIETIC_AND_LYMPHOID_TISSUE	KMS-27	haematopoietic_&_lymphoid_tissue	lymphoid_neoplasm	plasma_cell_myeloma	4.99E-03	2.59E-01	WT
146 KO52_HAEMATOPOIETIC_AND_LYMPHOID_TISSUE	KO52	haematopoietic_&_lymphoid_tissue	haematopoietic_neoplasm	acute_myeloid_leukaemia	1.05E-02	2.40E-01	WT
147 KMS28BM_HAEMATOPOIETIC_AND_LYMPHOID_TISSUE	KMS-28BM	haematopoietic_&_lymphoid_tissue	lymphoid_neoplasm	plasma_cell_myeloma	2.43E-03	2.76E-01	WT
148 KNS60_CENTRAL_NERVOUS_SYSTEM	KNS-60	central_nervous_system	glioma	astrocytoma_GradeIV	4.64E-19	7.30E-01	MUT_p.V292L/Missense
149 KNS81_CENTRAL_NERVOUS_SYSTEM	KNS-81	central_nervous_system	glioma	astrocytoma_GradeIV	2.10E-19	7.36E-01	WT
150 KPNSI9S_AUTONOMIC_GANGLIA	KP-N-SI9s	autonomic_ganglia	neuroblastoma	---	3.46E-20	7.52E-01	WT
151 LU99_LUNG	LU99	lung	carcinoma	large_cell_carcinoma	1.01E-15	6.60E-01	WT
152 KMS34_HAEMATOPOIETIC_AND_LYMPHOID_TISSUE	KMS-34	haematopoietic_&_lymphoid_tissue	lymphoid_neoplasm	plasma_cell_myeloma	1.19E-02	2.36E-01	WT
153 MKN1_STOMACH	MKN1	stomach	carcinoma	mixed_adeno-squamous_carcinoma	2.59E-11	5.54E-01	WT
154 MKN74_STOMACH	MKN74	stomach	carcinoma	tubular_adenocarcinoma	7.04E-03	-2.50E-01	WT
155 KP3_PANCREAS	KP-3	pancreas	carcinoma	ductal_carcinoma	4.08E-01	-1.06E-01	WT

156 NCO2_HAEMATOPOIETIC_AND_LYMPHOID_TISSUE	NCO2	haematopoietic_&_lymphoid_tissue	haematopoietic_neoplasm	chronic_myeloid_leukaemia	1.00E-03	2.95E-01	WT
157 KHM1B_HAEMATOPOIETIC_AND_LYMPHOID_TISSUE	KHM-1B	haematopoietic_&_lymphoid_tissue	lymphoid_neoplasm	plasma_cell_myeloma	1.94E-01	1.44E-01	WT
158 HSC2_UPPER_AERODIGESTIVE_TRACT	HSC-2	upper_aerodigestive_tract	carcinoma	squamous_cell_carcinoma	2.80E-01	-1.27E-01	WT
159 KG1C_CENTRAL_NERVOUS_SYSTEM	KG-1-C	central_nervous_system	glioma	astrocytoma_GradeIV	6.23E-19	7.27E-01	WT
160 HUH7_LIVER	HuH-7	liver	carcinoma	hepato_cellular_carcinoma	1.67E-05	3.72E-01	WT
161 KMBC2_URINARY_TRACT	KMBC-2	urinary_tract	carcinoma	---	3.49E-11	-5.51E-01	WT
162 KMRC20_KIDNEY	KMRC-20	kidney	carcinoma	renal_cell_carcinoma	7.75E-06	3.85E-01	WT
163 HUCCT1_BILIARY_TRACT	HuCCT1	biliary_tract	carcinoma	---	2.85E-01	-1.26E-01	WT
164 KIJK_HAEMATOPOIETIC_AND_LYMPHOID_TISSUE	Ki-JK	haematopoietic_&_lymphoid_tissue	lymphoid_neoplasm	anaplastic_large_cell_lymphoma	4.81E-04	3.10E-01	WT
165 A4FUK_HAEMATOPOIETIC_AND_LYMPHOID_TISSUE	A4/Fuk	haematopoietic_&_lymphoid_tissue	lymphoid_neoplasm	diffuse_large_B-cell_lymphoma	8.37E-02	1.77E-01	WT
166 KALS1_CENTRAL_NERVOUS_SYSTEM	KALS-1	central_nervous_system	glioma	---	1.88E-20	7.57E-01	WT
167 HEC108_ENDOMETRIUM	HEC-108	endometrium	carcinoma	adenocarcinoma	6.99E-01	-6.72E-02	MUT_3UTR
168 HEL9217_HAEMATOPOIETIC_AND_LYMPHOID_TISSUE	HEL 92.1.7	haematopoietic_&_lymphoid_tissue	haematopoietic_neoplasm	acute_myeloid_leukaemia	2.04E-06	4.06E-01	WT
169 HUH28_BILIARY_TRACT	HuH28	biliary_tract	carcinoma	---	9.00E-15	6.39E-01	WT
170 CMK115_HAEMATOPOIETIC_AND_LYMPHOID_TISSUE	CMK-11-5	haematopoietic_&_lymphoid_tissue	haematopoietic_neoplasm	acute_myeloid_leukaemia	6.17E-04	3.05E-01	WT
171 HEC265_ENDOMETRIUM	HEC-265	endometrium	carcinoma	adenocarcinoma	4.81E-02	-1.96E-01	WT
172 CAL12T_LUNG	CAL-12T	lung	carcinoma	non-small_cell_carcinoma	1.15E-02	-2.37E-01	WT
173 THP1_HAEMATOPOIETIC_AND_LYMPHOID_TISSUE	THP-1	haematopoietic_&_lymphoid_tissue	haematopoietic_neoplasm	acute_myeloid_leukaemia	6.70E-04	3.04E-01	WT
174 A3KAW_HAEMATOPOIETIC_AND_LYMPHOID_TISSUE	A3/KAW	haematopoietic_&_lymphoid_tissue	lymphoid_neoplasm	diffuse_large_B-cell_lymphoma	6.17E-04	3.05E-01	WT
175 SKNSH_AUTONOMIC_GANGLIA	SK-N-SH	autonomic_ganglia	neuroblastoma	---	1.88E-20	7.57E-01	WT
176 HCC1954_BREAST	HCC1954	breast	carcinoma	ductal_carcinoma	1.31E-06	-4.13E-01	WT
177 CAMA1_BREAST	CAMA-1	breast	carcinoma	---	1.73E-06	-4.09E-01	WT
178 HEC6_ENDOMETRIUM	HEC-6	endometrium	carcinoma	adenocarcinoma	5.23E-04	3.09E-01	MUT_p.A289V/Missense
179 AM38_CENTRAL_NERVOUS_SYSTEM	AM-38	central_nervous_system	glioma	astrocytoma_GradeIV	1.02E-17	7.02E-01	WT
180 HEC50B_ENDOMETRIUM	HEC-50B	endometrium	carcinoma	adenocarcinoma	1.24E-09	5.09E-01	MUT_3UTR
181 HSC3_UPPER_AERODIGESTIVE_TRACT	HSC-3	upper_aerodigestive_tract	carcinoma	squamous_cell_carcinoma	1.15E-02	2.37E-01	WT
182 HSC4_UPPER_AERODIGESTIVE_TRACT	HSC-4	upper_aerodigestive_tract	carcinoma	squamous_cell_carcinoma	1.35E-02	-2.33E-01	WT
183 KMS11_HAEMATOPOIETIC_AND_LYMPHOID_TISSUE	KMS-11	haematopoietic_&_lymphoid_tissue	lymphoid_neoplasm	plasma_cell_myeloma	4.99E-03	2.59E-01	WT
184 KP4_PANCREAS	KP4	pancreas	carcinoma	ductal_carcinoma	1.31E-11	5.62E-01	WT
185 KE39_STOMACH	KE-39	stomach	carcinoma	---	1.63E-12	-5.85E-01	WT
186 COV504_OVARY	COV504	ovary	carcinoma	---	1.05E-10	5.38E-01	WT
187 KE97_STOMACH	KE-97	stomach	carcinoma	---	8.37E-02	1.77E-01	WT
188 VMRCRCW_KIDNEY	VMRC-RCW	kidney	carcinoma	---	6.29E-07	4.24E-01	WT
189 HGC27_STOMACH	HGC-27	stomach	carcinoma	undifferentiated_adenocarcinoma	1.07E-14	6.37E-01	WT
190 LUDLU1_LUNG	LUDLU-1	lung	carcinoma	squamous_cell_carcinoma	1.43E-05	-3.75E-01	WT
191 HUG1N_STOMACH	HuG1-N	stomach	carcinoma	---	1.28E-12	-5.88E-01	WT
192 U266B1_HAEMATOPOIETIC_AND_LYMPHOID_TISSUE	U266B1	haematopoietic_&_lymphoid_tissue	lymphoid_neoplasm	plasma_cell_myeloma	2.62E-02	2.14E-01	WT
193 NUGC4_STOMACH	NUGC-4	stomach	carcinoma	signet_ring_adenocarcinoma	2.59E-11	-5.54E-01	WT
194 CAS1_CENTRAL_NERVOUS_SYSTEM	CAS-1	central_nervous_system	glioma	astrocytoma_GradeIV	6.72E-21	7.65E-01	WT
195 KMM1_HAEMATOPOIETIC_AND_LYMPHOID_TISSUE	KMM-1	haematopoietic_&_lymphoid_tissue	lymphoid_neoplasm	plasma_cell_myeloma	1.63E-04	3.32E-01	WT
196 CORL23_LUNG	COR-L23	lung	carcinoma	large_cell_carcinoma	9.86E-02	-1.71E-01	WT
197 MORCPR_LUNG	MOR/CPR	lung	carcinoma	adenocarcinoma	2.04E-05	-3.69E-01	WT
198 HARA_LUNG	HARA	lung	carcinoma	squamous_cell_carcinoma	5.25E-01	9.01E-02	WT
199 SKOV3_OVARY	SK-OV-3	ovary	carcinoma	adenocarcinoma	4.54E-08	4.62E-01	WT
200 HPAFII_PANCREAS	HPAF-II	pancreas	carcinoma	ductal_carcinoma	2.23E-11	-5.56E-01	WT
201 MC116_HAEMATOPOIETIC_AND_LYMPHOID_TISSUE	MC116	haematopoietic_&_lymphoid_tissue	lymphoid_neoplasm	B-cell_lymphoma_unspecified	5.35E-03	2.57E-01	WT
202 NCIH1568_LUNG	NCI-H1568	lung	carcinoma	non-small_cell_carcinoma	4.06E-04	-3.14E-01	WT
203 CA46_HAEMATOPOIETIC_AND_LYMPHOID_TISSUE	CA46	haematopoietic_&_lymphoid_tissue	lymphoid_neoplasm	Burkitt_lymphoma	7.78E-02	1.79E-01	WT
204 A498_KIDNEY	A-498	kidney	carcinoma	renal_cell_carcinoma	3.43E-09	4.96E-01	WT
205 BCP1_HAEMATOPOIETIC_AND_LYMPHOID_TISSUE	BCP-1	haematopoietic_&_lymphoid_tissue	lymphoid_neoplasm	B-cell_lymphoma_unspecified	2.47E-02	2.16E-01	WT
206 A673_BONE	A-673	bone	Ewings_sarcoma-peripheral_primitive_neuro_ectodermal_tumour	---	6.78E-11	5.43E-01	WT
207 AU565_BREAST	AU565	breast	carcinoma	---	3.28E-13	-6.02E-01	WT
208 CAPAN1_PANCREAS	Capan-1	pancreas	carcinoma	ductal_carcinoma	2.75E-08	-4.69E-01	WT

209	NCIH1563_LUNG	NCI-H1563	lung	carcinoma	adenocarcinoma	1.63E-09	5.05E-01	WT
210	NCIH358_LUNG	NCI-H358	lung	carcinoma	bronchiolo_alveolar_adenocarcinoma	1.58E-02	-2.29E-01	WT
211	CFPAC1_PANCREAS	CFPAC-1	pancreas	carcinoma	ductal_carcinoma	6.14E-03	-2.53E-01	WT
212	NIHOVCAR3_OVARY	NIH:OVCAR-3	ovary	carcinoma	---	5.89E-01	-8.16E-02	WT
213	NCIH1650_LUNG	NCI-H1650	lung	carcinoma	bronchiolo_alveolar_adenocarcinoma	2.75E-01	1.28E-01	MUT_p.ELREA746del/In_Frame_Deletion
214	NCIH1793_LUNG	NCI-H1793	lung	carcinoma	non-small_cell_carcinoma	1.19E-04	3.38E-01	MUT_p.C311F/Missense
215	NCIH1437_LUNG	NCI-H1437	lung	carcinoma	adenocarcinoma	2.34E-07	-4.39E-01	WT
216	KNS62_LUNG	KNS-62	lung	carcinoma	squamous_cell_carcinoma	2.47E-02	-2.16E-01	WT
217	HS578T_BREAST	Hs 578T	breast	carcinoma	ductal_carcinoma	1.41E-19	7.40E-01	WT
218	BT20_BREAST	BT-20	breast	carcinoma	ductal_carcinoma	5.10E-06	-3.92E-01	WT
219	SNU182_LIVER	SNU-182	liver	carcinoma	hepato_cellular_carcinoma	1.23E-13	6.12E-01	WT
220	SKNMC_BONE	SK-N-MC	bone	Ewings_sarcoma-peripheral_primitive_neuro_ectodermal_tumour	---	3.31E-10	5.25E-01	WT
221	HH_HAEMATOPOIETIC_AND_LYMPHOID_TISSUE	HH	haematopoietic_&_lymphoid_tissue	lymphoid_neoplasm	adult_T-cell_lymphoma-leukaemia	4.42E-04	3.12E-01	WT
222	FADU_UPPER_AERODIGESTIVE_TRACT	FaDu	upper_aerodigestive_tract	carcinoma	squamous_cell_carcinoma	2.43E-03	-2.76E-01	WT
223	SJRH30_SOFT_TISSUE	SJRH30	soft_tissue	rhabdomyosarcoma	---	7.58E-19	7.25E-01	WT
224	MDAMB361_BREAST	MDA-MB-361	breast	carcinoma	---	1.23E-02	-2.36E-01	WT
225	MDAMB436_BREAST	MDA-MB-436	breast	carcinoma	---	3.17E-15	6.49E-01	WT
226	PANC0213_PANCREAS	Panc 02.13	pancreas	carcinoma	---	2.10E-01	-1.40E-01	WT
227	DB_HAEMATOPOIETIC_AND_LYMPHOID_TISSUE	DB	haematopoietic_&_lymphoid_tissue	lymphoid_neoplasm	diffuse_large_B-cell_lymphoma	1.53E-01	1.54E-01	WT
228	RDES_BONE	RD-ES	bone	Ewings_sarcoma-peripheral_primitive_neuro_ectodermal_tumour	---	3.55E-10	5.24E-01	WT
229	CAL27_UPPER_AERODIGESTIVE_TRACT	CAL 27	upper_aerodigestive_tract	carcinoma	squamous_cell_carcinoma	2.43E-03	-2.76E-01	WT
230	SW1271_LUNG	SW 1271	lung	carcinoma	small_cell_carcinoma	7.57E-15	6.40E-01	WT
231	NCIH1792_LUNG	NCI-H1792	lung	carcinoma	adenocarcinoma	7.14E-12	5.69E-01	WT
232	GCT_SOFT_TISSUE	GCT	soft_tissue	malignant_fibrous_histiocytoma-pleomorphic_sarcoma	---	1.02E-18	7.23E-01	MUT_Intron
233	SKBR3_BREAST	SK-BR-3	breast	carcinoma	---	6.13E-12	-5.71E-01	WT
234	SNU16_STOMACH	SNU-16	stomach	carcinoma	undifferentiated_adenocarcinoma	9.59E-14	-6.15E-01	WT
235	NCIH82_LUNG	NCI-H82	lung	carcinoma	small_cell_carcinoma	1.07E-07	4.50E-01	WT
236	NCIN87_STOMACH	NCI-N87	stomach	carcinoma	---	1.12E-08	-4.80E-01	WT
237	U2OS_BONE	U-2 OS	bone	osteosarcoma	---	1.92E-11	5.58E-01	WT
238	H4_CENTRAL_NERVOUS_SYSTEM	H4	central_nervous_system	glioma	---	3.93E-18	7.11E-01	WT
239	GDM1_HAEMATOPOIETIC_AND_LYMPHOID_TISSUE	GDM-1	haematopoietic_&_lymphoid_tissue	haematopoietic_neoplasm	acute_myeloid_leukaemia	1.30E-04	3.36E-01	WT
240	HCC1143_BREAST	HCC1143	breast	carcinoma	ductal_carcinoma	7.24E-01	6.38E-02	WT
241	T24_URINARY_TRACT	T24	urinary_tract	carcinoma	transitional_cell_carcinoma	1.01E-12	5.90E-01	WT
242	G401_SOFT_TISSUE	G-401	soft_tissue	rhabdoid_tumour	---	9.76E-11	5.39E-01	WT
243	HCC1937_BREAST	HCC1937	breast	carcinoma	ductal_carcinoma	1.67E-05	-3.72E-01	WT
244	OCUM1_STOMACH	OCUM-1	stomach	carcinoma	diffuse_adenocarcinoma	1.05E-11	-5.65E-01	WT
245	D283MED_CENTRAL_NERVOUS_SYSTEM	D283 Med	central_nervous_system	primitive_neuro-ectodermal_tumour-medulloblastoma	---	3.21E-09	4.97E-01	WT
246	SCC9_UPPER_AERODIGESTIVE_TRACT	SCC-9	upper_aerodigestive_tract	carcinoma	squamous_cell_carcinoma	2.85E-01	1.26E-01	WT
247	HCC1395_BREAST	HCC1395	breast	carcinoma	ductal_carcinoma	7.71E-12	5.68E-01	WT
248	NMCG1_CENTRAL_NERVOUS_SYSTEM	NMC-G1	central_nervous_system	glioma	---	5.76E-18	7.07E-01	WT
249	HS604T_HAEMATOPOIETIC_AND_LYMPHOID_TISSUE	Hs 604.T	haematopoietic_&_lymphoid_tissue	lymphoid_neoplasm	Hodgkin_lymphoma	2.59E-11	5.54E-01	MUT_Intron
250	TE9_OESOPHAGUS	TE-9	oesophagus	carcinoma	squamous_cell_carcinoma	4.90E-05	-3.54E-01	WT
251	KNS42_CENTRAL_NERVOUS_SYSTEM	KNS-42	central_nervous_system	glioma	---	1.07E-14	6.37E-01	WT
252	KYSE520_OESOPHAGUS	KYSE-520	oesophagus	carcinoma	squamous_cell_carcinoma	5.92E-05	3.50E-01	WT
253	KYSE270_OESOPHAGUS	KYSE-270	oesophagus	carcinoma	squamous_cell_carcinoma	3.11E-02	2.09E-01	WT
254	JHUEM1_ENDOMETRIUM	JHUEM-1	endometrium	carcinoma	adenocarcinoma	1.36E-04	-3.35E-01	WT
255	YH13_CENTRAL_NERVOUS_SYSTEM	YH-13	central_nervous_system	glioma	astrocytoma_GradeIV	7.14E-23	8.02E-01	WT
256	IALM_LUNG	IA-LM	lung	carcinoma	large_cell_carcinoma	2.32E-10	5.29E-01	WT
257	NCIH2052_PLEURA	NCI-H2052	pleura	mesothelioma	---	5.90E-16	6.65E-01	WT

258	EB2_HAEMATOPOIETIC_AND_LYMPHOID_TISSUE	EB2	haematopoietic_&_lymphoid_tissue	lymphoid_neoplasm	Burkitt_lymphoma	6.17E-04	3.05E-01	WT
259	SKNFI_AUTONOMIC_GANGLIA	SK-N-FI	autonomic_ganglia	neuroblastoma	---	7.85E-11	5.42E-01	WT
260	SKNAS_AUTONOMIC_GANGLIA	SK-N-AS	autonomic_ganglia	neuroblastoma	---	2.21E-18	7.16E-01	WT
261	GA10_HAEMATOPOIETIC_AND_LYMPHOID_TISSUE	GA-10	haematopoietic_&_lymphoid_tissue	lymphoid_neoplasm	Burkitt_lymphoma	4.33E-02	1.99E-01	WT
262	SNU398_LIVER	SNU-398	liver	carcinoma	hepato_cellular_carcinoma	1.51E-14	6.34E-01	WT
263	SNU423_LIVER	SNU-423	liver	carcinoma	hepato_cellular_carcinoma	1.12E-17	7.02E-01	WT
264	SCC4_UPPER_AERODIGESTIVE_TRACT	SCC-4	upper_aerodigestive_tract	carcinoma	squamous_cell_carcinoma	9.63E-02	1.72E-01	WT
265	EB1_HAEMATOPOIETIC_AND_LYMPHOID_TISSUE	EB1	haematopoietic_&_lymphoid_tissue	lymphoid_neoplasm	Burkitt_lymphoma	9.26E-04	2.97E-01	WT
266	HCC1187_BREAST	HCC1187	breast	carcinoma	ductal_carcinoma	3.15E-01	-1.21E-01	WT
267	SNU387_LIVER	SNU-387	liver	carcinoma	hepato_cellular_carcinoma	3.15E-17	6.92E-01	WT
268	DAUDI_HAEMATOPOIETIC_AND_LYMPHOID_TISSUE	Daudi	haematopoietic_&_lymphoid_tissue	lymphoid_neoplasm	Burkitt_lymphoma	1.23E-01	1.62E-01	MUT_Intron
269	HT_HAEMATOPOIETIC_AND_LYMPHOID_TISSUE	HT	haematopoietic_&_lymphoid_tissue	lymphoid_neoplasm	B-cell_lymphoma_unspecified	7.60E-02	1.80E-01	WT
270	MJ_HAEMATOPOIETIC_AND_LYMPHOID_TISSUE	MJ	haematopoietic_&_lymphoid_tissue	lymphoid_neoplasm	mycosisfungoides-Sezary_syndrome	1.60E-03	2.85E-01	WT
271	JM1_HAEMATOPOIETIC_AND_LYMPHOID_TISSUE	JM1	haematopoietic_&_lymphoid_tissue	lymphoid_neoplasm	B-cell_lymphoma_unspecified	2.09E-03	2.79E-01	WT
272	C2BBE1_LARGE_INTESTINE	C2BBe1	large_intestine	carcinoma	adenocarcinoma	3.26E-03	-2.69E-01	WT
273	SKES1_BONE	SK-ES-1	bone	Ewings_sarcoma-peripheral_primitive_neuro_ectodermal_tumour	---	3.30E-12	5.77E-01	WT
274	EPLC272H_LUNG	EPLC-272H	lung	carcinoma	squamous_cell_carcinoma	2.47E-02	2.16E-01	WT
275	BXPC3_PANCREAS	BxPC-3	pancreas	carcinoma	ductal_carcinoma	1.04E-04	-3.40E-01	WT
276	NCIH1355_LUNG	NCI-H1355	lung	carcinoma	adenocarcinoma	7.15E-05	3.47E-01	MUT_p.Q1159H/Missense
277	SNU5_STOMACH	SNU-5	stomach	carcinoma	undifferentiated_adenocarcinoma	1.76E-01	1.48E-01	WT
278	SNU475_LIVER	SNU-475	liver	carcinoma	hepato_cellular_carcinoma	1.51E-14	6.34E-01	WT
279	HEC251_ENDOMETRIUM	HEC-251	endometrium	carcinoma	adenocarcinoma	2.27E-02	-2.19E-01	WT
280	HCC95_LUNG	HCC-95	lung	carcinoma	squamous_cell_carcinoma	5.25E-01	9.01E-02	WT
281	RPMI7951_SKIN	RPMI-7951	skin	malignant_melanoma	---	7.75E-20	7.45E-01	WT
282	COLO783_SKIN	COLO-783	skin	malignant_melanoma	---	8.33E-12	5.67E-01	WT
283	BICR31_UPPER_AERODIGESTIVE_TRACT	BICR 31	upper_aerodigestive_tract	carcinoma	squamous_cell_carcinoma	6.67E-01	-7.14E-02	WT
284	COV362_OVARY	COV362	ovary	carcinoma	---	8.80E-10	5.13E-01	MUT_p.H1124Q/Missense
285	COLO792_SKIN	COLO 792	skin	malignant_melanoma	---	1.05E-08	4.81E-01	WT
286	COV434_OVARY	COV434	ovary	sexcord-stromal_tumour	granulosa_cell_tumour	2.40E-11	5.55E-01	WT
287	WM983B_SKIN	WM-983B	skin	malignant_melanoma	---	3.76E-11	5.50E-01	MUT_3UTR
288	HS751T_HAEMATOPOIETIC_AND_LYMPHOID_TISSUE	Hs 751.T	haematopoietic_&_lymphoid_tissue	lymphoid_neoplasm	Hodgkin_lymphoma	1.03E-21	7.81E-01	WT
289	LS513_LARGE_INTESTINE	LS513	large_intestine	carcinoma	adenocarcinoma	2.57E-19	-7.35E-01	WT
290	NCIH2110_LUNG	NCI-H2110	lung	carcinoma	non-small_cell_carcinoma	1.04E-04	-3.40E-01	WT
291	CHAGOK1_LUNG	ChaGo-K-1	lung	carcinoma	---	3.90E-03	-2.64E-01	WT
292	DMS454_LUNG	DMS 454	lung	carcinoma	small_cell_carcinoma	8.77E-02	-1.75E-01	WT
293	CORL47_LUNG	COR-L47	lung	carcinoma	small_cell_carcinoma	2.75E-01	1.28E-01	WT
294	DMS273_LUNG	DMS 273	lung	carcinoma	small_cell_carcinoma	1.53E-11	5.60E-01	WT
295	CORL311_LUNG	COR-L311	lung	carcinoma	small_cell_carcinoma	2.10E-01	1.40E-01	WT
296	NCIH2286_LUNG	NCI-H2286	lung	carcinoma	small_cell_carcinoma	4.16E-14	6.23E-01	WT
297	WM793_SKIN	WM-793	skin	malignant_melanoma	---	4.57E-17	6.89E-01	WT
298	A704_KIDNEY	A-704	kidney	carcinoma	renal_cell_carcinoma	4.24E-05	3.56E-01	WT
299	MEWO_SKIN	MeWo	skin	malignant_melanoma	---	4.38E-10	5.21E-01	WT
300	WM88_SKIN	WM-88	skin	malignant_melanoma	---	6.45E-16	6.64E-01	MUT_p.E758K/Missense
301	NCIH2081_LUNG	NCI-H2081	lung	carcinoma	small_cell_carcinoma	9.80E-03	2.41E-01	WT
302	NCIH1092_LUNG	NCI-H1092	lung	carcinoma	small_cell_carcinoma	4.66E-03	2.60E-01	WT
303	SW837_LARGE_INTESTINE	SW837	large_intestine	carcinoma	adenocarcinoma	2.26E-03	-2.77E-01	WT
304	IGR1_SKIN	IGR-1	skin	malignant_melanoma	---	6.78E-11	5.43E-01	WT
305	NCIH1105_LUNG	NCI-H1105	lung	carcinoma	small_cell_carcinoma	1.68E-02	2.27E-01	WT
306	NCIH1876_LUNG	NCI-H1876	lung	carcinoma	small_cell_carcinoma	1.08E-02	2.39E-01	WT
307	CORL24_LUNG	COR-L24	lung	carcinoma	small_cell_carcinoma	1.87E-03	2.81E-01	WT
308	COV644_OVARY	COV644	ovary	carcinoma	---	1.70E-04	-3.31E-01	WT
309	GP2D_LARGE_INTESTINE	GP2d	large_intestine	carcinoma	adenocarcinoma	3.85E-12	-5.76E-01	WT
310	PECAPJ15_UPPER_AERODIGESTIVE_TRACT	PE/CA-PJ15	upper_aerodigestive_tract	carcinoma	squamous_cell_carcinoma	4.66E-03	-2.60E-01	WT

311 CORL88_LUNG	COR-L88	lung	carcinoma	small_cell_carcinoma	5.12E-01	9.18E-02	MUT_Intron
312 CORL95_LUNG	COR-L95	lung	carcinoma	small_cell_carcinoma	4.50E-01	-1.00E-01	WT
313 PECAPJ49_UPPER_AERODIGESTIVE_TRACT	PE/CA-PJ49	upper_aerodigestive_tract	carcinoma	squamous_cell_carcinoma	4.94E-02	1.95E-01	WT
314 PECAPJ34CLONEC12_UPPER_AERODIGESTIVE_TRACT	PE/CA-PJ34 (clone C12)	upper_aerodigestive_tract	carcinoma	squamous_cell_carcinoma	2.95E-01	-1.24E-01	WT
315 RT11284_URINARY_TRACT	RT112/84	urinary_tract	carcinoma	---	5.04E-10	-5.20E-01	WT
316 PECAPJ41CLONED2_UPPER_AERODIGESTIVE_TRACT	PE/CA-PJ41 (clone D2)	upper_aerodigestive_tract	carcinoma	squamous_cell_carcinoma	5.00E-01	9.35E-02	WT
317 OV7_OVARY	OV7	ovary	carcinoma	---	1.51E-14	6.34E-01	WT
318 OAW42_OVARY	OAW42	ovary	carcinoma	---	3.58E-02	2.05E-01	WT
319 SUIT2_PANCREAS	SUIT-2	pancreas	carcinoma	ductal_carcinoma	3.03E-03	2.70E-01	WT
320 LC1SQSF_LUNG	LC-1/sq-SF	lung	carcinoma	squamous_cell_carcinoma	5.00E-07	4.28E-01	WT
321 KASUMI1_HAEMATOPOIETIC_AND_LYMPHOID_TISSUE	KASUMI-1	haematopoietic_&_lymphoid_tissue	haematopoietic_neoplasm	acute_myeloid_leukaemia	2.02E-03	2.80E-01	WT
322 SUPM2_HAEMATOPOIETIC_AND_LYMPHOID_TISSUE	SUP-M2	haematopoietic_&_lymphoid_tissue	lymphoid_neoplasm	anaplastic_large_cell_lymphoma	3.14E-03	2.70E-01	WT
323 SKHEP1_LIVER	SK-HEP-1	liver	carcinoma	adenocarcinoma	2.62E-16	6.73E-01	WT
324 OVCAR8_OVARY	OVCAR-8	ovary	carcinoma	---	8.43E-16	6.62E-01	WT
325 PANC0203_PANCREAS	Panc 02.03	pancreas	carcinoma	---	4.82E-03	-2.59E-01	WT
326 NCIH2172_LUNG	NCI-H2172	lung	carcinoma	non-small_cell_carcinoma	2.16E-10	5.30E-01	MUT_3UTR
327 ISHIKAWAHERAKLIO02ER_ENDOMETRIUM	Ishikawa (Heraklio) 02 ER-	endometrium	carcinoma	adenocarcinoma	4.94E-02	1.95E-01	MUT_p.R973Q/Missense
328 NCIH596_LUNG	NCI-H596	lung	carcinoma	mixed_adeno-squamous_carcinoma	2.90E-01	1.25E-01	WT
329 NCIH650_LUNG	NCI-H650	lung	carcinoma	bronchiolo_alveolar_adenocarcinoma	1.97E-17	6.96E-01	MUT_Intron
330 SW1573_LUNG	SW 1573	lung	carcinoma	squamous_cell_carcinoma	3.56E-13	6.01E-01	WT
331 OVMANA_OVARY	OVMANA	ovary	carcinoma	clear_cell_carcinoma	7.53E-03	-2.48E-01	WT
332 SKCO1_LARGE_INTESTINE	SK-CO-1	large_intestine	carcinoma	adenocarcinoma	7.47E-14	-6.17E-01	WT
333 KARPAS299_HAEMATOPOIETIC_AND_LYMPHOID_TISSUE	KARPAS-299	haematopoietic_&_lymphoid_tissue	lymphoid_neoplasm	anaplastic_large_cell_lymphoma	7.88E-04	3.00E-01	WT
334 YAPC_PANCREAS	YAPC	pancreas	carcinoma	---	8.22E-05	-3.44E-01	WT
335 HT1197_URINARY_TRACT	HT-1197	urinary_tract	carcinoma	---	6.08E-02	-1.88E-01	WT
336 A253_SALIVARY_GLAND	A-253	salivary_gland	carcinoma	muco0epidermoid_carcinoma	8.05E-03	2.47E-01	WT
337 HCC366_LUNG	HCC-366	lung	carcinoma	mixed_adeno-squamous_carcinoma	7.11E-09	4.86E-01	WT
338 DKMG_CENTRAL_NERVOUS_SYSTEM	DK-MG	central_nervous_system	glioma	astrocytoma_GradeIV	7.96E-23	8.01E-01	WT
339 SNU1040_LARGE_INTESTINE	SNU-1040	large_intestine	carcinoma	---	9.91E-07	-4.18E-01	WT
340 SNU1076_UPPER_AERODIGESTIVE_TRACT	SNU-1076	upper_aerodigestive_tract	carcinoma	squamous_cell_carcinoma	2.14E-05	-3.68E-01	WT
341 SNU175_LARGE_INTESTINE	SNU-175	large_intestine	carcinoma	---	1.17E-05	-3.78E-01	MUT_p.A864V/Missense
342 SNU407_LARGE_INTESTINE	SNU-407	large_intestine	carcinoma	---	1.86E-13	-6.08E-01	MUT_p.G983/Splice_Site_Insertion
343 SNU201_CENTRAL_NERVOUS_SYSTEM	SNU-201	central_nervous_system	glioma	astrocytoma_GradeIV	1.02E-20	7.62E-01	WT
344 KMS20_HAEMATOPOIETIC_AND_LYMPHOID_TISSUE	KMS-20	haematopoietic_&_lymphoid_tissue	lymphoid_neoplasm	plasma_cell_myeloma	1.39E-02	2.32E-01	WT
345 SNU216_STOMACH	SNU-216	stomach	carcinoma	---	3.73E-04	-3.15E-01	WT
346 SNU1079_BILIARY_TRACT	SNU-1079	biliary_tract	carcinoma	---	4.81E-02	1.96E-01	WT
347 HCC1195_LUNG	HCC-1195	lung	carcinoma	mixed_adeno-squamous_carcinoma	2.07E-02	-2.21E-01	WT
348 SNU466_CENTRAL_NERVOUS_SYSTEM	SNU-466	central_nervous_system	glioma	astrocytoma_GradeIV	5.35E-14	6.21E-01	WT
349 SNU1105_CENTRAL_NERVOUS_SYSTEM	SNU-1105	central_nervous_system	glioma	astrocytoma_GradeIV	5.75E-23	8.04E-01	MUT_p.V742I/Missense
350 SNU283_LARGE_INTESTINE	SNU-283	large_intestine	carcinoma	---	8.26E-21	-7.64E-01	WT
351 YD15_SALIVARY_GLAND	YD-15	salivary_gland	carcinoma	muco0epidermoid_carcinoma	2.13E-04	-3.27E-01	WT
352 SNU308_BILIARY_TRACT	SNU-308	biliary_tract	carcinoma	---	1.31E-06	-4.13E-01	WT
353 SNU245_BILIARY_TRACT	SNU-245	biliary_tract	carcinoma	---	1.12E-20	-7.61E-01	WT
354 SNU478_BILIARY_TRACT	SNU-478	biliary_tract	carcinoma	---	8.22E-05	-3.44E-01	WT
355 SNU620_STOMACH	SNU-620	stomach	carcinoma	---	1.01E-15	-6.60E-01	WT
356 SNU410_PANCREAS	SNU-410	pancreas	carcinoma	---	4.48E-09	4.92E-01	WT
357 SNU899_UPPER_AERODIGESTIVE_TRACT	SNU-899	upper_aerodigestive_tract	carcinoma	squamous_cell_carcinoma	4.58E-06	-3.94E-01	WT
358 SNU503_LARGE_INTESTINE	SNU-503	large_intestine	carcinoma	---	1.43E-03	-2.87E-01	WT
359 SET2_HAEMATOPOIETIC_AND_LYMPHOID_TISSUE	Set-2	haematopoietic_&_lymphoid_tissue	haematopoietic_neoplasm	essential_thrombo-cythaemia	6.43E-04	3.04E-01	WT
360 SNU738_CENTRAL_NERVOUS_SYSTEM	SNU-738	central_nervous_system	glioma	oligo-dendro_glioma	7.14E-12	5.69E-01	WT
361 SNU626_CENTRAL_NERVOUS_SYSTEM	SNU-626	central_nervous_system	glioma	astrocytoma_GradeIV	3.14E-16	6.71E-01	WT
362 SNU878_LIVER	SNU-878	liver	carcinoma	hepato_cellular_carcinoma	1.70E-04	3.31E-01	WT

363	SNU840_OVARY	SNU-840	ovary	carcinoma	Brenner_tumour	1.18E-01	1.64E-01	WT
364	IGR39_SKIN	IGR-39	skin	malignant_melanoma	---	1.27E-14	6.35E-01	MUT_Intron
365	ALEXANDERCELLS_LIVER	Alexander cells	liver	carcinoma	hepato_cellular_carcinoma	6.51E-05	3.49E-01	WT
366	DAOY_CENTRAL_NERVOUS_SYSTEM	Daoy	central_nervous_system	primitive_neuro-ectodermal_tumour-medulloblastoma	---	5.51E-17	6.87E-01	WT
367	HEP3B217_LIVER	Hep 3B2.1-7	liver	carcinoma	hepato_cellular_carcinoma	1.23E-01	1.62E-01	WT
368	KYM1_SOFT_TISSUE	KYM-1	soft_tissue	rhabdomyosarcoma	---	5.84E-09	4.89E-01	WT
369	HLE_LIVER	HLE	liver	carcinoma	hepato_cellular_carcinoma	4.33E-18	7.10E-01	WT
370	CMK_HAEMATOPOIETIC_AND_LYMPHOID_TISSUE	CMK	haematopoietic_&_lymphoid_tissue	haematopoietic_neoplasm	acute_myeloid_leukaemia	2.62E-03	2.74E-01	WT
371	JMSU1_URINARY_TRACT	JMSU-1	urinary_tract	carcinoma	---	2.44E-15	6.51E-01	WT
372	IMR32_AUTONOMIC_GANGLIA	IMR-32	autonomic_ganglia	neuroblastoma	---	7.35E-13	5.94E-01	WT
373	SUDHL8_HAEMATOPOIETIC_AND_LYMPHOID_TISSUE	SU-DHL-8	haematopoietic_&_lymphoid_tissue	lymphoid_neoplasm	diffuse_large_B-cell_lymphoma	1.60E-03	2.85E-01	WT
374	T3M4_PANCREAS	T3M-4	pancreas	carcinoma	ductal_carcinoma	3.74E-07	-4.32E-01	WT
375	COLO818_SKIN	COLO-818	skin	malignant_melanoma	---	1.04E-13	6.14E-01	WT
376	PK45H_PANCREAS	PK-45H	pancreas	carcinoma	---	2.27E-08	4.71E-01	WT
377	HEC59_ENDOMETRIUM	HEC-59	endometrium	carcinoma	adenocarcinoma	7.06E-02	1.83E-01	p.R531*
378	CPCN_LUNG	CPC-N	lung	carcinoma	small_cell_carcinoma	2.99E-06	4.01E-01	WT
379	NUGC2_STOMACH	NUGC-2	stomach	carcinoma	adenocarcinoma	2.18E-01	-1.39E-01	WT
380	PLCPRF5_LIVER	PLC/PRF/5	liver	carcinoma	hepato_cellular_carcinoma	2.32E-04	3.25E-01	WT
381	KPNYN_AUTONOMIC_GANGLIA	KP-N-YN	autonomic_ganglia	neuroblastoma	---	5.05E-11	5.47E-01	WT
382	CCFSTTG1_CENTRAL_NERVOUS_SYSTEM	CCF-STTG1	central_nervous_system	glioma	astrocytoma	2.33E-22	7.93E-01	WT
383	NCIH929_HAEMATOPOIETIC_AND_LYMPHOID_TISSUE	NCI-H929	haematopoietic_&_lymphoid_tissue	lymphoid_neoplasm	plasma_cell_myeloma	4.94E-02	1.95E-01	WT
384	KMRC3_KIDNEY	KMRC-3	kidney	carcinoma	clear_cell_renal_cell_carcinoma	1.54E-07	4.45E-01	WT
385	NCIH2023_LUNG	NCI-H2023	lung	carcinoma	adenocarcinoma	7.49E-05	3.46E-01	WT
386	DMS153_LUNG	DMS 153	lung	carcinoma	small_cell_carcinoma	1.76E-01	-1.48E-01	WT
387	NCIH1048_LUNG	NCI-H1048	lung	carcinoma	small_cell_carcinoma	2.14E-02	2.20E-01	WT
388	D341MED_CENTRAL_NERVOUS_SYSTEM	D341 Med	central_nervous_system	primitive_neuro-ectodermal_tumour-medulloblastoma	---	4.20E-07	4.30E-01	WT
389	NCIH660_PROSTATE	NCI-H660	prostate	carcinoma	small_cell_carcinoma	5.49E-02	-1.91E-01	MUT_Intron
390	NCIH1618_LUNG	NCI-H1618	lung	carcinoma	small_cell_carcinoma	3.63E-01	1.13E-01	WT
391	HS675T_LARGE_INTESTINE	Hs 675.T	large_intestine	carcinoma	---	8.74E-17	6.83E-01	WT
392	PEER_HAEMATOPOIETIC_AND_LYMPHOID_TISSUE	PEER	haematopoietic_&_lymphoid_tissue	lymphoid_neoplasm	acute_lymphoblastic_T-cell_leukaemia	3.28E-04	3.18E-01	WT
393	SKM1_HAEMATOPOIETIC_AND_LYMPHOID_TISSUE	SKM-1	haematopoietic_&_lymphoid_tissue	haematopoietic_neoplasm	acute_myeloid_leukaemia	5.14E-05	3.53E-01	WT
394	HT144_SKIN	HT-144	skin	malignant_melanoma	---	1.64E-17	6.98E-01	WT
395	SKMEL31_SKIN	SK-MEL-31	skin	malignant_melanoma	---	4.50E-12	5.74E-01	WT
396	HS839T_SKIN	Hs 839.T	skin	malignant_melanoma	---	1.23E-22	7.98E-01	WT
397	HS939T_SKIN	Hs 939.T	skin	malignant_melanoma	---	2.68E-18	7.14E-01	WT
398	OCILY3_HAEMATOPOIETIC_AND_LYMPHOID_TISSUE	OCI-LY3	haematopoietic_&_lymphoid_tissue	lymphoid_neoplasm	diffuse_large_B-cell_lymphoma	2.02E-03	2.80E-01	WT
399	HS819T_BONE	Hs 819.T	bone	chondrosarcoma	---	2.15E-21	7.75E-01	WT
400	NCIH69_LUNG	NCI-H69	lung	carcinoma	small_cell_carcinoma	1.50E-01	1.55E-01	WT
401	DV90_LUNG	DV-90	lung	carcinoma	adenocarcinoma	4.83E-08	-4.61E-01	WT
402	ZR751_BREAST	ZR-75-1	breast	carcinoma	ductal_carcinoma	2.07E-11	-5.57E-01	WT
403	KG1_HAEMATOPOIETIC_AND_LYMPHOID_TISSUE	KG-1	haematopoietic_&_lymphoid_tissue	haematopoietic_neoplasm	acute_myeloid_leukaemia	1.04E-03	2.94E-01	WT
404	SKNBE2_AUTONOMIC_GANGLIA	SK-N-BE(2)	autonomic_ganglia	neuroblastoma	---	1.28E-12	5.88E-01	WT
405	HCC70_BREAST	HCC70	breast	carcinoma	ductal_carcinoma	1.37E-07	-4.46E-01	WT
406	NCIH2452_PLEURA	NCI-H2452	pleura	mesothelioma	---	6.88E-14	6.18E-01	WT
407	SNU1_STOMACH	SNU-1	stomach	carcinoma	undifferentiated_adenocarcinoma	9.45E-05	3.42E-01	WT
408	LOUCY_HAEMATOPOIETIC_AND_LYMPHOID_TISSUE	Loucy	haematopoietic_&_lymphoid_tissue	lymphoid_neoplasm	acute_lymphoblastic_T-cell_leukaemia	2.53E-04	3.23E-01	WT
409	HCC56_LARGE_INTESTINE	HCC-56	large_intestine	carcinoma	adenocarcinoma	1.23E-13	-6.12E-01	WT
410	NCIH1648_LUNG	NCI-H1648	lung	carcinoma	adenocarcinoma	1.55E-06	-4.11E-01	WT
411	TO175T_HAEMATOPOIETIC_AND_LYMPHOID_TISSUE	TO 175.T	haematopoietic_&_lymphoid_tissue	lymphoid_neoplasm	Hodgkin_lymphoma	1.16E-19	7.41E-01	WT
412	MPP89_PLEURA	MPP 89	pleura	mesothelioma	---	2.66E-15	6.51E-01	MUT_Intron
413	PANC0813_PANCREAS	Panc 08.13	pancreas	carcinoma	ductal_carcinoma	4.01E-08	-4.63E-01	WT

414	MG63_BONE	MG-63	bone	osteosarcoma	---	7.27E-17	6.85E-01	WT
415	PK1_PANCREAS	PK-1	pancreas	carcinoma	---	2.13E-04	-3.27E-01	WT
416	HS840T_UPPER_AERODIGESTIVE_TRACT	Hs 840.T	upper_aerodigestive_tract	other	papilloma	2.55E-20	7.54E-01	WT
417	NCIH23_LUNG	NCI-H23	lung	carcinoma	non-small_cell_carcinoma	4.50E-12	5.74E-01	WT
418	TE617T_SOFT_TISSUE	TE 617.T	soft_tissue	rhabdomyosarcoma	---	1.64E-14	6.33E-01	WT
419	SW1088_CENTRAL_NERVOUS_SYSTEM	SW 1088	central_nervous_system	glioma	---	2.55E-20	7.54E-01	WT
420	ISTMES2_PLEURA	IST-MES2	pleura	mesothelioma	---	6.13E-12	5.71E-01	WT
421	NCIH1373_LUNG	NCI-H1373	lung	carcinoma	adenocarcinoma	1.78E-02	2.25E-01	MUT_Intron
422	NCIH1930_LUNG	NCI-H1930	lung	carcinoma	small_cell_carcinoma	1.95E-04	3.28E-01	WT
423	NCIH1623_LUNG	NCI-H1623	lung	carcinoma	adenocarcinoma	2.53E-04	-3.23E-01	WT
424	HS229T_LUNG	Hs 229.T	lung	carcinoma	adenocarcinoma	3.57E-22	7.89E-01	WT
425	NCIH1838_LUNG	NCI-H1838	lung	carcinoma	non-small_cell_carcinoma	3.52E-01	1.15E-01	WT
426	NCIH1836_LUNG	NCI-H1836	lung	carcinoma	small_cell_carcinoma	6.67E-01	7.14E-02	WT
427	HS688AT_SKIN	Hs 688(A).T	skin	malignant_melanoma	---	3.81E-19	7.31E-01	WT
428	PANC0504_PANCREAS	Panc 05.04	pancreas	carcinoma	---	2.00E-08	-4.73E-01	WT
429	HS822T_BONE	Hs 822.T	bone	Ewings_sarcoma-peripheral_primitive_neuro_ectodermal_tumour	---	4.91E-22	7.87E-01	WT
430	BL70_HAEMATOPOIETIC_AND_LYMPHOID_TISSUE	BL-70	haematopoietic_&_lymphoid_tissue	lymphoid_neoplasm	Burkitt_lymphoma	1.31E-02	2.34E-01	WT
431	CI1_HAEMATOPOIETIC_AND_LYMPHOID_TISSUE	CI-1	haematopoietic_&_lymphoid_tissue	lymphoid_neoplasm	B-cell_lymphoma_unspecified	2.52E-03	2.75E-01	WT
432	G292CLONEA141B1_BONE	G-292, clone A141B1	bone	osteosarcoma	---	3.11E-25	8.44E-01	WT
433	RCM1_LARGE_INTESTINE	RCM-1	large_intestine	carcinoma	adenocarcinoma	1.13E-11	-5.64E-01	WT
434	HS737T_BONE	Hs 737.T	bone	other	giant_cell_tumour	1.21E-24	8.33E-01	WT
435	HS616T_HAEMATOPOIETIC_AND_LYMPHOID_TISSUE	Hs 616.T	haematopoietic_&_lymphoid_tissue	lymphoid_neoplasm	Hodgkin_lymphoma	7.75E-20	7.45E-01	WT
436	HS600T_SKIN	Hs 600.T	skin	malignant_melanoma	---	1.25E-23	8.15E-01	WT
437	A101D_SKIN	A101D	skin	malignant_melanoma	---	1.01E-12	5.90E-01	WT
438	NCIH1755_LUNG	NCI-H1755	lung	carcinoma	adenocarcinoma	4.36E-11	5.48E-01	WT
439	143B_BONE	143B	bone	osteosarcoma	---	4.63E-23	8.05E-01	WT
440	RERFLCKJ_LUNG	RERF-LC-KJ	lung	carcinoma	non-small_cell_carcinoma	1.22E-11	-5.63E-01	WT
441	NCIH1693_LUNG	NCI-H1693	lung	carcinoma	adenocarcinoma	1.15E-02	2.37E-01	WT
442	SW780_URINARY_TRACT	SW 780	urinary_tract	carcinoma	transitional_cell_carcinoma	1.53E-09	-5.06E-01	WT
443	HS683_CENTRAL_NERVOUS_SYSTEM	Hs 683	central_nervous_system	glioma	astrocytoma_GradeIV	1.03E-21	7.81E-01	MUT_p.A289D/Missense
444	RKO_LARGE_INTESTINE	RKO	large_intestine	carcinoma	adenocarcinoma	1.36E-05	3.76E-01	WT
445	HS729_SOFT_TISSUE	Hs 729	soft_tissue	rhabdomyosarcoma	---	7.19E-24	8.20E-01	WT
446	JHOS2_OVARY	JHOS-2	ovary	carcinoma	adenocarcinoma	2.48E-01	-1.33E-01	WT
447	MCF7_BREAST	MCF7	breast	carcinoma	---	2.63E-07	-4.37E-01	WT
448	U937_HAEMATOPOIETIC_AND_LYMPHOID_TISSUE	U-937	haematopoietic_&_lymphoid_tissue	lymphoid_neoplasm	diffuse_large_B-cell_lymphoma	5.35E-03	2.57E-01	WT
449	SKLMS1_SOFT_TISSUE	SK-LMS-1	soft_tissue	sarcoma	---	5.12E-19	7.29E-01	WT
450	HUTU80_SMALL_INTESTINE	HuTu 80	small_intestine	carcinoma	adenocarcinoma	2.12E-14	6.30E-01	WT
451	HCC202_BREAST	HCC202	breast	carcinoma	ductal_carcinoma	4.05E-11	-5.49E-01	WT
452	MEG01_HAEMATOPOIETIC_AND_LYMPHOID_TISSUE	MEG-01	haematopoietic_&_lymphoid_tissue	haematopoietic_neoplasm	blast_phase_chronic_myeloid_leukaemia	6.63E-06	3.88E-01	WT
453	REH_HAEMATOPOIETIC_AND_LYMPHOID_TISSUE	Reh	haematopoietic_&_lymphoid_tissue	lymphoid_neoplasm	acute_lymphoblastic_B-cell_leukaemia	1.19E-04	3.38E-01	WT
454	ONS76_CENTRAL_NERVOUS_SYSTEM	ONS-76	central_nervous_system	primitive_neuro-ectodermal_tumour-medulloblastoma	---	8.65E-09	4.84E-01	WT
455	RPMI6666_HAEMATOPOIETIC_AND_LYMPHOID_TISSUE	RPMI 6666	haematopoietic_&_lymphoid_tissue	lymphoid_neoplasm	Hodgkin_lymphoma	3.78E-02	2.03E-01	WT
456	SNUC1_LARGE_INTESTINE	SNU-C1	large_intestine	carcinoma	adenocarcinoma	3.61E-21	-7.70E-01	WT
457	SKNDZ_AUTONOMIC_GANGLIA	SK-N-DZ	autonomic_ganglia	neuroblastoma	---	1.40E-10	5.35E-01	WT
458	DMS114_LUNG	DMS 114	lung	carcinoma	small_cell_carcinoma	1.87E-15	6.54E-01	WT
459	TT_OESOPHAGUS	T.T	oesophagus	carcinoma	squamous_cell_carcinoma	5.37E-06	-3.91E-01	WT
460	ST486_HAEMATOPOIETIC_AND_LYMPHOID_TISSUE	ST486	haematopoietic_&_lymphoid_tissue	lymphoid_neoplasm	Burkitt_lymphoma	7.24E-02	1.82E-01	WT
461	OC314_OVARY	OC 314	ovary	carcinoma	serous_carcinoma	1.24E-04	3.37E-01	WT
462	SW1353_BONE	SW 1353	bone	chondrosarcoma	---	2.95E-18	7.13E-01	WT
463	MDAMB157_BREAST	MDA-MB-157	breast	carcinoma	ductal_carcinoma	5.65E-19	7.28E-01	WT
464	LN18_CENTRAL_NERVOUS_SYSTEM	LN-18	central_nervous_system	glioma	astrocytoma_GradeIV	1.01E-12	5.90E-01	WT

465	769P_KIDNEY	769-P	kidney	carcinoma	clear_cell_renal_cell_carcinoma	2.74E-05	3.64E-01	WT
466	A2058_SKIN	A2058	skin	malignant_melanoma	---	5.35E-14	6.21E-01	WT
467	786O_KIDNEY	786-O	kidney	carcinoma	clear_cell_renal_cell_carcinoma	5.25E-12	5.72E-01	WT
468	CAOV3_OVARY	Caov-3	ovary	carcinoma	adenocarcinoma	2.22E-01	1.38E-01	MUT_p.R255Q/Missense
469	HEPG2_LIVER	Hep G2	liver	carcinoma	hepato_cellular_carcinoma	4.69E-02	1.96E-01	WT
470	MOLT4_HAEMATOPOIETIC_AND_LYMPHOID_TISSUE	MOLT-4	haematopoietic_&_lymphoid_tissue	lymphoid_neoplasm	acute_lymphoblastic_T-cell_leukaemia	4.24E-04	3.13E-01	WT
471	NCIH524_LUNG	NCI-H524	lung	carcinoma	small_cell_carcinoma	7.00E-08	4.56E-01	WT
472	NCIH209_LUNG	NCI-H209	lung	carcinoma	small_cell_carcinoma	3.97E-01	1.08E-01	WT
473	MIAPACA2_PANCREAS	MIA PaCa-2	pancreas	carcinoma	ductal_carcinoma	3.33E-07	4.34E-01	WT
474	MCAS_OVARY	MCAS	ovary	carcinoma	adenocarcinoma	1.56E-04	-3.32E-01	WT
475	SBC5_LUNG	SBC-5	lung	carcinoma	small_cell_carcinoma	3.08E-10	5.26E-01	WT
476	COLO829_SKIN	COLO 829	skin	malignant_melanoma	---	4.92E-14	6.22E-01	WT
477	VCAP_PROSTATE	VCaP	prostate	carcinoma	adenocarcinoma	6.43E-04	-3.04E-01	WT
478	IPC298_SKIN	IPC-298	skin	malignant_melanoma	---	2.16E-10	5.30E-01	WT
479	CAPAN2_PANCREAS	Capan-2	pancreas	carcinoma	ductal_carcinoma	7.91E-08	-4.54E-01	WT
480	SKMEL28_SKIN	SK-MEL-28	skin	malignant_melanoma	---	5.41E-10	5.19E-01	MUT_p.P753S/Missense
481	EFM192A_BREAST	EFM-192A	breast	carcinoma	---	1.36E-08	-4.78E-01	WT
482	HS766T_PANCREAS	Hs 766T	pancreas	carcinoma	ductal_carcinoma	2.36E-05	3.66E-01	WT
483	EFO27_OVARY	EFO-27	ovary	carcinoma	mucinous_carcinoma	1.87E-03	2.81E-01	WT
484	A2780_OVARY	A2780	ovary	carcinoma	adenocarcinoma	1.80E-17	6.97E-01	WT
485	NCIH196_LUNG	NCI-H196	lung	carcinoma	small_cell_carcinoma	2.83E-19	7.34E-01	WT
486	IGROV1_OVARY	IGROV1	ovary	carcinoma	adenocarcinoma	8.55E-04	2.98E-01	MUT_Intron
487	NCIH889_LUNG	NCI-H889	lung	carcinoma	small_cell_carcinoma	3.01E-04	3.20E-01	WT
488	NCIH211_LUNG	NCI-H211	lung	carcinoma	small_cell_carcinoma	2.70E-01	1.28E-01	WT
489	MDAPCA2B_PROSTATE	MDA PCa 2b	prostate	carcinoma	adenocarcinoma	4.35E-06	-3.95E-01	WT
490	SKMEL24_SKIN	SK-MEL-24	skin	malignant_melanoma	---	1.10E-15	6.59E-01	WT
491	K029AX_SKIN	K029AX	skin	malignant_melanoma	---	1.76E-08	4.74E-01	WT
492	HT1376_URINARY_TRACT	HT-1376	urinary_tract	carcinoma	transitional_cell_carcinoma	1.43E-03	-2.87E-01	WT
493	NCIH2171_LUNG	NCI-H2171	lung	carcinoma	small_cell_carcinoma	1.05E-02	2.40E-01	WT
494	EVSAT_BREAST	EVSA-T	breast	carcinoma	---	3.76E-11	-5.50E-01	MUT_p.E711K/Missense
495	HS695T_SKIN	Hs 695T	skin	malignant_melanoma	---	1.04E-13	6.14E-01	MUT_Intron
496	CHL1_SKIN	CHL-1	skin	malignant_melanoma	---	3.24E-11	5.52E-01	MUT_p.V1011M/Missense
497	LOUNH91_LUNG	LOU-NH91	lung	carcinoma	squamous_cell_carcinoma	4.19E-09	4.93E-01	MUT_p.A755D/Missense
498	LK2_LUNG	LK-2	lung	carcinoma	squamous_cell_carcinoma	3.91E-01	1.09E-01	WT
499	TOV21G_OVARY	TOV-21G	ovary	carcinoma	clear_cell_carcinoma	1.11E-05	3.79E-01	WT
500	SKMEL3_SKIN	SK-MEL-3	skin	malignant_melanoma	---	3.14E-07	4.35E-01	WT
501	ASPC1_PANCREAS	AsPC-1	pancreas	carcinoma	ductal_carcinoma	1.60E-03	-2.85E-01	WT
502	OVSAHO_OVARY	OVSAHO	ovary	carcinoma	adenocarcinoma	9.18E-03	2.43E-01	WT
503	NCIH441_LUNG	NCI-H441	lung	carcinoma	adenocarcinoma	2.08E-07	-4.40E-01	WT
504	RERFLCSQ1_LUNG	RERF-LC-Sq1	lung	carcinoma	squamous_cell_carcinoma	2.62E-16	6.73E-01	WT
505	NCIH2030_LUNG	NCI-H2030	lung	carcinoma	non-small_cell_carcinoma	3.76E-08	4.64E-01	WT
506	HMCB_SKIN	HMCB	skin	malignant_melanoma	---	2.41E-12	5.81E-01	WT
507	FUOV1_OVARY	FU-OV-1	ovary	carcinoma	serous_carcinoma	4.38E-10	5.21E-01	WT
508	MALME3M_SKIN	Malme-3M	skin	malignant_melanoma	---	9.70E-12	5.65E-01	WT
509	KMS12BM_HAEMATOPOIETIC_AND_LYMPHOID_TISSUE	KMS-12-BM	haematopoietic_&_lymphoid_tissue	lymphoid_neoplasm	plasma_cell_myeloma	6.23E-02	1.87E-01	WT
510	T98G_CENTRAL_NERVOUS_SYSTEM	T98G	central_nervous_system	glioma	astrocytoma_GradeIV	6.94E-15	6.41E-01	WT
511	HL60_HAEMATOPOIETIC_AND_LYMPHOID_TISSUE	HL-60	haematopoietic_&_lymphoid_tissue	haematopoietic_neoplasm	acute_myeloid_leukaemia	6.39E-02	1.86E-01	WT
512	DBTRG05MG_CENTRAL_NERVOUS_SYSTEM	DBTRG-05MG	central_nervous_system	glioma	astrocytoma_GradeIV	6.04E-17	6.86E-01	MUT_Intron
513	HEYA8_OVARY	Hey-A8	ovary	carcinoma	---	1.64E-14	6.33E-01	WT
514	NCIH2122_LUNG	NCI-H2122	lung	carcinoma	adenocarcinoma	1.05E-08	-4.81E-01	WT
515	MELJUSO_SKIN	MEL-JUSO	skin	malignant_melanoma	---	2.67E-10	5.27E-01	WT
516	SW620_LARGE_INTESTINE	SW620	large_intestine	carcinoma	adenocarcinoma	1.43E-01	1.56E-01	WT
517	HCT116_LARGE_INTESTINE	HCT 116	large_intestine	carcinoma	---	2.01E-02	2.22E-01	WT
518	VMRCRCZ_KIDNEY	VMRC-RCZ	kidney	carcinoma	renal_cell_carcinoma	3.67E-09	4.95E-01	WT

519	DU145_PROSTATE	DU 145	prostate	carcinoma	---	3.73E-04	3.15E-01	WT
520	MM1S_HAEMATOPOIETIC_AND_LYMPHOID_TISSUE	MM1-S	haematopoietic_&_lymphoid_tissue	lymphoid_neoplasm	plasma_cell_myeloma	8.98E-02	1.74E-01	MUT_p.G917R/Missense
521	L33_PANCREAS	L3.3	pancreas	carcinoma	---	1.31E-02	-2.34E-01	WT
522	SKMEL2_SKIN	SK-MEL-2	skin	malignant_melanoma	---	5.05E-11	5.47E-01	WT
523	A549_LUNG	A549	lung	carcinoma	non-small_cell_carcinoma	7.90E-07	4.21E-01	WT
524	A172_CENTRAL_NERVOUS_SYSTEM	A172	central_nervous_system	glioma	astrocytoma_GradeIV	1.12E-17	7.02E-01	WT
525	NCIH1975_LUNG	NCI-H1975	lung	carcinoma	non-small_cell_carcinoma	2.89E-04	3.21E-01	MUT_p.L858R/Missense
526	COLO679_SKIN	COLO-679	skin	malignant_melanoma	---	1.75E-09	5.04E-01	WT
527	KP2_PANCREAS	KP-2	pancreas	carcinoma	---	4.83E-06	-3.93E-01	WT
528	HUH1_LIVER	huH-1	liver	carcinoma	hepato_cellular_carcinoma	2.55E-02	2.15E-01	WT
529	MDAMB453_BREAST	MDA-MB-453	breast	carcinoma	---	2.41E-12	-5.81E-01	WT
530	PANC0403_PANCREAS	Panc 04.03	pancreas	carcinoma	---	7.15E-05	-3.47E-01	WT
531	TYKNU_OVARY	TYK-nu	ovary	carcinoma	undifferentiated_carcinoma	1.45E-13	6.11E-01	WT
532	OVISE_OVARY	OVISE	ovary	carcinoma	clear_cell_carcinoma	8.57E-02	-1.76E-01	WT
533	EFM19_BREAST	EFM-19	breast	carcinoma	ductal_carcinoma	6.21E-05	-3.49E-01	WT
534	MELHO_SKIN	MEL-HO	skin	malignant_melanoma	---	7.90E-07	4.21E-01	WT
535	RL_HAEMATOPOIETIC_AND_LYMPHOID_TISSUE	RL	haematopoietic_&_lymphoid_tissue	lymphoid_neoplasm	B-cell_lymphoma_unspecified	1.43E-01	1.56E-01	WT
536	G361_SKIN	G-361	skin	malignant_melanoma	---	1.33E-09	5.08E-01	WT
537	NCIH647_LUNG	NCI-H647	lung	carcinoma	mixed_adeno-squamous_carcinoma	1.45E-07	4.46E-01	WT
538	GMS10_CENTRAL_NERVOUS_SYSTEM	GMS-10	central_nervous_system	glioma	astrocytoma_GradeIV	1.12E-17	7.02E-01	WT
539	SEM_HAEMATOPOIETIC_AND_LYMPHOID_TISSUE	SEM	haematopoietic_&_lymphoid_tissue	lymphoid_neoplasm	acute_lymphoblastic_B-cell_leukaemia	4.24E-04	3.13E-01	WT
540	HLFA_LIVER	HLF-a	liver	carcinoma	hepato_cellular_carcinoma	1.56E-19	7.39E-01	WT
541	42MGBA_CENTRAL_NERVOUS_SYSTEM	42-MG-BA	central_nervous_system	glioma	astrocytoma_GradeIV	2.33E-22	7.93E-01	WT
542	HCC44_LUNG	HCC-44	lung	carcinoma	adenocarcinoma	1.84E-07	4.42E-01	WT
543	KYSE70_OESOPHAGUS	KYSE-70	oesophagus	carcinoma	squamous_cell_carcinoma	1.15E-02	2.37E-01	MUT_Intron
544	GAMG_CENTRAL_NERVOUS_SYSTEM	GAMG	central_nervous_system	glioma	---	9.47E-20	7.43E-01	WT
545	NCIH522_LUNG	NCI-H522	lung	carcinoma	non-small_cell_carcinoma	1.87E-09	5.03E-01	WT
546	EN_ENDOMETRIUM	EN	endometrium	carcinoma	---	5.78E-13	5.96E-01	MUT_p.N234D/Missense
547	PK59_PANCREAS	PK-59	pancreas	carcinoma	---	2.26E-03	-2.77E-01	MUT_p.W1157C/Missense
548	SNB19_CENTRAL_NERVOUS_SYSTEM	SNB-19	central_nervous_system	glioma	astrocytoma_GradeIV	1.64E-17	6.98E-01	WT
549	COLO741_SKIN	COLO 741	skin	malignant_melanoma	---	1.28E-08	4.79E-01	WT
550	HDMYZ_HAEMATOPOIETIC_AND_LYMPHOID_TISSUE	HD-MY-Z	haematopoietic_&_lymphoid_tissue	lymphoid_neoplasm	Hodgkin_lymphoma	6.34E-20	7.47E-01	WT
551	NCIH661_LUNG	NCI-H661	lung	carcinoma	large_cell_carcinoma	9.58E-17	6.82E-01	WT
552	LNCAPCLONEFGC_PROSTATE	LNCaP clone FGC	prostate	carcinoma	adenocarcinoma	9.64E-04	-2.96E-01	WT
553	LS411N_LARGE_INTESTINE	LS411N	large_intestine	carcinoma	adenocarcinoma	7.59E-09	-4.86E-01	MUT_p.T940A/Missense
554	HCC78_LUNG	HCC-78	lung	carcinoma	adenocarcinoma	3.28E-04	-3.18E-01	WT
555	YKG1_CENTRAL_NERVOUS_SYSTEM	YKG1	central_nervous_system	glioma	astrocytoma_GradeIV	1.90E-19	7.37E-01	WT
556	ESS1_ENDOMETRIUM	ESS-1	endometrium	carcinoma	carcinosarcoma-malignant_mesodermal_mixed_tumour	1.87E-15	6.54E-01	WT
557	U251MG_CENTRAL_NERVOUS_SYSTEM	U-251 MG	central_nervous_system	glioma	astrocytoma	1.16E-19	7.41E-01	WT
558	TE11_OESOPHAGUS	TE-11	oesophagus	carcinoma	squamous_cell_carcinoma	1.44E-02	-2.31E-01	WT
559	KS1_CENTRAL_NERVOUS_SYSTEM	KS-1	central_nervous_system	glioma	astrocytoma_GradeIV	1.90E-19	7.37E-01	WT
560	HEC1A_ENDOMETRIUM	HEC-1-A	endometrium	carcinoma	adenocarcinoma	2.40E-02	-2.17E-01	WT
561	TE1_OESOPHAGUS	TE-1	oesophagus	carcinoma	squamous_cell_carcinoma	4.45E-02	1.98E-01	WT
562	JHUEM2_ENDOMETRIUM	JHUEM-2	endometrium	carcinoma	adenocarcinoma	4.17E-17	6.90E-01	WT
563	RERFLCAD1_LUNG	RERF-LC-Ad1	lung	carcinoma	adenocarcinoma	1.95E-02	-2.23E-01	WT
564	TE5_OESOPHAGUS	TE-5	oesophagus	carcinoma	squamous_cell_carcinoma	1.43E-03	-2.87E-01	MUT_Intron
565	KELLY_AUTONOMIC_GANGLIA	KELLY	autonomic_ganglia	neuroblastoma	---	4.38E-10	5.21E-01	WT
566	NCIH1155_LUNG	NCI-H1155	lung	carcinoma	large_cell_carcinoma	8.94E-08	4.52E-01	WT
567	TE15_OESOPHAGUS	TE-15	oesophagus	carcinoma	squamous_cell_carcinoma	4.99E-03	-2.59E-01	WT
568	ABC1_LUNG	ABC-1	lung	carcinoma	non-small_cell_carcinoma	2.09E-03	-2.79E-01	WT
569	8MGBA_CENTRAL_NERVOUS_SYSTEM	8-MG-BA	central_nervous_system	glioma	astrocytoma_GradeIV	5.90E-16	6.65E-01	WT
570	NCIH1703_LUNG	NCI-H1703	lung	carcinoma	adenocarcinoma	2.37E-13	6.05E-01	WT
571	AGS_STOMACH	AGS	stomach	carcinoma	adenocarcinoma	6.29E-07	-4.24E-01	WT

572 QGP1_PANCREAS	QGP-1	pancreas	carcinoma	---	3.02E-02	-2.10E-01	WT
573 DETROIT562_UPPER_AERODIGESTIVE_TRACT	Detroit 562	upper_aerodigestive_tract	carcinoma	---	4.02E-01	-1.07E-01	WT
574 BT474_BREAST	BT-474	breast	carcinoma	ductal_carcinoma	4.69E-11	-5.48E-01	WT
575 ACHN_KIDNEY	ACHN	kidney	carcinoma	renal_cell_carcinoma	3.43E-09	4.96E-01	WT
576 LOVO_LARGE_INTESTINE	LoVo	large_intestine	carcinoma	adenocarcinoma	6.14E-03	-2.53E-01	WT
577 NCIH1581_LUNG	NCI-H1581	lung	carcinoma	large_cell_carcinoma	6.22E-10	5.17E-01	WT
578 SCABER_URINARY_TRACT	SCaBER	urinary_tract	carcinoma	transitional_cell_carcinoma	2.57E-01	1.31E-01	WT
579 SKMEL30_SKIN	SK-MEL-30	skin	malignant_melanoma	---	3.49E-11	5.51E-01	WT
580 OVKATE_OVARY	OVKATE	ovary	carcinoma	adenocarcinoma	4.57E-02	-1.97E-01	WT
581 22RV1_PROSTATE	22Rv1	prostate	carcinoma	---	1.08E-02	-2.39E-01	WT
582 C32_SKIN	C32	skin	malignant_melanoma	---	3.01E-11	5.53E-01	WT
583 PANC0327_PANCREAS	Panc 03.27	pancreas	carcinoma	ductal_carcinoma	1.66E-01	-1.51E-01	WT
584 TCCSUP_URINARY_TRACT	TCCSUP	urinary_tract	carcinoma	transitional_cell_carcinoma	5.41E-10	5.19E-01	WT
585 KURAMOCHI_OVARY	KURAMOCHI	ovary	carcinoma	undifferentiated_carcinoma	3.74E-07	4.32E-01	WT
586 RPMI8226_HAEMATOPOIETIC_AND_LYMPHOID_TISSUE	RPMI 8226	haematopoietic_&_lymphoid_tissue	lymphoid_neoplasm	plasma_cell_myeloma	2.95E-01	1.24E-01	MUT_p.T751I/Missense
587 DLD1_LARGE_INTESTINE	DLD-1	large_intestine	carcinoma	adenocarcinoma	2.83E-06	-4.01E-01	WT
588 ES2_OVARY	ES-2	ovary	carcinoma	clear_cell_carcinoma	2.17E-17	6.96E-01	WT
589 NCIH1395_LUNG	NCI-H1395	lung	carcinoma	adenocarcinoma	1.59E-05	-3.73E-01	WT
590 NCIH1781_LUNG	NCI-H1781	lung	carcinoma	bronchiolo_alveolar_adenocarcinoma	2.59E-11	-5.54E-01	WT
591 LS1034_LARGE_INTESTINE	LS1034	large_intestine	carcinoma	adenocarcinoma	9.35E-13	-5.91E-01	WT
592 G402_SOFT_TISSUE	G-402	soft_tissue	rhabdoid_tumour	---	1.91E-12	5.83E-01	WT
593 NCIH146_LUNG	NCI-H146	lung	carcinoma	small_cell_carcinoma	2.52E-01	1.32E-01	WT
594 SW48_LARGE_INTESTINE	SW48	large_intestine	carcinoma	adenocarcinoma	3.15E-04	-3.19E-01	MUT_p.G719S/Missense
595 CHP212_AUTONOMIC_GANGLIA	CHP-212	autonomic_ganglia	neuroblastoma	---	2.87E-16	6.72E-01	WT
596 LS180_LARGE_INTESTINE	LS 180	large_intestine	carcinoma	adenocarcinoma	1.50E-12	-5.86E-01	WT
597 SW403_LARGE_INTESTINE	SW403	large_intestine	carcinoma	adenocarcinoma	1.56E-19	-7.39E-01	WT
598 NCIH747_LARGE_INTESTINE	NCI-H747	large_intestine	carcinoma	adenocarcinoma	1.76E-08	-4.74E-01	MUT_p.E282K/Missense
599 RT4_URINARY_TRACT	RT4	urinary_tract	carcinoma	transitional_cell_carcinoma	4.38E-10	-5.21E-01	WT
600 KATOIII_STOMACH	KATO III	stomach	carcinoma	adenocarcinoma	1.44E-15	-6.56E-01	WT
601 BDCM_HAEMATOPOIETIC_AND_LYMPHOID_TISSUE	BDCM	haematopoietic_&_lymphoid_tissue	lymphoid_neoplasm	acute_lymphoblastic_B-cell_leukaemia	2.40E-02	2.17E-01	WT
602 SW1417_LARGE_INTESTINE	SW1417	large_intestine	carcinoma	adenocarcinoma	1.36E-04	-3.35E-01	WT
603 SW948_LARGE_INTESTINE	SW948	large_intestine	carcinoma	adenocarcinoma	2.39E-16	-6.73E-01	WT
604 HS606T_BREAST	Hs 606.T	breast	carcinoma	---	2.41E-23	8.10E-01	WT
605 MDAMB415_BREAST	MDA-MB-415	breast	carcinoma	---	1.45E-07	-4.46E-01	WT
606 DMS79_LUNG	DMS 79	lung	carcinoma	small_cell_carcinoma	2.99E-01	1.23E-01	WT
607 NCIH2106_LUNG	NCI-H2106	lung	carcinoma	non-small_cell_carcinoma	3.10E-01	-1.22E-01	WT
608 SW579_THYROID	SW579	thyroid	carcinoma	anaplastic_carcinoma	1.03E-21	7.81E-01	WT
609 MDAMB468_BREAST	MDA-MB-468	breast	carcinoma	---	3.97E-07	-4.31E-01	WT
610 NCIH526_LUNG	NCI-H526	lung	carcinoma	small_cell_carcinoma	1.47E-01	-1.56E-01	WT
611 BT549_BREAST	BT-549	breast	carcinoma	ductal_carcinoma	9.27E-18	7.03E-01	WT
612 NCIH2196_LUNG	NCI-H2196	lung	carcinoma	small_cell_carcinoma	1.73E-01	1.49E-01	WT
613 CAL851_BREAST	CAL-85-1	breast	carcinoma	---	5.94E-03	-2.54E-01	WT
614 EFO21_OVARY	EFO-21	ovary	carcinoma	serous_carcinoma	4.58E-06	3.94E-01	WT
615 A375_SKIN	A-375	skin	malignant_melanoma	---	6.94E-15	6.41E-01	WT
616 HT29_LARGE_INTESTINE	HT-29	large_intestine	carcinoma	adenocarcinoma	5.90E-16	-6.65E-01	WT
617 NCIH446_LUNG	NCI-H446	lung	carcinoma	small_cell_carcinoma	3.56E-12	5.77E-01	MUT_Intron
618 HS294T_SKIN	Hs 294T	skin	malignant_melanoma	---	1.91E-12	5.83E-01	WT
619 SKMEL5_SKIN	SK-MEL-5	skin	malignant_melanoma	---	1.76E-08	4.74E-01	WT
620 HS944T_SKIN	Hs 944.T	skin	malignant_melanoma	---	1.38E-14	6.34E-01	MUT_Intron
621 TCCPAN2_PANCREAS	TCC-PAN2	pancreas	carcinoma	---	1.40E-01	-1.57E-01	WT
622 RMGI_OVARY	RMG-I	ovary	carcinoma	clear_cell_carcinoma	1.12E-02	-2.38E-01	WT
623 PC3_PROSTATE	PC-3	prostate	carcinoma	adenocarcinoma	1.78E-02	2.25E-01	WT
624 K562_HAEMATOPOIETIC_AND_LYMPHOID_TISSUE	K-562	haematopoietic_&_lymphoid_tissue	haematopoietic_neoplasm	blast_phase_chronic_myeloid_leukaemia	5.02E-04	3.10E-01	WT

625 RT112_URINARY_TRACT	RT-112	urinary_tract	carcinoma	transitional_cell_carcinoma	3.08E-10	-5.26E-01	WT
626 NUGC3_STOMACH	NUGC-3	stomach	carcinoma	---	5.74E-03	-2.55E-01	WT
627 U87MG_CENTRAL_NERVOUS_SYSTEM	U-87 MG	central_nervous_system	glioma	astrocytoma	6.06E-21	7.66E-01	WT
628 CAKI2_KIDNEY	Caki-2	kidney	carcinoma	clear_cell_renal_cell_carcinoma	4.12E-06	3.95E-01	WT
629 OE19_OESOPHAGUS	OE19	oesophagus	carcinoma	adenocarcinoma	3.46E-20	-7.52E-01	WT
630 KMRC2_KIDNEY	KMRC-2	kidney	carcinoma	clear_cell_renal_cell_carcinoma	1.30E-10	5.36E-01	WT
631 JHH2_LIVER	JHH-2	liver	carcinoma	hepato_cellular_carcinoma	1.65E-08	4.75E-01	WT
632 IM95_STOMACH	IM95	stomach	carcinoma	intestinal_adenocarcinoma	1.72E-15	-6.55E-01	MUT_Intron
633 MV411_HAEMATOPOIETIC_AND_LYMPHOID_TISSUE	MV-4-11	haematopoietic_&_lymphoid_tissue	haematopoietic_neoplasm	acute_myeloid_leukaemia	1.63E-02	2.28E-01	WT
634 WM2664_SKIN	WM-266-4	skin	malignant_melanoma	---	3.23E-14	6.26E-01	WT
635 KMRC1_KIDNEY	KMRC-1	kidney	carcinoma	clear_cell_renal_cell_carcinoma	2.91E-15	6.50E-01	WT
636 KM12_LARGE_INTESTINE	KM12	large_intestine	carcinoma	adenocarcinoma	4.58E-06	-3.94E-01	WT
637 COLO849_SKIN	COLO-849	skin	malignant_melanoma	---	3.30E-12	5.77E-01	WT
638 T84_LARGE_INTESTINE	T84	large_intestine	carcinoma	adenocarcinoma	2.73E-14	-6.28E-01	WT
639 CALU1_LUNG	Calu-1	lung	carcinoma	squamous_cell_carcinoma	2.01E-10	5.31E-01	WT
640 LOXIMVI_SKIN	LOX IMVI	skin	malignant_melanoma	---	2.00E-18	7.17E-01	WT
641 NCIH2170_LUNG	NCI-H2170	lung	carcinoma	squamous_cell_carcinoma	1.75E-09	-5.04E-01	WT
642 SQ1_LUNG	Sq-1	lung	carcinoma	squamous_cell_carcinoma	4.82E-03	-2.59E-01	WT
643 AN3CA_ENDOMETRIUM	AN3 CA	endometrium	carcinoma	adenocarcinoma	7.29E-11	5.43E-01	WT
644 NCIH2444_LUNG	NCI-H2444	lung	carcinoma	non-small_cell_carcinoma	4.93E-01	-9.44E-02	WT
645 UACC257_SKIN	UACC-257	skin	malignant_melanoma	---	1.51E-05	3.74E-01	WT
646 KYSE510_OESOPHAGUS	KYSE-510	oesophagus	carcinoma	squamous_cell_carcinoma	5.21E-02	-1.93E-01	WT
647 MOLM13_HAEMATOPOIETIC_AND_LYMPHOID_TISSUE	MOLM-13	haematopoietic_&_lymphoid_tissue	haematopoietic_neoplasm	acute_myeloid_leukaemia	2.55E-02	2.15E-01	WT
648 HUPT4_PANCREAS	HUP-T4	pancreas	carcinoma	---	2.32E-10	-5.29E-01	WT
649 KYSE30_OESOPHAGUS	KYSE-30	oesophagus	carcinoma	squamous_cell_carcinoma	5.84E-09	-4.89E-01	WT
650 KYSE150_OESOPHAGUS	KYSE-150	oesophagus	carcinoma	squamous_cell_carcinoma	4.21E-02	-2.00E-01	WT
651 PSN1_PANCREAS	PSN1	pancreas	carcinoma	ductal_carcinoma	3.74E-07	4.32E-01	WT
652 NCIH1299_LUNG	NCI-H1299	lung	carcinoma	non-small_cell_carcinoma	2.23E-15	6.52E-01	WT
653 IGR37_SKIN	IGR-37	skin	malignant_melanoma	---	7.91E-08	4.54E-01	MUT_Intron
654 MOLP2_HAEMATOPOIETIC_AND_LYMPHOID_TISSUE	MOLP-2	haematopoietic_&_lymphoid_tissue	lymphoid_neoplasm	plasma_cell_myeloma	2.18E-01	-1.39E-01	WT
655 SCC25_UPPER_AERODIGESTIVE_TRACT	SCC-25	upper_aerodigestive_tract	carcinoma	squamous_cell_carcinoma	7.27E-04	3.02E-01	WT
656 UT7_HAEMATOPOIETIC_AND_LYMPHOID_TISSUE	UT-7	haematopoietic_&_lymphoid_tissue	haematopoietic_neoplasm	acute_myeloid_leukaemia	2.01E-02	2.22E-01	WT
657 MHHNB11_AUTONOMIC_GANGLIA	MHH-NB-11	autonomic_ganglia	neuroblastoma	---	2.61E-12	5.80E-01	WT
658 HCC1806_BREAST	HCC1806	breast	carcinoma	ductal_carcinoma	8.55E-04	-2.98E-01	WT
659 GRANTA519_HAEMATOPOIETIC_AND_LYMPHOID_TISSUE	GRANTA-519	haematopoietic_&_lymphoid_tissue	lymphoid_neoplasm	mantle_cell_lymphoma	8.90E-04	2.98E-01	WT
660 MKN7_STOMACH	MKN7	stomach	carcinoma	tubular_adenocarcinoma	3.85E-01	-1.10E-01	WT
661 SW1710_URINARY_TRACT	SW-1710	urinary_tract	carcinoma	transitional_cell_carcinoma	6.67E-10	5.16E-01	WT
662 MOLT13_HAEMATOPOIETIC_AND_LYMPHOID_TISSUE	MOLT-13	haematopoietic_&_lymphoid_tissue	lymphoid_neoplasm	acute_lymphoblastic_T-cell_leukaemia	8.90E-04	2.98E-01	WT
663 SR786_HAEMATOPOIETIC_AND_LYMPHOID_TISSUE	SR-786	haematopoietic_&_lymphoid_tissue	lymphoid_neoplasm	anaplastic_large_cell_lymphoma	3.43E-04	3.17E-01	WT
664 SCC15_UPPER_AERODIGESTIVE_TRACT	SCC-15	upper_aerodigestive_tract	carcinoma	squamous_cell_carcinoma	3.68E-02	2.04E-01	WT
665 NCIH2126_LUNG	NCI-H2126	lung	carcinoma	adenocarcinoma	1.13E-11	-5.64E-01	WT
666 NCIH2141_LUNG	NCI-H2141	lung	carcinoma	small_cell_carcinoma	6.39E-02	1.86E-01	WT
667 HCC827_LUNG	HCC827	lung	carcinoma	adenocarcinoma	1.32E-01	1.60E-01	MUT_p.ELREA746del/In_Frame_ Deletion
668 RD_SOFT_TISSUE	RD	soft_tissue	rhabdomyosarcoma	embryonal	9.64E-25	8.35E-01	WT
669 TT_THYROID	TT	thyroid	carcinoma	medullary_carcinoma	3.97E-01	1.08E-01	WT
670 TF1_HAEMATOPOIETIC_AND_LYMPHOID_TISSUE	TF-1	haematopoietic_&_lymphoid_tissue	haematopoietic_neoplasm	acute_myeloid_leukaemia	2.13E-04	3.27E-01	WT
671 HS172T_URINARY_TRACT	Hs 172.T	urinary_tract	carcinoma	---	7.75E-20	7.45E-01	WT
672 UACC812_BREAST	UACC-812	breast	carcinoma	ductal_carcinoma	1.87E-09	-5.03E-01	WT
673 ME1_HAEMATOPOIETIC_AND_LYMPHOID_TISSUE	ME-1	haematopoietic_&_lymphoid_tissue	haematopoietic_neoplasm	acute_myeloid_leukaemia	1.09E-03	2.93E-01	WT
674 JEKO1_HAEMATOPOIETIC_AND_LYMPHOID_TISSUE	JeKo-1	haematopoietic_&_lymphoid_tissue	lymphoid_neoplasm	mantle_cell_lymphoma	8.89E-03	2.44E-01	WT
675 NUDHL1_HAEMATOPOIETIC_AND_LYMPHOID_TISSUE	NU-DHL-1	haematopoietic_&_lymphoid_tissue	lymphoid_neoplasm	diffuse_large_B-cell_lymphoma	5.49E-02	1.91E-01	WT
676 KYO1_HAEMATOPOIETIC_AND_LYMPHOID_TISSUE	KYO-1	haematopoietic_&_lymphoid_tissue	haematopoietic_neoplasm	blast_phase_chronic_myeloid_leukaemia	2.62E-03	2.74E-01	WT

677 NALM19_HAEMATOPOIETIC_AND_LYMPHOID_TISSUE	NALM-19	haematopoietic_&_lymphoid_tissue	lymphoid_neoplasm	acute_lymphoblastic_B-cell_leukaemia	1.26E-01	1.62E-01	WT
678 HPBALL_HAEMATOPOIETIC_AND_LYMPHOID_TISSUE	HPB-ALL	haematopoietic_&_lymphoid_tissue	lymphoid_neoplasm	acute_lymphoblastic_T-cell_leukaemia	1.67E-05	3.72E-01	WT
679 SUPT11_HAEMATOPOIETIC_AND_LYMPHOID_TISSUE	SUP-T11	haematopoietic_&_lymphoid_tissue	lymphoid_neoplasm	peripheral_T-cell_lymphoma_unspecified	1.06E-05	3.80E-01	WT
680 MDAMB134VI_BREAST	MDA-MB-134-VI	breast	carcinoma	ductal_carcinoma	4.61E-04	-3.11E-01	WT
681 HCC1500_BREAST	HCC1500	breast	carcinoma	ductal_carcinoma	4.58E-06	-3.94E-01	WT
682 HCC1599_BREAST	HCC1599	breast	carcinoma	ductal_carcinoma	1.78E-02	-2.25E-01	WT
683 SUPHD1_HAEMATOPOIETIC_AND_LYMPHOID_TISSUE	SUP-HD1	haematopoietic_&_lymphoid_tissue	lymphoid_neoplasm	Hodgkin_lymphoma	3.90E-03	2.64E-01	MUT_p.S811F/Missense
684 OCIAML3_HAEMATOPOIETIC_AND_LYMPHOID_TISSUE	OCI-AML3	haematopoietic_&_lymphoid_tissue	haematopoietic_neoplasm	acute_myeloid_leukaemia	7.49E-05	3.46E-01	WT
685 SUDHL10_HAEMATOPOIETIC_AND_LYMPHOID_TISSUE	SU-DHL-10	haematopoietic_&_lymphoid_tissue	lymphoid_neoplasm	diffuse_large_B-cell_lymphoma	1.83E-01	1.46E-01	WT
686 SUDHL4_HAEMATOPOIETIC_AND_LYMPHOID_TISSUE	SU-DHL-4	haematopoietic_&_lymphoid_tissue	lymphoid_neoplasm	diffuse_large_B-cell_lymphoma	3.88E-02	2.02E-01	WT
687 OCILY19_HAEMATOPOIETIC_AND_LYMPHOID_TISSUE	OCI-LY-19	haematopoietic_&_lymphoid_tissue	lymphoid_neoplasm	diffuse_large_B-cell_lymphoma	3.73E-04	3.15E-01	WT
688 MOLM6_HAEMATOPOIETIC_AND_LYMPHOID_TISSUE	MOLM-6	haematopoietic_&_lymphoid_tissue	haematopoietic_neoplasm	blast_phase_chronic_myeloid_leukaemia	2.94E-02	2.11E-01	WT
689 SKMM2_HAEMATOPOIETIC_AND_LYMPHOID_TISSUE	SK-MM-2	haematopoietic_&_lymphoid_tissue	lymphoid_neoplasm	plasma_cell_myeloma	1.50E-01	1.55E-01	WT
690 KASUMI6_HAEMATOPOIETIC_AND_LYMPHOID_TISSUE	Kasumi-6	haematopoietic_&_lymphoid_tissue	haematopoietic_neoplasm	acute_myeloid_leukaemia	1.89E-02	2.24E-01	WT
691 HS698T_LARGE_INTESTINE	Hs 698.T	large_intestine	carcinoma	adenocarcinoma	1.25E-20	7.60E-01	WT
692 MINO_HAEMATOPOIETIC_AND_LYMPHOID_TISSUE	Mino	haematopoietic_&_lymphoid_tissue	lymphoid_neoplasm	mantle_cell_lymphoma	3.03E-03	2.70E-01	WT
693 CHP126_AUTONOMIC_GANGLIA	CHP-126	autonomic_ganglia	neuroblastoma	---	5.80E-10	5.18E-01	WT
694 COLO680N_OESOPHAGUS	COLO-680N	oesophagus	carcinoma	squamous_cell_carcinoma	2.70E-01	-1.28E-01	WT
695 COLO678_LARGE_INTESTINE	COLO-678	large_intestine	carcinoma	adenocarcinoma	2.42E-08	-4.70E-01	WT
696 LAMA84_HAEMATOPOIETIC_AND_LYMPHOID_TISSUE	LAMA-84	haematopoietic_&_lymphoid_tissue	haematopoietic_neoplasm	blast_phase_chronic_myeloid_leukaemia	2.43E-03	2.76E-01	WT
697 CMLT1_HAEMATOPOIETIC_AND_LYMPHOID_TISSUE	CML-T1	haematopoietic_&_lymphoid_tissue	haematopoietic_neoplasm	blast_phase_chronic_myeloid_leukaemia	1.94E-03	2.81E-01	WT
698 EHEB_HAEMATOPOIETIC_AND_LYMPHOID_TISSUE	EHEB	haematopoietic_&_lymphoid_tissue	lymphoid_neoplasm	chronic_lymphocytic_leukaemia-small_lymphocytic_lymphoma	7.42E-02	1.81E-01	WT
699 CAL54_KIDNEY	CAL-54	kidney	carcinoma	renal_cell_carcinoma	3.85E-05	3.58E-01	WT
700 BV173_HAEMATOPOIETIC_AND_LYMPHOID_TISSUE	BV-173	haematopoietic_&_lymphoid_tissue	haematopoietic_neoplasm	blast_phase_chronic_myeloid_leukaemia	2.09E-03	2.79E-01	WT
701 DOHH2_HAEMATOPOIETIC_AND_LYMPHOID_TISSUE	DOHH-2	haematopoietic_&_lymphoid_tissue	lymphoid_neoplasm	diffuse_large_B-cell_lymphoma	9.20E-02	1.73E-01	WT
702 EOL1_HAEMATOPOIETIC_AND_LYMPHOID_TISSUE	EOL-1	haematopoietic_&_lymphoid_tissue	haematopoietic_neoplasm	acute_myeloid_leukaemia	3.90E-03	2.64E-01	WT
703 NALM6_HAEMATOPOIETIC_AND_LYMPHOID_TISSUE	NALM-6	haematopoietic_&_lymphoid_tissue	lymphoid_neoplasm	acute_lymphoblastic_B-cell_leukaemia	5.97E-06	3.89E-01	MUT_p.R1068*/Nonsense
704 VMCUB1_URINARY_TRACT	VM-CUB1	urinary_tract	carcinoma	transitional_cell_carcinoma	1.67E-03	-2.84E-01	WT
705 LP1_HAEMATOPOIETIC_AND_LYMPHOID_TISSUE	LP-1	haematopoietic_&_lymphoid_tissue	lymphoid_neoplasm	plasma_cell_myeloma	4.06E-04	3.14E-01	WT
706 LXF289_LUNG	LXF-289	lung	carcinoma	adenocarcinoma	1.71E-13	6.09E-01	WT
707 PL21_HAEMATOPOIETIC_AND_LYMPHOID_TISSUE	PL-21	haematopoietic_&_lymphoid_tissue	haematopoietic_neoplasm	acute_myeloid_leukaemia	4.69E-02	1.96E-01	WT
708 PF382_HAEMATOPOIETIC_AND_LYMPHOID_TISSUE	PF-382	haematopoietic_&_lymphoid_tissue	lymphoid_neoplasm	acute_lymphoblastic_T-cell_leukaemia	5.68E-04	3.07E-01	WT
709 NALM1_HAEMATOPOIETIC_AND_LYMPHOID_TISSUE	NALM-1	haematopoietic_&_lymphoid_tissue	haematopoietic_neoplasm	blast_phase_chronic_myeloid_leukaemia	1.48E-03	2.87E-01	WT
710 OCIAML2_HAEMATOPOIETIC_AND_LYMPHOID_TISSUE	OCI-AML2	haematopoietic_&_lymphoid_tissue	haematopoietic_neoplasm	acute_myeloid_leukaemia	2.82E-03	2.72E-01	WT
711 MHHCALL3_HAEMATOPOIETIC_AND_LYMPHOID_TISSUE	MHH-CALL-3	haematopoietic_&_lymphoid_tissue	lymphoid_neoplasm	acute_lymphoblastic_B-cell_leukaemia	1.18E-03	2.92E-01	WT
712 JURLMK1_HAEMATOPOIETIC_AND_LYMPHOID_TISSUE	JURL-MK1	haematopoietic_&_lymphoid_tissue	haematopoietic_neoplasm	blast_phase_chronic_myeloid_leukaemia	1.14E-07	4.49E-01	WT
713 OCIAML5_HAEMATOPOIETIC_AND_LYMPHOID_TISSUE	OCI-AML5	haematopoietic_&_lymphoid_tissue	haematopoietic_neoplasm	acute_myeloid_leukaemia	3.26E-03	2.69E-01	WT
714 KE37_HAEMATOPOIETIC_AND_LYMPHOID_TISSUE	KE-37	haematopoietic_&_lymphoid_tissue	haematopoietic_neoplasm	acute_lymphoblastic_leukaemia	1.44E-02	2.31E-01	WT
715 JK1_HAEMATOPOIETIC_AND_LYMPHOID_TISSUE	JK-1	haematopoietic_&_lymphoid_tissue	haematopoietic_neoplasm	blast_phase_chronic_myeloid_leukaemia	9.49E-03	2.42E-01	WT
716 RI1_HAEMATOPOIETIC_AND_LYMPHOID_TISSUE	RI-1	haematopoietic_&_lymphoid_tissue	lymphoid_neoplasm	B-cell_lymphoma_unspecified	2.66E-01	1.29E-01	WT
717 L540_HAEMATOPOIETIC_AND_LYMPHOID_TISSUE	L-540	haematopoietic_&_lymphoid_tissue	lymphoid_neoplasm	Hodgkin_lymphoma	1.73E-03	2.83E-01	WT
718 CAL78_BONE	CAL-78	bone	chondrosarcoma	dedifferentiated	7.14E-23	8.02E-01	WT
719 HDQP1_BREAST	HDQ-P1	breast	carcinoma	ductal_carcinoma	1.23E-01	-1.62E-01	WT
720 MHHCALL4_HAEMATOPOIETIC_AND_LYMPHOID_TISSUE	MHH-CALL-4	haematopoietic_&_lymphoid_tissue	lymphoid_neoplasm	acute_lymphoblastic_B-cell_leukaemia	3.78E-02	2.03E-01	WT
721 MUTZ5_HAEMATOPOIETIC_AND_LYMPHOID_TISSUE	MUTZ-5	haematopoietic_&_lymphoid_tissue	lymphoid_neoplasm	acute_lymphoblastic_B-cell_leukaemia	3.48E-02	2.06E-01	WT

722	HDLM2_HAEMATOPOIETIC_AND_LYMPHOID_TISSUE	HDLM-2	haematopoietic_&_lymphoid_tissue	lymphoid_neoplasm	Hodgkin_lymphoma	1.01E-02	2.41E-01	WT
723	CAL29_URINARY_TRACT	CAL-29	urinary_tract	carcinoma	transitional_cell_carcinoma	4.99E-03	-2.59E-01	WT
724	KOPN8_HAEMATOPOIETIC_AND_LYMPHOID_TISSUE	KOPN-8	haematopoietic_&_lymphoid_tissue	lymphoid_neoplasm	acute_lymphoblastic_B-cell_leukaemia	1.27E-02	2.35E-01	WT
725	SCLC21H_LUNG	SCLC-21H	lung	carcinoma	small_cell_carcinoma	2.86E-02	2.12E-01	WT
726	CALU3_LUNG	Calu-3	lung	carcinoma	adenocarcinoma	1.18E-03	-2.92E-01	WT
727	NCIH1435_LUNG	NCI-H1435	lung	carcinoma	non-small_cell_carcinoma	3.14E-07	-4.35E-01	WT
728	NCIH2009_LUNG	NCI-H2009	lung	carcinoma	adenocarcinoma	1.53E-01	1.54E-01	MUT_Intron
729	HCC1419_BREAST	HCC1419	breast	carcinoma	ductal_carcinoma	1.19E-12	-5.88E-01	WT
730	BT483_BREAST	BT-483	breast	carcinoma	ductal_carcinoma	1.13E-10	-5.37E-01	WT
731	HS281T_BREAST	Hs 281.T	breast	carcinoma	---	8.03E-24	8.19E-01	WT
732	HS934T_SKIN	Hs 934.T	skin	malignant_melanoma	---	7.14E-23	8.02E-01	WT
733	HCC4006_LUNG	HCC4006	lung	carcinoma	adenocarcinoma	1.23E-02	-2.36E-01	MUT_p.ELR746del/In_Frame_Deletion
734	KU812_HAEMATOPOIETIC_AND_LYMPHOID_TISSUE	KU812	haematopoietic_&_lymphoid_tissue	haematopoietic_neoplasm	chronic_myeloid_leukaemia	3.29E-02	2.07E-01	WT
735	SHP77_LUNG	SHP-77	lung	carcinoma	small_cell_carcinoma	1.08E-04	3.39E-01	WT
736	TE441T_SOFT_TISSUE	TE 441.T	soft_tissue	rhabdomyosarcoma	---	1.31E-11	5.62E-01	WT
737	NB1_AUTONOMIC_GANGLIA	NB-1	autonomic_ganglia	neuroblastoma	---	6.27E-13	5.95E-01	WT
738	HS274T_BREAST	Hs 274.T	breast	carcinoma	---	1.65E-18	7.19E-01	WT
739	PFEIFFER_HAEMATOPOIETIC_AND_LYMPHOID_TISSUE	Pfeiffer	haematopoietic_&_lymphoid_tissue	lymphoid_neoplasm	diffuse_large_B-cell_lymphoma	1.18E-01	1.64E-01	WT
740	ZR7530_BREAST	ZR-75-30	breast	carcinoma	ductal_carcinoma	9.23E-09	-4.83E-01	WT
741	HS863T_BONE	Hs 863.T	bone	Ewings_sarcoma-peripheral_primitive_neuro_ectodermal_tumour	---	9.27E-22	7.81E-01	WT
742	NCIH841_LUNG	NCI-H841	lung	carcinoma	small_cell_carcinoma	1.87E-15	6.54E-01	WT
743	HS821T_BONE	Hs 821.T	bone	giant_cell_tumour	---	4.45E-21	7.69E-01	WT
744	NCIH1944_LUNG	NCI-H1944	lung	carcinoma	non-small_cell_carcinoma	1.29E-01	1.61E-01	WT
745	NCIH2405_LUNG	NCI-H2405	lung	carcinoma	adenocarcinoma	2.13E-08	4.72E-01	WT
746	DEL_HAEMATOPOIETIC_AND_LYMPHOID_TISSUE	DEL	haematopoietic_&_lymphoid_tissue	lymphoid_neoplasm	---	3.58E-04	3.16E-01	WT
747	BEN_LUNG	BEN	lung	carcinoma	---	1.94E-03	-2.81E-01	WT
748	COLO320_LARGE_INTESTINE	COLO-320	large_intestine	carcinoma	adenocarcinoma	1.88E-08	4.74E-01	WT
749	RPMI8402_HAEMATOPOIETIC_AND_LYMPHOID_TISSUE	RPMI-8402	haematopoietic_&_lymphoid_tissue	lymphoid_neoplasm	acute_lymphoblastic_T-cell_leukaemia	1.94E-03	2.81E-01	WT
750	SUDHL1_HAEMATOPOIETIC_AND_LYMPHOID_TISSUE	SU-DHL-1	haematopoietic_&_lymphoid_tissue	lymphoid_neoplasm	anaplastic_large_cell_lymphoma	2.54E-06	4.03E-01	MUT_p.R98Q/Missense
751	P12ICHIKAWA_HAEMATOPOIETIC_AND_LYMPHOID_TISSUE	P12-ICHIKAWA	haematopoietic_&_lymphoid_tissue	lymphoid_neoplasm	acute_lymphoblastic_T-cell_leukaemia	6.36E-03	2.53E-01	MUT_Intron
752	BFTC905_URINARY_TRACT	BFTC-905	urinary_tract	carcinoma	transitional_cell_carcinoma	4.24E-04	-3.13E-01	WT
753	BFTC909_KIDNEY	BFTC-909	kidney	carcinoma	---	1.41E-19	7.40E-01	WT
754	MHHES1_BONE	MHH-ES-1	bone	Ewings_sarcoma-peripheral_primitive_neuro_ectodermal_tumour	---	3.85E-12	5.76E-01	WT
755	JVM3_HAEMATOPOIETIC_AND_LYMPHOID_TISSUE	JVM-3	haematopoietic_&_lymphoid_tissue	lymphoid_neoplasm	chronic_lymphocytic_leukaemia-small_lymphocytic_lymphoma	6.14E-03	2.53E-01	WT
756	NOMO1_HAEMATOPOIETIC_AND_LYMPHOID_TISSUE	NOMO-1	haematopoietic_&_lymphoid_tissue	haematopoietic_neoplasm	acute_myeloid_leukaemia	2.30E-01	1.36E-01	WT
757	NCIH727_LUNG	NCI-H727	lung	carcinoid-endocrine_tumour	---	1.27E-02	-2.35E-01	WT
758	HS343T_BREAST	Hs 343.T	breast	carcinoma	---	8.03E-24	8.19E-01	WT
759	HS888T_BONE	Hs 888.T	bone	osteosarcoma	---	1.03E-21	7.81E-01	WT
760	MKN45_STOMACH	MKN-45	stomach	carcinoma	diffuse_adenocarcinoma	2.79E-07	-4.36E-01	WT
761	8505C_THYROID	8505C	thyroid	carcinoma	anaplastic_carcinoma	8.99E-12	5.66E-01	WT
762	KU1919_URINARY_TRACT	KU-19-19	urinary_tract	carcinoma	transitional_cell_carcinoma	2.78E-02	2.13E-01	WT
763	KCL22_HAEMATOPOIETIC_AND_LYMPHOID_TISSUE	KCL-22	haematopoietic_&_lymphoid_tissue	haematopoietic_neoplasm	blast_phase_chronic_myeloid_leukaemia	5.97E-06	3.89E-01	WT
764	CADOES1_BONE	CADO-ES1	bone	Ewings_sarcoma-peripheral_primitive_neuro_ectodermal_tumour	---	5.25E-12	5.72E-01	WT
765	S117_SOFT_TISSUE	S-117	soft_tissue	sarcoma	---	1.02E-17	7.02E-01	WT
766	EM2_HAEMATOPOIETIC_AND_LYMPHOID_TISSUE	EM-2	haematopoietic_&_lymphoid_tissue	haematopoietic_neoplasm	blast_phase_chronic_myeloid_leukaemia	1.10E-01	1.67E-01	WT
767	OPM2_HAEMATOPOIETIC_AND_LYMPHOID_TISSUE	OPM-2	haematopoietic_&_lymphoid_tissue	lymphoid_neoplasm	plasma_cell_myeloma	2.78E-02	2.13E-01	WT
768	CAL62_THYROID	CAL-62	thyroid	carcinoma	anaplastic_carcinoma	2.06E-12	5.82E-01	WT

769	LCLC103H_LUNG	LCLC-103H	lung	carcinoma	large_cell_carcinoma	8.99E-12	5.66E-01	WT
770	JURKAT_HAEMATOPOIETIC_AND_LYMPHOID_TISSUE	JURKAT	haematopoietic_&_lymphoid_tissue	lymphoid_neoplasm	acute_lymphoblastic_T-cell_leukaemia	5.68E-04	3.07E-01	WT
771	697_HAEMATOPOIETIC_AND_LYMPHOID_TISSUE	697	haematopoietic_&_lymphoid_tissue	haematopoietic_neoplasm	acute_lymphoblastic_leukaemia	3.33E-05	3.61E-01	WT
772	MHHCALL2_HAEMATOPOIETIC_AND_LYMPHOID_TISSUE	MHH-CALL-2	haematopoietic_&_lymphoid_tissue	lymphoid_neoplasm	acute_lymphoblastic_B-cell_leukaemia	1.43E-03	2.87E-01	WT
773	M07E_HAEMATOPOIETIC_AND_LYMPHOID_TISSUE	M-07e	haematopoietic_&_lymphoid_tissue	haematopoietic_neoplasm	acute_myeloid_leukaemia	1.13E-03	2.93E-01	WT
774	L428_HAEMATOPOIETIC_AND_LYMPHOID_TISSUE	L-428	haematopoietic_&_lymphoid_tissue	lymphoid_neoplasm	Hodgkin_lymphoma	6.70E-04	3.04E-01	WT
775	MOLT16_HAEMATOPOIETIC_AND_LYMPHOID_TISSUE	MOLT-16	haematopoietic_&_lymphoid_tissue	lymphoid_neoplasm	acute_lymphoblastic_T-cell_leukaemia	2.92E-03	2.71E-01	MUT_p.T39T/Silent
776	NB4_HAEMATOPOIETIC_AND_LYMPHOID_TISSUE	NB-4	haematopoietic_&_lymphoid_tissue	haematopoietic_neoplasm	acute_myeloid_leukaemia	1.04E-03	2.94E-01	WT
777	LCLC97TM1_LUNG	LCLC-97TM1	lung	carcinoma	large_cell_carcinoma	4.57E-02	-1.97E-01	WT
778	2313287_STOMACH	23132/87	stomach	carcinoma	adenocarcinoma	3.46E-17	-6.91E-01	WT
779	BHT101_THYROID	BHT-101	thyroid	carcinoma	anaplastic_carcinoma	1.00E-05	3.81E-01	WT
780	JVM2_HAEMATOPOIETIC_AND_LYMPHOID_TISSUE	JVM-2	haematopoietic_&_lymphoid_tissue	lymphoid_neoplasm	chronic_lymphocytic_leukaemia-small_lymphocytic_lymphoma	6.39E-02	1.86E-01	WT
781	CAL51_BREAST	CAL-51	breast	carcinoma	---	1.36E-05	3.76E-01	WT
782	L363_HAEMATOPOIETIC_AND_LYMPHOID_TISSUE	L-363	haematopoietic_&_lymphoid_tissue	lymphoid_neoplasm	plasma_cell_myeloma	2.53E-04	3.23E-01	WT
783	KMH2_HAEMATOPOIETIC_AND_LYMPHOID_TISSUE	KM-H2	haematopoietic_&_lymphoid_tissue	lymphoid_neoplasm	Hodgkin_lymphoma	3.50E-05	3.60E-01	MUT_p.W1157C/Missense
784	SIMA_AUTONOMIC_GANGLIA	SIMA	autonomic_ganglia	neuroblastoma	---	1.92E-11	5.58E-01	WT
785	BC3C_URINARY_TRACT	BC-3C	urinary_tract	carcinoma	transitional_cell_carcinoma	7.36E-06	3.86E-01	WT
786	SUDHL6_HAEMATOPOIETIC_AND_LYMPHOID_TISSUE	SU-DHL-6	haematopoietic_&_lymphoid_tissue	lymphoid_neoplasm	diffuse_large_B-cell_lymphoma	3.38E-02	2.07E-01	WT
787	KARPAS620_HAEMATOPOIETIC_AND_LYMPHOID_TISSUE	KARPAS-620	haematopoietic_&_lymphoid_tissue	lymphoid_neoplasm	plasma_cell_myeloma	3.68E-02	2.04E-01	WT
788	REC1_HAEMATOPOIETIC_AND_LYMPHOID_TISSUE	REC-1	haematopoietic_&_lymphoid_tissue	lymphoid_neoplasm	mantle_cell_lymphoma	4.06E-04	3.14E-01	WT
789	OCIM1_HAEMATOPOIETIC_AND_LYMPHOID_TISSUE	OCI-M1	haematopoietic_&_lymphoid_tissue	haematopoietic_neoplasm	acute_myeloid_leukaemia	4.04E-03	2.64E-01	WT
790	SIGM5_HAEMATOPOIETIC_AND_LYMPHOID_TISSUE	SIG-M5	haematopoietic_&_lymphoid_tissue	haematopoietic_neoplasm	acute_myeloid_leukaemia	1.39E-02	2.32E-01	WT
791	SUDHL5_HAEMATOPOIETIC_AND_LYMPHOID_TISSUE	SU-DHL-5	haematopoietic_&_lymphoid_tissue	lymphoid_neoplasm	diffuse_large_B-cell_lymphoma	6.23E-02	1.87E-01	WT
792	BCPAP_THYROID	B-CPAP	thyroid	carcinoma	papillary_carcinoma	9.70E-12	5.65E-01	WT
793	CL11_LARGE_INTESTINE	CL-11	large_intestine	carcinoma	adenocarcinoma	4.38E-01	-1.02E-01	WT
794	TT2609C02_THYROID	TT2609-C02	thyroid	carcinoma	follicular_carcinoma	7.57E-15	6.40E-01	WT
795	MEC2_HAEMATOPOIETIC_AND_LYMPHOID_TISSUE	MEC-2	haematopoietic_&_lymphoid_tissue	lymphoid_neoplasm	chronic_lymphocytic_leukaemia-small_lymphocytic_lymphoma	4.57E-02	1.97E-01	WT
796	CL34_LARGE_INTESTINE	CL-34	large_intestine	carcinoma	adenocarcinoma	1.99E-16	-6.75E-01	WT
797	RCHACV_HAEMATOPOIETIC_AND_LYMPHOID_TISSUE	RCH-ACV	haematopoietic_&_lymphoid_tissue	lymphoid_neoplasm	acute_lymphoblastic_leukaemia	5.37E-06	3.91E-01	WT
798	CAL120_BREAST	CAL-120	breast	carcinoma	---	1.13E-10	5.37E-01	WT
799	647V_URINARY_TRACT	647-V	urinary_tract	carcinoma	transitional_cell_carcinoma	3.41E-01	1.16E-01	WT
800	MOLM16_HAEMATOPOIETIC_AND_LYMPHOID_TISSUE	MOLM-16	haematopoietic_&_lymphoid_tissue	haematopoietic_neoplasm	acute_myeloid_leukaemia	2.89E-04	3.21E-01	WT
801	F36P_HAEMATOPOIETIC_AND_LYMPHOID_TISSUE	F-36P	haematopoietic_&_lymphoid_tissue	haematopoietic_neoplasm	acute_myeloid_leukaemia	1.27E-03	2.90E-01	WT
802	EJM_HAEMATOPOIETIC_AND_LYMPHOID_TISSUE	EJM	haematopoietic_&_lymphoid_tissue	lymphoid_neoplasm	plasma_cell_myeloma	1.94E-01	1.44E-01	WT
803	WSUDLCL2_HAEMATOPOIETIC_AND_LYMPHOID_TISSUE	WSU-DLCL2	haematopoietic_&_lymphoid_tissue	lymphoid_neoplasm	diffuse_large_B-cell_lymphoma	2.07E-02	2.21E-01	WT
804	JIMT1_BREAST	JIMT-1	breast	carcinoma	ductal_carcinoma	2.43E-01	1.34E-01	WT
805	ML1_THYROID	ML-1	thyroid	carcinoma	follicular_carcinoma	8.33E-12	5.67E-01	WT
806	AMO1_HAEMATOPOIETIC_AND_LYMPHOID_TISSUE	AMO-1	haematopoietic_&_lymphoid_tissue	lymphoid_neoplasm	plasma_cell_myeloma	5.92E-02	1.89E-01	MUT_p.K209N/Missense
807	MOLP8_HAEMATOPOIETIC_AND_LYMPHOID_TISSUE	MOLP-8	haematopoietic_&_lymphoid_tissue	lymphoid_neoplasm	plasma_cell_myeloma	1.67E-03	2.84E-01	WT

Abbreviation: WT, wild type; MUT, Mutant;

Table S4C. EMT score (generic cell line EMT signature) of SANGER/COSMIC cell line data.

Index	Source Name	CosmicID	Label	Disease	Histology	PrimarySite	PrimaryHist	Generic.EMT.CL.pv	Generic.EMT.CL.Ksscore	EGFR.Mutation_Amino Acid/Type
1	BxPC-3	906693	pancreatic	pancreas	Carcinoma	pancreas	carcinoma	2.58E-08	-4.69E-01	WT
2	KMOE-2	907280	H.L.M.	AML	AML	haematopoietic and lymphoid tissue	haematopoietic neoplasm	1.15E-02	2.37E-01	WT
3	MFM-223	910948	breast	breast	DuctalCarcinoma	breast	carcinoma	5.00E-07	-4.28E-01	---
4	NUGC-3	908455	gastric	stomach	Carcinoma	stomach	carcinoma	1.36E-05	-3.76E-01	WT
5	OC-314	909257	ovarian	ovary	Carcinoma	ovary	carcinoma	2.65E-04	3.22E-01	WT
6	COLO-741	906815	colorectal	large_intestine	Carcinoma	large intestine	carcinoma	7.00E-08	4.56E-01	WT
7	KARPAS-45	907272	H.L.M.	lymphoblastic Leukaemia	ALL	haematopoietic and lymphoid tissue	haematopoietic neoplasm	1.14E-04	3.38E-01	WT
8	JAR	907175	---	other	Carcinoma	placenta	choriocarcinoma	9.63E-02	-1.72E-01	WT
9	DU-4475	906844	breast	breast	DuctalCarcinoma	breast	carcinoma	2.02E-01	-1.42E-01	---
10	MONO-MAC-6	908148	H.L.M.	AML	AML	haematopoietic and lymphoid tissue	haematopoietic neoplasm	7.53E-03	2.48E-01	WT
11	SKM-1	909722	H.L.M.	haematopoietic_neoplasm other	MDS	haematopoietic and lymphoid tissue	haematopoietic neoplasm	5.37E-06	3.91E-01	WT
12	TGBC11TKB	909770	gastric	stomach	Carcinoma	stomach	carcinoma	2.48E-07	-4.38E-01	WT
13	CCF-STTG1	906823	CNS	glioma	Glioma	central nervous system	glioma	3.13E-19	7.33E-01	WT
14	RKO	909698	colorectal	large_intestine	Carcinoma	large intestine	carcinoma	1.30E-04	3.36E-01	WT
15	LXF-289	753592	lung	lung: NSCLC: adenocarcinoma	AdenoCarcinoma	lung	carcinoma	1.65E-11	5.60E-01	WT
16	IMR-5	907170	CNS	neuroblastoma	Neuroblastoma	autonomic ganglia	neuroblastoma	3.05E-12	5.78E-01	WT
17	L-540	907323	H.L.M.	Hodgkin lymphoma	HodgekinsLymphoma	haematopoietic and lymphoid tissue	lymphoid neoplasm	9.80E-03	2.41E-01	WT
18	MDA-MB-468	908123	breast	breast	Carcinoma	breast	carcinoma	1.39E-06	-4.12E-01	WT
19	MHH-NB-11	908135	CNS	neuroblastoma	Neuroblastoma	autonomic ganglia	neuroblastoma	3.85E-12	5.76E-01	WT
20	MHH-CALL-4	908133	H.L.M.	ALL	ALL	haematopoietic and lymphoid tissue	lymphoid neoplasm	3.20E-02	2.08E-01	WT
21	NCI-H508	908442	colorectal	large instestine	AdenoCarcinoma	large intestine	carcinoma	3.15E-17	-6.92E-01	WT
22	NB4	924050	H.L.M.	AML	AML	haematopoietic and lymphoid tissue	he	1.00E-03	2.95E-01	---
23	P31-FUJ	909253	H.L.M.	AML	AML	haematopoietic and lymphoid tissue	haematopoietic neoplasm	9.49E-03	2.42E-01	WT
24	KMS-12-PE	753568	H.L.M.	Myeloma	Myeloma	haematopoietic and lymphoid tissue	lymphoid neoplasm	3.20E-02	2.08E-01	WT
25	MKN1	908138	gastric	stomach	Carcinoma	stomach	carcinoma	9.53E-06	3.82E-01	WT
26	KARPAS-299	907273	H.L.M.	lymphoid_neoplasm other	Lymphoma	haematopoietic and lymphoid tissue	lymphoid neoplasm	1.43E-03	2.87E-01	WT
27	CFPAC-1	906821	pancreatic	pancreas	DuctalCarcinoma	pancreas	carcinoma	1.83E-06	-4.08E-01	WT
28	SCC-9	909709	head & neck	upper_aerodigestive_tract	Squamous	upper aerodigestive tract	carcinoma	7.88E-04	-3.00E-01	WT

29	KNS-62		753569	lung	lung: NSCLC: squamous_cell_carcinoma	Squamous	lung	carcinoma	1.09E-03	-2.93E-01	WT
30	LAN-1		801740	CNS	neuroblastoma	Neuroblastoma	autonomic ganglia	neuroblastoma	6.19E-08	4.57E-01	---
31	CRO-AP5		906806	H.L.M.	B-cell Lymphoma	Lymphoma	haematopoietic and lymphoid tissue	lymphoid neoplasm	1.98E-01	1.43E-01	WT
32	KMS-12-BM	---		H.L.M.	Myeloma	Myeloma	haematopoietic and lymphoid tissue	lymphoid neoplasm	7.60E-02	1.80E-01	---
33	NCI-H1666		908473	lung	lung: NSCLC: adenocarcinoma	AdenoCarcinoma	lung	carcinoma	1.32E-03	-2.89E-01	WT
34	647-V		906797	bladder	bladder	Carcinoma	urinary tract	carcinoma	2.82E-03	-2.72E-01	---
35	SNU-423		909737	liver	liver	HCC	liver	carcinoma	1.87E-10	5.31E-01	WT
36	KY821		907300	H.L.M.	Leukaemia	Leukaemia	haematopoietic and lymphoid tissue	haematopoietic neoplasm	1.73E-01	1.49E-01	WT
37	LU-99A		907796	lung	lung other	GiantCell	lung	carcinoma	5.41E-10	5.19E-01	WT
38	HC-1		907044	H.L.M.	hairy_cell_leukaemia	Lymphoma	haematopoietic and lymphoid tissue	lymphoid neoplasm	9.86E-02	1.71E-01	WT
39	639-V		906798	bladder	bladder	Carcinoma	urinary tract	carcinoma	1.65E-11	5.60E-01	WT
40	D-283MED		906834	CNS	medulloblastoma	Medulloblastoma	central nervous system	primitive neuroectodermal tumour-medulloblastoma	5.04E-10	5.20E-01	WT
41	KP-4		753572	pancreatic	pancreas	DuctalCarcinoma	pancreas	carcinoma	1.31E-11	5.62E-01	WT
42	OCI-AML2		910947	H.L.M.	AML	AML	haematopoietic and lymphoid tissue	haematopoietic neoplasm	1.87E-03	2.81E-01	WT
43	KASUMI-1		907275	H.L.M.	AML	AML	haematopoietic and lymphoid tissue	haematopoietic neoplasm	1.01E-02	2.41E-01	WT
44	KE-37		907277	H.L.M.	lymphoblastic Leukaemia	ALL	haematopoietic and lymphoid tissue	haematopoietic neoplasm	2.78E-02	2.13E-01	---
45	Saos-2		909707	Ewings/Osteosarcoma	osteosarcoma	Sarcoma	bone	osteosarcoma	5.65E-19	7.28E-01	WT
46	MKN28		908139	gastric	stomach	AdenoCarcinoma	stomach	carcinoma	3.56E-12	-5.77E-01	WT
47	JVM-2		907269	H.L.M.	lymphoid_neoplasm other	CLL	haematopoietic and lymphoid tissue	lymphoid neoplasm	6.39E-02	1.86E-01	WT
48	C8166		906829	H.L.M.	lymphoid_neoplasm other	ALL	haematopoietic and lymphoid tissue	lymphoid neoplasm	4.90E-05	3.54E-01	WT
49	GCIY		906869	gastric	stomach	AdenoCarcinoma	stomach	carcinoma	1.80E-01	1.47E-01	WT
50	LoVo		907790	colorectal	large_intestine	AdenoCarcinoma	large intestine	carcinoma	1.67E-05	-3.72E-01	WT
51	KARPAS-422		907274	H.L.M.	B cell lymphoma	Lymphoma	haematopoietic and lymphoid tissue	lymphoid neoplasm	3.20E-02	2.08E-01	---
52	MN-60		908143	H.L.M.	B cell Leukaemia	ALL	haematopoietic and lymphoid tissue	lymphoid neoplasm	3.29E-02	2.07E-01	WT
53	MFE-296		908130	uterus	endometrium	AdenoCarcinoma	endometrium	carcinoma	2.14E-09	5.02E-01	WT
54	HMV-II		907058	melanoma	malignant_melanoma	Melanoma	skin	malignant melanoma	1.54E-07	4.45E-01	WT
55	GOTO		906875	CNS	neuroblastoma	Neuroblastoma	autonomic ganglia	neuroblastoma	4.19E-13	5.99E-01	WT
56	P30-OHK		909252	H.L.M.	lymphoblastic Leukaemia	ALL	haematopoietic and lymphoid tissue	lymphoid neoplasm	1.67E-05	3.72E-01	WT
57	NCI-H2452		908462	lung	mesothelioma	Mesothelioma	pleura	mesothelioma	9.85E-09	4.82E-01	WT

58	HuCCT1	907069	biliary_tract	biliary_tract	Carcinoma	biliary tract	carcinoma	1.35E-01	-1.59E-01	WT
59	DSH1	753552	bladder	bladder	Carcinoma	urinary tract	carcinoma	1.86E-13	-6.08E-01	WT
60	BONNA-12	906695	H.L.M.	HCL (hairy cell Leukaemia)	Lymphoma	haematopoietic and lymphoid tissue	lymphoid neoplasm	7.78E-02	1.79E-01	WT
61	ACN	906803	CNS	neuroblastoma	Neuroblastoma	autonomic ganglia	neuroblastoma	2.23E-12	5.82E-01	WT
62	MPP-89	908150	lung	mesothelioma	Mesothelioma	pleura	mesothelioma	1.16E-14	6.36E-01	WT
63	ONS-76	909248	CNS	medulloblastoma	Medulloblastoma	central nervous system	primitive neuroectodermal tumour-medulloblastoma	4.35E-06	3.95E-01	WT
64	GDM-1	906870	H.L.M.	AML	AML	haematopoietic and lymphoid tissue	haematopoietic neoplasm	1.27E-03	2.90E-01	WT
65	OPM-2	909249	H.L.M.	Myeloma	Myeloma	haematopoietic and lymphoid tissue	haematopoietic neoplasm	5.35E-03	2.57E-01	WT
66	HCE-T	907049	head & neck	upper_aerodigestive_tract	Squamous	upper aerodigestive tract	carcinoma	8.98E-02	-1.74E-01	WT
67	MEG-01	908126	H.L.M.	CML	CML	haematopoietic and lymphoid tissue	haematopoietic neoplasm	5.66E-06	3.90E-01	WT
68	MJ	908137	H.L.M.	lymphoid_neoplasm other	Other	haematopoietic and lymphoid tissue	lymphoid neoplasm	1.30E-04	3.36E-01	WT
69	LC4-1	907787	H.L.M.	lymphoblastic Leukaemia	ALL	haematopoietic and lymphoid tissue	haematopoietic neoplasm	1.95E-02	2.23E-01	WT
70	ESS-1	907000	uterus	endometrium	Other	endometrium	carcinoma	3.56E-13	6.01E-01	WT
71	EB2	906846	H.L.M.	Burkitt lymphoma	BurkittsLymphoma	haematopoietic and lymphoid tissue	lymphoid neoplasm	5.23E-04	3.09E-01	WT
72	SW1710	909749	bladder	bladder	Carcinoma	urinary tract	carcinoma	3.76E-08	4.64E-01	WT
73	FTC-133	906864	thyroid	thyroid	FollicularCarcinoma	thyroid	carcinoma	3.81E-10	5.23E-01	WT
74	GI-ME-N	906872	CNS	neuroblastoma	Neuroblastoma	autonomic ganglia	neuroblastoma	2.95E-18	7.13E-01	WT
75	COR-L105	906805	lung	lung: NSCLC: adenocarcinoma	AdenoCarcinoma	lung	carcinoma	3.68E-02	-2.04E-01	WT
76	H-EMC-SS	907290	Ewings/Osteosarcoma	other	Sarcoma	bone	chondrosarcoma	1.30E-10	5.36E-01	WT
77	MHH-CALL-2	908132	H.L.M.	B cell Leukaemia	ALL	haematopoietic and lymphoid tissue	lymphoid neoplasm	1.63E-02	2.28E-01	WT
78	TGBC1TKB	909769	biliary_tract	biliary_tract	AdenoCarcinoma	biliary tract	carcinoma	2.14E-05	-3.68E-01	WT
79	P12-ICHIKAWA	909251	H.L.M.	lymphoblastic T cell leukaemia	ALL	haematopoietic and lymphoid tissue	lymphoid neoplasm	1.58E-02	2.29E-01	WT
80	NOMO-1	908451	H.L.M.	AML	AML	haematopoietic and lymphoid tissue	haematopoietic neoplasm	2.90E-01	1.25E-01	---
81	EFO-21	911905	ovarian	ovary	Carcinoma	ovary	carcinoma	8.61E-05	3.44E-01	WT
82	KP-N-YN	907314	CNS	neuroblastoma	Neuroblastoma	autonomic ganglia	neuroblastoma	3.67E-09	4.95E-01	WT
83	A704	910920	kidney	Renal cell carcinoma	Carcinoma	kidney	carcinoma	7.53E-03	2.48E-01	---
84	COLO-720E	688126	ovarian	ovary	AdenoCarcinoma	ovary	carcinoma	7.66E-10	5.14E-01	---
85	DMS-53	907295	lung	lung: small_cell_carcinoma	Carcinoma	lung	carcinoma	5.45E-04	-3.08E-01	WT

86	DMS-79	753551	lung	lung: small_cell_carcinoma	Carcinoma	lung	carcinoma	1.91E-01	-1.45E-01	WT
87	EFO-27	906852	ovarian	ovary	Carcinoma	ovary	carcinoma	1.05E-02	2.40E-01	WT
88	RVH-421	909706	melanoma	malignant_melanoma	Melanoma	skin	malignant melanoma	4.09E-10	5.22E-01	WT
89	DEL	906836	H.L.M.	lymphoid_neoplasm other	Other	haematopoietic and lymphoid tissue	lymphoid neoplasm	2.82E-03	2.72E-01	WT
90	MOLT-13	908146	H.L.M.	lymphoblastic T cell leukaemia	ALL	haematopoietic and lymphoid tissue	lymphoid neoplasm	1.54E-03	2.86E-01	WT
91	MFE-280	908129	uterus	endometrium	AdenoCarcinoma	endometrium	carcinoma	1.91E-01	1.45E-01	WT
92	SUP-B8	910209	H.L.M.	ALL	ALL	haematopoietic and lymphoid tissue	lymphoid neoplasm	5.35E-02	1.92E-01	WT
93	JVM-3	907270	H.L.M.	lymphoid_neoplasm other	CLL	haematopoietic and lymphoid tissue	lymphoid neoplasm	8.05E-03	2.47E-01	WT
94	HuP-T4	907286	pancreatic	pancreas	Carcinoma	pancreas	carcinoma	8.99E-12	-5.66E-01	WT
95	Ca9-22	753538	head & neck	upper_aerodigestive_tract	Squamous	upper aerodigestive tract	carcinoma	1.59E-01	-1.52E-01	WT
96	RPMI-8402	909702	H.L.M.	lymphoblastic T cell leukaemia	ALL	haematopoietic and lymphoid tissue	lymphoid neoplasm	3.63E-03	2.66E-01	WT
97	NCI-H661	687829	lung	lung: NSCLC: large cell	LargeCell	lung	carcinoma	4.69E-11	5.48E-01	WT
98	DBTRG-05MG	906835	CNS	glioma	Glioma	central nervous system	glioma	1.76E-12	5.84E-01	WT
99	SK-HEP-1	909719	liver	liver	AdenoCarcinoma	liver	carcinoma	3.76E-11	5.50E-01	WT
100	NCI-N417	934543	lung	lung: carcinoma	Carcinoma	lung	carcinoma	4.16E-12	5.75E-01	WT
101	NCI-H82	688031	lung	lung: small_cell_carcinoma	Carcinoma	lung	carcinoma	1.87E-09	5.03E-01	WT
102	MDA-MB-134-VI	908119	breast	breast	DuctalCarcinoma	breast	carcinoma	4.24E-05	-3.56E-01	WT
103	HT	907063	H.L.M.	B cell lymphoma	Lymphoma	haematopoietic and lymphoid tissue	lymphoid neoplasm	2.90E-01	1.25E-01	WT
104	LP-1	907791	H.L.M.	Myeloma	Myeloma	haematopoietic and lymphoid tissue	lymphoid neoplasm	1.39E-02	2.32E-01	WT
105	SW982	909759	sacrcoma	soft tissue other	Sarcoma	soft tissue	synovial sarcoma	9.27E-18	7.03E-01	WT
106	MSTO-211H	908152	lung	mesothelioma	Mesothelioma	pleura	mesothelioma	5.90E-16	6.65E-01	WT
107	NCI-H1299	724831	lung	lung: NSCLC: large cell	LargeCell	lung	carcinoma	7.47E-14	6.17E-01	WT
108	ETK-1	906861	biliary_tract	biliary_tract	AdenoCarcinoma	biliary tract	carcinoma	8.90E-04	-2.98E-01	WT
109	RF-48	909697	gastric	stomach	AdenoCarcinoma	stomach	carcinoma	1.38E-01	1.58E-01	WT
110	SW13	909744	---	other	Carcinoma	adrenal gland	adrenal cortical carcinoma	1.08E-09	5.10E-01	WT
111	NCI-SNU-5	908445	gastric	stomach	Carcinoma	stomach	carcinoma	6.22E-01	-7.74E-02	WT
112	NCI-H524	908483	lung	lung: small_cell_carcinoma	Carcinoma	lung	carcinoma	1.01E-07	4.51E-01	---
113	MDA-MB-453	908122	breast	breast	Carcinoma	breast	carcinoma	6.30E-11	-5.44E-01	WT
114	JEG-3	907176	---	other	Carcinoma	placenta	choriocarcinoma	6.80E-03	-2.51E-01	WT

115	LNCaP-Clone-FGC	907788	prostate	prostate	AdenoCarcinoma	prostate	carcinoma	2.32E-04	-3.25E-01	WT
116	YAPC	909904	pancreatic	pancreas	Carcinoma	pancreas	carcinoma	1.28E-08	-4.79E-01	WT
117	MEL-HO	908124	melanoma	malignant_melanoma	Melanoma	skin	malignant melanoma	1.55E-06	4.11E-01	WT
118	SU-DHL-1	909742	H.L.M.	lymphoid_neoplasm other	Lymphoma	haematopoietic and lymphoid tissue	lymphoid neoplasm	5.65E-05	3.51E-01	---
119	EM-2	906855	H.L.M.	CML	CML	haematopoietic and lymphoid tissue	haematopoietic neoplasm	1.43E-01	1.56E-01	WT
120	EB-3	906847	H.L.M.	Burkitt lymphoma	BurkittsLymphoma	haematopoietic and lymphoid tissue	lymphoid neoplasm	1.66E-01	1.51E-01	WT
121	Ramos-2G6-4C10	910401	H.L.M.	Burkitt lymphoma	BurkittsLymphoma	haematopoietic and lymphoid tissue	lymphoid neoplasm	4.44E-01	1.01E-01	WT
122	SW1088	909745	CNS	glioma	Glioma	central nervous system	glioma	4.33E-18	7.10E-01	WT
123	NCI-SNU-16	908446	gastric	stomach	Carcinoma	stomach	carcinoma	2.18E-16	-6.74E-01	WT
124	NCI-H520	908443	lung	lung: NSCLC: squamous_cell_carcinoma	Squamous	lung	carcinoma	2.43E-01	-1.34E-01	WT
125	KATOIII	907276	gastric	stomach	Carcinoma	stomach	carcinoma	1.63E-12	-5.85E-01	WT
126	JiyoyeP-2003	907268	H.L.M.	Burkitt lymphoma	BurkittsLymphoma	haematopoietic and lymphoid tissue	lymphoid neoplasm	2.75E-01	-1.28E-01	WT
127	LS-411N	907794	colorectal	large_intestine	AdenoCarcinoma	large intestine	carcinoma	1.45E-08	-4.77E-01	WT
128	WERI-Rb-1	909782	Eye	retinoblastma	Carcinoma	eye	retinoblastoma	1.09E-03	2.93E-01	WT
129	NB19	801738	CNS	neuroblastoma	Neuroblastoma	autonomic ganglia	neuroblastoma	1.28E-08	4.79E-01	---
130	EGI-1	906853	biliary_tract	biliary_tract	AdenoCarcinoma	biliary tract	carcinoma	8.65E-09	-4.84E-01	WT
131	DG-75	906838	H.L.M.	Burkitt lymphoma	BurkittsLymphoma	haematopoietic and lymphoid tissue	lymphoid neoplasm	3.99E-02	2.02E-01	WT
132	DV-90	753553	lung	lung: NSCLC: adenocarcinoma	AdenoCarcinoma	lung	carcinoma	4.48E-09	-4.92E-01	WT
133	SNU-C2B	909740	colorectal	large_intestine	AdenoCarcinoma	large intestine	carcinoma	1.16E-09	-5.09E-01	WT
134	SW1417	909747	colorectal	large_intestine	AdenoCarcinoma	large intestine	carcinoma	7.85E-11	-5.42E-01	WT
135	NCI-SNU-1	908444	gastric	stomach	Carcinoma	stomach	carcinoma	2.26E-03	2.77E-01	WT
136	NCI-H28	908470	lung	mesothelioma	Mesothelioma	pleura	mesothelioma	5.39E-05	3.52E-01	WT
137	HuNS1	683664	H.L.M.	myeloma	Myeloma	haematopoietic and lymphoid tissue	lymphoid neoplasm	4.69E-02	1.96E-01	---
138	KS-1	907313	CNS	glioma	Glioma	central nervous system	glioma	2.97E-14	6.27E-01	WT
139	LS-513	907795	colorectal	large_intestine	AdenoCarcinoma	large intestine	carcinoma	2.09E-22	-7.93E-01	WT
140	MEL-JUSO	908125	melanoma	malignant_melanoma	Melanoma	skin	malignant melanoma	3.31E-10	5.25E-01	WT
141	NCI-H1650	687800	lung	lung: NSCLC: adenocarcinoma	AdenoCarcinoma	lung	carcinoma	3.20E-02	-2.08E-01	MUT_p.E746_A750del/In_Frame_Deletion
142	UMC-11	909779	lung	lung: NSCLC: carcinoid	Carcinoid	lung	carcinoid-endocrine tumour	8.37E-02	-1.77E-01	WT
143	GA-10-Clone-4	906866	H.L.M.	Burkitt lymphoma	BurkittsLymphoma	haematopoietic and lymphoid tissue	lymphoid neoplasm	2.57E-01	1.31E-01	WT
144	Daudi	906831	H.L.M.	Burkitt lymphoma	BurkittsLymphoma	haematopoietic and lymphoid tissue	lymphoid neoplasm	3.74E-01	1.11E-01	---

145	RPMI-6666	909701	H.L.M.	Hodgkin lymphoma	HodgekinsLymphoma	haematopoietic and lymphoid tissue	lymphoid neoplasm	1.63E-01	1.51E-01	WT
146	SW1463	909748	colorectal	large_intestine	AdenoCarcinoma	large intestine	carcinoma	1.97E-17	-6.96E-01	WT
147	NCI-H889	908456	lung	lung: small_cell_carcinoma	Carcinoma	lung	carcinoma	1.26E-01	1.62E-01	WT
148	NCI-H2347	687820	lung	lung: NSCLC: NOS	NSCLC	lung	carcinoma	3.03E-03	-2.70E-01	WT
149	ECC4	906850	GI tract	other	Carcinoma	gastrointestinal tract (site indeterminate)	carcinoma	6.14E-03	-2.53E-01	WT
150	KYSE-180	907318	oesophagus	oesophagus	Squamous	oesophagus	carcinoma	1.05E-02	-2.40E-01	WT
151	ML-2	908141	H.L.M.	AML	AML	haematopoietic and lymphoid tissue	haematopoietic neoplasm	3.20E-02	2.08E-01	WT
152	NCI-H1993	908476	lung	lung: NSCLC: adenocarcinoma	AdenoCarcinoma	lung	carcinoma	3.31E-10	-5.25E-01	WT
153	NCI-H1703	908474	lung	lung: NSCLC: adenocarcinoma	Carcinoma	lung	carcinoma	1.74E-10	5.32E-01	WT
154	EC-GI-10	753555	oesophagus	oesophagus	Carcinoma	oesophagus	carcinoma	1.08E-02	-2.39E-01	WT
155	SK-MEL-30	909726	melanoma	malignant_melanoma	Melanoma	skin	malignant melanoma	1.39E-06	4.12E-01	WT
156	RTSG	909705	ovarian	ovary	AdenoCarcinoma	ovary	carcinoma	1.08E-01	1.68E-01	WT
157	SW837	909755	colorectal	large_intestine	AdenoCarcinoma	large intestine	carcinoma	1.37E-07	-4.46E-01	WT
158	NCI-H716	908458	colorectal	large_intestine	AdenoCarcinoma	large intestine	carcinoma	1.19E-04	3.38E-01	WT
159	NCI-H2030	722045	lung	lung: NSCLC: adenocarcinoma	AdenoCarcinoma	lung	carcinoma	3.33E-05	3.61E-01	WT
160	DB	906832	H.L.M.	B cell lymphoma	Lymphoma	haematopoietic and lymphoid tissue	lymphoid neoplasm	1.91E-01	1.45E-01	WT
161	KYSE-70	753576	oesophagus	oesophagus	Squamous	oesophagus	carcinoma	5.35E-02	1.92E-01	WT
162	LU-139	713878	lung	lung: small_cell_carcinoma	Carcinoma	lung	carcinoma	2.52E-01	1.32E-01	WT
163	MS-1	753594	lung	lung: small_cell_carcinoma	Carcinoma	lung	carcinoma	3.14E-07	4.35E-01	WT
164	NCCIT	908441	germ_cell_tumour	germ_cell_tumour	GermCell	testis	germ cell tumour	7.59E-09	4.86E-01	WT
165	RPMI-8866	910544	H.L.M.	CML	CML	haematopoietic and lymphoid tissue	haematopoietic neoplasm	8.57E-02	1.76E-01	WT
166	G-401	907299	kidney	Renal other	Rabdoid	kidney	rhabdoid tumour	1.64E-07	4.44E-01	WT
167	SNU-449	909738	liver	liver	HCC	liver	carcinoma	1.74E-07	4.43E-01	WT
168	RT-112	909704	bladder	bladder	Carcinoma	urinary tract	carcinoma	4.92E-14	-6.22E-01	WT
169	SW872	909756	sacrcoma	soft tissue other	Other	soft tissue	liposarcoma	4.38E-10	5.21E-01	WT
170	NCI-H747	908457	colorectal	large_intestine	AdenoCarcinoma	large intestine	carcinoma	9.23E-09	-4.83E-01	WT
171	NCI-H1755	908475	lung	lung: NSCLC: adenocarcinoma	AdenoCarcinoma	lung	carcinoma	8.17E-02	-1.78E-01	WT
172	Daoy	906833	CNS	medulloblastoma	Medulloblastoma	central nervous system	primitive neuroectodermal tumour-medulloblastoma	1.92E-11	5.58E-01	WT

173	KINGS-1	907279	CNS	glioma	Glioma	central nervous system	glioma	4.17E-17	6.90E-01	WT
174	LCLC-103H	753586	lung	lung: NSCLC: large cell	LargeCell	lung	carcinoma	3.21E-09	4.97E-01	WT
175	MOLT-16	908147	H.L.M.	lymphoblastic T cell leukaemia	ALL	haematopoietic and lymphoid tissue	lymphoid neoplasm	1.58E-02	2.29E-01	WT
176	NALM-6	908158	H.L.M.	B cell Leukaemia	ALL	haematopoietic and lymphoid tissue	lymphoid neoplasm	9.64E-04	2.96E-01	WT
177	NALM-1	908157	H.L.M.	CML	CML	haematopoietic and lymphoid tissue	haematopoietic neoplasm	4.94E-02	1.95E-01	---
178	Detroit562	906837	head & neck	upper_aerodigestive_tract	Carcinoma	upper aerodigestive tract	carcinoma	3.91E-06	-3.96E-01	WT
179	SJRH30	909716	sacrcoma	rhabdomyosarcoma	Sarcoma	soft tissue	rhabdomyosarcoma	5.33E-13	5.97E-01	WT
180	SF126	909712	CNS	glioma	Glioma	central nervous system	glioma	1.65E-18	7.19E-01	---
181	SW684	909754	sacrcoma	soft tissue other	Sarcoma	soft tissue	fibrosarcoma	1.97E-17	6.96E-01	WT
182	NCI-H727	724855	lung	lung: NSCLC: carcinoid	Carcinoid	lung	carcinoid-endocrine tumour	1.08E-04	-3.39E-01	WT
183	NCI-H1838	687807	lung	lung: NSCLC: adenocarcinoma	AdenoCarcinoma	lung	carcinoma	5.51E-01	8.67E-02	WT
184	HH	907056	H.L.M.	lymphoid_neoplasm other	ALL	haematopoietic and lymphoid tissue	lymphoid neoplasm	8.90E-04	2.98E-01	WT
185	L-428	907322	H.L.M.	Hodgkin lymphoma	HodgekinsLymphoma	haematopoietic and lymphoid tissue	lymphoid neoplasm	9.18E-03	2.43E-01	WT
186	SW948	909757	colorectal	large_intestine	AdenoCarcinoma	large intestine	carcinoma	2.68E-18	-7.14E-01	WT
187	MUTZ-1	908155	H.L.M.	myelodysplatic	MDS	haematopoietic and lymphoid tissue	haematopoietic neoplasm	1.23E-01	1.62E-01	WT
188	NCI-H1792	724868	lung	lung: NSCLC: adenocarcinoma	AdenoCarcinoma	lung	carcinoma	5.11E-09	4.91E-01	WT
189	RS4-11	909703	H.L.M.	Leukaemia	Leukaemia	haematopoietic and lymphoid tissue	haematopoietic neoplasm	1.32E-01	1.60E-01	WT
190	NCI-H146	910899	lung	lung: small_cell_carcinoma	Carcinoma	lung	carcinoma	3.10E-01	1.22E-01	WT
191	Calu-3	687777	lung	lung: NSCLC: adenocarcinoma	AdenoCarcinoma	lung	carcinoma	7.53E-03	-2.48E-01	WT
192	CPC-N	753547	lung	lung: small_cell_carcinoma	Carcinoma	lung	carcinoma	2.99E-06	4.01E-01	WT
193	NCI-H2405	687821	lung	lung: NSCLC: adenocarcinoma	AdenoCarcinoma	lung	carcinoma	6.99E-06	3.87E-01	WT
194	J-RT3-T3-5	907391	H.L.M.	lymphoblastic T cell leukaemia	ALL	haematopoietic and lymphoid tissue	lymphoid neoplasm	2.34E-03	2.76E-01	WT
195	NCI-N87	908461	gastric	stomach	AdenoCarcinoma	stomach	carcinoma	9.08E-11	-5.40E-01	WT
196	SCH	909711	gastric	stomach	Carcinoma	stomach	other	6.55E-02	-1.85E-01	WT
197	IST-MEL1	907172	melanoma	malignant_melanoma	Melanoma	skin	malignant melanoma	8.83E-14	6.16E-01	WT
198	A4-Fuk	910934	melanoma	malignant_melanoma	Melanoma	skin	malignant melanoma	1.70E-04	3.31E-01	WT

199	LU-135	713899	lung	lung: small_cell_carcinoma	Carcinoma	lung	carcinoma	5.14E-05	3.53E-01	WT
200	PC-14	753608	lung	lung: NSCLC: adenocarcinoma	AdenoCarcinoma	lung	carcinoma	9.91E-07	-4.18E-01	MUT_p.E746_A750del/In_Frame_Deletion
201	EVSA-T	906862	breast	breast	Carcinoma	breast	carcinoma	6.78E-11	-5.43E-01	WT
202	ATN-1	910687	H.L.M.	lymphoblastic T cell leukaemia	ALL	haematopoietic and lymphoid tissue	lymphoid neoplasm	3.04E-01	1.22E-01	WT
203	Calu-6	724859	lung	lung: NSCLC: adenocarcinoma	AdenoCarcinoma	lung	carcinoma	2.59E-11	5.54E-01	WT
204	COLO-824	906812	breast	breast	Carcinoma	breast	carcinoma	1.59E-01	-1.52E-01	WT
205	NCI-H2227	688018	lung	lung: small_cell_carcinoma	Carcinoma	lung	carcinoma	2.08E-07	4.40E-01	WT
206	MC-1010	907798	H.L.M.	AML	AML	haematopoietic and lymphoid tissue	haematopoietic neoplasm	4.94E-02	1.95E-01	WT
207	OCUB-M	909256	breast	breast	Carcinoma	breast	carcinoma	4.19E-09	-4.93E-01	WT
208	T98G	687586	CNS	glioma	Glioma	central nervous system	glioma	7.97E-13	5.93E-01	WT
209	IGR-1	907169	melanoma	malignant_melanoma	Melanoma	skin	malignant melanoma	9.37E-07	4.18E-01	WT
210	CAL-120	906826	breast	breast	Carcinoma	breast	carcinoma	2.63E-07	4.37E-01	WT
211	LN-405	910694	CNS	glioma	Glioma	central nervous system	glioma	2.23E-12	5.82E-01	WT
212	PSN1	910546	pancreatic	pancreas	Carcinoma	pancreas	carcinoma	1.13E-03	2.93E-01	WT
213	AsPC-1	910702	pancreatic	pancreas	Carcinoma	pancreas	carcinoma	1.05E-02	-2.40E-01	WT
214	CESS	910688	H.L.M.	AML	AML	haematopoietic and lymphoid tissue	haematopoietic neoplasm	1.53E-01	1.54E-01	WT
215	COLO-680N	906817	oesophagus	oesophagus	Squamous	oesophagus	carcinoma	3.58E-04	-3.16E-01	WT
216	NCI-H1522	908464	lung	lung: small_cell_carcinoma	Carcinoma	lung	carcinoma	5.92E-02	1.89E-01	WT
217	MHH-PREB-1	908136	H.L.M.	B cell Leukaemia	ALL	haematopoietic and lymphoid tissue	lymphoid neoplasm	3.02E-02	2.10E-01	WT
218	OAW-42	910548	ovarian	ovary	Carcinoma	ovary	carcinoma	1.87E-01	1.45E-01	WT
219	TUR	909773	H.L.M.	B cell lymphoma	Lymphoma	haematopoietic and lymphoid tissue	lymphoid neoplasm	1.23E-02	2.36E-01	WT
220	LS-174T	907793	colorectal	large_intestine	AdenoCarcinoma	large intestine	carcinoma	5.02E-17	-6.88E-01	WT
221	KURAMOCHI	909975	ovarian	ovary	Carcinoma	ovary	carcinoma	5.74E-03	2.55E-01	WT
222	MDA-MB-361	908121	breast	breast	Carcinoma	breast	carcinoma	1.63E-12	-5.85E-01	WT
223	RCC10RGB	909974	kidney	Renal cell carcinoma	Carcinoma	kidney	carcinoma	1.70E-04	3.31E-01	WT
224	NCI-H1770	687804	lung	lung: NSCLC: NOS	NSCLC	lung	carcinoma	1.87E-03	2.81E-01	WT
225	AN3-CA	910781	uterus	endometrium	AdenoCarcinoma	endometrium	carcinoma	3.33E-07	4.34E-01	WT
226	UM-UC-3	724838	bladder	bladder	Carcinoma	urinary tract	carcinoma	1.10E-15	6.59E-01	WT
227	REH	909696	H.L.M.	B cell Leukaemia	ALL	haematopoietic and lymphoid tissue	lymphoid neoplasm	8.32E-03	2.46E-01	WT

228	NCI-H1563	753600	lung	lung: NSCLC: adenocarcinoma	AdenoCarcinoma	lung	carcinoma	1.74E-07	4.43E-01	WT
229	MHH-ES-1	908134	Ewings/Osteosarcoma	Ewings sarcoma	EwingsSarcoma	bone	Ewings sarcoma-peripheral primitive neuroectodermal tumour	7.59E-09	4.86E-01	---
230	PFSK-1	683667	CNS	medulloblastoma	Medulloblastoma	central nervous system	primitive neuroectodermal tumour-medulloblastoma	1.05E-11	5.65E-01	WT
231	UACC-812	910910	breast	breast	DuctalCarcinoma	breast	carcinoma	6.79E-13	-5.94E-01	WT
232	KYSE-510	907321	oesophagus	oesophagus	Squamous	oesophagus	carcinoma	3.28E-04	-3.18E-01	WT
233	MES-SA	908127	sacrcoma	soft tissue other	Sarcoma	soft tissue	sarcoma	2.99E-06	4.01E-01	WT
234	ME-180	687514	uterus	cervix	Carcinoma	cervix	carcinoma	1.93E-06	-4.07E-01	---
235	SCCH-26	909710	CNS	neuroblastoma	Neuroblastoma	autonomic ganglia	neuroblastoma	1.55E-08	4.76E-01	WT
236	A427	910851	lung	lung: NOS	Carcinoma	lung	carcinoma	1.53E-02	2.30E-01	WT
237	VA-ES-BJ	688121	sacrcoma	soft tissue other	Sarcoma	soft tissue	epithelioid sarcoma	1.01E-07	4.51E-01	WT
238	BV-173	910710	H.L.M.	CML	CML	haematopoietic and lymphoid tissue	haematopoietic neoplasm	1.05E-02	2.40E-01	WT
239	NCI-H1355	724866	lung	lung: NSCLC: adenocarcinoma	AdenoCarcinoma	lung	carcinoma	1.59E-01	1.52E-01	WT
240	NCI-H1651	910900	lung	lung: NSCLC: adenocarcinoma	AdenoCarcinoma	lung	carcinoma	4.35E-06	3.95E-01	WT
241	QIMR-WIL	910545	H.L.M.	AML	AML	haematopoietic and lymphoid tissue	haematopoietic neoplasm	8.98E-02	1.74E-01	WT
242	EFM-19	906851	breast	breast	DuctalCarcinoma	breast	carcinoma	8.37E-07	-4.20E-01	WT
243	Mewo	908128	melanoma	malignant_melanoma	Melanoma	skin	malignant melanoma	2.68E-06	4.02E-01	WT
244	COR-L279	910937	lung	lung: small_cell_carcinoma	Carcinoma	lung	carcinoma	3.68E-01	-1.12E-01	WT
245	NCI-H1048	687995	lung	lung: small_cell_carcinoma	Carcinoma	lung	carcinoma	1.94E-01	1.44E-01	WT
246	C-4-II	910553	uterus	cervix	Carcinoma	cervix	carcinoma	3.54E-08	-4.65E-01	WT
247	SW48	909751	colorectal	large_intestine	AdenoCarcinoma	large intestine	carcinoma	1.04E-04	-3.40E-01	MUT_p.G719S/Missense
248	LAMA-84	907783	H.L.M.	CML	CML	haematopoietic and lymphoid tissue	haematopoietic neoplasm	1.08E-02	2.39E-01	WT
249	NY	910849	Ewings/Osteosarcoma	osteosarcoma	Sarcoma	bone	osteosarcoma	4.05E-11	5.49E-01	WT
250	NCI-H2170	687815	lung	lung: NSCLC: squamous_cell_carcinoma	Squamous	lung	carcinoma	9.76E-11	-5.39E-01	WT
251	RD	909264	sacrcoma	rhabdomyosarcoma	Sarcoma	soft tissue	rhabdomyosarcoma	1.36E-18	7.20E-01	---
252	FADU	906863	head & neck	upper_aerodigestive_tract	Squamous	upper aerodigestive tract	carcinoma	8.94E-08	-4.52E-01	WT

253	MG-63	908131	Ewings/Osteosarcoma	osteosarcoma	Sarcoma	bone	osteosarcoma	6.33E-14	6.19E-01	WT
254	NCI-H64	688026	lung	lung: small_cell_carcinoma	Carcinoma	lung	carcinoma	1.10E-01	1.67E-01	---
255	HEL	907053	H.L.M.	AML	AML	haematopoietic and lymphoid tissue	haematopoietic neoplasm	5.35E-03	2.57E-01	---
256	C3A	910850	liver	liver	HCC	liver	carcinoma	2.10E-01	-1.40E-01	---
257	U-118-MG	687588	CNS	glioma	Glioma	central nervous system	glioma	1.82E-18	7.18E-01	WT
258	CML-T1	910951	H.L.M.	CML	CML	haematopoietic and lymphoid tissue	haematopoietic neoplasm	3.14E-03	2.70E-01	WT
259	MOG-G-CCM	908144	CNS	glioma	Glioma	central nervous system	glioma	9.35E-13	5.91E-01	---
260	NCI-H441	908460	lung	lung: NSCLC: adenocarcinoma	AdenoCarcinoma	lung	carcinoma	3.08E-10	-5.26E-01	WT
261	RL	910861	H.L.M.	B cell lymphoma	Lymphoma	haematopoietic and lymphoid tissue	lymphoid neoplasm	3.57E-01	1.14E-01	WT
262	IST-MES1	907173	lung	mesothelioma	Mesothelioma	pleura	mesothelioma	3.55E-10	5.24E-01	WT
263	PA-1	909255	ovarian	ovary	GermCell	ovary	germ cell tumour	4.16E-14	6.23E-01	WT
264	KALS-1	907271	CNS	glioma	Glioma	central nervous system	glioma	1.38E-14	6.34E-01	WT
265	SCC-25	910701	head & neck	upper_aerodigestive_tract	Squamous	upper aerodigestive tract	carcinoma	1.18E-01	-1.64E-01	WT
266	no-10	908452	CNS	glioma	Glioma	central nervous system	glioma	4.50E-15	6.45E-01	WT
267	Calu-1	724858	lung	lung: NSCLC: adenocarcinoma	Carcinoma	lung	carcinoma	8.80E-10	5.13E-01	WT
268	YKG-1	687592	CNS	glioma	Glioma	central nervous system	glioma	1.27E-14	6.35E-01	WT
269	SNU-475	909739	liver	liver	HCC	liver	carcinoma	4.86E-12	5.73E-01	WT
270	NCI-H630	908482	colorectal	large_intestine	AdenoCarcinoma	large intestine	carcinoma	8.43E-16	-6.62E-01	WT
271	RPMI-2650	909700	head & neck	upper_aerodigestive_tract	Squamous	upper aerodigestive tract	carcinoma	1.09E-03	2.93E-01	WT
272	IPC-298	907171	melanoma	malignant_melanoma	Melanoma	skin	malignant melanoma	1.84E-07	4.42E-01	WT
273	RMG-I	909699	ovarian	ovary	ClearCell	ovary	carcinoma	2.34E-03	-2.76E-01	WT
274	KYSE-140	753573	oesophagus	oesophagus	Squamous	oesophagus	carcinoma	6.14E-03	-2.53E-01	WT
275	NCI-H1092	687997	lung	lung: small_cell_carcinoma	Carcinoma	lung	carcinoma	2.40E-02	2.17E-01	WT
276	SW1573	724878	lung	lung: NSCLC: adenocarcinoma	AdenoCarcinoma	lung	carcinoma	3.30E-12	5.77E-01	WT
277	TGBC24TKB	910695	biliary_tract	biliary_tract	Carcinoma	biliary tract	carcinoma	1.71E-13	-6.09E-01	WT
278	A673	684052	sacrcoma	rhabdomyosarcoma (putative Ewings)	Sarcoma	soft tissue	rhabdomyosarcoma	6.22E-10	5.17E-01	WT
279	CGTH-W-1	910568	thyroid	thyroid	FollicularCarcinoma	thyroid	carcinoma	4.33E-18	7.10E-01	WT
280	CHL-1	910853	melanoma	malignant_melanoma	Melanoma	skin	malignant melanoma	2.63E-07	4.37E-01	WT

281	CAL-62	906828	thyroid	thyroid	Anaplastic	thyroid	carcinoma	6.51E-05	3.49E-01	WT
282	GT3TKB	907041	gastric	stomach	AdenoCarcinoma	stomach	carcinoma	4.69E-11	5.48E-01	WT
283	NMC-G1	908449	CNS	glioma	Glioma	central nervous system	glioma	1.78E-11	5.59E-01	WT
284	SJSA-1	909717	Ewings/Osteosarcoma	other	Sarcoma	bone	other	5.33E-13	5.97E-01	WT
285	HUTU-80	907073	GI tract	other	AdenoCarcinoma	small intestine	carcinoma	5.85E-11	5.45E-01	WT
286	LU-65	724863	lung	lung other	GiantCell	lung	carcinoma	3.85E-01	-1.10E-01	WT
287	AM-38	910933	CNS	glioma	Glioma	central nervous system	glioma	2.16E-10	5.30E-01	WT
288	B2-17	911904	CNS	glioma	Glioma	central nervous system	glioma	6.04E-17	6.86E-01	---
289	CAL-12T	753540	lung	lung: NSCLC: NOS	NSCLC	lung	carcinoma	1.04E-04	-3.40E-01	WT
290	CAL-85-1	910852	breast	breast	Carcinoma	breast	carcinoma	3.03E-03	-2.70E-01	---
291	CHP-134	910941	CNS	neuroblastoma	Neuroblastoma	autonomic ganglia	neuroblastoma	3.74E-07	4.32E-01	WT
292	GCT	906999	sacrcoma	soft tissue other	Sarcoma	soft tissue	malignant fibrous histiocytoma-pleomorphic sarcoma	1.86E-13	6.08E-01	WT
293	HPAF-II	724869	pancreatic	pancreas	DuctalCarcinoma	pancreas	carcinoma	2.23E-11	-5.56E-01	WT
294	NCI-H1155	908467	lung	lung: NSCLC: large cell	LargeCell	lung	carcinoma	1.09E-03	2.93E-01	WT
295	SK-LMS-1	909720	sacrcoma	soft tissue other	Sarcoma	soft tissue	sarcoma	5.39E-16	6.66E-01	WT
296	HCC1954	749709	breast	breast	DuctalCarcinoma	breast	carcinoma	1.30E-10	-5.36E-01	WT
297	T-24	724812	bladder	bladder	Carcinoma	urinary tract	carcinoma	4.24E-05	3.56E-01	WT
298	no-11	908450	CNS	glioma	Glioma	central nervous system	glioma	1.64E-17	6.98E-01	WT
299	A204	910784	sacrcoma	rhabdomyosarcoma	Sarcoma	soft tissue	rhabdomyosarcoma	1.53E-11	5.60E-01	WT
300	BEN	753534	lung	lung: NOS	Carcinoma	lung	carcinoma	3.15E-06	-4.00E-01	WT
301	CAL-33	753541	head & neck	upper_aerodigestive_tract	Squamous	upper aerodigestive tract	carcinoma	1.54E-07	-4.45E-01	WT
302	COLO-320-HSR	910569	colorectal	large_intestine	AdenoCarcinoma	large intestine	carcinoma	9.53E-06	3.82E-01	WT
303	GAMG	906868	CNS	glioma	Glioma	central nervous system	glioma	2.79E-13	6.04E-01	WT
304	HT-1080	907064	sacrcoma	soft tissue other	Sarcoma	soft tissue	fibrosarcoma	1.28E-12	5.88E-01	WT
305	HuH-7	907071	liver	liver	HCC	liver	carcinoma	3.02E-05	3.62E-01	WT
306	SCC-3	910930	H.L.M.	B cell lymphoma	Lymphoma	haematopoietic and lymphoid tissue	lymphoid neoplasm	4.24E-04	3.13E-01	WT
307	HCC2157	749715	breast	breast	DuctalCarcinoma	breast	carcinoma	2.65E-04	-3.22E-01	WT
308	TGW	910780	CNS	neuroblastoma	Neuroblastoma	autonomic ganglia	neuroblastoma	1.96E-07	4.41E-01	---
309	23132-87	910924	gastric	stomach	AdenoCarcinoma	stomach	carcinoma	5.90E-16	-6.65E-01	WT
310	ALL-PO	910944	H.L.M.	lymphoblastic Leukaemia	ALL	haematopoietic and lymphoid tissue	lymphoid neoplasm	2.27E-02	2.19E-01	WT
311	BL-41	910706	H.L.M.	Burkitt lymphoma	BurkittsLymphoma	haematopoietic and lymphoid tissue	lymphoid neoplasm	1.08E-01	1.68E-01	---
312	Caov-3	906825	ovarian	ovary	AdenoCarcinoma	ovary	carcinoma	2.20E-02	-2.19E-01	WT

313	BL-70	910707	H.L.M.	Burkitt lymphoma	BurkittsLymphoma	haematopoietic and lymphoid tissue	lymphoid neoplasm	1.53E-01	1.54E-01	WT
314	HCC70	907048	breast	breast	DuctalCarcinoma	breast	carcinoma	2.58E-08	-4.69E-01	WT
315	HN	907059	head & neck	upper_aerodigestive_tract	Squamous	upper aerodigestive tract	carcinoma	3.57E-01	1.14E-01	WT
316	HT-144	907067	melanoma	malignant_melanoma	Melanoma	skin	malignant melanoma	3.03E-13	6.03E-01	WT
317	SW1990	910907	pancreatic	pancreas	Carcinoma	pancreas	carcinoma	5.47E-08	-4.59E-01	WT
318	HCC1599	749713	breast	breast	DuctalCarcinoma	breast	carcinoma	1.04E-04	-3.40E-01	WT
319	TALL-1	909762	H.L.M.	lymphoblastic T cell leukaemia	ALL	haematopoietic and lymphoid tissue	lymphoid neoplasm	1.87E-03	2.81E-01	---
320	769-P	910922	kidney	clear cell carcinoma	ClearCell	kidney	carcinoma	7.57E-04	3.01E-01	WT
321	A101D	910921	melanoma	malignant_melanoma	Melanoma	skin	malignant melanoma	1.74E-10	5.32E-01	WT
322	C-33-A	687505	uterus	cervix	Carcinoma	cervix	carcinoma	2.07E-11	5.57E-01	---
323	COLO-678	910689	colorectal	large_intestine	Carcinoma	large intestine	carcinoma	8.33E-12	-5.67E-01	WT
324	CAS-1	910943	CNS	glioma	Glioma	central nervous system	glioma	1.35E-17	7.00E-01	WT
325	DMS-114	687983	lung	lung: small_cell_carcinoma	Carcinoma	lung	carcinoma	1.01E-09	5.11E-01	---
326	H4	907042	CNS	glioma	Glioma	central nervous system	glioma	1.79E-14	6.32E-01	WT
327	HOS	907060	Ewings/Osteosarcoma	osteosarcoma	Sarcoma	bone	osteosarcoma	8.63E-13	5.92E-01	WT
328	HUH-6-clone5	907070	liver	liver	HCC	liver	carcinoma	1.26E-01	-1.62E-01	WT
329	SW900	724879	lung	lung: NSCLC: squamous_cell_carcinoma	Squamous	lung	carcinoma	2.43E-04	3.24E-01	WT
330	HCC1937	749714	breast	breast	DuctalCarcinoma	breast	carcinoma	4.83E-08	-4.61E-01	WT
331	TI-73	910696	Ewings/Osteosarcoma	osteosarcoma	Sarcoma	bone	osteosarcoma	1.44E-15	6.56E-01	WT
332	8-MG-BA	687562	CNS	glioma	Glioma	central nervous system	glioma	2.79E-13	6.04E-01	WT
333	BALL-1	910705	H.L.M.	B cell Leukaemia	ALL	haematopoietic and lymphoid tissue	lymphoid neoplasm	1.47E-01	1.56E-01	WT
334	CTV-1	753548	H.L.M.	AML	AML	haematopoietic and lymphoid tissue	haematopoietic neoplasm	2.52E-03	2.75E-01	WT
335	CW-2	910554	colorectal	large_intestine	Carcinoma	large intestine	carcinoma	9.22E-16	-6.61E-01	WT
336	C2BBe1	910700	colorectal	large_intestine	AdenoCarcinoma	large intestine	carcinoma	1.20E-08	-4.80E-01	WT
337	GAK	910932	melanoma	malignant_melanoma	Melanoma	skin	malignant melanoma	6.63E-06	3.88E-01	WT
338	H9	907043	H.L.M.	lymphoid_neoplasm other	Other	haematopoietic and lymphoid tissue	lymphoid neoplasm	5.94E-03	2.54E-01	WT
339	HEC-1	907051	uterus	endometrium	AdenoCarcinoma	endometrium	carcinoma	1.58E-02	2.29E-01	WT
340	ST486	910906	H.L.M.	Burkitt lymphoma	BurkittsLymphoma	haematopoietic and lymphoid tissue	lymphoid neoplasm	3.68E-02	2.04E-01	WT
341	SW1783	909750	CNS	glioma	Glioma	central nervous system	glioma	4.92E-14	6.22E-01	WT

342	HCC1143	749710	breast	breast	DuctalCarcinoma	breast	carcinoma	9.86E-02	-1.71E-01	WT
343	VMRC-RCZ	909781	kidney	Renal cell carcinoma	Carcinoma	kidney	carcinoma	7.90E-07	4.21E-01	WT
344	A3-KAW	910935	gastric	stomach	Carcinoma	stomach	carcinoma	3.58E-02	2.05E-01	WT
345	BC-1	910919	H.L.M.	B cell lymphoma	Lymphoma	haematopoietic and lymphoid tissue	lymphoid neoplasm	8.05E-03	2.47E-01	WT
346	COLO-684	910691	uterus	endometrium	AdenoCarcinoma	endometrium	carcinoma	1.07E-07	4.50E-01	WT
347	COR-L51	910555	lung	lung: small_cell_carcinoma	Carcinoma	lung	carcinoma	1.43E-01	1.56E-01	---
348	COR-L88	906808	lung	lung: small_cell_carcinoma	Carcinoma	lung	carcinoma	4.32E-01	1.03E-01	WT
349	GI-1	906871	CNS	glioma	Glioma	central nervous system	glioma	2.23E-15	6.52E-01	WT
350	GP5d	907291	colorectal	large_intestine	AdenoCarcinoma	large intestine	carcinoma	1.24E-09	-5.09E-01	WT
351	HLE	907057	liver	liver	HCC	liver	carcinoma	9.70E-12	5.65E-01	WT
352	SUP-T1	909743	H.L.M.	lymphoblastic T cell leukaemia	ALL	haematopoietic and lymphoid tissue	lymphoid neoplasm	1.49E-04	3.33E-01	WT
353	SW780	687457	bladder	bladder	Carcinoma	urinary tract	carcinoma	2.17E-17	-6.96E-01	WT
354	HCC2218	749716	breast	breast	DuctalCarcinoma	breast	carcinoma	5.35E-14	-6.21E-01	WT
355	WSU-NHL	909785	H.L.M.	lymphoid_neoplasm other	Lymphoma	haematopoietic and lymphoid tissue	lymphoid neoplasm	6.89E-02	1.84E-01	---
356	A172	687563	CNS	glioma	Glioma	central nervous system	glioma	1.23E-13	6.12E-01	WT
357	BFTC-909	910698	kidney	Renal other	Carcinoma	kidney	carcinoma	1.63E-12	5.85E-01	WT
358	CMK	910566	H.L.M.	AML	AML	haematopoietic and lymphoid tissue	haematopoietic neoplasm	8.05E-03	2.47E-01	WT
359	CA46	910703	H.L.M.	Burkitt lymphoma	BurkittsLymphoma	haematopoietic and lymphoid tissue	lymphoid neoplasm	1.18E-01	1.64E-01	WT
360	ChaGo-K-1	687596	lung	lung: NOS	Carcinoma	lung	carcinoma	2.04E-06	-4.06E-01	WT
361	Gp2D	995380	colorectal	large_intestine	AdenoCarcinoma	large intestine	carcinoma	1.01E-12	-5.90E-01	---
362	HCC1806	907047	breast	breast	DuctalCarcinoma	breast	carcinoma	9.45E-05	-3.42E-01	WT
363	SCLC-21H	753611	lung	lung: small_cell_carcinoma	Carcinoma	lung	carcinoma	8.57E-02	1.76E-01	WT
364	SK-UT-1	909732	sacrcoma	soft tissue other	Other	soft tissue	leiomyosarcoma	1.42E-11	5.61E-01	WT
365	SK-N-AS	724828	CNS	neuroblastoma	Neuroblastoma	autonomic ganglia	neuroblastoma	2.82E-12	5.79E-01	WT
366	HCC38	749717	breast	breast	DuctalCarcinoma	breast	carcinoma	2.22E-01	1.38E-01	WT
367	NCI-H1436	908469	lung	lung: small_cell_carcinoma	Carcinoma	lung	carcinoma	2.27E-02	2.19E-01	WT
368	NCI-H2171	688015	lung	lung: small_cell_carcinoma	Carcinoma	lung	carcinoma	1.95E-02	2.23E-01	WT
369	NCI-H358	908465	lung	lung: NSCLC: adenocarcinoma	AdenoCarcinoma	lung	carcinoma	4.72E-07	-4.29E-01	WT

370	NEC8	910942	germ_cell_tumour	germ_cell_tumour	GermCell	testis	germ cell tumour	2.01E-02	2.22E-01	WT
371	OMC-1	949154	uterus	cervix	Squamous	cervix	carcinoma	1.08E-04	-3.39E-01	WT
372	RO82-W-1	930083	thyroid	thyroid	FollicularCarcinoma	thyroid	carcinoma	1.01E-15	6.60E-01	WT
373	SHP-77	724872	lung	lung: small_cell_carcinoma	Carcinoma	lung	carcinoma	1.19E-02	2.36E-01	WT
374	SK-NEP-1	909730	kidney	Renal other (putative Ewings)	Other	kidney	Wilms tumour	2.62E-09	4.99E-01	WT
375	SW954	924247	uterus	vulva	Squamous	vulva	carcinoma	2.92E-03	-2.71E-01	WT
376	TE-12	946356	oesophagus	oesophagus	Squamous	oesophagus	carcinoma	4.58E-06	-3.94E-01	WT
377	NCI-H1573	908472	lung	lung: NSCLC: adenocarcinoma	AdenoCarcinoma	lung	carcinoma	3.55E-10	-5.24E-01	WT
378	NCI-H1975	924244	lung	lung: NSCLC: adenocarcinoma	AdenoCarcinoma	lung	carcinoma	1.39E-02	2.32E-01	MUT_p.T790M/Missense
379	NCI-H2196	908481	lung	lung: small_cell_carcinoma	Carcinoma	lung	carcinoma	7.57E-04	3.01E-01	WT
380	NCI-H378	688022	lung	lung: small_cell_carcinoma	Carcinoma	lung	carcinoma	1.12E-02	2.38E-01	WT
381	NH-6	925344	CNS	neuroblastoma	Neuroblastoma	autonomic ganglia	neuroblastoma	3.85E-12	5.76E-01	WT
382	OS-RC-2	909250	kidney	Renal cell carcinoma	Carcinoma	kidney	carcinoma	9.06E-06	3.83E-01	WT
383	RPMI-7951	910903	melanoma	malignant_melanoma	Melanoma	skin	malignant melanoma	9.27E-18	7.03E-01	WT
384	SK-MG-1	909729	CNS	glioma	Glioma	central nervous system	glioma	6.04E-17	6.86E-01	WT
385	T84	909761	colorectal	large_intestine	Carcinoma	large intestine	carcinoma	8.83E-14	-6.16E-01	WT
386	TE-15	753614	oesophagus	oesophagus	Squamous	oesophagus	carcinoma	1.29E-07	-4.47E-01	WT
387	NB17	949175	CNS	neuroblastoma	Neuroblastoma	autonomic ganglia	neuroblastoma	1.01E-09	5.11E-01	WT
388	NCI-H1618	753601	lung	lung: small_cell_carcinoma	Carcinoma	lung	carcinoma	1.32E-01	1.60E-01	WT
389	NCI-H2009	724873	lung	lung: NSCLC: adenocarcinoma	AdenoCarcinoma	lung	carcinoma	1.44E-02	-2.31E-01	WT
390	NCI-H2228	687816	lung	lung: NSCLC: adenocarcinoma	AdenoCarcinoma	lung	carcinoma	3.03E-03	2.70E-01	WT
391	NCI-H446	688023	lung	lung: small_cell_carcinoma	Carcinoma	lung	carcinoma	1.74E-10	5.32E-01	WT
392	NKM-1	908448	H.L.M.	AML	AML	haematopoietic and lymphoid tissue	haematopoietic neoplasm	3.50E-03	2.67E-01	WT
393	PF-382	909260	H.L.M.	lymphoblastic T cell leukaemia	ALL	haematopoietic and lymphoid tissue	lymphoid neoplasm	3.28E-04	3.18E-01	WT
394	S-117	910946	sacrcoma	soft tissue other	Sarcoma	soft tissue	sarcoma	1.64E-14	6.33E-01	WT
395	SiHa	930297	uterus	cervix	Squamous	cervix	carcinoma	6.98E-04	3.03E-01	WT
396	SK-N-FI	688087	CNS	neuroblastoma	Neuroblastoma	autonomic ganglia	neuroblastoma	9.76E-11	5.39E-01	---
397	TCCSUP	687459	bladder	bladder	Carcinoma	urinary tract	carcinoma	4.79E-09	4.91E-01	WT

398	TE-161-T	---	---	---	BurkittsLymphoma	haematopoietic and lymphoid tissue	lymphoid neoplasm	2.93E-21	7.72E-01	WT
399	NB5	949176	CNS	neuroblastoma	Neuroblastoma	autonomic ganglia	neuroblastoma	4.93E-16	6.67E-01	WT
400	NCI-H1693	687802	lung	lung: NSCLC: adenocarcinoma	AdenoCarcinoma	lung	carcinoma	2.26E-03	2.77E-01	WT
401	NCI-H2052	688058	lung	mesothelioma	Mesothelioma	pleura	mesothelioma	2.51E-14	6.28E-01	WT
402	NCI-H2291	724874	lung	lung: NSCLC: adenocarcinoma	AdenoCarcinoma	lung	carcinoma	5.92E-02	-1.89E-01	WT
403	NCI-H510A	753605	lung	lung: small_cell_carcinoma	Carcinoma	lung	carcinoma	2.06E-01	-1.41E-01	WT
404	NOS-1	925345	Ewings/Osteosarcoma	osteosarcoma	Sarcoma	bone	osteosarcoma	3.05E-12	5.78E-01	WT
405	PLC-PRF-5	925349	liver	liver	HCC	liver	carcinoma	1.19E-02	2.36E-01	WT
406	SAS	909708	head & neck	upper_aerodigestive_tract	Squamous	upper aerodigestive tract	carcinoma	1.50E-01	1.55E-01	WT
407	SIMA	753620	CNS	neuroblastoma	Neuroblastoma	autonomic ganglia	neuroblastoma	3.12E-08	4.67E-01	WT
408	SNG-M	909735	uterus	endometrium	AdenoCarcinoma	endometrium	carcinoma	4.50E-01	1.00E-01	WT
409	TCO-1	---	---	---	Carcinoma	thyroid	carcinoma	1.75E-05	-3.72E-01	---
410	TE-5	735784	oesophagus	oesophagus	Squamous	oesophagus	carcinoma	3.91E-06	-3.96E-01	WT
411	NB6	949173	CNS	neuroblastoma	Neuroblastoma	autonomic ganglia	neuroblastoma	1.47E-06	4.12E-01	WT
412	NCI-H2081	908480	lung	lung: small_cell_carcinoma	Carcinoma	lung	carcinoma	1.22E-03	2.91E-01	WT
413	NCI-H2330	688019	lung	lung: small_cell_carcinoma	Carcinoma	lung	carcinoma	1.91E-01	1.45E-01	WT
414	NCI-H526	688025	lung	lung: small_cell_carcinoma	Carcinoma	lung	carcinoma	5.77E-01	-8.33E-02	WT
415	NTERA-S-cl-D1	908454	germ_cell_tumour	germ_cell_tumour	GermCell	testis	germ cell tumour	3.08E-10	5.26E-01	WT
416	Raji	909262	H.L.M.	Burkitt lymphoma	BurkittsLymphoma	haematopoietic and lymphoid tissue	lymphoid neoplasm	4.33E-02	1.99E-01	WT
417	SBC-1	713885	lung	lung: small_cell_carcinoma	Carcinoma	lung	carcinoma	1.95E-02	2.23E-01	WT
418	SK-CO-1	909718	colorectal	large_intestine	AdenoCarcinoma	large intestine	carcinoma	2.49E-10	-5.28E-01	WT
419	SNU-387	909736	liver	liver	HCC	liver	carcinoma	3.56E-13	6.01E-01	WT
420	TC-YIK	946357	uterus	cervix	Carcinoma	cervix	carcinoma	8.37E-02	-1.77E-01	WT
421	TE-6	946355	oesophagus	oesophagus	Squamous	oesophagus	carcinoma	8.32E-03	-2.46E-01	WT
422	NCI-H209	688013	lung	lung: small_cell_carcinoma	Carcinoma	lung	carcinoma	5.21E-02	1.93E-01	WT
423	NCI-H292	753604	lung	lung: NSCLC: adenocarcinoma	Carcinoma	lung	carcinoma	2.04E-06	-4.06E-01	WT
424	NCI-H596	908459	lung	lung: NSCLC: adenocarcinoma	Carcinoma	lung	carcinoma	6.41E-01	-7.48E-02	WT
425	OAW-28	946360	ovarian	ovary	Carcinoma	ovary	carcinoma	3.58E-02	-2.05E-01	WT

426	RERF-LC-FM	930081	lung	lung: small_cell_carcinoma	Carcinoma	lung	carcinoma	2.07E-02	2.21E-01	WT
427	SBC-5	713880	lung	lung: small_cell_carcinoma	Carcinoma	lung	carcinoma	2.00E-08	4.73E-01	WT
428	SK-LU-1	909721	lung	lung: NSCLC: adenocarcinoma	AdenoCarcinoma	lung	carcinoma	4.92E-13	5.98E-01	WT
429	SW626	909753	ovarian	ovary	AdenoCarcinoma	ovary	carcinoma	9.86E-02	-1.71E-01	WT
430	TE-1	753621	oesophagus	oesophagus	Squamous	oesophagus	carcinoma	1.23E-01	-1.62E-01	WT
431	TE-8	753623	oesophagus	oesophagus	Squamous	oesophagus	carcinoma	7.05E-07	4.23E-01	WT
432	NB7	949174	CNS	neuroblastoma	Neuroblastoma	autonomic ganglia	neuroblastoma	2.96E-07	4.35E-01	WT
433	NCI-H1793	908463	lung	lung: NSCLC: adenocarcinoma	AdenoCarcinoma	lung	carcinoma	5.68E-04	3.07E-01	WT
434	NCI-H2126	687814	lung	lung: NSCLC: adenocarcinoma	AdenoCarcinoma	lung	carcinoma	4.09E-10	-5.22E-01	WT
435	NCI-H295	908466	---	Adrenal cortical carcinoma	Carcinoma	adrenal gland	adrenal cortical carcinoma	4.70E-10	5.20E-01	WT
436	NCI-H650	722066	lung	lung: NSCLC: adenocarcinoma	AdenoCarcinoma	lung	carcinoma	3.52E-14	6.25E-01	WT
437	OE19	910079	oesophagus	oesophagus	AdenoCarcinoma	oesophagus	carcinoma	2.38E-21	-7.74E-01	WT
438	RH-18	971774	sacrcoma	rhabdomyosarcoma	Sarcoma	soft tissue	rhabdomyosarcoma	1.82E-18	7.18E-01	WT
439	SCC-4	910904	head & neck	upper_aerodigestive_tract	Squamous	upper aerodigestive tract	carcinoma	7.97E-02	-1.79E-01	WT
440	SW1116	909746	colorectal	large_intestine	AdenoCarcinoma	large intestine	carcinoma	3.15E-17	-6.92E-01	WT
441	TE-10	753622	oesophagus	oesophagus	Squamous	oesophagus	carcinoma	3.58E-02	-2.05E-01	WT
442	TE-9	946353	oesophagus	oesophagus	Squamous	oesophagus	carcinoma	1.60E-03	-2.85E-01	WT
443	NCI-H1304	753599	lung	lung: small_cell_carcinoma	Carcinoma	lung	carcinoma	6.70E-04	3.04E-01	WT
444	NCI-H2141	688014	lung	lung: small_cell_carcinoma	Carcinoma	lung	carcinoma	1.48E-02	2.30E-01	WT
445	NCI-H345	688021	lung	lung: small_cell_carcinoma	Carcinoma	lung	carcinoma	2.99E-01	1.23E-01	WT
446	NCI-H810	925341	lung	lung: NSCLC: large cell	LargeCell	lung	carcinoma	4.10E-02	-2.01E-01	WT
447	OE33	910549	oesophagus	oesophagus	Other	oesophagus	other	7.14E-12	-5.69E-01	WT
448	SK-MEL-24	909725	melanoma	malignant_melanoma	Melanoma	skin	malignant melanoma	1.87E-09	5.03E-01	WT
449	SW403	995394	colorectal	large_intestine	AdenoCarcinoma	large intestine	carcinoma	3.57E-01	-1.14E-01	---
450	TE-11	946354	oesophagus	oesophagus	Squamous	oesophagus	carcinoma	2.16E-06	-4.06E-01	WT
451	TT	930299	thyroid	thyroid	Carcinoma	thyroid	carcinoma	4.94E-02	-1.95E-01	---
452	697	906800	H.L.M.	lymphoblastic Leukaemia	ALL	haematopoietic and lymphoid tissue	haematopoietic neoplasm	2.74E-05	3.64E-01	WT
453	BHY	753535	head & neck	upper_aerodigestive_tract	Squamous	upper aerodigestive tract	carcinoma	3.25E-01	-1.19E-01	WT

454	CAL-54	910952	kidney	Renal cell carcinoma	Carcinoma	kidney	carcinoma	3.28E-04	3.18E-01	WT
455	D-247MG	946367	CNS	glioma	Glioma	central nervous system	glioma	5.65E-19	7.28E-01	WT
456	D-538MG	946376	CNS	glioma	Glioma	central nervous system	glioma	3.21E-22	7.90E-01	WT
457	ES3	684055	Ewings/Osteosarcoma	Ewings sarcoma	EwingsSarcoma	bone	Ewings sarcoma-peripheral primitive neuroectodermal tumour	8.33E-12	5.67E-01	WT
458	EW-13	949166	Ewings/Osteosarcoma	Ewings sarcoma	EwingsSarcoma	bone	Ewings sarcoma-peripheral primitive neuroectodermal tumour	1.10E-12	5.89E-01	WT
459	HT-1197	907065	bladder	bladder	Carcinoma	urinary tract	carcinoma	4.82E-03	-2.59E-01	---
460	IST-SL2	753565	lung	lung: small_cell_carcinoma	Carcinoma	lung	carcinoma	1.18E-01	-1.64E-01	WT
461	KYSE-150	907317	oesophagus	oesophagus	Squamous	oesophagus	carcinoma	3.10E-01	-1.22E-01	---
462	LS-1034	917486	colorectal	large_intestine	AdenoCarcinoma	large intestine	carcinoma	1.38E-14	-6.34E-01	WT
463	MIA-PaCa-2	724870	pancreatic	pancreas	Carcinoma	pancreas	carcinoma	3.14E-07	4.35E-01	WT
464	BOKU	753536	uterus	cervix	Squamous	cervix	carcinoma	2.28E-06	4.05E-01	WT
465	CAMA-1	946382	breast	breast	Carcinoma	breast	carcinoma	1.74E-07	-4.43E-01	WT
466	D-263MG	946368	CNS	glioma	Glioma	central nervous system	glioma	9.47E-20	7.43E-01	WT
467	D-542MG	753549	CNS	glioma	Glioma	central nervous system	glioma	5.18E-20	7.48E-01	WT
468	EW-7	949160	Ewings/Osteosarcoma	Ewings sarcoma	EwingsSarcoma	bone	Ewings sarcoma-peripheral primitive neuroectodermal tumour	1.40E-10	5.35E-01	WT
469	HT-1376	907066	bladder	bladder	Carcinoma	urinary tract	carcinoma	1.56E-04	-3.32E-01	WT
470	ITO-II	924163	germ_cell_tumour	germ_cell_tumour	GermCell	testis	germ cell tumour	1.23E-02	2.36E-01	WT
471	KYSE-270	907319	oesophagus	oesophagus	Squamous	oesophagus	carcinoma	2.07E-02	2.21E-01	WT
472	LS-123	907792	colorectal	large_intestine	AdenoCarcinoma	large intestine	carcinoma	2.62E-03	-2.74E-01	WT
473	MKN45	925340	gastric	stomach	AdenoCarcinoma	stomach	carcinoma	7.71E-12	-5.68E-01	WT
474	BPH-1	924105	prostate	prostate	Other	prostate	hyperplasia	4.35E-06	-3.95E-01	WT
475	Caov-4	949090	ovarian	ovary	AdenoCarcinoma	ovary	carcinoma	6.39E-02	-1.86E-01	WT
476	D-336MG	946369	CNS	glioma	Glioma	central nervous system	glioma	9.80E-15	6.38E-01	WT
477	D-556MED	946381	CNS	medulloblastoma	Medulloblastoma	central nervous system	primitive neuroectodermal tumour-medulloblastoma	1.55E-06	4.11E-01	WT

478	ES5	684057	Ewings/Osteosarcoma	Ewings sarcoma	EwingsSarcoma	bone	Ewings sarcoma-peripheral primitive neuroectodermal tumour	9.76E-11	5.39E-01	WT
479	HT-3	907068	uterus	cervix	Carcinoma	cervix	carcinoma	3.26E-03	-2.69E-01	WT
480	K052	907284	H.L.M.	AML	AML	haematopoietic and lymphoid tissue	haematopoietic neoplasm	4.45E-02	1.98E-01	WT
481	KYSE-520	753575	oesophagus	oesophagus	Squamous	oesophagus	carcinoma	2.10E-01	-1.40E-01	WT
482	M059J	949094	CNS	glioma	Glioma	central nervous system	glioma	6.04E-17	6.86E-01	WT
483	MOG-G-UVW	908145	CNS	glioma	Glioma	central nervous system	glioma	7.05E-16	6.63E-01	---
484	8505C	924102	thyroid	thyroid	Anaplastic	thyroid	carcinoma	1.11E-05	3.79E-01	WT
485	BT-20	906801	breast	breast	Carcinoma	breast	carcinoma	5.04E-10	-5.20E-01	WT
486	CAPAN-1	753624	pancreatic	pancreas	DuctalCarcinoma	pancreas	carcinoma	1.82E-16	-6.76E-01	WT
487	D-384MED	946379	CNS	medulloblastoma	Medulloblastoma	central nervous system	primitive neuroectodermal tumour-medulloblastoma	6.58E-08	4.57E-01	WT
488	DOK	910936	head & neck	upper_aerodigestive_tract	Other	upper aerodigestive tract	other	9.20E-02	-1.73E-01	WT
489	ES6	949157	Ewings/Osteosarcoma	Ewings sarcoma	EwingsSarcoma	bone	Ewings sarcoma-peripheral primitive neuroectodermal tumour	1.10E-12	5.89E-01	WT
490	GA-10-Clone-20	906866	H.L.M.	Burkitt lymphoma	BurkittsLymphoma	haematopoietic and lymphoid tissue	lymphoid neoplasm	4.16E-12	-5.75E-01	---
491	HT55	907287	colorectal	large_intestine	AdenoCarcinoma	large intestine	carcinoma	5.76E-18	-7.07E-01	WT
492	K5	924238	thyroid	thyroid	FollicularCarcinoma	thyroid	carcinoma	1.44E-15	6.56E-01	---
493	LAN-5	923828	CNS	neuroblastoma	Neuroblastoma	autonomic ganglia	neuroblastoma	9.08E-11	5.40E-01	---
494	MRK-nu-1	908151	breast	breast	Carcinoma	breast	carcinoma	7.35E-13	-5.94E-01	WT
495	A431	910925	melanoma	other	Squamous	skin	carcinoma	1.86E-04	-3.29E-01	WT
496	CAL-148	924106	breast	breast	DuctalCarcinoma	breast	carcinoma	8.85E-07	-4.19E-01	WT
497	Capan-2	910915	pancreatic	pancreas	AdenoCarcinoma	pancreas	carcinoma	3.32E-08	-4.66E-01	WT
498	D-392MG	946370	CNS	glioma	Glioma	central nervous system	glioma	6.04E-17	6.86E-01	---
499	DoTc2-4510	906843	uterus	cervix	Carcinoma	cervix	carcinoma	6.58E-08	-4.57E-01	WT
500	ES7	684059	Ewings/Osteosarcoma	Ewings sarcoma	EwingsSarcoma	bone	Ewings sarcoma-peripheral primitive neuroectodermal tumour	2.79E-13	6.04E-01	WT
501	GOTO-P3	906876	CNS	neuroblastoma	Neuroblastoma	autonomic ganglia	neuroblastoma	2.01E-13	6.07E-01	---
502	HTC-C3	924151	thyroid	thyroid	Carcinoma	thyroid	carcinoma	3.63E-01	-1.13E-01	WT
503	KGN	924186	ovarian	ovary	Other	ovary	other	1.38E-14	6.34E-01	WT
504	LAN-6	949170	CNS	neuroblastoma	Neuroblastoma	autonomic ganglia	neuroblastoma	5.41E-10	5.19E-01	WT

505	MC-IXC	908118	CNS	neuroblastoma	Neuroblastoma	autonomic ganglia	neuroblastoma	2.81E-09	4.98E-01	WT
506	MZ7-mel	753596	melanoma	malignant_melanoma	Melanoma	skin	malignant melanoma	7.59E-09	4.86E-01	WT
507	AGS	906790	gastric	stomach	AdenoCarcinoma	stomach	carcinoma	1.65E-08	-4.75E-01	---
508	CAL-27	910916	head & neck	upper_aerodigestive_tract	Squamous	upper aerodigestive tract	carcinoma	9.53E-06	-3.82E-01	WT
509	CHP-212	906820	CNS	neuroblastoma	Neuroblastoma	autonomic ganglia	neuroblastoma	2.37E-13	6.05E-01	WT
510	D-397MG	946371	CNS	glioma	Glioma	central nervous system	glioma	7.05E-16	6.63E-01	WT
511	ECC12	906849	gastric	stomach	Carcinoma	stomach	carcinoma	1.86E-04	3.29E-01	WT
512	ES8	949155	Ewings/Osteosarcoma	Ewings sarcoma	EwingsSarcoma	bone	Ewings sarcoma-peripheral primitive neuroectodermal tumour	6.30E-11	5.44E-01	WT
513	HCC1419	907045	breast	breast	DuctalCarcinoma	breast	carcinoma	1.53E-11	-5.60E-01	WT
514	HuH-28	924163	biliary_tract	biliary_tract	Carcinoma	biliary tract	carcinoma	1.31E-11	5.62E-01	---
515	KNS-42	907282	CNS	glioma	Glioma	central nervous system	glioma	3.05E-12	5.78E-01	WT
516	LB373-MEL-D	753581	melanoma	malignant_melanoma	Melanoma	skin	malignant melanoma	3.56E-12	5.77E-01	WT
517	MDA-MB-175-VII	908120	breast	breast	DuctalCarcinoma	breast	carcinoma	2.30E-14	-6.29E-01	WT
518	NBsusSR	925342	CNS	neuroblastoma	Neuroblastoma	autonomic ganglia	neuroblastoma	1.08E-09	5.10E-01	WT
519	AML-193	949089	H.L.M.	AML	AML	haematopoietic and lymphoid tissue	haematopoietic neoplasm	9.20E-02	1.73E-01	WT
520	CAL-39	924107	uterus	vulva	Squamous	vulva	carcinoma	1.37E-03	-2.88E-01	WT
521	COLO-668	910692	lung	lung: small_cell_carcinoma	Carcinoma	lung	carcinoma	1.12E-02	-2.38E-01	WT
522	D-458MED	946381	CNS	medulloblastoma	Medulloblastoma	central nervous system	primitive neuroectodermal tumour-medulloblastoma	6.29E-07	4.24E-01	WT
523	EW-1	949163	Ewings/Osteosarcoma	Ewings sarcoma	EwingsSarcoma	bone	Ewings sarcoma-peripheral primitive neuroectodermal tumour	4.27E-08	4.63E-01	WT
524	HCE-4	753559	oesophagus	oesophagus	Squamous	oesophagus	carcinoma	4.45E-07	4.29E-01	WT
525	HuO9	907072	Ewings/Osteosarcoma	osteosarcoma	Sarcoma	bone	osteosarcoma	1.28E-12	5.88E-01	WT
526	KNS-81-FD	924188	CNS	glioma	Glioma	central nervous system	glioma	2.43E-18	7.15E-01	MUT_p.G598V/Missense
527	LB831-BLC	753584	bladder	bladder	Carcinoma	urinary tract	carcinoma	9.00E-15	6.39E-01	WT
528	MDA-MB-415	924240	breast	breast	Carcinoma	breast	carcinoma	5.33E-13	-5.97E-01	WT
529	NB1	949179	CNS	neuroblastoma	Neuroblastoma	autonomic ganglia	neuroblastoma	2.79E-11	5.54E-01	WT
530	BCPAP	924104	thyroid	thyroid	Carcinoma	thyroid	carcinoma	3.43E-09	4.96E-01	WT
531	CAL-51	910927	breast	breast	Carcinoma	breast	carcinoma	8.98E-02	1.74E-01	WT

532	D-245MG	946366	CNS	glioma	Glioma	central nervous system	glioma	7.71E-16	6.62E-01	WT
533	D-502MG	946373	CNS	glioma	Glioma	central nervous system	glioma	9.80E-15	6.38E-01	WT
534	ES1	949158	Ewings/Osteosarcoma	Ewings sarcoma	EwingsSarcoma	bone	Ewings sarcoma-peripheral primitive neuroectodermal tumour	4.38E-10	5.21E-01	WT
535	EW-11	684062	Ewings/Osteosarcoma	Ewings sarcoma	EwingsSarcoma	bone	Ewings sarcoma-peripheral primitive neuroectodermal tumour	9.43E-10	5.12E-01	WT
536	HeLaSF	687569	uterus	cervix	AdenoCarcinoma	cervix	carcinoma	1.37E-07	4.46E-01	WT
537	IM-9	753563	H.L.M.	Myeloma	Myeloma	haematopoietic and lymphoid tissue	lymphoid neoplasm	2.27E-02	2.19E-01	WT
538	KP-N-YS	946363	CNS	neuroblastoma	Neuroblastoma	autonomic ganglia	neuroblastoma	5.84E-15	6.43E-01	WT
539	LC-1F	907785	lung	lung: NSCLC: squamous_cell_carcinoma	Squamous	lung	carcinoma	3.91E-01	-1.09E-01	WT
540	MFH-ino	925343	sacrcoma	soft tissue other	Sarcoma	soft tissue	malignant fibrous histiocytoma-pleomorphic sarcoma	3.15E-17	6.92E-01	WT
541	NB12	949172	CNS	neuroblastoma	Neuroblastoma	autonomic ganglia	neuroblastoma	4.86E-12	5.73E-01	WT
542	U-266	753615	H.L.M.	Myeloma	Myeloma	haematopoietic and lymphoid tissue	lymphoid neoplasm	3.29E-02	2.07E-01	---
543	BB30-HNC	753531	head & neck	upper_aerodigestive_tract	Squamous	upper aerodigestive tract	carcinoma	4.81E-02	-1.96E-01	WT
544	CP67-MEL	949092	melanoma	malignant_melanoma	Melanoma	skin	malignant melanoma	1.13E-11	5.64E-01	WT
545	LB1047-RCC	753577	kidney	Renal cell carcinoma	Carcinoma	kidney	carcinoma	2.75E-08	4.69E-01	---
546	NCI-H2107	924246	lung	lung: small_cell_carcinoma	Carcinoma	lung	carcinoma	2.06E-01	1.41E-01	WT
547	SK-N-DZ	688086	CNS	neuroblastoma	Neuroblastoma	autonomic ganglia	neuroblastoma	1.42E-09	5.07E-01	WT
548	EW-24	949168	Ewings/Osteosarcoma	Ewings sarcoma	EwingsSarcoma	bone	Ewings sarcoma-peripheral primitive neuroectodermal tumour	1.13E-13	6.13E-01	WT
549	HSC-2	753562	head & neck	upper_aerodigestive_tract	Squamous	upper aerodigestive tract	carcinoma	2.94E-02	-2.11E-01	---
550	MMAC-SF	925339	melanoma	malignant_melanoma	Melanoma	skin	malignant melanoma	1.76E-08	4.74E-01	WT
551	NCI-H1395	684681	lung	lung: NSCLC: adenocarcinoma	AdenoCarcinoma	lung	carcinoma	2.36E-05	-3.66E-01	---
552	NCI-H2342	687819	lung	lung: NSCLC: adenocarcinoma	AdenoCarcinoma	lung	carcinoma	4.09E-10	-5.22E-01	WT

553	U-2-OS	909776	Ewings/Osteosarcoma	osteosarcoma	Sarcoma	bone	osteosarcoma	1.42E-11	5.61E-01	WT
554	BB49-HNC	753532	head & neck	upper_aerodigestive_tract	Squamous	upper aerodigestive tract	carcinoma	1.74E-10	5.32E-01	WT
555	HA7-RCC	753558	kidney	Renal cell carcinoma	Carcinoma	kidney	carcinoma	3.28E-04	3.18E-01	WT
556	LB2241-RCC	753578	kidney	Renal cell carcinoma	Carcinoma	kidney	carcinoma	3.51E-06	3.98E-01	WT
557	D-392MG	946370	CNS	glioma	Glioma	central nervous system	glioma	8.44E-18	7.04E-01	WT
558	NCI-H2087	724834	lung	lung: NSCLC: adenocarcinoma	AdenoCarcinoma	lung	carcinoma	1.94E-05	-3.70E-01	---
559	U-698-M	909777	H.L.M.	B cell Leukaemia	ALL	haematopoietic and lymphoid tissue	lymphoid neoplasm	2.27E-02	2.19E-01	WT
560	D-423MG	946372	CNS	glioma	Glioma	central nervous system	glioma	2.21E-18	7.16E-01	WT
561	MKN7	924250	gastric	stomach	Carcinoma	stomach	carcinoma	1.18E-03	-2.92E-01	---
562	NCI-H1437	687794	lung	lung: NSCLC: adenocarcinoma	AdenoCarcinoma	lung	carcinoma	3.56E-12	-5.77E-01	---
563	BFTC-905	910926	bladder	bladder	Carcinoma	urinary tract	carcinoma	2.68E-06	-4.02E-01	---
564	BB65-RCC	753533	kidney	Renal cell carcinoma	Carcinoma	kidney	carcinoma	9.26E-04	2.97E-01	WT
565	HO-1-N-1	924111	head & neck	upper_aerodigestive_tract	Squamous	upper aerodigestive tract	carcinoma	5.35E-03	-2.57E-01	WT
566	LB2518-MEL	753579	melanoma	malignant_melanoma	Melanoma	skin	malignant melanoma	9.51E-08	4.52E-01	WT
567	PANC-03-27	925346	pancreatic	pancreas	DuctalCarcinoma	pancreas	carcinoma	2.55E-02	-2.15E-01	WT
568	HAL-01	949153	H.L.M.	lymphoblastic Leukaemia	ALL	haematopoietic and lymphoid tissue	lymphoid neoplasm	1.04E-03	2.94E-01	WT
569	EW-16	949165	Ewings/Osteosarcoma	Ewings sarcoma	EwingsSarcoma	bone	Ewings sarcoma-peripheral primitive neuroectodermal tumour	2.20E-07	4.40E-01	WT
570	KYSE-450	907320	oesophagus	oesophagus	Squamous	oesophagus	carcinoma	2.90E-01	-1.25E-01	MUT_p.S768I/Missense
571	IA-LM	910779	lung	lung: NSCLC: large cell	LargeCell	lung	carcinoma	5.41E-10	5.19E-01	WT
572	NCI-H2087	724834	lung	lung: NSCLC: adenocarcinoma	AdenoCarcinoma	lung	carcinoma	5.00E-07	-4.28E-01	WT
573	PD1503a	---	---	---	Other	#N/A	#N/A	1.32E-01	-1.60E-01	---
574	CTB-1	949088	H.L.M.	B cell lymphoma	Lymphoma	haematopoietic and lymphoid tissue	lymphoid neoplasm	2.55E-02	2.15E-01	---
575	VM-CUB-1	909780	bladder	bladder	Carcinoma	urinary tract	carcinoma	5.45E-04	-3.08E-01	WT
576	C32	906830	melanoma	malignant_melanoma	Melanoma	skin	malignant melanoma	2.42E-08	4.70E-01	WT
577	IST-SL1	753564	lung	lung: small_cell_carcinoma	Carcinoma	lung	carcinoma	8.98E-02	1.74E-01	WT

578	LB647-SCLC	753582	lung	lung: small_cell_carcinoma	Carcinoma	lung	carcinoma	1.87E-03	2.81E-01	WT
579	PANC-10-05	925348	pancreatic	pancreas	DuctalCarcinoma	pancreas	carcinoma	4.83E-06	-3.93E-01	WT
580	NB14	949178	CNS	neuroblastoma	Neuroblastoma	autonomic ganglia	neuroblastoma	6.22E-10	5.17E-01	WT
581	HCC1395	749712	breast	breast	DuctalCarcinoma	breast	carcinoma	4.53E-14	6.22E-01	WT
582	NH-12	908447	CNS	neuroblastoma	Neuroblastoma	autonomic ganglia	neuroblastoma	3.76E-08	4.64E-01	WT
583	BHT-101	906696	thyroid	thyroid	Anaplastic	thyroid	carcinoma	2.65E-04	3.22E-01	WT
584	NCI-H1930	924245	lung	lung: small_cell_carcinoma	Carcinoma	lung	carcinoma	2.20E-02	2.19E-01	WT
585	DMS-273	687985	lung	lung: small_cell_carcinoma	Carcinoma	lung	carcinoma	3.85E-12	5.76E-01	WT
586	WM-115	909784	melanoma	malignant_melanoma	Melanoma	skin	malignant melanoma	4.91E-15	6.45E-01	WT
587	CaR-1	924108	colorectal	large_intestine	AdenoCarcinoma	large intestine	carcinoma	1.63E-04	-3.32E-01	WT
588	J82	753566	bladder	bladder	Carcinoma	urinary tract	carcinoma	7.66E-10	5.14E-01	WT
589	LB996-RCC	753585	kidney	Renal cell carcinoma	Carcinoma	kidney	carcinoma	3.14E-03	2.70E-01	WT
590	SKG-IIIa	930298	uterus	cervix	Carcinoma	cervix	carcinoma	4.99E-03	-2.59E-01	WT
591	SK-MM-2	753612	H.L.M.	Myeloma	Myeloma	haematopoietic and lymphoid tissue	lymphoid neoplasm	4.26E-01	1.04E-01	---
592	NCI-H1437	687794	lung	lung: NSCLC: adenocarcinoma	AdenoCarcinoma	lung	carcinoma	1.63E-12	-5.85E-01	---
593	KM-H2	909976	H.L.M.	Hodgkin lymphoma	HodgekinsLymphoma	haematopoietic and lymphoid tissue	lymphoid neoplasm	7.15E-05	3.47E-01	---
594	HDLM-2	924110	H.L.M.	Hodgkin lymphoma	HodgekinsLymphoma	haematopoietic and lymphoid tissue	lymphoid neoplasm	6.72E-02	1.85E-01	---
595	NCI-H1395	684681	lung	lung: NSCLC: adenocarcinoma	AdenoCarcinoma	lung	carcinoma	1.19E-04	-3.38E-01	WT
596	NCI-H1105	908468	lung	lung: small_cell_carcinoma	Carcinoma	lung	carcinoma	4.69E-02	1.96E-01	WT
597	KU-19-19	907312	bladder	bladder	Carcinoma	urinary tract	carcinoma	1.32E-01	1.60E-01	WT
598	ZR-75-30	909907	breast	breast	DuctalCarcinoma	breast	carcinoma	5.84E-09	-4.89E-01	WT
599	COR-L96CAR	971776	lung	lung: small_cell_carcinoma	Carcinoma	lung	carcinoma	1.66E-01	-1.51E-01	WT
600	LCLC-97TM1	946361	lung	lung: NSCLC: large cell	LargeCell	lung	carcinoma	8.21E-04	-2.99E-01	WT
601	SK-MES-1	909728	lung	lung: NSCLC: squamous_cell_carcinoma	Squamous	lung	carcinoma	5.37E-06	3.91E-01	---
602	EW-3	949161	Ewings/Osteosarcoma	Ewings sarcoma	EwingsSarcoma	bone	Ewings sarcoma-peripheral primitive neuroectodermal tumour	8.10E-09	4.85E-01	---

603	SCC-15		910911	head & neck	upper_aerodigestive_tract	Squamous	upper aerodigestive tract	carcinoma	1.08E-04	3.39E-01	WT
604	SK-MM-2		753612	H.L.M.	Myeloma	Myeloma	haematopoietic and lymphoid tissue	lymphoid neoplasm	3.68E-01	1.12E-01	WT
605	NCI-H250		924241	lung	lung: small_cell_carcinoma	Carcinoma	lung	carcinoma	3.30E-01	-1.18E-01	---
606	GMS-10		906873	CNS	glioma	Glioma	central nervous system	glioma	2.21E-18	7.16E-01	WT
607		380	924101	H.L.M.	ALL	ALL	haematopoietic and lymphoid tissue	lymphoid neoplasm	2.86E-02	2.12E-01	WT
608	KP-N-RT-BM-1		924189	CNS	neuroblastoma	Neuroblastoma	autonomic ganglia	neuroblastoma	9.85E-09	4.82E-01	WT
609	MDA-MB-157		925338	breast	breast	DuctalCarcinoma	breast	carcinoma	7.05E-16	6.63E-01	WT
610	EW-22		949167	Ewings/Osteosarcoma	Ewings sarcoma	EwingsSarcoma	bone	Ewings sarcoma-peripheral primitive neuroectodermal tumour	8.10E-09	4.85E-01	WT
611	LB771-HNC		753583	head & neck	upper_aerodigestive_tract	Squamous	upper aerodigestive tract	carcinoma	7.04E-03	2.50E-01	WT
612	D-566MG		946377	CNS	glioma	Glioma	central nervous system	glioma	5.46E-22	7.86E-01	WT
613	DOHH-2		906842	H.L.M.	B cell lymphoma	Lymphoma	haematopoietic and lymphoid tissue	lymphoid neoplasm	6.23E-02	1.87E-01	WT
614	HSC-2		753562	head & neck	upper_aerodigestive_tract	Squamous	upper aerodigestive tract	carcinoma	2.47E-02	-2.16E-01	WT
615	NCI-H2029		688011	lung	lung: small_cell_carcinoma	Carcinoma	lung	carcinoma	3.48E-02	-2.06E-01	WT
616	RH-1		971773	sacrcoma	rhabdomyosarcoma (putative Ewings)	Sarcoma	soft tissue	rhabdomyosarcoma	3.56E-13	6.01E-01	---
617	22RV1		924100	prostate	prostate	AdenoCarcinoma	prostate	carcinoma	2.33E-02	-2.18E-01	WT
618	CP66-MEL		753546	melanoma	malignant_melanoma	Melanoma	skin	malignant melanoma	1.63E-09	5.05E-01	WT
619	L-363		924239	H.L.M.	Myeloma	Myeloma	haematopoietic and lymphoid tissue	lymphoid neoplasm	4.33E-02	1.99E-01	WT
620	MZ2-MEL.		971777	melanoma	malignant_melanoma	Melanoma	skin	malignant melanoma	3.85E-12	5.76E-01	---
621	VMRC-MELG	---		---	---	Melanoma	skin	malignant melanoma	2.44E-15	6.51E-01	WT
622	EW-18		949164	Ewings/Osteosarcoma	Ewings sarcoma	EwingsSarcoma	bone	Ewings sarcoma-peripheral primitive neuroectodermal tumour	8.65E-09	4.84E-01	WT
623	EW-12		949162	Ewings/Osteosarcoma	Ewings sarcoma	EwingsSarcoma	bone	Ewings sarcoma-peripheral primitive neuroectodermal tumour	8.37E-07	4.20E-01	WT
624	A253		906794	---	other	Carcinoma	salivary gland	carcinoma	5.49E-02	-1.91E-01	WT

625	MKN7		924250	gastric	stomach	Carcinoma	stomach	carcinoma	4.24E-04	-3.13E-01	WT
626	KM-H2		909976	H.L.M.	Hodgkin lymphoma	HodgekinsLymphoma	haematopoietic and lymphoid tissue	lymphoid neoplasm	4.61E-04	3.11E-01	WT
627	SNU-C1		910905	colorectal	large_intestine	AdenoCarcinoma	large intestine	carcinoma	7.19E-24	-8.20E-01	---
628	COLO-792		906814	melanoma	malignant_melanoma	Melanoma	skin	malignant melanoma	1.23E-05	3.78E-01	---
629	SF295		905985	CNS	glioma	Glioma	central nervous system	glioma	1.15E-16	6.80E-01	WT
630	SK-MEL-2		905955	melanoma	malignant_melanoma	Melanoma	skin	malignant melanoma	7.47E-07	4.22E-01	WT
631	NCI-H838		910399	lung	lung: NSCLC: NOS	NSCLC	lung	carcinoma	1.53E-09	5.06E-01	WT
632	LOXIMVI		905974	melanoma	malignant_melanoma	Melanoma	skin	malignant melanoma	8.25E-15	6.39E-01	WT
633	LK-2		687787	lung	lung: NSCLC: squamous_cell_carcinoma	Squamous	lung	carcinoma	2.43E-01	-1.34E-01	WT
634	U-87-MG		687590	CNS	glioma	Glioma	central nervous system	glioma	2.17E-17	6.96E-01	WT
635	HCC1187		749711	breast	breast	DuctalCarcinoma	breast	carcinoma	3.36E-01	-1.17E-01	WT
636		5637	687452	bladder	bladder	Carcinoma	urinary tract	carcinoma	3.78E-02	-2.03E-01	WT
637	NB16		801737	CNS	neuroblastoma	Neuroblastoma	autonomic ganglia	neuroblastoma	4.01E-08	4.63E-01	---
638	HT-1197		907065	bladder	bladder	Carcinoma	urinary tract	carcinoma	1.78E-04	-3.30E-01	WT
639	NCI-H23		905942	lung	lung: NSCLC: NOS	NSCLC	lung	carcinoma	8.33E-12	5.67E-01	WT
640	OVCAR-5		905969	ovarian	ovary	Carcinoma	ovary	carcinoma	1.18E-03	-2.92E-01	---
641	RPMI-8226		905964	H.L.M.	Myeloma	Myeloma	haematopoietic and lymphoid tissue	haematopoietic neoplasm	7.06E-02	-1.83E-01	MUT_p.T751I/Missense
642	DMS-153		687984	lung	lung: small_cell_carcinoma	Carcinoma	lung	carcinoma	6.72E-02	-1.85E-01	WT
643	LU-134-A		753588	lung	lung: small_cell_carcinoma	Carcinoma	lung	carcinoma	2.18E-01	-1.39E-01	WT
644	NB13		949177	CNS	neuroblastoma	Neuroblastoma	autonomic ganglia	neuroblastoma	2.83E-06	4.01E-01	WT
645	NCI-ADR-RES		905987	ovarian	ovary	Carcinoma	breast	carcinoma	5.46E-09	4.90E-01	WT
646	U251		905983	CNS	glioma	Glioma	central nervous system	glioma	3.83E-14	6.24E-01	WT
647	PC-3		905934	prostate	prostate	AdenoCarcinoma	prostate	carcinoma	3.04E-01	1.22E-01	---
648	CAL-72		906827	Ewings/Osteosarcoma	osteosarcoma	Sarcoma	bone	osteosarcoma	8.12E-14	6.16E-01	---
649	HL-60		905938	H.L.M.	AML	AML	haematopoietic and lymphoid tissue	haematopoietic neoplasm	1.21E-01	1.63E-01	WT
650	RERF-LC-MS		910931	lung	lung: NSCLC: adenocarcinoma	AdenoCarcinoma	lung	carcinoma	2.34E-07	4.39E-01	WT
651	BE-13		906763	H.L.M.	lymphoblastic Leukaemia	ALL	haematopoietic and lymphoid tissue	haematopoietic neoplasm	7.24E-02	1.82E-01	WT
652	NCI-H157		911847	lung	lung: NSCLC: squamous_cell_carcinoma	Squamous	lung	carcinoma	3.08E-10	5.26E-01	WT
653	HGC-27		907055	gastric	stomach	Carcinoma	stomach	carcinoma	2.79E-11	5.54E-01	WT

654	KM12	905989	colorectal	large_intestine	AdenoCarcinoma	large intestine	carcinoma	1.30E-10	-5.36E-01	WT
655	SK-N-FI	688087	CNS	neuroblastoma	Neuroblastoma	autonomic ganglia	neuroblastoma	7.15E-10	5.15E-01	WT
656	OVCAR-4	905990	ovarian	ovary	Carcinoma	ovary	carcinoma	4.10E-02	-2.01E-01	---
657	PANC-08-13	925347	pancreatic	pancreas	DuctalCarcinoma	pancreas	carcinoma	7.35E-13	-5.94E-01	---
658	NCI-H522	905944	lung	lung: NSCLC: NOS	NSCLC	lung	carcinoma	8.94E-08	4.52E-01	WT
659	Hs-578-T	905957	breast	breast	Carcinoma	breast	carcinoma	1.26E-16	6.79E-01	WT
660	SK-MEL-28	905954	melanoma	malignant_melanoma	Melanoma	skin	malignant melanoma	1.84E-07	4.42E-01	MUT_p.P753S/Missense
661	LB1047-RCC	753577	kidney	Renal cell carcinoma	Carcinoma	kidney	carcinoma	1.24E-06	4.14E-01	WT
662	TT	930299	thyroid	thyroid	Carcinoma	thyroid	carcinoma	5.92E-02	-1.89E-01	WT
663	U-266	753615	H.L.M.	Myeloma	Myeloma	haematopoietic and lymphoid tissue	lymphoid neoplasm	3.99E-02	2.02E-01	WT
664	Becker	906746	CNS	glioma	Glioma	central nervous system	glioma	8.33E-12	5.67E-01	---
665	NB10	949171	CNS	neuroblastoma	Neuroblastoma	autonomic ganglia	neuroblastoma	2.04E-04	3.27E-01	---
666	MOLT-4	905958	H.L.M.	lymphoblastic Leukaemia	ALL	haematopoietic and lymphoid tissue	haematopoietic neoplasm	8.90E-04	2.98E-01	WT
667	BT-549	905951	breast	breast	DuctalCarcinoma	breast	carcinoma	1.10E-15	6.59E-01	WT
668	HuO-3N1	909977	Ewings/Osteosarcoma	osteosarcoma	Sarcoma	bone	osteosarcoma	2.43E-18	7.15E-01	WT
669	CRO-AP2	906807	H.L.M.	B cell lymphoma	Lymphoma	haematopoietic and lymphoid tissue	lymphoid neoplasm	1.40E-01	1.57E-01	WT
670	SNB19	905966	CNS	glioma	Glioma	central nervous system	glioma	6.79E-13	5.94E-01	WT
671	NCI-H1437	687794	lung	lung: NSCLC: adenocarcinoma	AdenoCarcinoma	lung	carcinoma	6.13E-12	-5.71E-01	WT
672	BFTC-905	910926	bladder	bladder	Carcinoma	urinary tract	carcinoma	3.53E-07	-4.33E-01	WT
673	SK-MEL-3	909724	melanoma	malignant_melanoma	Melanoma	skin	malignant melanoma	1.47E-06	4.12E-01	---
674	A498	905948	kidney	Renal other	Carcinoma	kidney	carcinoma	2.04E-06	4.06E-01	WT
675	EHEB	906854	H.L.M.	lymphoid_neoplasm other	CLL	haematopoietic and lymphoid tissue	lymphoid neoplasm	2.75E-01	-1.28E-01	WT
676	EPLC-272H	753556	lung	lung: NSCLC: squamous_cell_carcinoma	Squamous	lung	carcinoma	4.50E-01	-1.00E-01	WT
677	KYSE-410	753574	oesophagus	oesophagus	Squamous	oesophagus	carcinoma	5.41E-10	-5.19E-01	WT
678	KYSE-150	907317	oesophagus	oesophagus	Squamous	oesophagus	carcinoma	3.38E-02	-2.07E-01	WT
679	CTB-1	949088	H.L.M.	B cell lymphoma	Lymphoma	haematopoietic and lymphoid tissue	lymphoid neoplasm	1.18E-01	1.64E-01	WT
680	HDLM-2	924110	H.L.M.	Hodgkin lymphoma	HodgekinsLymphoma	haematopoietic and lymphoid tissue	lymphoid neoplasm	1.32E-01	1.60E-01	WT
681	RXF393	905978	kidney	Renal cell carcinoma	Carcinoma	kidney	carcinoma	1.57E-13	6.10E-01	WT
682	EoL-1-cell	906856	H.L.M.	haematopoietic_neoplasm other	CML	haematopoietic and lymphoid tissue	haematopoietic neoplasm	5.35E-02	1.92E-01	---
683	CCRF-CEM	905952	H.L.M.	lymphoblastic Leukaemia	ALL	haematopoietic and lymphoid tissue	haematopoietic neoplasm	2.78E-02	2.13E-01	WT

684	GR-ST	906877	H.L.M.	lymphoblastic Leukaemia	ALL	haematopoietic and lymphoid tissue	haematopoietic neoplasm	2.01E-02	2.22E-01	WT
685	NCI-H460	905943	lung	lung: NSCLC: large cell	LargeCell	lung	carcinoma	5.97E-06	3.89E-01	WT
686	K5	924238	thyroid	thyroid	FollicularCarcinoma	thyroid	carcinoma	7.47E-14	6.17E-01	WT
687	EW-3	949161	Ewings/Osteosarcoma	Ewings sarcoma	EwingsSarcoma	bone	Ewings sarcoma-peripheral primitive neuroectodermal tumour	1.96E-07	4.41E-01	WT
688	NCI-H250	924241	lung	lung: small_cell_carcinoma	Carcinoma	lung	carcinoma	1.53E-01	-1.54E-01	WT
689	RH-1	971773	sacrcoma	rhabdomyosarcoma (putative Ewings)	Sarcoma	soft tissue	rhabdomyosarcoma	2.51E-14	6.28E-01	WT
690	SR	905965	H.L.M.	lymphoid_neoplasm other	Other	haematopoietic and lymphoid tissue	lymphoid neoplasm	7.57E-04	3.01E-01	WT
691	EKVX	905970	lung	lung: NSCLC: adenocarcinoma	AdenoCarcinoma	lung	carcinoma	8.77E-02	1.75E-01	---
692	LOUCY	907789	H.L.M.	lymphoblastic T cell leukaemia	ALL	haematopoietic and lymphoid tissue	lymphoid neoplasm	4.34E-03	2.62E-01	WT
693	DJM-1	906841	melanoma	other	Other	skin	adnexal tumour	2.88E-05	-3.63E-01	WT
694	HuP-T3	907285	pancreatic	pancreas	Carcinoma	pancreas	carcinoma	1.05E-02	-2.40E-01	WT
695	HOP-92	905973	lung	lung: NSCLC: large cell	LargeCell	lung	carcinoma	1.53E-11	5.60E-01	WT
696	SK-MES-1	909728	lung	lung: NSCLC: squamous_cell_carcinoma	Squamous	lung	carcinoma	3.28E-04	3.18E-01	WT
697	OVCAR-8	905991	ovarian	ovary	Carcinoma	ovary	carcinoma	1.62E-10	5.33E-01	WT
698	NB69	908440	CNS	neuroblastoma	Neuroblastoma	autonomic ganglia	neuroblastoma	7.29E-11	5.43E-01	WT
699	SNU-C1	910905	colorectal	large_intestine	AdenoCarcinoma	large intestine	carcinoma	3.31E-24	-8.26E-01	WT
700	MZ2-MEL.	971777	melanoma	malignant_melanoma	Melanoma	skin	malignant melanoma	6.61E-12	5.70E-01	---
701	Malme-3M	905953	melanoma	malignant_melanoma	Melanoma	skin	malignant melanoma	1.12E-08	4.80E-01	WT
702	ACHN	905950	kidney	Renal cell carcinoma	Carcinoma	kidney	carcinoma	1.19E-04	3.38E-01	WT
703	SK-OV-3	905959	ovarian	ovary	AdenoCarcinoma	ovary	carcinoma	3.20E-02	2.08E-01	WT
704	SN12C	905979	kidney	Renal cell carcinoma	Carcinoma	kidney	carcinoma	2.57E-13	6.05E-01	WT
705	SW620	905962	colorectal	large_intestine	AdenoCarcinoma	large intestine	carcinoma	2.57E-01	1.31E-01	WT
706	Becker	906746	CNS	glioma	Glioma	central nervous system	glioma	2.07E-11	5.57E-01	WT
707	G-361	906865	melanoma	malignant_melanoma	Melanoma	skin	malignant melanoma	5.00E-07	4.28E-01	WT
708	HSC-4	907062	head & neck	upper_aerodigestive_tract	Squamous	upper aerodigestive tract	carcinoma	6.80E-03	-2.51E-01	WT
709	BL-41	910706	H.L.M.	Burkitt lymphoma	BurkittsLymphoma	haematopoietic and lymphoid tissue	lymphoid neoplasm	1.32E-01	1.60E-01	WT
710	TGW	910780	CNS	neuroblastoma	Neuroblastoma	autonomic ganglia	neuroblastoma	1.30E-05	3.77E-01	WT

711	MFM-223	910948	breast	breast	DuctalCarcinoma	breast	carcinoma	5.04E-10	-5.20E-01	WT
712	DU-4475	906844	breast	breast	DuctalCarcinoma	breast	carcinoma	1.32E-01	-1.60E-01	WT
713	NCI-H226	905941	lung	lung: NSCLC: squamous_cell_carcinoma	Squamous	lung	carcinoma	8.65E-09	4.84E-01	WT
714	HCC2998	905971	colorectal	large_intestine	AdenoCarcinoma	large intestine	carcinoma	6.97E-18	-7.06E-01	WT
715	IGROV-1	905968	ovarian	ovary	Carcinoma	ovary	carcinoma	8.32E-03	2.46E-01	WT
716	SK-MEL-5	905956	melanoma	malignant_melanoma	Melanoma	skin	malignant melanoma	8.60E-06	3.84E-01	WT
717	Daudi	906831	H.L.M.	Burkitt lymphoma	BurkittsLymphoma	haematopoietic and lymphoid tissue	lymphoid neoplasm	6.80E-03	-2.51E-01	WT
718	SK-MEL-3	909724	melanoma	malignant_melanoma	Melanoma	skin	malignant melanoma	1.93E-06	4.07E-01	WT
719	COLO-679	906818	melanoma	malignant_melanoma	Melanoma	skin	malignant melanoma	7.75E-06	3.85E-01	WT
720	THP-1	909771	H.L.M.	AML	AML	haematopoietic and lymphoid tissue	haematopoietic neoplasm	3.50E-03	2.67E-01	WT
721	C-33-A	687505	uterus	cervix	Carcinoma	cervix	carcinoma	1.28E-08	4.79E-01	WT
722	NCI-H64	688026	lung	lung: small_cell_carcinoma	Carcinoma	lung	carcinoma	2.02E-01	1.42E-01	WT
723	KE-37	907277	H.L.M.	lymphoblastic Leukaemia	ALL	haematopoietic and lymphoid tissue	haematopoietic neoplasm	3.20E-01	1.20E-01	WT
724	KARPAS-422	907274	H.L.M.	B cell lymphoma	Lymphoma	haematopoietic and lymphoid tissue	lymphoid neoplasm	9.20E-02	-1.73E-01	WT
725	HCT-116	905936	colorectal	large_intestine	Carcinoma	large intestine	carcinoma	3.04E-01	-1.22E-01	WT
726	M14	905975	melanoma	malignant_melanoma	Melanoma	skin	malignant melanoma	1.29E-07	4.47E-01	WT
727	U031	905981	kidney	Renal cell carcinoma	Carcinoma	kidney	carcinoma	1.67E-05	3.72E-01	WT
728	NALM-1	908157	H.L.M.	CML	CML	haematopoietic and lymphoid tissue	haematopoietic neoplasm	1.03E-01	1.69E-01	WT
729	ARH-77	906765	H.L.M.	Myeloma	Myeloma	haematopoietic and lymphoid tissue	haematopoietic neoplasm	8.89E-03	2.44E-01	WT
730	Ca-Ski	906824	uterus	cervix	Squamous	cervix	carcinoma	1.32E-01	-1.60E-01	WT
731	TYK-nu	909774	ovarian	ovary	Carcinoma	ovary	carcinoma	6.78E-11	5.43E-01	WT
732	CAL-72	906827	Ewings/Osteosarcoma	osteosarcoma	Sarcoma	bone	osteosarcoma	1.23E-13	6.12E-01	WT
733	NB10	949171	CNS	neuroblastoma	Neuroblastoma	autonomic ganglia	neuroblastoma	6.21E-05	3.49E-01	WT
734	A704	910920	kidney	Renal cell carcinoma	Carcinoma	kidney	carcinoma	4.81E-02	1.96E-01	WT
735	NOMO-1	908451	H.L.M.	AML	AML	haematopoietic and lymphoid tissue	haematopoietic neoplasm	7.78E-02	-1.79E-01	WT
736	A549	905949	lung	lung: NSCLC: NOS	Carcinoma	lung	carcinoma	7.57E-04	3.01E-01	WT
737	DU-145	905935	prostate	prostate	AdenoCarcinoma	prostate	carcinoma	1.08E-01	1.68E-01	WT
738	MDA-MB-231	905960	breast	breast	Carcinoma	breast	carcinoma	5.37E-06	3.91E-01	WT
739	SF539	905984	CNS	glioma	Glioma	central nervous system	glioma	2.21E-18	7.16E-01	WT
740	647-V	906797	bladder	bladder	Carcinoma	urinary tract	carcinoma	2.89E-04	-3.21E-01	WT

741	ABC-1	906791	lung	lung: NSCLC: adenocarcinoma	AdenoCarcinoma	lung	carcinoma	2.83E-06	-4.01E-01	WT
742	HSC-3	907061	head & neck	upper_aerodigestive_tract	Squamous	upper aerodigestive tract	carcinoma	4.20E-01	-1.05E-01	WT
743	RCM-1	909263	colorectal	large_intestine	AdenoCarcinoma	large intestine	carcinoma	7.71E-12	-5.68E-01	WT
744	YH-13	909905	CNS	glioma	Glioma	central nervous system	glioma	1.66E-16	6.77E-01	WT
745	NCI-H524	908483	lung	lung: small_cell_carcinoma	Carcinoma	lung	carcinoma	9.37E-07	4.18E-01	WT
746	SU-DHL-1	909742	H.L.M.	lymphoid_neoplasm other	Lymphoma	haematopoietic and lymphoid tissue	lymphoid neoplasm	6.17E-04	3.05E-01	WT
747	786-0	905947	kidney	clear cell carcinoma	ClearCell	kidney	carcinoma	2.79E-11	5.54E-01	WT
748	HOP-62	905972	lung	lung: NSCLC: adenocarcinoma	AdenoCarcinoma	lung	carcinoma	6.22E-10	5.17E-01	WT
749	UACC-62	905976	melanoma	malignant_melanoma	Melanoma	skin	malignant melanoma	1.40E-10	5.35E-01	WT
750	UACC-257	905977	melanoma	malignant_melanoma	Melanoma	skin	malignant melanoma	7.49E-05	3.46E-01	WT
751	COLO-792	906814	melanoma	malignant_melanoma	Melanoma	skin	malignant melanoma	3.43E-04	3.17E-01	WT
752	A2058	906792	melanoma	malignant_melanoma	Melanoma	skin	malignant melanoma	2.49E-10	5.28E-01	WT
753	HD-MY-Z	907050	H.L.M.	Hodgkin lymphoma	HodgekinsLymphoma	haematopoietic and lymphoid tissue	lymphoid neoplasm	3.81E-19	7.31E-01	WT
754	WSU-NHL	909785	H.L.M.	lymphoid_neoplasm other	Lymphoma	haematopoietic and lymphoid tissue	lymphoid neoplasm	1.35E-01	1.59E-01	WT
755	TALL-1	909762	H.L.M.	lymphoblastic T cell leukaemia	ALL	haematopoietic and lymphoid tissue	lymphoid neoplasm	6.80E-03	2.51E-01	WT
756	SF126	909712	CNS	glioma	Glioma	central nervous system	glioma	3.24E-18	7.13E-01	WT
757	MDA-MB-435	905988	melanoma	malignant_melanoma	Carcinoma	breast	carcinoma	2.00E-08	4.73E-01	WT
758	HCT-15	905937	colorectal	large_intestine	Carcinoma	large intestine	carcinoma	1.45E-08	-4.77E-01	WT
759	MCF7	905946	breast	breast	Carcinoma	breast	carcinoma	7.36E-06	-3.86E-01	WT
760	OVCAR-3	905933	ovarian	ovary	AdenoCarcinoma	ovary	carcinoma	7.04E-03	-2.50E-01	WT
761	OVCAR-5	905969	ovarian	ovary	Carcinoma	ovary	carcinoma	1.54E-03	-2.86E-01	WT
762	COR-L23	687780	lung	lung: NSCLC: large cell	LargeCell	lung	carcinoma	4.81E-02	-1.96E-01	WT
763	MHH-ES-1	908134	Ewings/Osteosarcoma	Ewings sarcoma	EwingsSarcoma	bone	Ewings sarcoma-peripheral primitive neuroectodermal tumour	7.47E-07	4.22E-01	WT
764	CAL-85-1	910852	breast	breast	Carcinoma	breast	carcinoma	4.81E-04	-3.10E-01	WT
765	EoL-1-cell	906856	H.L.M.	haematopoietic_neoplasm other	CML	haematopoietic and lymphoid tissue	haematopoietic neoplasm	4.69E-02	1.96E-01	WT
766	ME-180	687514	uterus	cervix	Carcinoma	cervix	carcinoma	8.60E-06	-3.84E-01	WT
767	RD	909264	sacrcoma	rhabdomyosarcoma	Sarcoma	soft tissue	rhabdomyosarcoma	1.65E-18	7.19E-01	WT

768	COLO-205	905961	colorectal	large_intestine	AdenoCarcinoma	large intestine	carcinoma	1.16E-14	-6.36E-01	WT
769	HT-29	905939	colorectal	large_intestine	Carcinoma	large intestine	carcinoma	1.64E-17	-6.98E-01	WT
770	T47D	905945	breast	breast	DuctalCarcinoma	breast	carcinoma	5.14E-08	-4.60E-01	WT
771	PC-3	905934	prostate	prostate	AdenoCarcinoma	prostate	carcinoma	2.66E-01	1.29E-01	WT
772	COLO-800	906813	melanoma	malignant_melanoma	Melanoma	skin	malignant melanoma	1.74E-10	5.32E-01	WT
773	KOSC-2	753570	head & neck	upper_aerodigestive_tract	Squamous	upper aerodigestive tract	carcinoma	5.45E-04	-3.08E-01	WT
774	DK-MG	906839	CNS	glioma	Glioma	central nervous system	glioma	8.74E-17	6.83E-01	WT
775	SW962	909758	uterus	vulva	Squamous	vulva	carcinoma	4.99E-03	2.59E-01	WT
776	AGS	906790	gastric	stomach	AdenoCarcinoma	stomach	carcinoma	5.80E-10	-5.18E-01	WT
777	HEL	907053	H.L.M.	AML	AML	haematopoietic and lymphoid tissue	haematopoietic neoplasm	3.38E-02	2.07E-01	WT
778	C3A	910850	liver	liver	HCC	liver	carcinoma	5.21E-02	-1.93E-01	WT
779	CAKI-1	905963	kidney	clear cell carcinoma	ClearCell	kidney	carcinoma	7.75E-06	3.85E-01	WT
780	SF268	905986	CNS	glioma	Glioma	central nervous system	glioma	1.99E-16	6.75E-01	MUT_p.A289V/Missense
781	TK10	905980	kidney	Renal cell carcinoma	Carcinoma	kidney	carcinoma	2.62E-02	2.14E-01	WT
782	A2780	906804	ovarian	ovary	AdenoCarcinoma	ovary	carcinoma	2.62E-16	6.73E-01	WT
783	OVCAR-4	905990	ovarian	ovary	Carcinoma	ovary	carcinoma	2.86E-02	-2.12E-01	WT
784	A375	906793	melanoma	malignant_melanoma	Melanoma	skin	malignant melanoma	1.30E-10	5.36E-01	WT
785	KP-N-S19s	907315	CNS	neuroblastoma	Neuroblastoma	autonomic ganglia	neuroblastoma	1.57E-15	6.56E-01	WT
786	BC-3	910918	H.L.M.	B cell lymphoma	Lymphoma	haematopoietic and lymphoid tissue	lymphoid neoplasm	2.95E-01	-1.24E-01	WT
787	PANC-08-13	925347	pancreatic	pancreas	DuctalCarcinoma	pancreas	carcinoma	1.50E-12	-5.86E-01	WT
788	EKVX	905970	lung	lung: NSCLC: adenocarcinoma	AdenoCarcinoma	lung	carcinoma	1.01E-01	1.70E-01	WT
789	DMS-114	687983	lung	lung: small_cell_carcinoma	Carcinoma	lung	carcinoma	1.76E-08	4.74E-01	WT

Abbreviation: WT, wild type; MUT, Mutant; AML, Acute Myeloid Leukaemia; ALL, Acute Lymphoblastic Leukaemia; CML, Chronic Myeloid Leukaemia; CLL, Chronic Lymphoblastic Leukaemia; H. L. M., Haematopoietic & Lymphoid Malignancies

Table S4D. EMT score (generic cell line EMT signature) of SANGER/COSMIC + CCLE/Broad-Novartis cell line data.

Cell line primary name	Site Primary	Sanger.Generic.EMT.CL.pv	Sanger.Generic.EMT.CL.Ksscore	CCLE.Generic.EMT.CL.pv	CCLE.Generic.EMT.CL.Ksscore	Avg.Generic.EMT.CL.pv	Avg.Generic.EMT.CL.Ksscore
EGI-1	biliary_tract	8.65E-09	-4.84E-01	---	---	8.65E-09	-4.84E-01
ETK-1	biliary_tract	8.90E-04	-2.98E-01	---	---	8.90E-04	-2.98E-01
HuCCT1	biliary_tract	1.35E-01	-1.59E-01	2.85E-01	-1.26E-01	2.10E-01	-1.43E-01
HuH28	biliary_tract	9.00E-15	6.39E-01	9.00E-15	6.39E-01	9.00E-15	6.39E-01
SNU1079	biliary_tract	---	---	4.81E-02	1.96E-01	4.81E-02	1.96E-01
SNU245	biliary_tract	---	---	1.12E-20	-7.61E-01	1.12E-20	-7.61E-01
SNU308	biliary_tract	---	---	1.31E-06	-4.13E-01	1.31E-06	-4.13E-01
SNU478	biliary_tract	---	---	8.22E-05	-3.44E-01	8.22E-05	-3.44E-01
TGBC1TKB	biliary_tract	2.14E-05	-3.68E-01	---	---	2.14E-05	-3.68E-01
TGBC24TKB	biliary_tract	1.71E-13	-6.09E-01	---	---	1.71E-13	-6.09E-01
5637	bladder	3.78E-02	-2.03E-01	4.10E-02	2.01E-01	3.94E-02	-1.00E-03
639-V	bladder	1.65E-11	5.60E-01	---	---	1.65E-11	5.60E-01
647-V	bladder	2.82E-03	-2.72E-01	---	---	---	---
647-V	bladder	2.89E-04	-3.21E-01	3.41E-01	1.16E-01	8.67E-02	-1.87E-01
BC-3C	bladder	7.36E-06	3.86E-01	---	---	7.36E-06	3.86E-01
BFTC-905	bladder	2.68E-06	-4.02E-01	---	---	---	---
BFTC-905	bladder	3.53E-07	-4.33E-01	4.24E-04	-3.13E-01	1.07E-04	-3.88E-01
CAL-29	bladder	4.99E-03	-2.59E-01	---	---	4.99E-03	-2.59E-01
DSH1	bladder	1.86E-13	-6.08E-01	---	---	1.86E-13	-6.08E-01
Hs 172.T	bladder	7.75E-20	7.45E-01	---	---	7.75E-20	7.45E-01
HT-1197	bladder	4.82E-03	-2.59E-01	---	---	---	---
HT-1197	bladder	1.78E-04	-3.30E-01	6.08E-02	-1.88E-01	1.77E-02	-2.59E-01
HT-1376	bladder	1.56E-04	-3.32E-01	1.43E-03	-2.87E-01	7.93E-04	-3.10E-01
J82	bladder	7.66E-10	5.14E-01	5.35E-15	6.44E-01	3.83E-10	5.79E-01
JMSU-1	bladder	2.44E-15	6.51E-01	---	---	2.44E-15	6.51E-01
KMBC-2	bladder	3.49E-11	-5.51E-01	---	---	3.49E-11	-5.51E-01
KU-19-19	bladder	1.32E-01	1.60E-01	2.78E-02	2.13E-01	7.99E-02	1.87E-01
LB831-BLC	bladder	9.00E-15	6.39E-01	---	---	9.00E-15	6.39E-01
RT112	bladder	4.92E-14	-6.22E-01	3.08E-10	-5.26E-01	1.54E-10	-5.74E-01
RT112/84	bladder	5.04E-10	-5.20E-01	---	---	5.04E-10	-5.20E-01
RT4	bladder	4.38E-10	-5.21E-01	---	---	4.38E-10	-5.21E-01
SCABER	bladder	2.57E-01	1.31E-01	---	---	2.57E-01	1.31E-01
SW1710	bladder	3.76E-08	4.64E-01	6.67E-10	5.16E-01	1.91E-08	4.90E-01
SW780	bladder	2.17E-17	-6.96E-01	1.53E-09	-5.06E-01	7.65E-10	-6.01E-01
T24	bladder	4.24E-05	3.56E-01	1.01E-12	5.90E-01	2.12E-05	4.73E-01
TCCSUP	bladder	4.79E-09	4.91E-01	5.41E-10	5.19E-01	2.67E-09	5.05E-01
UMUC3	bladder	1.10E-15	6.59E-01	2.83E-19	7.34E-01	5.50E-16	6.97E-01
VMCUB1	bladder	5.45E-04	-3.08E-01	1.67E-03	-2.84E-01	1.11E-03	-2.96E-01
380	blood	2.86E-02	2.12E-01	---	---	2.86E-02	2.12E-01
697	blood	2.74E-05	3.64E-01	3.33E-05	3.61E-01	3.04E-05	3.63E-01
ALL-PO	blood	2.27E-02	2.19E-01	---	---	2.27E-02	2.19E-01
AML-193	blood	9.20E-02	1.73E-01	7.78E-02	1.79E-01	8.49E-02	1.76E-01
AMO-1	blood	---	---	5.92E-02	1.89E-01	5.92E-02	1.89E-01
ARH-77	blood	8.89E-03	2.44E-01	---	---	8.89E-03	2.44E-01
ATN-1	blood	3.04E-01	1.22E-01	---	---	3.04E-01	1.22E-01
BALL-1	blood	1.47E-01	1.56E-01	---	---	1.47E-01	1.56E-01
BC-1	blood	8.05E-03	2.47E-01	---	---	8.05E-03	2.47E-01
BC-3	blood	2.95E-01	-1.24E-01	---	---	2.95E-01	-1.24E-01
BCP-1	blood	---	---	2.47E-02	2.16E-01	2.47E-02	2.16E-01
BDCM	blood	---	---	2.40E-02	2.17E-01	2.40E-02	2.17E-01
BE-13	blood	7.24E-02	1.82E-01	---	---	7.24E-02	1.82E-01
BL-41	blood	1.08E-01	1.68E-01	---	---	---	---
BL-41	blood	1.32E-01	1.60E-01	---	---	1.20E-01	1.64E-01
BL-70	blood	1.53E-01	1.54E-01	1.31E-02	2.34E-01	8.31E-02	1.94E-01
BONNA-12	blood	7.78E-02	1.79E-01	---	---	7.78E-02	1.79E-01

BV-173	blood	1.05E-02	2.40E-01	2.09E-03	2.79E-01	6.30E-03	2.60E-01
C8166	blood	4.90E-05	3.54E-01	---	---	4.90E-05	3.54E-01
CA46	blood	1.18E-01	1.64E-01	7.78E-02	1.79E-01	9.79E-02	1.72E-01
CCRF-CEM	blood	2.78E-02	2.13E-01	---	---	2.78E-02	2.13E-01
CESS	blood	1.53E-01	1.54E-01	---	---	1.53E-01	1.54E-01
CI-1	blood	---	---	2.52E-03	2.75E-01	2.52E-03	2.75E-01
CMK	blood	8.05E-03	2.47E-01	2.62E-03	2.74E-01	5.34E-03	2.61E-01
CMK-11-5	blood	---	---	6.17E-04	3.05E-01	6.17E-04	3.05E-01
CML-T1	blood	3.14E-03	2.70E-01	1.94E-03	2.81E-01	2.54E-03	2.76E-01
CRO-AP2	blood	1.40E-01	1.57E-01	---	---	1.40E-01	1.57E-01
CRO-AP5	blood	1.98E-01	1.43E-01	---	---	1.98E-01	1.43E-01
CTB-1	blood	2.55E-02	2.15E-01	---	---	2.55E-02	2.15E-01
CTB-1	blood	1.18E-01	1.64E-01	---	---	1.18E-01	1.64E-01
CTV-1	blood	2.52E-03	2.75E-01	---	---	2.52E-03	2.75E-01
Daudi	blood	3.74E-01	1.11E-01	---	---	---	---
Daudi	blood	6.80E-03	-2.51E-01	1.23E-01	1.62E-01	2.19E-01	3.33E-02
DB	blood	1.91E-01	1.45E-01	1.53E-01	1.54E-01	1.72E-01	1.50E-01
DEL	blood	2.82E-03	2.72E-01	3.58E-04	3.16E-01	1.59E-03	2.94E-01
DG-75	blood	3.99E-02	2.02E-01	---	---	3.99E-02	2.02E-01
DOHH-2	blood	6.23E-02	1.87E-01	9.20E-02	1.73E-01	7.72E-02	1.80E-01
EB1	blood	---	---	9.26E-04	2.97E-01	9.26E-04	2.97E-01
EB2	blood	5.23E-04	3.09E-01	6.17E-04	3.05E-01	5.70E-04	3.07E-01
EB-3	blood	1.66E-01	1.51E-01	---	---	1.66E-01	1.51E-01
EHEB	blood	2.75E-01	-1.28E-01	7.42E-02	1.81E-01	1.75E-01	2.65E-02
EJM	blood	---	---	1.94E-01	1.44E-01	1.94E-01	1.44E-01
EM-2	blood	1.43E-01	1.56E-01	1.10E-01	1.67E-01	1.27E-01	1.62E-01
EoL-1-cell	blood	4.69E-02	1.96E-01	---	---	4.69E-02	1.96E-01
EoL-1-cell	blood	5.35E-02	1.92E-01	3.90E-03	2.64E-01	2.87E-02	2.28E-01
F-36P	blood	---	---	1.27E-03	2.90E-01	1.27E-03	2.90E-01
GA-10	blood	---	---	4.33E-02	1.99E-01	4.33E-02	1.99E-01
GA-10-Clone-20	blood	4.16E-12	-5.75E-01	---	---	4.16E-12	-5.75E-01
GA-10-Clone-4	blood	2.57E-01	1.31E-01	---	---	2.57E-01	1.31E-01
GDM-1	blood	1.27E-03	2.90E-01	1.30E-04	3.36E-01	7.00E-04	3.13E-01
GRANTA-519	blood	---	---	8.90E-04	2.98E-01	8.90E-04	2.98E-01
GR-ST	blood	2.01E-02	2.22E-01	---	---	2.01E-02	2.22E-01
H9	blood	5.94E-03	2.54E-01	---	---	5.94E-03	2.54E-01
HAL-01	blood	1.04E-03	2.94E-01	---	---	1.04E-03	2.94E-01
HC-1	blood	9.86E-02	1.71E-01	---	---	9.86E-02	1.71E-01
HDLM-2	blood	6.72E-02	1.85E-01	---	---	---	---
HDLM-2	blood	1.32E-01	1.60E-01	1.01E-02	2.41E-01	6.91E-02	1.93E-01
HD-MY-Z	blood	3.81E-19	7.31E-01	6.34E-20	7.47E-01	2.22E-19	7.39E-01
HEL	blood	5.35E-03	2.57E-01	---	---	---	---
HEL	blood	3.38E-02	2.07E-01	---	---	1.96E-02	2.32E-01
HEL 92.1.7	blood	---	---	2.04E-06	4.06E-01	2.04E-06	4.06E-01
HH	blood	8.90E-04	2.98E-01	4.42E-04	3.12E-01	6.66E-04	3.05E-01
HL-60	blood	1.21E-01	1.63E-01	6.39E-02	1.86E-01	9.25E-02	1.75E-01
HPB-ALL	blood	---	---	1.67E-05	3.72E-01	1.67E-05	3.72E-01
Hs 604.T	blood	---	---	2.59E-11	5.54E-01	2.59E-11	5.54E-01
Hs 611.T	blood	---	---	1.84E-05	3.71E-01	1.84E-05	3.71E-01
Hs 616.T	blood	---	---	7.75E-20	7.45E-01	7.75E-20	7.45E-01
Hs 751.T	blood	---	---	1.03E-21	7.81E-01	1.03E-21	7.81E-01
HT	blood	2.90E-01	1.25E-01	7.60E-02	1.80E-01	1.83E-01	1.53E-01
HuNS1	blood	4.69E-02	1.96E-01	4.10E-02	2.01E-01	4.40E-02	1.99E-01
HuT 102	blood	---	---	4.67E-05	3.55E-01	4.67E-05	3.55E-01
HuT 78	blood	---	---	1.36E-04	3.35E-01	1.36E-04	3.35E-01
IM-9	blood	2.27E-02	2.19E-01	---	---	2.27E-02	2.19E-01
JeKo-1	blood	---	---	8.89E-03	2.44E-01	8.89E-03	2.44E-01
JiyoyeP-2003	blood	2.75E-01	-1.28E-01	---	---	2.75E-01	-1.28E-01
JK-1	blood	---	---	9.49E-03	2.42E-01	9.49E-03	2.42E-01
JM1	blood	---	---	2.09E-03	2.79E-01	2.09E-03	2.79E-01

J-RT3-T3-5	blood	2.34E-03	2.76E-01	---	---	2.34E-03	2.76E-01
JURKAT	blood	---	---	5.68E-04	3.07E-01	5.68E-04	3.07E-01
JURL-MK1	blood	---	---	1.14E-07	4.49E-01	1.14E-07	4.49E-01
JVM-2	blood	6.39E-02	1.86E-01	6.39E-02	1.86E-01	6.39E-02	1.86E-01
JVM-3	blood	8.05E-03	2.47E-01	6.14E-03	2.53E-01	7.10E-03	2.50E-01
K052	blood	4.45E-02	1.98E-01	---	---	4.45E-02	1.98E-01
K-562	blood	---	---	5.02E-04	3.10E-01	5.02E-04	3.10E-01
KARPAS-299	blood	1.43E-03	2.87E-01	7.88E-04	3.00E-01	1.11E-03	2.94E-01
KARPAS-422	blood	3.20E-02	2.08E-01	---	---	---	---
KARPAS-422	blood	9.20E-02	-1.73E-01	---	---	6.20E-02	1.75E-02
KARPAS-45	blood	1.14E-04	3.38E-01	---	---	1.14E-04	3.38E-01
KARPAS-620	blood	---	---	3.68E-02	2.04E-01	3.68E-02	2.04E-01
KASUMI-1	blood	1.01E-02	2.41E-01	2.02E-03	2.80E-01	6.06E-03	2.61E-01
Kasumi-6	blood	---	---	1.89E-02	2.24E-01	1.89E-02	2.24E-01
KCL-22	blood	---	---	5.97E-06	3.89E-01	5.97E-06	3.89E-01
KE-37	blood	2.78E-02	2.13E-01	---	---	---	---
KE-37	blood	3.20E-01	1.20E-01	1.44E-02	2.31E-01	9.75E-02	1.94E-01
KG-1	blood	---	---	1.04E-03	2.94E-01	1.04E-03	2.94E-01
KHM-1B	blood	---	---	1.94E-01	1.44E-01	1.94E-01	1.44E-01
Ki-JK	blood	---	---	4.81E-04	3.10E-01	4.81E-04	3.10E-01
KM-H2	blood	7.15E-05	3.47E-01	---	---	7.15E-05	3.47E-01
KM-H2	blood	4.61E-04	3.11E-01	3.50E-05	3.60E-01	1.60E-04	3.41E-01
KMM-1	blood	---	---	1.63E-04	3.32E-01	1.63E-04	3.32E-01
KMOE-2	blood	1.15E-02	2.37E-01	---	---	1.15E-02	2.37E-01
KMS-11	blood	---	---	4.99E-03	2.59E-01	4.99E-03	2.59E-01
KMS-12-BM	blood	7.60E-02	1.80E-01	6.23E-02	1.87E-01	6.92E-02	1.84E-01
KMS-12-PE	blood	3.20E-02	2.08E-01	---	---	3.20E-02	2.08E-01
KMS-20	blood	---	---	1.39E-02	2.32E-01	1.39E-02	2.32E-01
KMS-21BM	blood	---	---	3.78E-02	2.03E-01	3.78E-02	2.03E-01
KMS-26	blood	---	---	1.54E-03	2.86E-01	1.54E-03	2.86E-01
KMS-27	blood	---	---	4.99E-03	2.59E-01	4.99E-03	2.59E-01
KMS-28BM	blood	---	---	2.43E-03	2.76E-01	2.43E-03	2.76E-01
KMS-34	blood	---	---	1.19E-02	2.36E-01	1.19E-02	2.36E-01
KO52	blood	---	---	1.05E-02	2.40E-01	1.05E-02	2.40E-01
KOPN-8	blood	---	---	1.27E-02	2.35E-01	1.27E-02	2.35E-01
KU812	blood	---	---	3.29E-02	2.07E-01	3.29E-02	2.07E-01
KY821	blood	1.73E-01	1.49E-01	---	---	1.73E-01	1.49E-01
KYO-1	blood	---	---	2.62E-03	2.74E-01	2.62E-03	2.74E-01
L-1236	blood	---	---	9.26E-04	2.97E-01	9.26E-04	2.97E-01
L-363	blood	4.33E-02	1.99E-01	2.53E-04	3.23E-01	2.18E-02	2.61E-01
L-428	blood	9.18E-03	2.43E-01	6.70E-04	3.04E-01	4.93E-03	2.74E-01
L-540	blood	9.80E-03	2.41E-01	1.73E-03	2.83E-01	5.77E-03	2.62E-01
LAMA-84	blood	1.08E-02	2.39E-01	2.43E-03	2.76E-01	6.62E-03	2.58E-01
LC4-1	blood	1.95E-02	2.23E-01	---	---	1.95E-02	2.23E-01
LOUCY	blood	4.34E-03	2.62E-01	2.53E-04	3.23E-01	2.30E-03	2.93E-01
LP-1	blood	1.39E-02	2.32E-01	4.06E-04	3.14E-01	7.15E-03	2.73E-01
M-07e	blood	---	---	1.13E-03	2.93E-01	1.13E-03	2.93E-01
MC-1010	blood	4.94E-02	1.95E-01	---	---	4.94E-02	1.95E-01
MC116	blood	---	---	5.35E-03	2.57E-01	5.35E-03	2.57E-01
ME-1	blood	---	---	1.09E-03	2.93E-01	1.09E-03	2.93E-01
MEC-2	blood	---	---	4.57E-02	1.97E-01	4.57E-02	1.97E-01
MEG-01	blood	5.66E-06	3.90E-01	6.63E-06	3.88E-01	6.15E-06	3.89E-01
MHH-CALL-2	blood	1.63E-02	2.28E-01	1.43E-03	2.87E-01	8.87E-03	2.58E-01
MHH-CALL-3	blood	---	---	1.18E-03	2.92E-01	1.18E-03	2.92E-01
MHH-CALL-4	blood	3.20E-02	2.08E-01	3.78E-02	2.03E-01	3.49E-02	2.06E-01
MHH-PREB-1	blood	3.02E-02	2.10E-01	---	---	3.02E-02	2.10E-01
Mino	blood	---	---	3.03E-03	2.70E-01	3.03E-03	2.70E-01
MJ	blood	1.30E-04	3.36E-01	1.60E-03	2.85E-01	8.65E-04	3.11E-01
ML-2	blood	3.20E-02	2.08E-01	---	---	3.20E-02	2.08E-01
MM1-S	blood	---	---	8.98E-02	1.74E-01	8.98E-02	1.74E-01

MN-60	blood	3.29E-02	2.07E-01	---	---	3.29E-02	2.07E-01
MOLM-13	blood	---	---	2.55E-02	2.15E-01	2.55E-02	2.15E-01
MOLM-16	blood	---	---	2.89E-04	3.21E-01	2.89E-04	3.21E-01
MOLM-6	blood	---	---	2.94E-02	2.11E-01	2.94E-02	2.11E-01
MOLP-2	blood	---	---	2.18E-01	-1.39E-01	2.18E-01	-1.39E-01
MOLP-8	blood	---	---	1.67E-03	2.84E-01	1.67E-03	2.84E-01
MOLT13	blood	1.54E-03	2.86E-01	8.90E-04	2.98E-01	1.22E-03	2.92E-01
MOLT16	blood	1.58E-02	2.29E-01	2.92E-03	2.71E-01	9.36E-03	2.50E-01
MOLT4	blood	8.90E-04	2.98E-01	4.24E-04	3.13E-01	6.57E-04	3.06E-01
MONOMAC6	blood	7.53E-03	2.48E-01	---	---	7.53E-03	2.48E-01
MUTZ1	blood	1.23E-01	1.62E-01	---	---	1.23E-01	1.62E-01
MUTZ5	blood	---	---	3.48E-02	2.06E-01	3.48E-02	2.06E-01
MV411	blood	---	---	1.63E-02	2.28E-01	1.63E-02	2.28E-01
NALM1	blood	4.94E-02	1.95E-01	---	---	---	---
NALM1	blood	1.03E-01	1.69E-01	1.48E-03	2.87E-01	5.08E-02	2.12E-01
NALM19	blood	---	---	1.26E-01	1.62E-01	1.26E-01	1.62E-01
NALM6	blood	9.64E-04	2.96E-01	5.97E-06	3.89E-01	4.85E-04	3.43E-01
NB4	blood	1.00E-03	2.95E-01	1.04E-03	2.94E-01	1.02E-03	2.95E-01
NCIH929	blood	---	---	4.94E-02	1.95E-01	4.94E-02	1.95E-01
NCO2	blood	---	---	1.00E-03	2.95E-01	1.00E-03	2.95E-01
NKM1	blood	3.50E-03	2.67E-01	---	---	3.50E-03	2.67E-01
NOMO1	blood	2.90E-01	1.25E-01	---	---	---	---
NOMO1	blood	7.78E-02	-1.79E-01	2.30E-01	1.36E-01	2.22E-01	5.18E-02
NUDHL1	blood	---	---	5.49E-02	1.91E-01	5.49E-02	1.91E-01
OCIAML2	blood	1.87E-03	2.81E-01	2.82E-03	2.72E-01	2.35E-03	2.77E-01
OCIAML3	blood	---	---	7.49E-05	3.46E-01	7.49E-05	3.46E-01
OCIAML5	blood	---	---	3.26E-03	2.69E-01	3.26E-03	2.69E-01
OCILY19	blood	---	---	3.73E-04	3.15E-01	3.73E-04	3.15E-01
OCILY3	blood	---	---	2.02E-03	2.80E-01	2.02E-03	2.80E-01
OCIM1	blood	---	---	4.04E-03	2.64E-01	4.04E-03	2.64E-01
OPM2	blood	5.35E-03	2.57E-01	2.78E-02	2.13E-01	1.66E-02	2.35E-01
P12ICHIKAWA	blood	1.58E-02	2.29E-01	6.36E-03	2.53E-01	1.11E-02	2.41E-01
P30OHK	blood	1.67E-05	3.72E-01	---	---	1.67E-05	3.72E-01
P31FUJ	blood	9.49E-03	2.42E-01	6.58E-03	2.52E-01	8.04E-03	2.47E-01
P3HR1	blood	---	---	1.08E-02	2.39E-01	1.08E-02	2.39E-01
PEER	blood	---	---	3.28E-04	3.18E-01	3.28E-04	3.18E-01
PF382	blood	3.28E-04	3.18E-01	5.68E-04	3.07E-01	4.48E-04	3.13E-01
PFEIFFER	blood	---	---	1.18E-01	1.64E-01	1.18E-01	1.64E-01
PL21	blood	---	---	4.69E-02	1.96E-01	4.69E-02	1.96E-01
QIMRWIL	blood	8.98E-02	1.74E-01	---	---	8.98E-02	1.74E-01
RAJI	blood	4.33E-02	1.99E-01	1.48E-02	2.30E-01	2.91E-02	2.15E-01
RAMOS2G64C10	blood	4.44E-01	1.01E-01	---	---	4.44E-01	1.01E-01
RCHACV	blood	---	---	5.37E-06	3.91E-01	5.37E-06	3.91E-01
REC1	blood	---	---	4.06E-04	3.14E-01	4.06E-04	3.14E-01
REH	blood	8.32E-03	2.46E-01	1.19E-04	3.38E-01	4.22E-03	2.92E-01
RI1	blood	---	---	2.66E-01	1.29E-01	2.66E-01	1.29E-01
RL	blood	3.57E-01	1.14E-01	1.43E-01	1.56E-01	2.50E-01	1.35E-01
RPMI6666	blood	1.63E-01	1.51E-01	3.78E-02	2.03E-01	1.00E-01	1.77E-01
RPMI8226	blood	7.06E-02	-1.83E-01	2.95E-01	1.24E-01	1.83E-01	-2.95E-02
RPMI8402	blood	3.63E-03	2.66E-01	1.94E-03	2.81E-01	2.79E-03	2.74E-01
RPMI8866	blood	8.57E-02	1.76E-01	---	---	8.57E-02	1.76E-01
RS411	blood	1.32E-01	1.60E-01	1.48E-02	2.30E-01	7.34E-02	1.95E-01
SCC3	blood	4.24E-04	3.13E-01	---	---	4.24E-04	3.13E-01
SEM	blood	---	---	4.24E-04	3.13E-01	4.24E-04	3.13E-01
SET2	blood	---	---	6.43E-04	3.04E-01	6.43E-04	3.04E-01
SIGM5	blood	---	---	1.39E-02	2.32E-01	1.39E-02	2.32E-01
SKM1	blood	5.37E-06	3.91E-01	5.14E-05	3.53E-01	2.84E-05	3.72E-01
SKMM2	blood	4.26E-01	1.04E-01	---	---	---	---
SKMM2	blood	3.68E-01	1.12E-01	1.50E-01	1.55E-01	3.43E-01	1.19E-01
SR	blood	7.57E-04	3.01E-01	---	---	7.57E-04	3.01E-01

SR786	blood	---	---	3.43E-04	3.17E-01	3.43E-04	3.17E-01
ST486	blood	3.68E-02	2.04E-01	7.24E-02	1.82E-01	5.46E-02	1.93E-01
SUDHL1	blood	5.65E-05	3.51E-01	---	---	---	---
SUDHL1	blood	6.17E-04	3.05E-01	2.54E-06	4.03E-01	1.83E-04	3.53E-01
SUDHL10	blood	---	---	1.83E-01	1.46E-01	1.83E-01	1.46E-01
SUDHL4	blood	---	---	3.88E-02	2.02E-01	3.88E-02	2.02E-01
SUDHL5	blood	---	---	6.23E-02	1.87E-01	6.23E-02	1.87E-01
SUDHL6	blood	---	---	3.38E-02	2.07E-01	3.38E-02	2.07E-01
SUDHL8	blood	---	---	1.60E-03	2.85E-01	1.60E-03	2.85E-01
SUPB15	blood	---	---	1.24E-04	3.37E-01	1.24E-04	3.37E-01
SUPB8	blood	5.35E-02	1.92E-01	---	---	5.35E-02	1.92E-01
SUPHD1	blood	---	---	3.90E-03	2.64E-01	3.90E-03	2.64E-01
SUPM2	blood	---	---	3.14E-03	2.70E-01	3.14E-03	2.70E-01
SUPT1	blood	1.49E-04	3.33E-01	5.37E-06	3.91E-01	7.72E-05	3.62E-01
SUPT11	blood	---	---	1.06E-05	3.80E-01	1.06E-05	3.80E-01
TALL1	blood	1.87E-03	2.81E-01	---	---	1.87E-03	2.81E-01
TF1	blood	---	---	2.13E-04	3.27E-01	2.13E-04	3.27E-01
THP1	blood	3.50E-03	2.67E-01	6.70E-04	3.04E-01	2.09E-03	2.86E-01
TO 175.T	blood	---	---	1.16E-19	7.41E-01	1.16E-19	7.41E-01
TOLEDO	blood	---	---	3.76E-03	2.65E-01	3.76E-03	2.65E-01
TUR	blood	1.23E-02	2.36E-01	---	---	1.23E-02	2.36E-01
U266	blood	3.29E-02	2.07E-01	---	---	3.29E-02	2.07E-01
U266B1	blood	---	---	2.62E-02	2.14E-01	2.62E-02	2.14E-01
U698M	blood	2.27E-02	2.19E-01	---	---	2.27E-02	2.19E-01
U937	blood	---	---	5.35E-03	2.57E-01	5.35E-03	2.57E-01
UT7	blood	---	---	2.01E-02	2.22E-01	2.01E-02	2.22E-01
WSUDLCL2	blood	---	---	2.07E-02	2.21E-01	2.07E-02	2.21E-01
WSUNHL	blood	6.89E-02	1.84E-01	---	---	6.89E-02	1.84E-01
A3/KAW	blood/stomach	3.58E-02	2.05E-01	6.17E-04	3.05E-01	1.82E-02	2.55E-01
143B	bone	---	---	4.63E-23	8.05E-01	4.63E-23	8.05E-01
A673	bone	6.22E-10	5.17E-01	6.78E-11	5.43E-01	3.45E-10	5.30E-01
CADO-ES1	bone	---	---	5.25E-12	5.72E-01	5.25E-12	5.72E-01
CAL-72	bone	8.12E-14	6.16E-01	---	---	---	---
CAL-72	bone	1.23E-13	6.12E-01	7.14E-23	8.02E-01	7.14E-14	6.62E-01
ES1	bone	4.38E-10	5.21E-01	---	---	4.38E-10	5.21E-01
ES3	bone	8.33E-12	5.67E-01	---	---	8.33E-12	5.67E-01
ES5	bone	9.76E-11	5.39E-01	---	---	9.76E-11	5.39E-01
ES6	bone	1.10E-12	5.89E-01	---	---	1.10E-12	5.89E-01
ES7	bone	2.79E-13	6.04E-01	---	---	2.79E-13	6.04E-01
ES8	bone	6.30E-11	5.44E-01	---	---	6.30E-11	5.44E-01
EW-1	bone	4.27E-08	4.63E-01	---	---	4.27E-08	4.63E-01
EW-11	bone	9.43E-10	5.12E-01	---	---	9.43E-10	5.12E-01
EW-12	bone	8.37E-07	4.20E-01	---	---	8.37E-07	4.20E-01
EW-13	bone	1.10E-12	5.89E-01	---	---	1.10E-12	5.89E-01
EW-16	bone	2.20E-07	4.40E-01	---	---	2.20E-07	4.40E-01
EW-18	bone	8.65E-09	4.84E-01	---	---	8.65E-09	4.84E-01
EW-22	bone	8.10E-09	4.85E-01	---	---	8.10E-09	4.85E-01
EW-24	bone	1.13E-13	6.13E-01	---	---	1.13E-13	6.13E-01
EW-3	bone	8.10E-09	4.85E-01	---	---	---	---
EW-3	bone	1.96E-07	4.41E-01	---	---	1.02E-07	4.63E-01
EW-7	bone	1.40E-10	5.35E-01	---	---	1.40E-10	5.35E-01
G-292, clone A141B1	bone	---	---	3.11E-25	8.44E-01	3.11E-25	8.44E-01
H-EMC-SS	bone	1.30E-10	5.36E-01	---	---	1.30E-10	5.36E-01
HOS	bone	8.63E-13	5.92E-01	3.93E-18	7.11E-01	4.32E-13	6.52E-01
Hs 706.T	bone	---	---	2.15E-21	7.75E-01	2.15E-21	7.75E-01
Hs 737.T	bone	---	---	1.21E-24	8.33E-01	1.21E-24	8.33E-01
Hs 819.T	bone	---	---	2.15E-21	7.75E-01	2.15E-21	7.75E-01
Hs 821.T	bone	---	---	4.45E-21	7.69E-01	4.45E-21	7.69E-01
Hs 822.T	bone	---	---	4.91E-22	7.87E-01	4.91E-22	7.87E-01
Hs 863.T	bone	---	---	9.27E-22	7.81E-01	9.27E-22	7.81E-01

Hs 870.T	bone	---	---	1.73E-23	8.13E-01	1.73E-23	8.13E-01
Hs 888.T	bone	---	---	1.03E-21	7.81E-01	1.03E-21	7.81E-01
HuO-3N1	bone	2.43E-18	7.15E-01	---	---	2.43E-18	7.15E-01
HuO9	bone	1.28E-12	5.88E-01	---	---	1.28E-12	5.88E-01
MG-63	bone	6.33E-14	6.19E-01	7.27E-17	6.85E-01	3.17E-14	6.52E-01
MHH-ES-1	bone	7.59E-09	4.86E-01	---	---	---	---
MHH-ES-1	bone	7.47E-07	4.22E-01	3.85E-12	5.76E-01	1.91E-07	4.93E-01
NOS1	bone	3.05E-12	5.78E-01	---	---	3.05E-12	5.78E-01
NY	bone	4.05E-11	5.49E-01	---	---	4.05E-11	5.49E-01
OUMS27	bone	1.25E-20	7.60E-01	---	---	1.25E-20	7.60E-01
RDES	bone	---	---	3.55E-10	5.24E-01	3.55E-10	5.24E-01
SAOS2	bone	---	---	5.65E-19	7.28E-01	5.65E-19	7.28E-01
SJSA1	bone	5.33E-13	5.97E-01	5.65E-19	7.28E-01	2.67E-13	6.63E-01
SKES1	bone	---	---	3.30E-12	5.77E-01	3.30E-12	5.77E-01
SKNMC	bone	---	---	3.31E-10	5.25E-01	3.31E-10	5.25E-01
SW 1353	bone	---	---	2.95E-18	7.13E-01	2.95E-18	7.13E-01
TC71	bone	1.51E-10	5.34E-01	---	---	1.51E-10	5.34E-01
TI73	bone	1.44E-15	6.56E-01	9.16E-21	7.63E-01	7.20E-16	7.10E-01
U2OS	bone	1.42E-11	5.61E-01	1.92E-11	5.58E-01	1.67E-11	5.60E-01
AU565	breast	---	---	3.28E-13	-6.02E-01	3.28E-13	-6.02E-01
BT-20	breast	5.04E-10	-5.20E-01	5.10E-06	-3.92E-01	2.55E-06	-4.56E-01
BT-474	breast	4.69E-11	-5.48E-01	---	---	4.69E-11	-5.48E-01
BT-483	breast	1.13E-10	-5.37E-01	---	---	1.13E-10	-5.37E-01
BT-549	breast	1.10E-15	6.59E-01	9.27E-18	7.03E-01	5.55E-16	6.81E-01
CAL-120	breast	2.63E-07	4.37E-01	1.13E-10	5.37E-01	1.32E-07	4.87E-01
CAL-148	breast	8.85E-07	-4.19E-01	---	---	8.85E-07	-4.19E-01
CAL-51	breast	8.98E-02	1.74E-01	1.36E-05	3.76E-01	4.49E-02	2.75E-01
CAL-85-1	breast	3.03E-03	-2.70E-01	---	---	---	---
CAL-85-1	breast	4.81E-04	-3.10E-01	5.94E-03	-2.54E-01	3.12E-03	-2.76E-01
CAMA-1	breast	1.74E-07	-4.43E-01	1.73E-06	-4.09E-01	9.52E-07	-4.26E-01
COLO-824	breast	1.59E-01	-1.52E-01	---	---	1.59E-01	-1.52E-01
DU-4475	breast	1.32E-01	-1.60E-01	---	---	---	---
DU-4475	breast	2.02E-01	-1.42E-01	3.36E-01	-1.17E-01	2.01E-01	-1.45E-01
EFM-19	breast	8.37E-07	-4.20E-01	6.21E-05	-3.49E-01	3.15E-05	-3.85E-01
EFM-192A	breast	1.36E-08	-4.78E-01	---	---	1.36E-08	-4.78E-01
EVSA-T	breast	6.78E-11	-5.43E-01	3.76E-11	-5.50E-01	5.27E-11	-5.47E-01
HCC1143	breast	9.86E-02	-1.71E-01	7.24E-01	6.38E-02	4.11E-01	-5.36E-02
HCC1187	breast	3.36E-01	-1.17E-01	3.15E-01	-1.21E-01	3.26E-01	-1.19E-01
HCC1395	breast	4.53E-14	6.22E-01	7.71E-12	5.68E-01	3.88E-12	5.95E-01
HCC1419	breast	1.53E-11	-5.60E-01	1.19E-12	-5.88E-01	8.25E-12	-5.74E-01
HCC1500	breast	4.58E-06	-3.94E-01	---	---	4.58E-06	-3.94E-01
HCC1599	breast	1.04E-04	-3.40E-01	1.78E-02	-2.25E-01	8.95E-03	-2.83E-01
HCC1806	breast	9.45E-05	-3.42E-01	8.55E-04	-2.98E-01	4.75E-04	-3.20E-01
HCC1937	breast	4.83E-08	-4.61E-01	1.67E-05	-3.72E-01	8.37E-06	-4.17E-01
HCC1954	breast	1.30E-10	-5.36E-01	1.31E-06	-4.13E-01	6.55E-07	-4.75E-01
HCC202	breast	4.05E-11	-5.49E-01	---	---	4.05E-11	-5.49E-01
HCC2157	breast	2.65E-04	-3.22E-01	1.80E-03	-2.82E-01	1.03E-03	-3.02E-01
HCC2218	breast	5.35E-14	-6.21E-01	1.19E-12	-5.88E-01	6.22E-13	-6.05E-01
HCC38	breast	2.22E-01	1.38E-01	2.18E-01	1.39E-01	2.20E-01	1.39E-01
HCC70	breast	2.58E-08	-4.69E-01	1.37E-07	-4.46E-01	8.14E-08	-4.58E-01
HDQ-P1	breast	---	---	1.23E-01	-1.62E-01	1.23E-01	-1.62E-01
Hs 274.T	breast	---	---	1.65E-18	7.19E-01	1.65E-18	7.19E-01
Hs 281.T	breast	---	---	8.03E-24	8.19E-01	8.03E-24	8.19E-01
Hs 343.T	breast	---	---	8.03E-24	8.19E-01	8.03E-24	8.19E-01
Hs 578T	breast	1.26E-16	6.79E-01	1.41E-19	7.40E-01	6.31E-17	7.10E-01
Hs 606.T	breast	---	---	2.41E-23	8.10E-01	2.41E-23	8.10E-01
Hs 739.T	breast	---	---	1.57E-21	7.77E-01	1.57E-21	7.77E-01
JIMT-1	breast	2.43E-01	1.34E-01	---	---	2.43E-01	1.34E-01
MCF7	breast	7.36E-06	-3.86E-01	2.63E-07	-4.37E-01	3.81E-06	-4.12E-01
MDA-MB-134-VI	breast	4.24E-05	-3.56E-01	4.61E-04	-3.11E-01	2.52E-04	-3.34E-01

MDA-MB-157	breast	7.05E-16	6.63E-01	5.65E-19	7.28E-01	3.53E-16	6.96E-01
MDA-MB-175-VII	breast	2.30E-14	-6.29E-01	2.21E-18	-7.16E-01	1.15E-14	-6.73E-01
MDA-MB-231	breast	5.37E-06	3.91E-01	6.67E-10	5.16E-01	2.69E-06	4.54E-01
MDA-MB-361	breast	1.63E-12	-5.85E-01	1.23E-02	-2.36E-01	6.15E-03	-4.11E-01
MDA-MB-415	breast	5.33E-13	-5.97E-01	1.45E-07	-4.46E-01	7.25E-08	-5.22E-01
MDA-MB-436	breast	3.17E-15	6.49E-01	---	---	3.17E-15	6.49E-01
MDA-MB-453	breast	6.30E-11	-5.44E-01	2.41E-12	-5.81E-01	3.27E-11	-5.63E-01
MDA-MB-468	breast	1.39E-06	-4.12E-01	3.97E-07	-4.31E-01	8.94E-07	-4.22E-01
MFM-223	breast	5.04E-10	-5.20E-01	---	---	---	---
MFM-223	breast	5.00E-07	-4.28E-01	---	---	2.50E-07	-4.74E-01
MRKNU1	breast	7.35E-13	-5.94E-01	---	---	7.35E-13	-5.94E-01
OCUBM	breast	4.19E-09	-4.93E-01	---	---	4.19E-09	-4.93E-01
SKBR3	breast	6.13E-12	-5.71E-01	---	---	6.13E-12	-5.71E-01
T47D	breast	5.14E-08	-4.60E-01	1.17E-05	-3.78E-01	5.88E-06	-4.19E-01
UACC812	breast	6.79E-13	-5.94E-01	1.87E-09	-5.03E-01	9.35E-10	-5.49E-01
YMB1	breast	4.16E-12	-5.75E-01	---	---	4.16E-12	-5.75E-01
ZR751	breast	2.07E-11	-5.57E-01	---	---	2.07E-11	-5.57E-01
ZR7530	breast	5.84E-09	-4.89E-01	9.23E-09	-4.83E-01	7.54E-09	-4.86E-01
42-MG-BA	CNS	---	---	2.33E-22	7.93E-01	2.33E-22	7.93E-01
8-MG-BA	CNS	2.79E-13	6.04E-01	5.90E-16	6.65E-01	1.40E-13	6.35E-01
A172	CNS	1.23E-13	6.12E-01	1.12E-17	7.02E-01	6.15E-14	6.57E-01
ACN	CNS	2.23E-12	5.82E-01	---	---	2.23E-12	5.82E-01
AM-38	CNS	2.16E-10	5.30E-01	1.02E-17	7.02E-01	1.08E-10	6.16E-01
B2-17	CNS	6.04E-17	6.86E-01	---	---	6.04E-17	6.86E-01
Becker	CNS	8.33E-12	5.67E-01	---	---	---	---
Becker	CNS	2.07E-11	5.57E-01	1.82E-16	6.76E-01	9.37E-12	5.92E-01
CAS-1	CNS	1.35E-17	7.00E-01	6.72E-21	7.65E-01	6.75E-18	7.33E-01
CCF-STTG1	CNS	3.13E-19	7.33E-01	2.33E-22	7.93E-01	1.57E-19	7.63E-01
CHP-126	CNS	---	---	5.80E-10	5.18E-01	5.80E-10	5.18E-01
CHP-134	CNS	3.74E-07	4.32E-01	---	---	3.74E-07	4.32E-01
CHP-212	CNS	2.37E-13	6.05E-01	2.87E-16	6.72E-01	1.19E-13	6.39E-01
D-245MG	CNS	7.71E-16	6.62E-01	---	---	7.71E-16	6.62E-01
D-247MG	CNS	5.65E-19	7.28E-01	---	---	5.65E-19	7.28E-01
D-263MG	CNS	9.47E-20	7.43E-01	---	---	9.47E-20	7.43E-01
D-283MED	CNS	5.04E-10	5.20E-01	3.21E-09	4.97E-01	1.86E-09	5.09E-01
D-336MG	CNS	9.80E-15	6.38E-01	---	---	9.80E-15	6.38E-01
D341 Med	CNS	---	---	4.20E-07	4.30E-01	4.20E-07	4.30E-01
D-384MED	CNS	6.58E-08	4.57E-01	---	---	6.58E-08	4.57E-01
D-392MG	CNS	8.44E-18	7.04E-01	---	---	8.44E-18	7.04E-01
D-392MG	CNS	6.04E-17	6.86E-01	---	---	6.04E-17	6.86E-01
D-397MG	CNS	7.05E-16	6.63E-01	---	---	7.05E-16	6.63E-01
D-423MG	CNS	2.21E-18	7.16E-01	---	---	2.21E-18	7.16E-01
D-458MED	CNS	6.29E-07	4.24E-01	---	---	6.29E-07	4.24E-01
D-502MG	CNS	9.80E-15	6.38E-01	---	---	9.80E-15	6.38E-01
D-538MG	CNS	3.21E-22	7.90E-01	---	---	3.21E-22	7.90E-01
D-542MG	CNS	5.18E-20	7.48E-01	---	---	5.18E-20	7.48E-01
D-556MED	CNS	1.55E-06	4.11E-01	---	---	1.55E-06	4.11E-01
D-566MG	CNS	5.46E-22	7.86E-01	---	---	5.46E-22	7.86E-01
Daoy	CNS	1.92E-11	5.58E-01	5.51E-17	6.87E-01	9.60E-12	6.23E-01
DBTRG-05MG	CNS	1.76E-12	5.84E-01	6.04E-17	6.86E-01	8.80E-13	6.35E-01
DK-MG	CNS	8.74E-17	6.83E-01	7.96E-23	8.01E-01	4.37E-17	7.42E-01
GAMG	CNS	2.79E-13	6.04E-01	9.47E-20	7.43E-01	1.40E-13	6.74E-01
GI-1	CNS	2.23E-15	6.52E-01	1.41E-21	7.78E-01	1.12E-15	7.15E-01
GI-ME-N	CNS	2.95E-18	7.13E-01	---	---	2.95E-18	7.13E-01
GMS-10	CNS	2.21E-18	7.16E-01	1.12E-17	7.02E-01	6.71E-18	7.09E-01
GOS-3	CNS	---	---	1.49E-17	6.99E-01	1.49E-17	6.99E-01
GOTO	CNS	4.19E-13	5.99E-01	---	---	4.19E-13	5.99E-01
GOTO-P3	CNS	2.01E-13	6.07E-01	---	---	2.01E-13	6.07E-01
H4	CNS	1.79E-14	6.32E-01	3.93E-18	7.11E-01	8.95E-15	6.72E-01
Hs 683	CNS	---	---	1.03E-21	7.81E-01	1.03E-21	7.81E-01

IMR-32	CNS	---	---	7.35E-13	5.94E-01	7.35E-13	5.94E-01
IMR-5	CNS	3.05E-12	5.78E-01	---	---	3.05E-12	5.78E-01
KALS-1	CNS	1.38E-14	6.34E-01	1.88E-20	7.57E-01	6.90E-15	6.96E-01
KELLY	CNS	---	---	4.38E-10	5.21E-01	4.38E-10	5.21E-01
KG-1-C	CNS	---	---	6.23E-19	7.27E-01	6.23E-19	7.27E-01
KINGS-1	CNS	4.17E-17	6.90E-01	---	---	4.17E-17	6.90E-01
KNS-42	CNS	3.05E-12	5.78E-01	1.07E-14	6.37E-01	1.53E-12	6.08E-01
KNS-60	CNS	---	---	4.64E-19	7.30E-01	4.64E-19	7.30E-01
KNS-81	CNS	---	---	2.10E-19	7.36E-01	2.10E-19	7.36E-01
KNS-81-FD	CNS	2.43E-18	7.15E-01	---	---	2.43E-18	7.15E-01
KP-N-RT-BM-1	CNS	9.85E-09	4.82E-01	---	---	9.85E-09	4.82E-01
KP-N-S19s	CNS	1.57E-15	6.56E-01	3.46E-20	7.52E-01	7.85E-16	7.04E-01
KP-N-YN	CNS	3.67E-09	4.95E-01	5.05E-11	5.47E-01	1.86E-09	5.21E-01
KP-N-YS	CNS	5.84E-15	6.43E-01	---	---	5.84E-15	6.43E-01
KS-1	CNS	2.97E-14	6.27E-01	1.90E-19	7.37E-01	1.49E-14	6.82E-01
LAN-1	CNS	6.19E-08	4.57E-01	---	---	6.19E-08	4.57E-01
LAN-5	CNS	9.08E-11	5.40E-01	---	---	9.08E-11	5.40E-01
LAN-6	CNS	5.41E-10	5.19E-01	---	---	5.41E-10	5.19E-01
LN-18	CNS	---	---	1.01E-12	5.90E-01	1.01E-12	5.90E-01
LN-229	CNS	---	---	6.63E-17	6.85E-01	6.63E-17	6.85E-01
LN-405	CNS	2.23E-12	5.82E-01	---	---	2.23E-12	5.82E-01
M059J	CNS	6.04E-17	6.86E-01	---	---	6.04E-17	6.86E-01
M059K	CNS	---	---	6.07E-22	7.85E-01	6.07E-22	7.85E-01
MC-IXC	CNS	2.81E-09	4.98E-01	---	---	2.81E-09	4.98E-01
MHH-NB-11	CNS	3.85E-12	5.76E-01	2.61E-12	5.80E-01	3.23E-12	5.78E-01
MOG-G-CCM	CNS	9.35E-13	5.91E-01	---	---	9.35E-13	5.91E-01
MOG-G-UVW	CNS	7.05E-16	6.63E-01	---	---	7.05E-16	6.63E-01
NB1	CNS	2.79E-11	5.54E-01	6.27E-13	5.95E-01	1.43E-11	5.75E-01
NB10	CNS	2.04E-04	3.27E-01	---	---	2.04E-04	3.27E-01
NB12	CNS	4.86E-12	5.73E-01	---	---	4.86E-12	5.73E-01
NB13	CNS	2.83E-06	4.01E-01	---	---	2.83E-06	4.01E-01
NB14	CNS	6.22E-10	5.17E-01	---	---	6.22E-10	5.17E-01
NB16	CNS	4.01E-08	4.63E-01	---	---	4.01E-08	4.63E-01
NB17	CNS	1.01E-09	5.11E-01	---	---	1.01E-09	5.11E-01
NB19	CNS	1.28E-08	4.79E-01	---	---	1.28E-08	4.79E-01
NB5	CNS	4.93E-16	6.67E-01	---	---	4.93E-16	6.67E-01
NB6	CNS	1.47E-06	4.12E-01	---	---	1.47E-06	4.12E-01
NB69	CNS	7.29E-11	5.43E-01	---	---	7.29E-11	5.43E-01
NB7	CNS	2.96E-07	4.35E-01	---	---	2.96E-07	4.35E-01
NBSUSSR	CNS	1.08E-09	5.10E-01	---	---	1.08E-09	5.10E-01
NH12	CNS	3.76E-08	4.64E-01	---	---	3.76E-08	4.64E-01
NH6	CNS	3.85E-12	5.76E-01	3.13E-19	7.33E-01	1.93E-12	6.55E-01
NMCG1	CNS	1.78E-11	5.59E-01	5.76E-18	7.07E-01	8.90E-12	6.33E-01
NO10	CNS	4.50E-15	6.45E-01	---	---	4.50E-15	6.45E-01
NO11	CNS	1.64E-17	6.98E-01	---	---	1.64E-17	6.98E-01
ONS76	CNS	4.35E-06	3.95E-01	8.65E-09	4.84E-01	2.18E-06	4.40E-01
PFSK1	CNS	1.05E-11	5.65E-01	---	---	1.05E-11	5.65E-01
SCCH26	CNS	1.55E-08	4.76E-01	---	---	1.55E-08	4.76E-01
SF126	CNS	1.65E-18	7.19E-01	---	---	---	---
SF126	CNS	3.24E-18	7.13E-01	2.15E-21	7.75E-01	1.64E-18	7.32E-01
SF268	CNS	1.99E-16	6.75E-01	---	---	1.99E-16	6.75E-01
SF295	CNS	1.15E-16	6.80E-01	8.44E-18	7.04E-01	6.17E-17	6.92E-01
SF539	CNS	2.21E-18	7.16E-01	---	---	2.21E-18	7.16E-01
SIMA	CNS	3.12E-08	4.67E-01	1.92E-11	5.58E-01	1.56E-08	5.13E-01
SKMG1	CNS	6.04E-17	6.86E-01	---	---	6.04E-17	6.86E-01
SKNAS	CNS	2.82E-12	5.79E-01	2.21E-18	7.16E-01	1.41E-12	6.48E-01
SKNBE(2)	CNS	---	---	1.28E-12	5.88E-01	1.28E-12	5.88E-01
SKNDZ	CNS	1.42E-09	5.07E-01	1.40E-10	5.35E-01	7.80E-10	5.21E-01
SKNFI	CNS	9.76E-11	5.39E-01	---	---	---	---
SKNFI	CNS	7.15E-10	5.15E-01	7.85E-11	5.42E-01	2.47E-10	5.34E-01

SKNSH	CNS	---	---	1.88E-20	7.57E-01	1.88E-20	7.57E-01
SNB19	CNS	6.79E-13	5.94E-01	1.64E-17	6.98E-01	3.40E-13	6.46E-01
SNU1105	CNS	---	---	5.75E-23	8.04E-01	5.75E-23	8.04E-01
SNU201	CNS	---	---	1.02E-20	7.62E-01	1.02E-20	7.62E-01
SNU466	CNS	---	---	5.35E-14	6.21E-01	5.35E-14	6.21E-01
SNU626	CNS	---	---	3.14E-16	6.71E-01	3.14E-16	6.71E-01
SNU738	CNS	---	---	7.14E-12	5.69E-01	7.14E-12	5.69E-01
SW1088	CNS	4.33E-18	7.10E-01	2.55E-20	7.54E-01	2.18E-18	7.32E-01
SW1783	CNS	4.92E-14	6.22E-01	1.28E-19	7.41E-01	2.46E-14	6.82E-01
T98G	CNS	7.97E-13	5.93E-01	6.94E-15	6.41E-01	4.02E-13	6.17E-01
TGW	CNS	1.96E-07	4.41E-01	---	---	1.96E-07	4.41E-01
U118MG	CNS	1.82E-18	7.18E-01	---	---	1.82E-18	7.18E-01
U251	CNS	3.83E-14	6.24E-01	---	---	3.83E-14	6.24E-01
U251 MG	CNS	---	---	1.16E-19	7.41E-01	1.16E-19	7.41E-01
U87MG	CNS	2.17E-17	6.96E-01	6.06E-21	7.66E-01	1.09E-17	7.31E-01
YH13	CNS	1.66E-16	6.77E-01	7.14E-23	8.02E-01	8.30E-17	7.40E-01
YKG1	CNS	1.27E-14	6.35E-01	1.90E-19	7.37E-01	6.35E-15	6.86E-01
WERIRB1	Eye	1.09E-03	2.93E-01	---	---	1.09E-03	2.93E-01
ITO-II	germ_cell_tumour	1.23E-02	2.36E-01	---	---	1.23E-02	2.36E-01
NCCIT	germ_cell_tumour	7.59E-09	4.86E-01	---	---	7.59E-09	4.86E-01
NEC8	germ_cell_tumour	2.01E-02	2.22E-01	---	---	2.01E-02	2.22E-01
NTERASCLD1	germ_cell_tumour	3.08E-10	5.26E-01	---	---	3.08E-10	5.26E-01
ECC4	GI tract	6.14E-03	-2.53E-01	---	---	6.14E-03	-2.53E-01
HUTU-80	GI tract	5.85E-11	5.45E-01	2.12E-14	6.30E-01	2.93E-11	5.88E-01
769-P	kidney	7.57E-04	3.01E-01	2.74E-05	3.64E-01	3.92E-04	3.33E-01
786-0	kidney	2.79E-11	5.54E-01	5.25E-12	5.72E-01	1.66E-11	5.63E-01
A498	kidney	2.04E-06	4.06E-01	3.43E-09	4.96E-01	1.02E-06	4.51E-01
A704	kidney	7.53E-03	2.48E-01	---	---	---	---
A704	kidney	4.81E-02	1.96E-01	4.24E-05	3.56E-01	1.58E-02	2.62E-01
ACHN	kidney	1.19E-04	3.38E-01	3.43E-09	4.96E-01	5.95E-05	4.17E-01
BB65-RCC	kidney	9.26E-04	2.97E-01	---	---	9.26E-04	2.97E-01
BFTC-909	kidney	1.63E-12	5.85E-01	1.41E-19	7.40E-01	8.15E-13	6.63E-01
CAKI-1	kidney	7.75E-06	3.85E-01	8.33E-12	5.67E-01	3.88E-06	4.76E-01
Caki-2	kidney	---	---	4.12E-06	3.95E-01	4.12E-06	3.95E-01
CAL-54	kidney	3.28E-04	3.18E-01	3.85E-05	3.58E-01	1.83E-04	3.38E-01
G-401	kidney	1.64E-07	4.44E-01	9.76E-11	5.39E-01	8.20E-08	4.92E-01
HA7-RCC	kidney	3.28E-04	3.18E-01	---	---	3.28E-04	3.18E-01
KMRC-1	kidney	---	---	2.91E-15	6.50E-01	2.91E-15	6.50E-01
KMRC-2	kidney	---	---	1.30E-10	5.36E-01	1.30E-10	5.36E-01
KMRC-20	kidney	---	---	7.75E-06	3.85E-01	7.75E-06	3.85E-01
KMRC-3	kidney	---	---	1.54E-07	4.45E-01	1.54E-07	4.45E-01
LB1047-RCC	kidney	2.75E-08	4.69E-01	---	---	---	---
LB1047-RCC	kidney	1.24E-06	4.14E-01	---	---	6.34E-07	4.42E-01
LB2241-RCC	kidney	3.51E-06	3.98E-01	---	---	3.51E-06	3.98E-01
LB996-RCC	kidney	3.14E-03	2.70E-01	---	---	3.14E-03	2.70E-01
OSRC2	kidney	9.06E-06	3.83E-01	---	---	9.06E-06	3.83E-01
RCC10RGB	kidney	1.70E-04	3.31E-01	---	---	1.70E-04	3.31E-01
RXF393	kidney	1.57E-13	6.10E-01	---	---	1.57E-13	6.10E-01
SKNEP1	kidney	2.62E-09	4.99E-01	---	---	2.62E-09	4.99E-01
SN12C	kidney	2.57E-13	6.05E-01	---	---	2.57E-13	6.05E-01
TK10	kidney	2.62E-02	2.14E-01	---	---	2.62E-02	2.14E-01
U031	kidney	1.67E-05	3.72E-01	---	---	1.67E-05	3.72E-01
VMRCRCW	kidney	---	---	6.29E-07	4.24E-01	6.29E-07	4.24E-01
VMRCRCZ	kidney	7.90E-07	4.21E-01	3.67E-09	4.95E-01	3.97E-07	4.58E-01
C2BBe1	large_intestine	1.20E-08	-4.80E-01	3.26E-03	-2.69E-01	1.63E-03	-3.75E-01
CaR-1	large_intestine	1.63E-04	-3.32E-01	---	---	1.63E-04	-3.32E-01
CCK-81	large_intestine	---	---	9.35E-13	-5.91E-01	9.35E-13	-5.91E-01
CL-11	large_intestine	---	---	4.38E-01	-1.02E-01	4.38E-01	-1.02E-01
CL-34	large_intestine	---	---	1.99E-16	-6.75E-01	1.99E-16	-6.75E-01
COLO-205	large_intestine	1.16E-14	-6.36E-01	1.71E-13	-6.09E-01	9.13E-14	-6.23E-01

COLO-320	large_intestine	---	---	1.88E-08	4.74E-01	1.88E-08	4.74E-01
COLO-320-HSR	large_intestine	9.53E-06	3.82E-01	---	---	9.53E-06	3.82E-01
COLO-678	large_intestine	8.33E-12	-5.67E-01	2.42E-08	-4.70E-01	1.21E-08	-5.19E-01
COLO-741	large_intestine	7.00E-08	4.56E-01	---	---	7.00E-08	4.56E-01
CW-2	large_intestine	9.22E-16	-6.61E-01	---	---	9.22E-16	-6.61E-01
DLD-1	large_intestine	---	---	2.83E-06	-4.01E-01	2.83E-06	-4.01E-01
Gp2D	large_intestine	1.01E-12	-5.90E-01	3.85E-12	-5.76E-01	2.43E-12	-5.83E-01
GP5d	large_intestine	1.24E-09	-5.09E-01	---	---	1.24E-09	-5.09E-01
HCC2998	large_intestine	6.97E-18	-7.06E-01	---	---	6.97E-18	-7.06E-01
HCC-56	large_intestine	---	---	1.23E-13	-6.12E-01	1.23E-13	-6.12E-01
HCT-116	large_intestine	3.04E-01	-1.22E-01	2.01E-02	2.22E-01	1.62E-01	5.00E-02
HCT-15	large_intestine	1.45E-08	-4.77E-01	4.67E-05	-3.55E-01	2.34E-05	-4.16E-01
Hs 675.T	large_intestine	---	---	8.74E-17	6.83E-01	8.74E-17	6.83E-01
Hs 698.T	large_intestine	---	---	1.25E-20	7.60E-01	1.25E-20	7.60E-01
HT-29	large_intestine	1.64E-17	-6.98E-01	5.90E-16	-6.65E-01	3.03E-16	-6.82E-01
HT55	large_intestine	5.76E-18	-7.07E-01	---	---	5.76E-18	-7.07E-01
KM12	large_intestine	1.30E-10	-5.36E-01	4.58E-06	-3.94E-01	2.29E-06	-4.65E-01
LoVo	large_intestine	1.67E-05	-3.72E-01	6.14E-03	-2.53E-01	3.08E-03	-3.13E-01
LS 180	large_intestine	---	---	1.50E-12	-5.86E-01	1.50E-12	-5.86E-01
LS-1034	large_intestine	1.38E-14	-6.34E-01	9.35E-13	-5.91E-01	4.74E-13	-6.13E-01
LS-123	large_intestine	2.62E-03	-2.74E-01	---	---	2.62E-03	-2.74E-01
LS-174T	large_intestine	5.02E-17	-6.88E-01	---	---	5.02E-17	-6.88E-01
LS-411N	large_intestine	1.45E-08	-4.77E-01	7.59E-09	-4.86E-01	1.10E-08	-4.82E-01
LS-513	large_intestine	2.09E-22	-7.93E-01	2.57E-19	-7.35E-01	1.29E-19	-7.64E-01
NCIH508	large_intestine	3.15E-17	-6.92E-01	1.97E-17	-6.96E-01	2.56E-17	-6.94E-01
NCIH630	large_intestine	8.43E-16	-6.62E-01	---	---	8.43E-16	-6.62E-01
NCIH716	large_intestine	1.19E-04	3.38E-01	6.99E-06	3.87E-01	6.30E-05	3.63E-01
NCIH747	large_intestine	9.23E-09	-4.83E-01	1.76E-08	-4.74E-01	1.34E-08	-4.79E-01
OUMS23	large_intestine	---	---	8.77E-02	-1.75E-01	8.77E-02	-1.75E-01
RCM1	large_intestine	7.71E-12	-5.68E-01	1.13E-11	-5.64E-01	9.51E-12	-5.66E-01
RKO	large_intestine	1.30E-04	3.36E-01	1.36E-05	3.76E-01	7.18E-05	3.56E-01
SKCO1	large_intestine	2.49E-10	-5.28E-01	7.47E-14	-6.17E-01	1.25E-10	-5.73E-01
SNU1040	large_intestine	---	---	9.91E-07	-4.18E-01	9.91E-07	-4.18E-01
SNU175	large_intestine	---	---	1.17E-05	-3.78E-01	1.17E-05	-3.78E-01
SNU283	large_intestine	---	---	8.26E-21	-7.64E-01	8.26E-21	-7.64E-01
SNU407	large_intestine	---	---	1.86E-13	-6.08E-01	1.86E-13	-6.08E-01
SNU503	large_intestine	---	---	1.43E-03	-2.87E-01	1.43E-03	-2.87E-01
SNUC1	large_intestine	7.19E-24	-8.20E-01	---	---	---	---
SNUC1	large_intestine	3.31E-24	-8.26E-01	3.61E-21	-7.70E-01	9.09E-22	-8.09E-01
SNUC2A	large_intestine	---	---	5.00E-07	-4.28E-01	5.00E-07	-4.28E-01
SNUC2B	large_intestine	1.16E-09	-5.09E-01	---	---	1.16E-09	-5.09E-01
SW1116	large_intestine	3.15E-17	-6.92E-01	3.56E-13	-6.01E-01	1.78E-13	-6.47E-01
SW1417	large_intestine	7.85E-11	-5.42E-01	1.36E-04	-3.35E-01	6.80E-05	-4.39E-01
SW1463	large_intestine	1.97E-17	-6.96E-01	5.90E-16	-6.65E-01	3.05E-16	-6.81E-01
SW403	large_intestine	3.57E-01	-1.14E-01	1.56E-19	-7.39E-01	1.79E-01	-4.27E-01
SW48	large_intestine	1.04E-04	-3.40E-01	3.15E-04	-3.19E-01	2.10E-04	-3.30E-01
SW480	large_intestine	---	---	1.73E-02	2.26E-01	1.73E-02	2.26E-01
SW620	large_intestine	2.57E-01	1.31E-01	1.43E-01	1.56E-01	2.00E-01	1.44E-01
SW837	large_intestine	1.37E-07	-4.46E-01	2.26E-03	-2.77E-01	1.13E-03	-3.62E-01
SW948	large_intestine	2.68E-18	-7.14E-01	2.39E-16	-6.73E-01	1.21E-16	-6.94E-01
T84	large_intestine	8.83E-14	-6.16E-01	2.73E-14	-6.28E-01	5.78E-14	-6.22E-01
Alexander cells	liver	---	---	6.51E-05	3.49E-01	6.51E-05	3.49E-01
C3A	liver	2.10E-01	-1.40E-01	---	---	---	---
C3A	liver	5.21E-02	-1.93E-01	4.50E-01	-1.00E-01	2.31E-01	-1.43E-01
Hep 3B2.1-7	liver	---	---	1.23E-01	1.62E-01	1.23E-01	1.62E-01
Hep G2	liver	---	---	4.69E-02	1.96E-01	4.69E-02	1.96E-01
HLE	liver	9.70E-12	5.65E-01	4.33E-18	7.10E-01	4.85E-12	6.38E-01
HLF	liver	---	---	1.23E-17	7.01E-01	1.23E-17	7.01E-01
HLF-a	liver	---	---	1.56E-19	7.39E-01	1.56E-19	7.39E-01
huH-1	liver	---	---	2.55E-02	2.15E-01	2.55E-02	2.15E-01

HUH-6-clone5	liver	1.26E-01	-1.62E-01	---	---	1.26E-01	-1.62E-01
HuH-7	liver	3.02E-05	3.62E-01	1.67E-05	3.72E-01	2.35E-05	3.67E-01
JHH-1	liver	---	---	7.24E-02	-1.82E-01	7.24E-02	-1.82E-01
JHH-2	liver	---	---	1.65E-08	4.75E-01	1.65E-08	4.75E-01
JHH-4	liver	---	---	2.34E-07	4.39E-01	2.34E-07	4.39E-01
JHH-5	liver	---	---	1.53E-01	-1.54E-01	1.53E-01	-1.54E-01
JHH-7	liver	---	---	2.10E-01	1.40E-01	2.10E-01	1.40E-01
PLC/PRF/5	liver	---	---	2.32E-04	3.25E-01	2.32E-04	3.25E-01
PLCPRF5	liver	1.19E-02	2.36E-01	---	---	1.19E-02	2.36E-01
SKHEP1	liver	3.76E-11	5.50E-01	2.62E-16	6.73E-01	1.88E-11	6.12E-01
SNU182	liver	---	---	1.23E-13	6.12E-01	1.23E-13	6.12E-01
SNU387	liver	3.56E-13	6.01E-01	3.15E-17	6.92E-01	1.78E-13	6.47E-01
SNU398	liver	---	---	1.51E-14	6.34E-01	1.51E-14	6.34E-01
SNU423	liver	1.87E-10	5.31E-01	1.12E-17	7.02E-01	9.35E-11	6.17E-01
SNU449	liver	1.74E-07	4.43E-01	7.29E-11	5.43E-01	8.70E-08	4.93E-01
SNU475	liver	4.86E-12	5.73E-01	1.51E-14	6.34E-01	2.44E-12	6.04E-01
SNU878	liver	---	---	1.70E-04	3.31E-01	1.70E-04	3.31E-01
A427	lung	1.53E-02	2.30E-01	---	---	1.53E-02	2.30E-01
A549	lung	7.57E-04	3.01E-01	7.90E-07	4.21E-01	3.79E-04	3.61E-01
ABC-1	lung	2.83E-06	-4.01E-01	2.09E-03	-2.79E-01	1.05E-03	-3.40E-01
BEN	lung	3.15E-06	-4.00E-01	1.94E-03	-2.81E-01	9.72E-04	-3.41E-01
CAL-12T	lung	1.04E-04	-3.40E-01	1.15E-02	-2.37E-01	5.80E-03	-2.89E-01
Calu-1	lung	8.80E-10	5.13E-01	2.01E-10	5.31E-01	5.41E-10	5.22E-01
Calu-3	lung	7.53E-03	-2.48E-01	1.18E-03	-2.92E-01	4.36E-03	-2.70E-01
Calu-6	lung	2.59E-11	5.54E-01	5.82E-14	6.20E-01	1.30E-11	5.87E-01
ChaGo-K-1	lung	2.04E-06	-4.06E-01	3.90E-03	-2.64E-01	1.95E-03	-3.35E-01
COLO-668	lung	1.12E-02	-2.38E-01	---	---	1.12E-02	-2.38E-01
COR-L105	lung	3.68E-02	-2.04E-01	---	---	3.68E-02	-2.04E-01
COR-L23	lung	4.81E-02	-1.96E-01	9.86E-02	-1.71E-01	7.34E-02	-1.84E-01
COR-L24	lung	---	---	1.87E-03	2.81E-01	1.87E-03	2.81E-01
COR-L279	lung	3.68E-01	-1.12E-01	---	---	3.68E-01	-1.12E-01
COR-L311	lung	---	---	2.10E-01	1.40E-01	2.10E-01	1.40E-01
COR-L47	lung	---	---	2.75E-01	1.28E-01	2.75E-01	1.28E-01
COR-L51	lung	1.43E-01	1.56E-01	---	---	1.43E-01	1.56E-01
COR-L88	lung	4.32E-01	1.03E-01	5.12E-01	9.18E-02	4.72E-01	9.74E-02
COR-L95	lung	---	---	4.50E-01	-1.00E-01	4.50E-01	-1.00E-01
COR-L96CAR	lung	1.66E-01	-1.51E-01	---	---	1.66E-01	-1.51E-01
CPC-N	lung	2.99E-06	4.01E-01	2.99E-06	4.01E-01	2.99E-06	4.01E-01
DMS 454	lung	---	---	8.77E-02	-1.75E-01	8.77E-02	-1.75E-01
DMS-114	lung	1.76E-08	4.74E-01	---	---	---	---
DMS-114	lung	1.01E-09	5.11E-01	1.87E-15	6.54E-01	9.05E-09	5.28E-01
DMS-153	lung	6.72E-02	-1.85E-01	1.76E-01	-1.48E-01	1.22E-01	-1.67E-01
DMS-273	lung	3.85E-12	5.76E-01	1.53E-11	5.60E-01	9.58E-12	5.68E-01
DMS-53	lung	5.45E-04	-3.08E-01	1.54E-03	-2.86E-01	1.04E-03	-2.97E-01
DMS-79	lung	1.91E-01	-1.45E-01	2.99E-01	1.23E-01	2.45E-01	-1.10E-02
DV-90	lung	4.48E-09	-4.92E-01	4.83E-08	-4.61E-01	2.64E-08	-4.77E-01
EBC-1	lung	---	---	5.06E-01	9.27E-02	5.06E-01	9.27E-02
EKVX	lung	8.77E-02	1.75E-01	---	---	---	---
EKVX	lung	1.01E-01	1.70E-01	---	---	9.44E-02	1.73E-01
EPLC-272H	lung	4.50E-01	-1.00E-01	2.47E-02	2.16E-01	2.37E-01	5.80E-02
HARA	lung	---	---	5.25E-01	9.01E-02	5.25E-01	9.01E-02
HCC-1195	lung	---	---	2.07E-02	-2.21E-01	2.07E-02	-2.21E-01
HCC-15	lung	---	---	4.50E-03	2.61E-01	4.50E-03	2.61E-01
HCC-366	lung	---	---	7.11E-09	4.86E-01	7.11E-09	4.86E-01
HCC4006	lung	---	---	1.23E-02	-2.36E-01	1.23E-02	-2.36E-01
HCC-44	lung	---	---	1.84E-07	4.42E-01	1.84E-07	4.42E-01
HCC-78	lung	---	---	3.28E-04	-3.18E-01	3.28E-04	-3.18E-01
HCC827	lung	---	---	1.32E-01	1.60E-01	1.32E-01	1.60E-01
HCC-95	lung	---	---	5.25E-01	9.01E-02	5.25E-01	9.01E-02
HOP-62	lung	6.22E-10	5.17E-01	---	---	6.22E-10	5.17E-01

HOP-92	lung	1.53E-11	5.60E-01	---	---	1.53E-11	5.60E-01
Hs 229.T	lung	---	---	3.57E-22	7.89E-01	3.57E-22	7.89E-01
Hs 618.T	lung	---	---	4.91E-22	7.87E-01	4.91E-22	7.87E-01
IA-LM	lung	5.41E-10	5.19E-01	2.32E-10	5.29E-01	3.87E-10	5.24E-01
IST-MES1	lung	3.55E-10	5.24E-01	2.57E-13	6.05E-01	1.78E-10	5.65E-01
IST-MES2	lung	---	---	6.13E-12	5.71E-01	6.13E-12	5.71E-01
IST-SL1	lung	8.98E-02	1.74E-01	---	---	8.98E-02	1.74E-01
IST-SL2	lung	1.18E-01	-1.64E-01	---	---	1.18E-01	-1.64E-01
KNS-62	lung	1.09E-03	-2.93E-01	2.47E-02	-2.16E-01	1.29E-02	-2.55E-01
LB647-SCLC	lung	1.87E-03	2.81E-01	---	---	1.87E-03	2.81E-01
LC-1/sq-SF	lung	---	---	5.00E-07	4.28E-01	5.00E-07	4.28E-01
LC-1F	lung	3.91E-01	-1.09E-01	---	---	3.91E-01	-1.09E-01
LCLC-103H	lung	3.21E-09	4.97E-01	8.99E-12	5.66E-01	1.61E-09	5.32E-01
LCLC-97TM1	lung	8.21E-04	-2.99E-01	4.57E-02	-1.97E-01	2.33E-02	-2.48E-01
LK-2	lung	2.43E-01	-1.34E-01	3.91E-01	1.09E-01	3.17E-01	-1.25E-02
LOU-NH91	lung	---	---	4.19E-09	4.93E-01	4.19E-09	4.93E-01
LU-134-A	lung	2.18E-01	-1.39E-01	---	---	2.18E-01	-1.39E-01
LU-135	lung	5.14E-05	3.53E-01	---	---	5.14E-05	3.53E-01
LU-139	lung	2.52E-01	1.32E-01	---	---	2.52E-01	1.32E-01
LU-65	lung	3.85E-01	-1.10E-01	2.02E-01	1.42E-01	2.94E-01	1.60E-02
LU99	lung	---	---	1.01E-15	6.60E-01	1.01E-15	6.60E-01
LU-99A	lung	5.41E-10	5.19E-01	---	---	5.41E-10	5.19E-01
LUDLU-1	lung	---	---	1.43E-05	-3.75E-01	1.43E-05	-3.75E-01
LXF-289	lung	1.65E-11	5.60E-01	1.71E-13	6.09E-01	8.34E-12	5.85E-01
MOR/CPR	lung	---	---	2.04E-05	-3.69E-01	2.04E-05	-3.69E-01
MPP89	lung	1.16E-14	6.36E-01	2.66E-15	6.51E-01	7.13E-15	6.44E-01
MS1	lung	3.14E-07	4.35E-01	---	---	3.14E-07	4.35E-01
MSTO211H	lung	5.90E-16	6.65E-01	7.75E-20	7.45E-01	2.95E-16	7.05E-01
NCIH1048	lung	1.94E-01	1.44E-01	2.14E-02	2.20E-01	1.08E-01	1.82E-01
NCIH1092	lung	2.40E-02	2.17E-01	4.66E-03	2.60E-01	1.43E-02	2.39E-01
NCIH1105	lung	4.69E-02	1.96E-01	1.68E-02	2.27E-01	3.19E-02	2.12E-01
NCIH1155	lung	1.09E-03	2.93E-01	8.94E-08	4.52E-01	5.45E-04	3.73E-01
NCIH1184	lung	---	---	9.23E-09	4.83E-01	9.23E-09	4.83E-01
NCIH1299	lung	7.47E-14	6.17E-01	2.23E-15	6.52E-01	3.85E-14	6.35E-01
NCIH1304	lung	6.70E-04	3.04E-01	---	---	6.70E-04	3.04E-01
NCIH1341	lung	---	---	1.00E-05	3.81E-01	1.00E-05	3.81E-01
NCIH1355	lung	1.59E-01	1.52E-01	7.15E-05	3.47E-01	7.95E-02	2.50E-01
NCIH1373	lung	---	---	1.78E-02	2.25E-01	1.78E-02	2.25E-01
NCIH1395	lung	2.36E-05	-3.66E-01	---	---	---	---
NCIH1395	lung	1.19E-04	-3.38E-01	1.59E-05	-3.73E-01	4.56E-05	-3.61E-01
NCIH1435	lung	---	---	3.14E-07	-4.35E-01	3.14E-07	-4.35E-01
NCIH1436	lung	2.27E-02	2.19E-01	---	---	2.27E-02	2.19E-01
NCIH1437	lung	1.63E-12	-5.85E-01	---	---	---	---
NCIH1437	lung	3.56E-12	-5.77E-01	---	---	---	---
NCIH1437	lung	6.13E-12	-5.71E-01	2.34E-07	-4.39E-01	3.90E-08	-5.56E-01
NCIH146	lung	3.10E-01	1.22E-01	2.52E-01	1.32E-01	2.81E-01	1.27E-01
NCIH1522	lung	5.92E-02	1.89E-01	---	---	5.92E-02	1.89E-01
NCIH1563	lung	1.74E-07	4.43E-01	1.63E-09	5.05E-01	8.78E-08	4.74E-01
NCIH1568	lung	---	---	4.06E-04	-3.14E-01	4.06E-04	-3.14E-01
NCIH157	lung	3.08E-10	5.26E-01	---	---	3.08E-10	5.26E-01
NCIH1573	lung	3.55E-10	-5.24E-01	9.23E-09	-4.83E-01	4.79E-09	-5.04E-01
NCIH1581	lung	---	---	6.22E-10	5.17E-01	6.22E-10	5.17E-01
NCIH1618	lung	1.32E-01	1.60E-01	3.63E-01	1.13E-01	2.48E-01	1.37E-01
NCIH1623	lung	---	---	2.53E-04	-3.23E-01	2.53E-04	-3.23E-01
NCIH1648	lung	---	---	1.55E-06	-4.11E-01	1.55E-06	-4.11E-01
NCIH1650	lung	3.20E-02	-2.08E-01	2.75E-01	1.28E-01	1.54E-01	-4.00E-02
NCIH1651	lung	4.35E-06	3.95E-01	2.20E-07	4.40E-01	2.29E-06	4.18E-01
NCIH1666	lung	1.32E-03	-2.89E-01	5.02E-04	-3.10E-01	9.11E-04	-3.00E-01
NCIH1693	lung	2.26E-03	2.77E-01	1.15E-02	2.37E-01	6.88E-03	2.57E-01
NCIH1694	lung	---	---	1.84E-07	4.42E-01	1.84E-07	4.42E-01

NCIH1703	lung	1.74E-10	5.32E-01	2.37E-13	6.05E-01	8.71E-11	5.69E-01
NCIH1734	lung	---	---	4.99E-03	2.59E-01	4.99E-03	2.59E-01
NCIH1755	lung	8.17E-02	-1.78E-01	4.36E-11	5.48E-01	4.09E-02	1.85E-01
NCIH1770	lung	1.87E-03	2.81E-01	---	---	1.87E-03	2.81E-01
NCIH1781	lung	---	---	2.59E-11	-5.54E-01	2.59E-11	-5.54E-01
NCIH1792	lung	5.11E-09	4.91E-01	7.14E-12	5.69E-01	2.56E-09	5.30E-01
NCIH1793	lung	5.68E-04	3.07E-01	1.19E-04	3.38E-01	3.44E-04	3.23E-01
NCIH1836	lung	---	---	6.67E-01	7.14E-02	6.67E-01	7.14E-02
NCIH1838	lung	5.51E-01	8.67E-02	3.52E-01	1.15E-01	4.52E-01	1.01E-01
NCIH1876	lung	---	---	1.08E-02	2.39E-01	1.08E-02	2.39E-01
NCIH1930	lung	2.20E-02	2.19E-01	1.95E-04	3.28E-01	1.11E-02	2.74E-01
NCIH1944	lung	---	---	1.29E-01	1.61E-01	1.29E-01	1.61E-01
NCIH196	lung	---	---	2.83E-19	7.34E-01	2.83E-19	7.34E-01
NCIH1963	lung	---	---	2.34E-03	2.76E-01	2.34E-03	2.76E-01
NCIH1975	lung	1.39E-02	2.32E-01	2.89E-04	3.21E-01	7.09E-03	2.77E-01
NCIH1993	lung	3.31E-10	-5.25E-01	---	---	3.31E-10	-5.25E-01
NCIH2009	lung	1.44E-02	-2.31E-01	1.53E-01	1.54E-01	8.37E-02	-3.85E-02
NCIH2023	lung	---	---	7.49E-05	3.46E-01	7.49E-05	3.46E-01
NCIH2029	lung	3.48E-02	-2.06E-01	2.27E-02	-2.19E-01	2.88E-02	-2.13E-01
NCIH2030	lung	3.33E-05	3.61E-01	3.76E-08	4.64E-01	1.67E-05	4.13E-01
NCIH2052	lung	2.51E-14	6.28E-01	5.90E-16	6.65E-01	1.28E-14	6.47E-01
NCIH2066	lung	---	---	6.36E-03	2.53E-01	6.36E-03	2.53E-01
NCIH2081	lung	1.22E-03	2.91E-01	9.80E-03	2.41E-01	5.51E-03	2.66E-01
NCIH2085	lung	---	---	3.02E-02	2.10E-01	3.02E-02	2.10E-01
NCIH2087	lung	1.94E-05	-3.70E-01	---	---	---	---
NCIH2087	lung	5.00E-07	-4.28E-01	2.47E-02	-2.16E-01	6.18E-03	-3.46E-01
NCIH209	lung	5.21E-02	1.93E-01	3.97E-01	1.08E-01	2.25E-01	1.51E-01
NCIH2106	lung	---	---	3.10E-01	-1.22E-01	3.10E-01	-1.22E-01
NCIH2107	lung	2.06E-01	1.41E-01	---	---	2.06E-01	1.41E-01
NCIH211	lung	---	---	2.70E-01	1.28E-01	2.70E-01	1.28E-01
NCIH2110	lung	---	---	1.04E-04	-3.40E-01	1.04E-04	-3.40E-01
NCIH2122	lung	---	---	1.05E-08	-4.81E-01	1.05E-08	-4.81E-01
NCIH2126	lung	4.09E-10	-5.22E-01	1.13E-11	-5.64E-01	2.10E-10	-5.43E-01
NCIH2141	lung	1.48E-02	2.30E-01	6.39E-02	1.86E-01	3.94E-02	2.08E-01
NCIH2170	lung	9.76E-11	-5.39E-01	1.75E-09	-5.04E-01	9.24E-10	-5.22E-01
NCIH2171	lung	1.95E-02	2.23E-01	1.05E-02	2.40E-01	1.50E-02	2.32E-01
NCIH2172	lung	---	---	2.16E-10	5.30E-01	2.16E-10	5.30E-01
NCIH2196	lung	7.57E-04	3.01E-01	1.73E-01	1.49E-01	8.69E-02	2.25E-01
NCIH2227	lung	2.08E-07	4.40E-01	2.93E-08	4.68E-01	1.19E-07	4.54E-01
NCIH2228	lung	3.03E-03	2.70E-01	4.04E-05	3.57E-01	1.54E-03	3.14E-01
NCIH226	lung	8.65E-09	4.84E-01	2.59E-11	5.54E-01	4.34E-09	5.19E-01
NCIH2286	lung	---	---	4.16E-14	6.23E-01	4.16E-14	6.23E-01
NCIH2291	lung	5.92E-02	-1.89E-01	6.23E-02	-1.87E-01	6.08E-02	-1.88E-01
NCIH23	lung	8.33E-12	5.67E-01	4.50E-12	5.74E-01	6.42E-12	5.71E-01
NCIH2330	lung	1.91E-01	1.45E-01	---	---	1.91E-01	1.45E-01
NCIH2342	lung	4.09E-10	-5.22E-01	1.17E-06	-4.15E-01	5.85E-07	-4.69E-01
NCIH2347	lung	3.03E-03	-2.70E-01	1.12E-02	-2.38E-01	7.12E-03	-2.54E-01
NCIH2405	lung	6.99E-06	3.87E-01	2.13E-08	4.72E-01	3.51E-06	4.30E-01
NCIH2444	lung	---	---	4.93E-01	-9.44E-02	4.93E-01	-9.44E-02
NCIH2452	lung	9.85E-09	4.82E-01	6.88E-14	6.18E-01	4.93E-09	5.50E-01
NCIH250	lung	3.30E-01	-1.18E-01	---	---	3.30E-01	-1.18E-01
NCIH28	lung	5.39E-05	3.52E-01	1.84E-07	4.42E-01	2.70E-05	3.97E-01
NCIH292	lung	2.04E-06	-4.06E-01	---	---	2.04E-06	-4.06E-01
NCIH322	lung	---	---	3.17E-05	-3.61E-01	3.17E-05	-3.61E-01
NCIH345	lung	2.99E-01	1.23E-01	---	---	2.99E-01	1.23E-01
NCIH358	lung	4.72E-07	-4.29E-01	1.58E-02	-2.29E-01	7.90E-03	-3.29E-01
NCIH378	lung	1.12E-02	2.38E-01	---	---	1.12E-02	2.38E-01
NCIH441	lung	3.08E-10	-5.26E-01	2.08E-07	-4.40E-01	1.04E-07	-4.83E-01
NCIH446	lung	1.74E-10	5.32E-01	3.56E-12	5.77E-01	8.88E-11	5.55E-01
NCIH460	lung	5.97E-06	3.89E-01	5.44E-11	5.46E-01	2.99E-06	4.68E-01

NCIH510	lung	---	---	5.00E-01	-9.35E-02	5.00E-01	-9.35E-02
NCIH510A	lung	2.06E-01	-1.41E-01	---	---	2.06E-01	-1.41E-01
NCIH520	lung	2.43E-01	-1.34E-01	2.99E-01	1.23E-01	2.71E-01	-5.50E-03
NCIH522	lung	8.94E-08	4.52E-01	1.87E-09	5.03E-01	4.56E-08	4.78E-01
NCIH524	lung	1.01E-07	4.51E-01	---	---	---	---
NCIH524	lung	9.37E-07	4.18E-01	7.00E-08	4.56E-01	3.03E-07	4.44E-01
NCIH526	lung	5.77E-01	-8.33E-02	1.47E-01	-1.56E-01	3.62E-01	-1.20E-01
NCIH596	lung	6.41E-01	-7.48E-02	2.90E-01	1.25E-01	4.66E-01	2.51E-02
NCIH64	lung	1.10E-01	1.67E-01	---	---	1.10E-01	1.67E-01
NCIH647	lung	---	---	1.45E-07	4.46E-01	1.45E-07	4.46E-01
NCIH650	lung	3.52E-14	6.25E-01	1.97E-17	6.96E-01	1.76E-14	6.61E-01
NCIH661	lung	4.69E-11	5.48E-01	9.58E-17	6.82E-01	2.35E-11	6.15E-01
NCIH69	lung	---	---	1.50E-01	1.55E-01	1.50E-01	1.55E-01
NCIH727	lung	1.08E-04	-3.39E-01	1.27E-02	-2.35E-01	6.40E-03	-2.87E-01
NCIH810	lung	4.10E-02	-2.01E-01	4.14E-01	1.05E-01	2.28E-01	-4.80E-02
NCIH82	lung	1.87E-09	5.03E-01	1.07E-07	4.50E-01	5.44E-08	4.77E-01
NCIH838	lung	1.53E-09	5.06E-01	---	---	1.53E-09	5.06E-01
NCIH841	lung	---	---	1.87E-15	6.54E-01	1.87E-15	6.54E-01
NCIH889	lung	1.26E-01	1.62E-01	3.01E-04	3.20E-01	6.32E-02	2.41E-01
NCIN417	lung	4.16E-12	5.75E-01	---	---	4.16E-12	5.75E-01
PC14	lung	9.91E-07	-4.18E-01	3.38E-03	-2.68E-01	1.69E-03	-3.43E-01
RERFLCAD1	lung	---	---	1.95E-02	-2.23E-01	1.95E-02	-2.23E-01
RERFLCAD2	lung	---	---	4.33E-02	-1.99E-01	4.33E-02	-1.99E-01
RERFLCAI	lung	---	---	7.05E-16	6.63E-01	7.05E-16	6.63E-01
RERFLCFM	lung	2.07E-02	2.21E-01	---	---	2.07E-02	2.21E-01
RERFLCKJ	lung	---	---	1.22E-11	-5.63E-01	1.22E-11	-5.63E-01
RERFLCMS	lung	2.34E-07	4.39E-01	1.42E-11	5.61E-01	1.17E-07	5.00E-01
RERFLCSQ1	lung	---	---	2.62E-16	6.73E-01	2.62E-16	6.73E-01
SBC1	lung	1.95E-02	2.23E-01	---	---	1.95E-02	2.23E-01
SBC5	lung	2.00E-08	4.73E-01	3.08E-10	5.26E-01	1.02E-08	5.00E-01
SCLC21H	lung	8.57E-02	1.76E-01	2.86E-02	2.12E-01	5.72E-02	1.94E-01
SHP77	lung	1.19E-02	2.36E-01	1.08E-04	3.39E-01	6.00E-03	2.88E-01
SKLU1	lung	4.92E-13	5.98E-01	2.41E-12	5.81E-01	1.45E-12	5.90E-01
SKMES1	lung	5.37E-06	3.91E-01	---	---	---	---
SKMES1	lung	3.28E-04	3.18E-01	9.91E-07	4.18E-01	8.49E-05	3.80E-01
SQ1	lung	---	---	4.82E-03	-2.59E-01	4.82E-03	-2.59E-01
SW 1271	lung	---	---	7.57E-15	6.40E-01	7.57E-15	6.40E-01
SW1573	lung	3.30E-12	5.77E-01	3.56E-13	6.01E-01	1.83E-12	5.89E-01
SW900	lung	2.43E-04	3.24E-01	2.43E-01	1.34E-01	1.22E-01	2.29E-01
UMC11	lung	8.37E-02	-1.77E-01	---	---	8.37E-02	-1.77E-01
COLO-680N	oesophagus	3.58E-04	-3.16E-01	2.70E-01	-1.28E-01	1.35E-01	-2.22E-01
EC-GI-10	oesophagus	1.08E-02	-2.39E-01	---	---	1.08E-02	-2.39E-01
HCE-4	oesophagus	4.45E-07	4.29E-01	---	---	4.45E-07	4.29E-01
KYSE-140	oesophagus	6.14E-03	-2.53E-01	7.60E-02	1.80E-01	4.11E-02	-3.65E-02
KYSE-150	oesophagus	3.10E-01	-1.22E-01	---	---	---	---
KYSE-150	oesophagus	3.38E-02	-2.07E-01	4.21E-02	-2.00E-01	1.74E-01	-1.63E-01
KYSE-180	oesophagus	1.05E-02	-2.40E-01	5.51E-01	8.67E-02	2.81E-01	-7.67E-02
KYSE-270	oesophagus	2.07E-02	2.21E-01	3.11E-02	2.09E-01	2.59E-02	2.15E-01
KYSE-30	oesophagus	---	---	5.84E-09	-4.89E-01	5.84E-09	-4.89E-01
KYSE-410	oesophagus	5.41E-10	-5.19E-01	1.27E-02	-2.35E-01	6.35E-03	-3.77E-01
KYSE-450	oesophagus	2.90E-01	-1.25E-01	5.70E-01	8.42E-02	4.30E-01	-2.04E-02
KYSE-510	oesophagus	3.28E-04	-3.18E-01	5.21E-02	-1.93E-01	2.62E-02	-2.56E-01
KYSE-520	oesophagus	2.10E-01	-1.40E-01	5.92E-05	3.50E-01	1.05E-01	1.05E-01
KYSE-70	oesophagus	5.35E-02	1.92E-01	1.15E-02	2.37E-01	3.25E-02	2.15E-01
OE19	oesophagus	2.38E-21	-7.74E-01	3.46E-20	-7.52E-01	1.85E-20	-7.63E-01
OE33	oesophagus	7.14E-12	-5.69E-01	3.32E-08	-4.66E-01	1.66E-08	-5.18E-01
T.T	oesophagus	---	---	5.37E-06	-3.91E-01	5.37E-06	-3.91E-01
TE1	oesophagus	1.23E-01	-1.62E-01	4.45E-02	1.98E-01	8.38E-02	1.80E-02
TE10	oesophagus	3.58E-02	-2.05E-01	---	---	3.58E-02	-2.05E-01
TE11	oesophagus	2.16E-06	-4.06E-01	1.44E-02	-2.31E-01	7.20E-03	-3.19E-01

TE12	oesophagus	4.58E-06	-3.94E-01	---	---	4.58E-06	-3.94E-01
TE15	oesophagus	1.29E-07	-4.47E-01	4.99E-03	-2.59E-01	2.50E-03	-3.53E-01
TE5	oesophagus	3.91E-06	-3.96E-01	1.43E-03	-2.87E-01	7.17E-04	-3.42E-01
TE6	oesophagus	8.32E-03	-2.46E-01	---	---	8.32E-03	-2.46E-01
TE8	oesophagus	7.05E-07	4.23E-01	---	---	7.05E-07	4.23E-01
TE9	oesophagus	1.60E-03	-2.85E-01	4.90E-05	-3.54E-01	8.25E-04	-3.20E-01
A2780	ovary	2.62E-16	6.73E-01	1.80E-17	6.97E-01	1.40E-16	6.85E-01
Caov-3	ovary	2.20E-02	-2.19E-01	2.22E-01	1.38E-01	1.22E-01	-4.05E-02
Caov-4	ovary	6.39E-02	-1.86E-01	6.55E-02	-1.85E-01	6.47E-02	-1.86E-01
COLO-720E	ovary	7.66E-10	5.14E-01	---	---	7.66E-10	5.14E-01
COV318	ovary	---	---	6.66E-07	4.23E-01	6.66E-07	4.23E-01
COV362	ovary	---	---	8.80E-10	5.13E-01	8.80E-10	5.13E-01
COV434	ovary	---	---	2.40E-11	5.55E-01	2.40E-11	5.55E-01
COV504	ovary	---	---	1.05E-10	5.38E-01	1.05E-10	5.38E-01
COV644	ovary	---	---	1.70E-04	-3.31E-01	1.70E-04	-3.31E-01
EFO-21	ovary	8.61E-05	3.44E-01	4.58E-06	3.94E-01	4.53E-05	3.69E-01
EFO-27	ovary	1.05E-02	2.40E-01	1.87E-03	2.81E-01	6.19E-03	2.61E-01
ES-2	ovary	---	---	2.17E-17	6.96E-01	2.17E-17	6.96E-01
FU-OV-1	ovary	---	---	4.38E-10	5.21E-01	4.38E-10	5.21E-01
Hey-A8	ovary	---	---	1.64E-14	6.33E-01	1.64E-14	6.33E-01
IGROV-1	ovary	8.32E-03	2.46E-01	8.55E-04	2.98E-01	4.59E-03	2.72E-01
JHOS-2	ovary	---	---	2.48E-01	-1.33E-01	2.48E-01	-1.33E-01
JHOS-4	ovary	---	---	2.52E-03	-2.75E-01	2.52E-03	-2.75E-01
KGN	ovary	1.38E-14	6.34E-01	---	---	1.38E-14	6.34E-01
KURAMOCHI	ovary	5.74E-03	2.55E-01	3.74E-07	4.32E-01	2.87E-03	3.44E-01
MCAS	ovary	---	---	1.56E-04	-3.32E-01	1.56E-04	-3.32E-01
NCIADRRES	ovary	5.46E-09	4.90E-01	---	---	5.46E-09	4.90E-01
OAW28	ovary	3.58E-02	-2.05E-01	---	---	3.58E-02	-2.05E-01
OAW42	ovary	1.87E-01	1.45E-01	3.58E-02	2.05E-01	1.11E-01	1.75E-01
OC314	ovary	2.65E-04	3.22E-01	1.24E-04	3.37E-01	1.95E-04	3.30E-01
OV56	ovary	---	---	6.67E-10	5.16E-01	6.67E-10	5.16E-01
OV7	ovary	---	---	1.51E-14	6.34E-01	1.51E-14	6.34E-01
OV90	ovary	---	---	1.87E-01	1.45E-01	1.87E-01	1.45E-01
OVCAR3	ovary	7.04E-03	-2.50E-01	5.89E-01	-8.16E-02	2.98E-01	-1.66E-01
OVCAR4	ovary	4.10E-02	-2.01E-01	---	---	---	---
OVCAR4	ovary	2.86E-02	-2.12E-01	6.08E-02	1.88E-01	4.29E-02	-1.07E-01
OVCAR5	ovary	1.18E-03	-2.92E-01	---	---	---	---
OVCAR5	ovary	1.54E-03	-2.86E-01	---	---	1.36E-03	-2.89E-01
OVCAR8	ovary	1.62E-10	5.33E-01	8.43E-16	6.62E-01	8.10E-11	5.98E-01
OVISE	ovary	---	---	8.57E-02	-1.76E-01	8.57E-02	-1.76E-01
OVKATE	ovary	---	---	4.57E-02	-1.97E-01	4.57E-02	-1.97E-01
OVMANA	ovary	---	---	7.53E-03	-2.48E-01	7.53E-03	-2.48E-01
OVSAHO	ovary	---	---	9.18E-03	2.43E-01	9.18E-03	2.43E-01
OVTOKO	ovary	---	---	1.74E-07	4.43E-01	1.74E-07	4.43E-01
PA1	ovary	4.16E-14	6.23E-01	---	---	4.16E-14	6.23E-01
RKN	ovary	---	---	2.83E-19	7.34E-01	2.83E-19	7.34E-01
RMGI	ovary	2.34E-03	-2.76E-01	1.12E-02	-2.38E-01	6.77E-03	-2.57E-01
RMUGS	ovary	---	---	5.10E-06	-3.92E-01	5.10E-06	-3.92E-01
RTSG	ovary	1.08E-01	1.68E-01	---	---	1.08E-01	1.68E-01
SKOV3	ovary	3.20E-02	2.08E-01	4.54E-08	4.62E-01	1.60E-02	3.35E-01
SNU840	ovary	---	---	1.18E-01	1.64E-01	1.18E-01	1.64E-01
SW626	ovary	9.86E-02	-1.71E-01	---	---	9.86E-02	-1.71E-01
TOV21G	ovary	---	---	1.11E-05	3.79E-01	1.11E-05	3.79E-01
TYKNU	ovary	6.78E-11	5.43E-01	1.45E-13	6.11E-01	3.40E-11	5.77E-01
AsPC-1	pancreas	1.05E-02	-2.40E-01	1.60E-03	-2.85E-01	6.05E-03	-2.63E-01
BxPC-3	pancreas	2.58E-08	-4.69E-01	1.04E-04	-3.40E-01	5.20E-05	-4.05E-01
CAPAN-1	pancreas	1.82E-16	-6.76E-01	2.75E-08	-4.69E-01	1.38E-08	-5.73E-01
Capan-2	pancreas	3.32E-08	-4.66E-01	7.91E-08	-4.54E-01	5.62E-08	-4.60E-01
CFPAC-1	pancreas	1.83E-06	-4.08E-01	6.14E-03	-2.53E-01	3.07E-03	-3.31E-01
DAN-G	pancreas	---	---	1.98E-01	-1.43E-01	1.98E-01	-1.43E-01

HPAF-II	pancreas	2.23E-11	-5.56E-01	2.23E-11	-5.56E-01	2.23E-11	-5.56E-01
Hs 766T	pancreas	---	---	2.36E-05	3.66E-01	2.36E-05	3.66E-01
HuP-T3	pancreas	1.05E-02	-2.40E-01	8.37E-02	-1.77E-01	4.71E-02	-2.09E-01
HuP-T4	pancreas	8.99E-12	-5.66E-01	2.32E-10	-5.29E-01	1.20E-10	-5.48E-01
KP-2	pancreas	---	---	4.83E-06	-3.93E-01	4.83E-06	-3.93E-01
KP-3	pancreas	---	---	4.08E-01	-1.06E-01	4.08E-01	-1.06E-01
KP-4	pancreas	1.31E-11	5.62E-01	1.31E-11	5.62E-01	1.31E-11	5.62E-01
L3.3	pancreas	---	---	1.31E-02	-2.34E-01	1.31E-02	-2.34E-01
MIA-PaCa-2	pancreas	3.14E-07	4.35E-01	3.33E-07	4.34E-01	3.24E-07	4.35E-01
PANC 02.03	pancreas	---	---	4.82E-03	-2.59E-01	4.82E-03	-2.59E-01
PANC 02.13	pancreas	---	---	2.10E-01	-1.40E-01	2.10E-01	-1.40E-01
PANC 03.27	pancreas	---	---	1.66E-01	-1.51E-01	1.66E-01	-1.51E-01
PANC 04.03	pancreas	---	---	7.15E-05	-3.47E-01	7.15E-05	-3.47E-01
PANC 05.04	pancreas	---	---	2.00E-08	-4.73E-01	2.00E-08	-4.73E-01
PANC 08.13	pancreas	---	---	4.01E-08	-4.63E-01	4.01E-08	-4.63E-01
PANC0327	pancreas	2.55E-02	-2.15E-01	---	---	2.55E-02	-2.15E-01
PANC0813	pancreas	7.35E-13	-5.94E-01	---	---	7.35E-13	-5.94E-01
PANC1	pancreas	---	---	5.82E-08	4.58E-01	5.82E-08	4.58E-01
PANC1005	pancreas	4.83E-06	-3.93E-01	---	---	4.83E-06	-3.93E-01
PATU8902	pancreas	---	---	4.12E-06	-3.95E-01	4.12E-06	-3.95E-01
PATU8988S	pancreas	---	---	2.97E-14	-6.27E-01	2.97E-14	-6.27E-01
PK1	pancreas	---	---	2.13E-04	-3.27E-01	2.13E-04	-3.27E-01
PK45H	pancreas	---	---	2.27E-08	4.71E-01	2.27E-08	4.71E-01
PK59	pancreas	---	---	2.26E-03	-2.77E-01	2.26E-03	-2.77E-01
PSN1	pancreas	1.13E-03	2.93E-01	3.74E-07	4.32E-01	5.65E-04	3.63E-01
QGP1	pancreas	---	---	3.02E-02	-2.10E-01	3.02E-02	-2.10E-01
SNU410	pancreas	---	---	4.48E-09	4.92E-01	4.48E-09	4.92E-01
SUIT2	pancreas	---	---	3.03E-03	2.70E-01	3.03E-03	2.70E-01
SW1990	pancreas	5.47E-08	-4.59E-01	7.90E-07	4.21E-01	4.22E-07	-1.90E-02
T3M4	pancreas	---	---	3.74E-07	-4.32E-01	3.74E-07	-4.32E-01
TCCPAN2	pancreas	---	---	1.40E-01	-1.57E-01	1.40E-01	-1.57E-01
YAPC	pancreas	1.28E-08	-4.79E-01	8.22E-05	-3.44E-01	4.11E-05	-4.12E-01
22RV1	prostate	2.33E-02	-2.18E-01	1.08E-02	-2.39E-01	1.71E-02	-2.29E-01
BPH-1	prostate	4.35E-06	-3.95E-01	---	---	4.35E-06	-3.95E-01
DU-145	prostate	1.08E-01	1.68E-01	3.73E-04	3.15E-01	5.42E-02	2.42E-01
LNCaP-Clone-FGC	prostate	2.32E-04	-3.25E-01	9.64E-04	-2.96E-01	5.98E-04	-3.11E-01
MDA PCa 2b	prostate	---	---	4.35E-06	-3.95E-01	4.35E-06	-3.95E-01
NCIH660	prostate	---	---	5.49E-02	-1.91E-01	5.49E-02	-1.91E-01
PC3	prostate	3.04E-01	1.22E-01	---	---	---	---
PC3	prostate	2.66E-01	1.29E-01	1.78E-02	2.25E-01	2.23E-01	1.50E-01
VCAP	prostate	---	---	6.43E-04	-3.04E-01	6.43E-04	-3.04E-01
A-253	salivary_gland	---	---	8.05E-03	2.47E-01	8.05E-03	2.47E-01
YD15	salivary_gland	---	---	2.13E-04	-3.27E-01	2.13E-04	-3.27E-01
A101D	skin	1.74E-10	5.32E-01	1.01E-12	5.90E-01	8.75E-11	5.61E-01
A2058	skin	2.49E-10	5.28E-01	5.35E-14	6.21E-01	1.25E-10	5.75E-01
A375	skin	1.30E-10	5.36E-01	6.94E-15	6.41E-01	6.50E-11	5.89E-01
A431	skin	1.86E-04	-3.29E-01	---	---	1.86E-04	-3.29E-01
A4-Fuk	skin	1.70E-04	3.31E-01	8.37E-02	1.77E-01	4.19E-02	2.54E-01
C32	skin	2.42E-08	4.70E-01	3.01E-11	5.53E-01	1.21E-08	5.12E-01
CHL-1	skin	2.63E-07	4.37E-01	3.24E-11	5.52E-01	1.32E-07	4.95E-01
COLO 741	skin	---	---	1.28E-08	4.79E-01	1.28E-08	4.79E-01
COLO 792	skin	---	---	1.05E-08	4.81E-01	1.05E-08	4.81E-01
COLO 829	skin	---	---	4.92E-14	6.22E-01	4.92E-14	6.22E-01
COLO-679	skin	7.75E-06	3.85E-01	1.75E-09	5.04E-01	3.88E-06	4.45E-01
COLO-783	skin	---	---	8.33E-12	5.67E-01	8.33E-12	5.67E-01
COLO-792	skin	1.23E-05	3.78E-01	---	---	1.23E-05	3.78E-01
COLO-792	skin	3.43E-04	3.17E-01	---	---	3.43E-04	3.17E-01
COLO-800	skin	1.74E-10	5.32E-01	3.56E-12	5.77E-01	8.88E-11	5.55E-01
COLO-818	skin	---	---	1.04E-13	6.14E-01	1.04E-13	6.14E-01
COLO-849	skin	---	---	3.30E-12	5.77E-01	3.30E-12	5.77E-01

CP66-MEL	skin	1.63E-09	5.05E-01	---	---	1.63E-09	5.05E-01
CP67-MEL	skin	1.13E-11	5.64E-01	---	---	1.13E-11	5.64E-01
DJM-1	skin	2.88E-05	-3.63E-01	---	---	2.88E-05	-3.63E-01
G-361	skin	5.00E-07	4.28E-01	1.33E-09	5.08E-01	2.51E-07	4.68E-01
GAK	skin	6.63E-06	3.88E-01	---	---	6.63E-06	3.88E-01
HMCB	skin	---	---	2.41E-12	5.81E-01	2.41E-12	5.81E-01
HMV-II	skin	1.54E-07	4.45E-01	---	---	1.54E-07	4.45E-01
Hs 294T	skin	---	---	1.91E-12	5.83E-01	1.91E-12	5.83E-01
Hs 600.T	skin	---	---	1.25E-23	8.15E-01	1.25E-23	8.15E-01
Hs 688(A).T	skin	---	---	3.81E-19	7.31E-01	3.81E-19	7.31E-01
Hs 695T	skin	---	---	1.04E-13	6.14E-01	1.04E-13	6.14E-01
Hs 839.T	skin	---	---	1.23E-22	7.98E-01	1.23E-22	7.98E-01
Hs 895.T	skin	---	---	2.33E-22	7.93E-01	2.33E-22	7.93E-01
Hs 934.T	skin	---	---	7.14E-23	8.02E-01	7.14E-23	8.02E-01
Hs 936.T	skin	---	---	7.66E-10	5.14E-01	7.66E-10	5.14E-01
Hs 939.T	skin	---	---	2.68E-18	7.14E-01	2.68E-18	7.14E-01
Hs 940.T	skin	---	---	1.12E-17	7.02E-01	1.12E-17	7.02E-01
Hs 944.T	skin	---	---	1.38E-14	6.34E-01	1.38E-14	6.34E-01
HT-144	skin	3.03E-13	6.03E-01	1.64E-17	6.98E-01	1.52E-13	6.51E-01
IGR-1	skin	9.37E-07	4.18E-01	6.78E-11	5.43E-01	4.69E-07	4.81E-01
IGR-37	skin	---	---	7.91E-08	4.54E-01	7.91E-08	4.54E-01
IGR-39	skin	---	---	1.27E-14	6.35E-01	1.27E-14	6.35E-01
IPC-298	skin	1.84E-07	4.42E-01	2.16E-10	5.30E-01	9.21E-08	4.86E-01
IST-MEL1	skin	8.83E-14	6.16E-01	---	---	8.83E-14	6.16E-01
K029AX	skin	---	---	1.76E-08	4.74E-01	1.76E-08	4.74E-01
LB2518-MEL	skin	9.51E-08	4.52E-01	---	---	9.51E-08	4.52E-01
LB373-MEL-D	skin	3.56E-12	5.77E-01	---	---	3.56E-12	5.77E-01
LOXIMVI	skin	8.25E-15	6.39E-01	2.00E-18	7.17E-01	4.13E-15	6.78E-01
M14	skin	1.29E-07	4.47E-01	---	---	1.29E-07	4.47E-01
Malme-3M	skin	1.12E-08	4.80E-01	9.70E-12	5.65E-01	5.60E-09	5.23E-01
MDA-MB-435	skin	2.00E-08	4.73E-01	---	---	2.00E-08	4.73E-01
MEL-HO	skin	1.55E-06	4.11E-01	7.90E-07	4.21E-01	1.17E-06	4.16E-01
MEL-JUSO	skin	3.31E-10	5.25E-01	2.67E-10	5.27E-01	2.99E-10	5.26E-01
Mewo	skin	2.68E-06	4.02E-01	4.38E-10	5.21E-01	1.34E-06	4.62E-01
MMAC-SF	skin	1.76E-08	4.74E-01	---	---	1.76E-08	4.74E-01
MZ2MEL.	skin	3.85E-12	5.76E-01	---	---	---	---
MZ2MEL.	skin	6.61E-12	5.70E-01	---	---	5.23E-12	5.73E-01
MZ7MEL	skin	7.59E-09	4.86E-01	---	---	7.59E-09	4.86E-01
RPMI7951	skin	9.27E-18	7.03E-01	7.75E-20	7.45E-01	4.67E-18	7.24E-01
RVH421	skin	4.09E-10	5.22E-01	8.25E-15	6.39E-01	2.05E-10	5.81E-01
SKMEL1	skin	---	---	2.13E-08	4.72E-01	2.13E-08	4.72E-01
SKMEL2	skin	7.47E-07	4.22E-01	5.05E-11	5.47E-01	3.74E-07	4.85E-01
SKMEL24	skin	1.87E-09	5.03E-01	1.10E-15	6.59E-01	9.35E-10	5.81E-01
SKMEL28	skin	1.84E-07	4.42E-01	5.41E-10	5.19E-01	9.23E-08	4.81E-01
SKMEL3	skin	1.47E-06	4.12E-01	---	---	---	---
SKMEL3	skin	1.93E-06	4.07E-01	3.14E-07	4.35E-01	1.30E-06	4.17E-01
SKMEL30	skin	1.39E-06	4.12E-01	3.49E-11	5.51E-01	6.95E-07	4.82E-01
SKMEL31	skin	---	---	4.50E-12	5.74E-01	4.50E-12	5.74E-01
SKMEL5	skin	8.60E-06	3.84E-01	1.76E-08	4.74E-01	4.31E-06	4.29E-01
UACC257	skin	7.49E-05	3.46E-01	1.51E-05	3.74E-01	4.50E-05	3.60E-01
UACC62	skin	1.40E-10	5.35E-01	3.52E-14	6.25E-01	7.00E-11	5.80E-01
WM115	skin	4.91E-15	6.45E-01	1.26E-16	6.79E-01	2.52E-15	6.62E-01
WM2664	skin	---	---	3.23E-14	6.26E-01	3.23E-14	6.26E-01
WM793	skin	---	---	4.57E-17	6.89E-01	4.57E-17	6.89E-01
WM88	skin	---	---	6.45E-16	6.64E-01	6.45E-16	6.64E-01
WM983B	skin	---	---	3.76E-11	5.50E-01	3.76E-11	5.50E-01
A204	soft_tissue	1.53E-11	5.60E-01	2.66E-15	6.51E-01	7.65E-12	6.06E-01
G-402	soft_tissue	---	---	1.91E-12	5.83E-01	1.91E-12	5.83E-01
GCT	soft_tissue	1.86E-13	6.08E-01	1.02E-18	7.23E-01	9.30E-14	6.66E-01
Hs 729	soft_tissue	---	---	7.19E-24	8.20E-01	7.19E-24	8.20E-01

HT-1080	soft_tissue	1.28E-12	5.88E-01	1.82E-16	6.76E-01	6.40E-13	6.32E-01
KYM-1	soft_tissue	---	---	5.84E-09	4.89E-01	5.84E-09	4.89E-01
MES-SA	soft_tissue	2.99E-06	4.01E-01	---	---	2.99E-06	4.01E-01
MFH-ino	soft_tissue	3.15E-17	6.92E-01	---	---	3.15E-17	6.92E-01
RD	soft_tissue	1.36E-18	7.20E-01	---	---	---	---
RD	soft_tissue	1.65E-18	7.19E-01	9.64E-25	8.35E-01	1.09E-18	7.49E-01
RH1	soft_tissue	3.56E-13	6.01E-01	---	---	3.56E-13	6.01E-01
RH18	soft_tissue	1.82E-18	7.18E-01	---	---	1.82E-18	7.18E-01
S117	soft_tissue	1.64E-14	6.33E-01	1.02E-17	7.02E-01	8.21E-15	6.68E-01
SJRH30	soft_tissue	5.33E-13	5.97E-01	7.58E-19	7.25E-01	2.67E-13	6.61E-01
SKLMS1	soft_tissue	5.39E-16	6.66E-01	5.12E-19	7.29E-01	2.70E-16	6.98E-01
SKUT1	soft_tissue	1.42E-11	5.61E-01	1.20E-15	6.58E-01	7.10E-12	6.10E-01
SW684	soft_tissue	1.97E-17	6.96E-01	---	---	1.97E-17	6.96E-01
SW872	soft_tissue	4.38E-10	5.21E-01	---	---	4.38E-10	5.21E-01
SW982	soft_tissue	9.27E-18	7.03E-01	---	---	9.27E-18	7.03E-01
TE 441.T	soft_tissue	---	---	1.31E-11	5.62E-01	1.31E-11	5.62E-01
TE 617.T	soft_tissue	---	---	1.64E-14	6.33E-01	1.64E-14	6.33E-01
VAESBJ	soft_tissue	1.01E-07	4.51E-01	---	---	1.01E-07	4.51E-01
23132-87	stomach	5.90E-16	-6.65E-01	3.46E-17	-6.91E-01	3.12E-16	-6.78E-01
AGS	stomach	1.65E-08	-4.75E-01	---	---	---	---
AGS	stomach	5.80E-10	-5.18E-01	6.29E-07	-4.24E-01	1.66E-07	-4.73E-01
ECC12	stomach	1.86E-04	3.29E-01	---	---	1.86E-04	3.29E-01
FU97	stomach	---	---	5.07E-02	1.94E-01	5.07E-02	1.94E-01
GCIY	stomach	1.80E-01	1.47E-01	6.43E-04	3.04E-01	9.03E-02	2.26E-01
GT3TKB	stomach	4.69E-11	5.48E-01	---	---	4.69E-11	5.48E-01
HGC-27	stomach	2.79E-11	5.54E-01	1.07E-14	6.37E-01	1.40E-11	5.96E-01
Hs 746T	stomach	---	---	6.37E-15	6.42E-01	6.37E-15	6.42E-01
HuG1-N	stomach	---	---	1.28E-12	-5.88E-01	1.28E-12	-5.88E-01
IM95	stomach	---	---	1.72E-15	-6.55E-01	1.72E-15	-6.55E-01
KATOIII	stomach	1.63E-12	-5.85E-01	1.44E-15	-6.56E-01	8.16E-13	-6.21E-01
KE-39	stomach	---	---	1.63E-12	-5.85E-01	1.63E-12	-5.85E-01
KE-97	stomach	---	---	8.37E-02	1.77E-01	8.37E-02	1.77E-01
MKN1	stomach	9.53E-06	3.82E-01	2.59E-11	5.54E-01	4.77E-06	4.68E-01
MKN28	stomach	3.56E-12	-5.77E-01	---	---	3.56E-12	-5.77E-01
MKN45	stomach	7.71E-12	-5.68E-01	2.79E-07	-4.36E-01	1.40E-07	-5.02E-01
MKN7	stomach	1.18E-03	-2.92E-01	---	---	---	---
MKN7	stomach	4.24E-04	-3.13E-01	3.85E-01	-1.10E-01	9.69E-02	-2.52E-01
MKN74	stomach	---	---	7.04E-03	-2.50E-01	7.04E-03	-2.50E-01
NCIN87	stomach	9.08E-11	-5.40E-01	1.12E-08	-4.80E-01	5.65E-09	-5.10E-01
NCISNU1	stomach	2.26E-03	2.77E-01	---	---	2.26E-03	2.77E-01
NCISNU16	stomach	2.18E-16	-6.74E-01	---	---	2.18E-16	-6.74E-01
NCISNU5	stomach	6.22E-01	-7.74E-02	---	---	6.22E-01	-7.74E-02
NUGC2	stomach	---	---	2.18E-01	-1.39E-01	2.18E-01	-1.39E-01
NUGC3	stomach	1.36E-05	-3.76E-01	5.74E-03	-2.55E-01	2.88E-03	-3.16E-01
NUGC4	stomach	---	---	2.59E-11	-5.54E-01	2.59E-11	-5.54E-01
OCUM1	stomach	---	---	1.05E-11	-5.65E-01	1.05E-11	-5.65E-01
RERFGC1B	stomach	---	---	2.10E-01	-1.40E-01	2.10E-01	-1.40E-01
RF48	stomach	1.38E-01	1.58E-01	---	---	1.38E-01	1.58E-01
SCH	stomach	6.55E-02	-1.85E-01	---	---	6.55E-02	-1.85E-01
SH10TC	stomach	---	---	1.01E-12	5.90E-01	1.01E-12	5.90E-01
SNU1	stomach	---	---	9.45E-05	3.42E-01	9.45E-05	3.42E-01
SNU16	stomach	---	---	9.59E-14	-6.15E-01	9.59E-14	-6.15E-01
SNU216	stomach	---	---	3.73E-04	-3.15E-01	3.73E-04	-3.15E-01
SNU5	stomach	---	---	1.76E-01	1.48E-01	1.76E-01	1.48E-01
SNU620	stomach	---	---	1.01E-15	-6.60E-01	1.01E-15	-6.60E-01
TGBC11TKB	stomach	2.48E-07	-4.38E-01	---	---	2.48E-07	-4.38E-01
8505C	thyroid	1.11E-05	3.79E-01	8.99E-12	5.66E-01	5.55E-06	4.73E-01
BCPAP	thyroid	3.43E-09	4.96E-01	9.70E-12	5.65E-01	1.72E-09	5.31E-01
BHT-101	thyroid	2.65E-04	3.22E-01	1.00E-05	3.81E-01	1.38E-04	3.52E-01
CAL-62	thyroid	6.51E-05	3.49E-01	2.06E-12	5.82E-01	3.26E-05	4.66E-01

CGTH-W-1	thyroid	4.33E-18	7.10E-01	---	---	4.33E-18	7.10E-01
FTC-133	thyroid	3.81E-10	5.23E-01	---	---	3.81E-10	5.23E-01
HTC-C3	thyroid	3.63E-01	-1.13E-01	---	---	3.63E-01	-1.13E-01
K5	thyroid	1.44E-15	6.56E-01	---	---	---	---
K5	thyroid	7.47E-14	6.17E-01	---	---	3.81E-14	6.37E-01
ML-1	thyroid	---	---	8.33E-12	5.67E-01	8.33E-12	5.67E-01
RO82W1	thyroid	1.01E-15	6.60E-01	---	---	1.01E-15	6.60E-01
SW579	thyroid	---	---	1.03E-21	7.81E-01	1.03E-21	7.81E-01
TT	thyroid	4.94E-02	-1.95E-01	---	---	---	---
TT	thyroid	5.92E-02	-1.89E-01	3.97E-01	1.08E-01	1.39E-01	-1.18E-01
TT2609C02	thyroid	---	---	7.57E-15	6.40E-01	7.57E-15	6.40E-01
BB30-HNC	upper_aerodigestive_tract	4.81E-02	-1.96E-01	---	---	4.81E-02	-1.96E-01
BB49-HNC	upper_aerodigestive_tract	1.74E-10	5.32E-01	---	---	1.74E-10	5.32E-01
BHY	upper_aerodigestive_tract	3.25E-01	-1.19E-01	---	---	3.25E-01	-1.19E-01
BICR 31	upper_aerodigestive_tract	---	---	6.67E-01	-7.14E-02	6.67E-01	-7.14E-02
Ca9-22	upper_aerodigestive_tract	1.59E-01	-1.52E-01	---	---	1.59E-01	-1.52E-01
CAL 27	upper_aerodigestive_tract	---	---	2.43E-03	-2.76E-01	2.43E-03	-2.76E-01
CAL-27	upper_aerodigestive_tract	9.53E-06	-3.82E-01	---	---	9.53E-06	-3.82E-01
CAL-33	upper_aerodigestive_tract	1.54E-07	-4.45E-01	---	---	1.54E-07	-4.45E-01
Detroit562	upper_aerodigestive_tract	3.91E-06	-3.96E-01	4.02E-01	-1.07E-01	2.01E-01	-2.52E-01
DOK	upper_aerodigestive_tract	9.20E-02	-1.73E-01	---	---	9.20E-02	-1.73E-01
FADU	upper_aerodigestive_tract	8.94E-08	-4.52E-01	2.43E-03	-2.76E-01	1.22E-03	-3.64E-01
HCE-T	upper_aerodigestive_tract	8.98E-02	-1.74E-01	---	---	8.98E-02	-1.74E-01
HN	upper_aerodigestive_tract	3.57E-01	1.14E-01	---	---	3.57E-01	1.14E-01
HO-1-N-1	upper_aerodigestive_tract	5.35E-03	-2.57E-01	---	---	5.35E-03	-2.57E-01
Hs 840.T	upper_aerodigestive_tract	---	---	2.55E-20	7.54E-01	2.55E-20	7.54E-01
HSC-2	upper_aerodigestive_tract	2.94E-02	-2.11E-01	---	---	---	---
HSC-2	upper_aerodigestive_tract	2.47E-02	-2.16E-01	2.80E-01	-1.27E-01	9.09E-02	-1.91E-01
HSC-3	upper_aerodigestive_tract	4.20E-01	-1.05E-01	1.15E-02	2.37E-01	2.16E-01	6.60E-02
HSC-4	upper_aerodigestive_tract	6.80E-03	-2.51E-01	1.35E-02	-2.33E-01	1.02E-02	-2.42E-01
KOSC-2	upper_aerodigestive_tract	5.45E-04	-3.08E-01	---	---	5.45E-04	-3.08E-01
LB771-HNC	upper_aerodigestive_tract	7.04E-03	2.50E-01	---	---	7.04E-03	2.50E-01
PE/CAPJ15	upper_aerodigestive_tract	---	---	4.66E-03	-2.60E-01	4.66E-03	-2.60E-01
PE/CAPJ34 (CLONE C12)	upper_aerodigestive_tract	---	---	2.95E-01	-1.24E-01	2.95E-01	-1.24E-01
PE/CAPJ41 (CLONE D2)	upper_aerodigestive_tract	---	---	5.00E-01	9.35E-02	5.00E-01	9.35E-02
PE/CAPJ49	upper_aerodigestive_tract	---	---	4.94E-02	1.95E-01	4.94E-02	1.95E-01
RPMI2650	upper_aerodigestive_tract	1.09E-03	2.93E-01	---	---	1.09E-03	2.93E-01
SAS	upper_aerodigestive_tract	1.50E-01	1.55E-01	---	---	1.50E-01	1.55E-01
SCC15	upper_aerodigestive_tract	1.08E-04	3.39E-01	3.68E-02	2.04E-01	1.85E-02	2.72E-01
SCC25	upper_aerodigestive_tract	1.18E-01	-1.64E-01	7.27E-04	3.02E-01	5.94E-02	6.90E-02
SCC4	upper_aerodigestive_tract	7.97E-02	-1.79E-01	9.63E-02	1.72E-01	8.80E-02	-3.50E-03
SCC9	upper_aerodigestive_tract	7.88E-04	-3.00E-01	2.85E-01	1.26E-01	1.43E-01	-8.70E-02
SNU1076	upper_aerodigestive_tract	---	---	2.14E-05	-3.68E-01	2.14E-05	-3.68E-01
SNU899	upper_aerodigestive_tract	---	---	4.58E-06	-3.94E-01	4.58E-06	-3.94E-01
AN3-CA	uterus	3.33E-07	4.34E-01	7.29E-11	5.43E-01	1.67E-07	4.89E-01
BOKU	uterus	2.28E-06	4.05E-01	---	---	2.28E-06	4.05E-01
C-33-A	uterus	2.07E-11	5.57E-01	---	---	---	---
C-33-A	uterus	1.28E-08	4.79E-01	---	---	6.41E-09	5.18E-01
C-4-II	uterus	3.54E-08	-4.65E-01	---	---	3.54E-08	-4.65E-01
CAL-39	uterus	1.37E-03	-2.88E-01	---	---	1.37E-03	-2.88E-01
Ca-Ski	uterus	1.32E-01	-1.60E-01	---	---	1.32E-01	-1.60E-01
COLO-684	uterus	1.07E-07	4.50E-01	---	---	1.07E-07	4.50E-01
DoTc2-4510	uterus	6.58E-08	-4.57E-01	---	---	6.58E-08	-4.57E-01
EFE-184	uterus	---	---	4.34E-03	2.62E-01	4.34E-03	2.62E-01
EN	uterus	---	---	5.78E-13	5.96E-01	5.78E-13	5.96E-01
ESS-1	uterus	3.56E-13	6.01E-01	1.87E-15	6.54E-01	1.79E-13	6.28E-01
HEC-1	uterus	1.58E-02	2.29E-01	---	---	1.58E-02	2.29E-01
HEC-108	uterus	---	---	6.99E-01	-6.72E-02	6.99E-01	-6.72E-02
HEC-151	uterus	---	---	1.56E-01	1.53E-01	1.56E-01	1.53E-01
HEC-1-A	uterus	---	---	2.40E-02	-2.17E-01	2.40E-02	-2.17E-01

HEC-251	uterus	---	---	2.27E-02	-2.19E-01	2.27E-02	-2.19E-01
HEC-265	uterus	---	---	4.81E-02	-1.96E-01	4.81E-02	-1.96E-01
HEC-50B	uterus	---	---	1.24E-09	5.09E-01	1.24E-09	5.09E-01
HEC-59	uterus	---	---	7.06E-02	1.83E-01	7.06E-02	1.83E-01
HEC-6	uterus	---	---	5.23E-04	3.09E-01	5.23E-04	3.09E-01
HeLaSF	uterus	1.37E-07	4.46E-01	---	---	1.37E-07	4.46E-01
HT-3	uterus	3.26E-03	-2.69E-01	---	---	3.26E-03	-2.69E-01
Ishikawa (Heraklio) 02 ER-	uterus	---	---	4.94E-02	1.95E-01	4.94E-02	1.95E-01
JHUEM-1	uterus	---	---	1.36E-04	-3.35E-01	1.36E-04	-3.35E-01
JHUEM-2	uterus	---	---	4.17E-17	6.90E-01	4.17E-17	6.90E-01
KLE	uterus	---	---	4.24E-04	3.13E-01	4.24E-04	3.13E-01
ME-180	uterus	1.93E-06	-4.07E-01	---	---	---	---
ME-180	uterus	8.60E-06	-3.84E-01	---	---	5.27E-06	-3.96E-01
MFE-280	uterus	1.91E-01	1.45E-01	2.27E-02	2.19E-01	1.07E-01	1.82E-01
MFE-296	uterus	2.14E-09	5.02E-01	4.69E-11	5.48E-01	1.09E-09	5.25E-01
MFE-319	uterus	---	---	4.62E-01	9.86E-02	4.62E-01	9.86E-02
OMC1	uterus	1.08E-04	-3.39E-01	---	---	1.08E-04	-3.39E-01
RL952	uterus	---	---	3.15E-04	-3.19E-01	3.15E-04	-3.19E-01
SIHA	uterus	6.98E-04	3.03E-01	---	---	6.98E-04	3.03E-01
SKGIIIA	uterus	4.99E-03	-2.59E-01	---	---	4.99E-03	-2.59E-01
SNGM	uterus	4.50E-01	1.00E-01	4.56E-01	9.95E-02	4.53E-01	9.98E-02
SW954	uterus	2.92E-03	-2.71E-01	---	---	2.92E-03	-2.71E-01
SW962	uterus	4.99E-03	2.59E-01	---	---	4.99E-03	2.59E-01
TCYIK	uterus	8.37E-02	-1.77E-01	---	---	8.37E-02	-1.77E-01

Table S5. Spearman correlation of EMT and drug response in each cancer type.

Compund	Drug Target	All_Cancer.Rho	Glio-/Neuro-blastoma.Rho	Ewing/Osteosarcoma.Rho	Bladder.Rho	Haemopoietic/Lymphoid.Rho	Breast.Rho	Renal.Rho	Colorectal.Rho	Liver.Rho	Lung.Rho	Esophagus.Rho	Ovarian.Rho	Pancreatic.Rho	Malignant Melanoma.Rho	Gastric.Rho	Thyroid.Rho	Head&Neck.Rho	Uterus.Rho
AP-24534	ABL	-0.18	-0.17	-0.16	0.33	-0.09	-0.56	-0.21	-0.29	-0.15	-0.04	-0.24	-0.24	0.23	-0.02	-0.30	-0.43	-0.21	-0.14
Nilotinib	ABL	-0.05	-0.09	-0.02	0.08	-0.05	0.14	0.06	-0.14	-0.07	-0.05	-0.10	-0.41	-0.28	-0.07	0.11	0.23	0.15	-0.04
Imatinib	ABL, KIT, PDGFR	-0.14	-0.20	-0.16	-1.00	-0.05	0.52	-0.32	0.01	0.00	0.18	0.14	1.00	0.00	-0.50	0.14	0.00	-0.60	-1.00
Dasatinib	ABL, SRC, KIT, PDGFR	-0.19	-0.49	-0.27	-1.00	-0.06	-0.20	0.32	-0.49	0.00	-0.13	0.10	-1.00	0.00	-0.56	-0.18	0.00	0.40	0.40
AKT inhibitor VIII	AKT1/2	0.12	-0.27	0.29	0.35	-0.07	0.20	-0.41	0.06	0.00	0.12	0.13	-0.32	0.01	0.12	-0.11	0.28	0.21	0.15
MK-2206	AKT1/2	-0.05	-0.05	0.31	0.24	-0.04	0.52	-0.31	-0.04	-0.39	-0.03	0.24	0.21	-0.46	-0.11	-0.11	-0.12	-0.05	0.03
A-443654	AKT1/2/3	0.08	-0.13	-0.23	-1.00	0.14	0.48	0.26	-0.23	0.00	-0.08	-0.05	-0.50	0.00	-0.09	-0.14	0.00	0.80	-0.10
NVP-TAE684	ALK	-0.10	0.15	0.05	-1.00	-0.14	-0.48	-0.41	-0.64	0.00	-0.08	0.20	0.50	0.00	-0.06	0.14	0.00	0.50	-0.20
AICAR	AMPK agonist	0.15	0.10	-0.25	0.03	0.10	0.06	-0.22	-0.76	-0.68	0.10	-0.25	-0.53	0.05	-0.20	-0.13	0.43	-0.08	0.47
Metformin	AMPK agonist	0.08	-0.21	-0.09	0.05	-0.14	0.36	0.00	0.13	0.36	0.04	-0.07	-0.27	-0.14	0.18	0.04	0.22	0.39	0.02
Bicalutamide	Androgen receptor (ANDR)	0.03	-0.36	-0.20	0.07	0.02	0.16	-0.10	-0.18	-0.40	0.07	0.36	-0.05	-0.15	0.22	0.48	-0.28	-0.17	0.50
QS11	ARFGAP	-0.06	0.15	0.13	-0.07	-0.09	-0.16	-0.11	-0.27	-0.02	0.09	-0.35	-0.37	-0.24	0.05	-0.04	0.08	-0.44	-0.09
KU-55933	ATM	-0.08	-0.10	-0.38	-0.19	-0.03	-0.15	-0.15	-0.40	0.07	-0.03	-0.17	-0.41	-0.05	0.06	-0.40	0.63	0.21	0.19
Thapsigargin	ATPase, Ca++ transporting, cardiac muscle, slow twitch 2	-0.12	0.12	0.05	-0.38	0.01	-0.31	-0.32	-0.40	-0.10	0.02	-0.27	-0.34	-0.30	-0.16	-0.28	0.04	0.04	-0.18
ZM-447439	AURKB	-0.15	0.08	0.17	0.24	0.04	-0.25	-0.28	-0.37	0.04	-0.03	-0.04	-0.30	-0.42	-0.15	-0.21	-0.42	0.19	-0.20
VX-680	Aurora A/B/C, FLT3, ABL1, JAK2,	0.00	0.03	0.02	-1.00	0.00	-0.03	-0.02	-0.01	0.00	-0.05	0.13	-1.00	0.00	0.15	0.00	0.00	0.70	-0.10
TW 37	BCL-2, BCL-XL	-0.26	0.14	0.21	-0.37	-1.00	0.03	0.48	-0.41	-0.32	-0.29	-0.06	-0.16	-0.56	-0.08	-0.30	0.05	-0.23	-0.45
ABT-263	BCL2, BCL-XL, BCL-W	0.03	0.38	0.43	0.07	0.06	-0.01	0.44	-0.05	0.00	0.05	0.47	0.47	0.40	-0.06	0.26	0.05	-0.40	0.26
Obatoclax Mesylate	BCL-2, BCL-XL, MCL-1	-0.08	0.10	0.15	-0.16	-0.12	-0.39	0.28	-0.38	0.03	-0.01	-0.32	-0.50	-0.18	0.08	-0.58	0.22	-0.35	-0.35
GNF-2	BCR-ABL	-0.04	-0.16	-0.46	-1.00	0.02	0.48	0.13	-0.37	0.00	0.24	0.06	1.00	0.00	-0.69	-0.61	0.00	-0.30	-0.90
WZ-1-84	BMX, Tec-family	0.00	-0.30	-0.23	-1.00	0.02	-0.07	0.46	0.23	0.00	-0.04	0.35	1.00	0.00	0.18	0.32	0.00	0.10	0.40
AZ628	BRAF	-0.19	-0.24	-0.10	1.00	-0.07	-0.80	0.15	-0.02	0.00	-0.16	0.23	-1.00	0.00	0.07	-0.19	0.00	-0.30	-0.20
PLX4720	BRAF	-0.12	-0.09	-0.16	0.00	-0.09	0.20	0.27	-0.40	-0.14	0.02	-0.13	-0.32	-0.35	-0.17	-0.25	0.57	-0.14	0.24
SB590885	BRAF	0.00	0.22	-0.02	0.36	-0.25	0.13	-0.37	-0.27	0.21	0.04	0.24	0.31	-0.30	-0.17	-0.29	0.62	-0.13	0.06

LFM-A13	BTK	-0.09	-0.09	0.07	-0.02	-0.02	0.04	0.17	-0.21	-0.30	0.15	-0.19	0.06	0.53	0.13	-0.11	0.27	-0.09	-0.37
PAC-1	CASP3 activator	0.06	0.21	0.09	0.53	-0.05	-0.18	0.16	-0.06	-0.17	0.31	0.16	-0.22	0.04	0.03	0.12	-0.09	-0.35	-0.15
RO-3306	CDK1	-0.07	0.12	-0.01	-0.37	-0.07	0.00	-0.10	-0.25	-0.07	-0.10	-0.36	0.38	-0.33	-0.16	-0.69	0.18	0.35	0.47
CGP-60474	CDK1/2/5/7/9	-0.04	-0.13	-0.20	-1.00	0.12	0.24	-0.17	-0.57	0.00	-0.06	0.14	-1.00	0.00	-0.47	-0.46	0.00	0.60	-0.50
CGP-082996	CDK4	0.05	-0.32	-0.26	-1.00	0.03	-0.01	-0.05	-0.24	0.00	0.04	0.01	1.00	0.00	0.07	-0.68	0.00	0.30	-0.20
PD-0332991	CDK4/6	-0.13	0.25	0.27	0.38	-0.05	-0.07	-0.48	-0.39	-0.57	0.00	0.02	-0.02	-0.11	-0.26	-0.60	0.50	0.21	-0.12
Roscovitine	CDKs	0.02	-0.16	-0.25	-1.00	0.06	-0.09	0.42	-0.05	0.00	0.21	-0.45	-0.50	0.00	-0.18	-0.12	0.00	0.30	-1.00
AZD7762	CHK1/2	-0.14	0.22	0.12	0.02	0.07	-0.49	0.17	-0.16	-0.39	-0.15	-0.10	0.09	-0.38	-0.08	-0.11	-0.12	0.31	-0.14
Pyrimethamine	Dihydrofolate reductase (DHFR)	0.08	-0.20	-0.23	1.00	-0.05	-0.18	-0.01	-0.14	0.00	0.12	-0.20	1.00	0.00	-0.23	-0.19	0.00	0.20	-0.80
Methotrexate	Dihydrofolate reductase (DHFR)	0.04	0.27	0.12	0.31	0.15	0.19	-0.18	-0.22	0.43	-0.12	-0.02	-0.61	-0.10	-0.22	-0.04	-0.57	0.01	-0.03
Cisplatin	DNA crosslinker	-0.23	-0.19	0.11	-0.28	-0.01	0.00	-0.23	-0.18	-0.82	-0.18	-0.25	-0.37	-0.48	-0.33	-0.33	-0.13	0.36	-0.34
Mitomycin C	DNA crosslinker	-0.02	0.17	0.46	-0.31	-0.30	-0.01	0.09	-0.06	-0.17	0.10	-0.17	-0.53	-0.35	-0.17	-0.04	-0.28	0.12	0.35
Bleomycin	DNA damage	-0.09	0.08	0.16	-0.44	-0.25	-0.18	0.12	-0.27	0.27	-0.10	-0.39	-0.46	-0.48	-0.28	-0.03	-0.03	-0.11	0.03
Doxorubicin	DNA intercalating	-0.08	-0.01	0.35	-0.20	-0.16	-0.13	-0.07	-0.27	-0.28	0.05	-0.28	-0.51	-0.55	0.03	-0.20	-0.26	0.12	-0.21
Gemcitabine	DNA replication	-0.11	0.17	0.31	-0.07	-0.21	0.01	0.19	-0.34	0.12	0.05	-0.17	-0.63	-0.43	-0.06	-0.41	-0.19	0.15	-0.18
Cytarabine	DNA synthesis	-0.21	0.04	0.26	0.23	-0.08	-0.38	-0.18	-0.12	-0.79	-0.16	-0.29	-0.40	-0.56	-0.42	-0.16	0.18	-0.21	0.07
NU-7441	DNAPK	-0.23	-0.11	-0.47	-0.10	0.11	-0.10	-0.24	-0.21	0.14	-0.09	0.12	-0.23	0.10	-0.16	-0.01	0.00	0.10	-0.27
Gefitinib	EGFR	0.23	0.19	-0.01	0.02	0.02	0.14	0.17	0.01	0.29	0.21	-0.34	0.22	0.27	0.16	-0.14	0.23	0.26	0.76
Erlotinib	EGFR	0.01	-0.02	-0.25	-1.00	0.06	0.25	0.58	0.39	0.00	-0.04	0.37	0.50	0.00	0.15	0.12	0.00	0.90	0.40
BIBW2992	EGFR, ERBB2	0.37	0.01	-0.17	0.35	0.03	0.49	0.28	0.18	0.18	0.31	-0.04	0.36	0.42	-0.17	0.24	0.00	0.20	0.66
Lapatinib	EGFR, ERBB2	-0.03	-0.36	-0.49	1.00	-0.13	0.64	0.40	0.27	0.00	0.05	0.08	1.00	0.00	0.24	-0.36	0.00	0.80	-0.40
XMD8-85	ERK5 (MK07)	-0.03	-0.44	-0.20	1.00	0.03	-0.23	0.01	-0.17	0.00	-0.03	0.10	-0.50	0.00	0.39	-0.43	0.00	0.30	-0.40
PF-562271	FAK	-0.05	0.03	0.23	0.19	-0.11	-0.25	-0.39	-0.06	-0.27	0.02	0.34	-0.17	0.07	0.00	-0.51	-0.07	0.30	-0.17
FTI-277	Farnesyl transferase (FNTA)	-0.08	-0.23	-0.02	-0.17	0.03	-0.02	0.08	-0.12	-0.25	-0.05	-0.01	-0.36	-0.23	-0.08	-0.37	-0.61	0.18	0.10
Tipifarnib	Farnesyl-transferase (FNTA)	-0.01	0.09	-0.09	-0.06	-0.15	-0.11	0.05	-0.30	0.05	0.07	-0.28	-0.39	-0.35	0.24	-0.27	0.14	-0.04	0.00
PD-173074	FGFR1/3	-0.17	-0.18	0.02	-0.12	-0.13	-0.15	-0.11	-0.21	0.14	-0.17	0.09	-0.19	0.10	0.02	-0.14	-0.37	-0.31	-0.22
CEP-701	FLT3, JAK2, NTRK1, RET	-0.22	0.13	0.18	0.30	-0.10	-0.41	-0.06	-0.38	-0.71	-0.10	-0.21	-0.57	-0.75	0.08	-0.16	-0.25	0.38	0.04

Salubrinal	GADD34-PP1C phosphatase	0.09	-0.13	-0.27	-1.00	0.22	0.23	0.08	-0.10	0.00	0.06	-0.18	1.00	0.00	0.28	0.02	0.00	0.50	-0.80
BMS-708163	gamma-secretase	-0.03	0.06	0.22	0.33	-1.00	0.21	-0.25	0.08	0.21	-0.03	-0.27	-0.18	0.44	0.20	-0.11	0.48	-0.36	-0.01
Z-LLNle-CHO	g-secretase	-0.06	-0.31	-0.10	-1.00	0.02	-0.38	0.54	0.04	0.00	-0.15	-0.25	-1.00	0.00	-0.21	0.11	0.00	-0.60	0.40
SB 216763	GSK3A/B	-0.11	0.07	-0.31	-0.15	-0.01	-0.43	0.20	0.08	0.09	-0.10	0.29	-0.26	-0.14	-0.25	0.34	-0.40	0.06	-0.31
CHIR-99021	GSK3B	-0.14	-0.13	-0.05	0.03	-0.05	-0.26	0.51	0.22	-0.02	-0.12	0.16	-0.25	0.00	-0.46	0.24	-0.77	0.06	-0.13
MS-275	HDAC	0.07	0.18	-0.20	1.00	-0.03	0.22	-0.13	0.25	0.00	0.14	-0.37	1.00	0.00	-0.31	-0.17	0.00	0.60	-0.20
Vorinostat	HDAC inhibitor Class I, IIa, IIb, IV	-0.08	0.42	0.43	0.22	-0.05	0.14	-0.19	-0.29	0.00	-0.15	-0.45	0.04	-0.36	0.10	-0.17	0.28	0.09	0.19
Elesclomol	HSP70	-0.27	0.21	0.08	-0.39	-0.08	-0.54	-0.12	-0.50	-0.75	-0.17	-0.32	-0.01	-0.37	-0.37	-0.04	-0.15	0.13	-0.35
AUY922	HSP90	-0.13	-0.15	0.19	-0.19	-0.05	-0.01	-0.16	-0.32	0.08	0.01	-0.24	-0.35	-0.57	-0.07	-0.31	-0.28	-0.25	0.04
17-AAG	HSP90	-0.05	-0.43	-0.16	0.22	-0.12	0.07	-0.54	-0.18	0.43	-0.04	0.19	-0.24	-0.61	-0.40	-0.07	-0.42	0.19	0.25
CCT018159	HSP90	-0.03	0.05	-0.24	-0.04	-1.00	-0.08	-0.36	-0.43	0.40	0.12	-0.06	-0.62	-0.57	-0.29	-0.30	-0.02	0.39	0.24
OSI-906	IFG1R	-0.04	0.47	0.43	0.09	-0.05	-0.41	-0.11	0.16	-0.22	0.10	-0.01	0.03	0.20	-0.06	-0.11	0.25	-0.76	0.02
BMS-754807	IGF1R	-0.11	0.24	0.36	-0.31	-0.06	-0.40	-0.09	0.25	-0.52	0.09	0.21	-0.08	-0.03	0.07	-0.28	0.12	-0.34	-0.03
BMS-536924	IGF1R	-0.09	-0.10	-0.09	-1.00	-0.08	-0.17	0.21	0.00	0.00	-0.10	0.25	-1.00	0.00	-0.13	-0.04	0.00	0.60	0.40
KIN001-135	IKKE	-0.06	-0.19	-0.20	-1.00	0.01	0.07	0.21	-0.18	0.00	-0.02	-0.17	0.50	0.00	0.30	-0.07	0.00	0.80	-0.40
BMS-509744	ITK	-0.09	0.04	0.09	-1.00	-0.03	0.05	0.01	0.08	0.00	-0.08	-0.17	0.50	0.00	-0.15	-0.54	0.00	0.90	0.00
JNK Inhibitor VIII	JNK	-0.10	0.12	0.20	0.33	0.24	0.09	-0.17	-0.30	-0.43	-0.15	-0.12	0.08	-0.36	-0.20	-0.14	0.55	-0.20	-0.03
JNK-9L	JNK	-0.02	0.14	0.18	0.08	0.01	-0.31	-0.31	-0.13	-0.15	0.10	-0.15	-0.26	0.36	0.03	-0.53	-0.13	0.20	0.03
AS601245	JNK	-0.05	-0.07	-0.24	-0.31	-0.13	-0.33	0.11	-0.13	0.03	0.16	0.04	-0.51	-0.26	-0.09	-0.25	-0.19	-0.32	-0.38
S-Trityl-L-cysteine	KIF11	0.03	-0.07	-0.12	1.00	0.09	0.35	-0.25	-0.23	0.00	-0.06	-0.22	-0.50	0.00	-0.15	-0.43	0.00	0.90	-0.20
Midostaurin	KIT	-0.21	-0.26	0.11	-0.07	-0.16	-0.64	0.06	-0.25	-0.35	-0.02	-0.25	-0.41	-0.08	0.10	-0.30	-0.50	0.03	-0.04
JNJ-26854165	MDM2	-0.22	-0.07	0.24	0.26	1.00	-0.43	0.18	-0.28	-0.14	-0.19	-0.09	-0.79	-0.27	0.09	0.53	-0.37	0.41	-0.35
Nutlin-3a	MDM2	-0.10	0.22	0.01	0.37	-0.05	0.15	0.13	-0.22	0.32	-0.13	-0.22	-0.10	-0.13	-0.29	-0.22	0.05	-0.26	0.14
PD-0325901	MEK1/2	0.10	0.00	-0.09	0.49	-0.03	0.04	-0.18	0.05	-0.64	0.11	0.10	0.36	-0.25	-0.09	0.36	0.98	0.22	0.64
AZD6244	MEK1/2	0.09	-0.11	-0.05	0.41	0.04	0.08	0.18	-0.24	0.18	0.08	0.01	-0.16	-0.47	-0.11	0.08	0.74	0.20	0.27
RDEA119	MEK1/2	-0.01	-0.29	0.01	0.44	-0.16	-0.26	-0.12	0.07	0.04	0.04	0.14	-0.09	-0.14	-0.12	0.27	0.88	0.02	0.28
CI-1040	MEK1/2	0.01	0.04	-0.02	0.02	0.16	-0.15	-0.19	0.04	-0.20	0.04	-0.14	-0.25	-0.34	0.01	0.26	0.35	0.32	0.46
PHA-665752	MET	-0.12	-0.22	-0.02	-1.00	-0.08	0.09	-0.07	-0.38	0.00	0.25	0.22	-0.50	0.00	-0.47	0.14	0.00	-0.60	0.30
PF-02341066	MET, ALK	-0.05	-0.10	-0.01	-1.00	-0.07	-0.12	-0.74	0.01	0.00	0.06	0.22	0.50	0.00	0.29	0.46	0.00	0.00	-0.66
Vinblastine	Microtubules	-0.17	-0.01	0.03	0.23	0.14	-0.12	-0.73	-0.49	-0.93	-0.17	-0.03	-0.41	-0.68	-0.50	-0.34	-0.53	0.43	-0.06
Docetaxel	Microtubules	-0.17	-0.14	-0.02	0.22	0.03	-0.18	-0.63	-0.38	-0.79	-0.13	-0.04	-0.31	-0.61	-0.56	0.04	-0.27	0.33	-0.04

Vinorelbine	Microtubules	-0.04	0.10	0.15	0.31	-0.10	-0.11	-0.69	-0.07	-0.47	0.07	0.08	-0.32	-0.13	0.21	-0.22	-0.33	0.26	-0.03
Paclitaxel	Microtubules	0.03	-0.21	0.03	-1.00	0.05	0.13	-0.24	-0.30	0.00	-0.11	0.17	-1.00	0.00	0.06	0.00	0.00	1.00	-0.10
Epothilone B	Microtubules	-0.01	0.08	0.22	-0.29	-0.03	-0.11	-0.13	-0.21	-0.22	0.07	-0.14	-0.37	-0.55	0.12	-0.14	0.04	0.18	0.05
Temsirolimus	MTOR	-0.21	-0.14	0.21	-0.06	0.10	0.28	-0.43	-0.46	-0.29	-0.11	0.03	-0.56	-0.47	-0.13	-0.30	-0.35	-0.08	-0.18
Rapamycin	MTOR	0.08	-0.08	0.28	1.00	0.15	-0.19	-0.17	-0.62	0.00	0.13	0.35	-0.50	0.00	-0.06	0.07	0.00	0.30	-0.20
JW-7-52-1	MTOR	0.00	-0.22	-0.12	1.00	0.15	0.26	0.25	-0.43	0.00	-0.23	0.32	-1.00	0.00	-0.12	-0.25	0.00	0.80	0.00
AZD8055	mTORC1/2	-0.09	-0.06	-0.01	0.26	-0.02	0.12	-0.32	-0.43	-0.07	-0.17	0.04	-0.28	-0.07	-0.34	-0.50	0.74	-0.08	-0.11
Parthenolide	NFKB1	-0.13	-0.23	0.28	-1.00	-0.10	-0.25	0.25	-0.37	0.00	-0.06	-0.05	0.50	0.00	0.45	-0.17	0.00	0.80	0.10
GW 441756	NTRK1	0.04	0.37	0.30	0.13	-0.03	0.13	-0.17	0.02	-0.46	-0.03	-0.02	0.40	0.18	0.06	0.00	-0.02	0.14	-0.31
VX-702	p38	-0.07	0.12	0.07	0.06	-0.03	0.21	-0.15	-0.21	-0.54	-0.12	-0.10	-0.38	0.21	0.07	0.13	-0.55	-0.26	-0.10
BIRB 0796	p38, JNK2	0.01	0.40	-0.16	-0.13	0.05	0.22	-0.33	-0.08	-0.14	-0.04	0.18	0.00	-0.08	-0.08	-0.18	0.37	-0.16	0.24
PF-4708671	p70 S6KA	0.00	-0.03	-0.03	-0.17	-1.00	0.30	0.03	-0.26	0.75	0.02	-0.01	-0.24	0.07	0.19	-0.11	0.07	0.23	0.34
IPA-3	PAK	-0.08	0.23	0.04	-0.13	0.10	-0.05	-0.09	-0.59	-0.15	0.13	-0.09	-0.51	-0.39	0.11	-0.12	0.48	-0.42	-0.35
AG-014699	PARP1, PARP2	-0.28	0.02	0.47	0.32	-1.00	-0.43	-0.13	-0.21	0.32	-0.14	-0.19	-0.62	-0.19	-0.42	-0.10	0.12	0.24	0.14
AZD-2281	PARP1/2	-0.23	-0.06	0.51	0.00	-0.11	-0.33	-0.54	-0.36	-0.11	-0.12	-0.40	-0.42	-0.24	-0.27	-0.39	0.05	-0.29	-0.34
ABT-888	PARP1/2	0.03	0.18	0.17	-0.04	-0.02	0.15	-0.22	-0.19	-0.21	0.02	0.04	-0.14	-0.21	-0.31	-0.29	0.45	0.09	-0.24
Axitinib	PDGFR, KIT, VEGFR	-0.22	0.00	0.17	0.17	-0.18	-0.20	-0.05	-0.19	0.18	-0.20	-0.01	-0.02	-0.12	-0.25	-0.08	-0.60	-0.15	-0.40
Sunitinib	PDGFRA, PDGFRB, VEGFR(KDR), KIT, FLT3	-0.02	0.14	0.08	-1.00	-0.14	-0.24	-0.25	-0.25	0.00	-0.20	0.28	-0.50	0.00	0.00	-0.04	0.00	0.00	0.60
Sorafenib	PDGFRA, PDGFRB, VEGFR(KDR), KIT, FLT3	0.00	0.01	-0.24	-1.00	0.00	0.20	0.03	-0.06	0.00	-0.08	0.07	1.00	0.00	0.06	0.12	0.00	0.20	-0.40
GDC0941	PI3K (class 1)	-0.13	-0.12	-0.20	-0.11	0.06	0.32	-0.09	-0.41	0.07	-0.07	-0.48	-0.24	0.01	-0.15	-0.27	0.50	-0.17	0.00
NVP-BEZ235	PI3K (Class 1) and mTORC1/2	-0.25	-0.34	0.02	-0.07	-0.01	-0.06	-0.31	-0.49	-0.07	-0.12	0.11	-0.42	-0.50	-0.37	-0.51	-0.45	-0.12	-0.19
WO2009093972	PI3Kb	-0.20	-0.40	-0.36	-0.09	-1.00	0.39	0.18	-0.73	0.36	-0.10	-0.35	-0.22	-0.12	-0.05	0.03	0.40	0.13	-0.34
AZD6482	PI3Kb (P3C2B)	-0.12	-0.29	-0.19	-0.50	0.04	0.29	-0.36	-0.25	-0.02	0.04	0.20	-0.09	0.09	0.04	-0.38	-0.41	0.54	-0.19
GW843682X	PLK1	0.01	-0.01	-0.10	-1.00	-0.05	0.14	-0.25	-0.15	0.00	-0.31	-0.11	-1.00	0.00	-0.13	-0.07	0.00	0.80	-0.10
BI-2536	PLK1/2/3	0.01	-0.18	-0.16	-1.00	0.05	0.20	-0.42	-0.14	0.00	-0.16	-0.32	0.50	0.00	0.03	0.00	0.00	0.70	-0.10
CCT007093	PPM1D	-0.03	0.10	0.04	-0.05	1.00	0.37	-0.23	-0.14	-0.18	0.03	-0.19	-0.30	0.65	0.03	-0.25	-0.32	0.51	0.37
Bryostatin 1	PRKC	-0.05	-0.26	-0.22	-0.09	-0.03	-0.12	0.05	-0.04	0.78	0.06	0.14	0.33	-0.17	0.11	-0.30	0.70	0.04	-0.08
DMOG	Prolyl-4-Hydroxylase	-0.08	0.05	-0.29	0.35	0.10	-0.34	-0.04	0.05	0.13	0.13	0.28	0.00	0.28	-0.01	-0.50	0.09	0.07	0.31

Bortezomib	Proteasome	-0.20	-0.39	-0.43	1.00	0.11	-0.12	0.33	0.14	0.00	-0.03	0.03	1.00	0.00	-0.04	-0.57	0.00	0.90	0.10
MG-132	Proteasome	-0.20	-0.40	-0.16	1.00	-0.11	0.30	-0.02	0.13	0.00	-0.13	-0.23	0.50	0.00	0.11	-0.48	0.00	-0.10	0.00
EHT 1864	Rac GTPases	-0.02	-0.02	0.47	0.16	1.00	0.26	0.02	-0.28	0.75	-0.01	0.07	-0.26	0.21	0.26	-0.29	0.08	0.00	0.08
Bexarotene	Retinioic acid X family agonist	-0.11	-0.22	-0.46	-0.32	0.12	-0.38	-0.11	-0.30	0.03	0.16	0.05	-0.56	-0.03	-0.01	-0.40	0.03	-0.27	-0.04
ATRA	Retinoic acid and retinoid X receptor agonist	-0.04	0.19	0.02	-0.02	0.24	0.14	-0.33	-0.25	-0.21	-0.15	0.06	-0.33	0.19	0.21	-0.12	0.05	0.08	0.21
GSK269962A	ROCK	-0.24	-0.26	-0.29	1.00	0.01	0.26	-0.47	0.14	0.00	0.00	-0.30	-0.50	0.00	-0.42	-0.55	0.00	-0.40	0.20
CMK	RSK	-0.08	-0.11	0.25	-1.00	-0.05	0.27	-0.15	-0.46	0.00	-0.10	0.17	1.00	0.00	-0.24	0.25	0.00	0.90	0.30
SL 0101-1	RSK, AURKB, PIM3	0.03	0.20	0.15	-0.23	0.03	0.15	0.01	-0.41	0.37	-0.10	0.19	-0.01	-0.30	-0.11	-0.36	0.13	-0.08	-0.03
BI-D1870	RSK1/2/3/5, PLK1, AURKB	-0.13	0.19	0.30	-0.11	0.03	-0.42	0.04	-0.35	-0.71	-0.12	-0.35	0.09	-0.16	-0.17	-0.61	0.28	0.25	0.51
GSK-650394	SGK3	-0.02	0.04	0.06	-0.06	-0.18	-0.12	-0.14	-0.20	-0.03	0.05	-0.07	-0.40	-0.50	0.33	-0.20	-0.16	0.26	-0.06
NSC-87877	SHP1/2 (PTN6/11)	0.01	-0.08	-0.04	-0.15	0.09	-0.04	0.29	0.22	0.33	-0.01	-0.42	0.12	0.02	0.02	-0.10	0.43	0.20	-0.39
GDC-0449	SMO	-0.08	0.05	-0.32	-0.32	-0.01	-0.17	-0.17	-0.23	-0.21	-0.05	0.11	-0.36	-0.15	-0.21	0.09	0.15	0.09	-0.27
Cyclopamine	SMO	-0.03	-0.45	-0.18	-1.00	0.05	0.15	0.04	0.01	0.00	-0.14	0.12	0.50	0.00	-0.37	0.00	0.00	0.80	-1.00
A-770041	SRC family	-0.08	-0.26	-0.46	-1.00	0.13	0.35	0.32	-0.27	0.00	-0.05	0.03	-1.00	0.00	-0.72	-0.07	0.00	0.50	0.00
WH-4-023	SRC family, ABL	-0.19	-0.28	-0.18	-1.00	0.12	0.38	0.20	-0.21	0.00	-0.24	-0.05	-1.00	0.00	-0.42	0.46	0.00	0.10	0.00
Bosutinib	SRC, ABL, TEC	0.05	-0.03	0.40	0.29	-0.05	0.08	0.25	-0.29	-0.21	0.07	-0.11	-0.16	-0.26	-0.10	-0.36	0.40	0.14	0.52
AZD-0530	SRC, ABL1	-0.11	-0.05	-0.32	-1.00	-0.05	0.35	0.50	-0.44	0.00	0.00	0.36	-0.50	0.00	-0.74	-0.09	0.00	0.30	0.30
BAY 61-3606	SYK	-0.09	0.20	0.28	-0.57	-0.06	-0.13	0.01	-0.33	-0.38	0.08	-0.10	-0.51	-0.09	-0.12	-0.24	-0.32	-0.16	-0.21
BX-795	TBK1, PDK1, IKK, AURKB/C	-0.35	-0.20	-0.18	0.24	-0.03	-0.59	-0.50	-0.38	-0.54	-0.19	-0.08	-0.45	-0.32	-0.39	-0.55	-0.35	0.19	0.17
Lenalidomide	TNF alpha	0.04	0.29	0.29	-0.17	0.01	0.08	-0.12	-0.33	0.18	0.02	-0.15	-0.31	0.13	-0.18	-0.30	0.15	0.11	-0.18
Camptothecin	TOP1	-0.22	0.14	0.41	-0.15	0.02	-0.03	-0.37	-0.37	0.07	-0.25	-0.31	-0.61	-0.35	-0.48	-0.30	0.15	0.24	0.27
Etoposide	TOP2	-0.11	0.22	0.26	0.06	-0.18	-0.19	-0.40	-0.34	0.15	-0.01	-0.11	-0.66	-0.06	0.05	-0.53	-0.35	0.23	-0.12
Shikonin	unknown	-0.09	0.07	0.26	0.04	-0.14	-0.19	-0.21	-0.40	-0.03	0.03	-0.10	0.15	-0.44	-0.01	-0.37	-0.09	-0.11	0.30
FH535	unknown	-0.02	-0.04	-0.13	-0.31	0.08	0.13	-0.04	-0.28	0.68	0.06	-0.04	-0.55	-0.35	0.12	-0.13	0.25	-0.06	0.00
Pazopanib	VEGFR, PDGFRA, PDGFRB, KIT	-0.20	-0.24	-0.45	-0.07	-0.11	-0.03	0.07	-0.46	0.13	0.20	-0.20	-0.24	-0.54	-0.04	-0.45	-0.01	-0.30	-0.45
AMG-706	VEGFR, RET, c-KIT, PDGFR	-0.13	0.00	0.24	0.03	0.01	0.12	-0.07	-0.39	-0.14	0.03	-0.06	0.30	0.00	-0.15	-0.16	-0.08	0.08	-0.32

681640	WEE1, CHK1	-0.06	0.10	0.26	0.17	0.16	-0.22	-0.03	-0.33	-0.61	-0.10	0.00	-0.01	-0.21	0.23	0.10	0.26	-0.03	0.19
Embelin	XIAP	-0.10	-0.03	-0.23	-0.18	0.04	-0.26	0.05	-0.19	0.22	0.09	0.05	-0.43	-0.16	-0.11	-0.34	0.33	-0.01	-0.14

Abbreviation: *Rho* = Spearman Correlation *Rho* ;

Table S6. Cohorts used for cancer-specific tumour epithelial-mesenchymal transition signature generation.

							Quality Control (% of sample pass metric)				
**SN**	**ArrayExpress/ GEO ID**	**Disease**	**Project Title**	**Number of Tumour Sample**	**Pubmed ID**	**Reference**	**Average PM Intensity**	**Kernel Density**	**GAPDH 3':5'**	**βactin 3':5'**	**Positive/ Negative Ctrl COI**
1	GSE3149	Ovarian	Duke Cohort	146	16273092	Bild AH, Yao G, Chang JT, Wang Q et al. 2006 Nature **439** 353-7	100.00	99.32	96.58	96.58	100.00
2	GSE9891	Ovarian	Expression profile of 285 ovarian tumour samples	285	18698038	Tothill RW, Tinker AV, George J, Brown R et al. 2008 Clin Cancer Res **14** 5198-208.	100.00	100.00	99.30	99.65	100.00
3	GSE2109	Ovarian	Expression Project for Oncology (expO)	214	---	---	100.00	97.66	96.26	84.11	100.00
4	TCGA	Ovarian	The Cancer Genome Atlas	406	21720365	The Cancer Genome Atlas Research Network 2011 Nature **474** 60-15.	100.00	99.75	98.77	83.00	100.00
5	Oslo Cohort	Ovarian	Oslo Cohort	26	---	---	100.00	100.00	88.46	92.31	100.00
6	GSE6008	Ovarian	Human ovarian tumours and normal ovaries	99	16452189	Hendrix ND, Wu R, Kuick R, Schwartz DR et al. 2006 Cancer Res **66** 1354-62.	100.00	98.99	96.97	98.99	100.00
7	E-MEXP-935	Ovarian	Transcription profiling of human ovarian cancer tumour samples to investigate heterogeneity	27	17367315	Jochumsen KM, Tan Q, Hølund B, Kruse TA, Mogensen O 2007 Int J Gynecol Cancer. **17** 979-85	100.00	92.59	100.00	100.00	96.30
8	GSE10971	Ovarian	Gene expression data from non-malignant fallopian tube epithelium and high grade serous carcinoma.	13	18593983	Tone AA, Begley H, Sharma M, Murphy J et al. 2008 Clin Cancer Res **14** 4067-78.	92.31	92.31	0.00	0.00	100.00
9	GSE14001	Ovarian	PAX2: A Potential Biomarker for Low Malignant Potential Ovarian tumours and Low-Grade Serous Ovarian Carcinomas	20	19525924	Tung CS, Mok SC, Tsang YT, Zu Z et al. 2009 Mod Pathol **22** 1243-50.	100.00	100.00	80.00	85.00	100.00
10	GSE14407	Ovarian	Ovarian Cancer gene expression profiling identifies the surface of the ovary as a stem cell niche	12	20040092	Bowen NJ, Walker LD, Matyunina LV, Logani S et al. 2009 BMC Med Genomics **2** 71.	100.00	100.00	0.00	0.00	100.00
11	GSE14764	Ovarian	A Prognostic Gene Expression Index in Ovarian Cancer	80	19294737	Denkert C, Budczies J, Darb-Esfahani S, Gyorffy B et al. 2009 J Pathol **218** 273-80.	100.00	98.75	100.00	100.00	100.00
12	GSE18520	Ovarian	Whole-genome oligonucleotide expression analysis of papillary serous ovarian adenocarcinomas	53	19962670	Mok SC, Bonome T, Vathipadiekal V, Bell A et al. 2009 Cancer Cell **16** 521-32.	100.00	100.00	18.87	5.66	100.00
13	GSE19352	Ovarian	Activation of phosphatidylcholine-cycle enzymes in human epithelial ovarian cancer cells	20	20179205	Iorio E, Ricci A, Bagnoli M, Pisanu ME et al. 2010 Cancer Res **70** 2126-35.	100.00	100.00	70.00	70.00	100.00
14	E-MEXP-1085	Ovarian	Transcription profiling of human ovarian tumours from the MALOVA (MALignant OVArian) cancer study	43	19823056	Jochumsen KM, Tan Q, Høgdall EV, Høgdall C et al. 2009 Int J Gynecol Cancer. **19** 1205-13	97.67	97.67	20.93	20.93	97.67
15	GSE15578	Ovarian	Expression Data from Ovarian Surface Kinome	4	19956396	Pejovic T, Pande NT, Mori M, Mhawech-Fauceglia P, et al. 2009 Transl Oncol **2** 341-9	100.00	100.00	100.00	100.00	100.00
16	GSE12172	Ovarian	Common activation of RAS_MAPK pathway in serous LMP tumours	90	19010816	Anglesio MS, Arnold JM, George J, Tinker AV et al. 2008 Mol Cancer Res **6** 1678-90.	100.00	100.00	98.89	96.67	100.00
			Total:	1538							
17	E-TABM-158	Breast	Transcription profiling of human breast cancer samples	130	17157792	Chin, K., DeVries, S., Fridlyand, J., et al. 2006 Cancer cell **10**, 529-541.	99.23	99.23	100.00	90.77	100.00
18	GSE11121	Breast	The humoral immune system has a key prognostic impact in node-negative breast cancer	200	18593943	Schmidt M, Böhm D, von Törne C, et al. 2008 Cancer Res. **68** 5405-13	99.50	99.50	94.50	96.50	98.50
19	GSE12276	Breast	Expression data from primary breast Tumours	204	19421193	Bos PD, Zhang XH, Nadal C, et al. 2009 Nature **18** 1005-9	100.00	100.00	93.63	82.35	100.00
20	GSE1456	Breast	Gene expression of breast cancer tissue in a large population-based cohort of Swedish patients	159	16280042	Pawitan Y, Bjöhle J, Amler L, et al. 2005 Breast Cancer Res **7** R953-64	100.00	100.00	87.42	94.97	99.37
21	GSE1561	Breast	EORTC 10994 clinical trial	49	15897907	Farmer P, Bonnefoi H, Becette V, et al. 2005 Oncogene **24** 4660-71	100.00	100.00	79.59	48.98	100.00
22	GSE19615	Breast	Integrated genomic and function characterization of the 8q22 gain	115	20098429	Li Y, Zou L, Li Q, et al. 2010 Nat Med **16** 214-8	100.00	100.00	91.30	96.52	100.00
23	GSE20181	Breast	Letrozole (Femara) early and late responses to treatment	176	20646288	Miller WR, Larionov A 2010 Breast Cancer Res **12** R52	96.02	96.02	18.18	7.95	100.00
24	GSE2034	Breast	Breast cancer relapse free survival	286	15721472	Wang Y, Klijn JG, Zhang Y, et al. 2005 The Lancet **365** 671-9	100.00	100.00	14.69	54.20	100.00
25	GSE21653	Breast	A gene expression signature identifies two prognostic subgroups of basal breast cancer	266	20490655	Sabatier R, Finetti P, Cervera N, et al. 2011 Breast Cancer Res **126** 407-20	100.00	100.00	88.35	93.23	98.50
26	GSE23177	Breast	Prediction of lymph node involvement in breast cancer from primary Tumour tissue using gene expression profiling and miRNAs.	116	21116709	Smeets A, Daemen A, Vanden Bempt I, et al. 2011 **129** 767-76	100.00	100.00	100.00	100.00	100.00
27	GSE23593	Breast	IntraTumour Heterogeneity and Precision of Microarray-Based Predictors of Breast Cancer Biology and Clinical Outcome	50	20368555	Barry WT, Kernagis DN, Dressman HK, et al. 2010 J Clin Oncol **28** 2198-206	100.00	100.00	100.00	100.00	100.00
28	GSE23988	Breast	Expression data from breast cancer FNA biopsies from patients (US samples)	61	21191116	Iwamoto T, Bianchini G, Booser D, et al. 2011 J Natl Cancer Inst **103** 264-72	96.72	96.72	93.44	93.44	100.00
29	GSE25066	Breast	Genomic predictor of response and survival following neoadjuvant taxane-anthracycline chemotherapy in breast cancer	508	21558518	Hatzis C, Pusztai L, Valero V, et al. 2011 JAMA **305** 1873-81	99.80	99.80	89.57	92.13	100.00
30	GSE26639	Breast	Expression Data from 226 patients of the REMAGUS02 trial	226	21631949	de Cremoux P, Valet F, Gentien D, et al. 2011 BMC Cancer **11** 215	99.56	99.56	98.23	97.79	100.00

31	GSE31519	Breast	A Clinically Relevant Gene Signature in Triple-Negative and Basal-Like Breast Cancer	67	22220191	Karn T, Pusztai L, Holtrich U, et al. 2011 PloS One **6** e28403	97.01	97.01	97.01	100.00	100.00
32	GSE3494	Breast	An expression signature for p53 in breast cancer predicts mutation status, transcriptional effects, and patient survival	251	16141321	Miller LD, Smeds J, George J, et al. 2005 **102** 13550-5	100.00	100.00	84.06	99.20	100.00
33	GSE3744	Breast	Human breast Tumour expression	47	16473279	Richardson AL, Wang ZC, De Nicolo A, et al. 2006 **9** 121-32	100.00	100.00	89.36	89.36	100.00
34	GSE4922	Breast	Genetic Reclassification of Histologic Grade Delineates New Clinical Subtypes of Breast Cancer	40	17079448	Ivshina AV, George J, Senko O, et al. 2006 Cancer Res **66** 10292-301	100.00	100.00	75.00	100.00	100.00
35	GSE5327	Breast	Breast cancer relapse free survival and lung metastasis free survival	58	17420468	Minn AJ, Gupta GP, Padua D, et al. 2007 **104** 6740-5	98.28	98.28	62.07	89.66	100.00
36	GSE5460	Breast	Predicting Features of Breast Cancer with Gene Expression Patterns	127	18297396	Lu X, Lu X, Wang ZC, et al. 2008 Breast Cancer Res Treat **108** 191-201	100.00	100.00	92.13	97.64	100.00
37	GSE5764	Breast	Analysis of microdissected invasive lobular and ductal breast carcinomas in relation to normal ductal and lobular cells	10	17389037	Turashvili G, Bouchal J, Baumforth K, et al. 2007 BMC Cancer **7** 55	90.00	90.00	10.00	40.00	100.00
38	GSE6532	Breast	Definition of clinically distinct molecular subtypes in estrogen receptor positive breast carcinomas using genomic grade	414	17401012	Loi S, Haibe-Kains B, Desmedt C, et al. 2007 J Clin Oncol **25** 1239-46	100.00	100.00	89.86	93.00	98.79
39	GSE6596	Breast	Comparison of gene expression data from human and mouse breast cancers: Identification of conserved breast Tumour genes	24	17410534	Klein A, Wessel R, Graessmann M, et al. 2007 Int J Cancer **121** 683-8	79.17	79.17	66.67	66.67	100.00
40	GSE7390	Breast	Strong Time Dependence of the 76-Gene Prognostic Signature	198	17545524	Desmedt C, Piette F, Loi S, et al. 2007 Clin Cancer Res **13** 3207-14	100.00	100.00	98.99	100.00	99.49
41	GSE9195	Breast	Predicting prognosis using molecular profiling in estrogen receptor-positive breast cancer treated with tamoxifen	77	18498629	Loi S, Haibe-Kains B, Desmedt C, et al. 2008 BMC Genomics **9** 239	100.00	100.00	89.61	68.83	100.00
42	HESS	Breast	Pharmacogenomic predictor of sensitivity to preoperative chemotherapy with paclitaxel and fluorouracil, doxorubicin, and cyclophosphamide in breast cancer.	133	16896004	Hess KR, Anderson K, Symmans WF, et al. 2006 J Clin Oncol **24** 4236-44	100.00	100.00	89.47	93.98	100.00
			Total:	3992							
43	GSE31684	Bladder	Combination of a novel gene expression signature with a clinical nomogram improves the prediction of survival in high-risk bladder cancer	93	22228636	Riester M, Taylor JM, Feifer A, et al. 2012 Clin Cancer Res **18** 1323-33	100.00	100.00	89.25	89.25	100.00
44	GSE5287	Bladder	Prediction of response and survival following chemotherapy in patients with advanced bladder cancer	30	17671123	Als AB, Dyrskjøt L, von der Maase H, et al. 2007 Clin Cancer Res **13** 4407-14	100.00	100.00	83.33	100.00	96.67
45	GSE7476	Bladder	Analysis of clinical bladder cancer classification according to microarray expression profiles	9	19539325	Mengual L, Burset M, Ars E, et al. 2009 J Urol **182** 741-8	100.00	100.00	100.00	100.00	100.00
			Total:	132							
46	GSE10961	Colon	Gene expression profiling of liver metastases from colorectal cancer	18	18827815	Pantaleo MA, Astolfi A, Nannini M, et al. 2008 Br J Cancer **99** 1729-34	94.44	94.44	66.67	66.67	100.00
47	GSE12945	Colon	Expression data from colorectal cancers	62	19399471	Staub E, Groene J, Heinze M, et al. 2009 J Mol Med (Berl) **87** 633-44	100.00	100.00	1.61	0.00	98.39
48	GSE13067	Colon	Expression data from primary colorectal cancers	74	19088021	Jorissen RN, Lipton L, Gibbs P, et al. 2008 Clin Cancer Res **14** 8061-9	100.00	100.00	100.00	100.00	100.00
49	GSE13294	Colon	Expression data from primary colorectal cancers	155	19088021	Jorissen RN, Lipton L, Gibbs P, et al. 2008 Clin Cancer Res **14** 8061-9	99.35	99.35	84.52	93.55	100.00
50	GSE14333	Colon	Expression data from 290 primary colorectal cancers	290	19996206	Jorissen RN, Gibbs P, Christie M, et al. 2009 Clin Cancer Res **15** 7642-51	100.00	100.00	100.00	100.00	100.00
51	GSE15960	Colon	Expression data from human colonic epithelial cells normal (N), adenoma (AD) or colorectal cancer (CRC) tissues	12	20087348	Galamb O, Spisák S, Sipos F, et al. 2010 Br J Cancer **102** 765-73	100.00	100.00	0.00	0.00	100.00
52	GSE17536	Colon	Metastasis Gene Expression Profile Predicts Recurrence and Death in Colon Cancer Patients (Moffitt Samples)	177	19914252	Smith JJ, Deane NG, Wu F, et al. 2010 Gastroenterology **138** 958-68	98.87	98.87	100.00	100.00	100.00
53	GSE17537	Colon	Metastasis Gene Expression Profile Predicts Recurrence and Death in Colon Cancer Patients (VMC Samples)	55	22115830	Freeman TJ, Smith JJ, Chen X, et al. 2012 Gastroenterology **142** 562-71	100.00	100.00	96.36	94.55	100.00
54	GSE18088	Colon	Correlation of molecular profiles and clinical outcome of stage UICC II colon cancer patients	53	21465190	Gröne J, Lenze D, Jurinovic V, et al. 2011 Int J Colorectal Dis **26** 847-58	100.00	100.00	100.00	100.00	98.11
55	GSE18105	Colon	Stage II and stage III colorectal cancer	77	20162577	Matsuyama T, Ishikawa T, Mogushi K, et al. 2010 Int J Cancer **127** 2292-9	100.00	100.00	55.84	7.79	100.00
56	GSE20916	Colon	Modeling oncogenic signaling in colon Tumours by multidirectional analyses of microarray data	101	20957034	Skrzypczak M, Goryca K, Rubel T, et al. 2010 PLoS One **5** e13091	100.00	100.00	62.38	60.40	100.00
57	GSE23878	Colon	Genome Wide Expression Analysis of Middle Eastern Colorectal Cancer Reveals FOXM1 as a Novel Target for Cancer Therapy	35	21281787	Uddin S, Ahmed M, Hussain A, et al. Am J Pathol **178** 537-47	100.00	97.14	88.57	80.00	97.14
58	GSE24514	Colon	Expression data from human MSI colorectal cancer and normal colonic mucosa	34	21544814	Alhopuro P, Sammalkorpi H, Niittymäki I, et al. 2012 Int J Cancer **130** 1558-66	100.00	100.00	100.00	100.00	100.00
59	GSE26682	Colon	MRE11 Deficiency Increases Sensitivity to Poly(ADP-ribose) Polymerase Inhibition in Microsatellite Unstable Colorectal Cancers.	331	21300766	Vilar E, Bartnik CM, Stenzel SL, et al. 2011 Cancer Res **71** 2632-42	95.77	95.77	94.56	98.79	100.00
60	GSE31595	Colon	Gene Expression Profiles in Stage II and III Colon Cancer. Application of a 128-gene signature	37	22710688	Thorsteinsson M, Kirkeby LT, Hansen R, et al. 2012 Int J Colorectal Dis. **27** 1579-86	100.00	100.00	100.00	100.00	100.00

61	GSE33113	Colon	AMC colon cancer AJCCII	90	22056143	de Sousa E Melo F, Colak S, Buikhuisen J, et al. 2011 Cell Stem Cell **9** 476-85	100.00	100.00	100.00	100.00	100.00
62	GSE4045	Colon	Classification of serrated colorectal Tumours	37	16819509	Laiho P, Kokko A, Vanharanta S, et al. 2007 Oncogene **26** 312-20	100.00	100.00	86.49	100.00	100.00
63	GSE5851	Colon	Phase II exploratory pharmacogenomics study of cetuximab monotherapy in patients with advanced metastatic CRC	80	17664471	Khambata-Ford S, Garrett CR, Meropol NJ, et al. 2007 J Clin Oncol **25** 3230-7	100.00	100.00	100.00	100.00	100.00
64	GSE8671	Colon	Transcriptome profile of human colorectal adenomas.	32	18171984	Sabates-Bellver J, Van der Flier LG, de Palo M, et al. 2007 Mol Cancer Res **5** 1263-75	96.88	96.88	100.00	100.00	100.00
65	GSE9348	Colon	Expression data from healthy controls and early stage CRC patient's Tumour	70	20143136	Hong Y, Downey T, Eu KW, et al. 2010 Clin Exp Metastasis **27** 83-90	100.00	100.00	95.71	95.71	100.00
			Total:	1820							

66	GSE15460	Gastric	Oncogenic Pathway Combinations Predict Clinical Prognosis in Gastric Cancer	231	19798449	Ooi CH, Ivanova T, Wu J, et al. 2009 PLoS Genet **5** e1000676	97.40	97.40	95.67	97.40	89.61
			Total:	231							

67	GSE10245	Lung	Gene expression differences between adenocarcinoma and squamous cell carcinoma in human NSCLC	58	18486272	Kuner R, Muley T, Meister M, et al. 2009 Lung Cancer **63** 32-8	93.10	93.10	100.00	100.00	100.00
68	GSE10445	Lung	MERLION LUNG CANCER STUDY	72	19176396	Broët P, Camilleri-Broët S, Zhang S, et al. 2009 Cancer Res **69** 1055-62	86.11	86.11	48.61	52.78	100.00
69	GSE12667	Lung	Discovery of somatic mutations in lung adenocarcinomas	75	18948947	Ding L, Getz G, Wheeler DA, et al. 2008 Nature **455** 1069-75	98.67	98.67	94.67	98.67	98.67
70	GSE14814	Lung	Prognostic and Predictive Gene Signature for Adjuvant Chemotherapy in Resected Non-Small Cell Lung Cancer	90	20823422	Zhu CQ, Ding K, Strumpf D, et al. 2010 J Clin Oncol **28** 4417-24	94.44	94.44	95.56	98.89	100.00
71	GSE18842	Lung	Gene expression analysis of human lung cancer and control samples	46	20878980	Sanchez-Palencia A, Gomez-Morales M, Gomez-Capilla JA, et al. 2011 Int J Cancer **129** 355-64	95.65	95.65	58.70	93.48	45.65
72	GSE27716	Lung	Expression data from Columbia Lung Adenocarcinoma Human Tumour Cells	40	21911454	Borczuk AC, Sole M, Lu P, et al. 2011 Cancer Res **71** 6665-75	100.00	100.00	0.00	0.00	100.00
73	GSE28582	Lung	Gene Copy Number Aberrations are Associated with Survival in Histological Subgroups of Non-Small Cell Lung Cancer	100	22011649	Micke P, Edlund K, Holmberg L, et al. 2011 J Thorac Oncol **6** 18833-40	100.00	100.00	99.00	100.00	100.00
			Total:	481							

Abbreviation: GEO, Gene Expression Omnibus; PM, Perfect Match; COI, Centre of Intensity

Table S7. Collections used for cancer-specific cell line epithelial-mesenchymal transition signature generation.

SN	ArrayExpress/ GEO ID	Disease	Project Title	Number of Cell Lines	Pubmed ID	Reference	Quality Control (% of sample pass metric)				
							Average PM Intensity	Kernel Density	GAPDH 3':5'	βactin 3':5'	Positive/Negative Ctrl COI
1	GSE29175	Ovarian	Expression data from ovarian cancer cell lines	38	20062075	Yamaguchi K, Mandai M, Oura T, et al. 2010 Oncogene **29** 1741-52	97.37	97.37	100.00	100.00	97.37
2	GSE34615	Ovarian	Ovarian cancer cell lines [Baseline profiling]	41	---	---	100.00	100.00	87.80	82.93	100.00
3	GSE28724	Ovarian	Expression data from cultured human ovarian carcinoma cell lines	34	23666744	Tan TZ, Miow QH, Huang RY, et al. 2013 EMBO Mol Med	100.00	100.00	100.00	70.59	100.00
4	E-TABM-254	Ovarian	Transcription profiling of human ovarian cancer cell lines	30	17908964	Guan Y, Kuo WL, Stilwell JL, et al. 2007 Clincal Cancer Res. **13** 5745-55	100.00	100.00	100.00	100.00	100.00
			Total:	143							
5	GSE15026	Breast	Expression data from breast cancer cell lines with various colony-forming ability	30	19483725	Mori S, Chang JT, Andrechek ER, et al. 2009 Oncogene **28** 2796-805	100.00	100.00	100.00	100.00	100.00
6	E-TABM-157	Breast	Transcription profiling of 51 human breast cancer cell lines	51	19783824	Pavon-Eternod M, Gomes S, Geslain R, et al. 2009 Nucleic Acids Res. **37** 7268-80	96.08	96.08	100.00	100.00	98.04
			Total:	81							
7	GSE5845	Bladder	Bladder Cancer 40 Cell Lines	40	17666531	Lee JK, Havaleshko DM, Cho H, et al. 2007 Proc Natl Sci USA **104** 13086-91	---	---	---	---	---
			Total:	40							
7	GSE10843	Colorectal	mRNA Cancer Cell Line Profiles	48#	21673316	Stinson S, Lackner MR, Adai AT, et al. 2011 Sci Signal **4** ra41	100.00	100.00	100.00	100.00	97.92
8	GSE34211	Colorectal	Pfizer Molecular Profile Data for Cell Lines	14#	22222631	Hook KE, Garza SJ, Lira ME, et al. 2012 Mol Cancer Ther **11** 710-9	100.00	100.00	100.00	100.00	100.00
			Total:	62							
9	GSE15460	Gastric	Oncogenic Pathway Combinations Predict Clinical Prognosis in Gastric Cancer	70	19798449	Ooi CH, Ivanova T, Wu J, et al. 2009 PLoS Genet **5** e1000676	97.14	97.14	100.00	100.00	100.00
			Total:	70							
10	GSE4127	Lung	Anti-Tumour Activity of Histone Deacetylase Inhibitors in Non-Small Cell Lung Cancer Cells	29	18606719	Miyanaga A, Gemma A, Noro R, et al. 2008 Mol Cancer Ther **7** 1923-30	100.00	100.00	100.00	100.00	100.00
11	GSE10843	Lung	mRNA Cancer Cell Line Profiles	91#	21673316	Stinson S, Lackner MR, Adai AT, et al. 2011 Sci Signal **4** ra41	100.00	100.00	100.00	100.00	100.00
12	GSE34211	Lung	Pfizer Molecular Profile Data for Cell Lines	56#	22222631	Hook KE, Garza SJ, Lira ME, et al. 2012 Mol Cancer Ther **11** 710-9	96.43	96.43	100.00	100.00	96.43
13	GSE31625	Lung	Gene Expression Patterns that Predict Sensitivity to Epidermal Growth Factor Receptor Tyrosine Kinase Inhibitors in Lung Cancer Cell Lines and Human Lung Tumours	48	17096850	Balko JM, Potti A, Saunders C, et al. 2006 BMC Genomics **7** 289	100.00	100.00	91.67	93.75	100.00
14	GSE14925	Lung	Expression data from Non small cell lung cancer cell lines	54	19451690	Sos ML, Michel K, Zander T, et al. 2009 J Clin Invest. **119** 1727-40	100.00	100.00	66.67	74.07	72.72
15	GSE4824	Lung	Analysis of lung cancer cell lines	85	18391978	Lockwood WW, Chari R, Coe BP, et al. 2008 Oncogene **27** 4615-24	98.82	98.82	77.65	82.35	65.88
			Total:	363							

#Only subset of the series were used

Table S8. Dataset used for validation generic epithelial-mesenchymal transition signature.

SN	ArrayExpress/ GEO ID	Disease	Project Title	Number of Sample	Pubmed ID	Reference
1	GSE9691	Breast	Analysis of the effects of loss of E-cadherin and cell adhesion on human mammary epithelial cells	12	18483246	Onder TT, Gupta PB, Mani SA, et al. 2008 Cancer Res **68** 3645-54
2	GSE24202	Breast	Expression data from genetically modified HMLE human mammary epithelial cells	15	20713713	Taube JH, Herschkowitz JI, Komurov K, et al. 2010 Proc Natl Acad Sci USA **107** 15449-54
3	E-MEXP-3547	Breast	Transcription profiling by array of MDA-MB-231 cells with HMGA2 knock-down	4	---	---
4	GSE28569	Breast	Down regulation of CK2-Beta is sufficient to drive epithelial-to-mesenchymal transition through Snail 1 induction	8	22562247	Deshiere A, Duchemin-Pelletier E, Spreux E, et al. 2013 **32** 1373-83
5	GSE33146	Breast	Expression data from DKAT breast cancer cell line pre- and post-EMT	6	23049838	D'Amato NC, Ostrander JH, Bowie ML, et al. 2012 PLoS One **7** e45684
6	GSE39356	Breast	MiR-374a Promotes Epithelial-Mesenchymal Transition (EMT) and Metastasis of Breast Cancer (mRNA dataset)	4	23321667	Cai J, Guan H, Fang L, et al. 2013 J Clin Invest. **123** 566-79
7	GSE36081	Breast	Effect of GRHL2 in HMLE+Twist-ER+4-OHT cells	6	22379025	Cieply B, Riley P 4th, Pifer PM, et al. 2012 Cancer Res **72** 2440-53
8	GSE27424	Esophageal	NOTCH3 knockdown in non-transformed human oesophageal cells	12	21890822	Ohashi S, Natsuizaka M, Naganuma S, et al. Cancer Res **71** 6836-47
9	E-MTAB-114	foreskin	Transcription profiling of human foreskin keratinocytes expressing E6 or E7 compared to normal keratinocytes reveals HPV16 E7 induces EMT	6	19552933	Hellner K, Mar J, Fang F, et al. 2009 Virology **391** 57-63
10	GSE16194	Lung	Expression data from Snail over-expressing non-small cell lung cancer cell lines	6	19887480	Yanagawa J, Walser TC, Zhu LX, et al. 2009 Clin Cancer Res **15** 6820-9
11	GSE5457	Lung	Retinoic Acids Exposure Alters TGFbeta1 -Induced Epithelial Mesenchymal Transition via Wnt5b expression.	8	18621908	Malizia AP, Keating DT, Smith SM, et al. 2008 Am J Physiol Lung Cell Mol Physiol. **295** L451-60
12	GSE23952	Pancreas	Expression data from TGF-beta treated Panc-1 pancreatic adenocarcinoma cell line	6	20885998	Maupin KA, Sinha A, Eugster E, et al. 2010 PLoS One **5** e13002
13	GSE6891	Acute Myeloid Leukaemia	Acute myeloid Leukaemia samples of samples =< 60yrs on HG-U133 plus 2	460	18838472	Verhaak RG, Wouters BJ, Erpelinck CA, et al. 2009 Haematologica **94** 131-4
14	GSE14468	Acute Myeloid Leukaemia	Gene expression profiling of CEBPA double and single mutant and CEBPA wild type AML.	65	19171880	Wouters BJ, Löwenberg B, Erpelinck-Verschueren CA, et al. 2009 Blood **113** 3088-91
15	GSE12417	Acute Myeloid Leukaemia	Prognostic gene signature for normal karyotype AML	242	18716133	Metzeler KH, Hummel M, Bloomfield CD, et al. 2008 Blood **112** 4193-201
16	GSE10358	Acute Myeloid Leukaemia	Discovery and validation of expression data for the Genomics of Acute Myeloid Leukaemia Program at Washington University.	275	18270328	Tomasson MH, Xiang Z, Walgren R, et al. 2008 Blood **111** 4797-808
17	GSE16015	Acute Myeloid Leukaemia	AML with mutated NPM1 carrying a normal or aberrant karyotype show overlapping features	107	19429869	Haferlach C, Mecucci C, Schnittger S, et al. 2009 Blood **114** 3024-32
18	GSE24080	Multiple Myeloma	MAQC-II Project: Multiple myeloma (MM) data set	559	20064235	Popovici V, Chen W, Gallas BG, et al. 2010 Breast Cancer Res **12** R5
19	GSE2113	Multiple Myeloma	Plasma cell dyscrasias expression profiles associated with distinct IGH translocations in multiple myeloma	39	15735737	Mattioli M, Agnelli L, Fabris S, et al. 2005 Oncogene **24** 2461-73
20	GSE26760	Multiple Myeloma	MMRC expression reference collection	304	21430775	Chapman MA, Lawrence MS, Keats JJ, et al. Nature **471** 467-72
21	GSE21349	Multiple Myeloma	A compendium of myeloma associated chromosomal copy number abnormalities and their prognostic value	258	20616218	Walker BA, Leone PE, Chiecchio L, et al. 2010 Blood **116** e56-65
22	GSE19784	Multiple Myeloma	Gene expression profiling of multiple myeloma patients included in the HOVON65/GMMG-HD4 trial	320	20574050	Broyl A, Hose D, Lokhorst H, et al. 2010 Blood **116** 2543-53
23	GSE6401	Multiple Myeloma	Up-regulation of translational machinery and distinct genetic subgroups characterize hyperdiploidy in multiple myeloma	102	17367409	Agnelli L, Fabris S, Bicciato S, et al. 2007 Br J Haematol **136** 565-73
24	GSE6477	Multiple Myeloma	Expression data from different stages of plasma cell neoplasm	125	17409404	Chng WJ, Kumar S, Vanwier S, et al. 2007 Cancer Res **67** 2982-9
25	GSE13591	Multiple Myeloma	Integrated genomics approach to detect allelic imbalances in multiple myeloma	133	19396863	Agnelli L, Mosca L, Fabris S, et al. 2009 Genes Chromosomes Cancer **48** 603-14
26	GSE17306	Multiple Myeloma	Plasma cells from 52 patients with multiple myeloma: mRNA and miRNA expression profiling	51	20385818	Zhou Y, Chen L, Barlogie B, et al. 2010 Proc Natl Acad Sci USA **107** 7904-9
27	GSE17498	Multiple Myeloma	Identification of MicroRNA Expression Patterns and a MicroRNAs/mRNA Regulatory Network in Multiple Myeloma	38	19846888	Lionetti M, Biasiolo M, Agnelli L, et al. 2009 Blood **114** e20-6
28	GSE33382	Osteosarcoma	Genome-wide gene expression analysis of high-grade osteosarcoma	84	22454324	Kuijjer ML, Rydbeck H, Kresse SH, et al. 2012 Genes Chromosomes Cancer **51** 696-706
29	TCGA.GBM	Glioblastoma	Integrated genomic analysis identifies clinically relevant subtypes of glioblastoma characterized by abnormalities in PDGFRA, IDH1, EGFR, and NF1.	411	20129251	Verhaak RG, Hoadley KA, Purdom E, et al. 2010 Cancer Cell **17** 98-110
30	GSE19274	Neuroblastoma	Expression profiling in Neuroblastoma Primary Tumours and Cell lines	100	21289283	Cole KA, Huggins J, Laquaglia M, et al. 2011 Proc Natl Acad Sci USA **108** 3336-41
31	GSE3218	Germ Cell Tumour	Expression Profiling of Adult Male Germ Cell Tumours	100	16424014	Korkola JE, Houldsworth J, Chadalavada RS, et al. 2006 Cancer Res **66** 820-7
32	GSE19949	Renal	Integrative genome-wide expression profiling identifies three distinct molecular subgroups of renal cell carcinoma with different patient outcome	113	22824167	Beleut M, Zimmermann P, Baudis M, et al. 2012 BMC Cancer **12** 310
33	GSE36895	Renal	Molecular Genetic Classification of clear-cell Renal Cell Carcinoma (ccRCC) based on the Gene Expression Profiling of Tumours and Tumourgrafts deficient for BAP1 or PBRM1	29	22683710	Peña-Llopis S, Vega-Rubín-de-Celis S, Liao A, et al. 2012 Nat Genet **44** 751-9
34	GSE26574	Renal	An antioxidant response phenotype is shared between hereditary and sporadic type 2 papillary renal cell carcinoma	57	22014576	Ooi A, Wong JC, Petillo D, et al. 2011 Cancer Cell **20** 511-23
35	GSE22541	Renal	Expression data from pulmonary metastases and primary Tumours of clear-cell renal cell carcinoma (ccRCC) with different disease-free survivals	24	23125027	Toma MI, Wuttig D, Kaiser S, et al. 2013 Genes Chromosomes Cancer **52** 265-73
36	GSE23629	Renal	Expression data from RCC paired Tumours to study metastasis progression	16	20952505	López-Lago MA, Thodima VJ, Guttapalli A, et al. 2010 Cancer Res **70** 9682-92
37	GSE6344	Renal	Gene expression in Stage 1,2 Normal and Tumour kidney cancer	10	17699851	Gumz ML, Zou H, Kreinest PA, et al. 2007 Clin Cancer Res **13** 4740-9
38	GSE781	Renal	Normal and Renal Cell Carcinoma Kidney Tissue, Human	12	14641932	Lenburg ME, Liou LS, Gerry NP, et al. 2003 BMC Cancer **3** 31
39	GSE14520	Liver	Gene expression data of human hepatocellular carcinoma (HCC)	247	21159642	Roessler S, Jia HL, Budhu A, et al. 2010 Cancer Res **70** 10202-12
40	GSE36924	Pancreatic Ductal Adenocarcinoma	ICGC Pancreas: Genomic analysis reveals roles for chromatin modification and axon guidance in pancreatic cancer	91	22699621	Pérez-Mancera PA, Rust AG, van der Weyden L, et al. 2012 Nature **486** 266-70
41	GSE17951	Prostate	Gene expression analysis of prostate cancer samples using Affymetrix U133Plus2 array	154	21459804	Jia Z, Wang Y, Sawyers A, et al. 2011 Cancer Res **71** 2476-87
42	GSE6956	Prostate	Tumour Immunobiological Differences in Prostate Cancer between African-American and European-American Men	69	18245496	Wallace TA, Prueitt RL, Yi M, et al. 2008 Cancer Res **68** 927-36
43	GSE8218	Prostate	Gene expression data from prostate cancer samples	144	20663908	Wang Y, Xia XQ, Jia Z, et al. 2010 Cancer Res **70** 6448-55

44	GSE25136	Prostate	Optimizing molecular signatures for prostate cancer recurrence	79	19343730	Sun Y, Goodison S. 2009 Prostate **69** 1119-27
45	GSE28403	Prostate	Integrative genomic, transcriptomic and RNAi analysis indicates a potential oncogenic role for FAM110B in castration-resistant prostate cancer.	13	21919029	Vainio P, Wolf M, Edgren H, et al. 2012 Prostate **72** 789-802
46	GSE32269	Prostate	Expression data for primary localized prostate cancer versus castration-resistant bone metastatic prostate cancer	51	---	---
47	GSE46517	Malignant Melanoma	Human melanoma samples comparing nevi and primary and metastatic melanoma	104	20520718	Kabbarah O, Nogueira C, Feng B, et al. 2010 PLoS One **5** e10770

Table S9. Published or curated Epithelial-Mesenchymal Transition (EMT) genesets used for EMT markers correlation.

Index	Geneset	Source	Description	Reference	PMID
1	Alonso Metastasis EMT	MSigDB v4.0	http://www.broadinstitute.org/gsea/msigdb/cards/ALONSO_METASTASIS_EMT_UP<br>http://www.broadinstitute.org/gsea/msigdb/cards/ALONSO_METASTASIS_EMT_DN	Alonso SR, Tracey L, Ortiz P, et al. 2007 Cancer Res. **67** 3450-60	1740945
2	EMT-PATHWAY	Carretero et al.	EMT-related genes from literature	Carretero J, Shimamura T, Rikova K, et al. 2010 Cancer Cell **17** 547-59	20541700
3	EMT	Carretero et al.	Upregulated genes in TGFβ-induced EMT	Carretero J, Shimamura T, Rikova K, et al. 2010 Cancer Cell **17** 547-59	20541700
4	Mes_sg/Epi_sg	Curation	Mesenchymal and Epithelial markers curated from Lee et al.. The gene list was refined by removing Keratins that are predominantly basal and non-consensual according to Schweizer et al., and Moll etl al.	Lee JM, Dedhar S, Kalluri R, et al. 2006 J Cell Biol. **172** 973-81<br>Schweizer J, Bowden PE, Coulombe PA, et al. 2006 J Cell Biol. **174** 169-74<br>Moll R, Divo M, Langbein L 2008 Histochem Cell Biol. **129** 705-33	16567498<br>18461349<br>16831889
5	Epithelial to Mesenchymal Transition	MSigDB v4.0	http://www.broadinstitute.org/gsea/msigdb/cards/EPITHELIAL_TO_MESENCHYMAL_TRANSITION	Gene Ontology GO:0001837	---
6	Sarrio EMT	MSigDB v4.0	http://www.broadinstitute.org/gsea/msigdb/cards/SARRIO_EPITHELIAL_MESENCHYMAL_TRANSITION_UP<br>http://www.broadinstitute.org/gsea/msigdb/cards/SARRIO_EPITHELIAL_MESENCHYMAL_TRANSITION_DN	Sarrió D, Rodriguez-Pinilla SM, Hardisson D, et al. 2008 Cancer Res. **68** 989-97	18281472

Abbreviation: EMT, Epithelial-Mesenchymal Transition; MSigDB, Molecular signature database.
