# Supplementary material for: Epithelial-mesenchymal transition spectrum quantification and its efficacy in deciphering survival and drug responses of cancer patients
Source: EMBO Mol Med. 2014 Sep 11;6(10):1279–93. doi: 10.15252/emmm.201404208 (PMC4287932; doi:10.15252/emmm.201404208)
Supplement: Supplementary file 3 [file emmm0006-1279-sd3.pdf]

Epithelial-Mesenchymal Transition Spectrum Quantification and Its Efficacy in Deciphering Survival and Drug Responses of Cancer Patients

Tuan Zea Tan, Qing Hao Miow, Yoshio Miki, Tetsuo Noda, Seiichi Mori, Ruby Yun-Ju Huang and Jean Paul Thiery

Corresponding author: Jean Paul Thiery, Institute of Molecular and Cell Biology

Review timeline:

Submission date:	28 April 2014
Editorial Decision:	12 June 2014
Revision received:	30 July 2014
Editorial Decision:	04 August 2014
Revision received:	07 August 2014
Accepted:	08 August 2014

Transaction Report:

(Note: With the exception of the correction of typographical or spelling errors that could be a source of ambiguity, letters and reports are not edited. The original formatting of letters and referee reports may not be reflected in this compilation.)

Editor: Roberto Buccione

1st Editorial Decision 12 June 2014

Thank you for the submission of your manuscript to EMBO Molecular Medicine. We have now received comments from the Reviewers whom we asked to evaluate your manuscript.

We apologise that it has taken more time than we would have liked to return a decision, but unfortunately the Reviewers delivered their evaluations with some delay. I trust that the inevitable frustration due to the delay will be somewhat tempered by the fact that the Reviewers are all quite supportive and, in my opinion, offer valuable suggestions to improve your manuscript.

Reviewer 1 specifically acknowledges the significant usefulness of the scoring tool and in this respect would like you to provide an algorithm to allow others to readily apply the approach to their favourite system. S/he also would like you to verify whether the signatures perform well with a reduced set of genes. Finally, this Reviewer suggests that a parallel should be drawn with previously reported cancer stemness signatures.

Reviewer 2 raises interesting points with respect to pancreatic cancer (with reference to the alleged negative correlation between EMT and survival) and also suggests further analysis comparing mesenchymal vs. epithelial cancer types. These points appear very interesting and while to specifically address them might not be required, I believe that the information gained would be potentially very valuable and would increase the impact of your work. Reviewer 2 notes that some discrepancies between certain clinical samples and theoretically corresponding cell lines were not discussed. S/he also challenges the contention that EMT reversal leads to less metastases, mentions other points of interest and cautions against overstating the clinical applications of your tool.

Reviewer 3 would like more information on how the EMT signatures other than the breast cancer one were derived and validated. S/he also suggests verifying EMT status after patient stratification based on different subtypes of a given cancer. This Reviewer, similarly to Reviewer 2, also mentions the growing evidence that EMT reversal actually increases metastatic colonisation. Finally, Reviewer 3 suggests that overall and disease-free survival as well should be considered in your evaluations.

In conclusion, while publication of the paper cannot be considered at this stage, we would be pleased to consider a suitably revised submission, provided that the Reviewers' concerns are addressed as outlined above.

I look forward to receiving you revised manuscript as soon as possible.

***** Reviewer's comments *****

Referee #1 (Comments on Novelty/Model System):

The presented study provides a potentially useful tool to evaluate and objectify EMT signatures in primary cancers as well as cancer-derived tumor cells. Most importantly, it would substantially increase the impact of the study if the authors provide an easy to handle on line tool allowing researchers to rapidly assess EMT signatures in their biological samples of interest or primary patient material. This would also increase the impact of the study and improve signatures to be used in evaluating therapy regimes.

Referee #1 (Remarks):

Tan et al. developed a method to quantify the EMT status of tumors and tumor-derived cell lines by developing a generic EMT gene expression signature. Based on transcriptomics of ovarian, breast, bladder, lung, colorectal and gastric cancers Tan et al. defined a gene expression signature that discriminates between epithelial (low, negative EMT score), mesenchymal (high, positive EMT score), or intermediate phenotypes, which display a certain degree of plasticity. Consistent with the hypothesis that this generic EMT signature monitors the cellular phenotype, functional studies demonstrated increased EMT scores upon e.g. knockdown of CDH1 or overexpression of TWIST in epithelial cancer cells. By 'EMT scoring' clinical samples and cell lines from various cancers, the authors identified cancer type-specific EMT states with e.g. predominant epithelial phenotypes in colorectal cancers. Most strikingly, however, the correlation of primary signatures with those observed in cancer-derived cells revealed a striking correlation and appeared barely biased by stromal content in primary material. In contrast to previous suggestions, poor patient overall (OS) and disease-free survival DFS were not exclusively associated with mesenchymal, but also with the epithelial cancer phenotypes. Moreover, by correlating the EMT score with chemotherapeutic responses of cancer cell lines Tan et al. suggest that the EMT-score indicates prevalence in the chemo-sensitivity of certain tumors instead of a general resistance or reduced sensitivity of MC-like tumors. This suggestion was further evaluated by analyzing Paclitaxel sensitivity of ovarian cancer patients. Supporting the hypothesis of phenotype-specific responses, Paclitaxel increased OS and DFS of patients suffering from mesenchymal ovarian carcinomas, but decreased the prognosis for patients suffering from epithelial ovarian carcinomas.
The manuscript presents a highly interesting tool, that could substantially contribute to objectifying the evaluation of EMT phenotypes in vitro as well as malignancies. However, in its present form the scoring remains difficult to apply, involves too many genes whereas other potential markers are not considered and lacks an option to include the estimation and correlation with stemness phenotypes.

Major aspects to be addressed:
1) The authors should at least provide a easy to use algorithm allowing other researchers to evaluate the EMT-status of their biological system of choice by gene expression signatures derived by microarrays.
2) The authors should evaluate if their signatures performs equally well with a reduced set of genes analyzed. This would potentially allow evaluating EMT signatures based on smaller qRT-PCR arrays. At least the authors should identify the 'best performing' genes.

3) Along these lines it remains elusive why the authors did not include miRNAs as additional markers, since these were shown to be substantially deregulated in various cancer according to the EMT-status, for instance the miR-200 family.
4) The authors should attempt to assess the expression of putative CSC-markers reported for various cancers and try to correlate this with their EMT signature. In addition the authors have to correlate their signature with previously reported stemness signatures (e.g. Ben-Porath et al., 2008.)

Minor aspects:

1) Wherefrom were the data derived used for analyses in Figure E1? Where are the references? The same applies for Figure 1B, C. The data processing and experimental procedures are not or barely described in the methods section or in the figure legend.
2) Figure E2B should be included in the main figure section. I would prefer one main figure together with Figure 3B. Accordingly, Figure 3A should be one main figure.

Referee #2 (Comments on Novelty/Model System):

The reviewers wish to state that they are not able to evaluate in detail the soundness of the bioinformatics/statistics in the manuscript as they are not experts in that area. They were, however able to evaluate the importance and context of the question addressed, novelty and the biological background.

Referee #2 (Remarks):

The authors provide a universal EMT-signature that can be applied to several cancer types. They show that this tool allows classification of tumor samples or cell lines due to their epithelial or mesenchymal genetic profile. In doing so, the authors very well appreciate the limitations of in vitro cell line models and distinguish between gene signatures of primary tumors and cell lines. In the end, the generated tumor-specific EMT-signature is correlated to clinical outcome. The authors address one of the most pressing questions in the field, i.e., is there a general EMT-signature and how does it affect clinical behaviour in different entities. They do so in an unbiased and careful manner, thereby putting out a tool (the generic EMT-signature) that should be of broad interest and utility to a lot of researchers from different fields of inquiry.

A few minor issues might be of interest to address.

For the creation of the generic, cancer-specific EMT-signature, the authors use gene sets of different tissues (bladder, breast, colorectal, gastric, lung and ovarian) (Figure 2). Given the implications of EMT in pancreatic cancer (PC) and the emerging evidence for a negative correlation between EMT and the survival rates of patients with PC (Ref. 1-3), inclusion of PC-specific EMT-signatures should be taken into consideration.
In Figure 3A, the generic EMT score is applied to several cancer entities, including data belonging to primary tumors and cell lines. Although many clinical samples and cell lines of the same cancer type overlap in their Epi/Mes classification, there are also cases of discrepancy (bladder, ovarian, lung and prostate carcinomas). Unfortunately, the authors do not discuss these differences. One possibility could be that the EMT-score for cell lines is less reliable due to smaller cohorts. Alternatively, it would mean that cell lines indeed transform during in vitro culturing and do not always reflect the phenotype of the correspondent primary tumor.
In the text belonging to Figure 3A, the authors draw the conclusion that the presented analysis might help to predict the origin of the tumor. One should be careful with this presumption, since at least in breast cancer it has been shown that basal-like tumors arise from luminal/epithelial, rather than from basal/mesenchymal populations (Ref. 4, and 5).
Next, the authors investigate whether the generic EMT-signature has clinical implications (Figure 4). They find that the EMT status does not entirely correlate with clinical response in patients suffering from breast or ovarian cancer. It is most surprising that the distribution of Epi and Mes phenotypes is very similar between patients that show a good clinical response and those suffering

from a progressive disease. Together with the drug sensitivity data, this analysis shows that EMT should not be the only factor taken into consideration when deciding over treatment regimens. Nevertheless, the authors were able to show that mesenchymal ovarian cancers respond better to chemotherapy than their epithelial counterparts. To consolidate the ability of the EMT score to predict sensitivity to chemotherapy it would be necessary to assess additional data from several cancer types and maybe even compare cancer entities that tend to be more mesenchymal (e.g. colon) to the more epithelial ones (e.g.: liver or renal).
In the discussion, the authors mention the EMT score as a method to assess effectiveness of EMT reversion therapies, aiming to inhibit the metastatic tumor potential. While the idea that the EMT score can detect phenotypic transitions is uncontestable, stating that the reversion of the EMT process leads to less metastasis is not entirely correct. It was recently shown that reversion of EMT even promotes metastatic colonization (Ref. 6).
Taken altogether, EMT scoring can be very useful when classifying tumor entities, but one should be aware of its limitations when it comes to the choice of therapy and the prediction of clinical outcome. The authors therefore provide a versatile tool for tumor classification based on their EMT status, but its power in clinical applications should not be overrated.

1. Nakajima S. et al. N-cadherin expression and epithelial-mesenchymal transition in pancreatic carcinoma. Clin Cancer Res. 2004 Jun 15;10(12 Pt 1):4125-33.
2. Hotz B. et al. Epithelial to mesenchymal transition: expression of the regulators snail, slug, and twist in pancreatic cancer. Clin Cancer Res. 2007 Aug 15;13(16):4769-76.
3. Arumugam T. et al. Epithelial to mesenchymal transition contributes to drug resistance in pancreatic cancer. Cancer Res. 2009 Jul 15;69(14):5820-8.
4. Lim E. et al. Aberrant luminal progenitors as the candidate target population for basal tumor development in BRCA1 mutation carriers. Nat Med. 2009 Aug;15(8):907-13.
5. Molyneux G. et al. BRCA1 basal-like breast cancers originate from luminal epithelial progenitors and not from basal stem cells. Cell Stem Cell. 2010 Sep 3;7(3):403-17.
6. Tsai JH et al. Spatiotemporal regulation of epithelial-mesenchymal transition is essential for squamous cell carcinoma metastasis. Cancer Cell. 2012 Dec 11;22(6):725-36.

Referee #3 (Comments on Novelty/Model System):

Future cancer subtype analysis and metastasis-free survival analysis are strongly suggested.

Referee #3 (Remarks):

In this manuscript the authors report a novel EMT scoring method to quantitatively estimate the degree of EMT in cell lines and tumors. The authors first developed cancer-specific EMT signatures using transcriptomics of a large collection of cancer cell lines and clinical samples, and demonstrated that they exhibit good correlation with published cancer-specific EMT signatures. From the established cancer-specific EMT signatures, they further derived a generic EMT signature and a method for universal EMT scoring. The authors also applied this EMT scoring to examine its efficacy in correlating EMT status with patient survival rates and responses to treatment.

This is an interesting study that is aims at developing methods to quantitatively assess the correlation between EMT status and cancer characteristics. The authors developed and applied a valid methodology, and the quality and explanation of data is adequate, making the conclusion robust. The paper is therefore of high scientific interest.

I suggest the following possible revisions to improve the quality of this manuscript:

1. Figure 1 and E1. The breast cancer-specific EMT signature is well explained and validated. However, it is not sure how some of the other cancer-specific EMT signatures are derived and verified, for example the ovarian and bladder cancers, as depicted in Figure 2A.

2. Figure 3 and E2. It is claimed by the authors that EMT status does not necessarily correlate with poorer survival. Did the authors try performing the analyses after stratifying patients based on different subtypes of the same cancer? For example, will performing Kaplan-Meier analysis by

subtypes of tumors (e.g. luminal, basal, ERBB2+, triple-negative subtypes of breast cancer) give a different result?

3. Figure 3 and E2. Given the critical roles of EMT in tumor cell dissemination and cancer metastasis, it is suggested that the authors also check metastasis-free survival, not only OS and DFS.

4. There is growing evidence suggesting that the reversal of EMT, the mesenchymal-epithelial transition (MET), may be necessary for efficient metastatic colonization. The authors should discuss this point and the potential complications in the interpretation of their results.

Minor concerns:

1. What are the values of the y axis in Figure 1C (top panel)?

1st Revision - authors' response 30 July 2014

Referee #1 (Comments on Novelty/Model System):

The presented study provides a potentially useful tool to evaluate and objectify EMT signatures in primary cancers as well as cancer-derived tumor cells. Most importantly, it would substantially increase the impact of the study if the authors provide an easy to handle on line tool allowing researchers to rapidly assess EMT signatures in their biological samples of interest or primary patient material. This would also increase the impact of the study and improve signatures to be used in evaluating therapy regimes.

Referee #1 (Remarks):

Tan et al. developed a method to quantify the EMT status of tumors and tumor-derived cell lines by developing a generic EMT gene expression signature. Based on transcriptomics of ovarian, breast, bladder, lung, colorectal and gastric cancers Tan et al. defined a gene expression signature that discriminates between epithelial (low, negative EMT score), mesenchymal (high, positive EMT score), or intermediate phenotypes, which display a certain degree of plasticity. Consistent with the hypothesis that this generic EMT signature monitors the cellular phenotype, functional studies demonstrated increased EMT scores upon e.g. knockdown of CDH1 or overexpression of TWIST in epithelial cancer cells. By 'EMT scoring' clinical samples and cell lines from various cancers, the authors identified cancer type-specific EMT states with e.g. predominant epithelial phenotypes in colorectal cancers. Most strikingly, however, the correlation of primary signatures with those observed in cancer-derived cells revealed a striking correlation and appeared barely biased by stromal content in primary material. In contrast to previous suggestions, poor patient overall (OS) and disease-free survival DFS were not exclusively associated with mesenchymal, but also with the epithelial cancer phenotypes. Moreover, by correlating the EMT score with chemotherapeutic responses of cancer cell lines Tan et al. suggest that the EMT-score indicates prevalence in the chemo-sensitivity of certain tumors instead of a general resistance or reduced sensitivity of MC-like tumors. This suggestion was further evaluated by analyzing Paclitaxel sensitivity of ovarian cancer

patients. Supporting the hypothesis of phenotype-specific responses, Paclitaxel increased OS and DFS of patients suffering from mesenchymal ovarian carcinomas, but decreased the prognosis for patients suffering from epithelial ovarian carcinomas.

Remark:

The manuscript presents a highly interesting tool, that could substantially contribute to objectifying the evaluation of EMT phenotypes in vitro as well as malignancies. However, in its present form the scoring remains difficult to apply, involves too many genes whereas other potential markers are not considered and lacks an option to include the estimation and correlation with stemness phenotypes.

> We appreciate the reviewer's positive comments regarding our tool. We agree with the Referee#1 that the tool in its current form is relatively costly and difficult to apply, especially in the clinical setting. We have carefully considered the three limitations of our scoring system outlaid by Referee#1 and have provided a detailed response to each in the respective comments: i) involves too many genes (Comment 2); ii) Other potential markers are not considered (Comment 3); iii) Lacks an option to include the estimation and correlation with stemness (Comment 4).

Major aspects to be addressed:

Comment 1

The authors should at least provide an easy to use algorithm allowing other researchers to evaluate the EMT-status of their biological system of choice by gene expression signatures derived by microarrays.

> *Response:* We agree with the Referee that it would be more useful if an online tool for EMT scoring is available. However, as we are in the process of patent filing (patent publication number: WO2013043132 A1; http://www.google.com/patents/WO2013043132A1?cl=en), we are not able to make the tool online at this moment. However, researchers can approach us through a temporary link http://www.csi.nus.edu.sg/bioinfo/index.php for computation of EMT score either using our signature or their own in a blinded manner without the need to login. Alternatively, researchers can request for the Matlab script upon signing of a Material Transfer Agreement (MTA) with our Institution. Details for such requests are given in Materials and Methods under 'Computation of EMT score'. The current script implementing the algorithm takes input formats from GSEA: GCT (for input gene expression data) and GMT (for gene signature). Details of the format can be obtained from the following website: http://www.broadinstitute.org/cancer/software/gsea/wiki/index.php/Data_formats. We are currently preparing a follow-up paper in requirement of patent filing, in which we will make the online tool available once we are granted such clearance.

Comment 2

The authors should evaluate if their signatures perform equally well with a reduced set of genes analyzed. This would potentially allow evaluating EMT signatures based on smaller qRT-PCR arrays. At least the authors should identify the 'best performing' genes.

Response: We agree with Referee#1 that the tool will be more attractive in terms of cost and utility, especially in the clinical setting, if the number of genes could be reduced. Indeed, we are in the process of patent filing and are working on a follow-up paper using a reduced number of genes for diagnostic purposes (publication number: WO2013043132 A1). One possible way to identify the best-performing genes is based on the weights of differentially expressed genes between epithelial and mesenchymal states given in Table E1A. However, as pointed out by the reviewer, it is important that the reduced set will perform equally well. Thus, we have studied the effect of reducing the number of genes in the generic EMT signature in terms of correlating EMT scores between a reduced and the full EMT signature in the manuscript. We performed the analysis for both tumors and cell lines. We compared the overall concordance in tumor EMT phenotype estimated by the full and reduced EMT signatures, and re-validated the EMT scoring on the reduced set using the same set of functional intervention dataset as in Fig. E3 and Table E3 of the revised manuscript. The EMT score computed from the reduced set has been added to Table E3 and E4A. The analysis and result are given in the Expanded View section 'Effect of reducing genes in generic EMT signature' and in Fig. E5.

Comment 3

Along these lines it remains elusive why the authors did not include miRNAs as additional markers, since these were shown to be substantially deregulated in various cancer according to the EMT-status, for instance the miR-200 family.

Response: We agree with Referee#1 that including miRNAs in the signature might allow for the more precise quantification of EMT. However, as we seek to quantify EMT in various cancers, and because the development of our signature and scoring system was based on a relatively large number of cell lines and tumors, the amount of miRNA data is not as abundantly available as mRNA gene expression data. This prompted us instead to focus only on mRNA gene expression data. Furthermore, since our EMT scoring system is based on a generic method two-sample Kolmogorov-Smirnov test, the EMT signature is therefore customizable. The present generic EMT signature could be further refined or modified. In order to identify potential miRNA markers, we gathered 6 datasets where miRNA and mRNA expression data are available from GEO (bladder, GSE40355; pancreas, GSE32688; prostate, GSE21034; breast, ovarian, TCGA; multiple myeloma, GSE17498) (The Cancer Genome Atlas, 2012; The Cancer Genome Atlas Research, 2011;

Donahue *et al*, 2012; Hecker *et al*, 2013; Lionetti *et al*, 2009; Taylor *et al*, 2010), and correlated generic EMT score with miRNA expression. Looking at miRNA implicated in EMT (Hao *et al*, 2014; Zhang & Ma, 2012), we found that the miR-200 family members (miR-200a, miR-200b, miR-200c, miR-141, miR-429) negatively correlate with the generic EMT score (Fig. E2). However, we also noted that the miRNA involved in promoting or suppressing EMT might be cancer-dependent, as the correlation or anti-correlation were not consistent in all cancers or with previous reports (Hao *et al*, 2014; Zhang & Ma, 2012). This discrepancy may also stem from the platform-specific, cross-hybridization problem, as miRNA are short, closely related sequences (between family members), and hence measuring their expression is technically challenging (Mestdagh *et al*, 2014). Nevertheless, the expression of miR-200 and miR-34 (miR-34a, miR-34b, miR-34c) families were relatively consistent across bladder, breast, pancreas, prostate, and ovarian cancers; hence, scoring EMT using an miRNA-based EMT signature would complement and may allow for a more precise estimate of EMT when used in concert with an mRNA-based EMT signature. It is important to note that this assessment is preliminary, as only small cohorts were analysed. An in-depth investigation will be only possible when more data with both miRNA and mRNA gene expression data become available. We have included and discussed the result of the analysis in Fig. E2 and in the Expanded View section 'Generic EMT signature and miRNA' in the revised manuscript.

Comment 4

The authors should attempt to assess the expression of putative CSC-markers reported for various cancers and try to correlate this with their EMT signature. In addition the authors have to correlate their signature with previously reported stemness signatures (e.g. Ben-Porath et al., 2008.)

Response: Because EMT is associated with the acquisition of stemness (Frisch *et al*, 2013; Huang *et al*, 2012; Tam & Weinberg, 2013; Thiery *et al*, 2009), as suggested by the Referee, we have assessed the correlation of the generic EMT score and stemness using 21 published stem cell gene sets found in the Molecular Signature Database (Msigdb v4.0)(Subramanian *et al*, 2005), as well as those published stem cell markers (Medema, 2013). The enrichment score from ssGSEA (Verhaak *et al*, 2013) of the 21 stem cell related gene sets (*Beier Glioma stem cell*, *Ben-Porath embryonic stem cell v1*, *Ben-Porath embryonic stem cell v2*, *Ben-Porath NANOG targets*, *Ben-Porath NOS targets*, *Ben-Porath OCT4 targets*, *Ben-Porath SOX2 targets*, *Bhattacharya embryonic stem cell*, *BIOCARTA stem pathway*, *Boquest stem cell cultured vs fresh*, *Boquest stem cell*, *Conrad stem cell*, *Gal leukemic stem cell*, *Gentles leukemic stem cell*, *Hoebeke lymphoid stem cell*, *Jaatinen hematopoietic stem cell*, *Lee neural crest stem cell*, *Oswald hematopoietic stem cell in collagen gel*, *Pece mammary stem cell*, *Wong embryonic stem cell core*, *Yamashita liver cancer stem cell*) were computed and correlated with the EMT score for each cancer type.

The generic EMT score does not correlate universally with stemness in all cancer types. It is likely that different cancers employ different programs for expressing stem cell properties. Positive correlations between generic EMT score and stem cell signatures such as *Boquest stem cell*, *Pece mammary stem cell* or gene expression of stem cell markers such as *CD44* and *CXCR4* were observed in the majority of the cancer types. However, this analysis is preliminary due to certain limitations: the stem cell signatures were derived from different cell types, and no consideration was given to the different types of stem cells. For example, in breast cancer, there exist at least two types of stem cells (Liu *et al*, 2014). Nevertheless, we added this data into the Results section in the Expanded View, Fig. E11 and commented on it in the Discussion section of the main text.

Minor aspects:

1) Wherefrom were the data derived used for analyses in Figure E1? Where are the references? The same applies for Figure 1B, C. The data processing and experimental procedures are not or barely described in the methods section or in the figure legend.

Response: The data in Figures 1B, 1C and E1 (E3 in the revised manuscript) are from various datasets. The source of the downloaded dataset and the associated references were given in Tables E3 and E8. As pointed out by Referee#1, we now mention the source of the dataset and cite references in the figure legends of Figures 1B, 1C and E3. The data processing procedure and software used were given in 'Section Data preprocessing for Affymetrix microarray expression data'. Since the experimental procedures of these public datasets were described in their publications, and many datasets were involved, we did not duplicate these descriptions in the Materials and Methods. We instead provided the references in Tables E6 and E8.

2) Figure E2B should be included in the main figure section. I would prefer one main figure together with Figure 3B. Accordingly, Figure 3A should be one main figure.

Response: We have re-arranged the figures such that Figure E2B and Figure 3B form the new Figure 4 in the revised manuscript, and Figure 3A has been changed to the new Figure 3 in the revised manuscript, as suggested by Referee#1.

Referee #2 (Comments on Novelty/Model System):

The reviewers wish to state that they are not able to evaluate in detail the soundness of the bioinformatics/statistics in the manuscript as they are not experts in that area. They were, however able to evaluate the importance and context of the question addressed, novelty and the biological background.

Referee #2 (Remarks):

The authors provide a universal EMT-signature that can be applied to several cancer types. They show that this tool allows classification of tumour samples or cell lines due to their epithelial or mesenchymal genetic profile. In doing so, the authors very well appreciate the limitations of in vitro cell line models and distinguish between gene signatures of primary tumours and cell lines. In the end, the generated tumour-specific EMT-signature is correlated to clinical outcome. The authors address one of the most pressing questions in the field, i.e., is there a general EMT-signature and how does it affect clinical behaviour in different entities. They do so in an unbiased and careful manner, thereby putting out a tool (the generic EMT-signature) that should be of broad interest and utility to a lot of researchers from different fields of inquiry.

> *Response:* We thank Referee#2 for supporting the importance of the tool and for raising several issues critical to the improvement of our manuscript. We have provided detailed responses to each of the issues raised in the following sections.

A few minor issues might be of interest to address.

<u>Comment 1:</u>
For the creation of the generic, cancer-specific EMT-signature, the authors use gene sets of different tissues (bladder, breast, colorectal, gastric, lung and ovarian) (Figure 2). Given the implications of EMT in pancreatic cancer (PC) and the emerging evidence for a negative correlation between EMT and the survival rates of patients with PC (Ref. 1-3), inclusion of PC-specific EMT-signatures should be taken into consideration.

Ref. 1. Nakajima S. et al. N-cadherin expression and epithelial-mesenchymal transition in pancreatic carcinoma. Clin Cancer Res. 2004 Jun 15;10(12 Pt 1):4125-33.
Ref. 2. Hotz B. et al. Epithelial to mesenchymal transition: expression of the regulators snail, slug, and twist in pancreatic cancer. Clin Cancer Res. 2007 Aug 15;13(16):4769-76.
Ref. 3. Arumugam T. et al. Epithelial to mesenchymal transition contributes to drug resistance in pancreatic cancer. Cancer Res. 2009 Jul 15;69(14):5820-8.

> *Response:* We thank Referee#2 for the references and for pointing out the implications of EMT in pancreatic cancer. We are in complete agreement with Referee#2 that EMT could play a role in pancreatic cancer. Indeed, in Fig. 3 of the revised manuscript, we observed an EMTed pancreatic cancer population in both tumors and cell lines. As we are seeking a

generic EMT signature that is common amongst the different cancer types and since Figure E3 in the revised manuscript shows that EMT scoring is capable of accurately predicting EMT status of pancreatic cancer cell lines, we believe the present EMT scoring is also applicable to pancreatic cancer. In addition, we showed in Table E1C that this generic EMT scoring is highly correlated with cancer-specific EMT. Hence, we opine that it is highly likely that incorporating a pancreatic-specific EMT signature would not change significantly the present generic EMT signature. Thus, we did not repeat the procedure to generate a pancreatic cancer-specific EMT signature or generic EMT signature, but rather, we performed additional analyses validating the applicability of this generic EMT signature in pancreatic cancer and added the data to the Results section, Expanded View and Fig. E4 of the revised manuscript. Furthermore, we have included two recent pancreatic datasets that have overall survival information, and have studied the correlation of EMT and survival in pancreatic cancer as well (Fig. 4A in revised manuscript). Even though it is not significant, there is a trend that EMT correlates with poorer overall survival, consistent with previously published results (Arumugam *et al*, 2009; Hotz *et al*, 2007; Nakajima *et al*, 2004).

Comment 2.1:

In Figure 3A, the generic EMT score is applied to several cancer entities, including data belonging to primary tumours and cell lines. Although many clinical samples and cell lines of the same cancer type overlap in their Epi/Mes classification, there are also cases of discrepancy (bladder, ovarian, lung and prostate carcinomas). Unfortunately, the authors do not discuss these differences. One possibility could be that the EMT-score for cell lines is less reliable due to smaller cohorts. Alternatively, it would mean that cell lines indeed transform during in vitro culturing and do not always reflect the phenotype of the correspondent primary tumour.

Response: We thank Referee#2 for pointing out this oversight. We agree with Referee#2 that the EMT score distribution of clinical samples and cell lines of the same cancer type were largely similar in most cases but that there were also discrepancies in terms of EMT score mean and range in some cases such as ovarian and prostate cancers. Although it is true that cell lines acquire additional transformations in culture, we believe the discrepancies stemmed from the differences in the number of samples, and that the data of cell lines and tumours were not paired. Hence, the different compositions for each cancer type such as histology, grade, and stage may have caused the difference in the distribution of the EMT scores. For example, in prostate cancer, the tumours were made up of primary and metastatic, recurrent and non-recurrent prostate carcinoma, whereas the 8 prostate cancer cell lines were derived mainly from metastatic sites (Table R1). We have provided this explanation of the discrepancies in the Results section in the revised manuscript.

Table R1: Prostate cell lines in Fig. 3

Cell Line	Cancer	Tissue Source	ATCC code/PMID
22RV1	prostate	prostate	CRL-2505
BPH-1	prostate	transurethral resection	7535634
DU 145	prostate	derived from metastatic site: brain	HTB-81
LNCaP clone FGC	prostate	derived from metastatic site: left supraclavicular lymph node	CRL-1740
MDA PCa 2b	prostate	derived from metastatic site: bone	CRL-2422
NCI-H660	prostate	derived from metastatic site: lymph node	CRL-5813
PC-3	prostate	derived from metastatic site: bone	CRL-1435
VCaP	prostate	derived from metastatic site: vertebral metastasis	CRL-2876

Comment 2.2:

In the text belonging to Figure 3A, the authors draw the conclusion that the presented analysis might help to predict the origin of the tumour. One should be careful with this presumption, since at least in breast cancer it has been shown that basal-like tumours arise from luminal/epithelial, rather than from basal/mesenchymal populations (Ref. 4, and 5).

Ref. 4. Lim E. et al. Aberrant luminal progenitors as the candidate target population for basal tumour development in BRCA1 mutation carriers. Nat Med. 2009 Aug;15(8):907-13.
Ref. 5. Molyneux G. et al. BRCA1 basal-like breast cancers originate from luminal epithelial progenitors and not from basal stem cells. Cell Stem Cell. 2010 Sep 3;7(3):403-17.

Response: Our intention was to say that the wide range of the EMT score observed within a cancer type might reflect the difference in cell of origin in the cancer. For example, in breast cancer, subtypes Luminal-A and -B originated from mature luminal cells (hence more Epi), whereas the Basal subtype originated from luminal progenitors (hence more Mes) (Lim *et al*, 2009). We did not mean to imply that EMT is predictive of the cell of origin. We have removed these sentences from the Results section to avoid this confusion in the revised manuscript.

Comment 3:

Next, the authors investigate whether the generic EMT-signature has clinical implications (Figure 4). They find that the EMT status does not entirely correlate with clinical response in patients suffering from breast or ovarian cancer. It is most surprising that the distribution of Epi and Mes phenotypes is very similar between patients that show a good clinical response and those suffering from a progressive disease. Together with the drug sensitivity data, this analysis shows that EMT should not be the only factor taken into consideration when deciding over treatment regimens. Nevertheless, the authors were able to show that mesenchymal ovarian cancers respond better to chemotherapy than their epithelial counterparts. To consolidate the ability of the EMT score to

predict sensitivity to chemotherapy it would be necessary to assess additional data from several cancer types and maybe even compare cancer entities that tend to be more mesenchymal (e.g. colon) to the more epithelial ones (e.g.: liver or renal).

Response: As per the suggestions of Referee#2, we have assessed additional data from 11 breast cancer cohorts (GSE48905, GSE33658, GSE23428, GSE22226, GSE18864, GSE28796, GSE16646, GSE22513, GSE4779, GSE18728, GSE50948)(Bauer *et al*, 2010; Carey *et al*, 2012; Esserman *et al*, 2012; Evans *et al*, 2012; Farmer *et al*, 2009; Knudsen *et al*, 2014; Korde *et al*, 2010; Lehmann *et al*, 2011; Massarweh *et al*, 2011; Prat *et al*, 2014; Silver *et al*, 2010), 3 colorectal cancer cohorts (GSE19862, GSE35452, GSE46862) (Gim J, 2014), a head & neck cancer cohort (GSE32877) (Tomkiewicz *et al*, 2012) and a melanoma cohort (GSE22968) (Beasley *et al*, 2011) where clinical response data was available. There is no survival data available in these cohorts. Interestingly, the data from the 11 breast cancer cohorts subjected to various treatments, including fulvestrant, anastrazole, carboplatin, doxorubicin, and other drugs (Fig. E8A), showed a similar distribution of Epi, intermediate and Mes breast cancers in each of the clinical response groups as we noted in Fig. 5A. Notably, the worst response group (progressive disease or residual disease) was made up of mostly Mes breast cancers. There is a trend of either an increasing proportion of Mes or a decreasing proportion of Epi toward chemoresistant groups. In the predominantly Epi colorectal and head & neck cancers and the predominantly Mes melanoma, because the distribution of EMT score does not allow us to segregate into Epi, intermediate and Mes groups, we instead investigated the EMT score profiles of responders and non-responders in these cancers (Fig. E8B). We found no significant difference between responders and non-responders in terms of EMT score, albeit there is a very slight trend that responders tend to have higher EMT score in predominantly Epi cancers (colorectal cancer), and a slight trend that responders tend to have a lower EMT score in predominantly Mes cancers (melanoma). We were not able to compare the results with that of the cell lines (Table E5) because the majority of these cohorts were treated with a combination of chemotherapeutics. Furthermore, as these data are from a relatively small cohort, further study is required to validate the current observation. These analyses have been added to the Results section, and the Expanded View of the revised manuscript.

On the other hand, we were not able to verify the findings of either the benefit of Mes tumour to paclitaxel in other cancer types because the data were not available. Instead, we found three datasets with survival and treatment information (glioma, GSE43388; ER+ breast cancer, GSE16391, and multiple myeloma, GSE9782) (Desmedt *et al*, 2009; Erdem-Eraslan *et al*, 2013; Mulligan *et al*, 2007). Interestingly, we observed a differential response in Epi and Mes tumours (Fig. E10). Even though, in general, patients with glioma receiving radiotherapy and chemotherapy have a better overall survival, the benefit is greater in

patients with a Mes glioma (*p*=0.0117). In contrast, patients with Epi multiple myeloma have a better disease-free survival rate when administered bortezomib instead of dexamethasone (*p*=0.0349). We observed no difference in ER+ tumours in patients administered with letrozole or tamoxifen in terms of EMT stratification. These data provide preliminary evidence of the potential benefit in stratifying patients by EMT status using generic EMT scoring. We concede that the cohorts were relatively small. However, we believe that our analysis would open the door to opportunities for incorporating the EMT scoring system into translational research protocols of clinical trials in the future when more data is available to assess the benefit of stratification by EMT.

Comment 4:

In the discussion, the authors mention the EMT score as a method to assess effectiveness of EMT reversion therapies, aiming to inhibit the metastatic tumor potential. While the idea that the EMT score can detect phenotypic transitions is uncontestable, stating that the reversion of the EMT process leads to less metastasis is not entirely correct. It was recently shown that reversion of EMT even promotes metastatic colonization (Ref. 6).

Ref. 6. Tsai JH et al. Spatiotemporal regulation of epithelial-mesenchymal transition is essential for squamous cell carcinoma metastasis. Cancer Cell. 2012 Dec 11;22(6):725-36.

Response: We thank the reviewer for raising this important point. Tsai et al. is a landmark paper and we fully agree with Tsai et al. as well as with Referees#2 and #3 that reversing EMT may promote metastatic colonization. This paradigm, to an extent, fits our previously proposed model where mesenchymal micro-metastases must re-acquire an epithelial phenotype to proliferate at the metastatic site (Thiery, 2002). The original text in the manuscript was not accurate ("the EMT reversion therapy as a means to reduce the metastatic potential") in that we are not postulating that EMT reversal therapy could *cure* metastasis, but instead we meant that EMT reversal therapy may *sensitize* cancer cells to specific drugs. Furthermore, our intent was not to achieve a full reversal of EMT—which, as pointed out by Referees#2, #3 and Tsai et al. could be detrimental—rather, we were referring to modifying the EMT status *along the spectrum* to improve patient responses to treatment. In our experience, the use of a single agent does not fully reverse EMT but it is sufficient to reduce invasion (unpublished), anoikis, spheroidogenesis and clonogenicity *in vitro* (Huang *et al*, 2013), and *in vivo* using orthotopic grafting of a human cancer line (Sim & Thiery, 2014; manuscript in preparation). The main challenge, however, is that we do not know exactly at which intermediate states and under what conditions or context cancer cells in the primary—or at distant sites—can exit dormancy and resume growth or develop chemo-resistance. This is a very complex problem requiring further investigations which are beyond the scope of the current study. We have amended the sentence to better reflect what we meant in the Discussion section of the revised manuscript.

Comment 5:

Taken altogether, EMT scoring can be very useful when classifying tumor entities, but one should be aware of its limitations when it comes to the choice of therapy and the prediction of clinical outcome. The authors therefore provide a versatile tool for tumor classification based on their EMT status, but its power in clinical applications should not be overrated.

> *Response:* We thank you the reviewer for supporting the utility of this tool, and we have re-written sentences in the manuscript regarding its clinical application such that they do not overstate the clinical utility of our tool in the revised manuscript.

Referee #3 (Comments on Novelty/Model System):

Future cancer subtype analysis and metastasis-free survival analysis are strongly suggested.

Referee #3 (Remarks):

In this manuscript the authors report a novel EMT scoring method to quantitatively estimate the degree of EMT in cell lines and tumors. The authors first developed cancer-specific EMT signatures using transcriptomics of a large collection of cancer cell lines and clinical samples, and demonstrated that they exhibit good correlation with published cancer-specific EMT signatures. From the established cancer-specific EMT signatures, they further derived a generic EMT signature and a method for universal EMT scoring. The authors also applied this EMT scoring to examine its efficacy in correlating EMT status with patient survival rates and responses to treatment.

This is an interesting study that is aims at developing methods to quantitatively assess the correlation between EMT status and cancer characteristics. The authors developed and applied a valid methodology, and the quality and explanation of data is adequate, making the conclusion robust. The paper is therefore of high scientific interest.

I suggest the following possible revisions to improve the quality of this manuscript:

Comment 1:

Figure 1 and E1. The breast cancer-specific EMT signature is well explained and validated. However, it is not sure how some of the other cancer-specific EMT signatures are derived and verified, for example the ovarian and bladder cancers, as depicted in Figure 2A.

> *Response:* Figure 1 depicted the procedure for deriving the cancer-specific EMT signature for the bladder, colorectal, gastric, and lung cancer-specific EMT signatures. Breast and

ovarian cancer-specific EMT signatures were derived using a similar procedure (apart from Steps 1 and 2 depicted in Figure 1A) and were published elsewhere (Akalay *et al*, 2013; Miow *et al*, 2014); these were derived from comparing the profile of immunofluorescence staining of CDH1 and CDH2 (Materials and Methods). Since the cancer-specific EMT signatures were derived using the same procedure and have good correlation with the generic EMT signature (Table E1C), we performed additional analyses to verify only the validity of bladder cancer-specific EMT signature. The result is added in Fig. E1, Results section, and Expanded View of the revised manuscript.

<u>Comment 2</u>

Figure 3 and E2. It is claimed by the authors that EMT status does not necessarily correlate with poorer survival. Did the authors try performing the analyses after stratifying patients based on different subtypes of the same cancer? For example, will performing Kaplan-Meier analysis by subtypes of tumors (e.g. luminal, basal, ERBB2+, triple-negative subtypes of breast cancer) give a different result?

Response: We focused our analysis on correlating EMT status and survival only in molecular subtypes of breast cancer, as the molecular subtypes of breast cancer is more established (Prat *et al*, 2012). When stratified by molecular subtype, we observed a better disease-free survival (DFS) in patients with Basal and Claudin-Low breast cancers of a low EMT score than in those with a high EMT score (hazard ratio=0.6549, p=0.0089). There was no significant difference in other breast cancer subtypes when correlating EMT and DFS or overall survival. However, the correlation of EMT and DFS in Basal and Claudin-Low subtypes was not coherent in all breast cancer cohorts, probably due to the smaller sample sizes. Therefore, we did not report this finding initially. As pointed out by Referee#3, it might be of great interest to the reader, and we have added these results to Fig. E7, as well as the Results and Discussion sections.

<u>Comment 3</u>:

Figure 3 and E2. Given the critical roles of EMT in tumor cell dissemination and cancer metastasis, it is suggested that the authors also check metastasis-free survival, not only OS and DFS.

Response: We thank the Referee for pointing this out, as this made us aware that we were not clear in the definition of disease-free survival (DFS). The definition of DFS we adopted in the manuscript broadly encompassed (local) recurrence-free survival, progression-free survival, and distant metastasis-free survival (DMFS). The majority of the data were in fact distant metastasis-free survival. We have indicated in Fig. 3B which cohort data are DMFS, and inserted the definition of DFS in the text.

<u>Comment 4:</u>

There is growing evidence suggesting that the reversal of EMT, the mesenchymal-epithelial transition (MET), may be necessary for efficient metastatic colonization. The authors should discuss this point and the potential complications in the interpretation of their results.

Response: We thank the reviewer for raising this important point. Tsai et al. is a landmark paper and we fully agree with Tsai et al. as well as with Referees#2 and #3 that reversing EMT may promote metastatic colonization. This paradigm, to an extent, fits our previously proposed model where mesenchymal micro-metastases must re-acquire an epithelial phenotype to proliferate at the metastatic site (Thiery, 2002). The original text in the manuscript was not accurate ("the EMT reversion therapy as a means to reduce the metastatic potential") in that we are not postulating that EMT reversal therapy could *cure* metastasis, but instead we meant that EMT reversal therapy may *sensitize* cancer cells to specific drugs. Furthermore, our intent was not to achieve a full reversal of EMT—which, as pointed out by Referees#2, #3 and Tsai et al. could be detrimental—rather, we were referring to modifying the EMT status *along the spectrum* to improve patient responses to treatment. In our experience, the use of a single agent does not fully reverse EMT but it is sufficient to reduce invasion (unpublished), anoikis resistance, spheroidogenesis and clonogenicity *in vitro* (Huang *et al*, 2013), and *in vivo* using orthotopic grafting of a human cancer line (Sim & Thiery, 2014; manuscript in preparation). The main challenge, however, is that we do not know exactly at which intermediate states and under what conditions or context cancer cells in the primary—or at the distant sites—can exit dormancy and resume growth or develop chemo-resistance. This is a very complex problem requiring further investigations which are beyond the scope of the current study. We have amended the sentence to better reflect what we meant in the Discussion section of the revised manuscript.

Minor concerns:

1. What are the values of the y axis in Figure 1C (top panel)?

Response: We have put in the y-axis values on the top panel of Figure 1C.

References

Akalay I, Janji B, Hasmim M, Noman MZ, Andre F, De Cremoux P, Bertheau P, Badoual C, Vielh P, Larsen AK et al (2013) Epithelial-to-mesenchymal transition and autophagy induction in breast carcinoma promote escape from T-cell-mediated lysis. Cancer research 73: 2418-2427

Arumugam T, Ramachandran V, Fournier KF, Wang H, Marquis L, Abbruzzese JL, Gallick GE, Logsdon CD, McConkey DJ, Choi W (2009) Epithelial to mesenchymal transition contributes to drug resistance in pancreatic cancer. Cancer research 69: 5820-5828

Bauer JA, Chakravarthy AB, Rosenbluth JM, Mi D, Seeley EH, De Matos Granja-Ingram N, Olivares MG, Kelley MC, Mayer IA, Meszoely IM et al (2010) Identification of markers of taxane sensitivity using proteomic and genomic analyses of breast tumors from patients receiving

© EMBO

neoadjuvant paclitaxel and radiation. Clinical cancer research : an official journal of the American Association for Cancer Research 16: 681-690

Beasley GM, Riboh JC, Augustine CK, Zager JS, Hochwald SN, Grobmyer SR, Peterson B, Royal R, Ross MI, Tyler DS (2011) Prospective multicenter phase II trial of systemic ADH-1 in combination with melphalan via isolated limb infusion in patients with advanced extremity melanoma. Journal of clinical oncology : official journal of the American Society of Clinical Oncology 29: 1210-1215

The Cancer Genome Atlas Research (2012) Comprehensive molecular portraits of human breast tumours. Nature 490: 61-70

The Cancer Genome Atlas Research (2011) Integrated genomic analyses of ovarian carcinoma. Nature 474: 609-615

Carey LA, Rugo HS, Marcom PK, Mayer EL, Esteva FJ, Ma CX, Liu MC, Storniolo AM, Rimawi MF, Forero-Torres A et al (2012) TBCRC 001: randomized phase II study of cetuximab in combination with carboplatin in stage IV triple-negative breast cancer. Journal of clinical oncology : official journal of the American Society of Clinical Oncology 30: 2615-2623

Desmedt C, Giobbie-Hurder A, Neven P, Paridaens R, Christiaens MR, Smeets A, Lallemand F, Haibe-Kains B, Viale G, Gelber RD et al (2009) The Gene expression Grade Index: a potential predictor of relapse for endocrine-treated breast cancer patients in the BIG 1-98 trial. BMC medical genomics 2: 40

Donahue TR, Tran LM, Hill R, Li Y, Kovochich A, Calvopina JH, Patel SG, Wu N, Hindoyan A, Farrell JJ et al (2012) Integrative survival-based molecular profiling of human pancreatic cancer. Clinical cancer research : an official journal of the American Association for Cancer Research 18: 1352-1363

Erdem-Eraslan L, Gravendeel LA, de Rooi J, Eilers PH, Idbaih A, Spliet WG, den Dunnen WF, Teepen JL, Wesseling P, Sillevis Smitt PA et al (2013) Intrinsic molecular subtypes of glioma are prognostic and predict benefit from adjuvant procarbazine, lomustine, and vincristine chemotherapy in combination with other prognostic factors in anaplastic oligodendroglial brain tumors: a report from EORTC study 26951. Journal of clinical oncology : official journal of the American Society of Clinical Oncology 31: 328-336

Esserman LJ, Berry DA, Cheang MC, Yau C, Perou CM, Carey L, DeMichele A, Gray JW, Conway-Dorsey K, Lenburg ME et al (2012) Chemotherapy response and recurrence-free survival in neoadjuvant breast cancer depends on biomarker profiles: results from the I-SPY 1 TRIAL (CALGB 150007/150012; ACRIN 6657). Breast cancer research and treatment 132: 1049-1062

Evans AL, Faial T, Gilchrist MJ, Down T, Vallier L, Pedersen RA, Wardle FC, Smith JC (2012) Genomic targets of Brachyury (T) in differentiating mouse embryonic stem cells. PloS one 7: e33346

Farmer P, Bonnefoi H, Anderle P, Cameron D, Wirapati P, Becette V, Andre S, Piccart M, Campone M, Brain E et al (2009) A stroma-related gene signature predicts resistance to neoadjuvant chemotherapy in breast cancer. Nature medicine 15: 68-74

Frisch SM, Schaller M, Cieply B (2013) Mechanisms that link the oncogenic epithelial-mesenchymal transition to suppression of anoikis. Journal of cell science 126: 21-29

Gim J CY, Hong SH, Kim HC, Chun HK, Park T*, Park WY, Lee WY (2014) Predicting multi-class response to preoperative chemoradiotherapy in rectal cancer patients, manuscript in preparation.

Hao J, Zhang Y, Deng M, Ye R, Zhao S, Wang Y, Li J, Zhao Z (2014) MicroRNA control of epithelial-mesenchymal transition in cancer stem cells. International journal of cancer Journal international du cancer 135: 1019-1027

Hecker N, Stephan C, Mollenkopf HJ, Jung K, Preissner R, Meyer HA (2013) A new algorithm for integrated analysis of miRNA-mRNA interactions based on individual classification reveals insights into bladder cancer. PloS one 8: e64543

Hotz B, Arndt M, Dullat S, Bhargava S, Buhr HJ, Hotz HG (2007) Epithelial to mesenchymal transition: expression of the regulators snail, slug, and twist in pancreatic cancer. Clinical cancer research : an official journal of the American Association for Cancer Research 13: 4769-4776

Huang RY, Chung VY, Thiery JP (2012) Targeting pathways contributing to epithelial-mesenchymal transition (EMT) in epithelial ovarian cancer. Current drug targets 13: 1649-1653

Huang RY, Wong MK, Tan TZ, Kuay KT, Ng AHC, Chung VY, Chu Y-S, Matsumura N, Lai H-C, Lee YF et al (2013) An EMT Spectrum defines an anoikis resistant and spheroidogenic Intermediate Mesenchymal state that is sensitive to E-cadherin restoration by a Src-kinase inhibitor, Saracatinib (AZD0530). Cell Death and Disease: e915

Knudsen S, Jensen T, Hansen A, Mazin W, Lindemann J, Kuter I, Laing N, Anderson E (2014) Development and validation of a gene expression score that predicts response to fulvestrant in breast cancer patients. PloS one 9: e87415

Korde LA, Lusa L, McShane L, Lebowitz PF, Lukes L, Camphausen K, Parker JS, Swain SM, Hunter K, Zujewski JA (2010) Gene expression pathway analysis to predict response to neoadjuvant docetaxel and capecitabine for breast cancer. Breast cancer research and treatment 119: 685-699

Lehmann BD, Bauer JA, Chen X, Sanders ME, Chakravarthy AB, Shyr Y, Pietenpol JA (2011) Identification of human triple-negative breast cancer subtypes and preclinical models for selection of targeted therapies. The Journal of clinical investigation 121: 2750-2767

Lim E, Vaillant F, Wu D, Forrest NC, Pal B, Hart AH, Asselin-Labat ML, Gyorki DE, Ward T, Partanen A et al (2009) Aberrant luminal progenitors as the candidate target population for basal tumor development in BRCA1 mutation carriers. Nature medicine 15: 907-913

Lionetti M, Biasiolo M, Agnelli L, Todoerti K, Mosca L, Fabris S, Sales G, Deliliers GL, Bicciato S, Lombardi L et al (2009) Identification of microRNA expression patterns and definition of a microRNA/mRNA regulatory network in distinct molecular groups of multiple myeloma. Blood 114: e20-26

Liu S, Cong Y, Wang D, Sun Y, Deng L, Liu Y, Martin-Trevino R, Shang L, McDermott SP, Landis MD et al (2014) Breast Cancer Stem Cells Transition between Epithelial and Mesenchymal States Reflective of their Normal Counterparts. Stem cell reports 2: 78-91

Massarweh S, Tham YL, Huang J, Sexton K, Weiss H, Tsimelzon A, Beyer A, Rimawi M, Cai WY, Hilsenbeck S et al (2011) A phase II neoadjuvant trial of anastrozole, fulvestrant, and gefitinib in patients with newly diagnosed estrogen receptor positive breast cancer. Breast cancer research and treatment 129: 819-827

Medema JP (2013) Cancer stem cells: the challenges ahead. Nature cell biology 15: 338-344

Mestdagh P, Hartmann N, Baeriswyl L, Andreasen D, Bernard N, Chen C, Cheo D, D'Andrade P, DeMayo M, Dennis L et al (2014) Evaluation of quantitative miRNA expression platforms in the microRNA quality control (miRQC) study. Nature methods

Miow QH, Tan TZ, Ye J, Lau JA, Yokomizo T, Thiery J-P, Mori S (2014) Epithelial-Mesenchymal Status Renders Differential Responses to Cisplatin in Ovarian Cancer. Oncogene in press

Mulligan G, Mitsiades C, Bryant B, Zhan F, Chng WJ, Roels S, Koenig E, Fergus A, Huang Y, Richardson P et al (2007) Gene expression profiling and correlation with outcome in clinical trials of the proteasome inhibitor bortezomib. Blood 109: 3177-3188

© EMBO

Nakajima S, Doi R, Toyoda E, Tsuji S, Wada M, Koizumi M, Tulachan SS, Ito D, Kami K, Mori T et al (2004) N-cadherin expression and epithelial-mesenchymal transition in pancreatic carcinoma. Clinical cancer research : an official journal of the American Association for Cancer Research 10: 4125-4133

Prat A, Bianchini G, Thomas M, Belousov A, Cheang MC, Koehler A, Gomez P, Semiglazov V, Eiermann W, Tjulandin S et al (2014) Research-based PAM50 subtype predictor identifies higher responses and improved survival outcomes in HER2-positive breast cancer in the NOAH study. Clinical cancer research : an official journal of the American Association for Cancer Research 20: 511-521

Prat A, Ellis MJ, Perou CM (2012) Practical implications of gene-expression-based assays for breast oncologists. Nature reviews Clinical oncology 9: 48-57

Silver DP, Richardson AL, Eklund AC, Wang ZC, Szallasi Z, Li Q, Juul N, Leong CO, Calogrias D, Buraimoh A et al (2010) Efficacy of neoadjuvant Cisplatin in triple-negative breast cancer. Journal of clinical oncology : official journal of the American Society of Clinical Oncology 28: 1145-1153

Subramanian A, Tamayo P, Mootha VK, Mukherjee S, Ebert BL, Gillette MA, Paulovich A, Pomeroy SL, Golub TR, Lander ES et al (2005) Gene set enrichment analysis: a knowledge-based approach for interpreting genome-wide expression profiles. Proceedings of the National Academy of Sciences of the United States of America 102: 15545-15550

Tam WL, Weinberg RA (2013) The epigenetics of epithelial-mesenchymal plasticity in cancer. Nature medicine 19: 1438-1449

Taylor BS, Schultz N, Hieronymus H, Gopalan A, Xiao Y, Carver BS, Arora VK, Kaushik P, Cerami E, Reva B et al (2010) Integrative genomic profiling of human prostate cancer. Cancer cell 18: 11-22

Thiery JP (2002) Epithelial-mesenchymal transitions in tumour progression. Nature reviews Cancer 2: 442-454

Thiery JP, Acloque H, Huang RY, Nieto MA (2009) Epithelial-mesenchymal transitions in development and disease. Cell 139: 871-890

Tomkiewicz C, Hans S, Mucchielli MH, Agier N, Delacroix H, Marisa L, Brasnu D, Aggerbeck LP, Badoual C, Barouki R et al (2012) A head and neck cancer tumor response-specific gene signature for cisplatin, 5-fluorouracil induction chemotherapy fails with added taxanes. PloS one 7: e47170

Verhaak RG, Tamayo P, Yang JY, Hubbard D, Zhang H, Creighton CJ, Fereday S, Lawrence M, Carter SL, Mermel CH et al (2013) Prognostically relevant gene signatures of high-grade serous ovarian carcinoma. The Journal of clinical investigation 123: 517-525

Zhang J, Ma L (2012) MicroRNA control of epithelial-mesenchymal transition and metastasis. Cancer metastasis reviews 31: 653-662

2nd Editorial Decision 04 August 2014

Thank you for the submission of your revised manuscript to EMBO Molecular Medicine.

We have now received the enclosed reports from the Reviewers that were asked to re-assess it.

As you will see the Reviewers 2 and 3 are now globally supportive. Reviewer 1 was not available and I therefore asked Reviewer 2 to evaluate your response and action to his/her comments. Reviewer 2 considered your reply to Reviewer 1 satisfactory. However, concerning the availability of the easy-to-use algorithm to calculate the EMT signature, while your explanation is deemed

satisfactory, s/he would like you to make the availability of the Matlab script clear in the revised manuscript. I have checked the manuscript and feel that you have clearly stated this availability in the Materials and Methods section. Therefore no further action is required on your part regarding this issue.

I am thus pleased to inform you that we will be able to accept your manuscript.
